# Personalized whole‐body models integrate metabolism, physiology, and the gut microbiome

**DOI:** 10.15252/msb.20198982

**Published:** 2020-05-28

**Authors:** Ines Thiele, Swagatika Sahoo, Almut Heinken, Johannes Hertel, Laurent Heirendt, Maike K Aurich, Ronan MT Fleming

**Affiliations:** ^1^ School of Medicine National University of Ireland Galway Ireland; ^2^ Discipline of Microbiology School of Natural Sciences National University of Ireland Galway Ireland; ^3^ APC Microbiome Cork Ireland; ^4^ Luxembourg Centre for Systems Biomedicine University of Luxembourg Esch‐sur‐Alzette Luxembourg; ^5^ Department of Psychiatry and Psychotherapy University Medicine Greifswald Greifswald Germany; ^6^ Division of Analytical Biosciences Leiden Academic Centre for Drug Research Faculty of Science University of Leiden Leiden The Netherlands; ^7^ Present address: Department of Chemical Engineering, and Initiative for Biological Systems Engineering (IBSE) Indian Institute of Technology Chennai India

**Keywords:** flux balance analysis, human metabolism, metabolic modeling, microbiome, Computational Biology, Metabolism

## Abstract

Comprehensive molecular‐level models of human metabolism have been generated on a cellular level. However, models of whole‐body metabolism have not been established as they require new methodological approaches to integrate molecular and physiological data. We developed a new metabolic network reconstruction approach that used organ‐specific information from literature and omics data to generate two sex‐specific whole‐body metabolic (WBM) reconstructions. These reconstructions capture the metabolism of 26 organs and six blood cell types. Each WBM reconstruction represents whole‐body organ‐resolved metabolism with over 80,000 biochemical reactions in an anatomically and physiologically consistent manner. We parameterized the WBM reconstructions with physiological, dietary, and metabolomic data. The resulting WBM models could recapitulate known inter‐organ metabolic cycles and energy use. We also illustrate that the WBM models can predict known biomarkers of inherited metabolic diseases in different biofluids. Predictions of basal metabolic rates, by WBM models personalized with physiological data, outperformed current phenomenological models. Finally, integrating microbiome data allowed the exploration of host–microbiome co‐metabolism. Overall, the WBM reconstructions, and their derived computational models, represent an important step toward virtual physiological humans.

## Graphical abstract

Sex‐specific, whole‐body human metabolic models were developed and constrained with physiological, dietary, and metabolomic data. They recapitulate known whole‐body metabolic functions and enable mechanistic exploration of host‐microbiome co‐metabolism.

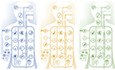

## Introduction

A key aim of precision medicine is the development of predictive, personalizable, and computational models of the human body that can be interrogated to predict potential therapeutic approaches (Kell, 2007). Molecular biology has yielded great insights into the molecular constituents of human cells, but there still remains substantial progress to be made to integrate these parts into a virtual whole human body. While the Virtual Physiological Human and the Physiome projects have generated many anatomically and physiologically representative human body models (Hunter *et al*, 2013), their integration with underlying networks of genes, proteins, and biochemical reactions (Tan *et al*, 2017) is relatively less developed.

The constraint‐based reconstruction and analysis approach (Palsson, 2015) generates a detailed description of molecular‐level processes through the construction of comprehensive, genome‐scale metabolic reconstructions. A reconstruction is assembled based on an organism's genome annotation and current biochemical and physiological knowledge. A metabolic reconstruction is amenable to computational modeling by the imposition of condition‐specific constraints based on experimental data, such as transcriptomics (Opdam *et al*, 2017), proteomics (Yizhak *et al*, 2010), and metabolomics (Aurich *et al*, 2016). Importantly, a metabolic reconstruction serves as a blueprint for many condition‐specific metabolic models, making it a versatile tool for diverse biomedical applications (Aurich & Thiele, 2016; Nielsen, 2017).

A comprehensive *in silico* molecular‐level description of human metabolism is available (Brunk *et al*, 2018); however, the generation of accurate organ‐ and tissue‐specific metabolic models remains challenging using automated approaches and omics data (Opdam *et al*, 2017). At the same time, a solely manual curation approach based on extensive literature review is not tractable due to the large number of organs and cell types in the human body as well as the variable depths of literature on organ‐specific metabolic functions. Hence, a combined algorithmic and manual curation approach is needed, which has already been developed for microbial reconstructions (Magnusdottir *et al*, 2017).

To contribute toward the ambitious goal to develop a human whole‐body model (Kitano, 2010), current systems biology approaches need to go beyond molecular networks to also represent anatomical and physiological properties in the computational modeling framework. For instance, Bordbar *et al* (2011a) created the first multi‐tissue metabolic model where three organ‐specific metabolic models were connected through a blood compartment. However, this model does not accurately describe the mass flow occurring in the human body, which starts with dietary intake followed by transport, metabolism, and elimination of the nutrients and its by‐products. In the absence of such detailed representation, the generic human metabolic reconstruction has been used as a proxy for whole‐body metabolism (Nilsson *et al*, 2017), but it does not yet capture metabolic pathways that occur in parallel in multiple organs to give rise to known physiology, such as the Cori cycle.

**Figure 1:**
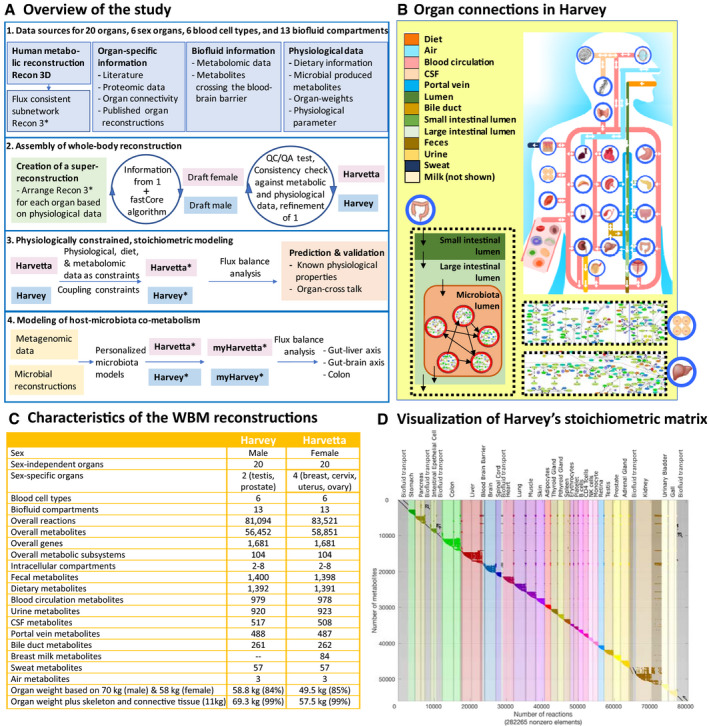
Overview of the reconstruction approach and key features of the organ‐resolved, sex‐specific, curated whole‐body metabolism (WBM) reconstructions Overall approach to reconstruction and analysis of the WBM reconstructions and derived models. The generic genome‐scale reconstruction of human metabolism, Recon3D (Brunk *et al*, 2018), containing 13,543 metabolic reactions and 4,140 unique metabolites, was used to derive a stoichiometrically and flux‐consistent submodel, named Recon3*, to serve as a starting point for the construction of the WBM reconstructions. During the reconstruction process, we reviewed over 600 publications and included numerous omics data sets. myHarvey refers to the microbiome‐associated WBM models. See Materials and Methods and Dataset EV2 for more details.Schematic overview of the included organs and their anatomical connections in the male WBM reconstruction. The arrows indicate the organ exchange with the biofluid compartments.Statistics of the content of the WBM reconstructions.Stoichiometric matrix of the male reconstruction is shown with all non‐zero entries highlighted. The columns of the stoichiometric matrix represent the reactions, while the rows correspond to the metabolites. If a metabolite (j) participates in a reaction (i), the stoichiometric coefficient is entered in the corresponding cell (i, j). Each row represents the mass‐balance equation for a given metabolite (dx/dt). In flux balance analysis (FBA) (Orth *et al*, 2010), the modeled system is assumed to be at a steady state (i.e., dx/dt = 0). This mathematical problem can be efficiently solved at a large‐scale (Materials and Methods, Conversion from reconstruction to condition‐specific models).

To address these challenges, we developed the first two molecular‐level, organ‐resolved, physiologically consistent, sex‐specific, genome‐scale reconstruction of whole‐body metabolism (WBM; Fig 1A). We demonstrate that these WBM reconstructions can be converted into personalized WBM models by integration with physiological, quantitative metabolomics, and microbiome data, thereby allowing, e.g., the assessment of microbial metabolism on host metabolism in an organ‐resolved, person‐dependent manner.

## Results

### A novel approach yields curated whole‐body metabolic reconstructions

We developed a novel, iterative approach to assemble WBM reconstructions by leveraging an existing generic human metabolic reconstruction, Recon3D (Brunk *et al*, 2018), reconstruction algorithms (Vlassis *et al*, 2014), omics data (Wishart *et al*, 2013; Kim *et al*, 2014; Uhlen *et al*, 2015), and manual curation of more than 600 scientific literature articles and books (Fig 1A). The female WBM, termed Harvetta 1.0, was generated by starting with a meta‐reconstruction, composed of a set of 30 identical Recon3D models connected through anatomically consistent biofluid compartments (Fig 1B, Materials and Methods, Reconstruction details). Similarly, the male WBM, termed Harvey 1.0, started from a meta‐reconstruction with one copy of Recon3D for each of its 28 organs, tissues, and cell types (Table 1). Note that we will refer in the following to all cell types, tissues, and organs collectively as organs. We extensively reviewed organ‐specific literature for genes, reactions, and pathways known to be present in the different organs (Materials and Methods, Reconstruction details, Table EV1). This review effort yielded 9,258 organ‐specific reactions, which we added to a *core reaction set*, defining reactions that had to be present in the WBM reconstructions. We removed 912 organ‐specific reactions reported to be absent in the literature (Materials and Methods, Reconstruction details, Table EV1). Moreover, evidence for the presence of organ‐specific proteins for all organs except the small intestine was obtained from the human proteome map (Kim *et al*, 2014) and the human proteome atlas (Uhlen *et al*, 2015) (Materials and Methods, Reconstruction details, Tables EV2 and EV3). For four organs, metabolic reconstructions have been published, namely the red blood cell (Bordbar *et al*, 2011b), adipocyte (Bordbar *et al*, 2011a), small intestine (Sahoo & Thiele, 2013), and liver (Gille *et al*, 2010). Thus, we added the respective organ‐specific reactions to the core reaction set, after mapping the reaction identifiers to the Virtual Metabolic Human (VMH, http://www.vmh.life) reaction identifiers (Materials and Methods, Reconstruction details). The literature was also reviewed for the absence of cellular organelles in the different organs (Materials and Methods, Reconstruction details, Table EV4), which were removed accordingly from the relevant organs in the meta‐reconstructions. As a next step, we added transport reactions to the core reaction set, based on organ‐specific transporter expression data collected from the literature (Materials and Methods, Reconstruction details, Table EV5; Sahoo *et al*, 2014), and organ‐specific sink reactions for metabolites known to be stored in different organs (Materials and Methods, Reconstruction details, Tables EV6 and EV7). We used metabolomics data from 16 different resources to enforce the presence of metabolites detected in the different biofluid compartments (Fig 1, Materials and Methods, Reconstruction details, Table EV8), and added the corresponding reactions to the core reaction set. Furthermore, we used literature information to define metabolites that are known to cross, or not, the blood–brain barrier and either added them to the core reaction set or removed them from the meta‐reconstructions, respectively (Materials and Methods, Reconstruction details, Table EV9). Additionally, we included the dietary uptake reactions to the core reaction set for metabolites that have been identified in food (Materials and Methods, Reconstruction details, Table EV10). Finally, to enable the integration of the gut microbiome with the WBM reconstructions (see below), we added sink reactions to the core reaction set for metabolites known to be produced by human gut microbes (Table EV11). Each organ contains a biomass maintenance reaction representing the macromolecular precursors (e.g., amino acids) required for organ maintenance (Materials and Methods, Reconstruction details). To represent the energy required to maintain the body's cellular function and integrity, we added a whole‐body maintenance reaction to both meta‐reconstructions, in which each organ biomass maintenance reaction is weighted based on its respective organ weight in the reference man or woman (Snyder *et al*, 1975) (Materials and Methods, Reconstruction details).

**Table 1:** List of organs present in the male (Harvey) and female (Harvetta) WBM reconstructions

Organ name	Organ abbreviations	Present in WBM reconstruction
Adipose tissue	Adipocytes_	Harvey, Harvetta
Adrenal gland	Agland_	Harvey, Harvetta
B‐cells	Bcells_	Harvey, Harvetta
Brain	Brain_	Harvey, Harvetta
Breast	Breast_	Harvetta
CD4^+^ T‐cells	CD4Tcells_	Harvey, Harvetta
Cervix	Cervix_	Harvetta
Colon	Colon_	Harvey, Harvetta
Gallbladder	Gall_	Harvey, Harvetta
Heart	Heart_	Harvey, Harvetta
Kidney	Kidney_	Harvey, Harvetta
Liver	Liver_	Harvey, Harvetta
Lung	Lung_	Harvey, Harvetta
Monocytes	Monocyte_	Harvey, Harvetta
Muscle	Muscle_	Harvey, Harvetta
Natural killer cells	Nkcells_	Harvey, Harvetta
Ovary	Ovary_	Harvetta
Pancreas	Pancreas_	Harvey, Harvetta
Platelet	Platelet_	Harvey, Harvetta
Prostate	Prostate_	Harvey
Parathyroid gland	Pthyroidgland_	Harvey, Harvetta
Red blood cell	RBC_	Harvey, Harvetta
Retina	Retina_	Harvey, Harvetta
Spinal cord	Scord_	Harvey, Harvetta
Small intestine	sIEC_	Harvey, Harvetta
Skin	Skin_	Harvey, Harvetta
Spleen	Spleen_	Harvey, Harvetta
Stomach	Stomach_	Harvey, Harvetta
Testis	Testis_	Harvey
Thyroid gland	Thyroidgland_	Harvey, Harvetta
Urinary bladder	Urinarybladder_	Harvey, Harvetta
Uterus	Uterus_	Harvetta

The collected information, in the form of the core reaction set, and the tailored meta‐reconstructions were used as inputs for a model extraction algorithm (Vlassis *et al*, 2014) to generate sex‐specific draft WBM reconstructions (Fig 1A, Materials and Methods, Reconstruction details). This network extraction algorithm returns a compact subnetwork containing all core reactions and a minimal number of additional reactions necessary to render the subnetwork flux consistent, i.e., each network reaction can carry a non‐zero flux value. As the algorithm adds a minimal number of reactions to the subnetwork, it should be evaluated whether such additions can be supported or rejected based on biological evidence. A similar approach has been suggested for gap filling (Rolfsson *et al*, 2011). Consequently, we revisited the literature for evidence to support reaction inclusion and either expanded the organ‐specific core reaction set or removed organ‐specific reactions from the meta‐reconstructions as appropriate (Fig 1A). We then generated new draft WBM reconstructions using the model extraction algorithm with this updated input. This cycle was iterated over a hundred times, focusing on different organs and metabolic pathways each time, which was necessary due to the large size of the reconstructions (Materials and Methods, Reconstruction details). Throughout the reconstruction process, we followed an established quality control and quality assurance protocol (Thiele & Palsson, 2010) and performed core tests proposed by the systems biology community (preprint: Lieven *et al*, 2018).

The final sex‐specific WBM reconstructions account for 83,521 and 81,094 reactions for Harvetta and Harvey, respectively (Fig 1C, Tables EV12 and EV13). Harvetta contained more reactions than Harvey due to two more sex‐specific organs (Fig 1C and D). In total, 73,215 reactions were shared between both sexes, while 7,879 were unique to Harvey and 10,306 unique to Harvetta. Excluding sex organs, < 4% of all WBM reactions were sex‐specific, mostly involving alternative transport reactions (Dataset EV2: 3.6). Overall, these WBM reconstructions account for about 84% of the body weight. The remaining 16% correspond mostly to bones and connective tissue (Snyder *et al*, 1975; Fig 1C), both of which are as yet insufficiently represented with corresponding reactions in Recon3D. The resulting, sex‐specific WBM reconstructions comprehensively capture human whole‐body metabolism consistent with current knowledge.

### An organ compendium was derived from the WBM reconstructions

**Figure 2:**
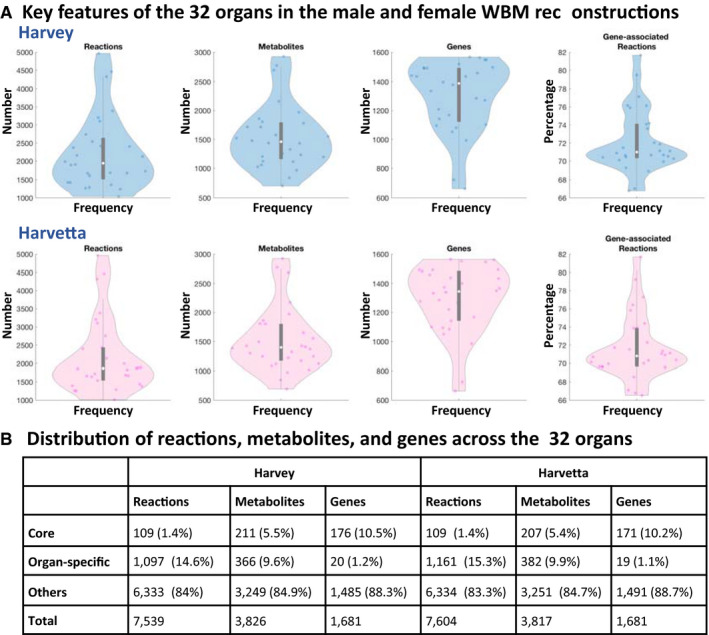
The organ compendium contains sex‐specific metabolic reconstructions Distribution of content in the male (blue) and female (pink) organ‐specific reconstructions.Overall statistics of the organ compendium. Core reactions, genes, and metabolites refer to those that are shared between all organs in Harvetta or Harvey. The category “other” refers to all reactions, genes, or metabolites that appear in more than two but < 30 (Harvetta) or 28 (Harvey) organs (Dataset EV2: 3.7).

A compendium of organ‐specific reconstructions was extracted from the WBM reconstructions (Figs 1B and 2, Tables 1 and EV14). All organ‐specific reconstructions can be obtained from the VMH at http://www.vmh.life. The technical quality of each organ was assessed using a comprehensive test suite, implemented in the COBRA Toolbox (Heirendt *et al*, 2019), that checks for adherence to quality standards developed for metabolic reconstructions (Thiele & Palsson, 2010; Brunk *et al*, 2018; preprint: Lieven *et al*, 2018). These tests included, e.g., testing for realistic ATP yields on various carbon sources under aerobic and anaerobic conditions or the ability to perform defined metabolic tasks (Dataset EV2: 3.7). A typical organ contained 2,947 ± 1,980 reactions, 2,076 ± 1,081 metabolites, and 1,298 ± 245 genes (average ± SD, Fig 2A). As one would expect, the organs with the most reactions were liver, kidney, and colon (Fig 1D). On average, 72% of reactions in each organ were gene‐associated, when excluding transport and exchange reactions (Fig 2A). About 10% of all metabolites were organ‐specific metabolites (Fig 2B), which may be used as biomarker metabolites for organ dysfunction. Notably, an additional 330 metabolites could only be found in two organs, potentially further expanding the set of potential organ dysfunction markers. Only 176 (10%) of the genes were core genes, which can be explained by the absence of the mitochondrially localized gene products from the core set, as the RBCs are lacking this organelle. When excluding the RBCs, an additional 142 genes were present in all remaining organs. The organ compendium represents a comprehensive set of manually curated, self‐consistent organ‐specific reconstructions that may be used for a range of biomedical applications (Aurich & Thiele, 2016; Nielsen, 2017).

### Combining metabolism with physiology enables physiologically constrained, stoichiometric modeling

**Figure 3:**
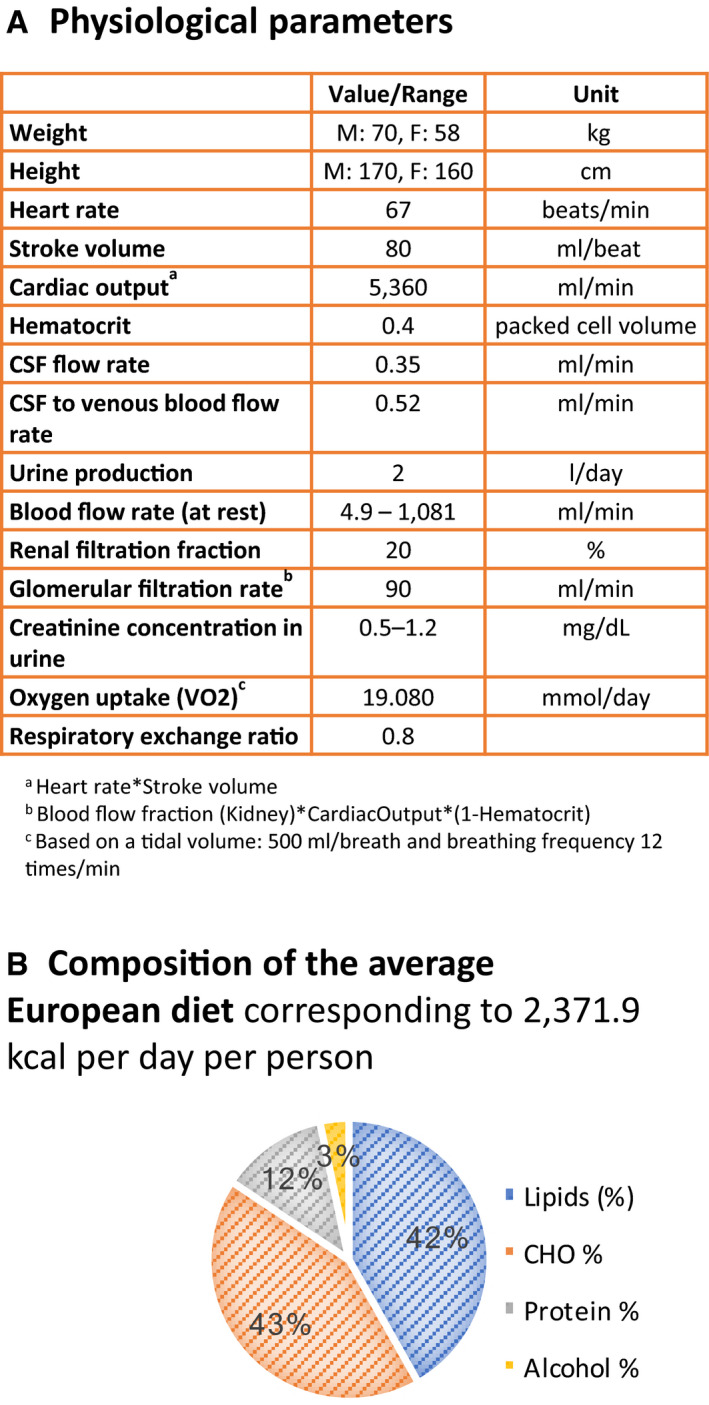
Combining metabolism with physiology enabled physiologically constrained, stoichiometric modeling List of physiological parameters used to constrain the reactions. The physiological values were retrieved for a reference man and woman (Snyder *et al*, 1975). A complete list of simulation constraints can be found in Materials and Methods (Conversion from reconstruction to condition‐specific models) and Dataset EV2: 3.2.Nutritional composition of the average European diet.

The conversion of a reconstruction into a model involves the imposition of condition‐specific constraints (Orth *et al*, 2010). Each WBM reconstruction was constrained with 15 physiological parameters (Fig 3A) and metabolomics data (Wishart *et al*, 2013; Materials and Methods, Conversion from reconstruction to condition‐specific models, Dataset EV2: 3.2). For instance, metabolite transport across the capillaries of well‐perfused organs is bulk flow limited rather than diffusion‐limited (Feher, 2012). Using organ‐specific blood flow rates at rest (Price *et al*, 2003) and plasma metabolite concentrations (Wishart *et al*, 2013), we placed upper bounds on the corresponding organ‐specific metabolite transport reactions. Hence, by combining physiological and metabolomic constraints, we defined how much of each metabolite can be maximally taken up by an organ in the WBM, thereby addressing the challenge of appropriately constraining organ‐specific models. Additionally, we assumed that the kidney filters 20% of blood plasma along with all the metabolites, irrespective of their nature (Martini & Bartholomew, 2006). Accordingly, multiplying the glomerular filtration rate by healthy plasma metabolite concentration ranges (Wishart *et al*, 2013) allowed us to set lower and upper bounds for filtration of each metabolite by the kidney. Similarly, we assumed that cerebrospinal fluid (CSF) metabolites are unselectively returned into the bloodstream, permitting us to use healthy CSF metabolite concentration (Wishart *et al*, 2013) as constraints on the rate at which CSF metabolites enter the bloodstream. Using the daily urine excretion rate and urine metabolite concentrations (Wishart *et al*, 2013), we constrained the rate of urinary metabolite excretion (Fig 3A). We also applied constraints corresponding to an average European diet (Fig 3B; Elmadfa, 2012). All applied constraints are described in detail in the Dataset EV2: 3.2.1. In total, 12.5% of the WBM model reactions had a constraint placed on their bounds, leading to a substantially reduced steady‐state solution flux space (Appendix Fig S1A, Table EV12). We will refer to this novel paradigm in constraint‐based modeling as physiologically and stoichiometrically constrained modeling (PSCM) and provide a PSCM toolbox (http://www.opencobra.github.io/cobratoolbox) as well as a MATLAB (Mathworks, Inc.) Live Script (Dataset EV2, https://opencobra.github.io/cobratoolbox/stable/) enabling the reproducibility of all presented simulations.

### The physiologically constrained whole‐body metabolic models can predict organ metabolic essentiality, known biomarkers of inherited metabolic diseases, and inter‐organ metabolic cycles

**Figure 4:**
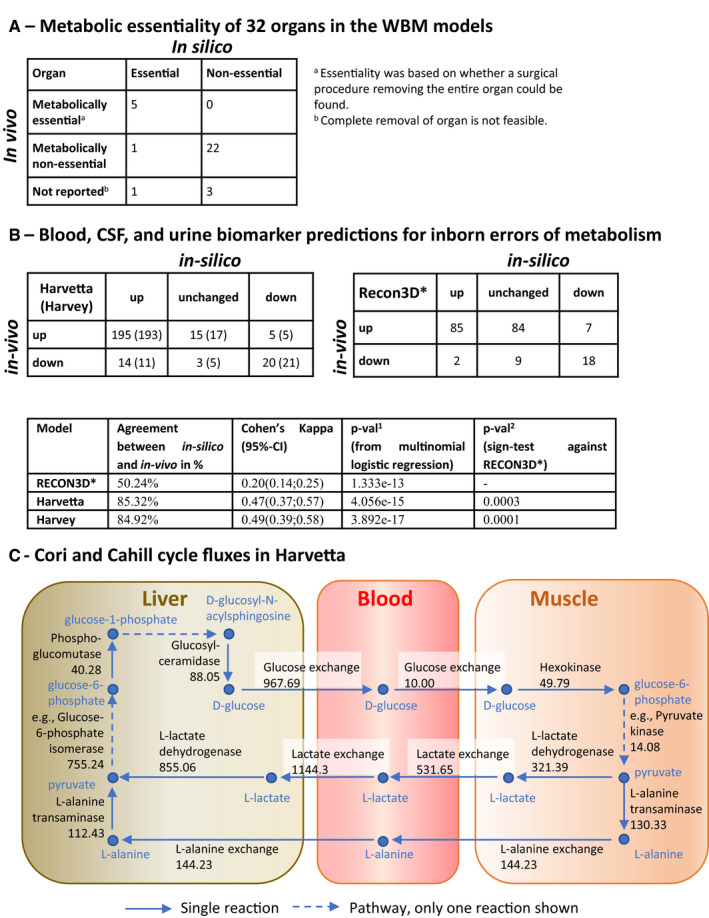
Assessment of the predictive potential of the WBM model Comparison of predicted and *in vivo* organ metabolic essentiality (Table EV15).Statistical agreement between *in silico* and *in vivo* predictions for reported biomarkers of 57 inborn errors of metabolism using the WBM models and a Recon3D model (Table EV16).Cori and Cahill cycle fluxes involving liver and muscles (in mmol/day/person). Key reactions from a single flux distribution for the female WBM model, obtained by minimizing the Euclidian norm, subject to mass‐balance and physiological constraints. Only a subset of the active intra‐organ pathways is shown; hence, the values of influx and efflux to a metabolite are not equal in the figure. Please refer to Dataset EV2: 3.3.2.2 or vmh.life for reaction details.

To assess the predictive potential of the WBM models against current knowledge, we computed the metabolic essentiality of an organ by maximizing a whole‐body maintenance reaction, without the corresponding organ biomass contribution, under the aforementioned physiological and dietary constraints. We predicted an organ to be metabolically non‐essential if a non‐zero whole‐body maintenance reaction flux was possible in a WBM model deficient in that organ (Dataset EV2: 3.5). We defined an organ to be metabolically non‐essential *in vivo* if it can be surgically removed or replaced by an artificial organ (e.g., heart; Table EV15). Of the 32 organs, for 28 we could find corresponding information and predicted the metabolic essentiality correctly in all but one case (Fig 4A). It is important to note that the WBM models can only assess metabolic essentiality but do not capture other essential organ functions (e.g., those of the brain).

To further assess the predictive capacity of the WBM models, we simulated 57 inborn errors of metabolism (IEMs) by deleting the corresponding reaction(s) in all organs having the known defective gene(s) (Dataset EV2: 3.8, Materials and Methods, Statistical analyses). We then predicted and compared for each IEM the known biomarkers in either blood, urine, or CSF. Across the 57 IEMs, a total of 215 out of 252 (85.3%) biofluid specific biomarkers were qualitatively predicted correctly by Harvetta and 214 out of 252 (84.9%) by Harvey (Fig 4B, Table EV16). We compared this with similar predictions using the Recon3D model, which has only one extracellular compartment. As expected, the number of biomarkers was smaller than could be predicted (205 metabolites). Notably, the predictive potential of the Recon3D model was lower than that of the WBM models, with only 103 biomarker metabolites predicted correctly (50.2%, Fig 4B). Overall, the biomarker predictions with Harvetta and Harvey showed higher agreement percentages, increased Cohen's kappa metrics, and significantly better agreement percentages per disease (*P* = 0.0003 and *P* = 0.0001 for Harvetta and Harvey, respectively; Fig 4B). The better prediction capabilities of the WBM models were mainly caused by having fewer inconclusive or unchanged predictions, meaning that those metabolites were not predicted to be biomarkers. While for the Recon3D model, 45.3% (93 of 205) of the predictions failed to predict any changes, only 7.1% (18 of 252) fell into this category for Harvetta and 8.7% (22 of 252) for Harvey. Hence, the ability to account for physiological and metabolic constraints as well as organ‐level separation of metabolic pathways enhanced the prediction capacity considerably and presented a key advantage of using a WBM model over the generic Recon3D model.

In the WBM models, we investigated the constraints sufficient for the activity of two inter‐organ metabolic cycles (Frayn, 2010), when minimizing the Euclidean norm of the flux vector, with the rate of the whole‐body maintenance reaction set to one (Dataset EV2: 3.3.2.2). In the Cori cycle, glucose is secreted by the liver and taken up by the muscle, which produces lactate, which is then taken up by the liver to produce glucose (Fig 4C). Elevated postprandial blood glucose concentration, mediated by insulin levels, determines the rate at which glucose is taken up *in vivo* (Wasserman *et al*, 2011), but the regulatory functions of insulin are beyond the scope of the present WBM models. Consistently, only enforcing muscle glucose uptake was sufficient to achieve activity of the Cori cycle in the WBM models, at rest (Dataset EV2: 3.3.2.2). In the Cahill cycle, glucose is secreted by the liver and taken up by the muscle, which converts it to alanine, which is taken up by the liver to produce glucose (Fig 4C). The Cahill cycle is increased during exercise, when muscles degrade amino acids for energy needs, as a means to remove ammonium from the body via urea production in the liver (Frayn, 2010). Consistently, enforcing muscle glucose uptake, and enforcing either liver alanine uptake or increasing urinary urea excretion was sufficient to achieve activity of the Cahill cycle in the WBM models (Dataset EV2: 3.3.2.2).

To demonstrate that the aforementioned results are a consequence of including reactions and constraints based on organ‐level data, we then tested the degree of functional redundancy in the WBM models. Therefore, we removed random subsets of reactions and checking the feasibility of a unit flux through the whole‐body maintenance reaction. We found that there was an exponential drop in the number of feasible models as the fraction of reactions removed was increased (Appendix Fig S1B). Specifically, 60% of the WBM models (600/1000) were feasible if 0.1% of reactions (81/81,094) were randomly removed and none of the models was feasible when 1% of the reactions were randomly removed.

This reliable prediction of organ and gene essentiality, as well as the feasibility of inter‐organ metabolic cycles, and the infeasibility of whole‐body maintenance upon random reaction removal, demonstrates that the topology of the WBM models is a specific and sensitive representation of whole‐body metabolism but that constraints are required to enforce condition‐dependent metabolic activity.

### The whole‐body metabolic models predict basal metabolic flux and activity‐induced energy expenditure

The basal metabolic rate (BMR) varies between individuals, depending on their age, sex, weight, height, and body composition. Phenomenological methods (Harris & Benedict, 1918; Mifflin *et al*, 1990) have been developed for estimating BMRs, but they do not account for differences in metabolism. Here, we investigated whether the WBM models, which incorporate metabolism and physiological constraints (Fig 3), could be used to predict BMRs with higher accuracy than the phenomenological methods. To this end, we assumed that BMR is equal to the energy yield from ATP hydrolysis (64 kJ; Wackerhage *et al*, 1998) times the resting rate of steady‐state ATP consumption. The latter was predicted by setting the whole‐body maintenance reaction flux to one, minimizing the Euclidean norm to obtain a unique steady‐state basal metabolic flux vector (Heirendt *et al*, 2019) and summing the rate of ATP consumption of all reactions in all organs (Dataset EV2: 3.3). The whole‐body maintenance reaction represents the material and energy (ATP) required to maintain the non‐metabolic cellular functions of the body and was constructed based on the fractional weight contribution for each organ for a reference man or woman (Snyder *et al*, 1975) (Dataset EV2: 3.2). In the basal metabolic flux vector, ATP consumption by the whole‐body maintenance reaction is < 2% of the total ATP consumption. Without any fitting, or accounting for age, we predicted overall energy consumption rates of 1,344 kcal for Harvetta and 1,455 kcal for Harvey, which were consistent with the expected values for a reference man and woman, respectively.

**Figure 5:**
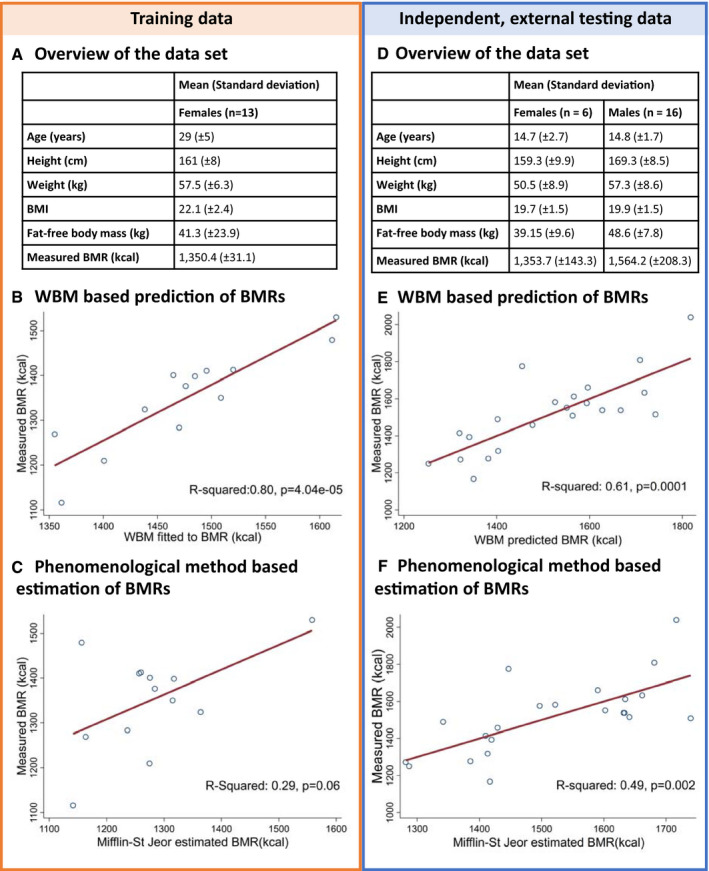
Application of the WBM models to predict basal metabolic rates (BMRs) A‐CA training data set (Prentice *et al*, 1986) was used to identify parameters to improve the prediction of the BMRs (A). Therefore, the WBM models were first constrained based on age, height, and weight (Dataset EV2: 3.9, Table EV17). We then adjusted fat‐free mass and the ATP requirements in the muscle, to obtain a better fit of the predicted and measured BMRs (B). For comparison, we estimated the BMRs using a phenomenological method, being the Mifflin‐St. Jeor equations, and compared the estimates with the measurements (C).D–FSubsequently, we validated the identified tuning parameters, being adjusted fat‐free mass and the ATP requirements in the muscle, using an independent, external data set (D) (Loureiro *et al*, 2015). The WBM model based predicted BMRs for the independent data set (E) outperformed the phenomenological method (F).

Age, weight, and height from 13 healthy women with normal body mass index (BMI < 22.1 ± 2.4) (Prentice *et al*, 1986) (Fig 5A, Dataset EV2: 3.9, Table EV17) were used, together with established polynomial functions (Dataset EV2: 3.10.1.5; Young *et al*, 2009), to fix the physiological parameters (organ weights, blood flow rate, and cardiac output) of a set of personalized Harvetta models. We then tuned Harvetta to fit corresponding personalized BMR measurements by performing a sensitivity analysis (Dataset EV2: 3.9.1) thereby identifying parameters in Harvetta that could be adjusted to fit the BMR data better. As the fat‐free body mass was provided for each individual, we adjusted the muscle and adipocyte coefficients in the whole‐body maintenance reactions, instead of solely relying on the estimated values based on age, weight, and height (Young *et al*, 2009). We also increased the ATP coefficient in the muscle biomass maintenance reaction, starting from a previously estimated coefficient (Bordbar *et al*, 2011a), to better reflect the energetic requirements associated with protein turnover in the muscle. After optimizing the ATP coefficient, the WBM model was able to predict the measured BMR with high accuracy (explained variance = 79.7%, F(1, 11) = 43.14, *P* = 4.04e‐05, Fig 5B) and better than the Mifflin‐St Jeor equations (Fig 5C). However, as parameter tuning was involved, potentially leading to overfitting, we validated the WBM BMR prediction models with an independent data set not utilized in parameter estimation.

An independent, external data set, composed of age, weight, height, fat‐free body mass, and BMR data from six female and 16 male athletes (Loureiro *et al*, 2015) (Fig 5D) was used to assess the ability of the previously tuned WBM models to predict BMR (Dataset EV2: 3.9.2, Table EV17). Good correlation was obtained between predicted and measured BMR (explained variance = 61.3%, *F*(2, 19) = 15.06, *P* = 1.10e‐4) (Fig 5E). In comparison, the Mifflin‐St Jeor equations, a standard phenomenological method of estimating the BMR from age, sex, height, and weight (Mifflin *et al*, 1990), gave a lower correlation (*R* ^2^ = 0.49, *P* = 0.002) (Fig 5F). Furthermore, given the WBM models, the Mifflin‐St Jeor equations were not informative for the true BMR (linear regression *b* = 0.01, 95%‐CI:(−0.99, 1.00), *P *= 0.90). In contrast, the WBM prediction added predictive value to the Mifflin‐St Jeor equation (linear regression *b* = 2.24, 95%‐CI: (0.32; 4.16), *P* = 0.025). The last result implies that the WBM models are significantly superior to the Mifflin‐St Jeor equations in terms of prediction accuracy. Taken together, the results demonstrate that the WBM models provide good and replicable estimations of BMRs that improve on standard phenomenological methods. However, the analyses also highlight the need for personalized parameterization of the WBM models for a given simulation and that the use of further parameters is likely to improve the prediction fit. Equally, confounding factors, beyond age and sex, should be considered and validation in a larger cohort will be required.

Beyond the basal metabolic rate, activity‐induced energy expenditure consumes the remaining energy and nutrient resources provided by the diet (Biesalski & Grimm, 2005). With the aforementioned basal metabolic flux distribution (Dataset EV2: 3.3.2.3), the resting brain energy consumption rate was initially predicted to be 10.5 mole ATP/female/day (Dataset EV2: 3.3.2.3). Assuming a conversion of 1 mole of glucose into 31 moles of ATP, this value is lower than a total brain consumption rate of 20.65 mole ATP/day/person, based on the reported consumption rate of 120 g of glucose per day (Berg *et al*, 2002). To achieve a 20 mole ATP/female/day total brain consumption rate, we had to set a requirement for the brain to hydrolyze 3.5 mole ATP/female/day for electrophysiological processes (VMH ID: Brain_DM_atp_c_). Note that this demand for hydrolysis of ATP requires further hydrolysis of ATP by supporting metabolic pathways elsewhere in the brain. Similarly, the pumping of blood through the body by the heart requires the hydrolysis of ATP. However, literature reports differ in their estimated energy requirements with one source reporting 6,000 g ATP/day/person to be consumed (https://heartmdinstitute.com/heart-health/metabolic-cardiology-basics/), which corresponds to 11.8 mole ATP/day/person with the molecular weight of ATP being 507.18 g/mole (Dataset EV2: 3.2.2.7). A lower bound on a heart demand for hydrolysis of ATP (VMH ID: Heart_DM_atp_c_) was set to 6 mole ATP/day/person to account for the energy required for pumping blood through the body while allowing the remained to be consumed by supporting heart metabolic reactions. Despite the lack of comparable measurements of activity‐induced ATP consumption rates, we set these parameters on brain and heart ATP hydrolysis because each organ contributes to the overall energy consumption in a whole‐body model. After accounting for whole‐body maintenance and energy demand due to brain and heart activity, the basal metabolic flux distribution predicted that the remaining energy that the WBM models could extract from the dietary input was 515.7 kcal for Harvetta and 502.5 kcal for Harvey. These values correspond to approximately 20% of the average European dietary input (2,372 kcal) used for the WBM simulations. This result is consistent with a 15–30% allocation of dietary input toward physical activity (Biesalski & Grimm, 2005).

Overall, we demonstrated that WBM model predictions of basal metabolism and energy requirements are overall consistent with the literature and they provide an opportunity for integration of both physiological and molecular biological data to enable enhanced predictions when compared with purely phenomenological methods.

### Personalized microbiome‐associated WBM models to simulate gut–organ co‐metabolism

The human gut microbiota influences host organ metabolism, including the brain (“gut‐brain axis”), the liver (“gut‐liver‐axis”), and the colon (Clarke *et al*, 2014). However, our understanding of host–microbiome co‐metabolism remains limited. The WBM models can be used to predict how the microbiome may modulate human metabolism, thereby complementing existing *in vitro* and *in vivo* analysis approaches.

**Figure 6:**
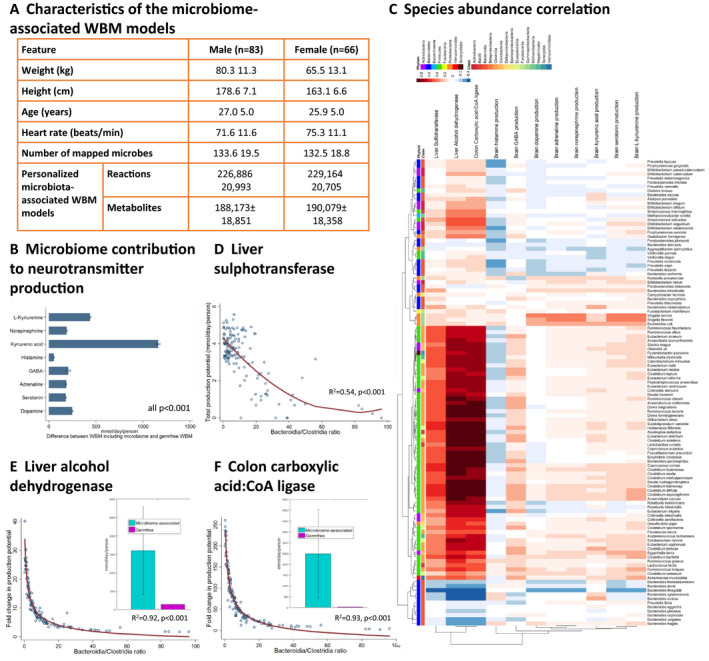
Application of the 149 personalized microbiome‐associated (my) WBM models to predict host–microbiome co‐metabolism ACharacteristics of the myWBM models.BAverage brain biosynthesis potential for eight neurotransmitters in the personalized WBM models displayed as the difference between with and without microbiome (germ‐free). *P*‐values from paired *t*‐tests.CSpearman correlations between species‐level abundances and fold changes in fluxes (microbiome‐associated vs. germ‐free) for flux through 11 objective functions. Each data point is the Spearman correlation between 149 changes in fluxes for one reaction and 149 abundances for one species.D–FBacteroidia/Clostridia ratio against maximal flux (mmol/day/person) against (relative abundance) through the different reactions. Regression line from fractional polynomial regression and p‐values from likelihood ratio tests. Inlet: Average maximal reaction flux in microbiome‐associated and germ‐free WBM models.

To constrain Harvetta and Harvey in a gut microbial context, we used published shotgun metagenomics and clinical data on 149 healthy individuals provided by the Human Microbiome Project (HMP) Consortium (Consortium, 2012a; Peterson *et al,* 2009) (Dataset EV2: 3.10, Fig 6A). First, we generated personalized germ‐free WBM models using clinical data on sex, weight, height, and heart rate of each individual. Second, we mapped the reported strain‐level abundances for each individual onto a resource of 773 gut microbial metabolic reconstructions (Magnusdottir *et al*, 2017; Dataset EV2: 3.10.1). The resulting 149 personalized microbial community models captured 131 ± 19 microbes per HMP individual, accounting for 91 ± 7% of the reported relative abundance (Appendix Fig S2). Third**,** the microbial community models were added to the large‐intestinal lumen of the corresponding personalized WBM models, resulting in personalized microbiome‐associated (my) WBM models. In the large‐intestinal lumen of these personalized myWBM models, gut microbes can take up dietary metabolites after they transit from the small intestinal lumen where small intestinal enterocytes may also absorb them. Metabolites produced by gut microbes can be taken up by colonocytes or excreted via the feces.

The human gut microbiome is a source of amino acids and neuroactive compounds (Clarke *et al*, 2014; Lin *et al*, 2017). Hence, we investigated whether the presence of the gut microbiota in personalized myWBM models could increase the maximal possible production rate of eight prominent neurotransmitters. We first maximized the neurotransmitter production in the brain in the personalized germ‐free WBM models and observed substantial inter‐individual variation (Fig 6B). This variation could be attributed to the changed bounds on the organ uptake reactions due to the personalization of the physiological parameters. We then used the personalized myWBM and observed substantial increase in the brain neurotransmitter production capability. No clear correlations between brain neurotransmitter synthesis potential and individual microbial species were observed (Fig 6C). The high inter‐individual variation in these metabolic objectives could neither be explained by the presence of a single species or genus (Fig 6B and C) nor by the provided meta‐data. Consequently, we hypothesized that the simulation results were the direct consequence of the host–gut microbial co‐metabolism and could not be derived from the metagenomic data alone. Using the 149 personalized microbiome models without the WBM, we calculated the neurotransmitters and neurotransmitter precursors predicted to be secreted by each strain (Appendix Fig S3). For instance, GABA was predicted to be produced by *Akkermansia muciniphila* and strains of the *Bacteroides*,* Escherichia*, and *Shigella* genera. Consistently, GABA‐producing capability has been shown for *Bacteroides*,* Parabacteroides,* and *Escherichia* species (Strandwitz *et al*, 2019). Glutamate, the precursor of GABA and itself a neurotransmitter, was predicted to be mainly produced by *Eubacterium rectale*,* Faecalibacterium*,* Roseburia*, and *Bacteroides*. Histamine was predicted to be produced mainly by Gammaproteobacteria species and *Streptococcus* sp., in agreement with reports that members of these taxa synthesize histamine (Clarke *et al*, 2014). Histidine, the precursor of histamine, was predicted to be produced mainly by commensals of the *Bifidobacterium* genus and of the Clostridiales order (e.g., *Faecalibacterium*,* Roseburia*). *Alistipes* and *Bacteroides* sp. were predicted to produce the majority of L‐tryptophan, a precursor of serotonin. Finally, L‐tyrosine, a precursor of dopamine, was predicted to be produced by a variety of species belonging to the Actinobacteria, Firmicutes, and Proteobacteria phyla. Taken together, the modeling results predicted that gut microbes have the molecular mechanisms that may influence host brain metabolism directly by synthesizing neurotransmitters and indirectly by providing the amino acid precursors to be further metabolized by human enzymes. The predicted maximum secretion potential, as well as the contributing strains, depended on each individual's microbiome composition. Thus, the WBM models can be used to generate novel mechanistic hypothesis as to how the gut microbiota might influence brain metabolism (Clarke *et al*, 2014; Lin *et al*, 2017).

Ethanol causes liver toxicity as its product, acetaldehyde, is cytotoxic and carcinogenic (Neuman *et al*, 2017). When maximizing the flux through the liver alcohol dehydrogenase (VMH ID: ALCD2if), the myWBM models predict an 11.4 ± 8.5 fold increase in maximal potential flux compared with corresponding personalized germ‐free WBM models (Fig 6E). This fold change in flux strongly correlated with species belonging to the Clostridia class and negatively correlated with representatives of the Bacteroidia class (Fig 6E). Consequently, the Bacteroidia/Clostridia ratio determined 92.1% of the variation in the predicted maximum alcohol dehydrogenase flux (*F*(2, 144) = 852.21, *P* = 1.56e‐80, see Table EV18). Species that affected the most the alcohol dehydrogenase flux included *Clostridium scindens*,* Blautia hydrogenotrophica*, and the well‐known pathobiont *Clostridium difficile* (Fig 6C). Endogenous ethanol production by the microbiota has been shown in non‐alcoholic steatohepatitis (NASH) patients (Zhu *et al*, 2013). Further support that microbial endogenous ethanol production can perturb liver function and cause non‐alcoholic fatty liver disease has been recently published (Yuan *et al*, 2019).

Microbially produced butyrate serves as the main energy source for the colonocyte (Donohoe *et al*, 2011). We computed the maximum flux through the butyrate‐CoA ligase in the colonocytes in the personalized myWBM models. The predicted maximum butyrate‐CoA ligase flux was on average 78.7 ± 64.7‐fold increased in the presence of the microbiome (Fig 6F) and strongly correlated with the abundance of known butyrate producers, such as *Faecalibacterium prausnitzii*,* Butyrivibrio crossotus*, and *Subdoligranulum variabile* (Louis & Flint, 2009). The Bacteroidia/Clostridia ratio determined 92.9% of the variance in the predicted maximum butyrate‐CoA ligase flux (*F*(2, 144) = 953.32, *P* = 8.8e‐84, Table EV18). Consistently, it is known that butyrate is mainly produced by microbes belonging to the Clostridia class (Louis & Flint, 2009).

The human gut microbiota can also influence drug metabolism (Spanogiannopoulos *et al*, 2016). Flux balance analysis was used to predict the maximal possible flux through the liver sulphotransferase reaction (VMH ID: PCSF) in each myWBM model (Fig 6D). myWBM models with low *Clostridioides* genus abundance were predicted to have lower maximum liver sulfonation flux (Fig 6D). The potential for liver sulfonation was inversely correlated with the abundance of the Bacteroidia class (Fig 6D), such that the Bacteroidia/Clostridia ratio contributed 53.6% of variance to maximal liver sulfonation rates (*F*(2, 144) = 85.37, *P* = 3.547e‐25, Table EV18). Note that, at higher *Clostridioides* genus abundances, substrate availability was limiting for p‐cresol production and subsequent sulfonation (Appendix Fig S4). Overall, consistent with our results, it is known that the production of p‐cresol, e.g., by *Clostridium difficile*, competes with drugs, such as acetaminophen, for sulfonation in the liver and interferes with drug detoxification (Spanogiannopoulos *et al*, 2016).

Taken together, using the personalized myWBM models, we demonstrate that this modeling framework can generate predictions of how microbial metabolism may modulate host metabolism. Importantly, some of these predictions have already been shown experimentally to be consistent with known host–microbe co‐metabolism. Thus, the myWBMs are suitable for the generation of novel, experimentally testable hypotheses on host‐microbe co‐metabolism, the development of microbe‐modulated biomarkers, and for the identification of individuals with increased risk of toxicity.

## Discussion

We presented two sex‐specific, organ‐resolved, molecular‐level, anatomically and physiologically consistent reconstructions of human whole‐body metabolism. The underlying reconstruction of human metabolic pathways (Brunk *et al*, 2018) has been developed over the past decade based on over 2,000 literature articles and books, and provided an indispensable foundation for the WBM reconstructions. Organ‐specific metabolism (Figs 1 and 2) was based on more than 600 published studies and books, and accounts for comprehensive proteomic and metabolomics data. Known inter‐organ metabolic interactions, as illustrated with the classical examples of the Cori and the Cahill cycles, as well as organ‐essentiality and inborn errors of metabolism (Fig 4) were captured with the WBM reconstructions, as were whole‐body functions, such as BMR and the energy use (Fig 5). Using the microbiome‐associated WBM models, we could show that individual microbes, or microbial communities, may be used to explore their potential influences on the metabolism of different host organs (Fig 6). Importantly, for some of these predictions, we could find supporting evidence in the literature that such host–microbe co‐metabolism does indeed occur. Finally, the personalized WBM models reflected inter‐individual variability in metabolism due to varied physiological parameters, which were broadly consistent with phenomenological observations. Taken together, the WBM reconstructions represent a molecular‐level description of organ‐specific processes built on current knowledge and underpinned by basic physicochemical principles.

The creation of organ‐specific metabolic reconstructions is challenging despite the myriad of omics data and sophisticated algorithms (Opdam *et al*, 2017). We tackled this challenge with an iterative approach combining extensive literature review, omics data, and high‐performance computing (Fig 1). Moreover, the inclusion of biofluid compartments in the WBM reconstructions enabled the integration of quantitative metabolomics data, while the use of microbiome information and dietary information increased the comprehensiveness of the WBM reconstructions. The interlinked metabolic complexity captured in the WBM reconstructions could not have been achieved by using an organ‐by‐organ reconstruction approach, as such an approach would not consider the inherent cooperation between the body's organs. Importantly, this novel whole‐body reconstruction paradigm may present a blueprint for other multi‐cellular model organisms, such as the virtual physiological rat (Beard *et al*, 2012).

In addition to our genome and diet, our (gut) microbiome contributes to inter‐individual variation in disease development and progression (Sonnenburg & Backhed, 2016). Computational models aimed at accounting for these factors have been, in part, hampered by the lack of a molecular‐level, organ‐resolved description of human metabolism. While the gut microbiota can modulate human metabolism on an organ level (Zhu *et al*, 2013; Yuan *et al*, 2019), the underlying pathways are typically elucidated using animal models (Claus *et al*, 2008; Sampson *et al*, 2016; Virtue *et al*, 2019). The WBM models, when associated with microbiomes, enable such analysis *in silico* and a comparison with their germ‐free counterpart. This capability facilitates the formulation of novel, mechanistic hypotheses on how gut microbes, individually and collectively, may modulate human metabolism, and vice versa. While these hypotheses require experimental validation, the WBM models permit prioritization of experimental studies, thus accelerating knowledge creation through a systems biology approach.

One may question whether it is enough to use the germ‐free WBM models, considering the impact of the microbiome on human metabolism, as also illustrated with our examples (Fig 6). We argue that using the germ‐free WBM models without the gut microbiome is valuable and valid, as we applied measured blood metabolite concentration ranges as constraints for each organ. These metabolite concentration ranges have been obtained from, e.g., the Human Metabolome Database (Wishart *et al*, 2013). These ranges are assumed to represent the concerted metabolic activity of host and microbial metabolism. Nonetheless, we believe that the inclusion of the microbiome adds another dimension to the possible applications of the WBM models. While, in this study, we only demonstrated the inclusion of the human gut microbiome, microbiomes from other body sites (Costello *et al*, 2009; Consortium THMP, 2012a; Pasolli *et al*, 2019), such as the small intestine, the vagina, the skin, or the oral cavities, should eventually be also considered.

The WBM reconstructions represent anatomically consistent topological connections between organs. Current approaches have either assumed a whole‐body human metabolic network without organ boundaries (Aurich & Thiele, 2016; Nilsson *et al*, 2017) or simplified it (Bordbar *et al*, 2011a), which ultimately limits its general usability and predictive capacity. Here, in contrast, we considered individual‐level physiological, nutritional, and microbial parameters for personalization of the WBM models and provide a novel tool to study inter‐individual variability in physiological processes as well as drug metabolism (Thiele *et al*, 2017). Multi‐omic data for healthy and diseased individuals are becoming increasingly available (Price *et al*, 2017), requiring novel tools for the integrative analysis of such diverse data. The omics data also provide a unique opportunity to further constrain and personalize the WBM models. Such personalization of computational human models is also a requirement for enabling *in silico* clinical trials (Viceconti *et al*, 2016).

The presented WBM reconstructions describe the metabolism of a large number of organs in the human body but are not yet resolved to the tissue or cellular level. For instance, the kidney and the liver are anatomically complex organs, consisting of many different cell types with distinct metabolic functions. The WBM reconstructions will need to be expanded to include cell‐specific metabolism within the individual organs, which will be enabled through advances in single‐cell omics technologies (Han *et al*, 2018). Another limitation is that our WBM reconstructions capture only metabolism, ignoring important processes, such as signal transduction and regulation, immune response, and the action of hormones. For some of these processes, computational models have been already constructed (Chelliah *et al*, 2015). Using metabolic models as a framework for integration of other types of models has been already demonstrated, most notably in a model of *Mycoplasma genitalium* (Karr *et al*, 2012).

This new physiologically and stoichiometrically constrained modeling (PSCM) approach allows the integration of physiological parameters and quantitative metabolomics data to restrict organ uptake rates to physiologically relevant values and thereby limit the achievable intra‐organ metabolic flux states. Our computational approach is tractable, as it relies either on solutions to linear or quadratic optimization problems. In contrast, hybrid modeling approaches, such as the ones integrating physiology‐based pharmacokinetic modeling with genome‐scale models (Eissing *et al*, 2011; Krauss *et al*, 2012; Guebila & Thiele, 2016), require a flux solution for a metabolic submodel at each time step. All processes in the human body are intrinsically dynamic, but many whole‐body metabolic questions can be addressed while assuming a steady state. In those cases, WBM modeling is a valuable, time‐efficient alternative to hybrid modeling as it represents human metabolism at a more comprehensive level, and yet it is computationally tractable.

Taken together, the WBM reconstructions and personalizable, versatile models of Harvetta and Harvey represent a significant step toward the “virtual human” envisioned in the Tokyo declaration (Kitano, 2010).

## Materials and Methods

### 1 Reconstruction details

In this part, we describe the reconstruction approach and the information used to generate the WBM reconstructions, Harvey and Harvetta. The conversion of the reconstructions into condition‐specific WBM metabolic models is then described Statistical analyses details can be found at the end of the Materials and Methods section. Simulation‐relevant information and methods can be found in the Dataset EV2 (or here: https://opencobra.github.io/cobratoolbox/stable/).

#### 1.1 The human metabolic reconstruction served as a starting point for the WBM reconstructions

As a starting point for the WBM reconstructions, we used the global human metabolic reconstruction, Recon3D (Brunk *et al*, 2018), which accounts comprehensively for transport and biochemical transformation reactions, known to occur in at least one cell type. Recon3D was assembled based on more than 2,000 literature sources. Recon3D was obtained from the Virtual Metabolic Human database (https://www.vmh.life/#downloadview, version 3.01). In Recon3D, many of the enzyme‐catalyzed reactions or transport reactions are associated with the corresponding gene(s) that encode the protein(s). These so‐called gene‐protein‐reaction associations (GPRs) are represented through Boolean rules (“AND”, “OR”) for isozymes or protein complexes, respectively. The reconstruction of Recon3D consists of 13,543 reactions, 4,140 unique metabolites, and 3,288 genes. The Recon3D publication also contained a flux and stoichiometrically consistent global metabolic model (Recon3D model) accounting for 10,600 reactions, 5,835 non‐unique metabolites (2,797 unique metabolites), and 1,882 unique genes. Hence, we used the Recon3D model, rather than Recon3D as starting point, as we wished the WBM reconstructions, and their derived models to be also flux and stoichiometrically consistent. From the Recon3D model, we removed from all reactions, and metabolites, involved in protein and drug metabolism, as these pathways were beyond the anticipated scope of the first version of the WBM reconstructions. We then removed flux inconsistent reactions (i.e., those reactions that did not admit non‐zero net flux) using the COBRA toolbox (Heirendt *et al*, 2019). The resulting metabolic flux and stoichiometrically consistent model, named Recon3D*, contained 8,418 reactions, 4,489 (non‐unique) metabolites, 2,053 transcripts, and 1,709 genes, and was used in the following to assemble the WBM reconstructions.

#### 1.2 Setup of the multi‐organ, sex‐specific meta‐reconstructions

The central idea of the novel reconstruction paradigm was to generate sex‐specific, organ‐resolved WBM reconstructions that were not built by connecting the separate organ‐specific metabolic reconstructions but which would emerge as one functional, self‐consistent whole‐body metabolic system from the metabolic capabilities and known interactions between the organs.

##### 1.2.1 Preparation of the organ‐specific metabolic reconstructions to be included in the meta‐reconstructions

**Figure 7:**
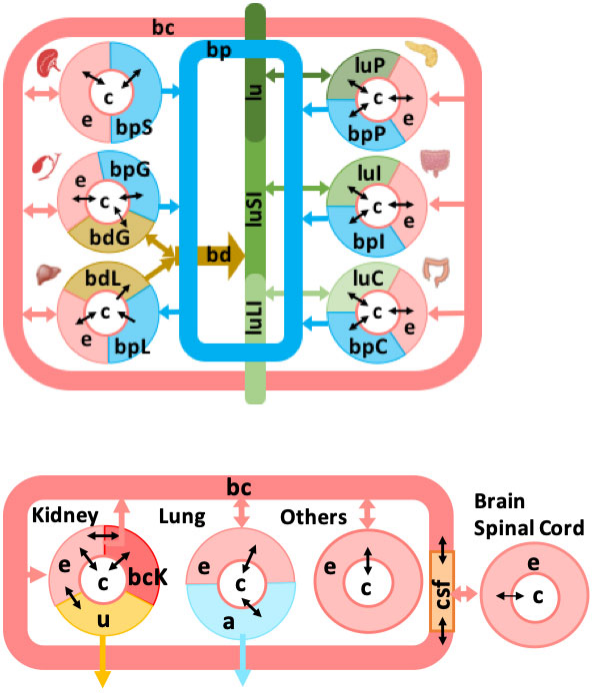
Biofluid compartment definitions for the individual organs in the WBM reconstructions Refer to Table 2 for the abbreviations of the biofluid compartments.

We considered 20 organs, six sex‐specific organs, and six blood cells (Table 1). For simplicity, we refer to all of these as organs in the following. We defined 13 biofluid compartments to be considered in the WBM reconstructions (Fig 1B, Table 2). For each organ, transport reactions from the extracellular compartment ([e]) to the blood compartment ([bc]) were added to Recon3D*. An example of such transport reactions is the transport of metabolite A from and to the blood compartment into the extra‐organ space of the heart: Heart_EX_A[bc]_[e]: 1 A[bc] <=> 1 Heart_A[e]. Additional transport reactions were added to those organs that are connected to a third, or forth, biofluid (e.g., liver, Fig 7, Table 2). For organs, which can only take up from or secrete into a particular biofluid (see arrows in Figs 1B and 7), the reaction directionality was set accordingly. The corresponding transport mechanism was always set to occur through facilitated transport, which assumes that the metabolites can be transported from the biofluid to the interstitial fluid surrounding the organ cells and that the transport is driven either by concentration difference (diffusion) or pressure difference (bulk flow). Each reaction in Recon3D* and the newly added transport reactions received a suffix corresponding to one organ (Table 1). Numerous organs are known to store metabolites, e.g., the liver. We included sink reactions for stored metabolites in the corresponding organs (Table EV6).

**Table 2:** Biofluid compartments and the connected organs present in the WBM reconstructions

Biofluid compartment	Abbreviation	Connected organs
Diet	[d]	–
Lumen	[lu]	–
Lumen, small intestine	[luSI]	Small intestinal cells
Lumen, large intestine	[luLI]	Colonocytes
Feces	[fe]	–
Blood, circulation	[bc]	All except brain and spinal cord
Blood, portal vein	[bp]	Liver, colonocytes, small intestinal cells, pancreas, spleen
Bile duct	[bd]	Liver, gallbladder
Cerebrospinal fluid	[csf]	Brain, spinal cord
Urine	[u]	Kidney
Sweat	[sw]	Skin
Breast milk (female only)	[mi]	Breast
Air	[a]	Lung

The male meta‐reconstruction was constructed such that it contained 28 times Recon3D* in an anatomically correct manner as described in the next section (Fig 1B). The female meta‐reconstruction was constructed such that it contained 30 times Recon3D* in an anatomically correct manner. Each draft meta‐reconstruction contained more than 300,000 reactions.

##### 1.2.2 Anatomically accurate organ connectivity

The dietary input compartment represents the exchange medium consisting of all the dietary ingredients that the human body can consume. We added diet uptake reactions for all metabolites with defined exchange reactions in Recon3D*, as well as transport reactions along the gastrointestinal tract (Fig 1B, Table 2) to the draft meta‐reconstructions. The dietary inputs from the [d] enter the gastrointestinal lumen represented by [lu] in the WBM reconstructions (Table 2). The lumen compartment setup in the model represents the gastrointestinal lumen, which is unidirectional and exits into the fecal excretion compartment [fe]. The fecal excretion compartment represents the excretory end‐products comprising the undigested and unabsorbed part of the dietary input. In the WBM reconstructions, the gastrointestinal lumen compartment is further divided into the small intestinal lumen [luSI] and the large‐intestinal lumen [luLI]. While the gastrointestinal lumen receives the diet input [d], the small intestinal lumen receives metabolite drainage from the gallbladder (via the bile duct [bd]), pancreas, and the small intestine. The large intestinal lumen is specific for the large intestine receiving metabolites only from the colonocytes and from the small intestinal lumen. Both small intestinal epithelial cells (sIEC) and colonocytes were allowed to take up metabolites from their corresponding luminal compartment and could secrete some metabolites into the lumen (Tables EV12A and EV13A). Gut microbes are known to produce in the gastrointestinal tract valuable metabolic precursors to the human host. To enable the uptake of such metabolites by the small intestinal cells and the colonocytes, we added corresponding sink reactions into the corresponding luminal compartments of the meta‐reconstructions (Table EV11).

The portal venous blood [bp] receives metabolites from the colon, small intestine, spleen, pancreas, and gallbladder, which drains into the liver for further metabolism and for exposure to the systemic blood circulation [bc]. The bile duct [bd] is a special compartment, which is specific for liver and gallbladder. Bile is synthesized in the liver and stored in the gallbladder (Murray *et al*, 2000). From the liver, bile flows into the bile duct. The flow of bile into the small intestinal lumen via the bile duct depends on the Sphincter of Oddi that closes during the inter‐digestive period, increasing the pressure in the bile duct, and resulting in backflow of bile into the gallbladder, where it is further concentrated. During the digestive phase, the Sphincter of Oddi opens causing the concentrated bile flow into the small intestinal lumen to aid in digestion (Gropper *et al*, 2009).

The systemic blood circulation [bc] is represented by the circulatory blood in the WBM reconstruction, which provides nutrients to all organs. Since the brain and the spinal cord are specific in its metabolite exchange, we introduced the blood–brain barrier and cerebrospinal fluid [csf] as an additional compartment. The blood–brain barrier selectively allows the exchange of metabolites, to and from the brain (explained below), and the cerebrospinal fluid receives metabolites from the brain, finally draining into the circulatory blood compartment (Fig 1). The lung takes up oxygen from the environment and gives out carbon dioxide, which is captured as [a] in the WBM reconstructions. Finally, the urine compartment [u] contains filtered metabolites from the kidney for final excretion. These biofluid compartments signify precise anatomical barriers of the organs of the human body.

To ensure that metabolites found in different biofluids based on metabolomics data (see below) would also be present in the WBM reconstructions, we added demand reactions for all those metabolites to the corresponding biofluid compartments of the draft meta‐reconstructions. By doing so, we enforced that at least one organ could produce a given metabolite, or that it would be taken up from the diet (and the lumen).

#### 1.3 Manual curation of the WBM reconstruction content

We defined a core reaction set containing all reactions, which should be present in the WBM reconstructions based on literature and omics data evidence. For each organ, the core reaction set was expanded to include organ‐specific (i) protein information from the human proteome map (HPM) (Kim *et al*, 2014) and the human protein atlas (HPA) (Uhlen *et al*, 2015), (ii) extensive manual curation of more than 600 literature sources for the presence or absence of metabolic and transport reactions, genes, or pathways in all organs, and (iii) reactions presence in four published tissue‐specific organs, i.e., red blood cell (Bordbar *et al*, 2011b), adipocyte (Bordbar *et al*, 2011a), small intestine (Sahoo & Thiele, 2013), and liver (Gille *et al*, 2010). Note that these input data were not sex‐specific, due to the absence of corresponding data on an organ‐specific scale.

This manual curation yielded a comprehensive core reaction set, which served as input for the extraction algorithm (see section 1.3). For reactions that were found to be absent in an organ, we set the corresponding lower and upper bounds to 0 in the draft meta‐reconstructions, which corresponds to removing them from the meta‐reconstructions. These absent reactions were then removed from the meta‐reconstructions by the algorithm as they were not flux‐consistent.

##### 1.3.1 Proteomic data

The HPM resource (Kim *et al*, 2014) provides protein information for 17,294 genes that accounted for 84% of the protein‐encoded part of the human genome. These proteins were obtained from normal histological tissue samples, accounting for 17 adult tissue types and 6 primary hematopoietic cells, from three deceased individuals. We queried the database (10/12/2015) for all genes present in Recon3D*. For 1601/1709 Recon3D* genes/proteins (94% coverage) to obtain their distributions in the 23 tissue/cell types. The protein expression data were scaled to range from 0 to 1. Only those proteins with an expression level of greater or equal to 0.2 were assumed to be expressed in an organ (Table EV2).

Moreover, to complement the HPM resource, we used HPA (Uhlen *et al*, 2015). The protein expression data for the normal tissues were downloaded and sorted according to the tissue type. The proteins in the human protein atlas have been analyzed with a single antibody, and the expression levels have been reported based on the antibody staining (Uhlen *et al*, 2010). The expression levels depend on the quality of the antibody used (Uhlen *et al*, 2010). Hence, we considered only those proteins, which had a high and medium expression level for a given tissue/organ. For the brain, we combined the protein information of cerebellum, cerebral cortex, hippocampus, and lateral ventricle. The Ensembl gene IDs were converted to their corresponding EntrezGene IDs using the BioMart tool from Ensembl (Flicek *et al*, 2014). Overall, HPA provided protein expression information for 1504/1709 (88%) of the Recon3D* genes (Table EV3). Together, these two proteomic resources provided expression information for 1690/1709 (99%) of the Recon3D* genes.

We used the GPR associations given in Recon3D* to identify reactions to be present in the core reaction set. Note that in the case of protein complexes, the presence of one of the proteins from the complex was deemed sufficient to require the presence of at least one reaction in the WBM reconstruction.

##### 1.3.2 Literature‐based curation of WBM reconstruction content

###### 1.3.2.1 Organ‐specific metabolic information

We performed manual curation of reactions, genes, and pathways based on literature for all included organs (Table EV1) but focused in particular depth on the metabolism occurring in eight organs (skeletal muscle, skin, spleen, kidney, lung, retina, heart, and brain), which contribute substantially to inter‐organ metabolism (Murray *et al*, 2009). We followed the bottom‐up, manual reconstruction approach established for metabolic reconstructions (Thiele & Palsson, 2010).

To facilitate this large‐scale, manual literature curation effort, we defined “metabolic units” that not only accounted for the Recon3D* sub‐systems but were also metabolite specific. We categorized individual metabolic and transport reactions in Recon3D* as metabolic units. Each metabolic unit represented the first and last reaction step of a particular pathway and contained three components: (i) major metabolic pathway, (ii) product formed, and (iii) cellular location. The reaction content of Recon3D* was classified into 427 metabolic units when only the major metabolic pathway was considered, and the cellular compartments ignored. When the whole metabolic unit, along with all its components, was taken into account, 5,637 metabolic units resulted. Usage of the metabolic units greatly accelerated the reconstruction process as they account for individual metabolite‐specific pathways as well as key enzymes of the biochemical pathways, in the same way that they are frequently reported and referred to in the biochemical literature. This literature information was translated into the occurrence and non‐occurrence of metabolic units. Additionally, we noted tasks that an organ can carry out (e.g., storage of glycogen) or the inability to carry out a particular task (e.g., storage of vitamins occurs only in a limited number of organs), leading to the formulation of organ‐specific metabolic objectives.

We also collected information on the pathways/reactions that are absent across organs (Table EV1). Primary literature articles, review articles, and books on organ‐specific metabolism were thoroughly studied to derive the pathway information. In the case of reported absence in an organ, the corresponding organ‐specific reactions were set to have a lower and upper bound of 0 in the meta‐reconstruction(s), thus effectively removing these reactions from the organ.

Brief descriptions of the organ‐specific metabolism for those organs that we curated extensively for their metabolic content can be found at the Virtual Metabolic Human database (http://www.vmh.life).

###### 1.3.2.2 Presence of cellular organelles in organs

Within the blood tissues, differences in the presence of cellular organelles exist between fully matured red blood cells and others. For instance, fully matured red blood cells are devoid of a nucleus and any other organelles (Berg *et al*, 2001). The other hematopoietic cells, such as the B‐lymphocytes, T‐lymphocytes, natural killer cells, and monocytes, contain all the cellular organelles (Takahashi *et al*, 1986; Kaneda *et al*, 1991; Delva *et al*, 2006). Therefore, we performed a thorough manual search and obtained a definitive occurrence of cellular organelles for 19/32 organs from the literature (Table EV4). This search was important to accurately represent the organs’ metabolic capabilities in the WBM reconstructions. For the remaining organs, no information could be found in the literature and the presence of all organelles was assumed.

###### 1.3.2.3 Nutrient storage within organs

Maintenance of certain metabolite pools and metabolite storage as a reserve for energy demands within the cells is crucial for maintaining organ‐specific functions. For instance, glycogen is stored in liver and in the skeletal muscle (Murray *et al*, 2000), and fatty acids are stored in the adipocytes (Summers *et al*, 2000). During periods of fasting, liver glycogen serves to maintain the blood glucose levels. Additionally, triglyceride stores in the adipocytes are broken down to supply fatty acids to skeletal muscle and heart to serve as an energy resource (Lanham‐New *et al*, 2013). A thorough manual search of the storage capacity for metabolites and dietary nutrients by various organs was performed. Known storage capacities were represented by adding specific demand/sink reactions (Table EV6) to the corresponding organs, which were then added to the core reaction set. The demand reactions serve as cellular demand, or usage, of the metabolites in the feeding stage and can be opened or closed when setting up the simulation conditions (Dataset EV2). Similarly, the sink reactions can release stored metabolites from the respective organs during nutrient deprivation or overnight fasting state.

###### 1.3.2.4 Metabolic objectives for organs

As a result of the literature search for the organ‐specific metabolic pathways, we described each organ by its chief metabolic functions, e.g., arginine synthesis by the kidney, citrulline synthesis by the small intestine, cholesterol synthesis by the spleen, vitamin D synthesis by the skin, and the Cori cycle between liver and skeletal muscle. Glucose from liver enters skeletal muscle, where it is converted to lactate via anaerobic glycolysis. The muscle then releases lactate back into the circulation to be utilized for gluconeogenesis by the liver, contributing to the muscle‐liver‐Cori cycle (Murray *et al*, 2000). The kidney is the major organ for the synthesis of arginine from citrulline (van de Poll *et al*, 2004). Citrulline synthesized in the small intestine reaches kidney for further metabolism by urea cycle reactions, thereby contributing to inter‐organ amino acid metabolism. The spleen is an important hematopoietic organ, and synthesis of dolichol and cholesterol from acetate are important indicators of this process (Potter *et al*, 1981). The human skin is mainly responsible for the synthesis of vitamin D from 7‐dehydrocholesterol in multiple reaction steps (Chen *et al*, 2007). These physiological functions and their representative biochemical reactions were set as metabolic tasks for each organ (Table EV1).

###### 1.3.2.5 Bile composition

Bile salts aid in the digestion and absorption of fat constituents through their micellar properties (Murray *et al*, 2009). Recon3D describes the human metabolism of bile acids comprehensively (Brunk *et al*, 2018). On an organ level, bile is synthesized in the liver and drained into the gallbladder, via the bile duct. The gallbladder stores the bile constituents and releases it into the intestinal lumen, i.e., into the duodenum (first part of small intestine) for efficient digestion and absorption of food. To capture the bile composition, we used a large‐scale proteomic analysis of human bile, which also measured metabolites in the human bile (Fuda *et al*, 2006; Farina *et al*, 2009). Conclusive evidence concerning their presence in bile was available for 84 exchanged metabolites in the Recon3D* model; and for 459 exchanged metabolites, absence in the bile was concluded (Table EV7). The remaining transport reactions into the bile duct, without evidence of presence or absence, were unconstrained and algorithmically added when extracting the subnetworks depending on their secretion from the gallbladder and its internal metabolism. The storage (aka demand) reactions for 26 bile salts were added to the gallbladder.

###### 1.3.2.6 Recon3D* exchange metabolites present/absent in diet

Comprehensive information for the presence in the diet was found for 300 metabolites, and for 50 metabolites the absence in the diet was reported. For the remaining exchange metabolites, no information could be found in the literature. Hence, these were left unconstrained in the meta‐reconstruction (Table EV10).

###### 1.3.2.7 Metabolomic data

The WBM reconstructions account for 13 biofluid compartments (Table 2). The core reaction set accounted for biofluid‐specificity of 1,105 metabolites such that they were incorporated into the respective biofluid compartments (i.e., in the blood circulation, portal blood, cerebrospinal fluid, feces, and urine). This was represented by adding the corresponding demand reactions to the biofluids in the meta‐reconstructions (see above). This information was extracted from various literature references as well as databases, including the Human Metabolome database (Wishart *et al*, 2013) (Table EV8). Many of these metabolites have been reported to be changed in pathological states, and highlight the potential of the WBM reconstructions in capturing the known biomarkers and prediction of new ones.

###### 1.3.2.8 Transport reaction information

Our previous work on human membrane transporters (Sahoo *et al*, 2014) served as a compendium of transport proteins. These transport proteins were noted with their organ distribution from the relevant scientific literature (Table EV5). Again, the GPR associations within Recon3D* model were used, and the corresponding transport reactions were extracted and incorporated into the core reaction set of the specific organ.

Conclusive evidence for the presence of 166 transport proteins distributed across 26 organs formed the transport protein part of the core reaction set. For the remaining organs, the presence of transport proteins was derived from HPA and HPM. While the presence of the transport protein and its associated reaction was included in the core reaction set, the non‐occurrence was ignored. This is because the absence of a transport protein across an organ or tissue is difficult to establish. Interestingly, amino acids transport proteins, ABC transporters, and lipid transporters were found to be more ubiquitously expressed across organs. Most transport protein information was found for kidney, brain, liver, heart, and skeletal muscle, while for hematopoietic cells (e.g., red blood cells and platelets), the least information could be found. We enabled the secretion of mucin degradative products, glycans, and ethanolamine into the large‐intestinal lumen from the colon (Chang *et al*, 2004; Fabich *et al*, 2008).

###### 1.3.2.9 Defining the blood–brain barrier

We represented the blood–brain barrier in the WBM reconstructions. The brain is separated from the blood/extracellular compartment by the blood–brain barrier (Redzic, 2011). This barrier formed by the brain endothelial cells and exhibits restricted entry of small molecules. Molecules with a molecular mass below 500 Da and possessing high lipid solubility can enter the brain (Pardridge, 2005). This information was used to add the blood–brain barrier transport reactions to the core reaction set (Table EV9). 240 metabolites have been reported not to pass the blood–brain barrier, which includes lecithin, triglycerides, lysolecithin, and cholesterol (Pardridge & Mietus, 1980; Redzic, 2011). Thus, the corresponding blood–brain barrier transport reactions were constrained to zero in the meta‐reconstructions, thereby eliminating them from the WBM reconstructions. The remaining transport reactions were unconstrained enabling their addition during the subnetwork generation process. Their addition, therefore, depended on the internal metabolic architecture of the brain (and spinal cord).

###### 1.3.2.10 Biomass reactions

The WBM reconstructions contain three different versions of the biomass reaction. These are (i) biomass_reaction, (ii) biomass_maintenance, and (iii) biomass_maintenance_noTrTr. The biomass_reaction is the general biomass reaction as in Recon3D* model, biomass_ maintenance is same as biomass_reaction except for the nuclear deoxynucleotides, and biomass_maintenance_noTrTr, where _noTrTr stands for no transcription and translation, is devoid of amino acids, nuclear deoxynucleotides, and cellular deoxynucleotides except for adenosine‐triphosphate.

The biomass reaction was retained only for tissues/organs known to possess regenerative capacity, i.e., liver (Malhi *et al*, 2002), heart (Senyo *et al*, 2013), and kidney (Li & Wingert, 2013). For the remaining organs, only the biomass_maintenance reaction was added, requiring the maintenance of cellular metabolic profiles, i.e., the organs capability to synthesize all the biomass components except the nuclear deoxynucleotides. The biomass_maintenance_noTrTr reaction may be used to model fasting conditions, as amino acids if stored intracellularly, increase the osmotic pressure, necessitating their rapid catabolism (Lanham‐New *et al*, 2013).

In particular, the biomass reactions were formulated as follows:

“biomass_maintenance: 20.6508 h2o[c] + 20.7045 atp[c] + 0.38587 glu_L[c] + 0.35261 asp_L[c] + 0.036117 gtp[c] + 0.50563 ala_L[c] + 0.27942 asn_L[c] + 0.046571 cys_L[c] + 0.326 gln_L[c] + 0.53889 gly[c] + 0.39253 ser_L[c] + 0.31269 thr_L[c] + 0.59211 lys_L[c] + 0.35926 arg_L[c] + 0.15302 met_L[c] + 0.023315 pail_hs[c] + 0.039036 ctp[c] + 0.15446 pchol_hs[c] + 0.055374 pe_hs[c] + 0.020401 chsterol[c] + 0.002914 pglyc_hs[c] + 0.011658 clpn_hs[c] + 0.053446 utp[c] + 0.27519 g6p[c] + 0.12641 his_L[c] + 0.15967 tyr_L[c] + 0.28608 ile_L[c] + 0.54554 leu_L[c] + 0.013306 trp_L[c] + 0.25947 phe_L[c] + 0.41248 pro_L[c] + 0.005829 ps_hs[c] + 0.017486 sphmyln_hs[c] + 0.35261 val_L[c] ‐> 20.6508 h[c] + 20.6508 adp[c] + 20.6508 pi[c] + lipid_membrane[c] + proteome[c] + transcriptome[c] + biomass_maintenance_dummy_objective”

where the lipid_membrane[c] is degraded via the reaction

“LIPID_DEGRx: lipid_membrane[c] ‐> 0.023315 pail_hs[c] + 0.154463 pchol_hs[c] + 0.055374 pe_hs[c] + 0.020401 chsterol[c] + 0.002914 pglyc_hs[c] + 0.011658 clpn_hs[c] + 0.005829 ps_hs[c] + 0.017486 sphmyln_hs[c]”

the proteome[c] is degraded via the reaction

“PROTEOME_DEGRx: proteome[c] ‐> 0.385872 glu_L[c] + 0.352607 asp_L[c] + 0.505626 ala_L[c] + 0.279425 asn_L[c] + 0.046571 cys_L[c] + 0.325996 gln_L[c] + 0.538891 gly[c] + 0.392525 ser_L[c] + 0.31269 thr_L[c] + 0.592114 lys_L[c] + 0.35926 arg_L[c] + 0.153018 met_L[c] + 0.126406 his_L[c] + 0.159671 tyr_L[c] + 0.286078 ile_L[c] + 0.545544 leu_L[c] + 0.013306 trp_L[c] + 0.259466 phe_L[c] + 0.412484 pro_L[c] + 0.352607 val_L[c]”

and the transcriptome[c] is degraded via the reaction

“TRANSCRIPTOME_DEGRx: transcriptome[c] ‐> 0.053446 amp[c] + 0.039036 cmp[c] + 0.036117 gmp[c] + 0.053446 ump[c]”.

Finally, the biomass_maintenance_dummy_objective is part of the whole‐body biomass reaction, which is defined as:

“Whole_body_objective_rxn: 21.4286 Adipocytes_biomass_maintenance_dummy_objective + 0.02 Agland_biomass_maintenance_dummy_objective + 2 Brain_biomass_maintenance_dummy_objective + 0.428571 Colon_biomass_maintenance_dummy_objective + 0.472857 Heart_biomass_maintenance_dummy_objective + 0.442857 Kidney_biomass_maintenance_dummy_objective + 2.57143 Liver_biomass_maintenance_dummy_objective + 0.765714 Lung_biomass_maintenance_dummy_objective + 40 Muscle_biomass_maintenance_dummy_objective + 0.142857 Pancreas_biomass_maintenance_dummy_objective + 0.0228571 Prostate_biomass_maintenance_dummy_objective + 0.000171429 Pthyroidgland_biomass_maintenance_dummy_objective + 0.000465714 Retina_biomass_maintenance_dummy_objective + 0.0428571 Scord_biomass_maintenance_dummy_objective + 0.914286 sIEC_biomass_reactionIEC01b_dummy_objective + 3.71429 Skin_biomass_maintenance_dummy_objective + 0.257143 Spleen_biomass_maintenance_dummy_objective + 0.214286 Stomach_biomass_maintenance_dummy_objective + 0.05 Testis_biomass_maintenance_dummy_objective + 0.0285714 Thyroidgland_biomass_maintenance_dummy_objective + 0.0642857 Urinarybladder_biomass_maintenance_dummy_objective + 0.00212143 Bcells_biomass_maintenance_dummy_objective + 0.0117857 CD4Tcells_biomass_maintenance_dummy_objective + 0.00353571 Nkcells_biomass_maintenance_dummy_objective + 0.00392857 Monocyte_biomass_maintenance_dummy_objective + 0.0285714 Platelet_biomass_maintenance_noTrTr_dummy_objective + 3.53571 RBC_biomass_maintenance_noTrTr_dummy_objective + 0.0142857 Gall_biomass_maintenance_dummy_objective ‐>”

Please note that the stoichiometric coefficients in the “Whole_body_objective_rxn” can vary between sex and individuals, depending on their organ fractions, which are either derived from experimental data (e.g., whole‐body scans), or calculated using phenomenological methods (see Dataset EV2: 3.2 for more details) (Young *et al*, 2009).

##### 1.3.3 Published metabolic reconstructions

For the red blood cell (Bordbar *et al*, 2011b), the adipocytes (Bordbar *et al*, 2011a), the small intestine (Sahoo & Thiele, 2013), and the liver (Gille *et al*, 2010), genome‐scale metabolic reconstructions have been published; hence, their reactions were also used for defining present reactions in the corresponding organs of the WBM reconstructions. Note that these reconstructions have been assembled based on Recon 1 (Duarte *et al*, 2007).

The published red blood cell reconstruction has been assembled using multiple proteomic data sets (Bordbar *et al*, 2011b). The published adipocyte reconstruction was generated by tailoring Recon 1 based on genome annotation data, physiological, and biochemical data from online databases (e.g., KEGG (Okuda *et al*, 2008), NCBI, UniProt (Consortium, 2012b), and BRENDA (Scheer *et al*, 2011), and literature (Bordbar *et al*, 2011a). The liver/hepatocyte reconstruction has been built through manual curation of the relevant scientific literature, using Recon 1 and KEGG as starting points (Gille *et al*, 2010). Additionally, gene expression data sets of normal human liver samples have served as a secondary line of evidence (Gille *et al*, 2010). The small intestinal epithelial cell reconstruction (Sahoo & Thiele, 2013) has been assembled using primary literature, organ‐specific books, and databases. Since the small intestinal epithelial cell model maintained different extracellular compartments representing the apical and basolateral polarity of the cell, the reactions were added as such to the core set. However, the GPR association was updated with those used in the Recon3D* model.

Mapping of the reaction content in these published reconstructions onto Recon3D* was done manually using the reaction abbreviation, reaction description, and reaction formula. In the case of the adipocyte, the blood compartment was replaced with the extracellular compartment to find the correct matches with the Recon3D* reactions. Additionally, the published adipocyte model (Bordbar *et al*, 2011a) contained a lumped version of the fatty acid oxidation reactions; hence, the corresponding un‐lumped versions were mapped onto Recon3D*. The mapped reactions of all four reconstructions were added to the core reaction set, after adding the corresponding organ‐specific prefix to the reaction abbreviations.

#### 1.4 Algorithmic generation of draft sex‐specific WBM reconstructions from the tailored meta‐reconstructions

To achieve the algorithmic generation of draft sex‐specific WBM reconstructions from the tailored draft meta‐reconstructions, we used the fastCore algorithm (Vlassis *et al*, 2014). Briefly, fastCore takes as input a (meta‐)metabolic reconstruction and the set of core reactions, known to be active in the network, to identify a flux‐consistent subnetwork containing all core reactions (as long as they are flux consistent) and a minimal number of additional, flux‐consistent reactions.

We considered for each sex two different scenarios and defined constraints on the meta‐reconstructions accordingly. In the end, we obtained four meta‐reconstructions.

First, a feeding condition was defined, in which all dietary exchange reactions were open (i.e., the lower bound was set to –inf and the upper bound was set to zero) and the storage of metabolites in the various organs was enabled (lower bound on the sink reactions were set to be zero and the upper bound was set to be 1,000,000 mmol/day/person).
Second, a fasting condition was defined, in which all dietary uptake reactions were closed (lower and upper bound were set to zero) but access to stored metabolites from the different organs was enabled (sink reactions had a lower bound of −1,000,000 mmol/day/person and an upper bound of zero).

Using these four differently setup meta‐reconstructions along with the corresponding (i.e., sex‐specific) core reaction sets and the fastCore algorithm, we generated four flux‐consistent subnetworks. We then removed the demand reactions for the biofluid metabolites, defined all reactions present in each subnetwork to be the core reaction set for the given setup, and repeated the extraction of flux‐consistent subsets from the meta‐reconstructions. By doing so, we also enforced that at least one reaction could either transport or excrete a given biofluid metabolite or that it is catabolized in at least one organ.

Finally, we joined the fasting and the feeding subnetworks for each sex. The rationale for having the feeding and the fasting condition is that the human body can fast overnight, and thus the WBM reconstructions should capture this capability regarding catabolic as well as anabolic metabolic reactions. Note that the WBM reconstructions are not able to starve, as this would require the degradation of muscle proteins, which we did not explicitly capture in Recon3D*, and thus in the WBM reconstructions.

The fastCore adds a minimal number of additional reactions to the core reaction sets to form the flux consistent, compact subnetwork. Hence, the added reactions represent hypotheses of which reactions and pathways would be needed to make the subnetworks flux consistent, given a set of core reactions. It does not mean that the proposed solution is biologically relevant. Consequently, after the generation of the male and female draft WBM reconstructions, we manually inspected that the added reactions were consistent with the current knowledge about organ‐specific metabolism. The core reactions and the absence of organ‐specific reactions were updated based on literature evidence, and the subnetwork generation was repeated.

As one example, we encountered genes/proteins that were present in an organ‐specific manner as per the human proteome data set (Kim *et al*, 2014), but, which were not added to the respective organs in the draft WBM reconstructions by fastCore, due to missing transport reactions. These reactions were subsequently added to the human metabolic reconstruction, which ultimately yielded Recon3D, and thus Recon3D* model. This is because the development of Recon3D and the WBM reconstructions occurred in parallel. In brief, for each of these instances where core reactions were not added to the subnetwork, we analyzed them manually. A typical example is the addition of the reactions SALMCOM and SALMCOM2 to the core reaction set for colon, rectum, adrenal gland, platelet, lung, heart, brain, retina, B cells, CD4 cells, CD8 cells, NK cells, testis, and prostate. While SALMCOM was correctly added by fastCore to the liver, gallbladder, pancreas, and kidney, it was missing in the remaining organs, despite proteomic expression evidence. Hence, we added transport reactions for the participating metabolites of SALMCOM and SALMCOM2, i.e., normetanephrine (VMH ID: normete_L) and metanephrine (VMH ID: mepi) as well as their demand reactions in the blood circulation, as both compounds have been detected in blood and urine (HMDB00819, HMDB04063). The addition of these transport reactions allowed the metabolites to be transported across the whole body and excreted in the urine by the kidney. Similar to the discussed case, many transport reactions were added during the debugging process that enabled the whole‐body routing of phospholipids, cholesterol ester species, and acylcarnitines. These examples show that the WBM reconstructions can be effectively used to investigate inter‐organ metabolite cross‐talk.

Overall, due to the complexity of the WBM reconstructions and the large number of reactions (more than 80 k, Fig 1B), we iterated this process more than 100 times, each time focusing on different organs or pathways (spanning one or multiple organs).

At the end of the reconstruction part that relied on fastCore, we removed all artificial reactions from the WBM reconstructions, such as sink/demand reactions for biofluid metabolites and the sinks for metabolites of microbial origin in the lumen.

#### 1.5 Refinement and curation of the WBM reconstructions

We then proceeded to further manually curate the reconstructions by adding reactions that have not been included using fastCore but for which biological evidence could be found in the literature (e.g., certain amino acid transport reactions). We also added transport reactions for all metabolites present in the extracellular space of the organs to the corresponding biofluids. For instance, certain metabolites were present in the CSF compartment due to brain and spinal cord metabolism but no corresponding transport reactions from CSF [csf] to blood circulation [bc] were included by fastCore. Hence, we added those reactions as the entire CSF drains into the blood circulation. Again, the reason that such reactions were not included into the reconstructions by fastCore as it returns a most compact subnetwork.

Moreover, WBM model predictions were compared with organ‐specific data and known whole‐body metabolic functions (e.g., multi‐organ metabolism of glucose (Cori cycle), amino acid cycle (Cahill cycle)). In this step, we added missing reactions to the sex‐specific draft WBM reconstructions. During the entire reconstruction process, we performed the same quality control and assurance tests as defined for genome‐scale metabolic reconstructions (Thiele & Palsson, 2010).

### 2 Conversion from reconstruction to condition‐specific models

To ensure reproducibility of the presented work, and also to reduce the number of supplemental tables, we provide a MATLAB (Mathworks, Inc) live script along with a PSCM toolbox, which is an extension to the COBRA Toolbox (Heirendt *et al*, 2019) (to be obtained from https://github.com/opencobra/cobratoolbox).

2.1

##### 2.1.1 Unit of WBM models

The unit of all WBM model reactions is in mmol/day/person (Harvey, 70 kg; Harvetta: 58 kg), if not stated differently.

##### 2.1.2 Flux balance analysis

The conversion of a metabolic reconstruction into a condition‐specific model includes the transformation of the biochemical reaction list into a computable, mathematical matrix format (*S matrix*), where the columns correspond to reactions (variables) and the rows correspond to the metabolites. If a metabolite (j) participates in a reaction (i), the stoichiometric coefficient is entered in the corresponding cell (j, i). Consequently, each row represents the mass‐balance equation for a given metabolite (dx/dt).

The conversion into a model also requires the imposition of physicochemical constraints (e.g., mass conservation) and systems boundaries, in the form of so‐called exchange reactions (Palsson, 2015). The constraint‐based modeling and reconstruction (COBRA) approach assumes that the modeled system is in a steady state (S.v = dc/dt = 0), where v is a flux vector for n reactions and dx/dt the change in metabolite concentration (dx) over time (dt). The S matrix gives rise to an underdetermined system of linear equations; i.e., there are fewer equations (mass‐balances) than variables (reaction fluxes). Consequently, a polyhedral convex steady‐state solution space contains all feasible steady‐state solutions. By adding further constraints (e.g., nutrient uptake rates, enzyme reaction rates) to the model, one restricts the solution space to solutions that are biologically relevant under this condition. Despite incomplete knowledge about many reaction rates, kinetic parameters, metabolite and enzyme concentrations, the COBRA approach permits the computation of possible phenotypic properties of a metabolic model, derived from a reconstruction.

In flux balance analysis (Orth *et al*, 2010), the modeled system is assumed to be at a steady state (i.e., dx/dt = 0). Consequently, the underlying mathematical problem is a linear programming problem that can be efficiently solved and at a large scale, as only one global optimal solution exists. The linear programming problem is formulated as:

$$\text{Optimize z}=c^{T}.v$$

Such that S.v=0

$${\text{lb}}_{i}\leq v_{i}\leq {\text{ub}}_{i}$$

where c is a vector (n,1), lb the lower bound, and ub the upper bound of reaction i. For irreversible reactions, lb was set to 0 mmol/day/person and ub was > 0 mmol/day/person. In the case of reversible reactions, lb was set to < 0 mmol/day/person. In absence of specific simulation constraints, ub was set to the arbitrary value 1,000,000 mmol/day/person, and lb to ‐1,000,000 mmol/day/person. Uptake reactions (e.g., dietary uptake reactions) were defined to have a negative flux value, while excretion reaction (e.g., urine excretion, fecal excretion) was defined to have a positive flux value.

##### 2.1.3 Coupling constraints

Coupling constraints were implemented in the WBM reconstructions as described previously (Thiele *et al*, 2010; Heinken *et al*, 2013). Briefly, coupling constraints enforce that the flux through a set of coupled reactions is proportional to a specified reaction (e.g., biomass reaction). The metabolic and transport reactions in every organ were coupled to the respective organ's biomass objective function (BOF). The coupling constraints prevent biologically implausible solutions where the reactions in an organs carry flux even though the flux through the organ's BOF is zero. They were realized by implementing a coupling factor of 20,000 for each reaction. This allowed each forward and reverse reaction to carry a flux of up to 20,000 and −20,000 times the flux through the BOF, respectively.

##### 2.1.4 Flux variability analysis of the unconstrained and constrained WBM models

We performed flux variability analysis (Gudmundsson & Thiele, 2010) on the unconstrained and physiologically constrained male WBM model using a Julia implementation (Heirendt *et al*, 2017) of flux balance analysis (Orth *et al*, 2010) by minimizing and maximizing each model reaction. The flux span of a reaction is defined as maximal possible flux value (v_max,i_) minus maximal possible flux value for a reaction i (v_min,i_): fluxSpan_i_ = (v_max,i_ − v_min,i_).

2.2

##### 2.2.1 “Sanity checks” on the WBM and organ‐specific reconstructions

Quality control and quality assurance tests, or sanity checks have been compiled, expended, and implemented by the systems biology research community (Thiele & Palsson, 2010; Agren *et al*, 2013; Fleming *et al*, 2016; Swainston *et al*, 2016; Brunk *et al*, 2018), e.g., in MEMOTE (preprint: Lieven *et al*, 2018) and in the COBRA Toolbox (Heirendt *et al*, 2019). We ensured that the whole‐body metabolic reconstructions, and the organ‐specific reconstructions, passed the quality control and quality assurance tests implemented in the COBRA Toolbox (Heirendt *et al*, 2019). Additionally, we ensured that the organ‐specific reconstructions passed further quality control and quality assurance tests (Tables 3 and EV19).

**Table 3:** Lists of tests performed for all organ models derived from the WBM reconstructions

Number	Quality assurance and quality control tests
1	“fastLeakTest”
2	“Exchanges, sinks, and demands have lb = 0, except h2o”
3	“Exchanges, sinks, and demands have lb = 0, except h2o and o2”
4	“Exchanges, sinks, and demands have lb = 0, allow DM_atp_c_ to be reversible”
5	“Exchanges, sinks, and demands have lb = 0, test flux through DM_h[m] (max)”
6	“Exchanges, sinks, and demands have lb = 0, test flux through DM_h[c] (max)”
7	“Exchanges, sinks, and demands have lb = 0, ub of EX_h[e] = 0, test flux through DM_h[c] (min)”
8	“Test metabolic objective functions with open sinks”
9	“Test metabolic objective functions with closed sinks (lb)”
10	“Compute ATP yield”
11	“Check duplicate reactions”
12	“Check empty columns in rxnGeneMat”
13	“Check that demand reactions have a lb >= 0”
14	“Check consistency of model.rev with model.lb”
15	“Check whether singleGeneDeletion runs smoothly”
16	“Check for flux consistency”

We converted the WBM reconstructions into computational models (see also, Simulation details). First, we tested for secretion or production of metabolites when all the exchanges and sinks were closed (see Simulation details). We shall refer to this test as leak test. Thereafter, the models were tested for carrying non‐zero fluxes for reactions known to be required for important metabolic functions. We refer to these latter tests as metabolic function tests (see Simulation details).

###### 2.2.1.1 Leak tests

The WBM reconstructions were tested for thermodynamically infeasible loops that could generate metabolites or compounds when no mass enters the model. Such a test was done in two steps. Firstly, all the exchange, sink, and demand reactions were constrained to zero for the lower bound. Then, all the exchange reactions were optimized to check whether the model was carrying any non‐zero flux. After that, the biomass was optimized to check whether the model was carrying any zero flux. Secondly, a demand reaction for each compartment‐specific metabolite in the model was created and optimized. The basic purpose of running such a leak test was to check whether the model can generate anything from nothing. In case any of the demand or exchange reactions carry a non‐zero flux, the respective reaction was optimized while minimizing the Euclidian norm (Heirendt *et al*, 2019). Note that both Recon3D model and also Recon3D* model passed the leak test, as did the WBM reconstruction derived organ‐specific models (see Dataset EV2: 3.7).

###### 2.2.1.2 Metabolic function tests

Each organ‐specific reconstruction in the organ compendium has been tested for being able to have a non‐zero optimal flux value for up to 460 metabolic functions (Table EV19). Please note that not all organs were required to carry a non‐zero optimal flux value for all 460 metabolic functions, as each organ has its own primary metabolic functions.

###### 2.2.1.3 ATP yield

We also tested each organ model for the ATP yield and flux through the ATP synthetase for 15 carbon sources under aerobic and anaerobic conditions in accordance with the tests introduced by (Swainston *et al*, 2016; Table EV20).

2.3

##### 2.3.1 Computing considerations when working with the WBM models

In order to run a flux variability analysis on such large‐scale models efficiently, dedicated high‐performance was required. Most high‐performance computers have computing nodes with a relatively high base frequency per core, but with a low memory per node ratio. However, in order to perform large‐scale biological simulations efficiently, a high‐memory computing node is recommended with memory around 768 GB. The entire model must be able to be loaded into memory together with the result files, which may occupy more than 10–20 times the original model size. Memory access speed was critical to load the model and perform mathematical operations quickly. It is recommended that the computing node has a state‐of‐the‐art dual processor with 18 cores (2 threads per core) or more. In order to perform efficient flux variability analysis on the WBM models with acceptable solution times, it is recommended to run the optimization problems on all cores, each spawning 2 threads.

In addition to appropriate hardware, appropriately tuned software was important. For large‐scale models, poorly performing code and an untuned solver are bottlenecks. As the optimization solver accounts for most of the simulation time, parameter tuning together with an appropriate interface are critical. The Tomlab interface is recommended when interfacing the industrial‐quality CPLEX solver from MATLAB. Some analyses that were computing intensive, such as flux variability analysis, were run from MATLAB, but the solver is interfaced directly through a compiled MEX interface written in C. The newly developed Julia language has proven itself to be a valuable alternative in launching a multi core flux variability analysis (Heirendt *et al*, 2017), in particular for launching the analysis across multiple nodes.

### 3 Simulation details

Please refer to the accompanying Matlab LiveScript and its html version. Dataset EV2. The PSCM Toolbox contains all information, data, scripts, and functions to repeat the simulations in this paper Dataset EV1.

### 4 Statistical analyses

#### 4.1 Inherited metabolic diseases

To analyze the prediction accuracies and compare them across models and IEMs, the predictions for each metabolite were categorized (increased, inconclusive, and decreased) according to the simulation results (Table EV16). Then, the agreement in percentage with the *in vivo* measurements was calculated for Recon3D*, Harvey, and Harvetta separately. Moreover, to deliver a metric taking agreement by chance into account, Cohen's kappa and corresponding confidence intervals were calculated. For deriving p‐values on prediction accuracies, multinomial logistic regressions were utilized with *in vivo* observations as response variable (three categories: increased/inconclusive/decreased) and *in silico* prediction (three categories: increased/inconclusive/decreased) as the predictor. Finally, respecting potential dependencies of predictions coming from the same IEM, the agreement percentages per IEM was calculated. For example, if four of five biomarkers in a certain IEM were correctly predicted, the agreement percentage would be 80%. The models (Harvey/Harvetta vs Recon3D*) were then tested against each other using a non‐parametric sign test with agreement percentages per IEM as the response variable. This test effectively provided statistical inference on the question of whether Harvey/Harvetta showed higher accuracy in predicting biomarkers per IEM in comparison to Recon3D* in a majority of IEMs. All statistical calculations were performed in STATA 14/MP.

#### 4.2 BMR predictions

To validate the WBM‐based BMR predictions, we calculated the Pearson correlations and corresponding R‐squared values of the WBM predictions when regressing them on the empirically measured BMRs from (Prentice *et al*, 1986) in 13 women by standard linear ordinary least squares (OLS) regression. The provided fat‐free body mass was utilized to adjust the muscle and adipocyte coefficients in the whole‐body maintenance reaction. To optimize the prediction, we variegated systematically the ATP coefficient of the muscle biomass maintenance. Then, the parametrization of the WBM predictions was validated in an independent data set (Loureiro *et al*, 2015), which consisted of six female and 16 male athletes. The measured BMRs were the response variable, while the WBM predictions were the predictor of interest. We also allowed for sex‐specific intercepts by including sex as a covariate into the standard linear OLS regression equation. Once again, the correlations and R‐squared values regarding the empirically measured BMR were calculated. The prediction accuracies operationalized by the Pearson correlations were then compared with the accuracies of estimations based on sex, height, and weight, such as the Mifflin‐St Jeor equations. Further, for statistical inference, we fitted in the larger data set (Loureiro *et al*, 2015) a multivariable regression using the measured BMR as the response variable and the WBM predictions as well as the Mifflin‐St Jeor predictions as the predictor variables. We investigated whether adding the Mifflin‐St Jeor predictions to the WBM predictions or vice versa increased the overall model‐fit via likelihood ratio tests. This procedure derived inference on the question of whether the WBM predictions contain information not represented in the Mifflin‐St Jeor equation and vice versa. The calculations were performed in STATA 14/MP.

#### 4.3 Microbiome host interactions

To analyze the relation between microbiome traits and fluxes in the organ‐specific reactions, we calculated the maximal fluxes through the liver sulphotransferase, the liver alcohol dehydrogenase, and the colonic carboxylic acid:CoA ligase. Then, we performed OLS regressions with the fluxes as the dependent variable and the Bacteroidetes/Clostridia ratio as the predictor, allowing for non‐linear transformations of the predictor using multivariable fractional polynomials. Besides the classical parametric test on the fit of the regression models, we used two non‐parametrical methods to assess the significance of the regression models in sensitivity analyses. First, we used bootstrapping using 4,000 replications to determine p‐values. Second, we used quantile regression as an outlier robust method. The full results can be found in Table EV18.

## Author contributions

IT, RMTF, and SS conceived the study. IT, SS, MKA, and AH contributed to the reconstructions. RMTF and LH contributed tools and methods. IT and RMTF performed the simulations. IT, AH, and RMTF analyzed the data. IT, JH, and AH performed the statistical analysis. IT, RMTF, SS, JH, and AH wrote the manuscript. All authors edited the manuscript.

## Conflict of interest

The authors declare that they have no conflict of interest.

## Supporting information



Appendix

Table EV1

Table EV2

Table EV3

Table EV4

Table EV5

Table EV6

Table EV7

Table EV8

Table EV9

Table EV10

Table EV11

Table EV12

Table EV13

Table EV14

Table EV15

Table EV16

Table EV17

Table EV18

Table EV19

Table EV20

Dataset EV1

Dataset EV2

Review Process File

## Data Availability

Modeling computer scripts:
Physiological and stoichiometrically constrained modeling (PSCM) toolbox: GitHub (https://opencobra.github.io/cobratoolbox/stable/). Constraint‐based reconstruction and analysis (COBRA) toolbox: GitHub (https://opencobra.github.io/cobratoolbox/stable/). Microbiome modeling toolbox: GitHub (https://opencobra.github.io/cobratoolbox/stable/). Computational models:
Whole‐body metabolic reconstructions: Virtual Metabolic Human database (https://www.vmh.life/#downloadview). Microbial metabolic reconstructions: Virtual Metabolic Human database (https://www.vmh.life/#downloadview). Preecomputed results: https://github.com/ThieleLab
