# Supplementary material for: Personalized whole‐body models integrate metabolism, physiology, and the gut microbiome
Source: Mol Syst Biol. 2020 May 28;16(5):e8982. doi: 10.15252/msb.20198982 (PMC7285886; doi:10.15252/msb.20198982)
Supplement: Supplementary file 1 — Appendix [file MSB-16-e8982-s001.pdf]

Appendix Figures

For the manuscript "Personalized whole-body models integrate metabolism, physiology, and the gut microbiome"

Ines Thiele[1,2,3,4]*, Swagatika Sahoo[4]†, Almut Heinken[1], Johannes Hertel[1,5], Laurent Heirendt[4], Maike K. Aurich[4], Ronan M.T. Fleming[1,4,5]*

1 School of Medicine, National University of Ireland, Galway, Ireland.
2 Discipline of Microbiology, School of Natural Sciences, National University of Ireland, Galway, Ireland.
3 APC Microbiome, Ireand.
4 Luxembourg Centre for Systems Biomedicine, University of Luxembourg, Campus Belval, Esch-sur-Alzette, L-4362, Luxembourg.
4 Department of Psychiatry and Psychotherapy, University Medicine Greifswald, Greifswald, D-17489, Germany.
5 Division of Analytical Biosciences, Leiden Academic Centre for Drug Research, Faculty of Science, University of Leiden, Leiden, 2333, The Netherlands.
*Correspondence to: I.T. (ines.thiele@nuigalway.ie) and R.M.T.F. (ronan.mt.fleming@gmail.com)
†Current address: Department of Chemical Engineering, and Initiative for Biological Systems Engineering (IBSE), Indian Institute of Technology, Madras, Chennai, India

Appendix Table of Contents

Supplemental material

Figure S1. A. Flux span of the unconstrained (left) and physiologically (right) constrained Harvey model. B. Degree of functional redundancy in the WBM models.

Figure S2. Microbiome project data of 149 shotgun metagenomic samples of healthy individuals (Peterson et al, 2009) mapped onto the 773 gut microbial reconstructions (AGORA) (Magnusdottir et al, 2017).

Figure S3. Strain-level contributions to overall amino acid and neurotransmitter production in 149 personalized microbiomes without the host.

Figure S4. Dependence of liver sulfotransferase flux on the abundance of the *Clostridioides* genus in 149 personalized WBM models.

A

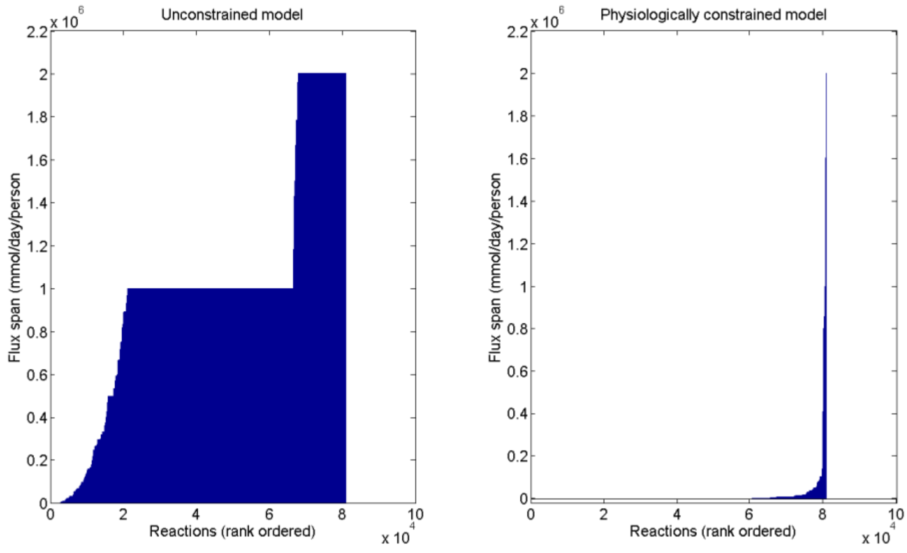

B

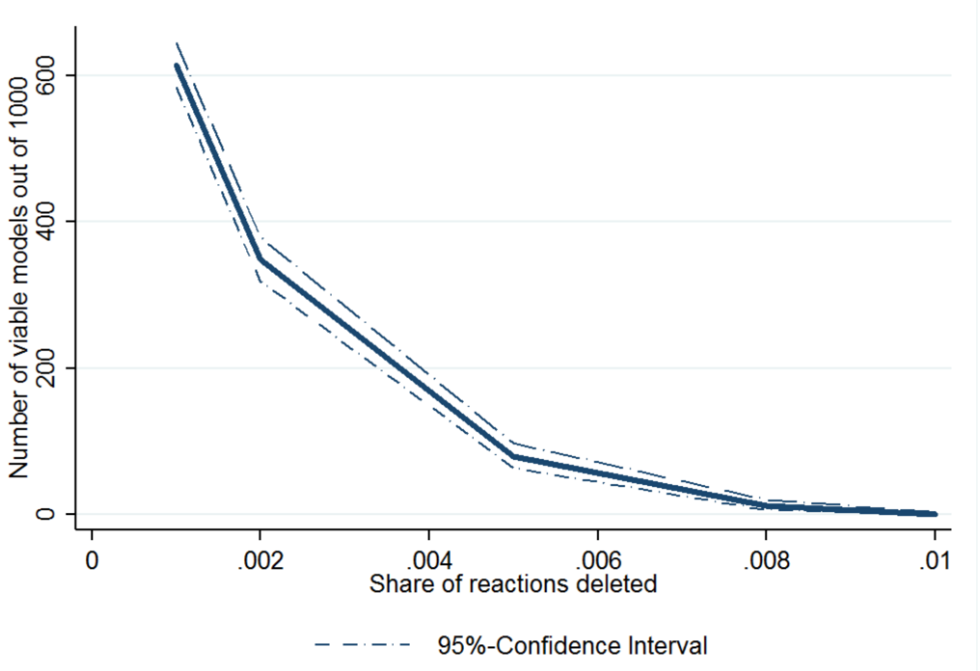

Figure S1. A. Flux span of the unconstrained (left) and physiologically (right) constrained Harvey model. Flux variability analysis was performed on all model reactions to calculate the flux span of each reaction ($fluxSpan_i = v_{max,i} - v_{min,i}$). The addition of the physiological constraints on 12.5% of the model reactions, resulted in a reduction of median flux span from 1,000,000 mmol/day/person (**left**) to 9.11 mmol/day/person (**right**). **B.** Degree of functional redundancy in the WBM models. We removed random subsets of reactions from the male WBM model and checked the feasibility of a unit flux through the whole-body maintenance reaction (1000 experiments per random reaction subset). We found that there was an exponential drop in the number of feasible models as the fraction of reactions removed was increased.

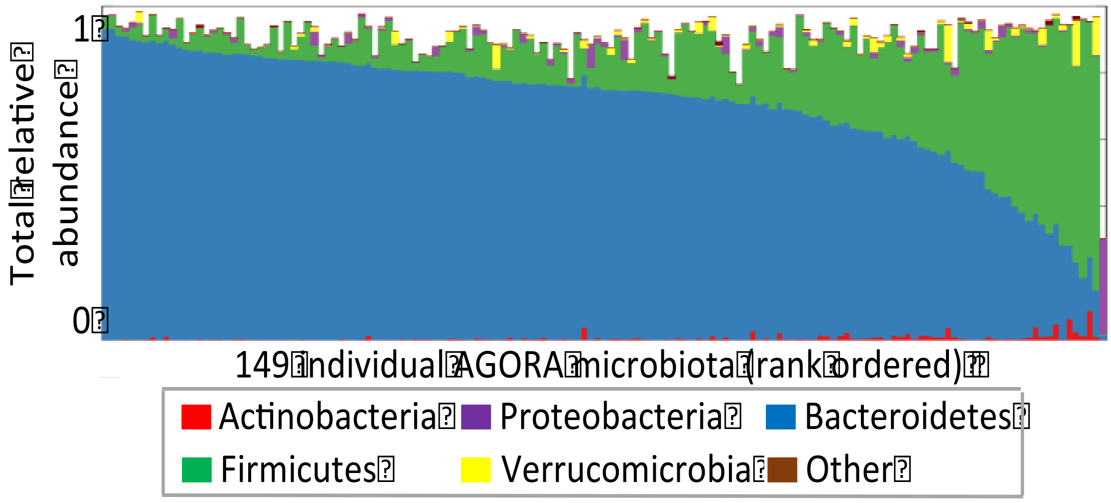

Figure S2. Microbiome project data of 149 shotgun metagenomic samples of healthy individuals (Peterson et al, 2009) mapped onto the 773 gut microbial reconstructions (AGORA) (Magnusdottir et al, 2017). On average, AGORA captures 131 +/- 19 microbes per HMP individual, accounting for 91 +/- 7% of the relative abundance.

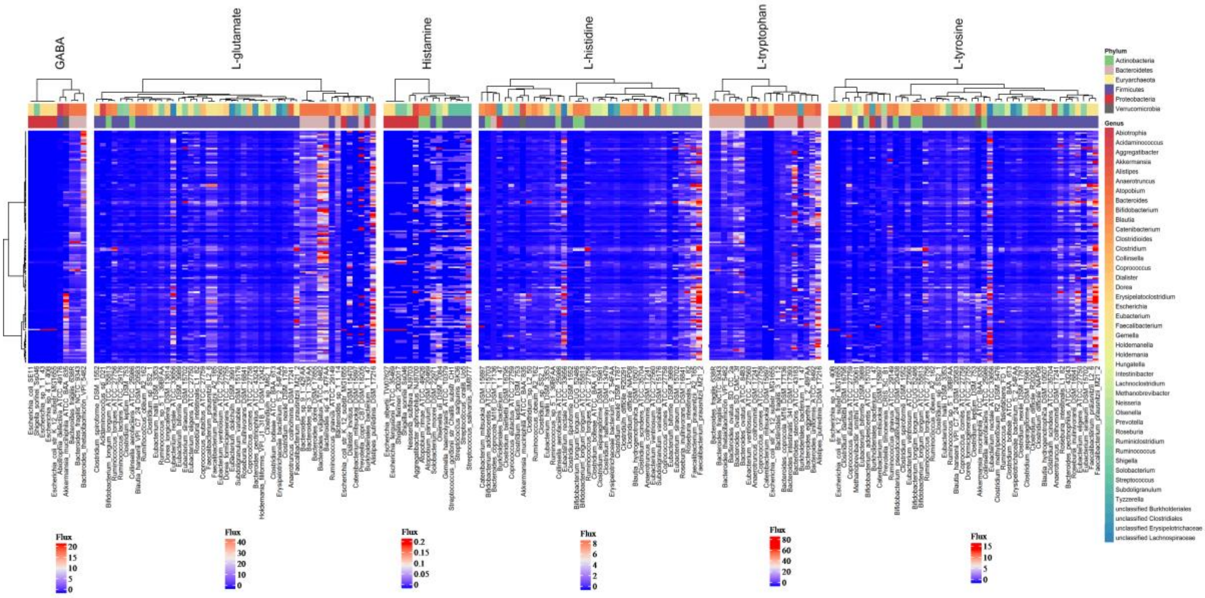

Figure S3. Strain-level contributions to overall amino acid and neurotransmitter production in 149 personalized microbiomes without the host. Strain-level contributions in 149 personalized microbiome models (without the WBM models) to the overall production of GABA, histamine, L-glutamate, L-histidine, L-tryptophan, and L-tyrosine. The contributions were determined by computing the maximal secretion flux in mmol/day/person through the internal exchange reaction of each strain for the respective metabolite. The columns show strain to metabolite contributions annotation by class and phylum, and the rows show the 149 microbiomes.

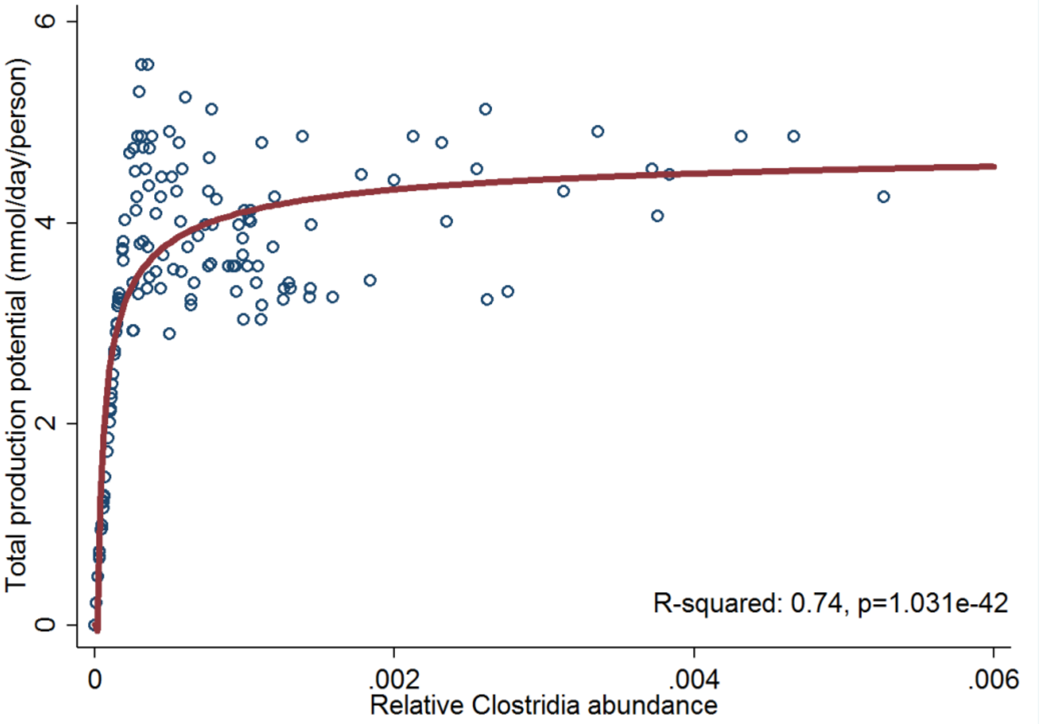

Figure S4. Dependence of liver sulfotransferase flux on the abundance of the *Clostridioides* genus in 149 personalized WBM models. The flux for p-cresol sulfate formation by the liver sulfotransferase (mmol/person/day) is plotted against the relative abundance of the *Clostridioides* genus in 149 personalized WBM models. The *Clostridioides* genus includes *Clostridioides difficile*, which produces p-cresol. Regression line and R-squared value from linear regression with fractional polynomials, p-value from corresponding likelihood ration test.

References

Magnusdottir S, Heinken A, Kutt L, Ravcheev DA, Bauer E, Noronha A, Greenhalgh K, Jager C, Baginska J, Wilmes P, Fleming RM, Thiele I (2017) Generation of genome-scale metabolic reconstructions for 773 members of the human gut microbiota. *Nat Biotechnol* **35:** 81-89

Peterson J, Garges S, Giovanni M, McInnes P, Wang L, Schloss JA, Bonazzi V, McEwen JE, Wetterstrand KA, Deal C, Baker CC, Di Francesco V, Howcroft TK, Karp RW, Lunsford RD, Wellington CR, Belachew T, Wright M, Giblin C, David H et al (2009) The NIH Human Microbiome Project. *Genome Res* **19:** 2317-2323
