# Supplementary material for: Personalized whole‐body models integrate metabolism, physiology, and the gut microbiome
Source: Mol Syst Biol. 2020 May 28;16(5):e8982. doi: 10.15252/msb.20198982 (PMC7285886; doi:10.15252/msb.20198982)
Supplement: Supplementary file 22 — Dataset EV1 [file MSB-16-e8982-s022.zip › PSCM_toolbox/PSCM_toolbox_doc/src/annotateModel.html]

Description of annotateModel


# annotateModel

## PURPOSE

**This function annotates a model with VMH reaction and metabolite**

## SYNOPSIS

**function model = annotateModel(model, annotateRxns, annotateMets,modelID,modelName,modelAnnotation)**

## DESCRIPTION

```
 This function annotates a model with VMH reaction and metabolite
 identifiers.

 function model = annotateModel(model, annotateRxns,annotateMets)

 INPUT
 model         Model structure
 annotateRxns  default: 1
 annotateMets  default: 1

 Optional:
 modelID       ID of model
 modelName     model Name

 OUTPUT
 model         Updated model structure

 Ines Thiele October 2019
```

## CROSS-REFERENCE INFORMATION

This function calls:


This function is called by:

- getOrgansFromHarvey This function cuts the organs from the whole-body metabolic model. Note that the different
- annotateHH annotate Harvey and Harvetta
- annotateOrganAtlas annotate OrganAtlas

## SOURCE CODE

```
0001 function model = annotateModel(model, annotateRxns, annotateMets,modelID,modelName,modelAnnotation)
0002 % This function annotates a model with VMH reaction and metabolite
0003 % identifiers.
0004 %
0005 % function model = annotateModel(model, annotateRxns,annotateMets)
0006 %
0007 % INPUT
0008 % model         Model structure
0009 % annotateRxns  default: 1
0010 % annotateMets  default: 1
0011 %
0012 % Optional:
0013 % modelID       ID of model
0014 % modelName     model Name
0015 %
0016 % OUTPUT
0017 % model         Updated model structure
0018 %
0019 % Ines Thiele October 2019
0020 
0021 if ~exist('annotateRxns','var')
0022     annotateRxns = 1;
0023 end
0024 
0025 if ~exist('annotateMets','var')
0026     annotateMets = 1;
0027 end
0028 
0029 if exist('modelID','var')
0030     model.modelID = modelID;
0031 end
0032 
0033 if exist('modelID','var')
0034     model.modelName = modelName;
0035 end
0036 if exist('modelAnnotation','var')
0037     model.modelAnnotation = modelAnnotation;
0038 end
0039 %% read input files
0040 if annotateRxns == 1
0041     % load reactions from VMH
0042     VMH_rxns = tdfread('VMH_Reactions.tsv');
0043 end
0044 
0045 if annotateMets == 1
0046     % load metabolites from VMH
0047     VMH_mets = tdfread('VMH_Metabolites.tsv');
0048 end
0049 %% reactions
0050 if annotateRxns == 1
0051     % redefine fields that should be updated
0052     %model.rxnNames = {};
0053     %model.rxnNames(1:length(model.rxns),1) = {''};
0054     model.rxnECNumbers = {};
0055     model.rxnECNumbers(1:length(model.rxns),1) = {''};
0056     %model.subSystems = {};
0057     %model.subSystems(1:length(model.rxns),1) = {''};
0058     model.rxnMetaNetXID = {};
0059     model.rxnMetaNetXID(1:length(model.rxns),1) = {''};
0060     model.rxnKEGGID = {};
0061     model.rxnKEGGID(1:length(model.rxns),1) = {''};
0062     model.rxnSBOTerms = {};
0063     model.rxnSBOTerms(1:length(model.rxns),1) = {''};
0064     model.rxnSEEDID = {};
0065     model.rxnSEEDID(1:length(model.rxns),1) = {''};
0066     
0067     VMH_rxns2.abbreviation = strcat('_',VMH_rxns.abbreviation);
0068     [a,remDash] = strtok(model.rxns,'_');
0069     [remDashBrak,rem2] = strtok(remDash,'['); % remove [
0070     [remBrak] = strtok(model.rxns,'['); % remove [
0071     [remDBrak,rem2] = strtok(model.rxns,'_['); % remove [
0072     for i = 1 : length(VMH_rxns.abbreviation)
0073         m =[];
0074         % check for organ in front and [
0075         tokVMH = strtok(VMH_rxns2.abbreviation(i,:),'['); % remove [
0076         tokVMH = strtok(tokVMH,' '); % remove space
0077         m = strmatch(tokVMH,remDash,'exact');
0078         if isempty(m)
0079             % no organ in front but [
0080             tokVMH = strtok(VMH_rxns.abbreviation(i,:),'['); % remove [
0081             m = strmatch(tokVMH,remBrak,'exact');
0082             if isempty(m)
0083                 m = strmatch(tokVMH,remDBrak,'exact');
0084             end
0085         end
0086         if ~isempty(m)
0087             model.rxnECNumbers(m,1) = cellstr(VMH_rxns.ecnumber(i,:));
0088             model.rxnNames(m,1) = cellstr(VMH_rxns.description(i,:));
0089             model.subSystems(m,1) = cellstr(regexprep(VMH_rxns.subsystem(i,:),'"',''));
0090             model.rxnMetaNetXID(m,1) = cellstr(VMH_rxns.metanetx(i,:));
0091             model.rxnKEGGID(m,1) = cellstr(VMH_rxns.keggId(i,:));
0092             model.rxnSEEDID(m,1) = cellstr(VMH_rxns.seed(i,:));
0093             % annotate reaction SBO terms:
0094             if  ~isempty(strfind(VMH_rxns.abbreviation(i,:),'biomass')) ||  ~isempty(strfind(VMH_rxns.abbreviation(i,:),'Biomass'))
0095                 % biomass reaction
0096                 model.rxnSBOTerms(m,1) = cellstr('SBO:0000629');
0097                 % Exchange reaction
0098             elseif ~isempty(strmatch('EX_',VMH_rxns.abbreviation(i,:))) ||  ~isempty(strmatch('Ex_',VMH_rxns.abbreviation(i,:)))
0099                 model.rxnSBOTerms(m,1) = cellstr('SBO:0000627');
0100             elseif ~isempty(strmatch('DM_',VMH_rxns.abbreviation(i,:)))
0101                 model.rxnSBOTerms(m,1) = cellstr('SBO:0000628');
0102             elseif  ~isempty(strmatch('sink_',VMH_rxns.abbreviation(i,:)))
0103                 model.rxnSBOTerms(m,1) = cellstr('SBO:0000632');
0104             elseif  ~isempty(strmatch('Transport',regexprep(VMH_rxns.subsystem(i,:),'"','')))
0105                 model.rxnSBOTerms(m,1) = cellstr('SBO:0000185');
0106             elseif  ~isempty(strmatch('Exchange',VMH_rxns.subsystem(i,:)))
0107                 model.rxnSBOTerms(m,1) = cellstr('SBO:0000628');
0108             else % metabolicx reaction
0109                 model.rxnSBOTerms(m,1) = cellstr('SBO:0000176');
0110             end
0111         end
0112         
0113     end
0114 end
0115 % check for biomass reaction separately again in case that the named one
0116 % was not in the vmh file
0117 BM1 = strmatch('biomass',model.rxns);
0118 BM2 = strmatch('Biomass',model.rxns);
0119 BM3 = strmatch('bio',model.rxns);
0120 BM = unique([BM1;BM2;BM3]);
0121 for i = 1 : length(BM)
0122     model.rxnSBOTerms(BM(i),1) = cellstr('SBO:0000629');
0123 end
0124 
0125 Sink = strmatch('sink_',model.rxns);
0126 for i = 1 : length(Sink)
0127     model.rxnSBOTerms(Sink(i),1) = cellstr('SBO:0000632');
0128 end
0129 
0130 % annotate missing transport reactions based on subSystem with SBO term
0131 for i = 1:length(model.rxns)
0132     if ~isempty(strmatch('Transport',model.subSystems(i)))
0133         model.rxnSBOTerms(i,1) = cellstr('SBO:0000185');
0134     elseif ~isempty(find(~cellfun(@isempty,strfind(model.rxns,'EX_'))))
0135         model.rxnSBOTerms(i,1) = cellstr('SBO:0000628');
0136     elseif ~isempty(find(~cellfun(@isempty,strfind(model.rxns,'DM_'))))
0137         model.rxnSBOTerms(i,1) = cellstr('SBO:0000627');
0138     elseif  ~isempty(strmatch('Demand',model.subSystems(i)))
0139         model.rxnSBOTerms(i,1) = cellstr('SBO:0000185');
0140     end
0141 end
0142 
0143 %% Metabolites
0144 if annotateMets == 1
0145     model.metNames= {};
0146     model.metNames(1:length(model.mets),1) = {''};
0147     model.metFormulas= {};
0148     model.metFormulas(1:length(model.mets),1) = {''};
0149     model.metCharges = [];
0150     model.metCharges(1:length(model.mets),1) = 0;
0151     model.metChEBIID={};
0152     model.metChEBIID(1:length(model.mets),1) = {''};
0153     model.metHMDBID= {};
0154     model.metHMDBID(1:length(model.mets),1) = {''};
0155     model.metInChIString= {};
0156     model.metInChIString(1:length(model.mets),1) = {''};
0157     model.metKEGGID= {};
0158     model.metKEGGID(1:length(model.mets),1) = {''};
0159     model.metSmiles= {};
0160     model.metSmiles(1:length(model.mets),1) = {''};
0161     model.metMetaNetXID= {};
0162     model.metMetaNetXID(1:length(model.mets),1) = {''};
0163     model.metPubChemID= {};
0164     model.metPubChemID(1:length(model.mets),1) = {''};
0165     model.metBiGGID= {};
0166     model.metBiGGID(1:length(model.mets),1) = {''};
0167     model.metBioCycID= {};
0168     model.metBioCycID(1:length(model.mets),1) = {''};
0169     model.metSEEDID= {};
0170     model.metSEEDID(1:length(model.mets),1) = {''};
0171     model.metSBOTerms= {};
0172     model.metSBOTerms(1:length(model.mets),1) = {''};
0173     model.metChemSpider= {};
0174     model.metChemSpider(1:length(model.mets),1) = {''};
0175     model.metInchiKey= {};
0176     model.metInchiKey(1:length(model.mets),1) = {''};
0177     
0178     % note that there are a few metabolites that are not present
0179     [a,rem] = strtok(model.mets,'_');
0180     [rem,rem2] = strtok(rem,'[');
0181     
0182     % add _ to Recon3D.mets
0183     VMH_mets2 = VMH_mets;
0184     VMH_mets2.abbreviation = strcat('_',VMH_mets.abbreviation);
0185     % match both metabolite lists
0186     [LIA,LOCB] = ismember(rem,VMH_mets2.abbreviation);
0187     for i = 1 : length(LOCB)
0188         if LOCB(i) >0 && ~strcmp(a(i),'slack')
0189             model.metCharges(i,1) = VMH_mets2.charge(LOCB(i),:);
0190             model.metFormulas(i,1) = cellstr(VMH_mets2.chargedFormula(LOCB(i),:));
0191             model.metNames(i,1) =  cellstr(VMH_mets2.fullName(LOCB(i),:));
0192             if ~isempty(regexprep(VMH_mets2.cheBlId(LOCB(i),:),' ',''))% remove space in string
0193                 model.metChEBIID(i,1) =  cellstr(strcat('CHEBI:',num2str(VMH_mets2.cheBlId(LOCB(i),:))));
0194             end
0195             model.metHMDBID(i,1) =  cellstr(VMH_mets2.hmdb(LOCB(i),:));
0196             model.metInChIString(i,1) =  cellstr(regexprep(VMH_mets2.inchiString(LOCB(i),:),'"',''));
0197             model.metKEGGID(i,1) =  cellstr(VMH_mets2.keggId(LOCB(i),:));
0198             model.metSmiles(i,1) =  cellstr(VMH_mets2.smile(LOCB(i),:));
0199             model.metPubChemID(i,1) =  cellstr(num2str(VMH_mets2.pubChemId(LOCB(i),:)));
0200             model.metMetaNetXID(i,1) =  cellstr(VMH_mets2.metanetx(LOCB(i),:));
0201             model.metBiGGID(i,1) =  cellstr(VMH_mets2.biggId(LOCB(i),:));
0202             model.metBioCycID(i,1) =  cellstr(VMH_mets2.biocyc(LOCB(i),:));
0203             model.metSEEDID(i,1) =  cellstr(VMH_mets2.seed(LOCB(i),:));
0204             model.metSBOTerms(i,1) =  cellstr('SBO:0000247'); % simple molecule
0205             if ~isempty(VMH_mets2.chemspider(LOCB(i),:)) && ~isnan(VMH_mets2.chemspider(LOCB(i),:))
0206                 model.metChemSpider(i,1) =  cellstr(num2str(VMH_mets2.chemspider(LOCB(i),:)));
0207             end
0208             model.metInchiKey(i,1) =  cellstr(VMH_mets2.inchiKey(LOCB(i),:));
0209         end
0210     end
0211     % catch also those metabolites that have no organ_ in front of it
0212     [rem,rem2] = strtok(model.mets,'[');
0213     
0214     [LIA,LOCBa] = ismember(rem,VMH_mets.abbreviation);
0215     for i = 1 : length(LOCBa)
0216         if LOCBa(i) >0 && ~strcmp(a(i),'slack')
0217             model.metCharges(i,1) = VMH_mets2.charge(LOCBa(i),:);
0218             model.metFormulas(i,1) = cellstr(VMH_mets2.chargedFormula(LOCBa(i),:));
0219             model.metNames(i,1) =  cellstr(VMH_mets2.fullName(LOCBa(i),:));
0220             if ~isempty(regexprep(VMH_mets2.cheBlId(LOCBa(i),:),' ',''))% remove space in string
0221                 model.metChEBIID(i,1) =  cellstr(strcat('CHEBI:',num2str(VMH_mets2.cheBlId(LOCBa(i),:))));
0222             end
0223             model.metHMDBID(i,1) =  cellstr(VMH_mets2.hmdb(LOCBa(i),:));
0224             model.metInChIString(i,1) =  cellstr(regexprep(VMH_mets2.inchiString(LOCBa(i),:),'"',''));
0225             model.metKEGGID(i,1) =  cellstr(VMH_mets2.keggId(LOCBa(i),:));
0226             model.metSmiles(i,1) =  cellstr(VMH_mets2.smile(LOCBa(i),:));
0227             model.metPubChemID(i,1) =  cellstr(num2str(VMH_mets2.pubChemId(LOCBa(i),:)));
0228             model.metMetaNetXID(i,1) =  cellstr(VMH_mets2.metanetx(LOCBa(i),:));
0229             model.metBiGGID(i,1) =  cellstr(VMH_mets2.biggId(LOCBa(i),:));
0230             model.metBioCycID(i,1) =  cellstr(VMH_mets2.biocyc(LOCBa(i),:));
0231             model.metSEEDID(i,1) =  cellstr(VMH_mets2.seed(LOCBa(i),:));
0232             model.metSBOTerms(i,1) =  cellstr('SBO:0000247'); % simple molecule
0233             if ~isempty(VMH_mets2.chemspider(LOCBa(i),:)) && ~isnan(VMH_mets2.chemspider(LOCBa(i),:))
0234                 model.metChemSpider(i,1) =  cellstr(num2str(VMH_mets2.chemspider(LOCBa(i),:)));
0235             end
0236             model.metInchiKey(i,1) =  cellstr(VMH_mets2.inchiKey(LOCBa(i),:));
0237         end
0238     end
0239 end
```

---

Generated on Thu 14-May-2020 13:05:49 by **m2html** © 2005
