# Supplementary material for: Personalized whole‐body models integrate metabolism, physiology, and the gut microbiome
Source: Mol Syst Biol. 2020 May 28;16(5):e8982. doi: 10.15252/msb.20198982 (PMC7285886; doi:10.15252/msb.20198982)
Supplement: Supplementary file 22 — Dataset EV1 [file MSB-16-e8982-s022.zip › PSCM_toolbox/PSCM_toolbox_doc/src/compareBounds2Models.html]

Description of compareBounds2Models


# compareBounds2Models

## PURPOSE

**This function compares the bounds between two models**

## SYNOPSIS

**function [R1\_missing,R2\_missing,R12\_bounds,R12\_bounds\_mismatch] = compareBounds2Models(model1,model2)**

## DESCRIPTION

```
 This function compares the bounds between two models
```

## CROSS-REFERENCE INFORMATION

This function calls:


This function is called by:

## SOURCE CODE

```
0001 function [R1_missing,R2_missing,R12_bounds,R12_bounds_mismatch] = compareBounds2Models(model1,model2)
0002 % This function compares the bounds between two models
0003 %
0004 %
0005 %
0006 
0007 
0008 % find the overlapping set of reaction between the two models
0009 R1 = model1.rxns;
0010 R2 = model2.rxns;
0011 R12 = unique([R1;R2]);
0012 R1_missing = '';
0013 R2_missing = '';
0014 R12_bounds_mismatch = {};
0015 mismatch_lb = 0;
0016 mismatch_ub = 0;
0017 cnt = 1;
0018 cntM = 1;
0019 for i = 1 : length(R12)
0020         r1 = find(ismember(R1,R12(i)));
0021         r2 = find(ismember(R2,R12{i}));
0022     if isempty(r1) % reaction does not exist in model1
0023         R1_missing = [R1_missing;R12{i}];
0024     elseif isempty(r2) % reaction does not exist in model2
0025         R2_missing = [R2_missing;R12{i}];
0026     else
0027         % compare bounds in both models
0028         % lower bound
0029         if model1.lb(r1) == model2.lb(r2)
0030             R12_bounds(cnt,1) = R12(i);
0031             R12_bounds{cnt,2} = 'identical lower bound';
0032             R12_bounds(cnt,3) = num2cell(model1.lb(r1));
0033             R12_bounds(cnt,4) = num2cell(model2.lb(r2));
0034             cnt = cnt+1;
0035         else
0036             R12_bounds_mismatch(cntM,1) = R12(i);
0037             R12_bounds_mismatch{cntM,2} = 'NOT identical lower bound';
0038             R12_bounds_mismatch(cntM,3) = num2cell(model1.lb(r1));
0039             R12_bounds_mismatch(cntM,4) = num2cell(model2.lb(r2));
0040             mismatch_lb = mismatch_lb +1;
0041             cntM = cntM+1;
0042         end
0043         if model1.ub(r1) == model2.ub(r2)
0044             R12_bounds(cnt,1) = R12(i);
0045             R12_bounds{cnt,2} = 'identical upper bound';
0046             R12_bounds(cnt,3) = num2cell(model1.ub(r1));
0047             R12_bounds(cnt,4) = num2cell(model2.ub(r2));
0048             cnt = cnt+1;
0049         else
0050             R12_bounds_mismatch(cntM,1) = R12(i);
0051             R12_bounds_mismatch{cntM,2} = 'NOT identical upper bound';
0052             R12_bounds_mismatch(cntM,3) = num2cell(model1.ub(r1));
0053             R12_bounds_mismatch(cntM,4) = num2cell(model2.ub(r2));
0054             mismatch_ub = mismatch_ub +1;
0055             cntM = cntM+1;
0056         end
0057     end
0058 end
0059 mismatch_lb
0060 mismatch_ub
0061
```

---

Generated on Thu 14-May-2020 13:05:49 by **m2html** © 2005
