# Supplementary material for: Personalized whole‐body models integrate metabolism, physiology, and the gut microbiome
Source: Mol Syst Biol. 2020 May 28;16(5):e8982. doi: 10.15252/msb.20198982 (PMC7285886; doi:10.15252/msb.20198982)
Supplement: Supplementary file 22 — Dataset EV1 [file MSB-16-e8982-s022.zip › PSCM_toolbox/PSCM_toolbox_doc/src/getBasicHarveyStats.html]

Description of getBasicHarveyStats


# getBasicHarveyStats

## PURPOSE

**This function compiles basic statistics on the male and female whole-body**

## SYNOPSIS

**function [TableHHStats] = getBasicHarveyStats(male,female)**

## DESCRIPTION

```
 This function compiles basic statistics on the male and female whole-body
 metabolic model

 function [TableHHStats] = getBasicHarveyStats(male,female)

 INPUT
 male          Model structure containing the male model
 female        Model structure containing the female model

 OUTPUT
 TableHHStats  Table containing the statistics
 
 Ines Thiele 2016- 2019
```

## CROSS-REFERENCE INFORMATION

This function calls:


This function is called by:

## SOURCE CODE

```
0001 function [TableHHStats] = getBasicHarveyStats(male,female)
0002 % This function compiles basic statistics on the male and female whole-body
0003 % metabolic model
0004 %
0005 % function [TableHHStats] = getBasicHarveyStats(male,female)
0006 %
0007 % INPUT
0008 % male          Model structure containing the male model
0009 % female        Model structure containing the female model
0010 %
0011 % OUTPUT
0012 % TableHHStats  Table containing the statistics
0013 %
0014 % Ines Thiele 2016- 2019
0015 
0016 % build table
0017 clear TableHHStats
0018 c = 1;
0019 
0020 SL = (find(~cellfun(@isempty,strfind(female.mets,'slack_'))));
0021 SL_female = SL;
0022 SL = (find(~cellfun(@isempty,strfind(male.mets,'slack_'))));
0023 SL_male = SL;
0024 
0025 TableHHStats{c , 1} = '';
0026 TableHHStats{c , 2} = 'Harvetta'; 
0027 TableHHStats{c , 3} = 'Harvey'; c = c+1;
0028 
0029 
0030 TableHHStats{c , 1} = 'Number of Reactions';      
0031 
0032 TableHHStats{c , 2} = num2str(length(female.rxns));
0033 TableHHStats{c , 3} = num2str(length(male.rxns)); c = c+1;
0034 
0035 
0036 TableHHStats{c , 1} = 'Number of Metabolites';      
0037 
0038 TableHHStats{c , 2} = num2str(length(female.mets)-length(SL_female));
0039 TableHHStats{c , 3} = num2str(length(male.mets)-length(SL_male)); c = c+1;
0040 
0041 TableHHStats{c , 1} = 'Number of Genes (transcripts)';      
0042 TableHHStats{c , 2} = num2str(length(female.genes));
0043 TableHHStats{c , 3} = num2str(length(male.genes)); c = c+1;
0044 
0045 TableHHStats{c , 1} = 'Number of Genes (unique)';     
0046 [a,b]=strtok(female.genes,'.');
0047 
0048 TableHHStats{c , 2} = num2str(length(unique(a)));
0049 [a,b]=strtok(male.genes,'.');
0050 TableHHStats{c , 3} = num2str(length(unique(a))); c = c+1;
0051 
0052 TableHHStats{c , 1} = 'Number of Subsystems';      
0053 
0054 % Feb 2020, moved this to loadPSCMfile
0055 % Oct 2017 % clean up final remaining subsystem names
0056 % female.subSystems(strmatch('Transport, endoplasmic reticular',female.subSystems,'exact'))={'Transport, endoplasmic reticulum'};
0057 % female.subSystems(strmatch('Arginine and Proline Metabolism',female.subSystems,'exact'))={'Arginine and proline Metabolism'};
0058 % female.subSystems(strmatch(' ',female.subSystems,'exact'))={'Miscellaneous'};
0059 %
0060 % male.subSystems(strmatch('Transport, endoplasmic reticular',male.subSystems,'exact'))={'Transport, endoplasmic reticulum'};
0061 % male.subSystems(strmatch('Arginine and Proline Metabolism',male.subSystems,'exact'))={'Arginine and proline Metabolism'};
0062 % male.subSystems(strmatch(' ',male.subSystems,'exact'))={'Miscellaneous'};
0063 
0064 TableHHStats{c , 2} = num2str(length(unique(female.subSystems))-1);%do not count dummy reactions
0065 TableHHStats{c , 3} = num2str(length(unique(male.subSystems))-1); c = c+1;%do not count dummy reactions
0066 
0067 TableHHStats{c , 1} = 'Blood compartment metabolites';
0068 BC_mets = (find(~cellfun(@isempty,strfind(female.mets,'[bc]'))));
0069 SL_female = (find(~cellfun(@isempty,strfind(female.mets,'slack_'))));
0070 BC_mets_female = setdiff(BC_mets,SL_female);
0071 
0072 BC_mets = (find(~cellfun(@isempty,strfind(male.mets,'[bc]'))));
0073 SL_male = (find(~cellfun(@isempty,strfind(male.mets,'slack_'))));
0074 BC_mets_male = setdiff(BC_mets,SL_male);
0075 
0076 TableHHStats{c , 2} = num2str(length(BC_mets_female));
0077 TableHHStats{c , 3} = num2str(length(BC_mets_male)); c = c+1;
0078 
0079 TableHHStats{c , 1} = 'Urine metabolites';
0080 U_mets = (find(~cellfun(@isempty,strfind(female.mets,'[u]'))));
0081 U_mets_female = setdiff(U_mets,SL_female);
0082 
0083 U_mets = (find(~cellfun(@isempty,strfind(male.mets,'[u]'))));
0084 U_mets_male = setdiff(U_mets,SL_male);
0085 
0086 TableHHStats{c , 2} = num2str(length(U_mets_female));
0087 TableHHStats{c , 3} = num2str(length(U_mets_male)); c = c+1;
0088 
0089 TableHHStats{c , 1} = 'Portal vein metabolites';
0090 BP_mets = (find(~cellfun(@isempty,strfind(female.mets,'[bp]'))));
0091 BP_mets_female = setdiff(BP_mets,SL_female);
0092 
0093 BP_mets = (find(~cellfun(@isempty,strfind(male.mets,'[bp]'))));
0094 BP_mets_male = setdiff(BP_mets,SL_male);
0095 
0096 TableHHStats{c , 2} = num2str(length(BP_mets_female));
0097 TableHHStats{c , 3} = num2str(length(BP_mets_male)); c = c+1;
0098 
0099 TableHHStats{c , 1} = 'Bile duct metabolites';
0100 
0101 BD_mets = (find(~cellfun(@isempty,strfind(female.mets,'[bd]'))));
0102 BD_mets_female = setdiff(BD_mets,SL_female);
0103 
0104 BD_mets = (find(~cellfun(@isempty,strfind(male.mets,'[bd]'))));
0105 BD_mets_male = setdiff(BD_mets,SL_male);
0106 
0107 TableHHStats{c , 2} = num2str(length(BD_mets_female));
0108 TableHHStats{c , 3} = num2str(length(BD_mets_male)); c = c+1;
0109 
0110 TableHHStats{c , 1} = 'CSF metabolites';
0111 
0112 CSF_mets = (find(~cellfun(@isempty,strfind(female.mets,'[csf]'))));
0113 CSF_mets_female = setdiff(CSF_mets,SL_female);
0114 
0115 CSF_mets = (find(~cellfun(@isempty,strfind(male.mets,'[csf]'))));
0116 CSF_mets_male = setdiff(CSF_mets,SL_male);
0117 
0118 TableHHStats{c , 2} = num2str(length(CSF_mets_female));
0119 TableHHStats{c , 3} = num2str(length(CSF_mets_male)); c = c+1;
0120 
0121 TableHHStats{c , 1} = 'Diet metabolites';
0122 
0123 D_mets = (find(~cellfun(@isempty,strfind(female.mets,'[d]'))));
0124 D_mets_female = setdiff(D_mets,SL_female);
0125 D_mets = (find(~cellfun(@isempty,strfind(male.mets,'[d]'))));
0126 D_mets_male = setdiff(D_mets,SL_male);
0127 
0128 TableHHStats{c , 2} = num2str(length(D_mets_female));
0129 TableHHStats{c , 3} = num2str(length(D_mets_male)); c = c+1;
0130 
0131 TableHHStats{c , 1} = 'Fecal metabolites';
0132 
0133 Fe_mets = (find(~cellfun(@isempty,strfind(female.mets,'[fe]'))));
0134 Fe_mets_female = setdiff(Fe_mets,SL_female);
0135 
0136 Fe_mets = (find(~cellfun(@isempty,strfind(male.mets,'[fe]'))));
0137 Fe_mets_male = setdiff(Fe_mets,SL_male);
0138 
0139 TableHHStats{c , 2} = num2str(length(Fe_mets_female));
0140 TableHHStats{c , 3} = num2str(length(Fe_mets_male)); c = c+1;
0141 
0142 
0143 TableHHStats{c , 1} = 'Sweat metabolites';
0144 
0145 Fe_mets = (find(~cellfun(@isempty,strfind(female.mets,'[sw]'))));
0146 Fe_mets_female = setdiff(Fe_mets,SL_female);
0147 
0148 Fe_mets = (find(~cellfun(@isempty,strfind(male.mets,'[sw]'))));
0149 Fe_mets_male = setdiff(Fe_mets,SL_male);
0150 
0151 TableHHStats{c , 2} = num2str(length(Fe_mets_female));
0152 TableHHStats{c , 3} = num2str(length(Fe_mets_male)); c = c+1;
0153 
0154 
0155 TableHHStats{c , 1} = 'Air metabolites';
0156 
0157 A_mets = (find(~cellfun(@isempty,strfind(female.mets,'[a]'))));
0158 A_mets_female = setdiff(A_mets,SL_female);
0159 
0160 A_mets = (find(~cellfun(@isempty,strfind(male.mets,'[a]'))));
0161 A_mets_male = setdiff(A_mets,SL_male);
0162 
0163 TableHHStats{c , 2} = num2str(length(A_mets_female));
0164 TableHHStats{c , 3} = num2str(length(A_mets_male)); c = c+1;
0165 
0166 
0167 TableHHStats{c , 1} = 'Milk metabolites';
0168 
0169 Fe_mets = (find(~cellfun(@isempty,strfind(female.mets,'[mi]'))));
0170 Fe_mets_female = setdiff(Fe_mets,SL_female);
0171 
0172 Fe_mets = (find(~cellfun(@isempty,strfind(male.mets,'[mi]'))));
0173 Fe_mets_male = setdiff(Fe_mets,SL_male);
0174 
0175 TableHHStats{c , 2} = num2str(length(Fe_mets_female));
0176 TableHHStats{c , 3} = num2str(length(Fe_mets_male)); c = c+1;
```

---

Generated on Thu 14-May-2020 13:05:49 by **m2html** © 2005
