# Supplementary material for: Personalized whole‐body models integrate metabolism, physiology, and the gut microbiome
Source: Mol Syst Biol. 2020 May 28;16(5):e8982. doi: 10.15252/msb.20198982 (PMC7285886; doi:10.15252/msb.20198982)
Supplement: Supplementary file 22 — Dataset EV1 [file MSB-16-e8982-s022.zip › PSCM_toolbox/PSCM_toolbox_doc/src/linearRegression.html]

Description of linearRegression


# linearRegression

## PURPOSE

**This function calculates the linear regression in the form of y = a0 + a1\*x**

## SYNOPSIS

**function [a0,a1,Rsqr,Residuals] = linearRegression(x,y)**

## DESCRIPTION

```
 This function calculates the linear regression in the form of y = a0 + a1*x
 
 function [a0,a1,Rsqr,Residuals] = linearRegression(x,y)

 INPUT
 x             Values for the explanatory variable x in y = a0 + a1*x
 y             Values for the dependent variable y in y = a0 + a1*x

 OUTPUT
 a0            Intercept
 a1            Slope of the line
 Rsqr          Square of the correlation coefficient
 Residuals     Regression residuals, provides an objective measure of the
               goodness of fit of the linear regression equation
 
 Ines Thiele 2018
```

## CROSS-REFERENCE INFORMATION

This function calls:


This function is called by:

## SOURCE CODE

```
0001 function [a0,a1,Rsqr,Residuals] = linearRegression(x,y)
0002 % This function calculates the linear regression in the form of y = a0 + a1*x
0003 %
0004 % function [a0,a1,Rsqr,Residuals] = linearRegression(x,y)
0005 %
0006 % INPUT
0007 % x             Values for the explanatory variable x in y = a0 + a1*x
0008 % y             Values for the dependent variable y in y = a0 + a1*x
0009 %
0010 % OUTPUT
0011 % a0            Intercept
0012 % a1            Slope of the line
0013 % Rsqr          Square of the correlation coefficient
0014 % Residuals     Regression residuals, provides an objective measure of the
0015 %               goodness of fit of the linear regression equation
0016 %
0017 % Ines Thiele 2018
0018 
0019 
0020 %
0021 x = [ones(length(x),1) x];
0022 b = x\y;
0023 yCalc2 = x*b;
0024 % R2
0025 Rsqr = 1 - sum((y - yCalc2).^2)/sum((y - mean(y)).^2);
0026 a0 = b(1);
0027 a1 = b(2);
0028 Residuals = (y - yCalc2);
```

---

Generated on Thu 14-May-2020 13:05:49 by **m2html** © 2005
