# Supplementary material for: Personalized whole‐body models integrate metabolism, physiology, and the gut microbiome
Source: Mol Syst Biol. 2020 May 28;16(5):e8982. doi: 10.15252/msb.20198982 (PMC7285886; doi:10.15252/msb.20198982)
Supplement: Supplementary file 22 — Dataset EV1 [file MSB-16-e8982-s022.zip › PSCM_toolbox/PSCM_toolbox_doc/src/compareMaleFemale.html]

Description of compareMaleFemale


# compareMaleFemale

## PURPOSE

**This function compares basic features of the male and female whole-body**

## SYNOPSIS

**function [ResultsMaleFemale] = compareMaleFemale(male,female)**

## DESCRIPTION

```
 This function compares basic features of the male and female whole-body
 metabolic models

 [ResultsMaleFemale] = compareMaleFemale(male,female)

 INPUT
 male                  model structure (male whole-body metabolic model)
 female                model structure (female whole-body metabolic model)

 OUTPUT
 ResultsMaleFemale     structure containing the basic differences and
                       commenalities between male and female model

 Ines Thiele 2017
```

## CROSS-REFERENCE INFORMATION

This function calls:


This function is called by:

## SOURCE CODE

```
0001 function [ResultsMaleFemale] = compareMaleFemale(male,female)
0002 % This function compares basic features of the male and female whole-body
0003 % metabolic models
0004 %
0005 % [ResultsMaleFemale] = compareMaleFemale(male,female)
0006 %
0007 % INPUT
0008 % male                  model structure (male whole-body metabolic model)
0009 % female                model structure (female whole-body metabolic model)
0010 %
0011 % OUTPUT
0012 % ResultsMaleFemale     structure containing the basic differences and
0013 %                       commenalities between male and female model
0014 %
0015 % Ines Thiele 2017
0016 
0017 % reactions unique to male
0018 ResultsMaleFemale.MaleOnly = setdiff(male.rxns,female.rxns);
0019 ResultsMaleFemale.FemaleOnly = setdiff(female.rxns,male.rxns);
0020 ResultsMaleFemale.BothGender = intersect(female.rxns,male.rxns);
0021 
0022 [maleOrgans]=unique(strtok(male.rxns,'_'));
0023 [femaleOrgans]=unique(strtok(female.rxns,'_'));
0024 
0025 for i = 1 : length(maleOrgans)
0026     ResultsMaleFemale.OrgansNumRxnMale(i,1) = length(strmatch(maleOrgans(i),male.rxns));   
0027     ResultsMaleFemale.OrgansNumRxnMale(i,2) = length(strmatch(maleOrgans(i),ResultsMaleFemale.MaleOnly));
0028     % fraction
0029     ResultsMaleFemale.OrgansNumRxnMale(i,3) = ResultsMaleFemale.OrgansNumRxnMale(i,2)/ResultsMaleFemale.OrgansNumRxnMale(i,1); 
0030 end
0031 
0032 for i = 1 : length(femaleOrgans)
0033     ResultsMaleFemale.OrgansNumRxnFemale(i,1) = length(strmatch(femaleOrgans(i),female.rxns));   
0034     ResultsMaleFemale.OrgansNumRxnFemale(i,2) = length(strmatch(femaleOrgans(i),ResultsMaleFemale.FemaleOnly));
0035     % fraction
0036     ResultsMaleFemale.OrgansNumRxnFemale(i,3) = ResultsMaleFemale.OrgansNumRxnFemale(i,2)/ResultsMaleFemale.OrgansNumRxnFemale(i,1); 
0037 end
0038 
0039 ResultsMaleFemale.maleOrgans = maleOrgans;
0040 ResultsMaleFemale.femaleOrgans = femaleOrgans;
0041 
0042 %get subsystems for gall rxns
0043 FemaleSS = female.subSystems(find(ismember(female.rxns,ResultsMaleFemale.FemaleOnly(strmatch('Gall_',ResultsMaleFemale.FemaleOnly)))));
0044 ResultsMaleFemale.FemaleGallSSEnrich = unique(FemaleSS);
0045 for i = 1 : length(ResultsMaleFemale.FemaleGallSSEnrich)
0046     ResultsMaleFemale.FemaleGallSSEnrich{i,2} = num2str(length(strmatch(ResultsMaleFemale.FemaleGallSSEnrich{i},FemaleSS,'exact')));
0047 end
0048 MaleSS = male.subSystems(find(ismember(male.rxns,ResultsMaleFemale.MaleOnly(strmatch('Gall_',ResultsMaleFemale.MaleOnly)))));
0049 ResultsMaleFemale.MaleGallSSEnrich = unique(MaleSS);
0050 for i = 1 : length(ResultsMaleFemale.MaleGallSSEnrich)
0051     ResultsMaleFemale.MaleGallSSEnrich{i,2} = num2str(length(strmatch(ResultsMaleFemale.MaleGallSSEnrich{i},MaleSS,'exact')));
0052 end
0053 
0054 % unique biofluid exchange reactions
0055 
0056 ResultsMaleFemale.MaleOnlyBiofluid = ResultsMaleFemale.MaleOnly(find(~cellfun(@isempty,strfind(ResultsMaleFemale.MaleOnly,'_EX_'))));
0057 ResultsMaleFemale.FemaleOnlyBiofluid = ResultsMaleFemale.FemaleOnly(find(~cellfun(@isempty,strfind(ResultsMaleFemale.FemaleOnly,'_EX_'))));
0058
```

---

Generated on Thu 14-May-2020 13:05:49 by **m2html** © 2005
