# Supplementary material for: Personalized whole‐body models integrate metabolism, physiology, and the gut microbiome
Source: Mol Syst Biol. 2020 May 28;16(5):e8982. doi: 10.15252/msb.20198982 (PMC7285886; doi:10.15252/msb.20198982)
Supplement: Supplementary file 22 — Dataset EV1 [file MSB-16-e8982-s022.zip › PSCM_toolbox/PSCM_toolbox_doc/src/createModelNewCompartment.html]

Description of createModelNewCompartment


# createModelNewCompartment

## PURPOSE

**This function converts a two compartment metabolic model into a three compartment metabolic model model**

## SYNOPSIS

**function [modelComp] = createModelNewCompartment(model,OldComp,NewComp,NewCompName,LB,UB,RemoveExch)**

## DESCRIPTION

```
 This function converts a two compartment metabolic model into a three compartment metabolic model model
 
 function [modelComp] = createModelNewCompartment(model,OldComp,NewComp,NewCompName,LB,UB,RemoveExch)
 
 INPUT 
 model         Model structure
 OldComp       Name of the current (to be replicated) compartment to be
               replicated and added
 NewCompName   Name of new compartment e.g., 'lumen'
 LB            Value for Lower bound on new exchange compartment (default:-1000)
 UB            Value for Upper bound on new exchange compartment (default:1000)
 
 NewComp       Abrreviation for the new compartment,m e.g., 'lu'
 RemoveExch    Option (if set to 1) to remove the old compartment

 OUTPUT
 modelComp     Model with three compartments

 Ines Thiele 2016-2017
```

## CROSS-REFERENCE INFORMATION

This function calls:


This function is called by:

- combineHarveyMicrotiota This function combines harvey and a microbial community model
- runIEM\_HH This script predicts known biomarker metabolites in

## SOURCE CODE

```
0001 function [modelComp] = createModelNewCompartment(model,OldComp,NewComp,NewCompName,LB,UB,RemoveExch)
0002 % This function converts a two compartment metabolic model into a three compartment metabolic model model
0003 %
0004 % function [modelComp] = createModelNewCompartment(model,OldComp,NewComp,NewCompName,LB,UB,RemoveExch)
0005 %
0006 % INPUT
0007 % model         Model structure
0008 % OldComp       Name of the current (to be replicated) compartment to be
0009 %               replicated and added
0010 % NewCompName   Name of new compartment e.g., 'lumen'
0011 % LB            Value for Lower bound on new exchange compartment (default:-1000)
0012 % UB            Value for Upper bound on new exchange compartment (default:1000)
0013 %
0014 % NewComp       Abrreviation for the new compartment,m e.g., 'lu'
0015 % RemoveExch    Option (if set to 1) to remove the old compartment
0016 %
0017 % OUTPUT
0018 % modelComp     Model with three compartments
0019 %
0020 % Ines Thiele 2016-2017
0021 
0022 warning off
0023 if ~exist('LB','var')
0024     LB = -1000;
0025 end
0026 
0027 if ~exist('UB','var')
0028     UB = 1000;
0029 end
0030 
0031 if ~exist('RemoveExch','var')
0032     RemoveExch = 0;
0033 end
0034 % find exchange reactions
0035 cnt = 1;
0036 for t=1:length(model.rxns)
0037     if  strfind(model.rxns{t}, 'EX_')
0038         Ex_RxnsAll(cnt,1) =model.rxns(t); %make exchange reaction list
0039         cnt=cnt+1;
0040     elseif  strfind(model.rxns{t}, 'Ex_')
0041         Ex_RxnsAll(cnt,1) =model.rxns(t); %make exchange reaction list
0042         cnt=cnt+1;
0043     end
0044 end
0045 
0046 % duplicate all ExtRxns in a new compartment [i] for the New Compartment and
0047 % for new exchange reactions
0048 %a = printRxnFormulaOri(model, Ex_RxnsAll,[],[],[],[],false);
0049 a = printRxnFormula(model,'rxnAbbrList',Ex_RxnsAll,'printFlag',0,'lineChangeFlag',0,'metNameFlag',0,'fid',0,'directionFlag',0);
0050 
0051 aOri = a;
0052 modelComp = model;
0053 
0054 for i = 1 : length(Ex_RxnsAll)
0055     b = find(ismember(model.rxns,Ex_RxnsAll(i)));
0056     if ~isempty(strfind(a{i},strcat('[',OldComp,']'))) && length(find(model.S(:,b))) ==1
0057         % add new reactions to model
0058         [metaboliteList,stoichCoeffList] = parseRxnFormula(a{i});
0059         metaboliteListA = regexprep(metaboliteList,strcat('\[',OldComp,'\]'),strcat('\[',NewComp,'\]'));
0060         if UB == 0
0061             UBO = -1*LB;
0062             LBO = 0;
0063             revFlag = 0;
0064             RxnName = strcat(model.rxnNames{b},' (from ',NewCompName,'to ',strcat('[',OldComp,']'));
0065             modelComp = addReaction(modelComp,{strcat(Ex_RxnsAll{i},'_[',NewComp,']'),RxnName},[metaboliteListA metaboliteList],...
0066                 [-1 1], revFlag,LBO,UBO,0,strcat('Transport'),'',[],[],0,0);
0067         else
0068             metaboliteList = [metaboliteList metaboliteListA];
0069             stoichCoeffList = [-1 1];
0070             if LB < 0
0071                 revFlag = 1;
0072             else
0073                 revFlag = 0;
0074             end
0075             RxnName = strcat(model.rxnNames{b},' (from ',strcat('[',OldComp,']'),' to ',NewCompName,')');
0076             %replaced addReactionOri - Ronan
0077             modelComp = addReaction(modelComp,{strcat(Ex_RxnsAll{i},'_[',NewComp,']'),RxnName},metaboliteList,stoichCoeffList,...
0078                 revFlag,LB,UB,0,strcat('Transport'),'',[],[],0,0);
0079             %modelComp = addReaction(modelComp,{strcat(Ex_RxnsAll{i},'_[',NewComp,']'),RxnName},'metaboliteList',metaboliteList,'stoichCoeffList',stoichCoeffList,...
0080              %'lowerBound',LB,'upperBound',UB,'objectiveCoef',0,'subSystem',{'Transport'},'geneRule','','geneNameList',[],'systNameList',[],'checkDuplicate',0,'printLevel',0);
0081         end
0082     end
0083 end
0084 a=aOri;
0085 if RemoveExch == 1
0086     for i = 1 : length(Ex_RxnsAll)
0087         % remove original reactions
0088         b = find(ismember(modelComp.rxns,Ex_RxnsAll(i)));
0089         if ~isempty(strfind(a{i},strcat('[',OldComp,']'))) && length(find(modelComp.S(:,b))) ==1
0090             if isfield(modelComp,'rxnGeneMat')
0091             modelComp = rmfield(modelComp,'rxnGeneMat');
0092             end
0093             modelComp = removeRxns(modelComp,Ex_RxnsAll{i});
0094         end
0095     end
0096 end
```

---

Generated on Thu 14-May-2020 13:05:49 by **m2html** © 2005
