# Supplementary material for: Personalized whole‐body models integrate metabolism, physiology, and the gut microbiome
Source: Mol Syst Biol. 2020 May 28;16(5):e8982. doi: 10.15252/msb.20198982 (PMC7285886; doi:10.15252/msb.20198982)
Supplement: Supplementary file 22 — Dataset EV1 [file MSB-16-e8982-s022.zip › PSCM_toolbox/PSCM_toolbox_doc/src/calcOrganFract.html]

Description of calcOrganFract


# calcOrganFract

## PURPOSE

**This function extrapolates the organ weight fractions based on polynomials given in**

## SYNOPSIS

**function [organs,OrganWeight,OrganWeightFract,IndividualParameters] = calcOrganFract(model, IndividualParameters)**

## DESCRIPTION

```
 This function extrapolates the organ weight fractions based on polynomials given in
 http://www.ams.sunysb.edu/~hahn/psfile/pap_obesity.pdf (PMID 19267313), Table 3. Those ones that are not given are assumed to remain constant with weight
 using the fractions from the reference man and reference woman.

 [organs,OrganWeight,OrganWeightFract,IndividualParameters] = calcOrganFract(model, IndividualParameters)

 INPUT
 model                 Model structure
 IndividualParameters  Structure of individual parameters (sex, weight,
                       blood volume)
 
 OUTPUT
 organs                List of organs
 OrganWeight           List of organ weights (same order as organs)
 OrganWeightFract      List of organ weight fractions (same order as organs)
 IndividualParameters  Updated structure of individual parameters
 
 Ines Thiele Nov 2017
```

## CROSS-REFERENCE INFORMATION

This function calls:

- getOrganWeightFraction This script reads in the organ file and assign biomass\_maintenance coefficient according

This function is called by:

- perform\_BMR\_newData This script repeats the simulation described in Thiele et al., "Personalized whole-body models integrate metabolism, physiology, and the gut microbiome", Method section 3.9.2 Validation of the parameters in an independent data set.
- perform\_sensi\_BMR\_all This script repeats the simulation described in Thiele et al.,

## SOURCE CODE

```
0001 function [organs,OrganWeight,OrganWeightFract,IndividualParameters] = calcOrganFract(model, IndividualParameters)
0002 % This function extrapolates the organ weight fractions based on polynomials given in
0003 % http://www.ams.sunysb.edu/~hahn/psfile/pap_obesity.pdf (PMID 19267313), Table 3. Those ones that are not given are assumed to remain constant with weight
0004 % using the fractions from the reference man and reference woman.
0005 %
0006 % [organs,OrganWeight,OrganWeightFract,IndividualParameters] = calcOrganFract(model, IndividualParameters)
0007 %
0008 % INPUT
0009 % model                 Model structure
0010 % IndividualParameters  Structure of individual parameters (sex, weight,
0011 %                       blood volume)
0012 %
0013 % OUTPUT
0014 % organs                List of organs
0015 % OrganWeight           List of organ weights (same order as organs)
0016 % OrganWeightFract      List of organ weight fractions (same order as organs)
0017 % IndividualParameters  Updated structure of individual parameters
0018 %
0019 % Ines Thiele Nov 2017
0020 %
0021 
0022 % get sex from individual parameters
0023 sex = IndividualParameters.sex;
0024 
0025 % load reference organ weights/fractions from Ref man and Ref woman
0026 getOrganWeightFraction;
0027 RefOrganWeights = OrganWeight;
0028 RefOrganWeightFract = OrganWeightFract;
0029 RefBodyWeight = BodyWeight;
0030 RefOrganNames = OrganNames;
0031 
0032 % get individual parameters
0033 Wt = IndividualParameters.bodyWeight*1000;
0034 
0035 % IT - 27.02.2018 - I changed this default statement
0036 % see line 100 onwards - important for calc of platelets and red blood
0037 % cells
0038 BloodVolume = IndividualParameters.CardiacOutput; % given in ml
0039 %
0040 
0041 % pre-define variables
0042 OrganWeight = [];
0043 OrganWeightFract = [];
0044 organs = [];
0045 
0046 % define polynomials for organ weights based on body weight
0047 % based on polynomials given in
0048 % http://www.ams.sunysb.edu/~hahn/psfile/pap_obesity.pdf, Table 3
0049 %
0050 if strcmp(sex,'male')
0051     OF={
0052         'Brain' '1.41e-01' '-5.54e-06' '9.30e-11' '-6.83e-16' '1.80e-21' '0.0'
0053         'Heart' '6.32e-03' '-1.67e-08' '0.0' '0.0' '0.0' '0.0'
0054         'Kidney' '7.26e-03' '-6.69e-08' '3.33e-13' '0.0' '0.0' '0.0'
0055         'Liver' '4.25e-02' '-1.01e-06' '1.99e-11' '-1.66e-16' '4.83e-22' '0.0'
0056         'Lungs' '1.86e-02' '-4.55e-08' '0.0' '0.0' '0.0' '0.0'
0057         'Spleen' '3.12e-03' '-5.57e-09' '0.0' '0.0' '0.0' '0.0'
0058         'Agland' '8.04e-04' '-1.98e-08' '2.01e-13' '-6.11e-19' '0.0' '0.0'
0059         'Pancreas' '1.48e-03' '0.0' '0.0' '0.0' '0.0' '0.0'
0060         'Thymus' '3.70e-03' '-1.05e-07' '7.94e-13' '0.0' '0.0' '0.0'
0061         'Thyroidgland' '2.42e-04' '0.0' '0.0' '0.0' '0.0' '0.0'
0062         'Adipocytes' '1.61e-01' '-3.59e-06' '8.28e-11' '-3.57e-16' '4.73e-22' '0.0'
0063         'Muscle' '9.68e-02' '-3.32e-06' '1.83e-10' '-1.24e-15' '0.0' '0.0'
0064         'Skin' '1.03e-01' '-2.56e-06' '3.68e-11' '-2.58e-16' '8.62e-22' '-1.10e-27'
0065         'Blood' '8.97e-02' '-3.50e-07' '6.54e-13' '0.0' '0.0' '0.0'
0066         };
0067     
0068 elseif strcmp(sex,'female')
0069     OF={
0070         'Brain' '1.12e-01' '-3.33e-06' '4.30e-11' '-2.45e-16' '5.03e-22' '0.0'
0071         'Heart' '5.40e-03' '-1.07e-08' '0.0' '0.0' '0.0' '0.0'
0072         'Kidney' '7.56e-03' '-5.58e-08' '1.54e-13' '0.0' '0.0' '0.0'
0073         'Liver' '3.34e-02' '-1.89e-07' '5.34e-13' '0.0' '0.0' '0.0'
0074         'Lungs' '1.89e-02' '-5.94e-08' '0.0' '0.0' '0.0' '0.0'
0075         'Spleen' '2.96e-03' '-7.72e-09' '0.0' '0.0' '0.0' '0.0'
0076         'Agland' '8.04e-04' '-1.98e-08' '2.01e-13' '-6.11e-19' '0.0' '0.0'
0077         'Pancreas' '1.48e-03' '0.0' '0.0' '0.0' '0.0' '0.0'
0078         'Thymus' '3.70e-03' '-1.05e-07' '7.94e-13' '0.0' '0.0' '0.0'
0079         'Thyroidgland' '2.42e-04' '0.0' '0.0' '0.0' '0.0' '0.0'
0080         'Adipocytes' '1.84e-01' '-6.86e-06' '2.46e-10' '-2.11e-15' '7.58e-21' '-9.94e-27'
0081         'Muscle' '3.65e-02' '7.91e-06' '-5.74e-11' '0.0' '0.0' '0.0'
0082         'Skin' '9.81e-02' '-2.28e-06' '2.74e-11' '-1.58e-16' '4.30e-22' '-4.43e-28'
0083         'Blood' '8.97e-02' '-3.50e-07' '6.54e-13' '0.0' '0.0' '0.0'
0084         % these numbers come from Luebcke et al 2007
0085         % Postnatal Growth Considerations for PBPK Modeling
0086         'Breast' '0.01' '0.0' '0.0' '0.0' '0.0' '0.0'
0087         };
0088 end
0089 
0090 for i =1 : size(OF,1)
0091     x0= str2num(OF{i,2});
0092     x1=str2num(OF{i,3});
0093     x2=str2num(OF{i,4});
0094     x3=str2num(OF{i,5});
0095     x4=str2num(OF{i,6});
0096     x5=str2num(OF{i,7});
0097     % organ fraction for given weight
0098     OrganWeightFract(i,1) = x0 + x1*Wt + x2*Wt^2 + x3*Wt^3 + x4*Wt^4 + x5*Wt^5;
0099     % weight per organ for given weight
0100     OrganWeight(i,1) = OrganWeightFract(i,1)*Wt;
0101 end
0102 organs = OF(:,1);
0103 r = size(organs,1)+1;
0104 %% calculate blood cells
0105 
0106 BloodRow = strmatch('Blood',OF(:,1));
0107 BloodWeight = OrganWeight(BloodRow,1);
0108 
0109 % IT 27.02.2018 - after HH submission I changed the calculation of the
0110 % blood volume
0111 % density of plasma: 1.0506 kg/m3 at 37 degrees: http://clinchem.aaccjnls.org/content/20/5/615
0112 % => 1 l of blood = 1.0506 kg
0113 %
0114 BloodVolume = BloodWeight/1.0506; % in ml
0115 IndividualParameters.BloodVolume = BloodVolume;
0116 
0117 % WBC make about 1% of blood
0118 WBCWeight = 0.01*BloodWeight;
0119 % Lymphocytes    15-40% of White Blood Cells; assumed 30% of WBC weight
0120 LympWeight = 0.3*WBCWeight;
0121 % Bcells    9% of Lymphocytes
0122 organs{r} = 'Bcells';
0123 OrganWeight(r,1) = 0.09*LympWeight;
0124 OrganWeightFract(r,1) = OrganWeight(r,1)/Wt;
0125 r = r+1;
0126 % CD4Tcells    "45-75% of lymphocytes; 4 � 20% of leukocyte; assumed 15% of leukocytes"
0127 organs{r} = 'CD4Tcells';
0128 OrganWeight(r,1) = 0.15*WBCWeight;
0129 OrganWeightFract(r,1) = OrganWeight(r,1)/Wt;
0130 r = r+1;
0131 % CD8Tcells    2 � 11% of leukocytes; assumed 8%
0132 % Nkcells    Human and mouse NK cells constitute approximately 15% of all circulating lymphocytes
0133 organs{r} = 'Nkcells';
0134 OrganWeight(r,1) = 0.15*LympWeight;
0135 OrganWeightFract(r,1) = OrganWeight(r,1)/Wt;
0136 r = r+1;
0137 % Monocyte    Monocyte�(2-8% of peripheral WBCs); assumed 5%
0138 organs{r} = 'Monocyte';
0139 OrganWeight(r,1) = 0.05*WBCWeight;
0140 OrganWeightFract(r,1) = OrganWeight(r,1)/Wt;
0141 r = r+1;
0142 % Platelet    150,000 to 400,000/mm3; 1mm3 = 1e-6 l; 10 pg per platelet (wet weight); assumed 400k/mm3; --> 4g/l; --> 20g/5l
0143 organs{r} = 'Platelet';
0144 OrganWeight(r,1) = 4*BloodVolume/1000;%495g/l
0145 OrganWeightFract(r,1) = OrganWeight(r,1)/Wt;
0146 r = r+1;
0147 % RBC    The normal range in men is approximately 4.7 to 6.1 million cells/ul (microliter). The normal range in women range from 4.2 to 5.4 million cells/ul, according to NIH (National Institutes of Health) data. 27 pg dry weight of one RBC. Assumed 70% water in RBC --> 90 pg/RBC; --> 495g/l --> 2475 g/5l for male (assumed 5.5M) and 2050 g/l for female (assumed 4.5M)
0148 organs{r} = 'RBC';
0149 OrganWeight(r,1) = 495*BloodVolume/1000;%495g/l
0150 OrganWeightFract(r,1) = OrganWeight(r,1)/Wt;
0151 r = r+1;
0152 
0153 % now readjust organ weight fraction from all other organs not covered by
0154 % the polynomials and the equation based on the individual weight and the
0155 % it is assumed that the weight of these organs does not change with
0156 % increasing or decreasing body weight hence the fractions will be adjusted
0157 % to the new body weight
0158 
0159 % get all organs defined in the model
0160 ObjectiveComponents = model.rxns(find(~cellfun(@isempty,strfind(model.rxns,'_biomass_maintenance'))));
0161 ObjectiveComponents(end+1) ={'sIEC_biomass_reactionIEC01b'};
0162 OrgansInModel = strtok(ObjectiveComponents,'_');
0163 
0164 % organs not captured by polynomials
0165 MissingOrgans = setdiff(unique(OrgansInModel), organs);
0166 % their original weight:
0167 
0168 for i = 1 : length(MissingOrgans)
0169     organs{r} = MissingOrgans{i};
0170     X = find(ismember(RefOrganNames,MissingOrgans{i}));
0171     OrganWeight(r,1) = RefOrganWeights(X);
0172     OrganWeightFract(r,1) =  RefOrganWeights(X)/Wt;
0173     r = r+1;
0174 end
0175 
0176 IndividualParameters.OrgansWeightsRefMan = IndividualParameters.OrgansWeights;
0177 IndividualParameters = rmfield(IndividualParameters,'OrgansWeights');
0178 for i = 1 : length(organs)
0179     IndividualParameters.OrgansWeights(i,:) = {organs{i}, num2str(OrganWeight(i)), num2str(OrganWeightFract(i)*100)};
0180 end
```

---

Generated on Thu 14-May-2020 13:05:49 by **m2html** © 2005
