# Supplementary material for: Personalized whole‐body models integrate metabolism, physiology, and the gut microbiome
Source: Mol Syst Biol. 2020 May 28;16(5):e8982. doi: 10.15252/msb.20198982 (PMC7285886; doi:10.15252/msb.20198982)
Supplement: Supplementary file 22 — Dataset EV1 [file MSB-16-e8982-s022.zip › PSCM_toolbox/PSCM_toolbox_doc/src/adjustWholeBodyRxnCoeff.html]

Description of adjustWholeBodyRxnCoeff


# adjustWholeBodyRxnCoeff

## PURPOSE

**[model] = adjustWholeBodyRxnCoeff(model, listOrgan, listCoeff)**

## SYNOPSIS

**function [model] = adjustWholeBodyRxnCoeff(model, listOrgan, listCoeff)**

## DESCRIPTION

```
 [model] = adjustWholeBodyRxnCoeff(model, listOrgan, listCoeff)

 This function adjusts the coefficients of the whole-body biomass
 maintenance (WBM) reaction. The WBM reaction contains each organ present
 in the whole-body metabolic reconstructions. For each organ, the
 stoichiometric coefficients represent the fractional weight contribution
 of the respective organ to the whole body weight. These coefficients can
 be updated to reflect individual specific body contributions. E.g., in
 obese individuals the ratio of muscle and adipose tissue is different
 than in a normal BMI individual. Hence, they can be updated with this
 function.

 INPUT
 model         whole-body metabolic model
 listOrgan     List of organs, whose stoichiometric coefficient should be
               updated
 listCoeff     List of coefficients that replace current ones in the WBM
               reaction (order must match the order of organs in
               ListOrgan)

 OUTPUT
 model         whole-body metabolic model with adjusted stoichiometric
               coefficients in the whole-body metabolic model

 Ines Thiele, 2012 - 2020
```

## CROSS-REFERENCE INFORMATION

This function calls:


This function is called by:

- perform\_BMR\_newData This script repeats the simulation described in Thiele et al., "Personalized whole-body models integrate metabolism, physiology, and the gut microbiome", Method section 3.9.2 Validation of the parameters in an independent data set.
- perform\_sensi\_BMR\_all This script repeats the simulation described in Thiele et al.,

## SOURCE CODE

```
0001 function [model] = adjustWholeBodyRxnCoeff(model, listOrgan, listCoeff)
0002 % [model] = adjustWholeBodyRxnCoeff(model, listOrgan, listCoeff)
0003 %
0004 % This function adjusts the coefficients of the whole-body biomass
0005 % maintenance (WBM) reaction. The WBM reaction contains each organ present
0006 % in the whole-body metabolic reconstructions. For each organ, the
0007 % stoichiometric coefficients represent the fractional weight contribution
0008 % of the respective organ to the whole body weight. These coefficients can
0009 % be updated to reflect individual specific body contributions. E.g., in
0010 % obese individuals the ratio of muscle and adipose tissue is different
0011 % than in a normal BMI individual. Hence, they can be updated with this
0012 % function.
0013 %
0014 % INPUT
0015 % model         whole-body metabolic model
0016 % listOrgan     List of organs, whose stoichiometric coefficient should be
0017 %               updated
0018 % listCoeff     List of coefficients that replace current ones in the WBM
0019 %               reaction (order must match the order of organs in
0020 %               ListOrgan)
0021 %
0022 % OUTPUT
0023 % model         whole-body metabolic model with adjusted stoichiometric
0024 %               coefficients in the whole-body metabolic model
0025 %
0026 % Ines Thiele, 2012 - 2020
0027 
0028 wholeBodyRxn = 'Whole_body_objective_rxn';
0029 wholeBodyRxnID = find(ismember( model.rxns, wholeBodyRxn));
0030 if ~isfield(model,'A')
0031     model.A = model.S;
0032     removeA = 1;
0033 else 
0034     removeA = 0;
0035 end
0036 for i = 1 :length(listOrgan)
0037     % find dummy reaction for organ
0038     organID = strmatch(strcat(listOrgan{i},'_biomass'),model.mets); 
0039     organID2= (find(~cellfun(@isempty,strfind(model.mets,'_dummy_objective'))));
0040     organID = intersect(organID,organID2);
0041     if ~isempty(organID)
0042         % set stoichiometric coefficient to new value
0043         model.A(organID, wholeBodyRxnID) = -listCoeff(i)*100; % as the organ fractions are given in fraction but should be incorporated as percentage in whole body objective
0044     end
0045 end
0046 model.S = model.A;
0047 if removeA == 1
0048     model = rmfield(model,'A');
0049 end
```

---

Generated on Thu 14-May-2020 13:05:49 by **m2html** © 2005
