# Supplementary material for: Personalized whole‐body models integrate metabolism, physiology, and the gut microbiome
Source: Mol Syst Biol. 2020 May 28;16(5):e8982. doi: 10.15252/msb.20198982 (PMC7285886; doi:10.15252/msb.20198982)
Supplement: Supplementary file 22 — Dataset EV1 [file MSB-16-e8982-s022.zip › PSCM_toolbox/PSCM_toolbox_doc/src/optimizeWBModel.html]

Description of optimizeWBModel


# optimizeWBModel

## PURPOSE

**Solves flux balance analysis problems, and variants thereof**

## SYNOPSIS

**function solution = optimizeWBModel(model, param)**

## DESCRIPTION

```
 Solves flux balance analysis problems, and variants thereof

 Solves LP problems of the form

 .. math::

    max/min  ~& c^T v \\
    s.t.     ~& S v = dxdt ~~~~~~~~~~~:y \\
             ~& C v \leq d~~~~~~~~:y \\
             ~& lb \leq v \leq ub~~~~:w

 USAGE:

    solution = optimizeCbModel(model, osenseStr, minNorm, allowLoops, zeroNormApprox)

 INPUT:
    model:             (the following fields are required - others can be supplied)

                         * S  - `m x 1` Stoichiometric matrix
                         * c  - `n x 1` Linear objective coefficients
                         * lb - `n x 1` Lower bounds
                         * ub - `n x 1` Upper bounds
                         * dxdt - `m x 1` change in concentration with time
                         * csense - `m x 1` character array with entries in {L,E,G} 
                           (The code is backward compatible with an m + k x 1 csense vector,
                           where k is the number of coupling constraints)

                         * C - `k x n` Left hand side of C*v <= d
                         * d - `k x n` Right hand side of C*v <= d
                         * dsense - `k x 1` character array with entries in {L,E,G}

 OPTIONAL INPUT:
    param:      Additional parameters as a parameter struct
                   All fields of the struct which are not COBRA parameters
                   (see `getCobraSolverParamsOptionsForType`) for this
                   problem type will be passed on to the solver in a
                   solver specific manner.

                   Some optional parameters which can be passed to the function
                   as part of the options struct (DONE), or as parameter value
                   pairs (TODO), or are listed below:

    * osenseStr:         Maximize ('max')/minimize ('min') (opt, default =
                         'max') linear part of the objective. Nonlinear
                         parts of the objective are always assumed to be
                         minimised.

    * solverName:    Solver name {'tomlab_cplex','ibm_cplex','cplex_direct'}

    * printLevel:    verbose level
                      *   if `0`, warnings and errors are silenced. (default: 0)
                      *   if `> 0`, warnings and errors are thrown.

    * minNorm:       {(0), scalar , `n x 1` vector}, where `[m, n] = size(S)`;
                   If not zero then, minimise the Euclidean length
                   of the solution to the LP problem. minNorm ~1e-6 should be
                   high enough for regularisation yet maintain the same value for
                   the linear part of the objective. However, this should be
                   checked on a case by case basis, by optimization with and
                   without regularisation.

 OUTPUT:
    solution:       solution object:

                          * f - Objective value
                          * v - Reaction rates (Optimal primal variable, legacy FBAsolution.x)
                          * y - Dual for the molecular species
                          * w - Reduced costs of the reactions
                          * s - Slacks of the molecular species
                          * stat - Solver status in standardized form:
                            * `-1` - No solution reported (timelimit, numerical problem etc)
                            *  `0` - Infeasible
                            *  `1` - Optimal solution
                            *  `2` - Unbounded solution
                          * origStat - Original status returned by the specific solver
                          * ctrs_y - the duals for the constraints from C
                          * ctrs_slack - Slacks of the additional constraints
```

## CROSS-REFERENCE INFORMATION

This function calls:


This function is called by:

- checkIEM\_WBM This function performs the inborn error of metabolism simulations by
- organEssentiality This function computes the organ essentiality in a whole-body model by
- perform\_BMR\_newData This script repeats the simulation described in Thiele et al., "Personalized whole-body models integrate metabolism, physiology, and the gut microbiome", Method section 3.9.2 Validation of the parameters in an independent data set.
- perform\_sensi\_BMR\_all This script repeats the simulation described in Thiele et al.,

## SOURCE CODE

```
0001 function solution = optimizeWBModel(model, param)
0002 % Solves flux balance analysis problems, and variants thereof
0003 %
0004 % Solves LP problems of the form
0005 %
0006 % .. math::
0007 %
0008 %    max/min  ~& c^T v \\
0009 %    s.t.     ~& S v = dxdt ~~~~~~~~~~~:y \\
0010 %             ~& C v \leq d~~~~~~~~:y \\
0011 %             ~& lb \leq v \leq ub~~~~:w
0012 %
0013 % USAGE:
0014 %
0015 %    solution = optimizeCbModel(model, osenseStr, minNorm, allowLoops, zeroNormApprox)
0016 %
0017 % INPUT:
0018 %    model:             (the following fields are required - others can be supplied)
0019 %
0020 %                         * S  - `m x 1` Stoichiometric matrix
0021 %                         * c  - `n x 1` Linear objective coefficients
0022 %                         * lb - `n x 1` Lower bounds
0023 %                         * ub - `n x 1` Upper bounds
0024 %                         * dxdt - `m x 1` change in concentration with time
0025 %                         * csense - `m x 1` character array with entries in {L,E,G}
0026 %                           (The code is backward compatible with an m + k x 1 csense vector,
0027 %                           where k is the number of coupling constraints)
0028 %
0029 %                         * C - `k x n` Left hand side of C*v <= d
0030 %                         * d - `k x n` Right hand side of C*v <= d
0031 %                         * dsense - `k x 1` character array with entries in {L,E,G}
0032 %
0033 % OPTIONAL INPUT:
0034 %    param:      Additional parameters as a parameter struct
0035 %                   All fields of the struct which are not COBRA parameters
0036 %                   (see `getCobraSolverParamsOptionsForType`) for this
0037 %                   problem type will be passed on to the solver in a
0038 %                   solver specific manner.
0039 %
0040 %                   Some optional parameters which can be passed to the function
0041 %                   as part of the options struct (DONE), or as parameter value
0042 %                   pairs (TODO), or are listed below:
0043 %
0044 %    * osenseStr:         Maximize ('max')/minimize ('min') (opt, default =
0045 %                         'max') linear part of the objective. Nonlinear
0046 %                         parts of the objective are always assumed to be
0047 %                         minimised.
0048 %
0049 %    * solverName:    Solver name {'tomlab_cplex','ibm_cplex','cplex_direct'}
0050 %
0051 %    * printLevel:    verbose level
0052 %                      *   if `0`, warnings and errors are silenced. (default: 0)
0053 %                      *   if `> 0`, warnings and errors are thrown.
0054 %
0055 %    * minNorm:       {(0), scalar , `n x 1` vector}, where `[m, n] = size(S)`;
0056 %                   If not zero then, minimise the Euclidean length
0057 %                   of the solution to the LP problem. minNorm ~1e-6 should be
0058 %                   high enough for regularisation yet maintain the same value for
0059 %                   the linear part of the objective. However, this should be
0060 %                   checked on a case by case basis, by optimization with and
0061 %                   without regularisation.
0062 %
0063 % OUTPUT:
0064 %    solution:       solution object:
0065 %
0066 %                          * f - Objective value
0067 %                          * v - Reaction rates (Optimal primal variable, legacy FBAsolution.x)
0068 %                          * y - Dual for the molecular species
0069 %                          * w - Reduced costs of the reactions
0070 %                          * s - Slacks of the molecular species
0071 %                          * stat - Solver status in standardized form:
0072 %                            * `-1` - No solution reported (timelimit, numerical problem etc)
0073 %                            *  `0` - Infeasible
0074 %                            *  `1` - Optimal solution
0075 %                            *  `2` - Unbounded solution
0076 %                          * origStat - Original status returned by the specific solver
0077 %                          * ctrs_y - the duals for the constraints from C
0078 %                          * ctrs_slack - Slacks of the additional constraints
0079 
0080 
0081 if isfield(model,'osenseStr')
0082     if ~any(strcmp(model.osenseStr,{'min','max'}))
0083         error('model.osenseStr can only be either min or max')
0084     end
0085 else
0086     %this is in for backward compatibility only, use model.osenseStr
0087     %instead
0088     if isfield(model,'osense')
0089         if model.osense == 1
0090             model.osenseStr = 'min';
0091         elseif model.osense == -1
0092             model.osenseStr = 'max';
0093         else
0094             error('model.osense can only be either 1 or -1')
0095         end
0096     else
0097         %check in case there is no linear objective
0098         linearObjective = any(model.c);
0099         quadraticObjective = ~isempty(param.minNorm);
0100         if linearObjective && quadraticObjective
0101             model.osenseStr = 'min';
0102         elseif linearObjective && ~quadraticObjective
0103             model.osenseStr = 'max';
0104         elseif ~linearObjective && quadraticObjective
0105             model.osenseStr = 'min';
0106         elseif ~linearObjective && ~quadraticObjective
0107             model.osenseStr = 'min';
0108         end
0109         warning(['optimizeWBModel: assuming model.osenseStr is ' model.osenseStr ', but this should be specified explicitly.'])
0110     end
0111 end
0112 
0113 if exist('param','var')
0114     if isfield(param,'minNorm')
0115         if param.minNorm == 0
0116             param.minNorm = [];
0117         end
0118     else
0119         param.minNorm=[];
0120     end
0121     if ~isfield(param,'verify')
0122         param.verify=0;
0123     end
0124 else
0125     param.minNorm=[];
0126     param.verify = 0;
0127 end
0128 
0129 validatedSolvers={'tomlab_cplex','ibm_cplex','cplex_direct'};
0130 
0131 if 1
0132     %mlb = magnitude of a large bound
0133     mlb = 1000000; %original
0134 else
0135     mlb = inf;
0136 end
0137 
0138 allowLoops =1;
0139 zeroNormApprox = [];
0140 
0141 if isempty(param.minNorm) %Linear optimisation
0142     
0143     [solverName, solverOK] = getCobraSolver('LP');
0144     if ~any(strcmp(solverName,validatedSolvers))
0145         fprintf('%s\n','Note that the solvers validated for use with the PSCM toolbox are:')
0146         disp(validatedSolvers)
0147         [solverOK, solverInstalled] = changeCobraSolver('tomlab_cplex', 'LP',1,1);
0148         if ~solverOK
0149             error([solverName ' has not been validated for use with the PSCM toolbox. Tried to change to tomlab_cplex, but it failed.'])
0150         end
0151     end
0152     
0153     solution = optimizeCbModel(model, model.osenseStr, param.minNorm, allowLoops, zeroNormApprox, param);
0154     
0155 elseif isnumeric(param.minNorm) %quadratic optimisation, proceeds in two steps
0156     
0157     %check in case there is no linear objective
0158     noLinearObjective = all(model.c==0);
0159 
0160     if noLinearObjective
0161         [tmp, solverOK] = getCobraSolver('QP');
0162         solverName{1,1} = tmp;
0163         solverName{1,2} = 'QP';
0164     else
0165         [solverName{1,1}, solverOK] = getCobraSolver('LP');
0166         solverName{1,2} = 'QP';
0167         [solverName{2,1}, solverOK] = getCobraSolver('QP');
0168         solverName{2,2} = 'QP';
0169     end
0170     
0171     for i = 1:size(solverName,1)
0172         if ~any(strcmp(solverName{i,1},validatedSolvers))
0173             fprintf('%s\n','Note that the solvers validated for use with the PSCM toolbox are:')
0174             disp(validatedSolvers)
0175             %switch over to a validated solver
0176             [solverOK, solverInstalled] = changeCobraSolver('tomlab_cplex', solverName{i,2},1,1);
0177             if solverOK
0178                 fprintf('%s\n',[solverName{i,1} ' has not been validated for use with the PSCM toolbox. Tried to change to tomlab_cplex, but it failed.'])
0179             else
0180                 error([solverName{i,1} ' has not been validated for use with the PSCM toolbox. Tried to change to tomlab_cplex, but it failed.'])
0181             end
0182         end
0183     end
0184     
0185     param.printLevel = getCobraSolverParams('QP','printLevel',param);
0186     
0187     solution = optimizeCbModel(model, model.osenseStr, param.minNorm, allowLoops, zeroNormApprox, param);
0188     
0189     if param.printLevel>0
0190         fprintf('%s%i\n','First solution.stat = ', solution.stat)
0191         fprintf('%s%i\n','First solution.origStat = ', solution.origStat)
0192         if param.printLevel>1 && any(contains(solverName(:,1),'cplex'))
0193             [ExitText,~] = cplexStatus(solution.origStat);
0194             fprintf('%s%s\n','First solution.origStatText = ', ExitText)
0195         end
0196     end
0197     
0198 %       1 (S,B) Optimal solution found
0199 %       2 (S,B) Model has an unbounded ray
0200 %       3 (S,B) Model has been proven infeasible
0201 %       4 (S,B) Model has been proven either infeasible or unbounded
0202 %       5 (S,B) Optimal solution is available, but with infeasibilities after unscaling
0203 %       6 (S,B) Solution is available, but not proven optimal, due to numeric difficulties
0204 
0205     % origStat == 5 means Optimal solution is available, but with infeasibilities after unscaling
0206     % origStat == 6 means Solution is available, but not proved optimal, due to numeric difficulties during optimization
0207     %if solution.stat~=0 && (solution.origStat == 5 || solution.origStat == 6)
0208     if solution.stat == 3
0209         %rescale the problem and try to solve it again
0210         if 1
0211             if ~isempty(solution.v)
0212                 %rescale with help from previous solution
0213                 bigN=max(abs(solution.v));
0214             else
0215                 %rescale without any previous solution
0216                 bigN = 500000;
0217             end
0218             % all high flux values in the solution vector of the first QP retain a high bound
0219             model.lb(solution.v<-1e4) = -bigN;
0220             model.ub(solution.v>1e4)  =  bigN;
0221         end
0222         
0223         if 1
0224             % reduce the "infinity" bounds on all other reactions.
0225             % note this step does not affect any non-infinity bounds set on the
0226             % whole-body metabolic model
0227             model.lb(model.lb==-mlb)= -10000; % reduce the effective unbound constraints to lower number, representing inf
0228             model.ub(model.ub==mlb)=   10000;% reduce the effective unbound constraints to lower number, representing inf
0229         end
0230         
0231         if 1
0232             % we then rescale all bounds on the model reactions by a factor of 1/1000,
0233             % which proven to result in an optimal QP solution
0234             model.lb=model.lb/1000;
0235             model.ub=model.ub/1000;
0236         end
0237         
0238         solution = optimizeCbModel(model, model.osenseStr, param.minNorm, allowLoops, zeroNormApprox, param);
0239         
0240         
0241         % rescale the computed solution by the factor of 1000
0242         %  * f - Objective value
0243         %  * v - Reaction rates (Optimal primal variable, legacy FBAsolution.x)
0244         %  * y - Dual for the molecular species
0245         %  * w - Reduced costs of the reactions
0246         %  * s - Slacks of the molecular species
0247         %  * ctrs_y - the duals for the constraints from C
0248         %  * ctrs_slack - Slacks of the additional constraints
0249         
0250         % rescale the computed solution by the factor of 1000
0251         solution.f = solution.f*1000; 
0252         solution.v = solution.v*1000;
0253         solution.y = solution.y*1000;
0254         solution.w = solution.w*1000;
0255         solution.s = solution.s*1000;
0256         if isfield(solution,'ctrs_y')
0257             solution.ctrs_y = solution.ctrs_y*1000;
0258         end
0259         if isfield(solution,'ctrs_slack')
0260             solution.ctrs_slack = solution.ctrs_slack*1000;
0261         end
0262         
0263         if param.printLevel>0
0264             fprintf('%s%i\n','Second solution.stat = ', solution.stat)
0265             fprintf('%s%i\n','Second solution.origStat = ', solution.origStat)
0266             if param.printLevel>1 && any(contains(solverName(:,1),'cplex'))
0267                 [ExitText,~] = cplexStatus(solution.origStat);
0268                 fprintf('%s%s\n','Second solution.origStatText = ', ExitText)
0269             end
0270         end
0271     else
0272         if 0
0273             %return NaN of correct dimensions if problem does not solve properly
0274             solution.f = NaN;
0275             solution.v = NaN*ones(size(model.S,2),1);
0276             solution.y = NaN*ones(size(model.S,1),1);
0277             solution.w = NaN*ones(size(model.S,2),1);
0278             solution.s = NaN*ones(size(model.S,1),1);
0279             if isfield(model,'C')
0280                 solution.ctrs_y = NaN*ones(size(model.C,1),1);
0281                 solution.ctrs_slack = NaN*ones(size(model.C,1),1);
0282             end
0283             if isfield(model,'E')
0284                 solution.vars_v = NaN*ones(size(model.E,2),1);
0285                 solution.vars_w = NaN*ones(size(model.E,2),1);
0286             end
0287         else
0288             %return empty fields if problem does not solve properly (backward
0289             %compatible)
0290             solution.f = NaN;
0291             solution.v = [];
0292             solution.y = [];
0293             solution.w = [];
0294             solution.s = [];
0295             if isfield(model,'C')
0296                 solution.ctrs_y = [];
0297             end
0298             if isfield(model,'E')
0299                 solution.vars_v = [];
0300                 solution.vars_w = [];
0301             end
0302         end
0303         solution.x = solution.v;
0304         if param.printLevel>1 && any(contains(solverName(:,1),'cplex'))
0305             [ExitText,~] = cplexStatus(solution.origStat);
0306             warning(['Second solution.origStatText = ', ExitText])
0307         end
0308     end
0309 end
0310 
0311 if 1 %this may not be very backward compatible
0312     %remove fields coming from solveCobraLP/QP but not part of the specification
0313     %of the output from optimizeCbModel
0314     if isfield(solution,'obj')
0315         solution = rmfield(solution,'obj');
0316     end
0317     if isfield(solution,'full')
0318         solution = rmfield(solution,'full');
0319     end
0320     if isfield(solution,'rcost')
0321         solution = rmfield(solution,'rcost');
0322     end
0323     if isfield(solution,'slack')
0324         solution = rmfield(solution,'slack');
0325     end
0326 end
0327
```

---

Generated on Thu 14-May-2020 13:05:49 by **m2html** © 2005
