# Supplementary material for: Personalized whole‐body models integrate metabolism, physiology, and the gut microbiome
Source: Mol Syst Biol. 2020 May 28;16(5):e8982. doi: 10.15252/msb.20198982 (PMC7285886; doi:10.15252/msb.20198982)
Supplement: Supplementary file 22 — Dataset EV1 [file MSB-16-e8982-s022.zip › PSCM_toolbox/PSCM_toolbox_doc/src/getRxnsFromGene.html]

Description of getRxnsFromGene


# getRxnsFromGene

## PURPOSE

**This function gets all reaction(s) associated with a particular gene by**

## SYNOPSIS

**function [Rxns, grRules] = getRxnsFromGene(model,gene,causal)**

## DESCRIPTION

```
 This function gets all reaction(s) associated with a particular gene by
 screening through the grRules provided in the model structure
 

 [Rxns, grRules] = getRxnsFromGene(model,gene,causal)
 
 INPUT
 model     model structure
 gene      gene of interest
 causal    if causal == 1 get only genes that would lead to loss of function of the
           associated reactions, otw get all associated reactions
           (default)
 
 OUTPUT
 Rxns      List of reaction(s) associated with the input gene
 grRules   List of grRules containing the input gene, same order as Rxns
 
 Ines Thiele 10/2019
```

## CROSS-REFERENCE INFORMATION

This function calls:


This function is called by:

## SOURCE CODE

```
0001 function [Rxns, grRules] = getRxnsFromGene(model,gene,causal)
0002 % This function gets all reaction(s) associated with a particular gene by
0003 % screening through the grRules provided in the model structure
0004 %
0005 %
0006 % [Rxns, grRules] = getRxnsFromGene(model,gene,causal)
0007 %
0008 % INPUT
0009 % model     model structure
0010 % gene      gene of interest
0011 % causal    if causal == 1 get only genes that would lead to loss of function of the
0012 %           associated reactions, otw get all associated reactions
0013 %           (default)
0014 %
0015 % OUTPUT
0016 % Rxns      List of reaction(s) associated with the input gene
0017 % grRules   List of grRules containing the input gene, same order as Rxns
0018 %
0019 % Ines Thiele 10/2019
0020 
0021 if  ~exist('causal','var')
0022     causal = 0;
0023 end
0024 
0025 assoR = [];
0026 for i = 1 : length(model.grRules)
0027     if ~isempty(strfind(model.grRules{i},gene))
0028         if causal == 1
0029             % case 1 - 1 gene
0030             if  ~isempty(strmatch(model.grRules{i},gene,'exact')) % perfect match
0031                 assoR(i,1)=1;
0032                 % case 2 - 1 complex
0033             elseif ~isempty(strfind(model.grRules{i},{' and '})) &&  isempty(strfind(model.grRules{i},{' or '}))
0034                 [c,d] = split(model.grRules{i},' and ');
0035                 if ~isempty(strmatch(gene,c)) % works only for single genes or when at the beginning of and statement
0036                     assoR(i,1)=1;
0037                 end
0038             elseif ~isempty(strfind(model.grRules{i},{' or '})) % consider cases of alt splices and ' or '
0039                 cnt = 0;
0040                 [geneTok] = strtok(gene,'.');
0041                 if isempty(strfind(model.grRules{i},{' and '})) % only 'or's
0042                     [c,d] = split(model.grRules{i},' or ');
0043                     for j = 1 : length(c)
0044                         cTok = strtok(c{j},'.');
0045                         if ~isempty(strmatch(geneTok,cTok,'exact')) % perfect match
0046                             cnt = cnt +1;
0047                         end
0048                     end
0049                     if cnt == length(c) % all genes in or are alt splice forms
0050                         assoR(i,1)=1;
0051                     end
0052                 else % contains 'and'
0053                     [c,d] = split(model.grRules{i},' or '); % split first the 'or's
0054                     for j = 1 : length(c)
0055                         if ~isempty(strfind(c{j},{' and '})) % if 'and'
0056                             [a,b] = split(c{j},' and '); % split the 'and's
0057                             for k = 1 : length(a)
0058                                 aTok = strtok(a{k},'.');
0059                                 if ~isempty(strmatch(geneTok,aTok,'exact')) % perfect match
0060                                     cnt = cnt +1;
0061                                 end
0062                             end
0063                         else % no 'and'
0064                             cTok = strtok(c{j},'.');
0065                             if ~isempty(strcmp(geneTok,cTok)) % perfect match
0066                                 cnt = cnt +1;
0067                             end
0068                         end
0069                     end
0070                     % if there are as many counts as c's
0071                     if cnt == length(c) % all genes in or are alt splice forms
0072                         assoR(i,1)=1;
0073                     end
0074                 end
0075             end
0076         else
0077             assoR(i,1) = 1;
0078         end
0079         
0080     end
0081 end
0082 Rxns=  model.rxns(find(assoR))
0083 grRules= model.grRules(find(assoR))
```

---

Generated on Thu 14-May-2020 13:05:49 by **m2html** © 2005
