# Supplementary material for: Personalized whole‐body models integrate metabolism, physiology, and the gut microbiome
Source: Mol Syst Biol. 2020 May 28;16(5):e8982. doi: 10.15252/msb.20198982 (PMC7285886; doi:10.15252/msb.20198982)
Supplement: Supplementary file 22 — Dataset EV1 [file MSB-16-e8982-s022.zip › PSCM_toolbox/PSCM_toolbox_doc/src/calculateBMR.html]

Description of calculateBMR


# calculateBMR

## PURPOSE

**This function calculates the basal metabolic rate using the**

## SYNOPSIS

**function BMR = calculateBMR(sex, weight, height, age)**

## DESCRIPTION

```
 This function calculates the basal metabolic rate using the
 phenomenological model proposed by Harris-Benedict and derivations
 thereof (see below.
 Please also refer to the corresponding wikipedia entry:
 https://en.wikipedia.org/wiki/Harris%E2%80%93Benedict_equation for more
 details
 
 function BMR = calculateBMR(sex, weight, height, age)
 
 INPUT
 sex    'male' or 'female'
 weight    in kg
 height    in cm
 age       in years

 OUTPUT
 BMR       array with 3 numbers calculated based on 
                1. original Harris Benedict equations [1],[2]
                2. Harris Benedict equations revised by Roza and Shizgal in 1984.[3]
                3. The Harris Benedict equations revised by Mifflin and St Jeor in 1990:[4]

 References:
 [1]    Harris JA, Benedict FG (1918). "A Biometric Study of Human Basal Metabolism". Proceedings of the National Academy of Sciences of the United States of America. 4 (12): 370?3. doi:10.1073/pnas.4.12.370. PMC 1091498?Freely accessible. PMID 16576330.
 [2]    A Biometric Study of Basal Metabolism in Man. J. Arthur Harris and Francis G. Benedict. Washington, DC: Carnegie Institution, 1919.
 [3]    Roza AM, Shizgal HM (1984). "The Harris Benedict equation reevaluated: resting energy requirements and the body cell mass". The American Journal of Clinical Nutrition. 40 (1): 168?82. PMID 6741850.
 [4]    Mifflin MD, St Jeor ST, Hill LA, Scott BJ, Daugherty SA, Koh YO (1990). "A new predictive equation for resting energy expenditure in healthy individuals". The American Journal of Clinical Nutrition. 51 (2): 241?7. PMID 2305711.

 Ines Thiele 01/2018
```

## CROSS-REFERENCE INFORMATION

This function calls:


This function is called by:

- perform\_BMR\_newData This script repeats the simulation described in Thiele et al., "Personalized whole-body models integrate metabolism, physiology, and the gut microbiome", Method section 3.9.2 Validation of the parameters in an independent data set.
- perform\_sensi\_BMR\_all This script repeats the simulation described in Thiele et al.,

## SOURCE CODE

```
0001 function BMR = calculateBMR(sex, weight, height, age)
0002 % This function calculates the basal metabolic rate using the
0003 % phenomenological model proposed by Harris-Benedict and derivations
0004 % thereof (see below.
0005 % Please also refer to the corresponding wikipedia entry:
0006 % https://en.wikipedia.org/wiki/Harris%E2%80%93Benedict_equation for more
0007 % details
0008 %
0009 % function BMR = calculateBMR(sex, weight, height, age)
0010 %
0011 % INPUT
0012 % sex    'male' or 'female'
0013 % weight    in kg
0014 % height    in cm
0015 % age       in years
0016 %
0017 % OUTPUT
0018 % BMR       array with 3 numbers calculated based on
0019 %                1. original Harris Benedict equations [1],[2]
0020 %                2. Harris Benedict equations revised by Roza and Shizgal in 1984.[3]
0021 %                3. The Harris Benedict equations revised by Mifflin and St Jeor in 1990:[4]
0022 %
0023 % References:
0024 % [1]    Harris JA, Benedict FG (1918). "A Biometric Study of Human Basal Metabolism". Proceedings of the National Academy of Sciences of the United States of America. 4 (12): 370?3. doi:10.1073/pnas.4.12.370. PMC 1091498?Freely accessible. PMID 16576330.
0025 % [2]    A Biometric Study of Basal Metabolism in Man. J. Arthur Harris and Francis G. Benedict. Washington, DC: Carnegie Institution, 1919.
0026 % [3]    Roza AM, Shizgal HM (1984). "The Harris Benedict equation reevaluated: resting energy requirements and the body cell mass". The American Journal of Clinical Nutrition. 40 (1): 168?82. PMID 6741850.
0027 % [4]    Mifflin MD, St Jeor ST, Hill LA, Scott BJ, Daugherty SA, Koh YO (1990). "A new predictive equation for resting energy expenditure in healthy individuals". The American Journal of Clinical Nutrition. 51 (2): 241?7. PMID 2305711.
0028 %
0029 % Ines Thiele 01/2018
0030 
0031 %% The original Harris-Benedict equations published in 1918 and 1919.
0032 if strcmp(sex,'male') || strcmp(sex,'Male')
0033     % BMR = 66.5 + ( 13.75 �weight in kg ) + ( 5.003 �height in cm ) ? ( 6.755 �age in years )
0034     BMR(1,1) = 66.5 + ( 13.75 *weight ) + ( 5.003 * height) - ( 6.755 * age);
0035 else % female
0036     %   BMR = 655.1 + ( 9.563 �weight in kg ) + ( 1.850 �height in cm ) ? ( 4.676 �age in years )
0037     BMR(1,1) = 655.1 + ( 9.563 * weight  ) + ( 1.850 * height  ) - ( 4.676 * age );
0038 end
0039 
0040 %% The Harris?Benedict equations revised by Roza and Shizgal in 1984.[3]
0041 %
0042 if strcmp(sex,'male')|| strcmp(sex,'Male')
0043     % Men    BMR = 88.362 + (13.397 �weight in kg) + (4.799 �height in cm) - (5.677 �age in years)
0044     BMR(2,1) = 88.362 + (13.397 * weight ) + (4.799 * height ) - (5.677 * age);
0045 else % female
0046     % Women    BMR = 447.593 + (9.247 �weight in kg) + (3.098 �height in cm) - (4.330 �age in years)
0047     BMR(2,1) = 447.593 + (9.247 * weight ) + (3.098 * height ) - (4.330 * age );
0048 end
0049 % The 95% confidence range for men is �213.0 kcal/day, and �201.0 kcal/day for women.
0050 %
0051 %% The Harris?Benedict equations revised by Mifflin and St Jeor in 1990:[4]
0052 %
0053 if strcmp(sex,'male')|| strcmp(sex,'Male')
0054     % Men    BMR = (10 �weight in kg) + (6.25 �height in cm) - (5 �age in years) + 5
0055     BMR(3,1) = (10 * weight ) + (6.25 * height ) - (5 * age ) + 5;
0056 else % female
0057     % Women    BMR = (10 �weight in kg) + (6.25 �height in cm) - (5 �age in years) - 161
0058     BMR(3,1) = (10 * weight ) + (6.25 * height ) - (5 * age ) -161;
0059 end
0060
```

---

Generated on Thu 14-May-2020 13:05:49 by **m2html** © 2005
