# Supplementary material for: Personalized whole‐body models integrate metabolism, physiology, and the gut microbiome
Source: Mol Syst Biol. 2020 May 28;16(5):e8982. doi: 10.15252/msb.20198982 (PMC7285886; doi:10.15252/msb.20198982)
Supplement: Supplementary file 22 — Dataset EV1 [file MSB-16-e8982-s022.zip › PSCM_toolbox/PSCM_toolbox_doc/src/organEssentiality.html]

Description of organEssentiality


# organEssentiality

## PURPOSE

**This function computes the organ essentiality in a whole-body model by**

## SYNOPSIS

**function [ResultsOrganEss] = organEssentiality(model, LPSolver)**

## DESCRIPTION

```
 This function computes the organ essentiality in a whole-body model by
 setting each organ's value to zero in the whole-body objective reaction,
 setting all organ-specific reaction bounds to zero (lower and upper),
 and then computes whether a non-zero flux through this objective is still
 possible.

 function [ResultsOrganEss] = organEssentiality(model, LPSolver)

 INPUT
 model             model structure (whole-body metabolic model)
 LPSolver          Define LPSolver ('ILOGcomplex';
                   'tomlab_cplex' -default)

 OUTPUT
 ResultsOrganEss   Contains the maximally possible flux value for the
                   whole-body reaction for each organ. Col 1: Organ name,
                   Col 2: Max flux value, Col 3: Min flux value (if
                   minimization is activated; default: inactive), Col 4:
                   Solver status: 1 = feasible, 5 = feasible with
                   numerical difficulties (rescaling issues), 3 =
                   infeasible)

 Ines Thiele 2016
 Ines Thiele, added option to specify LPSolver - 10/2018
```

## CROSS-REFERENCE INFORMATION

This function calls:

- OrganLists This file contains lists of ograns as they are used in the whole-body
- optimizeWBModel Solves flux balance analysis problems, and variants thereof
- getOrganWeightFraction This script reads in the organ file and assign biomass\_maintenance coefficient according

This function is called by:

## SOURCE CODE

```
0001 function [ResultsOrganEss] = organEssentiality(model, LPSolver)
0002 % This function computes the organ essentiality in a whole-body model by
0003 % setting each organ's value to zero in the whole-body objective reaction,
0004 % setting all organ-specific reaction bounds to zero (lower and upper),
0005 % and then computes whether a non-zero flux through this objective is still
0006 % possible.
0007 %
0008 % function [ResultsOrganEss] = organEssentiality(model, LPSolver)
0009 %
0010 % INPUT
0011 % model             model structure (whole-body metabolic model)
0012 % LPSolver          Define LPSolver ('ILOGcomplex';
0013 %                   'tomlab_cplex' -default)
0014 %
0015 % OUTPUT
0016 % ResultsOrganEss   Contains the maximally possible flux value for the
0017 %                   whole-body reaction for each organ. Col 1: Organ name,
0018 %                   Col 2: Max flux value, Col 3: Min flux value (if
0019 %                   minimization is activated; default: inactive), Col 4:
0020 %                   Solver status: 1 = feasible, 5 = feasible with
0021 %                   numerical difficulties (rescaling issues), 3 =
0022 %                   infeasible)
0023 %
0024 % Ines Thiele 2016
0025 % Ines Thiele, added option to specify LPSolver - 10/2018
0026 
0027 if ~exist('LPSolver','var')
0028     LPSolver = 'tomlab_cplex';
0029 end
0030 
0031 global useSolveCobraLPCPLEX
0032 useSolveCobraLPCPLEX
0033 
0034 [solverOK, solverInstalled] = changeCobraSolver(LPSolver, 'LP');
0035 
0036 % reset the bounds on the whole-body objective
0037 model = changeRxnBounds(model,'Whole_body_objective_rxn',0,'l');
0038 model = changeRxnBounds(model,'Whole_body_objective_rxn',1000,'u');
0039 
0040 % define whole-body objective as objective function
0041 model = changeObjective(model,'Whole_body_objective_rxn');
0042 
0043 sex = model.sex;
0044 
0045 % List of organs
0046 getOrganWeightFraction;
0047 OrganLists;
0048 for i  = 1 : length(OrgansListShort)
0049     modelOrganEss = model;
0050     % redefine objective by removing the organ from the whole-body reaction
0051     O = strmatch(OrgansListShort{i},ObjectiveComponents);
0052     R = find(ismember(modelOrganEss.rxns,'Whole_body_objective_rxn'));
0053     M = find(ismember(modelOrganEss.mets,strcat(ObjectiveComponents{O},'_dummy_objective')));
0054     if useSolveCobraLPCPLEX 
0055         if ~isfield(modelOrganEss,'A')
0056             error('model.A missing')
0057         end
0058         modelOrganEss.A(M,R)=0; % no requirement of this objective part in OF
0059     else
0060         modelOrganEss.S(M,R)=0; % no requirement of this objective part in OF
0061     end
0062     modelOrganEss = changeObjective(modelOrganEss,'Whole_body_objective_rxn');
0063     % set all reaction bounds in this organ to 0
0064     R1 = strmatch(OrgansListShort{i},modelOrganEss.rxns);
0065     modelOrganEss.lb(R1)=0;
0066     modelOrganEss.ub(R1)=0;
0067     % maximize the whole-body reaction
0068     modelOrganEss.osenseStr = 'max';
0069     tic;
0070     if useSolveCobraLPCPLEX
0071         [FBA,~]=solveCobraLPCPLEX(modelOrganEss,1,0,0,[],0,LPSolver);
0072         FBA.f=FBA.obj;
0073         FBA.v=FBA.full;
0074     else
0075         FBA = optimizeWBModel(modelOrganEss);
0076     end
0077     timeTaken = toc;
0078     fprintf('%u%s%s%s%f\n',timeTaken,' sec. ',OrgansListShort{i},' obj = ',FBA.f)
0079     ResultsOrganEss(i,1)=OrgansListShort(i);
0080     
0081     if useSolveCobraLPCPLEX
0082         feasible = FBA.origStat == 1 || FBA.origStat == 5;
0083     else
0084         feasible = FBA.stat == 1;
0085     end
0086     if feasible
0087         ResultsOrganEss{i,2}=num2str(FBA.v(modelOrganEss.c~=0)); % max
0088         if 0 % also compute the minimal possible flux through the objective
0089             modelOrganEss.osenseStr = 'min';
0090             tic;
0091             if useSolveCobraLPCPLEX
0092                 [FBA,LPProblem]=solveCobraLPCPLEX(modelOrganEss,1,0,0,[],0,LPSolver);
0093             else
0094                 FBA = optimizeWBModel(modelOrganEss);
0095             end
0096             timeTaken = toc;
0097             ResultsOrganEss{i,3}=num2str(FBA.v(modelOrganEss.c)~=0);%min
0098         end
0099         %feasible
0100         ResultsOrganEss{i,4}=num2str(FBA.origStat);
0101     else
0102         if FBA.stat == -1 ||  FBA.stat == 2
0103             %display solution in case there is a problem
0104             fprinf('%s\n',['Problem with FBA for organ: ' ResultsOrganEss{i,1} '. FBA solution is:'])
0105             FBA
0106         end
0107             
0108         %infeasible or otherwise
0109         ResultsOrganEss{i,2}=NaN; % min
0110         ResultsOrganEss{i,3}=NaN;%max
0111         ResultsOrganEss{i,4}=num2str(FBA.origStat);
0112     end
0113 end
```

---

Generated on Thu 14-May-2020 13:05:49 by **m2html** © 2005
