# Supplementary material for: Personalized whole‐body models integrate metabolism, physiology, and the gut microbiome
Source: Mol Syst Biol. 2020 May 28;16(5):e8982. doi: 10.15252/msb.20198982 (PMC7285886; doi:10.15252/msb.20198982)
Supplement: Supplementary file 22 — Dataset EV1 [file MSB-16-e8982-s022.zip › PSCM_toolbox/PSCM_toolbox_doc/src/checkIEM_WBM.html]

Description of checkIEM\_WBM


# checkIEM\_WBM

## PURPOSE

**This function performs the inborn error of metabolism simulations by**

## SYNOPSIS

**function [IEMSol] = checkIEM\_WBM(model,IEMRxns, BiomarkerRxns,minRxnsFluxHealthy, reverseDirObj, fractionKO,minBiomarker,fixIEMlb, LPSolver)**

## DESCRIPTION

```
 This function performs the inborn error of metabolism simulations by
 deleting (or reducing) the flux through reaction(s) affected by a gene
 defect and optimized the flux through a defined set of biomarker
 reactions.

 function [IEMSol] = checkIEM_WBM(model,IEMRxns, BiomarkerRxns,minRxnsFluxHealthy, reverseDirObj, fractionKO,minBiomarker,fixIEMlb)

 INPUT
 model                 whole-body metabolic reconstruction or Recon3Dmodel
 IEMRxns               Reaction(s) affected by the inborn error of
                       metabolism
 minRxnsFluxHealthy    min flux value(s) through the IEMRxns
 reverseDirObj         the function maximizes the objective flux by
                       default. If set to 1, the function also checks the minimization problem.
 fractionKO            By default, a complete knowckout of BiomarkerRxnsthe IEM
                       reactions is computed but it is possible to set a fraction (default = 1
                       for 100% knockout)
 minBiomarker          Minimization through biomarker reaction (default = 0)
 fixIEMlb              fix IEM to lb = ub
                       =(1-fractionKO)*solution.v(find(model.c)) (default = 0, i.e., lb =0,
                       while ub = (1-fractionKO)*solution.v(find(model.c))
 LPSolver              Define LPSolver ('ILOGcomplex' - default;
                       'tomlab_cplex')

 OUTPUT
 IEMSol                Predicted biomarker fluxes and comparison with the
                       reported biomarkers

 USAGE:
 Exampe of preparation of a set of inputs to checkIEM_WBM
     R = {'_2OXOADPTm';'_2AMADPTm';'_r0879'};
     RxnsAll2 = '';
     for i = 1: length(R)
         RxnsAll = model.rxns(find(~cellfun(@isempty,strfind(model.rxns,R{i}))));
         RxnsAll2 =[RxnsAll2;RxnsAll];
     end
     IEMRxns = unique(RxnsAll2);
     RxnMic = model.rxns(find(~cellfun(@isempty,strfind(model.rxns,'Micro_')))) ;
     IEMRxns = setdiff(IEMRxns,RxnMic);

     if ~strcmp(modelName,'Recon3D')
         % add demand reactions to blood compartment for those biomarkers reported for blood
         % biomarker based on https://www.omim.org/entry/204750
         model = addDemandReaction(model, 'L2aadp[bc]');

         BiomarkerRxns = {
             'DM_L2aadp[bc]'    'Increased (blood)'
             'EX_2oxoadp[u]'    'Increased (urine)'
             'EX_adpoh[u]'    'Increased (urine)'
             };
     else
         BiomarkerRxns = {
             'EX_2oxoadp[u]'    'Increased (urine)'
             'EX_adpoh[u]'    'Increased (urine)'
             };
     end

 Then call the checkIEM_WBM function
     [IEMSol_2OAA] = checkIEM_WBM(model,IEMRxns, BiomarkerRxns,minRxnsFluxHealthy);

 Example IEMSol returned from the above
     {'IEM Rxns All obj - Healthy'}    {'65403.5393' }    {0×0 double                    }
     {'IEM Rxns All obj - Disease'}    {'65403.5393' }    {0×0 double                    }
     {'WB obj - Healthy'          }    {'NA'         }    {0×0 double                    }
     {'WB obj - Disease'          }    {'1'          }    {'1'                           }
     {'Healthy:DM_L2aadp[bc]'     }    {'-5.5324e-08'}    {'Disease - Reported:Increas…'}
     {'Disease:DM_L2aadp[bc]'     }    {'40.2418'    }    {'Disease - Reported:Increas…'}
     {'Healthy:EX_2oxoadp[u]'     }    {'-2.3603e-10'}    {'Disease - Reported:Increas…'}
     {'Disease:EX_2oxoadp[u]'     }    {'3.7368'     }    {'Disease - Reported:Increas…'}
     {'Healthy:EX_adpoh[u]'       }    {'-1.6985e-10'}    {'Disease - Reported:Increas…'}
     {'Disease:EX_adpoh[u]'       }    {'3.7368'     }    {'Disease - Reported:Increas…'}

 2018 - Ines Thiele
 2019 - Ines Thiele, included more options (minBiomarker,fixIEMlb,LPSolver)
```

## CROSS-REFERENCE INFORMATION

This function calls:

- optimizeWBModel Solves flux balance analysis problems, and variants thereof

This function is called by:

- runIEM\_HH This script predicts known biomarker metabolites in

## SOURCE CODE

```
0001 function [IEMSol] = checkIEM_WBM(model,IEMRxns, BiomarkerRxns,minRxnsFluxHealthy, reverseDirObj, fractionKO,minBiomarker,fixIEMlb, LPSolver)
0002 % This function performs the inborn error of metabolism simulations by
0003 % deleting (or reducing) the flux through reaction(s) affected by a gene
0004 % defect and optimized the flux through a defined set of biomarker
0005 % reactions.
0006 %
0007 % function [IEMSol] = checkIEM_WBM(model,IEMRxns, BiomarkerRxns,minRxnsFluxHealthy, reverseDirObj, fractionKO,minBiomarker,fixIEMlb)
0008 %
0009 % INPUT
0010 % model                 whole-body metabolic reconstruction or Recon3Dmodel
0011 % IEMRxns               Reaction(s) affected by the inborn error of
0012 %                       metabolism
0013 % minRxnsFluxHealthy    min flux value(s) through the IEMRxns
0014 % reverseDirObj         the function maximizes the objective flux by
0015 %                       default. If set to 1, the function also checks the minimization problem.
0016 % fractionKO            By default, a complete knowckout of BiomarkerRxnsthe IEM
0017 %                       reactions is computed but it is possible to set a fraction (default = 1
0018 %                       for 100% knockout)
0019 % minBiomarker          Minimization through biomarker reaction (default = 0)
0020 % fixIEMlb              fix IEM to lb = ub
0021 %                       =(1-fractionKO)*solution.v(find(model.c)) (default = 0, i.e., lb =0,
0022 %                       while ub = (1-fractionKO)*solution.v(find(model.c))
0023 % LPSolver              Define LPSolver ('ILOGcomplex' - default;
0024 %                       'tomlab_cplex')
0025 %
0026 % OUTPUT
0027 % IEMSol                Predicted biomarker fluxes and comparison with the
0028 %                       reported biomarkers
0029 %
0030 % USAGE:
0031 % Exampe of preparation of a set of inputs to checkIEM_WBM
0032 %     R = {'_2OXOADPTm';'_2AMADPTm';'_r0879'};
0033 %     RxnsAll2 = '';
0034 %     for i = 1: length(R)
0035 %         RxnsAll = model.rxns(find(~cellfun(@isempty,strfind(model.rxns,R{i}))));
0036 %         RxnsAll2 =[RxnsAll2;RxnsAll];
0037 %     end
0038 %     IEMRxns = unique(RxnsAll2);
0039 %     RxnMic = model.rxns(find(~cellfun(@isempty,strfind(model.rxns,'Micro_')))) ;
0040 %     IEMRxns = setdiff(IEMRxns,RxnMic);
0041 %
0042 %     if ~strcmp(modelName,'Recon3D')
0043 %         % add demand reactions to blood compartment for those biomarkers reported for blood
0044 %         % biomarker based on https://www.omim.org/entry/204750
0045 %         model = addDemandReaction(model, 'L2aadp[bc]');
0046 %
0047 %         BiomarkerRxns = {
0048 %             'DM_L2aadp[bc]'    'Increased (blood)'
0049 %             'EX_2oxoadp[u]'    'Increased (urine)'
0050 %             'EX_adpoh[u]'    'Increased (urine)'
0051 %             };
0052 %     else
0053 %         BiomarkerRxns = {
0054 %             'EX_2oxoadp[u]'    'Increased (urine)'
0055 %             'EX_adpoh[u]'    'Increased (urine)'
0056 %             };
0057 %     end
0058 %
0059 % Then call the checkIEM_WBM function
0060 %     [IEMSol_2OAA] = checkIEM_WBM(model,IEMRxns, BiomarkerRxns,minRxnsFluxHealthy);
0061 %
0062 % Example IEMSol returned from the above
0063 %     {'IEM Rxns All obj - Healthy'}    {'65403.5393' }    {0×0 double                    }
0064 %     {'IEM Rxns All obj - Disease'}    {'65403.5393' }    {0×0 double                    }
0065 %     {'WB obj - Healthy'          }    {'NA'         }    {0×0 double                    }
0066 %     {'WB obj - Disease'          }    {'1'          }    {'1'                           }
0067 %     {'Healthy:DM_L2aadp[bc]'     }    {'-5.5324e-08'}    {'Disease - Reported:Increas…'}
0068 %     {'Disease:DM_L2aadp[bc]'     }    {'40.2418'    }    {'Disease - Reported:Increas…'}
0069 %     {'Healthy:EX_2oxoadp[u]'     }    {'-2.3603e-10'}    {'Disease - Reported:Increas…'}
0070 %     {'Disease:EX_2oxoadp[u]'     }    {'3.7368'     }    {'Disease - Reported:Increas…'}
0071 %     {'Healthy:EX_adpoh[u]'       }    {'-1.6985e-10'}    {'Disease - Reported:Increas…'}
0072 %     {'Disease:EX_adpoh[u]'       }    {'3.7368'     }    {'Disease - Reported:Increas…'}
0073 %
0074 % 2018 - Ines Thiele
0075 % 2019 - Ines Thiele, included more options (minBiomarker,fixIEMlb,LPSolver)
0076 
0077 if ~exist('minRxnsFluxHealthy','var')
0078     minRxnsFluxHealthy = 0.75;
0079 end
0080 if ~exist('reverseDirObj','var')
0081     reverseDirObj = 0;
0082 end
0083 
0084 if ~exist('fractionKO','var')
0085     fractionKO = 1;% complete KO
0086 end
0087 if ~exist('minBiomarker','var')
0088     minBiomarker = 0;% no minimization of flux through biomarkers
0089 end
0090 
0091 if ~exist('fixIEMlb','var')
0092     fixIEMlb = 0;% lb = 0 for IEM rxns, while ub is constraint to (1-fractionKO)*solution.v(find(model.c));
0093 end
0094 
0095 if ~exist('LPSolver','var')
0096     LPSolver = 'ILOGcomplex';
0097     LPSolver = 'tomlab_cplex';
0098 end
0099 [solverOK, solverInstalled] = changeCobraSolver(LPSolver, 'LP',0,1);
0100 
0101 global useSolveCobraLPCPLEX
0102 %
0103 cnt = 1;
0104 tol = 1e-6;
0105 
0106 if useSolveCobraLPCPLEX
0107     [r,c] = size(model.A);
0108     % dummy rxn obj
0109     model.A(r+1,c+1) = -1;
0110     for i = 1 : length(IEMRxns)
0111         model.A(r+1,strmatch(IEMRxns{i},model.rxns,'exact')) = 1;
0112     end
0113     model.b(r+1) = 0;
0114     model.csense(r+1)='E';
0115     model.lb(c+1)=-100000;
0116     model.ub(c+1)=100000;
0117     model.c = zeros(size(model.A,2),1);
0118     model.c(c+1) = 1;
0119 else
0120     [r,c] = size(model.S);
0121     % dummy rxn obj
0122     model.S(r+1,c+1) = -1;
0123     for i = 1 : length(IEMRxns)
0124         model.S(r+1,strmatch(IEMRxns{i},model.rxns,'exact')) = 1;
0125     end
0126     model.b(r+1) = 0;
0127     model.csense(r+1)='E';
0128     model.lb(c+1)=-100000;
0129     model.ub(c+1)=100000;
0130     model.c = zeros(size(model.S,2),1);
0131     model.c(c+1) = 1;
0132     if isfield(model,'C')
0133         %pad coupling constraints
0134         model.C = [model.C, sparse(size(model.C,1),1)];
0135     end
0136 end
0137 
0138 if reverseDirObj ==  1 % minimize obj
0139     model.osense = 1;
0140 else
0141     model.osense = -1;
0142 end
0143 
0144 % maximize joint objective in healthy model
0145 tic;
0146 if useSolveCobraLPCPLEX
0147     [solution,~]=solveCobraLPCPLEX(model,1,0,0,[],0,'ILOGcomplex');
0148     solution.v=solution.full;
0149 else
0150     model.osenseStr='max';
0151     solution = optimizeWBModel(model);
0152 end
0153 timeTaken=toc;
0154 IEMSol{cnt,1} = 'IEM Rxns All obj - Healthy';
0155 
0156 %cplex origStat code meanings
0157 %       1 (S,B) Optimal solution found
0158 %       2 (S,B) Model has an unbounded ray
0159 %       3 (S,B) Model has been proved infeasible
0160 %       4 (S,B) Model has been proved either infeasible or unbounded
0161 %       5 (S,B) Optimal solution is available, but with infeasibilities after unscaling
0162 %       6 (S,B) Solution is available, but not proved optimal, due to numeric difficulties
0163 
0164 %the original status codes
0165 %if solution.origStat ~= 3 && solution.origStat ~= 5
0166 if solution.stat == 1 || solution.stat == 3 %only proceed if the wild type was optimal, or almost optimal
0167     IEMSol{cnt,2} = num2str(solution.v(model.c~=0));cnt = cnt + 1;
0168     if  abs(solution.v(model.c~=0)) > 1e-6
0169         
0170         % Healthy: set organ reactions to be at least 75% of max value
0171         model.lb(c+1) = minRxnsFluxHealthy*solution.v(model.c~=0);
0172         model.lb(c+1)=fix(model.lb(c+1)*1000000)/1000000;% remove the last few digits from the 16 digits allowed by matlab
0173         % Disease: set them all to 0
0174         modelIEM = model;
0175         if fixIEMlb == 1
0176             modelIEM.lb(c+1) = (1-fractionKO)*solution.v(model.c~=0);
0177             modelIEM.lb(c+1)=fix(modelIEM.lb(c+1)*1000000)/1000000;% remove the last few digits from the 16 digits allowed by matlab
0178             
0179         else
0180             modelIEM.lb(c+1) = 0;
0181         end
0182         modelIEM.ub(c+1) = (1-fractionKO)*solution.v(model.c~=0);
0183         modelIEM.ub(c+1)=fix(modelIEM.lb(c+1)*1000000)/1000000;% remove the last few digits from the 16 digits allowed by matlab
0184         
0185         tic;
0186         if useSolveCobraLPCPLEX
0187             [solution,~]=solveCobraLPCPLEX(modelIEM,1,0,0,[],0,'ILOGcomplex');
0188             solution.v=solution.full;
0189         else
0190             %note model.osense set above ~line 135
0191             solution = optimizeWBModel(modelIEM);
0192         end
0193         timeTaken=toc;
0194         IEMSol{cnt,1} = 'IEM Rxns All obj - Disease';
0195         
0196         %if solution.origStat ~= 3 && solution.origStat ~= 5% feasible solution
0197         if solution.stat == 1 || solution.stat == 3
0198             f = solution.v(modelIEM.c~=0);
0199             if abs(f) <= tol
0200                 f = 0;
0201             end
0202             IEMSol{cnt,2} = num2str(f);cnt = cnt + 1;
0203             % IEMSol{cnt,3} = num2str(solution.origStat);cnt = cnt + 1;
0204         else
0205             IEMSol{cnt,2} = 'NaN';
0206             cnt = cnt + 1;
0207         end
0208         
0209         % check that healthy model is still feasible
0210         model = changeObjective(model,'Whole_body_objective_rxn');
0211         model.osenseStr = 'max';
0212         IEMSol{cnt,1} = 'WB obj - Healthy';
0213         IEMSol{cnt,2} = 'ND';cnt = cnt + 1;
0214         
0215         %if solution.origStat ~= 3 && solution.origStat ~= 5% feasible solution
0216         if solution.stat == 1 || solution.stat == 3
0217             modelO = model;
0218             modelIEMO = modelIEM;
0219             
0220             % is biomass maintenance feasible
0221             modelIEM = changeObjective(modelIEM,'Whole_body_objective_rxn');
0222             modelIEM.osense = -1;
0223             tic;
0224             if useSolveCobraLPCPLEX
0225                 [solution,~]=solveCobraLPCPLEX(modelIEM,1,0,0,[],0,'ILOGcomplex');
0226                 solution.v=solution.full;
0227             else
0228                 solution = optimizeWBModel(modelIEM);
0229             end
0230             timeTaken=toc;
0231             IEMSol{cnt,1} = 'WB obj - Disease';
0232             if solution.stat == 1 || solution.stat == 3
0233                 f = solution.v(modelIEM.c~=0);
0234                 if abs(f) <= tol
0235                     f = 0;
0236                 end
0237                 IEMSol{cnt,2} = num2str(f);
0238             else
0239                 IEMSol{cnt,2} = NaN;
0240             end
0241             IEMSol{cnt,3} = num2str(solution.origStat);cnt = cnt + 1;
0242             % IEMSol{cnt,2} = 'NA';cnt = cnt + 1;
0243             
0244             %if solution.origStat ~= 3 && solution.origStat ~= 5% feasible
0245             if solution.stat == 1 || solution.stat == 3
0246                 for i = 1 : size(BiomarkerRxns,1)
0247                     model = modelO;
0248                     %displaying the biomarker being predicted helps to
0249                     %track progress
0250                     disp(BiomarkerRxns{i,1})
0251                     % Healthy
0252                     model.ub(strmatch(BiomarkerRxns{i,1},model.rxns)) = 100000;
0253                     model = changeObjective(model,BiomarkerRxns{i,1});
0254                     % max of biomarker
0255                     model.osenseStr = 'max';
0256                     model.osense = -1;
0257                     tic;
0258                     if useSolveCobraLPCPLEX
0259                         [solution,~]=solveCobraLPCPLEX(model,1,0,0,[],0,'ILOGcomplex');
0260                         solution.v=solution.full;
0261                     else
0262                         solution = optimizeWBModel(model);
0263                         if solution.origStat == 3 % in the case that the solution is returned infeasible, which can happen due to numerical difficulties of the cplex solver, remove some more digits from the constrain. This does not change the solution. Note if the function went until here the model itself is feasible as only the objective function is changed from the previous simulation.
0264                             model.lb(c+1)=fix(model.lb(c+1)*10000)/10000;
0265                             solution = optimizeWBModel(model);
0266                         end
0267                         
0268                     end
0269                     timeTaken=toc;
0270                     IEMSol{cnt,1} = strcat('Healthy:',BiomarkerRxns{i,1});
0271                     if solution.stat == 1 || solution.stat == 3
0272                         f = solution.v(model.c~=0);
0273                         if abs(f) <= tol
0274                             f = 0;
0275                         end
0276                         IEMSol{cnt,2} = num2str(f);
0277                     else
0278                         IEMSol{cnt,2} = 'NaN';
0279                     end
0280                     IEMSol{cnt,3} = strcat('Disease - Reported:',BiomarkerRxns{i,2});
0281                     % minimization of biomarker
0282                     if minBiomarker == 1
0283                         % min of biomarker
0284                         model.osense = 1;
0285                         model.osenseStr = 'min';
0286                         tic;
0287                         if useSolveCobraLPCPLEX
0288                             [solution,~]=solveCobraLPCPLEX(model,1,0,0,[],0,'ILOGcomplex');
0289                             solution.v=solution.full;
0290                         else
0291                             solution = optimizeWBModel(model);
0292                         end
0293                         timeTaken=toc;
0294                         if solution.stat == 1 || solution.stat == 3
0295                             f = solution.v(model.c~=0);
0296                             if abs(f) <= tol
0297                                 f = 0;
0298                             end
0299                             IEMSol{cnt,4} = num2str(solution.v(model.c~=0));
0300                         else
0301                             IEMSol{cnt,4} = 'NaN';
0302                         end
0303                     end
0304                     cnt = cnt + 1;
0305                     %KO
0306                     modelIEM = modelIEMO;
0307                     modelIEM.ub(strmatch(BiomarkerRxns{i,1},modelIEM.rxns)) = 100000;
0308                     modelIEM = changeObjective(modelIEM,BiomarkerRxns{i,1});
0309                     modelIEM.osense = -1;
0310                     tic;
0311                     if useSolveCobraLPCPLEX
0312                         [solution,~]=solveCobraLPCPLEX(modelIEM,1,0,0,[],0,'ILOGcomplex');
0313                         solution.v=solution.full;
0314                     else
0315                         solution = optimizeWBModel(modelIEM);
0316                         if solution.origStat == 3 % in the case that the solution is returned infeasible, which can happen due to numerical difficulties of the cplex solver, remove some more digits from the constrain. This does not change the solution. Note if the function went until here the model itself is feasible as only the objective function is changed from the previous simulation.
0317                             modelIEM.lb(c+1)=fix(modelIEM.lb(c+1)*10000)/10000;
0318                             solution = optimizeWBModel(modelIEM);
0319                         end
0320                     end
0321                     timeTaken=toc;
0322                     IEMSol{cnt,1} = strcat('Disease:',BiomarkerRxns{i,1});
0323                     if solution.stat == 1 || solution.stat == 3
0324                         f = solution.v(modelIEM.c~=0);
0325                         if abs(f) <= tol
0326                             f = 0;
0327                         end
0328                         IEMSol{cnt,2} = num2str(solution.v(modelIEM.c~=0));
0329                     else
0330                         IEMSol{cnt,2} = 'NaN';
0331                     end
0332                     IEMSol{cnt,3} =strcat('Disease - Reported:',BiomarkerRxns{i,2});
0333                     % minimization of biomarker
0334                     if minBiomarker == 1
0335                         % min of biomarker
0336                         modelIEM.osense = 1;
0337                         tic;
0338                         if useSolveCobraLPCPLEX
0339                             [solution,~]=solveCobraLPCPLEX(modelIEM,1,0,0,[],0,'ILOGcomplex');
0340                             solution.v=solution.full;
0341                         else
0342                             solution = optimizeWBModel(modelIEM);
0343                         end
0344                         timeTaken=toc;
0345                         if solution.stat == 1 || solution.stat == 3
0346                             f = solution.v(modelIEM.c~=0);
0347                             if abs(f) <= tol
0348                                 f = 0;
0349                             end
0350                             IEMSol{cnt,4} = num2str(f);
0351                         else
0352                             IEMSol{cnt,4} = 'NaN';
0353                         end
0354                     end
0355                     cnt = cnt + 1;
0356                 end
0357             end
0358         end
0359     end
0360 end
```

---

Generated on Thu 14-May-2020 13:05:49 by **m2html** © 2005
