# Supplementary material for: Personalized whole‐body models integrate metabolism, physiology, and the gut microbiome
Source: Mol Syst Biol. 2020 May 28;16(5):e8982. doi: 10.15252/msb.20198982 (PMC7285886; doi:10.15252/msb.20198982)
Supplement: Supplementary file 22 — Dataset EV1 [file MSB-16-e8982-s022.zip › PSCM_toolbox/PSCM_toolbox_doc/src/performSanityChecksonRecon.html]

Description of performSanityChecksonRecon


# performSanityChecksonRecon

## PURPOSE

**This function performs various quality control and quality assurance**

## SYNOPSIS

**function [TableChecks, Table\_csources, CSourcesTestedRxns, TestSolutionNameOpenSinks,TestSolutionNameClosedSinks] = performSanityChecksonRecon(model,resultsFileName,ExtraCellCompIn,ExtraCellCompOut,runSingleGeneDeletion)**

## DESCRIPTION

```
 This function performs various quality control and quality assurance
 tests.
 [TableChecks, Table_csources, CSourcesTestedRxns, TestSolutionNameOpenSinks,TestSolutionNameClosedSinks] = performSanityChecksonRecon(model,resultsFileName,ExtraCellCompIn,ExtraCellCompOut,runSingleGeneDeletion)

 INPUT
 model                         model structure
 resultsFileName               File name of the generated output file 
 ExtraCellCompIn               [e] compartment by default, if extracellular
                               uptake compartment is named differently, it
                               can be specified here
 ExtraCellCompOut              [e] compartment by default, if extracellular
                               secretion compartment is named differently, it
                               can be specified here
 runSingleGeneDeletion         if 0 (default): function does not run single gene deletion otw choose 1

 OUTPUT
 TableChecks                   Table overview of the performed tests and
                               their outcomes
 Table_csources                Table the test results for ATP yield from
                               various carbon scources under aerobic and anaerobic conditions
 CSourcesTestedRxns            List of reactions active when testing the ATP
                               yield from the various carbon sources
```

## CROSS-REFERENCE INFORMATION

This function calls:

- Test4HumanFctExtv5 % function [TestSolution,TestSolutionName] = Test4HumanFct(model,test)
- LeakTestRecon changeCobraSolver('tomlab\_cplex','lp');

This function is called by:

- getOrgansFromHarvey This function cuts the organs from the whole-body metabolic model. Note that the different

## SOURCE CODE

```
0001 function [TableChecks, Table_csources, CSourcesTestedRxns, TestSolutionNameOpenSinks,TestSolutionNameClosedSinks] = performSanityChecksonRecon(model,resultsFileName,ExtraCellCompIn,ExtraCellCompOut,runSingleGeneDeletion)
0002 % This function performs various quality control and quality assurance
0003 % tests.
0004 % [TableChecks, Table_csources, CSourcesTestedRxns, TestSolutionNameOpenSinks,TestSolutionNameClosedSinks] = performSanityChecksonRecon(model,resultsFileName,ExtraCellCompIn,ExtraCellCompOut,runSingleGeneDeletion)
0005 %
0006 % INPUT
0007 % model                         model structure
0008 % resultsFileName               File name of the generated output file
0009 % ExtraCellCompIn               [e] compartment by default, if extracellular
0010 %                               uptake compartment is named differently, it
0011 %                               can be specified here
0012 % ExtraCellCompOut              [e] compartment by default, if extracellular
0013 %                               secretion compartment is named differently, it
0014 %                               can be specified here
0015 % runSingleGeneDeletion         if 0 (default): function does not run single gene deletion otw choose 1
0016 %
0017 % OUTPUT
0018 % TableChecks                   Table overview of the performed tests and
0019 %                               their outcomes
0020 % Table_csources                Table the test results for ATP yield from
0021 %                               various carbon scources under aerobic and anaerobic conditions
0022 % CSourcesTestedRxns            List of reactions active when testing the ATP
0023 %                               yield from the various carbon sources
0024 
0025 % TestSolutionNameOpenSinks     List of results when testing for 460 metabolic
0026 %                               functions with all sinks open
0027 % TestSolutionNameClosedSinks   List of results when testing for 460 metabolic
0028 %                               functions with all sinks closed
0029 %
0030 % Ines Thiele 2016-2019
0031 
0032 global resultsPath
0033 resultsPath = which('MethodSection3.mlx');
0034 resultsPath = strrep(resultsPath,'MethodSection3.mlx',['Results' filesep]);
0035 
0036 if ~exist([resultsPath 'OrganChecks'],'dir')
0037     mkdir([resultsPath 'OrganChecks'])
0038 end
0039 
0040 if ~exist('ExtraCellCompIn','var')
0041     ExtraCellCompIn = '[e]'; % [e] compartment by default
0042 end
0043 if ~exist('ExtraCellCompOut','var')
0044     ExtraCellCompOut = '[e]'; % [e] compartment by default
0045 end
0046 
0047 if ~exist('runSingleGeneDeletion','var')
0048     runSingleGeneDeletion = 0; % do not run single gene deletion by default
0049 end
0050 
0051 global saveDiary
0052 if saveDiary
0053     %save each diary to PSCM/Results/OrganChecks/
0054     global resultsPath
0055     resultsPath = which('MethodSection3.mlx');
0056     resultsPath = strrep(resultsPath,'MethodSection3.mlx',['Results' filesep]);
0057     resultsFileName=[resultsFileName '_diary'];
0058     diary([resultsPath 'OrganChecks' filesep resultsFileName])
0059 end
0060 
0061 % if 1
0062 %     changeCobraSolver('tomlab_cplex','lp');
0063 %     changeCobraSolver('tomlab_cplex','qp');
0064 % end
0065 cnt = 1;
0066 tol = 1e-6;
0067 
0068 model.rxns(find(ismember(model.rxns,'ATPM')))={'DM_atp_c_'};
0069 model.rxns(find(ismember(model.rxns,'ATPhyd')))={'DM_atp_c_'};
0070 % adds DM_atp to model if not exist
0071 
0072 if isempty(strmatch('DM_atp_c_',model.rxns))
0073     [model, rxnIDexists] = addReaction(model,'DM_atp_c_', 'reactionFormula', 'h2o[c] + atp[c]  -> adp[c] + h[c] + pi[c] ');
0074 end
0075 
0076 model.rxns(find(ismember(model.rxns,'EX_biomass_reaction')))={'biomass_reaction'};
0077 model.rxns(find(ismember(model.rxns,'EX_biomass_maintenance')))={'biomass_maintenance'};
0078 model.rxns(find(ismember(model.rxns,'EX_biomass_maintenance_noTrTr')))={'biomass_maintenance_noTrTr'};
0079 model.lb(find(ismember(model.rxns,'biomass_reaction')))=0;
0080 model.lb(find(ismember(model.rxns,'biomass_maintenance_noTrTr')))=0;
0081 model.lb(find(ismember(model.rxns,'biomass_maintenance')))=0;
0082 
0083 
0084 TestSolutionNameOpenSinks ='';
0085 TestSolutionNameClosedSinks = '';
0086 
0087 model.rxns = regexprep(model.rxns,'\(','\[');
0088 model.rxns = regexprep(model.rxns,'\)','\]');
0089 % vanilla leak test
0090 
0091 if 1
0092     modelClosed = model;
0093     LeakTestRecon;
0094     TableChecks{cnt,1} = 'fastLeakTest 1';
0095     if length(LeakMets)>0
0096         warning('model leaks metabolites!')
0097         TableChecks{cnt,2} = 'Model leaks metabolites!';
0098     else
0099         TableChecks{cnt,2} = 'Leak free!';
0100     end
0101     cnt = cnt + 1;
0102 end
0103 
0104 if 0
0105     % test if something leaks when demand are added
0106     modelClosed = model;
0107     modelexchanges1 = strmatch('Ex_',modelClosed.rxns);
0108     modelexchanges4 = strmatch('EX_',modelClosed.rxns);
0109     modelexchanges2 = strmatch('DM_',modelClosed.rxns);
0110     modelexchanges3 = strmatch('sink_',modelClosed.rxns);
0111     selExc = (find( full((sum(abs(modelClosed.S)==1,1) ==1) & (sum(modelClosed.S~=0) == 1))))';
0112     
0113     modelexchanges = unique([modelexchanges1;modelexchanges2;modelexchanges3;modelexchanges4;selExc]);
0114     modelClosed.lb(find(ismember(modelClosed.rxns,modelClosed.rxns(modelexchanges))))=0;
0115     [modelClosed,rxnNames] = addDemandReaction(modelClosed,modelClosed.mets);
0116     
0117     [LeakRxns,modelTested] = fastLeakTest(modelClosed,modelClosed.rxns(selExc));
0118     
0119     TableChecks{cnt,1} = 'fastLeakTest 2 - add demand reactions for each metabolite in the model';
0120     if length(LeakMets)>0
0121         TableChecks{cnt,2} = 'Model leaks metabolites when demand reactions are added!';
0122     else
0123         TableChecks{cnt,2} = 'Leak free when demand reactions are added!';
0124     end
0125     cnt = cnt + 1;
0126 end
0127 
0128 if 1
0129     %%model produces energy from water!
0130     modelClosed = model;
0131     
0132     modelexchanges1 = strmatch('Ex_',modelClosed.rxns);
0133     modelexchanges4 = strmatch('EX_',modelClosed.rxns);
0134     modelexchanges2 = strmatch('DM_',modelClosed.rxns);
0135     modelexchanges3 = strmatch('sink_',modelClosed.rxns);
0136     selExc = (find( full((sum(abs(modelClosed.S)==1,1) ==1) & (sum(modelClosed.S~=0) == 1))))';
0137     
0138     modelexchanges = unique([modelexchanges1;modelexchanges2;modelexchanges3;modelexchanges4;selExc]);
0139     modelClosed.lb(find(ismember(modelClosed.rxns,modelClosed.rxns(modelexchanges))))=0;
0140     modelClosedATP = changeObjective(modelClosed,'DM_atp_c_');
0141     modelClosedATP = changeRxnBounds(modelClosedATP,'DM_atp_c_',0,'l');
0142     modelClosedATP = changeRxnBounds(modelClosedATP,strcat('EX_h2o',ExtraCellCompIn),-1,'l');
0143     FBA3=optimizeCbModel(modelClosedATP);
0144     TableChecks{cnt,1} = 'Exchanges, sinks, and demands have  lb = 0, except h2o';
0145     if abs(FBA3.f) > 1e-6
0146         TableChecks{cnt,2} = 'model produces energy from water!';
0147     else
0148         TableChecks{cnt,2} = 'model DOES NOT produce energy from water!';
0149     end
0150     cnt = cnt + 1;
0151 end
0152 %% model produces energy from water and oxygen!
0153 if 1
0154     modelClosed = model;
0155     modelexchanges1 = strmatch('Ex_',modelClosed.rxns);
0156     modelexchanges4 = strmatch('EX_',modelClosed.rxns);
0157     modelexchanges2 = strmatch('DM_',modelClosed.rxns);
0158     modelexchanges3 = strmatch('sink_',modelClosed.rxns);
0159     selExc = (find( full((sum(abs(modelClosed.S)==1,1) ==1) & (sum(modelClosed.S~=0) == 1))))';
0160     
0161     modelexchanges = unique([modelexchanges1;modelexchanges2;modelexchanges3;modelexchanges4;selExc]);
0162     modelClosed.lb(find(ismember(modelClosed.rxns,modelClosed.rxns(modelexchanges))))=0;
0163     modelClosedATP = changeObjective(modelClosed,'DM_atp_c_');
0164     modelClosedATP = changeRxnBounds(modelClosedATP,'DM_atp_c_',0,'l');
0165     modelClosedATP = changeRxnBounds(modelClosedATP,strcat('EX_h2o',ExtraCellCompIn),-1,'l');
0166     modelClosedATP = changeRxnBounds(modelClosedATP,strcat('EX_o2',ExtraCellCompIn),-1,'l');
0167     
0168     FBA6=optimizeCbModel(modelClosedATP);
0169     TableChecks{cnt,1} = 'Exchanges, sinks, and demands have  lb = 0, except h2o and o2';
0170     if abs(FBA6.f) > 1e-6
0171         TableChecks{cnt,2} = 'model produces energy from water and oxygen!';
0172     else
0173         TableChecks{cnt,2} = 'model DOES NOT produce energy from water and oxygen!';
0174     end
0175     cnt = cnt + 1;
0176 end
0177 %% model produces matter when atp demand is reversed!
0178 if 1
0179     modelClosed = model;
0180     modelexchanges1 = strmatch('Ex_',modelClosed.rxns);
0181     modelexchanges4 = strmatch('EX_',modelClosed.rxns);
0182     modelexchanges2 = strmatch('DM_',modelClosed.rxns);
0183     modelexchanges3 = strmatch('sink_',modelClosed.rxns);
0184     selExc = (find( full((sum(abs(modelClosed.S)==1,1) ==1) & (sum(modelClosed.S~=0) == 1))))';
0185     
0186     modelexchanges = unique([modelexchanges1;modelexchanges2;modelexchanges3;modelexchanges4;selExc]);
0187     modelClosed.lb(find(ismember(modelClosed.rxns,modelClosed.rxns(modelexchanges))))=0;
0188     
0189     modelClosed = changeObjective(modelClosed,'DM_atp_c_');
0190     modelClosed.lb(find(ismember(modelClosed.rxns,'DM_atp_c_'))) = -1000;
0191     modelClosed.ub(selExc)=1000;
0192     FBA = optimizeCbModel(modelClosed);
0193     TableChecks{cnt,1} = 'Exchanges, sinks, and demands have  lb = 0, allow DM_atp_c_ to be reversible';
0194     if abs(FBA.f) > 1e-6
0195         TableChecks{cnt,2} = 'model produces matter when atp demand is reversed!';
0196     else
0197         TableChecks{cnt,2} = 'model DOES NOT produce matter when atp demand is reversed!';
0198     end
0199     cnt = cnt + 1;
0200 end
0201 %% model has flux through h[m] demand !
0202 if 1
0203     modelClosed = model;
0204     modelexchanges1 = strmatch('Ex_',modelClosed.rxns);
0205     modelexchanges4 = strmatch('EX_',modelClosed.rxns);
0206     modelexchanges2 = strmatch('DM_',modelClosed.rxns);
0207     modelexchanges3 = strmatch('sink_',modelClosed.rxns);
0208     selExc = (find( full((sum(abs(modelClosed.S)==1,1) ==1) & (sum(modelClosed.S~=0) == 1))))';
0209     
0210     modelexchanges = unique([modelexchanges1;modelexchanges2;modelexchanges3;modelexchanges4;selExc]);
0211     modelClosed.lb(find(ismember(modelClosed.rxns,modelClosed.rxns(modelexchanges))))=0;
0212     modelClosed = addDemandReaction(modelClosed,'h[m]');
0213     modelClosed = changeObjective(modelClosed,'DM_h[m]');
0214     modelClosed.ub(find(ismember(modelClosed.rxns,'DM_h[m]'))) = 1000;
0215     modelClosed.ub(selExc)=1000;
0216     FBA = optimizeCbModel(modelClosed,'max');
0217     TableChecks{cnt,1} = 'Exchanges, sinks, and demands have  lb = 0, test flux through DM_h[m] (max)';
0218     if abs(FBA.f) > 1e-6
0219         TableChecks{cnt,2} = 'model has flux through h[m] demand (max)!';
0220     else
0221         TableChecks{cnt,2} = 'model has NO flux through h[m] demand (max)!';
0222     end
0223     cnt = cnt + 1;
0224 end
0225 if 0
0226     modelClosed = model;
0227     modelexchanges1 = strmatch('Ex_',modelClosed.rxns);
0228     modelexchanges4 = strmatch('EX_',modelClosed.rxns);
0229     modelexchanges2 = strmatch('DM_',modelClosed.rxns);
0230     modelexchanges3 = strmatch('sink_',modelClosed.rxns);
0231     selExc = (find( full((sum(abs(modelClosed.S)==1,1) ==1) & (sum(modelClosed.S~=0) == 1))))';
0232     
0233     modelexchanges = unique([modelexchanges1;modelexchanges2;modelexchanges3;modelexchanges4;selExc]);
0234     modelClosed.lb(find(ismember(modelClosed.rxns,modelClosed.rxns(modelexchanges))))=0;
0235     modelClosed = addDemandReaction(modelClosed,'h[m]');
0236     modelClosed = changeObjective(modelClosed,'DM_h[m]');
0237     modelClosed.ub(find(ismember(modelClosed.rxns,'DM_h[m]'))) = 1000;
0238     modelClosed.lb(find(ismember(modelClosed.rxns,'DM_h[m]'))) = -1000;
0239     modelClosed.ub(selExc)=1000;
0240     FBA = optimizeCbModel(modelClosed,'min');
0241     TableChecks{cnt,1} = 'Exchanges, sinks, and demands have  lb = 0, test flux through DM_h[m] (min)';
0242     if abs(FBA.f) > 1e-6
0243         TableChecks{cnt,2} = 'model has flux through h[m] demand (min)!';
0244     else
0245         TableChecks{cnt,2} = 'model has NO flux through h[m] demand (min)!';
0246     end
0247     cnt = cnt + 1;
0248 end
0249 %% model has flux through h[c] demand !
0250 if 1
0251     modelClosed = model;
0252     modelexchanges1 = strmatch('Ex_',modelClosed.rxns);
0253     modelexchanges4 = strmatch('EX_',modelClosed.rxns);
0254     modelexchanges2 = strmatch('DM_',modelClosed.rxns);
0255     modelexchanges3 = strmatch('sink_',modelClosed.rxns);
0256     selExc = (find( full((sum(abs(modelClosed.S)==1,1) ==1) & (sum(modelClosed.S~=0) == 1))))';
0257     
0258     modelexchanges = unique([modelexchanges1;modelexchanges2;modelexchanges3;modelexchanges4;selExc]);
0259     modelClosed.lb(find(ismember(modelClosed.rxns,modelClosed.rxns(modelexchanges))))=0;
0260     modelClosed = addDemandReaction(modelClosed,'h[c]');
0261     modelClosed = changeObjective(modelClosed,'DM_h[c]');
0262     modelClosed.ub(find(ismember(modelClosed.rxns,'DM_h[c]'))) = 1000;
0263     modelClosed.ub(selExc)=1000;
0264     FBA = optimizeCbModel(modelClosed,'max');
0265     TableChecks{cnt,1} = 'Exchanges, sinks, and demands have  lb = 0, test flux through DM_h[c] (max)';
0266     if abs(FBA.f) > 1e-6
0267         TableChecks{cnt,2} = 'model has flux through h[c] demand (max)!';
0268     else
0269         TableChecks{cnt,2} = 'model has NO flux through h[c] demand (max)!';
0270     end
0271     cnt = cnt + 1;
0272 end
0273 if 1
0274     modelClosed = model;
0275     modelexchanges1 = strmatch('Ex_',modelClosed.rxns);
0276     modelexchanges4 = strmatch('EX_',modelClosed.rxns);
0277     modelexchanges2 = strmatch('DM_',modelClosed.rxns);
0278     modelexchanges3 = strmatch('sink_',modelClosed.rxns);
0279     selExc = (find( full((sum(abs(modelClosed.S)==1,1) ==1) & (sum(modelClosed.S~=0) == 1))))';
0280     
0281     modelexchanges = unique([modelexchanges1;modelexchanges2;modelexchanges3;modelexchanges4;selExc]);
0282     modelClosed.lb(find(ismember(modelClosed.rxns,modelClosed.rxns(modelexchanges))))=0;
0283     modelClosed = addDemandReaction(modelClosed,'h[c]');
0284     modelClosed = changeObjective(modelClosed,'DM_h[c]');
0285     modelClosed.ub(find(ismember(modelClosed.rxns,'DM_h[c]'))) = 1000;
0286     modelClosed.lb(find(ismember(modelClosed.rxns,'DM_h[c]'))) = -1000;
0287     modelClosed.ub(selExc)=1000;
0288     modelClosed.ub(find(ismember(modelClosed.rxns,strcat('EX_h',ExtraCellCompOut)))) = 0;
0289     FBA = optimizeCbModel(modelClosed,'min');
0290     TableChecks{cnt,1} = 'Exchanges, sinks, and demands have  lb = 0, ub of EX_h[e] = 0, test flux through DM_h[c] (min)';
0291     if abs(FBA.f) > 1e-6
0292         TableChecks{cnt,2} = 'model has flux through h[c] demand (min)!';
0293     else
0294         TableChecks{cnt,2} = 'model has NO flux through h[c] demand (min)!';
0295     end
0296     cnt = cnt + 1;
0297 end
0298 %% model produces too much atp demand from glc -- old remove
0299 if 0
0300     modelClosed = model;
0301     modelexchanges1 = strmatch('Ex_',modelClosed.rxns);
0302     modelexchanges4 = strmatch('EX_',modelClosed.rxns);
0303     modelexchanges2 = strmatch('DM_',modelClosed.rxns);
0304     modelexchanges3 = strmatch('sink_',modelClosed.rxns);
0305     selExc = (find( full((sum(abs(modelClosed.S)==1,1) ==1) & (sum(modelClosed.S~=0) == 1))))';
0306     
0307     modelexchanges = unique([modelexchanges1;modelexchanges2;modelexchanges3;modelexchanges4;selExc]);
0308     modelClosed.lb(find(ismember(modelClosed.rxns,modelClosed.rxns(modelexchanges))))=0;
0309     modelClosed.c = zeros(length(modelClosed.rxns),1);
0310     modelClosed = changeObjective(modelClosed,'DM_atp_c_');
0311     modelClosed.lb(find(ismember(modelClosed.rxns,strcat('EX_o2',ExtraCellCompIn)))) = -1000;
0312     modelClosed.lb(find(ismember(modelClosed.rxns,strcat('EX_h2o',ExtraCellCompIn)))) = -1000;
0313     modelClosed.ub(find(ismember(modelClosed.rxns,strcat('EX_h2o',ExtraCellCompOut)))) = 1000;
0314     modelClosed.ub(find(ismember(modelClosed.rxns,strcat('EX_co2',ExtraCellCompOut)))) = 1000;
0315     %modelClosed.lb(find(ismember(modelClosed.rxns,'EX_pi[e]'))) = -1000;
0316     %modelClosed = addExchangeRxn(modelClosed,{'glc_D[e]'});
0317     modelClosed.lb(find(ismember(modelClosed.rxns,strcat('EX_glc_D',ExtraCellCompIn)))) = -1;
0318     modelClosed.ub(find(ismember(modelClosed.rxns,strcat('EX_glc_D',ExtraCellCompIn)))) = -1;
0319     
0320     modelClosed.ub(selExc)=1000;
0321     %    FBA = optimizeCbModel(modelClosed,'max',1e-6);
0322     FBA = optimizeCbModel(modelClosed,'max');
0323     
0324     TableChecks{cnt,1} = 'ATP yield ';
0325     if abs(FBA.f) > 1e-6
0326         FBA.f
0327         %  modelClosed = changeObjective(modelClosed,'ATPS4m');
0328         %  FBA = optimizeCbModel(modelClosed,'max',1e-6);
0329         FBA.x(find(ismember(modelClosed.rxns,'ATPS4m')))
0330         % prepare table
0331 
0332         warning('model produces too much atp demand from glc!');
0333     else
0334         warning('model DOES NOT produce too much atp demand from glc!');
0335     end
0336     
0337     
0338 end
0339 
0340 TableChecks{cnt,1} = 'Test metabolic objective functions with open sinks';
0341 if 1 % perform test function
0342     [TestSolution,TestSolutionNameOpenSinks, TestedRxnsSinks, PercSinks] = Test4HumanFctExtv5(model,'all');
0343     TableChecks{cnt,2} = strcat('Done. See variable TestSolutionNameOpenSinks for results. The model passes ', num2str(length(find(abs(TestSolution)>tol))),' out of ', num2str(length(TestSolution)), 'tests');
0344 else
0345     TableChecks{cnt,2} = 'Not performed.';
0346 end
0347 cnt =  cnt + 1;
0348 
0349 TableChecks{cnt,1} = 'Test metabolic objective functions with closed sinks (lb)';
0350 if 0 % perform test functions
0351     [TestSolution,TestSolutionNameClosedSinks, TestedRxnsClosedSinks, PercClosedSinks] = Test4HumanFctExtv5(model,'all',0);
0352     TableChecks{cnt,2} = strcat('Done. See variable TestSolutionNameClosedSinks for results. The model passes ', num2str(length(find(abs(TestSolution)>tol))),' out of ', num2str(length(TestSolution)), 'tests');
0353 else
0354     TableChecks{cnt,2} = 'Not performed.';
0355 end
0356 cnt =  cnt + 1;
0357 
0358 TableChecks{cnt,1} = 'Compute ATP yield';
0359 if 1 % test ATP yield
0360     [Table_csources, CSourcesTestedRxns, Perc] = testATPYieldFromCsources(model,[],ExtraCellCompIn,ExtraCellCompOut);
0361     TableChecks{cnt,2} = 'Done. See variable Table_csources for results.';
0362 else
0363     TableChecks{cnt,2} = 'Not performed.';
0364     CSourcesTestedRxns = '';
0365 end
0366 cnt = cnt + 1;
0367 
0368 TableChecks{cnt,1} = 'Check duplicated reactions';
0369 if 0
0370     method='FR';
0371     removeFlag=0;
0372     [modelOut,removedRxnInd, keptRxnInd] = checkDuplicateRxn(model,method,removeFlag);
0373     if isempty(removedRxnInd)
0374         TableChecks{cnt,2} = 'No duplicated reactions in model.';
0375     else
0376         TableChecks{cnt,2} = 'Duplicated reactions in model.';
0377     end
0378 else
0379     TableChecks{cnt,2} = 'Not performed.';
0380 end
0381 cnt = cnt + 1;
0382 
0383 TableChecks{cnt,1} = 'Check empty columns in rxnGeneMat';
0384 if 1
0385     E = find(sum(model.rxnGeneMat)==0);
0386     if isempty(E)
0387         TableChecks{cnt,2} = 'No empty columns in rxnGeneMat.';
0388     else
0389         TableChecks{cnt,2} = 'Empty columns in rxnGeneMat.';
0390     end
0391 else
0392     TableChecks{cnt,2} = 'Not performed.';
0393 end
0394 cnt = cnt + 1;
0395 
0396 TableChecks{cnt,1} = 'Check that demand reactions have a lb >= 0';
0397 if 1
0398     DMlb = find(model.lb(strmatch('DM_',model.rxns))<0);
0399     if isempty(DMlb)
0400         TableChecks{cnt,2} = 'No demand reaction can have flux in backward direction.';
0401     else
0402         TableChecks{cnt,2} = 'Demand reaction can have flux in backward direction.';
0403     end
0404 else
0405     TableChecks{cnt,2} = 'Not performed.';
0406 end
0407 cnt = cnt + 1;
0408 
0409 TableChecks{cnt,1} = 'Check consistency of model.rev with model.lb';
0410 if 1
0411     Rev = setdiff(find(model.lb<0), find(model.rev==1));
0412     if isempty(Rev)
0413         TableChecks{cnt,2} = 'model.rev and model.lb are consistent.';
0414     else
0415         TableChecks{cnt,2} = 'model.rev and model.lb are NOT consistent.';
0416     end
0417 else
0418     TableChecks{cnt,2} = 'Not performed.';
0419 end
0420 cnt = cnt + 1;
0421 
0422 TableChecks{cnt,1} = 'Check whether singleGeneDeletion runs smoothly';
0423 if runSingleGeneDeletion == 1
0424     try
0425         [grRatio,grRateKO,grRateWT,hasEffect,delRxns,fluxSolution] = singleGeneDeletion(model);
0426         TableChecks{cnt,2} = 'singleGeneDeletion finished without problems.';
0427     catch
0428         TableChecks{cnt,2} = 'There are problems with singleGeneDeletion.';
0429     end
0430 else
0431     TableChecks{cnt,2} = 'Not performed.';
0432 end
0433 cnt = cnt + 1;
0434 
0435 TableChecks{cnt,1} = 'Check for flux consistency';
0436 if 0
0437     param.epsilon=1e-4;
0438     param.modeFlag=0;
0439     %param.method='null_fastcc';
0440     %param.method='fastcc';
0441     printLevel = 1;
0442     [fluxConsistentMetBool,fluxConsistentRxnBool,fluxInConsistentMetBool,fluxInConsistentRxnBool,model] = findFluxConsistentSubset(model,param,printLevel)
0443     if isempty(find(fluxInConsistentRxnBool))
0444         TableChecks{cnt,2} = 'Model is flux consistent.';
0445     else
0446         TableChecks{cnt,2} = 'Model is NOT flux consistent';
0447     end
0448 else
0449     TableChecks{cnt,2} = 'Not performed.';
0450 end
0451 cnt = cnt + 1;
0452 
0453 
0454 save([resultsPath 'OrganChecks' filesep resultsFileName,'.mat'],'TableChecks', 'Table_csources', 'CSourcesTestedRxns', 'TestSolutionNameOpenSinks','TestSolutionNameClosedSinks');
0455 
0456 if saveDiary
0457     diary off;
0458 end
```

---

Generated on Thu 14-May-2020 13:05:49 by **m2html** © 2005
