# Supplementary material for: Personalized whole‐body models integrate metabolism, physiology, and the gut microbiome
Source: Mol Syst Biol. 2020 May 28;16(5):e8982. doi: 10.15252/msb.20198982 (PMC7285886; doi:10.15252/msb.20198982)
Supplement: Supplementary file 22 — Dataset EV1 [file MSB-16-e8982-s022.zip › PSCM_toolbox/PSCM_toolbox_doc/src/getOrgansFromHarvey.html]

Description of getOrgansFromHarvey


# getOrgansFromHarvey

## PURPOSE

**This function cuts the organs from the whole-body metabolic model. Note that the different**

## SYNOPSIS

**function [OrganCompendium,TableCSources] = getOrgansFromHarvey(modelWBM,runTestsOnly,OrganCompendium, printLevel)**

## DESCRIPTION

```
 This function cuts the organs from the whole-body metabolic model. Note that the different
 biofluid compartments are retained but all constraints on the exchange and transport reactions are overwritten
 Once the organs are extracted, the function runs the sanity check on each
 organ. This step can be also done independently by setting runTestsOnly
 to 1. In this case,  the OrganCompendium must be provided as input.
 The function also loads Recon 3* so that the test results can be compared
 with the organ test results.

 [OrganCompendium,TableCSources] = getOrgansFromHarvey(modelWBM, runTestsOnly, OrganCompendium)
 
 INPUT
 modelWBM                  model structure of whole-body metabolic model
 runTestsOnly              

 OUTPUT
 OrganCompendium           Structure containing the individal organs as
                           well as the basic tests that this organ passed
 TableCSources             Overview table of ATP yield per carbon source
                           under aerobic and anaerobic conditions for each organ in the model
                           structrure
 
 The organ compendium for each sex will be saved as
 OrganAtlas_Harvetta.mat and OrganAtlas_Harvey.mat along with the test
 results.

 Ines Thiele, 2017 - 2019
```

## CROSS-REFERENCE INFORMATION

This function calls:

- annotateModel This function annotates a model with VMH reaction and metabolite
- OrganLists This file contains lists of ograns as they are used in the whole-body
- performSanityChecksonRecon This function performs various quality control and quality assurance

This function is called by:

## SOURCE CODE

```
0001 function [OrganCompendium,TableCSources] = getOrgansFromHarvey(modelWBM,runTestsOnly,OrganCompendium, printLevel)
0002 % This function cuts the organs from the whole-body metabolic model. Note that the different
0003 % biofluid compartments are retained but all constraints on the exchange and transport reactions are overwritten
0004 % Once the organs are extracted, the function runs the sanity check on each
0005 % organ. This step can be also done independently by setting runTestsOnly
0006 % to 1. In this case,  the OrganCompendium must be provided as input.
0007 % The function also loads Recon 3* so that the test results can be compared
0008 % with the organ test results.
0009 %
0010 % [OrganCompendium,TableCSources] = getOrgansFromHarvey(modelWBM, runTestsOnly, OrganCompendium)
0011 %
0012 % INPUT
0013 % modelWBM                  model structure of whole-body metabolic model
0014 % runTestsOnly
0015 %
0016 % OUTPUT
0017 % OrganCompendium           Structure containing the individal organs as
0018 %                           well as the basic tests that this organ passed
0019 % TableCSources             Overview table of ATP yield per carbon source
0020 %                           under aerobic and anaerobic conditions for each organ in the model
0021 %                           structrure
0022 %
0023 % The organ compendium for each sex will be saved as
0024 % OrganAtlas_Harvetta.mat and OrganAtlas_Harvey.mat along with the test
0025 % results.
0026 %
0027 % Ines Thiele, 2017 - 2019
0028 
0029 global resultsPath
0030 resultsPath = which('MethodSection3.mlx');
0031 resultsPath = strrep(resultsPath,'MethodSection3.mlx',['Results' filesep]);
0032 
0033 if ~exist('printLevel','var')
0034     printLevel=0;
0035 end
0036 
0037 if ~exist('runTestsOnly','var')
0038     runTestsOnly=0;
0039 end
0040 
0041 % turn off warnings
0042 warning('off','all')
0043 
0044 sex = modelWBM.sex;
0045 OrganLists;
0046 if runTestsOnly ~= 1 
0047     if isfield(modelWBM,'A')
0048         % remove all slack variables
0049         modelWBM.S = modelWBM.A;
0050         SL = strmatch('slack_',modelWBM.mets);
0051         modelWBM.mets(SL) = [];
0052         modelWBM.b(SL) = [];
0053         modelWBM.S(SL,:) = [];
0054     end
0055     modelWBM.mets(strmatch('RBC_o2[bc]',modelWBM.mets,'exact'))={'o2[bc]'};
0056     modelWBM.mets(strmatch('RBC_co2[bc]',modelWBM.mets,'exact'))={'co2[bc]'};
0057     sex = modelWBM.sex;
0058     clear OrganCompendium
0059     for i = 1 : length(OrgansListShort)
0060         % metabolic reactions
0061         modelTmp = modelWBM;
0062         
0063         R1 = strmatch(OrgansListShort{i},modelTmp.rxns);
0064         if ~isempty(R1)
0065             % modelTmp.rxns(R1) = regexprep(modelTmp.rxns(R1),strcat(OrgansListShort{i},'_'),'');
0066             M1 = strmatch(OrgansListShort{i},modelTmp.mets(1:size(modelTmp.S,1)));
0067             %modelTmp.mets = regexprep(modelTmp.mets,strcat(OrgansListShort{i},'_'),'');
0068             model = struct();
0069             model.mets=cell(0,1);model.metNames=cell(0,1);model.metFormulas=cell(0,1);
0070             model.rxns=cell(0,1);model.rxnNames=cell(0,1);model.subSystems=cell(0,1);
0071             model.lb=zeros(0,1);model.ub=zeros(0,1);model.rev=zeros(0,1);
0072             model.c=zeros(0,1);model.b=zeros(0,1);
0073             model.S=sparse(0,0);
0074             % get organs
0075             % grap the corresponding Recon reactions
0076             for j =1 : length(R1)
0077                 %a= printRxnFormulaOri(modelTmp,modelTmp.rxns(R1(j)),0,1,0,1,0);
0078                 a = printRxnFormula(modelTmp,'rxnAbbrList',modelTmp.rxns(R1(j)),'printFlag',0,'lineChangeFlag',1,'metNameFlag',0,'fid',1,'directionFlag',0);
0079                 
0080                 model = addReaction(model,modelTmp.rxns{R1(j)},a{1});
0081                 % same constraints as in coupled model!
0082                 model.lb(end) = modelTmp.lb(R1(j));
0083                 model.ub(end) = modelTmp.ub(R1(j));
0084             end
0085             
0086             % remove dummy's from model
0087             Dummy = (find(~cellfun(@isempty,strfind(model.mets,'dummy'))));
0088             model.mets(Dummy) = [];
0089             model.metNames(Dummy) = [];
0090             model.metFormulas(Dummy) = [];
0091             model.S(Dummy,:)= [];
0092             model.c = modelTmp.c(R1);
0093             model.grRules = [modelTmp.grRules(R1)];
0094             [a,b] = ismember( modelTmp.mets(M1),model.mets);
0095             model.b = zeros(length(model.mets),1);
0096             model.rev = zeros(length(model.lb),1);
0097             model.rev(find(model.lb<0))=1;
0098             
0099             model.genes = cell(0,1);
0100             % keep all model compartments
0101             modelAllComp = model;
0102             % find all exchange metabolites
0103             for j = 1 : length(modelAllComp.mets)
0104                 if ~isempty(strfind(modelAllComp.mets{j},'[bd]'))||~isempty(strfind(modelAllComp.mets{j},'[luLI]'))...
0105                         ||~isempty(strfind(modelAllComp.mets{j},'[luSI]'))||~isempty(strfind(modelAllComp.mets{j},'[bc]'))...
0106                         ||~isempty(strfind(modelAllComp.mets{j},'[fe]'))||~isempty(strfind(modelAllComp.mets{j},'[u]'))...
0107                         ||~isempty(strfind(modelAllComp.mets{j},'[bp]'))||~isempty(strfind(modelAllComp.mets{j},'[a]'))...
0108                         ||~isempty(strfind(modelAllComp.mets{j},'[sw]'))||~isempty(strfind(modelAllComp.mets{j},'[csf]'))...
0109                         ||~isempty(strfind(modelAllComp.mets{j},'[lu]'))
0110                     modelAllComp = addExchangeRxn(modelAllComp,modelAllComp.mets(j),-1000,1000);
0111                 end
0112             end
0113             modelAllComp.rxns=regexprep(modelAllComp.rxns,strcat(OrgansListShort(i),'_'),'');
0114             modelAllComp.mets=regexprep(modelAllComp.mets,strcat(OrgansListShort(i),'_'),'');
0115             %a = printRxnFormulaOri(modelAllComp,modelAllComp.rxns,0,0,0,'',0);
0116             a = printRxnFormula(modelAllComp,'rxnAbbrList',modelAllComp.rxns,'printFlag',0,'lineChangeFlag',0,'metNameFlag',0,'fid',0,'directionFlag',0);
0117             modelAllComp.reactions =a;
0118             modelAllComp.genes = [];
0119             modelAllComp.rxnGeneMat = [];
0120             % rewrite GPRs
0121             modelAllCompgrRule = modelAllComp.grRules;
0122             for j = 1 : length(modelAllCompgrRule)
0123                 %modelAllComp = changeGeneAssociationOri(modelAllComp,modelAllComp.rxns{j},char(modelAllCompgrRule{j}));
0124                 modelAllComp = changeGeneAssociation(modelAllComp,modelAllComp.rxns{j},char(modelAllCompgrRule{j}),0);%do
0125             end
0126             % rename all EX_ reactions that have 2 entries, e.g., 'EX_2m3hbu(e)_[bc]'    '2m3hbu[e]  <=> 2m3hbu[bc] ' but keeps
0127             % 'EX_2m3hbu[bc]'    '2m3hbu[bc]  <=> ' as is
0128             % find all reactions starting with EX_ in abbr
0129             EXAll = strmatch('EX_',modelAllComp.rxns);
0130             % find all reactions that have only one non-zero entry in the S matrizx
0131             selExc = (find( full((sum(abs(modelAllComp.S)==1,1) ==1) & (sum(modelAllComp.S~=0) == 1))))';
0132             EX2Rename = setdiff(EXAll,selExc);
0133             modelAllComp.rxns(EX2Rename) = strcat('Tr_',modelAllComp.rxns(EX2Rename));
0134             modelAllComp.rxns(strmatch('biomass_reactionIEC01b_trtr',modelAllComp.rxns)) = {'biomass_reaction_trtr'};
0135             modelAllComp.rxns(strmatch('biomass_reactionIEC01b',modelAllComp.rxns)) ={ 'biomass_maintenance'};
0136             OrganCompendium.(OrgansListShort{i}).modelAllComp = modelAllComp;
0137         end
0138 
0139     end
0140     % annotate organ compendium with Recon 3D data
0141     annotateRxns = 1;
0142     annotateMets = 1;
0143     O = fieldnames(OrganCompendium);
0144     for i = 1 : length(O)
0145         if ~(strcmp(O{i},'sex')) && ~(strcmp(O{i},'Recon3DHarvey'))
0146             OrganCompendium.(O{i}).modelAllComp = annotateModel(OrganCompendium.(O{i}).modelAllComp, annotateRxns,annotateMets);
0147         end
0148     end
0149     if strcmp(sex,'female')
0150         OrganCompendium.sex = 'female';
0151         %save OrganAtlas_Harvetta OrganCompendium  modelWBM
0152         save([resultsPath 'OrganAtlas_Harvetta'],'OrganCompendium','modelWBM')
0153     else
0154         OrganCompendium.sex = 'male';
0155         save([resultsPath 'OrganAtlas_Harvey'],'OrganCompendium','modelWBM')
0156     end
0157 end
0158 %% run Metabolic function tests and quality assurance/quality control tests.
0159 
0160 organ = fieldnames(OrganCompendium);
0161 clear TestSolutionName TestSolution OR FBA_OR R
0162 for i =1 :length(organ)
0163     if ~strcmp('sex',organ{i}) &&  ~strcmp('Recon3DHarvey',organ{i}) 
0164         model = OrganCompendium.(organ{i}).modelAllComp;
0165         model.lb(find(model.lb<0))=-1000;
0166         model.ub(find(model.ub<0))=0;
0167         model.ub(find(model.ub>0))=1000;
0168         model.lb(find(model.lb>0))=0;
0169         % [X,TestSolutionName] = Test4HumanFctExtv4(model,'Harvey');
0170         resultsFileName = strcat(sex,organ{i});
0171         if strcmp('Brain',organ{i}) || strcmp('Scord',organ{i})
0172             extraCellCompIn = '[csf]';
0173             extraCellCompOut = '[csf]';
0174         elseif ~isempty(find(~cellfun(@isempty,strfind(model.rxns,'[bp]')))) && ~strcmp('Liver',organ{i})
0175             extraCellCompIn = '[bc]';
0176             % the out compartment does not matter as all out are anyway open
0177             extraCellCompOut = '[bp]';
0178         elseif ~isempty(find(~cellfun(@isempty,strfind(model.rxns,'[bd]')))) && ~strcmp('Liver',organ{i})
0179             extraCellCompIn = '[bc]';
0180             % the out compartment does not matter as all out are anyway open
0181             extraCellCompOut = '[bd]';
0182         else
0183             extraCellCompIn = '[bc]';
0184             % the out compartment does not matter as all out are anyway open
0185             extraCellCompOut = '[bc]';
0186         end
0187         %NOTE I have to test also Liver with bp input
0188         
0189         % revert the direction of sinks
0190         SI = strmatch('sink_',model.rxns);
0191         for k = 1 : length(SI)
0192             if ~isempty(find(model.S(:,SI(k))==1))% positive entry
0193                 % flip it
0194                 model.S(find(model.S(:,SI(k))==1),SI(k)) = -1;
0195                 model.lb(SI(k)) = min(model.lb(SI(k)),model.ub(SI(k)));
0196                 model.ub(SI(k)) = max(model.lb(SI(k)),model.ub(SI(k)));
0197             end
0198         end
0199         % revert the direction of demands
0200         SI = strmatch('DM_',model.rxns);
0201         for k = 1 : length(SI)
0202             if ~isempty(find(model.S(:,SI(k))==1)) && length(find(model.S(:,SI(k))==1))==1% positive entry
0203                 % flip it
0204                 model.S(find(model.S(:,SI(k))==1),SI(k)) = -1;
0205                 model.lb(SI(k)) = 0%min(model.lb(SI(k)),model.ub(SI(k)));
0206                 model.lb(SI(k)) = max(model.lb(SI(k)),model.ub(SI(k)));
0207             end
0208         end
0209         
0210         modelClosed = model;
0211         % prepare models for test - these changes are needed for the different
0212         % recon versions to match the rxn abbr definitions in this script
0213         modelClosed.rxns = regexprep(modelClosed.rxns,'\(','\[');
0214         modelClosed.rxns = regexprep(modelClosed.rxns,'\)','\]');
0215         modelClosed.mets = regexprep(modelClosed.mets,'\(','\[');
0216         modelClosed.mets = regexprep(modelClosed.mets,'\)','\]');
0217         modelClosed.rxns = regexprep(modelClosed.rxns,'ATPS4mi','ATPS4m');
0218         
0219         % replace older abbreviation of glucose exchange reaction with the one used
0220         % in this script
0221         if length(strmatch(strcat('EX_glc',extraCellCompIn),modelClosed.rxns))>0
0222             modelClosed.rxns{find(ismember(modelClosed.rxns,strcat('EX_glc',extraCellCompIn)))} = strcat('EX_glc_D',extraCellCompIn);
0223         end
0224         if length(strmatch(strcat('EX_glc',extraCellCompOut),modelClosed.rxns))>0
0225             modelClosed.rxns{find(ismember(modelClosed.rxns,strcat('EX_glc',extraCellCompOut)))} = strcat('EX_glc_D',extraCellCompOut);
0226         end
0227         
0228         % add reaction if it does not exist
0229         [modelClosed, rxnIDexists] = addReaction(modelClosed,'DM_atp_c_','reactionFormula','h2o[c] + atp[c] -> adp[c] + h[c] + pi[c] ','printLevel',0);
0230         if length(rxnIDexists)>0
0231             modelClosed.rxns{rxnIDexists} = 'DM_atp_c_'; % rename reaction in case that it exists already
0232         end
0233         
0234         modelClosed.lb(find(ismember(modelClosed.rxns, 'Tr_EX_o2[e]_[bc]')))=-1000;
0235         modelClosed.lb(find(ismember(modelClosed.rxns, 'Tr_EX_co2[e]_[bc]')))=-1000;
0236         modelClosed = changeRxnBounds(modelClosed, 'DCMPtm',-1000,'l');
0237         
0238         OrganCompendium.(organ{i}).modelAllComp = modelClosed;
0239         [OrganCompendium.(organ{i}).Sanity.TableChecks,...
0240             OrganCompendium.(organ{i}).Sanity.Table_csources,...
0241             OrganCompendium.(organ{i}).Sanity.CSourcesTestedRxns,...
0242             OrganCompendium.(organ{i}).Sanity.TestSolutionNameOpenSinks,...
0243             OrganCompendium.(organ{i}).Sanity.TestSolutionNameClosedSinks]...
0244             = performSanityChecksonRecon(modelClosed,resultsFileName,extraCellCompIn,extraCellCompOut);
0245     end
0246 end
0247 
0248 load('Recon3D_Harvey_Used_in_Script_120502.mat')
0249 %load('Y:\SemiAutomated_Organ_Models\_InesProteomeMapData\Recon2.1\Recon3_and_alike\2017_05_18_Recon3d_consistencyCheckHarvey.mat');
0250 OrganCompendium.Recon3DHarvey.model = modelConsistent;
0251 [OrganCompendium.Recon3DHarvey.Sanity.TableChecks, OrganCompendium.Recon3DHarvey.Sanity.Table_csources,OrganCompendium.Recon3DHarvey.Sanity.CSourcesTestedRxns,  OrganCompendium.Recon3DHarvey.Sanity.TestSolutionNameOpenSinks,OrganCompendium.Recon3DHarvey.Sanity.TestSolutionNameClosedSinks] = performSanityChecksonRecon(modelConsistent,'Recon3DHarvey');
0252 
0253 % get results from all Csources
0254 organ = fieldnames(OrganCompendium);
0255 TableCSources(:,1) = OrganCompendium.(organ{1}).Sanity.Table_csources(:,1);
0256 % theoretical
0257 TableCSources(:,2) = OrganCompendium.(organ{1}).Sanity.Table_csources(:,4);
0258 % Recon2.2
0259 TableCSources(:,3) = OrganCompendium.(organ{1}).Sanity.Table_csources(:,5);
0260 
0261 for i = 1 : length(organ)
0262     if ~strcmp('sex',organ{i})
0263         TableCSources(:,i+3) = OrganCompendium.(organ{i}).Sanity.Table_csources(:,2);
0264         TableCSources(1,i+3) = organ(i);
0265     end
0266 end
```

---

Generated on Thu 14-May-2020 13:05:49 by **m2html** © 2005
