# Supplementary material for: Personalized whole‐body models integrate metabolism, physiology, and the gut microbiome
Source: Mol Syst Biol. 2020 May 28;16(5):e8982. doi: 10.15252/msb.20198982 (PMC7285886; doi:10.15252/msb.20198982)
Supplement: Supplementary file 22 — Dataset EV1 [file MSB-16-e8982-s022.zip › PSCM_toolbox/PSCM_toolbox_doc/src/Test4HumanFctExtv5.html]

Description of Test4HumanFctExtv5


# Test4HumanFctExtv5

## PURPOSE

**% function [TestSolution,TestSolutionName] = Test4HumanFct(model,test)**

## SYNOPSIS

**function [TestSolution,TestSolutionName,TestedRxns,PercTestedRxns] = Test4HumanFctExtv4(model,test,optionSinks)**

## DESCRIPTION

```
% function [TestSolution,TestSolutionName] = Test4HumanFct(model,test)
 This functions test for the ~460 human functions -  I removed duplicates

 INPUT
 model             model structure (Recon1, with desired in silico
                   condition)
 test              possible statements: Recon1, IECori, IEC, all (default)
                   (choose IECori if you intend to test the IEC model OR a model that
                   contains lumen ('u') as compartment otw choose IEC);
                   all check for Recon1 and IEC
 option            if true = set sink reactions to 0 (default, leave unchanged).
                   Note that all lb's of exchanges and demands will be set to 0

 OUTPUT
 TestSolution      array containing the optimal value for the different
                   tests
 TestSolutionName  array containing the names  for the different tests

 Ines Thiele, 09/05/09
 MKA, 03/04/12 some of the reaction names have changed in newer versions of Recon1
 and Recon2. Comment setup til line 146 if using an old version.
 MKA, 24/05/12 finds correct EX_reactions and changes these to zero
 IT, 07/20/12 added tests for sIEC model
 IT 2017 more tests added
```

## CROSS-REFERENCE INFORMATION

This function calls:


This function is called by:

- performSanityChecksonRecon This function performs various quality control and quality assurance

## SOURCE CODE

```
0001 function [TestSolution,TestSolutionName,TestedRxns,PercTestedRxns] = Test4HumanFctExtv4(model,test,optionSinks)
0002 %% function [TestSolution,TestSolutionName] = Test4HumanFct(model,test)
0003 % This functions test for the ~460 human functions -  I removed duplicates
0004 %
0005 % INPUT
0006 % model             model structure (Recon1, with desired in silico
0007 %                   condition)
0008 % test              possible statements: Recon1, IECori, IEC, all (default)
0009 %                   (choose IECori if you intend to test the IEC model OR a model that
0010 %                   contains lumen ('u') as compartment otw choose IEC);
0011 %                   all check for Recon1 and IEC
0012 % option            if true = set sink reactions to 0 (default, leave unchanged).
0013 %                   Note that all lb's of exchanges and demands will be set to 0
0014 %
0015 % OUTPUT
0016 % TestSolution      array containing the optimal value for the different
0017 %                   tests
0018 % TestSolutionName  array containing the names  for the different tests
0019 %
0020 % Ines Thiele, 09/05/09
0021 % MKA, 03/04/12 some of the reaction names have changed in newer versions of Recon1
0022 % and Recon2. Comment setup til line 146 if using an old version.
0023 % MKA, 24/05/12 finds correct EX_reactions and changes these to zero
0024 % IT, 07/20/12 added tests for sIEC model
0025 % IT 2017 more tests added
0026 
0027 %%
0028 if nargin<2
0029     test = 'all';
0030 end
0031 if nargin<3
0032     optionSinks = 0; % do not close
0033 end
0034 
0035 global saveDiary
0036 
0037 if optionSinks
0038     % close sink reactions
0039     model.lb(strmatch('sink_',model.rxns))=0;
0040 end
0041 
0042 TestSolution = [];
0043 %%
0044 % S ='';
0045 %  S = warning('QUERY','VERBOSE');
0046 %% Setup
0047 
0048 % for organ atlas derived from Harvey only
0049 if strcmp(test,'Harvey') || strcmp(test,'all')
0050     model.rxns = regexprep(model.rxns,'\[bc\]','\(e\)');
0051     model.rxns = regexprep(model.rxns,'\(bc\)','\(e\)');
0052     model.rxns = regexprep(model.rxns,'\[a\]','\(e\)');
0053     model.rxns = regexprep(model.rxns,'\(a\)','\(e\)');
0054     model.rxns = regexprep(model.rxns,'\[csf\]','\(e\)');
0055     model.rxns = regexprep(model.rxns,'\(csf\)','\(e\)');
0056     model.rxns(strmatch('biomass_reactionIEC01b_trtr',model.rxns)) = {'biomass_maintenance'};
0057    model.rxns(strmatch('biomass_reactionIEC01b',model.rxns)) ={ 'biomass_reaction'};
0058 end
0059 
0060 if saveDiary
0061     %save each diary to PSCM_toolbox/Files/OrganChecks/
0062     aPath=which('MethodSection3.mlx');
0063     aPath=strrep(aPath,'MethodSection3.mlx',['Files' filesep 'OrganChecks' filesep]);
0064     diary([aPath filesep 'Test4Functions_diary.txt']);
0065 end
0066 TestedRxns =[];
0067 tol = 1e-6;
0068 % fixes the model met names in cases the compartments are not given with ()
0069 model.mets = regexprep(model.mets,'[','(');
0070 model.mets = regexprep(model.mets,']',')');
0071 model.mets = regexprep(model.mets,'_','-');
0072 model.mets = regexprep(model.mets,'-FSLASH-','/');
0073 
0074 model.rxns = regexprep(model.rxns,'\[','\(');
0075 model.rxns = regexprep(model.rxns,'\]','\)');
0076 
0077 % replace reaction names
0078 new = {'DM_atp_c_'
0079     'EX_gln_L(e)'
0080     'EX_glu_L(e)'
0081     'EX_lac_L(e)'
0082     'EX_pro_L(e)'
0083     'EX_cys_L(e)'
0084     'EX_lys_L(e)'
0085     'EX_arg_L(e)'
0086     'EX_his_L(e)'
0087     'EX_glc_D(e)'
0088     'CYOR_u10m'
0089     'NADH2_u10m'
0090     'EX_4hpro(e)'
0091     % due to innermitochondrial membrane representation in recon3
0092     'ASPGLUmi'
0093     'ATPS4mi'
0094     'CYOR_u10mi'
0095     'Htmi'
0096     'NADH2_u10mi'
0097     'CYOOm3i'
0098     'CYOOm2i'};
0099 
0100 original = {'DM_atp(c)'
0101     'EX_gln-L(e)'
0102     'EX_glu-L(e)'
0103     'EX_lac-L(e)'
0104     'EX_pro-L(e)'
0105     'EX_cys-L(e)'
0106     'EX_lys-L(e)'
0107     'EX_arg-L(e)'
0108     'EX_his-L(e)'
0109     'EX_glc(e)'
0110     'CYOR-u10m'
0111     'NADH2-u10m'
0112     'EX_4HPRO'
0113     'ASPGLUm'
0114     'ATPS4m'
0115     'CYOR-u10m'
0116     'Htm'
0117     'NADH2-u10m'
0118     'CYOOm3'
0119     'CYOOm2'};
0120 
0121 for i=1:length(new)
0122     A = find(ismember(model.rxns,new(i,1)));
0123     model.rxns(A,1)= original(i,1);
0124 end
0125 
0126 %replace metabolite names
0127 
0128 new_mets = {'Ser-Gly-Ala-X-Gly(r)'
0129     'Ser-Thr(g)'
0130     'Ser-Thr(l)'
0131     'ksii-core2(g)'
0132     'ksii-core4(g)'
0133     'ksii-core2(l)'
0134     'ksii-core4(l)'
0135     'cspg-a(l)'
0136     'cspg-b(l)'
0137     'cspg-c(l)'
0138     'cspg-d(l)'
0139     'cspg-e(l)'
0140     'cspg-a(g)'
0141     'cspg-b(g)'
0142     'cspg-c(g)'
0143     'cspg-d(g)'
0144     'cspg-e(g)'
0145     'galgluside-hs(g)'
0146     'gluside-hs(g)'
0147     'galgalgalthcrm-hs(g)'
0148     'acgagbside-hs(g)'
0149     'acnacngalgbside-hs(g)'
0150     'gd1b2-hs(g)'
0151     'gd1c-hs(g)'
0152     'gq1balpha-hs(g)'
0153     'dag-hs(c)'
0154     'pe-hs(c)'
0155     'tag-hs(c)'
0156     'cs-pre(g)'
0157     'crmp-hs(c)'
0158     'sphmyln-hs(c)'
0159     'pail-hs(c)'
0160     'pail45p-hs(c)'
0161     'pail4p-hs(c)'
0162     'dolichol-L(c)'
0163     'dolmanp-L(r)'
0164     'dolichol-U(c)'
0165     'dolmanp-U(r)'
0166     'dolichol-L(r)'
0167     'dolichol-U(r)'
0168     'gpi-prot-hs(r)'
0169     'g3m8mpdol-L(r)'
0170     'g3m8mpdol-U(r)'
0171     'gp1c-hs(g)'
0172     'dsTn-antigen(g)'
0173     'sTn-antigen(g)'
0174     'Tn-antigen(g)'
0175     };
0176 original_mets = {'Ser-Gly/Ala-X-Gly(r)'
0177     'Ser/Thr(g)'
0178     'Ser/Thr(l)'
0179     'ksii_core2(g)'
0180     'ksii_core4(g)'
0181     'ksii_core2(l)'
0182     'ksii_core4(l)'
0183     'cspg_a(l)'
0184     'cspg_b(l)'
0185     'cspg_c(l)'
0186     'cspg_d(l)'
0187     'cspg_e(l)'
0188     'cspg_a(g)'
0189     'cspg_b(g)'
0190     'cspg_c(g)'
0191     'cspg_d(g)'
0192     'cspg_e(g)'
0193     'galgluside_hs(g)'
0194     'gluside_hs(g)'
0195     'galgalgalthcrm_hs(g)'
0196     'acgagbside_hs(g)'
0197     'acnacngalgbside_hs(g)'
0198     'gd1b2_hs(g)'
0199     'gd1c_hs(g)'
0200     'gq1balpha_hs(g)'
0201     'dag_hs(c)'
0202     'pe_hs(c)'
0203     'tag_hs(c)'
0204     'cs_pre(g)'
0205     'crmp_hs(c)'
0206     'sphmyln_hs(c)'
0207     'pail_hs(c)'
0208     'pail45p_hs(c)'
0209     'pail4p_hs(c)'
0210     'dolichol_L(c)'
0211     'dolmanp_L(r)'
0212     'dolichol_U(c)'
0213     'dolmanp_U(r)'
0214     'dolichol_L(r)'
0215     'dolichol_U(r)'
0216     'gpi_prot_hs(r)'
0217     'g3m8mpdol_L(r)'
0218     'g3m8mpdol_U(r)'
0219     'gp1c_hs(g)'
0220     'dsTn_antigen(g)'
0221     'sTn_antigen(g)'
0222     'Tn_antigen(g)'
0223     };
0224 for i=1:length(new_mets)
0225     met = new_mets(i,1);
0226     A = find(ismember(model.mets,met));
0227     model.mets(A,1)= original_mets(i,1);
0228 end
0229 
0230 for i=1:length(new_mets)
0231     M = regexprep(new_mets(i,1),'(','[');
0232     M = regexprep(M,')',']');
0233     met = new_mets(i,1);
0234     A = find(ismember(M,met));
0235     model.mets(A,1)= original_mets(i,1);
0236 end
0237 % close sink reactions
0238 model.lb(strmatch('DM_',model.rxns))=0;
0239 %aerobic
0240 model.lb(ismember(model.rxns,'EX_o2(e)'))=-40;model.ub(ismember(model.rxns,'EX_o2(e)'))=0;
0241 
0242 model.c(find(model.c)) = 0;
0243 modelOri = model;
0244 k = 1;
0245 RPMI_composition={'EX_ala_L(e)','EX_arg-L(e)','EX_asn_L(e)','EX_asp_L(e)','EX_cys-L(e)','EX_gln-L(e)','EX_glu-L(e)','EX_gly(e)','EX_his-L(e)','EX_ile_L(e)','EX_leu_L(e)','EX_lys-L(e)','EX_met_L(e)','EX_phe_L(e)','EX_4HPRO','EX_pro-L(e)','EX_ser_L(e)','EX_thr_L(e)','EX_trp_L(e)','EX_tyr_L(e)','EX_val_L(e)','EX_ascb_L(e)','EX_btn(e)','EX_chol(e)','EX_pnto_R(e)','EX_fol(e)','EX_ncam(e)','EX_pydxn(e)','EX_ribflv(e)','EX_thm(e)','EX_cbl1(e)','EX_inost(e)','EX_ca2(e)','EX_fe3(e)','EX_k(e)','EX_hco3(e)','EX_na1(e)','EX_pi(e)','EX_glc(e)','EX_hxan(e)','EX_lnlc(e)','EX_lipoate(e)','EX_ptrc(e)','EX_pyr(e)','EX_thymd(e)','EX_etha(e)','EX_gthrd(e)'};
0246 
0247 if strcmp(test,'Recon1') || strcmp(test,'all') || strcmp(test,'Harvey')
0248     
0249     % %      %% "Human Recon 1 test mouse biomass"
0250     % %     model = modelOri;
0251     % %     model.c(find(model.c)) = 0;
0252     % %     model.c(ismember(model.rxns,'biomass_mm_1_no_glygln'))=1;
0253     % %       FBA = optimizeCbModel(model,'max');
0254     % %     TestSolution(k,1) = FBA.f);
0255     % %     TestSolutionName{k,1} = 'Human Recon 1 test mouse biomass';
0256     % %  if ~isnan(TestSolution(k,1)); TestedRxns = [TestedRxns; model.rxns(find(abs(FBA.x)>tol))]; end ;k = k +1;clear FBA
0257     %% "Human Recon 1 human  biomass"
0258     model = modelOri;
0259     model.c(find(model.c)) = 0;
0260     model.c(ismember(model.rxns,'biomass_reaction'))=1;
0261     if find(model.c)>0
0262         FBA = optimizeCbModel(model,'max');
0263         TestSolution(k,1) = FBA.f;
0264     else
0265         TestSolution(k,1) = NaN;
0266     end
0267     TestSolutionName{k,1} = 'Test human biomass reaction';
0268     if ~isnan(TestSolution(k,1)); TestedRxns = [TestedRxns; model.rxns(find(abs(FBA.x)>tol))]; end ;k = k +1;clear FBA
0269     %% "Human Recon 1 human  biomass"
0270     model = modelOri;
0271     model.c(find(model.c)) = 0;
0272     model.c(ismember(model.rxns,'biomass_maintenance_noTrTr'))=1;
0273     if find(model.c)>0
0274         FBA = optimizeCbModel(model,'max');
0275         TestSolution(k,1) = FBA.f;
0276     else
0277         TestSolution(k,1) = NaN;
0278     end
0279     TestSolutionName{k,1} = 'Test human biomass reaction (noTrTr)';
0280     if ~isnan(TestSolution(k,1)); TestedRxns = [TestedRxns; model.rxns(find(abs(FBA.x)>tol))]; end ;k = k +1;clear FBA
0281     %% "Human Recon 1 human  biomass"
0282     model = modelOri;
0283     model.c(find(model.c)) = 0;
0284     model.c(ismember(model.rxns,'biomass_maintenance'))=1;
0285     if find(model.c)>0
0286         FBA = optimizeCbModel(model,'max');
0287         TestSolution(k,1) = FBA.f;
0288     else
0289         TestSolution(k,1) = NaN;
0290     end
0291     TestSolutionName{k,1} = 'Test human biomass reaction (maintenance)';
0292     if ~isnan(TestSolution(k,1)); TestedRxns = [TestedRxns; model.rxns(find(abs(FBA.x)>tol))]; end ;k = k +1;clear FBA
0293     %% do not apply base medium for Harvey
0294     if strcmp(test,'Harvey') == 0
0295         model = modelOri;
0296         mediumCompounds = {'EX_co2(e)', 'EX_h(e)', 'EX_h2o(e)', 'EX_hco3(e)', 'EX_nh4(e)', 'EX_o2(e)', 'EX_pi(e)', 'EX_so4(e)'};
0297         ions={'EX_ca2(e)', 'EX_cl(e)', 'EX_co(e)', 'EX_fe2(e)', 'EX_fe3(e)', 'EX_k(e)', 'EX_na1(e)', 'EX_i(e)', 'EX_sel(e)'};
0298         I = strmatch('EX_', modelOri.rxns);
0299         
0300         for i=1:length(I);
0301             Ex= I(i);
0302             modelOri.lb(Ex,1) = 0;
0303             if modelOri.ub(Ex,1) < 0;
0304                 modelOri.ub(Ex,1)=1;
0305             end
0306             %  modelOri.ub(Ex,1) = 1;% uncomment to run for tcell models
0307         end
0308         modelOri.lb(find(ismember(modelOri.rxns,mediumCompounds)))=-100;
0309         modelOri.lb(find(ismember(modelOri.rxns,ions)))=-1;
0310     end
0311     %% ATP max aerobic, glc, v0.05
0312     model = modelOri;
0313     model.c(find(model.c)) = 0;
0314     model.lb(ismember(model.rxns,'EX_glc(e)'))=-1;model.ub(ismember(model.rxns,'EX_glc(e)'))=-1;
0315     model.lb(ismember(model.rxns,'EX_o2(e)'))=-40;model.ub(ismember(model.rxns,'EX_o2(e)'))=-1;
0316     model.c(ismember(model.rxns,'DM_atp(c)'))=1;
0317     if find(model.c)>0
0318         FBA = optimizeCbModel(model,'max');
0319         TestSolution(k,1) = FBA.f;
0320     else
0321         TestSolution(k,1) = NaN;
0322     end
0323     TestSolutionName{k,1} = 'ATP max, aerobic, glc';
0324     if ~isnan(TestSolution(k,1)); TestedRxns = [TestedRxns; model.rxns(find(abs(FBA.x)>tol))]; end ;k = k +1;clear FBA
0325     %% ATP max, anaerobic glc, v0.05
0326     model = modelOri;
0327     model.c(find(model.c)) = 0;
0328     model.lb(ismember(model.rxns,'EX_glc(e)'))=-1;model.ub(ismember(model.rxns,'EX_glc(e)'))=-1;
0329     model.lb(ismember(model.rxns,'EX_o2(e)'))=0;model.ub(ismember(model.rxns,'EX_o2(e)'))=0;
0330     model.c(ismember(model.rxns,'DM_atp(c)'))=1;
0331     if find(model.c)>0
0332         FBA = optimizeCbModel(model,'max');
0333         TestSolution(k,1) = FBA.f;
0334     else
0335         TestSolution(k,1) = NaN;
0336     end
0337     TestSolutionName{k,1} = 'ATP max, anaerobic, glc';
0338     if ~isnan(TestSolution(k,1)); TestedRxns = [TestedRxns; model.rxns(find(abs(FBA.x)>tol))]; end ;k = k +1;clear FBA
0339     %% ATP max, aerobic, citrate
0340     model = modelOri;
0341     model.c(find(model.c)) = 0;
0342     model.lb(ismember(model.rxns,'EX_cit(e)'))=-1;model.ub(ismember(model.rxns,'EX_cit(e)'))=-1;
0343     model.lb(ismember(model.rxns,'EX_o2(e)'))=-40;model.ub(ismember(model.rxns,'EX_o2(e)'))=-1;
0344     model.c(ismember(model.rxns,'DM_atp(c)'))=1;
0345     if find(model.c)>0
0346         FBA = optimizeCbModel(model,'max');
0347         TestSolution(k,1) = FBA.f;
0348     else
0349         TestSolution(k,1) = NaN;
0350     end
0351     TestSolutionName{k,1} = 'ATP max, aerobic, citrate';
0352     if ~isnan(TestSolution(k,1)); TestedRxns = [TestedRxns; model.rxns(find(abs(FBA.x)>tol))]; end ;k = k +1;clear FBA
0353     %% ATP max, aerobic, EtOH substrate v0.05
0354     model = modelOri;
0355     model.c(find(model.c)) = 0;
0356     model.lb(ismember(model.rxns,'EX_etoh(e)'))=-1;model.ub(ismember(model.rxns,'EX_etoh(e)'))=-1;
0357     model.lb(ismember(model.rxns,'EX_o2(e)'))=-40;model.ub(ismember(model.rxns,'EX_o2(e)'))=-1;
0358     model.c(ismember(model.rxns,'DM_atp(c)'))=1;
0359     if find(model.c)>0
0360         FBA = optimizeCbModel(model,'max');
0361         TestSolution(k,1) = FBA.f;
0362     else
0363         TestSolution(k,1) = NaN;
0364     end
0365     TestSolutionName{k,1} = 'ATP max, aerobic, etoh';
0366     if ~isnan(TestSolution(k,1)); TestedRxns = [TestedRxns; model.rxns(find(abs(FBA.x)>tol))]; end ;k = k +1;clear FBA
0367     %% ATP max, aerobic, glutamate v0.05
0368     model = modelOri;
0369     model.c(find(model.c)) = 0;
0370     model.lb(ismember(model.rxns,'EX_glu-L(e)'))=-1;model.ub(ismember(model.rxns,'EX_glu-L(e)'))=-1;
0371     model.lb(ismember(model.rxns,'EX_o2(e)'))=-40;model.ub(ismember(model.rxns,'EX_o2(e)'))=-1;
0372     model.c(ismember(model.rxns,'DM_atp(c)'))=1;
0373     if find(model.c)>0
0374         FBA = optimizeCbModel(model,'max');
0375         TestSolution(k,1) = FBA.f;
0376     else
0377         TestSolution(k,1) = NaN;
0378     end
0379     TestSolutionName{k,1} = 'ATP max, aerobic, glu-L';
0380     if ~isnan(TestSolution(k,1)); TestedRxns = [TestedRxns; model.rxns(find(abs(FBA.x)>tol))]; end ;k = k +1;clear FBA
0381     %% ATP max, aerobic, glutamine substrate
0382     model = modelOri;
0383     model.c(find(model.c)) = 0;
0384     model.lb(ismember(model.rxns,'EX_gln-L(e)'))=-1;model.ub(ismember(model.rxns,'EX_gln-L(e)'))=-1;
0385     model.lb(ismember(model.rxns,'EX_o2(e)'))=-40;model.ub(ismember(model.rxns,'EX_o2(e)'))=-1;
0386     model.c(ismember(model.rxns,'DM_atp(c)'))=1;
0387     if find(model.c)>0
0388         FBA = optimizeCbModel(model,'max');
0389         TestSolution(k,1) = FBA.f;
0390     else
0391         TestSolution(k,1) = NaN;
0392     end
0393     TestSolutionName{k,1} = 'ATP max, aerobic, gln-L';
0394     if ~isnan(TestSolution(k,1)); TestedRxns = [TestedRxns; model.rxns(find(abs(FBA.x)>tol))]; end ;k = k +1;clear FBA
0395     %% ATP max, aerobic, glycine substrate v0.05
0396     model = modelOri;
0397     model.c(find(model.c)) = 0;
0398     model.lb(ismember(model.rxns,'EX_gly(e)'))=-1;model.ub(ismember(model.rxns,'EX_gly(e)'))=-1;
0399     model.lb(ismember(model.rxns,'EX_o2(e)'))=-40;model.ub(ismember(model.rxns,'EX_o2(e)'))=-1;
0400     model.c(ismember(model.rxns,'DM_atp(c)'))=1;
0401     if find(model.c)>0
0402         FBA = optimizeCbModel(model,'max');
0403         TestSolution(k,1) = FBA.f;
0404     else
0405         TestSolution(k,1) = NaN;
0406     end
0407     TestSolutionName{k,1} = 'ATP max, aerobic, gly';
0408     if ~isnan(TestSolution(k,1)); TestedRxns = [TestedRxns; model.rxns(find(abs(FBA.x)>tol))]; end ;k = k +1;clear FBA
0409     %% ATP max, aerobic, lactate substrate v0.05
0410     model = modelOri;
0411     model.c(find(model.c)) = 0;
0412     model.lb(ismember(model.rxns,'EX_lac-L(e)'))=-1;model.ub(ismember(model.rxns,'EX_lac-L(e)'))=-1;
0413     model.lb(ismember(model.rxns,'EX_o2(e)'))=-40;model.ub(ismember(model.rxns,'EX_o2(e)'))=-1;
0414     model.c(ismember(model.rxns,'DM_atp(c)'))=1;
0415     if find(model.c)>0
0416         FBA = optimizeCbModel(model,'max');
0417         TestSolution(k,1) = FBA.f;
0418     else
0419         TestSolution(k,1) = NaN;
0420     end
0421     TestSolutionName{k,1} = 'ATP max, aerobic, lac-L';
0422     if ~isnan(TestSolution(k,1)); TestedRxns = [TestedRxns; model.rxns(find(abs(FBA.x)>tol))]; end ;k = k +1;clear FBA
0423     %% ATP max, aerobic, proline substrate v0.05
0424     model = modelOri;
0425     model.c(find(model.c)) = 0;
0426     model.lb(ismember(model.rxns,'EX_pro-L(e)'))=-1;model.ub(ismember(model.rxns,'EX_pro-L(e)'))=-1;
0427     model.lb(ismember(model.rxns,'EX_o2(e)'))=-40;model.ub(ismember(model.rxns,'EX_o2(e)'))=-1;
0428     model.c(ismember(model.rxns,'DM_atp(c)'))=1;
0429     if find(model.c)>0
0430         FBA = optimizeCbModel(model,'max');
0431         TestSolution(k,1) = FBA.f;
0432     else
0433         TestSolution(k,1) = NaN;
0434     end
0435     TestSolutionName{k,1} = 'ATP max, aerobic, pro-L';
0436     if ~isnan(TestSolution(k,1)); TestedRxns = [TestedRxns; model.rxns(find(abs(FBA.x)>tol))]; end ;k = k +1;clear FBA
0437     %% ATP production via electron transport chain
0438     model = modelOri;
0439     model.c(find(model.c)) = 0;
0440     model.lb(ismember(model.rxns,'EX_glc(e)'))=-1;model.ub(ismember(model.rxns,'EX_glc(e)'))=0;
0441     %model.lb(ismember(model.rxns,'CYOOm3'))=1; % there is an alternative
0442     %reaction
0443     if ~isempty(find(ismember(model.rxns,'CYOR-u10m'))) && ~isempty(find(ismember(model.rxns,'NADH2-u10m')))
0444         if  model.ub(ismember(model.rxns,'CYOR-u10m'))>=1 && model.ub(ismember(model.rxns,'NADH2-u10m'))>=1
0445             model.lb(ismember(model.rxns,'CYOR-u10m'))=1;
0446             model.lb(ismember(model.rxns,'NADH2-u10m'))=1;
0447             model.c(ismember(model.rxns,'DM_atp(c)'))=1;
0448             if find(model.c)>0
0449                 FBA = optimizeCbModel(model,'max');
0450                 TestSolution(k,1) = FBA.f;
0451             else
0452                 TestSolution(k,1) = NaN;
0453             end
0454         else
0455             TestSolution(k,1) = NaN;
0456         end
0457     else
0458         TestSolution(k,1) = NaN;
0459     end
0460     TestSolutionName{k,1} = 'ATP production via electron transport chain';
0461     if ~isnan(TestSolution(k,1)); TestedRxns = [TestedRxns; model.rxns(find(abs(FBA.x)>tol))]; end ;k = k +1;clear FBA
0462     %% add RMPI medium to model
0463     if 0
0464         if strcmp(test,'Harvey') == 0
0465             RPMI_composition={'EX_ala_L(e)','EX_arg_L(e)','EX_asn_L(e)','EX_asp_L(e)','EX_cys_L(e)','EX_gln-L(e)','EX_glu-L(e)','EX_gly(e)','EX_his_L(e)','EX_ile_L(e)','EX_leu_L(e)','EX_lys_L(e)','EX_met_L(e)','EX_phe_L(e)','EX_4HPRO','EX_pro-L(e)','EX_ser_L(e)','EX_thr_L(e)','EX_trp_L(e)','EX_tyr_L(e)','EX_val_L(e)','EX_ascb_L(e)','EX_btn(e)','EX_chol(e)','EX_pnto_R(e)','EX_fol(e)','EX_ncam(e)','EX_pydxn(e)','EX_ribflv(e)','EX_thm(e)','EX_cbl1(e)','EX_inost(e)','EX_ca2(e)','EX_fe3(e)','EX_k(e)','EX_hco3(e)','EX_na1(e)','EX_pi(e)','EX_glc(e)','EX_hxan(e)','EX_lnlc(e)','EX_lipoate(e)','EX_ptrc(e)','EX_pyr(e)','EX_thymd(e)','EX_etha(e)','EX_gthrd(e)'};
0466             for i = 1 : length(RPMI_composition)
0467                 modelOri = changeRxnBounds(modelOri,RPMI_composition{i},-1,'l');
0468             end
0469         end
0470     end
0471     
0472     %% gthrd reduces h2o2
0473     model = modelOri;
0474     model.c(find(model.c)) = 0;
0475     model.lb(ismember(model.rxns,'EX_gthrd(e)'))=-1;
0476     model.c(ismember(model.rxns,'GTHP'))=1;
0477     if find(model.c)>0
0478         FBA = optimizeCbModel(model,'max');
0479         TestSolution(k,1) = FBA.f;
0480     else
0481         TestSolution(k,1) = NaN;
0482     end
0483     TestSolutionName{k,1} = 'gthrd reduces h2o2, GTHP (c) ';
0484     if ~isnan(TestSolution(k,1)); TestedRxns = [TestedRxns; model.rxns(find(abs(FBA.x)>tol))]; end ;k = k +1;clear FBA
0485     
0486     model = modelOri;
0487     model.lb(ismember(model.rxns,'EX_gthrd(e)'))=-1;model.ub(ismember(model.rxns,'gthox(e)'))=1;
0488     model.c(find(model.c)) = 0;
0489     model.c(ismember(model.rxns,'GTHPe'))=1;
0490     if find(model.c)>0
0491         FBA = optimizeCbModel(model,'max');
0492         TestSolution(k,1) = FBA.f;
0493     else
0494         TestSolution(k,1) = NaN;
0495     end
0496     TestSolutionName{k,1} = 'gthrd reduces h2o2, GTHP (e) ';
0497     if ~isnan(TestSolution(k,1)); TestedRxns = [TestedRxns; model.rxns(find(abs(FBA.x)>tol))]; end ;k = k +1;clear FBA
0498     
0499     model = modelOri;
0500     model.c(find(model.c)) = 0;
0501     model.lb(ismember(model.rxns,'EX_gthrd(e)'))=-1;
0502     model.c(ismember(model.rxns,'GTHPm'))=1;
0503     if find(model.c)>0
0504         FBA = optimizeCbModel(model,'max');
0505         TestSolution(k,1) = FBA.f;
0506     else
0507         TestSolution(k,1) = NaN;
0508     end
0509     TestSolutionName{k,1} = 'gthrd reduces h2o2, GTHP (m) ';
0510     if ~isnan(TestSolution(k,1)); TestedRxns = [TestedRxns; model.rxns(find(abs(FBA.x)>tol))]; end ;k = k +1;clear FBA
0511     %% gly -> co2 and nh4 (via glycine cleavage system)
0512     model = modelOri;
0513     model.c(find(model.c)) = 0;
0514     [model] = addSinkReactions(model,{'gly(c)','co2(c)','nh4(c)'},[-1 -1; 0.1 100; 0.1 100]);
0515     model.lb(ismember(model.rxns,'EX_nh4(e)'))=0;model.ub(ismember(model.rxns,'EX_nh4(e)'))=1000;
0516     model.c(ismember(model.rxns,'sink_nh4(c)'))=1;
0517     if find(model.c)>0
0518         FBA = optimizeCbModel(model,'max');
0519         TestSolution(k,1) = FBA.f;
0520     else
0521         TestSolution(k,1) = NaN;
0522     end
0523     TestSolutionName{k,1} = 'gly -> co2 + nh4';
0524     if ~isnan(TestSolution(k,1)); TestedRxns = [TestedRxns; model.rxns(find(abs(FBA.x)>tol))]; end ;k = k +1;clear FBA
0525     
0526     %% 12ppd-S -> mthgxl
0527     model = modelOri;
0528     model.c(find(model.c)) = 0;
0529     [model] = addSinkReactions(model,{'12ppd-S(c)','mthgxl(c)'},[-1 -1; 0 100]);
0530     model.c(ismember(model.rxns,'sink_mthgxl(c)'))=1;
0531     if find(model.c)>0
0532         FBA = optimizeCbModel(model,'max');
0533         TestSolution(k,1) = FBA.f;
0534     else
0535         TestSolution(k,1) = NaN;
0536     end
0537     TestSolutionName{k,1} = '12ppd-S(c) -> mthgxl(c)';
0538     if ~isnan(TestSolution(k,1)); TestedRxns = [TestedRxns; model.rxns(find(abs(FBA.x)>tol))]; end ;k = k +1;clear FBA
0539     %% 12ppd-S -> pyr
0540     model = modelOri;
0541     model.c(find(model.c)) = 0;
0542     [model] = addSinkReactions(model,{'12ppd-S(c)','pyr(c)'},[-1 -1; 0 100]);
0543     model.c(ismember(model.rxns,'sink_pyr(c)'))=1;
0544     if find(model.c)>0
0545         FBA = optimizeCbModel(model,'max');
0546         TestSolution(k,1) = FBA.f;
0547     else
0548         TestSolution(k,1) = NaN;
0549     end
0550     TestSolutionName{k,1} = '12ppd-S(c) -> pyr(c)';
0551     if ~isnan(TestSolution(k,1)); TestedRxns = [TestedRxns; model.rxns(find(abs(FBA.x)>tol))]; end ;k = k +1;clear FBA
0552     %% 3pg -> gly
0553     model = modelOri;
0554     model.c(find(model.c)) = 0;
0555     [model] = addSinkReactions(model,{'3pg(c)','gly(c)'},[-1 -1; 0 100]);
0556     model.c(ismember(model.rxns,'sink_gly(c)'))=1;
0557     if find(model.c)>0
0558         FBA = optimizeCbModel(model,'max');
0559         TestSolution(k,1) = FBA.f;
0560     else
0561         TestSolution(k,1) = NaN;
0562     end
0563     TestSolutionName{k,1} = '3pg(c) -> gly(c)';
0564     if ~isnan(TestSolution(k,1)); TestedRxns = [TestedRxns; model.rxns(find(abs(FBA.x)>tol))]; end ;k = k +1;clear FBA
0565     %% 3pg -> ser-L
0566     model = modelOri;
0567     model.c(find(model.c)) = 0;
0568     [model] = addSinkReactions(model,{'3pg(c)','ser-L(c)'},[-1 -1; 0 100]);
0569     model.c(ismember(model.rxns,'sink_ser-L(c)'))=1;
0570     if find(model.c)>0
0571         FBA = optimizeCbModel(model,'max');
0572         TestSolution(k,1) = FBA.f;
0573     else
0574         TestSolution(k,1) = NaN;
0575     end
0576     TestSolutionName{k,1} = '3pg(c) -> ser-L(c)';
0577     if ~isnan(TestSolution(k,1)); TestedRxns = [TestedRxns; model.rxns(find(abs(FBA.x)>tol))]; end ;k = k +1;clear FBA
0578     %% 4abut -> succ[m]
0579     model = modelOri;
0580     model.c(find(model.c)) = 0;
0581     [model] = addSinkReactions(model,{'4abut(c)','succ(m)'},[-1 -1; 0 100]);
0582     model.c(ismember(model.rxns,'sink_succ(m)'))=1;
0583     if find(model.c)>0
0584         FBA = optimizeCbModel(model,'max');
0585         TestSolution(k,1) = FBA.f;
0586     else
0587         TestSolution(k,1) = NaN;
0588     end
0589     TestSolutionName{k,1} = '4abut(c) -> succ(m)';
0590     if ~isnan(TestSolution(k,1)); TestedRxns = [TestedRxns; model.rxns(find(abs(FBA.x)>tol))]; end ;k = k +1;clear FBA
0591     %% 4hpro-LT(m) -> glx(m)
0592     model = modelOri;
0593     model.c(find(model.c)) = 0;
0594     [model] = addSinkReactions(model,{'4hpro-LT(m)','glx(m)'},[-1 -1; 0 100]);
0595     model.c(ismember(model.rxns,'sink_glx(m)'))=1;
0596     if find(model.c)>0
0597         FBA = optimizeCbModel(model,'max');
0598         TestSolution(k,1) = FBA.f;
0599     else
0600         TestSolution(k,1) = NaN;
0601     end
0602     TestSolutionName{k,1} = '4hpro-LT(m) -> glx(m)';
0603     if ~isnan(TestSolution(k,1)); TestedRxns = [TestedRxns; model.rxns(find(abs(FBA.x)>tol))]; end ;k = k +1;clear FBA
0604     %% 5aop -> pheme
0605     model = modelOri;
0606     model.c(find(model.c)) = 0;
0607     [model] = addSinkReactions(model,{'5aop(c)','pheme(c)'},[-1 -1; 0 100]);
0608     model.c(ismember(model.rxns,'sink_pheme(c)'))=1;
0609     if find(model.c)>0
0610         FBA = optimizeCbModel(model,'max');
0611         TestSolution(k,1) = FBA.f;
0612     else
0613         TestSolution(k,1) = NaN;
0614     end
0615     TestSolutionName{k,1} = '5aop(c) -> pheme(c)';
0616     if ~isnan(TestSolution(k,1)); TestedRxns = [TestedRxns; model.rxns(find(abs(FBA.x)>tol))]; end ;k = k +1;clear FBA
0617     %% aact -> mthgxl
0618     model = modelOri;
0619     model.c(find(model.c)) = 0;
0620     [model] = addSinkReactions(model,{'aact(c)','mthgxl(c)'},[-1 -1; 0 100]);
0621     model.c(ismember(model.rxns,'sink_mthgxl(c)'))=1;
0622     if find(model.c)>0
0623         FBA = optimizeCbModel(model,'max');
0624         TestSolution(k,1) = FBA.f;
0625     else
0626         TestSolution(k,1) = NaN;
0627     end
0628     TestSolutionName{k,1} = 'aact(c) -> mthgxl(c)';
0629     if ~isnan(TestSolution(k,1)); TestedRxns = [TestedRxns; model.rxns(find(abs(FBA.x)>tol))]; end ;k = k +1;clear FBA
0630     %% acac[m] -> acetone[m]
0631     model = modelOri;
0632     model.c(find(model.c)) = 0;
0633     [model] = addSinkReactions(model,{'acac(m)','acetone(m)'},[-1 -1; 0 100]);
0634     model.c(ismember(model.rxns,'sink_acetone(m)'))=1;
0635     if find(model.c)>0
0636         FBA = optimizeCbModel(model,'max');
0637         TestSolution(k,1) = FBA.f;
0638     else
0639         TestSolution(k,1) = NaN;
0640     end
0641     TestSolutionName{k,1} = 'acac(m) -> acetone(m)';
0642     if ~isnan(TestSolution(k,1)); TestedRxns = [TestedRxns; model.rxns(find(abs(FBA.x)>tol))]; end ;k = k +1;clear FBA
0643     %% acac[m] -> bhb[m]
0644     model = modelOri;
0645     model.c(find(model.c)) = 0;
0646     [model] = addSinkReactions(model,{'acac(m)','bhb(m)'},[-1 -1; 0 100]);
0647     model.c(ismember(model.rxns,'sink_bhb(m)'))=1;
0648     if find(model.c)>0
0649         FBA = optimizeCbModel(model,'max');
0650         TestSolution(k,1) = FBA.f;
0651     else
0652         TestSolution(k,1) = NaN;
0653     end
0654     TestSolutionName{k,1} = 'acac(m) -> bhb(m)';
0655     if ~isnan(TestSolution(k,1)); TestedRxns = [TestedRxns; model.rxns(find(abs(FBA.x)>tol))]; end ;k = k +1;clear FBA
0656     %% acald -> ac
0657     model = modelOri;
0658     model.c(find(model.c)) = 0;
0659     [model] = addSinkReactions(model,{'acald(c)','ac(c)'},[-1 -1; 0 100]);
0660     model.c(ismember(model.rxns,'sink_ac(c)'))=1;
0661     if find(model.c)>0
0662         FBA = optimizeCbModel(model,'max');
0663         TestSolution(k,1) = FBA.f;
0664     else
0665         TestSolution(k,1) = NaN;
0666     end
0667     TestSolutionName{k,1} = 'acald(c) -> ac(c)';
0668     if ~isnan(TestSolution(k,1)); TestedRxns = [TestedRxns; model.rxns(find(abs(FBA.x)>tol))]; end ;k = k +1;clear FBA
0669     %% accoa(c) -> pmtcoa(c) -> malcoa(m)
0670     model = modelOri;
0671     model.c(find(model.c)) = 0;
0672     [model] = addSinkReactions(model,{'accoa(c)','pmtcoa(c)'},[-1 -1; 0 100]);
0673     model.c(ismember(model.rxns,'sink_pmtcoa(c)'))=1;
0674     if find(model.c)>0
0675         FBA = optimizeCbModel(model,'max');
0676         TestSolution(k,1) = FBA.f;
0677     else
0678         TestSolution(k,1) = NaN;
0679     end
0680     TestSolutionName{k,1} = 'accoa(c) -> pmtcoa(c)';
0681     if ~isnan(TestSolution(k,1)); TestedRxns = [TestedRxns; model.rxns(find(abs(FBA.x)>tol))]; end ;k = k +1;clear FBA
0682     %% accoa(c) -> pmtcoa(c) -> malcoa(m)
0683     model = modelOri;
0684     model.c(find(model.c)) = 0;
0685     [model] = addSinkReactions(model,{'pmtcoa(c)','malcoa(m)'},[-1 -1; 0 100]);
0686     model.c(ismember(model.rxns,'sink_malcoa(m)'))=1;
0687     if find(model.c)>0
0688         FBA = optimizeCbModel(model,'max');
0689         TestSolution(k,1) = FBA.f;
0690     else
0691         TestSolution(k,1) = NaN;
0692     end
0693     TestSolutionName{k,1} = 'pmtcoa(c) -> malcoa(m)';
0694     if ~isnan(TestSolution(k,1)); TestedRxns = [TestedRxns; model.rxns(find(abs(FBA.x)>tol))]; end ;k = k +1;clear FBA
0695     %% acetone -> mthgxl
0696     model = modelOri;
0697     model.c(find(model.c)) = 0;
0698     [model] = addSinkReactions(model,{'acetone(c)','mthgxl(c)'},[-1 -1; 0 100]);
0699     model.c(ismember(model.rxns,'sink_mthgxl(c)'))=1;
0700     if find(model.c)>0
0701         FBA = optimizeCbModel(model,'max');
0702         TestSolution(k,1) = FBA.f;
0703     else
0704         TestSolution(k,1) = NaN;
0705     end
0706     TestSolutionName{k,1} = 'acetone(c) -> mthgxl(c)';
0707     if ~isnan(TestSolution(k,1)); TestedRxns = [TestedRxns; model.rxns(find(abs(FBA.x)>tol))]; end ;k = k +1;clear FBA
0708     %% acgal -> udpacgal
0709     model = modelOri;
0710     model.c(find(model.c)) = 0;
0711     [model] = addSinkReactions(model,{'acgal(c)','udpacgal(c)'},[-1 -1; 0 100]);
0712     model.c(ismember(model.rxns,'sink_udpacgal(c)'))=1;
0713     if find(model.c)>0
0714         FBA = optimizeCbModel(model,'max');
0715         TestSolution(k,1) = FBA.f;
0716     else
0717         TestSolution(k,1) = NaN;
0718     end
0719     TestSolutionName{k,1} = 'acgal(c) -> udpacgal(c)';
0720     if ~isnan(TestSolution(k,1)); TestedRxns = [TestedRxns; model.rxns(find(abs(FBA.x)>tol))]; end ;k = k +1;clear FBA
0721     %% acgam -> cmpacna
0722     model = modelOri;
0723     model.c(find(model.c)) = 0;
0724     [model] = addSinkReactions(model,{'acgam(c)','cmpacna(c)'},[-1 -1; 0 100]);
0725     model.c(ismember(model.rxns,'sink_cmpacna(c)'))=1;
0726     if find(model.c)>0
0727         FBA = optimizeCbModel(model,'max');
0728         TestSolution(k,1) = FBA.f;
0729     else
0730         TestSolution(k,1) = NaN;
0731     end
0732     TestSolutionName{k,1} = 'acgam(c) -> cmpacna(c)';
0733     if ~isnan(TestSolution(k,1)); TestedRxns = [TestedRxns; model.rxns(find(abs(FBA.x)>tol))]; end ;k = k +1;clear FBA
0734     %% acorn -> orn
0735     model = modelOri;
0736     model.c(find(model.c)) = 0;
0737     [model] = addSinkReactions(model,{'acorn(c)','orn(c)'},[-1 -1; 0 100]);
0738     model.c(ismember(model.rxns,'sink_orn(c)'))=1;
0739     if find(model.c)>0
0740         FBA = optimizeCbModel(model,'max');
0741         TestSolution(k,1) = FBA.f;
0742     else
0743         TestSolution(k,1) = NaN;
0744     end
0745     TestSolutionName{k,1} = 'acorn(c) -> orn(c)';
0746     if ~isnan(TestSolution(k,1)); TestedRxns = [TestedRxns; model.rxns(find(abs(FBA.x)>tol))]; end ;k = k +1;clear FBA
0747     %% adrnl -> 34dhoxpeg
0748     model = modelOri;
0749     model.c(find(model.c)) = 0;
0750     [model] = addSinkReactions(model,{'adrnl(c)','34dhoxpeg(c)'},[-1 -1; 0 100]);
0751     model.c(ismember(model.rxns,'sink_34dhoxpeg(c)'))=1;
0752     if find(model.c)>0
0753         FBA = optimizeCbModel(model,'max');
0754         TestSolution(k,1) = FBA.f;
0755     else
0756         TestSolution(k,1) = NaN;
0757     end
0758     TestSolutionName{k,1} = 'adrnl(c) -> 34dhoxpeg(c)';
0759     if ~isnan(TestSolution(k,1)); TestedRxns = [TestedRxns; model.rxns(find(abs(FBA.x)>tol))]; end ;k = k +1;clear FBA
0760     %% adrnl -> 34dhoxpeg (2) % duplicate
0761     %% adrnl -> 34dhoxpeg (3) % duplicate
0762     %% akg[c] -> glu-L[c] % I adjusted lb since otherwise not feasible
0763     % model = modelOri;
0764     % model.c(find(model.c)) = 0;
0765     % [model] = addSinkReactions(model,{'akg(c)','glu-L(c)'},[-1 -1; 0 100]);
0766     % model.c(ismember(model.rxns,'sink_glu-L(c)'))=1;
0767     %   FBA = optimizeCbModel(model,'max');
0768     % TestSolution(k,1) = FBA.f;
0769     % TestSolutionName{k,1} = 'akg(c) -> glu-L(c)';
0770     % k = k +1;clear FBA
0771     %% akg[c] -> glu-L[c] % I adjusted lb since otherwise not feasible
0772     model = modelOri;
0773     model.c(find(model.c)) = 0;
0774     model.lb(ismember(model.rxns,'EX_akg(e)'))=-1;model.ub(ismember(model.rxns,'EX_akg(e)'))=-1;
0775     model.lb(ismember(model.rxns,'EX_o2(e)'))=-40;model.ub(ismember(model.rxns,'EX_o2(e)'))=-1;
0776     if ~isempty(strmatch('ALATA_L',model.rxns,'exact'))
0777         model.c(ismember(model.rxns,'ALATA_L'))=1;
0778         FBA = optimizeCbModel(model,'max');
0779         TestSolution(k,1) = FBA.f;
0780     else
0781         TestSolution(k,1) = NaN;
0782     end
0783     TestSolutionName{k,1} = 'akg(c) -> glu-L(c) (ALATA_L)';
0784     if ~isnan(TestSolution(k,1)); TestedRxns = [TestedRxns; model.rxns(find(abs(FBA.x)>tol))]; end ;k = k +1;clear FBA
0785     
0786     %% akg[c] -> glu-L[c]
0787     model = modelOri;
0788     model.c(find(model.c)) = 0;
0789     model.lb(ismember(model.rxns,'EX_akg(e)'))=-1;model.ub(ismember(model.rxns,'EX_akg(e)'))=-1;
0790     model.lb(ismember(model.rxns,'EX_o2(e)'))=-40;model.ub(ismember(model.rxns,'EX_o2(e)'))=-1;
0791     if ~isempty(strmatch('ASPTA',model.rxns,'exact'))
0792         model.c(ismember(model.rxns,'ASPTA'))=1;
0793         FBA = optimizeCbModel(model,'max');
0794         TestSolution(k,1) = FBA.f;
0795     else
0796         TestSolution(k,1) = NaN;
0797     end
0798     TestSolutionName{k,1} = 'akg(c) -> glu-L(c) (ASPTA)';
0799     if ~isnan(TestSolution(k,1)); TestedRxns = [TestedRxns; model.rxns(find(abs(FBA.x)>tol))]; end ;k = k +1;clear FBA
0800     
0801     %% akg[m[ -> oaa[m]
0802     model = modelOri;
0803     model.c(find(model.c)) = 0;
0804     [model] = addSinkReactions(model,{'akg(m)','oaa(m)'},[-1 -1; 0 100]);
0805     model.c(ismember(model.rxns,'sink_oaa(m)'))=1;
0806     if find(model.c)>0
0807         FBA = optimizeCbModel(model,'max');
0808         TestSolution(k,1) = FBA.f;
0809     else
0810         TestSolution(k,1) = NaN;
0811     end
0812     TestSolutionName{k,1} = 'akg(m) -> oaa(m)';
0813     if ~isnan(TestSolution(k,1)); TestedRxns = [TestedRxns; model.rxns(find(abs(FBA.x)>tol))]; end ;k = k +1;clear FBA
0814     %% akg[m] -> glu-L[m]
0815     model = modelOri;
0816     model.c(find(model.c)) = 0;
0817     [model] = addSinkReactions(model,{'akg(m)','glu-L(m)'},[-1 -1; 0 100]);
0818     model.c(ismember(model.rxns,'sink_glu-L(m)'))=1;
0819     if find(model.c)>0
0820         FBA = optimizeCbModel(model,'max');
0821         TestSolution(k,1) = FBA.f;
0822     else
0823         TestSolution(k,1) = NaN;
0824     end
0825     TestSolutionName{k,1} = 'akg(m) -> glu-L(m)';
0826     if ~isnan(TestSolution(k,1)); TestedRxns = [TestedRxns; model.rxns(find(abs(FBA.x)>tol))]; end ;k = k +1;clear FBA
0827     model = modelOri;
0828     model.c(find(model.c)) = 0;
0829     [model] = addSinkReactions(model,{'akg(m)'},-1 , -1);
0830     if ~isempty(strmatch('ASPTAm',model.rxns,'exact'))
0831         model.c(ismember(model.rxns,'ASPTAm'))=1;
0832         FBA = optimizeCbModel(model,'max');
0833         TestSolution(k,1) = FBA.f;
0834     else
0835         TestSolution(k,1) = NaN;
0836     end
0837     TestSolutionName{k,1} = 'akg(m) -> glu-L(m) (ASPTAm)';
0838     if ~isnan(TestSolution(k,1)); TestedRxns = [TestedRxns; model.rxns(find(abs(FBA.x)>tol))]; end ;k = k +1;clear FBA
0839     
0840     %% ala-B -> msa
0841     model = modelOri;
0842     model.c(find(model.c)) = 0;
0843     [model] = addSinkReactions(model,{'ala-B(c)','msa(m)'},[-1 -1; 0 100]);
0844     model.c(ismember(model.rxns,'sink_msa(m)'))=1;
0845     if find(model.c)>0
0846         FBA = optimizeCbModel(model,'max');
0847         TestSolution(k,1) = FBA.f;
0848     else
0849         TestSolution(k,1) = NaN;
0850     end
0851     TestSolutionName{k,1} = 'ala-B(c) -> msa(m)';
0852     if ~isnan(TestSolution(k,1)); TestedRxns = [TestedRxns; model.rxns(find(abs(FBA.x)>tol))]; end ;k = k +1;clear FBA
0853     %% ala-D -> pyr
0854     model = modelOri;
0855     model.c(find(model.c)) = 0;
0856     [model] = addSinkReactions(model,{'ala-D(c)','pyr(c)'},[-1 -1; 0 100]);
0857     model.c(ismember(model.rxns,'sink_pyr(c)'))=1;
0858     if find(model.c)>0
0859         FBA = optimizeCbModel(model,'max');
0860         TestSolution(k,1) = FBA.f;
0861     else
0862         TestSolution(k,1) = NaN;
0863     end
0864     TestSolutionName{k,1} = 'ala-D(c) -> pyr(c)';
0865     if ~isnan(TestSolution(k,1)); TestedRxns = [TestedRxns; model.rxns(find(abs(FBA.x)>tol))]; end ;k = k +1;clear FBA
0866     %% ala-L -> ala-D
0867     model = modelOri;
0868     model.c(find(model.c)) = 0;
0869     [model] = addSinkReactions(model,{'ala-L(c)','ala-D(c)'},[-1 -1; 0 100]);
0870     model.c(ismember(model.rxns,'sink_ala-D(c)'))=1;
0871     if find(model.c)>0
0872         FBA = optimizeCbModel(model,'max');
0873         TestSolution(k,1) = FBA.f;
0874     else
0875         TestSolution(k,1) = NaN;
0876     end
0877     TestSolutionName{k,1} = 'ala-L(c) -> ala-D(c)';
0878     if ~isnan(TestSolution(k,1)); TestedRxns = [TestedRxns; model.rxns(find(abs(FBA.x)>tol))]; end ;k = k +1;clear FBA
0879     %% ala-L -> pyr
0880     model = modelOri;
0881     model.c(find(model.c)) = 0;
0882     [model] = addSinkReactions(model,{'ala-L(c)','pyr(c)'},[-1 -1; 0 100]);
0883     model.c(ismember(model.rxns,'sink_pyr(c)'))=1;
0884     if find(model.c)>0
0885         FBA = optimizeCbModel(model,'max');
0886         TestSolution(k,1) = FBA.f;
0887     else
0888         TestSolution(k,1) = NaN;
0889     end
0890     TestSolutionName{k,1} = 'ala-L(c) -> pyr(c)';
0891     if ~isnan(TestSolution(k,1)); TestedRxns = [TestedRxns; model.rxns(find(abs(FBA.x)>tol))]; end ;k = k +1;clear FBA
0892     %% arachd(c) -> malcoa(m)
0893     model = modelOri;
0894     model.c(find(model.c)) = 0;
0895     [model] = addSinkReactions(model,{'arachd(c)','malcoa(m)'},[-1 -1; 0 100]);
0896     model.c(ismember(model.rxns,'sink_malcoa(m)'))=1;
0897     if find(model.c)>0
0898         FBA = optimizeCbModel(model,'max');
0899         TestSolution(k,1) = FBA.f;
0900     else
0901         TestSolution(k,1) = NaN;
0902     end
0903     TestSolutionName{k,1} = 'arachd(c) -> malcoa(m)';
0904     if ~isnan(TestSolution(k,1)); TestedRxns = [TestedRxns; model.rxns(find(abs(FBA.x)>tol))]; end ;k = k +1;clear FBA
0905     %% arachd(r) -> txa2(r)
0906     model = modelOri;
0907     model.c(find(model.c)) = 0;
0908     [model] = addSinkReactions(model,{'arachd(r)','txa2(r)'},[-1 -1; 0 100]);
0909     model.c(ismember(model.rxns,'sink_txa2(r)'))=1;
0910     if find(model.c)>0
0911         FBA = optimizeCbModel(model,'max');
0912         TestSolution(k,1) = FBA.f;
0913     else
0914         TestSolution(k,1) = NaN;
0915     end
0916     TestSolutionName{k,1} = 'arachd(r) -> txa2(r)';
0917     if ~isnan(TestSolution(k,1)); TestedRxns = [TestedRxns; model.rxns(find(abs(FBA.x)>tol))]; end ;k = k +1;clear FBA
0918     %% arg-L -> creat
0919     model = modelOri;
0920     model.c(find(model.c)) = 0;
0921     [model] = addSinkReactions(model,{'arg-L(c)','creat(c)'},[-1 -1; 0 100]);
0922     model.c(ismember(model.rxns,'sink_creat(c)'))=1;
0923     if find(model.c)>0
0924         FBA = optimizeCbModel(model,'max');
0925         TestSolution(k,1) = FBA.f;
0926     else
0927         TestSolution(k,1) = NaN;
0928     end
0929     TestSolutionName{k,1} = 'arg-L(c) -> creat(c)';
0930     if ~isnan(TestSolution(k,1)); TestedRxns = [TestedRxns; model.rxns(find(abs(FBA.x)>tol))]; end ;k = k +1;clear FBA
0931     %% arg-L -> glu-L (m)
0932     model = modelOri;
0933     model.c(find(model.c)) = 0;
0934     [model] = addSinkReactions(model,{'arg-L(c)','glu-L(m)'},[-1 -1; 0 100]);
0935     model.c(ismember(model.rxns,'sink_glu-L(m)'))=1;
0936     if find(model.c)>0
0937         FBA = optimizeCbModel(model,'max');
0938         TestSolution(k,1) = FBA.f;
0939     else
0940         TestSolution(k,1) = NaN;
0941     end
0942     TestSolutionName{k,1} = 'arg-L -> glu-L (m)';
0943     if ~isnan(TestSolution(k,1)); TestedRxns = [TestedRxns; model.rxns(find(abs(FBA.x)>tol))]; end ;k = k +1;clear FBA
0944     %% arg-L -> no
0945     model = modelOri;
0946     model.c(find(model.c)) = 0;
0947     [model] = addSinkReactions(model,{'arg-L(c)','no(c)'},[-1 -1; 0 100]);
0948     model.c(ismember(model.rxns,'sink_no(c)'))=1;
0949     if find(model.c)>0
0950         FBA = optimizeCbModel(model,'max');
0951         TestSolution(k,1) = FBA.f;
0952     else
0953         TestSolution(k,1) = NaN;
0954     end
0955     TestSolutionName{k,1} = 'arg-L -> no';
0956     if ~isnan(TestSolution(k,1)); TestedRxns = [TestedRxns; model.rxns(find(abs(FBA.x)>tol))]; end ;k = k +1;clear FBA
0957     %% arg-L -> pcreat
0958     model = modelOri;
0959     model.c(find(model.c)) = 0;
0960     [model] = addSinkReactions(model,{'arg-L(c)','pcreat(c)'},[-1 -1; 0 100]);
0961     model.c(ismember(model.rxns,'sink_pcreat(c)'))=1;
0962     if find(model.c)>0
0963         FBA = optimizeCbModel(model,'max');
0964         TestSolution(k,1) = FBA.f;
0965     else
0966         TestSolution(k,1) = NaN;
0967     end
0968     TestSolutionName{k,1} = 'arg-L(c) -> pcreat(c)';
0969     if ~isnan(TestSolution(k,1)); TestedRxns = [TestedRxns; model.rxns(find(abs(FBA.x)>tol))]; end ;k = k +1;clear FBA
0970     %% ascb -> eryth
0971     model = modelOri;
0972     model.c(find(model.c)) = 0;
0973     [model] = addSinkReactions(model,{'ascb-L(c)','eryth(c)'},[-1 -1; 0 100]);
0974     model.lb(find(ismember(model.rxns,'DM_ascb_L(c)')))=-1;
0975     model.ub(find(ismember(model.rxns,'DM_ascb_L(c)')))=-1;
0976     model.c(ismember(model.rxns,'sink_eryth(c)'))=1;
0977     if find(model.c)>0
0978         FBA = optimizeCbModel(model,'max');
0979         TestSolution(k,1) = FBA.f;
0980     else
0981         TestSolution(k,1) = NaN;
0982     end
0983     TestSolutionName{k,1} = 'ascb-L(c) -> eryth(c)';
0984     if ~isnan(TestSolution(k,1)); TestedRxns = [TestedRxns; model.rxns(find(abs(FBA.x)>tol))]; end ;k = k +1;clear FBA
0985     %% ascb -> lyxnt
0986     model = modelOri;
0987     model.c(find(model.c)) = 0;
0988     [model] = addSinkReactions(model,{'ascb-L(c)','lyxnt(c)'},[-1 -1; 0 100]);
0989     model.lb(find(ismember(model.rxns,'DM_ascb_L(c)')))=-1;
0990     model.ub(find(ismember(model.rxns,'DM_ascb_L(c)')))=-1;
0991     model.c(ismember(model.rxns,'sink_lyxnt(c)'))=1;
0992     if find(model.c)>0
0993         FBA = optimizeCbModel(model,'max');
0994         TestSolution(k,1) = FBA.f;
0995     else
0996         TestSolution(k,1) = NaN;
0997     end
0998     TestSolutionName{k,1} = 'ascb-L(c) -> lyxnt(c)';
0999     if ~isnan(TestSolution(k,1)); TestedRxns = [TestedRxns; model.rxns(find(abs(FBA.x)>tol))]; end ;k = k +1;clear FBA
1000     %% ascb -> thrnt
1001     model = modelOri;
1002     model.c(find(model.c)) = 0;
1003     [model] = addSinkReactions(model,{'ascb-L(c)','thrnt(c)'},[-1 -1; 0 100]);
1004     model.lb(find(ismember(model.rxns,'DM_ascb_L(c)')))=-1;
1005     model.ub(find(ismember(model.rxns,'DM_ascb_L(c)')))=-1;
1006     model.c(ismember(model.rxns,'sink_thrnt(c)'))=1;
1007     if find(model.c)>0
1008         FBA = optimizeCbModel(model,'max');
1009         TestSolution(k,1) = FBA.f;
1010     else
1011         TestSolution(k,1) = NaN;
1012     end
1013     TestSolutionName{k,1} = 'ascb-L(c) -> thrnt(c)';
1014     if ~isnan(TestSolution(k,1)); TestedRxns = [TestedRxns; model.rxns(find(abs(FBA.x)>tol))]; end ;k = k +1;clear FBA
1015     %% ascb -> xylnt
1016     model = modelOri;
1017     model.c(find(model.c)) = 0;
1018     [model] = addSinkReactions(model,{'ascb-L(c)','xylnt(c)'},[-1 -1; 0 100]);
1019     model.lb(find(ismember(model.rxns,'DM_ascb_L(c)')))=-1;
1020     model.ub(find(ismember(model.rxns,'DM_ascb_L(c)')))=-1;
1021     model.c(ismember(model.rxns,'sink_xylnt(c)'))=1;
1022     if find(model.c)>0
1023         FBA = optimizeCbModel(model,'max');
1024         TestSolution(k,1) = FBA.f;
1025     else
1026         TestSolution(k,1) = NaN;
1027     end
1028     TestSolutionName{k,1} = 'ascb-L(c) -> xylnt(c)';
1029     if ~isnan(TestSolution(k,1)); TestedRxns = [TestedRxns; model.rxns(find(abs(FBA.x)>tol))]; end ;k = k +1;clear FBA
1030     %% asn-L -> oaa
1031     model = modelOri;
1032     model.c(find(model.c)) = 0;
1033     [model] = addSinkReactions(model,{'asn-L(c)','oaa(c)'},[-1 -1; 0 100]);
1034     model.c(ismember(model.rxns,'sink_oaa(c)'))=1;
1035     if find(model.c)>0
1036         FBA = optimizeCbModel(model,'max');
1037         TestSolution(k,1) = FBA.f;
1038     else
1039         TestSolution(k,1) = NaN;
1040     end
1041     TestSolutionName{k,1} = 'asn-L(c) -> oaa(c)';
1042     if ~isnan(TestSolution(k,1)); TestedRxns = [TestedRxns; model.rxns(find(abs(FBA.x)>tol))]; end ;k = k +1;clear FBA
1043     %% asp-L + hco3 -> arg-L
1044     model = modelOri;
1045     model.c(find(model.c)) = 0;
1046     [model] = addSinkReactions(model,{'asp-L(c)','hco3(c)','arg-L(c)'},[-1 -1;-1 -1;0 100]);
1047     model.c(ismember(model.rxns,'sink_arg-L(c)'))=1;
1048     if find(model.c)>0
1049         FBA = optimizeCbModel(model,'max');
1050         TestSolution(k,1) = FBA.f;
1051     else
1052         TestSolution(k,1) = NaN;
1053     end
1054     TestSolutionName{k,1} = 'asp-L(c) + hco3(c) -> arg-L(c)';
1055     if ~isnan(TestSolution(k,1)); TestedRxns = [TestedRxns; model.rxns(find(abs(FBA.x)>tol))]; end ;k = k +1;clear FBA
1056     %% asp-L -> ala-B
1057     model = modelOri;
1058     model.c(find(model.c)) = 0;
1059     [model] = addSinkReactions(model,{'asp-L(c)','ala-B(c)'},[-1 -1; 0 100]);
1060     model.c(ismember(model.rxns,'sink_ala-B(c)'))=1;
1061     if find(model.c)>0
1062         FBA = optimizeCbModel(model,'max');
1063         TestSolution(k,1) = FBA.f;
1064     else
1065         TestSolution(k,1) = NaN;
1066     end
1067     TestSolutionName{k,1} = 'asp-L(c) -> ala-B(c)';
1068     if ~isnan(TestSolution(k,1)); TestedRxns = [TestedRxns; model.rxns(find(abs(FBA.x)>tol))]; end ;k = k +1;clear FBA
1069     %% asp-L -> asn-L
1070     model = modelOri;
1071     model.c(find(model.c)) = 0;
1072     [model] = addSinkReactions(model,{'asp-L(c)','asn-L(c)'},[-1 -1; 0 100]);
1073     model.c(ismember(model.rxns,'sink_asn-L(c)'))=1;
1074     if find(model.c)>0
1075         FBA = optimizeCbModel(model,'max');
1076         TestSolution(k,1) = FBA.f;
1077     else
1078         TestSolution(k,1) = NaN;
1079     end
1080     TestSolutionName{k,1} = 'asp-L(c) -> asn-L(c)';
1081     if ~isnan(TestSolution(k,1)); TestedRxns = [TestedRxns; model.rxns(find(abs(FBA.x)>tol))]; end ;k = k +1;clear FBA
1082     %% asp-L -> fum (via argsuc)
1083     model = modelOri;
1084     model.c(find(model.c)) = 0;
1085     [model] = addSinkReactions(model,{'asp-L(c)','argsuc(c)'},[-1 -1; 0 100]);
1086     model.c(ismember(model.rxns,'sink_argsuc(c)'))=1;
1087     if find(model.c)>0
1088         FBA = optimizeCbModel(model,'max');
1089         TestSolution(k,1) = FBA.f;
1090     else
1091         TestSolution(k,1) = NaN;
1092     end
1093     TestSolutionName{k,1} = 'asp-L(c) -> argsuc(c), asp-L -> fum (via argsuc), 1';
1094     if ~isnan(TestSolution(k,1)); TestedRxns = [TestedRxns; model.rxns(find(abs(FBA.x)>tol))]; end ;k = k +1;clear FBA
1095     model = modelOri;
1096     model.c(find(model.c)) = 0;
1097     [model] = addSinkReactions(model,{'argsuc(c)','fum(c)'},[-1 -1; 0 100]);
1098     model.c(ismember(model.rxns,'sink_fum(c)'))=1;
1099     if find(model.c)>0
1100         FBA = optimizeCbModel(model,'max');
1101         TestSolution(k,1) = FBA.f;
1102     else
1103         TestSolution(k,1) = NaN;
1104     end
1105     TestSolutionName{k,1} = 'argsuc(c) -> fum(c), asp-L -> fum (via argsuc), 2';
1106     if ~isnan(TestSolution(k,1)); TestedRxns = [TestedRxns; model.rxns(find(abs(FBA.x)>tol))]; end ;k = k +1;clear FBA
1107     %% asp-L -> fum (via dcamp)
1108     model = modelOri;
1109     model.c(find(model.c)) = 0;
1110     [model] = addSinkReactions(model,{'asp-L(c)','dcamp(c)'},[-1 -1; 0 100]);
1111     model.lb(find(ismember(model.rxns,'sink_asp_L(c)')))=-1;
1112     model.ub(find(ismember(model.rxns,'sink_asp_L(c)')))=-1;
1113     model.c(ismember(model.rxns,'sink_dcamp(c)'))=1;
1114     if find(model.c)>0
1115         FBA = optimizeCbModel(model,'max');
1116         TestSolution(k,1) = FBA.f;
1117     else
1118         TestSolution(k,1) = NaN;
1119     end
1120     TestSolutionName{k,1} = 'asp-L(c) -> dcamp(c), asp-L -> fum (via dcamp), 1';
1121     if ~isnan(TestSolution(k,1)); TestedRxns = [TestedRxns; model.rxns(find(abs(FBA.x)>tol))]; end ;k = k +1;clear FBA
1122     model = modelOri;
1123     model.c(find(model.c)) = 0;
1124     model.lb(find(ismember(model.rxns,'sink_asp_L(c)')))=-1;
1125     model.ub(find(ismember(model.rxns,'sink_asp_L(c)')))=-1;
1126     [model] = addSinkReactions(model,{'dcamp(c)','fum(c)'},[-1 -1; 0 100]);
1127     model.c(ismember(model.rxns,'sink_fum(c)'))=1;
1128     if find(model.c)>0
1129         FBA = optimizeCbModel(model,'max');
1130         TestSolution(k,1) = FBA.f;
1131     else
1132         TestSolution(k,1) = NaN;
1133     end
1134     TestSolutionName{k,1} = 'dcamp(c) -> fum(c), asp-L -> fum (via dcamp), 2';
1135     if ~isnan(TestSolution(k,1)); TestedRxns = [TestedRxns; model.rxns(find(abs(FBA.x)>tol))]; end ;k = k +1;clear FBA
1136     model = modelOri;
1137     model.c(find(model.c)) = 0;
1138     model.lb(find(ismember(model.rxns,'sink_asp_L(c)')))=-1;
1139     model.ub(find(ismember(model.rxns,'sink_asp_L(c)')))=-1;
1140     [model] = addSinkReactions(model,{'dcamp(c)','fum(c)'},[-1 -1; 0 100]);
1141     if ~isempty(strmatch('ADSS',model.rxns,'exact'))
1142         model.c(ismember(model.rxns,'ADSS'))=1;
1143         FBA = optimizeCbModel(model,'max');
1144         TestSolution(k,1) = FBA.f;
1145     else
1146         TestSolution(k,1) = NaN;
1147     end
1148     TestSolutionName{k,1} = 'dcamp(c) -> fum(c), asp-L -> fum (via dcamp), 3';
1149     if ~isnan(TestSolution(k,1)); TestedRxns = [TestedRxns; model.rxns(find(abs(FBA.x)>tol))]; end ;k = k +1;clear FBA
1150     
1151     %% asp-L -> oaa
1152     model = modelOri;
1153     model.c(find(model.c)) = 0;
1154     [model] = addSinkReactions(model,{'asp-L(c)','oaa(c)'},[-1 -1; 0 100]);
1155     model.c(ismember(model.rxns,'sink_oaa(c)'))=1;
1156     if find(model.c)>0
1157         FBA = optimizeCbModel(model,'max');
1158         TestSolution(k,1) = FBA.f;
1159     else
1160         TestSolution(k,1) = NaN;
1161     end
1162     TestSolutionName{k,1} = 'asp-L(c) -> oaa(c)';
1163     if ~isnan(TestSolution(k,1)); TestedRxns = [TestedRxns; model.rxns(find(abs(FBA.x)>tol))]; end ;k = k +1;clear FBA
1164     %% carn -> ala-B
1165     model = modelOri;
1166     model.c(find(model.c)) = 0;
1167     [model] = addSinkReactions(model,{'carn(c)','ala-B(c)'},[-1 -1; 0 100]);
1168     model.c(ismember(model.rxns,'sink_ala-B(c)'))=1;
1169     if find(model.c)>0
1170         FBA = optimizeCbModel(model,'max');
1171         TestSolution(k,1) = FBA.f;
1172     else
1173         TestSolution(k,1) = NaN;
1174     end
1175     TestSolutionName{k,1} = 'carn -> ala-B';
1176     if ~isnan(TestSolution(k,1)); TestedRxns = [TestedRxns; model.rxns(find(abs(FBA.x)>tol))]; end ;k = k +1;clear FBA
1177     %% chol(c) + dag_hs(c) -> pe_hs(c)
1178     model = modelOri;
1179     model.c(find(model.c)) = 0;
1180     [model] = addSinkReactions(model,{'chol(c)','dag_hs(c)','pe_hs(c)'},[-1 -1;-1 -1;0 100]);
1181     model.c(ismember(model.rxns,'sink_pe_hs(c)'))=1;
1182     if find(model.c)>0
1183         FBA = optimizeCbModel(model,'max');
1184         TestSolution(k,1) = FBA.f;
1185     else
1186         TestSolution(k,1) = NaN;
1187     end
1188     TestSolutionName{k,1} = 'chol(c) + dag_hs(c) -> pe_hs(c)';
1189     if ~isnan(TestSolution(k,1)); TestedRxns = [TestedRxns; model.rxns(find(abs(FBA.x)>tol))]; end ;k = k +1;clear FBA
1190     %% choline -> betaine -> glycine
1191     model = modelOri;
1192     model.c(find(model.c)) = 0;
1193     [model] = addSinkReactions(model,{'chol(m)','glyb(m)'},[-1 -1; 0 100]);
1194     model.c(ismember(model.rxns,'sink_glyb(m)'))=1;
1195     if find(model.c)>0
1196         FBA = optimizeCbModel(model,'max');
1197         TestSolution(k,1) = FBA.f;
1198     else
1199         TestSolution(k,1) = NaN;
1200     end
1201     TestSolutionName{k,1} = 'choline -> betaine (glyb) -> glycine, 1 [m]';
1202     if ~isnan(TestSolution(k,1)); TestedRxns = [TestedRxns; model.rxns(find(abs(FBA.x)>tol))]; end ;k = k +1;clear FBA
1203     model = modelOri;
1204     model.c(find(model.c)) = 0;
1205     [model] = addSinkReactions(model,{'glyb(m)','gly(m)'},[-1 -1; 0 100]);
1206     model.c(ismember(model.rxns,'sink_gly(m)'))=1;
1207     if find(model.c)>0
1208         FBA = optimizeCbModel(model,'max');
1209         TestSolution(k,1) = FBA.f;
1210     else
1211         TestSolution(k,1) = NaN;
1212     end
1213     TestSolutionName{k,1} = 'choline -> betaine (glyb) -> glycine, 2 [m]';
1214     if ~isnan(TestSolution(k,1)); TestedRxns = [TestedRxns; model.rxns(find(abs(FBA.x)>tol))]; end ;k = k +1;clear FBA
1215     %% coke(r) -> pecgoncoa(r)
1216     model = modelOri;
1217     model.c(find(model.c)) = 0;
1218     model.lb(ismember(model.rxns,'EX_glc(e)'))=-1;model.ub(ismember(model.rxns,'EX_glc(e)'))=0;
1219     model.lb(ismember(model.rxns,'EX_o2(e)'))=-40;model.ub(ismember(model.rxns,'EX_o2(e)'))=-1;
1220     [model] = addSinkReactions(model,{'coke(r)','pecgoncoa(r)'},[-1 -1; 0 100]);
1221     model.c(ismember(model.rxns,'sink_pecgoncoa(r)'))=1;
1222     if find(model.c)>0
1223         FBA = optimizeCbModel(model,'max');
1224         TestSolution(k,1) = FBA.f;
1225     else
1226         TestSolution(k,1) = NaN;
1227     end
1228     TestSolutionName{k,1} = 'coke(r) -> pecgoncoa(r)';
1229     if ~isnan(TestSolution(k,1)); TestedRxns = [TestedRxns; model.rxns(find(abs(FBA.x)>tol))]; end ;k = k +1;clear FBA
1230     %% core2[g] -> ksii_core2[g]
1231     model = modelOri;
1232     model.c(find(model.c)) = 0;
1233     [model] = addSinkReactions(model,{'core2(g)','ksii_core2(g)'},[-1 -1; 0 100]);
1234     model.c(ismember(model.rxns,'sink_ksii_core2(g)'))=1;
1235     if find(model.c)>0
1236         FBA = optimizeCbModel(model,'max');
1237         TestSolution(k,1) = FBA.f;
1238     else
1239         TestSolution(k,1) = NaN;
1240     end
1241     TestSolutionName{k,1} = 'core2(g) -> ksii_core2(g)';
1242     if ~isnan(TestSolution(k,1)); TestedRxns = [TestedRxns; model.rxns(find(abs(FBA.x)>tol))]; end ;k = k +1;clear FBA
1243     %% core4[g] -> ksii_core4[g]
1244     model = modelOri;
1245     model.c(find(model.c)) = 0;
1246     [model] = addSinkReactions(model,{'core4(g)','ksii_core4(g)'},[-1 -1; 0 100]);
1247     model.c(ismember(model.rxns,'sink_ksii_core4(g)'))=1;
1248     if find(model.c)>0
1249         FBA = optimizeCbModel(model,'max');
1250         TestSolution(k,1) = FBA.f;
1251     else
1252         TestSolution(k,1) = NaN;
1253     end
1254     TestSolutionName{k,1} = 'core4(g) -> ksii_core4(g)';
1255     if ~isnan(TestSolution(k,1)); TestedRxns = [TestedRxns; model.rxns(find(abs(FBA.x)>tol))]; end ;k = k +1;clear FBA
1256     %% cspg_a[ly] -> 2 gal[ly] + glcur[ly] + xyl-D[ly] %I adjusted lb since otw infeasible
1257     model = modelOri;
1258     model.c(find(model.c)) = 0;
1259     [model] = addSinkReactions(model,{'cspg_a(l)','gal(l)','glcur(l)','xyl-D(l)'},[-1 -1; 0.1 100; 0.1 100; 0.1 100]);
1260     model.c(ismember(model.rxns,'sink_xyl-D(l)'))=1;
1261     if find(model.c)>0
1262         FBA = optimizeCbModel(model,'max');
1263         TestSolution(k,1) = FBA.f;
1264     else
1265         TestSolution(k,1) = NaN;
1266     end
1267     TestSolutionName{k,1} = 'cspg_a[ly] -> gal[ly] + glcur[ly] + xyl-D[ly]';
1268     if ~isnan(TestSolution(k,1)); TestedRxns = [TestedRxns; model.rxns(find(abs(FBA.x)>tol))]; end ;k = k +1;clear FBA
1269     %% cspg_b[ly] -> 2gal[ly] + glcur[ly] + xyl-D[ly]
1270     model = modelOri;
1271     model.c(find(model.c)) = 0;
1272     [model] = addSinkReactions(model,{'cspg_b(l)','gal(l)','glcur(l)','xyl-D(l)'},[-1 -1; 0.1 100; 0.1 100; 0.1 100]);
1273     model.c(ismember(model.rxns,'sink_xyl-D(l)'))=1;
1274     if find(model.c)>0
1275         FBA = optimizeCbModel(model,'max');
1276         TestSolution(k,1) = FBA.f;
1277     else
1278         TestSolution(k,1) = NaN;
1279     end
1280     TestSolutionName{k,1} = 'cspg_b[ly] -> gal[ly] + glcur[ly] + xyl-D[ly]';
1281     if ~isnan(TestSolution(k,1)); TestedRxns = [TestedRxns; model.rxns(find(abs(FBA.x)>tol))]; end ;k = k +1;clear FBA
1282     %% cspg_c[ly] -> 2 gal[ly] + glcur[ly] + xyl-D[ly]
1283     model = modelOri;
1284     model.c(find(model.c)) = 0;
1285     [model] = addSinkReactions(model,{'cspg_c(l)','gal(l)','glcur(l)','xyl-D(l)'},[-1 -1; 0.1 100; 0.1 100; 0.1 100]);
1286     model.c(ismember(model.rxns,'sink_xyl-D(l)'))=1;
1287     if find(model.c)>0
1288         FBA = optimizeCbModel(model,'max');
1289         TestSolution(k,1) = FBA.f;
1290     else
1291         TestSolution(k,1) = NaN;
1292     end
1293     TestSolutionName{k,1} = 'cspg_c[ly] -> gal[ly] + glcur[ly] + xyl-D[ly]';
1294     if ~isnan(TestSolution(k,1)); TestedRxns = [TestedRxns; model.rxns(find(abs(FBA.x)>tol))]; end ;k = k +1;clear FBA
1295     %% cspg_d[ly] -> 2 gal[ly] + glcur[ly] + xyl-D[ly]
1296     model = modelOri;
1297     model.c(find(model.c)) = 0;
1298     [model] = addSinkReactions(model,{'cspg_d(l)','gal(l)','glcur(l)','xyl-D(l)'},[-1 -1; 0.1 100; 0.1 100; 0.1 100]);
1299     model.c(ismember(model.rxns,'sink_xyl-D(l)'))=1;
1300     if find(model.c)>0
1301         FBA = optimizeCbModel(model,'max');
1302         TestSolution(k,1) = FBA.f;
1303     else
1304         TestSolution(k,1) = NaN;
1305     end
1306     TestSolutionName{k,1} = 'cspg_d[ly] -> gal[ly] + glcur[ly] + xyl-D[ly]';
1307     if ~isnan(TestSolution(k,1)); TestedRxns = [TestedRxns; model.rxns(find(abs(FBA.x)>tol))]; end ;k = k +1;clear FBA
1308     %% cspg_e[ly] -> 2 gal[ly] + glcur[ly] + xyl-D[ly]
1309     model = modelOri;
1310     model.c(find(model.c)) = 0;
1311     [model] = addSinkReactions(model,{'cspg_e(l)','gal(l)','glcur(l)','xyl-D(l)'},[-1 -1; 0.1 100; 0.1 100; 0.1 100]);
1312     model.c(ismember(model.rxns,'sink_xyl-D(l)'))=1;
1313     if find(model.c)>0
1314         FBA = optimizeCbModel(model,'max');
1315         TestSolution(k,1) = FBA.f;
1316     else
1317         TestSolution(k,1) = NaN;
1318     end
1319     TestSolutionName{k,1} = 'cspg_e[ly] -> gal[ly] + glcur[ly] + xyl-D[ly]';
1320     if ~isnan(TestSolution(k,1)); TestedRxns = [TestedRxns; model.rxns(find(abs(FBA.x)>tol))]; end ;k = k +1;clear FBA
1321     %% cys-L + glu-L + gly -> ghtrd
1322     model = modelOri;
1323     model.c(find(model.c)) = 0;
1324     [model] = addSinkReactions(model,{'cys-L(c)', 'glu-L(c)','gly(c)','gthrd(c)'},[-1 -1;-1 -1;-1 -1; 0 100]);
1325     model.c(ismember(model.rxns,'sink_gthrd(c)'))=1;
1326     if find(model.c)>0
1327         FBA = optimizeCbModel(model,'max');
1328         TestSolution(k,1) = FBA.f;
1329     else
1330         TestSolution(k,1) = NaN;
1331     end
1332     TestSolutionName{k,1} = 'cys-L + glu-L + gly -> ghtrd';
1333     if ~isnan(TestSolution(k,1)); TestedRxns = [TestedRxns; model.rxns(find(abs(FBA.x)>tol))]; end ;k = k +1;clear FBA
1334     %% cys-L -> 3sala -> so4 %I adjusted lb since otw infeasible
1335     model = modelOri;
1336     model.c(find(model.c)) = 0;
1337     [model] = addSinkReactions(model,{'cys-L(c)','3sala(c)'},[-1 -1; 0 100]);
1338     model.lb(ismember(model.rxns,'EX_so4(e)'))=0;model.ub(ismember(model.rxns,'EX_so4(e)'))=1000;
1339     model.c(ismember(model.rxns,'sink_3sala(c)'))=1;
1340     if find(model.c)>0
1341         FBA = optimizeCbModel(model,'max');
1342         TestSolution(k,1) = FBA.f;
1343     else
1344         TestSolution(k,1) = NaN;
1345     end
1346     TestSolutionName{k,1} = 'cys-L -> 3sala -> so4, 1';
1347     if ~isnan(TestSolution(k,1)); TestedRxns = [TestedRxns; model.rxns(find(abs(FBA.x)>tol))]; end ;k = k +1;clear FBA
1348     model = modelOri;
1349     model.c(find(model.c)) = 0;
1350     [model] = addSinkReactions(model,{'3sala(c)','so4(c)'},[-1 -1; 0 100]);
1351     model.lb(ismember(model.rxns,'EX_so4(e)'))=0;model.ub(ismember(model.rxns,'EX_so4(e)'))=1000;
1352     model.c(ismember(model.rxns,'sink_so4(c)'))=1;
1353     if find(model.c)>0
1354         FBA = optimizeCbModel(model,'max');
1355         TestSolution(k,1) = FBA.f;
1356     else
1357         TestSolution(k,1) = NaN;
1358     end
1359     TestSolutionName{k,1} = 'cys-L -> 3sala -> so4, 2';
1360     if ~isnan(TestSolution(k,1)); TestedRxns = [TestedRxns; model.rxns(find(abs(FBA.x)>tol))]; end ;k = k +1;clear FBA
1361     %% cys-L -> hyptaur
1362     model = modelOri;
1363     model.c(find(model.c)) = 0;
1364     [model] = addSinkReactions(model,{'cys-L(c)','hyptaur(c)'},[-1 -1; 0 100]);
1365     model.c(ismember(model.rxns,'sink_hyptaur(c)'))=1;
1366     if find(model.c)>0
1367         FBA = optimizeCbModel(model,'max');
1368         TestSolution(k,1) = FBA.f;
1369     else
1370         TestSolution(k,1) = NaN;
1371     end
1372     TestSolutionName{k,1} = 'cys-L(c) -> hyptaur(c)';
1373     if ~isnan(TestSolution(k,1)); TestedRxns = [TestedRxns; model.rxns(find(abs(FBA.x)>tol))]; end ;k = k +1;clear FBA
1374     %% cystine -> cys-L
1375     model = modelOri;
1376     model.c(find(model.c)) = 0;
1377     [model] = addSinkReactions(model,{'Lcystin(c)','cys-L(c)'},[-1 -1; 0 100]);
1378     model.c(ismember(model.rxns,'sink_cys-L(c)'))=1;
1379     if find(model.c)>0
1380         FBA = optimizeCbModel(model,'max');
1381         TestSolution(k,1) = FBA.f;
1382     else
1383         TestSolution(k,1) = NaN;
1384     end
1385     TestSolutionName{k,1} = 'cystine (Lcystin) -> cys-L';
1386     if ~isnan(TestSolution(k,1)); TestedRxns = [TestedRxns; model.rxns(find(abs(FBA.x)>tol))]; end ;k = k +1;clear FBA
1387     %% dhap -> mthgxl
1388     model = modelOri;
1389     model.c(find(model.c)) = 0;
1390     [model] = addSinkReactions(model,{'dhap(c)','mthgxl(c)'},[-1 -1; 0 100]);
1391     model.c(ismember(model.rxns,'sink_mthgxl(c)'))=1;
1392     if find(model.c)>0
1393         FBA = optimizeCbModel(model,'max');
1394         TestSolution(k,1) = FBA.f;
1395     else
1396         TestSolution(k,1) = NaN;
1397     end
1398     TestSolutionName{k,1} = 'dhap(c) -> mthgxl(c)';
1399     if ~isnan(TestSolution(k,1)); TestedRxns = [TestedRxns; model.rxns(find(abs(FBA.x)>tol))]; end ;k = k +1;clear FBA
1400     %% dmpp -> ggdp
1401     model = modelOri;
1402     model.c(find(model.c)) = 0;
1403     
1404     for i = 1 : length(RPMI_composition)
1405         model = changeRxnBounds(model,RPMI_composition{i},-1,'l');
1406     end
1407     
1408     [model] = addSinkReactions(model,{'dmpp(c)','ggdp(c)'},[-1 -1; 0 100]);
1409     model.c(ismember(model.rxns,'sink_ggdp(c)'))=1;
1410     if find(model.c)>0
1411         FBA = optimizeCbModel(model,'max');
1412         TestSolution(k,1) = FBA.f;
1413     else
1414         TestSolution(k,1) = NaN;
1415     end
1416     TestSolutionName{k,1} = 'dmpp(c) -> ggdp(c) (with RPMI medium)';
1417     if ~isnan(TestSolution(k,1)); TestedRxns = [TestedRxns; model.rxns(find(abs(FBA.x)>tol))]; end ;k = k +1;clear FBA
1418     %% dna(n) -> dna5mtc(n)
1419     model = modelOri;
1420     model.c(find(model.c)) = 0;
1421     for i = 1 : length(RPMI_composition)
1422         model = changeRxnBounds(model,RPMI_composition{i},-1,'l');
1423     end
1424     [model] = addSinkReactions(model,{'dna(n)','dna5mtc(n)'},[-1 -1; 0 100]);
1425     model.c(ismember(model.rxns,'sink_dna5mtc(n)'))=1;
1426     if find(model.c)>0
1427         FBA = optimizeCbModel(model,'max');
1428         TestSolution(k,1) = FBA.f;
1429     else
1430         TestSolution(k,1) = NaN;
1431     end
1432     TestSolutionName{k,1} = 'dna(n) -> dna5mtc(n) (with RPMI medium)';
1433     if ~isnan(TestSolution(k,1)); TestedRxns = [TestedRxns; model.rxns(find(abs(FBA.x)>tol))]; end ;k = k +1;clear FBA
1434     %% dolichol_L -> dolmanp_L(r)
1435     model = modelOri;
1436     model.c(find(model.c)) = 0;
1437     for i = 1 : length(RPMI_composition)
1438         model = changeRxnBounds(model,RPMI_composition{i},-1,'l');
1439     end
1440     [model] = addSinkReactions(model,{'dolichol_L(c)','dolmanp_L(r)'},[-1 -1; 0 100]);
1441     model.c(ismember(model.rxns,'sink_dolmanp_L(r)'))=1;
1442     if find(model.c)>0
1443         FBA = optimizeCbModel(model,'max');
1444         TestSolution(k,1) = FBA.f;
1445     else
1446         TestSolution(k,1) = NaN;
1447     end
1448     TestSolutionName{k,1} = 'dolichol_L(c) -> dolmanp_L(r) (with RPMI medium)';
1449     if ~isnan(TestSolution(k,1)); TestedRxns = [TestedRxns; model.rxns(find(abs(FBA.x)>tol))]; end ;k = k +1;clear FBA
1450     %% dolichol_L -> g3m8mpdol_L[r]
1451     model = modelOri;
1452     model.c(find(model.c)) = 0;
1453     for i = 1 : length(RPMI_composition)
1454         model = changeRxnBounds(model,RPMI_composition{i},-1,'l');
1455     end
1456     [model] = addSinkReactions(model,{'dolichol_L(c)','g3m8mpdol_L(r)'},[-1 -1; 0 100]);
1457     model.c(ismember(model.rxns,'sink_g3m8mpdol_L(r)'))=1;
1458     if find(model.c)>0
1459         FBA = optimizeCbModel(model,'max');
1460         TestSolution(k,1) = FBA.f;
1461     else
1462         TestSolution(k,1) = NaN;
1463     end
1464     TestSolutionName{k,1} = 'dolichol_L(c) -> g3m8mpdol_L(r) (with RPMI medium)';
1465     if ~isnan(TestSolution(k,1)); TestedRxns = [TestedRxns; model.rxns(find(abs(FBA.x)>tol))]; end ;k = k +1;clear FBA
1466     %% dolichol_U -> dolmanp_U[r]
1467     model = modelOri;
1468     for i = 1 : length(RPMI_composition)
1469         model = changeRxnBounds(model,RPMI_composition{i},-1,'l');
1470     end
1471     model.c(find(model.c)) = 0;
1472     [model] = addSinkReactions(model,{'dolichol_U(c)','dolmanp_U(r)'},[-1 -1; 0 100]);
1473     model.c(ismember(model.rxns,'sink_dolmanp_U(r)'))=1;
1474     if find(model.c)>0
1475         FBA = optimizeCbModel(model,'max');
1476         TestSolution(k,1) = FBA.f;
1477     else
1478         TestSolution(k,1) = NaN;
1479     end
1480     TestSolutionName{k,1} = 'dolichol_U(c) -> dolmanp_U(r) (with RPMI medium)';
1481     if ~isnan(TestSolution(k,1)); TestedRxns = [TestedRxns; model.rxns(find(abs(FBA.x)>tol))]; end ;k = k +1;clear FBA
1482     %% dolichol_U -> g3m8mpdol_U[r]
1483     model = modelOri;
1484     for i = 1 : length(RPMI_composition)
1485         model = changeRxnBounds(model,RPMI_composition{i},-1,'l');
1486     end
1487     model.c(find(model.c)) = 0;
1488     [model] = addSinkReactions(model,{'dolichol_U(c)','g3m8mpdol_U(r)'},[-1 -1; 0 100]);
1489     model.c(ismember(model.rxns,'sink_g3m8mpdol_U(r)'))=1;
1490     if find(model.c)>0
1491         FBA = optimizeCbModel(model,'max');
1492         TestSolution(k,1) = FBA.f;
1493     else
1494         TestSolution(k,1) = NaN;
1495     end
1496     TestSolutionName{k,1} = 'dolichol_U(c) -> g3m8mpdol_U(r) (with RPMI medium)';
1497     if ~isnan(TestSolution(k,1)); TestedRxns = [TestedRxns; model.rxns(find(abs(FBA.x)>tol))]; end ;k = k +1;clear FBA
1498     %% dopa -> homoval (1)
1499     model = modelOri;
1500     model.c(find(model.c)) = 0;
1501     model.lb(ismember(model.rxns,'EX_glc(e)'))=-1;model.ub(ismember(model.rxns,'EX_glc(e)'))=0;
1502     [model] = addSinkReactions(model,{'dopa(c)','homoval(c)'},[-1 -1; 0 100]);
1503     model.lb(find(ismember(model.rxns,'DM_dopa(c)')))=-1;
1504     model.ub(find(ismember(model.rxns,'DM_dopa(c)')))=-1;
1505     model.c(ismember(model.rxns,'sink_homoval(c)'))=1;
1506     if find(model.c)>0
1507         FBA = optimizeCbModel(model,'max');
1508         TestSolution(k,1) = FBA.f;
1509     else
1510         TestSolution(k,1) = NaN;
1511     end
1512     TestSolutionName{k,1} = 'dopa(c) -> homoval(c)';
1513     if ~isnan(TestSolution(k,1)); TestedRxns = [TestedRxns; model.rxns(find(abs(FBA.x)>tol))]; end ;k = k +1;clear FBA
1514     %% dopa -> homoval (2) %duplicate
1515     %% etoh -> acald
1516     model = modelOri;
1517     model.c(find(model.c)) = 0;
1518     [model] = addSinkReactions(model,{'etoh(c)','acald(c)'},[-1 -1; 0 100]);
1519     model.c(ismember(model.rxns,'sink_acald(c)'))=1;
1520     if find(model.c)>0
1521         FBA = optimizeCbModel(model,'max');
1522         TestSolution(k,1) = FBA.f;
1523     else
1524         TestSolution(k,1) = NaN;
1525     end
1526     TestSolutionName{k,1} = 'etoh(c) -> acald(c)';
1527     if ~isnan(TestSolution(k,1)); TestedRxns = [TestedRxns; model.rxns(find(abs(FBA.x)>tol))]; end ;k = k +1;clear FBA
1528     %% f6p + g3p -> r5p
1529     model = modelOri;
1530     model.c(find(model.c)) = 0;
1531     [model] = addSinkReactions(model,{'f6p(c)','g3p(c)','r5p(c)'},[-1 -1; -1 -1;0 100]);
1532     model.c(ismember(model.rxns,'sink_r5p(c)'))=1;
1533     if find(model.c)>0
1534         FBA = optimizeCbModel(model,'max');
1535         TestSolution(k,1) = FBA.f;
1536     else
1537         TestSolution(k,1) = NaN;
1538     end
1539     TestSolutionName{k,1} = 'f6p(c) + g3p(c) -> r5p(c)';
1540     if ~isnan(TestSolution(k,1)); TestedRxns = [TestedRxns; model.rxns(find(abs(FBA.x)>tol))]; end ;k = k +1;clear FBA
1541     %% frdp -> dolichol_L
1542     model = modelOri;
1543     model.c(find(model.c)) = 0;
1544     for i = 1 : length(RPMI_composition)
1545         model = changeRxnBounds(model,RPMI_composition{i},-1,'l');
1546     end
1547     [model] = addSinkReactions(model,{'frdp(c)','dolichol_L(r)'},[-1 -1; 0 100]);
1548     model.c(ismember(model.rxns,'sink_dolichol_L(r)'))=1;
1549     if find(model.c)>0
1550         FBA = optimizeCbModel(model,'max');
1551         TestSolution(k,1) = FBA.f;
1552     else
1553         TestSolution(k,1) = NaN;
1554     end
1555     TestSolutionName{k,1} = 'frdp(c) -> dolichol_L(r) (with RPMI medium)';
1556     if ~isnan(TestSolution(k,1)); TestedRxns = [TestedRxns; model.rxns(find(abs(FBA.x)>tol))]; end ;k = k +1;clear FBA
1557     %% frdp -> dolichol_U
1558     model = modelOri;
1559     model.c(find(model.c)) = 0;
1560     for i = 1 : length(RPMI_composition)
1561         model = changeRxnBounds(model,RPMI_composition{i},-1,'l');
1562     end
1563     [model] = addSinkReactions(model,{'frdp(c)','dolichol_U(r)'},[-1 -1; 0 100]);
1564     model.c(ismember(model.rxns,'sink_dolichol_U(r)'))=1;
1565     if find(model.c)>0
1566         FBA = optimizeCbModel(model,'max');
1567         TestSolution(k,1) = FBA.f;
1568     else
1569         TestSolution(k,1) = NaN;
1570     end
1571     TestSolutionName{k,1} = 'frdp(c) -> dolichol_U(r) (with RPMI medium)';
1572     if ~isnan(TestSolution(k,1)); TestedRxns = [TestedRxns; model.rxns(find(abs(FBA.x)>tol))]; end ;k = k +1;clear FBA
1573     %% from ade(c) to amp(c)
1574     model = modelOri;
1575     model.c(find(model.c)) = 0;
1576     model.lb(ismember(model.rxns,'EX_glc(e)'))=-1;model.ub(ismember(model.rxns,'EX_glc(e)'))=0;
1577     model.lb(ismember(model.rxns,'EX_o2(e)'))=-40;model.ub(ismember(model.rxns,'EX_o2(e)'))=-1;
1578     [model] = addSinkReactions(model,{'ade(c)','amp(c)'},[-1 -1; 0 100]);
1579     model.c(ismember(model.rxns,'sink_amp(c)'))=1;
1580     if find(model.c)>0
1581         FBA = optimizeCbModel(model,'max');
1582         TestSolution(k,1) = FBA.f;
1583     else
1584         TestSolution(k,1) = NaN;
1585     end
1586     TestSolutionName{k,1} = 'ade(c) -> amp(c)';
1587     if ~isnan(TestSolution(k,1)); TestedRxns = [TestedRxns; model.rxns(find(abs(FBA.x)>tol))]; end ;k = k +1;clear FBA
1588     %% from adn(c) to urate(x)
1589     model = modelOri;
1590     model.c(find(model.c)) = 0;
1591     [model] = addSinkReactions(model,{'adn(c)','urate(x)'},[-1 -1; 0 100]);
1592     model.c(ismember(model.rxns,'sink_urate(x)'))=1;
1593     if find(model.c)>0
1594         FBA = optimizeCbModel(model,'max');
1595         TestSolution(k,1) = FBA.f;
1596     else
1597         TestSolution(k,1) = NaN;
1598     end
1599     TestSolutionName{k,1} = 'adn(c) -> urate(x)';
1600     if ~isnan(TestSolution(k,1)); TestedRxns = [TestedRxns; model.rxns(find(abs(FBA.x)>tol))]; end ;k = k +1;clear FBA
1601     %% from ADP(c) to dATP(n)
1602     model = modelOri;
1603     model.c(find(model.c)) = 0;
1604     [model,rxnsInModel] = addSinkReactions(model,{'adp(c)','datp(n)'},[-1 -1; 0 100]);
1605     if (rxnsInModel(2) >-1) % reaction exits already in model
1606         model=changeObjective(model,model.rxns(rxnsInModel(2),1));
1607     else
1608         model=changeObjective(model,'sink_datp(n)',1);
1609     end
1610     if find(model.c)>0
1611         FBA = optimizeCbModel(model,'max');
1612         TestSolution(k,1) = FBA.f;
1613     else
1614         TestSolution(k,1) = NaN;
1615     end
1616     TestSolutionName{k,1} = 'adp(c) -> datp(n)';
1617     if ~isnan(TestSolution(k,1)); TestedRxns = [TestedRxns; model.rxns(find(abs(FBA.x)>tol))]; end ;k = k +1;clear FBA
1618     %% from CDP(c) to dCTP(n)
1619     model = modelOri;
1620     model.c(find(model.c)) = 0;
1621     [model,rxnsInModel] = addSinkReactions(model,{'cdp(c)','dctp(n)'},[-1 -1; 0 100]);
1622     if (rxnsInModel(2) >-1) % reaction exits already in model
1623         model=changeObjective(model,model.rxns(rxnsInModel(2),1));
1624     else
1625         model=changeObjective(model,'sink_dctp(n)',1);
1626     end
1627     if find(model.c)>0
1628         FBA = optimizeCbModel(model,'max');
1629         TestSolution(k,1) = FBA.f;
1630     else
1631         TestSolution(k,1) = NaN;
1632     end
1633     TestSolutionName{k,1} = 'cdp(c) -> dctp(n)';
1634     if ~isnan(TestSolution(k,1)); TestedRxns = [TestedRxns; model.rxns(find(abs(FBA.x)>tol))]; end ;k = k +1;clear FBA
1635     %% from cmp to cytd
1636     model = modelOri;
1637     model.c(find(model.c)) = 0;
1638     [model] = addSinkReactions(model,{'cmp(c)','cytd(c)'},[-1 -1; 0 100]);
1639     model.c(ismember(model.rxns,'sink_cytd(c)'))=1;
1640     if find(model.c)>0
1641         FBA = optimizeCbModel(model,'max');
1642         TestSolution(k,1) = FBA.f;
1643     else
1644         TestSolution(k,1) = NaN;
1645     end
1646     TestSolutionName{k,1} = 'cmp(c) -> cytd(c)';
1647     if ~isnan(TestSolution(k,1)); TestedRxns = [TestedRxns; model.rxns(find(abs(FBA.x)>tol))]; end ;k = k +1;clear FBA
1648     %% from cytd to ala-B
1649     model = modelOri;
1650     model.c(find(model.c)) = 0;
1651     [model] = addSinkReactions(model,{'cytd(c)','ala-B(c)'},[-1 -1; 0 100]);
1652     model.c(ismember(model.rxns,'sink_ala-B(c)'))=1;
1653     if find(model.c)>0
1654         FBA = optimizeCbModel(model,'max');
1655         TestSolution(k,1) = FBA.f;
1656     else
1657         TestSolution(k,1) = NaN;
1658     end
1659     TestSolutionName{k,1} = 'cytd(c) -> ala-B(c)';
1660     if ~isnan(TestSolution(k,1)); TestedRxns = [TestedRxns; model.rxns(find(abs(FBA.x)>tol))]; end ;k = k +1;clear FBA
1661     %% from dcmp to ala-B
1662     model = modelOri;
1663     model.c(find(model.c)) = 0;
1664     [model] = addSinkReactions(model,{'dcmp(c)','ala-B(c)'},[-1 -1; 0 100]);
1665     model.c(ismember(model.rxns,'sink_ala-B(c)'))=1;
1666     if find(model.c)>0
1667         FBA = optimizeCbModel(model,'max');
1668         TestSolution(k,1) = FBA.f;
1669     else
1670         TestSolution(k,1) = NaN;
1671     end
1672     TestSolutionName{k,1} = 'dcmp(c) -> ala-B(c)';
1673     if ~isnan(TestSolution(k,1)); TestedRxns = [TestedRxns; model.rxns(find(abs(FBA.x)>tol))]; end ;k = k +1;clear FBA
1674     %% from GDP(c) to dGTP(n)
1675     model = modelOri;
1676     model.c(find(model.c)) = 0;
1677     [model] = addSinkReactions(model,{'gdp(c)','dgtp(n)'},[-1 -1; 0 100]);
1678     if (rxnsInModel(2) >-1) % reaction exits already in model
1679         model=changeObjective(model,model.rxns(rxnsInModel(2),1));
1680     else
1681         model=changeObjective(model,'sink_dgtp(n)',1);
1682     end
1683     if find(model.c)>0
1684         FBA = optimizeCbModel(model,'max');
1685         TestSolution(k,1) = FBA.f;
1686     else
1687         TestSolution(k,1) = NaN;
1688     end
1689     TestSolutionName{k,1} = 'gdp(c) -> dgtp(n)';
1690     if ~isnan(TestSolution(k,1)); TestedRxns = [TestedRxns; model.rxns(find(abs(FBA.x)>tol))]; end ;k = k +1;clear FBA
1691     %% from gln-L + HCO3 to UMP(c)
1692     model = modelOri;
1693     model.c(find(model.c)) = 0;
1694     [model] = addSinkReactions(model,{'gln-L(c)','hco3(c)','ump(c)'},[-1 -1;-1 -1; 0 100]);
1695     model.c(ismember(model.rxns,'sink_ump(c)'))=1;
1696     if find(model.c)>0
1697         FBA = optimizeCbModel(model,'max');
1698         TestSolution(k,1) = FBA.f;
1699     else
1700         TestSolution(k,1) = NaN;
1701     end
1702     TestSolutionName{k,1} = 'gln-L + HCO3 -> UMP(c)';
1703     if ~isnan(TestSolution(k,1)); TestedRxns = [TestedRxns; model.rxns(find(abs(FBA.x)>tol))]; end ;k = k +1;clear FBA
1704     %% from gsn(c) to urate(x)
1705     model = modelOri;
1706     model.c(find(model.c)) = 0;
1707     [model] = addSinkReactions(model,{'gsn(c)','urate(x)'},[-1 -1; 0 100]);
1708     model.c(ismember(model.rxns,'sink_urate(x)'))=1;
1709     if find(model.c)>0
1710         FBA = optimizeCbModel(model,'max');
1711         TestSolution(k,1) = FBA.f;
1712     else
1713         TestSolution(k,1) = NaN;
1714     end
1715     TestSolutionName{k,1} = 'gsn(c) -> urate(x)';
1716     if ~isnan(TestSolution(k,1)); TestedRxns = [TestedRxns; model.rxns(find(abs(FBA.x)>tol))]; end ;k = k +1;clear FBA
1717     %% from gua(c) to gmp(c)
1718     model = modelOri;
1719     model.c(find(model.c)) = 0;
1720     model.lb(ismember(model.rxns,'EX_glc(e)'))=-1;model.ub(ismember(model.rxns,'EX_glc(e)'))=0;
1721     model.lb(ismember(model.rxns,'EX_o2(e)'))=-40;model.ub(ismember(model.rxns,'EX_o2(e)'))=-1;
1722     [model] = addSinkReactions(model,{'gua(c)','gmp(c)'},[-1 -1; 0 100]);
1723     model.c(ismember(model.rxns,'sink_gmp(c)'))=1;
1724     if find(model.c)>0
1725         FBA = optimizeCbModel(model,'max');
1726         TestSolution(k,1) = FBA.f;
1727     else
1728         TestSolution(k,1) = NaN;
1729     end
1730     TestSolutionName{k,1} = 'gua(c) -> gmp(c)';
1731     if ~isnan(TestSolution(k,1)); TestedRxns = [TestedRxns; model.rxns(find(abs(FBA.x)>tol))]; end ;k = k +1;clear FBA
1732     %% from hxan(c) to imp(c)
1733     model = modelOri;
1734     model.c(find(model.c)) = 0;
1735     model.lb(ismember(model.rxns,'EX_glc(e)'))=-1;model.ub(ismember(model.rxns,'EX_glc(e)'))=0;
1736     model.lb(ismember(model.rxns,'EX_o2(e)'))=-40;model.ub(ismember(model.rxns,'EX_o2(e)'))=-1;
1737     [model] = addSinkReactions(model,{'hxan(c)','imp(c)'},[-1 -1; 0 100]);
1738     model.c(ismember(model.rxns,'sink_imp(c)'))=1;
1739     if find(model.c)>0
1740         FBA = optimizeCbModel(model,'max');
1741         TestSolution(k,1) = FBA.f;
1742     else
1743         TestSolution(k,1) = NaN;
1744     end
1745     TestSolutionName{k,1} = 'hxan(c) -> imp(c)';
1746     if ~isnan(TestSolution(k,1)); TestedRxns = [TestedRxns; model.rxns(find(abs(FBA.x)>tol))]; end ;k = k +1;clear FBA
1747     %% from imp to ATP
1748     model = modelOri;
1749     model.c(find(model.c)) = 0;
1750     [model] = addSinkReactions(model,{'imp(c)','atp(c)'},[-1 -1; 0 100]);
1751     model.c(ismember(model.rxns,'sink_atp(c)'))=1;
1752     if find(model.c)>0
1753         FBA = optimizeCbModel(model,'max');
1754         TestSolution(k,1) = FBA.f;
1755     else
1756         TestSolution(k,1) = NaN;
1757     end
1758     TestSolutionName{k,1} = 'imp(c) -> atp(c)';
1759     if ~isnan(TestSolution(k,1)); TestedRxns = [TestedRxns; model.rxns(find(abs(FBA.x)>tol))]; end ;k = k +1;clear FBA
1760     %% from imp to gtp
1761     model = modelOri;
1762     model.c(find(model.c)) = 0;
1763     [model] = addSinkReactions(model,{'imp(c)','gtp(c)'},[-1 -1; 0 100]);
1764     model.c(ismember(model.rxns,'sink_gtp(c)'))=1;
1765     if find(model.c)>0
1766         FBA = optimizeCbModel(model,'max');
1767         TestSolution(k,1) = FBA.f;
1768     else
1769         TestSolution(k,1) = NaN;
1770     end
1771     TestSolutionName{k,1} = 'imp(c) -> gtp(c)';
1772     if ~isnan(TestSolution(k,1)); TestedRxns = [TestedRxns; model.rxns(find(abs(FBA.x)>tol))]; end ;k = k +1;clear FBA
1773     %% from imp(c) to urate(x)
1774     model = modelOri;
1775     model.c(find(model.c)) = 0;
1776     [model] = addSinkReactions(model,{'imp(c)','urate(x)'},[-1 -1; 0 100]);
1777     model.c(ismember(model.rxns,'sink_urate(x)'))=1;
1778     if find(model.c)>0
1779         FBA = optimizeCbModel(model,'max');
1780         TestSolution(k,1) = FBA.f;
1781     else
1782         TestSolution(k,1) = NaN;
1783     end
1784     TestSolutionName{k,1} = 'imp(c) -> urate(x)';
1785     if ~isnan(TestSolution(k,1)); TestedRxns = [TestedRxns; model.rxns(find(abs(FBA.x)>tol))]; end ;k = k +1;clear FBA
1786     %% from prpp to imp
1787     model = modelOri;
1788     model.c(find(model.c)) = 0;
1789     [model] = addSinkReactions(model,{'prpp(c)','imp(c)'},[-1 -1; 0 100]);
1790     model.c(ismember(model.rxns,'sink_imp(c)'))=1;
1791     if find(model.c)>0
1792         FBA = optimizeCbModel(model,'max');
1793         TestSolution(k,1) = FBA.f;
1794     else
1795         TestSolution(k,1) = NaN;
1796     end
1797     TestSolutionName{k,1} = 'prpp(c) -> imp(c)';
1798     if ~isnan(TestSolution(k,1)); TestedRxns = [TestedRxns; model.rxns(find(abs(FBA.x)>tol))]; end ;k = k +1;clear FBA
1799     %% from pydx(c) to pydx5p(c)
1800     model = modelOri;
1801     model.c(find(model.c)) = 0;
1802     model.lb(ismember(model.rxns,'EX_glc(e)'))=-1;model.ub(ismember(model.rxns,'EX_glc(e)'))=0;
1803     model.lb(ismember(model.rxns,'EX_o2(e)'))=-40;model.ub(ismember(model.rxns,'EX_o2(e)'))=-1;
1804     [model] = addSinkReactions(model,{'pydx(c)','pydx5p(c)'},[-1 -1; 0 100]);
1805     model.lb(find(ismember(model.rxns,'sink_pydx(c)')))=-1;
1806     model.ub(find(ismember(model.rxns,'sink_pydx(c)')))=-1;
1807     model.c(ismember(model.rxns,'sink_pydx5p(c)'))=1;
1808     if find(model.c)>0
1809         FBA = optimizeCbModel(model,'max');
1810         TestSolution(k,1) = FBA.f;
1811     else
1812         TestSolution(k,1) = NaN;
1813     end
1814     TestSolutionName{k,1} = 'pydx(c) -> pydx5p(c)';
1815     if ~isnan(TestSolution(k,1)); TestedRxns = [TestedRxns; model.rxns(find(abs(FBA.x)>tol))]; end ;k = k +1;clear FBA
1816     %% from thm(c) to thmpp(c)
1817     model = modelOri;
1818     model.c(find(model.c)) = 0;
1819     for i = 1 : length(RPMI_composition)
1820         model = changeRxnBounds(model,RPMI_composition{i},-1,'l');
1821     end
1822     [model] = addSinkReactions(model,{'thm(c)','thmpp(c)'},[-1 -1; 0 100]);
1823     model.c(ismember(model.rxns,'sink_thmpp(c)'))=1;
1824     if find(model.c)>0
1825         FBA = optimizeCbModel(model,'max');
1826         TestSolution(k,1) = FBA.f;
1827     else
1828         TestSolution(k,1) = NaN;
1829     end
1830     TestSolutionName{k,1} = 'thm(c) -> thmpp(c) (with RPMI medium)';
1831     if ~isnan(TestSolution(k,1)); TestedRxns = [TestedRxns; model.rxns(find(abs(FBA.x)>tol))]; end ;k = k +1;clear FBA
1832     %% from thm(e) to thmpp(m) %does not work; changing lb has no effect
1833     model = modelOri;
1834     model.c(find(model.c)) = 0;
1835     for i = 1 : length(RPMI_composition)
1836         model = changeRxnBounds(model,RPMI_composition{i},-1,'l');
1837     end
1838     [model] = addSinkReactions(model,{'thm(e)','thmpp(m)'},[-1 -1; 0 100]);
1839     model.lb(find(ismember(model.rxns,'EX_thm(e)')))=-1;
1840     model.ub(find(ismember(model.rxns,'EX_thm(e)')))=-1;
1841     model.c(ismember(model.rxns,'sink_thmpp(m)'))=1;
1842     if find(model.c)>0
1843         FBA = optimizeCbModel(model,'max');
1844         TestSolution(k,1) = FBA.f;
1845     else
1846         TestSolution(k,1) = NaN;
1847     end
1848     TestSolutionName{k,1} = 'thm(e) -> thmpp(m) (with RPMI medium)';
1849     if ~isnan(TestSolution(k,1)); TestedRxns = [TestedRxns; model.rxns(find(abs(FBA.x)>tol))]; end ;k = k +1;clear FBA
1850     %% from thmmp(e) to thmpp(c)
1851     model = modelOri;
1852     model.c(find(model.c)) = 0;
1853     for i = 1 : length(RPMI_composition)
1854         model = changeRxnBounds(model,RPMI_composition{i},-1,'l');
1855     end
1856     [model] = addSinkReactions(model,{'thmmp(e)','thmpp(c)'},[-1 -1; 0 100]);
1857     model.lb(find(ismember(model.rxns,'EX_thmmp(e)')))=-1;
1858     model.ub(find(ismember(model.rxns,'EX_thmmp(e)')))=-1;
1859     model.c(ismember(model.rxns,'sink_thmpp(c)'))=1;
1860     if find(model.c)>0
1861         FBA = optimizeCbModel(model,'max');
1862         TestSolution(k,1) = FBA.f;
1863     else
1864         TestSolution(k,1) = NaN;
1865     end
1866     TestSolutionName{k,1} = 'thmmp(e) -> thmpp(c) (with RPMI medium)';
1867     if ~isnan(TestSolution(k,1)); TestedRxns = [TestedRxns; model.rxns(find(abs(FBA.x)>tol))]; end ;k = k +1;clear FBA
1868     %% from thmmp(e) to thmpp(m)%does not work; changing lb has no effect
1869     model = modelOri;
1870     model.c(find(model.c)) = 0;
1871     for i = 1 : length(RPMI_composition)
1872         model = changeRxnBounds(model,RPMI_composition{i},-1,'l');
1873     end
1874     model.lb(find(ismember(model.rxns,'EX_thmmp(e)')))=-1;
1875     model.ub(find(ismember(model.rxns,'EX_thmmp(e)')))=-1;
1876     [model] = addSinkReactions(model,{'thmpp(m)'},[ 0 100]);
1877     model.c(ismember(model.rxns,'sink_thmpp(m)'))=1;
1878     if find(model.c)>0
1879         FBA = optimizeCbModel(model,'max');
1880         TestSolution(k,1) = FBA.f;
1881     else
1882         TestSolution(k,1) = NaN;
1883     end
1884     TestSolutionName{k,1} = 'thmmp(e) -> thmpp(m) (with RPMI medium)';
1885     if ~isnan(TestSolution(k,1)); TestedRxns = [TestedRxns; model.rxns(find(abs(FBA.x)>tol))]; end ;k = k +1;clear FBA
1886     %% from tyr-L(m) to q10(m)
1887     model = modelOri;
1888     model.c(find(model.c)) = 0;
1889     [model] = addSinkReactions(model,{'tyr-L(m)','q10(m)'},[-1 -1; 0 100]);
1890     model.c(ismember(model.rxns,'sink_q10(m)'))=1;
1891     if find(model.c)>0
1892         FBA = optimizeCbModel(model,'max');
1893         TestSolution(k,1) = FBA.f;
1894     else
1895         TestSolution(k,1) = NaN;
1896     end
1897     TestSolutionName{k,1} = 'tyr-L(m) -> q10(m)';
1898     if ~isnan(TestSolution(k,1)); TestedRxns = [TestedRxns; model.rxns(find(abs(FBA.x)>tol))]; end ;k = k +1;clear FBA
1899     %% from UDP(c) to dTTP(n)
1900     model = modelOri;
1901     model.c(find(model.c)) = 0;
1902     [model] = addSinkReactions(model,{'udp(c)','dttp(n)'},[-1 -1; 0 100]);
1903     if (rxnsInModel(2) >-1) % reaction exits already in model
1904         model=changeObjective(model,model.rxns(rxnsInModel(2),1));
1905     else
1906         model=changeObjective(model,'sink_dttp(n)',1);
1907     end
1908     if find(model.c)>0
1909         FBA = optimizeCbModel(model,'max');
1910         TestSolution(k,1) = FBA.f;
1911     else
1912         TestSolution(k,1) = NaN;
1913     end
1914     TestSolutionName{k,1} = 'udp(c) -> dttp(n)';
1915     if ~isnan(TestSolution(k,1)); TestedRxns = [TestedRxns; model.rxns(find(abs(FBA.x)>tol))]; end ;k = k +1;clear FBA
1916     %% from ump to ala-B
1917     model = modelOri;
1918     model.c(find(model.c)) = 0;
1919     [model] = addSinkReactions(model,{'ump(c)','ala-B(c)'},[-1 -1; 0 100]);
1920     model.c(ismember(model.rxns,'sink_ala-B(c)'))=1;
1921     if find(model.c)>0
1922         FBA = optimizeCbModel(model,'max');
1923         TestSolution(k,1) = FBA.f;
1924     else
1925         TestSolution(k,1) = NaN;
1926     end
1927     TestSolutionName{k,1} = 'ump(c) -> ala-B(c)';
1928     if ~isnan(TestSolution(k,1)); TestedRxns = [TestedRxns; model.rxns(find(abs(FBA.x)>tol))]; end ;k = k +1;clear FBA
1929     %% fru -> dhap
1930     model = modelOri;
1931     model.c(find(model.c)) = 0;
1932     [model] = addSinkReactions(model,{'fru(c)','dhap(c)'},[-1 -1; 0 100]);
1933     model.c(ismember(model.rxns,'sink_dhap(c)'))=1;
1934     if find(model.c)>0
1935         FBA = optimizeCbModel(model,'max');
1936         TestSolution(k,1) = FBA.f;
1937     else
1938         TestSolution(k,1) = NaN;
1939     end
1940     TestSolutionName{k,1} = 'fru(c) -> dhap(c)';
1941     if ~isnan(TestSolution(k,1)); TestedRxns = [TestedRxns; model.rxns(find(abs(FBA.x)>tol))]; end ;k = k +1;clear FBA
1942     %% fru -> g3p
1943     model = modelOri;
1944     model.c(find(model.c)) = 0;
1945     [model] = addSinkReactions(model,{'fru(c)','g3p(c)'},[-1 -1; 0 100]);
1946     model.c(ismember(model.rxns,'sink_g3p(c)'))=1;
1947     if find(model.c)>0
1948         FBA = optimizeCbModel(model,'max');
1949         TestSolution(k,1) = FBA.f;
1950     else
1951         TestSolution(k,1) = NaN;
1952     end
1953     TestSolutionName{k,1} = 'fru(c) -> g3p(c)';
1954     if ~isnan(TestSolution(k,1)); TestedRxns = [TestedRxns; model.rxns(find(abs(FBA.x)>tol))]; end ;k = k +1;clear FBA
1955     %% fuc -> gdpfuc
1956     model = modelOri;
1957     model.c(find(model.c)) = 0;
1958     
1959     for i = 1 : length(RPMI_composition)
1960         model = changeRxnBounds(model,RPMI_composition{i},-1,'l');
1961     end
1962     [model] = addSinkReactions(model,{'fuc-L(c)','gdpfuc(c)'},[-1 -1; 0 100]);
1963     model.c(ismember(model.rxns,'sink_gdpfuc(c)'))=1;
1964     if find(model.c)>0
1965         FBA = optimizeCbModel(model,'max');
1966         TestSolution(k,1) = FBA.f;
1967     else
1968         TestSolution(k,1) = NaN;
1969     end
1970     TestSolutionName{k,1} = 'fuc-L(c) -> gdpfuc(c) (with RPMI medium)';
1971     if ~isnan(TestSolution(k,1)); TestedRxns = [TestedRxns; model.rxns(find(abs(FBA.x)>tol))]; end ;k = k +1;clear FBA
1972     %% fum[m] -> oaa[m]
1973     model = modelOri;
1974     model.c(find(model.c)) = 0;
1975     [model] = addSinkReactions(model,{'fum(m)','oaa(m)'},[-1 -1; 0 100]);
1976     model.c(ismember(model.rxns,'sink_oaa(m)'))=1;
1977     if find(model.c)>0
1978         FBA = optimizeCbModel(model,'max');
1979         TestSolution(k,1) = FBA.f;
1980     else
1981         TestSolution(k,1) = NaN;
1982     end
1983     TestSolutionName{k,1} = 'fum(m) -> oaa(m)';
1984     if ~isnan(TestSolution(k,1)); TestedRxns = [TestedRxns; model.rxns(find(abs(FBA.x)>tol))]; end ;k = k +1;clear FBA
1985     %% g1p -> dtdprmn
1986     model = modelOri;
1987     model.c(find(model.c)) = 0;
1988     for i = 1 : length(RPMI_composition)
1989         model = changeRxnBounds(model,RPMI_composition{i},-1,'l');
1990     end
1991     [model] = addSinkReactions(model,{'g1p(c)','dtdprmn(c)'},[-1 -1; 0 100]);
1992     model.c(ismember(model.rxns,'sink_dtdprmn(c)'))=1;
1993     if find(model.c)>0
1994         FBA = optimizeCbModel(model,'max');
1995         TestSolution(k,1) = FBA.f;
1996     else
1997         TestSolution(k,1) = NaN;
1998     end
1999     TestSolutionName{k,1} = 'g1p(c) -> dtdprmn(c) (with RPMI medium)';
2000     if ~isnan(TestSolution(k,1)); TestedRxns = [TestedRxns; model.rxns(find(abs(FBA.x)>tol))]; end ;k = k +1;clear FBA
2001     %% g3p -> mthgxl
2002     model = modelOri;
2003     model.c(find(model.c)) = 0;
2004     [model] = addSinkReactions(model,{'g3p(c)','mthgxl(c)'},[-1 -1; 0 100]);
2005     model.c(ismember(model.rxns,'sink_mthgxl(c)'))=1;
2006     if find(model.c)>0
2007         FBA = optimizeCbModel(model,'max');
2008         TestSolution(k,1) = FBA.f;
2009     else
2010         TestSolution(k,1) = NaN;
2011     end
2012     TestSolutionName{k,1} = 'g3p(c) -> mthgxl(c)';
2013     if ~isnan(TestSolution(k,1)); TestedRxns = [TestedRxns; model.rxns(find(abs(FBA.x)>tol))]; end ;k = k +1;clear FBA
2014     %% g6p -> r5p
2015     model = modelOri;
2016     model.c(find(model.c)) = 0;
2017     [model] = addSinkReactions(model,{'g6p(c)','r5p(c)'},[-1 -1; 0 100]);
2018     model.c(ismember(model.rxns,'sink_r5p(c)'))=1;
2019     if find(model.c)>0
2020         FBA = optimizeCbModel(model,'max');
2021         TestSolution(k,1) = FBA.f;
2022     else
2023         TestSolution(k,1) = NaN;
2024     end
2025     TestSolutionName{k,1} = 'g6p(c) -> r5p(c)';
2026     if ~isnan(TestSolution(k,1)); TestedRxns = [TestedRxns; model.rxns(find(abs(FBA.x)>tol))]; end ;k = k +1;clear FBA
2027     %% g6p -> ru5p
2028     model = modelOri;
2029     model.c(find(model.c)) = 0;
2030     [model] = addSinkReactions(model,{'g6p(c)','ru5p-D(c)'},[-1 -1; 0 100]);
2031     model.c(ismember(model.rxns,'sink_ru5p-D(c)'))=1;
2032     if find(model.c)>0
2033         FBA = optimizeCbModel(model,'max');
2034         TestSolution(k,1) = FBA.f;
2035     else
2036         TestSolution(k,1) = NaN;
2037     end
2038     TestSolutionName{k,1} = 'g6p(c) -> ru5p-D(c)';
2039     if ~isnan(TestSolution(k,1)); TestedRxns = [TestedRxns; model.rxns(find(abs(FBA.x)>tol))]; end ;k = k +1;clear FBA
2040     %% gal -> glc
2041     model = modelOri;
2042     model.c(find(model.c)) = 0;
2043     [model] = addSinkReactions(model,{'gal(c)','glc-D(c)'},[-1 -1; 0 100]);
2044     model.c(ismember(model.rxns,'sink_glc-D(c)'))=1;
2045     if find(model.c)>0
2046         FBA = optimizeCbModel(model,'max');
2047         TestSolution(k,1) = FBA.f;
2048     else
2049         TestSolution(k,1) = NaN;
2050     end
2051     TestSolutionName{k,1} = 'gal(c) -> glc-D(c)';
2052     if ~isnan(TestSolution(k,1)); TestedRxns = [TestedRxns; model.rxns(find(abs(FBA.x)>tol))]; end ;k = k +1;clear FBA
2053     %% gal -> udpgal
2054     model = modelOri;
2055     model.c(find(model.c)) = 0;
2056     [model] = addSinkReactions(model,{'gal(c)','udpgal(c)'},[-1 -1; 0 100]);
2057     model.c(ismember(model.rxns,'sink_udpgal(c)'))=1;
2058     if find(model.c)>0
2059         FBA = optimizeCbModel(model,'max');
2060         TestSolution(k,1) = FBA.f;
2061     else
2062         TestSolution(k,1) = NaN;
2063     end
2064     TestSolutionName{k,1} = 'gal(c) -> udpgal(c)';
2065     if ~isnan(TestSolution(k,1)); TestedRxns = [TestedRxns; model.rxns(find(abs(FBA.x)>tol))]; end ;k = k +1;clear FBA
2066     %% galgluside(g) -> galgalgalthcrm_hs(g)
2067     model = modelOri;
2068     model.c(find(model.c)) = 0;
2069     [model] = addSinkReactions(model,{'galgluside_hs(g)','galgalgalthcrm_hs(g)'},[-1 -1; 0 100]);
2070     model.c(ismember(model.rxns,'sink_galgalgalthcrm_hs(g)'))=1;
2071     if find(model.c)>0
2072         FBA = optimizeCbModel(model,'max');
2073         TestSolution(k,1) = FBA.f;
2074     else
2075         TestSolution(k,1) = NaN;
2076     end
2077     TestSolutionName{k,1} = 'galgluside_hs(g) -> galgalgalthcrm_hs(g)';
2078     if ~isnan(TestSolution(k,1)); TestedRxns = [TestedRxns; model.rxns(find(abs(FBA.x)>tol))]; end ;k = k +1;clear FBA
2079     %% galgluside_hs(g) -> acgagbside_hs(g)
2080     model = modelOri;
2081     model.c(find(model.c)) = 0;
2082     [model] = addSinkReactions(model,{'galgluside_hs(g)','acgagbside_hs(g)'},[-1 -1; 0 100]);
2083     model.c(ismember(model.rxns,'sink_acgagbside_hs(g)'))=1;
2084     if find(model.c)>0
2085         FBA = optimizeCbModel(model,'max');
2086         TestSolution(k,1) = FBA.f;
2087     else
2088         TestSolution(k,1) = NaN;
2089     end
2090     TestSolutionName{k,1} = 'galgluside_hs(g) -> acgagbside_hs(g)';
2091     if ~isnan(TestSolution(k,1)); TestedRxns = [TestedRxns; model.rxns(find(abs(FBA.x)>tol))]; end ;k = k +1;clear FBA
2092     %% galgluside_hs(g) -> acnacngalgbside_hs(g)
2093     model = modelOri;
2094     model.c(find(model.c)) = 0;
2095     [model] = addSinkReactions(model,{'galgluside_hs(g)','acnacngalgbside_hs(g)'},[-1 -1; 0 100]);
2096     model.c(ismember(model.rxns,'sink_acnacngalgbside_hs(g)'))=1;
2097     if find(model.c)>0
2098         FBA = optimizeCbModel(model,'max');
2099         TestSolution(k,1) = FBA.f;
2100     else
2101         TestSolution(k,1) = NaN;
2102     end
2103     TestSolutionName{k,1} = 'galgluside_hs(g) -> acnacngalgbside_hs(g)';
2104     if ~isnan(TestSolution(k,1)); TestedRxns = [TestedRxns; model.rxns(find(abs(FBA.x)>tol))]; end ;k = k +1;clear FBA
2105     %% galgluside_hs(g) -> gd1b2_hs(g)
2106     model = modelOri;
2107     model.c(find(model.c)) = 0;
2108     [model] = addSinkReactions(model,{'galgluside_hs(g)','gd1b2_hs(g)'},[-1 -1; 0 100]);
2109     model.c(ismember(model.rxns,'sink_gd1b2_hs(g)'))=1;
2110     if find(model.c)>0
2111         FBA = optimizeCbModel(model,'max');
2112         TestSolution(k,1) = FBA.f;
2113     else
2114         TestSolution(k,1) = NaN;
2115     end
2116     TestSolutionName{k,1} = 'galgluside_hs(g) -> gd1b2_hs(g)';
2117     if ~isnan(TestSolution(k,1)); TestedRxns = [TestedRxns; model.rxns(find(abs(FBA.x)>tol))]; end ;k = k +1;clear FBA
2118     %% galgluside_hs(g) -> gd1c_hs(g)
2119     model = modelOri;
2120     model.c(find(model.c)) = 0;
2121     [model] = addSinkReactions(model,{'galgluside_hs(g)','gd1c_hs(g)'},[-1 -1; 0 100]);
2122     model.c(ismember(model.rxns,'sink_gd1c_hs(g)'))=1;
2123     if find(model.c)>0
2124         FBA = optimizeCbModel(model,'max');
2125         TestSolution(k,1) = FBA.f;
2126     else
2127         TestSolution(k,1) = NaN;
2128     end
2129     TestSolutionName{k,1} = 'galgluside_hs(g) -> gd1c_hs(g)';
2130     if ~isnan(TestSolution(k,1)); TestedRxns = [TestedRxns; model.rxns(find(abs(FBA.x)>tol))]; end ;k = k +1;clear FBA
2131     %% galgluside_hs(g) -> gp1c_hs(g)
2132     model = modelOri;
2133     model.c(find(model.c)) = 0;
2134     [model] = addSinkReactions(model,{'galgluside_hs(g)','gp1c_hs(g)'},[-1 -1; 0 100]);
2135     model.c(ismember(model.rxns,'sink_gp1c_hs(g)'))=1;
2136     if find(model.c)>0
2137         FBA = optimizeCbModel(model,'max');
2138         TestSolution(k,1) = FBA.f;
2139     else
2140         TestSolution(k,1) = NaN;
2141     end
2142     TestSolutionName{k,1} = 'galgluside_hs(g) -> gp1c_hs(g)';
2143     if ~isnan(TestSolution(k,1)); TestedRxns = [TestedRxns; model.rxns(find(abs(FBA.x)>tol))]; end ;k = k +1;clear FBA
2144     %% galgluside_hs(g) -> gq1balpha_hs(g)
2145     model = modelOri;
2146     model.c(find(model.c)) = 0;
2147     [model] = addSinkReactions(model,{'galgluside_hs(g)','gq1balpha_hs(g)'},[-1 -1; 0 100]);
2148     model.c(ismember(model.rxns,'sink_gq1balpha_hs(g)'))=1;
2149     if find(model.c)>0
2150         FBA = optimizeCbModel(model,'max');
2151         TestSolution(k,1) = FBA.f;
2152     else
2153         TestSolution(k,1) = NaN;
2154     end
2155     TestSolutionName{k,1} = 'galgluside_hs(g) -> gq1balpha_hs(g)';
2156     if ~isnan(TestSolution(k,1)); TestedRxns = [TestedRxns; model.rxns(find(abs(FBA.x)>tol))]; end ;k = k +1;clear FBA
2157     %% gam6p -> uacgam
2158     model = modelOri;
2159     model.c(find(model.c)) = 0;
2160     [model] = addSinkReactions(model,{'gam6p(c)','uacgam(c)'},[-1 -1; 0 100]);
2161     model.c(ismember(model.rxns,'sink_uacgam(c)'))=1;
2162     if find(model.c)>0
2163         FBA = optimizeCbModel(model,'max');
2164         TestSolution(k,1) = FBA.f;
2165     else
2166         TestSolution(k,1) = NaN;
2167     end
2168     TestSolutionName{k,1} = 'gam6p(c) -> uacgam(c)';
2169     if ~isnan(TestSolution(k,1)); TestedRxns = [TestedRxns; model.rxns(find(abs(FBA.x)>tol))]; end ;k = k +1;clear FBA
2170     %% gdpmann -> gdpfuc
2171     model = modelOri;
2172     model.c(find(model.c)) = 0;
2173     model.lb(ismember(model.rxns,'EX_glc(e)'))=-1;model.ub(ismember(model.rxns,'EX_glc(e)'))=0;
2174     model.lb(ismember(model.rxns,'EX_o2(e)'))=-40;model.ub(ismember(model.rxns,'EX_o2(e)'))=-1;
2175     [model] = addSinkReactions(model,{'gdpmann(c)','gdpfuc(c)'},[-1 -1; 0 100]);
2176     model.c(ismember(model.rxns,'sink_gdpfuc(c)'))=1;
2177     if find(model.c)>0
2178         FBA = optimizeCbModel(model,'max');
2179         TestSolution(k,1) = FBA.f;
2180     else
2181         TestSolution(k,1) = NaN;
2182     end
2183     TestSolutionName{k,1} = 'gdpmann(c) -> gdpfuc(c)';
2184     if ~isnan(TestSolution(k,1)); TestedRxns = [TestedRxns; model.rxns(find(abs(FBA.x)>tol))]; end ;k = k +1;clear FBA
2185     %% glc -> inost
2186     model = modelOri;
2187     model.c(find(model.c)) = 0;
2188     [model] = addSinkReactions(model,{'glc-D(c)','inost(c)'},[-1 -1; 0 100]);
2189     model.c(ismember(model.rxns,'sink_inost(c)'))=1;
2190     if find(model.c)>0
2191         FBA = optimizeCbModel(model,'max');
2192         TestSolution(k,1) = FBA.f;
2193     else
2194         TestSolution(k,1) = NaN;
2195     end
2196     TestSolutionName{k,1} = 'glc-D(c) -> inost(c)';
2197     if ~isnan(TestSolution(k,1)); TestedRxns = [TestedRxns; model.rxns(find(abs(FBA.x)>tol))]; end ;k = k +1;clear FBA
2198     %% glc -> lac + atp + h2o % I assumed lac-L
2199     model = modelOri;
2200     model.c(find(model.c)) = 0;
2201     [model] = addSinkReactions(model,{'glc-D(c)','lac-L(c)','atp(c)','h2o(c)'},[-1 -1; 0.1 100; 0.1 100; 0.1 100]);
2202     model.c(ismember(model.rxns,'sink_lac-L(c)'))=1;
2203     if find(model.c)>0
2204         FBA = optimizeCbModel(model,'max');
2205         TestSolution(k,1) = FBA.f;
2206     else
2207         TestSolution(k,1) = NaN;
2208     end
2209     TestSolutionName{k,1} = 'glc-D(c) -> lac-L(c) + atp(c) + h2o(c)';
2210     if ~isnan(TestSolution(k,1)); TestedRxns = [TestedRxns; model.rxns(find(abs(FBA.x)>tol))]; end ;k = k +1;clear FBA
2211     %% glc -> lac-D
2212     model = modelOri;
2213     model.c(find(model.c)) = 0;
2214     [model] = addSinkReactions(model,{'glc-D(c)','lac-D(c)'},[-1 -1; 0 100]);
2215     model.c(ismember(model.rxns,'sink_lac-D(c)'))=1;
2216     if find(model.c)>0
2217         FBA = optimizeCbModel(model,'max');
2218         TestSolution(k,1) = FBA.f;
2219     else
2220         TestSolution(k,1) = NaN;
2221     end
2222     TestSolutionName{k,1} = 'glc-D(c) -> lac-D(c)';
2223     if ~isnan(TestSolution(k,1)); TestedRxns = [TestedRxns; model.rxns(find(abs(FBA.x)>tol))]; end ;k = k +1;clear FBA
2224     %% glc -> lcts[g] (2)
2225     model = modelOri;
2226     model.c(find(model.c)) = 0;
2227     [model] = addSinkReactions(model,{'glc-D(c)','lcts(g)'},[-1 -1; 0 100]);
2228     model.c(ismember(model.rxns,'sink_lcts(g)'))=1;
2229     if find(model.c)>0
2230         FBA = optimizeCbModel(model,'max');
2231         TestSolution(k,1) = FBA.f;
2232     else
2233         TestSolution(k,1) = NaN;
2234     end
2235     TestSolutionName{k,1} = 'glc-D(c) -> lcts(g)';
2236     if ~isnan(TestSolution(k,1)); TestedRxns = [TestedRxns; model.rxns(find(abs(FBA.x)>tol))]; end ;k = k +1;clear FBA
2237     %% glc -> pyr
2238     model = modelOri;
2239     model.c(find(model.c)) = 0;
2240     [model] = addSinkReactions(model,{'glc-D(c)','pyr(c)'},[-1 -1; 0 100]);
2241     model.c(ismember(model.rxns,'sink_pyr(c)'))=1;
2242     if find(model.c)>0
2243         FBA = optimizeCbModel(model,'max');
2244         TestSolution(k,1) = FBA.f;
2245     else
2246         TestSolution(k,1) = NaN;
2247     end
2248     TestSolutionName{k,1} = 'glc-D(c) -> pyr(c)';
2249     if ~isnan(TestSolution(k,1)); TestedRxns = [TestedRxns; model.rxns(find(abs(FBA.x)>tol))]; end ;k = k +1;clear FBA
2250     %% gln -> nh4
2251     model = modelOri;
2252     model.c(find(model.c)) = 0;
2253     [model] = addSinkReactions(model,{'gln-L(c)','nh4(c)'},[-1 -1; 0 100]);
2254     model.lb(ismember(model.rxns,'EX_nh4(e)'))=0;model.ub(ismember(model.rxns,'EX_nh4(e)'))=1000;
2255     model.c(ismember(model.rxns,'sink_nh4(c)'))=1;
2256     if find(model.c)>0
2257         FBA = optimizeCbModel(model,'max');
2258         TestSolution(k,1) = FBA.f;
2259     else
2260         TestSolution(k,1) = NaN;
2261     end
2262     TestSolutionName{k,1} = 'gln-L(c) -> nh4(c)';
2263     if ~isnan(TestSolution(k,1)); TestedRxns = [TestedRxns; model.rxns(find(abs(FBA.x)>tol))]; end ;k = k +1;clear FBA
2264     %% gln-L(m) -> glu-L(m)
2265     model = modelOri;
2266     model.c(find(model.c)) = 0;
2267     [model] = addSinkReactions(model,{'gln-L(m)','glu-L(m)'},[-1 -1; 0 100]);
2268     model.c(ismember(model.rxns,'sink_glu-L(m)'))=1;
2269     if find(model.c)>0
2270         FBA = optimizeCbModel(model,'max');
2271         TestSolution(k,1) = FBA.f;
2272     else
2273         TestSolution(k,1) = NaN;
2274     end
2275     TestSolutionName{k,1} = 'gln-L(m) -> glu-L(m)';
2276     if ~isnan(TestSolution(k,1)); TestedRxns = [TestedRxns; model.rxns(find(abs(FBA.x)>tol))]; end ;k = k +1;clear FBA
2277     %% gln-L[m] -> glu-L[m]
2278     model = modelOri;
2279     model.c(find(model.c)) = 0;
2280     [model] = addSinkReactions(model,{'gln-L(m)','glu-L(m)'},[-1 -1; 0 100]);
2281     model.c(ismember(model.rxns,'sink_glu-L(m)'))=1;
2282     if find(model.c)>0
2283         FBA = optimizeCbModel(model,'max');
2284         TestSolution(k,1) = FBA.f;
2285     else
2286         TestSolution(k,1) = NaN;
2287     end
2288     TestSolutionName{k,1} = 'gln-L(m) -> glu-L(m)';
2289     if ~isnan(TestSolution(k,1)); TestedRxns = [TestedRxns; model.rxns(find(abs(FBA.x)>tol))]; end ;k = k +1;clear FBA
2290     %% glu5sa -> pro-L
2291     model = modelOri;
2292     model.c(find(model.c)) = 0;
2293     [model] = addSinkReactions(model,{'glu5sa(c)','pro-L(c)'},[-1 -1; 0 100]);
2294     model.c(ismember(model.rxns,'sink_pro-L(c)'))=1;
2295     if find(model.c)>0
2296         FBA = optimizeCbModel(model,'max');
2297         TestSolution(k,1) = FBA.f;
2298     else
2299         TestSolution(k,1) = NaN;
2300     end
2301     TestSolutionName{k,1} = 'glu5sa(c) -> pro-L(c)';
2302     if ~isnan(TestSolution(k,1)); TestedRxns = [TestedRxns; model.rxns(find(abs(FBA.x)>tol))]; end ;k = k +1;clear FBA
2303     %% glu-L -> 4abut
2304     model = modelOri;
2305     model.c(find(model.c)) = 0;
2306     [model] = addSinkReactions(model,{'glu-L(c)','4abut(c)'},[-1 -1; 0 100]);
2307     model.c(ismember(model.rxns,'sink_4abut(c)'))=1;
2308     if find(model.c)>0
2309         FBA = optimizeCbModel(model,'max');
2310         TestSolution(k,1) = FBA.f;
2311     else
2312         TestSolution(k,1) = NaN;
2313     end
2314     TestSolutionName{k,1} = 'glu-L(c) -> 4abut(c)';
2315     if ~isnan(TestSolution(k,1)); TestedRxns = [TestedRxns; model.rxns(find(abs(FBA.x)>tol))]; end ;k = k +1;clear FBA
2316     %% glu-L -> gln-L[c] %I adjusted lb since otw infeasible
2317     model = modelOri;
2318     model.c(find(model.c)) = 0;
2319     [model] = addSinkReactions(model,{'glu-L(c)','gln-L(c)'},[-1 -1; 0 100]);
2320     model.c(ismember(model.rxns,'sink_gln-L(c)'))=1;
2321     if find(model.c)>0
2322         FBA = optimizeCbModel(model,'max');
2323         TestSolution(k,1) = FBA.f;
2324     else
2325         TestSolution(k,1) = NaN;
2326     end
2327     TestSolutionName{k,1} = 'glu-L(c) -> gln-L(c)';
2328     if ~isnan(TestSolution(k,1)); TestedRxns = [TestedRxns; model.rxns(find(abs(FBA.x)>tol))]; end ;k = k +1;clear FBA
2329     %% glu-L -> pro-L
2330     model = modelOri;
2331     model.c(find(model.c)) = 0;
2332     [model] = addSinkReactions(model,{'glu-L(c)','pro-L(c)'},[-1 -1; 0 100]);
2333     model.c(ismember(model.rxns,'sink_pro-L(c)'))=1;
2334     if find(model.c)>0
2335         FBA = optimizeCbModel(model,'max');
2336         TestSolution(k,1) = FBA.f;
2337     else
2338         TestSolution(k,1) = NaN;
2339     end
2340     TestSolutionName{k,1} = 'glu-L -> pro-L';
2341     if ~isnan(TestSolution(k,1)); TestedRxns = [TestedRxns; model.rxns(find(abs(FBA.x)>tol))]; end ;k = k +1;clear FBA
2342     %% glu-L(m) -> akg(m)
2343     model = modelOri;
2344     model.c(find(model.c)) = 0;
2345     [model] = addSinkReactions(model,{'glu-L(m)','akg(m)'},[-1 -1; 0 100]);
2346     model.c(ismember(model.rxns,'sink_akg(m)'))=1;
2347     if find(model.c)>0
2348         FBA = optimizeCbModel(model,'max');
2349         TestSolution(k,1) = FBA.f;
2350     else
2351         TestSolution(k,1) = NaN;
2352     end
2353     TestSolutionName{k,1} = 'glu-L(m) -> akg(m)';
2354     if ~isnan(TestSolution(k,1)); TestedRxns = [TestedRxns; model.rxns(find(abs(FBA.x)>tol))]; end ;k = k +1;clear FBA
2355     %% gluside_hs(g) -> galgluside_hs(g)
2356     model = modelOri;
2357     model.c(find(model.c)) = 0;
2358     [model] = addSinkReactions(model,{'gluside_hs(g)','galgluside_hs(g)'},[-1 -1; 0 100]);
2359     model.c(ismember(model.rxns,'sink_galgluside_hs(g)'))=1;
2360     if find(model.c)>0
2361         FBA = optimizeCbModel(model,'max');
2362         TestSolution(k,1) = FBA.f;
2363     else
2364         TestSolution(k,1) = NaN;
2365     end
2366     TestSolutionName{k,1} = 'gluside_hs(g) -> galgluside_hs(g)';
2367     if ~isnan(TestSolution(k,1)); TestedRxns = [TestedRxns; model.rxns(find(abs(FBA.x)>tol))]; end ;k = k +1;clear FBA
2368     %% glx[m] -> glyclt[m]
2369     model = modelOri;
2370     model.c(find(model.c)) = 0;
2371     [model] = addSinkReactions(model,{'glx(m)','glyclt(m)'},[-1 -1; 0 100]);
2372     model.c(ismember(model.rxns,'sink_glyclt(m)'))=1;
2373     if find(model.c)>0
2374         FBA = optimizeCbModel(model,'max');
2375         TestSolution(k,1) = FBA.f;
2376     else
2377         TestSolution(k,1) = NaN;
2378     end
2379     TestSolutionName{k,1} = 'glx(m) -> glyclt(m)';
2380     if ~isnan(TestSolution(k,1)); TestedRxns = [TestedRxns; model.rxns(find(abs(FBA.x)>tol))]; end ;k = k +1;clear FBA
2381     %% gly -> ser-L -> pyr (via SERD_L) % SERD_L does not exist in human
2382     % (L-serine deaminase)
2383     model = modelOri;
2384     model.c(find(model.c)) = 0;
2385     [model] = addSinkReactions(model,{'gly(c)','ser-L(c)'},[-1 -1; 0 100]);
2386     model.c(ismember(model.rxns,'sink_ser-L(c)'))=1;
2387     if find(model.c)>0
2388         FBA = optimizeCbModel(model,'max');
2389         TestSolution(k,1) = FBA.f;
2390     else
2391         TestSolution(k,1) = NaN;
2392     end
2393     TestSolutionName{k,1} = 'gly(c) -> ser-L(c) -> pyr(c), 1';
2394     if ~isnan(TestSolution(k,1)); TestedRxns = [TestedRxns; model.rxns(find(abs(FBA.x)>tol))]; end ;k = k +1;clear FBA
2395     model = modelOri;
2396     model.c(find(model.c)) = 0;
2397     [model] = addSinkReactions(model,{'ser-L(c)','pyr(c)'},[-1 -1; 0 100]);
2398     model.c(ismember(model.rxns,'sink_pyr(c)'))=1;
2399     if find(model.c)>0
2400         FBA = optimizeCbModel(model,'max');
2401         TestSolution(k,1) = FBA.f;
2402     else
2403         TestSolution(k,1) = NaN;
2404     end
2405     TestSolutionName{k,1} = 'gly(c) -> ser-L(c) -> pyr(c), 2';
2406     if ~isnan(TestSolution(k,1)); TestedRxns = [TestedRxns; model.rxns(find(abs(FBA.x)>tol))]; end ;k = k +1;clear FBA
2407     %% glyc -> glc
2408     model = modelOri;
2409     model.c(find(model.c)) = 0;
2410     [model] = addSinkReactions(model,{'glyc(c)','glc-D(c)'},[-1 -1; 0 100]);
2411     model.c(ismember(model.rxns,'sink_glc-D(c)'))=1;
2412     if find(model.c)>0
2413         FBA = optimizeCbModel(model,'max');
2414         TestSolution(k,1) = FBA.f;
2415     else
2416         TestSolution(k,1) = NaN;
2417     end
2418     TestSolutionName{k,1} = 'glyc(c) -> glc-D(c)';
2419     if ~isnan(TestSolution(k,1)); TestedRxns = [TestedRxns; model.rxns(find(abs(FBA.x)>tol))]; end ;k = k +1;clear FBA
2420     %% glyc(c) + Rtotal(c) + Rtotal2(c) -> dag_hs(c)
2421     model = modelOri;
2422     model.c(find(model.c)) = 0;
2423     [model] = addSinkReactions(model,{'glyc(c)','Rtotal(c)','Rtotal2(c)','dag_hs(c)'},[-1 -1;-1 -1;-1 -1; 0 100]);
2424     model.c(ismember(model.rxns,'sink_dag_hs(c)'))=1;
2425     if find(model.c)>0
2426         FBA = optimizeCbModel(model,'max');
2427         TestSolution(k,1) = FBA.f;
2428     else
2429         TestSolution(k,1) = NaN;
2430     end
2431     TestSolutionName{k,1} = 'glyc(c) + Rtotal(c) + Rtotal2(c) -> dag_hs(c)';
2432     if ~isnan(TestSolution(k,1)); TestedRxns = [TestedRxns; model.rxns(find(abs(FBA.x)>tol))]; end ;k = k +1;clear FBA
2433     %% glyc(c) + Rtotal(c) -> tag_hs(c)
2434     model = modelOri;
2435     model.c(find(model.c)) = 0;
2436     for i = 1 : length(RPMI_composition)
2437         model = changeRxnBounds(model,RPMI_composition{i},-1,'l');
2438     end
2439     [model] = addSinkReactions(model,{'glyc(c)','Rtotal(c)','tag_hs(c)'},[-1 -1;-1 -1; 0 100]);
2440     model.c(ismember(model.rxns,'sink_tag_hs(c)'))=1;
2441     if find(model.c)>0
2442         FBA = optimizeCbModel(model,'max');
2443         TestSolution(k,1) = FBA.f;
2444     else
2445         TestSolution(k,1) = NaN;
2446     end
2447     TestSolutionName{k,1} = 'glyc(c) + Rtotal(c) -> tag_hs(c) (with RPMI medium)';
2448     if ~isnan(TestSolution(k,1)); TestedRxns = [TestedRxns; model.rxns(find(abs(FBA.x)>tol))]; end ;k = k +1;clear FBA
2449     %% glyclt -> gly
2450     model = modelOri;
2451     model.c(find(model.c)) = 0;
2452     [model] = addSinkReactions(model,{'glyclt(c)','gly(c)'},[-1 -1; 0 100]);
2453     model.c(ismember(model.rxns,'sink_gly(c)'))=1;
2454     if find(model.c)>0
2455         FBA = optimizeCbModel(model,'max');
2456         TestSolution(k,1) = FBA.f;
2457     else
2458         TestSolution(k,1) = NaN;
2459     end
2460     TestSolutionName{k,1} = 'glyclt(c) -> gly(c)';
2461     if ~isnan(TestSolution(k,1)); TestedRxns = [TestedRxns; model.rxns(find(abs(FBA.x)>tol))]; end ;k = k +1;clear FBA
2462     %% glygn2 -> glc % changing lb has no effect
2463     model = modelOri;
2464     model.c(find(model.c)) = 0;
2465     [model] = addSinkReactions(model,{'glygn2(c)','glc-D(c)'},[-1 -1; 0 100]);
2466     model.c(ismember(model.rxns,'sink_glc-D(c)'))=1;
2467     if find(model.c)>0
2468         FBA = optimizeCbModel(model,'max');
2469         TestSolution(k,1) = FBA.f;
2470     else
2471         TestSolution(k,1) = NaN;
2472     end
2473     TestSolutionName{k,1} = 'glygn2(c) -> glc-D(c)';
2474     if ~isnan(TestSolution(k,1)); TestedRxns = [TestedRxns; model.rxns(find(abs(FBA.x)>tol))]; end ;k = k +1;clear FBA
2475     %% glygn2[e] -> glc[e]
2476     model = modelOri;
2477     model.c(find(model.c)) = 0;
2478     [model] = addSinkReactions(model,{'glygn2(e)','glc-D(e)'},[-1 -1; 0 100]);
2479     %model.c(ismember(model.rxns,'sink_glc-D(e)'))=1;
2480     if ~isempty(strmatch('AMY2e',model.rxns,'exact'))
2481         model = changeObjective(model,'AMY2e',1);
2482         FBA = optimizeCbModel(model,'max');
2483         TestSolution(k,1) = FBA.f;
2484     else
2485         TestSolution(k,1) = NaN;
2486     end
2487     TestSolutionName{k,1} = 'glygn2(e) -> glc-D(e) - via AMY2e';
2488     if ~isnan(TestSolution(k,1)); TestedRxns = [TestedRxns; model.rxns(find(abs(FBA.x)>tol))]; end ;k = k +1;clear FBA
2489     %% glyx -> oxa % I assumed glx
2490     model = modelOri;
2491     model.c(find(model.c)) = 0;
2492     [model] = addSinkReactions(model,{'glx(c)','oxa(c)'},[-1 -1; 0 100]);
2493     model.c(ismember(model.rxns,'sink_oxa(c)'))=1;
2494     if find(model.c)>0
2495         FBA = optimizeCbModel(model,'max');
2496         TestSolution(k,1) = FBA.f;
2497     else
2498         TestSolution(k,1) = NaN;
2499     end
2500     TestSolutionName{k,1} = 'glx(c) -> oxa(c)';
2501     if ~isnan(TestSolution(k,1)); TestedRxns = [TestedRxns; model.rxns(find(abs(FBA.x)>tol))]; end ;k = k +1;clear FBA
2502     %% ha[l] -> acgam[l] + glcur[l]
2503     model = modelOri;
2504     model.c(find(model.c)) = 0;
2505     [model] = addSinkReactions(model,{'ha(l)','acgam(l)','glcur(l)'},[-1 -1; 0.1 100; 0.1 100]);
2506     model.c(ismember(model.rxns,'sink_acgam(l)'))=1;
2507     if find(model.c)>0
2508         FBA = optimizeCbModel(model,'max');
2509         TestSolution(k,1) = FBA.f;
2510     else
2511         TestSolution(k,1) = NaN;
2512     end
2513     TestSolutionName{k,1} = 'ha[l] -> acgam[l] + glcur[l]';
2514     if ~isnan(TestSolution(k,1)); TestedRxns = [TestedRxns; model.rxns(find(abs(FBA.x)>tol))]; end ;k = k +1;clear FBA
2515     %% His -> glu-L
2516     model = modelOri;
2517     model.c(find(model.c)) = 0;
2518     [model] = addSinkReactions(model,{'his-L(c)','glu-L(c)'},[-1 -1; 0 100]);
2519     model.c(ismember(model.rxns,'sink_glu-L(c)'))=1;
2520     if find(model.c)>0
2521         FBA = optimizeCbModel(model,'max');
2522         TestSolution(k,1) = FBA.f;
2523     else
2524         TestSolution(k,1) = NaN;
2525     end
2526     TestSolutionName{k,1} = 'his-L(c) -> glu-L(c)';
2527     if ~isnan(TestSolution(k,1)); TestedRxns = [TestedRxns; model.rxns(find(abs(FBA.x)>tol))]; end ;k = k +1;clear FBA
2528     %% his-L -> hista
2529     model = modelOri;
2530     model.c(find(model.c)) = 0;
2531     [model] = addSinkReactions(model,{'his-L(c)','hista(c)'},[-1 -1; 0 100]);
2532     model.lb(find(ismember(model.rxns,'sink_his_L(c)')))=-1;
2533     model.ub(find(ismember(model.rxns,'sink_his_L(c)')))=-1;
2534     model.c(ismember(model.rxns,'sink_hista(c)'))=1;
2535     if find(model.c)>0
2536         FBA = optimizeCbModel(model,'max');
2537         TestSolution(k,1) = FBA.f;
2538     else
2539         TestSolution(k,1) = NaN;
2540     end
2541     TestSolutionName{k,1} = 'his-L(c) -> hista(c)';
2542     if ~isnan(TestSolution(k,1)); TestedRxns = [TestedRxns; model.rxns(find(abs(FBA.x)>tol))]; end ;k = k +1;clear FBA
2543     %% hista -> 3mlda
2544     model = modelOri;
2545     model.c(find(model.c)) = 0;
2546     for i = 1 : length(RPMI_composition)
2547         model = changeRxnBounds(model,RPMI_composition{i},-1,'l');
2548     end
2549     [model] = addSinkReactions(model,{'hista(c)','3mlda(c)'},[-1 -1; 0 100]);
2550     model.lb(find(ismember(model.rxns,'DM_hista(c)')))=-1;
2551     model.ub(find(ismember(model.rxns,'DM_hista(c)')))=-1;
2552     model.c(ismember(model.rxns,'sink_3mlda(c)'))=1;
2553     if find(model.c)>0
2554         FBA = optimizeCbModel(model,'max');
2555         TestSolution(k,1) = FBA.f;
2556     else
2557         TestSolution(k,1) = NaN;
2558     end
2559     TestSolutionName{k,1} = 'hista(c) -> 3mlda(c) (with RPMI medium)';
2560     if ~isnan(TestSolution(k,1)); TestedRxns = [TestedRxns; model.rxns(find(abs(FBA.x)>tol))]; end ;k = k +1;clear FBA
2561     %% hista -> im4ac
2562     model = modelOri;
2563     model.c(find(model.c)) = 0;
2564     [model] = addSinkReactions(model,{'hista(c)','im4act(c)'},[-1 -1; 0 100]);
2565     model.c(ismember(model.rxns,'sink_im4act(c)'))=1;
2566     if find(model.c)>0
2567         FBA = optimizeCbModel(model,'max');
2568         TestSolution(k,1) = FBA.f;
2569     else
2570         TestSolution(k,1) = NaN;
2571     end
2572     TestSolutionName{k,1} = 'hista(c) -> im4ac(c)';
2573     if ~isnan(TestSolution(k,1)); TestedRxns = [TestedRxns; model.rxns(find(abs(FBA.x)>tol))]; end ;k = k +1;clear FBA
2574     %% hmgcoa(x) -> chsterol(r)
2575     model = modelOri;
2576     model.c(find(model.c)) = 0;
2577     [model] = addSinkReactions(model,{'hmgcoa(x)','chsterol(r)'},[-1 -1; 0 100]);
2578     model.c(ismember(model.rxns,'sink_chsterol(r)'))=1;
2579     if find(model.c)>0
2580         FBA = optimizeCbModel(model,'max');
2581         TestSolution(k,1) = FBA.f;
2582     else
2583         TestSolution(k,1) = NaN;
2584     end
2585     TestSolutionName{k,1} = 'hmgcoa(x) -> chsterol(r)';
2586     if ~isnan(TestSolution(k,1)); TestedRxns = [TestedRxns; model.rxns(find(abs(FBA.x)>tol))]; end ;k = k +1;clear FBA
2587     %% hmgcoa(x) -> frdp(x)
2588     model = modelOri;
2589     model.c(find(model.c)) = 0;
2590     [model] = addSinkReactions(model,{'hmgcoa(x)','frdp(x)'},[-1 -1; 0 100]);
2591     model.c(ismember(model.rxns,'sink_frdp(x)'))=1;
2592     if find(model.c)>0
2593         FBA = optimizeCbModel(model,'max');
2594         TestSolution(k,1) = FBA.f;
2595     else
2596         TestSolution(k,1) = NaN;
2597     end
2598     TestSolutionName{k,1} = 'hmgcoa(x) -> frdp(x)';
2599     if ~isnan(TestSolution(k,1)); TestedRxns = [TestedRxns; model.rxns(find(abs(FBA.x)>tol))]; end ;k = k +1;clear FBA
2600     %% hmgcoa(x) -> xoldiolone(r)
2601     model = modelOri;
2602     model.c(find(model.c)) = 0;
2603     [model] = addSinkReactions(model,{'hmgcoa(x)','xoldiolone(r)'},[-1 -1; 0 100]);
2604     model.c(ismember(model.rxns,'sink_xoldiolone(r)'))=1;
2605     if find(model.c)>0
2606         FBA = optimizeCbModel(model,'max');
2607         TestSolution(k,1) = FBA.f;
2608     else
2609         TestSolution(k,1) = NaN;
2610     end
2611     TestSolutionName{k,1} = 'hmgcoa(x) -> xoldiolone(r)';
2612     if ~isnan(TestSolution(k,1)); TestedRxns = [TestedRxns; model.rxns(find(abs(FBA.x)>tol))]; end ;k = k +1;clear FBA
2613     %% hmgcoa(x) -> xoltriol(c)
2614     model = modelOri;
2615     model.c(find(model.c)) = 0;
2616     [model] = addSinkReactions(model,{'hmgcoa(x)','xoltriol(c)'},[-1 -1; 0 100]);
2617     model.c(ismember(model.rxns,'sink_xoltriol(c)'))=1;
2618     if find(model.c)>0
2619         FBA = optimizeCbModel(model,'max');
2620         TestSolution(k,1) = FBA.f;
2621     else
2622         TestSolution(k,1) = NaN;
2623     end
2624     TestSolutionName{k,1} = 'hmgcoa(x) -> xoltriol(c)';
2625     if ~isnan(TestSolution(k,1)); TestedRxns = [TestedRxns; model.rxns(find(abs(FBA.x)>tol))]; end ;k = k +1;clear FBA
2626     %% hmgcoa(x)-chsterol(r) %duplicate
2627     % model = modelOri;
2628     % model.c(find(model.c)) = 0;
2629     % [model] = addSinkReactions(model,{'hmgcoa(x)','chsterol(r)'},[-1 -1; 0 100]);
2630     % model.c(ismember(model.rxns,'sink_chsterol(r)'))=1;
2631     %   FBA = optimizeCbModel(model,'max');
2632     % TestSolution(k,1) = FBA.f;
2633     % TestSolutionName{k,1} = 'hmgcoa(x) -> chsterol(r)';
2634     % k = k +1;clear FBA
2635     %% hpyr -> 2pg
2636     model = modelOri;
2637     model.c(find(model.c)) = 0;
2638     [model] = addSinkReactions(model,{'hpyr(c)','2pg(c)'},[-1 -1; 0 100]);
2639     model.c(ismember(model.rxns,'sink_2pg(c)'))=1;
2640     if find(model.c)>0
2641         FBA = optimizeCbModel(model,'max');
2642         TestSolution(k,1) = FBA.f;
2643     else
2644         TestSolution(k,1) = NaN;
2645     end
2646     TestSolutionName{k,1} = 'hpyr(c) -> 2pg(c)';
2647     if ~isnan(TestSolution(k,1)); TestedRxns = [TestedRxns; model.rxns(find(abs(FBA.x)>tol))]; end ;k = k +1;clear FBA
2648     %% hpyr -> glyclt
2649     model = modelOri;
2650     model.c(find(model.c)) = 0;
2651     [model] = addSinkReactions(model,{'hpyr(c)','glyclt(c)'},[-1 -1; 0 100]);
2652     model.c(ismember(model.rxns,'sink_glyclt(c)'))=1;
2653     if find(model.c)>0
2654         FBA = optimizeCbModel(model,'max');
2655         TestSolution(k,1) = FBA.f;
2656     else
2657         TestSolution(k,1) = NaN;
2658     end
2659     TestSolutionName{k,1} = 'hpyr(c) -> glyclt(c)';
2660     if ~isnan(TestSolution(k,1)); TestedRxns = [TestedRxns; model.rxns(find(abs(FBA.x)>tol))]; end ;k = k +1;clear FBA
2661     %% hpyr -> glyc-S
2662     model = modelOri;
2663     model.c(find(model.c)) = 0;
2664     [model] = addSinkReactions(model,{'hpyr(c)','glyc-S(c)'},[-1 -1; 0 100]);
2665     model.c(ismember(model.rxns,'sink_glyc-S(c)'))=1;
2666     if find(model.c)>0
2667         FBA = optimizeCbModel(model,'max');
2668         TestSolution(k,1) = FBA.f;
2669     else
2670         TestSolution(k,1) = NaN;
2671     end
2672     TestSolutionName{k,1} = 'hpyr(c) -> glyc-S(c)';
2673     if ~isnan(TestSolution(k,1)); TestedRxns = [TestedRxns; model.rxns(find(abs(FBA.x)>tol))]; end ;k = k +1;clear FBA
2674     %% hspg(l) -> 2 gal(l) + glcur(l) + xyl-D(l) %changing lb has no effect
2675     model = modelOri;
2676     model.c(find(model.c)) = 0;
2677     [model] = addSinkReactions(model,{'hspg(l)','gal(l)','glcur(l)','xyl-D(l)'},[-1 -1; 0.1 100; 0.1 100; 0.1 100]);
2678     model.c(ismember(model.rxns,'sink_xyl-D(l)'))=1;
2679     if find(model.c)>0
2680         FBA = optimizeCbModel(model,'max');
2681         TestSolution(k,1) = FBA.f;
2682     else
2683         TestSolution(k,1) = NaN;
2684     end
2685     TestSolutionName{k,1} = 'hspg(l) -> gal(l) + glcur(l) + xyl-D(l)';
2686     if ~isnan(TestSolution(k,1)); TestedRxns = [TestedRxns; model.rxns(find(abs(FBA.x)>tol))]; end ;k = k +1;clear FBA
2687     %% hyptaur(c) -> taur(x)
2688     model = modelOri;
2689     model.c(find(model.c)) = 0;
2690     [model] = addSinkReactions(model,{'hyptaur(c)','taur(x)'},[-1 -1; 0 100]);
2691     model.c(ismember(model.rxns,'sink_taur(x)'))=1;
2692     if find(model.c)>0
2693         FBA = optimizeCbModel(model,'max');
2694         TestSolution(k,1) = FBA.f;
2695     else
2696         TestSolution(k,1) = NaN;
2697     end
2698     TestSolutionName{k,1} = 'hyptaur(c) -> taur(x)';
2699     if ~isnan(TestSolution(k,1)); TestedRxns = [TestedRxns; model.rxns(find(abs(FBA.x)>tol))]; end ;k = k +1;clear FBA
2700     %% ile-L -> accoa
2701     model = modelOri;
2702     model.c(find(model.c)) = 0;
2703     [model] = addSinkReactions(model,{'ile-L(c)','accoa(c)'},[-1 -1; 0 100]);
2704     model.lb(find(ismember(model.rxns,'sink_ile_L(c)')))=-1;
2705     model.ub(find(ismember(model.rxns,'sink_ile_L(c)')))=-1;
2706     model.lb(find(ismember(model.rxns,'sink_coa(c)')))=-1;
2707     model.ub(find(ismember(model.rxns,'sink_coa(c)')))=1;
2708     model.c(ismember(model.rxns,'sink_accoa(c)'))=1;
2709     if find(model.c)>0
2710         FBA = optimizeCbModel(model,'max');
2711         TestSolution(k,1) = FBA.f;
2712     else
2713         TestSolution(k,1) = NaN;
2714     end
2715     TestSolutionName{k,1} = 'ile-L(c) -> accoa(c)';
2716     if ~isnan(TestSolution(k,1)); TestedRxns = [TestedRxns; model.rxns(find(abs(FBA.x)>tol))]; end ;k = k +1;clear FBA
2717     %% inost -> pail_hs
2718     model = modelOri;
2719     model.c(find(model.c)) = 0;
2720     for i = 1 : length(RPMI_composition)
2721         model = changeRxnBounds(model,RPMI_composition{i},-1,'l');
2722     end
2723     [model] = addSinkReactions(model,{'inost(c)','pail_hs(c)'},[-1 -1; 0 100]);
2724     model.c(ismember(model.rxns,'sink_pail_hs(c)'))=1;
2725     if find(model.c)>0
2726         FBA = optimizeCbModel(model,'max');
2727         TestSolution(k,1) = FBA.f;
2728     else
2729         TestSolution(k,1) = NaN;
2730     end
2731     TestSolutionName{k,1} = 'inost(c) -> pail_hs(c) (with RPMI medium)';
2732     if ~isnan(TestSolution(k,1)); TestedRxns = [TestedRxns; model.rxns(find(abs(FBA.x)>tol))]; end ;k = k +1;clear FBA
2733     %% inost -> pail45p_hs
2734     model = modelOri;
2735     model.c(find(model.c)) = 0;
2736     for i = 1 : length(RPMI_composition)
2737         model = changeRxnBounds(model,RPMI_composition{i},-1,'l');
2738     end
2739     [model] = addSinkReactions(model,{'inost(c)','pail45p_hs(c)'},[-1 -1; 0 100]);
2740     model.c(ismember(model.rxns,'sink_pail45p_hs(c)'))=1;
2741     if find(model.c)>0
2742         FBA = optimizeCbModel(model,'max');
2743         TestSolution(k,1) = FBA.f;
2744     else
2745         TestSolution(k,1) = NaN;
2746     end
2747     TestSolutionName{k,1} = 'inost(c) -> pail45p_hs(c) (with RPMI medium)';
2748     if ~isnan(TestSolution(k,1)); TestedRxns = [TestedRxns; model.rxns(find(abs(FBA.x)>tol))]; end ;k = k +1;clear FBA
2749     %% inost -> pail4p_hs
2750     model = modelOri;
2751     for i = 1 : length(RPMI_composition)
2752         model = changeRxnBounds(model,RPMI_composition{i},-1,'l');
2753     end
2754     model.c(find(model.c)) = 0;
2755     [model] = addSinkReactions(model,{'inost(c)','pail4p_hs(c)'},[-1 -1; 0 100]);
2756     model.c(ismember(model.rxns,'sink_pail4p_hs(c)'))=1;
2757     if find(model.c)>0
2758         FBA = optimizeCbModel(model,'max');
2759         TestSolution(k,1) = FBA.f;
2760     else
2761         TestSolution(k,1) = NaN;
2762     end
2763     TestSolutionName{k,1} = 'inost(c) -> pail4p_hs(c) (with RPMI medium)';
2764     if ~isnan(TestSolution(k,1)); TestedRxns = [TestedRxns; model.rxns(find(abs(FBA.x)>tol))]; end ;k = k +1;clear FBA
2765     %% inost -> xu5p-D
2766     model = modelOri;
2767     model.c(find(model.c)) = 0;
2768     [model] = addSinkReactions(model,{'inost(c)','xu5p-D(c)'},[-1 -1; 0 100]);
2769     model.c(ismember(model.rxns,'sink_xu5p-D(c)'))=1;
2770     if find(model.c)>0
2771         FBA = optimizeCbModel(model,'max');
2772         TestSolution(k,1) = FBA.f;
2773     else
2774         TestSolution(k,1) = NaN;
2775     end
2776     TestSolutionName{k,1} = 'inost(c) -> xu5p-D(c)';
2777     if ~isnan(TestSolution(k,1)); TestedRxns = [TestedRxns; model.rxns(find(abs(FBA.x)>tol))]; end ;k = k +1;clear FBA
2778     %% ipdp(x) -> sql(r)
2779     model = modelOri;
2780     model.c(find(model.c)) = 0;
2781     model.lb(ismember(model.rxns,'EX_glc(e)'))=-1;model.ub(ismember(model.rxns,'EX_glc(e)'))=0;
2782     model.lb(ismember(model.rxns,'EX_o2(e)'))=-40;model.ub(ismember(model.rxns,'EX_o2(e)'))=-1;
2783     [model] = addSinkReactions(model,{'ipdp(x)','sql(r)'},[-1 -1; 0 100]);
2784     model.c(ismember(model.rxns,'sink_sql(r)'))=1;
2785     if find(model.c)>0
2786         FBA = optimizeCbModel(model,'max');
2787         TestSolution(k,1) = FBA.f;
2788     else
2789         TestSolution(k,1) = NaN;
2790     end
2791     TestSolutionName{k,1} = 'ipdp(x) -> sql(r)';
2792     if ~isnan(TestSolution(k,1)); TestedRxns = [TestedRxns; model.rxns(find(abs(FBA.x)>tol))]; end ;k = k +1;clear FBA
2793     %% itacon[m] -> pyr[m] %changing lb has no effect
2794     model = modelOri;
2795     model.c(find(model.c)) = 0;
2796     [model] = addSinkReactions(model,{'itacon(m)','pyr(m)'},[-1 -1; 0 100]);
2797     model.c(ismember(model.rxns,'sink_pyr(m)'))=1;
2798     if find(model.c)>0
2799         FBA = optimizeCbModel(model,'max');
2800         TestSolution(k,1) = FBA.f;
2801     else
2802         TestSolution(k,1) = NaN;
2803     end
2804     TestSolutionName{k,1} = 'itacon(m) -> pyr(m)';
2805     if ~isnan(TestSolution(k,1)); TestedRxns = [TestedRxns; model.rxns(find(abs(FBA.x)>tol))]; end ;k = k +1;clear FBA
2806     %% ksi[l] -> man[l] + acgam[l]
2807     model = modelOri;
2808     model.c(find(model.c)) = 0;
2809     for i = 1 : length(RPMI_composition)
2810         model = changeRxnBounds(model,RPMI_composition{i},-1,'l');
2811     end
2812     [model] = addSinkReactions(model,{'ksi(l)','man(l)','acgam(l)'},[-1 -1; 0.1 100; 0.1 100]);
2813     model.c(ismember(model.rxns,'sink_acgam(l)'))=1;
2814     if find(model.c)>0
2815         FBA = optimizeCbModel(model,'max');
2816         TestSolution(k,1) = FBA.f;
2817     else
2818         TestSolution(k,1) = NaN;
2819     end
2820     TestSolutionName{k,1} = 'ksi[l] -> man[l] + acgam[l] (with RPMI medium)';
2821     if ~isnan(TestSolution(k,1)); TestedRxns = [TestedRxns; model.rxns(find(abs(FBA.x)>tol))]; end ;k = k +1;clear FBA
2822     %% ksii_core2(l) -> Ser/Thr(l)
2823     model = modelOri;
2824     model.c(find(model.c)) = 0;
2825     [model,rxnsInModel] = addSinkReactions(model,{'ksii_core2(l)','Ser/Thr(l)'},[-1 -1; 0 100]);
2826     if (rxnsInModel(2) >-1) % reaction exits already in model
2827         model=changeObjective(model,model.rxns(rxnsInModel(2),1));
2828     else
2829         model=changeObjective(model,'sink_Ser/Thr(l)',1);
2830     end
2831     if find(model.c)>0
2832         FBA = optimizeCbModel(model,'max');
2833         TestSolution(k,1) = FBA.f;
2834     else
2835         TestSolution(k,1) = NaN;
2836     end
2837     TestSolutionName{k,1} = 'ksii_core2(l) -> Ser/Thr(l)';
2838     if ~isnan(TestSolution(k,1)); TestedRxns = [TestedRxns; model.rxns(find(abs(FBA.x)>tol))]; end ;k = k +1;clear FBA
2839     %% ksii_core4(l) -> Ser/Thr(l)
2840     model = modelOri;
2841     model.c(find(model.c)) = 0;
2842     [model,rxnsInModel] = addSinkReactions(model,{'ksii_core4(l)','Ser/Thr(l)'},[-1 -1; 0 100]);
2843     if (rxnsInModel(2) >-1) % reaction exits already in model
2844         model=changeObjective(model,model.rxns(rxnsInModel(2),1));
2845     else
2846         model=changeObjective(model,'sink_Ser/Thr(l)',1);
2847     end
2848     if find(model.c)>0
2849         FBA = optimizeCbModel(model,'max');
2850         TestSolution(k,1) = FBA.f;
2851     else
2852         TestSolution(k,1) = NaN;
2853     end
2854     TestSolutionName{k,1} = 'ksii_core4(l) -> Ser/Thr(l)';
2855     if ~isnan(TestSolution(k,1)); TestedRxns = [TestedRxns; model.rxns(find(abs(FBA.x)>tol))]; end ;k = k +1;clear FBA
2856     %% l2fn2m2masn[g] -> ksi[g]
2857     model = modelOri;
2858     model.c(find(model.c)) = 0;
2859     for i = 1 : length(RPMI_composition)
2860         model = changeRxnBounds(model,RPMI_composition{i},-1,'l');
2861     end
2862     [model] = addSinkReactions(model,{'l2fn2m2masn(g)','ksi(g)'},[-1 -1; 0 100]);
2863     model.c(ismember(model.rxns,'sink_ksi(g)'))=1;
2864     if find(model.c)>0
2865         FBA = optimizeCbModel(model,'max');
2866         TestSolution(k,1) = FBA.f;
2867     else
2868         TestSolution(k,1) = NaN;
2869     end
2870     TestSolutionName{k,1} = 'l2fn2m2masn(g) -> ksi(g) (with RPMI medium)';
2871     if ~isnan(TestSolution(k,1)); TestedRxns = [TestedRxns; model.rxns(find(abs(FBA.x)>tol))]; end ;k = k +1;clear FBA
2872     %% lac -> glc % i assumed lac-L
2873     model = modelOri;
2874     model.c(find(model.c)) = 0;
2875     [model] = addSinkReactions(model,{'lac-L(c)','glc-D(c)'},[-1 -1; 0 100]);
2876     model.c(ismember(model.rxns,'sink_glc-D(c)'))=1;
2877     if find(model.c)>0
2878         FBA = optimizeCbModel(model,'max');
2879         TestSolution(k,1) = FBA.f;
2880     else
2881         TestSolution(k,1) = NaN;
2882     end
2883     TestSolutionName{k,1} = 'lac-L(c) -> glc-D(c)';
2884     if ~isnan(TestSolution(k,1)); TestedRxns = [TestedRxns; model.rxns(find(abs(FBA.x)>tol))]; end ;k = k +1;clear FBA
2885     %% Lcyst(c) -> taur(x)
2886     model = modelOri;
2887     model.c(find(model.c)) = 0;
2888     [model] = addSinkReactions(model,{'Lcyst(c)','taur(x)'},[-1 -1; 0 100]);
2889     model.c(ismember(model.rxns,'sink_taur(x)'))=1;
2890     if find(model.c)>0
2891         FBA = optimizeCbModel(model,'max');
2892         TestSolution(k,1) = FBA.f;
2893     else
2894         TestSolution(k,1) = NaN;
2895     end
2896     TestSolutionName{k,1} = 'Lcyst(c) -> taur(x)';
2897     if ~isnan(TestSolution(k,1)); TestedRxns = [TestedRxns; model.rxns(find(abs(FBA.x)>tol))]; end ;k = k +1;clear FBA
2898     %% leu-L -> accoa
2899     model = modelOri;
2900     model.c(find(model.c)) = 0;
2901     [model] = addSinkReactions(model,{'leu-L(c)','accoa(c)'},[-1 -1; 0 100]);
2902     model.lb(find(ismember(model.rxns,'sink_leu_L(c)')))=-1;
2903     model.ub(find(ismember(model.rxns,'sink_leu_L(c)')))=-1;
2904     model.lb(find(ismember(model.rxns,'sink_coa(c)')))=-1;
2905     model.ub(find(ismember(model.rxns,'sink_coa(c)')))=1;
2906     model.c(ismember(model.rxns,'sink_accoa(c)'))=1;
2907     if find(model.c)>0
2908         FBA = optimizeCbModel(model,'max');
2909         TestSolution(k,1) = FBA.f;
2910     else
2911         TestSolution(k,1) = NaN;
2912     end
2913     TestSolutionName{k,1} = 'leu-L(c) -> accoa(c)';
2914     if ~isnan(TestSolution(k,1)); TestedRxns = [TestedRxns; model.rxns(find(abs(FBA.x)>tol))]; end ;k = k +1;clear FBA
2915     %% lys-L[c] -> accoa[m] (via saccrp-L pathway)
2916     model = modelOri;
2917     model.c(find(model.c)) = 0;
2918     [model] = addSinkReactions(model,{'lys-L(c)','accoa(m)'},[-1 -1; 0 100]);
2919     model.lb(find(ismember(model.rxns,'sink_lys_L(c)')))=-1;
2920     model.ub(find(ismember(model.rxns,'sink_lys_L(c)')))=-1;
2921     model.lb(find(ismember(model.rxns,'sink_coa(c)')))=-1;
2922     model.ub(find(ismember(model.rxns,'sink_coa(c)')))=1;
2923     model.c(ismember(model.rxns,'sink_accoa(m)'))=1;
2924     if find(model.c)>0
2925         FBA = optimizeCbModel(model,'max');
2926         TestSolution(k,1) = FBA.f;
2927     else
2928         TestSolution(k,1) = NaN;
2929     end
2930     TestSolutionName{k,1} = 'lys-L[c] -> accoa[m] (via saccrp-L pathway)';
2931     if ~isnan(TestSolution(k,1)); TestedRxns = [TestedRxns; model.rxns(find(abs(FBA.x)>tol))]; end ;k = k +1;clear FBA
2932     %% lys-L[x] -> aacoa[m] (via Lpipecol pathway)
2933     model = modelOri;
2934     model.c(find(model.c)) = 0;
2935     model.lb(find(ismember(model.rxns,'EX_lys-L(e)')))=-1;
2936     model.ub(find(ismember(model.rxns,'EX_lys-L(e)')))=-1;
2937     [model] = addSinkReactions(model,{'lys-L(x)','aacoa(m)'},[-1 -1; 0 100]);
2938     model.lb(find(ismember(model.rxns,'sink_coa(c)')))=-1;
2939     model.ub(find(ismember(model.rxns,'sink_coa(c)')))=1;
2940     model.c(ismember(model.rxns,'sink_aacoa(m)'))=1;
2941     if find(model.c)>0
2942         FBA = optimizeCbModel(model,'max');
2943         TestSolution(k,1) = FBA.f;
2944     else
2945         TestSolution(k,1) = NaN;
2946     end
2947     TestSolutionName{k,1} = 'lys-L[x] -> aacoa[m] (via Lpipecol pathway)';
2948     if ~isnan(TestSolution(k,1)); TestedRxns = [TestedRxns; model.rxns(find(abs(FBA.x)>tol))]; end ;k = k +1;clear FBA
2949     %% m8masn[r] -> nm4masn[g]
2950     model = modelOri;
2951     model.c(find(model.c)) = 0;
2952     model.lb(ismember(model.rxns,'EX_glc(e)'))=-1;model.ub(ismember(model.rxns,'EX_glc(e)'))=0;
2953     model.lb(ismember(model.rxns,'EX_o2(e)'))=-40;model.ub(ismember(model.rxns,'EX_o2(e)'))=0;
2954     [model] = addSinkReactions(model,{'m8masn(r)','nm4masn(g)'},[-1 -1; 0 100]);
2955     model.c(ismember(model.rxns,'sink_nm4masn(g)'))=1;
2956     if find(model.c)>0
2957         FBA = optimizeCbModel(model,'max');
2958         TestSolution(k,1) = FBA.f;
2959     else
2960         TestSolution(k,1) = NaN;
2961     end
2962     TestSolutionName{k,1} = 'm8masn(r) -> nm4masn(g)';
2963     if ~isnan(TestSolution(k,1)); TestedRxns = [TestedRxns; model.rxns(find(abs(FBA.x)>tol))]; end ;k = k +1;clear FBA
2964     %% man -> gdpmann
2965     model = modelOri;
2966     model.c(find(model.c)) = 0;
2967     [model] = addSinkReactions(model,{'man(c)','gdpmann(c)'},[-1 -1; 0 100]);
2968     model.c(ismember(model.rxns,'sink_gdpmann(c)'))=1;
2969     if find(model.c)>0
2970         FBA = optimizeCbModel(model,'max');
2971         TestSolution(k,1) = FBA.f;
2972     else
2973         TestSolution(k,1) = NaN;
2974     end
2975     TestSolutionName{k,1} = 'man(c) -> gdpmann(c)';
2976     if ~isnan(TestSolution(k,1)); TestedRxns = [TestedRxns; model.rxns(find(abs(FBA.x)>tol))]; end ;k = k +1;clear FBA
2977     %% man6p -> kdn
2978     model = modelOri;
2979     model.c(find(model.c)) = 0;
2980     [model] = addSinkReactions(model,{'man6p(c)','kdn(c)'},[-1 -1; 0 100]);
2981     if ~isempty(strmatch('ACNAM9PL2',model.rxns,'exact'))
2982         model.c(ismember(model.rxns,'ACNAM9PL2'))=1;
2983         FBA = optimizeCbModel(model,'max');
2984         TestSolution(k,1) = FBA.f;
2985     else
2986         TestSolution(k,1) = NaN;
2987     end
2988     TestSolutionName{k,1} = 'man6p(c) -> kdn(c) - via ACNAM9PL2';
2989     if ~isnan(TestSolution(k,1)); TestedRxns = [TestedRxns; model.rxns(find(abs(FBA.x)>tol))]; end ;k = k +1;clear FBA
2990     
2991     %% mescon[m] -> pyr[m] %changing lb has no effect
2992     model = modelOri;
2993     model.c(find(model.c)) = 0;
2994     [model] = addSinkReactions(model,{'mescon(m)','pyr(m)'},[-1 -1; 0 100]);
2995     model.c(ismember(model.rxns,'sink_pyr(m)'))=1;
2996     if find(model.c)>0
2997         FBA = optimizeCbModel(model,'max');
2998         TestSolution(k,1) = FBA.f;
2999     else
3000         TestSolution(k,1) = NaN;
3001     end
3002     TestSolutionName{k,1} = 'mescon(m) -> pyr(m)';
3003     if ~isnan(TestSolution(k,1)); TestedRxns = [TestedRxns; model.rxns(find(abs(FBA.x)>tol))]; end ;k = k +1;clear FBA
3004     %% met-L -> cys-L
3005     model = modelOri;
3006     model.c(find(model.c)) = 0;
3007     [model] = addSinkReactions(model,{'met-L(c)','cys-L(c)'},[-1 -1; 0 100]);
3008     model.c(ismember(model.rxns,'sink_cys-L(c)'))=1;
3009     if find(model.c)>0
3010         FBA = optimizeCbModel(model,'max');
3011         TestSolution(k,1) = FBA.f;
3012     else
3013         TestSolution(k,1) = NaN;
3014     end
3015     TestSolutionName{k,1} = 'met-L(c) -> cys-L(c)';
3016     if ~isnan(TestSolution(k,1)); TestedRxns = [TestedRxns; model.rxns(find(abs(FBA.x)>tol))]; end ;k = k +1;clear FBA
3017     %% mi145p -> inost
3018     model = modelOri;
3019     model.c(find(model.c)) = 0;
3020     [model] = addSinkReactions(model,{'mi145p(c)','inost(c)'},[-1 -1; 0 100]);
3021     model.c(ismember(model.rxns,'sink_inost(c)'))=1;
3022     if find(model.c)>0
3023         FBA = optimizeCbModel(model,'max');
3024         TestSolution(k,1) = FBA.f;
3025     else
3026         TestSolution(k,1) = NaN;
3027     end
3028     TestSolutionName{k,1} = 'mi145p(c) -> inost(c)';
3029     if ~isnan(TestSolution(k,1)); TestedRxns = [TestedRxns; model.rxns(find(abs(FBA.x)>tol))]; end ;k = k +1;clear FBA
3030     %% missing dtmp-3aib testing ???
3031     %% msa -> ala-B %changing lb has no effect
3032     model = modelOri;
3033     model.c(find(model.c)) = 0;
3034     [model] = addSinkReactions(model,{'msa(m)','ala-B(m)'},[-1 -1; 0 100]);
3035     model.c(ismember(model.rxns,'sink_ala-B(m)'))=1;
3036     if find(model.c)>0
3037         FBA = optimizeCbModel(model,'max');
3038         TestSolution(k,1) = FBA.f;
3039     else
3040         TestSolution(k,1) = NaN;
3041     end
3042     TestSolutionName{k,1} = 'msa(m) -> ala-B(m)';
3043     if ~isnan(TestSolution(k,1)); TestedRxns = [TestedRxns; model.rxns(find(abs(FBA.x)>tol))]; end ;k = k +1;clear FBA
3044     %% mthgxl -> 12ppd-S
3045     model = modelOri;
3046     model.c(find(model.c)) = 0;
3047     [model] = addSinkReactions(model,{'mthgxl(c)','12ppd-S(c)'},[-1 -1; 0 100]);
3048     model.c(ismember(model.rxns,'sink_12ppd-S(c)'))=1;
3049     if find(model.c)>0
3050         FBA = optimizeCbModel(model,'max');
3051         TestSolution(k,1) = FBA.f;
3052     else
3053         TestSolution(k,1) = NaN;
3054     end
3055     TestSolutionName{k,1} = 'mthgxl(c) -> 12ppd-S(c)';
3056     if ~isnan(TestSolution(k,1)); TestedRxns = [TestedRxns; model.rxns(find(abs(FBA.x)>tol))]; end ;k = k +1;clear FBA
3057     %% mthgxl -> lac-D
3058     model = modelOri;
3059     model.c(find(model.c)) = 0;
3060     [model] = addSinkReactions(model,{'mthgxl(c)','lac-D(c)'},[-1 -1; 0 100]);
3061     model.c(ismember(model.rxns,'sink_lac-D(c)'))=1;
3062     if find(model.c)>0
3063         FBA = optimizeCbModel(model,'max');
3064         TestSolution(k,1) = FBA.f;
3065     else
3066         TestSolution(k,1) = NaN;
3067     end
3068     TestSolutionName{k,1} = 'mthgxl(c) -> lac-D(c)';
3069     if ~isnan(TestSolution(k,1)); TestedRxns = [TestedRxns; model.rxns(find(abs(FBA.x)>tol))]; end ;k = k +1;clear FBA
3070     %% n2m2nmasn[l] -> man[l] + acgam[l]
3071     model = modelOri;
3072     model.c(find(model.c)) = 0;
3073     for i = 1 : length(RPMI_composition)
3074         model = changeRxnBounds(model,RPMI_composition{i},-1,'l');
3075     end
3076     [model] = addSinkReactions(model,{'n2m2nmasn(l)','man(l)','acgam(l)'},[-1 -1; 0.1 100; 0.1 100]);
3077     model.c(ismember(model.rxns,'sink_acgam(l)'))=1;
3078     if find(model.c)>0
3079         FBA = optimizeCbModel(model,'max');
3080         TestSolution(k,1) = FBA.f;
3081     else
3082         TestSolution(k,1) = NaN;
3083     end
3084     TestSolutionName{k,1} = 'n2m2nmasn[l] -> man[l] + acgam[l] (with RPMI medium)';
3085     if ~isnan(TestSolution(k,1)); TestedRxns = [TestedRxns; model.rxns(find(abs(FBA.x)>tol))]; end ;k = k +1;clear FBA
3086     %% nm4masn[g] -> l2fn2m2masn[g]
3087     model = modelOri;
3088     model.c(find(model.c)) = 0;
3089     for i = 1 : length(RPMI_composition)
3090         model = changeRxnBounds(model,RPMI_composition{i},-1,'l');
3091     end
3092     [model] = addSinkReactions(model,{'nm4masn(g)','l2fn2m2masn(g)'},[-1 -1; 0 100]);
3093     model.c(ismember(model.rxns,'sink_l2fn2m2masn(g)'))=1;
3094     if find(model.c)>0
3095         FBA = optimizeCbModel(model,'max');
3096         TestSolution(k,1) = FBA.f;
3097     else
3098         TestSolution(k,1) = NaN;
3099     end
3100     TestSolutionName{k,1} = 'nm4masn(g) -> l2fn2m2masn(g) (with RPMI medium)';
3101     if ~isnan(TestSolution(k,1)); TestedRxns = [TestedRxns; model.rxns(find(abs(FBA.x)>tol))]; end ;k = k +1;clear FBA
3102     %% nm4masn[g] -> n2m2nmasn[g]
3103     model = modelOri;
3104     model.c(find(model.c)) = 0;
3105     for i = 1 : length(RPMI_composition)
3106         model = changeRxnBounds(model,RPMI_composition{i},-1,'l');
3107     end
3108     [model] = addSinkReactions(model,{'nm4masn(g)','n2m2nmasn(g)'},[-1 -1; 0 100]);
3109     model.c(ismember(model.rxns,'sink_n2m2nmasn(g)'))=1;
3110     if find(model.c)>0
3111         FBA = optimizeCbModel(model,'max');
3112         TestSolution(k,1) = FBA.f;
3113     else
3114         TestSolution(k,1) = NaN;
3115     end
3116     TestSolutionName{k,1} = 'nm4masn(g) -> n2m2nmasn(g) (with RPMI medium)';
3117     if ~isnan(TestSolution(k,1)); TestedRxns = [TestedRxns; model.rxns(find(abs(FBA.x)>tol))]; end ;k = k +1;clear FBA
3118     %% nm4masn[g] -> s2l2fn2m2masn[g]
3119     model = modelOri;
3120     model.c(find(model.c)) = 0;
3121     for i = 1 : length(RPMI_composition)
3122         model = changeRxnBounds(model,RPMI_composition{i},-1,'l');
3123     end
3124     [model] = addSinkReactions(model,{'nm4masn(g)','s2l2fn2m2masn(g)'},[-1 -1; 0 100]);
3125     model.c(ismember(model.rxns,'sink_s2l2fn2m2masn(g)'))=1;
3126     if find(model.c)>0
3127         FBA = optimizeCbModel(model,'max');
3128         TestSolution(k,1) = FBA.f;
3129     else
3130         TestSolution(k,1) = NaN;
3131     end
3132     TestSolutionName{k,1} = 'nm4masn(g) -> s2l2fn2m2masn(g) (with RPMI medium)';
3133     if ~isnan(TestSolution(k,1)); TestedRxns = [TestedRxns; model.rxns(find(abs(FBA.x)>tol))]; end ;k = k +1;clear FBA
3134     %% npphr -> 34dhoxpeg %npphr does not exists
3135     % model = modelOri;
3136     % model.c(find(model.c)) = 0;
3137     % [model] = addSinkReactions(model,{'npphr(c)','34dhoxpeg(c)'},[-1 -1; 0 100]);
3138     % model.c(ismember(model.rxns,'sink_34dhoxpeg(c)'))=1;
3139     %   FBA = optimizeCbModel(model,'max');
3140     % TestSolution(k,1) = FBA.f;
3141     % TestSolutionName{k,1} = 'npphr(c) -> 34dhoxpeg(c)';
3142     % k = k +1;clear FBA
3143     %% o2- -> h2o2 -> o2 + h2o
3144     model = modelOri;
3145     model.c(find(model.c)) = 0;
3146     [model] = addSinkReactions(model,{'o2s(c)','h2o2(c)'},[-1 -1; 0 100]);
3147     model.c(ismember(model.rxns,'sink_h2o2(c)'))=1;
3148     if find(model.c)>0
3149         FBA = optimizeCbModel(model,'max');
3150         TestSolution(k,1) = FBA.f;
3151     else
3152         TestSolution(k,1) = NaN;
3153     end
3154     TestSolutionName{k,1} = 'o2- -> h2o2 -> o2 + h2o, 1';
3155     if ~isnan(TestSolution(k,1)); TestedRxns = [TestedRxns; model.rxns(find(abs(FBA.x)>tol))]; end ;k = k +1;clear FBA
3156     model = modelOri;
3157     model.c(find(model.c)) = 0;
3158     
3159     model.lb(ismember(model.rxns,'EX_h2o(e)'))=0;
3160     model.lb(ismember(model.rxns,'EX_o2(e)'))=0;
3161     [model] = addSinkReactions(model,{'h2o2(c)','o2(c)','h2o(c)'},[-1 -1; -1 -1; 0.1 100]);
3162     model.c(ismember(model.rxns,'sink_h2o(c)'))=1;
3163     if find(model.c)>0
3164         FBA = optimizeCbModel(model,'max');
3165         TestSolution(k,1) = FBA.f;
3166     else
3167         TestSolution(k,1) = NaN;
3168     end
3169     TestSolutionName{k,1} = 'o2- -> h2o2 -> o2 + h2o, 2';
3170     if ~isnan(TestSolution(k,1)); TestedRxns = [TestedRxns; model.rxns(find(abs(FBA.x)>tol))]; end ;k = k +1;clear FBA
3171     %% orn -> nh4 v0.05
3172     model = modelOri;
3173     model.c(find(model.c)) = 0;
3174     [model] = addSinkReactions(model,{'orn(c)','nh4(c)'},[-1 -1; 0 100]);
3175     model.lb(ismember(model.rxns,'EX_nh4(e)'))=0;model.ub(ismember(model.rxns,'EX_nh4(e)'))=1000;
3176     model.c(ismember(model.rxns,'sink_nh4(c)'))=1;
3177     if find(model.c)>0
3178         FBA = optimizeCbModel(model,'max');
3179         TestSolution(k,1) = FBA.f;
3180     else
3181         TestSolution(k,1) = NaN;
3182     end
3183     TestSolutionName{k,1} = 'orn(c) -> nh4(c)';
3184     if ~isnan(TestSolution(k,1)); TestedRxns = [TestedRxns; model.rxns(find(abs(FBA.x)>tol))]; end ;k = k +1;clear FBA
3185     %% orn -> ptrc
3186     model = modelOri;
3187     model.c(find(model.c)) = 0;
3188     [model] = addSinkReactions(model,{'orn(c)','ptrc(c)'},[-1 -1; 0 100]);
3189     model.c(ismember(model.rxns,'sink_ptrc(c)'))=1;
3190     if find(model.c)>0
3191         FBA = optimizeCbModel(model,'max');
3192         TestSolution(k,1) = FBA.f;
3193     else
3194         TestSolution(k,1) = NaN;
3195     end
3196     TestSolutionName{k,1} = 'orn(c) -> ptrc(c)';
3197     if ~isnan(TestSolution(k,1)); TestedRxns = [TestedRxns; model.rxns(find(abs(FBA.x)>tol))]; end ;k = k +1;clear FBA
3198     %% orn -> spmd
3199     model = modelOri;
3200     model.c(find(model.c)) = 0;
3201     [model] = addSinkReactions(model,{'orn(c)','spmd(c)'},[-1 -1; 0 100]);
3202     model.c(ismember(model.rxns,'sink_spmd(c)'))=1;
3203     if find(model.c)>0
3204         FBA = optimizeCbModel(model,'max');
3205         TestSolution(k,1) = FBA.f;
3206     else
3207         TestSolution(k,1) = NaN;
3208     end
3209     TestSolutionName{k,1} = 'orn(c) -> spmd(c)';
3210     if ~isnan(TestSolution(k,1)); TestedRxns = [TestedRxns; model.rxns(find(abs(FBA.x)>tol))]; end ;k = k +1;clear FBA
3211     %% orn -> sprm
3212     model = modelOri;
3213     model.c(find(model.c)) = 0;
3214     [model,rxnsInModel] = addSinkReactions(model,{'orn(c)','sprm(c)'},[-1 -1; 0 100]);
3215     if (rxnsInModel(2) >-1) % reaction exits already in model
3216         model=changeObjective(model,model.rxns(rxnsInModel(2),1));
3217     else
3218         model=changeObjective(model,'sink_sprm(c)',1);
3219     end
3220     if find(model.c)>0
3221         FBA = optimizeCbModel(model,'max');
3222         TestSolution(k,1) = FBA.f;
3223     else
3224         TestSolution(k,1) = NaN;
3225     end
3226     TestSolutionName{k,1} = 'orn(c) -> sprm(c)';
3227     if ~isnan(TestSolution(k,1)); TestedRxns = [TestedRxns; model.rxns(find(abs(FBA.x)>tol))]; end ;k = k +1;clear FBA
3228     %% pail_hs -> gpi_prot_hs[r]
3229     model = modelOri;
3230     model.c(find(model.c)) = 0;
3231     for i = 1 : length(RPMI_composition)
3232         model = changeRxnBounds(model,RPMI_composition{i},-1,'l');
3233     end
3234     [model] = addSinkReactions(model,{'pail_hs(c)','gpi_prot_hs(r)'},[-1 -1; 0 100]);
3235     model.c(ismember(model.rxns,'sink_gpi_prot_hs(r)'))=1;
3236     if find(model.c)>0
3237         FBA = optimizeCbModel(model,'max');
3238         TestSolution(k,1) = FBA.f;
3239     else
3240         TestSolution(k,1) = NaN;
3241     end
3242     TestSolutionName{k,1} = 'pail_hs(c) -> gpi_prot_hs(r) (with RMPI medium)';
3243     if ~isnan(TestSolution(k,1)); TestedRxns = [TestedRxns; model.rxns(find(abs(FBA.x)>tol))]; end ;k = k +1;clear FBA
3244     %% pail45p -> mi145p
3245     model = modelOri;
3246     model.c(find(model.c)) = 0;
3247     [model] = addSinkReactions(model,{'pail45p_hs(c)','mi145p(c)'},[-1 -1; 0 100]);
3248     model.c(ismember(model.rxns,'sink_mi145p(c)'))=1;
3249     if find(model.c)>0
3250         FBA = optimizeCbModel(model,'max');
3251         TestSolution(k,1) = FBA.f;
3252     else
3253         TestSolution(k,1) = NaN;
3254     end
3255     TestSolutionName{k,1} = 'pail45p(c) -> mi145p(c)';
3256     if ~isnan(TestSolution(k,1)); TestedRxns = [TestedRxns; model.rxns(find(abs(FBA.x)>tol))]; end ;k = k +1;clear FBA
3257     %% phe-L -> pac
3258     model = modelOri;
3259     model.c(find(model.c)) = 0;
3260     [model] = addSinkReactions(model,{'phe-L(c)','pac(c)'},[-1 -1; 0 100]);
3261     model.c(ismember(model.rxns,'sink_pac(c)'))=1;
3262     if find(model.c)>0
3263         FBA = optimizeCbModel(model,'max');
3264         TestSolution(k,1) = FBA.f;
3265     else
3266         TestSolution(k,1) = NaN;
3267     end
3268     TestSolutionName{k,1} = 'phe-L(c) -> pac(c)';
3269     if ~isnan(TestSolution(k,1)); TestedRxns = [TestedRxns; model.rxns(find(abs(FBA.x)>tol))]; end ;k = k +1;clear FBA
3270     %% phe-L -> pacald
3271     model = modelOri;
3272     model.c(find(model.c)) = 0;
3273     [model] = addSinkReactions(model,{'phe-L(c)','pacald(c)'},[-1 -1; 0 100]);
3274     model.c(ismember(model.rxns,'sink_pacald(c)'))=1;
3275     if find(model.c)>0
3276         FBA = optimizeCbModel(model,'max');
3277         TestSolution(k,1) = FBA.f;
3278     else
3279         TestSolution(k,1) = NaN;
3280     end
3281     TestSolutionName{k,1} = 'phe-L(c) -> pacald(c)';
3282     if ~isnan(TestSolution(k,1)); TestedRxns = [TestedRxns; model.rxns(find(abs(FBA.x)>tol))]; end ;k = k +1;clear FBA
3283     %% phe-L -> peamn
3284     model = modelOri;
3285     model.c(find(model.c)) = 0;
3286     [model] = addSinkReactions(model,{'phe-L(c)','peamn(c)'},[-1 -1; 0 100]);
3287     model.c(ismember(model.rxns,'sink_peamn(c)'))=1;
3288     if find(model.c)>0
3289         FBA = optimizeCbModel(model,'max');
3290         TestSolution(k,1) = FBA.f;
3291     else
3292         TestSolution(k,1) = NaN;
3293     end
3294     TestSolutionName{k,1} = 'phe-L(c) -> peamn(c)';
3295     if ~isnan(TestSolution(k,1)); TestedRxns = [TestedRxns; model.rxns(find(abs(FBA.x)>tol))]; end ;k = k +1;clear FBA
3296     %% phe-L -> phaccoa
3297     model = modelOri;
3298     model.c(find(model.c)) = 0;
3299     model.lb(ismember(model.rxns,'EX_glc(e)'))=-1;model.ub(ismember(model.rxns,'EX_glc(e)'))=0;
3300     model.lb(ismember(model.rxns,'EX_o2(e)'))=-40;model.ub(ismember(model.rxns,'EX_o2(e)'))=-1;
3301     [model] = addSinkReactions(model,{'phe-L(c)','phaccoa(c)'},[-1 -1; 0 100]);
3302     model.lb(find(ismember(model.rxns,'sink_phe_L(c)')))=-1;
3303     model.ub(find(ismember(model.rxns,'sink_phe_L(c)')))=-1;
3304     model.lb(find(ismember(model.rxns,'sink_coa(c)')))=-1;
3305     model.ub(find(ismember(model.rxns,'sink_coa(c)')))=1;
3306     model.c(ismember(model.rxns,'sink_phaccoa(c)'))=1;
3307     if find(model.c)>0
3308         FBA = optimizeCbModel(model,'max');
3309         TestSolution(k,1) = FBA.f;
3310     else
3311         TestSolution(k,1) = NaN;
3312     end
3313     TestSolutionName{k,1} = 'phe-L(c) -> phaccoa(c)';
3314     if ~isnan(TestSolution(k,1)); TestedRxns = [TestedRxns; model.rxns(find(abs(FBA.x)>tol))]; end ;k = k +1;clear FBA
3315     %% phe-L -> pheacgln
3316     model = modelOri;
3317     model.c(find(model.c)) = 0;
3318     [model] = addSinkReactions(model,{'phe-L(c)','pheacgln(c)'},[-1 -1; 0 100]);
3319     model.lb(find(ismember(model.rxns,'sink_phe_L(c)')))=-1;
3320     model.ub(find(ismember(model.rxns,'sink_phe_L(c)')))=-1;
3321     model.c(ismember(model.rxns,'sink_pheacgln(c)'))=1;
3322     if find(model.c)>0
3323         FBA = optimizeCbModel(model,'max');
3324         TestSolution(k,1) = FBA.f;
3325     else
3326         TestSolution(k,1) = NaN;
3327     end
3328     TestSolutionName{k,1} = 'phe-L(c) -> pheacgln(c)';
3329     if ~isnan(TestSolution(k,1)); TestedRxns = [TestedRxns; model.rxns(find(abs(FBA.x)>tol))]; end ;k = k +1;clear FBA
3330     %% phe-L -> phpyr
3331     model = modelOri;
3332     model.c(find(model.c)) = 0;
3333     [model] = addSinkReactions(model,{'phe-L(c)','phpyr(c)'},[-1 -1; 0 100]);
3334     model.lb(find(ismember(model.rxns,'sink_phe_L(c)')))=-1;
3335     model.ub(find(ismember(model.rxns,'sink_phe_L(c)')))=-1;
3336     model.c(ismember(model.rxns,'sink_phpyr(c)'))=1;
3337     if find(model.c)>0
3338         FBA = optimizeCbModel(model,'max');
3339         TestSolution(k,1) = FBA.f;
3340     else
3341         TestSolution(k,1) = NaN;
3342     end
3343     TestSolutionName{k,1} = 'phe-L(c) -> phpyr(c)';
3344     if ~isnan(TestSolution(k,1)); TestedRxns = [TestedRxns; model.rxns(find(abs(FBA.x)>tol))]; end ;k = k +1;clear FBA
3345     %% phe-L -> tyr-L
3346     model = modelOri;
3347     model.c(find(model.c)) = 0;
3348     [model] = addSinkReactions(model,{'phe-L(c)','tyr-L(c)'},[-1 -1; 0 100]);
3349     model.lb(find(ismember(model.rxns,'sink_phe_L(c)')))=-1;
3350     model.ub(find(ismember(model.rxns,'sink_phe_L(c)')))=-1;
3351     model.c(ismember(model.rxns,'sink_tyr-L(c)'))=1;
3352     if find(model.c)>0
3353         FBA = optimizeCbModel(model,'max');
3354         TestSolution(k,1) = FBA.f;
3355     else
3356         TestSolution(k,1) = NaN;
3357     end
3358     TestSolutionName{k,1} = 'phe-L(c) -> tyr-L(c)';
3359     if ~isnan(TestSolution(k,1)); TestedRxns = [TestedRxns; model.rxns(find(abs(FBA.x)>tol))]; end ;k = k +1;clear FBA
3360     %% pheme -> bilirub %changing lb has no effect
3361     model = modelOri;
3362     model.c(find(model.c)) = 0;
3363     model.lb(ismember(model.rxns,'EX_glc(e)'))=-1;model.ub(ismember(model.rxns,'EX_glc(e)'))=0;
3364     model.lb(ismember(model.rxns,'EX_o2(e)'))=-40;model.ub(ismember(model.rxns,'EX_o2(e)'))=-1;
3365     [model] = addSinkReactions(model,{'pheme(c)','bilirub(c)'},[-1 -1; 0 100]);
3366     model.lb(find(ismember(model.rxns,'sink_pheme(c)')))=-1;
3367     model.ub(find(ismember(model.rxns,'sink_pheme(c)')))=-1;
3368     model.c(ismember(model.rxns,'sink_bilirub(c)'))=1;
3369     if find(model.c)>0
3370         FBA = optimizeCbModel(model,'max');
3371         TestSolution(k,1) = FBA.f;
3372     else
3373         TestSolution(k,1) = NaN;
3374     end
3375     TestSolutionName{k,1} = 'pheme(c) -> bilirub(c)';
3376     if ~isnan(TestSolution(k,1)); TestedRxns = [TestedRxns; model.rxns(find(abs(FBA.x)>tol))]; end ;k = k +1;clear FBA
3377     %% phytcoa(x) -> dmnoncoa(m)
3378     model = modelOri;
3379     model.c(find(model.c)) = 0;
3380     [model] = addSinkReactions(model,{'phytcoa(x)','dmnoncoa(m)'},[-1 -1; 0 100]);
3381     model.c(ismember(model.rxns,'sink_dmnoncoa(m)'))=1;
3382     if find(model.c)>0
3383         FBA = optimizeCbModel(model,'max');
3384         TestSolution(k,1) = FBA.f;
3385     else
3386         TestSolution(k,1) = NaN;
3387     end
3388     TestSolutionName{k,1} = 'phytcoa(x) -> dmnoncoa(m)';
3389     if ~isnan(TestSolution(k,1)); TestedRxns = [TestedRxns; model.rxns(find(abs(FBA.x)>tol))]; end ;k = k +1;clear FBA
3390     %% pmtcoa(c) -> crmp_hs(c)
3391     model = modelOri;
3392     for i = 1 : length(RPMI_composition)
3393         model = changeRxnBounds(model,RPMI_composition{i},-1,'l');
3394     end
3395     model.c(find(model.c)) = 0;
3396     [model] = addSinkReactions(model,{'pmtcoa(c)','crmp_hs(c)'},[-1 -1; 0 100]);
3397     model.c(ismember(model.rxns,'sink_crmp_hs(c)'))=1;
3398     if find(model.c)>0
3399         FBA = optimizeCbModel(model,'max');
3400         TestSolution(k,1) = FBA.f;
3401     else
3402         TestSolution(k,1) = NaN;
3403     end
3404     TestSolutionName{k,1} = 'pmtcoa(c) -> crmp_hs(c) (with RPMI medium)';
3405     if ~isnan(TestSolution(k,1)); TestedRxns = [TestedRxns; model.rxns(find(abs(FBA.x)>tol))]; end ;k = k +1;clear FBA
3406     %% pmtcoa(c) -> sphmyln_hs(c)
3407     model = modelOri;
3408     for i = 1 : length(RPMI_composition)
3409         model = changeRxnBounds(model,RPMI_composition{i},-1,'l');
3410     end
3411     model.c(find(model.c)) = 0;
3412     [model] = addSinkReactions(model,{'pmtcoa(c)','sphmyln_hs(c)'},[-1 -1; 0 100]);
3413     model.c(ismember(model.rxns,'sink_sphmyln_hs(c)'))=1;
3414     if find(model.c)>0
3415         FBA = optimizeCbModel(model,'max');
3416         TestSolution(k,1) = FBA.f;
3417     else
3418         TestSolution(k,1) = NaN;
3419     end
3420     TestSolutionName{k,1} = 'pmtcoa(c) -> sphmyln_hs(c) (with RPMI medium)';
3421     if ~isnan(TestSolution(k,1)); TestedRxns = [TestedRxns; model.rxns(find(abs(FBA.x)>tol))]; end ;k = k +1;clear FBA
3422     %% ppcoa[m] -> succoa[m]
3423     model = modelOri;
3424     model.c(find(model.c)) = 0;
3425     [model] = addSinkReactions(model,{'ppcoa(m)','succoa(m)'},[-1 -1; 0 100]);
3426     model.c(ismember(model.rxns,'sink_succoa(m)'))=1;
3427     if find(model.c)>0
3428         FBA = optimizeCbModel(model,'max');
3429         TestSolution(k,1) = FBA.f;
3430     else
3431         TestSolution(k,1) = NaN;
3432     end
3433     TestSolutionName{k,1} = 'ppcoa(m) -> succoa(m)';
3434     if ~isnan(TestSolution(k,1)); TestedRxns = [TestedRxns; model.rxns(find(abs(FBA.x)>tol))]; end ;k = k +1;clear FBA
3435     %% pro-L -> glu-L
3436     model = modelOri;
3437     model.c(find(model.c)) = 0;
3438     [model] = addSinkReactions(model,{'pro-L(c)','glu-L(c)'},[-1 -1; 0 100]);
3439     model.lb(find(ismember(model.rxns,'sink_pro_L(c)')))=-1;
3440     model.ub(find(ismember(model.rxns,'sink_pro_L(c)')))=-1;
3441     model.c(ismember(model.rxns,'sink_glu-L(c)'))=1;
3442     model.c(ismember(model.rxns,'sink_glu_L(c)'))=1;
3443     if find(model.c)>0
3444         FBA = optimizeCbModel(model,'max');
3445         TestSolution(k,1) = FBA.f;
3446     else
3447         TestSolution(k,1) = NaN;
3448     end
3449     TestSolutionName{k,1} = 'pro-L(c) -> glu-L(c)';
3450     if ~isnan(TestSolution(k,1)); TestedRxns = [TestedRxns; model.rxns(find(abs(FBA.x)>tol))]; end ;k = k +1;clear FBA
3451     %% ptrc -> ala-B
3452     model = modelOri;
3453     model.c(find(model.c)) = 0;
3454     [model] = addSinkReactions(model,{'ptrc(c)','ala-B(c)'},[-1 -1; 0 100]);
3455     model.c(ismember(model.rxns,'sink_ala-B(c)'))=1;
3456     if find(model.c)>0
3457         FBA = optimizeCbModel(model,'max');
3458         TestSolution(k,1) = FBA.f;
3459     else
3460         TestSolution(k,1) = NaN;
3461     end
3462     TestSolutionName{k,1} = 'ptrc(c) -> ala-B(c)';
3463     if ~isnan(TestSolution(k,1)); TestedRxns = [TestedRxns; model.rxns(find(abs(FBA.x)>tol))]; end ;k = k +1;clear FBA
3464     %% ptrc -> spmd
3465     model = modelOri;
3466     model.c(find(model.c)) = 0;
3467     [model] = addSinkReactions(model,{'ptrc(c)','spmd(c)'},[-1 -1; 0 100]);
3468     model.c(ismember(model.rxns,'sink_spmd(c)'))=1;
3469     if find(model.c)>0
3470         FBA = optimizeCbModel(model,'max');
3471         TestSolution(k,1) = FBA.f;
3472     else
3473         TestSolution(k,1) = NaN;
3474     end
3475     TestSolutionName{k,1} = 'ptrc(c) -> spmd(c)';
3476     if ~isnan(TestSolution(k,1)); TestedRxns = [TestedRxns; model.rxns(find(abs(FBA.x)>tol))]; end ;k = k +1;clear FBA
3477     %% pyr -> fad[m] + h[m] %changing lb has no effect
3478     model = modelOri;
3479     model.c(find(model.c)) = 0;
3480     for i = 1 : length(RPMI_composition)
3481         model = changeRxnBounds(model,RPMI_composition{i},-1,'l');
3482     end
3483     [model] = addSinkReactions(model,{'pyr(c)','fadh2(m)','fad(m)','h(m)'},[-1 -1;-1 0;  0.1 100; 0.1 100]);
3484     model.c(ismember(model.rxns,'sink_fad(m)'))=1;
3485     if find(model.c)>0
3486         FBA = optimizeCbModel(model,'max');
3487         TestSolution(k,1) = FBA.f;
3488     else
3489         TestSolution(k,1) = NaN;
3490     end
3491     TestSolutionName{k,1} = 'pyr -> fad[m] + h[m] (with RPMI medium)';
3492     if ~isnan(TestSolution(k,1)); TestedRxns = [TestedRxns; model.rxns(find(abs(FBA.x)>tol))]; end ;k = k +1;clear FBA
3493     %% pyr -> lac-D
3494     model = modelOri;
3495     model.c(find(model.c)) = 0;
3496     [model] = addSinkReactions(model,{'pyr(c)','lac-D(c)'},[-1 -1; 0 100]);
3497     model.c(ismember(model.rxns,'sink_lac-D(c)'))=1;
3498     if find(model.c)>0
3499         FBA = optimizeCbModel(model,'max');
3500         TestSolution(k,1) = FBA.f;
3501     else
3502         TestSolution(k,1) = NaN;
3503     end
3504     TestSolutionName{k,1} = 'pyr(c) -> lac-D(c)';
3505     if ~isnan(TestSolution(k,1)); TestedRxns = [TestedRxns; model.rxns(find(abs(FBA.x)>tol))]; end ;k = k +1;clear FBA
3506     %% pyr -> nad[m] + h[m] %changing lb has no effect
3507     model = modelOri;
3508     model.c(find(model.c)) = 0;
3509     for i = 1 : length(RPMI_composition)
3510         model = changeRxnBounds(model,RPMI_composition{i},-1,'l');
3511     end
3512     [model] = addSinkReactions(model,{'pyr(c)','nad(m)','h(m)'},[-1 -1; 0.1 100; 0.1 100]);
3513     model.c(ismember(model.rxns,'sink_nad(m)'))=1;
3514     if find(model.c)>0
3515         FBA = optimizeCbModel(model,'max');
3516         TestSolution(k,1) = FBA.f;
3517     else
3518         TestSolution(k,1) = NaN;
3519     end
3520     TestSolutionName{k,1} = 'pyr -> nad[m] + h[m] (with RPMI medium)';
3521     if ~isnan(TestSolution(k,1)); TestedRxns = [TestedRxns; model.rxns(find(abs(FBA.x)>tol))]; end ;k = k +1;clear FBA
3522     %% pyr[c] -> accoa[m] + co2[c] + nadh[m] %changing lb has no effect
3523     model = modelOri;
3524     model.c(find(model.c)) = 0;
3525     [model] = addSinkReactions(model,{'pyr(c)','accoa(m)','nadh(m)','co2(c)'},[-1 -1; 0.1 100; 0.1 100; 0.1 100]);
3526     model.lb(find(ismember(model.rxns,'sink_coa(c)')))=-1;
3527     model.ub(find(ismember(model.rxns,'sink_coa(c)')))=1;
3528     model.lb(find(ismember(model.rxns,'sink_nad(c)')))=-1;
3529     model.ub(find(ismember(model.rxns,'sink_nad(c)')))=1;
3530     model.c(ismember(model.rxns,'sink_accoa(m)'))=1;
3531     if find(model.c)>0
3532         FBA = optimizeCbModel(model,'max');
3533         TestSolution(k,1) = FBA.f;
3534     else
3535         TestSolution(k,1) = NaN;
3536     end
3537     TestSolutionName{k,1} = 'pyr[c] -> accoa[m] + co2(c) + nadh[m]';
3538     if ~isnan(TestSolution(k,1)); TestedRxns = [TestedRxns; model.rxns(find(abs(FBA.x)>tol))]; end ;k = k +1;clear FBA
3539     %% pyr<>ala-L
3540     model = modelOri;
3541     model.c(find(model.c)) = 0;
3542     [model] = addSinkReactions(model,{'pyr(c)','ala-L(c)'},[-1 -1; 0 100]);
3543     model.c(ismember(model.rxns,'sink_ala-L(c)'))=1;
3544     model.c(ismember(model.rxns,'sink_ala_L(c)'))=1;
3545     if find(model.c)>0
3546         FBA = optimizeCbModel(model,'max');
3547         TestSolution(k,1) = FBA.f;
3548     else
3549         TestSolution(k,1) = NaN;
3550     end
3551     TestSolutionName{k,1} = 'pyr(c) -> ala-L(c), 1';
3552     if ~isnan(TestSolution(k,1)); TestedRxns = [TestedRxns; model.rxns(find(abs(FBA.x)>tol))]; end ;k = k +1;clear FBA
3553     model = modelOri;
3554     model.c(find(model.c)) = 0;
3555     [model] = addSinkReactions(model,{'ala-L(c)','pyr(c)'},[-1 -1; 0 100]);
3556     model.lb(find(ismember(model.rxns,'sink_ala_L(c)')))=-1;
3557     model.ub(find(ismember(model.rxns,'sink_ala_L(c)')))=-1;
3558     model.c(ismember(model.rxns,'sink_pyr(c)'))=1;
3559     if find(model.c)>0
3560         FBA = optimizeCbModel(model,'max');
3561         TestSolution(k,1) = FBA.f;
3562     else
3563         TestSolution(k,1) = NaN;
3564     end
3565     TestSolutionName{k,1} = 'pyr(c) -> ala-L(c), 2';
3566     if ~isnan(TestSolution(k,1)); TestedRxns = [TestedRxns; model.rxns(find(abs(FBA.x)>tol))]; end ;k = k +1;clear FBA
3567     %% R_group
3568     %% s2l2fn2m2masn[l] -> man[l] + acgam[l]
3569     model = modelOri;
3570     model.c(find(model.c)) = 0;
3571     for i = 1 : length(RPMI_composition)
3572         model = changeRxnBounds(model,RPMI_composition{i},-1,'l');
3573     end
3574     [model] = addSinkReactions(model,{'s2l2fn2m2masn(l)','man(l)','acgam(l)'},[-1 -1; 0.1 100; 0.1 100]);
3575     model.c(ismember(model.rxns,'sink_man(l)'))=1;
3576     if find(model.c)>0
3577         FBA = optimizeCbModel(model,'max');
3578         TestSolution(k,1) = FBA.f;
3579     else
3580         TestSolution(k,1) = NaN;
3581     end
3582     TestSolutionName{k,1} = 's2l2fn2m2masn(l) -> man[l] + acgam[l] (with RPMI medium)';
3583     if ~isnan(TestSolution(k,1)); TestedRxns = [TestedRxns; model.rxns(find(abs(FBA.x)>tol))]; end ;k = k +1;clear FBA
3584     %% Ser/Thr[g] + udpacgal[g] -> core2[g] %changing lb has no effect
3585     model = modelOri;
3586     model.c(find(model.c)) = 0;
3587     for i = 1 : length(RPMI_composition)
3588         model = changeRxnBounds(model,RPMI_composition{i},-1,'l');
3589     end
3590     model = changeRxnBounds(model,'GALNTg',0.1,'l');
3591     [model] = addSinkReactions(model,{'Ser/Thr(g)';'udpacgal(g)';'core2(g)'},[-1 -1; -1 -1; 0 100]);
3592     model.c(ismember(model.rxns,'sink_core2(g)'))=1;
3593     if find(model.c)>0
3594         FBA = optimizeCbModel(model,'max');
3595         TestSolution(k,1) = FBA.f;
3596     else
3597         TestSolution(k,1) = NaN;
3598     end
3599     TestSolutionName{k,1} = 'Ser/Thr[g] + udpacgal[g] -> core2[g] - via GALNTg and DM_core4[g] (with RPMI medium)';
3600     if ~isnan(TestSolution(k,1)); TestedRxns = [TestedRxns; model.rxns(find(abs(FBA.x)>tol))]; end ;k = k +1;clear FBA
3601     %% Ser/Thr[g] + udpacgal[g] -> core4[g] %changing lb has no effect
3602     model = modelOri;
3603     model.c(find(model.c)) = 0;
3604     for i = 1 : length(RPMI_composition)
3605         model = changeRxnBounds(model,RPMI_composition{i},-1,'l');
3606     end
3607     model = changeRxnBounds(model,'GALNTg',0.1,'l');
3608     [model] = addSinkReactions(model,{'Ser/Thr(g)';'udpacgal(g)';'core4(g)'},[-1 -1; -1 -1; 0 100]);
3609     model.c(ismember(model.rxns,'sink_core4(g)'))=1;
3610     if find(model.c)>0
3611         FBA = optimizeCbModel(model,'max');
3612         TestSolution(k,1) = FBA.f;
3613     else
3614         TestSolution(k,1) = NaN;
3615     end
3616     TestSolutionName{k,1} = 'Ser/Thr[g] + udpacgal[g] -> core4[g] - via GALNTg and DM_core4[g] (with RPMI medium)';
3617     if ~isnan(TestSolution(k,1)); TestedRxns = [TestedRxns; model.rxns(find(abs(FBA.x)>tol))]; end ;k = k +1;clear FBA
3618     %% Ser/Thr[g] + udpacgal[g] -> dsTn_antigen[g] % dsTn_antigen does not exists
3619     % model = modelOri;
3620     % model.c(find(model.c)) = 0;
3621     % [model] = addSinkReactions(model,{'Ser/Thr(g)','udpacgal(g)','dsTn_antigen(g)'},[-1 -1;-1 -1; 0 100]);
3622     % model.c(ismember(model.rxns,'sink_dsTn_antigen(g)'))=1;
3623     %   FBA = optimizeCbModel(model,'max');
3624     % TestSolution(k,1) = FBA.f;
3625     % TestSolutionName{k,1} = 'Ser/Thr[g] + udpacgal[g] -> dsTn_antigen[g]';
3626     % k = k +1;clear FBA
3627     %% Ser/Thr[g] + udpacgal[g] -> Tn_antigen[g] % dsTn_antigen does not exists
3628     % - I used Tn_antigen instead %changing lb has no effect
3629     model = modelOri;
3630     model.c(find(model.c)) = 0;
3631     for i = 1 : length(RPMI_composition)
3632         model = changeRxnBounds(model,RPMI_composition{i},-1,'l');
3633     end
3634     [model] = addSinkReactions(model,{'Ser/Thr(g)','udpacgal(g)','Tn_antigen(g)'},[-1 -1;-1 -1; 0 100]);
3635     % model.c(ismember(model.rxns,'sink_Tn_antigen(g)'))=1;
3636     if ~isempty(strmatch('GALNTg',model.rxns,'exact'))
3637         model = changeObjective(model, 'GALNTg',1);
3638         FBA = optimizeCbModel(model,'max');
3639         TestSolution(k,1) = FBA.f;
3640     else
3641         TestSolution(k,1) = NaN;
3642     end
3643     TestSolutionName{k,1} = 'Ser/Thr[g] + udpacgal[g] -> Tn_antigen[g] - via GALNTg (with RPMI medium)';
3644     if ~isnan(TestSolution(k,1)); TestedRxns = [TestedRxns; model.rxns(find(abs(FBA.x)>tol))]; end ;k = k +1;clear FBA
3645     
3646     %% Ser/Thr[g] + udpacgal[g] -> sTn_antigen[g] %changing lb has no effect
3647     model = modelOri;
3648     model.c(find(model.c)) = 0;
3649     for i = 1 : length(RPMI_composition)
3650         model = changeRxnBounds(model,RPMI_composition{i},-1,'l');
3651     end
3652     if ~isempty(strmatch('GALNTg',model.rxns,'exact'))
3653         model = changeRxnBounds(model,'GALNTg',0.1,'l');
3654         [model] = addSinkReactions(model,{'Ser/Thr(g)','udpacgal(g)','sTn_antigen(g)'},[-1 -1;-1 -1; 0 100]);
3655         if (rxnsInModel(1) >-1) % reaction exits already in model
3656             model=changeObjective(model,model.rxns(rxnsInModel(1),1));
3657         else
3658             model=changeObjective(model,'sink_sTn_antigen(g)',1);
3659         end
3660         if find(model.c)>0
3661             FBA = optimizeCbModel(model,'max');
3662             TestSolution(k,1) = FBA.f;
3663         else
3664             TestSolution(k,1) = NaN;
3665         end
3666         TestSolutionName{k,1} = 'Ser/Thr[g] + udpacgal[g] -> sTn_antigen[g] - via GALNTg and DM_sTn_antigen(g) (with RPMI medium)';
3667         if ~isnan(TestSolution(k,1)); TestedRxns = [TestedRxns; model.rxns(find(abs(FBA.x)>tol))]; end ;k = k +1;clear FBA
3668     else
3669         TestSolution(k,1) = NaN;
3670         TestSolutionName{k,1} = 'Ser/Thr[g] + udpacgal[g] -> sTn_antigen[g] - via GALNTg and DM_sTn_antigen(g)';
3671         if ~isnan(TestSolution(k,1)); TestedRxns = [TestedRxns; model.rxns(find(abs(FBA.x)>tol))]; end ;k = k +1;clear FBA
3672     end
3673     %% Ser-Gly/Ala-X-Gly[er] -> cs_pre[g]
3674     model = modelOri;
3675     model.c(find(model.c)) = 0;
3676     model.lb(ismember(model.rxns,'EX_glc(e)'))=-1;model.ub(ismember(model.rxns,'EX_glc(e)'))=0;
3677     model.lb(ismember(model.rxns,'EX_o2(e)'))=-40;model.ub(ismember(model.rxns,'EX_o2(e)'))=-1;
3678     [model] = addSinkReactions(model,{'Ser-Gly/Ala-X-Gly(r)','cs_pre(g)'},[-1 -1; 0 100]);
3679     model.c(ismember(model.rxns,'sink_cs_pre(g)'))=1;
3680     if find(model.c)>0
3681         FBA = optimizeCbModel(model,'max');
3682         TestSolution(k,1) = FBA.f;
3683     else
3684         TestSolution(k,1) = NaN;
3685     end
3686     TestSolutionName{k,1} = 'Ser-Gly/Ala-X-Gly[r] -> cs_pre[g]';
3687     if ~isnan(TestSolution(k,1)); TestedRxns = [TestedRxns; model.rxns(find(abs(FBA.x)>tol))]; end ;k = k +1;clear FBA
3688     %% Ser-Gly/Ala-X-Gly[er] -> cspg_a[g]
3689     model = modelOri;
3690     model.c(find(model.c)) = 0;
3691     model.lb(ismember(model.rxns,'EX_glc(e)'))=-1;model.ub(ismember(model.rxns,'EX_glc(e)'))=0;
3692     model.lb(ismember(model.rxns,'EX_o2(e)'))=-40;model.ub(ismember(model.rxns,'EX_o2(e)'))=-1;
3693     [model] = addSinkReactions(model,{'Ser-Gly/Ala-X-Gly(r)','cspg_a(g)'},[-1 -1; 0 100]);
3694     model.c(ismember(model.rxns,'sink_cspg_a(g)'))=1;
3695     if find(model.c)>0
3696         FBA = optimizeCbModel(model,'max');
3697         TestSolution(k,1) = FBA.f;
3698     else
3699         TestSolution(k,1) = NaN;
3700     end
3701     TestSolutionName{k,1} = 'Ser-Gly/Ala-X-Gly[r] -> cspg_a[g]';
3702     if ~isnan(TestSolution(k,1)); TestedRxns = [TestedRxns; model.rxns(find(abs(FBA.x)>tol))]; end ;k = k +1;clear FBA
3703     %% Ser-Gly/Ala-X-Gly[er] -> cspg_c[g]
3704     model = modelOri;
3705     model.c(find(model.c)) = 0;
3706     model.lb(ismember(model.rxns,'EX_glc(e)'))=-1;model.ub(ismember(model.rxns,'EX_glc(e)'))=0;
3707     model.lb(ismember(model.rxns,'EX_o2(e)'))=-40;model.ub(ismember(model.rxns,'EX_o2(e)'))=-1;
3708     [model] = addSinkReactions(model,{'Ser-Gly/Ala-X-Gly(r)','cspg_c(g)'},[-1 -1; 0 100]);
3709     model.c(ismember(model.rxns,'sink_cspg_c(g)'))=1;
3710     if find(model.c)>0
3711         FBA = optimizeCbModel(model,'max');
3712         TestSolution(k,1) = FBA.f;
3713     else
3714         TestSolution(k,1) = NaN;
3715     end
3716     TestSolutionName{k,1} = 'Ser-Gly/Ala-X-Gly[r] -> cspg_c[g]';
3717     if ~isnan(TestSolution(k,1)); TestedRxns = [TestedRxns; model.rxns(find(abs(FBA.x)>tol))]; end ;k = k +1;clear FBA
3718     %% Ser-Gly/Ala-X-Gly[er] -> cspg_d[g]
3719     model = modelOri;
3720     model.c(find(model.c)) = 0;
3721     model.lb(ismember(model.rxns,'EX_glc(e)'))=-1;model.ub(ismember(model.rxns,'EX_glc(e)'))=0;
3722     model.lb(ismember(model.rxns,'EX_o2(e)'))=-40;model.ub(ismember(model.rxns,'EX_o2(e)'))=-1;
3723     [model] = addSinkReactions(model,{'Ser-Gly/Ala-X-Gly(r)','cspg_d(g)'},[-1 -1; 0 100]);
3724     model.c(ismember(model.rxns,'sink_cspg_d(g)'))=1;
3725     if find(model.c)>0
3726         FBA = optimizeCbModel(model,'max');
3727         TestSolution(k,1) = FBA.f;
3728     else
3729         TestSolution(k,1) = NaN;
3730     end
3731     TestSolutionName{k,1} = 'Ser-Gly/Ala-X-Gly[r] -> cspg_d[g]';
3732     if ~isnan(TestSolution(k,1)); TestedRxns = [TestedRxns; model.rxns(find(abs(FBA.x)>tol))]; end ;k = k +1;clear FBA
3733     %% Ser-Gly/Ala-X-Gly[er] -> cspg_e[g]
3734     model = modelOri;
3735     model.c(find(model.c)) = 0;
3736     model.lb(ismember(model.rxns,'EX_glc(e)'))=-1;model.ub(ismember(model.rxns,'EX_glc(e)'))=0;
3737     model.lb(ismember(model.rxns,'EX_o2(e)'))=-40;model.ub(ismember(model.rxns,'EX_o2(e)'))=-1;
3738     [model] = addSinkReactions(model,{'Ser-Gly/Ala-X-Gly(r)','cspg_e(g)'},[-1 -1; 0 100]);
3739     model.c(ismember(model.rxns,'sink_cspg_e(g)'))=1;
3740     if find(model.c)>0
3741         FBA = optimizeCbModel(model,'max');
3742         TestSolution(k,1) = FBA.f;
3743     else
3744         TestSolution(k,1) = NaN;
3745     end
3746     TestSolutionName{k,1} = 'Ser-Gly/Ala-X-Gly[r] -> cspg_e[g]';
3747     if ~isnan(TestSolution(k,1)); TestedRxns = [TestedRxns; model.rxns(find(abs(FBA.x)>tol))]; end ;k = k +1;clear FBA
3748     %% Ser-Gly/Ala-X-Gly[er] -> hspg[g]
3749     model = modelOri;
3750     model.c(find(model.c)) = 0;
3751     model.lb(ismember(model.rxns,'EX_glc(e)'))=-1;model.ub(ismember(model.rxns,'EX_glc(e)'))=0;
3752     model.lb(ismember(model.rxns,'EX_o2(e)'))=-40;model.ub(ismember(model.rxns,'EX_o2(e)'))=-1;
3753     [model] = addSinkReactions(model,{'Ser-Gly/Ala-X-Gly(r)','hspg(g)'},[-1 -1; 0 100]);
3754     model.c(ismember(model.rxns,'sink_hspg(g)'))=1;
3755     if find(model.c)>0
3756         FBA = optimizeCbModel(model,'max');
3757         TestSolution(k,1) = FBA.f;
3758     else
3759         TestSolution(k,1) = NaN;
3760     end
3761     TestSolutionName{k,1} = 'Ser-Gly/Ala-X-Gly[r] -> hspg[g]';
3762     if ~isnan(TestSolution(k,1)); TestedRxns = [TestedRxns; model.rxns(find(abs(FBA.x)>tol))]; end ;k = k +1;clear FBA
3763     %% Ser-Gly/Ala-X-Ser[er] -> cspg_b[g]
3764     model = modelOri;
3765     model.c(find(model.c)) = 0;
3766     model.lb(ismember(model.rxns,'EX_glc(e)'))=-1;model.ub(ismember(model.rxns,'EX_glc(e)'))=0;
3767     model.lb(ismember(model.rxns,'EX_o2(e)'))=-40;model.ub(ismember(model.rxns,'EX_o2(e)'))=-1;
3768     [model] = addSinkReactions(model,{'Ser-Gly/Ala-X-Gly(r)','cspg_b(g)'},[-1 -1; 0 100]);
3769     model.c(ismember(model.rxns,'sink_cspg_b(g)'))=1;
3770     if find(model.c)>0
3771         FBA = optimizeCbModel(model,'max');
3772         TestSolution(k,1) = FBA.f;
3773     else
3774         TestSolution(k,1) = NaN;
3775     end
3776     TestSolutionName{k,1} = 'Ser-Gly/Ala-X-Gly[r] -> cspg_b[g]';
3777     if ~isnan(TestSolution(k,1)); TestedRxns = [TestedRxns; model.rxns(find(abs(FBA.x)>tol))]; end ;k = k +1;clear FBA
3778     %% ser-L -> cys-L
3779     model = modelOri;
3780     model.c(find(model.c)) = 0;
3781     model.lb(ismember(model.rxns,'EX_glc(e)'))=-1;model.ub(ismember(model.rxns,'EX_glc(e)'))=0;
3782     model.lb(ismember(model.rxns,'EX_o2(e)'))=-40;model.ub(ismember(model.rxns,'EX_o2(e)'))=-1;
3783     [model] = addSinkReactions(model,{'ser-L(c)','cys-L(c)'},[-1 -1; 0 100]);
3784     model.lb(find(ismember(model.rxns,'sink_ser_L(c)')))=-1;
3785     model.ub(find(ismember(model.rxns,'sink_ser_L(c)')))=-1;
3786     model.c(ismember(model.rxns,'sink_cys-L(c)'))=1;
3787     model.c(ismember(model.rxns,'sink_cys_L(c)'))=1;
3788     if find(model.c)>0
3789         FBA = optimizeCbModel(model,'max');
3790         TestSolution(k,1) = FBA.f;
3791     else
3792         TestSolution(k,1) = NaN;
3793     end
3794     TestSolutionName{k,1} = 'ser-L(c) -> cys-L(c)';
3795     if ~isnan(TestSolution(k,1)); TestedRxns = [TestedRxns; model.rxns(find(abs(FBA.x)>tol))]; end ;k = k +1;clear FBA
3796     %% so4 -> PAPS
3797     model = modelOri;
3798     model.c(find(model.c)) = 0;
3799     model.lb(ismember(model.rxns,'EX_glc(e)'))=-1;model.ub(ismember(model.rxns,'EX_glc(e)'))=0;
3800     model.lb(ismember(model.rxns,'EX_o2(e)'))=-40;model.ub(ismember(model.rxns,'EX_o2(e)'))=-1;
3801     [model] = addSinkReactions(model,{'so4(c)','paps(c)'},[-1 -1; 0 100]);
3802     model.c(ismember(model.rxns,'sink_paps(c)'))=1;
3803     if find(model.c)>0
3804         FBA = optimizeCbModel(model,'max');
3805         TestSolution(k,1) = FBA.f;
3806     else
3807         TestSolution(k,1) = NaN;
3808     end
3809     TestSolutionName{k,1} = 'so4(c) -> paps(c)';
3810     if ~isnan(TestSolution(k,1)); TestedRxns = [TestedRxns; model.rxns(find(abs(FBA.x)>tol))]; end ;k = k +1;clear FBA
3811     %% spmd -> sprm
3812     model = modelOri;
3813     model.c(find(model.c)) = 0;
3814     [model,rxnsInModel] = addSinkReactions(model,{'spmd(c)','sprm(c)'},[-1 -1; 0 100]);
3815     %  model.c(ismember(model.rxns,'sink_sprm(c)'))=1;
3816     if (rxnsInModel(2) >-1) % reaction exits already in model
3817         model=changeObjective(model,model.rxns(rxnsInModel(2),1));
3818     else
3819         model=changeObjective(model,'sink_sprm(c)',1);
3820     end
3821     if find(model.c)>0
3822         FBA = optimizeCbModel(model,'max');
3823         TestSolution(k,1) = FBA.f;
3824     else
3825         TestSolution(k,1) = NaN;
3826     end
3827     TestSolutionName{k,1} = 'spmd(c) -> sprm(c)';
3828     if ~isnan(TestSolution(k,1)); TestedRxns = [TestedRxns; model.rxns(find(abs(FBA.x)>tol))]; end ;k = k +1;clear FBA
3829     %% srtn -> f5hoxkyn
3830     model = modelOri;
3831     model.c(find(model.c)) = 0;
3832     model.lb(ismember(model.rxns,'EX_glc(e)'))=-1;model.ub(ismember(model.rxns,'EX_glc(e)'))=0;
3833     [model] = addSinkReactions(model,{'srtn(c)','f5hoxkyn(c)'},[-1 -1; 0 100]);
3834     model.lb(find(ismember(model.rxns,'DM_srtn(c)')))=-1;
3835     model.ub(find(ismember(model.rxns,'DM_srtn(c)')))=-1;
3836     model.c(ismember(model.rxns,'sink_f5hoxkyn(c)'))=1;
3837     if find(model.c)>0
3838         FBA = optimizeCbModel(model,'max');
3839         TestSolution(k,1) = FBA.f;
3840     else
3841         TestSolution(k,1) = NaN;
3842     end
3843     TestSolutionName{k,1} = 'srtn(c) -> f5hoxkyn(c)';
3844     if ~isnan(TestSolution(k,1)); TestedRxns = [TestedRxns; model.rxns(find(abs(FBA.x)>tol))]; end ;k = k +1;clear FBA
3845     %% srtn -> fna5moxam
3846     model = modelOri;
3847     model.c(find(model.c)) = 0;
3848     model.lb(ismember(model.rxns,'EX_glc(e)'))=-1;model.ub(ismember(model.rxns,'EX_glc(e)'))=0;
3849     [model] = addSinkReactions(model,{'srtn(c)','fna5moxam(c)'},[-1 -1; 0 100]);
3850     model.lb(find(ismember(model.rxns,'DM_srtn(c)')))=-1;
3851     model.ub(find(ismember(model.rxns,'DM_srtn(c)')))=-1;
3852     model.c(ismember(model.rxns,'sink_fna5moxam(c)'))=1;
3853     if find(model.c)>0
3854         FBA = optimizeCbModel(model,'max');
3855         TestSolution(k,1) = FBA.f;
3856     else
3857         TestSolution(k,1) = NaN;
3858     end
3859     TestSolutionName{k,1} = 'srtn(c) -> fna5moxam(c)';
3860     if ~isnan(TestSolution(k,1)); TestedRxns = [TestedRxns; model.rxns(find(abs(FBA.x)>tol))]; end ;k = k +1;clear FBA
3861     %% srtn -> nmthsrtn
3862     model = modelOri;
3863     model.c(find(model.c)) = 0;
3864     [model] = addSinkReactions(model,{'srtn(c)','nmthsrtn(c)'},[-1 -1; 0 100]);
3865     model.lb(find(ismember(model.rxns,'DM_srtn(c)')))=-1;
3866     model.ub(find(ismember(model.rxns,'DM_srtn(c)')))=-1;
3867     model.c(find(ismember(model.rxns,'sink_nmthsrtn(c)')))=1;
3868     if find(model.c)>0
3869         FBA = optimizeCbModel(model,'max');
3870         TestSolution(k,1) = FBA.f;
3871     else
3872         TestSolution(k,1) = NaN;
3873     end
3874     TestSolutionName{k,1} = 'srtn(c) -> nmthsrtn(c)';
3875     if ~isnan(TestSolution(k,1)); TestedRxns = [TestedRxns; model.rxns(find(abs(FBA.x)>tol))]; end ;k = k +1;clear FBA
3876     %% strch1[e] -> glc[e]
3877     model = modelOri;
3878     model.c(find(model.c)) = 0;
3879     model = changeRxnBounds(model,'EX_strch1(e)',-1,'l');
3880     model = changeRxnBounds(model,'EX_strch1(e)',-1,'u');
3881     model = changeRxnBounds(model,'EX_glc(e)',0,'l');
3882     model = changeRxnBounds(model,'EX_glc(e)',1000,'u');
3883     model.lb(ismember(model.rxns,'EX_o2(e)'))=-40;model.ub(ismember(model.rxns,'EX_o2(e)'))=0;
3884     if ~isempty(strmatch('AMY1e',model.rxns,'exact'))
3885         model.c(ismember(model.rxns,'AMY1e'))=1;
3886         FBA = optimizeCbModel(model,'max');
3887         TestSolution(k,1) = FBA.f;
3888     else
3889         TestSolution(k,1) = NaN;
3890     end
3891     TestSolutionName{k,1} = 'strch1(e) -> glc-D(e) via AMY1e';
3892     if ~isnan(TestSolution(k,1)); TestedRxns = [TestedRxns; model.rxns(find(abs(FBA.x)>tol))]; end ;k = k +1;clear FBA
3893     
3894     %% succoa[m] -> oaa[m]
3895     model = modelOri;
3896     model.c(find(model.c)) = 0;
3897     [model] = addSinkReactions(model,{'succoa(m)','oaa(m)'},[-1 -1; 0 100]);
3898     model.c(ismember(model.rxns,'sink_oaa(m)'))=1;
3899     if find(model.c)>0
3900         FBA = optimizeCbModel(model,'max');
3901         TestSolution(k,1) = FBA.f;
3902     else
3903         TestSolution(k,1) = NaN;
3904     end
3905     TestSolutionName{k,1} = 'succoa(m) -> oaa(m)';
3906     if ~isnan(TestSolution(k,1)); TestedRxns = [TestedRxns; model.rxns(find(abs(FBA.x)>tol))]; end ;k = k +1;clear FBA
3907     %% taur(x) -> tchola(x)
3908     model = modelOri;
3909     model.c(find(model.c)) = 0;
3910     model.lb(ismember(model.rxns,'EX_glc(e)'))=-1;model.ub(ismember(model.rxns,'EX_glc(e)'))=0;
3911     model.lb(ismember(model.rxns,'EX_o2(e)'))=-40;model.ub(ismember(model.rxns,'EX_o2(e)'))=-1;
3912     [model] = addSinkReactions(model,{'taur(x)','tchola(x)'},[-1 -1; 0 100]);
3913     model.c(ismember(model.rxns,'sink_tchola(x)'))=1;
3914     if find(model.c)>0
3915         FBA = optimizeCbModel(model,'max');
3916         TestSolution(k,1) = FBA.f;
3917     else
3918         TestSolution(k,1) = NaN;
3919     end
3920     TestSolutionName{k,1} = 'taur(x) -> tchola(x)';
3921     if ~isnan(TestSolution(k,1)); TestedRxns = [TestedRxns; model.rxns(find(abs(FBA.x)>tol))]; end ;k = k +1;clear FBA
3922     %% thcholstoic(x) -> gchola(x)
3923     model = modelOri;
3924     model.c(find(model.c)) = 0;
3925     model.lb(ismember(model.rxns,'EX_glc(e)'))=-1;model.ub(ismember(model.rxns,'EX_glc(e)'))=0;
3926     model.lb(ismember(model.rxns,'EX_o2(e)'))=-40;model.ub(ismember(model.rxns,'EX_o2(e)'))=-1;
3927     [model] = addSinkReactions(model,{'thcholstoic(x)','gchola(x)'},[-1 -1; 0 100]);
3928     model.c(ismember(model.rxns,'sink_gchola(x)'))=1;
3929     if find(model.c)>0
3930         FBA = optimizeCbModel(model,'max');
3931         TestSolution(k,1) = FBA.f;
3932     else
3933         TestSolution(k,1) = NaN;
3934     end
3935     TestSolutionName{k,1} = 'thcholstoic(x) -> gchola(x)';
3936     if ~isnan(TestSolution(k,1)); TestedRxns = [TestedRxns; model.rxns(find(abs(FBA.x)>tol))]; end ;k = k +1;clear FBA
3937     %% thcholstoic(x) -> tchola(x)
3938     model = modelOri;
3939     model.c(find(model.c)) = 0;
3940     model.lb(ismember(model.rxns,'EX_glc(e)'))=-1;model.ub(ismember(model.rxns,'EX_glc(e)'))=0;
3941     model.lb(ismember(model.rxns,'EX_o2(e)'))=-40;model.ub(ismember(model.rxns,'EX_o2(e)'))=-1;
3942     [model] = addSinkReactions(model,{'thcholstoic(x)','tchola(x)'},[-1 -1; 0 100]);
3943     model.c(ismember(model.rxns,'sink_tchola(x)'))=1;
3944     if find(model.c)>0
3945         FBA = optimizeCbModel(model,'max');
3946         TestSolution(k,1) = FBA.f;
3947     else
3948         TestSolution(k,1) = NaN;
3949     end
3950     TestSolutionName{k,1} = 'thcholstoic(x) -> tchola(x)';
3951     if ~isnan(TestSolution(k,1)); TestedRxns = [TestedRxns; model.rxns(find(abs(FBA.x)>tol))]; end ;k = k +1;clear FBA
3952     %% thcholstoich(x) -> tchola(x) % thcholstoich does not exist in model
3953     % model = modelOri;
3954     % model.c(find(model.c)) = 0;
3955     % [model] = addSinkReactions(model,{'thcholstoich(x)','tchola(x)'},[-1 -1; 0 100]);
3956     % model.c(ismember(model.rxns,'sink_tchola(x)'))=1;
3957     %   FBA = optimizeCbModel(model,'max');
3958     % TestSolution(k,1) = FBA.f;
3959     % TestSolutionName{k,1} = 'thcholstoich(x) -> tchola(x)';
3960     % k = k +1;clear FBA
3961     %% thr-L -> ppcoa
3962     model = modelOri;
3963     model.c(find(model.c)) = 0;
3964     model.lb(ismember(model.rxns,'EX_o2(e)'))=-40;model.ub(ismember(model.rxns,'EX_o2(e)'))=-1;
3965     [model] = addSinkReactions(model,{'trp-L(c)','ppcoa(c)'},[-1 -1; 0 100]);
3966     model.lb(find(ismember(model.rxns,'sink_trp_L(c)')))=-1;
3967     model.ub(find(ismember(model.rxns,'sink_trp_L(c)')))=-1;
3968     model.lb(find(ismember(model.rxns,'sink_coa(c)')))=-1;
3969     model.ub(find(ismember(model.rxns,'sink_coa(c)')))=1;
3970     model.c(ismember(model.rxns,'sink_ppcoa(c)'))=1;
3971     if find(model.c)>0
3972         FBA = optimizeCbModel(model,'max');
3973         TestSolution(k,1) = FBA.f;
3974     else
3975         TestSolution(k,1) = NaN;
3976     end
3977     TestSolutionName{k,1} = 'trp-L(c) -> ppcoa(c)';
3978     if ~isnan(TestSolution(k,1)); TestedRxns = [TestedRxns; model.rxns(find(abs(FBA.x)>tol))]; end ;k = k +1;clear FBA
3979     %% trp-L -> accoa
3980     model = modelOri;
3981     model.c(find(model.c)) = 0;
3982     model.lb(ismember(model.rxns,'EX_o2(e)'))=-40;model.ub(ismember(model.rxns,'EX_o2(e)'))=-1;
3983     [model] = addSinkReactions(model,{'trp-L(c)','accoa(c)'},[-1 -1; 0 100]);
3984     model.lb(find(ismember(model.rxns,'sink_trp_L(c)')))=-1;
3985     model.ub(find(ismember(model.rxns,'sink_trp_L(c)')))=-1;
3986     model.lb(find(ismember(model.rxns,'sink_coa(c)')))=-1;
3987     model.ub(find(ismember(model.rxns,'sink_coa(c)')))=1;
3988     model.c(ismember(model.rxns,'sink_accoa(c)'))=1;
3989     if find(model.c)>0
3990         FBA = optimizeCbModel(model,'max');
3991         TestSolution(k,1) = FBA.f;
3992     else
3993         TestSolution(k,1) = NaN;
3994     end
3995     TestSolutionName{k,1} = 'trp-L(c) -> accoa(c)';
3996     if ~isnan(TestSolution(k,1)); TestedRxns = [TestedRxns; model.rxns(find(abs(FBA.x)>tol))]; end ;k = k +1;clear FBA
3997     %% trp-L -> anth
3998     model = modelOri;
3999     model.c(find(model.c)) = 0;
4000     [model,rxnsInModel] = addSinkReactions(model,{'trp-L(c)','anth(c)'},[-1 -1; 0 100]);
4001     model.lb(find(ismember(model.rxns,'sink_trp_L(c)')))=-1;
4002     model.ub(find(ismember(model.rxns,'sink_trp_L(c)')))=-1;
4003     if (rxnsInModel(2) >-1) % reaction exits already in model
4004         model=changeObjective(model,model.rxns(rxnsInModel(2),1));
4005     else
4006         model=changeObjective(model,'sink_anth(c)',1);
4007     end
4008     if find(model.c)>0
4009         FBA = optimizeCbModel(model,'max');
4010         TestSolution(k,1) = FBA.f;
4011     else
4012         TestSolution(k,1) = NaN;
4013     end
4014     TestSolutionName{k,1} = 'trp-L(c) -> anth(c)';
4015     if ~isnan(TestSolution(k,1)); TestedRxns = [TestedRxns; model.rxns(find(abs(FBA.x)>tol))]; end ;k = k +1;clear FBA
4016     %% trp-L -> id3acald
4017     model = modelOri;
4018     model.c(find(model.c)) = 0;
4019     [model] = addSinkReactions(model,{'trp-L(c)','id3acald(c)'},[-1 -1; 0 100]);
4020     model.lb(find(ismember(model.rxns,'sink_trp_L(c)')))=-1;
4021     model.ub(find(ismember(model.rxns,'sink_trp_L(c)')))=-1;
4022     model.c(ismember(model.rxns,'sink_id3acald(c)'))=1;
4023     if find(model.c)>0
4024         FBA = optimizeCbModel(model,'max');
4025         TestSolution(k,1) = FBA.f;
4026     else
4027         TestSolution(k,1) = NaN;
4028     end
4029     TestSolutionName{k,1} = 'trp-L(c) -> id3acald(c)';
4030     if ~isnan(TestSolution(k,1)); TestedRxns = [TestedRxns; model.rxns(find(abs(FBA.x)>tol))]; end ;k = k +1;clear FBA
4031     %% trp-L -> kynate
4032     model = modelOri;
4033     model.c(find(model.c)) = 0;
4034     [model] = addSinkReactions(model,{'trp-L(c)','kynate(c)'},[-1 -1; 0 100]);
4035     model.lb(find(ismember(model.rxns,'sink_trp_L(c)')))=-1;
4036     model.ub(find(ismember(model.rxns,'sink_trp_L(c)')))=-1;
4037     model.c(ismember(model.rxns,'sink_kynate(c)'))=1;
4038     if find(model.c)>0
4039         FBA = optimizeCbModel(model,'max');
4040         TestSolution(k,1) = FBA.f;
4041     else
4042         TestSolution(k,1) = NaN;
4043     end
4044     TestSolutionName{k,1} = 'trp-L(c) -> kynate(c)';
4045     if ~isnan(TestSolution(k,1)); TestedRxns = [TestedRxns; model.rxns(find(abs(FBA.x)>tol))]; end ;k = k +1;clear FBA
4046     %% trp-L -> melatn
4047     model = modelOri;
4048     model.c(find(model.c)) = 0;
4049     [model] = addSinkReactions(model,{'trp-L(c)','melatn(c)'},[-1 -1; 0 100]);
4050     model.lb(find(ismember(model.rxns,'sink_trp_L(c)')))=-1;
4051     model.ub(find(ismember(model.rxns,'sink_trp_L(c)')))=-1;
4052     model.c(ismember(model.rxns,'sink_melatn(c)'))=1;
4053     if find(model.c)>0
4054         FBA = optimizeCbModel(model,'max');
4055         TestSolution(k,1) = FBA.f;
4056     else
4057         TestSolution(k,1) = NaN;
4058     end
4059     TestSolutionName{k,1} = 'trp-L(c) -> melatn(c)';
4060     if ~isnan(TestSolution(k,1)); TestedRxns = [TestedRxns; model.rxns(find(abs(FBA.x)>tol))]; end ;k = k +1;clear FBA
4061     %% trp-L -> melatn
4062     model = modelOri;
4063     model.c(find(model.c)) = 0;
4064     [model] = addSinkReactions(model,{'trp-L(c)','Lfmkynr(c)'},[-1 -1; 0 100]);
4065     model.lb(find(ismember(model.rxns,'sink_trp_L(c)')))=-1;
4066     model.ub(find(ismember(model.rxns,'sink_trp_L(c)')))=-1;
4067     model.c(ismember(model.rxns,'sink_Lfmkynr(c)'))=1;
4068     if find(model.c)>0
4069         FBA = optimizeCbModel(model,'max');
4070         TestSolution(k,1) = FBA.f;
4071     else
4072         TestSolution(k,1) = NaN;
4073     end
4074     TestSolutionName{k,1} = 'trp-L(c) -> Lfmkynr(c)';
4075     if ~isnan(TestSolution(k,1)); TestedRxns = [TestedRxns; model.rxns(find(abs(FBA.x)>tol))]; end ;k = k +1;clear FBA
4076     %% trp-L -> melatn
4077     model = modelOri;
4078     model.c(find(model.c)) = 0;
4079     [model] = addSinkReactions(model,{'trp-L(c)','Lkynr(c)'},[-1 -1; 0 100]);
4080     model.lb(find(ismember(model.rxns,'sink_trp_L(c)')))=-1;
4081     model.ub(find(ismember(model.rxns,'sink_trp_L(c)')))=-1;
4082     model.c(ismember(model.rxns,'sink_Lkynr(c)'))=1;
4083     if find(model.c)>0
4084         FBA = optimizeCbModel(model,'max');
4085         TestSolution(k,1) = FBA.f;
4086     else
4087         TestSolution(k,1) = NaN;
4088     end
4089     TestSolutionName{k,1} = 'trp-L(c) -> Lkynr(c)';
4090     if ~isnan(TestSolution(k,1)); TestedRxns = [TestedRxns; model.rxns(find(abs(FBA.x)>tol))]; end ;k = k +1;clear FBA
4091     %% trp-L -> melatn
4092     model = modelOri;
4093     model.c(find(model.c)) = 0;
4094     [model] = addSinkReactions(model,{'trp-L(c)','nformanth(c)'},[-1 -1; 0 100]);
4095     model.lb(find(ismember(model.rxns,'sink_trp_L(c)')))=-1;
4096     model.ub(find(ismember(model.rxns,'sink_trp_L(c)')))=-1;
4097     model.c(ismember(model.rxns,'sink_nformanth(c)'))=1;
4098     if find(model.c)>0
4099         FBA = optimizeCbModel(model,'max');
4100         TestSolution(k,1) = FBA.f;
4101     else
4102         TestSolution(k,1) = NaN;
4103     end
4104     TestSolutionName{k,1} = 'trp-L(c) -> nformanth(c)';
4105     model.lb(find(ismember(model.rxns,'sink_trp_L(c)')))=-1;
4106     model.ub(find(ismember(model.rxns,'sink_trp_L(c)')))=-1;
4107     if ~isnan(TestSolution(k,1)); TestedRxns = [TestedRxns; model.rxns(find(abs(FBA.x)>tol))]; end ;k = k +1;clear FBA
4108     %% srtn(c) -> 5moxact(c)
4109     model = modelOri;
4110     model.c(find(model.c)) = 0;
4111     model.lb(ismember(model.rxns,'EX_glc(e)'))=-1;model.ub(ismember(model.rxns,'EX_glc(e)'))=0;
4112     [model] = addSinkReactions(model,{'srtn(c)','5moxact(c)'},[-1 -1; 0 100]);
4113     model.lb(find(ismember(model.rxns,'DM_srtn(c)')))=-1;
4114     model.ub(find(ismember(model.rxns,'DM_srtn(c)')))=-1;
4115     model.c(ismember(model.rxns,'sink_5moxact(c)'))=1;
4116     if find(model.c)>0
4117         FBA = optimizeCbModel(model,'max');
4118         TestSolution(k,1) = FBA.f;
4119     else
4120         TestSolution(k,1) = NaN;
4121     end
4122     TestSolutionName{k,1} = 'srtn(c) -> 5moxact(c)';
4123     if ~isnan(TestSolution(k,1)); TestedRxns = [TestedRxns; model.rxns(find(abs(FBA.x)>tol))]; end ;k = k +1;clear FBA
4124     %% srtn(c) -> 6hoxmelatn(c)
4125     model = modelOri;
4126     model.c(find(model.c)) = 0;
4127     model.lb(ismember(model.rxns,'EX_glc(e)'))=-1;model.ub(ismember(model.rxns,'EX_glc(e)'))=0;
4128     [model] = addSinkReactions(model,{'srtn(c)','6hoxmelatn(c)'},[-1 -1; 0 100]);
4129     model.lb(find(ismember(model.rxns,'DM_srtn(c)')))=-1;
4130     model.ub(find(ismember(model.rxns,'DM_srtn(c)')))=-1;
4131     model.c(ismember(model.rxns,'sink_6hoxmelatn(c)'))=1;
4132     if find(model.c)>0
4133         FBA = optimizeCbModel(model,'max');
4134         TestSolution(k,1) = FBA.f;
4135     else
4136         TestSolution(k,1) = NaN;
4137     end
4138     TestSolutionName{k,1} = 'srtn(c) -> 6hoxmelatn(c)';
4139     if ~isnan(TestSolution(k,1)); TestedRxns = [TestedRxns; model.rxns(find(abs(FBA.x)>tol))]; end ;k = k +1;clear FBA
4140     %% trp-L -> quln
4141     model = modelOri;
4142     model.c(find(model.c)) = 0;
4143     [model] = addSinkReactions(model,{'trp-L(c)','quln(c)'},[-1 -1; 0 100]);
4144     model.lb(find(ismember(model.rxns,'sink_trp_L(c)')))=-1;
4145     model.ub(find(ismember(model.rxns,'sink_trp_L(c)')))=-1;
4146     model.c(ismember(model.rxns,'sink_quln(c)'))=1;
4147     if find(model.c)>0
4148         FBA = optimizeCbModel(model,'max');
4149         TestSolution(k,1) = FBA.f;
4150     else
4151         TestSolution(k,1) = NaN;
4152     end
4153     TestSolutionName{k,1} = 'trp-L(c) -> quln(c)';
4154     if ~isnan(TestSolution(k,1)); TestedRxns = [TestedRxns; model.rxns(find(abs(FBA.x)>tol))]; end ;k = k +1;clear FBA
4155     %% trp-L -> srtn
4156     model = modelOri;
4157     model.c(find(model.c)) = 0;
4158     [model] = addSinkReactions(model,{'trp-L(c)','srtn(c)'},[-1 -1; 0 100]);
4159     model.lb(find(ismember(model.rxns,'sink_trp_L(c)')))=-1;
4160     model.ub(find(ismember(model.rxns,'sink_trp_L(c)')))=-1;
4161     model.c(ismember(model.rxns,'DM_srtn(c)'))=1;
4162     if find(model.c)>0
4163         FBA = optimizeCbModel(model,'max');
4164         TestSolution(k,1) = FBA.f;
4165     else
4166         TestSolution(k,1) = NaN;
4167     end
4168     TestSolutionName{k,1} = 'trp-L(c) -> srtn(c)';
4169     if ~isnan(TestSolution(k,1)); TestedRxns = [TestedRxns; model.rxns(find(abs(FBA.x)>tol))]; end ;k = k +1;clear FBA
4170     %% Tyr-ggn -> glygn2
4171     model = modelOri;
4172     model.c(find(model.c)) = 0;
4173     model.lb(ismember(model.rxns,'EX_o2(e)'))=-40;model.ub(ismember(model.rxns,'EX_o2(e)'))=-1;
4174     [model] = addSinkReactions(model,{'Tyr-ggn(c)','glygn2(c)'},[-1 -1; 0 100]);
4175     model.lb(find(ismember(model.rxns,'sink_Tyr-ggn(c)')))=-1;
4176     model.ub(find(ismember(model.rxns,'sink_Tyr-ggn(c)')))=-1;
4177     model.c(ismember(model.rxns,'sink_glygn2(c)'))=1;
4178     if find(model.c)>0
4179         FBA = optimizeCbModel(model,'max');
4180         TestSolution(k,1) = FBA.f;
4181     else
4182         TestSolution(k,1) = NaN;
4183     end
4184     TestSolutionName{k,1} = 'Tyr-ggn(c) -> glygn2(c)';
4185     if ~isnan(TestSolution(k,1)); TestedRxns = [TestedRxns; model.rxns(find(abs(FBA.x)>tol))]; end ;k = k +1;clear FBA
4186     %% tyr-L -> 34hpp
4187     model = modelOri;
4188     model.c(find(model.c)) = 0;
4189     [model] = addSinkReactions(model,{'tyr-L(c)','34hpp(c)'},[-1 -1; 0 100]);
4190     model.lb(find(ismember(model.rxns,'sink_tyr_L(c)')))=-1;
4191     model.ub(find(ismember(model.rxns,'sink_tyr_L(c)')))=-1;
4192     model.c(ismember(model.rxns,'sink_34hpp(c)'))=1;
4193     if find(model.c)>0
4194         FBA = optimizeCbModel(model,'max');
4195         TestSolution(k,1) = FBA.f;
4196     else
4197         TestSolution(k,1) = NaN;
4198     end
4199     TestSolutionName{k,1} = 'tyr-L(c) -> 34hpp(c)';
4200     if ~isnan(TestSolution(k,1)); TestedRxns = [TestedRxns; model.rxns(find(abs(FBA.x)>tol))]; end ;k = k +1;clear FBA
4201     %% tyr-L -> 4hphac
4202     model = modelOri;
4203     model.c(find(model.c)) = 0;
4204     [model] = addSinkReactions(model,{'tyr-L(c)','4hphac(c)'},[-1 -1; 0 100]);
4205     model.lb(find(ismember(model.rxns,'sink_tyr_L(c)')))=-1;
4206     model.ub(find(ismember(model.rxns,'sink_tyr_L(c)')))=-1;
4207     model.c(ismember(model.rxns,'sink_4hphac(c)'))=1;
4208     if find(model.c)>0
4209         FBA = optimizeCbModel(model,'max');
4210         TestSolution(k,1) = FBA.f;
4211     else
4212         TestSolution(k,1) = NaN;
4213     end
4214     TestSolutionName{k,1} = 'tyr-L(c) -> 4hphac(c)';
4215     if ~isnan(TestSolution(k,1)); TestedRxns = [TestedRxns; model.rxns(find(abs(FBA.x)>tol))]; end ;k = k +1;clear FBA
4216     %% tyr-L -> adrnl
4217     model = modelOri;
4218     model.c(find(model.c)) = 0;
4219     [model] = addSinkReactions(model,{'tyr-L(c)','adrnl(c)'},[-1 -1; 0 100]);
4220     model.lb(find(ismember(model.rxns,'sink_tyr_L(c)')))=-1;
4221     model.ub(find(ismember(model.rxns,'sink_tyr_L(c)')))=-1;
4222     model.c(ismember(model.rxns,'sink_adrnl(c)'))=1;
4223     if find(model.c)>0
4224         FBA = optimizeCbModel(model,'max');
4225         TestSolution(k,1) = FBA.f;
4226     else
4227         TestSolution(k,1) = NaN;
4228     end
4229     TestSolutionName{k,1} = 'tyr-L(c) -> adrnl(c)';
4230     if ~isnan(TestSolution(k,1)); TestedRxns = [TestedRxns; model.rxns(find(abs(FBA.x)>tol))]; end ;k = k +1;clear FBA
4231     %% tyr-L -> dopa
4232     model = modelOri;
4233     model.c(find(model.c)) = 0;
4234     [model] = addSinkReactions(model,{'tyr-L(c)','dopa(c)'},[-1 -1; 0 100]);
4235     model.lb(find(ismember(model.rxns,'sink_tyr_L(c)')))=-1;
4236     model.ub(find(ismember(model.rxns,'sink_tyr_L(c)')))=-1;
4237     model.c(ismember(model.rxns,'sink_dopa(c)'))=1;
4238     if find(model.c)>0
4239         FBA = optimizeCbModel(model,'max');
4240         TestSolution(k,1) = FBA.f;
4241     else
4242         TestSolution(k,1) = NaN;
4243     end
4244     TestSolutionName{k,1} = 'tyr-L(c) -> dopa(c)';
4245     if ~isnan(TestSolution(k,1)); TestedRxns = [TestedRxns; model.rxns(find(abs(FBA.x)>tol))]; end ;k = k +1;clear FBA
4246     %% tyr-L -> fum + acac
4247     model = modelOri;
4248     model.c(find(model.c)) = 0;
4249     [model] = addSinkReactions(model,{'tyr-L(c)','fum(c)','acac(c)'},[-1 -1; 0.1 100; 0.1 100]);
4250     model.lb(find(ismember(model.rxns,'sink_tyr_L(c)')))=-1;
4251     model.ub(find(ismember(model.rxns,'sink_tyr_L(c)')))=-1;
4252     model.c(ismember(model.rxns,'sink_fum(c)'))=1;
4253     if find(model.c)>0
4254         FBA = optimizeCbModel(model,'max');
4255         TestSolution(k,1) = FBA.f;
4256     else
4257         TestSolution(k,1) = NaN;
4258     end
4259     TestSolutionName{k,1} = 'tyr-L(c) -> fum(c) + acac(c)';
4260     if ~isnan(TestSolution(k,1)); TestedRxns = [TestedRxns; model.rxns(find(abs(FBA.x)>tol))]; end ;k = k +1;clear FBA
4261     %% tyr-L -> melanin
4262     model = modelOri;
4263     model.c(find(model.c)) = 0;
4264     [model,rxnsInModel] = addSinkReactions(model,{'tyr-L(c)','melanin(c)'},[-1 -1; 0 100]);
4265     model.lb(find(ismember(model.rxns,'sink_tyr_L(c)')))=-1;
4266     model.ub(find(ismember(model.rxns,'sink_tyr_L(c)')))=-1;
4267     if (rxnsInModel(2) >-1) % reaction exits already in model
4268         model=changeObjective(model,model.rxns(rxnsInModel(2),1));
4269     else
4270         model=changeObjective(model,'sink_melanin(c)',1);
4271     end
4272     if find(model.c)>0
4273         FBA = optimizeCbModel(model,'max');
4274         TestSolution(k,1) = FBA.f;
4275     else
4276         TestSolution(k,1) = NaN;
4277     end
4278     TestSolutionName{k,1} = 'tyr-L(c) -> melanin(c)';
4279     if ~isnan(TestSolution(k,1)); TestedRxns = [TestedRxns; model.rxns(find(abs(FBA.x)>tol))]; end ;k = k +1;clear FBA
4280     %% tyr-L -> nrpphr
4281     model = modelOri;
4282     model.c(find(model.c)) = 0;
4283     [model] = addSinkReactions(model,{'tyr-L(c)','nrpphr(c)'},[-1 -1; 0 100]);
4284     model.lb(find(ismember(model.rxns,'sink_tyr_L(c)')))=-1;
4285     model.ub(find(ismember(model.rxns,'sink_tyr_L(c)')))=-1;
4286     model.c(ismember(model.rxns,'sink_nrpphr(c)'))=1;
4287     if find(model.c)>0
4288         FBA = optimizeCbModel(model,'max');
4289         TestSolution(k,1) = FBA.f;
4290     else
4291         TestSolution(k,1) = NaN;
4292     end
4293     TestSolutionName{k,1} = 'tyr-L(c) -> nrpphr(c)';
4294     if ~isnan(TestSolution(k,1)); TestedRxns = [TestedRxns; model.rxns(find(abs(FBA.x)>tol))]; end ;k = k +1;clear FBA
4295     %% uacgam + udpglcur -> ha[e] %changing lb has no effect
4296     model = modelOri;
4297     model.c(find(model.c)) = 0;
4298     model.lb(ismember(model.rxns,'EX_glc(e)'))=-1;model.ub(ismember(model.rxns,'EX_glc(e)'))=0;
4299     model.lb(ismember(model.rxns,'EX_o2(e)'))=-40;model.ub(ismember(model.rxns,'EX_o2(e)'))=-1;
4300     [model] = addSinkReactions(model,{'uacgam(c)','udpglcur(c)','ha(e)'},[-1 -1; -1 -1;0 100]);
4301     %model.c(ismember(model.rxns,'sink_ha(c)'))=1;
4302     if ~isempty(strmatch('HAS2',model.rxns,'exact'))
4303         model=changeObjective(model,'HAS2',1);
4304         FBA = optimizeCbModel(model,'max');
4305         TestSolution(k,1) = FBA.f;
4306     else
4307         TestSolution(k,1) = NaN;
4308     end
4309     TestSolutionName{k,1} =  'uacgamv(c) + udpglcur(c) -> ha[e] - via HAS2';
4310     if ~isnan(TestSolution(k,1)); TestedRxns = [TestedRxns; model.rxns(find(abs(FBA.x)>tol))]; end ;k = k +1;clear FBA
4311     
4312     %% uacgam -> m8masn[r]
4313     model = modelOri;
4314     model.c(find(model.c)) = 0;
4315     model.lb(ismember(model.rxns,'EX_o2(e)'))=-40;model.ub(ismember(model.rxns,'EX_o2(e)'))=-1;
4316     [model] = addSinkReactions(model,{'uacgam(c)','m8masn(r)'},[-1 -1; 0 100]);
4317     model.c(ismember(model.rxns,'sink_m8masn(r)'))=1;
4318     if find(model.c)>0
4319         FBA = optimizeCbModel(model,'max');
4320         TestSolution(k,1) = FBA.f;
4321     else
4322         TestSolution(k,1) = NaN;
4323     end
4324     TestSolutionName{k,1} = 'uacgam(c) -> m8masn(r)';
4325     if ~isnan(TestSolution(k,1)); TestedRxns = [TestedRxns; model.rxns(find(abs(FBA.x)>tol))]; end ;k = k +1;clear FBA
4326     %% udpglcur -> xu5p-D
4327     model = modelOri;
4328     model.c(find(model.c)) = 0;
4329     [model] = addSinkReactions(model,{'udpglcur(c)','xu5p-D(c)'},[-1 -1; 0 100]);
4330     model.c(ismember(model.rxns,'sink_xu5p-D(c)'))=1;
4331     if find(model.c)>0
4332         FBA = optimizeCbModel(model,'max');
4333         TestSolution(k,1) = FBA.f;
4334     else
4335         TestSolution(k,1) = NaN;
4336     end
4337     TestSolutionName{k,1} = 'udpglcur(c) -> xu5p-D(c)';
4338     if ~isnan(TestSolution(k,1)); TestedRxns = [TestedRxns; model.rxns(find(abs(FBA.x)>tol))]; end ;k = k +1;clear FBA
4339     %% ura -> ala-B
4340     model = modelOri;
4341     model.c(find(model.c)) = 0;
4342     [model] = addSinkReactions(model,{'ura(c)','ala-B(c)'},[-1 -1; 0 100]);
4343     model.c(ismember(model.rxns,'sink_ala-B(c)'))=1;
4344     if find(model.c)>0
4345         FBA = optimizeCbModel(model,'max');
4346         TestSolution(k,1) = FBA.f;
4347     else
4348         TestSolution(k,1) = NaN;
4349     end
4350     TestSolutionName{k,1} = 'ura(c) -> ala-B(c)';
4351     if ~isnan(TestSolution(k,1)); TestedRxns = [TestedRxns; model.rxns(find(abs(FBA.x)>tol))]; end ;k = k +1;clear FBA
4352     %% val-L -> 3aib
4353     model = modelOri;
4354     model.c(find(model.c)) = 0;
4355     [model] = addSinkReactions(model,{'val-L(c)','3aib(c)'},[-1 -1; 0 100]);
4356     model.lb(find(ismember(model.rxns,'sink_val_L(c)')))=-1;
4357     model.ub(find(ismember(model.rxns,'sink_val_L(c)')))=-1;
4358     model.c(ismember(model.rxns,'sink_3aib(c)'))=1;
4359     if find(model.c)>0
4360         FBA = optimizeCbModel(model,'max');
4361         TestSolution(k,1) = FBA.f;
4362     else
4363         TestSolution(k,1) = NaN;
4364     end
4365     TestSolutionName{k,1} = 'val-L(c) -> 3aib(c)';
4366     if ~isnan(TestSolution(k,1)); TestedRxns = [TestedRxns; model.rxns(find(abs(FBA.x)>tol))]; end ;k = k +1;clear FBA
4367     %% val-L -> succoa
4368     model = modelOri;
4369     model.c(find(model.c)) = 0;
4370     model.lb(ismember(model.rxns,'EX_o2(e)'))=-40;model.ub(ismember(model.rxns,'EX_o2(e)'))=-1;
4371     [model] = addSinkReactions(model,{'val-L(c)','succoa(m)'},[-1 -1; 0 100]);
4372     model.lb(find(ismember(model.rxns,'sink_val_L(c)')))=-1;
4373     model.ub(find(ismember(model.rxns,'sink_val_L(c)')))=-1;
4374     model.lb(find(ismember(model.rxns,'sink_coa(c)')))=-1;
4375     model.ub(find(ismember(model.rxns,'sink_coa(c)')))=1;
4376     model.c(ismember(model.rxns,'sink_succoa(m)'))=1;
4377     if find(model.c)>0
4378         FBA = optimizeCbModel(model,'max');
4379         TestSolution(k,1) = FBA.f;
4380     else
4381         TestSolution(k,1) = NaN;
4382     end
4383     TestSolutionName{k,1} = 'val-L(c) -> succoa(m)';
4384     if ~isnan(TestSolution(k,1)); TestedRxns = [TestedRxns; model.rxns(find(abs(FBA.x)>tol))]; end ;k = k +1;clear FBA
4385     %% xoltriol(m) -> thcholstoic(m)
4386     model = modelOri;
4387     model.c(find(model.c)) = 0;
4388     [model] = addSinkReactions(model,{'xoltriol(m)','thcholstoic(m)'},[-1 -1; 0 100]);
4389     model.c(ismember(model.rxns,'sink_thcholstoic(m)'))=1;
4390     if find(model.c)>0
4391         FBA = optimizeCbModel(model,'max');
4392         TestSolution(k,1) = FBA.f;
4393     else
4394         TestSolution(k,1) = NaN;
4395     end
4396     TestSolutionName{k,1} = 'xoltriol(m) -> thcholstoic(m)';
4397     if ~isnan(TestSolution(k,1)); TestedRxns = [TestedRxns; model.rxns(find(abs(FBA.x)>tol))]; end ;k = k +1;clear FBA
4398     %% xylu-D -> glyclt
4399     model = modelOri;
4400     model.c(find(model.c)) = 0;
4401     [model] = addSinkReactions(model,{'xylu-D(c)','glyclt(c)'},[-1 -1; 0 100]);
4402     model.c(ismember(model.rxns,'sink_glyclt(c)'))=1;
4403     if find(model.c)>0
4404         FBA = optimizeCbModel(model,'max');
4405         TestSolution(k,1) = FBA.f;
4406     else
4407         TestSolution(k,1) = NaN;
4408     end
4409     TestSolutionName{k,1} = 'xylu-D(c) -> glyclt(c)';
4410     if ~isnan(TestSolution(k,1)); TestedRxns = [TestedRxns; model.rxns(find(abs(FBA.x)>tol))]; end ;k = k +1;clear FBA
4411 end
4412 
4413 %% Test for IEC ori  - works only for models with 'u'=lumen compartments
4414 if strcmp(test,'IECOri')
4415     % 1) glucose to lactate conversion
4416     model=modelOri;
4417     model=changeRxnBounds(model,'EX_gln_L(u)',0,'b');
4418     model=changeObjective(model,'EX_lac-L(e)');
4419     %FBA=optimizeCbModel(model,'min')
4420     FBA=optimizeCbModel(model,'max')
4421     TestSolution(k,1) = FBA.f;
4422     TestSolutionName{k,1} = 'glucose to lactate conversion';
4423     if ~isnan(TestSolution(k,1)); TestedRxns = [TestedRxns; model.rxns(find(abs(FBA.x)>tol))]; end ;k = k +1;clear FBA
4424     
4425     % 2); glutamine to glucose conversion
4426     model=modelOri;
4427     model=changeRxnBounds(model,'EX_gln-L(e)',-1,'b');
4428     model=changeRxnBounds(model,'EX_glc_D(u)',0,'b');
4429     model=changeRxnBounds(model,'EX_malt(u)',0,'b');
4430     model=changeRxnBounds(model,'EX_STRCH1(u)',0,'b');
4431     model=changeRxnBounds(model,'EX_strch2(u)',0,'b');
4432     model=changeRxnBounds(model,'EX_SUCR(u)',0,'b');
4433     model=changeObjective(model,'GLUNm');
4434     FBA=optimizeCbModel(model,'max');
4435     model=changeObjective(model,'ASPTAm');
4436     TestSolution(k,1) = FBA.f;
4437     TestSolutionName{k,1} = 'glutamine to glucose conversion - ASPTAm';
4438     if ~isnan(TestSolution(k,1)); TestedRxns = [TestedRxns; model.rxns(find(abs(FBA.x)>tol))]; end ;k = k +1;clear FBA
4439     model=changeObjective(model,'FUM');
4440     FBA=optimizeCbModel(model,'max');
4441     TestSolution(k,1) = FBA.f;
4442     TestSolutionName{k,1} = 'glutamine to glucose conversion - FUM';
4443     if ~isnan(TestSolution(k,1)); TestedRxns = [TestedRxns; model.rxns(find(abs(FBA.x)>tol))]; end ;k = k +1;clear FBA
4444     model=changeObjective(model,'MDH');
4445     FBA=optimizeCbModel(model,'max');
4446     TestSolution(k,1) = FBA.f;
4447     TestSolutionName{k,1} = 'glutamine to glucose conversion - MDH';
4448     if ~isnan(TestSolution(k,1)); TestedRxns = [TestedRxns; model.rxns(find(abs(FBA.x)>tol))]; end ;k = k +1;clear FBA
4449     model=changeObjective(model,'G6PPer');
4450     FBA=optimizeCbModel(model,'max');
4451     TestSolution(k,1) = FBA.f;
4452     TestSolutionName{k,1} = 'glutamine to glucose conversion - G6PPer';
4453     if ~isnan(TestSolution(k,1)); TestedRxns = [TestedRxns; model.rxns(find(abs(FBA.x)>tol))]; end ;k = k +1;clear FBA
4454     
4455     % 3); glutamine to proline conversion
4456     model=modelOri;
4457     model=changeRxnBounds(model,'EX_gln-L(e)',0,'b');
4458     model=changeRxnBounds(model,'EX_glc_D(u)',0,'b');
4459     model=changeRxnBounds(model,'EX_PRO-L(u)',0,'b');
4460     model=changeObjective(model,'P5CRm');
4461     %FBA=optimizeCbModel(model,'min');
4462     FBA=optimizeCbModel(model,'max');
4463     TestSolution(k,1) = FBA.f;
4464     TestSolutionName{k,1} = 'glutamine to proline conversion - P5CRm';
4465     if ~isnan(TestSolution(k,1)); TestedRxns = [TestedRxns; model.rxns(find(abs(FBA.x)>tol))]; end ;k = k +1;clear FBA
4466     model=changeObjective(model,'P5CRxm');
4467     %FBA=optimizeCbModel(model,'min');
4468     FBA=optimizeCbModel(model,'max');
4469     TestSolution(k,1) = FBA.f;
4470     TestSolutionName{k,1} = 'glutamine to proline conversion - P5CRxm';
4471     if ~isnan(TestSolution(k,1)); TestedRxns = [TestedRxns; model.rxns(find(abs(FBA.x)>tol))]; end ;k = k +1;clear FBA
4472     
4473     % 4); glutamine to ornithine conversion
4474     model=modelOri;
4475     model=changeRxnBounds(model,'EX_gln-L(e)',0,'b');
4476     model=changeRxnBounds(model,'EX_glc_D(u)',0,'b');
4477     model=changeRxnBounds(model,'EX_ORN(U)',0,'b');
4478     model=changeObjective(model,'ORNTArm');
4479     %FBA=optimizeCbModel(model,'min');
4480     FBA=optimizeCbModel(model,'max');
4481     TestSolution(k,1) = FBA.f;
4482     TestSolutionName{k,1} = 'glutamine to ornithine conversion - ORNTArm';
4483     if ~isnan(TestSolution(k,1)); TestedRxns = [TestedRxns; model.rxns(find(abs(FBA.x)>tol))]; end ;k = k +1;clear FBA
4484     
4485     % 5); glutamine to citrulline converion
4486     model=modelOri;
4487     model=changeRxnBounds(model,'EX_gln-L(e)',0,'b');
4488     model=changeRxnBounds(model,'EX_glc_D(u)',0,'b');
4489     model=changeObjective(model,'OCBTm');
4490     %FBA=optimizeCbModel(model,'min');
4491     FBA=optimizeCbModel(model,'max');
4492     TestSolution(k,1) = FBA.f;
4493     TestSolutionName{k,1} = 'glutamine to citrulline converion - OCBTm';
4494     if ~isnan(TestSolution(k,1)); TestedRxns = [TestedRxns; model.rxns(find(abs(FBA.x)>tol))]; end ;k = k +1;clear FBA
4495     
4496     % 6); glutamine to lactate
4497     model=modelOri;
4498     model=changeRxnBounds(model,'EX_gln-L(e)',0,'b');
4499     model=changeRxnBounds(model,'EX_glc_D(u)',0,'b');
4500     model=changeRxnBounds(model,'EX_malt(u)',0,'b');
4501     model=changeRxnBounds(model,'EX_STRCH1(u)',0,'b');
4502     model=changeRxnBounds(model,'EX_strch2(u)',0,'b');
4503     model=changeRxnBounds(model,'EX_SUCR(u)',0,'b');
4504     model=changeRxnBounds(model,'EX_LAC-L(u)',0,'b');
4505     model=changeObjective(model,'LDH_L');
4506     %FBA=optimizeCbModel(model,'min');
4507     FBA=optimizeCbModel(model,'max');
4508     TestSolution(k,1) = FBA.f;
4509     TestSolutionName{k,1} = 'glutamine to lactate - LDH_L';
4510     if ~isnan(TestSolution(k,1)); TestedRxns = [TestedRxns; model.rxns(find(abs(FBA.x)>tol))]; end ;k = k +1;clear FBA
4511     
4512     % 7); glutamine to aspartate
4513     model=modelOri;
4514     model=changeRxnBounds(model,'EX_glc_D(u)',0,'b');
4515     model=changeRxnBounds(model,'EX_ASP-L(u)',0,'b');
4516     model=changeRxnBounds(model,'EX_malt(u)',0,'b');
4517     model=changeRxnBounds(model,'EX_STRCH1(u)',0,'b');
4518     model=changeRxnBounds(model,'EX_strch2(u)',0,'b');
4519     model=changeRxnBounds(model,'EX_SUCR(u)',0,'b');
4520     model=changeObjective(model,'ASPTA');
4521     %FBA=optimizeCbModel(model,'min');
4522     FBA=optimizeCbModel(model,'max');
4523     TestSolution(k,1) = FBA.f;
4524     TestSolutionName{k,1} = 'glutamine to aspartate - ASPTA';
4525     if ~isnan(TestSolution(k,1)); TestedRxns = [TestedRxns; model.rxns(find(abs(FBA.x)>tol))]; end ;k = k +1;clear FBA
4526     
4527     % 8); glutamine to co2
4528     model=modelOri;
4529     model=changeRxnBounds(model,'EX_gln-L(e)',0,'b');
4530     model=changeRxnBounds(model,'EX_glc_D(u)',0,'b');
4531     model=changeRxnBounds(model,'EX_GLU-L(u)',0,'b');
4532     model=changeRxnBounds(model,'EX_malt(u)',0,'b');
4533     model=changeRxnBounds(model,'EX_STRCH1(u)',0,'b');
4534     model=changeRxnBounds(model,'EX_strch2(u)',0,'b');
4535     model=changeRxnBounds(model,'EX_SUCR(u)',0,'b');
4536     model=changeObjective(model,'AKGDm');
4537     %FBA=optimizeCbModel(model,'min');
4538     FBA=optimizeCbModel(model,'max');
4539     TestSolution(k,1) = FBA.f;
4540     TestSolutionName{k,1} = 'glutamine to co2 - AKGDm';
4541     if ~isnan(TestSolution(k,1)); TestedRxns = [TestedRxns; model.rxns(find(abs(FBA.x)>tol))]; end ;k = k +1;clear FBA
4542     
4543     % 9); glutamine to ammonia
4544     model=modelOri;
4545     model=changeRxnBounds(model,'EX_gln-L(e)',-1,'b');
4546     model=changeRxnBounds(model,'EX_glc_D(u)',0,'b');
4547     model=changeRxnBounds(model,'EX_GLU-L(u)',0,'b');
4548     model=changeObjective(model,'GLUNm');
4549     %FBA=optimizeCbModel(model,'min');
4550     FBA=optimizeCbModel(model,'max');
4551     TestSolution(k,1) = FBA.f;
4552     TestSolutionName{k,1} = 'glutamine to ammonia - GLUNm';
4553     if ~isnan(TestSolution(k,1)); TestedRxns = [TestedRxns; model.rxns(find(abs(FBA.x)>tol))]; end ;k = k +1;clear FBA
4554     
4555     % 10); putriscine to methionine (depends on oxygen uptake);
4556     model=modelOri;
4557     model=changeRxnBounds(model,'EX_gln-L(e)',0,'b');
4558     model=changeRxnBounds(model,'EX_glc_D(u)',0,'b');
4559     model=changeRxnBounds(model,'EX_GLU-L(u)',0,'b');
4560     model=changeRxnBounds(model,'EX_MET-L(u)',0,'b');
4561     model=changeRxnBounds(model,'EX_met_L(e)',0,'b');
4562     model=changeRxnBounds(model,'EX_o2(e)',-1,'l');
4563     model=changeObjective(model,'UNK2');
4564     %FBA=optimizeCbModel(model,'min');
4565     FBA=optimizeCbModel(model,'max');
4566     TestSolution(k,1) = FBA.f;
4567     TestSolutionName{k,1} = 'putriscine to methionine (depends on oxygen uptake) - UNK2';
4568     if ~isnan(TestSolution(k,1)); TestedRxns = [TestedRxns; model.rxns(find(abs(FBA.x)>tol))]; end ;k = k +1;clear FBA
4569     
4570     % 11); basolateral secretion of alanine
4571     model=modelOri;
4572     model=changeRxnBounds(model,'EX_gln-L(e)',0,'b');
4573     model=changeRxnBounds(model,'EX_glc_D(u)',0,'b');
4574     model=changeRxnBounds(model,'EX_GLU-L(u)',0,'b');
4575     model=changeRxnBounds(model,'EX_ala_L(u)',0,'b');
4576     model=changeObjective(model,'EX_ala_L(e)');
4577     %FBA=optimizeCbModel(model,'min');
4578     FBA=optimizeCbModel(model,'max');
4579     TestSolution(k,1) = FBA.f;
4580     TestSolutionName{k,1} = 'basolateral secretion of alanine';
4581     if ~isnan(TestSolution(k,1)); TestedRxns = [TestedRxns; model.rxns(find(abs(FBA.x)>tol))]; end ;k = k +1;clear FBA
4582     
4583     % 12); basolateral secretion of lactate
4584     model=modelOri;
4585     model=changeRxnBounds(model,'EX_gln-L(e)',0,'b');
4586     model=changeRxnBounds(model,'EX_glc_D(u)',0,'b');
4587     model=changeObjective(model,'EX_lac-L(e)');
4588     %FBA=optimizeCbModel(model,'min');
4589     FBA=optimizeCbModel(model,'max');
4590     TestSolution(k,1) = FBA.f;
4591     TestSolutionName{k,1} = 'basolateral secretion of lactate';
4592     if ~isnan(TestSolution(k,1)); TestedRxns = [TestedRxns; model.rxns(find(abs(FBA.x)>tol))]; end ;k = k +1;clear FBA
4593     
4594     % 13);synthesis of arginine from glutamine
4595     model=modelOri;
4596     model=changeRxnBounds(model,'EX_ARG-L(u)',0,'b');
4597     model=changeRxnBounds(model,'EX_arg_L(e)',0,'b');
4598     model=changeObjective(model,'ARGSL');
4599     %FBA=optimizeCbModel(model,'min');
4600     FBA=optimizeCbModel(model,'max');
4601     TestSolution(k,1) = FBA.f;
4602     TestSolutionName{k,1} = 'synthesis of arginine from glutamine - ARGSL';
4603     if ~isnan(TestSolution(k,1)); TestedRxns = [TestedRxns; model.rxns(find(abs(FBA.x)>tol))]; end ;k = k +1;clear FBA
4604     
4605     % 14);synthesis of proline from glutamine
4606     model=modelOri;
4607     model=changeRxnBounds(model,'EX_PRO-L(u)',0,'b');
4608     model=changeRxnBounds(model,'EX_pro-L(e)',0,'b');
4609     model=changeObjective(model,'P5CR');
4610     %FBA=optimizeCbModel(model,'min');
4611     FBA=optimizeCbModel(model,'max');
4612     TestSolution(k,1) = FBA.f;
4613     TestSolutionName{k,1} = 'synthesis of proline from glutamine - P5CR';
4614     if ~isnan(TestSolution(k,1)); TestedRxns = [TestedRxns; model.rxns(find(abs(FBA.x)>tol))]; end ;k = k +1;clear FBA
4615     model=changeObjective(model,'P5CRm');
4616     %FBA=optimizeCbModel(model,'min');
4617     FBA=optimizeCbModel(model,'max');
4618     TestSolution(k,1) = FBA.f;
4619     TestSolutionName{k,1} = 'synthesis of proline from glutamine - P5CRm';
4620     if ~isnan(TestSolution(k,1)); TestedRxns = [TestedRxns; model.rxns(find(abs(FBA.x)>tol))]; end ;k = k +1;clear FBA
4621     model=changeObjective(model,'P5CRxm');
4622     %FBA=optimizeCbModel(model,'min');
4623     FBA=optimizeCbModel(model,'max');
4624     TestSolution(k,1) = FBA.f;
4625     TestSolutionName{k,1} = 'synthesis of proline from glutamine - P5CRxm';
4626     if ~isnan(TestSolution(k,1)); TestedRxns = [TestedRxns; model.rxns(find(abs(FBA.x)>tol))]; end ;k = k +1;clear FBA
4627     
4628     % 15); synthesis of alanine from glutamine
4629     model=modelOri;
4630     model=changeRxnBounds(model,'EX_o2(e)',-1,'l');
4631     model=changeRxnBounds(model,'EX_glc(e)',0,'b');
4632     model=changeRxnBounds(model,'EX_GLU-L(e)',0,'b');
4633     model=changeRxnBounds(model,'EX_SUCR(e)',0,'b');
4634     model=changeRxnBounds(model,'EX_malt(e)',0,'b');
4635     model=changeRxnBounds(model,'EX_STRCH1(e)',0,'b');
4636     model=changeRxnBounds(model,'EX_strch2(e)',0,'b');
4637     model=changeRxnBounds(model,'EX_ala_L(e)',0,'b');
4638     model=changeObjective(model,'ALATA_L');
4639     %FBA=optimizeCbModel(model,'min');
4640     FBA=optimizeCbModel(model,'max');
4641     TestSolution(k,1) = FBA.f;
4642     TestSolutionName{k,1} = 'synthesis of alanine from glutamine - ALATA_L';
4643     if ~isnan(TestSolution(k,1)); TestedRxns = [TestedRxns; model.rxns(find(abs(FBA.x)>tol))]; end ;k = k +1;clear FBA
4644     
4645     % 16); basolateral secretion of proline
4646     model=modelOri;
4647     model=changeRxnBounds(model,'EX_PRO-L(u)',0,'b');
4648     model=changeObjective(model,'EX_pro-L(e)');
4649     %FBA=optimizeCbModel(model,'min');
4650     FBA=optimizeCbModel(model,'max');
4651     TestSolution(k,1) = FBA.f;
4652     TestSolutionName{k,1} = 'basolateral secretion of proline';
4653     if ~isnan(TestSolution(k,1)); TestedRxns = [TestedRxns; model.rxns(find(abs(FBA.x)>tol))]; end ;k = k +1;clear FBA
4654     
4655     % 17); basolateral secretion of arginine
4656     model=modelOri;
4657     model=changeRxnBounds(model,'EX_ARG-L(u)',0,'b');
4658     model=changeObjective(model,'EX_arg_L(e)');
4659     %FBA=optimizeCbModel(model,'min');
4660     FBA=optimizeCbModel(model,'max');
4661     TestSolution(k,1) = FBA.f;
4662     TestSolutionName{k,1} = 'basolateral secretion of arginine';
4663     if ~isnan(TestSolution(k,1)); TestedRxns = [TestedRxns; model.rxns(find(abs(FBA.x)>tol))]; end ;k = k +1;clear FBA
4664     
4665     % 18); basolateral secretion of ornithine
4666     model=modelOri;
4667     model=changeRxnBounds(model,'EX_ORN(U)',0,'b');
4668     model=changeObjective(model,'EX_orn(e)');
4669     %FBA=optimizeCbModel(model,'min');
4670     FBA=optimizeCbModel(model,'max');
4671     TestSolution(k,1) = FBA.f;
4672     TestSolutionName{k,1} = 'basolateral secretion of ornithine';
4673     if ~isnan(TestSolution(k,1)); TestedRxns = [TestedRxns; model.rxns(find(abs(FBA.x)>tol))]; end ;k = k +1;clear FBA
4674     
4675     % 19); synthesis of spermine from ornithine
4676     model=modelOri;
4677     model=changeRxnBounds(model,'EX_o2(e)',-1,'l');
4678     model=changeObjective(model,'SPRMS');
4679     %FBA=optimizeCbModel(model,'min');
4680     FBA=optimizeCbModel(model,'max');
4681     TestSolution(k,1) = FBA.f;
4682     TestSolutionName{k,1} = 'synthesis of spermine from ornithine - SPRMS';
4683     if ~isnan(TestSolution(k,1)); TestedRxns = [TestedRxns; model.rxns(find(abs(FBA.x)>tol))]; end ;k = k +1;clear FBA
4684     
4685     % 20);synthesis of spermidine from ornithine
4686     model=modelOri;
4687     model=changeRxnBounds(model,'EX_o2(e)',-1,'l');
4688     model=changeObjective(model,'SPMS');
4689     %FBA=optimizeCbModel(model,'min');
4690     FBA=optimizeCbModel(model,'max');
4691     TestSolution(k,1) = FBA.f;
4692     TestSolutionName{k,1} = 'synthesis of spermidine from ornithine - SPMS';
4693     if ~isnan(TestSolution(k,1)); TestedRxns = [TestedRxns; model.rxns(find(abs(FBA.x)>tol))]; end ;k = k +1;clear FBA
4694     
4695     % 21); synthesis of nitric oxide from arginine
4696     model=modelOri;
4697     model=changeRxnBounds(model,'EX_o2(e)',-1,'l');
4698     model=changeObjective(model,'NOS2');
4699     %FBA=optimizeCbModel(model,'min');
4700     FBA=optimizeCbModel(model,'max');
4701     TestSolution(k,1) = FBA.f;
4702     TestSolutionName{k,1} = 'synthesis of nitric oxide from arginine - NOS2';
4703     if ~isnan(TestSolution(k,1)); TestedRxns = [TestedRxns; model.rxns(find(abs(FBA.x)>tol))]; end ;k = k +1;clear FBA
4704     
4705     % 22); synthesis of cholesterol
4706     model=modelOri;
4707     model=changeRxnBounds(model,'EX_chsterol(u)',0,'b');
4708     model=changeRxnBounds(model,'EX_o2(e)',-1,'l');
4709     model=changeObjective(model,'DSREDUCr');
4710     %FBA=optimizeCbModel(model,'min');
4711     FBA=optimizeCbModel(model,'max');
4712     TestSolution(k,1) = FBA.f;
4713     TestSolutionName{k,1} = 'synthesis of cholesterol - DSREDUCr';
4714     if ~isnan(TestSolution(k,1)); TestedRxns = [TestedRxns; model.rxns(find(abs(FBA.x)>tol))]; end ;k = k +1;clear FBA
4715     
4716     % 23); denovo purine synthesis
4717     model=modelOri;
4718     model=changeObjective(model,'ADSL1');
4719     %FBA=optimizeCbModel(model,'min');
4720     FBA=optimizeCbModel(model,'max');
4721     TestSolution(k,1) = FBA.f;
4722     TestSolutionName{k,1} = 'de novo purine synthesis - ADSL1';
4723     if ~isnan(TestSolution(k,1)); TestedRxns = [TestedRxns; model.rxns(find(abs(FBA.x)>tol))]; end ;k = k +1;clear FBA
4724     model=changeRxnBounds(model,'EX_o2(e)',-1,'l');
4725     model=changeObjective(model,'GMPS2');
4726     %FBA=optimizeCbModel(model,'min');
4727     FBA=optimizeCbModel(model,'max');
4728     TestSolution(k,1) = FBA.f;
4729     TestSolutionName{k,1} = 'de novo purine synthesis - GMPS2';
4730     if ~isnan(TestSolution(k,1)); TestedRxns = [TestedRxns; model.rxns(find(abs(FBA.x)>tol))]; end ;k = k +1;clear FBA
4731     
4732     % 24); salvage of purine bases
4733     model=modelOri;
4734     model=changeRxnBounds(model,'EX_o2(e)',-1,'l');
4735     model=changeObjective(model,'ADPT');
4736     %FBA=optimizeCbModel(model,'min');
4737     FBA=optimizeCbModel(model,'max');
4738     TestSolution(k,1) = FBA.f;
4739     TestSolutionName{k,1} = 'salvage of purine bases - ADPT';
4740     if ~isnan(TestSolution(k,1)); TestedRxns = [TestedRxns; model.rxns(find(abs(FBA.x)>tol))]; end ;k = k +1;clear FBA
4741     model=changeObjective(model,'GUAPRT');
4742     %FBA=optimizeCbModel(model,'min');
4743     FBA=optimizeCbModel(model,'max');
4744     TestSolution(k,1) = FBA.f;
4745     TestSolutionName{k,1} = 'salvage of purine bases - GUAPRT';
4746     if ~isnan(TestSolution(k,1)); TestedRxns = [TestedRxns; model.rxns(find(abs(FBA.x)>tol))]; end ;k = k +1;clear FBA
4747     model=changeObjective(model,'HXPRT');
4748     %FBA=optimizeCbModel(model,'min');
4749     FBA=optimizeCbModel(model,'max');
4750     TestSolution(k,1) = FBA.f;
4751     TestSolutionName{k,1} = 'salvage of purine bases - HXPRT';
4752     if ~isnan(TestSolution(k,1)); TestedRxns = [TestedRxns; model.rxns(find(abs(FBA.x)>tol))]; end ;k = k +1;clear FBA
4753     
4754     % 25); purine catabolism
4755     model=modelOri;
4756     model=changeRxnBounds(model,'EX_o2(e)',-1,'l');
4757     model=changeObjective(model,'XAOx');
4758     %FBA=optimizeCbModel(model,'min');
4759     FBA=optimizeCbModel(model,'max');
4760     TestSolution(k,1) = FBA.f;
4761     TestSolutionName{k,1} = 'purine catabolism - XAOx';
4762     if ~isnan(TestSolution(k,1)); TestedRxns = [TestedRxns; model.rxns(find(abs(FBA.x)>tol))]; end ;k = k +1;clear FBA
4763     
4764     % 26); pyrimidine synthesis (check for both with and without bicarbonate uptake);
4765     model=modelOri;
4766     model=changeRxnBounds(model,'EX_o2(e)',-1,'l');
4767     model=changeRxnBounds(model,'EX_hco3(e)',-1,'l');
4768     model=changeObjective(model,'TMDS');
4769     %FBA=optimizeCbModel(model,'min');
4770     FBA=optimizeCbModel(model,'max');
4771     TestSolution(k,1) = FBA.f;
4772     TestSolutionName{k,1} = 'pyrimidine synthesis (with hco3 uptake) - TMDS';
4773     if ~isnan(TestSolution(k,1)); TestedRxns = [TestedRxns; model.rxns(find(abs(FBA.x)>tol))]; end ;k = k +1;clear FBA
4774     model=changeObjective(model,'CTPS2');
4775     %FBA=optimizeCbModel(model,'min');
4776     FBA=optimizeCbModel(model,'max');
4777     TestSolution(k,1) = FBA.f;
4778     TestSolutionName{k,1} = 'pyrimidine synthesis (with hco3 uptake) - CTPS2';
4779     if ~isnan(TestSolution(k,1)); TestedRxns = [TestedRxns; model.rxns(find(abs(FBA.x)>tol))]; end ;k = k +1;clear FBA
4780     
4781     % 27); pyrimidine catabolism
4782     model=modelOri;
4783     model=changeRxnBounds(model,'EX_o2(e)',-1,'l');
4784     model=changeRxnBounds(model,'EX_hco3(e)',-1,'l');
4785     model=changeObjective(model,'UPPN');
4786     %FBA=optimizeCbModel(model,'min');
4787     FBA=optimizeCbModel(model,'max');
4788     TestSolution(k,1) = FBA.f;
4789     TestSolutionName{k,1} = 'pyrimidine catabolism - UPPN';
4790     if ~isnan(TestSolution(k,1)); TestedRxns = [TestedRxns; model.rxns(find(abs(FBA.x)>tol))]; end ;k = k +1;clear FBA
4791     model=changeObjective(model,'BUP2');
4792     FBA=optimizeCbModel(model)
4793     TestSolution(k,1) = FBA.f;
4794     TestSolutionName{k,1} = 'pyrimidine catabolism - BUP2';
4795     if ~isnan(TestSolution(k,1)); TestedRxns = [TestedRxns; model.rxns(find(abs(FBA.x)>tol))]; end ;k = k +1;clear FBA
4796     
4797     % 28); fructose to glucose conversion
4798     model=modelOri;
4799     model=changeRxnBounds(model,'EX_o2(e)',-1,'l');
4800     model=changeRxnBounds(model,'EX_SUCR(u)',0,'b');
4801     model=changeRxnBounds(model,'EX_malt(u)',0,'b');
4802     model=changeRxnBounds(model,'EX_STRCH1(u)',0,'b');
4803     model=changeRxnBounds(model,'EX_strch2(u)',0,'b');
4804     model=changeRxnBounds(model,'EX_glc_D(u)',0,'b');
4805     model=changeObjective(model,'TRIOK');
4806     %FBA=optimizeCbModel(model,'min');
4807     FBA=optimizeCbModel(model,'max');
4808     TestSolution(k,1) = FBA.f;
4809     TestSolutionName{k,1} = 'fructose to glucose conversion - TRIOK';
4810     if ~isnan(TestSolution(k,1)); TestedRxns = [TestedRxns; model.rxns(find(abs(FBA.x)>tol))]; end ;k = k +1;clear FBA
4811     
4812     % 29); uptake and secretion of cholic acid
4813     model=modelOri;
4814     model=changeRxnBounds(model,'EX_o2(e)',-1,'l');
4815     model=changeRxnBounds(model,'EX_cholate(u)',-1,'l');
4816     model=changeObjective(model,'CHOLATEt2u');
4817     %FBA=optimizeCbModel(model,'min');
4818     FBA=optimizeCbModel(model,'max');
4819     TestSolution(k,1) = FBA.f;
4820     TestSolutionName{k,1} = 'uptake and secretion of cholic acid - CHOLATEt2u'; % SHOULD THIS BE MIN?
4821     if ~isnan(TestSolution(k,1)); TestedRxns = [TestedRxns; model.rxns(find(abs(FBA.x)>tol))]; end ;k = k +1;clear FBA
4822     model=changeObjective(model,'CHOLATEt3');
4823     %FBA=optimizeCbModel(model,'min');
4824     FBA=optimizeCbModel(model,'max');
4825     TestSolution(k,1) = FBA.f;
4826     TestSolutionName{k,1} = 'uptake and secretion of cholic acid - CHOLATEt3';
4827     if ~isnan(TestSolution(k,1)); TestedRxns = [TestedRxns; model.rxns(find(abs(FBA.x)>tol))]; end ;k = k +1;clear FBA
4828     
4829     % 30); Uptake and secretion of glycocholate
4830     model=modelOri;
4831     model=changeRxnBounds(model,'EX_o2(e)',-1,'l');
4832     model=changeRxnBounds(model,'EX_GCHOLA(u)',-1,'l');
4833     model=changeObjective(model,'GCHOLAt2u');
4834     %FBA=optimizeCbModel(model,'min');
4835     FBA=optimizeCbModel(model,'max');
4836     TestSolution(k,1) = FBA.f;
4837     TestSolutionName{k,1} = 'uptake and secretion of cholic glycocholate - GCHOLAt2u';
4838     if ~isnan(TestSolution(k,1)); TestedRxns = [TestedRxns; model.rxns(find(abs(FBA.x)>tol))]; end ;k = k +1;clear FBA
4839     model=changeObjective(model,'GCHOLAt3');
4840     %FBA=optimizeCbModel(model,'min');
4841     FBA=optimizeCbModel(model,'max');
4842     TestSolution(k,1) = FBA.f;
4843     TestSolutionName{k,1} = 'uptake and secretion of cholic glycocholate - GCHOLAt3';
4844     if ~isnan(TestSolution(k,1)); TestedRxns = [TestedRxns; model.rxns(find(abs(FBA.x)>tol))]; end ;k = k +1;clear FBA
4845     
4846     % 31); Uptake and secretion of tauro-cholate
4847     model=modelOri;
4848     model=changeRxnBounds(model,'EX_o2(e)',-1,'l');
4849     model=changeRxnBounds(model,'EX_TCHOLA(u)',-1,'l');
4850     model=changeObjective(model,'TCHOLAt2u');
4851     %FBA=optimizeCbModel(model,'min');
4852     FBA=optimizeCbModel(model,'max');
4853     TestSolution(k,1) = FBA.f;
4854     TestSolutionName{k,1} = 'uptake and secretion of tauro-cholate - TCHOLAt2u';
4855     if ~isnan(TestSolution(k,1)); TestedRxns = [TestedRxns; model.rxns(find(abs(FBA.x)>tol))]; end ;k = k +1;clear FBA
4856     model=changeObjective(model,'TCHOLAt3');
4857     %FBA=optimizeCbModel(model,'min');
4858     FBA=optimizeCbModel(model,'max');
4859     TestSolution(k,1) = FBA.f;
4860     TestSolutionName{k,1} = 'uptake and secretion of tauro-cholate - TCHOLAt3';
4861     if ~isnan(TestSolution(k,1)); TestedRxns = [TestedRxns; model.rxns(find(abs(FBA.x)>tol))]; end ;k = k +1;clear FBA
4862     
4863     % 32); Synthesis of fructose-6-phosphate from erythrose-4-phosphate (HMP shunt);
4864     model=modelOri;
4865     model=changeRxnBounds(model,'EX_o2(e)',-1,'l');
4866     model=changeObjective(model,'TKT2');
4867     %FBA=optimizeCbModel(model,'min');
4868     FBA=optimizeCbModel(model,'max');
4869     TestSolution(k,1) = FBA.f;
4870     TestSolutionName{k,1} = 'Synthesis of fructose-6-phosphate from erythrose-4-phosphate (HMP shunt) - TKT2';
4871     if ~isnan(TestSolution(k,1)); TestedRxns = [TestedRxns; model.rxns(find(abs(FBA.x)>tol))]; end ;k = k +1;clear FBA
4872     
4873     % 33); Malate to pyruvate (malic enzyme);
4874     model=modelOri;
4875     model=changeRxnBounds(model,'EX_o2(e)',-1,'l');
4876     model=changeObjective(model,'ME2');
4877     %FBA=optimizeCbModel(model,'min');
4878     FBA=optimizeCbModel(model,'max');
4879     TestSolution(k,1) = FBA.f;
4880     TestSolutionName{k,1} = 'Malate to pyruvate (malic enzyme) - ME2';
4881     if ~isnan(TestSolution(k,1)); TestedRxns = [TestedRxns; model.rxns(find(abs(FBA.x)>tol))]; end ;k = k +1;clear FBA
4882     model=changeObjective(model,'ME2m');
4883     %FBA=optimizeCbModel(model,'min');
4884     FBA=optimizeCbModel(model,'max');
4885     TestSolution(k,1) = FBA.f;
4886     TestSolutionName{k,1} = 'Malate to pyruvate (malic enzyme) - ME2m';
4887     if ~isnan(TestSolution(k,1)); TestedRxns = [TestedRxns; model.rxns(find(abs(FBA.x)>tol))]; end ;k = k +1;clear FBA
4888     
4889     % 34); Synthesis of urea (urea cycle);
4890     model=modelOri;
4891     model=changeRxnBounds(model,'EX_o2(e)',-1,'l');
4892     model=changeRxnBounds(model,'EX_arg_L(e)',-1,'l');
4893     model=changeObjective(model,'ARGN');
4894     %FBA=optimizeCbModel(model,'min');
4895     FBA=optimizeCbModel(model,'max');
4896     TestSolution(k,1) = FBA.f;
4897     TestSolutionName{k,1} = 'Synthesis of urea (urea cycle) - ARGN';
4898     if ~isnan(TestSolution(k,1)); TestedRxns = [TestedRxns; model.rxns(find(abs(FBA.x)>tol))]; end ;k = k +1;clear FBA
4899     
4900     % 35); Cysteine to pyruvate
4901     model=modelOri;
4902     model=changeRxnBounds(model,'EX_o2(e)',-1,'l');
4903     model=changeRxnBounds(model,'EX_cys-L(u)',-1,'b');
4904     model=changeRxnBounds(model,'EX_glc_D(u)',0,'b');
4905     model=changeRxnBounds(model,'EX_SUCR(u)',0,'b');
4906     model=changeRxnBounds(model,'EX_malt(u)',0,'b');
4907     model=changeRxnBounds(model,'EX_STRCH1(u)',0,'b');
4908     model=changeRxnBounds(model,'EX_strch2(u)',0,'b');
4909     model=changeObjective(model,'3SPYRSP');
4910     %FBA=optimizeCbModel(model,'min');
4911     FBA=optimizeCbModel(model,'max');
4912     TestSolution(k,1) = FBA.f;
4913     TestSolutionName{k,1} = 'Cysteine to pyruvate - 3SPYRSP';
4914     if ~isnan(TestSolution(k,1)); TestedRxns = [TestedRxns; model.rxns(find(abs(FBA.x)>tol))]; end ;k = k +1;clear FBA
4915     
4916     % 36); Methionine to cysteine  (check for dependancy over pe_hs);
4917     model=modelOri;
4918     model=changeRxnBounds(model,'EX_o2(e)',-1,'l');
4919     model=changeRxnBounds(model,'EX_pe_hs(u)',-1,'l');
4920     model=changeRxnBounds(model,'EX_cys_L(u)',0,'b');
4921     model=changeObjective(model,'CYSTGL');
4922     %FBA=optimizeCbModel(model,'min');
4923     FBA=optimizeCbModel(model,'max');
4924     TestSolution(k,1) = FBA.f;
4925     TestSolutionName{k,1} = 'Methionine to cysteine - CYSTGL';
4926     if ~isnan(TestSolution(k,1)); TestedRxns = [TestedRxns; model.rxns(find(abs(FBA.x)>tol))]; end ;k = k +1;clear FBA
4927     
4928     % 37); Synthesis of triacylglycerol (TAG reformation); (check for dependancy over dag_hs and RTOTAL3);
4929     model=modelOri;
4930     model=changeRxnBounds(model,'EX_o2(e)',-1,'l');
4931     model=changeRxnBounds(model,'EX_dag_hs(u)',-1,'l');
4932     model=changeRxnBounds(model,'EX_RTOTAL3(u)',-1,'l');
4933     model=changeRxnBounds(model,'EX_TAG_HS(u)',0,'b');
4934     model=changeObjective(model,'DGAT');
4935     %FBA=optimizeCbModel(model,'min');
4936     FBA=optimizeCbModel(model,'max');
4937     TestSolution(k,1) = FBA.f;
4938     TestSolutionName{k,1} = 'Synthesis of triacylglycerol (TAG reformation) - DGAT';
4939     if ~isnan(TestSolution(k,1)); TestedRxns = [TestedRxns; model.rxns(find(abs(FBA.x)>tol))]; end ;k = k +1;clear FBA
4940     
4941     % 38); Phosphatidylcholine synthesis (check for dependancy over pe_hs);
4942     model=modelOri;
4943     model=changeRxnBounds(model,'EX_o2(e)',-1,'l');
4944     model=changeRxnBounds(model,'EX_pe_hs(u)',-1,'l');
4945     model=changeRxnBounds(model,'EX_PCHOL_HS(u)',0,'b');
4946     model=changeObjective(model,'PETOHMm_hs');
4947     %FBA=optimizeCbModel(model,'min');
4948     FBA=optimizeCbModel(model,'max');
4949     TestSolution(k,1) = FBA.f;
4950     TestSolutionName{k,1} = 'Phosphatidylcholine synthesis - PETOHMm_hs';
4951     if ~isnan(TestSolution(k,1)); TestedRxns = [TestedRxns; model.rxns(find(abs(FBA.x)>tol))]; end ;k = k +1;clear FBA
4952     
4953     % 39); Synthesis of FMN from riboflavin
4954     model=modelOri;
4955     model=changeRxnBounds(model,'EX_o2(e)',-1,'l');
4956     model=changeRxnBounds(model,'EX_fmn(u)',0,'b');
4957     model=changeObjective(model,'RBFK');
4958     %FBA=optimizeCbModel(model,'min');
4959     FBA=optimizeCbModel(model,'max');
4960     TestSolution(k,1) = FBA.f;
4961     TestSolutionName{k,1} = 'Synthesis of FMN from riboflavin - RBFK';
4962     if ~isnan(TestSolution(k,1)); TestedRxns = [TestedRxns; model.rxns(find(abs(FBA.x)>tol))]; end ;k = k +1;clear FBA
4963     
4964     % 40); synthesis of FAD from riboflavin
4965     model=modelOri;
4966     model=changeRxnBounds(model,'EX_o2(e)',-1,'l');
4967     model=changeRxnBounds(model,'EX_FAD(u)',0,'b');
4968     model=changeObjective(model,'FMNAT');
4969     %FBA=optimizeCbModel(model,'min');
4970     FBA=optimizeCbModel(model,'max');
4971     TestSolution(k,1) = FBA.f;
4972     TestSolutionName{k,1} = 'synthesis of FAD from riboflavin - FMNAT';
4973     if ~isnan(TestSolution(k,1)); TestedRxns = [TestedRxns; model.rxns(find(abs(FBA.x)>tol))]; end ;k = k +1;clear FBA
4974     
4975     % 41); Synthesis of 5-methyl-tetrahydrofolate from folic acid
4976     model=modelOri;
4977     model=changeRxnBounds(model,'EX_o2(e)',-1,'l');
4978     model=changeRxnBounds(model,'EX_5mthf(u)',0,'b');
4979     model=changeObjective(model,'MTHFR3');
4980     %FBA=optimizeCbModel(model,'min');
4981     FBA=optimizeCbModel(model,'max');
4982     TestSolution(k,1) = FBA.f;
4983     TestSolutionName{k,1} = 'Synthesis of 5-methyl-tetrahydrofolate from folic acid - MTHFR3';
4984     if ~isnan(TestSolution(k,1)); TestedRxns = [TestedRxns; model.rxns(find(abs(FBA.x)>tol))]; end ;k = k +1;clear FBA
4985     
4986     % 42); Putriscine to GABA
4987     model=modelOri;
4988     model=changeRxnBounds(model,'EX_o2(e)',-1,'l');
4989     model=changeRxnBounds(model,'EX_4ABUT(u)',0,'b');
4990     model=changeObjective(model,'ABUTD');
4991     %FBA=optimizeCbModel(model,'min');
4992     FBA=optimizeCbModel(model,'max');
4993     TestSolution(k,1) = FBA.f;
4994     TestSolutionName{k,1} = 'Putriscine to GABA - ABUTD';
4995     if ~isnan(TestSolution(k,1)); TestedRxns = [TestedRxns; model.rxns(find(abs(FBA.x)>tol))]; end ;k = k +1;clear FBA
4996     
4997     % 43); Superoxide dismutase
4998     model=modelOri;
4999     model=changeRxnBounds(model,'EX_o2(e)',-1,'l');
5000     model=changeObjective(model,'SPODMm');
5001     %FBA=optimizeCbModel(model,'min');
5002     FBA=optimizeCbModel(model,'max');
5003     TestSolution(k,1) = FBA.f;
5004     TestSolutionName{k,1} = 'Superoxide dismutase - SPODMm';
5005     if ~isnan(TestSolution(k,1)); TestedRxns = [TestedRxns; model.rxns(find(abs(FBA.x)>tol))]; end ;k = k +1;clear FBA
5006     
5007     % 44); Availability of bicarbonate from Carbonic anhydrase reaction
5008     model=modelOri;
5009     model=changeRxnBounds(model,'EX_o2(e)',-1,'l');
5010     model=changeObjective(model,'H2CO3Dm');
5011     %FBA=optimizeCbModel(model,'min');
5012     FBA=optimizeCbModel(model,'max');
5013     TestSolution(k,1) = FBA.f;
5014     TestSolutionName{k,1} = 'Availability of bicarbonate from Carbonic anhydrase reaction - H2CO3Dm';
5015     if ~isnan(TestSolution(k,1)); TestedRxns = [TestedRxns; model.rxns(find(abs(FBA.x)>tol))]; end ;k = k +1;clear FBA
5016     
5017     % 45); Regeneration of citrate (TCA cycle);
5018     model=modelOri;
5019     model=changeRxnBounds(model,'EX_o2(e)',-1,'l');
5020     model=changeObjective(model,'CSm');
5021     %FBA=optimizeCbModel(model,'min');
5022     FBA=optimizeCbModel(model,'max');
5023     TestSolution(k,1) = FBA.f;
5024     TestSolutionName{k,1} = 'Regeneration of citrate (TCA cycle) - CSm';
5025     if ~isnan(TestSolution(k,1)); TestedRxns = [TestedRxns; model.rxns(find(abs(FBA.x)>tol))]; end ;k = k +1;clear FBA
5026     
5027     % 46); Histidine to FIGLU
5028     model=modelOri;
5029     model=changeRxnBounds(model,'EX_o2(e)',-1,'l');
5030     model=changeObjective(model,'IZPN');
5031     FBA=optimizeCbModel(model,'min');
5032     FBA=optimizeCbModel(model,'max');
5033     
5034     % 47); binding of guar gum fiber to bile acids
5035     model=modelOri;
5036     model=changeRxnBounds(model,'EX_GUM(u)',-1,'l');
5037     model=changeRxnBounds(model,'EX_GCHOLA(u)',-1,'l');
5038     model=changeObjective(model,'EX_GUMGCHOL(u)');
5039     FBA=optimizeCbModel(model,'min');
5040     %FBA=optimizeCbModel(model,'max');
5041     TestSolution(k,1) = FBA.f;
5042     TestSolutionName{k,1} = 'binding of guar gum fiber to bile acids - EX_GUMGCHOL(e)';
5043     if ~isnan(TestSolution(k,1)); TestedRxns = [TestedRxns; model.rxns(find(abs(FBA.x)>tol))]; end ;k = k +1;clear FBA
5044     model=changeRxnBounds(model,'EX_TCHOLA(u)',-1,'l');
5045     model=changeObjective(model,'GUMTCHOLe');
5046     %FBA=optimizeCbModel(model,'min');
5047     FBA=optimizeCbModel(model,'max');
5048     TestSolution(k,1) = FBA.f;
5049     TestSolutionName{k,1} = 'binding of guar gum fiber to bile acids - GUMTCHOLe';
5050     if ~isnan(TestSolution(k,1)); TestedRxns = [TestedRxns; model.rxns(find(abs(FBA.x)>tol))]; end ;k = k +1;clear FBA
5051     model=changeRxnBounds(model,'EX_DCHAC(u)',-1,'l');
5052     model=changeObjective(model,'GUMDCHAe');
5053     %FBA=optimizeCbModel(model,'min');
5054     FBA=optimizeCbModel(model,'max');
5055     TestSolution(k,1) = FBA.f;
5056     TestSolutionName{k,1} = 'binding of guar gum fiber to bile acids - GUMDCHAe';
5057     if ~isnan(TestSolution(k,1)); TestedRxns = [TestedRxns; model.rxns(find(abs(FBA.x)>tol))]; end ;k = k +1;clear FBA
5058     
5059     % 48); binding of psyllium fiber to bile acids
5060     model=modelOri;
5061     model=changeRxnBounds(model,'EX_PSYL(u)',-1,'l');
5062     model=changeRxnBounds(model,'EX_GCHOLA(u)',-1,'l');
5063     model=changeObjective(model,'PSYGCHe');
5064     %FBA=optimizeCbModel(model,'min');
5065     FBA=optimizeCbModel(model,'max');
5066     TestSolution(k,1) = FBA.f;
5067     TestSolutionName{k,1} = 'binding of psyllium fiber to bile acids - PSYGCHe';
5068     if ~isnan(TestSolution(k,1)); TestedRxns = [TestedRxns; model.rxns(find(abs(FBA.x)>tol))]; end ;k = k +1;clear FBA
5069     model=changeRxnBounds(model,'EX_TCHOLA(u)',-1,'l');
5070     model=changeObjective(model,'PSYTCHe');
5071     FBA=optimizeCbModel(model,'min');
5072     %FBA=optimizeCbModel(model,'max');
5073     TestSolution(k,1) = FBA.f;
5074     TestSolutionName{k,1} = 'binding of psyllium fiber to bile acids - PSYTCHe';
5075     if ~isnan(TestSolution(k,1)); TestedRxns = [TestedRxns; model.rxns(find(abs(FBA.x)>tol))]; end ;k = k +1;clear FBA
5076     model=changeRxnBounds(model,'EX_TDECHOLA(u)',-1,'l');
5077     model=changeObjective(model,'PSYTDECHe');
5078     %FBA=optimizeCbModel(model,'min');
5079     FBA=optimizeCbModel(model,'max');
5080     TestSolution(k,1) = FBA.f;
5081     TestSolutionName{k,1} = 'binding of psyllium fiber to bile acids - PSYTDECHe';
5082     if ~isnan(TestSolution(k,1)); TestedRxns = [TestedRxns; model.rxns(find(abs(FBA.x)>tol))]; end ;k = k +1;clear FBA
5083     
5084     % 49);binding to beta glucan fibers to bile acids
5085     model=modelOri;
5086     model=changeRxnBounds(model,'EX_BGLC(u)',-1,'l');
5087     model=changeRxnBounds(model,'EX_GCHOLA(u)',-1,'l');
5088     model=changeObjective(model,'BGLUGCHe');
5089     %FBA=optimizeCbModel(model,'min');
5090     FBA=optimizeCbModel(model,'max');
5091     TestSolution(k,1) = FBA.f;
5092     TestSolutionName{k,1} = 'binding to beta glucan fibers to bile acids - BGLUGCHe';
5093     if ~isnan(TestSolution(k,1)); TestedRxns = [TestedRxns; model.rxns(find(abs(FBA.x)>tol))]; end ;k = k +1;clear FBA
5094     model=changeRxnBounds(model,'EX_TCHOLA(u)',-1,'l');
5095     model=changeObjective(model,'BGLUTCHLe');
5096     %FBA=optimizeCbModel(model,'min');
5097     FBA=optimizeCbModel(model,'max');
5098     TestSolution(k,1) = FBA.f;
5099     TestSolutionName{k,1} = 'binding to beta glucan fibers to bile acids - BGLUTCHLe';
5100     if ~isnan(TestSolution(k,1)); TestedRxns = [TestedRxns; model.rxns(find(abs(FBA.x)>tol))]; end ;k = k +1;clear FBA
5101     model=changeRxnBounds(model,'EX_TDECHOLA(u)',-1,'l');
5102     model=changeObjective(model,'BGLUTDECHOe');
5103     %FBA=optimizeCbModel(model,'min');
5104     FBA=optimizeCbModel(model,'max');
5105     TestSolution(k,1) = FBA.f;
5106     TestSolutionName{k,1} = 'binding to beta glucan fibers to bile acids - BGLUTDECHOe';
5107     if ~isnan(TestSolution(k,1)); TestedRxns = [TestedRxns; model.rxns(find(abs(FBA.x)>tol))]; end ;k = k +1;clear FBA
5108     
5109     % 50); binding of pectin fiber to bile acids
5110     model=modelOri;
5111     model=changeRxnBounds(model,'EX_PECT(u)',-1,'l');
5112     model=changeRxnBounds(model,'EX_GCHOLA(u)',-1,'l');
5113     model=changeObjective(model,'PECGCHLe');
5114     %FBA=optimizeCbModel(model,'min');
5115     FBA=optimizeCbModel(model,'max');
5116     TestSolution(k,1) = FBA.f;
5117     TestSolutionName{k,1} = 'binding of pectin fiber to bile acids - PECGCHLe';
5118     if ~isnan(TestSolution(k,1)); TestedRxns = [TestedRxns; model.rxns(find(abs(FBA.x)>tol))]; end ;k = k +1;clear FBA
5119     model=changeRxnBounds(model,'EX_TCHOLA(u)',-1,'l');
5120     model=changeObjective(model,'PECTCHLe');
5121     %FBA=optimizeCbModel(model,'min');
5122     FBA=optimizeCbModel(model,'max');
5123     TestSolution(k,1) = FBA.f;
5124     TestSolutionName{k,1} = 'binding of pectin fiber to bile acids - PECTCHLe';
5125     if ~isnan(TestSolution(k,1)); TestedRxns = [TestedRxns; model.rxns(find(abs(FBA.x)>tol))]; end ;k = k +1;clear FBA
5126     model=changeRxnBounds(model,'EX_DCHAC(u)',-1,'l');
5127     model=changeObjective(model,'PECDCHe');
5128     %FBA=optimizeCbModel(model,'min');
5129     FBA=optimizeCbModel(model,'max');
5130     TestSolution(k,1) = FBA.f;
5131     TestSolutionName{k,1} = 'binding of pectin fiber to bile acids - PECDCHe';
5132     if ~isnan(TestSolution(k,1)); TestedRxns = [TestedRxns; model.rxns(find(abs(FBA.x)>tol))]; end ;k = k +1;clear FBA
5133     
5134     % 52); heme synthesis
5135     model=modelOri;
5136     model=changeRxnBounds(model,'EX_o2(e)',-1,'l');
5137     model=changeObjective(model,'FCLTm');
5138     %FBA=optimizeCbModel(model,'min');
5139     FBA=optimizeCbModel(model,'max');
5140     TestSolution(k,1) = FBA.f;
5141     TestSolutionName{k,1} = 'heme synthesis - FCLTm';
5142     if ~isnan(TestSolution(k,1)); TestedRxns = [TestedRxns; model.rxns(find(abs(FBA.x)>tol))]; end ;k = k +1;clear FBA
5143     
5144     % 53); heme degradation
5145     model=modelOri;
5146     model=changeRxnBounds(model,'EX_o2(e)',-1,'l');
5147     model=changeObjective(model,'HOXG');
5148     %FBA=optimizeCbModel(model,'min');
5149     FBA=optimizeCbModel(model,'max');
5150     TestSolution(k,1) = FBA.f;
5151     TestSolutionName{k,1} = 'heme degradation - HOXG';
5152     if ~isnan(TestSolution(k,1)); TestedRxns = [TestedRxns; model.rxns(find(abs(FBA.x)>tol))]; end ;k = k +1;clear FBA
5153 end
5154 
5155 %% metabolic tasks based on Enterocyte model - without original ('u')
5156 % compartment I deleted the last argument in changeObjective from here
5157 % onwards (SS)
5158 if strcmp(test,'IEC') || strcmp(test,'all')|| strcmp(test,'Harvey')
5159     %% glucose to lactate conversion
5160     model=modelOri;
5161     model=changeRxnBounds(model,'EX_glc(e)',-1,'b');
5162     if ~isempty(strmatch('EX_lac-L(e)',model.rxns,'exact'))
5163         model=changeObjective(model,'EX_lac-L(e)',1);
5164         FBA = optimizeCbModel(model,'max');
5165         TestSolution(k,1) = FBA.f;
5166     else
5167         TestSolution(k,1) = NaN;
5168     end
5169     TestSolutionName{k,1} = 'glucose to lactate conversion';
5170     if ~isnan(TestSolution(k,1)); TestedRxns = [TestedRxns; model.rxns(find(abs(FBA.x)>tol))]; end ;k = k +1;clear FBA
5171     
5172     %%  glutamine to glucose conversion
5173     model=modelOri;
5174     model=changeRxnBounds(model,'EX_gln-L(e)',-1,'b');
5175     model=changeRxnBounds(model,'EX_glc(e)',0,'b');
5176     model=changeRxnBounds(model,'EX_malt(e)',0,'b');
5177     model=changeRxnBounds(model,'EX_strch1(e)',0,'b');
5178     model=changeRxnBounds(model,'EX_strch2(e)',0,'b');
5179     model=changeRxnBounds(model,'EX_sucr(e)',0,'b');
5180     
5181     if ~isempty(strmatch('GLUNm',model.rxns,'exact'))
5182         model=changeObjective(model,'GLUNm',1);
5183         %FBA=optimizeCbModel(model,'min');
5184         FBA = optimizeCbModel(model,'max');
5185         TestSolution(k,1) = FBA.f;
5186     else
5187         TestSolution(k,1) = NaN;
5188     end
5189     TestSolutionName{k,1} = 'glutamine to glucose conversion - GLUNm';
5190     if ~isnan(TestSolution(k,1)); TestedRxns = [TestedRxns; model.rxns(find(abs(FBA.x)>tol))]; end ;k = k +1;clear FBA
5191     
5192     %% glutamine to glucose conversion - ASPTAm
5193     if ~isempty(strmatch('ASPTAm',model.rxns,'exact'))
5194         model=changeObjective(model,'ASPTAm',1);
5195         FBA = optimizeCbModel(model,'max');
5196         TestSolution(k,1) = FBA.f;
5197     else
5198         TestSolution(k,1) = NaN;
5199     end
5200     TestSolutionName{k,1} = 'glutamine to glucose conversion - ASPTAm';
5201     if ~isnan(TestSolution(k,1)); TestedRxns = [TestedRxns; model.rxns(find(abs(FBA.x)>tol))]; end ;k = k +1;clear FBA
5202     
5203     %% 'glutamine to glucose conversion - FUM'
5204     if ~isempty(strmatch('FUM',model.rxns,'exact'))
5205         model=changeObjective(model,'FUM',1);
5206         FBA = optimizeCbModel(model,'max');
5207         TestSolution(k,1) = FBA.f;
5208     else
5209         TestSolution(k,1) = NaN;
5210     end
5211     TestSolutionName{k,1} = 'glutamine to glucose conversion - FUM';
5212     if ~isnan(TestSolution(k,1)); TestedRxns = [TestedRxns; model.rxns(find(abs(FBA.x)>tol))]; end ;k = k +1;clear FBA
5213     %% glutamine to glucose conversion - MDH
5214     if ~isempty(strmatch('MDH',model.rxns,'exact'))
5215         model=changeObjective(model,'MDH',1);
5216         FBA = optimizeCbModel(model,'max');
5217         TestSolution(k,1) = FBA.f;
5218     else
5219         TestSolution(k,1) = NaN;
5220     end
5221     TestSolutionName{k,1} = 'glutamine to glucose conversion - MDH';
5222     if ~isnan(TestSolution(k,1)); TestedRxns = [TestedRxns; model.rxns(find(abs(FBA.x)>tol))]; end ;k = k +1;clear FBA
5223     %% glutamine to glucose conversion - G6PPer
5224     if ~isempty(strmatch('G6PPer',model.rxns,'exact'))
5225         model=changeObjective(model,'G6PPer',1);
5226         FBA = optimizeCbModel(model,'max');
5227         FBA = optimizeCbModel(model,'max');
5228         TestSolution(k,1) = FBA.f;
5229     else
5230         TestSolution(k,1) = NaN;
5231     end
5232     TestSolutionName{k,1} = 'glutamine to glucose conversion - G6PPer';
5233     if ~isnan(TestSolution(k,1)); TestedRxns = [TestedRxns; model.rxns(find(abs(FBA.x)>tol))]; end ;k = k +1;clear FBA
5234     %% glutamine to proline conversion
5235     model=modelOri;
5236     model=changeRxnBounds(model,'EX_gln-L(e)',-1,'b');
5237     model=changeRxnBounds(model,'EX_glc(e)',0,'b');
5238     model=changeRxnBounds(model,'EX_pro-L(e)',0,'b');
5239     
5240     if ~isempty(strmatch('P5CRm',model.rxns,'exact'))
5241         model=changeObjective(model,'P5CRm',1);
5242         FBA = optimizeCbModel(model,'max');
5243         TestSolution(k,1) = FBA.f;
5244     else
5245         TestSolution(k,1) = NaN;
5246     end
5247     TestSolutionName{k,1} = 'glutamine to proline conversion - P5CRm';
5248     if ~isnan(TestSolution(k,1)); TestedRxns = [TestedRxns; model.rxns(find(abs(FBA.x)>tol))]; end ;k = k +1;clear FBA
5249     %% glutamine to proline conversion - P5CRxm
5250     model=modelOri;
5251     model=changeRxnBounds(model,'EX_gln-L(e)',-1,'b');
5252     model=changeRxnBounds(model,'EX_glc(e)',0,'b');
5253     model=changeRxnBounds(model,'EX_pro-L(e)',0,'b');
5254     
5255     if ~isempty(strmatch('P5CRxm',model.rxns,'exact'))
5256         model=changeObjective(model,'P5CRxm',1);
5257         FBA = optimizeCbModel(model,'max');
5258         TestSolution(k,1) = FBA.f;
5259     else
5260         TestSolution(k,1) = NaN;
5261     end
5262     TestSolutionName{k,1} = 'glutamine to proline conversion - P5CRxm';
5263     if ~isnan(TestSolution(k,1)); TestedRxns = [TestedRxns; model.rxns(find(abs(FBA.x)>tol))]; end ;k = k +1;clear FBA
5264     
5265     
5266     %% glutamine to ornithine conversion
5267     model=modelOri;
5268     
5269     model=changeRxnBounds(model,'EX_gln-L(e)',-1,'l');
5270     if ~isempty(strmatch('ORNTArm',model.rxns,'exact'))
5271         model=changeObjective(model,'ORNTArm',1);
5272         FBA = optimizeCbModel(model,'max');
5273         TestSolution(k,1) = FBA.f;
5274     else
5275         TestSolution(k,1) = NaN;
5276     end
5277     TestSolutionName{k,1} = 'glutamine to ornithine conversion - ORNTArm';
5278     if ~isnan(TestSolution(k,1)); TestedRxns = [TestedRxns; model.rxns(find(abs(FBA.x)>tol))]; end ;k = k +1;clear FBA
5279     
5280     %% glutamine to citrulline converion
5281     model=modelOri;
5282     model=changeRxnBounds(model,'EX_gln-L(e)',-1,'b');
5283     if ~isempty(strmatch('OCBTm',model.rxns,'exact'))
5284         model=changeObjective(model,'OCBTm',1);
5285         FBA = optimizeCbModel(model,'max');
5286         TestSolution(k,1) = FBA.f;
5287     else
5288         TestSolution(k,1) = NaN;
5289     end
5290     TestSolutionName{k,1} = 'glutamine to citrulline converion - OCBTm';
5291     if ~isnan(TestSolution(k,1)); TestedRxns = [TestedRxns; model.rxns(find(abs(FBA.x)>tol))]; end ;k = k +1;clear FBA
5292     
5293     %% glutamine to lactate
5294     model=modelOri;
5295     model=changeRxnBounds(model,'EX_gln-L(e)',-1,'b');
5296     if ~isempty(strmatch('LDH_L',model.rxns,'exact'))
5297         model=changeObjective(model,'LDH_L',1);
5298         FBA = optimizeCbModel(model,'max');
5299         TestSolution(k,1) = FBA.f;
5300     else
5301         TestSolution(k,1) = NaN;
5302     end
5303     TestSolutionName{k,1} = 'glutamine to lactate - LDH_L';
5304     if ~isnan(TestSolution(k,1)); TestedRxns = [TestedRxns; model.rxns(find(abs(FBA.x)>tol))]; end ;k = k +1;clear FBA
5305     
5306     %% glutamine to aspartate
5307     model=modelOri;
5308     model=changeRxnBounds(model,'EX_gln-L(e)',-1,'b');
5309     if ~isempty(strmatch('ASPTA',model.rxns,'exact'))
5310         model=changeObjective(model,'ASPTA',1);
5311         FBA = optimizeCbModel(model,'max');
5312         TestSolution(k,1) = FBA.f;
5313     else
5314         TestSolution(k,1) = NaN;
5315     end
5316     TestSolutionName{k,1} = 'glutamine to aspartate - ASPTA';
5317     if ~isnan(TestSolution(k,1)); TestedRxns = [TestedRxns; model.rxns(find(abs(FBA.x)>tol))]; end ;k = k +1;clear FBA
5318     
5319     %% glutamine to co2
5320     model=modelOri;
5321     model=changeRxnBounds(model,'EX_gln-L(e)',-1,'b');
5322     if ~isempty(strmatch('AKGDm',model.rxns,'exact'))
5323         model=changeObjective(model,'AKGDm',1);
5324         FBA = optimizeCbModel(model,'max');
5325         TestSolution(k,1) = FBA.f;
5326     else
5327         TestSolution(k,1) = NaN;
5328     end
5329     TestSolutionName{k,1} = 'glutamine to co2 - AKGDm';
5330     if ~isnan(TestSolution(k,1)); TestedRxns = [TestedRxns; model.rxns(find(abs(FBA.x)>tol))]; end ;k = k +1;clear FBA
5331     %% glutamine to ammonia
5332     model=modelOri;
5333     model=changeRxnBounds(model,'EX_gln-L(e)',-1,'b');
5334     if ~isempty(strmatch('GLUNm',model.rxns,'exact'))
5335         model=changeObjective(model,'GLUNm',1);
5336         FBA = optimizeCbModel(model,'max');
5337         TestSolution(k,1) = FBA.f;
5338     else
5339         TestSolution(k,1) = NaN;
5340     end
5341     TestSolutionName{k,1} = 'glutamine to ammonia - GLUNm';
5342     if ~isnan(TestSolution(k,1)); TestedRxns = [TestedRxns; model.rxns(find(abs(FBA.x)>tol))]; end ;k = k +1;clear FBA
5343     
5344     %% putriscine to methionine (depends on oxygen uptake);
5345     model=modelOri;
5346     model=changeRxnBounds(model,'EX_ptrc(e)',-1,'b');
5347     model=changeRxnBounds(model,'EX_o2(e)',-1,'l');
5348     if ~isempty(strmatch('UNK2',model.rxns,'exact'))
5349         model=changeObjective(model,'UNK2',1);
5350         FBA = optimizeCbModel(model,'max');
5351         TestSolution(k,1) = FBA.f;
5352     else
5353         TestSolution(k,1) = NaN;
5354     end
5355     TestSolutionName{k,1} = 'putriscine to methionine (depends on oxygen uptake) - UNK2';
5356     if ~isnan(TestSolution(k,1)); if ~isnan(TestSolution(k,1)); TestedRxns = [TestedRxns; model.rxns(find(abs(FBA.x)>tol))]; end ; end ;k = k +1;clear FBA
5357     
5358     %%  secretion of alanine
5359     model=modelOri;
5360     
5361     model.lb(ismember(model.rxns,'EX_glc(e)'))=-1;model.ub(ismember(model.rxns,'EX_glc(e)'))=0;
5362     model.lb(ismember(model.rxns,'EX_o2(e)'))=-40;model.ub(ismember(model.rxns,'EX_o2(e)'))=-1;
5363     if ~isempty(strmatch('EX_ala_L(e)',model.rxns,'exact'))
5364         model=changeObjective(model,'EX_ala_L(e)',1);
5365         %FBA=optimizeCbModel(model,'min');
5366         FBA = optimizeCbModel(model,'max');
5367         TestSolution(k,1) = FBA.f;
5368     else
5369         TestSolution(k,1) = NaN;
5370     end
5371     TestSolutionName{k,1} = 'secretion of alanine';
5372     if ~isnan(TestSolution(k,1)); TestedRxns = [TestedRxns; model.rxns(find(abs(FBA.x)>tol))]; end ;k = k +1;clear FBA
5373     
5374     %%  secretion of lactate
5375     model=modelOri;
5376     model.lb(ismember(model.rxns,'EX_glc(e)'))=-1;model.ub(ismember(model.rxns,'EX_glc(e)'))=0;
5377     model.lb(ismember(model.rxns,'EX_o2(e)'))=-40;model.ub(ismember(model.rxns,'EX_o2(e)'))=-1;
5378     if ~isempty(strmatch('EX_lac-L(e)',model.rxns,'exact'))
5379         model=changeObjective(model,'EX_lac-L(e)');
5380         FBA = optimizeCbModel(model,'max');
5381         TestSolution(k,1) = FBA.f;
5382     else
5383         TestSolution(k,1) = NaN;
5384     end
5385     TestSolutionName{k,1} = 'secretion of lactate';
5386     if ~isnan(TestSolution(k,1)); TestedRxns = [TestedRxns; model.rxns(find(abs(FBA.x)>tol))]; end ;k = k +1;clear FBA
5387     
5388     %% synthesis of arginine from glutamine
5389     model=modelOri;
5390     model=changeRxnBounds(model,'EX_gln-L(e)',-1,'b');
5391     model.lb(ismember(model.rxns,'EX_o2(e)'))=-40;model.ub(ismember(model.rxns,'EX_o2(e)'))=-1;
5392     if ~isempty(strmatch('ARGSL',model.rxns,'exact'))
5393         model=changeObjective(model,'ARGSL',1);
5394         FBA = optimizeCbModel(model,'max');
5395         TestSolution(k,1) = FBA.f;
5396     else
5397         TestSolution(k,1) = NaN;
5398     end
5399     TestSolutionName{k,1} = 'synthesis of arginine from glutamine - ARGSL';
5400     if ~isnan(TestSolution(k,1)); TestedRxns = [TestedRxns; model.rxns(find(abs(FBA.x)>tol))]; end ;k = k +1;clear FBA
5401     
5402     
5403     %% synthesis of proline from glutamine
5404     model=modelOri;
5405     model=changeRxnBounds(model,'EX_gln-L(e)',-1,'b');
5406     model.lb(ismember(model.rxns,'EX_o2(e)'))=-40;model.ub(ismember(model.rxns,'EX_o2(e)'))=-1;
5407     if ~isempty(strmatch('P5CR',model.rxns,'exact'))
5408         model=changeObjective(model,'P5CR',1);
5409         FBA = optimizeCbModel(model,'max');
5410         TestSolution(k,1) = FBA.f;
5411     else
5412         TestSolution(k,1) = NaN;
5413     end
5414     TestSolutionName{k,1} = 'synthesis of proline from glutamine - P5CR';
5415     if ~isnan(TestSolution(k,1)); TestedRxns = [TestedRxns; model.rxns(find(abs(FBA.x)>tol))]; end ;k = k +1;clear FBA
5416     %% synthesis of proline from glutamine
5417     model=modelOri;
5418     model=changeRxnBounds(model,'EX_gln-L(e)',-1,'b');
5419     model.lb(ismember(model.rxns,'EX_o2(e)'))=-40;model.ub(ismember(model.rxns,'EX_o2(e)'))=-1;
5420     if ~isempty(strmatch('P5CRm',model.rxns,'exact'))
5421         model=changeObjective(model,'P5CRm',1);
5422         FBA = optimizeCbModel(model,'max');
5423         TestSolution(k,1) = FBA.f;
5424     else
5425         TestSolution(k,1) = NaN;
5426     end
5427     TestSolutionName{k,1} = 'synthesis of proline from glutamine - P5CRm';
5428     if ~isnan(TestSolution(k,1)); TestedRxns = [TestedRxns; model.rxns(find(abs(FBA.x)>tol))]; end ;k = k +1;clear FBA
5429     
5430     %% synthesis of proline from glutamine
5431     model=modelOri;
5432     model=changeRxnBounds(model,'EX_gln-L(e)',-1,'b');
5433     model.lb(ismember(model.rxns,'EX_o2(e)'))=-40;model.ub(ismember(model.rxns,'EX_o2(e)'))=-1;
5434     if ~isempty(strmatch('P5CRxm',model.rxns,'exact'))
5435         model=changeObjective(model,'P5CRxm',1);
5436         FBA = optimizeCbModel(model,'max');
5437         TestSolution(k,1) = FBA.f;
5438     else
5439         TestSolution(k,1) = NaN;
5440     end
5441     TestSolutionName{k,1} = 'synthesis of proline from glutamine - P5CRxm';
5442     if ~isnan(TestSolution(k,1)); TestedRxns = [TestedRxns; model.rxns(find(abs(FBA.x)>tol))]; end ;k = k +1;clear FBA
5443     
5444     %% synthesis of alanine from glutamine
5445     model=modelOri;
5446     model=changeRxnBounds(model,'EX_o2(e)',-1,'l');
5447     model=changeRxnBounds(model,'EX_gln-L(e)',-1,'b');
5448     if ~isempty(strmatch('ALATA_L',model.rxns,'exact'))
5449         model=changeObjective(model,'ALATA_L',1);
5450         FBA = optimizeCbModel(model,'max');
5451         TestSolution(k,1) = FBA.f;
5452     else
5453         TestSolution(k,1) = NaN;
5454     end
5455     TestSolutionName{k,1} = 'synthesis of alanine from glutamine - ALATA_L';
5456     if ~isnan(TestSolution(k,1)); TestedRxns = [TestedRxns; model.rxns(find(abs(FBA.x)>tol))]; end ;k = k +1;clear FBA
5457     
5458     %% basolateral secretion of proline
5459     model=modelOri;
5460     model.lb(ismember(model.rxns,'EX_glc(e)'))=-1;model.ub(ismember(model.rxns,'EX_glc(e)'))=0;
5461     model.lb(ismember(model.rxns,'EX_o2(e)'))=-40;model.ub(ismember(model.rxns,'EX_o2(e)'))=-1;
5462     if ~isempty(strmatch('EX_pro-L(e)',model.rxns,'exact'))
5463         model=changeObjective(model,'EX_pro-L(e)',1);
5464         FBA = optimizeCbModel(model,'max');
5465         TestSolution(k,1) = FBA.f;
5466     else
5467         TestSolution(k,1) = NaN;
5468     end
5469     TestSolutionName{k,1} = 'secretion of proline';
5470     if ~isnan(TestSolution(k,1)); TestedRxns = [TestedRxns; model.rxns(find(abs(FBA.x)>tol))]; end ;k = k +1;clear FBA
5471     
5472     
5473     %% basolateral secretion of arginine
5474     model=modelOri;
5475     model.lb(ismember(model.rxns,'EX_glc(e)'))=-1;model.ub(ismember(model.rxns,'EX_glc(e)'))=0;
5476     model.lb(ismember(model.rxns,'EX_o2(e)'))=-40;model.ub(ismember(model.rxns,'EX_o2(e)'))=-1;
5477     if ~isempty(strmatch('EX_arg-L(e)',model.rxns,'exact'))
5478         model=changeObjective(model,'EX_arg-L(e)',1);
5479         FBA = optimizeCbModel(model,'max');
5480         TestSolution(k,1) = FBA.f;
5481     else
5482         TestSolution(k,1) = NaN;
5483     end
5484     TestSolutionName{k,1} = 'secretion of arginine';
5485     if ~isnan(TestSolution(k,1)); TestedRxns = [TestedRxns; model.rxns(find(abs(FBA.x)>tol))]; end ;k = k +1;clear FBA
5486     
5487     %% basolateral secretion of ornithine
5488     model=modelOri;
5489     model.lb(ismember(model.rxns,'EX_glc(e)'))=-1;model.ub(ismember(model.rxns,'EX_glc(e)'))=0;
5490     model.lb(ismember(model.rxns,'EX_o2(e)'))=-40;model.ub(ismember(model.rxns,'EX_o2(e)'))=-1;
5491     if ~isempty(strmatch('EX_orn(e)',model.rxns,'exact'))
5492         model=changeObjective(model,'EX_orn(e)',1);
5493         FBA = optimizeCbModel(model,'max');
5494         TestSolution(k,1) = FBA.f;
5495     else
5496         TestSolution(k,1) = NaN;
5497     end
5498     TestSolutionName{k,1} = 'secretion of ornithine';
5499     if ~isnan(TestSolution(k,1)); TestedRxns = [TestedRxns; model.rxns(find(abs(FBA.x)>tol))]; end ;k = k +1;clear FBA
5500     
5501     
5502     %% synthesis of spermine from ornithine
5503     model=modelOri;
5504     model.lb(ismember(model.rxns,'EX_orn(e)'))=-1;model.ub(ismember(model.rxns,'EX_orn(e)'))=-1;
5505     model.lb(ismember(model.rxns,'EX_o2(e)'))=-40;model.ub(ismember(model.rxns,'EX_o2(e)'))=-1;
5506     if ~isempty(strmatch('SPRMS',model.rxns,'exact'))
5507         model=changeObjective(model,'SPRMS',1);
5508         FBA = optimizeCbModel(model,'max');
5509         TestSolution(k,1) = FBA.f;
5510     else
5511         TestSolution(k,1) = NaN;
5512     end
5513     TestSolutionName{k,1} = 'synthesis of spermine from ornithine - SPRMS';
5514     if ~isnan(TestSolution(k,1)); TestedRxns = [TestedRxns; model.rxns(find(abs(FBA.x)>tol))]; end ;k = k +1;clear FBA
5515     
5516     
5517     %% synthesis of spermidine from ornithine
5518     model=modelOri;
5519     model.lb(ismember(model.rxns,'EX_orn(e)'))=-1;model.ub(ismember(model.rxns,'EX_orn(e)'))=-1;
5520     model.lb(ismember(model.rxns,'EX_o2(e)'))=-40;model.ub(ismember(model.rxns,'EX_o2(e)'))=-1;
5521     if ~isempty(strmatch('SPMS',model.rxns,'exact'))
5522         model=changeObjective(model,'SPMS',1);
5523         %FBA=optimizeCbModel(model,'min');
5524         FBA = optimizeCbModel(model,'max');
5525         TestSolution(k,1) = FBA.f;
5526     else
5527         TestSolution(k,1) = NaN;
5528     end
5529     TestSolutionName{k,1} = 'synthesis of spermidine from ornithine - SPMS';
5530     if ~isnan(TestSolution(k,1)); TestedRxns = [TestedRxns; model.rxns(find(abs(FBA.x)>tol))]; end ;k = k +1;clear FBA
5531     
5532     %% synthesis of nitric oxide from arginine
5533     model=modelOri;
5534     model.lb(ismember(model.rxns,'EX_arg-L(e)'))=-1;model.ub(ismember(model.rxns,'EX_arg-L(e)'))=-1;
5535     model.lb(ismember(model.rxns,'EX_o2(e)'))=-40;model.ub(ismember(model.rxns,'EX_o2(e)'))=-1;
5536     if ~isempty(strmatch('NOS2',model.rxns,'exact'))
5537         model=changeObjective(model,'NOS2',1);
5538         %FBA=optimizeCbModel(model,'min');
5539         FBA = optimizeCbModel(model,'max');
5540         TestSolution(k,1) = FBA.f;
5541     else
5542         TestSolution(k,1) = NaN;
5543     end
5544     TestSolutionName{k,1} = 'synthesis of nitric oxide from arginine - NOS2';
5545     if ~isnan(TestSolution(k,1)); TestedRxns = [TestedRxns; model.rxns(find(abs(FBA.x)>tol))]; end ;k = k +1;clear FBA
5546     
5547     %%  synthesis of cholesterol
5548     model=modelOri;
5549     for i = 1 : length(RPMI_composition)
5550         model = changeRxnBounds(model,RPMI_composition{i},-1,'l');
5551     end
5552     model=changeRxnBounds(model,'EX_o2(e)',-1,'l');
5553     if ~isempty(strmatch('DSREDUCr',model.rxns,'exact'))
5554         model=changeObjective(model,'DSREDUCr',1);
5555         FBA = optimizeCbModel(model,'max');
5556         TestSolution(k,1) = FBA.f;
5557     else
5558         TestSolution(k,1) = NaN;
5559     end
5560     TestSolutionName{k,1} = 'synthesis of cholesterol - DSREDUCr (with RPMI medium)';
5561     if ~isnan(TestSolution(k,1)); TestedRxns = [TestedRxns; model.rxns(find(abs(FBA.x)>tol))]; end ;k = k +1;clear FBA
5562     
5563     %% denovo purine synthesis
5564     model=modelOri;
5565     model.lb(ismember(model.rxns,'EX_glc(e)'))=-1;model.ub(ismember(model.rxns,'EX_glc(e)'))=0;
5566     model.lb(ismember(model.rxns,'EX_o2(e)'))=-40;model.ub(ismember(model.rxns,'EX_o2(e)'))=-1;
5567     if ~isempty(strmatch('ADSL1',model.rxns,'exact'))
5568         model=changeObjective(model,'ADSL1',1);
5569         FBA = optimizeCbModel(model,'max');
5570         TestSolution(k,1) = FBA.f;
5571     else
5572         TestSolution(k,1) = NaN;
5573     end
5574     TestSolutionName{k,1} = 'de novo purine synthesis - ADSL1';
5575     if ~isnan(TestSolution(k,1)); TestedRxns = [TestedRxns; model.rxns(find(abs(FBA.x)>tol))]; end ;k = k +1;clear FBA
5576     
5577     %% de novo purine synthesis - GMPS2
5578     model=modelOri;
5579     model.lb(ismember(model.rxns,'EX_glc(e)'))=-1;model.ub(ismember(model.rxns,'EX_glc(e)'))=0;
5580     model.lb(ismember(model.rxns,'EX_o2(e)'))=-40;model.ub(ismember(model.rxns,'EX_o2(e)'))=-1;
5581     if ~isempty(strmatch('GMPS2',model.rxns,'exact'))
5582         model=changeObjective(model,'GMPS2');
5583         FBA = optimizeCbModel(model,'max');
5584         TestSolution(k,1) = FBA.f;
5585     else
5586         TestSolution(k,1) = NaN;
5587     end
5588     TestSolutionName{k,1} = 'de novo purine synthesis - GMPS2';
5589     if ~isnan(TestSolution(k,1)); TestedRxns = [TestedRxns; model.rxns(find(abs(FBA.x)>tol))]; end ;k = k +1;clear FBA
5590     
5591     %% salvage of purine bases
5592     model=modelOri;
5593     model.lb(ismember(model.rxns,'EX_glc(e)'))=-1;model.ub(ismember(model.rxns,'EX_glc(e)'))=0;
5594     model.lb(ismember(model.rxns,'EX_o2(e)'))=-40;model.ub(ismember(model.rxns,'EX_o2(e)'))=-1;
5595     if ~isempty(strmatch('ADPT',model.rxns,'exact'))
5596         model=changeObjective(model,'ADPT',1);
5597         FBA = optimizeCbModel(model,'max');
5598         TestSolution(k,1) = FBA.f;
5599     else
5600         TestSolution(k,1) = NaN;
5601     end
5602     TestSolutionName{k,1} = 'salvage of purine bases - ADPT';
5603     if ~isnan(TestSolution(k,1)); TestedRxns = [TestedRxns; model.rxns(find(abs(FBA.x)>tol))]; end ;k = k +1;clear FBA
5604     
5605     %% salvage of purine bases - GUAPRT
5606     model=modelOri;
5607     model.lb(ismember(model.rxns,'EX_glc(e)'))=-1;model.ub(ismember(model.rxns,'EX_glc(e)'))=0;
5608     model.lb(ismember(model.rxns,'EX_o2(e)'))=-40;model.ub(ismember(model.rxns,'EX_o2(e)'))=-1;
5609     if ~isempty(strmatch('GUAPRT',model.rxns,'exact'))
5610         model=changeObjective(model,'GUAPRT',1);
5611         FBA = optimizeCbModel(model,'max');
5612         TestSolution(k,1) = FBA.f;
5613     else
5614         TestSolution(k,1) = NaN;
5615     end
5616     TestSolutionName{k,1} = 'salvage of purine bases - GUAPRT';
5617     if ~isnan(TestSolution(k,1)); TestedRxns = [TestedRxns; model.rxns(find(abs(FBA.x)>tol))]; end ;k = k +1;clear FBA
5618     
5619     %% salvage of purine bases - HXPRT
5620     model=modelOri;
5621     model.lb(ismember(model.rxns,'EX_glc(e)'))=-1;model.ub(ismember(model.rxns,'EX_glc(e)'))=0;
5622     model.lb(ismember(model.rxns,'EX_o2(e)'))=-40;model.ub(ismember(model.rxns,'EX_o2(e)'))=-1;
5623     if ~isempty(strmatch('HXPRT',model.rxns,'exact'))
5624         model=changeObjective(model,'HXPRT',1);
5625         %FBA=optimizeCbModel(model,'min');
5626         FBA = optimizeCbModel(model,'max');
5627         TestSolution(k,1) = FBA.f;
5628     else
5629         TestSolution(k,1) = NaN;
5630     end
5631     TestSolutionName{k,1} = 'salvage of purine bases - HXPRT';
5632     if ~isnan(TestSolution(k,1)); TestedRxns = [TestedRxns; model.rxns(find(abs(FBA.x)>tol))]; end ;k = k +1;clear FBA
5633     
5634     %% purine catabolism
5635     model=modelOri;
5636     model.lb(ismember(model.rxns,'EX_glc(e)'))=-1;model.ub(ismember(model.rxns,'EX_glc(e)'))=0;
5637     model.lb(ismember(model.rxns,'EX_o2(e)'))=-40;model.ub(ismember(model.rxns,'EX_o2(e)'))=-1;
5638     if ~isempty(strmatch('XAOx',model.rxns,'exact'))
5639         model=changeObjective(model,'XAOx',1);
5640         FBA = optimizeCbModel(model,'max');
5641         TestSolution(k,1) = FBA.f;
5642     else
5643         TestSolution(k,1) = NaN;
5644     end
5645     TestSolutionName{k,1} = 'purine catabolism - XAOx';
5646     if ~isnan(TestSolution(k,1)); TestedRxns = [TestedRxns; model.rxns(find(abs(FBA.x)>tol))]; end ;k = k +1;clear FBA
5647     
5648     %% pyrimidine synthesis (with hco3 uptake) - TMDS
5649     model=modelOri;
5650     model.lb(ismember(model.rxns,'EX_glc(e)'))=-1;model.ub(ismember(model.rxns,'EX_glc(e)'))=0;
5651     model.lb(ismember(model.rxns,'EX_o2(e)'))=-40;model.ub(ismember(model.rxns,'EX_o2(e)'))=-1;
5652     model=changeRxnBounds(model,'EX_hco3(e)',-1,'b');
5653     if ~isempty(strmatch('TMDS',model.rxns,'exact'))
5654         model=changeObjective(model,'TMDS',1);
5655         FBA = optimizeCbModel(model,'max');
5656         TestSolution(k,1) = FBA.f;
5657     else
5658         TestSolution(k,1) = NaN;
5659     end
5660     TestSolutionName{k,1} = 'pyrimidine synthesis (with hco3 uptake) - TMDS';
5661     if ~isnan(TestSolution(k,1)); TestedRxns = [TestedRxns; model.rxns(find(abs(FBA.x)>tol))]; end ;k = k +1;clear FBA
5662     
5663     %% pyrimidine synthesis (with hco3 uptake) - CTPS2
5664     model=modelOri;
5665     model.lb(ismember(model.rxns,'EX_glc(e)'))=-1;model.ub(ismember(model.rxns,'EX_glc(e)'))=0;
5666     model.lb(ismember(model.rxns,'EX_o2(e)'))=-40;model.ub(ismember(model.rxns,'EX_o2(e)'))=-1;
5667     model=changeRxnBounds(model,'EX_hco3(e)',-1,'b');
5668     if ~isempty(strmatch('CTPS2',model.rxns,'exact'))
5669         model=changeObjective(model,'CTPS2',1);
5670         FBA = optimizeCbModel(model,'max');
5671         TestSolution(k,1) = FBA.f;
5672     else
5673         TestSolution(k,1) = NaN;
5674     end
5675     TestSolutionName{k,1} = 'pyrimidine synthesis (with hco3 uptake) - CTPS2';
5676     if ~isnan(TestSolution(k,1)); TestedRxns = [TestedRxns; model.rxns(find(abs(FBA.x)>tol))]; end ;k = k +1;clear FBA
5677     
5678     %% pyrimidine catabolism
5679     model=modelOri;
5680     model.lb(ismember(model.rxns,'EX_glc(e)'))=-1;model.ub(ismember(model.rxns,'EX_glc(e)'))=0;
5681     model.lb(ismember(model.rxns,'EX_o2(e)'))=-40;model.ub(ismember(model.rxns,'EX_o2(e)'))=-1;
5682     model=changeRxnBounds(model,'EX_hco3(e)',-1,'b');
5683     if ~isempty(strmatch('UPPN',model.rxns,'exact'))
5684         model=changeObjective(model,'UPPN',1);
5685         FBA = optimizeCbModel(model,'max');
5686         TestSolution(k,1) = FBA.f;
5687     else
5688         TestSolution(k,1) = NaN;
5689     end
5690     TestSolutionName{k,1} = 'pyrimidine catabolism - UPPN';
5691     if ~isnan(TestSolution(k,1)); TestedRxns = [TestedRxns; model.rxns(find(abs(FBA.x)>tol))]; end ;k = k +1;clear FBA
5692     
5693     %% 'pyrimidine catabolism - BUP2
5694     model=modelOri;
5695     model.lb(ismember(model.rxns,'EX_glc(e)'))=-1;model.ub(ismember(model.rxns,'EX_glc(e)'))=0;
5696     model.lb(ismember(model.rxns,'EX_o2(e)'))=-40;model.ub(ismember(model.rxns,'EX_o2(e)'))=-1;
5697     model=changeRxnBounds(model,'EX_hco3(e)',-1,'b');
5698     if ~isempty(strmatch('BUP2',model.rxns,'exact'))
5699         model=changeObjective(model,'BUP2',1);
5700         FBA=optimizeCbModel(model)
5701         TestSolution(k,1) = FBA.f;
5702     else
5703         TestSolution(k,1) = NaN;
5704     end
5705     TestSolutionName{k,1} = 'pyrimidine catabolism - BUP2';
5706     if ~isnan(TestSolution(k,1)); TestedRxns = [TestedRxns; model.rxns(find(abs(FBA.x)>tol))]; end ;k = k +1;clear FBA
5707     
5708     
5709     %% fructose to glucose conversion
5710     model=modelOri;
5711     
5712     model.lb(ismember(model.rxns,'EX_fru(e)'))=-1;model.ub(ismember(model.rxns,'EX_fru(e)'))=-1;
5713     model.lb(ismember(model.rxns,'EX_o2(e)'))=-40;model.ub(ismember(model.rxns,'EX_o2(e)'))=-1;
5714     if ~isempty(strmatch('TRIOK',model.rxns,'exact'))
5715         model=changeObjective(model,'TRIOK',1);
5716         FBA = optimizeCbModel(model,'max');
5717         TestSolution(k,1) = FBA.f;
5718     else
5719         TestSolution(k,1) = NaN;
5720     end
5721     TestSolutionName{k,1} = 'fructose to glucose conversion - TRIOK';
5722     if ~isnan(TestSolution(k,1)); TestedRxns = [TestedRxns; model.rxns(find(abs(FBA.x)>tol))]; end ;k = k +1;clear FBA
5723     
5724     
5725     %% uptake and secretion of cholic acid
5726     model=modelOri;
5727     model=changeRxnBounds(model,'EX_o2(e)',-1,'l');
5728     model=changeRxnBounds(model,'EX_cholate(e)',-1,'l');
5729     model=changeRxnBounds(model,'EX_cholate(e)',1000,'u');
5730     % model=changeObjective(model,'CHOLATEt2u');
5731     %FBA=optimizeCbModel(model,'min');
5732     %FBA=optimizeCbModel(model,'max');
5733     % TestSolution(k,1) = FBA.f;
5734     % TestSolutionName{k,1} = 'uptake and secretion of cholic acid - CHOLATEt2u'; % SHOULD THIS BE MIN?
5735     % k = k +1;clear FBA
5736     if ~isempty(strmatch('CHOLATEt3',model.rxns,'exact'))
5737         model=changeObjective(model,'CHOLATEt3',1);
5738         FBA = optimizeCbModel(model,'max');
5739         TestSolution(k,1) = FBA.f;
5740         TestSolutionName{k,1} = 'uptake of cholic acid - CHOLATEt3';
5741     else
5742         TestSolution(k,1) = NaN;
5743     end
5744     if ~isnan(TestSolution(k,1)); TestedRxns = [TestedRxns; model.rxns(find(abs(FBA.x)>tol))]; end ;k = k +1;clear FBA
5745     
5746     %     if ~isempty(strmatch('CHOLATEt3',model.rxns,'exact'))
5747     %         FBA=optimizeCbModel(model,'min');
5748     %         TestSolution(k,1) = FBA.f;
5749     %     else
5750     %         TestSolution(k,1) = NaN;
5751     %     end
5752     %     TestSolutionName{k,1} = 'secretion of cholic acid - CHOLATEt3';
5753     %  if ~isnan(TestSolution(k,1)); TestedRxns = [TestedRxns; model.rxns(find(abs(FBA.x)>tol))]; end ;k = k +1;clear FBA
5754     
5755     %% Uptake and secretion of glycocholate
5756     model=modelOri;
5757     model=changeRxnBounds(model,'EX_o2(e)',-1,'l');
5758     model=changeRxnBounds(model,'EX_gchola(e)',-1,'l');
5759     model=changeRxnBounds(model,'EX_gchola(e)',1000,'u');
5760     % model=changeObjective(model,'GCHOLAt2u');
5761     %FBA=optimizeCbModel(model,'min');
5762     %FBA=optimizeCbModel(model,'max');
5763     %TestSolution(k,1) = FBA.f;
5764     %TestSolutionName{k,1} = 'uptake and secretion of cholic glycocholate - GCHOLAt2u';
5765     %k = k +1;clear FBA
5766     if ~isempty(strmatch('GCHOLAt3',model.rxns,'exact'))
5767         model=changeObjective(model,'GCHOLAt3',1);
5768         %FBA=optimizeCbModel(model,'min');
5769         FBA = optimizeCbModel(model,'max');
5770         TestSolution(k,1) = FBA.f;
5771     else
5772         TestSolution(k,1) = NaN;
5773     end
5774     TestSolutionName{k,1} = 'uptake of cholic glycocholate - GCHOLAt3';
5775     if ~isnan(TestSolution(k,1)); TestedRxns = [TestedRxns; model.rxns(find(abs(FBA.x)>tol))]; end ;k = k +1;clear FBA
5776     
5777     %     if ~isempty(strmatch('GCHOLAt3',model.rxns,'exact'))
5778     %         FBA=optimizeCbModel(model,'min');
5779     %         TestSolution(k,1) = FBA.f;
5780     %     else
5781     %         TestSolution(k,1) = NaN;
5782     %     end
5783     %     TestSolutionName{k,1} = 'secretion of cholic glycocholate - GCHOLAt3';
5784     %  if ~isnan(TestSolution(k,1)); TestedRxns = [TestedRxns; model.rxns(find(abs(FBA.x)>tol))]; end ;k = k +1;clear FBA
5785     
5786     %% Uptake and secretion of tauro-cholate
5787     model=modelOri;
5788     model=changeRxnBounds(model,'EX_o2(e)',-1,'l');
5789     model=changeRxnBounds(model,'EX_tchola(e)',-1,'l');
5790     model=changeRxnBounds(model,'EX_tchola(e)',1000,'u');
5791     % model=changeObjective(model,'TCHOLAt2u');
5792     %FBA=optimizeCbModel(model,'min');
5793     %FBA=optimizeCbModel(model,'max');
5794     %TestSolution(k,1) = FBA.f;
5795     % TestSolutionName{k,1} = 'uptake and secretion of tauro-cholate - TCHOLAt2u';
5796     %k = k +1;clear FBA
5797     if ~isempty(strmatch('TCHOLAt3',model.rxns,'exact'))
5798         model=changeObjective(model,'TCHOLAt3',1);
5799         % FBA=optimizeCbModel(model,'min');
5800         FBA = optimizeCbModel(model,'max');
5801         TestSolution(k,1) = FBA.f;
5802     else
5803         TestSolution(k,1) = NaN;
5804     end
5805     TestSolutionName{k,1} = 'uptake of tauro-cholate - TCHOLAt3';
5806     if ~isnan(TestSolution(k,1)); TestedRxns = [TestedRxns; model.rxns(find(abs(FBA.x)>tol))]; end ;k = k +1;clear FBA
5807     %     if ~isempty(strmatch('TCHOLAt3',model.rxns,'exact'))
5808     %         FBA=optimizeCbModel(model,'min');
5809     %         TestSolution(k,1) = FBA.f;
5810     %     else
5811     %         TestSolution(k,1) = NaN;
5812     %     end
5813     %     TestSolutionName{k,1} = 'secretion of tauro-cholate - TCHOLAt3';
5814     %  if ~isnan(TestSolution(k,1)); TestedRxns = [TestedRxns; model.rxns(find(abs(FBA.x)>tol))]; end ;k = k +1;clear FBA
5815     
5816     %% Synthesis of fructose-6-phosphate from erythrose-4-phosphate (HMP shunt);
5817     model=modelOri;
5818     model.lb(ismember(model.rxns,'EX_glc(e)'))=-1;model.ub(ismember(model.rxns,'EX_glc(e)'))=0;
5819     model.lb(ismember(model.rxns,'EX_o2(e)'))=-40;model.ub(ismember(model.rxns,'EX_o2(e)'))=-1;
5820     if ~isempty(strmatch('TKT2',model.rxns,'exact'))
5821         model=changeObjective(model,'TKT2',1);
5822         FBA = optimizeCbModel(model,'max');
5823         TestSolution(k,1) = FBA.f;
5824     else
5825         TestSolution(k,1) = NaN;
5826     end
5827     TestSolutionName{k,1} = 'Synthesis of fructose-6-phosphate from erythrose-4-phosphate (HMP shunt) - TKT2';
5828     if ~isnan(TestSolution(k,1)); TestedRxns = [TestedRxns; model.rxns(find(abs(FBA.x)>tol))]; end ;k = k +1;clear FBA
5829     
5830     %% Malate to pyruvate (malic enzyme);
5831     model=modelOri;
5832     model.lb(ismember(model.rxns,'EX_glc(e)'))=-1;model.ub(ismember(model.rxns,'EX_glc(e)'))=0;
5833     model.lb(ismember(model.rxns,'EX_o2(e)'))=-40;model.ub(ismember(model.rxns,'EX_o2(e)'))=-1;
5834     if ~isempty(strmatch('ME2',model.rxns,'exact'))
5835         model=changeObjective(model,'ME2',1);
5836         FBA = optimizeCbModel(model,'max');
5837         TestSolution(k,1) = FBA.f;
5838     else
5839         TestSolution(k,1) = NaN;
5840     end
5841     TestSolutionName{k,1} = 'Malate to pyruvate (malic enzyme) - ME2';
5842     if ~isnan(TestSolution(k,1)); TestedRxns = [TestedRxns; model.rxns(find(abs(FBA.x)>tol))]; end ;k = k +1;clear FBA
5843     
5844     %% Malate to pyruvate (malic enzyme);
5845     model=modelOri;
5846     model.lb(ismember(model.rxns,'EX_glc(e)'))=-1;model.ub(ismember(model.rxns,'EX_glc(e)'))=0;
5847     model.lb(ismember(model.rxns,'EX_o2(e)'))=-40;model.ub(ismember(model.rxns,'EX_o2(e)'))=-1;
5848     if ~isempty(strmatch('ME2m',model.rxns,'exact'))
5849         model=changeObjective(model,'ME2m',1);
5850         FBA = optimizeCbModel(model,'max');
5851         TestSolution(k,1) = FBA.f;
5852     else
5853         TestSolution(k,1) = NaN;
5854     end
5855     TestSolutionName{k,1} = 'Malate to pyruvate (malic enzyme) - ME2m';
5856     if ~isnan(TestSolution(k,1)); TestedRxns = [TestedRxns; model.rxns(find(abs(FBA.x)>tol))]; end ;k = k +1;clear FBA
5857     
5858     
5859     %% Synthesis of urea (urea cycle);
5860     model=modelOri;
5861     for i = 1 : length(RPMI_composition)
5862         model = changeRxnBounds(model,RPMI_composition{i},-1,'l');
5863     end
5864     model=changeRxnBounds(model,'EX_o2(e)',-1,'l');
5865     if ~isempty(strmatch('ARGN',model.rxns,'exact'))
5866         model=changeObjective(model,'ARGN',1);
5867         FBA = optimizeCbModel(model,'max');
5868         TestSolution(k,1) = FBA.f;
5869     else
5870         TestSolution(k,1) = NaN;
5871     end
5872     TestSolutionName{k,1} = 'Synthesis of urea (urea cycle) - ARGN (with RPMI medium)';
5873     if ~isnan(TestSolution(k,1)); TestedRxns = [TestedRxns; model.rxns(find(abs(FBA.x)>tol))]; end ;k = k +1;clear FBA
5874     
5875     %% Cysteine to pyruvate
5876     model=modelOri;
5877     model=changeRxnBounds(model,'EX_o2(e)',-1,'l');
5878     model=changeRxnBounds(model,'EX_cys-L(e)',-1,'b');
5879     if ~isempty(strmatch('3SPYRSP',model.rxns,'exact'))
5880         model=changeObjective(model,'3SPYRSP',1);
5881         FBA = optimizeCbModel(model,'max');
5882         TestSolution(k,1) = FBA.f;
5883     else
5884         TestSolution(k,1) = NaN;
5885     end
5886     TestSolutionName{k,1} = 'Cysteine to pyruvate - 3SPYRSP';
5887     if ~isnan(TestSolution(k,1)); TestedRxns = [TestedRxns; model.rxns(find(abs(FBA.x)>tol))]; end ;k = k +1;clear FBA
5888     
5889     
5890     %% Methionine to cysteine  (check for dependancy over pe_hs);
5891     model=modelOri;
5892     model=changeRxnBounds(model,'EX_met_L(e)',-1,'b');
5893     model=changeRxnBounds(model,'EX_o2(e)',-1,'l');
5894     model=changeRxnBounds(model,'EX_pe_hs(e)',-1,'l');
5895     if ~isempty(strmatch('CYSTGL',model.rxns,'exact'))
5896         model=changeObjective(model,'CYSTGL',1);
5897         FBA = optimizeCbModel(model,'max');
5898         TestSolution(k,1) = FBA.f;
5899     else
5900         TestSolution(k,1) = NaN;
5901     end
5902     TestSolutionName{k,1} = 'Methionine to cysteine - CYSTGL';
5903     if ~isnan(TestSolution(k,1)); TestedRxns = [TestedRxns; model.rxns(find(abs(FBA.x)>tol))]; end ;k = k +1;clear FBA
5904     
5905     %% Synthesis of triacylglycerol (TAG reformation); (check for dependancy over dag_hs and RTOTAL3);
5906     model=modelOri;
5907     model=changeRxnBounds(model,'EX_o2(e)',-1,'l');
5908     model=changeRxnBounds(model,'EX_dag_hs(e)',-1,'l');
5909     model=changeRxnBounds(model,'EX_Rtotal3(e)',-1,'l');
5910     if ~isempty(strmatch('DGAT',model.rxns,'exact'))
5911         model=changeObjective(model,'DGAT');
5912         FBA = optimizeCbModel(model,'max');
5913         TestSolution(k,1) = FBA.f;
5914     else
5915         TestSolution(k,1) = NaN;
5916     end
5917     TestSolutionName{k,1} = 'Synthesis of triacylglycerol (TAG reformation) - DGAT';
5918     if ~isnan(TestSolution(k,1)); TestedRxns = [TestedRxns; model.rxns(find(abs(FBA.x)>tol))]; end ;k = k +1;clear FBA
5919     
5920     %% Phosphatidylcholine synthesis (check for dependancy over pe_hs);
5921     model=modelOri;
5922     model=changeRxnBounds(model,'EX_o2(e)',-1,'l');
5923     model=changeRxnBounds(model,'EX_pe_hs(e)',-1,'l');
5924     if ~isempty(strmatch('PETOHMm_hs',model.rxns,'exact'))
5925         model=changeObjective(model,'PETOHMm_hs',1);
5926         FBA = optimizeCbModel(model,'max');
5927         TestSolution(k,1) = FBA.f;
5928     else
5929         TestSolution(k,1) = NaN;
5930     end
5931     TestSolutionName{k,1} = 'Phosphatidylcholine synthesis - PETOHMm_hs';
5932     if ~isnan(TestSolution(k,1)); TestedRxns = [TestedRxns; model.rxns(find(abs(FBA.x)>tol))]; end ;k = k +1;clear FBA
5933     
5934     
5935     %% Synthesis of FMN from riboflavin
5936     model=modelOri;
5937     model.lb(ismember(model.rxns,'EX_glc(e)'))=-1;model.ub(ismember(model.rxns,'EX_glc(e)'))=0;
5938     model.lb(ismember(model.rxns,'EX_o2(e)'))=-40;model.ub(ismember(model.rxns,'EX_o2(e)'))=0;
5939     model=changeRxnBounds(model,'EX_ribflv(e)',-1,'b');
5940     if ~isempty(strmatch('RBFK',model.rxns,'exact'))
5941         model=changeObjective(model,'RBFK',1);
5942         FBA = optimizeCbModel(model,'max');
5943         TestSolution(k,1) = FBA.f;
5944     else
5945         TestSolution(k,1) = NaN;
5946     end
5947     TestSolutionName{k,1} = 'Synthesis of FMN from riboflavin - RBFK';
5948     if ~isnan(TestSolution(k,1)); TestedRxns = [TestedRxns; model.rxns(find(abs(FBA.x)>tol))]; end ;k = k +1;clear FBA
5949     
5950     %% synthesis of FAD from riboflavin
5951     model=modelOri;
5952     model.lb(ismember(model.rxns,'EX_glc(e)'))=-1;model.ub(ismember(model.rxns,'EX_glc(e)'))=0;
5953     model.lb(ismember(model.rxns,'EX_o2(e)'))=-40;model.ub(ismember(model.rxns,'EX_o2(e)'))=0;
5954     model=changeRxnBounds(model,'EX_ribflv(e)',-1,'b');
5955     if ~isempty(strmatch('FMNAT',model.rxns,'exact'))
5956         model=changeObjective(model,'FMNAT',1);
5957         FBA = optimizeCbModel(model,'max');
5958         TestSolution(k,1) = FBA.f;
5959     else
5960         TestSolution(k,1) = NaN;
5961     end
5962     TestSolutionName{k,1} = 'synthesis of FAD from riboflavin - FMNAT';
5963     if ~isnan(TestSolution(k,1)); TestedRxns = [TestedRxns; model.rxns(find(abs(FBA.x)>tol))]; end ;k = k +1;clear FBA
5964     
5965     
5966     %% Synthesis of 5-methyl-tetrahydrofolate from folic acid
5967     model=modelOri;
5968     model=changeRxnBounds(model,'EX_o2(e)',-1,'l');
5969     model=changeRxnBounds(model,'EX_fol(e)',-1,'b');
5970     if ~isempty(strmatch('MTHFR3',model.rxns,'exact'))
5971         model=changeObjective(model,'MTHFR3',1);
5972         FBA = optimizeCbModel(model,'max');
5973         TestSolution(k,1) = FBA.f;
5974     else
5975         TestSolution(k,1) = NaN;
5976     end
5977     TestSolutionName{k,1} = 'Synthesis of 5-methyl-tetrahydrofolate from folic acid - MTHFR3';
5978     if ~isnan(TestSolution(k,1)); TestedRxns = [TestedRxns; model.rxns(find(abs(FBA.x)>tol))]; end ;k = k +1;clear FBA
5979     
5980     
5981     %% Putriscine to GABA
5982     model=modelOri;
5983     model=changeRxnBounds(model,'EX_o2(e)',-1,'l');
5984     model=changeRxnBounds(model,'EX_ptrc(e)',-1,'b');
5985     if ~isempty(strmatch('ABUTD',model.rxns,'exact'))
5986         model=changeObjective(model,'ABUTD',1);
5987         FBA = optimizeCbModel(model,'max');
5988         TestSolution(k,1) = FBA.f;
5989     else
5990         TestSolution(k,1) = NaN;
5991     end
5992     TestSolutionName{k,1} = 'Putriscine to GABA - ABUTD';
5993     if ~isnan(TestSolution(k,1)); TestedRxns = [TestedRxns; model.rxns(find(abs(FBA.x)>tol))]; end ;k = k +1;clear FBA
5994     
5995     %% Superoxide dismutase
5996     model=modelOri;
5997     model.lb(ismember(model.rxns,'EX_glc(e)'))=-1;model.ub(ismember(model.rxns,'EX_glc(e)'))=0;
5998     model.lb(ismember(model.rxns,'EX_o2(e)'))=-40;model.ub(ismember(model.rxns,'EX_o2(e)'))=-1;
5999     if ~isempty(strmatch('SPODMm',model.rxns,'exact'))
6000         model=changeObjective(model,'SPODMm',1);
6001         FBA = optimizeCbModel(model,'max');
6002         TestSolution(k,1) = FBA.f;
6003     else
6004         TestSolution(k,1) = NaN;
6005     end
6006     TestSolutionName{k,1} = 'Superoxide dismutase - SPODMm';
6007     if ~isnan(TestSolution(k,1)); TestedRxns = [TestedRxns; model.rxns(find(abs(FBA.x)>tol))]; end ;k = k +1;clear FBA
6008     
6009     %% Availability of bicarbonate from Carbonic anhydrase reaction
6010     model=modelOri;
6011     model.lb(ismember(model.rxns,'EX_glc(e)'))=-1;model.ub(ismember(model.rxns,'EX_glc(e)'))=0;
6012     model.lb(ismember(model.rxns,'EX_o2(e)'))=-40;model.ub(ismember(model.rxns,'EX_o2(e)'))=-1;
6013     if ~isempty(strmatch('H2CO3Dm',model.rxns,'exact'))
6014         model=changeObjective(model,'H2CO3Dm',1);
6015         FBA = optimizeCbModel(model,'max');
6016         TestSolution(k,1) = FBA.f;
6017     else
6018         TestSolution(k,1) = NaN;
6019     end
6020     TestSolutionName{k,1} = 'Availability of bicarbonate from Carbonic anhydrase reaction - H2CO3Dm';
6021     if ~isnan(TestSolution(k,1)); TestedRxns = [TestedRxns; model.rxns(find(abs(FBA.x)>tol))]; end ;k = k +1;clear FBA
6022     
6023     %% Regeneration of citrate (TCA cycle);
6024     model=modelOri;
6025     model.lb(ismember(model.rxns,'EX_glc(e)'))=-1;model.ub(ismember(model.rxns,'EX_glc(e)'))=0;
6026     model.lb(ismember(model.rxns,'EX_o2(e)'))=-40;model.ub(ismember(model.rxns,'EX_o2(e)'))=-1;
6027     if ~isempty(strmatch('CSm',model.rxns,'exact'))
6028         model=changeObjective(model,'CSm',1);
6029         FBA = optimizeCbModel(model,'max');
6030         TestSolution(k,1) = FBA.f;
6031     else
6032         TestSolution(k,1) = NaN;
6033     end
6034     TestSolutionName{k,1} = 'Regeneration of citrate (TCA cycle) - CSm';
6035     if ~isnan(TestSolution(k,1)); TestedRxns = [TestedRxns; model.rxns(find(abs(FBA.x)>tol))]; end ;k = k +1;clear FBA
6036     
6037     
6038     %% Histidine to FIGLU
6039     model=modelOri;
6040     model.lb(find(ismember(model.rxns,'EX_his-L(e)')))=-1;
6041     model.ub(find(ismember(model.rxns,'EX_his-L(e)')))=-1;
6042     model=changeRxnBounds(model,'EX_o2(e)',-40,'l');
6043     model=changeRxnBounds(model,'EX_o2(e)',-1,'u');
6044     if ~isempty(strmatch('IZPN',model.rxns,'exact'))
6045         model=changeObjective(model,'IZPN',1);
6046         FBA = optimizeCbModel(model,'max');
6047         TestSolution(k,1) = FBA.f;
6048     else
6049         TestSolution(k,1) = NaN;
6050     end
6051     TestSolutionName{k,1} = 'Histidine to FIGLU - IZPN';
6052     if ~isnan(TestSolution(k,1)); TestedRxns = [TestedRxns; model.rxns(find(abs(FBA.x)>tol))]; end ;k = k +1;clear FBA
6053     
6054     
6055     %% binding of guar gum fiber to bile acids
6056     model=modelOri;
6057     model=changeRxnBounds(model,'EX_gum(e)',-1,'l');
6058     model=changeRxnBounds(model,'EX_gchola(e)',-1,'l');
6059     if ~isempty(strmatch('EX_gumgchol(e)',model.rxns,'exact'))
6060         model=changeObjective(model,'EX_gumgchol(e)',1);
6061         FBA = optimizeCbModel(model,'max');
6062         TestSolution(k,1) = FBA.f;
6063     else
6064         TestSolution(k,1) = NaN;
6065     end
6066     TestSolutionName{k,1} = 'binding of guar gum fiber to bile acids - EX_gumgchol(e)';
6067     if ~isnan(TestSolution(k,1)); TestedRxns = [TestedRxns; model.rxns(find(abs(FBA.x)>tol))]; end ;k = k +1;clear FBA
6068     
6069     model=modelOri;
6070     model=changeRxnBounds(model,'EX_tchola(e)',-1,'l');
6071     model=changeRxnBounds(model,'EX_gum(e)',-1,'l');
6072     
6073     if ~isempty(strmatch('GUMTCHOLe',model.rxns,'exact'))
6074         model=changeObjective(model,'GUMTCHOLe',1);
6075         FBA = optimizeCbModel(model,'max');
6076         TestSolution(k,1) = FBA.f;
6077     else
6078         TestSolution(k,1) = NaN;
6079     end
6080     TestSolutionName{k,1} = 'binding of guar gum fiber to bile acids - GUMTCHOLe';
6081     if ~isnan(TestSolution(k,1)); TestedRxns = [TestedRxns; model.rxns(find(abs(FBA.x)>tol))]; end ;k = k +1;clear FBA
6082     
6083     model=modelOri;
6084     if ~isempty(strmatch('GUMDCHAe',model.rxns,'exact'))
6085         model=changeRxnBounds(model,'EX_dchac(e)',-1,'l');
6086         model=changeRxnBounds(model,'EX_gum(e)',-1,'l');
6087         model=changeObjective(model,'GUMDCHAe',1);
6088         FBA = optimizeCbModel(model,'max');
6089         TestSolution(k,1) = FBA.f;
6090     else
6091         TestSolution(k,1) = NaN;
6092     end
6093     TestSolutionName{k,1} = 'binding of guar gum fiber to bile acids - GUMDCHAe';
6094     if ~isnan(TestSolution(k,1)); TestedRxns = [TestedRxns; model.rxns(find(abs(FBA.x)>tol))]; end ;k = k +1;clear FBA
6095     
6096     %% binding of psyllium fiber to bile acids
6097     model=modelOri;
6098     model=changeRxnBounds(model,'EX_psyl(e)',-1,'l');
6099     model=changeRxnBounds(model,'EX_gchola(e)',-1,'l');
6100     if ~isempty(strmatch('PSYGCHe',model.rxns,'exact'))
6101         model=changeObjective(model,'PSYGCHe',1);
6102         FBA = optimizeCbModel(model,'max');
6103         TestSolution(k,1) = FBA.f;
6104     else
6105         TestSolution(k,1) = NaN;
6106     end
6107     TestSolutionName{k,1} = 'binding of psyllium fiber to bile acids - PSYGCHe';
6108     if ~isnan(TestSolution(k,1)); TestedRxns = [TestedRxns; model.rxns(find(abs(FBA.x)>tol))]; end ;k = k +1;clear FBA
6109     
6110     
6111     model=modelOri;
6112     model=changeRxnBounds(model,'EX_psyl(e)',-1,'l');
6113     model=changeRxnBounds(model,'EX_tchola(e)',-1,'l');
6114     if ~isempty(strmatch('PSYTCHe',model.rxns,'exact'))
6115         model=changeObjective(model,'PSYTCHe',1);
6116         FBA = optimizeCbModel(model,'max');
6117         TestSolution(k,1) = FBA.f;
6118     else
6119         TestSolution(k,1) = NaN;
6120     end
6121     TestSolutionName{k,1} = 'binding of psyllium fiber to bile acids - PSYTCHe';
6122     if ~isnan(TestSolution(k,1)); TestedRxns = [TestedRxns; model.rxns(find(abs(FBA.x)>tol))]; end ;k = k +1;clear FBA
6123     
6124     model=modelOri;
6125     if ~isempty(strmatch('PSYTDECHe',model.rxns,'exact'))
6126         model=changeRxnBounds(model,'EX_tdechola(e)',-1,'l');
6127         model=changeRxnBounds(model,'EX_psyl(e)',-1,'l');
6128         model=changeObjective(model,'PSYTDECHe',1);
6129         FBA = optimizeCbModel(model,'max');
6130         TestSolution(k,1) = FBA.f;
6131     else
6132         TestSolution(k,1) = NaN;
6133     end
6134     TestSolutionName{k,1} = 'binding of psyllium fiber to bile acids - PSYTDECHe';
6135     if ~isnan(TestSolution(k,1)); TestedRxns = [TestedRxns; model.rxns(find(abs(FBA.x)>tol))]; end ;k = k +1;clear FBA
6136     
6137     %% binding to beta glucan fibers to bile acids
6138     model=modelOri;
6139     model=changeRxnBounds(model,'EX_bglc(e)',-1,'l');
6140     model=changeRxnBounds(model,'EX_gchola(e)',-1,'l');
6141     model.lb(ismember(model.rxns,'EX_o2(e)'))=-40;model.ub(ismember(model.rxns,'EX_o2(e)'))=-1;
6142     if ~isempty(strmatch('BGLUGCHe',model.rxns,'exact'))
6143         model=changeObjective(model,'BGLUGCHe',1);
6144         %FBA=optimizeCbModel(model,'min');
6145         FBA = optimizeCbModel(model,'max');
6146         TestSolution(k,1) = FBA.f;
6147     else
6148         TestSolution(k,1) = NaN;
6149     end
6150     TestSolutionName{k,1} = 'binding to beta glucan fibers to bile acids - BGLUGCHe';
6151     if ~isnan(TestSolution(k,1)); TestedRxns = [TestedRxns; model.rxns(find(abs(FBA.x)>tol))]; end ;k = k +1;clear FBA
6152     
6153     model=modelOri;
6154     model=changeRxnBounds(model,'EX_bglc(e)',-1,'l');
6155     model=changeRxnBounds(model,'EX_tchola(e)',-1,'l');
6156     model.lb(ismember(model.rxns,'EX_o2(e)'))=-40;model.ub(ismember(model.rxns,'EX_o2(e)'))=-1;
6157     if ~isempty(strmatch('BGLUTCHLe',model.rxns,'exact'))
6158         model=changeObjective(model,'BGLUTCHLe',1);
6159         FBA = optimizeCbModel(model,'max');
6160         TestSolution(k,1) = FBA.f;
6161     else
6162         TestSolution(k,1) = NaN;
6163     end
6164     TestSolutionName{k,1} = 'binding to beta glucan fibers to bile acids - BGLUTCHLe';
6165     if ~isnan(TestSolution(k,1)); TestedRxns = [TestedRxns; model.rxns(find(abs(FBA.x)>tol))]; end ;k = k +1;clear FBA
6166     
6167     model=modelOri;
6168     model=changeRxnBounds(model,'EX_bglc(e)',-1,'l');
6169     model=changeRxnBounds(model,'EX_tdechola(e)',-1,'l');
6170     model.lb(ismember(model.rxns,'EX_o2(e)'))=-40;model.ub(ismember(model.rxns,'EX_o2(e)'))=-1;
6171     if ~isempty(strmatch('BGLUTDECHOe',model.rxns,'exact'))
6172         model=changeObjective(model,'BGLUTDECHOe',1);
6173         FBA = optimizeCbModel(model,'max');
6174         TestSolution(k,1) = FBA.f;
6175     else
6176         TestSolution(k,1) = NaN;
6177     end
6178     TestSolutionName{k,1} = 'binding to beta glucan fibers to bile acids - BGLUTDECHOe';
6179     if ~isnan(TestSolution(k,1)); TestedRxns = [TestedRxns; model.rxns(find(abs(FBA.x)>tol))]; end ;k = k +1;clear FBA
6180     
6181     %% binding of pectin fiber to bile acids
6182     model=modelOri;
6183     model=changeRxnBounds(model,'EX_pect(e)',-1,'l');
6184     model=changeRxnBounds(model,'EX_gchola(e)',-1,'l');
6185     model.lb(ismember(model.rxns,'EX_o2(e)'))=-40;model.ub(ismember(model.rxns,'EX_o2(e)'))=-1;
6186     if ~isempty(strmatch('PECGCHLe',model.rxns,'exact'))
6187         model=changeObjective(model,'PECGCHLe',1);
6188         FBA = optimizeCbModel(model,'max');
6189         TestSolution(k,1) = FBA.f;
6190     else
6191         TestSolution(k,1) = NaN;
6192     end
6193     TestSolutionName{k,1} = 'binding of pectin fiber to bile acids - PECGCHLe';
6194     if ~isnan(TestSolution(k,1)); TestedRxns = [TestedRxns; model.rxns(find(abs(FBA.x)>tol))]; end ;k = k +1;clear FBA
6195     
6196     model=modelOri;
6197     model.lb(ismember(model.rxns,'EX_o2(e)'))=-40;model.ub(ismember(model.rxns,'EX_o2(e)'))=-1;
6198     model=changeRxnBounds(model,'EX_pect(e)',-1,'l');
6199     model=changeRxnBounds(model,'EX_tchola(e)',-1,'l');
6200     if ~isempty(strmatch('PECTCHLe',model.rxns,'exact'))
6201         model=changeObjective(model,'PECTCHLe',1);
6202         FBA = optimizeCbModel(model,'max');
6203         TestSolution(k,1) = FBA.f;
6204     else
6205         TestSolution(k,1) = NaN;
6206     end
6207     TestSolutionName{k,1} = 'binding of pectin fiber to bile acids - PECTCHLe';
6208     if ~isnan(TestSolution(k,1)); TestedRxns = [TestedRxns; model.rxns(find(abs(FBA.x)>tol))]; end ;k = k +1;clear FBA
6209     
6210     model=modelOri;
6211     if ~isempty(strmatch('PECDCHe',model.rxns,'exact'))
6212         model=changeRxnBounds(model,'EX_dchac(e)',-1,'l');
6213         model=changeRxnBounds(model,'EX_pect(e)',-1,'l');
6214         model.lb(ismember(model.rxns,'EX_o2(e)'))=-40;model.ub(ismember(model.rxns,'EX_o2(e)'))=-1;
6215         model=changeObjective(model,'PECDCHe',1);
6216         FBA = optimizeCbModel(model,'max');
6217         TestSolution(k,1) = FBA.f;
6218     else
6219         TestSolution(k,1) = NaN;
6220     end
6221     TestSolutionName{k,1} = 'binding of pectin fiber to bile acids - PECDCHe';
6222     if ~isnan(TestSolution(k,1)); TestedRxns = [TestedRxns; model.rxns(find(abs(FBA.x)>tol))]; end ;k = k +1;clear FBA
6223     
6224     %% heme synthesis
6225     model=modelOri;
6226     model.lb(ismember(model.rxns,'EX_glc(e)'))=-1;model.ub(ismember(model.rxns,'EX_glc(e)'))=0;
6227     model.lb(ismember(model.rxns,'EX_o2(e)'))=-40;model.ub(ismember(model.rxns,'EX_o2(e)'))=-1;
6228     if ~isempty(strmatch('FCLTm',model.rxns,'exact'))
6229         model=changeObjective(model,'FCLTm',1);
6230         FBA = optimizeCbModel(model,'max');
6231         TestSolution(k,1) = FBA.f;
6232     else
6233         TestSolution(k,1) = NaN;
6234     end
6235     TestSolutionName{k,1} = 'heme synthesis - FCLTm';
6236     if ~isnan(TestSolution(k,1)); TestedRxns = [TestedRxns; model.rxns(find(abs(FBA.x)>tol))]; end ;k = k +1;clear FBA
6237     
6238     %% heme degradation
6239     model=modelOri;
6240     model.lb(ismember(model.rxns,'EX_pheme(e)'))=-1;model.ub(ismember(model.rxns,'EX_pheme(e)'))=-1;
6241     model.lb(ismember(model.rxns,'EX_glc(e)'))=-1;model.ub(ismember(model.rxns,'EX_glc(e)'))=0;
6242     model.lb(ismember(model.rxns,'EX_o2(e)'))=-40;model.ub(ismember(model.rxns,'EX_o2(e)'))=0;
6243     if ~isempty(strmatch('HOXG',model.rxns,'exact'))
6244         model=changeObjective(model,'HOXG',1);
6245         FBA = optimizeCbModel(model,'max');
6246         TestSolution(k,1) = FBA.f;
6247     else
6248         TestSolution(k,1) = NaN;
6249     end
6250     TestSolutionName{k,1} = 'heme degradation - HOXG';
6251     if ~isnan(TestSolution(k,1)); TestedRxns = [TestedRxns; model.rxns(find(abs(FBA.x)>tol))]; end ;k = k +1;clear FBA
6252     
6253 end
6254 
6255 %% these functions are new based on muscle and kidney work of SS
6256 
6257 if strcmp(test,'all')|| strcmp(test,'Harvey')
6258     
6259     %% Muscle objectives: valine -> pyruvate
6260     model = modelOri;
6261     model.c(find(model.c)) = 0;
6262     model.lb(ismember(model.rxns,'EX_val_L(e)'))=-1;model.ub(ismember(model.rxns,'EX_val_L(e)'))=-1;
6263     model.lb(ismember(model.rxns,'EX_o2(e)'))=-40;model.ub(ismember(model.rxns,'EX_o2(e)'))=-1;
6264     [model] = addSinkReactions(model,{'pyr(m)'},[0 100]);
6265     model.c(ismember(model.rxns,'sink_pyr(m)'))=1;
6266     if find(model.c)>0
6267         FBA = optimizeCbModel(model,'max');
6268         TestSolution(k,1) = FBA.f;
6269     else
6270         TestSolution(k,1) = NaN;
6271     end
6272     TestSolutionName{k,1} = 'valine -> pyruvate';
6273     if ~isnan(TestSolution(k,1)); TestedRxns = [TestedRxns; model.rxns(find(abs(FBA.x)>tol))]; end ;k = k +1;clear FBA
6274     %% leucine -> pyruvate
6275     model = modelOri;
6276     model.c(find(model.c)) = 0;
6277     model.lb(ismember(model.rxns,'EX_leu_L(e)'))=-1;model.ub(ismember(model.rxns,'EX_leu_L(e)'))=-1;
6278     model.lb(ismember(model.rxns,'EX_o2(e)'))=-40;model.ub(ismember(model.rxns,'EX_o2(e)'))=-1;
6279     [model] = addSinkReactions(model,{'pyr(c)'},[0 100]);
6280     model.c(ismember(model.rxns,'sink_pyr(c)'))=1;
6281     if find(model.c)>0
6282         FBA = optimizeCbModel(model,'max');
6283         TestSolution(k,1) = FBA.f;
6284     else
6285         TestSolution(k,1) = NaN;
6286     end
6287     TestSolutionName{k,1} = 'leucine -> pyruvate';
6288     if ~isnan(TestSolution(k,1)); TestedRxns = [TestedRxns; model.rxns(find(abs(FBA.x)>tol))]; end ;k = k +1;clear FBA
6289     %% isoleucine -> pyruvate
6290     model = modelOri;
6291     model.c(find(model.c)) = 0;
6292     model.lb(ismember(model.rxns,'EX_glc(e)'))=0;model.ub(ismember(model.rxns,'EX_glc(e)'))=0;
6293     model.lb(ismember(model.rxns,'EX_ile_L(e)'))=-1;model.ub(ismember(model.rxns,'EX_ile_L(e)'))=-1;
6294     model.lb(ismember(model.rxns,'EX_o2(e)'))=-40;model.ub(ismember(model.rxns,'EX_o2(e)'))=-1;
6295     [model] = addSinkReactions(model,{'pyr(c)'},[0 100]);
6296     model.c(ismember(model.rxns,'sink_pyr(c)'))=1;
6297     if find(model.c)>0
6298         FBA = optimizeCbModel(model,'max');
6299         TestSolution(k,1) = FBA.f;
6300     else
6301         TestSolution(k,1) = NaN;
6302     end
6303     TestSolutionName{k,1} = 'isoleucine -> pyruvate';
6304     if ~isnan(TestSolution(k,1)); TestedRxns = [TestedRxns; model.rxns(find(abs(FBA.x)>tol))]; end ;k = k +1;clear FBA
6305     %% threonine -> alanine
6306     model = modelOri;
6307     model.c(find(model.c)) = 0;
6308     model.lb(ismember(model.rxns,'EX_glc(e)'))=0;model.ub(ismember(model.rxns,'EX_glc(e)'))=0;
6309     model.lb(ismember(model.rxns,'EX_thr_L(e)'))=-1;model.ub(ismember(model.rxns,'EX_thr_L(e)'))=-1;
6310     model.lb(ismember(model.rxns,'EX_o2(e)'))=-40;model.ub(ismember(model.rxns,'EX_o2(e)'))=0;
6311     [model] = addSinkReactions(model,{'ala-L(c)'},[0 100]);
6312     model.c(ismember(model.rxns,'sink_ala-L(c)'))=1;
6313     model.c(ismember(model.rxns,'sink_ala_L(c)'))=1;
6314     if find(model.c)>0
6315         FBA = optimizeCbModel(model,'max');
6316         TestSolution(k,1) = FBA.f;
6317     else
6318         TestSolution(k,1) = NaN;
6319     end
6320     TestSolutionName{k,1} = 'threonine -> alanine';
6321     if ~isnan(TestSolution(k,1)); TestedRxns = [TestedRxns; model.rxns(find(abs(FBA.x)>tol))]; end ;k = k +1;clear FBA
6322     %% aspartate -> pyruvate
6323     model = modelOri;
6324     model.c(find(model.c)) = 0;
6325     model.lb(ismember(model.rxns,'EX_glc(e)'))=0;model.ub(ismember(model.rxns,'EX_glc(e)'))=0;
6326     model.lb(ismember(model.rxns,'EX_asp_L(e)'))=-1;model.ub(ismember(model.rxns,'EX_asp_L(e)'))=-1;
6327     model.lb(ismember(model.rxns,'EX_o2(e)'))=-40;model.ub(ismember(model.rxns,'EX_o2(e)'))=-1;
6328     [model] = addSinkReactions(model,{'pyr(c)'},[0 100]);
6329     model.c(ismember(model.rxns,'sink_pyr(c)'))=1;
6330     if find(model.c)>0
6331         FBA = optimizeCbModel(model,'max');
6332         TestSolution(k,1) = FBA.f;
6333     else
6334         TestSolution(k,1) = NaN;
6335     end
6336     TestSolutionName{k,1} = 'aspartate -> pyruvate';
6337     if ~isnan(TestSolution(k,1)); TestedRxns = [TestedRxns; model.rxns(find(abs(FBA.x)>tol))]; end ;k = k +1;clear FBA
6338     %% serine -> alanine
6339     model = modelOri;
6340     model.c(find(model.c)) = 0;
6341     model.lb(ismember(model.rxns,'EX_glc(e)'))=0;model.ub(ismember(model.rxns,'EX_glc(e)'))=0;
6342     model.lb(ismember(model.rxns,'EX_ser_L(e)'))=-1;model.ub(ismember(model.rxns,'EX_ser_L(e)'))=-1;
6343     model.lb(ismember(model.rxns,'EX_o2(e)'))=-40;model.ub(ismember(model.rxns,'EX_o2(e)'))=-1;
6344     [model] = addSinkReactions(model,{'ala-L(c)'},[0 100]);
6345     model.c(ismember(model.rxns,'sink_ala-L(c)'))=1;
6346     model.c(ismember(model.rxns,'sink_ala_L(c)'))=1;
6347     if find(model.c)>0
6348         FBA = optimizeCbModel(model,'max');
6349         TestSolution(k,1) = FBA.f;
6350     else
6351         TestSolution(k,1) = NaN;
6352     end
6353     TestSolutionName{k,1} = 'serine -> alanine';
6354     if ~isnan(TestSolution(k,1)); TestedRxns = [TestedRxns; model.rxns(find(abs(FBA.x)>tol))]; end ;k = k +1;clear FBA
6355     %% glycine -> alanine
6356     model = modelOri;
6357     model.c(find(model.c)) = 0;
6358     model.lb(ismember(model.rxns,'EX_glc(e)'))=0;model.ub(ismember(model.rxns,'EX_glc(e)'))=0;
6359     model.lb(ismember(model.rxns,'EX_gly(e)'))=-1;model.ub(ismember(model.rxns,'EX_gly(e)'))=-1;
6360     model.lb(ismember(model.rxns,'EX_o2(e)'))=-40;model.ub(ismember(model.rxns,'EX_o2(e)'))=-1;
6361     [model] = addSinkReactions(model,{'ala-L(c)'},[0 100]);
6362     model.c(ismember(model.rxns,'sink_ala-L(c)'))=1;
6363     model.c(ismember(model.rxns,'sink_ala_L(c)'))=1;
6364     if find(model.c)>0
6365         FBA = optimizeCbModel(model,'max');
6366         TestSolution(k,1) = FBA.f;
6367     else
6368         TestSolution(k,1) = NaN;
6369     end
6370     TestSolutionName{k,1} = 'glycine -> alanine';
6371     if ~isnan(TestSolution(k,1)); TestedRxns = [TestedRxns; model.rxns(find(abs(FBA.x)>tol))]; end ;k = k +1;clear FBA
6372     %% aspartate -> alanine
6373     model = modelOri;
6374     model.c(find(model.c)) = 0;
6375     model.lb(ismember(model.rxns,'EX_glc(e)'))=0;model.ub(ismember(model.rxns,'EX_glc(e)'))=0;
6376     model.lb(ismember(model.rxns,'EX_asp_L(e)'))=-1;model.ub(ismember(model.rxns,'EX_asp_L(e)'))=-1;
6377     model.lb(ismember(model.rxns,'EX_o2(e)'))=-40;model.ub(ismember(model.rxns,'EX_o2(e)'))=-1;
6378     [model] = addSinkReactions(model,{'ala-L(c)'},[0 100]);
6379     model.c(ismember(model.rxns,'sink_ala-L(c)'))=1;
6380     model.c(ismember(model.rxns,'sink_ala_L(c)'))=1;
6381     if find(model.c)>0
6382         FBA = optimizeCbModel(model,'max');
6383         TestSolution(k,1) = FBA.f;
6384     else
6385         TestSolution(k,1) = NaN;
6386     end
6387     TestSolutionName{k,1} = 'aspartate -> alanine';
6388     if ~isnan(TestSolution(k,1)); TestedRxns = [TestedRxns; model.rxns(find(abs(FBA.x)>tol))]; end ;k = k +1;clear FBA
6389     %% tyrosine -> glutamine
6390     model = modelOri;
6391     model.c(find(model.c)) = 0;
6392     model.lb(ismember(model.rxns,'EX_glc(e)'))=0;model.ub(ismember(model.rxns,'EX_glc(e)'))=0;
6393     model.lb(ismember(model.rxns,'EX_tyr_L(e)'))=-1;model.ub(ismember(model.rxns,'EX_tyr_L(e)'))=-1;
6394     model.lb(ismember(model.rxns,'EX_o2(e)'))=-40;model.ub(ismember(model.rxns,'EX_o2(e)'))=-1;
6395     [model] = addSinkReactions(model,{'gln-L(c)'},[0 100]);
6396     model.c(ismember(model.rxns,'sink_gln-L(c)'))=1;
6397     model.c(ismember(model.rxns,'sink_gln_L(c)'))=1;
6398     if find(model.c)>0
6399         FBA = optimizeCbModel(model,'max');
6400         TestSolution(k,1) = FBA.f;
6401     else
6402         TestSolution(k,1) = NaN;
6403     end
6404     TestSolutionName{k,1} = 'tyrosine -> glutamine';
6405     if ~isnan(TestSolution(k,1)); TestedRxns = [TestedRxns; model.rxns(find(abs(FBA.x)>tol))]; end ;k = k +1;clear FBA
6406     %% lysine -> glutamine
6407     model = modelOri;
6408     model.c(find(model.c)) = 0;
6409     model.lb(ismember(model.rxns,'EX_glc(e)'))=0;model.ub(ismember(model.rxns,'EX_glc(e)'))=0;
6410     model.lb(ismember(model.rxns,'EX_lys-L(e)'))=-1;model.ub(ismember(model.rxns,'EX_lys-L(e)'))=-1;
6411     model.lb(ismember(model.rxns,'EX_o2(e)'))=-40;model.ub(ismember(model.rxns,'EX_o2(e)'))=-1;
6412     [model] = addSinkReactions(model,{'gln-L(c)'},[0 100]);
6413     model.c(ismember(model.rxns,'sink_gln-L(c)'))=1;
6414     model.c(ismember(model.rxns,'sink_gln_L(c)'))=1;
6415     if find(model.c)>0
6416         FBA = optimizeCbModel(model,'max');
6417         TestSolution(k,1) = FBA.f;
6418     else
6419         TestSolution(k,1) = NaN;
6420     end
6421     TestSolutionName{k,1} = 'lysine -> glutamine';
6422     if ~isnan(TestSolution(k,1)); TestedRxns = [TestedRxns; model.rxns(find(abs(FBA.x)>tol))]; end ;k = k +1;clear FBA
6423     %% phenylalanine -> glutamine
6424     model = modelOri;
6425     model.c(find(model.c)) = 0;
6426     model.lb(ismember(model.rxns,'EX_glc(e)'))=0;model.ub(ismember(model.rxns,'EX_glc(e)'))=0;
6427     model.lb(ismember(model.rxns,'EX_phe_L(e)'))=-1;model.ub(ismember(model.rxns,'EX_phe_L(e)'))=-1;
6428     model.lb(ismember(model.rxns,'EX_o2(e)'))=-40;model.ub(ismember(model.rxns,'EX_o2(e)'))=-1;
6429     [model] = addSinkReactions(model,{'gln-L(c)'},[0 100]);
6430     model.c(ismember(model.rxns,'sink_gln-L(c)'))=1;
6431     model.c(ismember(model.rxns,'sink_gln_L(c)'))=1;
6432     if find(model.c)>0
6433         FBA = optimizeCbModel(model,'max');
6434         TestSolution(k,1) = FBA.f;
6435     else
6436         TestSolution(k,1) = NaN;
6437     end
6438     TestSolutionName{k,1} = 'phenylalanine -> glutamine';
6439     if ~isnan(TestSolution(k,1)); TestedRxns = [TestedRxns; model.rxns(find(abs(FBA.x)>tol))]; end ;k = k +1;clear FBA
6440     %% cysteine -> glutamine
6441     model = modelOri;
6442     model.c(find(model.c)) = 0;
6443     model.lb(ismember(model.rxns,'EX_glc(e)'))=0;model.ub(ismember(model.rxns,'EX_glc(e)'))=0;
6444     model.lb(ismember(model.rxns,'EX_cys-L(e)'))=-1;model.ub(ismember(model.rxns,'EX_cys-L(e)'))=-1;
6445     model.lb(ismember(model.rxns,'EX_o2(e)'))=-40;model.ub(ismember(model.rxns,'EX_o2(e)'))=-1;
6446     [model] = addSinkReactions(model,{'gln-L(c)'},[0 100]);
6447     model.c(ismember(model.rxns,'sink_gln-L(c)'))=1;
6448     model.c(ismember(model.rxns,'sink_gln_L(c)'))=1;
6449     if find(model.c)>0
6450         FBA = optimizeCbModel(model,'max');
6451         TestSolution(k,1) = FBA.f;
6452     else
6453         TestSolution(k,1) = NaN;
6454     end
6455     TestSolutionName{k,1} = 'cysteine -> glutamine';
6456     if ~isnan(TestSolution(k,1)); TestedRxns = [TestedRxns; model.rxns(find(abs(FBA.x)>tol))]; end ;k = k +1;clear FBA
6457     %% cysteine -> alanine
6458     model = modelOri;
6459     model.c(find(model.c)) = 0;
6460     model.lb(ismember(model.rxns,'EX_glc(e)'))=0;model.ub(ismember(model.rxns,'EX_glc(e)'))=0;
6461     model.lb(ismember(model.rxns,'EX_cys-L(e)'))=-1;model.ub(ismember(model.rxns,'EX_cys-L(e)'))=-1;
6462     model.lb(ismember(model.rxns,'EX_o2(e)'))=-40;model.ub(ismember(model.rxns,'EX_o2(e)'))=-1;
6463     [model] = addSinkReactions(model,{'ala-L(c)'},[0 100]);
6464     model.c(ismember(model.rxns,'sink_ala-L(c)'))=1;
6465     model.c(ismember(model.rxns,'sink_ala_L(c)'))=1;
6466     if find(model.c)>0
6467         FBA = optimizeCbModel(model,'max');
6468         TestSolution(k,1) = FBA.f;
6469     else
6470         TestSolution(k,1) = NaN;
6471     end
6472     TestSolutionName{k,1} = 'cysteine -> alanine';
6473     if ~isnan(TestSolution(k,1)); TestedRxns = [TestedRxns; model.rxns(find(abs(FBA.x)>tol))]; end ;k = k +1;clear FBA
6474     %% leucine -> glutamine
6475     model = modelOri;
6476     model.c(find(model.c)) = 0;
6477     model.lb(ismember(model.rxns,'EX_glc(e)'))=0;model.ub(ismember(model.rxns,'EX_glc(e)'))=0;
6478     model.lb(ismember(model.rxns,'EX_leu_L(e)'))=-1;model.ub(ismember(model.rxns,'EX_leu_L(e)'))=-1;
6479     model.lb(ismember(model.rxns,'EX_o2(e)'))=-40;model.ub(ismember(model.rxns,'EX_o2(e)'))=-1;
6480     [model] = addSinkReactions(model,{'gln-L(c)'},[0 100]);
6481     model.c(ismember(model.rxns,'sink_gln-L(c)'))=1;
6482     model.c(ismember(model.rxns,'sink_gln_L(c)'))=1;
6483     if find(model.c)>0
6484         FBA = optimizeCbModel(model,'max');
6485         TestSolution(k,1) = FBA.f;
6486     else
6487         TestSolution(k,1) = NaN;
6488     end
6489     TestSolutionName{k,1} = 'leucine -> glutamine';
6490     if ~isnan(TestSolution(k,1)); TestedRxns = [TestedRxns; model.rxns(find(abs(FBA.x)>tol))]; end ;k = k +1;clear FBA
6491     %% leucine -> alanine
6492     model = modelOri;
6493     model.c(find(model.c)) = 0;
6494     model.lb(ismember(model.rxns,'EX_glc(e)'))=0;model.ub(ismember(model.rxns,'EX_glc(e)'))=0;
6495     model.lb(ismember(model.rxns,'EX_leu_L(e)'))=-1;model.ub(ismember(model.rxns,'EX_leu_L(e)'))=-1;
6496     model.lb(ismember(model.rxns,'EX_o2(e)'))=-40;model.ub(ismember(model.rxns,'EX_o2(e)'))=-1;
6497     [model] = addSinkReactions(model,{'ala-L(c)'},[0 100]);
6498     model.c(ismember(model.rxns,'sink_ala-L(c)'))=1;
6499     model.c(ismember(model.rxns,'sink_ala_L(c)'))=1;
6500     if find(model.c)>0
6501         FBA = optimizeCbModel(model,'max');
6502         TestSolution(k,1) = FBA.f;
6503     else
6504         TestSolution(k,1) = NaN;
6505     end
6506     TestSolutionName{k,1} = 'leucine -> alanine';
6507     if ~isnan(TestSolution(k,1)); TestedRxns = [TestedRxns; model.rxns(find(abs(FBA.x)>tol))]; end ;k = k +1;clear FBA
6508     %% valine -> glutamine
6509     model = modelOri;
6510     model.c(find(model.c)) = 0;
6511     model.lb(ismember(model.rxns,'EX_glc(e)'))=0;model.ub(ismember(model.rxns,'EX_glc(e)'))=0;
6512     model.lb(ismember(model.rxns,'EX_val_L(e)'))=-1;model.ub(ismember(model.rxns,'EX_val_L(e)'))=-1;
6513     model.lb(ismember(model.rxns,'EX_o2(e)'))=-40;model.ub(ismember(model.rxns,'EX_o2(e)'))=-1;
6514     [model] = addSinkReactions(model,{'gln-L(c)'},[0 100]);
6515     model.c(ismember(model.rxns,'sink_gln-L(c)'))=1;
6516     model.c(ismember(model.rxns,'sink_gln_L(c)'))=1;
6517     if find(model.c)>0
6518         FBA = optimizeCbModel(model,'max');
6519         TestSolution(k,1) = FBA.f;
6520     else
6521         TestSolution(k,1) = NaN;
6522     end
6523     TestSolutionName{k,1} = 'valine -> glutamine';
6524     if ~isnan(TestSolution(k,1)); TestedRxns = [TestedRxns; model.rxns(find(abs(FBA.x)>tol))]; end ;k = k +1;clear FBA
6525     %% valine -> alanine
6526     model = modelOri;
6527     model.c(find(model.c)) = 0;
6528     model.lb(ismember(model.rxns,'EX_glc(e)'))=0;model.ub(ismember(model.rxns,'EX_glc(e)'))=0;
6529     model.lb(ismember(model.rxns,'EX_val_L(e)'))=-1;model.ub(ismember(model.rxns,'EX_val_L(e)'))=-1;
6530     model.lb(ismember(model.rxns,'EX_o2(e)'))=-40;model.ub(ismember(model.rxns,'EX_o2(e)'))=-1;
6531     [model] = addSinkReactions(model,{'ala-L(c)'},[0 100]);
6532     model.c(ismember(model.rxns,'sink_ala-L(c)'))=1;
6533     model.c(ismember(model.rxns,'sink_ala_L(c)'))=1;
6534     if find(model.c)>0
6535         FBA = optimizeCbModel(model,'max');
6536         TestSolution(k,1) = FBA.f;
6537     else
6538         TestSolution(k,1) = NaN;
6539     end
6540     TestSolutionName{k,1} = 'valine -> alanine';
6541     if ~isnan(TestSolution(k,1)); TestedRxns = [TestedRxns; model.rxns(find(abs(FBA.x)>tol))]; end ;k = k +1;clear FBA
6542     %% isoleucine -> glutamine
6543     model = modelOri;
6544     model.c(find(model.c)) = 0;
6545     model.lb(ismember(model.rxns,'EX_glc(e)'))=0;model.ub(ismember(model.rxns,'EX_glc(e)'))=0;
6546     model.lb(ismember(model.rxns,'EX_ile_L(e)'))=-1;model.ub(ismember(model.rxns,'EX_ile_L(e)'))=-1;
6547     model.lb(ismember(model.rxns,'EX_o2(e)'))=-40;model.ub(ismember(model.rxns,'EX_o2(e)'))=-1;
6548     [model] = addSinkReactions(model,{'gln-L(c)'},[0 100]);
6549     model.c(ismember(model.rxns,'sink_gln-L(c)'))=1;
6550     model.c(ismember(model.rxns,'sink_gln_L(c)'))=1;
6551     if find(model.c)>0
6552         FBA = optimizeCbModel(model,'max');
6553         TestSolution(k,1) = FBA.f;
6554     else
6555         TestSolution(k,1) = NaN;
6556     end
6557     TestSolutionName{k,1} = 'isoleucine -> glutamine';
6558     if ~isnan(TestSolution(k,1)); TestedRxns = [TestedRxns; model.rxns(find(abs(FBA.x)>tol))]; end ;k = k +1;clear FBA
6559     %% isoleucine -> alanine
6560     model = modelOri;
6561     model.c(find(model.c)) = 0;
6562     model.lb(ismember(model.rxns,'EX_glc(e)'))=0;model.ub(ismember(model.rxns,'EX_glc(e)'))=0;
6563     model.lb(ismember(model.rxns,'EX_ile_L(e)'))=-1;model.ub(ismember(model.rxns,'EX_ile_L(e)'))=-1;
6564     model.lb(ismember(model.rxns,'EX_o2(e)'))=-40;model.ub(ismember(model.rxns,'EX_o2(e)'))=-1;
6565     [model] = addSinkReactions(model,{'ala-L(c)'},[0 100]);
6566     model.c(ismember(model.rxns,'sink_ala-L(c)'))=1;
6567     model.c(ismember(model.rxns,'sink_ala_L(c)'))=1;
6568     if find(model.c)>0
6569         FBA = optimizeCbModel(model,'max');
6570         TestSolution(k,1) = FBA.f;
6571     else
6572         TestSolution(k,1) = NaN;
6573     end
6574     TestSolutionName{k,1} = 'isoleucine -> alanine';
6575     if ~isnan(TestSolution(k,1)); TestedRxns = [TestedRxns; model.rxns(find(abs(FBA.x)>tol))]; end ;k = k +1;clear FBA
6576     %% methionine -> glutamine
6577     model = modelOri;
6578     model.c(find(model.c)) = 0;
6579     model.lb(ismember(model.rxns,'EX_glc(e)'))=0;model.ub(ismember(model.rxns,'EX_glc(e)'))=0;
6580     model.lb(ismember(model.rxns,'EX_met_L(e)'))=-1;model.ub(ismember(model.rxns,'EX_met_L(e)'))=-1;
6581     model.lb(ismember(model.rxns,'EX_o2(e)'))=-40;model.ub(ismember(model.rxns,'EX_o2(e)'))=-1;
6582     [model] = addSinkReactions(model,{'gln-L(c)'},[0 100]);
6583     model.c(ismember(model.rxns,'sink_gln-L(c)'))=1;
6584     model.c(ismember(model.rxns,'sink_gln_L(c)'))=1;
6585     if find(model.c)>0
6586         FBA = optimizeCbModel(model,'max');
6587         TestSolution(k,1) = FBA.f;
6588     else
6589         TestSolution(k,1) = NaN;
6590     end
6591     TestSolutionName{k,1} = 'methionine -> glutamine';
6592     if ~isnan(TestSolution(k,1)); TestedRxns = [TestedRxns; model.rxns(find(abs(FBA.x)>tol))]; end ;k = k +1;clear FBA
6593     %% methionine -> alanine
6594     model = modelOri;
6595     model.c(find(model.c)) = 0;
6596     model.lb(ismember(model.rxns,'EX_glc(e)'))=0;model.ub(ismember(model.rxns,'EX_glc(e)'))=0;
6597     model.lb(ismember(model.rxns,'EX_met_L(e)'))=-1;model.ub(ismember(model.rxns,'EX_met_L(e)'))=-1;
6598     model.lb(ismember(model.rxns,'EX_o2(e)'))=-40;model.ub(ismember(model.rxns,'EX_o2(e)'))=-1;
6599     [model] = addSinkReactions(model,{'ala-L(c)'},[0 100]);
6600     model.c(ismember(model.rxns,'sink_ala-L(c)'))=1;
6601     model.c(ismember(model.rxns,'sink_ala_L(c)'))=1;
6602     if find(model.c)>0
6603         FBA = optimizeCbModel(model,'max');
6604         TestSolution(k,1) = FBA.f;
6605     else
6606         TestSolution(k,1) = NaN;
6607     end
6608     TestSolutionName{k,1} = 'methionine -> alanine';
6609     if ~isnan(TestSolution(k,1)); TestedRxns = [TestedRxns; model.rxns(find(abs(FBA.x)>tol))]; end ;k = k +1;clear FBA
6610     %% arginine -> ornithine
6611     model = modelOri;
6612     model.c(find(model.c)) = 0;
6613     model.lb(ismember(model.rxns,'EX_glc(e)'))=0;model.ub(ismember(model.rxns,'EX_glc(e)'))=0;
6614     model.lb(ismember(model.rxns,'EX_arg-L(e)'))=-1;model.ub(ismember(model.rxns,'EX_arg-L(e)'))=-1;
6615     model.lb(ismember(model.rxns,'EX_o2(e)'))=-40;model.ub(ismember(model.rxns,'EX_o2(e)'))=-1;
6616     [model] = addSinkReactions(model,{'orn(c)'},[0 100]);
6617     model.c(ismember(model.rxns,'sink_orn(c)'))=1;
6618     if find(model.c)>0
6619         FBA = optimizeCbModel(model,'max');
6620         TestSolution(k,1) = FBA.f;
6621     else
6622         TestSolution(k,1) = NaN;
6623     end
6624     TestSolutionName{k,1} = 'arginine -> ornithine';
6625     if ~isnan(TestSolution(k,1)); TestedRxns = [TestedRxns; model.rxns(find(abs(FBA.x)>tol))]; end ;k = k +1;clear FBA
6626     %% arginine -> proline
6627     model = modelOri;
6628     model.c(find(model.c)) = 0;
6629     model.lb(ismember(model.rxns,'EX_glc(e)'))=0;model.ub(ismember(model.rxns,'EX_glc(e)'))=0;
6630     model.lb(ismember(model.rxns,'EX_arg-L(e)'))=-1;model.ub(ismember(model.rxns,'EX_arg-L(e)'))=-1;
6631     model.lb(ismember(model.rxns,'EX_o2(e)'))=-40;model.ub(ismember(model.rxns,'EX_o2(e)'))=-1;
6632     [model] = addSinkReactions(model,{'pro-L(c)'},[0 100]);
6633     model.c(ismember(model.rxns,'sink_pro-L(c)'))=1;
6634     model.c(ismember(model.rxns,'sink_pro_L(c)'))=1;
6635     if find(model.c)>0
6636         FBA = optimizeCbModel(model,'max');
6637         TestSolution(k,1) = FBA.f;
6638     else
6639         TestSolution(k,1) = NaN;
6640     end
6641     TestSolutionName{k,1} = 'arginine -> proline';
6642     if ~isnan(TestSolution(k,1)); TestedRxns = [TestedRxns; model.rxns(find(abs(FBA.x)>tol))]; end ;k = k +1;clear FBA
6643     %% ornithine -> putrescine
6644     model = modelOri;
6645     model.c(find(model.c)) = 0;
6646     model.lb(ismember(model.rxns,'EX_glc(e)'))=0;model.ub(ismember(model.rxns,'EX_glc(e)'))=0;
6647     model.lb(ismember(model.rxns,'EX_orn(e)'))=-1;model.ub(ismember(model.rxns,'EX_orn(e)'))=-1;
6648     model.lb(ismember(model.rxns,'EX_o2(e)'))=-40;model.ub(ismember(model.rxns,'EX_o2(e)'))=-1;
6649     [model] = addSinkReactions(model,{'ptrc(c)'},[0 100]);
6650     model.c(ismember(model.rxns,'sink_ptrc(c)'))=1;
6651     if find(model.c)>0
6652         FBA = optimizeCbModel(model,'max');
6653         TestSolution(k,1) = FBA.f;
6654     else
6655         TestSolution(k,1) = NaN;
6656     end
6657     TestSolutionName{k,1} = 'ornithine -> putrescine';
6658     if ~isnan(TestSolution(k,1)); TestedRxns = [TestedRxns; model.rxns(find(abs(FBA.x)>tol))]; end ;k = k +1;clear FBA
6659     %% glutamate -> glutamine
6660     model = modelOri;
6661     model.c(find(model.c)) = 0;
6662     model.lb(ismember(model.rxns,'EX_glc(e)'))=0;model.ub(ismember(model.rxns,'EX_glc(e)'))=0;
6663     model.lb(ismember(model.rxns,'EX_glu-L(e)'))=-1;model.ub(ismember(model.rxns,'EX_glu-L(e)'))=-1;
6664     model.lb(ismember(model.rxns,'EX_o2(e)'))=-40;model.ub(ismember(model.rxns,'EX_o2(e)'))=-1;
6665     [model] = addSinkReactions(model,{'gln-L(c)'},[0 100]);
6666     model.c(ismember(model.rxns,'sink_gln-L(c)'))=1;
6667     model.c(ismember(model.rxns,'sink_gln_L(c)'))=1;
6668     if find(model.c)>0
6669         FBA = optimizeCbModel(model,'max');
6670         TestSolution(k,1) = FBA.f;
6671     else
6672         TestSolution(k,1) = NaN;
6673     end
6674     TestSolutionName{k,1} = 'glutamate -> glutamine';
6675     if ~isnan(TestSolution(k,1)); TestedRxns = [TestedRxns; model.rxns(find(abs(FBA.x)>tol))]; end ;k = k +1;clear FBA
6676     %% methionine -> spermine
6677     model = modelOri;
6678     model.c(find(model.c)) = 0;
6679     model.lb(ismember(model.rxns,'EX_glc(e)'))=0;model.ub(ismember(model.rxns,'EX_glc(e)'))=0;
6680     model.lb(ismember(model.rxns,'EX_met_L(e)'))=-1;model.ub(ismember(model.rxns,'EX_met_L(e)'))=-1;
6681     model.lb(ismember(model.rxns,'EX_o2(e)'))=-40;model.ub(ismember(model.rxns,'EX_o2(e)'))=-1;
6682     [model] = addSinkReactions(model,{'sprm(c)'},[0 100]);
6683     model.c(ismember(model.rxns,'sink_sprm(c)'))=1;
6684     if find(model.c)>0
6685         FBA = optimizeCbModel(model,'max');
6686         TestSolution(k,1) = FBA.f;
6687     else
6688         TestSolution(k,1) = NaN;
6689     end
6690     TestSolutionName{k,1} = 'methionine -> spermine';
6691     if ~isnan(TestSolution(k,1)); TestedRxns = [TestedRxns; model.rxns(find(abs(FBA.x)>tol))]; end ;k = k +1;clear FBA
6692     %% methionine -> spermidine
6693     model = modelOri;
6694     model.c(find(model.c)) = 0;
6695     model.lb(ismember(model.rxns,'EX_glc(e)'))=0;model.ub(ismember(model.rxns,'EX_glc(e)'))=0;
6696     model.lb(ismember(model.rxns,'EX_met_L(e)'))=-1;model.ub(ismember(model.rxns,'EX_met_L(e)'))=-1;
6697     model.lb(ismember(model.rxns,'EX_o2(e)'))=-40;model.ub(ismember(model.rxns,'EX_o2(e)'))=-1;
6698     [model] = addSinkReactions(model,{'spmd(c)'},[0 100]);
6699     model.c(ismember(model.rxns,'sink_spmd(c)'))=1;
6700     if find(model.c)>0
6701         FBA = optimizeCbModel(model,'max');
6702         TestSolution(k,1) = FBA.f;
6703     else
6704         TestSolution(k,1) = NaN;
6705     end
6706     TestSolutionName{k,1} = 'methionine -> spermidine';
6707     if ~isnan(TestSolution(k,1)); TestedRxns = [TestedRxns; model.rxns(find(abs(FBA.x)>tol))]; end ;k = k +1;clear FBA
6708     %% spermidine -> putrescine
6709     model = modelOri;
6710     model.c(find(model.c)) = 0;
6711     model.lb(ismember(model.rxns,'EX_glc(e)'))=0;model.ub(ismember(model.rxns,'EX_glc(e)'))=0;
6712     model.lb(ismember(model.rxns,'EX_spmd(e)'))=-1;model.ub(ismember(model.rxns,'EX_spmd(e)'))=-1;
6713     model.lb(ismember(model.rxns,'EX_o2(e)'))=-40;model.ub(ismember(model.rxns,'EX_o2(e)'))=-1;
6714     [model] = addSinkReactions(model,{'ptrc(c)'},[0 100]);
6715     model.c(ismember(model.rxns,'sink_ptrc(c)'))=1;
6716     if find(model.c)>0
6717         FBA = optimizeCbModel(model,'max');
6718         TestSolution(k,1) = FBA.f;
6719     else
6720         TestSolution(k,1) = NaN;
6721     end
6722     TestSolutionName{k,1} = 'spermidine -> putrescine';
6723     if ~isnan(TestSolution(k,1)); TestedRxns = [TestedRxns; model.rxns(find(abs(FBA.x)>tol))]; end ;k = k +1;clear FBA
6724     %% ADP -> ATP/ adenylate kinase
6725     model = modelOri;
6726     model.c(find(model.c)) = 0;
6727     model.lb(ismember(model.rxns,'EX_o2(e)'))=-40;model.ub(ismember(model.rxns,'EX_o2(e)'))=-1;
6728     model.lb(ismember(model.rxns,'EX_glc(e)'))=-1;model.ub(ismember(model.rxns,'EX_glc(e)'))=0;
6729     if ~isempty(strmatch('AK1',model.rxns,'exact'))
6730         model.c(ismember(model.rxns,'AK1'))=1;
6731         FBA = optimizeCbModel(model,'max');
6732         TestSolution(k,1) = FBA.f;
6733     else
6734         TestSolution(k,1) = NaN;
6735     end
6736     TestSolutionName{k,1} = 'ADP -> ATP/ adenylate kinase';
6737     if ~isnan(TestSolution(k,1)); TestedRxns = [TestedRxns; model.rxns(find(abs(FBA.x)>tol))]; end ;k = k +1;clear FBA
6738     %% ADP -> ATP/ adenylate kinase
6739     model = modelOri;
6740     model.c(find(model.c)) = 0;
6741     model.lb(ismember(model.rxns,'EX_o2(e)'))=-40;model.ub(ismember(model.rxns,'EX_o2(e)'))=-1;
6742     model.lb(ismember(model.rxns,'EX_glc(e)'))=-1;model.ub(ismember(model.rxns,'EX_glc(e)'))=0;
6743     if ~isempty(strmatch('AK1',model.rxns,'exact'))
6744         model.c(ismember(model.rxns,'AK1m'))=1;
6745         FBA = optimizeCbModel(model,'max');
6746         TestSolution(k,1) = FBA.f;
6747     else
6748         TestSolution(k,1) = NaN;
6749     end
6750     TestSolutionName{k,1} = 'ADP -> ATP/ adenylate kinase (mitochondrial)';
6751     if ~isnan(TestSolution(k,1)); TestedRxns = [TestedRxns; model.rxns(find(abs(FBA.x)>tol))]; end ;k = k +1;clear FBA
6752     %% phosphocreatine -> creatine/ cytosolic creatine kinase
6753     model = modelOri;
6754     model.c(find(model.c)) = 0;
6755     model = addReaction(model,'EX_pcreat(e)','pcreat[e] <=>');
6756     model.lb(ismember(model.rxns,'EX_glc(e)'))=-1;model.ub(ismember(model.rxns,'EX_glc(e)'))=0;
6757     model.lb(ismember(model.rxns,'EX_o2(e)'))=-40;model.ub(ismember(model.rxns,'EX_o2(e)'))=0;
6758     model.lb(ismember(model.rxns,'EX_pcreat(e)'))=-1;model.ub(ismember(model.rxns,'EX_pcreat(e)'))=-1;
6759     [model] = addSinkReactions(model,{'creat(c)'},[0 100]);
6760     model.c(ismember(model.rxns,'sink_creat(c)'))=1;
6761     if find(model.c)>0
6762         FBA = optimizeCbModel(model,'max');
6763         TestSolution(k,1) = FBA.f;
6764     else
6765         TestSolution(k,1) = NaN;
6766     end
6767     TestSolutionName{k,1} = 'phosphocreatine -> creatine/ cytosolic creatine kinase';
6768     if ~isnan(TestSolution(k,1)); TestedRxns = [TestedRxns; model.rxns(find(abs(FBA.x)>tol))]; end ;k = k +1;clear FBA
6769     %% creatine -> phosphocreatine/mitochondrial creatine kinase
6770     model = modelOri;
6771     model.c(find(model.c)) = 0;
6772     model.lb(ismember(model.rxns,'EX_creat(e)'))=-1;model.ub(ismember(model.rxns,'EX_creat(e)'))=-1;
6773     model.lb(ismember(model.rxns,'EX_o2(e)'))=-40;model.ub(ismember(model.rxns,'EX_o2(e)'))=-1;
6774     [model] = addSinkReactions(model,{'pcreat(c)'},[0 100]);
6775     model.c(ismember(model.rxns,'sink_pcreat(c)'))=1;
6776     if find(model.c)>0
6777         FBA = optimizeCbModel(model,'max');
6778         TestSolution(k,1) = FBA.f;
6779     else
6780         TestSolution(k,1) = NaN;
6781     end
6782     TestSolutionName{k,1} = 'creatine -> phosphocreatine/mitochondrial creatine kinase';
6783     if ~isnan(TestSolution(k,1)); TestedRxns = [TestedRxns; model.rxns(find(abs(FBA.x)>tol))]; end ;k = k +1;clear FBA
6784     %% fructose -> lactate/ oxidation of fructose
6785     model = modelOri;
6786     model.c(find(model.c)) = 0;
6787     model.lb(ismember(model.rxns,'EX_fru(e)'))=-1;model.ub(ismember(model.rxns,'EX_fru(e)'))=-1;
6788     model.lb(ismember(model.rxns,'EX_o2(e)'))=-40;model.ub(ismember(model.rxns,'EX_o2(e)'))=0;
6789     [model] = addSinkReactions(model,{'lac-L(c)'},[0 100]);
6790     model.c(ismember(model.rxns,'sink_lac-L(c)'))=1;
6791     if find(model.c)>0
6792         FBA = optimizeCbModel(model,'max');
6793         TestSolution(k,1) = FBA.f;
6794     else
6795         TestSolution(k,1) = NaN;
6796     end
6797     TestSolutionName{k,1} = 'fructose -> lactate/ oxidation of fructose';
6798     if ~isnan(TestSolution(k,1)); TestedRxns = [TestedRxns; model.rxns(find(abs(FBA.x)>tol))]; end ;k = k +1;clear FBA
6799     %% fructose -> glycogen/ glycogenesis
6800     model = modelOri;
6801     model.c(find(model.c)) = 0;
6802     model.lb(ismember(model.rxns,'EX_fru(e)'))=-1;model.ub(ismember(model.rxns,'EX_fru(e)'))=-1;
6803     model.lb(ismember(model.rxns,'EX_o2(e)'))=-40;model.ub(ismember(model.rxns,'EX_o2(e)'))=0;
6804     [model] = addSinkReactions(model,{'glygn2(c)'},[0 100]);
6805     model.c(ismember(model.rxns,'sink_glygn2(c)'))=1;
6806     if find(model.c)>0
6807         FBA = optimizeCbModel(model,'max');
6808         TestSolution(k,1) = FBA.f;
6809     else
6810         TestSolution(k,1) = NaN;
6811     end
6812     TestSolutionName{k,1} = 'fructose -> glycogen/ glycogenesis';
6813     if ~isnan(TestSolution(k,1)); TestedRxns = [TestedRxns; model.rxns(find(abs(FBA.x)>tol))]; end ;k = k +1;clear FBA
6814     %% glucose -> erythrose/ HMP shunt
6815     model = modelOri;
6816     model.c(find(model.c)) = 0;
6817     model.lb(ismember(model.rxns,'EX_glc(e)'))=-1;model.ub(ismember(model.rxns,'EX_glc(e)'))=-1;
6818     model.lb(ismember(model.rxns,'EX_o2(e)'))=-40;model.ub(ismember(model.rxns,'EX_o2(e)'))=-1;
6819     [model] = addSinkReactions(model,{'e4p(c)'},[0 100]);
6820     model.c(ismember(model.rxns,'sink_e4p(c)'))=1;
6821     if find(model.c)>0
6822         FBA = optimizeCbModel(model,'max');
6823         TestSolution(k,1) = FBA.f;
6824     else
6825         TestSolution(k,1) = NaN;
6826     end
6827     TestSolutionName{k,1} = 'glucose -> erythrose/ HMP shunt';
6828     if ~isnan(TestSolution(k,1)); TestedRxns = [TestedRxns; model.rxns(find(abs(FBA.x)>tol))]; end ;k = k +1;clear FBA
6829     %% tag_hs(c) -> mag_hs(c)/ lipolysis
6830     model = modelOri;
6831     model.c(find(model.c)) = 0;
6832     model.lb(ismember(model.rxns,'EX_glc(e)'))=0;model.ub(ismember(model.rxns,'EX_glc(e)'))=0;
6833     model.lb(ismember(model.rxns,'EX_tag_hs(e)'))=-1;model.ub(ismember(model.rxns,'EX_tag_hs(e)'))=-1;
6834     model.lb(ismember(model.rxns,'EX_o2(e)'))=-40;model.ub(ismember(model.rxns,'EX_o2(e)'))=0;
6835     [model] = addSinkReactions(model,{'mag-hs(c)'},[0 100]);
6836     model.c(ismember(model.rxns,'sink_mag-hs(c)'))=1;
6837     if find(model.c)>0
6838         FBA = optimizeCbModel(model,'max');
6839         TestSolution(k,1) = FBA.f;
6840     else
6841         TestSolution(k,1) = NaN;
6842     end
6843     TestSolutionName{k,1} = 'tag_hs(c) -> mag_hs(c)/ lipolysis';
6844     if ~isnan(TestSolution(k,1)); TestedRxns = [TestedRxns; model.rxns(find(abs(FBA.x)>tol))]; end ;k = k +1;clear FBA
6845     %% tag_hs(c) -> glyc(c)/ lipolysis
6846     model = modelOri;
6847     model.c(find(model.c)) = 0;
6848     model.lb(ismember(model.rxns,'EX_glc(e)'))=0;model.ub(ismember(model.rxns,'EX_glc(e)'))=0;
6849     model.lb(ismember(model.rxns,'EX_tag_hs(e)'))=-1;model.ub(ismember(model.rxns,'EX_tag_hs(e)'))=-1;
6850     model.lb(ismember(model.rxns,'EX_o2(e)'))=-40;model.ub(ismember(model.rxns,'EX_o2(e)'))=-1;
6851     [model] = addSinkReactions(model,{'glyc(c)'},[0 100]);
6852     model.c(ismember(model.rxns,'sink_glyc(c)'))=1;
6853     if find(model.c)>0
6854         FBA = optimizeCbModel(model,'max');
6855         TestSolution(k,1) = FBA.f;
6856     else
6857         TestSolution(k,1) = NaN;
6858     end
6859     TestSolutionName{k,1} = 'tag_hs(c) -> glyc(c)/ lipolysis';
6860     if ~isnan(TestSolution(k,1)); TestedRxns = [TestedRxns; model.rxns(find(abs(FBA.x)>tol))]; end ;k = k +1;clear FBA
6861     %% pmtcoa -> acetylCoA/ beta oxidation from pmtcoa
6862     model = modelOri;
6863     %         for i = 1 : length(RPMI_composition)
6864     %         model = changeRxnBounds(model,RPMI_composition{i},-1,'l');
6865     %     end
6866     model.c(find(model.c)) = 0;
6867     model.lb(ismember(model.rxns,'EX_hdca(e)'))=-1;model.ub(ismember(model.rxns,'EX_hdca(e)'))=-1;
6868     model.lb(ismember(model.rxns,'EX_o2(e)'))=-40;model.ub(ismember(model.rxns,'EX_o2(e)'))=-1;
6869     model.lb(find(ismember(model.rxns,'sink_coa(c)')))=-1;
6870     model.ub(find(ismember(model.rxns,'sink_coa(c)')))=1;
6871     [model] = addSinkReactions(model,{'accoa(m)'},[0 100]);
6872     model.c(ismember(model.rxns,'sink_accoa(m)'))=1;
6873     if find(model.c)>0
6874         FBA = optimizeCbModel(model,'max');
6875         TestSolution(k,1) = FBA.f;
6876     else
6877         TestSolution(k,1) = NaN;
6878     end
6879     TestSolutionName{k,1} = 'pmtcoa -> acetylCoA/ beta oxidation from pmtcoa';
6880     if ~isnan(TestSolution(k,1)); TestedRxns = [TestedRxns; model.rxns(find(abs(FBA.x)>tol))]; end ;k = k +1;clear FBA
6881     %% odecoa -> acetylCoA/ beta oxidation from oleic acid
6882     model = modelOri;
6883     %         for i = 1 : length(RPMI_composition)
6884     %         model = changeRxnBounds(model,RPMI_composition{i},-1,'l');
6885     %     end
6886     model.c(find(model.c)) = 0;
6887     model.lb(ismember(model.rxns,'EX_ocdcea(e)'))=-1;model.ub(ismember(model.rxns,'EX_ocdcea(e)'))=-1;
6888     model.lb(ismember(model.rxns,'EX_o2(e)'))=-40;model.ub(ismember(model.rxns,'EX_o2(e)'))=0;
6889     model.lb(find(ismember(model.rxns,'sink_coa(c)')))=-1;
6890     model.ub(find(ismember(model.rxns,'sink_coa(c)')))=1;
6891     [model] = addSinkReactions(model,{'accoa(m)'},[0 100]);
6892     model.c(ismember(model.rxns,'sink_accoa(m)'))=1;
6893     if find(model.c)>0
6894         FBA = optimizeCbModel(model,'max');
6895         TestSolution(k,1) = FBA.f;
6896     else
6897         TestSolution(k,1) = NaN;
6898     end
6899     TestSolutionName{k,1} = 'odecoa -> acetylCoA/ beta oxidation from oleic acid (with RPMI medium)';
6900     if ~isnan(TestSolution(k,1)); TestedRxns = [TestedRxns; model.rxns(find(abs(FBA.x)>tol))]; end ;k = k +1;clear FBA
6901     %% lnlccoa -> acetylCoA/ beta oxidation from linoleic acid
6902     model = modelOri;
6903     %         for i = 1 : length(RPMI_composition)
6904     %         model = changeRxnBounds(model,RPMI_composition{i},-1,'l');
6905     %     end
6906     model.c(find(model.c)) = 0;
6907     model.lb(ismember(model.rxns,'EX_lnlc(e)'))=-1;model.ub(ismember(model.rxns,'EX_lnlc(e)'))=-1;
6908     model.lb(ismember(model.rxns,'EX_o2(e)'))=-40;model.ub(ismember(model.rxns,'EX_o2(e)'))=0;
6909     model.lb(find(ismember(model.rxns,'sink_coa(c)')))=-1;
6910     model.ub(find(ismember(model.rxns,'sink_coa(c)')))=1;
6911     [model] = addSinkReactions(model,{'accoa(m)'},[0 100]);
6912     model.c(ismember(model.rxns,'sink_accoa(m)'))=1;
6913     if find(model.c)>0
6914         FBA = optimizeCbModel(model,'max');
6915         TestSolution(k,1) = FBA.f;
6916     else
6917         TestSolution(k,1) = NaN;
6918     end
6919     TestSolutionName{k,1} = 'lnlccoa -> acetylCoA/ beta oxidation from linoleic acid (with RPMI medium)';
6920     if ~isnan(TestSolution(k,1)); TestedRxns = [TestedRxns; model.rxns(find(abs(FBA.x)>tol))]; end ;k = k +1;clear FBA
6921     %% glycerol -> dhap/ glycerol utilizing machinery
6922     model = modelOri;
6923     model.c(find(model.c)) = 0;
6924     model.lb(ismember(model.rxns,'EX_glc(e)'))=0;model.ub(ismember(model.rxns,'EX_glc(e)'))=0;
6925     model.lb(ismember(model.rxns,'EX_glyc(e)'))=-1;model.ub(ismember(model.rxns,'EX_glyc(e)'))=-1;
6926     model.lb(ismember(model.rxns,'EX_o2(e)'))=-40;model.ub(ismember(model.rxns,'EX_o2(e)'))=-1;
6927     [model] = addSinkReactions(model,{'dhap(c)'},[0 100]);
6928     model.c(ismember(model.rxns,'sink_dhap(c)'))=1;
6929     if find(model.c)>0
6930         FBA = optimizeCbModel(model,'max');
6931         TestSolution(k,1) = FBA.f;
6932     else
6933         TestSolution(k,1) = NaN;
6934     end
6935     TestSolutionName{k,1} = 'glycerol -> dhap/ glycerol utilizing machinery';
6936     if ~isnan(TestSolution(k,1)); TestedRxns = [TestedRxns; model.rxns(find(abs(FBA.x)>tol))]; end ;k = k +1;clear FBA
6937     %% adenine -> amp/ salvage of adenine
6938     model = modelOri;
6939     model.c(find(model.c)) = 0;
6940     model.lb(ismember(model.rxns,'EX_glc(e)'))=0;model.ub(ismember(model.rxns,'EX_glc(e)'))=0;
6941     model.lb(ismember(model.rxns,'EX_adn(e)'))=-1;model.ub(ismember(model.rxns,'EX_adn(e)'))=-1;
6942     model.lb(ismember(model.rxns,'EX_o2(e)'))=-40;model.ub(ismember(model.rxns,'EX_o2(e)'))=-1;
6943     [model] = addSinkReactions(model,{'amp(c)'},[0 100]);
6944     model.c(ismember(model.rxns,'sink_amp(c)'))=1;
6945     if find(model.c)>0
6946         FBA = optimizeCbModel(model,'max');
6947         TestSolution(k,1) = FBA.f;
6948     else
6949         TestSolution(k,1) = NaN;
6950     end
6951     TestSolutionName{k,1} = 'adenine -> amp/ salvage of adenine';
6952     if ~isnan(TestSolution(k,1)); TestedRxns = [TestedRxns; model.rxns(find(abs(FBA.x)>tol))]; end ;k = k +1;clear FBA
6953     %% hypoxanthine -> imp/ salvage of hypoxanthine
6954     model = modelOri;
6955     model.c(find(model.c)) = 0;
6956     model.lb(ismember(model.rxns,'EX_hxan(e)'))=-1;model.ub(ismember(model.rxns,'EX_hxan(e)'))=-1;
6957     model.lb(ismember(model.rxns,'EX_o2(e)'))=-40;model.ub(ismember(model.rxns,'EX_o2(e)'))=-1;
6958     [model] = addSinkReactions(model,{'imp(c)'},[0 100]);
6959     model.c(ismember(model.rxns,'INSK'))=1;
6960     if find(model.c)>0
6961         FBA = optimizeCbModel(model,'max');
6962         TestSolution(k,1) = FBA.f;
6963     else
6964         TestSolution(k,1) = NaN;
6965     end
6966     TestSolutionName{k,1} = 'hypoxanthine -> imp/ salvage of hypoxanthine';
6967     if ~isnan(TestSolution(k,1)); TestedRxns = [TestedRxns; model.rxns(find(abs(FBA.x)>tol))]; end ;k = k +1;clear FBA
6968     %% guanine -> gmp/ salvage of guanine
6969     model = modelOri;
6970     model.c(find(model.c)) = 0;
6971     model.lb(ismember(model.rxns,'EX_gua(e)'))=-1;model.ub(ismember(model.rxns,'EX_gua(e)'))=-1;
6972     model.lb(ismember(model.rxns,'EX_o2(e)'))=-40;model.ub(ismember(model.rxns,'EX_o2(e)'))=0;
6973     [model] = addSinkReactions(model,{'prpp(c)','gmp(c)'},[-1 0;0 100]);
6974     model.c(ismember(model.rxns,'GUAPRT'))=1;
6975     if find(model.c)>0
6976         FBA = optimizeCbModel(model,'max');
6977         TestSolution(k,1) = FBA.f;
6978     else
6979         TestSolution(k,1) = NaN;
6980     end
6981     TestSolutionName{k,1} = 'guanine -> gmp/ salvage of guanine';
6982     if ~isnan(TestSolution(k,1)); TestedRxns = [TestedRxns; model.rxns(find(abs(FBA.x)>tol))]; end ;k = k +1;clear FBA
6983     %% ribose -> imp/ denovo purine synthesis
6984     model = modelOri;
6985     model.c(find(model.c)) = 0;
6986     model.lb(ismember(model.rxns,'EX_glc(e)'))=0;model.ub(ismember(model.rxns,'EX_glc(e)'))=0;
6987     model.lb(ismember(model.rxns,'EX_rib_D(e)'))=-1;model.ub(ismember(model.rxns,'EX_rib_D(e)'))=-1;
6988     model.lb(ismember(model.rxns,'EX_o2(e)'))=-40;model.ub(ismember(model.rxns,'EX_o2(e)'))=-1;
6989     [model] = addSinkReactions(model,{'imp(c)'},[0 100]);
6990     model.c(ismember(model.rxns,'sink_imp(c)'))=1;
6991     if find(model.c)>0
6992         FBA = optimizeCbModel(model,'max');
6993         TestSolution(k,1) = FBA.f;
6994     else
6995         TestSolution(k,1) = NaN;
6996     end
6997     TestSolutionName{k,1} = 'ribose -> imp/ denovo purine synthesis';
6998     if ~isnan(TestSolution(k,1)); TestedRxns = [TestedRxns; model.rxns(find(abs(FBA.x)>tol))]; end ;k = k +1;clear FBA
6999     %% thymd -> thym/ thymidine phosphorylase
7000     model = modelOri;
7001     model.c(find(model.c)) = 0;
7002     model.lb(ismember(model.rxns,'EX_glc(e)'))=0;model.ub(ismember(model.rxns,'EX_glc(e)'))=0;
7003     model.lb(ismember(model.rxns,'EX_thymd(e)'))=-1;model.ub(ismember(model.rxns,'EX_thymd(e)'))=-1;
7004     model.lb(ismember(model.rxns,'EX_o2(e)'))=-40;model.ub(ismember(model.rxns,'EX_o2(e)'))=-1;
7005     [model] = addSinkReactions(model,{'thym(c)'},[0 100]);
7006     model.c(ismember(model.rxns,'sink_thym(c)'))=1;
7007     if find(model.c)>0
7008         FBA = optimizeCbModel(model,'max');
7009         TestSolution(k,1) = FBA.f;
7010     else
7011         TestSolution(k,1) = NaN;
7012     end
7013     TestSolutionName{k,1} = 'thymd -> thym/ thymidine phosphorylase';
7014     if ~isnan(TestSolution(k,1)); TestedRxns = [TestedRxns; model.rxns(find(abs(FBA.x)>tol))]; end ;k = k +1;clear FBA
7015     %% glutamine -> cmp/ pyrimidine synthesis
7016     model = modelOri;
7017     model.c(find(model.c)) = 0;
7018     model.lb(ismember(model.rxns,'EX_glc(e)'))=0;model.ub(ismember(model.rxns,'EX_glc(e)'))=0;
7019     model.lb(ismember(model.rxns,'EX_gln-L(e)'))=-1;model.ub(ismember(model.rxns,'EX_gln-L(e)'))=-1;
7020     model.lb(ismember(model.rxns,'EX_o2(e)'))=-40;model.ub(ismember(model.rxns,'EX_o2(e)'))=-1;
7021     [model] = addSinkReactions(model,{'cmp(c)'},[0 100]);
7022     model.c(ismember(model.rxns,'sink_cmp(c)'))=1;
7023     if find(model.c)>0
7024         FBA = optimizeCbModel(model,'max');
7025         TestSolution(k,1) = FBA.f;
7026     else
7027         TestSolution(k,1) = NaN;
7028     end
7029     TestSolutionName{k,1} = 'glutamine -> cmp/ pyrimidine synthesis';
7030     if ~isnan(TestSolution(k,1)); TestedRxns = [TestedRxns; model.rxns(find(abs(FBA.x)>tol))]; end ;k = k +1;clear FBA
7031     %% glutamine -> dtmp/ pyrimidine synthesis
7032     model = modelOri;
7033     model.c(find(model.c)) = 0;
7034     model.lb(ismember(model.rxns,'EX_glc(e)'))=0;model.ub(ismember(model.rxns,'EX_glc(e)'))=0;
7035     model.lb(ismember(model.rxns,'EX_gln-L(e)'))=-1;model.ub(ismember(model.rxns,'EX_gln-L(e)'))=-1;
7036     model.lb(ismember(model.rxns,'EX_o2(e)'))=-40;model.ub(ismember(model.rxns,'EX_o2(e)'))=-1;
7037     [model] = addSinkReactions(model,{'dtmp(c)'},[0 100]);
7038     model.c(ismember(model.rxns,'sink_dtmp(c)'))=1;
7039     if find(model.c)>0
7040         FBA = optimizeCbModel(model,'max');
7041         TestSolution(k,1) = FBA.f;
7042     else
7043         TestSolution(k,1) = NaN;
7044     end
7045     TestSolutionName{k,1} = 'glutamine -> dtmp/ pyrimidine synthesis';
7046     if ~isnan(TestSolution(k,1)); TestedRxns = [TestedRxns; model.rxns(find(abs(FBA.x)>tol))]; end ;k = k +1;clear FBA
7047     %% Kidney objectives: citr_L(c) -> arg_L(c)
7048     model = modelOri;
7049     model.c(find(model.c)) = 0;
7050     [model] = addSinkReactions(model,{'citr-L(c)','arg-L(c)'},[-1 -1; 0 100]);
7051     model.lb(find(ismember(model.rxns,'sink_citr(c)')))=-1;
7052     model.ub(find(ismember(model.rxns,'sink_citr(c)')))=-1;
7053     model.c(ismember(model.rxns,'sink_arg-L(c)'))=1;
7054     model.c(ismember(model.rxns,'sink_arg_L(c)'))=1;
7055     if find(model.c)>0
7056         FBA = optimizeCbModel(model,'max');
7057         TestSolution(k,1) = FBA.f;
7058     else
7059         TestSolution(k,1) = NaN;
7060     end
7061     TestSolutionName{k,1} = 'citr_L(c) -> arg_L(c)';
7062     if ~isnan(TestSolution(k,1)); TestedRxns = [TestedRxns; model.rxns(find(abs(FBA.x)>tol))]; end ;k = k +1;clear FBA
7063     %% cys_L(c) -> taur(c)
7064     model = modelOri;
7065     model.c(find(model.c)) = 0;
7066     [model] = addSinkReactions(model,{'cys-L(c)','taur(c)'},[-1 -1; 0 100]);
7067     model.c(ismember(model.rxns,'sink_taur(c)'))=1;
7068     if find(model.c)>0
7069         FBA = optimizeCbModel(model,'max');
7070         TestSolution(k,1) = FBA.f;
7071     else
7072         TestSolution(k,1) = NaN;
7073     end
7074     TestSolutionName{k,1} = 'cys_L(c) -> taur(c)';
7075     if ~isnan(TestSolution(k,1)); TestedRxns = [TestedRxns; model.rxns(find(abs(FBA.x)>tol))]; end ;k = k +1;clear FBA
7076     %% gly(c) -> orn(c)
7077     model = modelOri;
7078     model.c(find(model.c)) = 0;
7079     [model] = addSinkReactions(model,{'gly(c)','orn(c)'},[-1 -1; 0 100]);
7080     model.c(ismember(model.rxns,'sink_orn(c)'))=1;
7081     if find(model.c)>0
7082         FBA = optimizeCbModel(model,'max');
7083         TestSolution(k,1) = FBA.f;
7084     else
7085         TestSolution(k,1) = NaN;
7086     end
7087     TestSolutionName{k,1} = 'gly(c) -> orn(c)';
7088     if ~isnan(TestSolution(k,1)); TestedRxns = [TestedRxns; model.rxns(find(abs(FBA.x)>tol))]; end ;k = k +1;clear FBA
7089     %% citr_L(c) -> urea(c)/ partial urea cycle in kidney
7090     model = modelOri;
7091     model.c(find(model.c)) = 0;
7092     [model] = addSinkReactions(model,{'citr-L(c)','urea(c)'},[-1 -1; 0 100]);
7093     model.c(ismember(model.rxns,'sink_urea(c)'))=1;
7094     if find(model.c)>0
7095         FBA = optimizeCbModel(model,'max');
7096         TestSolution(k,1) = FBA.f;
7097     else
7098         TestSolution(k,1) = NaN;
7099     end
7100     TestSolutionName{k,1} = 'citr_L(c) -> urea(c)/ partial urea cycle in kidney';
7101     if ~isnan(TestSolution(k,1)); TestedRxns = [TestedRxns; model.rxns(find(abs(FBA.x)>tol))]; end ;k = k +1;clear FBA
7102     %% gthrd(c) -> glycine(c)/ glutathione breakdown via ?-glutamyl-transeptidase
7103     model = modelOri;
7104     model.c(find(model.c)) = 0;
7105     model.lb(ismember(model.rxns,'EX_glc(e)'))=-1;model.ub(ismember(model.rxns,'EX_glc(e)'))=0;
7106     model.lb(ismember(model.rxns,'EX_o2(e)'))=-40;model.ub(ismember(model.rxns,'EX_o2(e)'))=0;
7107     [model] = addSinkReactions(model,{'gly(c)','gthrd(c)'},[-1 -1; 0 100]);
7108     model.lb(find(ismember(model.rxns,'sink_gly(c)')))=-1;
7109     model.ub(find(ismember(model.rxns,'sink_gly(c)')))=-1;
7110     model.c(ismember(model.rxns,'sink_gthrd(c)'))=1;
7111     if find(model.c)>0
7112         FBA = optimizeCbModel(model,'max');
7113         TestSolution(k,1) = FBA.f;
7114     else
7115         TestSolution(k,1) = NaN;
7116     end
7117     TestSolutionName{k,1} = 'gthrd(c) -> glycine(c)/ glutathione breakdown via glutamyl-transeptidase';
7118     if ~isnan(TestSolution(k,1)); TestedRxns = [TestedRxns; model.rxns(find(abs(FBA.x)>tol))]; end ;k = k +1;clear FBA
7119     %% pro_L(c) -> GABA(c)/ GABA synthesis in kidney
7120     model = modelOri;
7121     model.c(find(model.c)) = 0;
7122     [model] = addSinkReactions(model,{'pro-L(c)','4abut(c)'},[-1 -1; 0 100]);
7123     model.c(ismember(model.rxns,'sink_4abut(c)'))=1;
7124     if find(model.c)>0
7125         FBA = optimizeCbModel(model,'max');
7126         TestSolution(k,1) = FBA.f;
7127     else
7128         TestSolution(k,1) = NaN;
7129     end
7130     TestSolutionName{k,1} = 'pro_L(c) -> GABA(c)/ GABA synthesis in kidney';
7131     if ~isnan(TestSolution(k,1)); TestedRxns = [TestedRxns; model.rxns(find(abs(FBA.x)>tol))]; end ;k = k +1;clear FBA
7132     %% pro_L(c) -> orn(c)
7133     model = modelOri;
7134     model.c(find(model.c)) = 0;
7135     [model] = addSinkReactions(model,{'pro-L(c)','orn(c)'},[-1 -1; 0 100]);
7136     model.c(ismember(model.rxns,'sink_orn(c)'))=1;
7137     if find(model.c)>0
7138         FBA = optimizeCbModel(model,'max');
7139         TestSolution(k,1) = FBA.f;
7140     else
7141         TestSolution(k,1) = NaN;
7142     end
7143     TestSolutionName{k,1} = 'pro_L(c) -> orn(c)';
7144     if ~isnan(TestSolution(k,1)); TestedRxns = [TestedRxns; model.rxns(find(abs(FBA.x)>tol))]; end ;k = k +1;clear FBA
7145     %% met_L(c) -> hcys_L(c)
7146     model = modelOri;
7147     model.c(find(model.c)) = 0;
7148     [model] = addSinkReactions(model,{'met-L(c)','hcys-L(c)'},[-1 -1; 0 100]);
7149     model.lb(find(ismember(model.rxns,'sink_met_L(c)')))=-1;
7150     model.ub(find(ismember(model.rxns,'sink_met_L(c)')))=-1;
7151     model.c(ismember(model.rxns,'sink_hcys-L(c)'))=1;
7152     if find(model.c)>0
7153         FBA = optimizeCbModel(model,'max');
7154         TestSolution(k,1) = FBA.f;
7155     else
7156         TestSolution(k,1) = NaN;
7157     end
7158     TestSolutionName{k,1} = 'met_L(c) -> hcys_L(c)';
7159     if ~isnan(TestSolution(k,1)); TestedRxns = [TestedRxns; model.rxns(find(abs(FBA.x)>tol))]; end ;k = k +1;clear FBA
7160     %% hcys_L(c) -> met_L(c)
7161     model = modelOri;
7162     model.c(find(model.c)) = 0;
7163     [model] = addSinkReactions(model,{'hcys-L(c)','met-L(c)'},[-1 -1; 0 100]);
7164     model.c(ismember(model.rxns,'sink_met-L(c)'))=1;
7165     model.c(ismember(model.rxns,'sink_met_L(c)'))=1;
7166     if find(model.c)>0
7167         FBA = optimizeCbModel(model,'max');
7168         TestSolution(k,1) = FBA.f;
7169     else
7170         TestSolution(k,1) = NaN;
7171     end
7172     TestSolutionName{k,1} = 'hcys_L(c) -> met_L(c)';
7173     if ~isnan(TestSolution(k,1)); TestedRxns = [TestedRxns; model.rxns(find(abs(FBA.x)>tol))]; end ;k = k +1;clear FBA
7174     %% hcys_L(c) -> cys_L(c)
7175     model = modelOri;
7176     model.c(find(model.c)) = 0;
7177     model.lb(ismember(model.rxns,'EX_o2(e)'))=-40;model.ub(ismember(model.rxns,'EX_o2(e)'))=0;
7178     [model] = addSinkReactions(model,{'hcys-L(c)','cys-L(c)'},[-1 -1; 0 100]);
7179     model.lb(find(ismember(model.rxns,'sink_ser_L(c)')))=-1;
7180     model.ub(find(ismember(model.rxns,'sink_ser_L(c)')))=-1;
7181     model.c(ismember(model.rxns,'sink_cys-L(c)'))=1;
7182     model.c(ismember(model.rxns,'sink_cys_L(c)'))=1;
7183     if find(model.c)>0
7184         FBA = optimizeCbModel(model,'max');
7185         TestSolution(k,1) = FBA.f;
7186     else
7187         TestSolution(k,1) = NaN;
7188     end
7189     TestSolutionName{k,1} = 'hcys_L(c) -> cys_L(c)';
7190     if ~isnan(TestSolution(k,1)); TestedRxns = [TestedRxns; model.rxns(find(abs(FBA.x)>tol))]; end ;k = k +1;clear FBA
7191     %% 'lys-L(c) -> glu_L(c) / lysine degradation
7192     model = modelOri;
7193     model.c(find(model.c)) = 0;
7194     [model] = addSinkReactions(model,{'lys-L(c)','glu-L(c)'},[-1 -1; 0 100]);
7195     model.c(ismember(model.rxns,'sink_glu-L(c)'))=1;
7196     model.c(ismember(model.rxns,'sink_glu_L(c)'))=1;
7197     if find(model.c)>0
7198         FBA = optimizeCbModel(model,'max');
7199         TestSolution(k,1) = FBA.f;
7200     else
7201         TestSolution(k,1) = NaN;
7202     end
7203     TestSolutionName{k,1} = 'lys-L(c) -> glu_L(c) / lysine degradation';
7204     if ~isnan(TestSolution(k,1)); TestedRxns = [TestedRxns; model.rxns(find(abs(FBA.x)>tol))]; end ;k = k +1;clear FBA
7205     %% trp-L(c) -> trypta(c) / tryptophan degradation
7206     model = modelOri;
7207     model.c(find(model.c)) = 0;
7208     [model] = addSinkReactions(model,{'trp-L(c)','trypta(c)'},[-1 -1; 0 100]);
7209     model.c(ismember(model.rxns,'sink_trypta(c)'))=1;
7210     if find(model.c)>0
7211         FBA = optimizeCbModel(model,'max');
7212         TestSolution(k,1) = FBA.f;
7213     else
7214         TestSolution(k,1) = NaN;
7215     end
7216     TestSolutionName{k,1} = 'trp-L(c) -> trypta(c) / tryptophan degradation';
7217     if ~isnan(TestSolution(k,1)); TestedRxns = [TestedRxns; model.rxns(find(abs(FBA.x)>tol))]; end ;k = k +1;clear FBA
7218     %% kynate(c) -> nicotinamide(c) / nicotinamide from tryptophan metabolite
7219     model = modelOri;
7220     model.c(find(model.c)) = 0;
7221     [model] = addSinkReactions(model,{'kynate(c)','nicrnt(c)'},[-1 -1; 0 100]);
7222     model.c(ismember(model.rxns,'sink_nicrnt(c)'))=1;
7223     if find(model.c)>0
7224         FBA = optimizeCbModel(model,'max');
7225         TestSolution(k,1) = FBA.f;
7226     else
7227         TestSolution(k,1) = NaN;
7228     end
7229     TestSolutionName{k,1} = 'kynate(c) -> nicotinamide(c) / nicotinamide from tryptophan metabolite';
7230     if ~isnan(TestSolution(k,1)); TestedRxns = [TestedRxns; model.rxns(find(abs(FBA.x)>tol))]; end ;k = k +1;clear FBA
7231     %% pyr(c) -> lac-L(c)/ lactate dehydrogenase
7232     model = modelOri;
7233     model.c(find(model.c)) = 0;
7234     [model] = addSinkReactions(model,{'pyr(c)','lac-L(c)'},[-1 -1; 0 100]);
7235     model.c(ismember(model.rxns,'sink_lac-L(c)'))=1;
7236     if find(model.c)>0
7237         FBA = optimizeCbModel(model,'max');
7238         TestSolution(k,1) = FBA.f;
7239     else
7240         TestSolution(k,1) = NaN;
7241     end
7242     TestSolutionName{k,1} = 'pyr(c) -> lac-L(c)/ lactate dehydrogenase';
7243     if ~isnan(TestSolution(k,1)); TestedRxns = [TestedRxns; model.rxns(find(abs(FBA.x)>tol))]; end ;k = k +1;clear FBA
7244     %% ATP max, aerobic, pyruvate/ pyruvate dehydrogenase-->TCA->energy
7245     model = modelOri;
7246     model.c(find(model.c)) = 0;
7247     model.lb(ismember(model.rxns,'EX_pyr(e)'))=-1;model.ub(ismember(model.rxns,'EX_pyr(e)'))=-1;
7248     model.lb(ismember(model.rxns,'EX_o2(e)'))=-40;model.ub(ismember(model.rxns,'EX_o2(e)'))=-1;
7249     
7250     if ~isempty(strmatch('DM_atp(c)',model.rxns,'exact'))
7251         model.c(ismember(model.rxns,'DM_atp(c)'))=1;
7252         FBA = optimizeCbModel(model,'max');
7253         TestSolution(k,1) = FBA.f;
7254     else
7255         TestSolution(k,1) = NaN;
7256     end
7257     TestSolutionName{k,1} = 'ATP max, aerobic, pyruvate/ pyruvate dehydrogenase-->TCA->energy';
7258     if ~isnan(TestSolution(k,1)); TestedRxns = [TestedRxns; model.rxns(find(abs(FBA.x)>tol))]; end ;k = k +1;clear FBA
7259     %% gal(c) -> udpg(c)/ galactose utilization
7260     model = modelOri;
7261     model.c(find(model.c)) = 0;
7262     [model] = addSinkReactions(model,{'gal(c)','udpg(c)'},[-1 -1; 0 100]);
7263     model.c(ismember(model.rxns,'sink_udpg(c)'))=1;
7264     if find(model.c)>0
7265         FBA = optimizeCbModel(model,'max');
7266         TestSolution(k,1) = FBA.f;
7267     else
7268         TestSolution(k,1) = NaN;
7269     end
7270     TestSolutionName{k,1} = 'gal(c) -> udpg(c)/ galactose utilization';
7271     if ~isnan(TestSolution(k,1)); TestedRxns = [TestedRxns; model.rxns(find(abs(FBA.x)>tol))]; end ;k = k +1;clear FBA
7272     %% fru(c) -> lac_L(c)/ fructose conversion to glucose & utilization
7273     model = modelOri;
7274     model.c(find(model.c)) = 0;
7275     [model] = addSinkReactions(model,{'fru(c)','lac-L(c)'},[-1 -1; 0 100]);
7276     model.c(ismember(model.rxns,'sink_lac-L(c)'))=1;
7277     if find(model.c)>0
7278         FBA = optimizeCbModel(model,'max');
7279         TestSolution(k,1) = FBA.f;
7280     else
7281         TestSolution(k,1) = NaN;
7282     end
7283     TestSolutionName{k,1} = 'fru(c) -> lac_L(c)/ fructose conversion to glucose & utilization';
7284     if ~isnan(TestSolution(k,1)); TestedRxns = [TestedRxns; model.rxns(find(abs(FBA.x)>tol))]; end ;k = k +1;clear FBA
7285     %% malcoa(c) -> eicostetcoa(c)/ fatty acid elongation
7286     model = modelOri;
7287     for i = 1 : length(RPMI_composition)
7288         model = changeRxnBounds(model,RPMI_composition{i},-1,'l');
7289     end
7290     model.c(find(model.c)) = 0;
7291     [model] = addSinkReactions(model,{'malcoa(c)','eicostetcoa(c)'},[-1 -1; 0 100]);
7292     model.c(ismember(model.rxns,'sink_eicostetcoa(c)'))=1;
7293     if find(model.c)>0
7294         FBA = optimizeCbModel(model,'max');
7295         TestSolution(k,1) = FBA.f;
7296     else
7297         TestSolution(k,1) = NaN;
7298     end
7299     TestSolutionName{k,1} = 'malcoa(c) -> eicostetcoa(c)/ fatty acid elongation (wtih RPMI medium)';
7300     if ~isnan(TestSolution(k,1)); TestedRxns = [TestedRxns; model.rxns(find(abs(FBA.x)>tol))]; end ;k = k +1;clear FBA
7301     %% accoa(c) -> chsterol(r)
7302     model = modelOri;
7303     model.c(find(model.c)) = 0;
7304     [model] = addSinkReactions(model,{'accoa(c)','chsterol(r)'},[-1 -1; 0 100]);
7305     model.c(ismember(model.rxns,'sink_chsterol(r)'))=1;
7306     if find(model.c)>0
7307         FBA = optimizeCbModel(model,'max');
7308         TestSolution(k,1) = FBA.f;
7309     else
7310         TestSolution(k,1) = NaN;
7311     end
7312     TestSolutionName{k,1} = 'accoa(c) -> chsterol(r)';
7313     if ~isnan(TestSolution(k,1)); TestedRxns = [TestedRxns; model.rxns(find(abs(FBA.x)>tol))]; end ;k = k +1;clear FBA
7314     %% inost(c) -> glac(r)
7315     model = modelOri;
7316     model.c(find(model.c)) = 0;
7317     [model] = addSinkReactions(model,{'inost(c)','glac(r)'},[-1 -1; 0 100]);
7318     model.c(ismember(model.rxns,'sink_glac(r)'))=1;
7319     if find(model.c)>0
7320         FBA = optimizeCbModel(model,'max');
7321         TestSolution(k,1) = FBA.f;
7322     else
7323         TestSolution(k,1) = NaN;
7324     end
7325     TestSolutionName{k,1} = 'inost(c) -> glac(r)';
7326     if ~isnan(TestSolution(k,1)); TestedRxns = [TestedRxns; model.rxns(find(abs(FBA.x)>tol))]; end ;k = k +1;clear FBA
7327     %% pail_hs(c) -> pail4p_hs(c)/ inositol kinase
7328     model = modelOri;
7329     model.c(find(model.c)) = 0;
7330     [model] = addSinkReactions(model,{'pail_hs(c)','pail4p_hs(c)'},[-1 -1; 0 100]);
7331     model.c(ismember(model.rxns,'sink_pail4p_hs(c)'))=1;
7332     if find(model.c)>0
7333         FBA = optimizeCbModel(model,'max');
7334         TestSolution(k,1) = FBA.f;
7335     else
7336         TestSolution(k,1) = NaN;
7337     end
7338     TestSolutionName{k,1} = 'pail_hs(c) -> pail4p_hs(c)/ inositol kinase';
7339     if ~isnan(TestSolution(k,1)); TestedRxns = [TestedRxns; model.rxns(find(abs(FBA.x)>tol))]; end ;k = k +1;clear FBA
7340     %% arachd(c) -> prostgh2(c)/ prostaglandin synthesis
7341     model = modelOri;
7342     model.c(find(model.c)) = 0;
7343     [model] = addSinkReactions(model,{'arachd(c)','prostgh2(c)'},[-1 -1; 0 100]);
7344     model.c(ismember(model.rxns,'sink_prostgh2(c)'))=1;
7345     if find(model.c)>0
7346         FBA = optimizeCbModel(model,'max');
7347         TestSolution(k,1) = FBA.f;
7348     else
7349         TestSolution(k,1) = NaN;
7350     end
7351     TestSolutionName{k,1} = 'arachd(c) -> prostgh2(c)/ prostaglandin synthesis';
7352     if ~isnan(TestSolution(k,1)); TestedRxns = [TestedRxns; model.rxns(find(abs(FBA.x)>tol))]; end ;k = k +1;clear FBA
7353     %% arachd(c) -> prostgd2(r)/ prostaglandin synthesis
7354     model = modelOri;
7355     model.c(find(model.c)) = 0;
7356     [model] = addSinkReactions(model,{'arachd(c)','prostgd2(r)'},[-1 -1; 0 100]);
7357     model.c(ismember(model.rxns,'sink_prostgd2(r)'))=1;
7358     if find(model.c)>0
7359         FBA = optimizeCbModel(model,'max');
7360         TestSolution(k,1) = FBA.f;
7361     else
7362         TestSolution(k,1) = NaN;
7363     end
7364     TestSolutionName{k,1} = 'arachd(c) -> prostgd2(r)/ prostaglandin synthesis';
7365     if ~isnan(TestSolution(k,1)); TestedRxns = [TestedRxns; model.rxns(find(abs(FBA.x)>tol))]; end ;k = k +1;clear FBA
7366     %% arachd(c) -> prostge2(r)/ prostaglandin synthesis
7367     model = modelOri;
7368     model.c(find(model.c)) = 0;
7369     [model] = addSinkReactions(model,{'arachd(c)','prostge2(r)'},[-1 -1; 0 100]);
7370     model.c(ismember(model.rxns,'sink_prostge2(r)'))=1;
7371     if find(model.c)>0
7372         FBA = optimizeCbModel(model,'max');
7373         TestSolution(k,1) = FBA.f;
7374     else
7375         TestSolution(k,1) = NaN;
7376     end
7377     TestSolutionName{k,1} = 'arachd(c) -> prostge2(r)/ prostaglandin synthesis';
7378     if ~isnan(TestSolution(k,1)); TestedRxns = [TestedRxns; model.rxns(find(abs(FBA.x)>tol))]; end ;k = k +1;clear FBA
7379     %% arachd(c) -> prostgi2(r)/ prostaglandin synthesis
7380     model = modelOri;
7381     model.c(find(model.c)) = 0;
7382     [model] = addSinkReactions(model,{'arachd(c)','prostgi2(r)'},[-1 -1; 0 100]);
7383     model.c(ismember(model.rxns,'sink_prostgi2(r)'))=1;
7384     if find(model.c)>0
7385         FBA = optimizeCbModel(model,'max');
7386         TestSolution(k,1) = FBA.f;
7387     else
7388         TestSolution(k,1) = NaN;
7389     end
7390     TestSolutionName{k,1} = 'arachd(c) -> prostgi2(r)/ prostaglandin synthesis';
7391     if ~isnan(TestSolution(k,1)); TestedRxns = [TestedRxns; model.rxns(find(abs(FBA.x)>tol))]; end ;k = k +1;clear FBA
7392     %% 25hvitd3(m) -> 2425dhvitd3(m)/ 24,25-dihydroxycalciol synthesis
7393     model = modelOri;
7394     model.c(find(model.c)) = 0;
7395     for i = 1 : length(RPMI_composition)
7396         model = changeRxnBounds(model,RPMI_composition{i},-1,'l');
7397     end
7398     model.lb(ismember(model.rxns,'EX_o2(e)'))=-40;model.ub(ismember(model.rxns,'EX_o2(e)'))=0;
7399     [model] = addSinkReactions(model,{'25hvitd3(m)','2425dhvitd3(m)'},[-1 -1; 0 100]);
7400     model.c(ismember(model.rxns,'sink_2425dhvitd3(m)'))=1;
7401     if find(model.c)>0
7402         FBA = optimizeCbModel(model,'max');
7403         TestSolution(k,1) = FBA.f;
7404     else
7405         TestSolution(k,1) = NaN;
7406     end
7407     TestSolutionName{k,1} = '25hvitd3(m) -> 2425dhvitd3(m)/ 24,25-dihydroxycalciol synthesis (with RPMI medium)';
7408     if ~isnan(TestSolution(k,1)); TestedRxns = [TestedRxns; model.rxns(find(abs(FBA.x)>tol))]; end ;k = k +1;clear FBA
7409     %% caro(c) -> retinal(c)/ vitamin A synthesis
7410     model = modelOri;
7411     model.c(find(model.c)) = 0;
7412     [model] = addSinkReactions(model,{'caro(c)','retinal(c)'},[-1 -1; 0 100]);
7413     model.c(ismember(model.rxns,'sink_retinal(c)'))=1;
7414     if find(model.c)>0
7415         FBA = optimizeCbModel(model,'max');
7416         TestSolution(k,1) = FBA.f;
7417     else
7418         TestSolution(k,1) = NaN;
7419     end
7420     TestSolutionName{k,1} = 'caro(c) -> retinal(c)/ vitamin A synthesis';
7421     if ~isnan(TestSolution(k,1)); TestedRxns = [TestedRxns; model.rxns(find(abs(FBA.x)>tol))]; end ;k = k +1;clear FBA
7422     
7423     %% missing part starts
7424     %% synthesis of glutamate from ornithine
7425     %% synthesis of glutamate from ornithine
7426     model = modelOri;
7427     model.c(find(model.c)) = 0;
7428     model.lb(ismember(model.rxns,'EX_orn(e)'))=-1;model.ub(ismember(model.rxns,'EX_orn(e)'))=-1;
7429     [model] = addDemandReaction(model,'glu-L(c)');
7430      model.c(ismember(model.rxns,'sink_retinal(c)'))=1;
7431     if find(model.c)>0
7432         FBA = optimizeCbModel(model,'max');
7433         TestSolution(k,1) = FBA.f;
7434     else
7435         TestSolution(k,1) = NaN;
7436     end
7437     TestSolutionName{k,1} = 'synthesis of glutamate from ornithine';
7438     if ~isnan(TestSolution(k,1)); TestedRxns = [TestedRxns; model.rxns(find(abs(FBA.x)>tol))]; end ;k = k +1;clear FBA
7439     %% synthesis of proline from ornithine
7440     model = modelOri;
7441     model.c(find(model.c)) = 0;
7442     model.lb(ismember(model.rxns,'EX_orn(e)'))=-1;model.ub(ismember(model.rxns,'EX_orn(e)'))=-1;
7443     [model] = addDemandReaction(model,'pro-L(m)');
7444     model.c(ismember(model.rxns,'DM_pro-L(m)'))=1;
7445     FBA = optimizeCbModel(model,'max');
7446     TestSolution(k,1) = FBA.f;
7447     TestSolutionName{k,1} = 'synthesis of proline from ornithine';
7448     if ~isnan(TestSolution(k,1)); TestedRxns = [TestedRxns; model.rxns(find(abs(FBA.x)>tol))]; end ;k = k +1;clear FBA
7449     %% visual cycle in retina
7450     model = modelOri;
7451     model.c(find(model.c)) = 0;
7452     [model] = addSinkReactions(model,{'retinol-cis-11(c)','retinal(c)'},[-1 -1; 0 1000]);
7453     model.c(ismember(model.rxns,'sink_retinal(c)'))=1;
7454     FBA = optimizeCbModel(model,'max');
7455     TestSolution(k,1) = FBA.f;
7456     TestSolutionName{k,1} = 'visual cycle in retina';
7457     if ~isnan(TestSolution(k,1)); TestedRxns = [TestedRxns; model.rxns(find(abs(FBA.x)>tol))]; end ;k = k +1;clear FBA
7458     %% pail_hs(c) -> pchol_hs(c)
7459     model = modelOri;
7460     model.c(find(model.c)) = 0;
7461     [model] = addSinkReactions(model,{'pail_hs(c)','pchol-hs(c)'},[-1 -1; 0 1000]);
7462     model.c(ismember(model.rxns,'sink_pchol-hs(c)'))=1;
7463     FBA = optimizeCbModel(model,'max');
7464     TestSolution(k,1) = FBA.f;
7465     TestSolutionName{k,1} = 'pail_hs(c) -> pchol_hs(c)';
7466     if ~isnan(TestSolution(k,1)); TestedRxns = [TestedRxns; model.rxns(find(abs(FBA.x)>tol))]; end ;k = k +1;clear FBA
7467     %% pail_hs(c) -> pe_hs(c)
7468     model = modelOri;
7469     model.c(find(model.c)) = 0;
7470     [model] = addSinkReactions(model,{'pail_hs(c)','pe_hs(c)'},[-1 -1; 0 1000]);
7471     model.c(ismember(model.rxns,'sink_pe_hs(c)'))=1;
7472     FBA = optimizeCbModel(model,'max');
7473     TestSolution(k,1) = FBA.f;
7474     TestSolutionName{k,1} = 'pail_hs(c) -> pe_hs(c)';
7475     if ~isnan(TestSolution(k,1)); TestedRxns = [TestedRxns; model.rxns(find(abs(FBA.x)>tol))]; end ;k = k +1;clear FBA
7476     %% pail_hs(c) -> ps_hs(c)
7477     model = modelOri;
7478     model.c(find(model.c)) = 0;
7479     [model] = addSinkReactions(model,{'pail_hs(c)','ps-hs(c)'},[-1 -1; 0 1000]);
7480     model.c(ismember(model.rxns,'sink_ps-hs(c)'))=1;
7481     FBA = optimizeCbModel(model,'max');
7482     TestSolution(k,1) = FBA.f;
7483     TestSolutionName{k,1} = 'pail_hs(c) -> ps_hs(c)';
7484     if ~isnan(TestSolution(k,1)); TestedRxns = [TestedRxns; model.rxns(find(abs(FBA.x)>tol))]; end ;k = k +1;clear FBA
7485     %% pail_hs(c) -> g3pc(c)
7486     model = modelOri;
7487     model.c(find(model.c)) = 0;
7488     [model] = addSinkReactions(model,{'pail_hs(c)','g3pc(c)'},[-1 -1; 0 1000]);
7489     model.c(ismember(model.rxns,'sink_g3pc(c)'))=1;
7490     FBA = optimizeCbModel(model,'max');
7491     TestSolution(k,1) = FBA.f;
7492     TestSolutionName{k,1} = 'pail_hs(c) -> g3pc(c)';
7493     if ~isnan(TestSolution(k,1)); TestedRxns = [TestedRxns; model.rxns(find(abs(FBA.x)>tol))]; end ;k = k +1;clear FBA
7494     %% dag_hs(c) -> pchol_hs(c)
7495     model = modelOri;
7496     model.c(find(model.c)) = 0;
7497     [model] = addSinkReactions(model,{'dag_hs(c)','pchol-hs(c)'},[-1 -1; 0 1000]);
7498     model.c(ismember(model.rxns,'sink_pchol-hs(c)'))=1;
7499     FBA = optimizeCbModel(model,'max');
7500     TestSolution(k,1) = FBA.f;
7501     TestSolutionName{k,1} = 'dag_hs(c) -> pchol_hs(c)';
7502     if ~isnan(TestSolution(k,1)); TestedRxns = [TestedRxns; model.rxns(find(abs(FBA.x)>tol))]; end ;k = k +1;clear FBA
7503     %% dag_hs(c) -> pe_hs(c)
7504     model = modelOri;
7505     model.c(find(model.c)) = 0;
7506     [model] = addSinkReactions(model,{'dag_hs(c)','pe_hs(c)'},[-1 -1; 0 1000]);
7507     model.c(ismember(model.rxns,'sink_pe_hs(c)'))=1;
7508     FBA = optimizeCbModel(model,'max');
7509     TestSolution(k,1) = FBA.f;
7510     TestSolutionName{k,1} = 'dag_hs(c) -> pe_hs(c)';
7511     if ~isnan(TestSolution(k,1)); TestedRxns = [TestedRxns; model.rxns(find(abs(FBA.x)>tol))]; end ;k = k +1;clear FBA
7512     %% dag_hs(c) -> clpn_hs(c)
7513     model = modelOri;
7514     model.c(find(model.c)) = 0;
7515     [model] = addSinkReactions(model,{'dag_hs(c)','clpn-hs(c)'},[-1 -1; 0 1000]);
7516     model.c(ismember(model.rxns,'sink_clpn-hs(c)'))=1;
7517     FBA = optimizeCbModel(model,'max');
7518     TestSolution(k,1) = FBA.f;
7519     TestSolutionName{k,1} = 'dag_hs(c) -> clpn_hs(c)';
7520     if ~isnan(TestSolution(k,1)); TestedRxns = [TestedRxns; model.rxns(find(abs(FBA.x)>tol))]; end ;k = k +1;clear FBA
7521     %% dag_hs(c) -> pgp_hs(c)
7522     model = modelOri;
7523     model.c(find(model.c)) = 0;
7524     [model] = addSinkReactions(model,{'dag_hs(c)','pgp-hs(c)'},[-1 -1; 0 1000]);
7525     model.c(ismember(model.rxns,'sink_pgp-hs(c)'))=1;
7526     FBA = optimizeCbModel(model,'max');
7527     TestSolution(k,1) = FBA.f;
7528     TestSolutionName{k,1} = 'dag_hs(c) -> pgp_hs(c)';
7529     if ~isnan(TestSolution(k,1)); TestedRxns = [TestedRxns; model.rxns(find(abs(FBA.x)>tol))]; end ;k = k +1;clear FBA
7530     %% bhb(m) -> acac(m)/ ketone body utilization
7531     model = modelOri;
7532     model.c(find(model.c)) = 0;
7533     [model] = addSinkReactions(model,{'bhb(m)','acac(m)'},[-1 -1; 0 1000]);
7534     model.c(ismember(model.rxns,'sink_acac(m)'))=1;
7535     FBA = optimizeCbModel(model,'max');
7536     TestSolution(k,1) = FBA.f;
7537     TestSolutionName{k,1} = 'bhb(m) -> acac(m)';
7538     if ~isnan(TestSolution(k,1)); TestedRxns = [TestedRxns; model.rxns(find(abs(FBA.x)>tol))]; end ;k = k +1;clear FBA
7539     %% mal_m(m) -> pyr(m)/ malic enzyme
7540     model = modelOri;
7541     model.c(find(model.c)) = 0;
7542     [model] = addSinkReactions(model,{'mal-L(m)','pyr(m)'},[-1 -1; 0 1000]);
7543     model.c(ismember(model.rxns,'sink_pyr(m)'))=1;
7544     FBA = optimizeCbModel(model,'max');
7545     TestSolution(k,1) = FBA.f;
7546     TestSolutionName{k,1} = 'mal_L(m) -> pyr(m)';
7547     if ~isnan(TestSolution(k,1)); TestedRxns = [TestedRxns; model.rxns(find(abs(FBA.x)>tol))]; end ;k = k +1;clear FBA
7548     %% glu_L(c) -> gln_L(c)/ glutamine synthase
7549     model = modelOri;
7550     model.c(find(model.c)) = 0;
7551     [model] = addSinkReactions(model,{'glu-L(c)','gln-L(c)'},[-1 -1; 0 1000]);
7552     model.c(ismember(model.rxns,'sink_gln-L(c)'))=1;
7553     FBA = optimizeCbModel(model,'max');
7554     TestSolution(k,1) = FBA.f;
7555     TestSolutionName{k,1} = 'glu_L(c) -> gln_L(c)';
7556     if ~isnan(TestSolution(k,1)); TestedRxns = [TestedRxns; model.rxns(find(abs(FBA.x)>tol))]; end ;k = k +1;clear FBA
7557     %% cys_L(c) -> coa(c)/ CoA synthesis from cysteine
7558     model = modelOri;
7559     model.c(find(model.c)) = 0;
7560     [model] = addSinkReactions(model,{'cys-L(c)','coa(c)'},[-1 -1; 0 100]);
7561     model.lb(find(ismember(model.rxns,'sink_cys-L(c)')))=-1;
7562     model.ub(find(ismember(model.rxns,'sink_cys-L(c)')))=-1;
7563     model.lb(find(ismember(model.rxns,'sink_cys_L(c)')))=-1;
7564     model.ub(find(ismember(model.rxns,'sink_cys_L(c)')))=-1;
7565     model.c(ismember(model.rxns,'DPCOAK'))=1;
7566     FBA = optimizeCbModel(model,'max');
7567     TestSolution(k,1) = FBA.f;
7568     TestSolutionName{k,1} = 'cys_L(c) -> coa(c)';
7569     if ~isnan(TestSolution(k,1)); TestedRxns = [TestedRxns; model.rxns(find(abs(FBA.x)>tol))]; end ;k = k +1;clear FBA
7570     %% occoa(m) -> accoa(m)/ octanoate oxidation
7571     model = modelOri;
7572     model.c(find(model.c)) = 0;
7573     [model] = addSinkReactions(model,{'occoa(m)','accoa(m)'},[-1 -1; 0 1000]);
7574     model.c(ismember(model.rxns,'sink_accoa(m)'))=1;
7575     FBA = optimizeCbModel(model,'max');
7576     TestSolution(k,1) = FBA.f;
7577     TestSolutionName{k,1} = 'occoa(m) -> accoa(m)';
7578     if ~isnan(TestSolution(k,1)); TestedRxns = [TestedRxns; model.rxns(find(abs(FBA.x)>tol))]; end ;k = k +1;clear FBA
7579     %% lnlncgcoa(c) -> dlnlcgcoa(c)/ fatty acid elongation
7580     model = modelOri;
7581     model.c(find(model.c)) = 0;
7582     [model] = addSinkReactions(model,{'lnlncgcoa(c)','dlnlcgcoa(c)'},[-1 -1; 0 1000]);
7583     model.c(ismember(model.rxns,'sink_dlnlcgcoa(c)'))=1;
7584     FBA = optimizeCbModel(model,'max');
7585     TestSolution(k,1) = FBA.f;
7586     TestSolutionName{k,1} = 'lnlncgcoa(c) -> dlnlcgcoa(c)';
7587     if ~isnan(TestSolution(k,1)); TestedRxns = [TestedRxns; model.rxns(find(abs(FBA.x)>tol))]; end ;k = k +1;clear FBA
7588     %% chol(c) -> ach(c)/ acetyl-choline synthesis in brain
7589     model = modelOri;
7590     model.c(find(model.c)) = 0;
7591     [model] = addSinkReactions(model,{'chol(c)','ach(c)'},[-1 -1; 0 1000]);
7592     model.c(ismember(model.rxns,'sink_ach(c)'))=1;
7593     FBA = optimizeCbModel(model,'max');
7594     TestSolution(k,1) = FBA.f;
7595     TestSolutionName{k,1} = 'chol(c) -> ach(c)';
7596     if ~isnan(TestSolution(k,1)); TestedRxns = [TestedRxns; model.rxns(find(abs(FBA.x)>tol))]; end ;k = k +1;clear FBA
7597     %% pyr(m) -> oaa(m)/ pyruvate carboxylase
7598     model = modelOri;
7599     model.c(find(model.c)) = 0;
7600     [model] = addSinkReactions(model,{'pyr(m)','oaa(m)'},[-1 -1; 0 1000]);
7601     model.c(ismember(model.rxns,'sink_oaa(m)'))=1;
7602     FBA = optimizeCbModel(model,'max');
7603     TestSolution(k,1) = FBA.f;
7604     TestSolutionName{k,1} = 'pyr(m) -> oaa(m)';
7605     if ~isnan(TestSolution(k,1)); TestedRxns = [TestedRxns; model.rxns(find(abs(FBA.x)>tol))]; end ;k = k +1;clear FBA
7606     %% GABA aminotransferase
7607     model = modelOri;
7608     model.c(find(model.c)) = 0;
7609     model.lb(ismember(model.rxns,'EX_glu-L(e)'))=-1;model.ub(ismember(model.rxns,'EX_glu-L(e)'))=-1;
7610     model.lb(ismember(model.rxns,'EX_o2(e)'))=-40;model.ub(ismember(model.rxns,'EX_o2(e)'))=-1;
7611     model.c(ismember(model.rxns,'ABTArm'))=1;
7612     FBA = optimizeCbModel(model,'max');
7613     TestSolution(k,1) = FBA.f;
7614     TestSolutionName{k,1} = 'GABA aminotransferase';
7615     if ~isnan(TestSolution(k,1)); TestedRxns = [TestedRxns; model.rxns(find(abs(FBA.x)>tol))]; end ;k = k +1;clear FBA
7616     %% methionine adenosyltransferase
7617     model = modelOri;
7618     model.c(find(model.c)) = 0;
7619     model.lb(ismember(model.rxns,'EX_met_L(e)'))=-1;model.ub(ismember(model.rxns,'EX_met_L(e)'))=-1;
7620     model.lb(ismember(model.rxns,'EX_o2(e)'))=-40;model.ub(ismember(model.rxns,'EX_o2(e)'))=-1;
7621     model.c(ismember(model.rxns,'METAT'))=1;
7622     FBA = optimizeCbModel(model,'max');
7623     TestSolution(k,1) = FBA.f;
7624     TestSolutionName{k,1} = 'methionine adenosyltransferase';
7625     if ~isnan(TestSolution(k,1)); TestedRxns = [TestedRxns; model.rxns(find(abs(FBA.x)>tol))]; end ;k = k +1;clear FBA
7626     %% creatine synthesis
7627     model = modelOri;
7628     model.c(find(model.c)) = 0;
7629     model.lb(ismember(model.rxns,'EX_arg_L(e)'))=-1;model.ub(ismember(model.rxns,'EX_arg_L(e)'))=-1;
7630     model.lb(ismember(model.rxns,'EX_gly(e)'))=-1;model.ub(ismember(model.rxns,'EX_gly(e)'))=-1;
7631     [model] = addSinkReactions(model,{'crtn(c)'},[0 1000]);
7632     model.c(ismember(model.rxns,'sink_crtn(c)'))=1;
7633     FBA = optimizeCbModel(model,'max');
7634     TestSolution(k,1) = FBA.f;
7635     TestSolutionName{k,1} = 'creatine synthesis';
7636     if ~isnan(TestSolution(k,1)); TestedRxns = [TestedRxns; model.rxns(find(abs(FBA.x)>tol))]; end ;k = k +1;clear FBA
7637     %% arachd(c) -> leuktrE4(c)/ leukotriene synthesis
7638     % requires multiple medium compounds --> RPMI
7639     
7640     model = modelOri;
7641     for i = 1 : length(RPMI_composition)
7642         model = changeRxnBounds(model,RPMI_composition{i},-1,'l');
7643     end
7644     model.c(find(model.c)) = 0;
7645     [model] = addSinkReactions(model,{'arachd(c)','leuktrE4(c)'},[-1 -1; 0 1000]);
7646     model.c(ismember(model.rxns,'sink_leuktrE4(c)'))=1;
7647     FBA = optimizeCbModel(model,'max');
7648     TestSolution(k,1) = FBA.f;
7649     TestSolutionName{k,1} = 'arachd(c) -> leuktrE4(c) (with RPMI medium)';
7650     if ~isnan(TestSolution(k,1)); TestedRxns = [TestedRxns; model.rxns(find(abs(FBA.x)>tol))]; end ;k = k +1;clear FBA
7651     %% arachd(c) -> C06314(c)/ lipoxin synthesis
7652     model = modelOri;
7653     for i = 1 : length(RPMI_composition)
7654         model = changeRxnBounds(model,RPMI_composition{i},-1,'l');
7655     end
7656     model.c(find(model.c)) = 0;
7657     [model] = addSinkReactions(model,{'arachd(c)','C06314(c)'},[-1 -1; 0 1000]);
7658     model.c(ismember(model.rxns,'sink_C06314(c)'))=1;
7659     FBA = optimizeCbModel(model,'max');
7660     TestSolution(k,1) = FBA.f;
7661     TestSolutionName{k,1} = 'arachd(c) -> C06314(c) (with RPMI medium)';
7662     if ~isnan(TestSolution(k,1)); TestedRxns = [TestedRxns; model.rxns(find(abs(FBA.x)>tol))]; end ;k = k +1;clear FBA
7663     %% nrpphr(c) -> 3mox4hoxm(c)/ degradation of norepinephrine
7664     model = modelOri;
7665     for i = 1 : length(RPMI_composition)
7666         model = changeRxnBounds(model,RPMI_composition{i},-1,'l');
7667     end
7668     model.c(find(model.c)) = 0;
7669     [model] = addSinkReactions(model,{'nrpphr(c)','3mox4hoxm(c)'},[-1 -1; 0 1000]);
7670     model.c(ismember(model.rxns,'sink_3mox4hoxm(c)'))=1;
7671     FBA = optimizeCbModel(model,'max');
7672     TestSolution(k,1) = FBA.f;
7673     TestSolutionName{k,1} = 'nrpphr(c) -> 3mox4hoxm(c) (with RPMI medium)';
7674     if ~isnan(TestSolution(k,1)); TestedRxns = [TestedRxns; model.rxns(find(abs(FBA.x)>tol))]; end ;k = k +1;clear FBA
7675     %% sbt_D(c) -> fru(c)/sorbitol pathway
7676     model = modelOri;
7677     model.c(find(model.c)) = 0;
7678     [model] = addSinkReactions(model,{'sbt-D(c)','fru(c)'},[-1 -1; 0 1000]);
7679     model.c(ismember(model.rxns,'sink_fru(c)'))=1;
7680     FBA = optimizeCbModel(model,'max');
7681     TestSolution(k,1) = FBA.f;
7682     TestSolutionName{k,1} = 'sbt_D(c) -> fru(c)/sorbitol pathway';
7683     if ~isnan(TestSolution(k,1)); TestedRxns = [TestedRxns; model.rxns(find(abs(FBA.x)>tol))]; end ;k = k +1;clear FBA
7684     
7685     %% new addition 26.04.2017
7686     model = modelOri;
7687     model.c(find(model.c)) = 0;
7688     model.lb(ismember(model.rxns,'EX_glc(e)'))=-1;model.ub(ismember(model.rxns,'EX_glc(e)'))=-1;
7689     model.lb(ismember(model.rxns,'EX_o2(e)'))=-40;model.ub(ismember(model.rxns,'EX_o2(e)'))=-1;
7690     [model] = addSinkReactions(model,{'accoa(m)'},0,  1000);
7691     model.lb(find(ismember(model.rxns,'sink_coa(c)')))=-1;
7692     model.ub(find(ismember(model.rxns,'sink_coa(c)')))=1;
7693     model.c(ismember(model.rxns,'sink_accoa(m)'))=1;
7694     FBA = optimizeCbModel(model,'max');
7695     TestSolution(k,1) = FBA.f;
7696     TestSolutionName{k,1} = 'Mitochondrial accoa de novo synthesis from glc';
7697     if ~isnan(TestSolution(k,1)); TestedRxns = [TestedRxns; model.rxns(find(abs(FBA.x)>tol))]; end ;k = k +1;clear FBA
7698     
7699     model = modelOri;
7700     model.c(find(model.c)) = 0;
7701     model.lb(ismember(model.rxns,'EX_glc(e)'))=-1;model.ub(ismember(model.rxns,'EX_glc(e)'))=-1;
7702     model.lb(ismember(model.rxns,'EX_o2(e)'))=-40;model.ub(ismember(model.rxns,'EX_o2(e)'))=-1;
7703     [model] = addSinkReactions(model,{'succoa(m)'},0,1000);
7704     model.c(ismember(model.rxns,'sink_succoa(m)'))=1;
7705     model.lb(find(ismember(model.rxns,'sink_coa(c)')))=-1;
7706     model.ub(find(ismember(model.rxns,'sink_coa(c)')))=1;
7707     FBA = optimizeCbModel(model,'max');
7708     TestSolution(k,1) = FBA.f;
7709     TestSolutionName{k,1} = 'Mitochondrial succoa de novo synthesis from glc';
7710     if ~isnan(TestSolution(k,1)); TestedRxns = [TestedRxns; model.rxns(find(abs(FBA.x)>tol))]; end ;k = k +1;clear FBA
7711 end
7712 TestSolution(find(abs(TestSolution)<tol))=0;
7713 TestSolutionName(:,2) = num2cell(TestSolution);
7714 TestedRxns = unique(TestedRxns);
7715 TestedRxns = intersect(modelOri.rxns,TestedRxns); % only those reactions that are also in modelOri not those that have been added to the network
7716 PercTestedRxns = length(TestedRxns)*100/length(modelOri.rxns);
7717 if saveDiary
7718     diary off
7719 end
```

---

Generated on Thu 14-May-2020 13:05:49 by **m2html** © 2005
