# Supplementary material for: Personalized whole‐body models integrate metabolism, physiology, and the gut microbiome
Source: Mol Syst Biol. 2020 May 28;16(5):e8982. doi: 10.15252/msb.20198982 (PMC7285886; doi:10.15252/msb.20198982)
Supplement: Supplementary file 22 — Dataset EV1 [file MSB-16-e8982-s022.zip › PSCM_toolbox/PSCM_toolbox_doc/src/convertATPflux2StepNumer.html]

Description of convertATPflux2StepNumer


# convertATPflux2StepNumer

## PURPOSE

**This function converts an ATP hydrolysis flux (e.g., Muscle\_DM\_atp\_c\_**

## SYNOPSIS

**function [Energy\_kJ,Energy\_kcal,Meter,StepNumber] = convertATPflux2StepNumber(ATP\_hydrolysis\_flux, sex, weight, height)**

## DESCRIPTION

```
 This function converts an ATP hydrolysis flux (e.g., Muscle_DM_atp_c_
 into distance walked and step number). See below for assumptions and
 calculation details

 function [Energy_kJ,Energy_kcal,Meter,StepNumber] = convertATPflux2StepNumber(ATP_hydrolysis_flux, sex, weight, height)
 INPUT
 ATP_hydrolysis_flux   Flux value through the Muscle_DM_atp_c_ reaction
 sex                'male' or 'female'
 weight                in kg
 height                in cm

 OUTPUT
 Energy_kJ             Energy value in kJ corresponding to the Flux value through the Muscle_DM_atp_c_ reaction
 Energy_kcal           Energy value in kcal corresponding to the Flux value through the Muscle_DM_atp_c_ reaction
 Meter                 Corresponding meter of walking that can be achieved
 StepNumber            Corresponding step number that can be achieved
 
 Ines Thiele 01/2018
```

## CROSS-REFERENCE INFORMATION

This function calls:


This function is called by:

## SOURCE CODE

```
0001 function [Energy_kJ,Energy_kcal,Meter,StepNumber] = convertATPflux2StepNumber(ATP_hydrolysis_flux, sex, weight, height)
0002 % This function converts an ATP hydrolysis flux (e.g., Muscle_DM_atp_c_
0003 % into distance walked and step number). See below for assumptions and
0004 % calculation details
0005 %
0006 % function [Energy_kJ,Energy_kcal,Meter,StepNumber] = convertATPflux2StepNumber(ATP_hydrolysis_flux, sex, weight, height)
0007 % INPUT
0008 % ATP_hydrolysis_flux   Flux value through the Muscle_DM_atp_c_ reaction
0009 % sex                'male' or 'female'
0010 % weight                in kg
0011 % height                in cm
0012 %
0013 % OUTPUT
0014 % Energy_kJ             Energy value in kJ corresponding to the Flux value through the Muscle_DM_atp_c_ reaction
0015 % Energy_kcal           Energy value in kcal corresponding to the Flux value through the Muscle_DM_atp_c_ reaction
0016 % Meter                 Corresponding meter of walking that can be achieved
0017 % StepNumber            Corresponding step number that can be achieved
0018 %
0019 % Ines Thiele 01/2018
0020 
0021 % energy cost of walking (1 step)
0022 % gross energy cost of 3 J/kg/m - taken from https://www.ncbi.nlm.nih.gov/pmc/articles/PMC4879834/
0023 %
0024 % ATP_hydrolysis_flux in mmol/person/day
0025 %
0026 % calculation
0027 % 1 mol ATP = 64KJ based on
0028 % http://www.physiology.org/doi/pdf/10.1152/jappl.1998.85.6.2140, Table 1
0029 % in resting human muscle
0030 Energy_kJ = (ATP_hydrolysis_flux/1000) * 64; %in kJ per person per day
0031 % 1 kJ = 0.239006 kcal
0032 Energy_kcal = Energy_kJ*0.239006; %in kcal per person per day
0033 % 1 meter = 3 J/kg
0034 Meter = Energy_kJ*1000/(3*weight); % per person per day
0035 % stride length - http://didyouknowstuff.blogspot.lu/p/how-many-kilometers-in-10000-steps.html
0036 % Stride length can be measured or calculated:
0037 % Women .413 * height in cm
0038 % Men .415 * height in cm
0039 if strcmp(sex,'male')
0040     Stride = 0.415 * height; %in cm
0041 else
0042     Stride = 0.413 * height; %in cm
0043 end
0044     
0045 StepNumber = Meter/(Stride/100);
0046
```

---

Generated on Thu 14-May-2020 13:05:49 by **m2html** © 2005
