# Supplementary material for: Personalized whole‐body models integrate metabolism, physiology, and the gut microbiome
Source: Mol Syst Biol. 2020 May 28;16(5):e8982. doi: 10.15252/msb.20198982 (PMC7285886; doi:10.15252/msb.20198982)
Supplement: Supplementary file 22 — Dataset EV1 [file MSB-16-e8982-s022.zip › PSCM_toolbox/PSCM_toolbox_doc/src/getStatsOrganComp.html]

Description of getStatsOrganComp


# getStatsOrganComp

## PURPOSE

**This function compiles general statistics on the male and the female**

## SYNOPSIS

**function [TableProp\_female,TableProp\_male, TableGRM,TableMetsNum\_female,TableMetsNum\_male,TableGenes\_femaleNum] = getStatsOrganComp(female, male, OrganCompendium\_female, OrganCompendium\_male, violinPlots)**

## DESCRIPTION

```
 This function compiles general statistics on the male and the female
 organ compendia derived from the male and female whole-body metabolic
 models.

 [TableProp_female,TableProp_male, TableGRM] = getStatsOrganComp(female, male, OrganCompendium_female, OrganCompendium_male, violinPlots)

 INPUT
 female                    model structure, female whole-body metabolic
                           model
 male                      model structure, male whole-body metabolic
                           model
 OrganCompendium_female    strucutre containing the different organs
                           (generated with the function getOrgansFromHarvey
 OrganCompendium_male      strucutre containing the different organs
                           (generated with the function getOrgansFromHarvey
 violinPlots               plot violin plots (does not work below Matlab
                           2016) (defaul = 0)

 OUTPUT
 TableProp_female          Table containing organ-specific information
 TableProp_male            Table containing organ-specific information

 A more comprehensive comparison output is provided in the file:
 Results_StatsOrganComp.mat that is created at the end of this function.

 Ines Thiele, 2017
```

## CROSS-REFERENCE INFORMATION

This function calls:


This function is called by:

## SUBFUNCTIONS

- function genesPerOrganFigure(data1, data2)

## SOURCE CODE

```
0001 function [TableProp_female,TableProp_male, TableGRM,TableMetsNum_female,TableMetsNum_male,TableGenes_femaleNum] = getStatsOrganComp(female, male, OrganCompendium_female, OrganCompendium_male, violinPlots)
0002 % This function compiles general statistics on the male and the female
0003 % organ compendia derived from the male and female whole-body metabolic
0004 % models.
0005 %
0006 % [TableProp_female,TableProp_male, TableGRM] = getStatsOrganComp(female, male, OrganCompendium_female, OrganCompendium_male, violinPlots)
0007 %
0008 % INPUT
0009 % female                    model structure, female whole-body metabolic
0010 %                           model
0011 % male                      model structure, male whole-body metabolic
0012 %                           model
0013 % OrganCompendium_female    strucutre containing the different organs
0014 %                           (generated with the function getOrgansFromHarvey
0015 % OrganCompendium_male      strucutre containing the different organs
0016 %                           (generated with the function getOrgansFromHarvey
0017 % violinPlots               plot violin plots (does not work below Matlab
0018 %                           2016) (defaul = 0)
0019 %
0020 % OUTPUT
0021 % TableProp_female          Table containing organ-specific information
0022 % TableProp_male            Table containing organ-specific information
0023 %
0024 % A more comprehensive comparison output is provided in the file:
0025 % Results_StatsOrganComp.mat that is created at the end of this function.
0026 %
0027 % Ines Thiele, 2017
0028 
0029 global resultsPath
0030 resultsPath = which('MethodSection3.mlx');
0031 resultsPath = strrep(resultsPath,'MethodSection3.mlx',['Results' filesep]);
0032 
0033 if  ~exist('violinPlots','var')
0034     violinPlots = 0;
0035 end
0036 
0037 % load Recon 3D* used for Harvey
0038 load('Recon3D_Harvey_Used_in_Script_120502.mat')
0039 Recon3DHarvey = modelConsistent;
0040 
0041 % female
0042 BC_mets = (find(~cellfun(@isempty,strfind(female.mets,'[bc]'))));
0043 SL = (find(~cellfun(@isempty,strfind(female.mets,'slack_'))));
0044 SL_female = SL;
0045 BC_mets_female = setdiff(BC_mets,SL);
0046 U_mets = (find(~cellfun(@isempty,strfind(female.mets,'[u]'))));
0047 U_mets_female = setdiff(U_mets,SL);
0048 BP_mets = (find(~cellfun(@isempty,strfind(female.mets,'[bp]'))));
0049 BP_mets_female = setdiff(BP_mets,SL);
0050 BD_mets = (find(~cellfun(@isempty,strfind(female.mets,'[bd]'))));
0051 BD_mets_female = setdiff(BD_mets,SL);
0052 CSF_mets = (find(~cellfun(@isempty,strfind(female.mets,'[csf]'))));
0053 CSF_mets_female = setdiff(CSF_mets,SL);
0054 D_mets = (find(~cellfun(@isempty,strfind(female.mets,'[d]'))));
0055 D_mets_female = setdiff(D_mets,SL);
0056 FE_mets = (find(~cellfun(@isempty,strfind(female.mets,'[fe]'))));
0057 FE_mets_female = setdiff(FE_mets,SL);
0058 SW_mets = (find(~cellfun(@isempty,strfind(female.mets,'[sw]'))));
0059 SW_mets_female = setdiff(SW_mets,SL);
0060 A_mets = (find(~cellfun(@isempty,strfind(female.mets,'[a]'))));
0061 A_mets_female = setdiff(A_mets,SL);
0062 M_mets = (find(~cellfun(@isempty,strfind(female.mets,'[mi]'))));
0063 M_mets_female = setdiff(M_mets,SL);
0064 
0065 % male
0066 BC_mets = (find(~cellfun(@isempty,strfind(male.mets,'[bc]'))));
0067 SL = (find(~cellfun(@isempty,strfind(male.mets,'slack_'))));
0068 SL_male = SL;
0069 BC_mets_male = setdiff(BC_mets,SL);
0070 U_mets = (find(~cellfun(@isempty,strfind(male.mets,'[u]'))));
0071 U_mets_male = setdiff(U_mets,SL);
0072 BP_mets = (find(~cellfun(@isempty,strfind(male.mets,'[bp]'))));
0073 BP_mets_male = setdiff(BP_mets,SL);
0074 BD_mets = (find(~cellfun(@isempty,strfind(male.mets,'[bd]'))));
0075 BD_mets_male = setdiff(BD_mets,SL);
0076 CSF_mets = (find(~cellfun(@isempty,strfind(male.mets,'[csf]'))));
0077 CSF_mets_male = setdiff(CSF_mets,SL);
0078 D_mets = (find(~cellfun(@isempty,strfind(male.mets,'[d]'))));
0079 D_mets_male = setdiff(D_mets,SL);
0080 FE_mets = (find(~cellfun(@isempty,strfind(male.mets,'[fe]'))));
0081 FE_mets_male = setdiff(FE_mets,SL);
0082 SW_mets = (find(~cellfun(@isempty,strfind(male.mets,'[sw]'))));
0083 SW_mets_male = setdiff(SW_mets,SL);
0084 A_mets = (find(~cellfun(@isempty,strfind(male.mets,'[a]'))));
0085 A_mets_male = setdiff(A_mets,SL);
0086 M_mets = (find(~cellfun(@isempty,strfind(male.mets,'[mi]'))));
0087 M_mets_male = setdiff(M_mets,SL);
0088 
0089 Table_Met_comp(1,:) ={' ';'Harvetta';'Harvey'};
0090 Table_Met_comp(2,:) ={'Number of Reactions';num2str(length(female.rxns));num2str(length(male.rxns))};
0091 Table_Met_comp(3,:) ={'Number of Metabolites'    num2str(length(female.mets)-length(SL_female))    num2str(length(male.mets)-length(SL_male))};
0092 Table_Met_comp(4,:) ={'Number of Genes (transcripts)'    num2str(length(female.genes))    num2str(length(male.genes))};
0093 Table_Met_comp(5,:) ={'Number of Subsystems'    num2str(length(unique(female.subSystems)))    num2str(length(unique(male.subSystems)))};
0094 Table_Met_comp(6,:) ={'Blood compartment metabolites'    num2str(length(BC_mets_female))    num2str(length(BC_mets_male))};
0095 Table_Met_comp(7,:) ={'Urine metabolites'    num2str(length(U_mets_female))    num2str(length(U_mets_male))};
0096 Table_Met_comp(8,:) ={'Portal vein metabolites'    num2str(length(BP_mets_female))    num2str(length(BP_mets_male))};
0097 Table_Met_comp(9,:) ={'Bile duct metabolites'    num2str(length(BD_mets_female))    num2str(length(BD_mets_male))};
0098 Table_Met_comp(10,:) ={'CSF metabolites'    num2str(length(CSF_mets_female))    num2str(length(CSF_mets_male))};
0099 Table_Met_comp(11,:) ={'Diet metabolites'    num2str(length(D_mets_female))    num2str(length(D_mets_male))};
0100 Table_Met_comp(12,:) ={'Fecal metabolites'    num2str(length(FE_mets_female))    num2str(length(FE_mets_male))};
0101 Table_Met_comp(13,:) ={'Sweat metabolites'    num2str(length(SW_mets_female))    num2str(length(SW_mets_male))};
0102 Table_Met_comp(14,:) ={'Air metabolites'    num2str(length(A_mets_female))    num2str(length(A_mets_male))};
0103 Table_Met_comp(15,:) ={'Milk metabolites'    num2str(length(M_mets_female))    num2str((0))};
0104 
0105 BC_male_only = setdiff(male.mets(BC_mets_male),female.mets(BC_mets_female));
0106 
0107 CSF_male_only = setdiff(male.mets(CSF_mets_male),female.mets(CSF_mets_female));
0108 CSF_female_only = setdiff(female.mets(CSF_mets_female),male.mets(CSF_mets_male));
0109 CSF_shared = intersect(female.mets(CSF_mets_female),male.mets(CSF_mets_male));
0110 
0111 
0112 U_male_only = setdiff(male.mets(U_mets_male),female.mets(U_mets_female));
0113 U_female_only = setdiff(female.mets(U_mets_female),male.mets(U_mets_male));
0114 U_shared = intersect(female.mets(U_mets_female),male.mets(U_mets_male));
0115 
0116 % reactions unique to each sex
0117 Rxns_male_only = setdiff(male.rxns,female.rxns);
0118 Rxns_female_only = setdiff(female.rxns,male.rxns);
0119 
0120 
0121 load('microbiota_model_samp_SRS011239.mat');
0122 
0123 % unique microbial metabolites [lu] compartment
0124 MyU_rxns = microbiota_model.rxns(find(~cellfun(@isempty,strfind(microbiota_model.rxns,'UFEt'))));
0125 MyU_rxns = unique(regexprep(MyU_rxns,'UFEt_',''));
0126 
0127 Omale = fieldnames(OrganCompendium_male);
0128 Ofemale = fieldnames(OrganCompendium_female);
0129 
0130 clear TableProp_male
0131 for i = 1 : length(Omale)
0132     if ~strcmp('sex',Omale{i}) && ~strcmp('gender',Omale{i}) && ~strcmp('Recon3DHarvey',Omale{i})
0133         clear remR3M
0134         cm =2;
0135         TableProp_male{i+1,1} = Omale{i};
0136         
0137         TableProp_male{1,cm} = {'Reactions'};
0138         TableProp_male{i+1,cm} = num2str(length(OrganCompendium_male.(Omale{i}).modelAllComp.rxns)); cm = cm + 1;
0139         
0140         TableProp_male{1,cm} = {'Reactions (without exchange/transport reactions)'};
0141         EX =find(~cellfun(@isempty,strfind(OrganCompendium_male.(Omale{i}).modelAllComp.rxns,'EX_')));
0142         DM =find(~cellfun(@isempty,strfind(OrganCompendium_male.(Omale{i}).modelAllComp.rxns,'DM_')));
0143         Sink =find(~cellfun(@isempty,strfind(OrganCompendium_male.(Omale{i}).modelAllComp.rxns,'sink_')));
0144         EX = [EX;DM;Sink];
0145         NoEx = length(OrganCompendium_male.(Omale{i}).modelAllComp.rxns)-length(EX);
0146         TableProp_male{i+1,cm} = num2str(NoEx); cm = cm + 1;
0147         
0148         TableProp_male{1,cm} = {'Percentage of all Recon Reactions (without exchange/transport reactions)'};
0149         EX =find(~cellfun(@isempty,strfind(OrganCompendium_male.(Omale{i}).modelAllComp.rxns,'EX_')));
0150         DM =find(~cellfun(@isempty,strfind(OrganCompendium_male.(Omale{i}).modelAllComp.rxns,'DM_')));
0151         Sink =find(~cellfun(@isempty,strfind(OrganCompendium_male.(Omale{i}).modelAllComp.rxns,'sink_')));
0152         EX = [EX;DM;Sink];
0153         NoEx = length(OrganCompendium_male.(Omale{i}).modelAllComp.rxns)-length(EX);
0154         Rxns = length(Recon3DHarvey.rxns);
0155         TableProp_male{i+1,cm} = num2str(NoEx*100/Rxns); cm = cm + 1;
0156         
0157         TableProp_male{1,cm} = {'Metabolites'};
0158         TableProp_male{i+1,cm} = num2str(length(OrganCompendium_male.(Omale{i}).modelAllComp.mets)); cm = cm + 1;
0159         
0160         TableProp_male{1,cm} = {'Percentage of all Recon Metabolites'};
0161         TableProp_male{i+1,cm} = num2str(length(OrganCompendium_male.(Omale{i}).modelAllComp.mets)*100/length(Recon3DHarvey.mets)); cm = cm + 1;
0162         
0163         TableProp_male{1,cm} = {'Metabolites (unique)'};
0164         [g,remR3M]=strtok(OrganCompendium_male.(Omale{i}).modelAllComp.mets,'[');
0165         TableProp_male{i+1,cm} = num2str(length(unique(g))); cm = cm + 1;
0166         
0167         TableProp_male{1,cm} = {'Percentage of all Metabolites (unique)'};
0168         [g,remR3M]=strtok(OrganCompendium_male.(Omale{i}).modelAllComp.mets,'[');
0169         [Mets]=strtok(male.mets,'[');
0170         TableProp_male{i+1,cm} = num2str(length(unique(g))*100/length(unique(Mets))); cm = cm + 1;
0171         
0172         % number of compartments
0173         TableProp_male{1,cm} = {'Compartments (unique)'};
0174         TableProp_male{i+1,cm} = num2str(length(unique(remR3M))); cm = cm + 1;
0175         
0176         % list of compartments
0177         TableProp_male{1,cm} = {'Compartment List (unique)'};
0178         C = unique(remR3M);
0179         for j= 1 : length(C)
0180             s= ' ';
0181             TableProp_male{i+1,cm} = strcat(TableProp_male{i+1,cm},',',s,C{j});
0182         end
0183         cm = cm + 1;
0184         
0185         %number of exchanges with [bc]
0186         TableProp_male{1,cm} = {'Number of exchanges with [bc]'};
0187         EX =find(~cellfun(@isempty,strfind(OrganCompendium_male.(Omale{i}).modelAllComp.rxns,'_EX_')));
0188         BC =find(~cellfun(@isempty,strfind(OrganCompendium_male.(Omale{i}).modelAllComp.rxns,'[bc]')));
0189         BCK =find(~cellfun(@isempty,strfind(OrganCompendium_male.(Omale{i}).modelAllComp.rxns,'[bcK]')));
0190         BC = intersect(EX,BC);
0191         BC = setdiff(BC,BCK);
0192         TableProp_male{i+1,cm} = num2str(length(BC)); cm = cm + 1;
0193         
0194         %percentage of all exchanges with [bc]
0195         TableProp_male{1,cm} = {'Percentage of all exchanges with [bc]'};
0196         EX =find(~cellfun(@isempty,strfind(OrganCompendium_male.(Omale{i}).modelAllComp.rxns,'_EX_')));
0197         BC =find(~cellfun(@isempty,strfind(OrganCompendium_male.(Omale{i}).modelAllComp.rxns,'[bc]')));
0198         BCK =find(~cellfun(@isempty,strfind(OrganCompendium_male.(Omale{i}).modelAllComp.rxns,'[bcK]')));
0199         BC = intersect(EX,BC);
0200         BC = setdiff(BC,BCK);
0201         TableProp_male{i+1,cm} = num2str(length(BC)*100/length(BC_mets_male)); cm = cm + 1;
0202         
0203         %number of exchanges with [bp]
0204         TableProp_male{1,cm} = {'Number of exchanges with [bp]'};
0205         EX =find(~cellfun(@isempty,strfind(OrganCompendium_male.(Omale{i}).modelAllComp.rxns,'_EX_')));
0206         BC =find(~cellfun(@isempty,strfind(OrganCompendium_male.(Omale{i}).modelAllComp.rxns,'[bp')));
0207         BC = intersect(EX,BC);
0208         TableProp_male{i+1,cm} = num2str(length(BC)); cm = cm + 1;
0209         
0210         %percentage of all exchanges with [bp]
0211         TableProp_male{1,cm} = {'Percentage of all exchanges with [bp]'};
0212         EX =find(~cellfun(@isempty,strfind(OrganCompendium_male.(Omale{i}).modelAllComp.rxns,'_EX_')));
0213         BC =find(~cellfun(@isempty,strfind(OrganCompendium_male.(Omale{i}).modelAllComp.rxns,'[bp')));
0214         BC = intersect(EX,BC);
0215         TableProp_male{i+1,cm} = num2str(length(BC)*100/length(BP_mets_male)); cm = cm + 1;
0216         
0217         %number of exchanges with [bd]
0218         TableProp_male{1,cm} = {'Number of exchanges with [bd]'};
0219         EX =find(~cellfun(@isempty,strfind(OrganCompendium_male.(Omale{i}).modelAllComp.rxns,'_EX_')));
0220         BC =find(~cellfun(@isempty,strfind(OrganCompendium_male.(Omale{i}).modelAllComp.rxns,'[bd')));
0221         BC = intersect(EX,BC);
0222         TableProp_male{i+1,cm} = num2str(length(BC)); cm = cm + 1;
0223         
0224         
0225         %percentage of all exchanges with [bd]
0226         TableProp_male{1,cm} = {'Percentage of all exchanges with [bd]'};
0227         EX =find(~cellfun(@isempty,strfind(OrganCompendium_male.(Omale{i}).modelAllComp.rxns,'_EX_')));
0228         BC =find(~cellfun(@isempty,strfind(OrganCompendium_male.(Omale{i}).modelAllComp.rxns,'[bd')));
0229         BC = intersect(EX,BC);
0230         TableProp_male{i+1,cm} = num2str(length(BC)*100/length(BD_mets_male)); cm = cm + 1;
0231         
0232         %number of exchanges with [lu]
0233         TableProp_male{1,cm} = {'Number of exchanges with [lu]'};
0234         EX =find(~cellfun(@isempty,strfind(OrganCompendium_male.(Omale{i}).modelAllComp.rxns,'_EX_')));
0235         BC =find(~cellfun(@isempty,strfind(OrganCompendium_male.(Omale{i}).modelAllComp.rxns,'[lu')));
0236         BC = intersect(EX,BC);
0237         TableProp_male{i+1,cm} = num2str(length(BC)); cm = cm + 1;
0238         
0239         
0240         %number of exchanges with [csf]
0241         TableProp_male{1,cm} = {'Number of exchanges with [csf]'};
0242         EX =find(~cellfun(@isempty,strfind(OrganCompendium_male.(Omale{i}).modelAllComp.rxns,'_EX_')));
0243         BC =find(~cellfun(@isempty,strfind(OrganCompendium_male.(Omale{i}).modelAllComp.rxns,'[csf')));
0244         BC = intersect(EX,BC);
0245         TableProp_male{i+1,cm} = num2str(length(BC)); cm = cm + 1;
0246         
0247         %percentage of all exchanges with [csf]
0248         TableProp_male{1,cm} = {'Percentage of all exchanges with [csf]'};
0249         EX =find(~cellfun(@isempty,strfind(OrganCompendium_male.(Omale{i}).modelAllComp.rxns,'_EX_')));
0250         BC =find(~cellfun(@isempty,strfind(OrganCompendium_male.(Omale{i}).modelAllComp.rxns,'[csf')));
0251         BC = intersect(EX,BC);
0252         TableProp_male{i+1,cm} = num2str(length(BC)*100/length(CSF_mets_male)); cm = cm + 1;
0253         
0254         %number of exchanges with [sw]
0255         TableProp_male{1,cm} = {'Number of exchanges with [sw]'};
0256         EX =find(~cellfun(@isempty,strfind(OrganCompendium_male.(Omale{i}).modelAllComp.rxns,'_EX_')));
0257         BC =find(~cellfun(@isempty,strfind(OrganCompendium_male.(Omale{i}).modelAllComp.rxns,'[sw')));
0258         BC = intersect(EX,BC);
0259         TableProp_male{i+1,cm} = num2str(length(BC)); cm = cm + 1;
0260         
0261         %number of exchanges with [a]
0262         TableProp_male{1,cm} = {'Number of exchanges with [a]'};
0263         EX =find(~cellfun(@isempty,strfind(OrganCompendium_male.(Omale{i}).modelAllComp.rxns,'_EX_')));
0264         BC =find(~cellfun(@isempty,strfind(OrganCompendium_male.(Omale{i}).modelAllComp.rxns,'[a')));
0265         BC = intersect(EX,BC);
0266         TableProp_male{i+1,cm} = num2str(length(BC)); cm = cm + 1;
0267         
0268         %number of exchanges with [u]
0269         TableProp_male{1,cm} = {'Number of exchanges with [u]'};
0270         EX =find(~cellfun(@isempty,strfind(OrganCompendium_male.(Omale{i}).modelAllComp.rxns,'_EX_')));
0271         BC =find(~cellfun(@isempty,strfind(OrganCompendium_male.(Omale{i}).modelAllComp.rxns,'[u]')));
0272         BC = intersect(EX,BC);
0273         TableProp_male{i+1,cm} = num2str(length(BC)); cm = cm + 1;
0274         
0275         %percentage of all exchanges with [u]
0276         TableProp_male{1,cm} = {'Percentage of all exchanges with [u]'};
0277         EX =find(~cellfun(@isempty,strfind(OrganCompendium_male.(Omale{i}).modelAllComp.rxns,'_EX_')));
0278         BC =find(~cellfun(@isempty,strfind(OrganCompendium_male.(Omale{i}).modelAllComp.rxns,'[u]')));
0279         BC = intersect(EX,BC);
0280         TableProp_male{i+1,cm} = num2str(length(BC)*100/length(U_mets_male)); cm = cm + 1;
0281         
0282         %[bc] overlap with microbiota metabolites
0283         TableProp_male{1,cm} = {'Percentage of overlap of [bc] with microbiota metabolites'};
0284         EX =find(~cellfun(@isempty,strfind(OrganCompendium_male.(Omale{i}).modelAllComp.rxns,'_EX_')));
0285         BC =find(~cellfun(@isempty,strfind(OrganCompendium_male.(Omale{i}).modelAllComp.rxns,'[bc')));
0286         BC = intersect(EX,BC);
0287         BCM = OrganCompendium_male.(Omale{i}).modelAllComp.rxns(BC);
0288         BCM = regexprep(BCM,'Tr_EX_','');
0289         BCM = regexprep(BCM,'\(e.+','');
0290         BCM_MY = intersect(BCM,MyU_rxns);
0291         if ~isempty(BC)
0292             TableProp_male{i+1,cm} = num2str(length(BCM_MY)*100/length(BC)); cm = cm + 1;
0293         else
0294             TableProp_male{i+1,cm} = 'NA'; cm = cm + 1;
0295         end
0296         
0297         
0298         %[bp] overlap with microbiota metabolites
0299         TableProp_male{1,cm} = {'Percentage of overlap of [bp] with microbiota metabolites'};
0300         EX =find(~cellfun(@isempty,strfind(OrganCompendium_male.(Omale{i}).modelAllComp.rxns,'_EX_')));
0301         BC =find(~cellfun(@isempty,strfind(OrganCompendium_male.(Omale{i}).modelAllComp.rxns,'[bp')));
0302         BC = intersect(EX,BC);
0303         BCM = OrganCompendium_male.(Omale{i}).modelAllComp.rxns(BC);
0304         BCM = regexprep(BCM,'Tr_EX_','');
0305         BCM = regexprep(BCM,'\[bp.+','');
0306         BCM_MY = intersect(BCM,MyU_rxns);
0307         if ~isempty(BC)
0308             TableProp_male{i+1,cm} = num2str(length(BCM_MY)*100/length(BC)); cm = cm + 1;
0309         else
0310             TableProp_male{i+1,cm} = 'NA'; cm = cm + 1;
0311         end
0312         
0313         %[bd] overlap with microbiota metabolites
0314         TableProp_male{1,cm} = {'Percentage of overlap of [bd] with microbiota metabolites'};
0315         EX =find(~cellfun(@isempty,strfind(OrganCompendium_male.(Omale{i}).modelAllComp.rxns,'_EX_')));
0316         BC =find(~cellfun(@isempty,strfind(OrganCompendium_male.(Omale{i}).modelAllComp.rxns,'[bd')));
0317         BC = intersect(EX,BC);
0318         BCM = OrganCompendium_male.(Omale{i}).modelAllComp.rxns(BC);
0319         BCM = regexprep(BCM,'Tr_EX_','');
0320         BCM = regexprep(BCM,'\[bd.+','');
0321         BCM_MY = intersect(BCM,MyU_rxns);
0322         if ~isempty(BC)
0323             TableProp_male{i+1,cm} = num2str(length(BCM_MY)*100/length(BC)); cm = cm + 1;
0324         else
0325             TableProp_male{i+1,cm} = 'NA'; cm = cm + 1;
0326         end
0327         
0328         %[csf] overlap with microbiota metabolites
0329         TableProp_male{1,cm} = {'Percentage of overlap of [csf] with microbiota metabolites'};
0330         EX =find(~cellfun(@isempty,strfind(OrganCompendium_male.(Omale{i}).modelAllComp.rxns,'_EX_')));
0331         BC =find(~cellfun(@isempty,strfind(OrganCompendium_male.(Omale{i}).modelAllComp.rxns,'[csf')));
0332         BC = intersect(EX,BC);
0333         BCM = OrganCompendium_male.(Omale{i}).modelAllComp.rxns(BC);
0334         BCM = regexprep(BCM,'Tr_EX_','');
0335         BCM = regexprep(BCM,'\(e.+','');
0336         BCM_MY = intersect(BCM,MyU_rxns);
0337         if ~isempty(BC)
0338             TableProp_male{i+1,cm} = num2str(length(BCM_MY)*100/length(BC)); cm = cm + 1;
0339         else
0340             TableProp_male{i+1,cm} = 'NA'; cm = cm + 1;
0341         end
0342         
0343         %[u] overlap with microbiota metabolites
0344         TableProp_male{1,cm} = {'Percentage of overlap of [u] with microbiota metabolites'};
0345         EX =find(~cellfun(@isempty,strfind(OrganCompendium_male.(Omale{i}).modelAllComp.rxns,'_EX_')));
0346         BC =find(~cellfun(@isempty,strfind(OrganCompendium_male.(Omale{i}).modelAllComp.rxns,'[u')));
0347         BC = intersect(EX,BC);
0348         BCM = OrganCompendium_male.(Omale{i}).modelAllComp.rxns(BC);
0349         BCM = regexprep(BCM,'Tr_EX_','');
0350         BCM = regexprep(BCM,'\(e.+','');
0351         BCM_MY = intersect(BCM,MyU_rxns);
0352         if ~isempty(BC)
0353             TableProp_male{i+1,cm} = num2str(length(BCM_MY)*100/length(BC)); cm = cm + 1;
0354         else
0355             TableProp_male{i+1,cm} = 'NA'; cm = cm + 1;
0356         end
0357         
0358         %[u] overlap with microbiota metabolites
0359         TableProp_male{1,cm} = {'Percentage of overlap of [u] with microbiota metabolites'};
0360         EX =find(~cellfun(@isempty,strfind(OrganCompendium_male.(Omale{i}).modelAllComp.rxns,'_EX_')));
0361         BC =find(~cellfun(@isempty,strfind(OrganCompendium_male.(Omale{i}).modelAllComp.rxns,'[u')));
0362         BC = intersect(EX,BC);
0363         BCM = OrganCompendium_male.(Omale{i}).modelAllComp.rxns(BC);
0364         BCM = regexprep(BCM,'Tr_EX_','');
0365         BCM = regexprep(BCM,'\(e.+','');
0366         BCM_MY = intersect(BCM,MyU_rxns);
0367         if ~isempty(BC)
0368             TableProp_male{i+1,cm} = num2str(length(BCM_MY)*100/length(BC)); cm = cm + 1;
0369         else
0370             TableProp_male{i+1,cm} = 'NA'; cm = cm + 1;
0371         end
0372         
0373         %[lu] overlap with microbiota metabolites
0374         TableProp_male{1,cm} = {'Percentage of overlap of [lu] with microbiota metabolites'};
0375         EX =find(~cellfun(@isempty,strfind(OrganCompendium_male.(Omale{i}).modelAllComp.rxns,'_EX_')));
0376         BC =find(~cellfun(@isempty,strfind(OrganCompendium_male.(Omale{i}).modelAllComp.rxns,'[lu')));
0377         BC = intersect(EX,BC);
0378         BCM = OrganCompendium_male.(Omale{i}).modelAllComp.rxns(BC);
0379         BCM = regexprep(BCM,'Tr_EX_','');
0380         BCM = regexprep(BCM,'\[lu.+','');
0381         BCM_MY = intersect(BCM,MyU_rxns);
0382         if ~isempty(BC)
0383             TableProp_male{i+1,cm} = num2str(length(BCM_MY)*100/length(BC)); cm = cm + 1;
0384         else
0385             TableProp_male{i+1,cm} = 'NA'; cm = cm + 1;
0386         end
0387         
0388         TableProp_male{1,cm} = {'Transcripts'};
0389         TableProp_male{i+1,cm} = num2str(length(OrganCompendium_male.(Omale{i}).modelAllComp.genes)); cm = cm + 1;
0390         
0391         TableProp_male{1,cm} = {'Genes (unique)'};
0392         [g,rem]=strtok(OrganCompendium_male.(Omale{i}).modelAllComp.genes,'.');
0393         TableProp_male{i+1,cm} = num2str(length(unique(g))); cm = cm + 1;
0394         
0395         TableProp_male{1,cm} = {'Number Genes Associated Reactions'};
0396         str = length(find(~cellfun(@isempty,OrganCompendium_male.(Omale{i}).modelAllComp.grRules)));
0397         TableProp_male{i+1,cm} = num2str(str); cm = cm + 1;
0398         
0399         TableProp_male{1,cm} = {'Percentage Genes Associated Reactions (Exchange Rxns excl)'};
0400         str = length(find(~cellfun(@isempty,OrganCompendium_male.(Omale{i}).modelAllComp.grRules)));
0401         TableProp_male{i+1,cm} = num2str(str*100/NoEx); cm = cm + 1;
0402         PercGeneAssRxns_male(i,1) = str*100/NoEx;
0403         
0404 %         TableProp_male{1,cm} = {'Subsystems'};
0405 %         if isempty(OrganCompendium_male.(Omale{i}).modelAllComp.subSystems{1})
0406 %             TableProp_male{i+1,cm}=NaN;
0407 %         else
0408 %             TableProp_male{i+1,cm} = num2str(length(unique(OrganCompendium_male.(Omale{i}).modelAllComp.subSystems)));
0409 %         end
0410 %         cm = cm + 1;
0411         
0412         TableProp_male{1,cm} = {'Size of S'};
0413         TableProp_male{i+1,cm} = strcat(num2str(size(OrganCompendium_male.(Omale{i}).modelAllComp.S,1)),'; ',num2str(size(OrganCompendium_male.(Omale{i}).modelAllComp.S,2))); cm = cm + 1;
0414         
0415         % rank of S
0416         TableProp_male{1,cm} = {'Rank of S'};
0417         %   TableProp_male{i+1,cm} = strcat(num2str(rank(full(OrganCompendium_male.(Omale{i}).modelAllComp.S)))); cm = cm + 1;
0418         
0419     end
0420 end
0421 TableProp_male=TableProp_male';
0422 
0423 %% compare reaction content
0424 clear TableRxns TableRxnsNum
0425 %Rxns = Recon3DHarvey.rxns;
0426 
0427 Rxns = [];
0428 % get set of unique reactions
0429 for i = 1 : length(Omale)
0430     if ~strcmp('sex',Omale{i}) && ~strcmp('gender',Omale{i}) && ~strcmp('Recon3DHarvey',Omale{i})
0431         % grab reactions
0432         ORxns = OrganCompendium_male.(Omale{i}).modelAllComp.rxns;
0433         ORxns = regexprep(ORxns,'Tr_','');
0434         ORxns = regexprep(ORxns,'_\[\w+\]','');
0435         ORxns = regexprep(ORxns,'\[\w\w\w\]','');
0436         ORxns = regexprep(ORxns,'\[\w\w\]','');
0437         ORxns = regexprep(ORxns,'\(','\[');
0438         ORxns = regexprep(ORxns,'\)','\]');
0439         ORxns = regexprep(ORxns,'_c_','\[c\]');
0440         ORxns = regexprep(ORxns,'_g_','\[g\]');
0441         ORxns = regexprep(ORxns,'_n_','\[n\]');
0442         ORxns = regexprep(ORxns,'_m_','\[m\]');
0443         ORxns = regexprep(ORxns,'_r_','\[r\]');
0444         ORxns = regexprep(ORxns,'_x_','\[x\]');
0445         ORxns = regexprep(ORxns,'\[u\]','');
0446         ORxns = regexprep(ORxns,'\[e\]','');
0447         ORxns = regexprep(ORxns,'\[mi\w\]','');
0448         ORxns = regexprep(ORxns,'\[sw\w\]','');
0449         ORxns = regexprep(ORxns,'\[lu\w\w\]','');
0450         Rxns = [Rxns;ORxns];
0451         
0452     end
0453 end
0454 Rxns = unique(Rxns);
0455 
0456 TableRxns_male(1:length(Rxns),1) = (Rxns);
0457 for i = 1 : length(Omale)
0458     if ~strcmp('sex',Omale{i}) && ~strcmp('gender',Omale{i}) && ~strcmp('Recon3DHarvey',Omale{i})
0459         % grab reactions
0460         ORxns = OrganCompendium_male.(Omale{i}).modelAllComp.rxns;
0461         ORxns = regexprep(ORxns,'Tr_','');
0462         ORxns = regexprep(ORxns,'_\[\w+\]','');
0463         ORxns = regexprep(ORxns,'\[\w\w\w\]','');
0464         ORxns = regexprep(ORxns,'\[\w\w\]','');
0465         ORxns = regexprep(ORxns,'\(','\[');
0466         ORxns = regexprep(ORxns,'\)','\]');
0467         ORxns = regexprep(ORxns,'_c_','\[c\]');
0468         ORxns = regexprep(ORxns,'_g_','\[g\]');
0469         ORxns = regexprep(ORxns,'_n_','\[n\]');
0470         ORxns = regexprep(ORxns,'_m_','\[m\]');
0471         ORxns = regexprep(ORxns,'_r_','\[r\]');
0472         ORxns = regexprep(ORxns,'_x_','\[x\]');
0473         ORxns = regexprep(ORxns,'\[u\]','');
0474         ORxns = regexprep(ORxns,'\[e\]','');
0475         ORxns = regexprep(ORxns,'\[mi\w\]','');
0476         ORxns = regexprep(ORxns,'\[sw\w\]','');
0477         ORxns = regexprep(ORxns,'\[lu\w\w\]','');
0478         ORxns = regexprep(ORxns,'_DIFF\[c\]','_DIFF');
0479         TableRxns_male(:,i+1)=num2cell(0);
0480         TableRxns_maleO{1,i+1} = Omale{i};
0481         TableRxns_male(find(ismember(Rxns,ORxns)),i+1)=num2cell(1);
0482         TableRxnsNum_male(find(ismember(Rxns,ORxns)),i)=1;
0483     end
0484 end
0485 
0486 TableRxns_male = [TableRxns_maleO;TableRxns_male];
0487 % correlation
0488 TableRxnsNumCorr_male = corrcoef(TableRxnsNum_male);
0489 
0490 % shared reactions
0491 TableRxns_maleNumInOrgans = TableRxnsNum_male*TableRxnsNum_male';
0492 RxnsInOrgans_male = diag(TableRxns_maleNumInOrgans);
0493 HousekeepingRxns_male = Rxns(find(RxnsInOrgans_male>=length(Omale)-2));%2 entries of Omale are no organs
0494 OrganSpecRxns_male = Rxns(find(RxnsInOrgans_male==1));%at most in 2 organs
0495 NoOrgaRxns_male = Rxns(find(RxnsInOrgans_male==0));%at most in 2 organs
0496 OtherOrganRxns_male = Rxns(find(RxnsInOrgans_male>1 & RxnsInOrgans_male<length(Omale)-2));%at most in 2 organs
0497 s = {' '};
0498 sumRxns = length(NoOrgaRxns_male)+length(OtherOrganRxns_male)+length(OrganSpecRxns_male)+length(HousekeepingRxns_male);
0499 TableGRM{1,1} = 'male';
0500 TableGRM{1,4} = 'Reactions';
0501 TableGRM{2,1} = 'core';
0502 TableGRM{2,4} = strcat(num2str(length(HousekeepingRxns_male)),s,'(',num2str(round(length(HousekeepingRxns_male)*100/sumRxns,1)),'%)');
0503 TableGRM{3,1} = 'organ-specific';
0504 TableGRM{3,4} = strcat(num2str(length(OrganSpecRxns_male)),s,'(',num2str(round(length(OrganSpecRxns_male)*100/sumRxns,1)),'%)');
0505 TableGRM{4,1} = 'others';
0506 TableGRM{4,4} = strcat(num2str(length(OtherOrganRxns_male)),s,'(',num2str(round(length(OtherOrganRxns_male)*100/sumRxns,1)),'%)');
0507 TableGRM{5,1} = 'absent';
0508 TableGRM{5,4} = strcat(num2str(length(NoOrgaRxns_male)),s,'(',num2str(round(length(NoOrgaRxns_male)*100/sumRxns,1)),'%)');
0509 TableGRM{6,1} = 'sum';
0510 TableGRM{6,4} = num2str(sumRxns);
0511 
0512 
0513 for i = 1 : length(Omale)
0514     if ~strcmp('sex',Omale{i}) && ~strcmp('gender',Omale{i}) && ~strcmp('Recon3DHarvey',Omale{i})
0515         % grab reactions
0516         ORxns = OrganCompendium_male.(Omale{i}).modelAllComp.rxns;
0517         HouseCoreORxns_male(i,1) = length(intersect(HousekeepingRxns_male ,ORxns))*100/length(ORxns);
0518         HouseCoreORxns_male(i,2) = length(intersect(OrganSpecRxns_male ,ORxns))*100/length(ORxns);
0519         CoreOrganRxns_male(i,1) = length(intersect(OrganSpecRxns_male ,ORxns));
0520         
0521     end
0522 end
0523 
0524 
0525 if 0
0526     figure;
0527     bar([HouseCoreORxns_male(:,1) HouseCoreORxns_male(:,2)],'stacked');
0528 end
0529 
0530 clear TableMets_male TableMetsNum_male
0531 %Mets = Recon3DHarvey.mets;
0532 
0533 Mets = [];
0534 % get set of unique metablites
0535 for i = 1 : length(Omale)
0536     if ~strcmp('sex',Omale{i}) && ~strcmp('gender',Omale{i}) && ~strcmp('Recon3DHarvey',Omale{i})
0537         % grab reactions
0538         OMets = OrganCompendium_male.(Omale{i}).modelAllComp.mets;
0539         OMets = regexprep(OMets,'\[b\w\w\]','\[e\]');
0540         OMets = regexprep(OMets,'\[b\w\]','\[e\]');
0541         OMets = regexprep(OMets,'\[u\]','\[e\]');
0542         OMets = regexprep(OMets,'\[u\]','\[e\]');
0543         OMets = regexprep(OMets,'\[lu\]','\[e\]');
0544         OMets = regexprep(OMets,'\[lu\w+\]','\[e\]');
0545         OMets = regexprep(OMets,'\[csf\]','\[e\]');
0546         OMets = regexprep(OMets,'\[s\w+\]','\[e\]');
0547         OMets = regexprep(OMets,'\[fe\]','\[e\]');
0548         OMets = regexprep(OMets,'\[a\]','\[e\]');
0549         OMets = regexprep(OMets,'\[e\]','');
0550         OMets = regexprep(OMets,'\[c\]','');
0551         OMets = regexprep(OMets,'\[m\]','');
0552         OMets = regexprep(OMets,'\[r\]','');
0553         OMets = regexprep(OMets,'\[g\]','');
0554         OMets = regexprep(OMets,'\[x\]','');
0555         OMets = regexprep(OMets,'\[n\]','');
0556         OMets = regexprep(OMets,'\[l\]','');
0557         Mets = [Mets;OMets];
0558     end
0559 end
0560 Mets = unique(Mets);
0561 
0562 clear TableMets_male
0563 TableMets_male(1:length(Mets),1) = (Mets);
0564 for i = 1 : length(Omale)
0565     if ~strcmp('sex',Omale{i}) && ~strcmp('gender',Omale{i}) && ~strcmp('Recon3DHarvey',Omale{i})
0566         % grab reactions
0567         OMets = OrganCompendium_male.(Omale{i}).modelAllComp.mets;
0568         OMets = regexprep(OMets,'\[b\w\w\]','\[e\]');
0569         OMets = regexprep(OMets,'\[b\w\]','\[e\]');
0570         OMets = regexprep(OMets,'\[u\]','\[e\]');
0571         OMets = regexprep(OMets,'\[u\]','\[e\]');
0572         OMets = regexprep(OMets,'\[lu\]','\[e\]');
0573         OMets = regexprep(OMets,'\[lu\w+\]','\[e\]');
0574         OMets = regexprep(OMets,'\[csf\]','\[e\]');
0575         OMets = regexprep(OMets,'\[s\w+\]','\[e\]');
0576         OMets = regexprep(OMets,'\[fe\]','\[e\]');
0577         OMets = regexprep(OMets,'\[a\]','\[e\]');
0578         OMets = regexprep(OMets,'\[e\]','');
0579         OMets = regexprep(OMets,'\[c\]','');
0580         OMets = regexprep(OMets,'\[m\]','');
0581         OMets = regexprep(OMets,'\[r\]','');
0582         OMets = regexprep(OMets,'\[g\]','');
0583         OMets = regexprep(OMets,'\[x\]','');
0584         OMets = regexprep(OMets,'\[n\]','');
0585         OMets = regexprep(OMets,'\[l\]','');
0586         TableMets_male(:,i+1)=num2cell(0);
0587         TableMets_maleO{1,i+1}=Omale{i};
0588         TableMets_male(find(ismember(Mets,OMets)),i+1)=num2cell(1);
0589         TableMetsNum_male(find(ismember(Mets,OMets)),i)=1;
0590     end
0591 end
0592 TableMets_male_unique = [TableMets_maleO;TableMets_male];
0593 
0594 
0595 Mets = [];
0596 % get set of unique metablites
0597 for i = 1 : length(Omale)
0598     if ~strcmp('sex',Omale{i}) && ~strcmp('gender',Omale{i}) && ~strcmp('Recon3DHarvey',Omale{i})
0599         % grab reactions
0600         OMets = OrganCompendium_male.(Omale{i}).modelAllComp.mets;
0601         OMets = regexprep(OMets,'\[b\w\w\]','\[e\]');
0602         OMets = regexprep(OMets,'\[b\w\]','\[e\]');
0603         OMets = regexprep(OMets,'\[u\]','\[e\]');
0604         OMets = regexprep(OMets,'\[u\]','\[e\]');
0605         OMets = regexprep(OMets,'\[lu\]','\[e\]');
0606         OMets = regexprep(OMets,'\[lu\w+\]','\[e\]');
0607         OMets = regexprep(OMets,'\[csf\]','\[e\]');
0608         OMets = regexprep(OMets,'\[s\w+\]','\[e\]');
0609         OMets = regexprep(OMets,'\[fe\]','\[e\]');
0610         OMets = regexprep(OMets,'\[a\]','\[e\]');
0611         Mets = [Mets;OMets];
0612     end
0613 end
0614 Mets = unique(Mets);
0615 
0616 clear TableMets_male
0617 TableMets_male(1:length(Mets),1) = (Mets);
0618 for i = 1 : length(Omale)
0619     if ~strcmp('sex',Omale{i}) && ~strcmp('gender',Omale{i}) && ~strcmp('Recon3DHarvey',Omale{i})
0620         % grab reactions
0621         OMets = OrganCompendium_male.(Omale{i}).modelAllComp.mets;
0622         OMets = regexprep(OMets,'\[b\w\w\]','\[e\]');
0623         OMets = regexprep(OMets,'\[b\w\]','\[e\]');
0624         OMets = regexprep(OMets,'\[u\]','\[e\]');
0625         OMets = regexprep(OMets,'\[u\]','\[e\]');
0626         OMets = regexprep(OMets,'\[lu\]','\[e\]');
0627         OMets = regexprep(OMets,'\[lu\w+\]','\[e\]');
0628         OMets = regexprep(OMets,'\[csf\]','\[e\]');
0629         OMets = regexprep(OMets,'\[s\w+\]','\[e\]');
0630         OMets = regexprep(OMets,'\[fe\]','\[e\]');
0631         OMets = regexprep(OMets,'\[a\]','\[e\]');
0632         TableMets_male(:,i+1)=num2cell(0);
0633         TableMets_maleO{1,i+1}=Omale{i};
0634         TableMets_male(find(ismember(Mets,OMets)),i+1)=num2cell(1);
0635         TableMetsNum_male(find(ismember(Mets,OMets)),i)=1;
0636     end
0637 end
0638 TableMets_male = [TableMets_maleO;TableMets_male];
0639 % correlation
0640 TableMetsNum_maleCorr = corrcoef(TableMetsNum_male);
0641 
0642 % shared metabolites
0643 TableMets_maleNumInOrgans = TableMetsNum_male*TableMetsNum_male';
0644 MetsInOrgans_male = diag(TableMets_maleNumInOrgans);
0645 HousekeepingMets_male = Mets(find(MetsInOrgans_male>=length(Omale)-2));%2 entries of Omale are no organs
0646 OrganSpecMets_male = Mets(find(MetsInOrgans_male==1));%at most in 2 organs
0647 NoOrgaMets_male = Mets(find(MetsInOrgans_male==0));%at most in 2 organs
0648 OtherOrganMets_male = Mets(find(MetsInOrgans_male>1 & MetsInOrgans_male<length(Omale)-2));%at most in 2 organs
0649 sumMets = length(NoOrgaMets_male)+length(OtherOrganMets_male)+length(OrganSpecMets_male)+length(HousekeepingMets_male);
0650 s = {' '};
0651 TableGRM{1,3} = 'Metabolites';
0652 TableGRM{2,1} = 'core';
0653 TableGRM{2,3} = strcat(num2str(length(HousekeepingMets_male)),s,'(',num2str(round(length(HousekeepingMets_male)*100/sumMets,1)),'%)');
0654 TableGRM{3,1} = 'organ-specific';
0655 TableGRM{3,3} = strcat(num2str(length(OrganSpecMets_male)),s,'(',num2str(round(length(OrganSpecMets_male)*100/sumMets,1)),'%)');
0656 TableGRM{4,1} = 'others';
0657 TableGRM{4,3} = strcat(num2str(length(OtherOrganMets_male)),s,'(',num2str(round(length(OtherOrganMets_male)*100/sumMets,1)),'%)');
0658 TableGRM{5,1} = 'absent';
0659 TableGRM{5,3} = strcat(num2str(length(NoOrgaMets_male)),s,'(',num2str(round(length(NoOrgaMets_male)*100/sumMets,1)),'%)');
0660 TableGRM{6,1} = 'sum';
0661 TableGRM{6,3} = num2str(sumMets);
0662 
0663 % get number of organ specific and core rxns per organ
0664 
0665 for i = 1 : length(Omale)
0666     if ~strcmp('sex',Omale{i}) && ~strcmp('gender',Omale{i}) && ~strcmp('Recon3DHarvey',Omale{i})
0667         % grab reactions
0668         OMets = OrganCompendium_male.(Omale{i}).modelAllComp.mets;
0669         HouseCoreOMets_male(i,1) = length(intersect(HousekeepingMets_male ,OMets))*100/length(OMets);
0670         HouseCoreOMets_male(i,2) = length(intersect(OrganSpecMets_male ,OMets))*100/length(OMets);
0671         CoreOrganMets_male(i,1) = length(intersect(OrganSpecMets_male ,ORxns));
0672         
0673     end
0674 end
0675 
0676 if 0
0677     figure
0678     bar([HouseCoreOMets_male(:,1) HouseCoreOMets_male(:,2)],'stacked');
0679 end
0680 
0681 clear TableGenes_male TableGenes_maleNum
0682 %[Genes,rem]=strtok(Recon3DHarvey.genes,'.');
0683 
0684 Genes = [];
0685 % get set of unique reactions
0686 for i = 1 : length(Omale)
0687     if ~strcmp('sex',Omale{i}) && ~strcmp('gender',Omale{i}) && ~strcmp('Recon3DHarvey',Omale{i})
0688         % grab reactions
0689         OGenes = OrganCompendium_male.(Omale{i}).modelAllComp.genes;
0690         Genes = [Genes;OGenes];
0691     end
0692 end
0693 %[Genes,rem]=strtok(Genes,'.');
0694 Genes = unique(Genes);
0695 
0696 Genes = unique(Genes);
0697 TableGenes_male(1:length(Genes),1) = (Genes);
0698 for i = 1 : length(Omale)
0699     if ~strcmp('sex',Omale{i}) && ~strcmp('gender',Omale{i}) && ~strcmp('Recon3DHarvey',Omale{i})
0700         % grab reactions
0701       %  [OGenes,rem]=strtok(OrganCompendium_male.(Omale{i}).modelAllComp.genes,'.');
0702      %   OGenes = unique(OGenes);
0703         OGenes = OrganCompendium_male.(Omale{i}).modelAllComp.genes;
0704         TableGenes_male(:,i+1)=num2cell(0);
0705         TableGenes_male(find(ismember(Genes,OGenes)),i+1)=num2cell(1);
0706         TableGenes_maleNum(find(ismember(Genes,OGenes)),i)=1;
0707         Organs{1,i+1} = Omale{i};
0708     end
0709 end
0710 TableGenes_male = [Organs;TableGenes_male];
0711 
0712 % correlation
0713 TableGenes_maleNumCorr = corrcoef(TableGenes_maleNum);
0714 % get stats
0715 
0716 % shared genes
0717 TableGenes_maleNumInOrgans = TableGenes_maleNum*TableGenes_maleNum';
0718 GenesInOrgans_male = diag(TableGenes_maleNumInOrgans);
0719 HousekeepingGenes_male = Genes(find(GenesInOrgans_male>=length(Omale)-2));%2 entries of Omale are no organs
0720 OrganSpecGenes_male = Genes(find(GenesInOrgans_male==1));%at most in 2 organs
0721 NoOrganGenes_male = Genes(find(GenesInOrgans_male==0));%at most in 2 organs
0722 OtherOrganGenes_male = Genes(find(GenesInOrgans_male>1 & GenesInOrgans_male<length(Omale)-2));%at most in 2 organs
0723 sumGenes = length(NoOrganGenes_male)+length(OtherOrganGenes_male)+length(OrganSpecGenes_male)+length(HousekeepingGenes_male);
0724 s = {' '};
0725 TableGRM{1,2} = 'Genes';
0726 TableGRM{2,1} = 'core';
0727 TableGRM{2,2} = strcat(num2str(length(HousekeepingGenes_male)),s,'(',num2str(round(length(HousekeepingGenes_male)*100/sumGenes,1)),'%)');
0728 TableGRM{3,1} = 'organ-specific';
0729 TableGRM{3,2} = strcat(num2str(length(OrganSpecGenes_male)),s,'(',num2str(round(length(OrganSpecGenes_male)*100/sumGenes,1)),'%)');
0730 TableGRM{4,1} = 'others';
0731 TableGRM{4,2} = strcat(num2str(length(OtherOrganGenes_male)),s,'(',num2str(round(length(OtherOrganGenes_male)*100/sumGenes,1)),'%)');
0732 TableGRM{5,1} = 'absent';
0733 TableGRM{5,2} = strcat(num2str(length(NoOrganGenes_male)),s,'(',num2str(round(length(NoOrganGenes_male)*100/sumGenes,1)),'%)');
0734 TableGRM{6,1} = 'sum';
0735 TableGRM{6,2} = num2str(sumGenes);
0736 
0737 
0738 if 0
0739     figure
0740     imagesc(TableRxnsNumCorr_male)
0741     figure
0742     imagesc(TableRxnsNum_male)
0743     figure
0744     imagesc(TableMetsNum_maleCorr)
0745     figure
0746     imagesc(TableMetsNum_male)
0747     figure
0748     imagesc(TableGenes_maleNumCorr)
0749     figure
0750     imagesc(TableGenes_maleNum)
0751 end
0752 
0753 % get stats table
0754 clear TableAverage_male
0755 c = 1;
0756 TableAverage_male{c,1} = {'Average number of reactions'};
0757 Sum = sum(TableRxnsNum_male);
0758 SumR_male = Sum;
0759 TableAverage_male{c,2} = num2str(mean(Sum));c= c+1;
0760 TableAverage_male{c,1} = {'Std number of reactions'};
0761 TableAverage_male{c,2} = num2str(std(Sum));c= c+1;
0762 TableAverage_male{c,1} = {'Min number of reactions'};
0763 TableAverage_male{c,2} = num2str(min(Sum));c= c+1;
0764 TableAverage_male{c,1} = {'Max number of reactions'};
0765 TableAverage_male{c,2} = num2str(max(Sum));c= c+1;
0766 
0767 TableAverage_male{c,1} = {'Average number of metabolites'};
0768 Sum = sum(TableMetsNum_male);
0769 SumM_male = Sum;
0770 TableAverage_male{c,2} = num2str(mean(Sum));c= c+1;
0771 TableAverage_male{c,1} = {'Std number of metabolites'};
0772 TableAverage_male{c,2} = num2str(std(Sum));c= c+1;
0773 TableAverage_male{c,1} = {'Min number of metabolites'};
0774 TableAverage_male{c,2} = num2str(min(Sum));c= c+1;
0775 TableAverage_male{c,1} = {'Max number of metabolites'};
0776 TableAverage_male{c,2} = num2str(max(Sum));c= c+1;
0777 
0778 TableAverage_male{c,1} = {'Average number of genes'};
0779 Sum = sum(TableGenes_maleNum);
0780 SumG_male = Sum;
0781 TableAverage_male{c,2} = num2str(mean(Sum));c= c+1;
0782 TableAverage_male{c,1} = {'Std number of genes'};
0783 TableAverage_male{c,2} = num2str(std(Sum));c= c+1;
0784 TableAverage_male{c,1} = {'Min number of genes'};
0785 TableAverage_male{c,2} = num2str(min(Sum));c= c+1;
0786 TableAverage_male{c,1} = {'Max number of genes'};
0787 TableAverage_male{c,2} = num2str(max(Sum));c= c+1;
0788 
0789 
0790 % does not work on 2013
0791 if violinPlots == 1
0792     figure;
0793     vs = violinplot([SumR_male' SumM_male' SumG_male'], {'Reactions';'Metabolites';'Genes (unique)'});
0794 end
0795 
0796 %% female
0797 
0798 Ofemale = fieldnames(OrganCompendium_female);
0799 
0800 clear TableProp_female
0801 for i = 1 : length(Ofemale)
0802     if ~strcmp('sex',Ofemale{i}) && ~strcmp('gender',Ofemale{i}) && ~strcmp('Recon3DHarvey',Ofemale{i})
0803         cm =2;
0804         TableProp_female{i+1,1} = Ofemale{i};
0805         
0806         TableProp_female{1,cm} = {'Reactions'};
0807         TableProp_female{i+1,cm} = num2str(length(OrganCompendium_female.(Ofemale{i}).modelAllComp.rxns)); cm = cm + 1;
0808         
0809         TableProp_female{1,cm} = {'Reactions (without exchange/transport reactions)'};
0810         EX =find(~cellfun(@isempty,strfind(OrganCompendium_female.(Ofemale{i}).modelAllComp.rxns,'EX_')));
0811         DM =find(~cellfun(@isempty,strfind(OrganCompendium_female.(Ofemale{i}).modelAllComp.rxns,'DM_')));
0812         Sink =find(~cellfun(@isempty,strfind(OrganCompendium_female.(Ofemale{i}).modelAllComp.rxns,'sink_')));
0813         EX = [EX;DM;Sink];
0814         NoEx = length(OrganCompendium_female.(Ofemale{i}).modelAllComp.rxns)-length(EX);
0815         TableProp_female{i+1,cm} = num2str(NoEx); cm = cm + 1;
0816         
0817         TableProp_female{1,cm} = {'Percentage of all Recon Reactions (without exchange/transport reactions)'};
0818         EX =find(~cellfun(@isempty,strfind(OrganCompendium_female.(Ofemale{i}).modelAllComp.rxns,'EX_')));
0819         DM =find(~cellfun(@isempty,strfind(OrganCompendium_female.(Ofemale{i}).modelAllComp.rxns,'DM_')));
0820         Sink =find(~cellfun(@isempty,strfind(OrganCompendium_female.(Ofemale{i}).modelAllComp.rxns,'sink_')));
0821         EX = [EX;DM;Sink];
0822         NoEx = length(OrganCompendium_female.(Ofemale{i}).modelAllComp.rxns)-length(EX);
0823         Rxns = length(Recon3DHarvey.rxns);
0824         TableProp_female{i+1,cm} = num2str(NoEx*100/Rxns); cm = cm + 1;
0825         
0826         TableProp_female{1,cm} = {'Metabolites'};
0827         TableProp_female{i+1,cm} = num2str(length(OrganCompendium_female.(Ofemale{i}).modelAllComp.mets)); cm = cm + 1;
0828         
0829         TableProp_female{1,cm} = {'Percentage of all Recon Metabolites'};
0830         TableProp_female{i+1,cm} = num2str(length(OrganCompendium_female.(Ofemale{i}).modelAllComp.mets)*100/length(Recon3DHarvey.mets)); cm = cm + 1;
0831         
0832         TableProp_female{1,cm} = {'Metabolites (unique)'};
0833         [g,remR3M]=strtok(OrganCompendium_female.(Ofemale{i}).modelAllComp.mets,'[');
0834         TableProp_female{i+1,cm} = num2str(length(unique(g))); cm = cm + 1;
0835         
0836         TableProp_female{1,cm} = {'Percentage of all Metabolites (unique)'};
0837         [g,remR3M]=strtok(OrganCompendium_female.(Ofemale{i}).modelAllComp.mets,'[');
0838         [Mets]=strtok(female.mets,'[');
0839         TableProp_female{i+1,cm} = num2str(length(unique(g))*100/length(unique(Mets))); cm = cm + 1;
0840         
0841         % number of compartments
0842         TableProp_female{1,cm} = {'Compartments (unique)'};
0843         TableProp_female{i+1,cm} = num2str(length(unique(remR3M))); cm = cm + 1;
0844         
0845         % list of compartments
0846         TableProp_female{1,cm} = {'Compartment List (unique)'};
0847         C = unique(remR3M);
0848         for j= 1 : length(C)
0849             s= ' ';
0850             TableProp_female{i+1,cm} = strcat(TableProp_female{i+1,cm},',',s,C{j});
0851         end
0852         cm = cm + 1;
0853         
0854         %number of exchanges with [bc]
0855         TableProp_female{1,cm} = {'Number of exchanges with [bc]'};
0856         EX =find(~cellfun(@isempty,strfind(OrganCompendium_female.(Ofemale{i}).modelAllComp.rxns,'_EX_')));
0857         BC =find(~cellfun(@isempty,strfind(OrganCompendium_female.(Ofemale{i}).modelAllComp.rxns,'[bc]')));
0858         BCK =find(~cellfun(@isempty,strfind(OrganCompendium_female.(Ofemale{i}).modelAllComp.rxns,'[bcK]')));
0859         BC = intersect(EX,BC);
0860         BC = setdiff(BC,BCK);
0861         TableProp_female{i+1,cm} = num2str(length(BC)); cm = cm + 1;
0862         
0863         %percentage of all exchanges with [bc]
0864         TableProp_female{1,cm} = {'Percentage of all exchanges with [bc]'};
0865         EX =find(~cellfun(@isempty,strfind(OrganCompendium_female.(Ofemale{i}).modelAllComp.rxns,'_EX_')));
0866         BC =find(~cellfun(@isempty,strfind(OrganCompendium_female.(Ofemale{i}).modelAllComp.rxns,'[bc]')));
0867         BCK =find(~cellfun(@isempty,strfind(OrganCompendium_female.(Ofemale{i}).modelAllComp.rxns,'[bcK]')));
0868         BC = intersect(EX,BC);
0869         BC = setdiff(BC,BCK);
0870         TableProp_female{i+1,cm} = num2str(length(BC)*100/length(BC_mets_female)); cm = cm + 1;
0871         
0872         %number of exchanges with [bp]
0873         TableProp_female{1,cm} = {'Number of exchanges with [bp]'};
0874         EX =find(~cellfun(@isempty,strfind(OrganCompendium_female.(Ofemale{i}).modelAllComp.rxns,'_EX_')));
0875         BC =find(~cellfun(@isempty,strfind(OrganCompendium_female.(Ofemale{i}).modelAllComp.rxns,'[bp')));
0876         BC = intersect(EX,BC);
0877         TableProp_female{i+1,cm} = num2str(length(BC)); cm = cm + 1;
0878         
0879         %percentage of all exchanges with [bp]
0880         TableProp_female{1,cm} = {'Percentage of all exchanges with [bp]'};
0881         EX =find(~cellfun(@isempty,strfind(OrganCompendium_female.(Ofemale{i}).modelAllComp.rxns,'_EX_')));
0882         BC =find(~cellfun(@isempty,strfind(OrganCompendium_female.(Ofemale{i}).modelAllComp.rxns,'[bp')));
0883         BC = intersect(EX,BC);
0884         TableProp_female{i+1,cm} = num2str(length(BC)*100/length(BP_mets_female)); cm = cm + 1;
0885         
0886         %number of exchanges with [bd]
0887         TableProp_female{1,cm} = {'Number of exchanges with [bd]'};
0888         EX =find(~cellfun(@isempty,strfind(OrganCompendium_female.(Ofemale{i}).modelAllComp.rxns,'_EX_')));
0889         BC =find(~cellfun(@isempty,strfind(OrganCompendium_female.(Ofemale{i}).modelAllComp.rxns,'[bd')));
0890         BC = intersect(EX,BC);
0891         TableProp_female{i+1,cm} = num2str(length(BC)); cm = cm + 1;
0892         
0893         
0894         %percentage of all exchanges with [bd]
0895         TableProp_female{1,cm} = {'Percentage of all exchanges with [bd]'};
0896         EX =find(~cellfun(@isempty,strfind(OrganCompendium_female.(Ofemale{i}).modelAllComp.rxns,'_EX_')));
0897         BC =find(~cellfun(@isempty,strfind(OrganCompendium_female.(Ofemale{i}).modelAllComp.rxns,'[bd')));
0898         BC = intersect(EX,BC);
0899         TableProp_female{i+1,cm} = num2str(length(BC)*100/length(BD_mets_female)); cm = cm + 1;
0900         
0901         %number of exchanges with [lu]
0902         TableProp_female{1,cm} = {'Number of exchanges with [lu]'};
0903         EX =find(~cellfun(@isempty,strfind(OrganCompendium_female.(Ofemale{i}).modelAllComp.rxns,'_EX_')));
0904         BC =find(~cellfun(@isempty,strfind(OrganCompendium_female.(Ofemale{i}).modelAllComp.rxns,'[lu')));
0905         BC = intersect(EX,BC);
0906         TableProp_female{i+1,cm} = num2str(length(BC)); cm = cm + 1;
0907         
0908         
0909         %number of exchanges with [csf]
0910         TableProp_female{1,cm} = {'Number of exchanges with [csf]'};
0911         EX =find(~cellfun(@isempty,strfind(OrganCompendium_female.(Ofemale{i}).modelAllComp.rxns,'_EX_')));
0912         BC =find(~cellfun(@isempty,strfind(OrganCompendium_female.(Ofemale{i}).modelAllComp.rxns,'[csf')));
0913         BC = intersect(EX,BC);
0914         TableProp_female{i+1,cm} = num2str(length(BC)); cm = cm + 1;
0915         
0916         %percentage of all exchanges with [csf]
0917         TableProp_female{1,cm} = {'Percentage of all exchanges with [csf]'};
0918         EX =find(~cellfun(@isempty,strfind(OrganCompendium_female.(Ofemale{i}).modelAllComp.rxns,'_EX_')));
0919         BC =find(~cellfun(@isempty,strfind(OrganCompendium_female.(Ofemale{i}).modelAllComp.rxns,'[csf')));
0920         BC = intersect(EX,BC);
0921         TableProp_female{i+1,cm} = num2str(length(BC)*100/length(CSF_mets_female)); cm = cm + 1;
0922         
0923         %number of exchanges with [sw]
0924         TableProp_female{1,cm} = {'Number of exchanges with [sw]'};
0925         EX =find(~cellfun(@isempty,strfind(OrganCompendium_female.(Ofemale{i}).modelAllComp.rxns,'_EX_')));
0926         BC =find(~cellfun(@isempty,strfind(OrganCompendium_female.(Ofemale{i}).modelAllComp.rxns,'[sw')));
0927         BC = intersect(EX,BC);
0928         TableProp_female{i+1,cm} = num2str(length(BC)); cm = cm + 1;
0929         
0930         %number of exchanges with [a]
0931         TableProp_female{1,cm} = {'Number of exchanges with [a]'};
0932         EX =find(~cellfun(@isempty,strfind(OrganCompendium_female.(Ofemale{i}).modelAllComp.rxns,'_EX_')));
0933         BC =find(~cellfun(@isempty,strfind(OrganCompendium_female.(Ofemale{i}).modelAllComp.rxns,'[a')));
0934         BC = intersect(EX,BC);
0935         TableProp_female{i+1,cm} = num2str(length(BC)); cm = cm + 1;
0936         
0937         %number of exchanges with [u]
0938         TableProp_female{1,cm} = {'Number of exchanges with [u]'};
0939         EX =find(~cellfun(@isempty,strfind(OrganCompendium_female.(Ofemale{i}).modelAllComp.rxns,'_EX_')));
0940         BC =find(~cellfun(@isempty,strfind(OrganCompendium_female.(Ofemale{i}).modelAllComp.rxns,'[u]')));
0941         BC = intersect(EX,BC);
0942         TableProp_female{i+1,cm} = num2str(length(BC)); cm = cm + 1;
0943         
0944         %percentage of all exchanges with [u]
0945         TableProp_female{1,cm} = {'Percentage of all exchanges with [u]'};
0946         EX =find(~cellfun(@isempty,strfind(OrganCompendium_female.(Ofemale{i}).modelAllComp.rxns,'_EX_')));
0947         BC =find(~cellfun(@isempty,strfind(OrganCompendium_female.(Ofemale{i}).modelAllComp.rxns,'[u]')));
0948         BC = intersect(EX,BC);
0949         TableProp_female{i+1,cm} = num2str(length(BC)*100/length(U_mets_female)); cm = cm + 1;
0950         
0951         %[bc] overlap with microbiota metabolites
0952         TableProp_female{1,cm} = {'Percentage of overlap of [bc] with microbiota metabolites'};
0953         EX =find(~cellfun(@isempty,strfind(OrganCompendium_female.(Ofemale{i}).modelAllComp.rxns,'_EX_')));
0954         BC =find(~cellfun(@isempty,strfind(OrganCompendium_female.(Ofemale{i}).modelAllComp.rxns,'[bc')));
0955         BC = intersect(EX,BC);
0956         BCM = OrganCompendium_female.(Ofemale{i}).modelAllComp.rxns(BC);
0957         BCM = regexprep(BCM,'Tr_EX_','');
0958         BCM = regexprep(BCM,'\(e.+','');
0959         BCM_MY = intersect(BCM,MyU_rxns);
0960         if ~isempty(BC)
0961             TableProp_female{i+1,cm} = num2str(length(BCM_MY)*100/length(BC)); cm = cm + 1;
0962         else
0963             TableProp_female{i+1,cm} = 'NA'; cm = cm + 1;
0964         end
0965         
0966         %[bp] overlap with microbiota metabolites
0967         TableProp_female{1,cm} = {'Percentage of overlap of [bp] with microbiota metabolites'};
0968         EX =find(~cellfun(@isempty,strfind(OrganCompendium_female.(Ofemale{i}).modelAllComp.rxns,'_EX_')));
0969         BC =find(~cellfun(@isempty,strfind(OrganCompendium_female.(Ofemale{i}).modelAllComp.rxns,'[bp')));
0970         BC = intersect(EX,BC);
0971         BCM = OrganCompendium_female.(Ofemale{i}).modelAllComp.rxns(BC);
0972         BCM = regexprep(BCM,'Tr_EX_','');
0973         BCM = regexprep(BCM,'\[bp.+','');
0974         BCM_MY = intersect(BCM,MyU_rxns);
0975         if ~isempty(BC)
0976             TableProp_female{i+1,cm} = num2str(length(BCM_MY)*100/length(BC)); cm = cm + 1;
0977         else
0978             TableProp_female{i+1,cm} = 'NA'; cm = cm + 1;
0979         end
0980         
0981         %[bd] overlap with microbiota metabolites
0982         TableProp_female{1,cm} = {'Percentage of overlap of [bd] with microbiota metabolites'};
0983         EX =find(~cellfun(@isempty,strfind(OrganCompendium_female.(Ofemale{i}).modelAllComp.rxns,'_EX_')));
0984         BC =find(~cellfun(@isempty,strfind(OrganCompendium_female.(Ofemale{i}).modelAllComp.rxns,'[bd')));
0985         BC = intersect(EX,BC);
0986         BCM = OrganCompendium_female.(Ofemale{i}).modelAllComp.rxns(BC);
0987         BCM = regexprep(BCM,'Tr_EX_','');
0988         BCM = regexprep(BCM,'\[bd.+','');
0989         BCM_MY = intersect(BCM,MyU_rxns);
0990         if ~isempty(BC)
0991             TableProp_female{i+1,cm} = num2str(length(BCM_MY)*100/length(BC)); cm = cm + 1;
0992         else
0993             TableProp_female{i+1,cm} = 'NA'; cm = cm + 1;
0994         end
0995         
0996         %[csf] overlap with microbiota metabolites
0997         TableProp_female{1,cm} = {'Percentage of overlap of [csf] with microbiota metabolites'};
0998         EX =find(~cellfun(@isempty,strfind(OrganCompendium_female.(Ofemale{i}).modelAllComp.rxns,'_EX_')));
0999         BC =find(~cellfun(@isempty,strfind(OrganCompendium_female.(Ofemale{i}).modelAllComp.rxns,'[csf')));
1000         BC = intersect(EX,BC);
1001         BCM = OrganCompendium_female.(Ofemale{i}).modelAllComp.rxns(BC);
1002         BCM = regexprep(BCM,'Tr_EX_','');
1003         BCM = regexprep(BCM,'\(e.+','');
1004         BCM_MY = intersect(BCM,MyU_rxns);
1005         if ~isempty(BC)
1006             TableProp_female{i+1,cm} = num2str(length(BCM_MY)*100/length(BC)); cm = cm + 1;
1007         else
1008             TableProp_female{i+1,cm} = 'NA'; cm = cm + 1;
1009         end
1010         
1011         %[u] overlap with microbiota metabolites
1012         TableProp_female{1,cm} = {'Percentage of overlap of [u] with microbiota metabolites'};
1013         EX =find(~cellfun(@isempty,strfind(OrganCompendium_female.(Ofemale{i}).modelAllComp.rxns,'_EX_')));
1014         BC =find(~cellfun(@isempty,strfind(OrganCompendium_female.(Ofemale{i}).modelAllComp.rxns,'[u')));
1015         BC = intersect(EX,BC);
1016         BCM = OrganCompendium_female.(Ofemale{i}).modelAllComp.rxns(BC);
1017         BCM = regexprep(BCM,'Tr_EX_','');
1018         BCM = regexprep(BCM,'\(e.+','');
1019         BCM_MY = intersect(BCM,MyU_rxns);
1020         if ~isempty(BC)
1021             TableProp_female{i+1,cm} = num2str(length(BCM_MY)*100/length(BC)); cm = cm + 1;
1022         else
1023             TableProp_female{i+1,cm} = 'NA'; cm = cm + 1;
1024         end
1025         
1026         %[u] overlap with microbiota metabolites
1027         TableProp_female{1,cm} = {'Percentage of overlap of [u] with microbiota metabolites'};
1028         EX =find(~cellfun(@isempty,strfind(OrganCompendium_female.(Ofemale{i}).modelAllComp.rxns,'_EX_')));
1029         BC =find(~cellfun(@isempty,strfind(OrganCompendium_female.(Ofemale{i}).modelAllComp.rxns,'[u')));
1030         BC = intersect(EX,BC);
1031         BCM = OrganCompendium_female.(Ofemale{i}).modelAllComp.rxns(BC);
1032         BCM = regexprep(BCM,'Tr_EX_','');
1033         BCM = regexprep(BCM,'\(e.+','');
1034         BCM_MY = intersect(BCM,MyU_rxns);
1035         if ~isempty(BC)
1036             TableProp_female{i+1,cm} = num2str(length(BCM_MY)*100/length(BC)); cm = cm + 1;
1037         else
1038             TableProp_female{i+1,cm} = 'NA'; cm = cm + 1;
1039         end
1040         
1041         %[lu] overlap with microbiota metabolites
1042         TableProp_female{1,cm} = {'Percentage of overlap of [lu] with microbiota metabolites'};
1043         EX =find(~cellfun(@isempty,strfind(OrganCompendium_female.(Ofemale{i}).modelAllComp.rxns,'_EX_')));
1044         BC =find(~cellfun(@isempty,strfind(OrganCompendium_female.(Ofemale{i}).modelAllComp.rxns,'[lu')));
1045         BC = intersect(EX,BC);
1046         BCM = OrganCompendium_female.(Ofemale{i}).modelAllComp.rxns(BC);
1047         BCM = regexprep(BCM,'Tr_EX_','');
1048         BCM = regexprep(BCM,'\[lu.+','');
1049         BCM_MY = intersect(BCM,MyU_rxns);
1050         if ~isempty(BC)
1051             TableProp_female{i+1,cm} = num2str(length(BCM_MY)*100/length(BC)); cm = cm + 1;
1052         else
1053             TableProp_female{i+1,cm} = 'NA'; cm = cm + 1;
1054         end
1055         
1056         TableProp_female{1,cm} = {'Transcripts'};
1057         TableProp_female{i+1,cm} = num2str(length(OrganCompendium_female.(Ofemale{i}).modelAllComp.genes)); cm = cm + 1;
1058         
1059         TableProp_female{1,cm} = {'Genes (unique)'};
1060         [g,rem]=strtok(OrganCompendium_female.(Ofemale{i}).modelAllComp.genes,'.');
1061         TableProp_female{i+1,cm} = num2str(length(unique(g))); cm = cm + 1;
1062         
1063         TableProp_female{1,cm} = {'Number Genes Associated Reactions'};
1064         str = length(find(~cellfun(@isempty,OrganCompendium_female.(Ofemale{i}).modelAllComp.grRules)));
1065         TableProp_female{i+1,cm} = num2str(str); cm = cm + 1;
1066         
1067         TableProp_female{1,cm} = {'Percentage Genes Associated Reactions (Exchange Rxns excl)'};
1068         str = length(find(~cellfun(@isempty,OrganCompendium_female.(Ofemale{i}).modelAllComp.grRules)));
1069         TableProp_female{i+1,cm} = num2str(str*100/NoEx); cm = cm + 1;
1070         PercGeneAssRxns_female(i,1) = str*100/NoEx;
1071         
1072 %         TableProp_female{1,cm} = {'Subsystems'};
1073 %         if isempty(OrganCompendium_female.(Ofemale{i}).modelAllComp.subSystems{1})
1074 %             TableProp_female{i+1,cm}=NaN;
1075 %         else
1076 %             TableProp_female{i+1,cm} = num2str(length(unique(OrganCompendium_female.(Ofemale{i}).modelAllComp.subSystems))); cm = cm + 1;
1077 %         end
1078         
1079         TableProp_female{1,cm} = {'Size of S'};
1080         TableProp_female{i+1,cm} = strcat(num2str(size(OrganCompendium_female.(Ofemale{i}).modelAllComp.S,1)),'; ',num2str(size(OrganCompendium_female.(Ofemale{i}).modelAllComp.S,2))); cm = cm + 1;
1081         
1082         % rank of S
1083         TableProp_female{1,cm} = {'Rank of S'};
1084         %     TableProp_female{i+1,cm} = strcat(num2str(rank(full(OrganCompendium_female.(Ofemale{i}).modelAllComp.S)))); cm = cm + 1;
1085         
1086     end
1087 end
1088 TableProp_female=TableProp_female';
1089 
1090 
1091 %% compare reaction content
1092 clear TableRxns TableRxnsNum
1093 %Rxns = Recon3DHarvey.rxns;
1094 
1095 Rxns = [];
1096 % get set of unique reactions
1097 for i = 1 : length(Ofemale)
1098     if ~strcmp('sex',Ofemale{i}) && ~strcmp('gender',Ofemale{i}) && ~strcmp('Recon3DHarvey',Ofemale{i})
1099         % grab reactions
1100         ORxns = OrganCompendium_female.(Ofemale{i}).modelAllComp.rxns;
1101         ORxns = regexprep(ORxns,'Tr_','');
1102         ORxns = regexprep(ORxns,'_\[\w+\]','');
1103         ORxns = regexprep(ORxns,'\[\w\w\w\]','');
1104         ORxns = regexprep(ORxns,'\[\w\w\]','');
1105         ORxns = regexprep(ORxns,'\(','\[');
1106         ORxns = regexprep(ORxns,'\)','\]');
1107         ORxns = regexprep(ORxns,'_c_','\[c\]');
1108         ORxns = regexprep(ORxns,'_g_','\[g\]');
1109         ORxns = regexprep(ORxns,'_n_','\[n\]');
1110         ORxns = regexprep(ORxns,'_m_','\[m\]');
1111         ORxns = regexprep(ORxns,'_r_','\[r\]');
1112         ORxns = regexprep(ORxns,'_x_','\[x\]');
1113         ORxns = regexprep(ORxns,'\[u\]','');
1114         ORxns = regexprep(ORxns,'\[e\]','');
1115         ORxns = regexprep(ORxns,'\[mi\w\]','');
1116         ORxns = regexprep(ORxns,'\[sw\w\]','');
1117         ORxns = regexprep(ORxns,'\[lu\w\w\]','');
1118         Rxns = [Rxns;ORxns];
1119     end
1120 end
1121 Rxns = unique(Rxns);
1122 
1123 TableRxns_female(1:length(Rxns),1) = (Rxns);
1124 for i = 1 : length(Ofemale)
1125     if ~strcmp('sex',Ofemale{i}) && ~strcmp('gender',Ofemale{i}) && ~strcmp('Recon3DHarvey',Ofemale{i})
1126         % grab reactions
1127         ORxns = OrganCompendium_female.(Ofemale{i}).modelAllComp.rxns;
1128         ORxns = regexprep(ORxns,'Tr_','');
1129         ORxns = regexprep(ORxns,'_\[\w+\]','');
1130         ORxns = regexprep(ORxns,'\[\w\w\w\]','');
1131         ORxns = regexprep(ORxns,'\[\w\w\]','');
1132         ORxns = regexprep(ORxns,'\(','\[');
1133         ORxns = regexprep(ORxns,'\)','\]');
1134         ORxns = regexprep(ORxns,'_c_','\[c\]');
1135         ORxns = regexprep(ORxns,'_g_','\[g\]');
1136         ORxns = regexprep(ORxns,'_n_','\[n\]');
1137         ORxns = regexprep(ORxns,'_m_','\[m\]');
1138         ORxns = regexprep(ORxns,'_r_','\[r\]');
1139         ORxns = regexprep(ORxns,'_x_','\[x\]');
1140         ORxns = regexprep(ORxns,'\[u\]','');
1141         ORxns = regexprep(ORxns,'\[e\]','');
1142         ORxns = regexprep(ORxns,'\[mi\w\]','');
1143         ORxns = regexprep(ORxns,'\[sw\w\]','');
1144         ORxns = regexprep(ORxns,'\[lu\w\w\]','');
1145         ORxns = regexprep(ORxns,'_DIFF\[c\]','_DIFF');
1146         TableRxns_female(:,i+1)=num2cell(0);
1147         TableRxns_femaleO{1,i+1} = Ofemale{i};
1148         TableRxns_female(find(ismember(Rxns,ORxns)),i+1)=num2cell(1);
1149         TableRxnsNum_female(find(ismember(Rxns,ORxns)),i)=1;
1150     end
1151 end
1152 TableRxns_female = [TableRxns_femaleO;TableRxns_female];
1153 % correlation
1154 TableRxnsNumCorr_female = corrcoef(TableRxnsNum_female);
1155 
1156 % shared reactions
1157 TableRxns_femaleNumInOrgans = TableRxnsNum_female*TableRxnsNum_female';
1158 RxnsInOrgans_female = diag(TableRxns_femaleNumInOrgans);
1159 HousekeepingRxns_female = Rxns(find(RxnsInOrgans_female>=length(Ofemale)-2));%2 entries of Ofemale are no organs
1160 OrganSpecRxns_female = Rxns(find(RxnsInOrgans_female==1));%at most in 2 organs
1161 NoOrgaRxns_female = Rxns(find(RxnsInOrgans_female==0));%at most in 2 organs
1162 OtherOrganRxns_female = Rxns(find(RxnsInOrgans_female>1 & RxnsInOrgans_female<length(Ofemale)-2));%at most in 2 organs
1163 s = {' '};
1164 sumRxns = length(NoOrgaRxns_female)+length(OtherOrganRxns_female)+length(OrganSpecRxns_female)+length(HousekeepingRxns_female);
1165 TableGRM{1,5} = 'Female';
1166 TableGRM{1,8} = 'Reactions';
1167 TableGRM{2,5} = 'core';
1168 TableGRM{2,8} = strcat(num2str(length(HousekeepingRxns_female)),s,'(',num2str(round(length(HousekeepingRxns_female)*100/sumRxns,1)),'%)');
1169 TableGRM{3,5} = 'organ-specific';
1170 TableGRM{3,8} = strcat(num2str(length(OrganSpecRxns_female)),s,'(',num2str(round(length(OrganSpecRxns_female)*100/sumRxns,1)),'%)');
1171 TableGRM{4,5} = 'others';
1172 TableGRM{4,8} = strcat(num2str(length(OtherOrganRxns_female)),s,'(',num2str(round(length(OtherOrganRxns_female)*100/sumRxns,1)),'%)');
1173 TableGRM{5,5} = 'absent';
1174 TableGRM{5,8} = strcat(num2str(length(NoOrgaRxns_female)),s,'(',num2str(round(length(NoOrgaRxns_female)*100/sumRxns,1)),'%)');
1175 TableGRM{6,5} = 'sum';
1176 TableGRM{6,8} = num2str(sumRxns);
1177 
1178 
1179 for i = 1 : length(Ofemale)
1180     if ~strcmp('sex',Ofemale{i}) && ~strcmp('gender',Ofemale{i}) && ~strcmp('Recon3DHarvey',Ofemale{i})
1181         % grab reactions
1182         ORxns = OrganCompendium_female.(Ofemale{i}).modelAllComp.rxns;
1183         HouseCoreORxns_female(i,1) = length(intersect(HousekeepingRxns_female ,ORxns))*100/length(ORxns);
1184         HouseCoreORxns_female(i,2) = length(intersect(OrganSpecRxns_female ,ORxns))*100/length(ORxns);
1185         CoreOrganRxns_female(i,1) = length(intersect(OrganSpecRxns_female ,ORxns));
1186     end
1187 end
1188 
1189 
1190 if 0
1191     % Create figure
1192     figure1 = figure;
1193     
1194     % Create axes
1195     axes1 = axes('Parent',figure1);
1196     hold(axes1,'on');
1197     % Create multiple lines using matrix input to bar
1198     %bar1 = bar(ymatrix1,'BarLayout','stacked','Parent',axes1);
1199     bar1 = bar(-1*[[HouseCoreORxns_female(1:24,1);0;0;HouseCoreORxns_female(25:end,1)] [HouseCoreORxns_female(1:24,2);0;0;HouseCoreORxns_female(25:end,2)]],'stacked','Parent',axes1);
1200     hold on
1201     bar2 =   bar(1*[[HouseCoreORxns_male(1:20,1);0;0;0;0;HouseCoreORxns_male(21:end,1)] [HouseCoreORxns_male(1:20,2);0;0;0;0;HouseCoreORxns_male(21:end,2)]],'stacked','Parent',axes1);
1202     
1203     set(bar2(2),'FaceColor',[0 0.447058826684952 0.74117648601532]);
1204     set(bar2(1),'FaceColor',[0.678431391716003 0.921568632125854 1]);
1205     set(bar1(2),'FaceColor',[1 0.600000023841858 0.7843137383461]);
1206     set(bar1(1),'FaceColor',[1 0.843137264251709 0]);
1207     
1208     box(axes1,'on');
1209     % Set the remaining axes properties
1210     set(axes1,'FontSize',16,'XAxisLocation','top','XDir','reverse','XTick',[1:32],'XTickLabel',...
1211         [Omale(1:20);Ofemale(21:24);Omale(21:end)],'XTickLabelRotation',270);
1212     
1213 end
1214 
1215 clear TableMets_female TableMetsNum_female
1216 %Mets = Recon3DHarvey.mets;
1217 Mets = [];
1218 % get set of unique reactions
1219 for i = 1 : length(Ofemale)
1220     if ~strcmp('sex',Ofemale{i}) && ~strcmp('gender',Ofemale{i}) && ~strcmp('Recon3DHarvey',Ofemale{i})
1221         % grab reactions
1222         OMets = OrganCompendium_female.(Ofemale{i}).modelAllComp.mets;
1223         OMets = regexprep(OMets,'\[b\w\w\]','\[e\]');
1224         OMets = regexprep(OMets,'\[b\w\]','\[e\]');
1225         OMets = regexprep(OMets,'\[u\]','\[e\]');
1226         OMets = regexprep(OMets,'\[u\]','\[e\]');
1227         OMets = regexprep(OMets,'\[lu\]','\[e\]');
1228         OMets = regexprep(OMets,'\[lu\w+\]','\[e\]');
1229         OMets = regexprep(OMets,'\[csf\]','\[e\]');
1230         OMets = regexprep(OMets,'\[s\w+\]','\[e\]');
1231         OMets = regexprep(OMets,'\[fe\]','\[e\]');
1232         OMets = regexprep(OMets,'\[mi\w]','\[e\]');
1233         OMets = regexprep(OMets,'\[mi]','\[e\]');
1234         OMets = regexprep(OMets,'\[a\]','\[e\]');
1235         OMets = regexprep(OMets,'\[e\]','');
1236         OMets = regexprep(OMets,'\[c\]','');
1237         OMets = regexprep(OMets,'\[m\]','');
1238         OMets = regexprep(OMets,'\[r\]','');
1239         OMets = regexprep(OMets,'\[g\]','');
1240         OMets = regexprep(OMets,'\[x\]','');
1241         OMets = regexprep(OMets,'\[n\]','');
1242         OMets = regexprep(OMets,'\[l\]','');
1243         Mets = [Mets;OMets];
1244     end
1245 end
1246 Mets = unique(Mets);
1247 
1248 TableMets_female(1:length(Mets),1) = (Mets);
1249 for i = 1 : length(Ofemale)
1250     if ~strcmp('sex',Ofemale{i}) && ~strcmp('gender',Ofemale{i}) && ~strcmp('Recon3DHarvey',Ofemale{i})
1251         % grab reactions
1252         OMets = OrganCompendium_female.(Ofemale{i}).modelAllComp.mets;
1253         OMets = regexprep(OMets,'\[b\w\w\]','\[e\]');
1254         OMets = regexprep(OMets,'\[b\w\]','\[e\]');
1255         OMets = regexprep(OMets,'\[u\]','\[e\]');
1256         OMets = regexprep(OMets,'\[u\]','\[e\]');
1257         OMets = regexprep(OMets,'\[lu\]','\[e\]');
1258         OMets = regexprep(OMets,'\[lu\w+\]','\[e\]');
1259         OMets = regexprep(OMets,'\[csf\]','\[e\]');
1260         OMets = regexprep(OMets,'\[s\w+\]','\[e\]');
1261         OMets = regexprep(OMets,'\[fe\]','\[e\]');
1262         OMets = regexprep(OMets,'\[mi\w]','\[e\]');
1263         OMets = regexprep(OMets,'\[mi]','\[e\]');
1264         OMets = regexprep(OMets,'\[a\]','\[e\]');
1265         OMets = regexprep(OMets,'\[e\]','');
1266         OMets = regexprep(OMets,'\[c\]','');
1267         OMets = regexprep(OMets,'\[m\]','');
1268         OMets = regexprep(OMets,'\[r\]','');
1269         OMets = regexprep(OMets,'\[g\]','');
1270         OMets = regexprep(OMets,'\[x\]','');
1271         OMets = regexprep(OMets,'\[n\]','');
1272         OMets = regexprep(OMets,'\[l\]','');
1273         TableMets_female(:,i+1)=num2cell(0);
1274         TableMets_female(find(ismember(Mets,OMets)),i+1)=num2cell(1);
1275         TableMets_femaleO{1,i+1}=Ofemale{i};
1276         TableMetsNum_female(find(ismember(Mets,OMets)),i)=1;
1277     end
1278 end
1279 TableMets_female_unique = [TableMets_femaleO;TableMets_female];
1280 
1281 
1282 
1283 Mets = [];
1284 % get set of unique reactions
1285 for i = 1 : length(Ofemale)
1286     if ~strcmp('sex',Ofemale{i}) && ~strcmp('gender',Ofemale{i}) && ~strcmp('Recon3DHarvey',Ofemale{i})
1287         % grab reactions
1288         OMets = OrganCompendium_female.(Ofemale{i}).modelAllComp.mets;
1289         OMets = regexprep(OMets,'\[b\w\w\]','\[e\]');
1290         OMets = regexprep(OMets,'\[b\w\]','\[e\]');
1291         OMets = regexprep(OMets,'\[u\]','\[e\]');
1292         OMets = regexprep(OMets,'\[u\]','\[e\]');
1293         OMets = regexprep(OMets,'\[lu\]','\[e\]');
1294         OMets = regexprep(OMets,'\[lu\w+\]','\[e\]');
1295         OMets = regexprep(OMets,'\[csf\]','\[e\]');
1296         OMets = regexprep(OMets,'\[s\w+\]','\[e\]');
1297         OMets = regexprep(OMets,'\[fe\]','\[e\]');
1298         OMets = regexprep(OMets,'\[mi\w]','\[e\]');
1299         OMets = regexprep(OMets,'\[mi]','\[e\]');
1300         OMets = regexprep(OMets,'\[a\]','\[e\]');
1301         Mets = [Mets;OMets];
1302     end
1303 end
1304 Mets = unique(Mets);
1305 
1306 TableMets_female(1:length(Mets),1) = (Mets);
1307 for i = 1 : length(Ofemale)
1308     if ~strcmp('sex',Ofemale{i}) && ~strcmp('gender',Ofemale{i}) && ~strcmp('Recon3DHarvey',Ofemale{i})
1309         % grab reactions
1310         OMets = OrganCompendium_female.(Ofemale{i}).modelAllComp.mets;
1311         OMets = regexprep(OMets,'\[b\w\w\]','\[e\]');
1312         OMets = regexprep(OMets,'\[b\w\]','\[e\]');
1313         OMets = regexprep(OMets,'\[u\]','\[e\]');
1314         OMets = regexprep(OMets,'\[u\]','\[e\]');
1315         OMets = regexprep(OMets,'\[lu\]','\[e\]');
1316         OMets = regexprep(OMets,'\[lu\w+\]','\[e\]');
1317         OMets = regexprep(OMets,'\[csf\]','\[e\]');
1318         OMets = regexprep(OMets,'\[s\w+\]','\[e\]');
1319         OMets = regexprep(OMets,'\[fe\]','\[e\]');
1320         OMets = regexprep(OMets,'\[mi\w]','\[e\]');
1321         OMets = regexprep(OMets,'\[mi]','\[e\]');
1322         OMets = regexprep(OMets,'\[a\]','\[e\]');
1323         TableMets_female(:,i+1)=num2cell(0);
1324         TableMets_female(find(ismember(Mets,OMets)),i+1)=num2cell(1);
1325         TableMets_femaleO{1,i+1}=Ofemale{i};
1326         TableMetsNum_female(find(ismember(Mets,OMets)),i)=1;
1327     end
1328 end
1329 TableMets_female = [TableMets_femaleO;TableMets_female];
1330 
1331 % correlation
1332 TableMetsNum_femaleCorr = corrcoef(TableMetsNum_female);
1333 
1334 % shared metabolites
1335 TableMets_femaleNumInOrgans = TableMetsNum_female*TableMetsNum_female';
1336 MetsInOrgans_female = diag(TableMets_femaleNumInOrgans);
1337 HousekeepingMets_female = Mets(find(MetsInOrgans_female>=length(Ofemale)-2));%2 entries of Ofemale are no organs
1338 OrganSpecMets_female = Mets(find(MetsInOrgans_female==1));%at most in 2 organs
1339 NoOrgaMets_female = Mets(find(MetsInOrgans_female==0));%at most in 2 organs
1340 OtherOrganMets_female = Mets(find(MetsInOrgans_female>1 & MetsInOrgans_female<length(Ofemale)-2));%at most in 2 organs
1341 sumMets = length(NoOrgaMets_female)+length(OtherOrganMets_female)+length(OrganSpecMets_female)+length(HousekeepingMets_female);
1342 s = {' '};
1343 TableGRM{1,7} = 'Metabolites';
1344 TableGRM{2,5} = 'core';
1345 TableGRM{2,7} = strcat(num2str(length(HousekeepingMets_female)),s,'(',num2str(round(length(HousekeepingMets_female)*100/sumMets,1)),'%)');
1346 TableGRM{3,5} = 'organ-specific';
1347 TableGRM{3,7} = strcat(num2str(length(OrganSpecMets_female)),s,'(',num2str(round(length(OrganSpecMets_female)*100/sumMets,1)),'%)');
1348 TableGRM{4,5} = 'others';
1349 TableGRM{4,7} = strcat(num2str(length(OtherOrganMets_female)),s,'(',num2str(round(length(OtherOrganMets_female)*100/sumMets,1)),'%)');
1350 TableGRM{5,5} = 'absent';
1351 TableGRM{5,7} = strcat(num2str(length(NoOrgaMets_female)),s,'(',num2str(round(length(NoOrgaMets_female)*100/sumMets,1)),'%)');
1352 TableGRM{6,5} = 'sum';
1353 TableGRM{6,7} = num2str(sumMets);
1354 
1355 clear TableGenes_female TableGenes_femaleNum
1356 %[Genes,rem]=strtok(Recon3DHarvey.genes,'.');
1357 Genes = [];
1358 % get set of unique reactions
1359 for i = 1 : length(Ofemale)
1360     if ~strcmp('sex',Ofemale{i}) && ~strcmp('gender',Ofemale{i}) && ~strcmp('Recon3DHarvey',Ofemale{i})
1361         % grab reactions
1362         OGenes = OrganCompendium_female.(Ofemale{i}).modelAllComp.genes;
1363         Genes = [Genes;OGenes];
1364     end
1365 end
1366 %[Genes,rem]=strtok(Genes,'.');
1367 Genes = unique(Genes);
1368 
1369 TableGenes_female(1:length(Genes),1) = (Genes);
1370 for i = 1 : length(Ofemale)
1371     if ~strcmp('sex',Ofemale{i}) && ~strcmp('gender',Ofemale{i}) && ~strcmp('Recon3DHarvey',Ofemale{i})
1372         % grab reactions
1373         %[OGenes,rem]=strtok(OrganCompendium_female.(Ofemale{i}).modelAllComp.genes,'.');
1374       %  OGenes = unique(OGenes);
1375           OGenes = OrganCompendium_female.(Ofemale{i}).modelAllComp.genes;
1376         TableGenes_female(:,i+1)=num2cell(0);
1377         TableGenes_female(find(ismember(Genes,OGenes)),i+1)=num2cell(1);
1378         TableGenes_femaleO{1,i+1} = Ofemale{i};
1379         TableGenes_femaleNum(find(ismember(Genes,OGenes)),i)=1;
1380     end
1381 end
1382 TableGenes_female = [TableGenes_femaleO;TableGenes_female];
1383 % correlation
1384 TableGenes_femaleNumCorr = corrcoef(TableGenes_femaleNum);
1385 % get stats
1386 
1387 % shared genes
1388 TableGenes_femaleNumInOrgans = TableGenes_femaleNum*TableGenes_femaleNum';
1389 GenesInOrgans_female = diag(TableGenes_femaleNumInOrgans);
1390 HousekeepingGenes_female = Genes(find(GenesInOrgans_female>=length(Ofemale)-2));%2 entries of Ofemale are no organs
1391 OrganSpecGenes_female = Genes(find(GenesInOrgans_female==1));%at most in 2 organs
1392 NoOrganGenes_female = Genes(find(GenesInOrgans_female==0));%at most in 2 organs
1393 OtherOrganGenes_female = Genes(find(GenesInOrgans_female>1 & GenesInOrgans_female<length(Ofemale)-2));%at most in 2 organs
1394 sumGenes = length(NoOrganGenes_female)+length(OtherOrganGenes_female)+length(OrganSpecGenes_female)+length(HousekeepingGenes_female);
1395 s = {' '};
1396 TableGRM{1,6} = 'Genes';
1397 TableGRM{2,5} = 'core';
1398 TableGRM{2,6} = strcat(num2str(length(HousekeepingGenes_female)),s,'(',num2str(round(length(HousekeepingGenes_female)*100/sumGenes,1)),'%)');
1399 TableGRM{3,5} = 'organ-specific';
1400 TableGRM{3,6} = strcat(num2str(length(OrganSpecGenes_female)),s,'(',num2str(round(length(OrganSpecGenes_female)*100/sumGenes,1)),'%)');
1401 TableGRM{4,5} = 'others';
1402 TableGRM{4,6} = strcat(num2str(length(OtherOrganGenes_female)),s,'(',num2str(round(length(OtherOrganGenes_female)*100/sumGenes,1)),'%)');
1403 TableGRM{5,5} = 'absent';
1404 TableGRM{5,6} = strcat(num2str(length(NoOrganGenes_female)),s,'(',num2str(round(length(NoOrganGenes_female)*100/sumGenes,1)),'%)');
1405 TableGRM{6,5} = 'sum';
1406 TableGRM{6,6} = num2str(sumGenes);
1407 
1408 if 0
1409     figure
1410     imagesc(TableRxnsNumCorr_female)
1411     figure
1412     imagesc(TableRxnsNum_female)
1413     figure
1414     imagesc(TableGenes_femaleNumCorr)
1415     figure
1416     imagesc(TableGenes_femaleNum)
1417     
1418     figure
1419     imagesc(TableMetsNum_femaleCorr)
1420     figure
1421     imagesc(TableMetsNum_female)
1422 end
1423 
1424 % get stats table
1425 clear TableAverage_female
1426 c = 1;
1427 TableAverage_female{c,1} = {'Average number of reactions'};
1428 Sum = sum(TableRxnsNum_female);
1429 SumR_female = Sum;
1430 TableAverage_female{c,2} = num2str(mean(Sum));c= c+1;
1431 TableAverage_female{c,1} = {'Std number of reactions'};
1432 TableAverage_female{c,2} = num2str(std(Sum));c= c+1;
1433 TableAverage_female{c,1} = {'Min number of reactions'};
1434 TableAverage_female{c,2} = num2str(min(Sum));c= c+1;
1435 TableAverage_female{c,1} = {'Max number of reactions'};
1436 TableAverage_female{c,2} = num2str(max(Sum));c= c+1;
1437 
1438 TableAverage_female{c,1} = {'Average number of metabolites'};
1439 Sum = sum(TableMetsNum_female);
1440 SumM_female = Sum;
1441 TableAverage_female{c,2} = num2str(mean(Sum));c= c+1;
1442 TableAverage_female{c,1} = {'Std number of metabolites'};
1443 TableAverage_female{c,2} = num2str(std(Sum));c= c+1;
1444 TableAverage_female{c,1} = {'Min number of metabolites'};
1445 TableAverage_female{c,2} = num2str(min(Sum));c= c+1;
1446 TableAverage_female{c,1} = {'Max number of metabolites'};
1447 TableAverage_female{c,2} = num2str(max(Sum));c= c+1;
1448 
1449 TableAverage_female{c,1} = {'Average number of genes'};
1450 Sum = sum(TableGenes_femaleNum);
1451 SumG_female = Sum;
1452 TableAverage_female{c,2} = num2str(mean(Sum));c= c+1;
1453 TableAverage_female{c,1} = {'Std number of genes'};
1454 TableAverage_female{c,2} = num2str(std(Sum));c= c+1;
1455 TableAverage_female{c,1} = {'Min number of genes'};
1456 TableAverage_female{c,2} = num2str(min(Sum));c= c+1;
1457 TableAverage_female{c,1} = {'Max number of genes'};
1458 TableAverage_female{c,2} = num2str(max(Sum));c= c+1;
1459 
1460 % does not work on 2013
1461 if violinPlots == 1
1462     figure
1463     vs = violinplot([SumR_female' SumM_female' SumG_female'], {'Reactions';'Metabolites';'Genes (unique)'});
1464     
1465     
1466     figure;
1467     subplot(2,4,1);
1468     vs = violinplot([SumR_male'  ], {'Male'});
1469     title('Reactions');
1470     subplot(2,4,2);
1471     vs = violinplot([SumM_male'  ], {'Male'});
1472     title('Metabolites');
1473     subplot(2,4,3);
1474     vs = violinplot([SumG_male'  ], {'Male'});
1475     title('Genes');
1476     subplot(2,4,4);
1477     vs = violinplot(PercGeneAssRxns_male , {'Male'});
1478     title('Gene-associated Reactions');
1479     subplot(2,4,5);
1480     vs = violinplot([SumR_female' ], {'Female'});
1481     title('Reactions');
1482     subplot(2,4,6);
1483     vs = violinplot([ SumM_female' ], {'Female'});
1484     title('Metabolites');
1485     subplot(2,4,7);
1486     vs = violinplot([ SumG_female' ], {'Female'});
1487     title('Genes');
1488     subplot(2,4,8);
1489     vs = violinplot(PercGeneAssRxns_female , {'Female'});
1490     title('Gene-associated Reactions');
1491     
1492 end
1493 
1494 %figures
1495 genesPerOrganFigure(GenesInOrgans_male, GenesInOrgans_female);
1496 
1497 if 0
1498     if 0 %Ronan
1499         clear s SL str Sum Sink rem* ans c C cm DM EX Exc* g i j vs BC BCK BCM
1500         save Results_StatsOrganComp
1501     else
1502         save([resultsPath 'Results_StatsOrganComp'],'Results_StatsOrganComp')
1503     end
1504 end
1505 
1506 function genesPerOrganFigure(data1, data2)
1507 %CREATEFIGURE(DATA1, DATA2)
1508 %  DATA1:  histogram data
1509 %  DATA2:  histogram data
1510 
1511 %  Auto-generated by MATLAB on 01-Nov-2017 13:29:33
1512 
1513 % Create figure
1514 figure1 = figure;
1515 
1516 % Create axes
1517 axes1 = axes('Parent',figure1,...
1518     'Position',[0.13 0.441422594142259 0.84158081705151 0.48357740585774]);
1519 hold(axes1,'on');
1520 
1521 % Create histogram
1522 histogram(data1,'DisplayName','Male','Parent',axes1,'BinMethod','auto');
1523 
1524 % Create histogram
1525 histogram(data2,'DisplayName','Female','Parent',axes1,'BinMethod','auto');
1526 
1527 % Create xlabel
1528 xlabel('Number of organs','FontWeight','bold');
1529 
1530 % Create ylabel
1531 ylabel('Number of genes','FontWeight','bold');
1532 
1533 % Uncomment the following line to preserve the X-limits of the axes
1534 % xlim(axes1,[-1 31]);
1535 % Uncomment the following line to preserve the Y-limits of the axes
1536 % ylim(axes1,[0 320]);
1537 box(axes1,'on');
1538 % Set the remaining axes properties
1539 set(axes1,'FontSize',16,'YGrid','on');
1540 % Create legend
1541 legend1 = legend(axes1,'show');
1542 set(legend1,...
1543     'Position',[0.199638917453492 0.822951332005227 0.0776315789473684 0.0538881309686221]);
1544 
1545 title('Genes per organ')
1546 
1547
```

---

Generated on Thu 14-May-2020 13:05:49 by **m2html** © 2005
