# Supplementary material for: Personalized whole‐body models integrate metabolism, physiology, and the gut microbiome
Source: Mol Syst Biol. 2020 May 28;16(5):e8982. doi: 10.15252/msb.20198982 (PMC7285886; doi:10.15252/msb.20198982)
Supplement: Supplementary file 22 — Dataset EV1 [file MSB-16-e8982-s022.zip › PSCM_toolbox/PSCM_toolbox_doc/src/determineFluxValuesOnBoundary.html]

Description of determineFluxValuesOnBoundary


# determineFluxValuesOnBoundary

## PURPOSE

**This function determines the number of reactions in the flux distributions that are on**

## SYNOPSIS

**function [OfConstraint, OfAll] = determineFluxValuesOnBoundary(model, solution)**

## DESCRIPTION

```
 This function determines the number of reactions in the flux distributions that are on
 the boundaries

 [OfConstraint, OfAll] = determineFluxValuesOnBoundary(model, solution)

 INPUT
 model         Model structure
 solution      Solution structure
 
 OUTPUT 
 OfConstraint  Fraction of flux values that are on the lower and upper bounds
               of all constrained reactions (assuming a minimum infinity
               of -1,000,000 and a  maximum infinity of 1,000,000
 OfAll         Fraction of flux values that are on the lower and upper bounds
               of all reactions in the model
 
 Ines Thiele, December 2018
```

## CROSS-REFERENCE INFORMATION

This function calls:


This function is called by:

## SOURCE CODE

```
0001 function [OfConstraint, OfAll] = determineFluxValuesOnBoundary(model, solution)
0002 % This function determines the number of reactions in the flux distributions that are on
0003 % the boundaries
0004 %
0005 % [OfConstraint, OfAll] = determineFluxValuesOnBoundary(model, solution)
0006 %
0007 % INPUT
0008 % model         Model structure
0009 % solution      Solution structure
0010 %
0011 % OUTPUT
0012 % OfConstraint  Fraction of flux values that are on the lower and upper bounds
0013 %               of all constrained reactions (assuming a minimum infinity
0014 %               of -1,000,000 and a  maximum infinity of 1,000,000
0015 % OfAll         Fraction of flux values that are on the lower and upper bounds
0016 %               of all reactions in the model
0017 %
0018 % Ines Thiele, December 2018
0019 %
0020 %
0021 %
0022 minInf = -1000000;
0023 maxInf = 1000000;
0024 % find reactions that have flux values on upper bound
0025 I = find(abs(solution.full)>1e-6);
0026 % find all non-zero and non-inf bounds
0027 J = find(abs(model.ub)~=0);
0028 Ji = find(abs(model.ub)<=abs(minInf));
0029 IJ = intersect(I,J);
0030 IJ = intersect(IJ,Ji);
0031 Usedub = model.ub(IJ);
0032 UsedF = solution.full(IJ);
0033 UsedR = model.rxns(IJ);
0034 OnUB= length(find(abs(Usedub-UsedF)<1e-5));
0035 
0036 % find reactions that have flux values on lower bound
0037 I = find(abs(solution.full)>1e-6);
0038 % find all non-zero and non-inf bounds
0039 J = find(abs(model.lb)~=0);
0040 Ji = find(abs(model.lb)<=abs(maxInf));
0041 IJ = intersect(I,J);
0042 IJ = intersect(IJ,Ji);
0043 Usedlb = model.lb(IJ);
0044 UsedF = solution.full(IJ);
0045 UsedR = model.rxns(IJ);
0046 OnLB= length(find(abs(Usedlb-UsedF)<1e-5));
0047 
0048 % constraint reactions
0049 minConstraints = length(intersect(find(model.lb>minInf),find(model.lb)));
0050 maxConstraints =length(intersect(find(model.ub<maxInf),find(model.ub)));
0051 PercentageConstraintRxns_model = (minConstraints + maxConstraints)*100/length(model.ub);
0052 %percentage of all reactions
0053 OfAll = (OnLB+OnUB)/length(model.rxns);
0054 OfConstraint = (OnLB+OnUB)/(minConstraints + maxConstraints);
```

---

Generated on Thu 14-May-2020 13:05:49 by **m2html** © 2005
