# Supplementary material for: Personalized whole‐body models integrate metabolism, physiology, and the gut microbiome
Source: Mol Syst Biol. 2020 May 28;16(5):e8982. doi: 10.15252/msb.20198982 (PMC7285886; doi:10.15252/msb.20198982)
Supplement: Supplementary file 22 — Dataset EV1 [file MSB-16-e8982-s022.zip › PSCM_toolbox/ext/m2html/doc/index.html]

Matlab Index


# Matlab Index

## Matlab Directories

- m2html
- m2html/@template
- m2html/@template/private
- m2html/private

## Matlab Files found in these Directories

|  |  |  |  |
| --- | --- | --- | --- |
| Contents | finish | mfileparse | set |
| char | get | mwizard | splitcode |
| display | loadtpl | mwizard2 | strtok |
| doxyread | m2html | openfile | subst |
| doxysearch | mdot | parse | template |
| doxywrite | mexexts | searchindex |  |

---

Generated on Tue 29-Oct-2019 21:04:53 by **m2html** © 2005
