# Supplementary material for: Personalized whole‐body models integrate metabolism, physiology, and the gut microbiome
Source: Mol Syst Biol. 2020 May 28;16(5):e8982. doi: 10.15252/msb.20198982 (PMC7285886; doi:10.15252/msb.20198982)
Supplement: Supplementary file 22 — Dataset EV1 [file MSB-16-e8982-s022.zip › PSCM_toolbox/PSCM_toolbox_doc/src/io/addReactionsHH.html]

Description of addReactionsHH


# addReactionsHH

## PURPOSE

**This function add reaction(s) to the whole-body metabolic model,**

## SYNOPSIS

**function [model] = addReactionsHH(model, rxnAbbrs,rxnNames, reactions, gprs, subSystems,couplingFactor)**

## DESCRIPTION

```
 This function add reaction(s) to the whole-body metabolic model,
 including the required coupling constraint.
 This function is based on model = addReaction(model,'newRxn1','A -> B + 2 C')

 [model] = addReactionsHH(model, rxnAbbrs,rxnNames, reactions, gprs, subSystems,couplingFactor)

 INPUT
 model             Model structure
 rxnAbbrs          List of reaction abbreviation(s) to be added
 rxnNames          List of reaction names
 reactions         List of reaction formula {'A -> B + 2 C'}
 gprs              List of grRules
 subSystems        List of subSystems
 couplingFactor    Coupling factor to be added, default 20000

 OUTPUT
 model             Updated model structure

 Ines Thiele 2018
```

## CROSS-REFERENCE INFORMATION

This function calls:


This function is called by:

## SOURCE CODE

```
0001 function [model] = addReactionsHH(model, rxnAbbrs,rxnNames, reactions, gprs, subSystems,couplingFactor)
0002 % This function add reaction(s) to the whole-body metabolic model,
0003 % including the required coupling constraint.
0004 % This function is based on model = addReaction(model,'newRxn1','A -> B + 2 C')
0005 %
0006 % [model] = addReactionsHH(model, rxnAbbrs,rxnNames, reactions, gprs, subSystems,couplingFactor)
0007 %
0008 % INPUT
0009 % model             Model structure
0010 % rxnAbbrs          List of reaction abbreviation(s) to be added
0011 % rxnNames          List of reaction names
0012 % reactions         List of reaction formula {'A -> B + 2 C'}
0013 % gprs              List of grRules
0014 % subSystems        List of subSystems
0015 % couplingFactor    Coupling factor to be added, default 20000
0016 %
0017 % OUTPUT
0018 % model             Updated model structure
0019 %
0020 % Ines Thiele 2018
0021 
0022 if ~exists('couplingFactor','var')
0023     couplingFactor = 20000;
0024 end
0025 
0026 for i = 1 : length(rxnAbbrs)
0027     
0028     % check that reaction does not exist yet in model
0029     if isempty(strmatch(rxnAbbrs(i),model.rxns,'exact'))
0030         % add reaction
0031         model = addReaction(model,rxnAbbrs{i},reactions{i});
0032         A = strmatch(rxnAbbrs(i),model.rxns,'exact');
0033         model.subSystems(A) = subSystems(i);
0034         model.grRules(A) = gprs(i);
0035         model.rxnNames(A) = rxnNames(i);
0036         token = strtok(rxnAbbrs{i},'_');
0037         % find organ biomass
0038         if strcmp(token,'sIEC')
0039             rxnC = strmatch('sIEC_biomass_reactionIEC01b',model.rxns);
0040         else
0041             rxnC = strmatch(strcat(token,'_biomass_maintenance'),model.rxns,'exact');
0042             if isempty(rxnC)
0043                 rxnC = strmatch(strcat(token,'_biomass_maintenance_noTrTr'),model.rxns);
0044             end
0045         end
0046         model.A = model.S;
0047         % if reaction does not start with Excretion or EX or Diet - add
0048         % coupling constraint
0049       
0050         if isempty(strmatch('EX_',rxnAbbrs(i))) && isempty(strmatch('Excretion_',rxnAbbrs(i))) && isempty(strmatch('Diet_',rxnAbbrs(i))) ...
0051                 && isempty(strmatch('LI_EX_',rxnAbbrs(i))) &&  isempty(strmatch('SI_EX_',rxnAbbrs(i))) ...
0052                 &&  isempty(strmatch('GI_EX_',rxnAbbrs(i))) && isempty(strmatch('BBB_',rxnAbbrs(i)))
0053             [model]=coupleRxnList2Rxn(model,rxnAbbrs(i),...
0054                 model.rxns(rxnC),couplingFactor,0.00);
0055         end
0056         model.S=model.A;
0057     else
0058         warning('Reaction with the same name already exists in the model');
0059     end
0060     
0061 end
```

---

Generated on Thu 14-May-2020 13:05:49 by **m2html** © 2005
