# Supplementary material for: Personalized whole‐body models integrate metabolism, physiology, and the gut microbiome
Source: Mol Syst Biol. 2020 May 28;16(5):e8982. doi: 10.15252/msb.20198982 (PMC7285886; doi:10.15252/msb.20198982)
Supplement: Supplementary file 22 — Dataset EV1 [file MSB-16-e8982-s022.zip › PSCM_toolbox/PSCM_toolbox_doc/src/io/loadPSCMfile.html]

Description of loadPSCMfile


# loadPSCMfile

## PURPOSE

**Loads a mat file into the workspace, given a nickname or a full filename**

## SYNOPSIS

**function variable = loadPSCMfile(fileName)**

## DESCRIPTION

```
 Loads a mat file into the workspace, given a nickname or a full filename
 Assumes that fileName points to a mat file with a single variable,
 otherwise the set of variables in the mat file are returned as a
 structure, where each field is a variable.

 INPUT: 
 fileName:     nickname of .mat file to load
               or 
               full name of .mat file to load
 
 OUTPUT:
 variable:     matlab variable returned
```

## CROSS-REFERENCE INFORMATION

This function calls:


This function is called by:

- perform\_sensi\_BMR\_all This script repeats the simulation described in Thiele et al.,
- annotateHH annotate Harvey and Harvetta
- runIEM\_HH This script predicts known biomarker metabolites in

## SOURCE CODE

```
0001 function variable = loadPSCMfile(fileName)
0002 % Loads a mat file into the workspace, given a nickname or a full filename
0003 % Assumes that fileName points to a mat file with a single variable,
0004 % otherwise the set of variables in the mat file are returned as a
0005 % structure, where each field is a variable.
0006 %
0007 % INPUT:
0008 % fileName:     nickname of .mat file to load
0009 %               or
0010 %               full name of .mat file to load
0011 %
0012 % OUTPUT:
0013 % variable:     matlab variable returned
0014 
0015 global useSolveCobraLPCPLEX
0016 useSolveCobraLPCPLEX
0017 
0018 useReadCbModel = 0;
0019 switch fileName
0020     case 'Harvey'
0021         if useSolveCobraLPCPLEX
0022             %COBRA v2 format
0023             load Harvey_1_01c
0024             
0025             male.subSystems(strmatch('Transport, endoplasmic reticular',male.subSystems,'exact'))={'Transport, endoplasmic reticulum'};
0026             male.subSystems(strmatch('Arginine and Proline Metabolism',male.subSystems,'exact'))={'Arginine and proline Metabolism'};
0027             male.subSystems(strmatch(' ',male.subSystems,'exact'))={'Miscellaneous'};
0028             
0029             if 1
0030                 %convert to v3 format except for coupling constraints
0031                 male   = convertOldStyleModel(male,0,0);
0032             end
0033         else
0034             if useReadCbModel
0035                 male = readCbModel('Harvey_1_03c', 'fileType','Matlab', 'modelName', 'male');
0036             else
0037                 %COBRA v3 format
0038                 %load Harvey_1_02c
0039                 load Harvey_1_03c
0040             end
0041         end
0042         if isfield(male,'gender')
0043             male.sex = male.gender;
0044             male = rmfield(male,'gender');
0045         else
0046             male.sex = 'male';
0047         end
0048         if isfield(male,'rxnGeneMat')
0049             male = rmfield(male,'rxnGeneMat');
0050         end
0051         variable = male;
0052     case 'Harvetta'
0053         if useSolveCobraLPCPLEX
0054             %COBRA v2 format
0055             load Harvetta_1_01c
0056             
0057             female.subSystems(strmatch('Transport, endoplasmic reticular',female.subSystems,'exact'))={'Transport, endoplasmic reticulum'};
0058             female.subSystems(strmatch('Arginine and Proline Metabolism',female.subSystems,'exact'))={'Arginine and proline Metabolism'};
0059             female.subSystems(strmatch(' ',female.subSystems,'exact'))={'Miscellaneous'};
0060                         
0061             if 1
0062                 %convert to v3 format except for coupling constraints
0063                 female = convertOldStyleModel(female,0,0);
0064             end
0065         else
0066             if useReadCbModel
0067                 female = readCbModel('Harvetta_1_03c', 'fileType','Matlab', 'modelName', 'male');
0068             else
0069                 %COBRA v3 format
0070                 %load Harvetta_1_02c
0071                 load Harvetta_1_03c
0072             end
0073         end
0074         if isfield(female,'gender')
0075             female.sex = female.gender;
0076             female = rmfield(female,'gender');
0077         else
0078             female.sex = 'female';
0079         end
0080         if isfield(female,'rxnGeneMat')
0081             female = rmfield(female,'rxnGeneMat');
0082         end
0083         variable = female;
0084     case 'Recon3D'
0085         load Recon3D_Harvey_Used_in_Script_120502
0086         variable = modelConsistent;
0087     otherwise
0088         load(fileName)
0089         s = whos;
0090         %attempt to return the file
0091         variable = s.name;
0092 end
0093 
0094
```

---

Generated on Thu 14-May-2020 13:05:49 by **m2html** © 2005
