# Supplementary material for: Personalized whole‐body models integrate metabolism, physiology, and the gut microbiome
Source: Mol Syst Biol. 2020 May 28;16(5):e8982. doi: 10.15252/msb.20198982 (PMC7285886; doi:10.15252/msb.20198982)
Supplement: Supplementary file 22 — Dataset EV1 [file MSB-16-e8982-s022.zip › PSCM_toolbox/PSCM_toolbox_doc/src/io/OrganLists.html]

Description of OrganLists


# OrganLists

## PURPOSE

**This file contains lists of ograns as they are used in the whole-body**

## SYNOPSIS

**This is a script file.**

## DESCRIPTION

```
 This file contains lists of ograns as they are used in the whole-body
 metabolic model.
 Please note that the desired sex has to be specified ('male' or 'female')

 Ines Thiele 2016 - 2017
```

## CROSS-REFERENCE INFORMATION

This function calls:


This function is called by:

- getOrgansFromHarvey This function cuts the organs from the whole-body metabolic model. Note that the different
- organEssentiality This function computes the organ essentiality in a whole-body model by
- physiologicalConstraintsHMDBbased This function applies constraints to the whole-body metabolic model
- physiologicalConstraintsHMDBbased\_old apply constraints to Harvey

## SOURCE CODE

```
0001 % This file contains lists of ograns as they are used in the whole-body
0002 % metabolic model.
0003 % Please note that the desired sex has to be specified ('male' or 'female')
0004 %
0005 % Ines Thiele 2016 - 2017
0006 
0007 OrgansListShort={ 'Heart'; 'Muscle'; 'Lung';'Skin'; 'Stomach'; 'sIEC'; 'Colon';...
0008     'Urinarybladder'; 'Retina'; 'Scord';'Brain';'Adipocytes'; ...
0009     'Liver';'Gall'; 'Kidney';'Pancreas'; 'Spleen';'Agland';'Thyroidgland';'Pthyroidgland';  ...
0010     'Ovary';'Uterus';...
0011     'Breast';'Cervix';...
0012     'Testis';'Prostate';...
0013     'Bcells';'CD4Tcells'; ...
0014     'Nkcells';'Monocyte';'Platelet'; 'RBC';%'Salvgland';
0015     %'Esophagus';'CD8Tcells';'Rectum';
0016     };
0017 if strcmp(sex,'female')
0018     OrgansListExt={'Heart'; 'Muscle'; 'Lung';'Skin'; 'Stomach'; 'sIEC'; 'Colon';...
0019         'Urinarybladder'; 'Retina'; 'Scord';'Brain';'Adipocytes'; ...
0020         'Liver';'Gall'; 'Kidney';'Pancreas'; 'Spleen';'Agland';'Thyroidgland';'Pthyroidgland';  ...
0021         'Ovary';'Uterus';...
0022         'Breast';'Cervix';...
0023         'Bcells';'CD4Tcells'; ...
0024         'Nkcells';'Monocyte';'Platelet'; 'RBC';...% 'Salvgland';
0025         'BBB';'Diet';'SI';'GI';'LI';'BileDuct';'Excretion'
0026         % 'Rectum';'CD8Tcells';'Esophagus';
0027         };
0028     OrgansList={'Heart'; 'Muscle'; 'Lung';'Skin'; 'Stomach'; 'sIEC'; 'Colon';...
0029         'Urinarybladder'; 'Retina'; 'Scord';'Brain';'Adipocytes'; ...
0030         'Liver';'Gall'; 'Kidney';'Pancreas'; 'Spleen';'Agland';'Thyroidgland';'Pthyroidgland';  ...
0031         'Ovary';'Uterus';...
0032         'Breast';'Cervix';...
0033         'Bcells';'CD4Tcells';...
0034         'Nkcells';'Monocyte';'Platelet'; 'RBC';...% 'Salvgland';
0035         %'Esophagus'; 'CD8Tcells';'Rectum';
0036         };% without 'BBB';'Diet';'SI';'GI';'LI';'BileDuct';'Excretion'
0037 elseif strcmp(sex,'male')
0038     OrgansListExt={'Heart'; 'Muscle'; 'Lung';'Skin'; 'Stomach'; 'sIEC'; 'Colon';...
0039         'Urinarybladder'; 'Retina'; 'Scord';'Brain';'Adipocytes'; ...
0040         'Liver';'Gall'; 'Kidney';'Pancreas'; 'Spleen';'Agland';'Thyroidgland';'Pthyroidgland';  ...
0041         'Testis';'Prostate';...
0042         'Bcells';'CD4Tcells'; ...
0043         'Nkcells';'Monocyte';'Platelet'; 'RBC';...% 'Salvgland';
0044         'BBB';'Diet';'SI';'GI';'LI';'BileDuct';'Excretion'
0045         %'Esophagus';'Rectum';'CD8Tcells';
0046         };
0047     OrgansList={'Heart'; 'Muscle'; 'Lung';'Skin'; 'Stomach'; 'sIEC'; 'Colon';...
0048         'Urinarybladder'; 'Retina'; 'Scord';'Brain';'Adipocytes'; ...
0049         'Liver';'Gall'; 'Kidney';'Pancreas'; 'Spleen';'Agland';'Thyroidgland';'Pthyroidgland';  ...
0050         'Testis';'Prostate';...
0051         'Bcells';'CD4Tcells'; ...
0052         'Nkcells';'Monocyte';'Platelet'; 'RBC';...% 'Salvgland';
0053         %'Esophagus';'Rectum'; 'CD8Tcells';
0054         };
0055 end
```

---

Generated on Thu 14-May-2020 13:05:49 by **m2html** © 2005
