# Supplementary material for: Personalized whole‐body models integrate metabolism, physiology, and the gut microbiome
Source: Mol Syst Biol. 2020 May 28;16(5):e8982. doi: 10.15252/msb.20198982 (PMC7285886; doi:10.15252/msb.20198982)
Supplement: Supplementary file 22 — Dataset EV1 [file MSB-16-e8982-s022.zip › PSCM_toolbox/PSCM_toolbox_doc/src/hostMicrobeInteraction/analyzeHMmodel.html]

Description of analyzeHMmodel


# analyzeHMmodel

## PURPOSE

**This function performs host-microbiome optimization for a set of defined**

## SYNOPSIS

**function [Results,ResultsSol,ResultsStats] = analyzeHMmodel(modelHM,Diet, Results,ResultsSol,setStandard,RxnMin,RxnMax,LPSolver)**

## DESCRIPTION

```
 This function performs host-microbiome optimization for a set of defined
 model reactions. Please note that the fecal secretion rate for the
 microbiome community biomass reaction is constrained to lb=0.4 and ub=1.

 [Results,ResultsSol,ResultsStats] = analyzeHMmodel(modelHM,Diet, Results,ResultsSol,setStandard,RxnMin,RxnMax)

 INPUT
 modelHM       model structure containing the host-microbiome model
 Diet          Diet option: 'EUAverageDiet' (default)
 Results       List of result names of FBA simulations using modelHM. The function provides the option to append the new results
               to a previous list of results. If not provided, a new list of results
               will be returned.
 ResultsSol    Array of FBA solution vectors corresponding to the optimization problems in Results. The function provides the option to append the new results
               to a previous list of results. If not provided, a new list of results
               will be returned.
 setStandard   default: 1
 RxnMin        Reaction(s) in modelHM to be minimized
 RxnMax        Reaction(s) in modelHM to be maximized
 LPSolver      Define LP solver to be used ('tomlab_cplex' or
               'ILOGcomplex' (default))

 OUTPUT
 Results       List of result names of FBA simulations using modelHM. 
 ResultsSol    Array of FBA solution vectors corresponding to the optimization problems in Results.
 ResultsStats  List of solver status for each FBA solution


 Ines Thiele 2016-2019


 define solver
```

## CROSS-REFERENCE INFORMATION

This function calls:

- EUAverageDietNew Average European Diet defintion. For details, please see https://www.vmh.life/#nutrition
- HighFiberDiet High fiber diet defintion. For details, please see https://www.vmh.life/#nutrition
- HighProteinDiet High protein diet defintion. For details, please see https://www.vmh.life/#nutrition
- UnhealthyDiet Unhealthy diet defintion. For details, please see https://www.vmh.life/#nutrition
- VegetarianDiet Vegetarian diet defintion. For details, please see https://www.vmh.life/#nutrition
- physiologicalConstraintsHMDBbased This function applies constraints to the whole-body metabolic model
- setDietConstraints This function sets diet constraints onto the bounds of the diet uptale
- setFeedingFastingConstraints This function sets constraints corresponding either to feeding (i.e.,
- standardPhysiolDefaultParameters This script creates the IndividualParameters structure which contains

This function is called by:

## SOURCE CODE

```
0001 function [Results,ResultsSol,ResultsStats] = analyzeHMmodel(modelHM,Diet, Results,ResultsSol,setStandard,RxnMin,RxnMax,LPSolver)
0002 % This function performs host-microbiome optimization for a set of defined
0003 % model reactions. Please note that the fecal secretion rate for the
0004 % microbiome community biomass reaction is constrained to lb=0.4 and ub=1.
0005 %
0006 % [Results,ResultsSol,ResultsStats] = analyzeHMmodel(modelHM,Diet, Results,ResultsSol,setStandard,RxnMin,RxnMax)
0007 %
0008 % INPUT
0009 % modelHM       model structure containing the host-microbiome model
0010 % Diet          Diet option: 'EUAverageDiet' (default)
0011 % Results       List of result names of FBA simulations using modelHM. The function provides the option to append the new results
0012 %               to a previous list of results. If not provided, a new list of results
0013 %               will be returned.
0014 % ResultsSol    Array of FBA solution vectors corresponding to the optimization problems in Results. The function provides the option to append the new results
0015 %               to a previous list of results. If not provided, a new list of results
0016 %               will be returned.
0017 % setStandard   default: 1
0018 % RxnMin        Reaction(s) in modelHM to be minimized
0019 % RxnMax        Reaction(s) in modelHM to be maximized
0020 % LPSolver      Define LP solver to be used ('tomlab_cplex' or
0021 %               'ILOGcomplex' (default))
0022 %
0023 % OUTPUT
0024 % Results       List of result names of FBA simulations using modelHM.
0025 % ResultsSol    Array of FBA solution vectors corresponding to the optimization problems in Results.
0026 % ResultsStats  List of solver status for each FBA solution
0027 %
0028 %
0029 % Ines Thiele 2016-2019
0030 %
0031 %
0032 % define solver
0033 if  ~exist('LPSolver','var')
0034     LPSolver = 'ILOGcomplex';
0035 end
0036 
0037 % define diet
0038 if ~exist('Diet','var')
0039     EUAverageDietNew;
0040 elseif strcmp(Diet,'EUAverageDiet')
0041     EUAverageDietNew;
0042 elseif strcmp(Diet,'HighFiberDiet')
0043     HighFiberDiet;
0044 elseif strcmp(Diet,'HighProteinDiet')
0045     HighProteinDiet;
0046 elseif strcmp(Diet,'UnhealthyDiet')
0047     UnhealthyDiet;
0048 elseif strcmp(Diet,'VegetarianDiet')
0049     VegetarianDiet;
0050 end
0051 if ~exist('setStandard','var')
0052     setStandard=1;
0053 end
0054 
0055 if ~exist('RxnMin','var')
0056     RxnMin ={'Kidney_EX_chsterol(e)_[bc]'
0057         % 'Kidney_EX_vldl_hs(e)_[bc]'
0058         % 'Kidney_EX_hdl_hs(e)_[bc]'
0059         'Kidney_EX_glc(e)_[bc]'
0060         'Kidney_EX_leuktrE4(e)_[bc]'
0061         'Kidney_EX_leuktrA4(e)_[bc]'
0062         'Brain_EX_dopa(e)_[csf]'
0063         'Brain_EX_srtn(e)_[csf]'
0064         'Brain_EX_bhb(e)_[csf]'
0065         'Brain_EX_acac(e)_[csf]'
0066         'Kidney_EX_etoh(e)_[bc]'
0067         'Kidney_EX_dopa(e)_[bc]'
0068         'Kidney_EX_fol(e)_[bc]'
0069         'Kidney_EX_prostgd2(e)_[bc]'
0070         'Kidney_EX_prostge2(e)_[bc]'
0071         'Kidney_EX_prostgf2(e)_[bc]'
0072         'Kidney_EX_prostgh2(e)_[bc]'
0073         'Kidney_EX_tststerone(e)_[bc]'
0074         'Kidney_EX_3ddcrn(e)_[bc]'
0075         'Kidney_EX_3deccrn(e)_[bc]'
0076         'Kidney_EX_3hdececrn(e)_[bc]'
0077         'Kidney_EX_3hexdcrn(e)_[bc]'
0078         'Kidney_EX_3octdec2crn(e)_[bc]'
0079         'Kidney_EX_3octdeccrn(e)_[bc]'
0080         'Kidney_EX_3tetd7ecoacrn(e)_[bc]'
0081         'Kidney_EX_3thexddcoacrn(e)_[bc]'
0082         'Kidney_EX_3ttetddcoacrn(e)_[bc]'
0083         'Kidney_EX_c10crn(e)_[bc]'
0084         'Kidney_EX_c12dc(e)_[bc]'
0085         'Kidney_EX_c16dc(e)_[bc]'
0086         'Kidney_EX_c3dc(e)_[bc]'
0087         'Kidney_EX_c51crn(e)_[bc]'
0088         'Kidney_EX_c5dc(e)_[bc]'
0089         'Kidney_EX_c6crn(e)_[bc]'
0090         'Kidney_EX_c8crn(e)_[bc]'
0091         'Kidney_EX_ddecrn(e)_[bc]'
0092         'Kidney_EX_ivcrn(e)_[bc]'
0093         'Kidney_EX_lac_L(e)_[bc]'
0094         'Kidney_EX_taur(e)_[bc]'
0095         'Kidney_EX_HC02191(e)_[bc]'
0096         'Kidney_EX_HC02192(e)_[bc]'
0097         'Kidney_EX_HC02193(e)_[bc]'
0098         'Kidney_EX_HC02195(e)_[bc]'
0099         'Kidney_EX_HC02196(e)_[bc]'
0100         'Kidney_EX_HC02220(e)_[bc]'
0101         'Kidney_EX_HC02194(e)_[bc]'
0102         'Kidney_EX_HC02197(e)_[bc]'
0103         'Kidney_EX_HC02198(e)_[bc]'
0104         'Kidney_EX_HC02199(e)_[bc]'
0105         'Brain_EX_HC02193(e)_[csf]'
0106         'Brain_EX_HC02195(e)_[csf]'
0107         'Brain_EX_HC02196(e)_[csf]'
0108         'Brain_EX_HC02199(e)_[csf]'
0109         'Brain_EX_HC02191(e)_[csf]'
0110         'Brain_EX_HC02194(e)_[csf]'
0111         'Brain_EX_HC02197(e)_[csf]'
0112         'Brain_EX_HC02198(e)_[csf]'
0113         'Brain_EX_HC02192(e)_[csf]'
0114         'Brain_EX_gchola(e)_[csf]'
0115         'Brain_EX_tchola(e)_[csf]'
0116         'Kidney_EX_gchola(e)_[bc]'
0117         'Kidney_EX_tchola(e)_[bc]'
0118         'Kidney_EX_tdchola(e)_[bc]'
0119         'Brain_EX_tdchola(e)_[csf]'
0120         'EX_acnam[u]'
0121         'EX_pheacgln[u]'
0122         'EX_3hmp[u]'
0123         'EX_succ[u]'
0124         'EX_cit[u]'
0125         'EX_glc_D[u]'
0126         'EX_urea[u]'
0127         'EX_glcur[u]'
0128         'Brain_EX_srtn(e)_[csf]'
0129         'Brain_EX_o2(e)_[csf]'
0130         'Heart_EX_o2(e)_[bc]'
0131         };
0132     RxnMin = unique(RxnMin);
0133 end
0134 if ~exist('RxnMax','var')
0135     RxnMax = {
0136         'Adipocytes_TAG_HSad'
0137         'Adipocytes_sink_c226coa(c)'
0138         'Adipocytes_sink_doco13ecoa(c)'
0139         'Adipocytes_sink_hdca(c)'
0140         'Adipocytes_sink_lnlc(c)'
0141         'Adipocytes_sink_lnlccoa(c)'
0142         'Adipocytes_sink_lnlncacoa(c)'
0143         'Adipocytes_sink_lnlncgcoa(c)'
0144         'Adipocytes_sink_odecoa(c)'
0145         'Adipocytes_sink_pmtcoa(c)'
0146         'Adipocytes_sink_stcoa(c)'
0147         'Adipocytes_sink_tag_hs(c)'
0148         'Adipocytes_sink_tmndnc(c)'
0149         'Adipocytes_sink_tmndnccoa(c)'
0150         'Muscle_DM_PROTEIN'
0151         'Muscle_DM_atp_c_'
0152         'Brain_3HLYTCL' % dopamine synthesis
0153         'Brain_5HLTDL' %Serotonin synthesis
0154         'Brain_NORANMT' % adrenaline
0155         'Brain_GLUDC' % gaba production
0156         'Brain_DOPAc'
0157         'Kidney_KYNATESYN' % kynate synthesis
0158         'Kidney_QUILSYN' % Quinolinate Synthesis
0159         'Brain_CHAT' % acetylcholine synthesis
0160         'Liver_VLDL_HSSYN'
0161         'Liver_IDL_HSSYN'
0162         'Liver_LDL_HSSYN'
0163         'Liver_HDL_HSSYN'
0164         'Liver_LDH_D'
0165         % 'Liver_BAAT2x'
0166         'Liver_ACS'
0167         % 'Liver_ACS2'
0168         'Liver_ALCD2if'
0169         % 'Liver_BGLYFm'
0170         'Liver_DGAT'
0171         'Liver_DHCR72r'
0172         'Liver_DHCR243r' %% add in next simulation round
0173         'Liver_GTHS'
0174         'Liver_PHACCOAGLNAC'
0175         'Liver_r0629'
0176         'Liver_r0630'
0177         'Liver_RE2637C'
0178         % 'Liver_RE2637X'
0179         'Liver_BGLYFm'
0180         'Liver_EX_chsterols(e)_[bc]'
0181         'Liver_VALTA'
0182         'Liver_VALTAm'
0183         'Adipocytes_VALTA'
0184         'Adipocytes_VALTAm'
0185         'Brain_VALTA'
0186         'Brain_VALTAm'
0187         'Liver_LEUTA'
0188         'Liver_LEUTAm'
0189         'Adipocytes_LEUTA'
0190         'Adipocytes_LEUTAm'
0191         'Brain_LEUTA'
0192         'Brain_LEUTAm'
0193         'Liver_ILETAA'
0194         'Liver_ILETAm'
0195         'Adipocytes_ILETA'
0196         'Adipocytes_ILETAm'
0197         'Brain_ILETA'
0198         'Brain_ILETAm'
0199         'Liver_OIVD2m'
0200         'Adipocytes_OIVD2m'
0201         'Brain_OIVD2m'
0202         'Liver_PDHm'
0203         'Adipocytes_PDHm'
0204         'Brain_PDHm'
0205         'Heart_PDHm'
0206         'Muscle_PDHm'
0207         'Brain_PHETHPTOX2'
0208         'Brain_PGM'
0209         'Brain_HMR_7749'
0210         'Brain_HMR_7746'
0211         'Brain_HMR_7748'
0212         'Brain_HMR_7745'
0213         'Brain_PHEMEtm'
0214         'Brain_PGK'
0215         'Muscle_PGK'
0216         'Heart_PGK'
0217         'Liver_PGK'
0218         'Adipocytes_PGK'
0219         'Brain_CYOOm3'
0220         'Muscle_CYOOm3'
0221         'Heart_CYOOm3'
0222         'Liver_CYOOm3'
0223         'Adipocytes_CYOOm3'
0224         'Brain_SUCOASm'
0225         'Muscle_SUCOASm'
0226         'Heart_SUCOASm'
0227         'Liver_SUCOASm'
0228         'Adipocytes_SUCOASm'
0229         'EX_acnam[u]'
0230         'EX_pheacgln[u]'
0231         'EX_3hmp[u]'
0232         'EX_succ[u]'
0233         'EX_cit[u]'
0234         'EX_glc_D[u]'
0235         'EX_urea[u]'
0236         'EX_glcur[u]'
0237         'CD4Tcells_EX_leuktrA4(e)_[bc]'
0238         'CD4Tcells_EX_leuktrD4(e)_[bc]'
0239         'Nkcells_EX_lac_L(e)_[bc]'
0240         'Nkcells_EX_leuktrA4(e)_[bc]'
0241         'Nkcells_EX_leuktrB4(e)_[bc]'
0242         'Nkcells_EX_leuktrF4(e)_[bc]'
0243         'Monocyte_EX_leuktrA4(e)_[bc]'
0244         'Monocyte_EX_leuktrB4(e)_[bc]'
0245         'Monocyte_EX_leuktrD4(e)_[bc]'
0246         'Monocyte_EX_leuktrE4(e)_[bc]'
0247         'Brain_G6PPer' %gluconeogenisis
0248         'Liver_G6PPer' %gluconeogenisis
0249         'Kidney_G6PPer' %gluconeogenisis
0250         'Brain_EX_o2(e)_[csf]'
0251         'Heart_EX_o2(e)_[bc]'
0252         'RBC_MTHFR3'
0253         'Kidney_MTHFR3'
0254         'Brain_CK'
0255         'Brain_CKc'
0256         'Muscle_CK'
0257         'Muscle_CKc'
0258         'Heart_CK' % increase linked to heart attack
0259         'Heart_CKc'
0260         };
0261     RxnMax = unique(RxnMax);
0262 end
0263 if exist('Results','var')
0264     [cnt,b] =size(Results);
0265     cnt = cnt +1; % append to existing Results
0266 else
0267     cnt = 1;
0268 end
0269 
0270 % modelHM = modelOHM;
0271 % set fasting or feeding state
0272 modelHM = setFeedingFastingConstraints(modelHM, 'feeding');
0273 % set diet - either AvAm or Bal
0274 modelHM = setDietConstraints(modelHM, Diet);
0275 if setStandard ==1
0276     % set constraints based on HMDB
0277     sex=modelHM.sex;
0278     standardPhysiolDefaultParameters;
0279     modelHM = physiologicalConstraintsHMDBbased(modelHM,IndividualParameters);
0280     [modelHM] = setDefaultModelingConstraints(modelHM);
0281 end
0282 % This allows me the use the script also for the GF version
0283 if ~isempty(strmatch('Excretion_EX_microbiota_LI_biomass[fe]',modelHM.rxns,'exact'))
0284     %%fecal microbiota
0285     modelHM = changeObjective(modelHM, 'Excretion_EX_microbiota_LI_biomass[fe]');
0286     tic;[solutionHM,LPProblem]=solveCobraLPCPLEX(modelHM,1,0,0,[],0,'tomlab_cplex');toc
0287     Results{cnt,1}='Excretion_EX_microbiota_LI_biomass[fe]';
0288     if solutionHM.origStat ~= -1 % problem is feasible
0289         Results{cnt,2}=num2str(solutionHM.full(find(modelHM.c)));
0290         ResultsSol(:,cnt)=solutionHM.full;
0291         ResultsStats(:,cnt)=solutionHM.origStat;
0292     else
0293         Results{cnt,2}='NaN';
0294         ResultsSol(:,cnt)=[];
0295         ResultsStats(:,cnt)=solutionHM.origStat;
0296     end
0297     cnt = cnt +1;
0298     % set faecal secretion constraint
0299     modelHM.lb(find(ismember(modelHM.rxns,'Excretion_EX_microbiota_LI_biomass[fe]')))=0.4; %
0300     modelHM.ub(find(ismember(modelHM.rxns,'Excretion_EX_microbiota_LI_biomass[fe]')))=1; %
0301 else
0302     Results{cnt,1}='Excretion_EX_microbiota_LI_biomass[fe]';
0303     Results{cnt,2}=num2str(0);
0304     ResultsSol(:,cnt)=zeros(length(modelHM.rxns),1);
0305     ResultsStats(:,cnt)=1;
0306     cnt = cnt +1;
0307 end
0308 %%Test flux through Whole_body_objective_rxn
0309 modelHM = changeObjective(modelHM, 'Whole_body_objective_rxn');
0310 tic;[solutionHM,LPProblem]=solveCobraLPCPLEX(modelHM,1,0,0,[],0,LPSolver);toc
0311 Results{cnt,1}='Whole_body_objective_rxn(max)';
0312 if solutionHM.origStat ~= -1 % problem is feasible
0313     Results{cnt,2}=num2str(solutionHM.full(find(modelHM.c)));
0314     ResultsSol(:,cnt)=solutionHM.full;
0315     ResultsStats(:,cnt)=solutionHM.origStat;
0316 else
0317     Results{cnt,2}='NaN';
0318     ResultsSol(:,cnt)=[];
0319     ResultsStats(:,cnt)=solutionHM.origStat;
0320 end
0321 cnt = cnt +1;
0322 LPProblem=modelHM;
0323 
0324 LPProblem.osense = 1; % minimization
0325 tic;[solutionHM,LPProblem]=solveCobraLPCPLEX(LPProblem,1,0,0,[],0,LPSolver);toc
0326 Results{cnt,1}='Whole_body_objective_rxn(min)';
0327 if solutionHM.origStat ~= -1 % problem is feasible
0328     Results{cnt,2}=num2str(solutionHM.full(find(LPProblem.c)));
0329     ResultsSol(:,cnt)=solutionHM.full;
0330     ResultsStats(:,cnt)=solutionHM.origStat;
0331 else
0332     Results{cnt,2}='NaN';
0333     ResultsSol(:,cnt)=[];
0334     ResultsStats(:,cnt)=solutionHM.origStat;
0335 end
0336 cnt = cnt +1;
0337 
0338 % set lb of RMR to 1U
0339 %modelHM.lb(find(ismember(modelHM.rxns,'Whole_body_objective_rxn')))=1;
0340 %% Maximize for alternatives - reuse basis
0341 
0342 for i = 1 : length(RxnMax)
0343     RxnMax{i}
0344     % check that reaction is in model
0345     if ~isempty(strmatch(RxnMax{i},LPProblem.rxns,'exact'))
0346         LPProblemMin = changeObjective(LPProblem,RxnMax{i});
0347         LPProblemMin.osense = -1;
0348         tic;[solutionHM,LPProblemMin]=solveCobraLPCPLEX(LPProblemMin,1,1,0,[],0,LPSolver);toc
0349         Results{cnt,1}=RxnMax{i};
0350         if solutionHM.origStat ~= -1 % problem is feasible
0351             Results{cnt,2}=num2str(solutionHM.full(find(LPProblemMin.c)));
0352             ResultsSol(:,cnt)=solutionHM.full;
0353             ResultsStats(:,cnt)=solutionHM.origStat;
0354         else
0355             Results{cnt,2}='NaN';
0356             ResultsSol(:,cnt)=[];
0357             ResultsStats(:,cnt)=solutionHM.origStat;
0358         end
0359         cnt = cnt +1;
0360     end
0361 end
0362 
0363 for i = 1 : length(RxnMin)
0364     % check that reaction is in model
0365     if ~isempty(strmatch(RxnMin{i},LPProblem.rxns,'exact'))
0366         LPProblemMin = changeObjective(LPProblem,RxnMin{i});
0367         LPProblemMin.osense = 1;
0368         tic;[solutionHM,LPProblemMin]=solveCobraLPCPLEX(LPProblemMin,1,1,0,[],0,LPSolver);toc
0369         Results{cnt,1}=RxnMin{i};
0370         if solutionHM.origStat ~= -1 % problem is feasible
0371             Results{cnt,2}=num2str(solutionHM.full(find(LPProblemMin.c)));
0372             ResultsSol(:,cnt)=solutionHM.full;
0373             ResultsStats(:,cnt)=solutionHM.origStat;
0374         else
0375             Results{cnt,2}='NaN';
0376             ResultsSol(:,cnt)=[];
0377             ResultsStats(:,cnt)=solutionHM.origStat;
0378         end
0379         cnt = cnt +1;
0380     end
0381 end
```

---

Generated on Thu 14-May-2020 13:05:49 by **m2html** © 2005
