# Supplementary material for: Personalized whole‐body models integrate metabolism, physiology, and the gut microbiome
Source: Mol Syst Biol. 2020 May 28;16(5):e8982. doi: 10.15252/msb.20198982 (PMC7285886; doi:10.15252/msb.20198982)
Supplement: Supplementary file 22 — Dataset EV1 [file MSB-16-e8982-s022.zip › PSCM_toolbox/PSCM_toolbox_doc/src/hostMicrobeInteraction/adjust.html]

Description of adjust


# adjust

## PURPOSE

**These constrains are specific to the host-microbiome interaction modeling**

## SYNOPSIS

**This is a script file.**

## DESCRIPTION

```
 These constrains are specific to the host-microbiome interaction modeling
 performed for the publication.
```

## CROSS-REFERENCE INFORMATION

This function calls:


This function is called by:

## SOURCE CODE

```
0001 % These constrains are specific to the host-microbiome interaction modeling
0002 % performed for the publication.
0003 
0004 Rx={'EX_nh4[u]'
0005     };
0006 
0007 Bx=[58.06444427    905.4203729
0008     ];
0009 for i =1 : 1%length(Rx)
0010     modelHM.lb(strmatch( Rx{i},modelHM.rxns,'exact')) = Bx(i,1); % currently -400 rendering many of the models to be infeasible in germfree state
0011     modelHM.ub(strmatch( Rx{i},modelHM.rxns,'exact')) = Bx(i,2); % currently -400 rendering many of the models to be infeasible in germfree state
0012 end
0013 Rx2 ={'Colon_2HBt2[bpC]'
0014     'Colon_2HBt2[luC]'
0015     'Colon_2MBUTtr[bpC]'
0016     'Colon_2MBUTtr[luC]'
0017     'Colon_2OBUTt'
0018     'Colon_2OBUTt[bpC]'
0019     'Colon_3MOPte[bpC]'
0020     'Colon_4HBZte[bpC]'
0021     'Colon_4HBZte[luC]'
0022     'Colon_4MCATtr[bpC]'
0023     'Colon_4MCATtr[luC]'
0024     'Colon_5APTNtr[bpC]'
0025     'Colon_5APTNtr[luC]'
0026     'Colon_5MTHFt2[luC]'
0027     'Colon_7OCHOLATEtr[bpC]'
0028     'Colon_7OCHOLATEtr[luC]'
0029     'Colon_ACALDt[bpC]'
0030     'Colon_ACt2r[bpC]'
0031     'Colon_ADEt[bpC]'
0032     'Colon_ADNt[bpC]'
0033     'Colon_BTNt2[bpC]'
0034     'Colon_BTOHtr[bpC]'
0035     'Colon_BTOHtr[luC]'
0036     'Colon_C04483t1[bpC]'
0037     'Colon_CE4890te'
0038     'Colon_CE4890te[bpC]'
0039     'Colon_CHOLATEt3[bpC]'
0040     'Colon_CHOLtu[bpC]'
0041     'Colon_CITt4_2[bpC]'
0042     'Colon_CLFORtex[bpC]'
0043     'Colon_CYTDt[bpC]'
0044     'Colon_D_LACt2[bpC]'
0045     'Colon_DADNt4[bpC]'
0046     'Colon_DURIt[bpC]'
0047     'Colon_ETHAt[bpC]'
0048     'Colon_EX_2hb[bpC]_[bp]'
0049     'Colon_EX_2hb[luC]_[luLI]'
0050     'Colon_EX_2hyoxplac[luC]_[luLI]'
0051     'Colon_EX_2mbut[bpC]_[bp]'
0052     'Colon_EX_2mbut[luC]_[luLI]'
0053     'Colon_EX_2obut[bpC]_[bp]'
0054     'Colon_EX_2obut[luC]_[luLI]'
0055     'Colon_EX_3mop[bpC]_[bp]'
0056     'Colon_EX_4hbz[bpC]_[bp]'
0057     'Colon_EX_4hbz[luC]_[luLI]'
0058     'Colon_EX_4mcat[bpC]_[bp]'
0059     'Colon_EX_4mcat[luC]_[luLI]'
0060     'Colon_EX_5aptn[bpC]_[bp]'
0061     'Colon_EX_5aptn[luC]_[luLI]'
0062     'Colon_EX_5mthf[luC]_[luLI]'
0063     'Colon_EX_7ocholate[bpC]_[bp]'
0064     'Colon_EX_7ocholate[luC]_[luLI]'
0065     'Colon_EX_ac[bpC]_[bp]'
0066     'Colon_EX_acald[bpC]_[bp]'
0067     'Colon_EX_ade[bpC]_[bp]'
0068     'Colon_EX_adn[bpC]_[bp]'
0069     'Colon_EX_aprgstrn[bpC]_[bp]'
0070     'Colon_EX_aprgstrn[luC]_[luLI]'
0071     'Colon_EX_btn[bpC]_[bp]'
0072     'Colon_EX_btoh[bpC]_[bp]'
0073     'Colon_EX_btoh[luC]_[luLI]'
0074     'Colon_EX_C02528[bpC]_[bp]'
0075     'Colon_EX_CE4890[bpC]_[bp]'
0076     'Colon_EX_CE4890[luC]_[luLI]'
0077     'Colon_EX_chol[bpC]_[bp]'
0078     'Colon_EX_cholate[bpC]_[bp]'
0079     'Colon_EX_cholate[luC]_[luLI]'
0080     'Colon_EX_cit[bpC]_[bp]'
0081     'Colon_EX_dad_2[bpC]_[bp]'
0082     'Colon_EX_dchac[bpC]_[bp]'
0083     'Colon_EX_duri[bpC]_[bp]'
0084     'Colon_EX_etha[bpC]_[bp]'
0085     'Colon_EX_fald[bpC]_[bp]'
0086     'Colon_EX_fe3[bpC]_[bp]'
0087     'Colon_EX_for[bpC]_[bp]'
0088     'Colon_EX_fru[bpC]_[bp]'
0089     'Colon_EX_fum[bpC]_[bp]'
0090     'Colon_EX_gam[bpC]_[bp]'
0091     'Colon_EX_gcald[bpC]_[bp]'
0092     'Colon_EX_gcald[luC]_[luLI]'
0093     'Colon_EX_glcn[bpC]_[bp]'
0094     'Colon_EX_glcn[luC]_[luLI]'
0095     'Colon_EX_glutar[bpC]_[bp]'
0096     'Colon_EX_glutar[luC]_[luLI]'
0097     'Colon_EX_glyc3p[bpC]_[bp]'
0098     'Colon_EX_gsn[bpC]_[bp]'
0099     'Colon_EX_gua[bpC]_[bp]'
0100     'Colon_EX_indole[bpC]_[bp]'
0101     'Colon_EX_indole[luC]_[luLI]'
0102     'Colon_EX_inost[bpC]_[bp]'
0103     'Colon_EX_ins[bpC]_[bp]'
0104     'Colon_EX_isobut[bpC]_[bp]'
0105     'Colon_EX_isobut[luC]_[luLI]'
0106     'Colon_EX_isocapr[bpC]_[bp]'
0107     'Colon_EX_isocapr[luC]_[luLI]'
0108     'Colon_EX_isoval[bpC]_[bp]'
0109     'Colon_EX_isoval[luC]_[luLI]'
0110     'Colon_EX_lac_D[bpC]_[bp]'
0111     'Colon_EX_mal_L[bpC]_[bp]'
0112     'Colon_EX_mlthf[bpC]_[bp]'
0113     'Colon_EX_mlthf[luC]_[luLI]'
0114     'Colon_EX_n8aspmd[bpC]_[bp]'
0115     'Colon_EX_n8aspmd[luC]_[luLI]'
0116     'Colon_EX_ncam[bpC]_[bp]'
0117     'Colon_EX_oxa[luC]_[luLI]'
0118     'Colon_EX_pheme[bpC]_[bp]'
0119     'Colon_EX_pheme[luC]_[luLI]'
0120     'Colon_EX_phpyr[bpC]_[bp]'
0121     'Colon_EX_phpyr[luC]_[luLI]'
0122     'Colon_EX_pnto_R[bpC]_[bp]'
0123     'Colon_EX_pydx[bpC]_[bp]'
0124     'Colon_EX_pydxn[bpC]_[bp]'
0125     'Colon_EX_pyr[bpC]_[bp]'
0126     'Colon_EX_so3[bpC]_[bp]'
0127     'Colon_EX_sprm[bpC]_[bp]'
0128     'Colon_EX_succ[bpC]_[bp]'
0129     'Colon_EX_thf[bpC]_[bp]'
0130     'Colon_EX_thmmp[bpC]_[bp]'
0131     'Colon_EX_thmmp[luC]_[luLI]'
0132     'Colon_EX_thmtp[bpC]_[bp]'
0133     'Colon_EX_thmtp[luC]_[luLI]'
0134     'Colon_EX_thym[bpC]_[bp]'
0135     'Colon_EX_tma[bpC]_[bp]'
0136     'Colon_EX_tma[luC]_[luLI]'
0137     'Colon_EX_ura[bpC]_[bp]'
0138     'Colon_EX_uri[bpC]_[bp]'
0139     'Colon_EX_xtsn[bpC]_[bp]'
0140     'Colon_2HYOXPLACtr[luC]'
0141     'Colon_CHOLATEte[bpC]'
0142     'Colon_CHOLATEte[luC]'
0143     'Colon_FE3MTP1[bpC]'
0144     'Colon_FOLt2[bpC]'
0145     'Colon_FRUt1r[bpC]'
0146     'Colon_FUMtr[bpC]'
0147     'Colon_GALt1r[bpC]'
0148     'Colon_GAMt1r[bpC]'
0149     'Colon_GCALDtr[bpC]'
0150     'Colon_GCALDtr[luC]'
0151     'Colon_GLCNte[luC]'
0152     'Colon_GLCNte[bpC]'
0153     'Colon_GLUTAROAT3t[bpC]'
0154     'Colon_GLUTAROAT3t[luC]'
0155     'Colon_GLUTARte[bpC]'
0156     'Colon_GLUTARte[luC]'
0157     'Colon_GLYC3tr[bpC]'
0158     'Colon_GSNt[bpC]'
0159     'Colon_GUAt[bpC]'
0160     'Colon_HMR_3951[bpC]'
0161     'Colon_HMR_6533[bpC]'
0162     'Colon_HMR_7977[bpC]'
0163     'Colon_HMR_7977[luC]'
0164     'Colon_INDOLEup[bpC]'
0165     'Colon_INDOLEup[luC]'
0166     'Colon_INSt[bpC]'
0167     'Colon_INSTt4[bpC]'
0168     'Colon_ISOBUTtr[bpC]'
0169     'Colon_ISOBUTtr[luC]'
0170     'Colon_ISOCAPRtr[bpC]'
0171     'Colon_ISOCAPRtr[luC]'
0172     'Colon_ISOVALtr[bpC]'
0173     'Colon_ISOVALtr[luC]'
0174     'Colon_MAL_Lte[bpC]'
0175     'Colon_MLTHFt[luC]'
0176     'Colon_MLTHFte2[bpC]'
0177     'Colon_N8ASPMDte[bpC]'
0178     'Colon_N8ASPMDte[luC]'
0179     'Colon_NCAMDe[bpC]'
0180     'Colon_OXAt[bpC]'
0181     'Colon_OXAt[luC]'
0182     'Colon_PHEMEt[bpC]'
0183     'Colon_PHEMEt[luC]'
0184     'Colon_PHPYRte[bpC]'
0185     'Colon_PHPYRte[luC]'
0186     'Colon_PNTOte[bpC]'
0187     'Colon_PYDXNtr[bpC]'
0188     'Colon_PYDXtr[bpC]'
0189     'Colon_PYRSMCT1[bpC]'
0190     'Colon_r0993[bpC]'
0191     'Colon_r1421[bpC]'
0192     'Colon_SO4OXAtex2[bpC]'
0193     'Colon_SPRMTDe[bpC]'
0194     'Colon_SUCCt4_2[bpC]'
0195     'Colon_THFt2[bpC]'
0196     'Colon_THMMPt4[bpC]'
0197     'Colon_THMMPt4[luC]'
0198     'Colon_THMTPt[bpC]'
0199     'Colon_THMTPt[luC]'
0200     'Colon_THYMDt1[bpC]'
0201     'Colon_THYMDtr2[bpC]'
0202     'Colon_THYMt[bpC]'
0203     'Colon_TMAtr[bpC]'
0204     'Colon_TMAtr[luC]'
0205     'Colon_URAt[bpC]'
0206     'Colon_URIt[bpC]'
0207     'Colon_XTSNtr[bpC]'
0208     'EX_2mbut[u]'
0209     'EX_4mcat[u]'
0210     'EX_5aptn[u]'
0211     'EX_7ocholate[u]'
0212     'EX_btoh[u]'
0213     'EX_gcald[u]'
0214     'EX_glutar[u]'
0215     'EX_indole[u]'
0216     'EX_isobut[u]'
0217     'EX_isocapr[u]'
0218     'EX_isoval[u]'
0219     'EX_ncam[u]'
0220     'EX_tma[u]'
0221     'EX_tmao[u]'
0222     'Kidney_EX_2mbut(e)_[bc]'
0223     'Kidney_EX_2mbut(e)_[bcK]'
0224     'Kidney_EX_2mbut(e)_[u]'
0225     'Kidney_EX_2mbut[bcK]_[bc]'
0226     'Kidney_EX_4mcat(e)_[bc]'
0227     'Kidney_EX_4mcat(e)_[bcK]'
0228     'Kidney_EX_4mcat(e)_[u]'
0229     'Kidney_EX_4mcat[bcK]_[bc]'
0230     'Kidney_EX_5aptn(e)_[bc]'
0231     'Kidney_EX_5aptn(e)_[bcK]'
0232     'Kidney_EX_5aptn(e)_[u]'
0233     'Kidney_EX_5aptn[bcK]_[bc]'
0234     'Kidney_EX_7ocholate(e)_[bc]'
0235     'Kidney_EX_7ocholate(e)_[bcK]'
0236     'Kidney_EX_7ocholate(e)_[u]'
0237     'Kidney_EX_7ocholate[bcK]_[bc]'
0238     'Kidney_EX_btoh(e)_[bc]'
0239     'Kidney_EX_btoh(e)_[bcK]'
0240     'Kidney_EX_btoh(e)_[u]'
0241     'Kidney_EX_btoh[bcK]_[bc]'
0242     'Kidney_EX_gcald(e)_[bc]'
0243     'Kidney_EX_gcald(e)_[bcK]'
0244     'Kidney_EX_gcald(e)_[u]'
0245     'Kidney_EX_gcald[bcK]_[bc]'
0246     'Kidney_EX_glutar(e)_[bcK]'
0247     'Kidney_EX_glutar(e)_[u]'
0248     'Kidney_EX_indole(e)_[bc]'
0249     'Kidney_EX_indole(e)_[bcK]'
0250     'Kidney_EX_indole(e)_[u]'
0251     'Kidney_EX_indole[bcK]_[bc]'
0252     'Kidney_EX_isobut(e)_[bc]'
0253     'Kidney_EX_isobut(e)_[bcK]'
0254     'Kidney_EX_isobut(e)_[u]'
0255     'Kidney_EX_isobut[bcK]_[bc]'
0256     'Kidney_EX_isocapr(e)_[bc]'
0257     'Kidney_EX_isocapr(e)_[bcK]'
0258     'Kidney_EX_isocapr(e)_[u]'
0259     'Kidney_EX_isocapr[bcK]_[bc]'
0260     'Kidney_EX_isoval(e)_[bc]'
0261     'Kidney_EX_isoval(e)_[bcK]'
0262     'Kidney_EX_isoval(e)_[u]'
0263     'Kidney_EX_isoval[bcK]_[bc]'
0264     'Kidney_EX_ncam(e)_[bc]'
0265     'Kidney_EX_ncam(e)_[bcK]'
0266     'Kidney_EX_ncam(e)_[u]'
0267     'Kidney_EX_ncam[bcK]_[bc]'
0268     'Kidney_EX_tma(e)_[bc]'
0269     'Kidney_EX_tma(e)_[bcK]'
0270     'Kidney_EX_tma(e)_[u]'
0271     'Kidney_EX_tma[bcK]_[bc]'
0272     'Kidney_EX_tmao(e)_[bc]'
0273     'Kidney_EX_tmao(e)_[bcK]'
0274     'Kidney_EX_tmao(e)_[u]'
0275     'Kidney_EX_tmao[bcK]_[bc]'
0276     'Liver_2HBt2'
0277     'Liver_2HBt2[bpL]'
0278     'Liver_2MBUTtr'
0279     'Liver_2MBUTtr[bpL]'
0280     'Liver_2OBUTt'
0281     'Liver_2OBUTt[bpL]'
0282     'Liver_3MOPte[bpL]'
0283     'Liver_4HBZte'
0284     'Liver_4HBZte[bpL]'
0285     'Liver_4MCATtr'
0286     'Liver_4MCATtr[bpL]'
0287     'Liver_5APTNtr'
0288     'Liver_5APTNtr[bpL]'
0289     'Liver_7OCHOLATEtr'
0290     'Liver_7OCHOLATEtr[bpL]'
0291     'Liver_ACALDt[bpL]'
0292     'Liver_ACt2r[bpL]'
0293     'Liver_ADEt[bpL]'
0294     'Liver_BTNt2[bpL]'
0295     'Liver_BTOHtr'
0296     'Liver_BTOHtr[bpL]'
0297     'Liver_C04483t1[bpL]'
0298     'Liver_C04483t2[bpL]'
0299     'Liver_CE4890te'
0300     'Liver_CE4890te[bpL]'
0301     'Liver_D_LACt2'
0302     'Liver_D_LACt2[bpL]'
0303     'Liver_ETHAt'
0304     'Liver_ETHAt[bpL]'
0305     'Liver_EX_2hb[bpL]_[bp]'
0306     'Liver_EX_2mbut(e)_[bc]'
0307     'Liver_EX_2mbut[bpL]_[bp]'
0308     'Liver_EX_2obut(e)_[bc]'
0309     'Liver_EX_2obut[bpL]_[bp]'
0310     'Liver_EX_3mop[bpL]_[bp]'
0311     'Liver_EX_4hbz(e)_[bc]'
0312     'Liver_EX_4hbz[bpL]_[bp]'
0313     'Liver_EX_4mcat(e)_[bc]'
0314     'Liver_EX_4mcat[bpL]_[bp]'
0315     'Liver_EX_5aptn(e)_[bc]'
0316     'Liver_EX_5aptn[bpL]_[bp]'
0317     'Liver_EX_7ocholate(e)_[bc]'
0318     'Liver_EX_7ocholate[bpL]_[bp]'
0319     'Liver_EX_acald[bpL]_[bp]'
0320     'Liver_EX_ade[bpL]_[bp]'
0321     'Liver_EX_aprgstrn[bpL]_[bp]'
0322     'Liver_EX_btoh(e)_[bc]'
0323     'Liver_EX_btoh[bpL]_[bp]'
0324     'Liver_EX_C02528[bpL]_[bp]'
0325     'Liver_EX_CE4890(e)_[bc]'
0326     'Liver_EX_CE4890[bpL]_[bp]'
0327     'Liver_EX_dchac[bpL]_[bp]'
0328     'Liver_EX_etha(e)_[bc]'
0329     'Liver_EX_etha[bpL]_[bp]'
0330     'Liver_EX_fald[bpL]_[bp]'
0331     'Liver_EX_fum(e)_[bc]'
0332     'Liver_EX_fum[bpL]_[bp]'
0333     'Liver_EX_gcald(e)_[bc]'
0334     'Liver_EX_gcald[bpL]_[bp]'
0335     'Liver_EX_glcn(e)_[bc]'
0336     'Liver_EX_glcn[bpL]_[bp]'
0337     'Liver_EX_glutar(e)_[bc]'
0338     'Liver_EX_glyc3p[bpL]_[bp]'
0339     'Liver_EX_gua[bpL]_[bp]'
0340     'Liver_EX_indole(e)_[bc]'
0341     'Liver_EX_indole[bpL]_[bp]'
0342     'Liver_EX_inost[bpL]_[bp]'
0343     'Liver_EX_isobut(e)_[bc]'
0344     'Liver_EX_isobut[bpL]_[bp]'
0345     'Liver_EX_isocapr(e)_[bc]'
0346     'Liver_EX_isocapr[bpL]_[bp]'
0347     'Liver_EX_isoval(e)_[bc]'
0348     'Liver_EX_isoval[bpL]_[bp]'
0349     'Liver_EX_lac_D(e)_[bc]'
0350     'Liver_EX_lac_D[bpL]_[bp]'
0351     'Liver_EX_mal_L(e)_[bc]'
0352     'Liver_EX_n8aspmd[bpL]_[bp]'
0353     'Liver_EX_ncam(e)_[bc]'
0354     'Liver_EX_phpyr[bpL]_[bp]'
0355     'Liver_EX_so3[bpL]_[bp]'
0356     'Liver_EX_succ[bpL]_[bp]'
0357     'Liver_EX_thf[bpL]_[bp]'
0358     'Liver_EX_thmmp[bpL]_[bp]'
0359     'Liver_EX_thmtp[bpL]_[bp]'
0360     'Liver_EX_thym[bpL]_[bp]'
0361     'Liver_EX_tma(e)_[bc]'
0362     'Liver_EX_tma[bpL]_[bp]'
0363     'Liver_EX_tmao(e)_[bc]'
0364     'Liver_EX_ura[bpL]_[bp]'
0365     'Liver_EX_xtsn[bpL]_[bp]'
0366     'Liver_FUMtr'
0367     'Liver_FUMtr[bpL]'
0368     'Liver_GCALDtr'
0369     'Liver_GCALDtr[bpL]'
0370     'Liver_GLCNte'
0371     'Liver_GLCNte[bpL]'
0372     'Liver_GLUTAROAT3t'
0373     'Liver_GLUTARte[bpL]'
0374     'Liver_GLYC3tr[bpL]'
0375     'Liver_GUAt[bpL]'
0376     'Liver_HMR_3951[bpL]'
0377     'Liver_HMR_7977[bpL]'
0378     'Liver_INDOLEup'
0379     'Liver_INDOLEup[bpL]'
0380     'Liver_INSTt4[bpL]'
0381     'Liver_ISOBUTtr'
0382     'Liver_ISOBUTtr[bpL]'
0383     'Liver_ISOCAPRtr'
0384     'Liver_ISOCAPRtr[bpL]'
0385     'Liver_ISOVALtr'
0386     'Liver_ISOVALtr[bpL]'
0387     'Liver_MAL_Lte'
0388     'Liver_MLTHFte2[bpL]'
0389     'Liver_N8ASPMDte[bpL]'
0390     'Liver_NCAMDe'
0391     'Liver_PHEMEt[bpL]'
0392     'Liver_PHPYRte[bpL]'
0393     'Liver_PNTOte[bpL]'
0394     'Liver_r1421[bpL]'
0395     'Liver_r1495[bpL]'
0396     'Liver_SPRMTDe[bpL]'
0397     'Liver_SUCCt4_2[bpL]'
0398     'Liver_THFt2[bpL]'
0399     'Liver_THMMPt4[bpL]'
0400     'Liver_THMTPt[bpL]'
0401     'Liver_THYMDtr2[bpL]'
0402     'Liver_THYMt[bpL]'
0403     'Liver_TMAOOx'
0404     'Liver_TMAOtr'
0405     'Liver_TMAtr'
0406     'Liver_TMAtr[bpL]'
0407     'Liver_URAt[bpL]'
0408     'Liver_XTSNtr[bpL]'};
0409 
0410 modelHM = changeRxnBounds(modelHM,Rx2,0,'b');
0411 
0412 Rx3={% 'EX_ac[u]'
0413     'EX_glyald[u]'
0414     'EX_HC00900[u]'
0415     'EX_acac[u]'
0416     'EX_acald[u]'
0417     'EX_2425dhvitd3[u]'
0418     'EX_4mptnl[u]'
0419     'EX_avite1[u]'
0420     'EX_chsterol[u]'
0421     'EX_tmndnc[u]'
0422     'EX_xolest_hs[u]'
0423     'EX_dha[u]'
0424     'EX_3bcrn[u]'
0425     'EX_3ddcrn[u]'
0426     'EX_3hdececrn[u]'
0427     'EX_3tdcrn[u]'
0428     'EX_c16dc[u]'
0429     'EX_c3dc[u]'
0430     'EX_c4dc[u]'
0431     'EX_c5dc[u]'
0432     'EX_c6crn[u]'
0433     'EX_idour[u]'
0434     'EX_3mtp[u]'
0435     'EX_adpoh[u]'
0436     'EX_C06314[u]'
0437     'EX_eidi1114ac[u]'
0438     'EX_magole_hs[u]'
0439     'EX_dxtrn[u]'
0440     'EX_xol7aone[u]'
0441     'EX_h[u]'
0442     'EX_succ[u]'
0443     'EX_xyl_D[u]'
0444     'EX_2m3hbu[u]'
0445     'EX_2m3hvac[u]'
0446     'EX_3mglutac[u]'
0447     'EX_3ohglutac[u]'
0448     'EX_thexdd[u]'
0449     'EX_sucsal[u]'
0450     'EX_xoltri24[u]'
0451     'EX_HC01444[u]'
0452     'EX_abt_D[u]'
0453     'EX_xol7ah3[u]'
0454     'EX_2h3mv[u]'
0455     'EX_Rtotal3[u]'
0456     'EX_glx[u]'
0457     'EX_2hb[u]'
0458     'EX_pac[u]'
0459     'EX_arachd[u]'
0460     'EX_2obut[u]'
0461     'EX_3mop[u]'
0462     'EX_C02356[u]'
0463     'EX_lnlc[u]'
0464     'EX_ribflv[u]'
0465     'EX_pmtcrn[u]'
0466     'EX_pcrn[u]'
0467     'EX_ttdca[u]'
0468     'EX_7dhchsterol[u]'
0469     'EX_crtsl[u]'
0470     'EX_tdchola[u]'
0471     'EX_estriol[u]'
0472     'EX_C05767[u]'
0473     %
0474     'EX_dca[u]'
0475     'EX_3hmp[u]'
0476     'EX_hestratriol[u]'
0477     'EX_34dhoxpeg[u]'
0478     'EX_C05299[u]'
0479     'EX_h2o2[u]'
0480     'EX_hexdtr[u]'
0481     'EX_3tetd7ecoacrn[u]'
0482     'EX_fum[u]'
0483     'EX_CE2028[u]'
0484     'EX_inost[u]'
0485     'EX_glyc_R[u]'
0486     'EX_q10[u]'
0487     'EX_glyc_S[u]'
0488     'EX_c51crn[u]'
0489     'EX_ppa[u]'
0490     'EX_lnlncg[u]'
0491     'EX_CE2510[u]'
0492     'EX_ptdca[u]'
0493     'EX_ttdcea[u]'
0494     'EX_34hpl[u]'
0495     'EX_pcresol[u]'
0496     'EX_12dhchol[u]'
0497     'EX_nac[u]'
0498     'EX_T4hcinnm[u]'
0499     'EX_C02528[u]'
0500     'EX_lnlccrn[u]'
0501     'EX_3hpp[u]'
0502     'EX_docosac[u]'
0503     'EX_lgnc[u]'
0504     'EX_estrone[u]'
0505     'EX_pydxn[u]'
0506     'EX_hpdece[u]'
0507     'EX_icit[u]'
0508     'EX_lanost[u]'
0509     'EX_CE2047[u]'
0510     'EX_xol27oh[u]'
0511     'EX_retn[u]'
0512     'EX_dca24g[u]'
0513     'EX_mqn8[u]'
0514     'EX_co[u]'
0515     'EX_bilglcur[u]'
0516     'EX_mqn7[u]'
0517     'EX_3thexddcoacrn[u]'
0518     'EX_C05302[u]'
0519     'EX_but[u]'
0520     'EX_c4crn[u]'
0521     'EX_CE4970[u]'
0522     'EX_dolichol_L[u]'
0523     'EX_2mcit[u]'
0524     'EX_4pyrdx[u]'
0525     'EX_tdechola[u]'
0526     'EX_aprgstrn[u]'
0527     'EX_3mlda[u]'
0528     'EX_11docrtstrn[u]'
0529     'EX_im4ac[u]'
0530     'EX_vitd3[u]'
0531     'EX_CE4968[u]'
0532     'EX_25hvitd3[u]'
0533     'EX_xol24oh[u]'
0534     'EX_xol25oh[u]'
0535     'EX_fucgalfucgalacglcgalgluside_hs[u]'
0536     'EX_galgalfucfucgalacglcgalacglcgal14acglcgalgluside_hs[u]'
0537     'EX_acmana[u]'
0538     'EX_M01966[u]'
0539     'EX_pail_hs[u]'
0540     'EX_gbside_hs[u]'
0541     'EX_CE2026[u]'
0542     'EX_M00979[u]'
0543     'EX_CE1292[u]'
0544     'EX_tststeroneglc[u]'
0545     'EX_5adtststeroneglc[u]'
0546     'EX_homoval[u]'
0547     'EX_3mox4hoxm[u]'
0548     'EX_eic21114tr[u]'
0549     'EX_C14768[u]'
0550     };
0551 for i =1 : length(Rx3)
0552     modelHM.ub(strmatch( Rx3{i},modelHM.rxns,'exact')) = modelHM.ub(strmatch( Rx3{i},modelHM.rxns,'exact'))*907.2;
0553 end
```

---

Generated on Thu 14-May-2020 13:05:49 by **m2html** © 2005
