# Supplementary material for: Personalized whole‐body models integrate metabolism, physiology, and the gut microbiome
Source: Mol Syst Biol. 2020 May 28;16(5):e8982. doi: 10.15252/msb.20198982 (PMC7285886; doi:10.15252/msb.20198982)
Supplement: Supplementary file 22 — Dataset EV1 [file MSB-16-e8982-s022.zip › PSCM_toolbox/PSCM_toolbox_doc/src/hostMicrobeInteraction/combineHarveyMicrotiota.html]

Description of combineHarveyMicrotiota


# combineHarveyMicrotiota

## PURPOSE

**This function combines harvey and a microbial community model**

## SYNOPSIS

**function modelHM = combineHarveyMicrotiota(modelH, modelM, couplingConstraint)**

## DESCRIPTION

```
 This function combines harvey and a microbial community model

 function modelHM = combineHarveyMicrotiota(modelH, modelM, couplingConstraint)

 INPUT
 modelH                Whole-body metabolic model structure
 modelM                Microbiota model structure
 couplingConstraint    coupling constraint for microbiome model (default:
                       20000, as used in the whole-body metabolic model

 OUTPUT
 modelHM               Model structure containing both Whole-body metabolic model and microbiota in one
                       matrix

 Ines Thiele May 2016
 replace Ex_AA[u] with an artificial transport reaction [luM] -- > [luLI]
 updated to new structure we use ([d],[fe],[u]) for microbes - Nov 2017 IT
```

## CROSS-REFERENCE INFORMATION

This function calls:

- createModelNewCompartment This function converts a two compartment metabolic model into a three compartment metabolic model model

This function is called by:

- runIEM\_HH This script predicts known biomarker metabolites in

## SOURCE CODE

```
0001 function modelHM = combineHarveyMicrotiota(modelH, modelM, couplingConstraint)
0002 % This function combines harvey and a microbial community model
0003 %
0004 % function modelHM = combineHarveyMicrotiota(modelH, modelM, couplingConstraint)
0005 %
0006 % INPUT
0007 % modelH                Whole-body metabolic model structure
0008 % modelM                Microbiota model structure
0009 % couplingConstraint    coupling constraint for microbiome model (default:
0010 %                       20000, as used in the whole-body metabolic model
0011 %
0012 % OUTPUT
0013 % modelHM               Model structure containing both Whole-body metabolic model and microbiota in one
0014 %                       matrix
0015 %
0016 % Ines Thiele May 2016
0017 % replace Ex_AA[u] with an artificial transport reaction [luM] -- > [luLI]
0018 % updated to new structure we use ([d],[fe],[u]) for microbes - Nov 2017 IT
0019 
0020 %TODO make this code COBRA v3 compatible
0021 if ~isfield(modelM,'A')
0022     modelM.A = [modelM.S;modelM.C];
0023     modelM.b = [modelM.b;modelM.d];
0024     modelM.b(1:end) = 0;
0025     modelM.csense = [modelM.csense;modelM.dsense];
0026     modelM.mets = [modelM.mets;modelM.ctrs];
0027     modelM = rmfield(modelM,'C');
0028     modelM = rmfield(modelM,'d');
0029     modelM = rmfield(modelM,'ctrs');
0030     modelM = rmfield(modelM,'dsense');
0031     modelM = rmfield(modelM,'S');
0032 end
0033 if ~isfield(modelH,'A')
0034     modelH.A = [modelH.S;modelH.C];
0035     modelH.b = [modelH.b;modelH.d];
0036     modelH.b(1:end) = 0;
0037     modelH.mets = [modelH.mets;modelH.ctrs];
0038     modelH.csense = [modelH.csense;modelH.dsense];
0039     modelH = rmfield(modelH,'C');
0040     modelH = rmfield(modelH,'d');
0041     modelH = rmfield(modelH,'ctrs');
0042     modelH = rmfield(modelH,'dsense');
0043     modelH = rmfield(modelH,'S');
0044 end
0045 modelM.b(1:end) = 0;
0046 
0047 % fix csense
0048 for i=1 :length(modelM.csense)
0049     tmp(i,1)=modelM.csense(i);
0050 end
0051 modelM.csense = tmp;
0052 % fix model.met
0053 if length(modelM.mets)<size(modelM.A,1)
0054     tmp1 = size(modelM.A,1)-length(modelM.mets);
0055     tmp2=length(modelM.mets);
0056     for i =1 : tmp1
0057         modelM.mets{tmp2+i}=num2str(i);
0058     end
0059 end
0060 if ~exist('couplingConstraint','var')
0061     couplingConstraint = 20000; % same as Harvey
0062 end
0063 
0064 factor = 1000; % to adjust to mmol
0065 modelH.S=modelH.A;
0066 modelM.S=modelM.A;
0067 % % remove a few reactions
0068 modelMO=modelM;
0069 
0070 % check whether the model contains [d] compartment
0071 if ~isempty(find(~cellfun(@isempty,strfind(modelM.rxns,'[d]'))))
0072     % remove exchange reactions
0073     % Diet exchange: 'EX_met[d]': 'met[d] <=>' and
0074     % Fecal exchanges: 'EX_met[fe]': 'met[fe] <=>'
0075     ExR = modelM.rxns(strmatch('EX_',modelM.rxns));
0076     % if isempty(ExR)
0077     %     ExR = (find(~cellfun(@isempty,strfind(modelM.rxns,'EX_'))));
0078     % end
0079     modelM.rev = zeros(length(modelM.rxns),1);
0080     modelM.rev(modelM.lb<0)=1;
0081     modelM = removeRxns(modelM,ExR);
0082     % convert Diet transport reactions
0083     % Diet transporter: 'DUt_met': 'met[d] -> met[u]'
0084     ExR = strmatch('DUt_',modelM.rxns);
0085     % get all [d] metabolites
0086     EXMD = modelM.mets(strmatch('\[d\]',modelM.mets));
0087     % rename those reactions
0088     modelM.rxns = regexprep(modelM.rxns, 'DUt_','Micro_EX_');
0089     modelM.rxns(ExR) = strcat(modelM.rxns(ExR), '[luLI]_[luM]');
0090     % make those reactions reversible
0091     modelM.mets = regexprep(modelM.mets, '\[d\]','\[luLI\]');
0092     modelM.mets = regexprep(modelM.mets, '\[u\]','\[luM\]');
0093 elseif ~isempty(find(~cellfun(@isempty,strfind(modelM.rxns,'[u]')))) % contains only [u] compartment
0094     % convert Ex_met[u] reactions into transport reactions
0095     [modelM] = createModelNewCompartment(modelM,'u','luLI','large intestinal lumen',-1000,1000,1);
0096     modelM.rxns = regexprep(modelM.rxns,'\[u\]_\[luLI\]','\[luLI\]_\[luM\]');
0097     modelM.rxns = regexprep(modelM.rxns,'^EX_','Micro_EX_');
0098     modelM.mets = regexprep(modelM.mets, '\[u\]','\[luM\]');
0099     ExR = strmatch('Micro_EX_',modelM.rxns);    
0100     EXMD = modelM.mets(strmatch('\[luM\]',modelM.mets));
0101     %  it seems that these models do not have a community biomass
0102     % I add it for the moment but
0103 end
0104 modelM.lb(ExR) = -1000;
0105 modelM.ub(ExR) = 1000;
0106 % remove fecal transport reactions
0107 % Fecal transporter: 'UFEt_met': 'met[u] -> met[fe]'
0108 ExR = strmatch('UFEt_',modelM.rxns);
0109 modelM = removeRxns(modelM,modelM.rxns(ExR));
0110 
0111 % remove slacks from exchanges
0112 ExR = strmatch('Micro_EX_',modelM.rxns);
0113 SL = strmatch('slack_',modelM.mets);
0114 modelM.S(SL,ExR)=0;
0115 
0116 % adjust further constraints on modelM
0117 for i = 1 : length(modelM.rxns)
0118     if ~isempty(strfind(modelM.rxns{i},'biomass[c]tr'))
0119         %    RM(i,1)=1;
0120         modelM=changeRxnBounds(modelM,modelM.rxns{i},0,'b');%Make sure microbes cannot share biomass between each other
0121     end
0122 end
0123 
0124 % add community biomass
0125 % rename biomass[c] to microbiota_LI_biomass
0126 if ~isempty(strmatch('microbeBiomass[luM]',modelM.mets))
0127     modelM.mets{strmatch('microbeBiomass[luM]',modelM.mets)} = 'microbiota_LI_biomass[luM]';
0128 elseif isempty(find(modelM.S(:,strmatch('communityBiomass',modelM.mets))>0))
0129     % it seems that in the newer version no product side has been defined
0130     modelM.mets{end+1}= 'microbiota_LI_biomass[luM]';
0131     modelM.S(end+1,strmatch('communityBiomass',modelM.rxns))=1;
0132     modelM.b(end+1)=0;
0133     modelM.csense(end+1,1)='E';
0134 else
0135     error
0136 end
0137 
0138 % remove the constraints on some of the microbial demands
0139 modelM.lb(find(modelM.lb>=0))=0;
0140 modelM=changeRxnBounds(modelM,'EX_biomass[c]',0,'b');%make sure biomass isn't being taken up or secreted
0141 
0142 modelM2=modelM;
0143 % add a reaction to modelM that transports the biomass to luLI and then the
0144 % fe and an excretion reaction
0145 [modelM2,rxnIDexists] = addReaction(modelM2,'LI_EX_microbiota_LI_biomass[luLI]_[fe]',{'microbiota_LI_biomass[luM]','microbiota_LI_biomass[fe]'},[-1 1],false);
0146 modelM2.subSystems{end}='Transport, biofluid';
0147 [modelM2,rxnIDexists] = addReaction(modelM2,'Excretion_EX_microbiota_LI_biomass[fe]',{'microbiota_LI_biomass[fe]'},[-1],false);
0148 modelM2.subSystems{end}='Exchange/demand reaction';
0149 a = length(modelM2.csense);
0150 for i = 1 : (length(modelM2.mets)-length(modelM2.csense))
0151     modelM2.csense(a+i,1)='E';
0152 end
0153 modelM2.lb(find(modelM2.lb<0))=-1000*1000;
0154 modelM2.ub(find(modelM2.ub>0))=1000*1000;
0155 
0156 modelM2.S(find(modelM2.S==400))=couplingConstraint;% change coupling constraint
0157 modelM2.S(find(modelM2.S==-400))=-couplingConstraint;% change coupling constraint
0158 modelM2.S(find(modelM2.S==200000))=couplingConstraint;% change coupling constraint - seems to be an error in Federico's scripts
0159 modelM2.S(find(modelM2.S==-200000))=-couplingConstraint;% change coupling constraint - seems to be an error in Federico's scripts
0160 
0161 modelH = rmfield(modelH,'A');
0162 modelM2 = rmfield(modelM2,'A');
0163 
0164 % check that both models do not have overlapping reactions
0165 Rem = intersect(modelH.rxns,modelM2.rxns);
0166 if ~isempty(Rem)
0167     % remove reactions from modelM2
0168     modelM2 = removeRxns(modelM2,Rem);
0169 end
0170 
0171 [modelHM] = mergeTwoModels(modelH,modelM2,1,0);
0172 modelHM.A = modelHM.S;
0173 % make sure that all new metabolites in luLI compartment can be excreted
0174 
0175 % EXMD = modelHM.mets(strmatch('\[d\]',modelHM.mets));
0176 for i = 1 : length(EXMD)
0177     Fe = regexrep(EXMD{i},'\[luLI\]','\[fe\]');
0178     if isempty(strmatch(strcat('LI_EX_',EXMD{i},'_[fe]'),modelHM.rxns))
0179         [modelHM,rxnIDexists] = addReaction(modelHM,strcat('LI_EX_',EXMD{i},'_[fe]'),{EXMD{i},Fe},[-1 1],false);
0180         modelHM.subSystems{end}='Transport, biofluid';
0181     end
0182     if isempty(strmatch(strcat('Excretion_EX_',fe),modelHM.rxns))
0183         [modelHM,rxnIDexists] = addReaction(modelHM,strcat('Excretion_EX_',fe),{fe},[-1],false);
0184         modelHM.subSystems{end}='Exchange/demand reaction';
0185     end
0186 end
0187 a = length(modelHM.csense);
0188 for i = 1 : (length(modelHM.mets)-length(modelHM.csense))
0189     modelHM.csense(a+i,1)='E';
0190 end
0191 
0192 % flag microbial reactions
0193 modelHM.Microbiota = ones(length(modelHM.rxns),1);
0194 modelHM.Microbiota(1:length(modelH.rxns))=0;
0195 
0196 % adjust communityBiomass to percentage rather than fraction, in accordance
0197 % to whole-body objective
0198 modelHM.S(:,strmatch('communityBiomass',modelHM.rxns)) = 100*modelHM.S(:,strmatch('communityBiomass',modelHM.rxns)) ;
0199 modelHM.A=modelHM.S;
0200 modelHM = rmfield(modelHM,'rules');
0201 modelHM = rmfield(modelHM,'grRules');
0202 modelHM = convertOldStyleModel(modelHM);
0203 
0204 for i = 1 : length(modelHM.rxns)
0205     modelHM.rules(i,1) = {''};
0206 end
0207 
0208 modelHM.genes = {''};
```

---

Generated on Thu 14-May-2020 13:05:49 by **m2html** © 2005
