# Supplementary material for: Personalized whole‐body models integrate metabolism, physiology, and the gut microbiome
Source: Mol Syst Biol. 2020 May 28;16(5):e8982. doi: 10.15252/msb.20198982 (PMC7285886; doi:10.15252/msb.20198982)
Supplement: Supplementary file 22 — Dataset EV1 [file MSB-16-e8982-s022.zip › PSCM_toolbox/PSCM_toolbox_doc/src/hostMicrobeInteraction/AGORAEssentialMetabolites.html]

Description of AGORAEssentialMetabolites


# AGORAEssentialMetabolites

## PURPOSE

**This file contains a list of metabolites required by the AGORA microbial**

## SYNOPSIS

**This is a script file.**

## DESCRIPTION

```
 This file contains a list of metabolites required by the AGORA microbial
 reconstructions to grow.
```

## CROSS-REFERENCE INFORMATION

This function calls:


This function is called by:

- setDietConstraints This function sets diet constraints onto the bounds of the diet uptale

## SOURCE CODE

```
0001 % This file contains a list of metabolites required by the AGORA microbial
0002 % reconstructions to grow.
0003 
0004 AGORAessential ={'EX_12dgr180[u]'
0005 'EX_26dap_M[u]'
0006 'EX_2obut[u]'
0007 'EX_3mop[u]'
0008 'EX_4hbz[u]'
0009 'EX_ac[u]'
0010 'EX_acgam[u]'
0011 'EX_acmana[u]'
0012 'EX_ade[u]'
0013 'EX_adn[u]'
0014 'EX_adocbl[u]'
0015 'EX_ala_D[u]'
0016 'EX_ala_L[u]'
0017 'EX_arab_D[u]'
0018 'EX_arg_L[u]'
0019 'EX_asn_L[u]'
0020 'EX_btn[u]'
0021 'EX_ca2[u]'
0022 'EX_cbl1[u]'
0023 'EX_cgly[u]'
0024 'EX_chor[u]'
0025 'EX_chsterol[u]'
0026 'EX_cit[u]'
0027 'EX_cl[u]'
0028 'EX_cobalt2[u]'
0029 'EX_csn[u]'
0030 'EX_cu2[u]'
0031 'EX_cys_L[u]'
0032 'EX_dad_2[u]'
0033 'EX_dcyt[u]'
0034 'EX_ddca[u]'
0035 'EX_dgsn[u]'
0036 'EX_fe2[u]'
0037 'EX_fe3[u]'
0038 'EX_fol[u]'
0039 'EX_for[u]'
0040 'EX_gal[u]'
0041 'EX_glc_D[u]'
0042 'EX_glu_L[u]'
0043 'EX_gly[u]'
0044 'EX_glyc3p[u]'
0045 'EX_glyc[u]'
0046 'EX_gthox[u]'
0047 'EX_gua[u]'
0048 'EX_h2s[u]'
0049 'EX_h[u]'
0050 'EX_his_L[u]'
0051 'EX_hxan[u]'
0052 'EX_ile_L[u]'
0053 'EX_k[u]'
0054 'EX_lanost[u]'
0055 'EX_leu_L[u]'
0056 'EX_lys_L[u]'
0057 'EX_met_L[u]'
0058 'EX_mg2[u]'
0059 'EX_mn2[u]'
0060 'EX_mqn7[u]'
0061 'EX_mqn8[u]'
0062 'EX_nac[u]'
0063 'EX_ncam[u]'
0064 'EX_nmn[u]'
0065 'EX_ocdca[u]'
0066 'EX_ocdcea[u]'
0067 'EX_orn[u]'
0068 'EX_phe_L[u]'
0069 'EX_pheme[u]'
0070 'EX_pi[u]'
0071 'EX_pnto_R[u]'
0072 'EX_pro_L[u]'
0073 'EX_ptrc[u]'
0074 'EX_pydx[u]'
0075 'EX_pydxn[u]'
0076 'EX_q8[u]'
0077 'EX_rib_D[u]'
0078 'EX_ribflv[u]'
0079 'EX_ser_L[u]'
0080 'EX_sheme[u]'
0081 'EX_so4[u]'
0082 'EX_spmd[u]'
0083 'EX_thm[u]'
0084 'EX_thr_L[u]'
0085 'EX_thymd[u]'
0086 'EX_trp_L[u]'
0087 'EX_ttdca[u]'
0088 'EX_tyr_L[u]'
0089 'EX_ura[u]'
0090 'EX_val_L[u]'
0091 'EX_xan[u]'
0092 'EX_xyl_D[u]'
0093 'EX_zn2[u]'
0094 % new
0095 'EX_arg_L[u]'
0096 'EX_ca2[u]'
0097 'EX_cgly[u]'
0098 'EX_cl[u]'
0099 'EX_cobalt2[u]'
0100 'EX_cu2[u]'
0101 'EX_fe2[u]'
0102 'EX_fol[u]'
0103 'EX_k[u]'
0104 'EX_mg2[u]'
0105 'EX_mn2[u]'
0106 'EX_mqn7[u]'
0107 'EX_ocdca[u]'
0108 'EX_pheme[u]'
0109 'EX_ribflv[u]'
0110 'EX_so4[u]'
0111 'EX_spmd[u]'
0112 'EX_thm[u]'
0113 'EX_trp_L[u]'
0114 'EX_zn2[u]'
0115 'EX_pnto_R[u]'
0116 'EX_sheme[u]'
0117 'EX_thymd[u]'
0118 'EX_pydx[u]'
0119 'EX_26dap_M[u]'
0120 'EX_ala_L[u]'
0121 'EX_fe3[u]'
0122 'EX_pi[u]'
0123 'EX_ttdca[u]'
0124 'EX_mqn8[u]'
0125 'EX_q8[u]'
0126 'EX_2dmmq8[u]'
0127 'EX_btn[u]'
0128 'EX_nmn[u]'
0129 'EX_asn_L[u]'
0130 'EX_adpcbl[u]'
0131 'EX_hxan[u]'
0132 'EX_gthrd[u]'
0133 'EX_4hbz[u]'
0134 'EX_12dgr180[u]'
0135 'EX_nac[u]'
0136 'EX_gthox[u]'
0137 'EX_ptrc[u]'
0138 'EX_ura[u]'
0139 'EX_ddca[u]'
0140 'EX_lys_L[u]'
0141 'EX_ala_D[u]'
0142 'EX_arab_D[u]'
0143 'EX_4abz[u]'
0144 'EX_ile_L[u]'
0145 'EX_met_L[u]'
0146 'EX_ocdcea[u]'
0147 'EX_tyr_L[u]'
0148 'EX_val_L[u]'
0149 'EX_his_L[u]'
0150 'EX_leu_L[u]'
0151 'EX_pro_L[u]'
0152 'EX_cit[u]'
0153 'EX_phe_L[u]'
0154 'EX_thr_L[u]'
0155 'EX_adn[u]'
0156 'EX_3mop[u]'
0157 'EX_cys_L[u]'
0158 'EX_gal[u]'
0159 'EX_ser_L[u]'
0160 'EX_orn[u]'
0161 'EX_h2s[u]'
0162 'EX_xyl_D[u]'
0163 'EX_2obut[u]'
0164 'EX_lanost[u]'
0165 'EX_rib_D[u]'
0166 'EX_dcyt[u]'
0167 'EX_glu_L[u]'
0168 'EX_ac[u]'
0169 'EX_ncam[u]'
0170 'EX_cytd[u]'
0171 'EX_amet[u]'
0172 'EX_no2[u]'
0173 'EX_cbl1[u]'
0174 'EX_gly[u]'
0175 'EX_gln_L[u]'
0176 'EX_acgam[u]'
0177 'EX_dad_2[u]'
0178 'EX_ade[u]'
0179 'EX_gua[u]'
0180 'EX_for[u]'
0181 'EX_h[u]'
0182 'EX_pydxn[u]'
0183 'EX_dgsn[u]'
0184 'EX_malt[u]'
0185 'EX_fald[u]'
0186 'EX_amp[u]'
0187 'EX_chor[u]'
0188 'EX_glyc[u]'
0189 'EX_glyc3p[u]'
0190 'EX_h2o[u]'
0191 'EX_chsterol[u]'
0192 'EX_acmana[u]'
0193 'EX_xan[u]'
0194 'EX_gsn[u]'};
0195 
0196 AGORAessential = unique(AGORAessential);
```

---

Generated on Thu 14-May-2020 13:05:49 by **m2html** © 2005
