# Supplementary material for: Personalized whole‐body models integrate metabolism, physiology, and the gut microbiome
Source: Mol Syst Biol. 2020 May 28;16(5):e8982. doi: 10.15252/msb.20198982 (PMC7285886; doi:10.15252/msb.20198982)
Supplement: Supplementary file 22 — Dataset EV1 [file MSB-16-e8982-s022.zip › PSCM_toolbox/PSCM_toolbox_doc/src/hostMicrobeInteraction/setSimulationConstraints.html]

Description of setSimulationConstraints


# setSimulationConstraints

## PURPOSE

**This function sets the remaining simulation constraints that I identified during the**

## SYNOPSIS

**function [model] = setSimulationConstraints(model)**

## DESCRIPTION

```
 This function sets the remaining simulation constraints that I identified during the
 debugging
 This function needs to be applied to GF and MyWB< models that have been created with GF
 before Dec 2017.

 function [model] = setSimulationConstraints(model)

 INPUT
 model     model structure (whole body metabolic model)

 OUTPUT
 model     model structure with updated constraints as defined in this file 

 Ines Thiele Dec 2017
```

## CROSS-REFERENCE INFORMATION

This function calls:


This function is called by:

- perform\_BMR\_newData This script repeats the simulation described in Thiele et al., "Personalized whole-body models integrate metabolism, physiology, and the gut microbiome", Method section 3.9.2 Validation of the parameters in an independent data set.
- perform\_sensi\_BMR\_all This script repeats the simulation described in Thiele et al.,

## SOURCE CODE

```
0001 function [model] = setSimulationConstraints(model)
0002 % This function sets the remaining simulation constraints that I identified during the
0003 % debugging
0004 % This function needs to be applied to GF and MyWB< models that have been created with GF
0005 % before Dec 2017.
0006 %
0007 % function [model] = setSimulationConstraints(model)
0008 %
0009 % INPUT
0010 % model     model structure (whole body metabolic model)
0011 %
0012 % OUTPUT
0013 % model     model structure with updated constraints as defined in this file
0014 %
0015 % Ines Thiele Dec 2017
0016 
0017 warning off;
0018 model = changeRxnBounds(model,'Liver_EX_4abut[bdL]_[bd]',0,'u');
0019 model = changeRxnBounds(model,'Liver_EX_tym[bdL]_[bd]',0,'u');
0020 model = changeRxnBounds(model,'Liver_EX_i[bdL]_[bd]',0,'u');
0021 model = changeRxnBounds(model,'Liver_EX_ca2[bdL]_[bd]',0,'u');
0022 model = changeRxnBounds(model,'Liver_EX_na1[bdL]_[bd]',0,'u');
0023 model = changeRxnBounds(model,'Liver_EX_h[bdL]_[bd]',0,'u');
0024 
0025 
0026 model = changeRxnBounds(model,'Colon_EX_o2[luC]_[luLI]',0,'l');
0027 model = changeRxnBounds(model,'Colon_EX_strch2[luC]_[luLI]',0,'l');
0028 model = changeRxnBounds(model,'Colon_EX_coke[luC]_[luLI]',0,'b');
0029 model = changeRxnBounds(model,'sIEC_EX_strch1[luI]_[luSI]',0,'b');
0030 model = changeRxnBounds(model,'sIEC_EX_strch2[luI]_[luSI]',0,'b');
0031 
0032 R = (find(~cellfun(@isempty,strfind(model.rxns, 'BileDuct_EX_'))));
0033 model.ub(R) = 0;
0034 % needed to allow pancreas to maintain
0035 model = changeRxnBounds(model,'BileDuct_EX_Rtotal[bd]_[luSI]',15,'u');
0036 model = changeRxnBounds(model,'BileDuct_EX_Rtotal2[bd]_[luSI]',10,'u');
0037 model = changeRxnBounds(model,'BileDuct_EX_pchol_hs[bd]_[luSI]',10,'u');
0038 model = changeRxnBounds(model,'BileDuct_EX_tag_hs[bd]_[luSI]',10,'u');
0039 model = changeRxnBounds(model,'BileDuct_EX_pe_hs[bd]_[luSI]',10,'u');
0040 model = changeRxnBounds(model,'BileDuct_EX_mag_hs[bd]_[luSI]',10,'u');
0041 model = changeRxnBounds(model,'BileDuct_EX_dag_hs[bd]_[luSI]',10,'u');
0042 
0043 model = changeRxnBounds(model,'BileDuct_EX_3dhcdchol[bd]_[luSI]',1000,'u');
0044 model = changeRxnBounds(model,'BileDuct_EX_3dhchol[bd]_[luSI]',1000,'u');
0045 model = changeRxnBounds(model,'BileDuct_EX_3dhdchol[bd]_[luSI]',1000,'u');
0046 %model = changeRxnBounds(model,'BileDuct_EX_ca3s[bd]_[luSI]',1000,'u');
0047 model = changeRxnBounds(model,'BileDuct_EX_cdca24g[bd]_[luSI]',1000,'u');
0048 %model = changeRxnBounds(model,'BileDuct_EX_dca3s[bd]_[luSI]',1000,'u');
0049 %model = changeRxnBounds(model,'BileDuct_EX_gca3s[bd]_[luSI]',1000,'u');
0050 %model = changeRxnBounds(model,'BileDuct_EX_gcdca3s[bd]_[luSI]',1000,'u');
0051 %model = changeRxnBounds(model,'BileDuct_EX_gdca3s[bd]_[luSI]',1000,'u');
0052 %model = changeRxnBounds(model,'BileDuct_EX_gudca3s[bd]_[luSI]',1000,'u');
0053 %model = changeRxnBounds(model,'BileDuct_EX_tca3s[bd]_[luSI]',1000,'u');
0054 %model = changeRxnBounds(model,'BileDuct_EX_tcdca3s[bd]_[luSI]',1000,'u');
0055 %model = changeRxnBounds(model,'BileDuct_EX_tdca3s[bd]_[luSI]',1000,'u');
0056 model = changeRxnBounds(model,'BileDuct_EX_thyochol[bd]_[luSI]',1000,'u');
0057 %model = changeRxnBounds(model,'BileDuct_EX_tudca3s[bd]_[luSI]',1000,'u');
0058 %model = changeRxnBounds(model,'BileDuct_EX_udca3s[bd]_[luSI]',1000,'u');
0059 
0060 model = changeRxnBounds(model,'Scord_EX_glc_D(e)_[csf]',0,'u');
0061 model = changeRxnBounds(model,'Brain_EX_glc_D(e)_[csf]',-400,'u');
0062 %
0063 % % set upper bound to 0
0064 % U = {'Liver_sink_hdca(c)'
0065 %     'Liver_sink_lnlc(c)'
0066 %     'Liver_sink_lnlccoa(c)'
0067 %     'Liver_sink_lnlncacoa(c)'
0068 %     'Liver_sink_lnlncgcoa(c)'
0069 %     'Liver_sink_odecoa(c)'
0070 %     'Liver_sink_pmtcoa(c)'
0071 %     'Liver_sink_stcoa(c)'
0072 %     'Liver_sink_tag_hs(c)'
0073 %     'Liver_sink_tmndnc(c)'
0074 %     'Liver_sink_tmndnccoa(c)'
0075 % %     'Muscle_EX_dag_hs(e)_[bc]'
0076 % %     'Muscle_EX_acetone(e)_[bc]'
0077 %     };
0078 % model.ub(find(ismember(model.rxns,U))) = 0;
0079 % % L = {'Muscle_EX_hco3(e)_[bc]'
0080 % % %     'Muscle_EX_glyald(e)_[bc]'
0081 % % %     'Muscle_EX_for(e)_[bc]'
0082 % % %     'Muscle_EX_pyr(e)_[bc]'
0083 % %  %   'Muscle_EX_k(e)_[bc]'
0084 % %     'Muscle_EX_h2o2(e)_[bc]'
0085 % %     };
0086 % % model.lb(find(ismember(model.rxns,L))) = 0;
0087 % %
0088 % %UE={'Muscle_EX_ala_L(e)_[bc]'};
0089 % % model.ub(find(ismember(model.rxns,UE))) = 1000000;
0090 %
```

---

Generated on Thu 14-May-2020 13:05:49 by **m2html** © 2005
