# Supplementary material for: Personalized whole‐body models integrate metabolism, physiology, and the gut microbiome
Source: Mol Syst Biol. 2020 May 28;16(5):e8982. doi: 10.15252/msb.20198982 (PMC7285886; doi:10.15252/msb.20198982)
Supplement: Supplementary file 22 — Dataset EV1 [file MSB-16-e8982-s022.zip › PSCM_toolbox/PSCM_toolbox_doc/src/hostMicrobeInteraction/calculateCorrelations.html]

Description of calculateCorrelations


# calculateCorrelations

## PURPOSE

**% This script creates microbiome models for HMP individuals using the Microbiome Modeling Toolbox**

## SYNOPSIS

**This is a script file.**

## DESCRIPTION

```
% This script creates microbiome models for HMP individuals using the Microbiome Modeling Toolbox 

 Almut Heinken
 
 path where to save results
```

## CROSS-REFERENCE INFORMATION

This function calls:


This function is called by:

## SOURCE CODE

```
0001 %% This script creates microbiome models for HMP individuals using the Microbiome Modeling Toolbox
0002 %
0003 % Almut Heinken
0004 %
0005 % path where to save results
0006 mkdir('microbiomeModels');
0007 resPath=(strcat(currentDir,'/microbiomeModels'));
0008 % path to where the COBRA Toolbox is located
0009 global CBTDIR
0010 toolboxPath=CBTDIR;
0011 % path to and name of the file with dietary information.
0012 dietFilePath=[CBTDIR filesep 'papers' filesep '2018_microbiomeModelingToolbox' filesep 'resource' filesep 'AverageEuropeanDiet'];
0013 % path to and name of the file with abundance information.
0014 abunFilePath=[CBTDIR filesep 'papers' filesep '2018_microbiomeModelingToolbox' filesep 'examples' filesep 'normCoverage.csv'];
0015 indInfoFilePath='';
0016 % name of objective function of organisms
0017 objre={'EX_biomass(e)'};
0018 %the output is vectorized picture, change to '-dpng' for .png
0019 figForm = '-depsc';
0020 % number of cores dedicated for parallelization
0021 numWorkers = 12;
0022 % autofix for names mismatch
0023 autoFix = 1;
0024 % if outputs in open formats should be produced for each section (1=T)
0025 compMod = 0; 
0026 % if documentations on patient health status is provided (0 not 1 yes)
0027 patStat = 0; 
0028 % to enable also rich diet simulations
0029 rDiet = 0; 
0030 % if if to use an external solver and save models with diet
0031 extSolve = 0; 
0032 % the type of FVA function to use to solve
0033 fvaType = 1; 
0034 % To tourn off the autorun to be able to manually execute each part of the pipeline.
0035 autorun=1; 
0036 %END OF REQUIRED INPUT VARIABLES
0037 %
0038 %PIPELINE LAUNCHER
0039 [init, modelPath, toolboxPath, resPath, dietFilePath, abunFilePath, indInfoFilePath, objre, figForm, numWorkers, autoFix, compMod, rDiet, extSolve, fvaType, autorun] = initMgPipe(modPath, toolboxPath, resPath, dietFilePath, abunFilePath, indInfoFilePath, objre, figForm, numWorkers, autoFix, compMod, rDiet, extSolve, fvaType, autorun);
0040 
0041 %% Calculate fold changes for the metabolic objectives betwene microbiome-associated and germfree
0042 % Here I need the part that puts RxnF./RxnF_GF together for all
0043 % individuals to create the FoldChanges variable.
0044 
0045 FoldChanges={[],'SRS011061','SRS011239','SRS011302','SRS011405','SRS011586','SRS012273','SRS012902','SRS013521','SRS014313','SRS014459','SRS014613','SRS014979','SRS015065','SRS015133','SRS015190','SRS015217','SRS015369','SRS016095','SRS016203','SRS016495','SRS016517','SRS017521','SRS017701','SRS018656','SRS019267','SRS019601','SRS019968','SRS020328','SRS021948','SRS022071','SRS022137','SRS022524','SRS022713','SRS023346','SRS023583','SRS023829','SRS024009','SRS024265','SRS024388','SRS042284','SRS043001','SRS043411','SRS048870','SRS049995','SRS050752','SRS051882','SRS052697','SRS053214','SRS053335','SRS053398','SRS054590','SRS054956','SRS055982','SRS057478','SRS057717','SRS058723','SRS063040','SRS063985','SRS064276','SRS064557','SRS065504','SRS075398','SRS077730','SRS078176','SRS011084','SRS011134','SRS011271','SRS011452','SRS011529','SRS013158','SRS013215','SRS013476','SRS013687','SRS013800','SRS013951','SRS014235','SRS014287','SRS014683','SRS014923','SRS015264','SRS015578','SRS015663','SRS015782','SRS015794','SRS015854','SRS015960','SRS016018','SRS016056','SRS016267','SRS016335','SRS016753','SRS016954','SRS016989','SRS017103','SRS017191','SRS017247','SRS017307','SRS017821','SRS018133','SRS018313','SRS018351','SRS018427','SRS018575','SRS018817','SRS019030','SRS019161','SRS019397','SRS019582','SRS019685','SRS019787','SRS019910','SRS020233','SRS020869','SRS021484','SRS022609','SRS023176','SRS023526','SRS023914','SRS023971','SRS024075','SRS024132','SRS024331','SRS024435','SRS024549','SRS024625','SRS042628','SRS043701','SRS045004','SRS045645','SRS045713','SRS047014','SRS047044','SRS048164','SRS049164','SRS049712','SRS049900','SRS049959','SRS050299','SRS050422','SRS050925','SRS051031','SRS052027','SRS056259','SRS056519','SRS058770','SRS062427','SRS064645';'Liver_PCSF',4.36838400000000,4.25779200000000,4.47897600000000,4.53427200000000,3.04128000000000,4.86604800000000,0.218000000000000,2.15000000000000,4.42368000000000,3.98131200000000,3.31776000000000,4.25779200000000,3.98131200000000,4.53427200000000,2.72800000000000,4.31308800000000,0,3.98131200000000,4.03660800000000,4.86604800000000,4.86604800000000,4.31308800000000,2.91000000000000,5.25312000000000,1.47000000000000,3.76012800000000,4.09190400000000,3.42835200000000,3.53894400000000,4.86604800000000,2.93068800000000,3.26246400000000,2.99978000000000,1.16600000000000,0.678000000000000,0.664000000000000,4.53427200000000,4.47897600000000,1.29200000000000,5.30841600000000,3.72800000000000,4.86604800000000,4.64486400000000,3.76012800000000,3.87072000000000,4.31308800000000,3.04128000000000,4.86604800000000,3.59424000000000,3.81542400000000,4.25779200000000,3.98131200000000,2.93068800000000,4.75545600000000,2.69328000000000,2.01800000000000,3.26246400000000,4.53427200000000,3.31776000000000,1.27400000000000,4.25779200000000,2.39800000000000,4.86604800000000,3.76012800000000,3.40213407012864,3.17904331143168,3.34636138045440,0,3.29058869078016,0.682000000000000,0.478000000000000,3.34636138045440,4.12717903589376,1.23400000000000,5.13108745003008,4.79645131198464,4.01563365654528,4.12717903589376,3.23481600110592,4.23872441524224,3.68099751849984,3.56945213915136,4.01563365654528,2.30600000000000,3.56945213915136,4.69646000000000,4.90799669133312,0.734000000000000,1.85600000000000,3.56945213915136,0.728000000000000,3.51367944947712,1.72600000000000,3.23481600110592,5.57726896742400,0,3.68099751849984,4.51758786361344,0,4.74067862231040,2.90017986306048,3.84831558752256,2.25372000000000,4.79645131198464,3.82068000000000,5.13108745003008,2.49400000000000,3.34636138045440,4.79645131198464,3.74652000000000,4.46181517393920,3.23481600110592,3.40213407012864,3.40213407012864,3.17904331143168,0.994000000000000,3.34636138045440,4.12717903589376,0.960000000000000,3.79254289784832,3.34636138045440,3.21000000000000,4.46181517393920,1.21400000000000,3.51367944947712,4.90799669133312,5.57726896742400,3.29800000000000,2.99000000000000,1.28800000000000,3.56945213915136,2.39800000000000,3.45790675980288,3.56945213915136,3.40213407012864,0.948000000000000,4.07140634621952,3.17624000000000,4.01563365654528,3.23481600110592,3.56945213915136,3.62522482882560,4.74067862231040,4.03404000000000,2.13200000000000,3.25778000000000,0;'Liver_ALCD2if',4.49130592214195,6.70573663189249,30.4727404152378,4.09612507291346,14.8829236294566,18.9989939073780,1.72282393109743,2.09190845358929,8.52342571208486,24.7129546728825,11.5617148236181,27.1992491620662,26.0534890914753,26.5000396720388,4.96142086300612,40.1756279223163,2.46225701335280,10.7350231441421,14.4978547229352,40.1891439860550,40.1891439860550,16.0490941305686,4.13517714184556,13.5152987166802,2.96855690508786,15.1995527379715,10.4176189063599,23.2960800732726,7.57717537297114,12.4847232994650,7.19513709889368,17.6596921028581,8.28685445393482,4.35045832971282,2.17752017660310,1.81125051866318,3.54193537313985,10.7481019486839,3.42296920431327,9.09108537124100,8.41575390823209,10.0184725489776,11.6948665049846,6.46305548109904,16.9778004428144,12.5445588995595,20.6377152160043,8.91756653874603,15.4156258331036,9.48805201080842,6.31636189402159,17.8796333795397,8.70463597677019,5.26920896212692,7.50025017380637,4.84155522288815,16.9930074936871,26.5000396720388,27.4527707920722,3.17257259182453,18.6751098078882,3.39851221590324,5.93639101048742,9.94124420836590,9.17336531097383,25.8299109242020,10.0079491898649,2.78116451705789,5.64607428097396,3.81788117902199,1.80880789815816,24.7424164311299,9.94985629762418,2.22281984572613,22.3798142325645,22.2630093566117,23.4373219380593,7.36303331160576,26.2871267225238,9.63594373175196,14.3149083620646,16.3788370925777,20.1095385217253,3.63615463544652,20.5307312389571,4.56403868362695,4.74297389450295,2.07428266953010,3.45751566997609,13.0548386602768,2.42565694922942,11.0326012630194,6.03768546904122,9.99814636043435,8.64399325546396,2.41124550246848,7.57083464067526,6.56831957121285,1.75141690917858,6.22983968026887,11.1146001712935,13.6077737070825,3.37643628127847,7.95624493679047,8.40442996848297,17.6481403804645,8.32032734103526,20.2930189948944,12.4465372169225,6.20104550840377,8.62622642247637,15.4561978748629,16.9662782951962,6.80497201780857,15.3723214660090,4.47995802398110,8.85095227218282,14.3154144746257,2.41071066825056,6.50220786255027,24.7424164311299,7.67295107892168,11.3057253083082,3.36724641750349,5.65178363447290,26.9632907252010,5.57946356907124,4.71191113831062,3.77290654805453,3.45203054446110,14.8473092015949,3.49004515018143,9.29716510714006,14.8314954412691,6.99327937544967,3.39889519502486,30.0697352606097,3.27203654241037,23.4373219380593,13.8161966751792,11.5866056938880,11.7410920920731,5.04368573097846,4.54974951718699,3.59044980612039,8.33871360916053,5.45004534762149;'Colon_HMR_0156',33.3343671619026,57.1830277806540,235.089230703793,22.3414075799136,96.9106654940477,93.7416149797852,1.85472889850761,9.66826127356424,42.1571680079888,224.481134893216,93.9281132512757,224.463236784488,213.221494652892,233.145783070221,31.1556320941105,236.462754743129,7.18438953729949,49.9102882001167,134.996647093676,245.530615264473,260.530335259203,55.3499439186155,26.7257735076059,70.9494394540617,14.2162544299025,68.1114092816234,79.4175251491045,124.212191405933,45.2705233141498,108.132231579656,51.3844103072022,158.992417954900,11.4612616811236,23.7366390502626,9.71909815224205,3.07769109124557,20.7992331593366,78.4014756978871,13.1559232450459,35.1488529227674,66.3405322931266,69.4705914606715,93.7274284066749,53.0072071514517,102.910418424557,81.5111074554793,146.228562560068,51.7942540010564,118.547624710459,75.3719143540268,23.0608766629638,143.983585706208,61.9779681328600,18.3605236592463,12.4886586611114,23.0174427424716,134.175317342056,233.040635354440,186.087994047878,20.7826875368177,136.683710543300,5.34262549217676,29.0094373375415,79.7228066815618,83.5944786475935,153.178491726405,76.4714644230326,11.8429977502465,33.9981779862785,20.8143003526723,3.74944285965715,156.145947946329,50.6521725381341,10.9360871366472,199.230600212219,162.771480057973,175.798121986508,67.2147180095363,156.797481505356,113.963600013100,113.102345301367,121.671839880314,140.733298056453,16.3032711117719,151.892587401361,35.5879679094798,24.6184954788350,9.26846458197039,11.2711969084679,113.110659580741,12.0612363025003,120.030459152878,29.4023258844412,43.8657934396799,36.4972492397437,11.5243019600015,54.4738589374853,43.9347922807090,4.45005880611572,27.5133160050510,87.6992163065903,92.1261755205012,31.2298061264885,64.5976616262538,77.0291834910107,143.409786082419,57.0923581655509,150.903976555088,86.6116221515309,27.2605440019020,54.1840481825032,89.5689106943506,101.600190371609,51.4931002335175,124.972693853338,7.48325690869873,62.5639551038227,98.5028936434513,7.23199672919750,40.9728620124868,156.208818598646,49.4105275262722,89.1681401153790,12.3074358998944,47.9744081347880,204.796859350298,34.5274170620750,34.4935088070435,22.8840721012859,18.1649938561928,165.535673976973,18.7974766764301,98.7271493777294,165.682773713132,46.1950653794541,16.0604410483487,183.425670538847,12.5211201814110,176.894571934782,120.277400263545,81.3046075001409,70.5811746880081,37.4150822394900,36.0442677368131,22.4589408015976,42.0586613345485,32.0296325192198;'Brain_DM_dopa[c]',7.59287834675601,7.61022172073524,7.86188972695927,7.77318176378420,5.43970205749773,8.13731501848345,7.65459646855330,6.74556205157672,7.70817529106449,7.11840920517429,5.92773367533089,7.59903877991365,7.11840920517426,7.75533533758696,7.60242159335553,7.73555122908044,6.74688396916200,7.13570707342333,7.21546526935760,8.23686298350763,8.24105684387549,7.72502576996030,6.03857651667414,8.46488769463467,7.30227149366460,6.73439729751351,7.29844518644328,6.11816027803932,6.32875162529247,8.02256141149320,5.22950637019717,5.83587128586362,8.01809060152326,7.28428369998612,6.22405635291876,7.74189463535971,7.65594531893250,7.81445816302733,5.62868062809201,7.75309955804274,7.50006913655789,7.99262599569661,7.86016536253748,6.74556205157706,6.90674701562913,7.73555122908111,5.44664970104298,8.14792738471460,6.43301167767380,6.82081811122763,7.36277078101336,7.11902923702556,4.94351236896975,8.08858231009140,7.48819875224008,7.37758322681167,5.83587128587374,7.75005343189656,5.92773367533398,6.74688396916268,7.60021863169596,7.67915280119119,8.15471125295106,6.74556205157638,6.27497312146640,5.86013420459473,5.99000599141900,5.68950795457328,6.06986751907902,6.26543634021641,6.10650484582413,5.96369816858760,7.15905723530318,6.96519518665598,6.59434552830675,6.41062186521777,6.15153482037256,7.29223258908153,5.96472173059335,5.87121889411161,5.77606250510709,6.24185640069838,7.00365843307274,6.88821751598885,6.17631201888398,6.84706175974014,7.03455575187989,6.13398231312534,6.42883289047519,6.48608967570998,7.45408359754920,6.06288337133337,5.94602854496153,5.96684917816002,7.51978293945083,6.80293119219494,6.05988592248559,6.02464312393598,6.26149344401070,7.30949148737080,5.34441076154572,5.78220558634813,6.99054962778965,6.79307293390810,7.05714600188035,6.59677637531659,6.53982725582672,5.43509400857548,6.50709955984879,6.57117113020697,6.88063107514646,5.96241815383891,6.27225695234885,6.27497312147062,5.86013420459506,6.04252787635902,6.05662427894964,7.08786021048593,6.22265913092661,7.00046091915189,5.97575847738644,5.96684917816019,7.24219441962713,6.41184136878696,6.06934763033725,7.04329892696466,7.02746692726371,6.29011721943930,6.29011721943930,6.80941092938328,6.22439394789946,6.58463059593263,6.38158130316129,6.25342270342995,6.27357886234738,5.57974823959900,6.98473417323672,5.90069491385668,6.24206131144285,5.96472173059335,6.17991176118784,5.94880512242438,6.98508354840367,7.25360580390034,6.62459360487461,6.15168839706765,5.39693571252046;'Brain_DM_srtn[c]',38.1416966238770,38.7969158989638,39.4786665395301,38.7756326855234,28.7447843294913,40.0835280665749,36.6115390749665,34.4926242677541,38.6182541653400,38.6586835726139,32.9605301217175,38.5725161509505,38.6491649591568,38.6639358637825,36.3808251421130,39.7356435946735,34.4937343874268,39.3788211325888,38.6013883099104,40.6710509114311,40.7003594582693,40.0781094108051,31.3005474672859,40.7449193085403,36.5368543160637,39.2057348751180,37.9915150972397,35.3308109274321,36.0226115092924,39.2801922044938,26.8114475785830,31.9519673125214,39.6264113056302,34.8527449984527,32.1900818714311,37.2217100194121,37.9541447726195,39.1469480015387,28.2714733184968,35.8791263282157,36.0694496360979,39.1148272183732,38.9803631203188,36.6668086770166,38.5911305611680,39.7503313328250,28.5050149755374,40.0495297038043,35.6313803956870,35.5492096983215,36.8141398873970,38.0121452143369,24.1520658625213,40.1259975364073,36.2845271924290,36.3055405813981,32.1442434566500,38.6269166948887,33.5552921529453,34.7496255977960,38.5822596487342,36.7831775336395,40.0969383654415,38.5842612683824,34.0000590698052,32.2470951439510,30.8692245018806,28.2602058425232,31.7646666008328,32.3575853054184,27.3658220903976,30.7036924063158,36.5152126505870,34.4559838827719,30.2628532730874,29.8998413420496,30.2018043483362,37.1991729958909,33.0834814729054,27.6283523323344,28.5132778101010,31.9667030469253,35.7855403873737,33.9836552576412,31.5404236317896,33.0421192124098,33.8834327330952,28.6647663475119,31.4237073830545,33.5313509202624,35.3810868072288,30.9259384137987,29.9170643638888,34.0597932835381,35.3831315688786,35.0004254648583,30.5012527474262,28.0705146235960,31.5335663829671,36.0125362132798,31.1144165795988,28.1568632993842,35.2914433719731,32.5125432230269,35.7344223607449,30.2796705162772,32.2669015155424,26.8700573591252,30.5800796891764,32.3349379051018,33.9392611012075,32.0730653416604,33.0520527639108,34.0034555360703,30.6882417481437,27.1927023908175,31.3027821017105,36.0522574591268,32.0793998792711,36.4957904426265,30.7837247078828,34.0669445020963,36.2521116210678,28.8878026702603,30.9679970291427,33.9216763482724,32.1234119245991,37.0922090613921,36.9117377600158,33.4472251341079,31.8291563673356,33.0664879822329,35.6435651887137,32.0179733180012,33.4002249387613,27.3955966029272,35.5359254467068,27.8373308225517,30.8498598470088,31.8200382914302,31.5635634958587,29.9178701203027,33.9388559307445,36.9378879326261,33.8805026213538,30.2026443116389,26.0794594779036;'Brain_DM_adrnl[c]',5.69587034436024,5.73193665052741,5.91007533974179,5.83674191327156,4.18547224686363,6.11688764393699,5.66585228312203,5.17719480168774,5.79388652193633,5.60967774529408,4.75338058911670,5.70178880538735,5.60877056352833,5.82234928241548,5.61892287585491,5.88051019208620,5.17814500409900,5.70713284082483,5.62357910828061,6.19269102064326,6.19584396967999,5.90765190857641,4.63461731419318,6.31219620565373,5.48947475691822,5.63401549504938,5.56019789180563,5.01292034923419,5.20285733667819,6.02934153763801,3.85341721570705,4.65990479474797,6.02751958714291,5.36424596685200,4.74398894982768,5.75130695765993,5.74954382026666,5.87445799488064,4.10374073845869,5.60320201348694,5.55942125688299,5.99992723432134,5.90598127991202,5.41533149940608,5.53988350972035,5.88209053388872,4.20671847935073,6.12582913149088,5.19920933295440,5.21320239156824,5.53258717179846,5.54004064782558,3.56879120701199,6.08018887538973,5.56261363350008,5.54630574848084,4.68056399323044,5.81852806415185,4.81573938550911,5.20617601372589,5.70275503819729,5.68989031872031,6.13092925493384,5.62442208837624,5.22195377043880,4.92486269980311,4.80049512608219,4.54009383890579,4.90786939952960,5.03230683196382,4.57965635377462,4.77088861839877,5.75399063197190,5.59527547698786,4.99896887520393,4.91182704389969,4.88778645890706,5.86102505683268,5.05343709223680,4.45877609604815,4.53814040564984,5.00341872068910,5.62909114537157,5.52094526078932,4.94852693429408,5.40152132050211,5.52856005418022,4.70968691502420,5.10550446524080,5.21032655708989,5.84460439814196,4.86398560577576,4.76282051889873,5.17958187824900,5.82294929887432,5.46775693581046,4.85767742261949,4.61331664885234,5.02395183759547,5.85624806834208,4.69119214460398,4.51978094896001,5.61855952129912,5.31356865051006,5.67208366772853,5.00146477520135,5.23618101184213,4.21646400860028,5.01279777030947,5.23857622711614,5.50652517606177,4.91818284463602,5.09751807783127,5.22237269352910,4.73138157628823,4.53137228511640,4.85181032987963,5.69676888947328,4.99692702759571,5.65934280185911,4.78146405800449,5.18046290654514,5.82082057412960,4.80687914330398,4.86889285803799,5.53748777976773,5.33356994512952,5.66464397474419,5.64183929074809,5.44314196185335,4.99915669050497,5.29231386359237,5.44588646420721,5.02398064942039,5.14202228197351,4.35909130819583,5.61388350263501,4.49057860777397,4.98241843846285,4.89886319277760,4.95157078673135,4.76525852717288,5.53973863136909,5.82998033492411,5.32442515081928,4.88790158066722,4.14943946088433;'Brain_DM_4abut[c]',1.29993088134610,1.22091302852764,1.08111994606998,1.23464560990853,1.48202788941865,1.16953227226940,1.05318863939131,1.08439933016393,1.10171250484353,1.21250493143716,1.51305664356201,1.24565595145285,1.21227781310856,1.25502669481212,1.15414418484022,1.05623259164703,1.07765988737576,1.10085397360789,1.21567138647466,1.06262782150974,1.06292193130629,1.09775366019356,1.24216320399724,1.05914849868698,1.06613050028877,1.17725359087481,1.44359774888322,1.63036016228527,1.31411938930240,1.16168644150515,2.13977533020646,1.39081011010037,1.13868634647310,1.28479703320373,1.36092851922113,1.05705319102636,1.21914231752328,1.08172249105570,1.63081041952104,1.17316573692303,1.15749701077848,1.20008505348272,1.20650354328699,1.09317697862523,1.40926615859703,1.05653600896792,1.30935111247214,1.05796696044936,1.22539294609307,1.19774468219057,1.11871066848316,1.19856890679013,2.05098934764526,1.14286767246885,1.31907077763648,1.08615765991566,1.39539655857676,1.25415808439454,1.52849999470386,1.07781548726071,1.24593214756658,1.05282762005026,1.05821652745707,1.10199907751715,1.08659367795950,1.09393723590323,1.09867886248436,1.08806538774286,1.07078955922026,1.07710057974500,1.14676304738220,1.11037277668609,1.09044525617201,1.07849968601728,1.12706845135545,1.14028235528779,1.13086640885362,1.08418881880227,1.08936569357333,1.19464375690319,1.16090984358608,1.10771854386546,1.09104672135867,1.09200355612231,1.10496467490236,1.07852948702440,1.09406871028796,1.09477682069738,1.10197492604291,1.09174974177716,1.07079241388758,1.08874844861952,1.09325579207114,1.08391954395568,1.09711657829582,1.07720105026954,1.08756575576627,1.13814650804291,1.07420355876681,1.08491732783702,1.09448353072389,1.13864183300535,1.07577060930581,1.10326672433105,1.07770166584576,1.12739242975122,1.08141563088793,1.23033676575672,1.14426526097663,1.10613938127150,1.09581925092769,1.09619870043947,1.09532282519991,1.08665189498777,1.08452478323746,1.11973533209970,1.10228333631397,1.08829171539263,1.07540414386935,1.08539492141584,1.11085608681810,1.08404383226357,1.08591773398763,1.16756843378310,1.08925477526619,1.09452125018864,1.07635012370652,1.05903745999209,1.05824141631673,1.07528958977280,1.08224590888700,1.07783524265683,1.08239417288259,1.08328652363033,1.08985931855201,1.13730652912220,1.08802301187815,1.19758765397760,1.13562135167105,1.08199628122057,1.10483545558465,1.09320636415735,1.08217253936293,1.08251384289353,1.08661405825666,1.13087273383102,1.12199861844625;'Brain_DM_hista[c]',1.84428309817850,1.79801861638269,1.89223942074575,1.91463637836250,1.28448295900334,2.05523307936222,2.05607300173312,1.58900935676447,1.86874267351869,1.68137521531878,1.40095260221749,1.79785644818514,1.68137521531863,1.91452418008970,2.05498628131203,1.82259050403741,1.58907009472442,1.68217287234423,1.70464354693333,2.05616018223054,2.05616018223054,1.82210608258922,1.42485783486078,2.22009720172664,1.77556932754333,1.58849560561315,1.72725995784325,1.44731859485670,1.49461770136214,2.05523307936212,1.23720198314012,1.37792413712805,2.00867345075258,1.98410820726947,1.47099618083782,2.05607300173323,1.91463637836274,1.89223942074575,1.33078258654520,2.24143331527307,2.00820370824650,2.05498628131203,1.96155658155654,1.58900935676425,1.63402658235578,1.82259050403810,1.28480343894070,2.05616018223054,1.51821873411737,1.61127333803377,1.79849991255936,1.68140386060240,1.23720198313985,2.00867345075391,1.98410820726940,1.86874267351869,1.37792413713026,1.91452418008970,1.40095260780975,1.58907009472442,1.79785644818514,2.05607300173323,2.05616018223054,1.58900935676442,1.53660333633086,1.43568272985776,1.51112169918880,1.61181202932080,1.48625392813705,1.56180715997488,2.19742839865672,1.51105297416863,1.86423643539509,2.01538749335155,2.31661685434186,2.16552226085430,1.81312321711712,1.86441723470735,1.46095498685088,1.91348742592477,1.66186371508609,1.61186703622298,1.81379518103337,2.01510264376479,1.61186422312654,2.14114050341345,2.21647419220279,2.06508012126649,1.93928780660446,1.61209920583952,2.39349510867695,1.58679243535473,1.63706063202312,1.46105690830828,2.51873217342725,1.71319057727815,1.66234628095136,2.03959852143063,1.71293931836695,2.14127850100882,1.30966699964800,1.73745497618889,1.86442240435844,2.16591472619801,1.86441723470747,2.31661685434207,1.91441090610973,1.51067087801905,2.16552226085400,1.93928780660441,2.01504871389532,1.46084456057170,1.53647318579893,1.53660333633053,1.43568272985732,2.22687081438167,1.51112169918880,1.86423643539509,1.56180715997488,1.71319057727815,1.51105297416885,1.46105690830839,2.01538749335202,2.36671176788663,1.58679243535473,2.21647419220253,2.51873217342735,1.53732734300543,1.53732734300543,2.01536759709069,1.61209920583951,1.81379518103335,1.56197218803005,1.61209920583946,1.53653653897817,1.63671134062857,1.83899954122168,1.91348742592477,1.81312321711912,1.46095498685088,1.61186703622298,1.63706063202476,2.14114050341486,1.86442240435493,1.71293931976533,1.81312321711701,1.63671134062867;'Brain_DM_kynate[c]',223.299078090903,223.189721560926,206.555245991028,224.177084246570,203.974846140545,227.229173904475,214.128509874701,188.318615983453,157.670461504839,221.379575292079,219.139866154805,222.962795012337,221.312167926636,197.393370845131,217.411171496947,177.611907753052,208.093226214754,219.513945212661,221.713635712470,225.116079705911,222.391632069325,216.231410148525,212.237855353573,224.877249334590,212.665674023222,221.474157247651,223.733516338500,222.520965177020,209.297924238430,224.211721061426,206.910379324001,209.721170371636,224.652483548929,216.281670755508,211.441621631437,216.184533588778,220.711395986664,221.129105908762,212.502958397418,218.563748692441,216.605840007787,224.716993681582,223.916498649717,213.731015583519,219.953249397997,220.272350370227,216.147187485424,222.882368265128,216.499303294453,212.912791946729,215.535714221959,219.736441705242,199.980739600139,226.436692966666,220.159470827673,137.054180153048,218.743570791446,201.032932984816,221.236367119035,208.583448742555,223.026127815620,214.440145451035,223.034032304072,152.219443475669,211.170068804108,220.806903805075,218.813024751929,212.840022423571,197.778298822344,216.715751675466,224.212452074679,221.295415392172,226.813282733930,219.942219870723,180.658801463704,226.575191996614,223.766351832338,224.835398954883,207.638577535395,223.612074875637,215.456985391643,201.271613107300,225.705917228305,225.165612444064,222.259419494135,219.743574271159,226.688786601528,209.941373840821,222.442611355393,222.488343299798,219.366415870969,217.226002806807,216.620113573551,221.467575025572,232.171239636706,218.943488876107,159.725043052519,221.134521199878,214.055202077040,226.718008506616,219.275925426605,220.681958198568,218.690661892204,226.377938613246,220.136842260518,227.612532393867,217.685682320378,219.296487266648,229.192524742525,224.779026634233,225.419048917670,212.098013212124,205.886392646293,221.986279675408,209.667945877472,219.298586422885,221.322574112962,222.047063695479,215.644549213808,223.670963718317,208.171719533268,221.506773058667,225.379880667167,228.899208663873,217.431207941835,213.537845915636,221.568256478602,213.534756852546,212.928740005105,217.028961879141,217.132261185471,107.111289439528,222.227156331160,217.816658205780,87.7329187743446,192.869723384230,224.119321350145,224.395235945038,226.331555394516,217.155564964139,194.757336407137,216.660074874206,222.725486557704,223.775084550057,221.652299926267,223.770874682391,188.427336698649;'Brain_DM_nrpphr[c]',5.79721294637122,5.83359559221434,6.00814313810317,5.93912724552938,4.32630203705875,6.21863181865494,5.78799518724985,5.28109277079887,5.89067453531739,5.70952766859441,4.90828250131141,5.80309507339786,5.70859124630181,5.92505896074144,5.74086370223971,5.97738352052270,5.28709776004883,5.80546744137924,5.72367976232678,6.29470540263888,6.29791030432200,6.00815294055223,4.75540980462725,6.44428406990135,5.58048080530809,5.74146238800095,5.65970296559475,5.17628048981561,5.33846124639227,6.13093808262236,3.98144841321981,4.78135704919992,6.12751827996774,5.48803931628298,4.88824271618309,5.87529244356583,5.84989730994319,5.97189646015664,4.24008808518397,5.72399191707130,5.68118884099007,6.10806165252058,6.00683004068620,5.52122520885688,5.63889681204249,5.97898991016022,4.32005807361268,6.22674179957378,5.33471784509240,5.30603719653142,5.62671515205609,5.63854202749287,3.70210186369807,6.18138725883870,5.69155976078127,5.63803973339025,4.80255476744125,5.92108595198712,4.97208363520682,5.31559115694992,5.80408763745575,5.81255152283294,6.23192595861771,5.73374697800825,5.32283767416556,5.02917610422583,4.90739395800576,4.66122061469452,5.00744425630534,5.13304095449020,4.75934323156596,4.88584151686875,5.86515105639804,5.70634180707539,5.17401319482466,5.08297557186842,5.03966320892314,5.97425363868682,5.15498473728081,4.69537947647729,4.73248671254891,5.11372658543950,5.73783875836498,5.64327851314511,5.06003002700588,5.53235342940369,5.67417533456556,4.86722495431382,5.23893576634174,5.31381225698082,5.98323375274256,4.96710315205320,4.87137441244133,5.27964796300575,5.97381356351709,5.57338832306586,4.96465262269826,4.77426966919163,5.12982098591319,5.98841142528221,4.79995157132304,4.71748058959098,5.72710303489264,5.46419756272289,5.78166140947868,5.17644474209006,5.35785665136684,4.39549598968199,5.17945391107925,5.38127400288733,5.63706391603725,5.02386652000374,5.20149260333638,5.32326470011976,4.83299651969006,4.70932825409176,4.96196978144951,5.80682361781726,5.09799617263576,5.76867613932696,4.89572179754613,5.28054599875299,5.93327052457654,4.99658804282240,4.97239892927994,5.68291850748124,5.48149755132618,5.79942573923893,5.77633424311084,5.57150163705224,5.09942076420106,5.39455457762605,5.55109704715824,5.12320226905995,5.24427912657101,4.57164460064480,5.72233696480837,4.72845105359279,5.11390795663177,4.99585911200532,5.06297908271595,4.87364908962099,5.67037523292080,5.94260905838513,5.42728700124239,5.03981678563343,4.36962967858627;'Brain_DM_Lkynr[c]',97.5251131775468,97.4714358417294,96.6019773115984,97.7132594584243,85.7551532075868,98.7042566286881,93.3141135138226,91.6006675726017,95.9939766946745,96.9517135529734,94.8541633423572,97.4213151776810,96.9144620430729,97.7733613541136,94.7543543971116,95.9884958089317,91.6518493108964,96.0279474443344,97.0958257159153,97.6781136592541,97.7320633936321,97.7562148161030,90.6753751847178,97.1863918466017,93.1984195958790,97.1292007727508,97.9894794230655,98.0403254926582,96.4666077271572,97.3578858748005,82.5406237753603,92.7166494168896,97.6542355396236,94.4897694806816,92.3647919280357,94.1973739051126,96.7629333196770,96.3792468084960,84.2809452145741,94.9682828528891,94.5358146868707,97.6094638146075,97.5008723292286,93.8511948411805,98.6175436003984,96.0324355065972,85.0748452677472,96.6983211997181,95.2353822840859,93.7683828147029,94.3759157507587,96.3506698763281,76.0463704547800,98.4387070947756,96.0115390347660,93.5700678580372,93.2113183251240,97.6908360236330,96.2906725159785,91.8064801025530,97.4538553047738,93.4092689452675,96.7612308507576,96.0108992826509,86.4408820659602,84.0063024822094,81.3118569379765,74.6488061714642,81.5239827507720,82.9775652200660,72.6646382022280,80.7909307496962,90.8307268112626,86.4144566202707,79.7084113098557,78.7422197181425,79.6327551258491,91.4361528899427,85.1340450349939,73.0692671295292,75.3851890330237,84.0519581766827,89.6006042816670,87.1284390553454,82.9367714186952,84.3935518393576,87.1175442071491,75.6015691497327,82.6914521268688,85.9560973454093,87.4700902929214,81.2540499342497,78.7533363003992,86.4338633659533,90.3503697448015,87.0292792821071,80.2399699786394,74.3694613286624,81.0926796282613,90.0644389451467,81.9324633794078,74.6106424819495,87.3453164814489,85.1732336097262,88.3515797852037,79.7530533627599,82.9072530014451,71.7623764996681,80.4699950181235,84.6689510456185,86.9649864470743,83.7284326390251,85.4585736541048,86.4499670714854,80.6955381958307,72.1033047762063,82.3346672621145,89.7112228777299,82.3507063402103,90.1453501176561,81.0076603633962,86.4536173301938,90.0221036275056,76.3185178864978,81.3563522883313,87.2118866952945,83.5393512424873,89.3690287899776,88.9619295228022,84.2415726331032,82.4910860013249,83.7997173042492,88.6652294476460,82.9154880519629,85.6523120095246,72.7127901469676,88.8422650166824,73.5975608073654,81.1921589919053,82.6174557632699,82.9425424672297,78.8023059842121,86.3465965439465,90.8431203135145,86.1927398334509,79.6349838440331,69.2274564609626};
0046 
0047 %% calculate the Spearman correlation between fold changes species abundances
0048 % get HMP abundances
0049 abundancePath=[CBTDIR filesep 'papers' filesep '2018_microbiomeModelingToolbox' filesep 'examples' filesep 'normCoverage.csv'];
0050 
0051 [FluxCorrelations, PValues, Abundances] = correlateFluxWithTaxonAbundance(abundancePath, FoldChanges, {}, 'Spearman');
0052 close all
0053 % replace IDs with descriptions
0054 replace={
0055     'Liver_PCSF'    'Liver Sulfotransferase'
0056     'Liver_ALCD2if' 'Liver Alcohol dehydrogenase'
0057     'Colon_HMR_0156'    'Colon Carboxylic acid:CoA ligase'
0058     'Brain_DM_dopa[c]'  'Brain dopamine production'
0059     'Brain_DM_srtn[c]'  'Brain serotonin production'
0060     'Brain_DM_adrnl[c]' 'Brain adrenaline production'
0061     'Brain_DM_4abut[c]' 'Brain GABA production'
0062     'Brain_DM_hista[c]' 'Brain histamine production'
0063     'Brain_DM_kynate[c]'    'Brain kynurenic acid production'
0064     'Brain_DM_nrpphr[c]'    'Brain norepinephrine production'
0065     'Brain_DM_Lkynr[c]' 'Brain L-kynurenine production'
0066     };
0067 % Export the species correlations
0068 for i=1:length(replace)
0069     FluxCorrelations.(taxa{i})(1,2:end)=strrep(FluxCorrelations.('Species')(1,2:end),replace{i,1},replace{i,2});
0070     PValues.(taxa{i})(1,2:end)=strrep(PValues.('Species')(1,2:end),replace{i,1},replace{i,2});
0071 end
0072 cell2csv('SpeciesCorrelations.csv',FluxCorrelations.('Species'));
0073 cell2csv('SpeciesPValues.csv',PValues.('Species'));
0074 cell2csv('SpeciesAbundances.csv',Abundances.('Species'));
0075 
0076 % Export summary table with the calculated values
0077 for i=2:size(FluxCorrelations.('Species'),1)
0078     Table{i,1}=FluxCorrelations.('Species'){i,1};
0079 end
0080 cnt=2;
0081 for i=2:size(FluxCorrelations.('Species'),2)
0082     Table{1,cnt}=strcat('CorrelationCoefficient_',FluxCorrelations.('Species'){1,i});
0083     for j=2:size(FluxCorrelations.('Species'),1)
0084         Table{j,cnt}=FluxCorrelations.('Species'){j,i};
0085     end
0086     cnt=cnt+1;
0087     Table{1,cnt}=strcat('pValue_',PValues.('Species'){1,i});
0088     for j=2:size(PValues.('Species'),1)
0089         Table{j,cnt}=PValues.('Species'){j,i};
0090     end
0091     cnt=cnt+1;
0092 end
0093 cell2csv('Table.csv',Table);
```

---

Generated on Thu 14-May-2020 13:05:49 by **m2html** © 2005
