# Supplementary material for: Personalized whole‐body models integrate metabolism, physiology, and the gut microbiome
Source: Mol Syst Biol. 2020 May 28;16(5):e8982. doi: 10.15252/msb.20198982 (PMC7285886; doi:10.15252/msb.20198982)
Supplement: Supplementary file 22 — Dataset EV1 [file MSB-16-e8982-s022.zip › PSCM_toolbox/PSCM_toolbox_doc/src/scripts/annotateOrganAtlas.html]

Description of annotateOrganAtlas


# annotateOrganAtlas

## PURPOSE

**annotate OrganAtlas**

## SYNOPSIS

**This is a script file.**

## DESCRIPTION

```
 annotate OrganAtlas
```

## CROSS-REFERENCE INFORMATION

This function calls:

- annotateModel This function annotates a model with VMH reaction and metabolite

This function is called by:

## SOURCE CODE

```
0001 % annotate OrganAtlas
0002 load('OrganAtlas_Harvetta_2.mat')
0003 
0004 annotateRxns = 1;
0005 annotateMets = 1;
0006  
0007 O = fieldnames(OrganCompendium_female);
0008 for i = 1 : length(O)
0009     if ~(strcmp(O{i},'sex')) && ~(strcmp(O{i},'Recon3DHarvey'))
0010         O{i}
0011         modelID = strcat(O{i},'_female');
0012         modelName = strcat(O{i},'extracted from the female whole body metabolic reconstruction, Harvetta.');
0013           modelAnnotation = {strcat('This is a metabolic reconstruction of: ', modelID);...
0014     'Authors: Ines Thiele, NUI Galway, Ireland';...
0015     'Please cite when using one or more reconstructions from the Organ compendium: Thiele et al.,Molecular Systems Biology, 2020.';...
0016     'This reconstruction has been extensively curated against experimental data from literature.';...
0017     'Please contact: ines(dot)thiele(at)nuigalway.ie';...
0018     'This work is licensed under a <a href="https://creativecommons.org/licenses/by-nc-nd/4.0/" target="_blank">Creative Commons Attribution-NonCommercial-NoDerivatives 4.0 International License</a>.'};
0019              tic;OrganCompendium_female.(O{i}).modelAllComp = annotateModel(OrganCompendium_female.(O{i}).modelAllComp, annotateRxns,annotateMets,modelID,modelName,modelAnnotation);toc;
0020                      fileNameOut = strcat(O{i},'_female.xml');
0021       %  writeCbModel(OrganCompendium_female.(O{i}).modelAllComp, 'format', 'sbml', 'fileName', fileNameOut);
0022 
0023     end
0024 end
0025 clearvars -except OrganCompendium_female
0026 save OrganAtlas_Harvetta OrganCompendium_female 
0027 clear
0028 
0029 load('OrganAtlas_Harvey_2.mat')
0030 
0031 annotateRxns = 1;
0032 annotateMets = 1;
0033 O = fieldnames(OrganCompendium_male);
0034 for i = 1 : length(O)
0035     if ~(strcmp(O{i},'sex')) && ~(strcmp(O{i},'Recon3DHarvey'))
0036         O{i}
0037             modelID = strcat(O{i},'_male');
0038         modelName = strcat(O{i},'extracted from the male whole body metabolic reconstruction, Harvey.');
0039   modelAnnotation = {strcat('This is a metabolic reconstruction of: ', modelID);...
0040     'Authors: Ines Thiele, NUI Galway, Ireland';...
0041     'Please cite when using one or more reconstructions from the Organ compendium: Thiele et al.,Molecular Systems Biology, 2020.';...
0042     'This reconstruction has been extensively curated against experimental data from literature.';...
0043     'Please contact: ines(dot)thiele(at)nuigalway.ie';...
0044     'This work is licensed under a <a href="https://creativecommons.org/licenses/by-nc-nd/4.0/" target="_blank">Creative Commons Attribution-NonCommercial-NoDerivatives 4.0 International License</a>.'};
0045         tic;OrganCompendium_male.(O{i}).modelAllComp = annotateModel(OrganCompendium_male.(O{i}).modelAllComp, annotateRxns,annotateMets,modelID,modelName,modelAnnotation);toc;
0046         fileNameOut = strcat(O{i},'_male.xml');
0047       %  writeCbModel(OrganCompendium_male.(O{i}).modelAllComp, 'format', 'sbml', 'fileName', fileNameOut);
0048 
0049     end
0050 end
0051 clearvars -except OrganCompendium_male
0052 save OrganAtlas_Harvey OrganCompendium_male
```

---

Generated on Thu 14-May-2020 13:05:49 by **m2html** © 2005
