# Supplementary material for: Personalized whole‐body models integrate metabolism, physiology, and the gut microbiome
Source: Mol Syst Biol. 2020 May 28;16(5):e8982. doi: 10.15252/msb.20198982 (PMC7285886; doi:10.15252/msb.20198982)
Supplement: Supplementary file 22 — Dataset EV1 [file MSB-16-e8982-s022.zip › PSCM_toolbox/PSCM_toolbox_doc/src/scripts/menu.html]

Index for Directory src/scripts


 Master index 

# Index for src/scripts

## Matlab files in this directory:

- LeakTestRecon
- annotateHH
- annotateOrganAtlas
- runIEM\_HH

## Subsequent directories:

- BMRcalculations

---

Generated by **m2html** © 2005
