# Supplementary material for: Personalized whole‐body models integrate metabolism, physiology, and the gut microbiome
Source: Mol Syst Biol. 2020 May 28;16(5):e8982. doi: 10.15252/msb.20198982 (PMC7285886; doi:10.15252/msb.20198982)
Supplement: Supplementary file 22 — Dataset EV1 [file MSB-16-e8982-s022.zip › PSCM_toolbox/PSCM_toolbox_doc/src/scripts/runIEM_HH.html]

Description of runIEM\_HH


# runIEM\_HH

## PURPOSE

**This script predicts known biomarker metabolites in**

## SYNOPSIS

**This is a script file.**

## DESCRIPTION

```
 This script predicts known biomarker metabolites in
 different biofluid compartments (urine, blood, csf) of the whole-body
 model for 57 inborn-errors of metabolism (IEMs).
 The meaning of the abbreviations for metabolites and IEMs used in this
 script can be found at www.vmh.life.
 The supported model options are 'male', 'female', and 'Recon3D'. Please
 define those using the variable 'sex' (e.g., sex = 'male').

 Ines Thiele 2018 - 2019
```

## CROSS-REFERENCE INFORMATION

This function calls:

- checkIEM\_WBM This function performs the inborn error of metabolism simulations by
- createModelNewCompartment This function converts a two compartment metabolic model into a three compartment metabolic model model
- combineHarveyMicrotiota This function combines harvey and a microbial community model
- loadPSCMfile Loads a mat file into the workspace, given a nickname or a full filename
- EUAverageDietNew Average European Diet defintion. For details, please see https://www.vmh.life/#nutrition
- physiologicalConstraintsHMDBbased This function applies constraints to the whole-body metabolic model
- setDietConstraints This function sets diet constraints onto the bounds of the diet uptale
- standardPhysiolDefaultParameters This script creates the IndividualParameters structure which contains

This function is called by:

## SOURCE CODE

```
0001 % This script predicts known biomarker metabolites in
0002 % different biofluid compartments (urine, blood, csf) of the whole-body
0003 % model for 57 inborn-errors of metabolism (IEMs).
0004 % The meaning of the abbreviations for metabolites and IEMs used in this
0005 % script can be found at www.vmh.life.
0006 % The supported model options are 'male', 'female', and 'Recon3D'. Please
0007 % define those using the variable 'sex' (e.g., sex = 'male').
0008 %
0009 % Ines Thiele 2018 - 2019
0010 
0011 if ~exist('useSolveCobraLPCPLEX','var')
0012     global useSolveCobraLPCPLEX
0013     useSolveCobraLPCPLEX = 0;
0014 end
0015 
0016 if ~exist('resultsPath','var')
0017     global resultsPath
0018     resultsPath = which('MethodSection3.mlx');
0019     resultsPath = strrep(resultsPath,'MethodSection3.mlx',['Results' filesep]);
0020 end
0021 
0022 if strcmp(modelName,'Harvey')
0023     %load file corresponding to fileName
0024     male = loadPSCMfile(modelName);
0025     
0026     %standardPhysiolDefaultParameters needs to know what sex it is dealing
0027     %with
0028     sex  = male.sex;
0029     standardPhysiolDefaultParameters;
0030     
0031     male = physiologicalConstraintsHMDBbased(male,IndividualParameters);
0032     EUAverageDietNew;
0033     male = setDietConstraints(male, Diet);
0034     model = male;
0035     modelO = model;
0036 elseif strcmp(modelName,'Harvetta')
0037     %load file corresponding to fileName
0038     female = loadPSCMfile(modelName);
0039     
0040     %standardPhysiolDefaultParameters needs to know what sex it is dealing
0041     %with
0042     sex  = female.sex;
0043     standardPhysiolDefaultParameters;
0044     
0045     female = physiologicalConstraintsHMDBbased(female,IndividualParameters);
0046     EUAverageDietNew;
0047     female = setDietConstraints(female, Diet);
0048     model = female;
0049     modelO = model;
0050 elseif strcmp(modelName,'Recon3D')
0051     if useSolveCobraLPCPLEX
0052         % load Recon3D* and
0053         load Recon3D_Harvey_Used_in_Script_120502
0054     else
0055         modelConsistent = model;
0056     end
0057     %makes modifications necessary to adjust Recon3D* for this script
0058     model = modelConsistent;
0059     model.rxns = regexprep(model.rxns,'\(e\)','[e]');
0060     model.rxns = strcat('_',model.rxns);
0061     model.rxns = regexprep(model.rxns,'_EX_','EX_');
0062     % add new compartment to Recon
0063     [model] = createModelNewCompartment(model,'e','u','urine');
0064     model.rxns = regexprep(model.rxns,'\[e\]_\[u\]','_tr_\[u\]');
0065     % add exchange reactions for the new [u] metabolites
0066     U = model.mets(~cellfun(@isempty,strfind(model.mets,'[u]')));
0067     for i = 1 : length(U)
0068         model = addExchangeRxn(model,U(i),0,1000);
0069     end
0070     
0071     % create diet reactions
0072     model.rxns = regexprep(model.rxns,'\[e\]','[d]');
0073     model.mets = regexprep(model.mets,'\[e\]','[d]');
0074     EX = model.rxns(~cellfun(@isempty,strfind(model.rxns,'EX_')));
0075     D = model.rxns(~cellfun(@isempty,strfind(model.rxns,'[d]')));
0076     EX_D = intersect(EX,D);
0077     model.rxns(ismember(model.rxns,EX_D)) = strcat('Diet_', model.rxns(ismember(model.rxns,EX_D)));
0078     % apply diet constraints
0079     model = setDietConstraints(model);
0080     % only force lower constraints of diet as no fecal outlet
0081     EX_D = model.rxns(strmatch('Diet_',model.rxns));
0082     model.ub(ismember(model.rxns,EX_D)) = 0;
0083     model.lb(ismember(model.rxns,'Diet_EX_o2[d]')) = -1000;
0084     
0085     if useSolveCobraLPCPLEX
0086         model.A = model.S;
0087     else
0088         if isfield(model,'A')
0089             model = rmfield(model,'A');
0090         end
0091     end
0092     model.rxns(ismember(model.rxns,'_biomass_maintenance')) = {'Whole_body_objective_rxn'};
0093     model.lb(ismember(model.rxns,'Whole_body_objective_rxn')) = 1;
0094     model.ub(ismember(model.rxns,'Whole_body_objective_rxn')) = 1;
0095     modelO = model;
0096 end
0097 cnt = 1;
0098 minRxnsFluxHealthy = 1;%0.9;
0099 
0100 %% integrate microbes into the whole-body reconstructions
0101 % load microbe model
0102 microbiome = 0;
0103 set = 0;
0104 if microbiome == 1
0105     files = {'SRS011239'};
0106     if set == 1
0107         S= load(strcat('/microbiota_model_samp_',files{1},'.mat'));
0108     end
0109     microbiota_model = S.microbiota_model;
0110     microbiota_model.rxns = strcat('Micro_',microbiota_model.rxns);
0111     modelHM = combineHarveyMicrotiota(model,microbiota_model,400);
0112     
0113     modelHM = changeRxnBounds(modelHM,'Whole_body_objective_rxn',1,'b');
0114     modelHMO = modelHM;
0115     % now set all strains to 0 but 1
0116     %Bacteroides_thetaiotaomicron_VPI_5482_biomass[c]
0117     % modelHM.S = modelHM.A;
0118     % modelHM.S(:,strmatch('communityBiomass',modelHM.rxns))=0;
0119     % modelHM.S(strmatch('Bacteroides_thetaiotaomicron_VPI_5482_biomass[c]',modelHM.mets),strmatch('communityBiomass',modelHM.rxns))=-1;
0120     
0121     % modelHM.S(strmatch('microbiota_LI_biomass[luM]',modelHM.mets),strmatch('communityBiomass',modelHM.rxns))=1;
0122     % modelHM.A = modelHM.S;
0123     
0124     modelHM.lb(ismember(modelHM.rxns,'Excretion_EX_microbiota_LI_biomass[fe]'))=0.1; %
0125     modelHM.ub(ismember(modelHM.rxns,'Excretion_EX_microbiota_LI_biomass[fe]'))=1; %
0126     
0127     model = modelHM;
0128 end
0129 
0130 %% set unified reaction constraints -- they are duplicated again in individual scripts
0131 
0132 R = {'_ARGSL';'_GACMTRc';'_FUM';'_FUMm';'_HMR_7698';'_UAG4E';'_UDPG4E';'_GALT'; '_G6PDH2c';'_G6PDH2r';'_G6PDH2rer';...
0133     '_GLUTCOADHm';'_r0541'; '_ACOAD8m';'_RE2410C';'_RE2410N'};
0134 RxnsAll2 = '';
0135 for i = 1: length(R)
0136     RxnsAll = model.rxns(~cellfun(@isempty,strfind(model.rxns,R{i})));
0137     RxnsAll2 =[RxnsAll2;RxnsAll];
0138 end
0139 
0140 %excluded reactions
0141 R2 = {'_FUMt';'_FUMAC';'_FUMS';'BBB'};
0142 RxnsAll4 = '';
0143 for i = 1: length(R2)
0144     RxnsAll3 = model.rxns(~cellfun(@isempty,strfind(model.rxns,R2{i})));
0145     RxnsAll4 =[RxnsAll4;RxnsAll3];
0146 end
0147 RxnsAll4 = unique(RxnsAll4);
0148 IEMRxns = setdiff(RxnsAll2,RxnsAll4);
0149 RxnMic = model.rxns(~cellfun(@isempty,strfind(model.rxns,'Micro_')));
0150 if ~isempty(RxnMic)
0151     RxnMic
0152 end
0153 IEMRxns = setdiff(IEMRxns,RxnMic);
0154 % set ARGSL to be irreversible
0155 model.lb(ismember(model.rxns,IEMRxns)) = 0;
0156 
0157 R2 = {'_r0784';'_r0463'};
0158 RxnsAll2 = '';
0159 for i = 1: length(R2)
0160     RxnsAll = model.rxns(~cellfun(@isempty,strfind(model.rxns,R2{i})));
0161     RxnsAll2 =[RxnsAll2;RxnsAll];
0162 end
0163 X = unique(RxnsAll2);
0164 RxnMic = model.rxns(~cellfun(@isempty,strfind(model.rxns,'Micro_')));
0165 if ~isempty(RxnMic)
0166     RxnMic
0167 end
0168 X = setdiff(X,RxnMic);
0169 model.lb(ismember(model.rxns,X)) = 0;
0170 model.ub(ismember(model.rxns,X)) = 0;
0171 
0172 %%%
0173 Rnew = {'BileDuct_EX_12dhchol[bd]_[luSI]';'BileDuct_EX_3dhcdchol[bd]_[luSI]';'BileDuct_EX_3dhchol[bd]_[luSI]';'BileDuct_EX_3dhdchol[bd]_[luSI]';'BileDuct_EX_3dhlchol[bd]_[luSI]';'BileDuct_EX_7dhcdchol[bd]_[luSI]';'BileDuct_EX_7dhchol[bd]_[luSI]';'BileDuct_EX_cdca24g[bd]_[luSI]';'BileDuct_EX_cdca3g[bd]_[luSI]';'BileDuct_EX_cholate[bd]_[luSI]';'BileDuct_EX_dca24g[bd]_[luSI]';'BileDuct_EX_dca3g[bd]_[luSI]';'BileDuct_EX_dchac[bd]_[luSI]';'BileDuct_EX_dgchol[bd]_[luSI]';'BileDuct_EX_gchola[bd]_[luSI]';'BileDuct_EX_hca24g[bd]_[luSI]';'BileDuct_EX_hca6g[bd]_[luSI]';'BileDuct_EX_hdca24g[bd]_[luSI]';'BileDuct_EX_hdca6g[bd]_[luSI]';'BileDuct_EX_hyochol[bd]_[luSI]';'BileDuct_EX_icdchol[bd]_[luSI]';'BileDuct_EX_isochol[bd]_[luSI]';'BileDuct_EX_lca24g[bd]_[luSI]';'BileDuct_EX_tchola[bd]_[luSI]';'BileDuct_EX_tdchola[bd]_[luSI]';'BileDuct_EX_tdechola[bd]_[luSI]';'BileDuct_EX_thyochol[bd]_[luSI]';'BileDuct_EX_uchol[bd]_[luSI]'};
0174 model.ub(ismember(model.rxns,Rnew)) = 100;
0175 
0176 modelO = model;
0177 if 1
0178     %% gene ID: 3034.1 - Histidinemia HIS
0179     model = modelO;
0180     R = '_HISD';
0181     RxnsAll = model.rxns(~cellfun(@isempty,strfind(model.rxns,R)));
0182     % exclude _HISDC reactions
0183     RxnsAll2 = model.rxns(~cellfun(@isempty,strfind(model.rxns,'_HISDC')));
0184     IEMRxns = setdiff(RxnsAll,RxnsAll2);
0185     RxnMic = model.rxns(~cellfun(@isempty,strfind(model.rxns,'Micro_')));
0186     IEMRxns = setdiff(IEMRxns,RxnMic);
0187     if ~strcmp(modelName,'Recon3D')
0188         % add demand reactions to blood compartment for those biomarkers reported for blood
0189         model = addDemandReaction(model, 'hista[bc]');
0190         model = addDemandReaction(model, 'his_L[bc]');
0191         
0192         if useSolveCobraLPCPLEX
0193             model.A = model.S;
0194         else
0195             if isfield(model,'A')
0196                 model = rmfield(model,'A');
0197             end
0198         end
0199         
0200         BiomarkerRxns ={'EX_hista[u]'    'Increased (blood/urine)'
0201             'DM_hista[bc]'    'Increased (blood/urine)'
0202             'EX_im4ac[u]'    'Increased (urine)'
0203             'EX_his_L[u]'    'Increased (blood/urine)'
0204             'DM_his_L[bc]'    'Increased (blood/urine)'
0205             };
0206     else
0207         BiomarkerRxns ={'EX_hista[u]'    'Increased (blood/urine)'
0208             'EX_im4ac[u]'    'Increased (urine)'
0209             'EX_his_L[u]'    'Increased (blood/urine)'
0210             };
0211     end
0212     [IEMSol_HIS] = checkIEM_WBM(model,IEMRxns, BiomarkerRxns,minRxnsFluxHealthy);
0213     
0214     %% %% gene ID: 2628.1 % AGAT def
0215     model = modelO;
0216     
0217     R = '_GLYAMDTRc';
0218     IEMRxns = model.rxns(~cellfun(@isempty,strfind(model.rxns,R)));
0219     RxnMic = model.rxns(~cellfun(@isempty,strfind(model.rxns,'Micro_'))) ;
0220     IEMRxns = setdiff(IEMRxns,RxnMic);
0221     % set GACMTRc reaction, which converts gudac into creat to irreversible
0222     X = model.rxns(~cellfun(@isempty,strfind(model.rxns,'_GACMTRc')));
0223     model.lb(ismember(model.rxns,X)) = 0;
0224     
0225     
0226     BiomarkerRxns = {'EX_creat[u]' 'Decreased (urine)'
0227         'EX_gudac[u]' 'Decreased (urine)' %
0228         };
0229     
0230     [IEMSol_AGAT] = checkIEM_WBM(model,IEMRxns, BiomarkerRxns,minRxnsFluxHealthy);
0231     
0232     %% %% gene ID: 383.1 % Arginase def ARG
0233     if 1
0234         model = modelO;
0235         
0236         R = '_ARGN';
0237         IEMRxns = model.rxns(~cellfun(@isempty,strfind(model.rxns,R)));
0238         RxnMic = model.rxns(~cellfun(@isempty,strfind(model.rxns,'Micro_'))) ;
0239         IEMRxns = setdiff(IEMRxns,RxnMic);
0240         % set GACMTRc reaction, which converts gudac into creat to irreversible
0241         X = model.rxns(~cellfun(@isempty,strfind(model.rxns,'_GACMTRc')));
0242         RxnMic = model.rxns(~cellfun(@isempty,strfind(model.rxns,'Micro_'))) ;
0243         X = setdiff(X,RxnMic);
0244         model.lb(ismember(model.rxns,X)) = 0;
0245         
0246         if ~strcmp(modelName,'Recon3D')
0247             % add demand reactions to blood compartment for those biomarkers reported for blood
0248             model = addDemandReaction(model, 'arg_L[bc]');
0249             model = addDemandReaction(model, 'creat[bc]');
0250             model = addDemandReaction(model, 'gudac[bc]');
0251             if useSolveCobraLPCPLEX
0252                 model.A = model.S;
0253             else
0254                 if isfield(model,'A')
0255                     model = rmfield(model,'A');
0256                 end
0257             end
0258             BiomarkerRxns = {
0259                 'EX_argsuc[u]' 'Increased (urine)'
0260                 'EX_orot[u]' 'Increased (urine)'
0261                 'EX_ura[u]' 'Increased  (urine)'
0262                 'DM_creat[bc]' 'Increased (blood)'
0263                 'DM_arg_L[bc]' 'Increased (blood)'
0264                 'DM_gudac[bc]' 'Increased (blood)'
0265                 };
0266         else
0267             BiomarkerRxns = {
0268                 'EX_argsuc[u]' 'Increased (urine)'
0269                 'EX_orot[u]' 'Increased (urine)'
0270                 'EX_ura[u]' 'Increased  (urine)'
0271                 'EX_creat[u]' 'Increased (blood)'
0272                 'EX_arg_L[u]' 'Increased (blood)'
0273                 'EX_gudac[u]' 'Increased (blood)'
0274                 };
0275         end
0276         [IEMSol_ARG] = checkIEM_WBM(model,IEMRxns, BiomarkerRxns,minRxnsFluxHealthy);
0277     end
0278     
0279     %% %% gene ID: 1373.1 % CPS1 'Carbamoyl phosphate synthetase I deficiency'
0280     model = modelO;
0281     
0282     R = {'_CBPSam';'_r0034'};
0283     RxnsAll = model.rxns(~cellfun(@isempty,strfind(model.rxns,R{1})));
0284     RxnsAll2 = model.rxns(~cellfun(@isempty,strfind(model.rxns,R{2})));
0285     IEMRxns = union(RxnsAll,RxnsAll2);
0286     RxnMic = model.rxns(~cellfun(@isempty,strfind(model.rxns,'Micro_'))) ;
0287     IEMRxns = setdiff(IEMRxns,RxnMic);
0288     if ~strcmp(modelName,'Recon3D')
0289         % add demand reactions to blood compartment for those biomarkers reported for blood
0290         model = addDemandReaction(model, 'gln_L[bc]');
0291         model = addDemandReaction(model, 'citr_L[bc]');
0292         if useSolveCobraLPCPLEX
0293             model.A = model.S;
0294         else
0295             if isfield(model,'A')
0296                 model = rmfield(model,'A');
0297             end
0298         end
0299         BiomarkerRxns = {'EX_lys_L[u]' 'Increased (urine)'
0300             'EX_gly[u]' 'Increased (urine)'
0301             'EX_ura[u]' 'Increased (urine)'
0302             'EX_5oxpro[u]' 'Increased (urine)'
0303             'DM_citr_L[bc]' 'Decreased (blood)'
0304             'DM_gln_L[bc]' 'Increased (blood)'
0305             };
0306     else
0307         
0308         BiomarkerRxns = {'EX_lys_L[u]' 'Increased (urine)'
0309             'EX_gly[u]' 'Increased (urine)'
0310             'EX_ura[u]' 'Increased (urine)'
0311             'EX_5oxpro[u]' 'Increased (urine)'
0312             'EX_citr_L[u]' 'Decreased (blood)'
0313             'EX_gln_L[u]' 'Increased (blood)'
0314             };
0315     end
0316     [IEMSol_CPS1] = checkIEM_WBM(model,IEMRxns, BiomarkerRxns,minRxnsFluxHealthy);
0317     
0318     %% gene ID: 549,1 % 3MGA 3-Methylglutaconic Aciduria Type I
0319     model = modelO;
0320     
0321     R = {'_MGCHrm'};
0322     IEMRxns = model.rxns(~cellfun(@isempty,strfind(model.rxns,R{1})));
0323     RxnMic = model.rxns(~cellfun(@isempty,strfind(model.rxns,'Micro_'))) ;
0324     IEMRxns = setdiff(IEMRxns,RxnMic);
0325     BiomarkerRxns = {'EX_3ivcrn[u]' 'Increased (urine)'
0326         % 'EX_3mglutac[u]' 'Increased (urine)'
0327         % 'EX_3mglutr[u]' 'Increased (urine)' % cannot be produced in healthy
0328         % stae
0329         };
0330     [IEMSol_3MGA] = checkIEM_WBM(model,IEMRxns, BiomarkerRxns,minRxnsFluxHealthy);
0331     
0332     %% 95.1 AMA1 Aminoacylase 1 Deficiency
0333     model = modelO;
0334     
0335     R = {'_ACODA';'_RE2640C'};
0336     RxnsAll = model.rxns(~cellfun(@isempty,strfind(model.rxns,R{1})));
0337     RxnsAll2 = model.rxns(~cellfun(@isempty,strfind(model.rxns,R{2})));
0338     IEMRxns = union(RxnsAll,RxnsAll2);
0339     RxnMic = model.rxns(~cellfun(@isempty,strfind(model.rxns,'Micro_'))) ;
0340     IEMRxns = setdiff(IEMRxns,RxnMic);
0341     
0342     BiomarkerRxns = {
0343         'EX_acglu[u]'    'Increased (urine)'
0344         'EX_acgly[u]'    'Increased (urine)'
0345         };
0346     [IEMSol_AMA1] = checkIEM_WBM(model,IEMRxns, BiomarkerRxns,minRxnsFluxHealthy);
0347     
0348     %% 1644.1 Aromatic L-amino acid decarboxylase deficiency
0349     model = modelO;
0350     
0351     R = {'_3HLYTCL';'_3HXKYNDCL';'_5HLTDL';'_5HXKYNDCL';'_LTDCL';'_PHYCBOXL';'_TYRCBOX'};
0352     RxnsAll2 = '';
0353     for i = 1: length(R)
0354         RxnsAll = model.rxns(~cellfun(@isempty,strfind(model.rxns,R{i})));
0355         RxnsAll2 =[RxnsAll2;RxnsAll];
0356     end
0357     IEMRxns = unique(RxnsAll2);
0358     RxnMic = model.rxns(~cellfun(@isempty,strfind(model.rxns,'Micro_'))) ;
0359     IEMRxns = setdiff(IEMRxns,RxnMic);
0360     if ~strcmp(modelName,'Recon3D')
0361         % add demand reactions to blood compartment for those biomarkers reported for blood
0362         model = addDemandReaction(model, '34dhphe[bc]');
0363         model = addDemandReaction(model, '5htrp[bc]');
0364         model = addDemandReaction(model, 'adrnl[bc]');
0365         model = addDemandReaction(model, 'CE2176[bc]');
0366         model = addDemandReaction(model, 'nrpphr[bc]');
0367         if useSolveCobraLPCPLEX
0368             model.A = model.S;
0369         else
0370             if isfield(model,'A')
0371                 model = rmfield(model,'A');
0372             end
0373         end
0374         BiomarkerRxns = {'EX_34dhphe[u]'    'Increased (urine/blood)'
0375             'DM_34dhphe[bc]'    'Increased (urine/blood)'
0376             'EX_5htrp[u]'    'Increased (urine/blood)'
0377             'DM_5htrp[bc]'    'Increased (urine/blood)'
0378             'DM_adrnl[bc]'    'Decreased (blood)'
0379             'EX_dopa[u]'    'Increased (urine)'
0380             'EX_CE2176[u]'    'Increased (urine/blood)'
0381             'DM_CE2176[bc]'    'Increased (urine/blood)'
0382             'DM_nrpphr[bc]'    'Decreased (blood)'
0383             'EX_3moxtyr[u]'    'Increased (urine)'
0384             };
0385     else
0386         
0387         BiomarkerRxns = {'EX_34dhphe[u]'    'Increased (urine/blood)'
0388             'EX_5htrp[u]'    'Increased (urine/blood)'
0389             'EX_adrnl[u]'    'Decreased (blood)'
0390             'EX_dopa[u]'    'Increased (urine)'
0391             'EX_CE2176[u]'    'Increased (urine/blood)'
0392             'EX_nrpphr[u]'    'Decreased (blood)'
0393             'EX_3moxtyr[u]'    'Increased (urine)'
0394             };
0395     end
0396     
0397     [IEMSol_AADC] = checkIEM_WBM(model,IEMRxns, BiomarkerRxns,minRxnsFluxHealthy);
0398     
0399     %% '56922.1' '3-methylcrotonyl coA carboxylase deficiency'
0400     model = modelO;
0401     
0402     R = {'_MCCCrm';'_RE2453M';'_RE2454M'};
0403     RxnsAll2 = '';
0404     for i = 1: length(R)
0405         RxnsAll = model.rxns(~cellfun(@isempty,strfind(model.rxns,R{i})));
0406         RxnsAll2 =[RxnsAll2;RxnsAll];
0407     end
0408     IEMRxns = unique(RxnsAll2);
0409     RxnMic = model.rxns(~cellfun(@isempty,strfind(model.rxns,'Micro_'))) ;
0410     IEMRxns = setdiff(IEMRxns,RxnMic);
0411     
0412     BiomarkerRxns = {'EX_3ivcrn[u]'    'Increased (urine)'
0413         %   'EX_acac[u]'    'Increased (urine)' % ketone bodies were not mentioned
0414         %   here: https://www.ncbi.nlm.nih.gov/pmc/articles/PMC1182108/
0415         %  'EX_acetone[u]'    'Increased (urine)'
0416         % 'EX_bhb[u]'    'Increased (urine)'
0417         'EX_CE2026[u]'    'Increased (urine)' % 3-methylcrotonylglycine
0418         'EX_3hivac[u]'    'Increased (urine)'
0419         };
0420     [IEMSol_3MCC] = checkIEM_WBM(model,IEMRxns, BiomarkerRxns,minRxnsFluxHealthy);
0421     
0422     %% '1589.1' CYP21D    '21-hydroxylase deficiency'
0423     if 1
0424         model = modelO;
0425         
0426         R = {'_P45021A1r';'_P45021A2r';'_RE2155R';'_21HPRGNLONE';'_HMR_1940';'_HMR_1948';'_HMR_1988';'_HMR_1990';'_HMR_1992';'_HMR_2007'};
0427         RxnsAll2 = '';
0428         for i = 1: length(R)
0429             RxnsAll = model.rxns(~cellfun(@isempty,strfind(model.rxns,R{i})));
0430             RxnsAll2 =[RxnsAll2;RxnsAll];
0431         end
0432         IEMRxns = unique(RxnsAll2);
0433         RxnMic = model.rxns(~cellfun(@isempty,strfind(model.rxns,'Micro_'))) ;
0434         IEMRxns = setdiff(IEMRxns,RxnMic);
0435         
0436         if ~strcmp(modelName,'Recon3D')
0437             % add demand reactions to blood compartment for those biomarkers reported for blood
0438             model = addDemandReaction(model, 'aldstrn[bc]');
0439             model = addDemandReaction(model, 'crtsl[bc]');
0440             model = addDemandReaction(model, 'prgstrn[bc]');
0441             model = addDemandReaction(model, '17ahprgnlone[bc]');
0442             model = addDemandReaction(model, '17ahprgstrn[bc]');
0443             model = addDemandReaction(model, 'M00603[bc]');
0444             model = addDemandReaction(model, 'andrstndn[bc]');
0445             model = addDemandReaction(model, 'andrstrn[bc]');
0446             model = addDemandReaction(model, 'dhea[bc]');
0447             model = addDemandReaction(model, 'C05284[bc]');
0448             model = addDemandReaction(model, 'CE2211[bc]');
0449             if useSolveCobraLPCPLEX
0450                 model.A = model.S;
0451             else
0452                 if isfield(model,'A')
0453                     model = rmfield(model,'A');
0454                 end
0455             end
0456             BiomarkerRxns = {'DM_aldstrn[bc]'    'Increased (blood)'%17-Ketotestosterone
0457                 'DM_crtsl[bc]'    'Decreased (blood)' %cortisol
0458                 'DM_prgstrn[bc]'    'Increased (blood)'  %Progesterone
0459                 'DM_andrstndn[bc]'    'Increased (blood)' %17-Ketotestosterone
0460                 'DM_andrstrn[bc]' 'Increased (blood)' % Androsterone,in male
0461                 'DM_dhea[bc]'    'Increased (blood)' %Dehydroepiandrosterone unchanged in this study: PMID: 28472487
0462                 };
0463         else
0464             BiomarkerRxns = {'EX_aldstrn[u]'    'Increased (blood)'%17-Ketotestosterone
0465                 'EX_crtsl[u]'    'Decreased (blood)' %cortisol
0466                 'EX_prgstrn[u]'    'Increased (blood)'  %Progesterone
0467                 'EX_andrstndn[u]'    'Increased (blood)' %17-Ketotestosterone
0468                 'EX_andrstrn[u]' 'Increased (blood)' % Androsterone,in male
0469                 'EX_dhea[u]'    'Increased (blood)' %Dehydroepiandrosterone unchanged in this study: PMID: 28472487
0470                 };
0471         end
0472         [IEMSol_CYP21D] = checkIEM_WBM(model,IEMRxns, BiomarkerRxns,minRxnsFluxHealthy);
0473     end
0474     %% '53630.1' Autosomal Dominant Hypercarotenemia And Vitamin A Deficiency
0475     model = modelO;
0476     
0477     R = {'_BCDO'};
0478     RxnsAll2 = '';
0479     for i = 1: length(R)
0480         RxnsAll = model.rxns(~cellfun(@isempty,strfind(model.rxns,R{i})));
0481         RxnsAll2 =[RxnsAll2;RxnsAll];
0482     end
0483     IEMRxns = unique(RxnsAll2);
0484     RxnMic = model.rxns(~cellfun(@isempty,strfind(model.rxns,'Micro_'))) ;
0485     IEMRxns = setdiff(IEMRxns,RxnMic);
0486     
0487     if ~strcmp(modelName,'Recon3D')
0488         % add demand reactions to blood compartment for those biomarkers reported for blood
0489         model = addDemandReaction(model, 'caro[bc]');
0490         if useSolveCobraLPCPLEX
0491             model.A = model.S;
0492         else
0493             if isfield(model,'A')
0494                 model = rmfield(model,'A');
0495             end
0496         end
0497         BiomarkerRxns = {'DM_caro[bc]'    'Increased (blood)'
0498             };
0499     else
0500         BiomarkerRxns = {'EX_caro[u]'    'Increased (blood)'
0501             };
0502     end
0503     [IEMSol_HYCARO] = checkIEM_WBM(model,IEMRxns, BiomarkerRxns,minRxnsFluxHealthy);
0504     
0505     %% '686.1' BTD Biotinidase Deficiency
0506     if 1
0507         model = modelO;
0508         
0509         % https://www.nature.com/articles/gim201784: Biochemically, untreated individuals may exhibit
0510         % metabolic ketoacidosis, lactic acidosis, and/or hyperammonemia.(PMID: 3930841)
0511         % Other metabolic abnormalities are more variable and may include elevated excretion
0512         % of 3-hydroxyisovaleric, lactic, and 3-hydroxypropionic acids and 3-methylcrotonylglycine
0513         % by urine organic acid analysis, as well as mildly elevated 3-hydroxyisovalerylcarnitine
0514         % (C5-OH) by plasma acylcarnitine analysis.(PMID: 6441143) These metabolic abnormalities are variable,
0515         % and affected children, whether symptomatic or asymptomatic, do not always
0516         % exhibit ketoacidosis or organic aciduria.(PMID: 3930841)
0517         
0518         R = {'_BTND1';'_BTND1n';'_BTNDe';'_BTNDm';...
0519             '_ACCOACm';'_ACCOAC';'_PCm';'_MCCCrm';'_RE2453M';'_RE2454M';'_PPCOACm'
0520             };
0521         RxnsAll2 = '';
0522         for i = 1: length(R)
0523             RxnsAll = model.rxns(~cellfun(@isempty,strfind(model.rxns,R{i})));
0524             RxnsAll2 =[RxnsAll2;RxnsAll];
0525         end
0526         IEMRxns = unique(RxnsAll2);
0527         RxnMic = model.rxns(~cellfun(@isempty,strfind(model.rxns,'Micro_'))) ;
0528         IEMRxns = setdiff(IEMRxns,RxnMic);
0529         
0530         if ~strcmp(modelName,'Recon3D')
0531             % add demand reactions to blood compartment for those biomarkers reported for blood
0532             model = addDemandReaction(model, 'acac[bc]');
0533             model = addDemandReaction(model, 'acetone[bc]');
0534             model = addDemandReaction(model, 'bhb[bc]');
0535             model = addDemandReaction(model, '3ivcrn[bc]');
0536             if useSolveCobraLPCPLEX
0537                 model.A = model.S;
0538             else
0539                 if isfield(model,'A')
0540                     model = rmfield(model,'A');
0541                 end
0542             end
0543             BiomarkerRxns = {%'DM_3ivcrn[bc]'    'Increased (blood)' %https://www.nature.com/articles/gim201784
0544                'DM_acac[bc]'    'Increased (blood)'
0545                'DM_acetone[bc]'    'Increased (blood)'
0546                 'DM_bhb[bc]'    'Increased (blood)'
0547                 'EX_lac_L[u]'    'Increased (urine)'
0548                 'EX_nh4[u]'    'Increased (urine)'
0549                 'EX_3hpp[u]'    'Increased (urine)'
0550                 'EX_CE2026[u]'    'Increased (urine)'
0551                 'EX_2mcit[u]'    'Increased (urine)'
0552                 };
0553         else
0554             BiomarkerRxns = {'EX_3ivcrn[u]'    'Increased (urine)'
0555                 'EX_acac[u]'    'Increased (blood)'
0556                 'EX_acetone[u]'    'Increased (blood)'
0557                 'EX_bhb[u]'    'Increased (blood)'
0558                 'EX_lac_L[u]'    'Increased (urine)'
0559                 'EX_nh4[u]'    'Increased (urine)'
0560                 'EX_3hpp[u]'    'Increased (urine)'
0561                 'EX_CE2026[u]'    'Increased (urine)'
0562                 'EX_2mcit[u]'    'Increased (urine)'
0563                 };
0564         end
0565         
0566         [IEMSol_BTD] = checkIEM_WBM(model,IEMRxns, BiomarkerRxns,minRxnsFluxHealthy,[],0.25);
0567         
0568     end
0569     %% '5264.1' CRFD Classic Refsum Disease
0570     if 0
0571         model = modelO;
0572         R = {'_PHYHx';'_RE3066X'};
0573         RxnsAll2 = '';
0574         for i = 1: length(R)
0575             RxnsAll = model.rxns(find(~cellfun(@isempty,strfind(model.rxns,R{i}))));
0576             RxnsAll2 =[RxnsAll2;RxnsAll];
0577         end
0578         IEMRxns = unique(RxnsAll2);
0579         RxnMic = model.rxns(find(~cellfun(@isempty,strfind(model.rxns,'Micro_')))) ;
0580         IEMRxns = setdiff(IEMRxns,RxnMic);
0581         if ~strcmp(modelName,'Recon3D')
0582             % add demand reactions to blood compartment for those biomarkers reported for blood
0583             model = addDemandReaction(model, 'phyt[bc]');
0584             model = addDemandReaction(model, 'prist[bc]');
0585             if useSolveCobraLPCPLEX
0586                 model.A = model.S;
0587             else
0588                 if isfield(model,'A')
0589                     model = rmfield(model,'A');
0590                 end
0591             end
0592             BiomarkerRxns = {'DM_phyt[bc]'   'Increased (blood)'
0593                 'DM_prist[bc]'   'Decreased (blood)'
0594                 };
0595         else
0596             BiomarkerRxns = {'EX_phyt[u]'   'Increased (blood)'
0597                 'EX_prist[u]'   'Decreased (blood)'
0598                 };
0599         end
0600         [IEMSol_CRFD] = checkIEM_WBM(model,IEMRxns, BiomarkerRxns,minRxnsFluxHealthy);
0601     end
0602     
0603     %% '1538.1' STAR Congenital Lipoid Adrenal Hyperplasia (Clah)/ Star Deficiency
0604     model = modelO;
0605     
0606     R = {'_P45011A1m';'_HMR_1928';'_HMR_1929';'_HMR_1932';'_HMR_1934';'_HMR_1935'};
0607     RxnsAll2 = '';
0608     for i = 1: length(R)
0609         RxnsAll = model.rxns(~cellfun(@isempty,strfind(model.rxns,R{i})));
0610         RxnsAll2 =[RxnsAll2;RxnsAll];
0611     end
0612     IEMRxns = unique(RxnsAll2);
0613     RxnMic = model.rxns(~cellfun(@isempty,strfind(model.rxns,'Micro_'))) ;
0614     IEMRxns = setdiff(IEMRxns,RxnMic);
0615     
0616     if ~strcmp(modelName,'Recon3D')
0617         % add demand reactions to blood compartment for those biomarkers reported for blood
0618         model = addDemandReaction(model, 'crtsl[bc]');
0619         model = addDemandReaction(model, 'crtstrn[bc]');
0620         model = addDemandReaction(model, '17ahprgstrn[bc]');
0621         model = addDemandReaction(model, 'andrstndn[bc]');
0622         model = addDemandReaction(model, 'dhea[bc]');
0623         model = addDemandReaction(model, '11docrtstrn[bc]');
0624         if useSolveCobraLPCPLEX
0625             model.A = model.S;
0626         else
0627             if isfield(model,'A')
0628                 model = rmfield(model,'A');
0629             end
0630         end
0631         BiomarkerRxns = {'DM_crtsl[bc]'    'Decreased (blood)'
0632             'DM_crtstrn[bc]'    'Decreased (blood)'
0633             'DM_andrstndn[bc]'    'Decreased (blood)'
0634             'DM_dhea[bc]'    'Decreased (blood)'
0635             'DM_11docrtstrn[bc]'    'Decreased (blood)'
0636             };
0637     else
0638         BiomarkerRxns = {'EX_crtsl[u]'    'Decreased (blood)'
0639             'EX_crtstrn[u]'    'Decreased (blood)'
0640             'EX_andrstndn[u]'    'Decreased (blood)'
0641             'EX_dhea[u]'    'Decreased (blood)'
0642             'EX_11docrtstrn[u]'    'Decreased (blood)'
0643             };
0644     end
0645     
0646     [IEMSol_STAR] = checkIEM_WBM(model,IEMRxns, BiomarkerRxns,minRxnsFluxHealthy);
0647     
0648     %% '1585.1' CMO1 Corticosterone Methyloxidase Type I Deficiency
0649     model = modelO;
0650     
0651     R = {'_P45011B21m'};
0652     RxnsAll2 = '';
0653     for i = 1: length(R)
0654         RxnsAll = model.rxns(~cellfun(@isempty,strfind(model.rxns,R{i})));
0655         RxnsAll2 =[RxnsAll2;RxnsAll];
0656     end
0657     IEMRxns = unique(RxnsAll2);
0658     RxnMic = model.rxns(~cellfun(@isempty,strfind(model.rxns,'Micro_'))) ;
0659     IEMRxns = setdiff(IEMRxns,RxnMic);
0660     
0661     if ~strcmp(modelName,'Recon3D')
0662         % add demand reactions to blood compartment for those biomarkers reported for blood
0663         model = addDemandReaction(model, 'aldstrn[bc]');
0664         model = addDemandReaction(model, 'M00429[bc]');
0665         if useSolveCobraLPCPLEX
0666             model.A = model.S;
0667         else
0668             if isfield(model,'A')
0669                 model = rmfield(model,'A');
0670             end
0671         end
0672         BiomarkerRxns = {'DM_aldstrn[bc]'    'Decreased (Not detectable, blood)'
0673             };
0674     else
0675         BiomarkerRxns = {'EX_aldstrn[u]'    'Decreased (Not detectable, blood)'
0676             };
0677     end
0678     [IEMSol_CMO1] = checkIEM_WBM(model,IEMRxns, BiomarkerRxns,minRxnsFluxHealthy);
0679     
0680     %% '1716.1' DGK Deoxyguanosine Kinase Deficiency
0681     if 1
0682         model = modelO;
0683         
0684         R = {'_r0456'};
0685         RxnsAll2 = '';
0686         for i = 1: length(R)
0687             RxnsAll = model.rxns(~cellfun(@isempty,strfind(model.rxns,R{i})));
0688             RxnsAll2 =[RxnsAll2;RxnsAll];
0689         end
0690         IEMRxns = unique(RxnsAll2);
0691         RxnMic = model.rxns(~cellfun(@isempty,strfind(model.rxns,'Micro_'))) ;
0692         IEMRxns = setdiff(IEMRxns,RxnMic);
0693         
0694         if ~strcmp(modelName,'Recon3D')
0695             % add demand reactions to blood compartment for those biomarkers reported for blood
0696             model = addDemandReaction(model, 'lac_L[bc]');
0697             if useSolveCobraLPCPLEX
0698                 model.A = model.S;
0699             else
0700                 if isfield(model,'A')
0701                     model = rmfield(model,'A');
0702                 end
0703             end
0704             BiomarkerRxns = {
0705                 'DM_lac_L[bc]'    'Increased (blood)'
0706                 };
0707         else
0708             BiomarkerRxns = {
0709                 'EX_lac_L[u]'    'Increased (blood)'
0710                 };
0711         end
0712         [IEMSol_DGK] = checkIEM_WBM(model,IEMRxns, BiomarkerRxns,minRxnsFluxHealthy);
0713     end
0714     %% '1807.1 'DPYR Dihydropyrimidinuria
0715     model = modelO;
0716     
0717     R = {'_DHPM2'};
0718     RxnsAll2 = '';
0719     for i = 1: length(R)
0720         RxnsAll = model.rxns(~cellfun(@isempty,strfind(model.rxns,R{i})));
0721         RxnsAll2 =[RxnsAll2;RxnsAll];
0722     end
0723     IEMRxns = unique(RxnsAll2);
0724     RxnMic = model.rxns(~cellfun(@isempty,strfind(model.rxns,'Micro_'))) ;
0725     IEMRxns = setdiff(IEMRxns,RxnMic);
0726     
0727     BiomarkerRxns = {
0728         'EX_thym[u]'    'Increased (urine)'
0729         'EX_ura[u]'    'Increased (urine)'
0730         'EX_56dura[u]'    'Increased (urine)'
0731         'EX_56dthm[u]'    'Increased (urine)'
0732         };
0733     [IEMSol_DPYR] = checkIEM_WBM(model,IEMRxns, BiomarkerRxns,minRxnsFluxHealthy);
0734     
0735     %% '3931.1' FED Fish-Eye Disease/ Lcat Deficiency
0736     if 1
0737         model = modelO;
0738         
0739         R = {'_HMR_0634';'_LCAT1e';'_LCAT10e';'_LCAT11e';'_LCAT12e';'_LCAT13e';'_LCAT14e';'_LCAT15e';'_LCAT16e';'_LCAT17e';'_LCAT18e';'_LCAT19e';...
0740             '_LCAT2e';'_LCAT20e';'_LCAT21e';'_LCAT22e';'_LCAT23e';'_LCAT24e';'_LCAT25e';'_LCAT26e';'_LCAT27e';'_LCAT28e';'_LCAT29e';...
0741             '_LCAT3e';'_LCAT30e';'_LCAT31e';'_LCAT32e';'_LCAT33e';'_LCAT34e';'_LCAT35e';'_LCAT36e';'_LCAT37e';'_LCAT38e';'_LCAT39e';...
0742             '_LCAT4e';'_LCAT40e';'_LCAT41e';'_LCAT42e';'_LCAT43e';'_LCAT44e';'_LCAT45e';'_LCAT46e';'_LCAT47e';'_LCAT48e';'_LCAT49e';...
0743             '_LCAT5e';'_LCAT50e';'_LCAT51e';'_LCAT52e';'_LCAT53e';'_LCAT54e';'_LCAT55e';'_LCAT56e';'_LCAT57e';'_LCAT58e';'_LCAT59e'
0744             };
0745         RxnsAll2 = '';
0746         for i = 1: length(R)
0747             RxnsAll = model.rxns(~cellfun(@isempty,strfind(model.rxns,R{i})));
0748             RxnsAll2 =[RxnsAll2;RxnsAll];
0749         end
0750         IEMRxns = unique(RxnsAll2);
0751         RxnMic = model.rxns(~cellfun(@isempty,strfind(model.rxns,'Micro_'))) ;
0752         IEMRxns = setdiff(IEMRxns,RxnMic);
0753         if ~strcmp(modelName,'Recon3D')
0754             % add demand reactions to blood compartment for those biomarkers reported for blood
0755             model = addDemandReaction(model, 'chsterol[bc]');
0756             model = addDemandReaction(model, 'tag_hs[bc]');
0757             if useSolveCobraLPCPLEX
0758                 model.A = model.S;
0759             else
0760                 if isfield(model,'A')
0761                     model = rmfield(model,'A');
0762                 end
0763             end
0764             BiomarkerRxns = {
0765                 'DM_chsterol[bc]'    'Decreased (blood)' %PMID: 8675648: "showed a highly significant reduction of HDL-cholesterol"
0766                 'DM_tag_hs[bc]'    'Increased (blood)' % PMID: 3141686: "They had fasting hypertriglyceridaemia." We are not modeling fasting condition
0767                 };
0768         else
0769             BiomarkerRxns = {
0770                 'EX_chsterol[u]'    'Decreased (blood)' %PMID: 8675648: "showed a highly significant reduction of HDL-cholesterol"
0771                 'EX_tag_hs[u]'    'Increased (blood)' % PMID: 3141686: "They had fasting hypertriglyceridaemia." We are not modeling fasting condition
0772                 };
0773         end
0774         [IEMSol_FED] = checkIEM_WBM(model,IEMRxns, BiomarkerRxns,minRxnsFluxHealthy);
0775     end
0776     %% '3795.1' EF Fructosuria(Essential Fructosuria)
0777     model = modelO;
0778     
0779     R = {'_HMR_8761';'_HMR_9800';'_KHK';'_KHK2';'_KHK3'};
0780     RxnsAll2 = '';
0781     for i = 1: length(R)
0782         RxnsAll = model.rxns(~cellfun(@isempty,strfind(model.rxns,R{i})));
0783         RxnsAll2 =[RxnsAll2;RxnsAll];
0784     end
0785     IEMRxns = unique(RxnsAll2);
0786     RxnMic = model.rxns(~cellfun(@isempty,strfind(model.rxns,'Micro_'))) ;
0787     IEMRxns = setdiff(IEMRxns,RxnMic);
0788     
0789     BiomarkerRxns = {
0790         'EX_fru[u]'    'Increased (urine)'
0791         };
0792     [IEMSol_EF] = checkIEM_WBM(model,IEMRxns, BiomarkerRxns,minRxnsFluxHealthy);
0793     
0794     %% '10841.1' FIGLU Glutamate Formiminotransferase Deficiency
0795     model = modelO;
0796     
0797     R = {'_FORTHFC';'_GluForTx';'_HMR_9726'};
0798     RxnsAll2 = '';
0799     for i = 1: length(R)
0800         RxnsAll = model.rxns(~cellfun(@isempty,strfind(model.rxns,R{i})));
0801         RxnsAll2 =[RxnsAll2;RxnsAll];
0802     end
0803     IEMRxns = unique(RxnsAll2);
0804     RxnMic = model.rxns(~cellfun(@isempty,strfind(model.rxns,'Micro_'))) ;
0805     IEMRxns = setdiff(IEMRxns,RxnMic);
0806     
0807     BiomarkerRxns = {
0808         'EX_forglu[u]'  'Increased (urine)'
0809         };
0810     [IEMSol_FIGLU] = checkIEM_WBM(model,IEMRxns, BiomarkerRxns,minRxnsFluxHealthy);
0811     
0812     %% '2639.1' GA1 Glutaric Acidemia Type I.
0813     model = modelO;
0814     
0815     R = {'_GLUTCOADHm';'_r0541'};
0816     RxnsAll2 = '';
0817     for i = 1: length(R)
0818         RxnsAll = model.rxns(~cellfun(@isempty,strfind(model.rxns,R{i})));
0819         RxnsAll2 =[RxnsAll2;RxnsAll];
0820     end
0821     IEMRxns = unique(RxnsAll2);
0822     RxnMic = model.rxns(~cellfun(@isempty,strfind(model.rxns,'Micro_'))) ;
0823     IEMRxns = setdiff(IEMRxns,RxnMic);
0824     model.lb(ismember(model.rxns,IEMRxns)) = 0;
0825     if ~strcmp(modelName,'Recon3D')
0826         % add demand reactions to blood compartment for those biomarkers reported for blood
0827         model = addDemandReaction(model, 'c5dc[bc]');
0828         if useSolveCobraLPCPLEX
0829             model.A = model.S;
0830         else
0831             if isfield(model,'A')
0832                 model = rmfield(model,'A');
0833             end
0834         end
0835         BiomarkerRxns = {
0836             'EX_c5dc[u]'    'Increased (blood/urine)'
0837             'DM_c5dc[bc]'    'Increased (blood/urine)'
0838             };
0839     else
0840         BiomarkerRxns = {
0841             'EX_c5dc[u]'    'Increased (blood/urine)'
0842             };
0843     end
0844     [IEMSol_GA1] = checkIEM_WBM(model,IEMRxns, BiomarkerRxns,minRxnsFluxHealthy);
0845     
0846     %% '2108.1' GA2 Glutaric Acidemia Type II
0847     model = modelO;
0848     
0849     R = {'_ETF';'_FADH2ETC'};
0850     RxnsAll2 = '';
0851     for i = 1: length(R)
0852         RxnsAll = model.rxns(~cellfun(@isempty,strfind(model.rxns,R{i})));
0853         RxnsAll2 =[RxnsAll2;RxnsAll];
0854     end
0855     IEMRxns = unique(RxnsAll2);
0856     RxnMic = model.rxns(~cellfun(@isempty,strfind(model.rxns,'Micro_'))) ;
0857     IEMRxns = setdiff(IEMRxns,RxnMic);
0858     
0859     if ~strcmp(modelName,'Recon3D')
0860         % add demand reactions to blood compartment for those biomarkers reported for blood
0861         model = addDemandReaction(model, 'c10crn[bc]');
0862         model = addDemandReaction(model, 'c4crn[bc]');
0863         model = addDemandReaction(model, 'ddeccrn[bc]');
0864         model = addDemandReaction(model, 'ttdcrn[bc]');
0865         model = addDemandReaction(model, 'pmtcrn[bc]');
0866         if useSolveCobraLPCPLEX
0867             model.A = model.S;
0868         else
0869             if isfield(model,'A')
0870                 model = rmfield(model,'A');
0871             end
0872         end
0873         BiomarkerRxns = {
0874             'DM_c10crn[bc]'    'Increased (blood)'
0875             'DM_c4crn[bc]'    'Increased (blood)'
0876             'DM_ddeccrn[bc]'    'Increased (blood)'
0877             'DM_ttdcrn[bc]'    'Increased (blood)'
0878             'DM_pmtcrn[bc]'    'Increased (blood)'
0879             'EX_4hpro_LT[u]'    'Increased (urine)'
0880             'EX_3hivac[u]'    'Increased (urine)'
0881             'EX_CE4970[u]'    'Increased (urine)'
0882             'EX_CE4969[u]'    'Increased (urine)'
0883             'EX_CE4968[u]'    'Increased (urine)'
0884             'EX_ethmalac[u]'    'Increased (urine)'
0885             'EX_adpac[u]'    'Increased (urine)'
0886             'EX_subeac[u]'    'Increased (urine)'
0887             'EX_sebacid[u]'    'Increased (urine)'
0888             'EX_pro_L[u]'    'Increased (urine)'
0889             };
0890     else
0891         BiomarkerRxns = {
0892             'EX_c10crn[u]'    'Increased (blood)'
0893             'EX_c4crn[u]'    'Increased (blood)'
0894             'EX_ddeccrn[u]'    'Increased (blood)'
0895             'EX_ttdcrn[u]'    'Increased (blood)'
0896             'EX_pmtcrn[u]'    'Increased (blood)'
0897             'EX_4hpro_LT[u]'    'Increased (urine)'
0898             'EX_pro_L[u]'    'Increased (urine)'
0899             'EX_3hivac[u]'    'Increased (urine)'
0900             'EX_CE4970[u]'    'Increased (urine)'
0901             'EX_CE4969[u]'    'Increased (urine)'
0902             'EX_CE4968[u]'    'Increased (urine)'
0903             'EX_ethmalac[u]'    'Increased (urine)'
0904             'EX_adpac[u]'    'Increased (urine)'
0905             'EX_subeac[u]'    'Increased (urine)'
0906             'EX_sebacid[u]'    'Increased (urine)'
0907             };
0908     end
0909     [IEMSol_GA2] = checkIEM_WBM(model,IEMRxns, BiomarkerRxns,minRxnsFluxHealthy);
0910     
0911     %% '2937.1' OXOP Glutathione Synthetase Deficiency And 5-Oxoprolinuria
0912     model = modelO;
0913     
0914     R = {'_GTHS'};
0915     RxnsAll2 = '';
0916     for i = 1: length(R)
0917         RxnsAll = model.rxns(~cellfun(@isempty,strfind(model.rxns,R{i})));
0918         RxnsAll2 =[RxnsAll2;RxnsAll];
0919     end
0920     IEMRxns = unique(RxnsAll2);
0921     RxnMic = model.rxns(~cellfun(@isempty,strfind(model.rxns,'Micro_'))) ;
0922     IEMRxns = setdiff(IEMRxns,RxnMic);
0923     
0924     if ~strcmp(modelName,'Recon3D')
0925         % add demand reactions to blood compartment for those biomarkers reported for blood
0926         model = addDemandReaction(model, '5oxpro[bc]');
0927         model = addDemandReaction(model, 'leuktrE4[bc]');
0928         model = addDemandReaction(model, 'leuktrC4[bc]');
0929         model = addDemandReaction(model, 'leuktrD4[bc]');
0930         model = addDemandReaction(model, 'pro_L[bc]');
0931         if useSolveCobraLPCPLEX
0932             model.A = model.S;
0933         else
0934             if isfield(model,'A')
0935                 model = rmfield(model,'A');
0936             end
0937         end
0938         BiomarkerRxns = {
0939             'EX_5oxpro[u]'    'Increased (urine)'
0940             'EX_leuktrE4[u]'    'Increased (urine/blood)'
0941             'EX_leuktrC4[u]'    'Increased (urine/blood)'
0942             'DM_5oxpro[bc]'    'Decreased (blood)'
0943             'DM_leuktrE4[bc]'    'Increased (urine/blood)'
0944             'DM_leuktrC4[bc]'    'Increased (urine/blood)'
0945             'DM_leuktrD4[bc]'    'Increased (urine/blood)'
0946             'DM_pro_L[bc]'    'Increased (blood)'
0947             };
0948     else
0949         
0950         BiomarkerRxns = {
0951             'EX_5oxpro[u]'    'Increased (urine)/ Decreased (blood)'
0952             'EX_leuktrE4[u]'    'Increased (urine/blood)'
0953             'EX_leuktrC4[u]'    'Increased (urine/blood)'
0954             'EX_leuktrD4[u]'    'Increased (urine/blood)'
0955             'EX_pro_L[u]'    'Increased (blood)'
0956             };
0957     end
0958     [IEMSol_OXOP] = checkIEM_WBM(model,IEMRxns, BiomarkerRxns,minRxnsFluxHealthy);
0959     
0960     %% '178.1'GSD6 Glycogen Storage Disease Type 6/Hers Disease
0961     if 0
0962         model = modelO;
0963         
0964         R = {'_r1393'};
0965         RxnsAll2 = '';
0966         for i = 1: length(R)
0967             RxnsAll = model.rxns(find(~cellfun(@isempty,strfind(model.rxns,R{i}))));
0968             RxnsAll2 =[RxnsAll2;RxnsAll];
0969         end
0970         IEMRxns = unique(RxnsAll2);
0971         RxnMic = model.rxns(find(~cellfun(@isempty,strfind(model.rxns,'Micro_')))) ;
0972         IEMRxns = setdiff(IEMRxns,RxnMic);
0973         
0974         if ~strcmp(modelName,'Recon3D')
0975             % add demand reactions to blood compartment for those biomarkers reported for blood
0976             model = addDemandReaction(model, 'chsterol[bc]');
0977             model = addDemandReaction(model, 'glc_D[bc]');
0978             model = addDemandReaction(model, 'lac_L[bc]');
0979             if useSolveCobraLPCPLEX
0980                 model.A = model.S;
0981             else
0982                 if isfield(model,'A')
0983                     model = rmfield(model,'A');
0984                 end
0985             end
0986             BiomarkerRxns = {
0987                 'EX_acac[u]'    'Increased (urine)'
0988                 'EX_acetone[u]'    'Increased (urine)'
0989                 'EX_bhb[u]'    'Increased (urine)'
0990                 'DM_chsterol[bc]'    'Increased (blood)'
0991                 'DM_glc_D[bc]'    'Increased (blood)'
0992                 'DM_lac_L[bc]'    'Increased (blood)'
0993                 };
0994         else
0995             BiomarkerRxns = {
0996                 'EX_acac[u]'    'Increased (urine)'
0997                 'EX_acetone[u]'    'Increased (urine)'
0998                 'EX_bhb[u]'    'Increased (urine)'
0999                 'EX_chsterol[u]'    'Increased (blood)'
1000                 'EX_glc_D[u]'    'Increased (blood)'
1001                 'EX_lac_L[u]'    'Increased (blood)'
1002                 };
1003         end
1004         [IEMSol_GSD6] = checkIEM_WBM(model,IEMRxns, BiomarkerRxns,minRxnsFluxHealthy);
1005     end
1006     
1007     %% '2593.1' GMT Guanidinoacetate Methyltransferase Deficiency
1008     model = modelO;
1009     
1010     R = {'_GACMTRc'};
1011     RxnsAll2 = '';
1012     for i = 1: length(R)
1013         RxnsAll = model.rxns(find(~cellfun(@isempty,strfind(model.rxns,R{i}))));
1014         RxnsAll2 =[RxnsAll2;RxnsAll];
1015     end
1016     IEMRxns = unique(RxnsAll2);
1017     RxnMic = model.rxns(find(~cellfun(@isempty,strfind(model.rxns,'Micro_')))) ;
1018     IEMRxns = setdiff(IEMRxns,RxnMic);
1019     
1020     if ~strcmp(modelName,'Recon3D')
1021         % add demand reactions to blood compartment for those biomarkers reported for blood
1022         model = addDemandReaction(model, 'creat[bc]');
1023         model = addDemandReaction(model, 'crtn[bc]');
1024         if useSolveCobraLPCPLEX
1025             model.A = model.S;
1026         else
1027             if isfield(model,'A')
1028                 model = rmfield(model,'A');
1029             end
1030         end
1031         BiomarkerRxns = {
1032             'EX_creat[u]'    'Decreased (urine/blood)'
1033             'DM_creat[bc]'    'Decreased (urine/blood)'
1034             'DM_crtn[bc]'    'Increased (blood)'
1035             'EX_urate[u]'    'Increased (urine)'
1036             'EX_gudac[u]'    'Increased (urine)'
1037             };
1038     else
1039         BiomarkerRxns = {
1040             'EX_creat[u]'    'Decreased (urine/blood)'
1041             'EX_crtn[u]'    'Increased (blood)'
1042             'EX_urate[u]'    'Increased (urine)'
1043             'EX_gudac[u]'    'Increased (urine)'
1044             };
1045     end
1046     [IEMSol_GMT] = checkIEM_WBM(model,IEMRxns, BiomarkerRxns,minRxnsFluxHealthy);
1047     %% '4942.1' GACR Gyrate Atrophy Of The Choroid And Retina
1048     model = modelO;
1049     
1050     R = {'_ORNTArm'};
1051     RxnsAll2 = '';
1052     for i = 1: length(R)
1053         RxnsAll = model.rxns(find(~cellfun(@isempty,strfind(model.rxns,R{i}))));
1054         RxnsAll2 =[RxnsAll2;RxnsAll];
1055     end
1056     IEMRxns = unique(RxnsAll2);
1057     RxnMic = model.rxns(find(~cellfun(@isempty,strfind(model.rxns,'Micro_')))) ;
1058     IEMRxns = setdiff(IEMRxns,RxnMic);
1059     
1060     if ~strcmp(modelName,'Recon3D')
1061         % add demand reactions to blood compartment for those biomarkers reported for blood
1062         model = addDemandReaction(model, 'gln_L[bc]');
1063         model = addDemandReaction(model, 'lys_L[bc]');
1064         model = addDemandReaction(model, 'orn[bc]');
1065         model = addDemandReaction(model, 'orn[csf]');
1066         if useSolveCobraLPCPLEX
1067             model.A = model.S;
1068         else
1069             if isfield(model,'A')
1070                 model = rmfield(model,'A');
1071             end
1072         end
1073         BiomarkerRxns = {
1074             'EX_arg_L[u]'    'Increased (urine)'
1075             'DM_gln_L[bc]'    'Decreased (blood)'
1076             'EX_glu_L[u]'    'Decreased (blood)'
1077             'EX_lys_L[u]'    'Decreased (urine/blood)'
1078             'DM_lys_L[bc]'    'Decreased (urine/blood)'
1079             'EX_orn[u]'    'Increased (urine/blood/CSF)'
1080             'DM_orn[bc]'    'Increased (urine/blood/CSF)'
1081             'DM_orn[csf]'    'Increased (CSF)'
1082             };
1083     else
1084         BiomarkerRxns = {
1085             'EX_arg_L[u]'    'Increased'
1086             'EX_gln_L[u]'    'Decreased (blood)'
1087             'EX_glu_L[u]'    'Decreased (blood)'
1088             'EX_lys_L[u]'    'Decreased (urine/blood)'
1089             'EX_orn[u]'    'Increased (urine/blood/CSF)'
1090             };
1091     end
1092     [IEMSol_GACR] = checkIEM_WBM(model,IEMRxns, BiomarkerRxns,minRxnsFluxHealthy);
1093     
1094     %% '229.1' HFI Hereditary Fructose Intolerance
1095     if 0
1096         model = modelO;
1097         
1098         R = {'_FBA5'};
1099         RxnsAll2 = '';
1100         for i = 1: length(R)
1101             RxnsAll = model.rxns(find(~cellfun(@isempty,strfind(model.rxns,R{i}))));
1102             RxnsAll2 =[RxnsAll2;RxnsAll];
1103         end
1104         IEMRxns = unique(RxnsAll2);
1105         RxnMic = model.rxns(find(~cellfun(@isempty,strfind(model.rxns,'Micro_')))) ;
1106         IEMRxns = setdiff(IEMRxns,RxnMic);
1107         
1108         if ~strcmp(modelName,'Recon3D')
1109             % add demand reactions to blood compartment for those biomarkers reported for blood
1110             model = addDemandReaction(model, 'fru[bc]');
1111             if useSolveCobraLPCPLEX
1112                 model.A = model.S;
1113             else
1114                 if isfield(model,'A')
1115                     model = rmfield(model,'A');
1116                 end
1117             end
1118             BiomarkerRxns = {
1119                 'EX_fru[u]'    'Increased (urine/blood)'
1120                 'DM_fru[bc]'    'Increased (urine/blood)'
1121                 };
1122         else
1123             BiomarkerRxns = {
1124                 'EX_fru[u]'    'Increased (urine/blood)'
1125                 };
1126         end
1127         [IEMSol_HFI] = checkIEM_WBM(model,IEMRxns, BiomarkerRxns,minRxnsFluxHealthy);
1128     end
1129     %% '3155.1 'HMG Hmg-Coa Lyase Deficiency
1130     model = modelO;
1131     
1132     R = {'_HMGLx'};
1133     RxnsAll2 = '';
1134     for i = 1: length(R)
1135         RxnsAll = model.rxns(find(~cellfun(@isempty,strfind(model.rxns,R{i}))));
1136         RxnsAll2 =[RxnsAll2;RxnsAll];
1137     end
1138     IEMRxns = unique(RxnsAll2);
1139     RxnMic = model.rxns(find(~cellfun(@isempty,strfind(model.rxns,'Micro_')))) ;;
1140     IEMRxns = setdiff(IEMRxns,RxnMic);
1141     if ~strcmp(modelName,'Recon3D')
1142         % add demand reactions to blood compartment for those biomarkers reported for blood
1143         model = addDemandReaction(model, 'c6dc[bc]');
1144         model = addDemandReaction(model, 'CE5068[bc]');
1145         if useSolveCobraLPCPLEX
1146             model.A = model.S;
1147         else
1148             if isfield(model,'A')
1149                 model = rmfield(model,'A');
1150             end
1151         end
1152         BiomarkerRxns = {
1153             'DM_c6dc[bc]'    'Increased (blood)'
1154             'EX_adpac[u]'    'Increased (urine)'
1155             };
1156     else
1157         BiomarkerRxns = {
1158             'EX_c6dc[u]'    'Increased (blood)'
1159             'EX_adpac[u]'    'Increased (urine)'
1160             };
1161     end
1162     [IEMSol_HMG] = checkIEM_WBM(model,IEMRxns, BiomarkerRxns,minRxnsFluxHealthy);
1163     
1164     %% '875.1 HCYS Homocystinuria
1165     % https://www.ncbi.nlm.nih.gov/pmc/articles/PMC5203861/: Patients with CBS deficiency have low to low normal cystathionine (reference range typically between 0.05-0.08 and 0.35-0.5 ?mol/L) and high to high normal methionine concentrations (reference range typically between 12-15 and 40-45 ?mol/L) with a grossly abnormal ratio of these two metabolites.
1166     % https://www.ncbi.nlm.nih.gov/pmc/articles/PMC5203861/: The major confounder that may mask the biochemical hallmarks of CBS deficiency is the intake of pyridoxine. Decreases in the tHcy concentration occur after pharmacological doses of pyridoxine in a substantial proportion of CBS deficient patients (Mudd et al 1985; Wilcken and Wilcken 1997; Magner et al 2011). In pyridoxine-responsive patients with some specific mutations (e.g. p.P49L), physiological doses of pyridoxine as low as 2 mg per day in an adult may decrease the tHcy concentrations into the reference range (Stabler et al 2013). Since pyridoxine is contained in many vitamin supplements as well as in fortified foods and drinks, it is important to avoid intake of any pyridoxine supplements for at least 2 weeks before sampling plasma for tHcy measurement, although occasionally a wash-out period of up to 1-2 months may be needed (Orendac et al 2003; Stabler et al 2013).
1167     
1168     model = modelO;
1169     
1170     R = {'_CYSTS';'_SELCYSTS'};
1171     RxnsAll2 = '';
1172     for i = 1: length(R)
1173         RxnsAll = model.rxns(find(~cellfun(@isempty,strfind(model.rxns,R{i}))));
1174         RxnsAll2 =[RxnsAll2;RxnsAll];
1175     end
1176     IEMRxns = unique(RxnsAll2);
1177     RxnMic = model.rxns(find(~cellfun(@isempty,strfind(model.rxns,'Micro_')))) ;
1178     IEMRxns = setdiff(IEMRxns,RxnMic);
1179     if ~strcmp(modelName,'Recon3D')
1180         % add demand reactions to blood compartment for those biomarkers reported for blood
1181         model = addDemandReaction(model, 'met_L[bc]');
1182         model = addDemandReaction(model, 'orn[bc]');
1183         model = addDemandReaction(model, 'Lhcystin[bc]');
1184         model = addDemandReaction(model, 'hcys_L[bc]');
1185         if useSolveCobraLPCPLEX
1186             model.A = model.S;
1187         else
1188             if isfield(model,'A')
1189                 model = rmfield(model,'A');
1190             end
1191         end
1192         BiomarkerRxns = {
1193             'DM_met_L[bc]'    'Increased (blood)'%
1194             'DM_orn[bc]'    'Increased (blood)' % % not mentioned in 27778219
1195             'EX_Lhcystin[u]'    'Increased (urine/blood)' % difficult to detect - only in higher concentrations
1196             'DM_Lhcystin[bc]'    'Increased (urine/blood)' % difficult to detect - only in higher concentrations
1197             'EX_hcys_L[u]'    'Increased (urine/blood)' % only total hcys (not necessarily free hcys) is Increased
1198             'DM_hcys_L[bc]'    'Increased (urine/blood)' % only total hcys (not necessarily free hcys) is Increased
1199             'EX_cyst_L[u]'    'Decreased (urine)' % PMID: 27778219
1200             };
1201     else
1202         BiomarkerRxns = {
1203             'EX_met_L[u]'    'Increased (blood)'%
1204             'EX_orn[u]'    'Increased (blood)' % not mentioned in 27778219
1205             'EX_Lhcystin[u]'    'Increased (urine/blood)' % difficult to detect - only in higher concentrations
1206             'EX_hcys_L[u]'    'Increased (urine/blood)' % only total hcys (not necessarily free hcys) is Increased
1207             'EX_cyst_L[u]'    'Decreased (urine)' % PMID: 27778219
1208             };
1209     end
1210     [IEMSol_HCYS] = checkIEM_WBM(model,IEMRxns, BiomarkerRxns,minRxnsFluxHealthy);
1211     %% '10157.1 HLYS1 Hyperlysinemia I, Familial
1212     model = modelO;
1213     
1214     R = {'_SACCD3m';'_SACCD4m';'_r0525'};
1215     RxnsAll2 = '';
1216     for i = 1: length(R)
1217         RxnsAll = model.rxns(find(~cellfun(@isempty,strfind(model.rxns,R{i}))));
1218         RxnsAll2 =[RxnsAll2;RxnsAll];
1219     end
1220     IEMRxns = unique(RxnsAll2);
1221     RxnMic = model.rxns(find(~cellfun(@isempty,strfind(model.rxns,'Micro_')))) ;;
1222     IEMRxns = setdiff(IEMRxns,RxnMic);
1223     if ~strcmp(modelName,'Recon3D')
1224         % add demand reactions to blood compartment for those biomarkers reported for blood
1225         model = addDemandReaction(model, 'lys_L[bc]');
1226         model = addDemandReaction(model, 'Lpipecol[bc]');
1227         if useSolveCobraLPCPLEX
1228             model.A = model.S;
1229         else
1230             if isfield(model,'A')
1231                 model = rmfield(model,'A');
1232             end
1233         end
1234         BiomarkerRxns = {
1235             'EX_lys_L[u]'    'Increased (urine/blood)'
1236             'DM_lys_L[bc]'    'Increased (urine/blood)'
1237             'DM_Lpipecol[bc]'    'Increased (blood)'
1238             };
1239     else
1240         BiomarkerRxns = {
1241             'EX_lys_L[u]'    'Increased (urine/blood)'
1242             'EX_Lpipecol[u]'    'Increased (blood)'
1243             };
1244     end
1245     [IEMSol_HLYS1] = checkIEM_WBM(model,IEMRxns, BiomarkerRxns,minRxnsFluxHealthy);
1246     
1247     %% '10157.1 HLYS2 Hyperlysinemia II Or Saccharopinuria
1248     model = modelO;
1249     
1250     R = {'_SACCD3m';'_SACCD4m';'_r0525'};
1251     RxnsAll2 = '';
1252     for i = 1: length(R)
1253         RxnsAll = model.rxns(find(~cellfun(@isempty,strfind(model.rxns,R{i}))));
1254         RxnsAll2 =[RxnsAll2;RxnsAll];
1255     end
1256     IEMRxns = unique(RxnsAll2);
1257     RxnMic = model.rxns(find(~cellfun(@isempty,strfind(model.rxns,'Micro_')))) ;;
1258     IEMRxns = setdiff(IEMRxns,RxnMic);
1259     if ~strcmp(modelName,'Recon3D')
1260         % add demand reactions to blood compartment for those biomarkers reported for blood
1261         model = addDemandReaction(model, 'lys_L[bc]');
1262         model = addDemandReaction(model, 'citr_L[bc]');
1263         model = addDemandReaction(model, 'saccrp_L[bc]');
1264         if useSolveCobraLPCPLEX
1265             model.A = model.S;
1266         else
1267             if isfield(model,'A')
1268                 model = rmfield(model,'A');
1269             end
1270         end
1271         BiomarkerRxns = {
1272             'EX_citr_L[u]'    'Increased (urine/blood)'
1273             'EX_lys_L[u]'    'Increased (urine/blood)'
1274             'DM_citr_L[bc]'    'Increased (urine/blood)'
1275             'DM_lys_L[bc]'    'Increased (urine/blood)'
1276             };
1277     else
1278         
1279         BiomarkerRxns = {
1280             'EX_citr_L[u]'    'Increased (urine/blood)'
1281             'EX_lys_L[u]'    'Increased (urine/blood)'
1282             };
1283     end
1284     [IEMSol_HLYS2] = checkIEM_WBM(model,IEMRxns, BiomarkerRxns,minRxnsFluxHealthy);
1285     
1286     %% '5625.1 HYPRO1 Hyperprolinemia Type I
1287     model = modelO;
1288     
1289     R = {'_r1453';'_PROD2m';'_PRO1xm'};
1290     RxnsAll2 = '';
1291     for i = 1: length(R)
1292         RxnsAll = model.rxns(find(~cellfun(@isempty,strfind(model.rxns,R{i}))));
1293         RxnsAll2 =[RxnsAll2;RxnsAll];
1294     end
1295     IEMRxns = unique(RxnsAll2);
1296     RxnMic = model.rxns(find(~cellfun(@isempty,strfind(model.rxns,'Micro_')))) ;;
1297     IEMRxns = setdiff(IEMRxns,RxnMic);
1298     
1299     if ~strcmp(modelName,'Recon3D')
1300         % add demand reactions to blood compartment for those biomarkers reported for blood
1301         model = addDemandReaction(model, 'pro_L[bc]');
1302         if useSolveCobraLPCPLEX
1303             model.A = model.S;
1304         else
1305             if isfield(model,'A')
1306                 model = rmfield(model,'A');
1307             end
1308         end
1309         BiomarkerRxns = {
1310             'EX_4hpro_LT[u]'    'Increased (urine)'
1311             'EX_gly[u]'    'Increased (urine)'
1312             'EX_pro_L[u]'    'Increased (urine/blood)'
1313             'DM_pro_L[bc]'    'Increased (urine/blood)'
1314             };
1315     else
1316         BiomarkerRxns = {
1317             'EX_4hpro_LT[u]'    'Increased (urine)'
1318             'EX_gly[u]'    'Increased (urine)'
1319             'EX_pro_L[u]'    'Increased (urine/blood)'
1320             };
1321     end
1322     
1323     [IEMSol_HYPRO1] = checkIEM_WBM(model,IEMRxns, BiomarkerRxns,minRxnsFluxHealthy);
1324     
1325     %% '8659.1 HPII Hyperprolinemia Type Ii
1326     model = modelO;
1327     
1328     R = {'_P5CDm';'_PHCDm';'_r0686';'_4HGLSDm';'_r0074'};
1329     RxnsAll2 = '';
1330     for i = 1: length(R)
1331         RxnsAll = model.rxns(find(~cellfun(@isempty,strfind(model.rxns,R{i}))));
1332         RxnsAll2 =[RxnsAll2;RxnsAll];
1333     end
1334     IEMRxns = unique(RxnsAll2);
1335     RxnMic = model.rxns(find(~cellfun(@isempty,strfind(model.rxns,'Micro_')))) ;;
1336     IEMRxns = setdiff(IEMRxns,RxnMic);
1337     
1338     if ~strcmp(modelName,'Recon3D')
1339         % add demand reactions to blood compartment for those biomarkers reported for blood
1340         model = addDemandReaction(model, 'pro_L[bc]');
1341         model = addDemandReaction(model, 'orn[bc]');
1342         if useSolveCobraLPCPLEX
1343             model.A = model.S;
1344         else
1345             if isfield(model,'A')
1346                 model = rmfield(model,'A');
1347             end
1348         end
1349         BiomarkerRxns = {
1350             'EX_4hpro_LT[u]'    'Increased (urine)'
1351             'EX_gly[u]'    'Increased (urine)'
1352             'EX_orn[u]'    'Increased (urine/blood)'
1353             'EX_pro_L[u]'    'Increased (urine/blood)'
1354             'DM_orn[bc]'    'Increased (urine/blood)'
1355             'DM_pro_L[bc]'    'Increased (urine/blood)'
1356             };
1357     else
1358         BiomarkerRxns = {
1359             'EX_4hpro_LT[u]'    'Increased'
1360             'EX_gly[u]'    'Increased (urine)'
1361             'EX_orn[u]'    'Increased (urine/blood)'
1362             'EX_pro_L[u]'    'Increased (urine/blood)'
1363             };
1364     end
1365     [IEMSol_HPII] = checkIEM_WBM(model,IEMRxns, BiomarkerRxns,minRxnsFluxHealthy);
1366     
1367     %% '3712.1 IVA Isovaleric Acidemia
1368     model = modelO;
1369     
1370     R = {'_ACOAD8m'};
1371     RxnsAll2 = '';
1372     for i = 1: length(R)
1373         RxnsAll = model.rxns(find(~cellfun(@isempty,strfind(model.rxns,R{i}))));
1374         RxnsAll2 =[RxnsAll2;RxnsAll];
1375     end
1376     IEMRxns = unique(RxnsAll2);
1377     RxnMic = model.rxns(find(~cellfun(@isempty,strfind(model.rxns,'Micro_')))) ;;
1378     IEMRxns = setdiff(IEMRxns,RxnMic);
1379     model.lb(find(ismember(model.rxns,IEMRxns))) = 0;
1380     
1381     if ~strcmp(modelName,'Recon3D')
1382         % add demand reactions to blood compartment for those biomarkers reported for blood
1383         model = addDemandReaction(model, 'ivcrn[bc]');
1384         if useSolveCobraLPCPLEX
1385             model.A = model.S;
1386         else
1387             if isfield(model,'A')
1388                 model = rmfield(model,'A');
1389             end
1390         end
1391         BiomarkerRxns = {
1392             'EX_3bcrn[u]'    'Increased (urine)'%
1393             'EX_3ivcrn[u]'    'Increased (urine)'
1394             'DM_ivcrn[bc]'    'Increased (blood)'
1395             };
1396     else
1397         
1398         BiomarkerRxns = {
1399             'EX_3bcrn[u]'    'Increased (urine)'
1400             'EX_3ivcrn[u]'    'Increased (urine)'
1401             'EX_ivcrn[u]'    'Increased (blood)'
1402             };
1403     end
1404     [IEMSol_IVA] = checkIEM_WBM(model,IEMRxns, BiomarkerRxns,minRxnsFluxHealthy);
1405     
1406     %% '3251.1 LNS Lesch-Nyhan Syndrome
1407     model = modelO;
1408     
1409     R = {'_GUAPRT';'_HXPRT'};
1410     RxnsAll2 = '';
1411     for i = 1: length(R)
1412         RxnsAll = model.rxns(find(~cellfun(@isempty,strfind(model.rxns,R{i}))));
1413         RxnsAll2 =[RxnsAll2;RxnsAll];
1414     end
1415     IEMRxns = unique(RxnsAll2);
1416     RxnMic = model.rxns(find(~cellfun(@isempty,strfind(model.rxns,'Micro_')))) ;
1417     IEMRxns = setdiff(IEMRxns,RxnMic);
1418     
1419     if ~strcmp(modelName,'Recon3D')
1420         % add demand reactions to blood compartment for those biomarkers reported for blood
1421         model = addDemandReaction(model, 'fol[bc]');
1422         model = addDemandReaction(model, 'urate[bc]');
1423         if useSolveCobraLPCPLEX
1424             model.A = model.S;
1425         else
1426             if isfield(model,'A')
1427                 model = rmfield(model,'A');
1428             end
1429         end
1430         BiomarkerRxns = {
1431             'DM_fol[bc]'    'Decreased (blood)'
1432             'EX_urate[u]'    'Increased (urine/blood)'
1433             'DM_urate[bc]'    'Increased (urine/blood)'
1434             };
1435     else
1436         BiomarkerRxns = {
1437             'EX_fol[u]'    'Decreased (blood)'
1438             'EX_urate[u]'    'Increased (urine/blood)'
1439             };
1440     end
1441     [IEMSol_LNS] = checkIEM_WBM(model,IEMRxns, BiomarkerRxns,minRxnsFluxHealthy);
1442     
1443     %% '4056.1 LTC4S Leukotriene C4 Synthase Deficiency (Ltc4 Synthase Deficiency)
1444     model = modelO;
1445     
1446     R = {'_HMR_1081';'_LTC4Sr'}; % there is another reaction in [r] that has a more complex GPR
1447     RxnsAll2 = '';
1448     for i = 1: length(R)
1449         RxnsAll = model.rxns(find(~cellfun(@isempty,strfind(model.rxns,R{i}))));
1450         RxnsAll2 =[RxnsAll2;RxnsAll];
1451     end
1452     IEMRxns = unique(RxnsAll2);
1453     RxnMic = model.rxns(find(~cellfun(@isempty,strfind(model.rxns,'Micro_')))) ;;
1454     IEMRxns = setdiff(IEMRxns,RxnMic);
1455     
1456     if ~strcmp(modelName,'Recon3D')
1457         % add demand reactions to blood compartment for those biomarkers reported for blood
1458         model = addDemandReaction(model, 'leuktrE4[bc]');
1459         model = addDemandReaction(model, 'leuktrC4[bc]');
1460         model = addDemandReaction(model, 'leuktrD4[bc]');
1461         model = addDemandReaction(model, 'leuktrE4[csf]');
1462         model = addDemandReaction(model, 'leuktrC4[csf]');
1463         model = addDemandReaction(model, 'leuktrD4[csf]');
1464         if useSolveCobraLPCPLEX
1465             model.A = model.S;
1466         else
1467             if isfield(model,'A')
1468                 model = rmfield(model,'A');
1469             end
1470         end
1471         BiomarkerRxns = {
1472             'EX_leuktrE4[u]'    'Decreased (urine/blood/CSF)'
1473             'EX_leuktrC4[u]'    'Decreased (urine/blood/CSF)'
1474             'DM_leuktrE4[bc]'    'Decreased (urine/blood/CSF)'
1475             'DM_leuktrC4[bc]'    'Decreased (urine/blood/CSF)'
1476             'DM_leuktrD4[bc]'    'Decreased (urine/blood/CSF)'
1477             'DM_leuktrE4[csf]'    'Decreased (urine/blood/CSF)'
1478             };
1479     else
1480         BiomarkerRxns = {
1481             'EX_leuktrE4[u]'    'Decreased (urine/blood/CSF)'
1482             'EX_leuktrC4[u]'    'Decreased (urine/blood/CSF)'
1483             'EX_leuktrD4[u]'    'Decreased (urine/blood/CSF)'
1484             };
1485     end
1486     
1487     [IEMSol_LTC4S] = checkIEM_WBM(model,IEMRxns, BiomarkerRxns,minRxnsFluxHealthy);
1488     
1489     %% '593.1 MSUD Maple Syrup Urine Disease
1490     model = modelO;
1491     
1492     R = {'_r0670';'_OIVD1m';'_OIVD2m';'_OIVD3m';'_r0385';'_r0386';'_r1154'};
1493     RxnsAll2 = '';
1494     for i = 1: length(R)
1495         RxnsAll = model.rxns(find(~cellfun(@isempty,strfind(model.rxns,R{i}))));
1496         RxnsAll2 =[RxnsAll2;RxnsAll];
1497     end
1498     IEMRxns = unique(RxnsAll2);
1499     RxnMic = model.rxns(find(~cellfun(@isempty,strfind(model.rxns,'Micro_')))) ;;
1500     IEMRxns = setdiff(IEMRxns,RxnMic);
1501     
1502     if ~strcmp(modelName,'Recon3D')
1503         % add demand reactions to blood compartment for those biomarkers reported for blood
1504         model = addDemandReaction(model, 'ile_L[bc]');
1505         model = addDemandReaction(model, 'leu_L[bc]');
1506         model = addDemandReaction(model, 'val_L[bc]');
1507         model = addDemandReaction(model, '3mop[bc]');
1508         if useSolveCobraLPCPLEX
1509             model.A = model.S;
1510         else
1511             if isfield(model,'A')
1512                 model = rmfield(model,'A');
1513             end
1514         end
1515         BiomarkerRxns = {
1516             'EX_ile_L[u]'    'Increased (urine/blood)'
1517             'EX_3mop[u]'    'Increased (urine/blood)'
1518             'DM_ile_L[bc]'    'Increased (urine/blood)'
1519             'DM_leu_L[bc]'    'Increased (blood)'
1520             'DM_val_L[bc]'    'Increased (blood)'
1521             'DM_3mop[bc]'    'Increased (urine/blood)'
1522             };
1523     else
1524         BiomarkerRxns = {
1525             'EX_ile_L[u]'    'Increased (urine/blood)'
1526             'EX_leu_L[u]'    'Increased (blood)'
1527             'EX_val_L[u]'    'Increased (blood)'
1528             'EX_3mop[u]'    'Increased (urine/blood)'
1529             };
1530     end
1531     [IEMSol_MSUD] = checkIEM_WBM(model,IEMRxns, BiomarkerRxns,minRxnsFluxHealthy);
1532     
1533     %% '4594.1 MMA Methylmalonic Acidemia (Mma)
1534     if 1
1535         model = modelO;
1536         
1537         R = {'_MMMm'};
1538         RxnsAll2 = '';
1539         for i = 1: length(R)
1540             RxnsAll = model.rxns(find(~cellfun(@isempty,strfind(model.rxns,R{i}))));
1541             RxnsAll2 =[RxnsAll2;RxnsAll];
1542         end
1543         IEMRxns = unique(RxnsAll2);
1544         RxnMic = model.rxns(find(~cellfun(@isempty,strfind(model.rxns,'Micro_')))) ;;
1545         IEMRxns = setdiff(IEMRxns,RxnMic);
1546         if ~strcmp(modelName,'Recon3D')
1547             % add demand reactions to blood compartment for those biomarkers reported for blood
1548             model = addDemandReaction(model, 'c4dc[bc]');
1549             model = addDemandReaction(model, 'crn[bc]');
1550             model = addDemandReaction(model, 'HC00900[bc]');
1551             model = addDemandReaction(model, '3hpp[bc]');
1552             model = addDemandReaction(model, 'pcrn[bc]');
1553             model = addDemandReaction(model, '3hdececrn[bc]');
1554             if useSolveCobraLPCPLEX
1555                 model.A = model.S;
1556             else
1557                 if isfield(model,'A')
1558                     model = rmfield(model,'A');
1559                 end
1560             end
1561             BiomarkerRxns = {
1562                 'EX_3aib[u]'    'Increased (urine)' %
1563                 'DM_c4dc[bc]'    'Increased (blood)' %
1564                 'DM_crn[bc]'    'Decreased (blood)' %
1565                 'DM_HC00900[bc]'    'Increased (blood)' %
1566                 'DM_3hpp[bc]'    'Increased (blood)' %
1567                 'DM_pcrn[bc]'    'Increased (blood)'
1568                 'DM_3hdececrn[bc]'    'Increased (blood)'
1569                 };
1570         else
1571             BiomarkerRxns = {
1572                 'EX_3aib[u]'    'Increased (urine)' %
1573                 'EX_c4dc[u]'    'Increased (blood)' %
1574                 'EX_crn[u]'    'Decreased (blood)' %
1575                 'EX_HC00900[u]'    'Increased (blood)' %
1576                 'EX_3hpp[u]'    'Increased (blood)' %
1577                 'EX_pcrn[u]'    'Increased (blood)'
1578                 'EX_3hdececrn[u]'    'Increased (blood)'
1579                 
1580                 };
1581         end
1582         [IEMSol_MMA] = checkIEM_WBM(model,IEMRxns, BiomarkerRxns,minRxnsFluxHealthy);
1583     end
1584     %% '162417.1 NAGS N-Acetylglutamate Synthase Deficiency
1585     model = modelO;
1586     
1587     R = {'_RE2030M';'_RE2031M';'_RE2032M';'_RE2156M';'_RE2223M';'_ACGSm'};
1588     RxnsAll2 = '';
1589     for i = 1: length(R)
1590         RxnsAll = model.rxns(find(~cellfun(@isempty,strfind(model.rxns,R{i}))));
1591         RxnsAll2 =[RxnsAll2;RxnsAll];
1592     end
1593     IEMRxns = unique(RxnsAll2);
1594     RxnMic = model.rxns(find(~cellfun(@isempty,strfind(model.rxns,'Micro_')))) ;;
1595     IEMRxns = setdiff(IEMRxns,RxnMic);
1596     if ~strcmp(modelName,'Recon3D')
1597         % add demand reactions to blood compartment for those biomarkers reported for blood
1598         model = addDemandReaction(model, 'ala_L[bc]');
1599         model = addDemandReaction(model, 'citr_L[bc]');
1600         model = addDemandReaction(model, 'gln_L[bc]');
1601         model = addDemandReaction(model, 'nh4[bc]');
1602         model = addDemandReaction(model, 'orn[bc]');
1603         if useSolveCobraLPCPLEX
1604             model.A = model.S;
1605         else
1606             if isfield(model,'A')
1607                 model = rmfield(model,'A');
1608             end
1609         end
1610         BiomarkerRxns = {
1611             'DM_ala_L[bc]'    'Increased (blood)'
1612             'DM_citr_L[bc]'    'Decreased (blood)'
1613             'DM_gln_L[bc]'    'Increased (blood)'
1614             'DM_nh4[bc]'    'Increased (blood)'
1615             'DM_orn[bc]'    'Increased (blood)'
1616             'EX_orot[u]'    'Decreased (urine)'
1617             };
1618     else
1619         BiomarkerRxns = {
1620             'EX_ala_L[u]'    'Increased (blood)'
1621             'EX_citr_L[u]'    'Decreased (blood)'
1622             'EX_gln_L[u]'    'Increased (blood)'
1623             'EX_nh4[u]'    'Increased (blood)'
1624             'EX_orn[u]'    'Increased (blood)'
1625             'EX_orot[u]'    'Decreased (urine)'
1626             };
1627     end
1628     [IEMSol_NAGS] = checkIEM_WBM(model,IEMRxns, BiomarkerRxns,minRxnsFluxHealthy);
1629     %% '5009.1 OTC Ornithine Transcarbamylase Deficiency
1630     model = modelO;
1631     
1632     R = {'_OCBTm'};
1633     RxnsAll2 = '';
1634     for i = 1: length(R)
1635         RxnsAll = model.rxns(find(~cellfun(@isempty,strfind(model.rxns,R{i}))));
1636         RxnsAll2 =[RxnsAll2;RxnsAll];
1637     end
1638     IEMRxns = unique(RxnsAll2);
1639     RxnMic = model.rxns(find(~cellfun(@isempty,strfind(model.rxns,'Micro_')))) ;;
1640     IEMRxns = setdiff(IEMRxns,RxnMic);
1641     if ~strcmp(modelName,'Recon3D')
1642         % add demand reactions to blood compartment for those biomarkers reported for blood
1643         model = addDemandReaction(model, 'ura[bc]');
1644         model = addDemandReaction(model, 'citr_L[bc]');
1645         model = addDemandReaction(model, 'gln_L[bc]');
1646         model = addDemandReaction(model, 'nh4[bc]');
1647         model = addDemandReaction(model, 'orn[bc]');
1648         if useSolveCobraLPCPLEX
1649             model.A = model.S;
1650         else
1651             if isfield(model,'A')
1652                 model = rmfield(model,'A');
1653             end
1654         end
1655         BiomarkerRxns = {
1656             'EX_5oxpro[u]'    'Increased (transient, urine)'
1657             'EX_gly[u]'    'Increased (urine)'
1658             'EX_lys_L[u]'    'Increased (urine)'
1659             'EX_orot[u]'    'Increased (urine)'
1660             'DM_citr_L[bc]'    'Decreased (blood)'
1661             'DM_gln_L[bc]'    'Increased (blood)'
1662             'DM_ura[bc]'    'Increased (urine)'
1663             'DM_nh4[bc]'    'Increased (blood)'
1664             'DM_orn[bc]'    'Increased (blood)'
1665             };
1666     else
1667         BiomarkerRxns = {
1668             'EX_5oxpro[u]'    'Increased (transient, urine)'
1669             'EX_citr_L[u]'    'Decreased (blood)'
1670             'EX_gln_L[u]'    'Increased (blood)'
1671             'EX_gly[u]'    'Increased (urine)'
1672             'EX_lys_L[u]'    'Increased (urine)'
1673             'EX_orot[u]'    'Increased (urine)'
1674             'EX_ura[u]'    'Increased (urine)'
1675             'EX_nh4[u]'    'Increased (blood)'
1676             'EX_orn[u]'    'Increased (blood)'
1677             };
1678     end
1679     [IEMSol_OTC] = checkIEM_WBM(model,IEMRxns, BiomarkerRxns,minRxnsFluxHealthy);
1680     
1681     %% '7372.1 OROA Orotic Aciduria
1682     if 0
1683         model = modelO;
1684         
1685         R = {'_ORPT';'_OMPDC'};
1686         RxnsAll2 = '';
1687         for i = 1: length(R)
1688             RxnsAll = model.rxns(find(~cellfun(@isempty,strfind(model.rxns,R{i}))));
1689             RxnsAll2 =[RxnsAll2;RxnsAll];
1690         end
1691         IEMRxns = unique(RxnsAll2);
1692         RxnMic = model.rxns(find(~cellfun(@isempty,strfind(model.rxns,'Micro_')))) ;;
1693         IEMRxns = setdiff(IEMRxns,RxnMic);
1694         
1695         if ~strcmp(modelName,'Recon3D')
1696             % add demand reactions to blood compartment for those biomarkers reported for blood
1697             model = addDemandReaction(model, 'orot[bc]');
1698             if useSolveCobraLPCPLEX
1699                 model.A = model.S;
1700             else
1701                 if isfield(model,'A')
1702                     model = rmfield(model,'A');
1703                 end
1704             end
1705             BiomarkerRxns = {
1706                 'EX_orot[u]'    'Increased (urine/blood)'
1707                 'DM_orot[bc]'    'Increased (urine/blood)'
1708                 };
1709         else
1710             BiomarkerRxns = {
1711                 'EX_orot[u]'    'Increased (urine/blood)'
1712                 };
1713         end
1714         [IEMSol_OROA] = checkIEM_WBM(model,IEMRxns, BiomarkerRxns,minRxnsFluxHealthy);
1715     end
1716     %% '5053.1 PKU Phenylketonuria
1717     model = modelO;
1718     
1719     R = {'_PHETHPTOX2';'_r0399'};
1720     RxnsAll2 = '';
1721     for i = 1: length(R)
1722         RxnsAll = model.rxns(find(~cellfun(@isempty,strfind(model.rxns,R{i}))));
1723         RxnsAll2 =[RxnsAll2;RxnsAll];
1724     end
1725     IEMRxns = unique(RxnsAll2);
1726     RxnMic = model.rxns(find(~cellfun(@isempty,strfind(model.rxns,'Micro_')))) ;;
1727     IEMRxns = setdiff(IEMRxns,RxnMic);
1728     
1729     if ~strcmp(modelName,'Recon3D')
1730         % add demand reactions to blood compartment for those biomarkers reported for blood
1731         model = addDemandReaction(model, 'phe_L[bc]');
1732         if useSolveCobraLPCPLEX
1733             model.A = model.S;
1734         else
1735             if isfield(model,'A')
1736                 model = rmfield(model,'A');
1737             end
1738         end
1739         BiomarkerRxns = {
1740             'DM_phe_L[bc]'    'Increased (blood)'
1741             'EX_2hyoxplac[u]'    'Increased (urine)'
1742             'EX_phpyr[u]'    'Increased (urine)'
1743             };
1744     else
1745         BiomarkerRxns = {
1746             'EX_phe_L[u]'    'Increased (blood)'
1747             'EX_2hyoxplac[u]'    'Increased (urine)'
1748             'EX_phpyr[u]'    'Increased (urine)'
1749             };
1750     end
1751     [IEMSol_PKU] = checkIEM_WBM(model,IEMRxns, BiomarkerRxns,minRxnsFluxHealthy);
1752     
1753     %% '1890.1 MNGIE Mitochondrial Neurogastrointestinal Encephalopathy (Mngie) Disease
1754     model = modelO;
1755     if 0
1756         R = {'_TMDPP'};
1757         RxnsAll2 = '';
1758         for i = 1: length(R)
1759             RxnsAll = model.rxns(find(~cellfun(@isempty,strfind(model.rxns,R{i}))));
1760             RxnsAll2 =[RxnsAll2;RxnsAll];
1761         end
1762         IEMRxns = unique(RxnsAll2);
1763         RxnMic = model.rxns(find(~cellfun(@isempty,strfind(model.rxns,'Micro_')))) ;;
1764         IEMRxns = setdiff(IEMRxns,RxnMic);
1765         
1766         if ~strcmp(modelName,'Recon3D')
1767             % add demand reactions to blood compartment for those biomarkers reported for blood
1768             model = addDemandReaction(model, 'duri[bc]');
1769             model = addDemandReaction(model, 'thymd[bc]');
1770             if useSolveCobraLPCPLEX
1771                 model.A = model.S;
1772             else
1773                 if isfield(model,'A')
1774                     model = rmfield(model,'A');
1775                 end
1776             end
1777             BiomarkerRxns = {
1778                 'DM_duri[bc]'    'Increased (blood)'
1779                 'DM_thymd[bc]'    'Increased (blood)'
1780                 };
1781         else
1782             BiomarkerRxns = {
1783                 'EX_duri[u]'    'Increased (blood)'
1784                 'EX_thymd[u]'    'Increased (blood)'
1785                 };
1786         end
1787         [IEMSol_MNGIE] = checkIEM_WBM(model,IEMRxns, BiomarkerRxns,minRxnsFluxHealthy);
1788     end
1789     %% '8803.1 SUCLA Succinate-Coenzyme A (Coa) Ligase Deficiency/Lactic Acidosis, Fatal Infantile
1790     model = modelO;
1791     
1792     R = {'_ITCOALm';'_MECOALm';'_SUCOASm';'_ITCOAL1m';'_MECOAS1m';'_SUCOAS1m'};
1793     RxnsAll2 = '';
1794     for i = 1: length(R)
1795         RxnsAll = model.rxns(find(~cellfun(@isempty,strfind(model.rxns,R{i}))));
1796         RxnsAll2 =[RxnsAll2;RxnsAll];
1797     end
1798     IEMRxns = unique(RxnsAll2);
1799     RxnMic = model.rxns(find(~cellfun(@isempty,strfind(model.rxns,'Micro_')))) ;;
1800     IEMRxns = setdiff(IEMRxns,RxnMic);
1801     
1802     if ~strcmp(modelName,'Recon3D')
1803         % add demand reactions to blood compartment for those biomarkers reported for blood
1804         model = addDemandReaction(model, 'lac_L[bc]');
1805         model = addDemandReaction(model, 'pyr[bc]');
1806         if useSolveCobraLPCPLEX
1807             model.A = model.S;
1808         else
1809             if isfield(model,'A')
1810                 model = rmfield(model,'A');
1811             end
1812         end
1813         BiomarkerRxns = {
1814             'DM_lac_L[bc]'    'Increased (blood)'
1815             'DM_pyr[bc]'    'Increased (blood)'
1816             };
1817     else
1818         BiomarkerRxns = {
1819             'EX_lac_L[u]'    'Increased (blood)'
1820             'EX_pyr[u]'    'Increased (blood)'
1821             };
1822     end
1823     [IEMSol_SUCLA] = checkIEM_WBM(model,IEMRxns, BiomarkerRxns,minRxnsFluxHealthy);
1824     
1825     %% '7915.1 SSADHD Succinic Semialdehyde Dehydrogenase Deficiency
1826     model = modelO;
1827     
1828     R = {'_r0178'};
1829     RxnsAll2 = '';
1830     for i = 1: length(R)
1831         RxnsAll = model.rxns(find(~cellfun(@isempty,strfind(model.rxns,R{i}))));
1832         RxnsAll2 =[RxnsAll2;RxnsAll];
1833     end
1834     IEMRxns = unique(RxnsAll2);
1835     RxnMic = model.rxns(find(~cellfun(@isempty,strfind(model.rxns,'Micro_')))) ;;
1836     IEMRxns = setdiff(IEMRxns,RxnMic);
1837     
1838     if ~strcmp(modelName,'Recon3D')
1839         % add demand reactions to blood compartment for those biomarkers reported for blood
1840         model = addDemandReaction(model, 'gly[bc]');
1841         model = addDemandReaction(model, '4hdxbutn[bc]');
1842         model = addDemandReaction(model, 'sucsal[bc]');
1843         model = addDemandReaction(model, '4abut[csf]');
1844         if useSolveCobraLPCPLEX
1845             model.A = model.S;
1846         else
1847             if isfield(model,'A')
1848                 model = rmfield(model,'A');
1849             end
1850         end
1851         BiomarkerRxns = {
1852             'EX_gly[u]'    'Increased (urine/blood)'
1853             'EX_sucsal[u]'    'Increased (urine/blood)'
1854             'DM_gly[bc]'    'Increased (urine/blood)'
1855             'DM_sucsal[bc]'    'Increased (urine/blood)'
1856             'DM_4abut[csf]'    'Increased (CSF)'
1857             };
1858     else
1859         BiomarkerRxns = {
1860             'EX_gly[u]'    'Increased (urine/blood)'
1861             'EX_sucsal[u]'    'Increased (urine/blood)'
1862             'EX_4abut[u]'    'Increased (CSF)'
1863             };
1864     end
1865     [IEMSol_SSADHD] = checkIEM_WBM(model,IEMRxns, BiomarkerRxns,minRxnsFluxHealthy);
1866     
1867     %% '2643.1 TETB Tetrahydrobiopterin Deficiency
1868     %  is a naturally occurring essential cofactor of the three aromatic amino
1869     %  acid hydroxylase enzymes, used in the degradation of amino acid
1870     %  phenylalanine and in the biosynthesis of the neurotransmitters serotonin
1871     %  (5-hydroxytryptamine, 5-HT), melatonin, dopamine,...
1872     model = modelO;
1873     
1874     R = {'_GTPCIn';'_r0120';'_r0121';'_r0708';'_r0775';'_r0777';'_GTPCI';...
1875         '_TYR3MO2';'_PHETHPTOX2';'_Tetrahydrobiopterin'
1876         };
1877     RxnsAll2 = '';
1878     for i = 1: length(R)
1879         RxnsAll = model.rxns(find(~cellfun(@isempty,strfind(model.rxns,R{i}))));
1880         RxnsAll2 =[RxnsAll2;RxnsAll];
1881     end
1882     IEMRxns = unique(RxnsAll2);
1883     RxnMic = model.rxns(find(~cellfun(@isempty,strfind(model.rxns,'Micro_')))) ;;
1884     IEMRxns = setdiff(IEMRxns,RxnMic);
1885     if ~strcmp(modelName,'Recon3D')
1886         % add demand reactions to blood compartment for those biomarkers reported for blood
1887         model = addDemandReaction(model, 'phe_L[bc]');
1888         if useSolveCobraLPCPLEX
1889             model.A = model.S;
1890         else
1891             if isfield(model,'A')
1892                 model = rmfield(model,'A');
1893             end
1894         end
1895         BiomarkerRxns = {
1896             'DM_phe_L[bc]'    'Increased (blood)'
1897             };
1898     else
1899         BiomarkerRxns = {
1900             'EX_phe_L[u]'    'Increased (blood)'
1901             };
1902     end
1903     [IEMSol_TETB] = checkIEM_WBM(model,IEMRxns, BiomarkerRxns,minRxnsFluxHealthy);
1904     
1905     %% '445.1 CIT1 Type I Citrullinemia
1906     model = modelO;
1907     
1908     R = {'_ARGSS' };
1909     RxnsAll2 = '';
1910     for i = 1: length(R)
1911         RxnsAll = model.rxns(find(~cellfun(@isempty,strfind(model.rxns,R{i}))));
1912         RxnsAll2 =[RxnsAll2;RxnsAll];
1913     end
1914     IEMRxns = unique(RxnsAll2);
1915     RxnMic = model.rxns(find(~cellfun(@isempty,strfind(model.rxns,'Micro_')))) ;;
1916     IEMRxns = setdiff(IEMRxns,RxnMic);
1917     
1918     if ~strcmp(modelName,'Recon3D')
1919         % add demand reactions to blood compartment for those biomarkers reported for blood
1920         model = addDemandReaction(model, 'citr_L[bc]');
1921         model = addDemandReaction(model, 'gly[bc]');
1922         model = addDemandReaction(model, 'nh4[bc]');
1923         if useSolveCobraLPCPLEX
1924             model.A = model.S;
1925         else
1926             if isfield(model,'A')
1927                 model = rmfield(model,'A');
1928             end
1929         end
1930         BiomarkerRxns = {
1931             'DM_citr_L[bc]'    'Increased (urine/blood)'
1932             'DM_gly[bc]'    'Increased (urine/blood)'
1933             'DM_nh4[bc]'    'Increased (blood)'
1934             'EX_orot[u]'    'Increased (urine)'
1935             'EX_citr_L[u]'    'Increased (urine/blood)'
1936             'EX_gly[u]'    'Increased (urine/blood)'
1937             };
1938     else
1939         
1940         BiomarkerRxns = {
1941             'EX_citr_L[u]'    'Increased (urine/blood)'
1942             'EX_gly[u]'    'Increased (urine/blood)'
1943             'EX_nh4[u]'    'Increased (blood)'
1944             'EX_orot[u]'    'Increased (urine)'
1945             };
1946     end
1947     [IEMSol_CIT1] = checkIEM_WBM(model,IEMRxns, BiomarkerRxns,minRxnsFluxHealthy);
1948     
1949     %% '2184.1 TYR1 Tyrosinemia Type I
1950     model = modelO;
1951     
1952     R = {'_FUMAC' };
1953     RxnsAll2 = '';
1954     for i = 1: length(R)
1955         RxnsAll = model.rxns(find(~cellfun(@isempty,strfind(model.rxns,R{i}))));
1956         RxnsAll2 =[RxnsAll2;RxnsAll];
1957     end
1958     IEMRxns = unique(RxnsAll2);
1959     RxnMic = model.rxns(find(~cellfun(@isempty,strfind(model.rxns,'Micro_')))) ;;
1960     IEMRxns = setdiff(IEMRxns,RxnMic);
1961     
1962     if ~strcmp(modelName,'Recon3D')
1963         % add demand reactions to blood compartment for those biomarkers reported for blood
1964         model = addDemandReaction(model, 'met_L[bc]');
1965         model = addDemandReaction(model, 'tyr_L[bc]');
1966         if useSolveCobraLPCPLEX
1967             model.A = model.S;
1968         else
1969             if isfield(model,'A')
1970                 model = rmfield(model,'A');
1971             end
1972         end
1973         BiomarkerRxns = {
1974             'DM_met_L[bc]'    'Increased (blood)'
1975             'DM_tyr_L[bc]'    'Increased (blood)'
1976             'EX_34hpl[u]'    'Increased (urine)'
1977             'EX_34hpp[u]'    'Increased (urine)'
1978             };
1979     else
1980         BiomarkerRxns = {
1981             'EX_met_L[u]'    'Increased (blood)'
1982             'EX_tyr_L[u]'    'Increased (blood)'
1983             'EX_34hpl[u]'    'Increased (urine)'
1984             'EX_34hpp[u]'    'Increased (urine)'
1985             };
1986     end
1987     [IEMSol_TYR1] = checkIEM_WBM(model,IEMRxns, BiomarkerRxns,minRxnsFluxHealthy);
1988     
1989     %% '3242.1 TYR3 Tyrosinemia Type III
1990     model = modelO;
1991     
1992     R = {'_34HPPOR';'_PPOR' };
1993     RxnsAll2 = '';
1994     for i = 1: length(R)
1995         RxnsAll = model.rxns(find(~cellfun(@isempty,strfind(model.rxns,R{i}))));
1996         RxnsAll2 =[RxnsAll2;RxnsAll];
1997     end
1998     IEMRxns = unique(RxnsAll2);
1999     RxnMic = model.rxns(find(~cellfun(@isempty,strfind(model.rxns,'Micro_')))) ;;
2000     IEMRxns = setdiff(IEMRxns,RxnMic);
2001     if ~strcmp(modelName,'Recon3D')
2002         % add demand reactions to blood compartment for those biomarkers reported for blood
2003         model = addDemandReaction(model, 'tyr_L[bc]');
2004         if useSolveCobraLPCPLEX
2005             model.A = model.S;
2006         else
2007             if isfield(model,'A')
2008                 model = rmfield(model,'A');
2009             end
2010         end
2011         BiomarkerRxns = {
2012             'DM_tyr_L[bc]'    'Increased (blood)'
2013             'EX_34hpl[u]'    'Increased (urine)'
2014             'EX_34hpp[u]'    'Increased (urine)'
2015             };
2016     else
2017         BiomarkerRxns = {
2018             'EX_tyr_L[u]'    'Increased (blood)'
2019             'EX_34hpl[u]'    'Increased (urine)'
2020             'EX_34hpp[u]'    'Increased (urine)'
2021             };
2022     end
2023     [IEMSol_TYR3] = checkIEM_WBM(model,IEMRxns, BiomarkerRxns,minRxnsFluxHealthy);
2024     
2025     %% '7498.1 XAN1 Xanthinuria Type 1
2026     model = modelO;
2027     
2028     R = {'_r0395';'_XANDp';'_XAO2x';'_XAOx';'_r0394';'_r0502';'_r0504' };
2029     RxnsAll2 = '';
2030     for i = 1: length(R)
2031         RxnsAll = model.rxns(find(~cellfun(@isempty,strfind(model.rxns,R{i}))));
2032         RxnsAll2 =[RxnsAll2;RxnsAll];
2033     end
2034     IEMRxns = unique(RxnsAll2);
2035     RxnMic = model.rxns(find(~cellfun(@isempty,strfind(model.rxns,'Micro_')))) ;;
2036     IEMRxns = setdiff(IEMRxns,RxnMic);
2037     if ~strcmp(modelName,'Recon3D')
2038         % add demand reactions to blood compartment for those biomarkers reported for blood
2039         model = addDemandReaction(model, 'xan[bc]');
2040         model = addDemandReaction(model, 'urate[bc]');
2041         if useSolveCobraLPCPLEX
2042             model.A = model.S;
2043         else
2044             if isfield(model,'A')
2045                 model = rmfield(model,'A');
2046             end
2047         end
2048         BiomarkerRxns = {
2049             'EX_hxan[u]'    'Increased (urine)'
2050             'EX_xan[u]'    'Increased (blood/urine)'
2051             'EX_urate[u]'    'Decreased (blood/urine)'
2052             'DM_xan[bc]'    'Increased (blood/urine)'
2053             'DM_urate[bc]'    'Decreased (blood/urine)'
2054             };
2055     else
2056         BiomarkerRxns = {
2057             'EX_hxan[u]'    'Increased (urine)'
2058             'EX_xan[u]'    'Increased (blood/urine)'
2059             'EX_urate[u]'    'Decreased (blood/urine)'
2060             };
2061     end
2062     [IEMSol_XAN1] = checkIEM_WBM(model,IEMRxns, BiomarkerRxns,minRxnsFluxHealthy);
2063     
2064     %% '4967.1 AKGD Alpha-Ketoglutarate Dehydrogenase Deficiency
2065     model = modelO;
2066     
2067     R = {'_AKGDm';'_2OXOADOXm';'_r0163';'_r0384';'_r0451';'_r0620' };
2068     RxnsAll2 = '';
2069     for i = 1: length(R)
2070         RxnsAll = model.rxns(find(~cellfun(@isempty,strfind(model.rxns,R{i}))));
2071         RxnsAll2 =[RxnsAll2;RxnsAll];
2072     end
2073     IEMRxns = unique(RxnsAll2);
2074     RxnMic = model.rxns(find(~cellfun(@isempty,strfind(model.rxns,'Micro_')))) ;;
2075     IEMRxns = setdiff(IEMRxns,RxnMic);
2076     if ~strcmp(modelName,'Recon3D')
2077         % add demand reactions to blood compartment for those biomarkers reported for blood
2078         model = addDemandReaction(model, 'lac_L[bc]');
2079         model = addDemandReaction(model, 'glu_L[bc]');
2080         model = addDemandReaction(model, 'gln_L[bc]');
2081         if useSolveCobraLPCPLEX
2082             model.A = model.S;
2083         else
2084             if isfield(model,'A')
2085                 model = rmfield(model,'A');
2086             end
2087         end
2088         BiomarkerRxns = {
2089             'EX_akg[u]'    'Increased (urine)'
2090             'EX_lac_L[u]'    'Increased (blood/urine)'
2091             'DM_lac_L[bc]'    'Increased (blood/urine)'
2092             'DM_glu_L[bc]'    'Increased (blood)'
2093             'DM_gln_L[bc]'    'Increased (blood)'
2094             };
2095     else
2096         BiomarkerRxns = {
2097             'EX_akg[u]'    'Increased (urine)'
2098             'EX_lac_L[u]'    'Increased (blood/urine)'
2099             'EX_glu_L[u]'    'Increased (blood)'
2100             'EX_gln_L[u]'    'Increased (blood)'
2101             };
2102     end
2103     [IEMSol_AKGD] = checkIEM_WBM(model,IEMRxns, BiomarkerRxns,minRxnsFluxHealthy);
2104     
2105     %% 1181.1 EP Essential Pentosuria
2106     if 1
2107         model = modelO;
2108         
2109         R = {'_XYLUR'};
2110         RxnsAll2 = '';
2111         for i = 1: length(R)
2112             RxnsAll = model.rxns(find(~cellfun(@isempty,strfind(model.rxns,R{i}))));
2113             RxnsAll2 =[RxnsAll2;RxnsAll];
2114         end
2115         IEMRxns = unique(RxnsAll2);
2116         RxnMic = model.rxns(find(~cellfun(@isempty,strfind(model.rxns,'Micro_')))) ;;
2117         IEMRxns = setdiff(IEMRxns,RxnMic);
2118         
2119         model.lb(find(ismember(model.rxns,IEMRxns))) = 0;
2120         
2121         R2 = {'_r0784'};
2122         RxnsAll2 = '';
2123         for i = 1: length(R2)
2124             RxnsAll = model.rxns(find(~cellfun(@isempty,strfind(model.rxns,R2{i}))));
2125             RxnsAll2 =[RxnsAll2;RxnsAll];
2126         end
2127         X = unique(RxnsAll2);
2128         RxnMic = model.rxns(find(~cellfun(@isempty,strfind(model.rxns,'Micro_')))) ;;
2129         X = setdiff(X,RxnMic);
2130         model.lb(find(ismember(model.rxns,X))) = 0;
2131         model.ub(find(ismember(model.rxns,X))) = 0;
2132         
2133         if ~strcmp(modelName,'Recon3D')
2134             % add demand reactions to blood compartment for those biomarkers reported for blood
2135             
2136             if useSolveCobraLPCPLEX
2137                 model.A = model.S;
2138             else
2139                 if isfield(model,'A')
2140                     model = rmfield(model,'A');
2141                 end
2142             end
2143             BiomarkerRxns = {
2144                 'EX_xylu_L[u]'    'Increased (urine)'
2145                 };
2146         else
2147             BiomarkerRxns = {
2148                 'EX_xylu_L[u]'    'Increased (urine)'
2149                 };
2150         end
2151         [IEMSol_EP] = checkIEM_WBM(model,IEMRxns, BiomarkerRxns,minRxnsFluxHealthy);
2152     end
2153     
2154     %% 587.1 HYPVLI Hypervalinemia And Hyperleucine-Isoleucinemia
2155     if 1
2156         model = modelO;
2157         
2158         R = {'_ILETAm';'_LEUTAm';'_VALTAm'};
2159         RxnsAll2 = '';
2160         for i = 1: length(R)
2161             RxnsAll = model.rxns(find(~cellfun(@isempty,strfind(model.rxns,R{i}))));
2162             RxnsAll2 =[RxnsAll2;RxnsAll];
2163         end
2164         IEMRxns = unique(RxnsAll2);
2165         RxnMic = model.rxns(find(~cellfun(@isempty,strfind(model.rxns,'Micro_')))) ;;
2166         IEMRxns = setdiff(IEMRxns,RxnMic);
2167         
2168         if ~strcmp(modelName,'Recon3D')
2169             % add demand reactions to blood compartment for those biomarkers reported for blood
2170             model = addDemandReaction(model, 'val_L[bc]');
2171             model = addDemandReaction(model, 'leu_L[bc]');
2172             if useSolveCobraLPCPLEX
2173                 model.A = model.S;
2174             else
2175                 if isfield(model,'A')
2176                     model = rmfield(model,'A');
2177                 end
2178             end
2179             BiomarkerRxns = {
2180                 'DM_val_L[bc]'    'Increased (blood/urine)'
2181                 'EX_val_L[u]'    'Increased (blood/urine)'
2182                 'EX_leu_L[u]'    'Increased (blood/urine)'
2183                 };
2184         else
2185             BiomarkerRxns = {
2186                 'EX_val_L[u]'    'Increased (blood/urine)'
2187                 'EX_leu_L[u]'    'Increased (blood/urine)'
2188                 };
2189         end
2190         [IEMSol_HYPVLI] = checkIEM_WBM(model,IEMRxns, BiomarkerRxns,minRxnsFluxHealthy);
2191     end
2192     
2193     
2194     %% 440.1 ASNSD Asparagine Synthetase Deficiency
2195     if 1
2196         model = modelO;
2197         
2198         R = {'_ASNS1'};
2199         RxnsAll2 = '';
2200         for i = 1: length(R)
2201             RxnsAll = model.rxns(find(~cellfun(@isempty,strfind(model.rxns,R{i}))));
2202             RxnsAll2 =[RxnsAll2;RxnsAll];
2203         end
2204         IEMRxns = unique(RxnsAll2);
2205         RxnMic = model.rxns(find(~cellfun(@isempty,strfind(model.rxns,'Micro_')))) ;;
2206         IEMRxns = setdiff(IEMRxns,RxnMic);
2207         
2208         if ~strcmp(modelName,'Recon3D') %&& ~strcmp(modelName,'Harvey') %somehow it gets stuck here
2209             % add demand reactions to blood compartment for those biomarkers reported for blood
2210             % biomarker based on https://www.ncbi.nlm.nih.gov/pmc/articles/PMC4486270/
2211             model = addDemandReaction(model, 'asn_L[bc]');
2212             model = addDemandReaction(model, 'asn_L[csf]');
2213             model = addDemandReaction(model, 'gln_L[bc]');
2214             model = addDemandReaction(model, 'gln_L[csf]');
2215             if useSolveCobraLPCPLEX
2216                 model.A = model.S;
2217             else
2218                 if isfield(model,'A')
2219                     model = rmfield(model,'A');
2220                 end
2221             end
2222             BiomarkerRxns = {
2223                 'DM_asn_L[bc]'    'Decreased (blood/csf)'
2224                 'DM_asn_L[csf]'    'Decreased (blood/csf)'
2225                 'DM_gln_L[bc]'    'Increased (blood/csf)'
2226                 'DM_gln_L[csf]'    'Increased (blood/csf)'
2227                 };
2228         else
2229             BiomarkerRxns = {
2230                 'EX_asn_L[u]'    'Decreased (blood/urine)'
2231                 'EX_gln_L[u]'    'Increased (blood/urine)'
2232                 };
2233         end
2234         [IEMSol_ASNSD] = checkIEM_WBM(model,IEMRxns, BiomarkerRxns,minRxnsFluxHealthy);
2235     end
2236 end
2237 
2238 
2239 %% 435.1: Argininosuccinic Aciduria
2240 if 1
2241     model = modelO;
2242     
2243     R = '_ARGSL';
2244     IEMRxns = model.rxns(find(~cellfun(@isempty,strfind(model.rxns,R))));
2245     RxnMic = model.rxns(find(~cellfun(@isempty,strfind(model.rxns,'Micro_')))) ;
2246     IEMRxns = setdiff(IEMRxns,RxnMic);
2247     % set ARGSL to be irreversible
2248     model.lb(find(ismember(model.rxns,IEMRxns))) = 0;
2249     if ~strcmp(modelName,'Recon3D')
2250         % add demand reactions to blood compartment for those biomarkers reported for blood
2251         model = addDemandReaction(model, 'gln_L[bc]');
2252         model = addDemandReaction(model, 'citr_L[bc]');
2253         if useSolveCobraLPCPLEX
2254             model.A = model.S;
2255         else
2256             if isfield(model,'A')
2257                 model = rmfield(model,'A');
2258             end
2259         end
2260         BiomarkerRxns = {
2261             'EX_argsuc[u]'    'Increased (urine)'
2262             'EX_gly[u]'    'Increased (urine)'
2263             'EX_orot[u]'    'Increased (urine)'
2264             'EX_lys_L[u]'    'Increased (urine)'
2265             'EX_ura[u]'    'Increased (urine)'
2266             'DM_gln_L[bc]'    'Increased (blood)'
2267             'DM_citr_L[bc]'    'Increased (blood)'
2268             };
2269     else
2270         BiomarkerRxns = {
2271             'EX_argsuc[u]'    'Increased (urine)'
2272             'EX_gly[u]'    'Increased (urine)'
2273             'EX_orot[u]'    'Increased (urine)'
2274             'EX_lys_L[u]'    'Increased (urine)'
2275             'EX_ura[u]'    'Increased (urine)'
2276             'EX_gln_L[u]'    'Increased (blood)'
2277             'EX_citr_L[u]'    'Increased (blood)'
2278             };
2279     end
2280     [IEMSol_ASA] = checkIEM_WBM(model,IEMRxns, BiomarkerRxns,minRxnsFluxHealthy);
2281 end
2282 
2283 %% '166785.1 MMA Methylmalonic Acidemia (Mma)
2284 if 0
2285     model = modelO;
2286     
2287     R = {'_CBLATm'};%;'_CBL2tm' % lethal in HH
2288     RxnsAll2 = '';
2289     for i = 1: length(R)
2290         RxnsAll = model.rxns(find(~cellfun(@isempty,strfind(model.rxns,R{i}))));
2291         RxnsAll2 =[RxnsAll2;RxnsAll];
2292     end
2293     IEMRxns = unique(RxnsAll2);
2294     RxnMic = model.rxns(find(~cellfun(@isempty,strfind(model.rxns,'Micro_')))) ;;
2295     IEMRxns = setdiff(IEMRxns,RxnMic);
2296     
2297     if ~strcmp(modelName,'Recon3D')
2298         % add demand reactions to blood compartment for those biomarkers reported for blood
2299         model = addDemandReaction(model, 'c4dc[bc]');
2300         model = addDemandReaction(model, 'crn[bc]');
2301         model = addDemandReaction(model, 'HC00900[bc]');
2302         model = addDemandReaction(model, '3hpp[bc]');
2303         if useSolveCobraLPCPLEX
2304             model.A = model.S;
2305         else
2306             if isfield(model,'A')
2307                 model = rmfield(model,'A');
2308             end
2309         end
2310         BiomarkerRxns = {
2311             'EX_3aib[u]'    'Increased (urine)'
2312             'DM_c4dc[bc]'    'Increased (blood)'
2313             'DM_crn[bc]'    'Decreased (blood)'
2314             'DM_HC00900[bc]'    'Increased (blood)'
2315             'DM_3hpp[bc]'    'Increased (blood)'
2316             };
2317     else
2318         BiomarkerRxns = {
2319             'EX_3aib[u]'    'Increased (urine)'
2320             'EX_c4dc[u]'    'Increased (blood)'
2321             'EX_crn[u]'    'Decreased (blood)'
2322             'EX_HC00900[u]'    'Increased (blood)'
2323             'EX_3hpp[u]'    'Increased (blood)'
2324             };
2325     end
2326     [IEMSol_MMA] = checkIEM_WBM(model,IEMRxns, BiomarkerRxns,minRxnsFluxHealthy);
2327 end
2328 %% '5091.1 PC Pyruvate Carboxylase Deficiency
2329 if 1
2330     model = modelO;
2331     
2332     R = {'_PCm'};
2333     RxnsAll2 = '';
2334     for i = 1: length(R)
2335         RxnsAll = model.rxns(find(~cellfun(@isempty,strfind(model.rxns,R{i}))));
2336         RxnsAll2 =[RxnsAll2;RxnsAll];
2337     end
2338     IEMRxns = unique(RxnsAll2);
2339     RxnMic = model.rxns(find(~cellfun(@isempty,strfind(model.rxns,'Micro_')))) ;;
2340     IEMRxns = setdiff(IEMRxns,RxnMic);
2341     if ~strcmp(modelName,'Recon3D')
2342         % add demand reactions to blood compartment for those biomarkers reported for blood
2343         model = addDemandReaction(model, 'acac[bc]');
2344         model = addDemandReaction(model, 'ala_L[bc]');
2345         model = addDemandReaction(model, 'bhb[bc]');
2346         model = addDemandReaction(model, 'citr_L[bc]');
2347         model = addDemandReaction(model, 'lys_L[bc]');
2348         model = addDemandReaction(model, 'pro_L[bc]');
2349         model = addDemandReaction(model, 'lac_L[bc]');
2350         model = addDemandReaction(model, 'nh4[bc]');
2351         model = addDemandReaction(model, 'glc_D[bc]');
2352         if useSolveCobraLPCPLEX
2353             model.A = model.S;
2354         else
2355             if isfield(model,'A')
2356                 model = rmfield(model,'A');
2357             end
2358         end
2359         BiomarkerRxns = {
2360             'DM_acac[bc]'    'Increased (urine/blood)'
2361             'DM_ala_L[bc]'    'Increased (blood)'
2362             'DM_bhb[bc]'    'Increased (urine/blood)'
2363             'DM_citr_L[bc]'    'Increased (blood)'
2364             'DM_lys_L[bc]'    'Increased (blood)'
2365             'DM_pro_L[bc]'    'Increased (blood)'
2366             'DM_lac_L[bc]'    'Increased (blood)' % after 12hrs of fasting,
2367             'DM_nh4[bc]'    'Increased (blood)' % after 12hrs of fasting,
2368             'DM_glc_D[bc]'    'Decreased (blood)' % after 12hrs of fasting,
2369             'EX_fum[u]'    'Increased (urine)'
2370             'EX_succ[u]'    'Increased (urine)'
2371             'EX_acetone[u]'    'Increased (urine)'
2372             'EX_akg[u]'    'Increased (urine)'
2373             'EX_acac[u]'    'Increased (urine/blood)'
2374             'EX_bhb[u]'    'Increased (urine/blood)'
2375             };
2376     else
2377         
2378         BiomarkerRxns = {
2379             'EX_acac[u]'    'Increased (urine/blood)'
2380             'EX_acetone[u]'    'Increased (urine)'
2381             'EX_akg[u]'    'Increased (urine)'
2382             'EX_ala_L[u]'    'Increased (blood)'
2383             'EX_bhb[u]'    'Increased (urine/blood)'
2384             'EX_citr_L[u]'    'Increased (blood)'
2385             'EX_lys_L[u]'    'Increased (blood)'
2386             'EX_pro_L[u]'    'Increased (blood)'
2387             'EX_succ[u]'    'Increased (urine)'
2388             'EX_lac_L[u]'    'Increased (blood)' % after 12hrs of fasting,
2389             'EX_nh4[u]'    'Increased (blood)' % after 12hrs of fasting,
2390             'EX_glc_D[u]'    'Decreased (blood)' % after 12hrs of fasting,
2391             'EX_fum[u]'    'Increased (urine)'
2392             };
2393     end
2394     [IEMSol_PC] = checkIEM_WBM(model,IEMRxns, BiomarkerRxns,minRxnsFluxHealthy);
2395 end
2396 %% '189.1 PHOX1 Primary Hyperoxaluria-Type 1
2397 if 1
2398     model = modelO;
2399     
2400     R = {'_AGTix';'_SPTix';'_r0160'};
2401     RxnsAll2 = '';
2402     for i = 1: length(R)
2403         RxnsAll = model.rxns(find(~cellfun(@isempty,strfind(model.rxns,R{i}))));
2404         RxnsAll2 =[RxnsAll2;RxnsAll];
2405     end
2406     IEMRxns = unique(RxnsAll2);
2407     RxnMic = model.rxns(find(~cellfun(@isempty,strfind(model.rxns,'Micro_')))) ;;
2408     IEMRxns = setdiff(IEMRxns,RxnMic);
2409     
2410     BiomarkerRxns = {
2411         'EX_oxa[u]'    'Increased (urine)'
2412         'EX_glyclt[u]'    'Increased (urine)'
2413         'EX_glx[u]'    'Increased (urine)'
2414         };
2415     [IEMSol_PHOX1] = checkIEM_WBM(model,IEMRxns, BiomarkerRxns,minRxnsFluxHealthy);
2416 end
2417 
2418 %% '4967.1 ADSL Adenylosuccinase Deficiency
2419 if 1
2420     model = modelO;
2421     
2422     R = {'_ADSL1';'_ADSL2'};
2423     RxnsAll2 = '';
2424     for i = 1: length(R)
2425         RxnsAll = model.rxns(find(~cellfun(@isempty,strfind(model.rxns,R{i}))));
2426         RxnsAll2 =[RxnsAll2;RxnsAll];
2427     end
2428     IEMRxns = unique(RxnsAll2);
2429     RxnMic = model.rxns(find(~cellfun(@isempty,strfind(model.rxns,'Micro_')))) ;;
2430     IEMRxns = setdiff(IEMRxns,RxnMic);
2431     if ~strcmp(modelName,'Recon3D')
2432         % add demand reactions to blood compartment for those biomarkers reported for blood
2433         model = addDemandReaction(model, 'Brain_25aics[c]');
2434         if useSolveCobraLPCPLEX
2435             model.A = model.S;
2436         else
2437             if isfield(model,'A')
2438                 model = rmfield(model,'A');
2439             end
2440         end
2441         BiomarkerRxns = {
2442             % 'EX_25aics[u]'    'Increased (urine/csf)' % not in HH
2443             'DM_Brain_25aics[c]'    'Increased (urine/csf)' % cannot be produced by HH
2444             };
2445     else
2446         BiomarkerRxns = {
2447             %    'EX_25aics[u]'    'Increased (urine/csf)'
2448             };
2449     end
2450     [IEMSol_ADSL] = checkIEM_WBM(model,IEMRxns, BiomarkerRxns,minRxnsFluxHealthy);
2451 end
2452 
2453 
2454 %% 443.1 CD Canavan Disease - only milder forms of this disease have been reported (there is an isozyme in Recon -- due to mild form report I included this IEM anyway)
2455 if 1
2456     model = modelO;
2457     
2458     R = {'_NACASPAH'};
2459     RxnsAll2 = '';
2460     for i = 1: length(R)
2461         RxnsAll = model.rxns(find(~cellfun(@isempty,strfind(model.rxns,R{i}))));
2462         RxnsAll2 =[RxnsAll2;RxnsAll];
2463     end
2464     IEMRxns = unique(RxnsAll2);
2465     RxnMic = model.rxns(find(~cellfun(@isempty,strfind(model.rxns,'Micro_')))) ;;
2466     IEMRxns = setdiff(IEMRxns,RxnMic);
2467     if ~strcmp(modelName,'Recon3D')
2468         % add demand reactions to blood compartment for those biomarkers reported for blood
2469         model = addDemandReaction(model, 'Nacasp[bc]');
2470         model = addDemandReaction(model, 'Nacasp[csf]');
2471         if useSolveCobraLPCPLEX
2472             model.A = model.S;
2473         else
2474             if isfield(model,'A')
2475                 model = rmfield(model,'A');
2476             end
2477         end
2478         BiomarkerRxns = {
2479             % 'EX_25aics[u]'    'Increased (urine/csf)' % not in HH
2480             'EX_Nacasp[u]'    'Increased (urine/csf/blood)' % cannot be produced by HH
2481             'DM_Nacasp[bc]'    'Increased (urine/csf/blood)'
2482             'DM_Nacasp[csf]'    'Increased (urine/csf/blood)'
2483             };
2484     else
2485         BiomarkerRxns = {
2486             'EX_Nacasp[u]'    'Increased (urine/csf/blood)'
2487             };
2488     end
2489     [IEMSol_CD] = checkIEM_WBM(model,IEMRxns, BiomarkerRxns,minRxnsFluxHealthy);
2490 end
2491 %% 1371.1 HPC Hereditary Coproporphyria
2492 if 1 %Rxn obj is 0
2493     model = modelO;
2494     
2495     R = {'_CPPPGO'};
2496     RxnsAll2 = '';
2497     for i = 1: length(R)
2498         RxnsAll = model.rxns(find(~cellfun(@isempty,strfind(model.rxns,R{i}))));
2499         RxnsAll2 =[RxnsAll2;RxnsAll];
2500     end
2501     IEMRxns = unique(RxnsAll2);
2502     RxnMic = model.rxns(find(~cellfun(@isempty,strfind(model.rxns,'Micro_')))) ;;
2503     IEMRxns = setdiff(IEMRxns,RxnMic);
2504     if ~strcmp(modelName,'Recon3D')
2505         % add demand reactions to blood compartment for those biomarkers reported for blood
2506         model = addDemandReaction(model, 'C05770[bc]');
2507         model = addDemandReaction(model, 'C05770[csf]');
2508         if useSolveCobraLPCPLEX
2509             model.A = model.S;
2510         else
2511             if isfield(model,'A')
2512                 model = rmfield(model,'A');
2513             end
2514         end
2515         BiomarkerRxns = {
2516             'EX_25aics[u]'    'Increased (urine/csf)' % not in HH
2517             'EX_C05770[u]'    'Increased (urine/csf/blood)'
2518             'DM_C05770[bc]'    'Increased (urine/csf/blood)'
2519             'DM_C05770[csf]'    'Increased (urine/csf/blood)'
2520             };
2521     else
2522         BiomarkerRxns = {
2523             'EX_C05770[u]'    'Increased (urine/csf/blood)'
2524             };
2525     end
2526     [IEMSol_HPC] = checkIEM_WBM(model,IEMRxns, BiomarkerRxns,minRxnsFluxHealthy);
2527 end
2528 
2529 %% 191.1 HMET Hypermethioninemia
2530 if 1
2531     model = modelO;
2532     
2533     R = {'_GNMT'};
2534     RxnsAll2 = '';
2535     for i = 1: length(R)
2536         RxnsAll = model.rxns(find(~cellfun(@isempty,strfind(model.rxns,R{i}))));
2537         RxnsAll2 =[RxnsAll2;RxnsAll];
2538     end
2539     IEMRxns = unique(RxnsAll2);
2540     RxnMic = model.rxns(find(~cellfun(@isempty,strfind(model.rxns,'Micro_')))) ;;
2541     IEMRxns = setdiff(IEMRxns,RxnMic);
2542     
2543     if ~strcmp(modelName,'Recon3D')
2544         % add demand reactions to blood compartment for those biomarkers reported for blood
2545         model = addDemandReaction(model, 'hcys_L[bc]');
2546         model = addDemandReaction(model, 'met_L[bc]');
2547         if useSolveCobraLPCPLEX
2548             model.A = model.S;
2549         else
2550             if isfield(model,'A')
2551                 model = rmfield(model,'A');
2552             end
2553         end
2554         BiomarkerRxns = {
2555             'EX_met_L[u]'    'Increased (urine/blood)' %
2556             'EX_hcys_L[u]'    'Increased (urine/blood)'
2557             'DM_hcys_L[bc]'    'Increased (urine/blood)'
2558             'DM_met_L[bc]'    'Increased (urine/blood)'
2559             };
2560     else
2561         BiomarkerRxns = {
2562             'EX_met_L[u]'    'Increased (urine/blood)'
2563             'EX_hcys_L[u]'    'Increased (urine/blood)'
2564             };
2565     end
2566     [IEMSol_HMET] = checkIEM_WBM(model,IEMRxns, BiomarkerRxns,minRxnsFluxHealthy);
2567 end
2568 
2569 %% 1718.1 DESMO Desmosterolosis
2570 if 1
2571     model = modelO;
2572     
2573     R = {'_DHCR241r';'_DHCR243r';'_r0783';'_r1380';'_DSREDUCr';'_HMR_1526';'_RE3129N'};
2574     RxnsAll2 = '';
2575     for i = 1: length(R)
2576         RxnsAll = model.rxns(find(~cellfun(@isempty,strfind(model.rxns,R{i}))));
2577         RxnsAll2 =[RxnsAll2;RxnsAll];
2578     end
2579     IEMRxns = unique(RxnsAll2);
2580     RxnMic = model.rxns(find(~cellfun(@isempty,strfind(model.rxns,'Micro_')))) ;;
2581     IEMRxns = setdiff(IEMRxns,RxnMic);
2582     
2583     R2 = {'_RE2410C';'_RE2410N'};
2584     RxnsAll2 = '';
2585     for i = 1: length(R2)
2586         RxnsAll = model.rxns(find(~cellfun(@isempty,strfind(model.rxns,R2{i}))));
2587         RxnsAll2 =[RxnsAll2;RxnsAll];
2588     end
2589     X = unique(RxnsAll2);
2590     RxnMic = model.rxns(find(~cellfun(@isempty,strfind(model.rxns,'Micro_')))) ;;
2591     X = setdiff(X,RxnMic);
2592     model.lb(find(ismember(model.rxns,X))) = 0;
2593     
2594     if ~strcmp(modelName,'Recon3D')
2595         % add demand reactions to blood compartment for those biomarkers reported for blood
2596         model = addDemandReaction(model, 'dsmsterol[bc]');
2597         if useSolveCobraLPCPLEX
2598             model.A = model.S;
2599         else
2600             if isfield(model,'A')
2601                 model = rmfield(model,'A');
2602             end
2603         end
2604         BiomarkerRxns = {
2605             'DM_dsmsterol[bc]'    'Increased (blood)'
2606             };
2607     else
2608         BiomarkerRxns = {
2609             'EX_dsmsterol[u]'    'Increased (blood)'
2610             };
2611     end
2612     [IEMSol_DESMO] = checkIEM_WBM(model,IEMRxns, BiomarkerRxns,minRxnsFluxHealthy);
2613 end
2614 
2615 
2616 %% 89874.1 2OAA 2-Oxoadipate Acidemia
2617 if 1 % not well studied - inconsistent biomarkers between reports
2618     model = modelO;
2619     
2620     R = {'_2OXOADPTm';'_2AMADPTm';'_r0879'};
2621     RxnsAll2 = '';
2622     for i = 1: length(R)
2623         RxnsAll = model.rxns(find(~cellfun(@isempty,strfind(model.rxns,R{i}))));
2624         RxnsAll2 =[RxnsAll2;RxnsAll];
2625     end
2626     IEMRxns = unique(RxnsAll2);
2627     RxnMic = model.rxns(find(~cellfun(@isempty,strfind(model.rxns,'Micro_')))) ;
2628     IEMRxns = setdiff(IEMRxns,RxnMic);
2629     
2630     if ~strcmp(modelName,'Recon3D')
2631         % add demand reactions to blood compartment for those biomarkers reported for blood
2632         % biomarker based on https://www.omim.org/entry/204750
2633         model = addDemandReaction(model, 'L2aadp[bc]');
2634         if useSolveCobraLPCPLEX
2635             model.A = model.S;
2636         else
2637             if isfield(model,'A')
2638                 model = rmfield(model,'A');
2639             end
2640         end
2641         BiomarkerRxns = {
2642             'DM_L2aadp[bc]'    'Increased (blood)'
2643             'EX_2oxoadp[u]'    'Increased (urine)'
2644             'EX_adpoh[u]'    'Increased (urine)'
2645             };
2646     else
2647         BiomarkerRxns = {
2648             'EX_2oxoadp[u]'    'Increased (urine)'
2649             'EX_adpoh[u]'    'Increased (urine)'
2650             };
2651     end
2652     [IEMSol_2OAA] = checkIEM_WBM(model,IEMRxns, BiomarkerRxns,minRxnsFluxHealthy);
2653 end
2654 
2655 
2656 %% Parse IEM results
2657 
2658 vars = who;
2659 vars_IEM = strmatch('IEMSol_',vars);
2660 % count results (in vivo vs in silico
2661 UpUp =0;
2662 DoDo = 0;
2663 UpDo = 0;
2664 DoUp = 0;
2665 UpUn = 0; %up in vivo, unchanged in silico
2666 DoUn = 0; %down in vivo, unchanged in silico
2667 UnUp = 0; %unchanged in vivo, up in silico
2668 UnDo = 0; %unchanged in vivo, down in silico
2669 UnUn =0;
2670 cnt = 1;
2671 
2672 clear Table_IEM
2673 for i = 1 : length(vars_IEM)
2674     % read in IEM solutions
2675     clear IEM
2676     IEM = evalin('base',vars{vars_IEM(i)});
2677     % get change of direction
2678     for j = 5 : 2 : size(IEM,1)
2679         H_D = str2num(char(IEM(j,2))) -  str2num(char(IEM(j+1,2))); % healthy minus disease
2680         if H_D < -1e-6 %Increased
2681             H_D_in_sil =1;
2682         elseif  H_D > 1e-6 %Increased
2683             H_D_in_sil =-1;
2684         else % unchanged
2685             H_D_in_sil =0;
2686         end
2687         % is the marker Increased or decreased in vivo?
2688         if ~isempty(strfind(IEM{j,3},'Incre'))
2689             H_D_in_vivo = 1;
2690         elseif  ~isempty(strfind(IEM{j,3},'Decre'))
2691             H_D_in_vivo = -1;
2692         else % unchanged
2693             H_D_in_vivo = 0;
2694         end
2695         % create new table with all results
2696         Table_IEM{cnt,1} = regexprep(vars{vars_IEM(i)},'IEMSol_',''); % IEM abbr
2697         Table_IEM{cnt,2} = regexprep(IEM{j,1},'Healthy:',''); % biomaker
2698         Table_IEM{cnt,3} = (IEM(j,2)); % healthy original values
2699         Table_IEM{cnt,4} = (IEM(j+1,2)); % disease original values
2700         Table_IEM{cnt,5} = num2str(H_D_in_sil); % in silico
2701         Table_IEM{cnt,6} = num2str(H_D_in_vivo); % in vivo
2702         Table_IEM{cnt,7} = IEM{j,3}; % original in vivo message (for biofluid info)
2703         Table_IEM{cnt,8} = (IEM(1,2));
2704         cnt = cnt +1;
2705         if H_D_in_vivo == 1 && H_D_in_sil == 1
2706             UpUp =  UpUp + 1;
2707         elseif H_D_in_vivo == -1 && H_D_in_sil == -1
2708             DoDo = DoDo + 1;
2709         elseif H_D_in_vivo == 1 && H_D_in_sil == -1
2710             UpDo =  UpDo +1;
2711         elseif H_D_in_vivo == -1 && H_D_in_sil == 1
2712             DoUp = DoUp + 1;
2713         elseif H_D_in_vivo == 0 && H_D_in_sil == 1
2714             UnUp = UnUp + 1;
2715         elseif H_D_in_vivo == 1 && H_D_in_sil == 0
2716             UpUn = UpUn + 1;
2717         elseif H_D_in_vivo == -1 && H_D_in_sil == 0
2718             DoUn = DoUn + 1;
2719         elseif H_D_in_vivo == 0 && H_D_in_sil == -1
2720             UnDo = UnDo + 1;
2721         elseif H_D_in_vivo == 0 && H_D_in_sil == 0
2722             UnUn = UnUn + 1;
2723         end
2724     end
2725 end
2726 
2727 clear Table_IEM_Grid
2728 Diseases = unique(Table_IEM(:,1));
2729 BioMU = unique(Table_IEM(find(~cellfun(@isempty,strfind(Table_IEM(:,2),'[u]'))),2));
2730 BioMCSF = unique(Table_IEM(find(~cellfun(@isempty,strfind(Table_IEM(:,2),'[csf]'))),2));
2731 BioMBC = unique(Table_IEM(find(~cellfun(@isempty,strfind(Table_IEM(:,2),'[bc]'))),2));
2732 BioM = [BioMBC; BioMU; BioMCSF];
2733 
2734 cnt=1;
2735 for i = 1 : length(vars_IEM)
2736     % read in IEM solutions
2737     clear IEM
2738     IEM = evalin('base',vars{vars_IEM(i)});
2739     % get change of direction
2740     for j = 5 : 2 : size(IEM,1)
2741         H_D = str2num(char(IEM(j,2))) -  str2num(char(IEM(j+1,2))); % healthy minus disease
2742         if H_D < -1e-6 %Increased
2743             H_D_in_sil =1;
2744         elseif  H_D > 1e-6 %Increased
2745             H_D_in_sil =-1;
2746         else % unchanged
2747             H_D_in_sil =0;
2748         end
2749         % is the marker Increased or decreased in vivo?
2750         if ~isempty(strfind(IEM{j,3},'Incre'))
2751             H_D_in_vivo = 1;
2752         elseif  ~isempty(strfind(IEM{j,3},'Decre'))
2753             H_D_in_vivo = -1;
2754         else % unchanged
2755             H_D_in_vivo = 0;
2756         end
2757         
2758         cnt = cnt +1;
2759     end
2760 end
2761 
2762 
2763 % make Table with overall results
2764 Table_sum_results{1,2} = 'up';
2765 Table_sum_results{1,3} = 'un';
2766 Table_sum_results{1,4} = 'down';
2767 Table_sum_results{2,1} = 'up';
2768 Table_sum_results{3,1} = 'un';
2769 Table_sum_results{4,1} = 'down';
2770 Table_sum_results{2,2} = num2str(UpUp);
2771 Table_sum_results{2,3} = num2str(UnUp);
2772 Table_sum_results{2,4} = num2str(DoUp);
2773 Table_sum_results{3,2} = num2str(UpUn);
2774 Table_sum_results{3,3} = num2str(UnUn);
2775 Table_sum_results{3,4} = num2str(DoUn);
2776 Table_sum_results{4,2} = num2str(UpDo);
2777 Table_sum_results{4,3} = num2str(UnDo);
2778 Table_sum_results{4,4} = num2str(DoDo);
2779 
2780 Accuracy = (UpUp + DoDo)/(UpUp+UnUp+DoUp+UpUn+UnUn+DoUn+UpDo+UnDo+DoDo)
2781 Precision = (UpUp)/(UpUp + UpDo)
2782 FalseDiscoveryRate = (UpDo)/(UpUp + UpDo)
2783 
2784 NumDiseases = length(unique(Table_IEM(:,1)))
2785 NumBiomarkers = length(unique(Table_IEM(:,2)))
2786 
2787 clear Bio* Do* H_* IEM IEMRxns R R2 RxnsA* Un* Up* X cnt i j minR* model vars*
2788 %clearvars -except Table_sum_results Accuracy Precision FalseDiscoveryRate NumDiseases NumBiomarkers
2789 
2790 if strcmp(modelName,'Harvey')
2791     % load  Harvey1_0
2792     if  microbiome == 1
2793         save([resultsPath 'Results_IEM_Harvey_1_03_Mic'])
2794     else
2795         save([resultsPath 'Results_IEM_Harvey_1_03'])
2796     end
2797     
2798 elseif strcmp(modelName,'Harvetta')
2799     % load  Harvetta1_0
2800     if  microbiome == 1
2801         save([resultsPath 'Results_IEM_Harvetta_1_03_Mic'])
2802     else
2803         save([resultsPath 'Results_IEM_Harvetta_1_03'])
2804     end
2805 elseif strcmp(modelName,'Recon3D')
2806     save([resultsPath 'Results_IEM_Recon3DStar'])
2807 end
```

---

Generated on Thu 14-May-2020 13:05:49 by **m2html** © 2005
