# Supplementary material for: Personalized whole‐body models integrate metabolism, physiology, and the gut microbiome
Source: Mol Syst Biol. 2020 May 28;16(5):e8982. doi: 10.15252/msb.20198982 (PMC7285886; doi:10.15252/msb.20198982)
Supplement: Supplementary file 22 — Dataset EV1 [file MSB-16-e8982-s022.zip › PSCM_toolbox/PSCM_toolbox_doc/src/scripts/annotateHH.html]

Description of annotateHH


# annotateHH

## PURPOSE

**annotate Harvey and Harvetta**

## SYNOPSIS

**This is a script file.**

## DESCRIPTION

```
 annotate Harvey and Harvetta
```

## CROSS-REFERENCE INFORMATION

This function calls:

- annotateModel This function annotates a model with VMH reaction and metabolite
- loadPSCMfile Loads a mat file into the workspace, given a nickname or a full filename

This function is called by:

## SOURCE CODE

```
0001 % annotate Harvey and Harvetta
0002 
0003 annotateRxns = 1;
0004 annotateMets = 1;
0005 
0006 male = loadPSCMfile('Harvey');
0007 modelID = strcat('Harvey');
0008 modelName = strcat('Male whole body metabolic reconstruction, Harvey.');
0009 modelAnnotation = {strcat('This is a metabolic reconstruction of: ', modelID);...
0010     'Authors: Ines Thiele, NUI Galway, Ireland';...
0011     'Please cite when using one or more reconstructions from the Organ compendium: Thiele et al.,Molecular Systems Biology, 2020.';...
0012     'This reconstruction has been extensively curated against experimental data from literature.';...
0013     'Please contact: ines(dot)thiele(at)nuigalway.ie';...
0014     'This work is licensed under a <a href="https://creativecommons.org/licenses/by-nc-nd/4.0/" target="_blank">Creative Commons Attribution-NonCommercial-NoDerivatives 4.0 International License</a>.'};
0015 tic;male = annotateModel(male, annotateRxns,annotateMets,modelID,modelName,modelAnnotation);toc;
0016 
0017 save Harvey male
0018 clear
0019 
0020 female = loadPSCMfile('Harvetta');
0021 annotateRxns = 1;
0022 annotateMets = 1;
0023 modelID = strcat('Harvetta');
0024 modelName = strcat('Male whole body metabolic reconstruction, Harvetta.');
0025 modelAnnotation = {strcat('This is a metabolic reconstruction of: ', modelID);...
0026     'Authors: Ines Thiele, NUI Galway, Ireland';...
0027     'Please cite when using one or more reconstructions from the Organ compendium: Thiele et al., Molecular Systems Biology, 2020.';...
0028     'This reconstruction has been extensively curated against experimental data from literature.';...
0029     'Please contact: ines(dot)thiele(at)nuigalway.ie';...
0030     'This work is licensed under a <a href="https://creativecommons.org/licenses/by-nc-nd/4.0/" target="_blank">Creative Commons Attribution-NonCommercial-NoDerivatives 4.0 International License</a>.'};
0031 tic;female = annotateModel(female, annotateRxns,annotateMets,modelID,modelName,modelAnnotation);toc;
0032 
0033 
0034 save Harvetta female
```

---

Generated on Thu 14-May-2020 13:05:49 by **m2html** © 2005
