# Supplementary material for: Personalized whole‐body models integrate metabolism, physiology, and the gut microbiome
Source: Mol Syst Biol. 2020 May 28;16(5):e8982. doi: 10.15252/msb.20198982 (PMC7285886; doi:10.15252/msb.20198982)
Supplement: Supplementary file 22 — Dataset EV1 [file MSB-16-e8982-s022.zip › PSCM_toolbox/PSCM_toolbox_doc/src/scripts/LeakTestRecon.html]

Description of LeakTestRecon


# LeakTestRecon

## PURPOSE

**changeCobraSolver('tomlab\_cplex','lp');**

## SYNOPSIS

**This is a script file.**

## DESCRIPTION

```
changeCobraSolver('tomlab_cplex','lp');
modelClosed = modelConsistent;
```

## CROSS-REFERENCE INFORMATION

This function calls:


This function is called by:

- performSanityChecksonRecon This function performs various quality control and quality assurance

## SOURCE CODE

```
0001 
0002 %changeCobraSolver('tomlab_cplex','lp');
0003 %modelClosed = modelConsistent;
0004 clear FF R
0005 % add demands for all metabolites in Recon
0006 modelClosed = model;
0007 %modelClosed = addDemandReaction(modelClosed,modelClosed.mets);
0008 
0009 modelexchanges1 = strmatch('Ex_',modelClosed.rxns);
0010 modelexchanges4 = strmatch('EX_',modelClosed.rxns);
0011 modelexchanges2 = strmatch('DM_',modelClosed.rxns);
0012 modelexchanges3 = strmatch('sink_',modelClosed.rxns);
0013 selExc = (find( full((sum(abs(modelClosed.S)==1,1) ==1) & (sum(modelClosed.S~=0) == 1))))';
0014 
0015 modelexchanges = unique([modelexchanges1;modelexchanges2;modelexchanges3;modelexchanges4;selExc]);
0016 modelClosed.lb(ismember(modelClosed.rxns,modelClosed.rxns(modelexchanges)))=0;
0017 %modelClosed.ub(find(ismember(modelClosed.rxns,modelClosed.rxns(modelexchanges))))=0;
0018 
0019 [LeakMets,modelClosed] = fastLeakTest(modelClosed, modelClosed.rxns(modelexchanges),0);
```

---

Generated on Thu 14-May-2020 13:05:49 by **m2html** © 2005
