# Supplementary material for: Personalized whole‐body models integrate metabolism, physiology, and the gut microbiome
Source: Mol Syst Biol. 2020 May 28;16(5):e8982. doi: 10.15252/msb.20198982 (PMC7285886; doi:10.15252/msb.20198982)
Supplement: Supplementary file 22 — Dataset EV1 [file MSB-16-e8982-s022.zip › PSCM_toolbox/PSCM_toolbox_doc/src/setConstraints/setFeedingFastingConstraints.html]

Description of setFeedingFastingConstraints


# setFeedingFastingConstraints

## PURPOSE

**This function sets constraints corresponding either to feeding (i.e.,**

## SYNOPSIS

**function model = setFeedingFastingConstraints(model, feedingStatus,fastingValue,storageValue)**

## DESCRIPTION

```
 This function sets constraints corresponding either to feeding (i.e.,
 storage reactions are set to have a lower bound of 0 while
 the upper bounds are set to >0 (storage is enabled) or to fasting (i.e.,
 storage reactions are set to have a lower bound of <0 (use of stored metabolites is enabled) while
 the upper bounds are set to 0 (storage is disabled).

 function model = setFeedingFastingConstraints(model, feedingStatus,fastingValue,storageValue)

 INPUT
 model             model structure
 feedingStatus     'feeding' = default bounds for diet uptake fastingValue for all diet exchanges, use diet script to refine, organ storage is turned on;
                   'fasting' = all diet uptakes are closed, organ storage release is turned on
 fastingValue      default: fastingValue = -10;
 storageValue      default: storageValue = 10;

 OUTPUT
 model             with updated constraints

 Ines Thiele, 2015/2016
```

## CROSS-REFERENCE INFORMATION

This function calls:

- AAStorage This script defines amino acids that can be stored by one or more organs

This function is called by:

- analyzeHMmodel This function performs host-microbiome optimization for a set of defined

## SOURCE CODE

```
0001 function model = setFeedingFastingConstraints(model, feedingStatus,fastingValue,storageValue)
0002 % This function sets constraints corresponding either to feeding (i.e.,
0003 % storage reactions are set to have a lower bound of 0 while
0004 % the upper bounds are set to >0 (storage is enabled) or to fasting (i.e.,
0005 % storage reactions are set to have a lower bound of <0 (use of stored metabolites is enabled) while
0006 % the upper bounds are set to 0 (storage is disabled).
0007 %
0008 % function model = setFeedingFastingConstraints(model, feedingStatus,fastingValue,storageValue)
0009 %
0010 % INPUT
0011 % model             model structure
0012 % feedingStatus     'feeding' = default bounds for diet uptake fastingValue for all diet exchanges, use diet script to refine, organ storage is turned on;
0013 %                   'fasting' = all diet uptakes are closed, organ storage release is turned on
0014 % fastingValue      default: fastingValue = -10;
0015 % storageValue      default: storageValue = 10;
0016 %
0017 % OUTPUT
0018 % model             with updated constraints
0019 %
0020 % Ines Thiele, 2015/2016
0021 if ~exist('fastingValue','var')
0022     fastingValue = -10;
0023 end
0024 if ~exist('storageValue','var')
0025     storageValue = 10;
0026 end
0027 
0028 % load amino acids that can be stored
0029 AAStorage;
0030 storedInLiver={'nad(c)';'nadp(c)';'fad(c)';...
0031     'thmtp(c)';'pydam(c)';'pydx(c)';'pydxn(c)';...
0032     'coa(c)';'thf(c)';'btn(c)';'retinol(c)';'retfa(c)';...
0033     '11_cis_retfa(c)';'9_cis_retfa(c)';'25hvitd2(c)';'vitd3(c)';...
0034     'avite1(c)';'avite2(c)';'chol(c)';'fe3(c)';'phyQ(c)';...
0035     'glygn2(c)'};
0036 StoreInKidney = {'ascb_L(c)';'chol(c)';};
0037 StoreInMuscle ={'thmpp(c)';'25hvitd2(c)';'vitd3(c)';'chol(c)';'glygn2(c)'};
0038 
0039 StoreInAdi={'25hvitd2(c)';'vitd3(c)';'avite1(c)';'avite2(c)';'phyQ(c)';...
0040     'hdca(c)';'tmndnc(c)';'lnlc(c)';'tag_hs(c)';...
0041     'c226coa(c)';'doco13ecoa(c)';'lnlccoa(c)';'lnlncacoa(c)';'lnlncgcoa(c)';...
0042     'odecoa(c)';'pmtcoa(c)';'stcoa(c)';'tmndnccoa(c)'
0043     };
0044 if isfield(model,'rxnGeneMat')
0045     model = rmfield(model,'rxnGeneMat');
0046 end
0047 % set all sinks to 0
0048 for i = 1 : length(model.rxns)
0049     if length(strfind(model.rxns{i},'sink_'))==1 ...
0050             && length(strfind(model.rxns{i},'sink_pre_prot(r)'))==0  ...
0051             && length(strfind(model.rxns{i},'sink_Ser_Gly_Ala_X_Gly(r)'))==0 ...
0052             && length(strfind(model.rxns{i},'sink_5hpet(c)'))==0
0053         %&& length(strfind(model.rxns{i},'sink_Tyr_ggn(c)'))==0 % ...
0054         %% && length(strfind(model.rxns{i},'sink_citr(c)'))==0
0055         % && length(strfind(model.rxns{i},'sink_Ser_Gly_Ala_X_Gly(r)'))==0 ...
0056         if isfield(model,'Microbiota') && model.Microbiota(i) ==0 %no microbe sink
0057             model.lb(i)=0;
0058         elseif ~isfield(model,'Microbiota')
0059             model.lb(i)=0;
0060         end
0061     end
0062 end
0063 
0064 DMs= (find(~cellfun(@isempty,strfind(model.rxns,'DM_'))));
0065 model.lb(DMs) = 0;
0066 
0067 % but not at the beginning of the abbr --> leaves in the
0068 % microbe sinks
0069 if 1
0070     tmp = strmatch('sink_',model.rxns);
0071     model.lb(tmp)=-10;
0072 end
0073 
0074 for i = 1 : length(model.rxns)
0075     if strfind(model.rxns{i},'sink_')
0076         model.rxns{i} = regexprep(model.rxns{i},'\[c\]','(c)');
0077         model.rxns{i} = regexprep(model.rxns{i},'\[r\]','(r)');
0078         %    model.rxns{i}
0079     end
0080 end
0081 modelexchanges1 = strmatch('Diet_EX_',model.rxns);
0082 modelexchanges2 = strmatch('Diet_Ex_',model.rxns);
0083 modelexchanges = [modelexchanges1;modelexchanges2];
0084 
0085 if strcmp(feedingStatus,'feeding') % storage no sinks
0086     model.lb(modelexchanges)=fastingValue;
0087     model.ub(modelexchanges)=0;
0088 elseif strcmp(feedingStatus,'fasting') % storage no sinks
0089     model.lb(modelexchanges)=0;
0090     model.ub(modelexchanges)=0;
0091     % exception for water
0092     
0093 end
0094 
0095 if strcmp(feedingStatus,'feeding') % storage no sinks
0096     for i = 1 : length(storedInLiver)
0097         storedInLiver{i};
0098         rxnName = strcat('Liver_sink_',storedInLiver{i});
0099         %   rxnName
0100         model = changeRxnBounds(model,rxnName,0,'l');
0101         model = changeRxnBounds(model,rxnName, storageValue,'u');
0102     end
0103     for i = 1 : length(StoreInKidney)
0104         StoreInKidney{i};
0105         rxnName = strcat('Kidney_sink_',StoreInKidney{i});
0106         %  rxnName
0107         model = changeRxnBounds(model,rxnName,0,'l');
0108         model = changeRxnBounds(model,rxnName, storageValue,'u');
0109     end
0110     for i = 1 : length(StoreInMuscle)
0111         rxnName = strcat('Muscle_sink_',StoreInMuscle{i});
0112         %  rxnName
0113         model = changeRxnBounds(model,rxnName,0,'l');
0114         model = changeRxnBounds(model,rxnName, storageValue,'u');
0115     end
0116     for i = 1 : length(storageAA)
0117         rxnName = strcat('Muscle_',storageAA{i});
0118         %    rxnName
0119         model = changeRxnBounds(model,rxnName,0,'l');
0120         model = changeRxnBounds(model,rxnName, storageValue,'u');
0121     end
0122     for i = 1 : length(StoreInAdi)
0123         rxnName = strcat('Adipocytes_sink_',StoreInAdi{i});
0124         %   rxnName
0125         StoreInAdi{i};
0126         model = changeRxnBounds(model,rxnName,0,'l');
0127         model = changeRxnBounds(model,rxnName, storageValue,'u');
0128     end
0129     rxnName = 'Retina_sink_crvnc(c)';
0130     model = changeRxnBounds(model,rxnName,0,'l');
0131     model = changeRxnBounds(model,rxnName, storageValue,'u');
0132     rxnName = 'Heart_sink_chol(c)';
0133     model = changeRxnBounds(model,rxnName,0,'l');
0134     model = changeRxnBounds(model,rxnName, storageValue,'u');
0135     rxnName = 'Brain_sink_crvnc(c)';
0136     model = changeRxnBounds(model,rxnName,0,'l');
0137     model = changeRxnBounds(model,rxnName, storageValue,'u');
0138     rxnName = 'Brain_sink_chol(c)';
0139     model = changeRxnBounds(model,rxnName,0,'l');
0140     model = changeRxnBounds(model,rxnName, storageValue,'u');
0141     rxnName = 'RBC_sink_glygn2(c)';
0142     model = changeRxnBounds(model,rxnName,0,'l');
0143     model = changeRxnBounds(model,rxnName, storageValue,'u');
0144     rxnName = 'Skin_sink_vitd3(c)';
0145     model = changeRxnBounds(model,rxnName,0,'l');
0146     model = changeRxnBounds(model,rxnName, storageValue,'u');
0147 elseif strcmp(feedingStatus,'fasting')
0148     for i = 1 : length(storedInLiver)
0149         rxnName = strcat('Liver_sink_',storedInLiver{i});
0150         model = changeRxnBounds(model,rxnName,fastingValue,'l');
0151         model = changeRxnBounds(model,rxnName,0,'u');
0152     end
0153     for i = 1 : length(StoreInKidney)
0154         rxnName = strcat('Kidney_sink_',StoreInKidney{i});
0155         model = changeRxnBounds(model,rxnName,fastingValue,'l');
0156         model = changeRxnBounds(model,rxnName,0,'u');
0157     end
0158     for i = 1 : length(StoreInMuscle)
0159         rxnName = strcat('Muscle_sink_',StoreInMuscle{i});
0160         model = changeRxnBounds(model,rxnName,fastingValue,'l');
0161         model = changeRxnBounds(model,rxnName,0,'u');
0162     end
0163     for i = 1 : length(storageAA)
0164         rxnName = strcat('Muscle_',storageAA{i});
0165         model = changeRxnBounds(model,rxnName,fastingValue,'l');
0166         model = changeRxnBounds(model,rxnName,0,'u');
0167     end
0168     for i = 1 : length(StoreInAdi)
0169         rxnName = strcat('Adipocytes_sink_',StoreInAdi{i});
0170         model = changeRxnBounds(model,rxnName,fastingValue,'l');
0171         model = changeRxnBounds(model,rxnName,0,'u');
0172     end
0173     rxnName = 'Retina_sink_crvnc(c)';
0174     model = changeRxnBounds(model,rxnName,fastingValue,'l');
0175     model = changeRxnBounds(model,rxnName,0,'u');
0176     rxnName = 'Heart_sink_chol(c)';
0177     model = changeRxnBounds(model,rxnName,fastingValue,'l');
0178     model = changeRxnBounds(model,rxnName,0,'u');
0179     rxnName = 'Brain_sink_crvnc(c)';
0180     model = changeRxnBounds(model,rxnName,fastingValue,'l');
0181     model = changeRxnBounds(model,rxnName,0,'u');
0182     rxnName = 'Brain_sink_chol(c)';
0183     model = changeRxnBounds(model,rxnName,fastingValue,'l');
0184     model = changeRxnBounds(model,rxnName,0,'u');
0185     rxnName = 'RBC_sink_glygn2(c)';
0186     model = changeRxnBounds(model,rxnName,fastingValue,'l');
0187     model = changeRxnBounds(model,rxnName,0,'u');
0188     rxnName = 'Skin_sink_vitd3(c)';
0189     model = changeRxnBounds(model,rxnName,fastingValue,'l');
0190     model = changeRxnBounds(model,rxnName,0,'u');
0191 end
0192 
0193 model.SetupInfo.FeedingStatus = feedingStatus;
```

---

Generated on Thu 14-May-2020 13:05:49 by **m2html** © 2005
