# Supplementary material for: Personalized whole‐body models integrate metabolism, physiology, and the gut microbiome
Source: Mol Syst Biol. 2020 May 28;16(5):e8982. doi: 10.15252/msb.20198982 (PMC7285886; doi:10.15252/msb.20198982)
Supplement: Supplementary file 22 — Dataset EV1 [file MSB-16-e8982-s022.zip › PSCM_toolbox/PSCM_toolbox_doc/src/setConstraints/setDietConstraints.html]

Description of setDietConstraints


# setDietConstraints

## PURPOSE

**This function sets diet constraints onto the bounds of the diet uptale**

## SYNOPSIS

**function model = setDietConstraints(model, Diet, factor)**

## DESCRIPTION

```
 This function sets diet constraints onto the bounds of the diet uptale
 reactions of a whole-body metabolic model.
 Units are given in mmol/day/person.

 function model = setDietConstraints(model, Diet, factor)

 INPUT
 model         model structure 
 Diet          Diet option: 'EUAverageDiet' (default)
 factor        value between 0 and 1; default is 1, i.e, 100% of the provided diet

 OUTPUT
 model         updated model strcution
 
 Ines Thiele 2016-2019
```

## CROSS-REFERENCE INFORMATION

This function calls:

- AGORAEssentialMetabolites This file contains a list of metabolites required by the AGORA microbial
- EUAverageDietNew Average European Diet defintion. For details, please see https://www.vmh.life/#nutrition
- HighFiberDiet High fiber diet defintion. For details, please see https://www.vmh.life/#nutrition
- HighProteinDiet High protein diet defintion. For details, please see https://www.vmh.life/#nutrition
- UnhealthyDiet Unhealthy diet defintion. For details, please see https://www.vmh.life/#nutrition
- VegetarianDiet Vegetarian diet defintion. For details, please see https://www.vmh.life/#nutrition

This function is called by:

- analyzeHMmodel This function performs host-microbiome optimization for a set of defined
- perform\_BMR\_newData This script repeats the simulation described in Thiele et al., "Personalized whole-body models integrate metabolism, physiology, and the gut microbiome", Method section 3.9.2 Validation of the parameters in an independent data set.
- perform\_sensi\_BMR\_all This script repeats the simulation described in Thiele et al.,
- runIEM\_HH This script predicts known biomarker metabolites in

## SOURCE CODE

```
0001 function model = setDietConstraints(model, Diet, factor)
0002 % This function sets diet constraints onto the bounds of the diet uptale
0003 % reactions of a whole-body metabolic model.
0004 % Units are given in mmol/day/person.
0005 %
0006 % function model = setDietConstraints(model, Diet, factor)
0007 %
0008 % INPUT
0009 % model         model structure
0010 % Diet          Diet option: 'EUAverageDiet' (default)
0011 % factor        value between 0 and 1; default is 1, i.e, 100% of the provided diet
0012 %
0013 % OUTPUT
0014 % model         updated model strcution
0015 %
0016 % Ines Thiele 2016-2019
0017 
0018 % define diet
0019 if ~exist('Diet','var')
0020     EUAverageDietNew;
0021 elseif strcmp(Diet,'EUAverageDiet')
0022     EUAverageDietNew;
0023 elseif strcmp(Diet,'HighFiberDiet')
0024     HighFiberDiet;
0025 elseif strcmp(Diet,'HighProteinDiet')
0026     HighProteinDiet;
0027 elseif strcmp(Diet,'UnhealthyDiet')
0028     UnhealthyDiet;
0029 elseif strcmp(Diet,'VegetarianDiet')
0030     VegetarianDiet;
0031 end
0032 
0033 if ~exist('factor','var')
0034     factor = 1; % 100% of the provided diet
0035 end
0036 microbiotaEnabling =1; % consider essential microbial metabolites to be added to diet
0037 
0038 % load essential metabolite list for AGORA models
0039 AGORAEssentialMetabolites;
0040 AGORAessential = regexprep(AGORAessential,'EX_','Diet_EX_');
0041 AGORAessential = regexprep(AGORAessential,'\[u\]','\[d\]');
0042 
0043 % set all  uptakes to 0
0044 tmp = strmatch('Diet_EX_',model.rxns);
0045 modelO = model;
0046 model.lb(tmp(1:end))=0;
0047 model.ub(tmp(1:end))=0;
0048 
0049 % ensure uptake of metabolites required for microbiota (may not all be
0050 % needed) -- I should really check which ones of those are need
0051 if microbiotaEnabling == 1
0052     MissingUptakes = setdiff(AGORAessential,Diet(:,1));
0053     % open uptake for those reactions
0054     % set constraints to a default
0055     model.lb(ismember(model.rxns,MissingUptakes))=-0.1;
0056 end
0057 
0058 % these compounds are in the diet and needed for the proper function of the
0059 % model but are not reported in our diet database
0060 if 1
0061     MissingDietCompounds={'Diet_EX_asn_L[d]'; 'Diet_EX_gln_L[d]';'Diet_EX_chol[d]';'Diet_EX_crn[d]';'Diet_EX_elaid[d]';...
0062         'Diet_EX_hdcea[d]';'Diet_EX_dlnlcg[d]';'Diet_EX_adrn[d]';'Diet_EX_hco3[d]';...
0063         %June 2nd 2017 debug for increased brain atp
0064         % adding these metabolites significantly incresed the DM_atp of brain
0065         % -- it would be very interesting to perform a sensitivity analysis and
0066         % see which metabolites have which effects --> could correlate with
0067         % brain activity and cognition/neurodegeneration
0068         % many of these are also microbially produced and should have a
0069         % positive effect.
0070         % DM_atp increased from 3600 to 5015
0071         'Diet_EX_sprm[d]'; 'Diet_EX_carn[d]';'Diet_EX_7thf[d]';...
0072         'Diet_EX_Lcystin[d]';%??
0073         'Diet_EX_hista[d]';'Diet_EX_orn[d]';...
0074         'Diet_EX_ptrc[d]';'Diet_EX_creat[d]';
0075         };
0076     
0077     model.lb(ismember(model.rxns,MissingDietCompounds))=-50;
0078     MissingDietCompounds={
0079         % 'Diet_EX_uri[d]'
0080         'Diet_EX_cytd[d]'
0081         % 'Diet_EX_gam[d]'
0082         %'Diet_EX_gal[d]'
0083         'Diet_EX_so4[d]'
0084         %'Diet_EX_fuc_L[d]'
0085         %'Colon_DM_Asn_X_Ser_Thr_ly_'
0086         %'Diet_EX_h[d]'
0087         
0088         };
0089     model.lb(ismember(model.rxns,MissingDietCompounds))=-50;
0090 
0091 model.lb(ismember(model.rxns,'Diet_EX_chol[d]'))=-41.251; %based on a daily intake of 396 mg in Av Am Diet per day (Sahoo 2013 paper)
0092 
0093 end
0094 
0095 % micronutrient - defined to have mole/day/person rate below 1e-6 mol/day/person
0096 % lower bounds will be relaxed by factor 10
0097 micronutrients ={%'Diet_EX_adpcbl[d]'
0098     % I changed the exchange ID since it was generated based on the
0099     % metabolite (ID adocbl). AH 16/12/01
0100     'Diet_EX_adocbl[d]'
0101     'Diet_EX_vitd2[d]'
0102     'Diet_EX_vitd3[d]'
0103     'Diet_EX_psyl[d]'
0104     'Diet_EX_gum[d]'
0105     'Diet_EX_bglc[d]'
0106     'Diet_EX_phyQ[d]'
0107     'Diet_EX_fol[d]'
0108     'Diet_EX_5mthf[d]'
0109     'Diet_EX_q10[d]'
0110     'Diet_EX_retinol_9_cis[d]'
0111     'Diet_EX_pydxn[d]'
0112     'Diet_EX_pydam[d]'
0113     'Diet_EX_pydx[d]'
0114     'Diet_EX_pheme[d]'
0115     'Diet_EX_ribflv[d]'
0116     'Diet_EX_thm[d]'
0117     % added 24.08.2016
0118     'Diet_EX_avite1[d]'
0119     'Diet_EX_pnto_R[d]'
0120     };
0121 %no uptake enforced but max uptake rate
0122 ions={    'Diet_EX_na1[d]'    %'0.056546816'
0123     'Diet_EX_cl[d]'    %'0.056412715'
0124     'Diet_EX_k[d]'    %'0.12020983'
0125     'Diet_EX_pi[d]'    %'0.007143207'
0126     'Diet_EX_zn2[d]'
0127     'Diet_EX_cu2[d]'
0128     };
0129 so4={
0130     %02.06.2017
0131     'Diet_EX_so4[d]' % see text above about addition of met for brain demand
0132     };
0133 
0134 for i = 1:  size(Diet,1)
0135     R = Diet{i,1};
0136     % exception for micronutrients to avoid numberical issues
0137     if ~isempty(find(ismember(micronutrients,R)))&& str2num(Diet{i,2})<=10
0138         model.lb(find(ismember(model.rxns,R))) = -10*factor;%-1.2*str2num(Diet{i,2})*factor;
0139     elseif ~isempty(find(ismember(micronutrients,R))) && str2num(Diet{i,2})>0.1
0140         model.lb(find(ismember(model.rxns,R))) = -1.2*str2num(Diet{i,2})*100*factor;
0141     elseif ~isempty(find(ismember(ions,R)))
0142         %  model.lb(find(ismember(model.rxns,R))) = -1.2*str2num(Diet{i,2});
0143         model.lb(find(ismember(model.rxns,R))) = -1.2*str2num(Diet{i,2})*100*factor;
0144     elseif ~isempty(find(ismember(so4,R)))
0145         model.lb(find(ismember(model.rxns,R))) = -1000*factor;
0146     else
0147         model.lb(find(ismember(model.rxns,R))) = -1.2*str2num(Diet{i,2})*factor;
0148     end
0149 end
0150 % do not enforce diet uptake --> set if statement to 0
0151 if 1
0152     for i = 1:size(Diet,1) % fine until 70
0153         R = Diet{i,1};
0154         if ~isempty(find(ismember(micronutrients,R)))
0155             model.ub(find(ismember(model.rxns,R))) =  -0.8*str2num(Diet{i,2})*factor;
0156         elseif ~isempty(find(ismember(ions,R)))
0157             model.ub(find(ismember(model.rxns,R))) = -0.8*str2num(Diet{i,2})*factor;
0158         else
0159             model.ub(find(ismember(model.rxns,R))) = -0.8*str2num(Diet{i,2})*factor;
0160         end
0161     end
0162 end
0163 
0164 model.SetupInfo.DietComposition = Diet;
0165
```

---

Generated on Thu 14-May-2020 13:05:49 by **m2html** © 2005
