# Supplementary material for: Personalized whole‐body models integrate metabolism, physiology, and the gut microbiome
Source: Mol Syst Biol. 2020 May 28;16(5):e8982. doi: 10.15252/msb.20198982 (PMC7285886; doi:10.15252/msb.20198982)
Supplement: Supplementary file 22 — Dataset EV1 [file MSB-16-e8982-s022.zip › PSCM_toolbox/PSCM_toolbox_doc/src/setConstraints/physiologicalConstraintsHMDBbased.html]

Description of physiologicalConstraintsHMDBbased


# physiologicalConstraintsHMDBbased

## PURPOSE

**This function applies constraints to the whole-body metabolic model**

## SYNOPSIS

**function modelConstraint = physiologicalConstraintsHMDBbased(model,IndividualParameters, ExclList, Type, InputData, Biofluid, setDefault,ExclMet,ExclMetAbbr)**

## DESCRIPTION

```
 This function applies  constraints to the whole-body metabolic model
 metabolite concentrations have to be given in uM
 organ weights have to be given in g
 Please note that reaction specific constraints are applied at the end of
 the function, which have been derived from the literature.

 function modelConstraint = physiologicalConstraintsHMDBbased(model,IndividualParameters, ExclList, Type, InputData, Biofluid, setDefault,ExclMet,ExclMetAbbr)

 INPUT
 model                     model structure
 IndividualParameters      Structure containing physiological parameters,
                           as generated in standardPhysiolDefaultParameters
 ExclList                  List of reaction(s) to which no updated bound
                           should be assigned to
 Type                      Input type (either 'xlsx' (default) --> loads by default
                           'Parsed_hmdbConc.xlsx' or 'direct'). If
                           'direct' InputData must be provided
 InputData                 first column corresponds to vmh id's of
                           metabolites, 2nd to data points (will be set as lb and ub)
 Biofluid                  'all' (default if type is xlsx). For direct:
                            'bc','u','csf'
 setDefault                If input data does not contain concentration information for a given metabolite
                           then a default concentration ranges will be used to calculate the constraints (default: 1)
                           Note that the default metabolite concentration
                           ranges are specified in IndividualParameters
                           for the different biofluid compartments.
 ExclMet                   Specify if certain metabolites, and thus their associated reactions, should be
                           excluded from the constraint application (default: 0)
 ExclMetAbbr               Provide list of metabolites that should be
                           excluded

 OUTPUT
 modelConstraint           model structure with updated constraints

 Ines Thiele, 2015-2019
```

## CROSS-REFERENCE INFORMATION

This function calls:

- OrganLists This file contains lists of ograns as they are used in the whole-body

This function is called by:

- analyzeHMmodel This function performs host-microbiome optimization for a set of defined
- perform\_BMR\_newData This script repeats the simulation described in Thiele et al., "Personalized whole-body models integrate metabolism, physiology, and the gut microbiome", Method section 3.9.2 Validation of the parameters in an independent data set.
- perform\_sensi\_BMR\_all This script repeats the simulation described in Thiele et al.,
- runIEM\_HH This script predicts known biomarker metabolites in
- individualizedLabReport This function computes personalized physiolgical parameters based on the

## SOURCE CODE

```
0001 function modelConstraint = physiologicalConstraintsHMDBbased(model,IndividualParameters, ExclList, Type, InputData, Biofluid, setDefault,ExclMet,ExclMetAbbr)
0002 % This function applies  constraints to the whole-body metabolic model
0003 % metabolite concentrations have to be given in uM
0004 % organ weights have to be given in g
0005 % Please note that reaction specific constraints are applied at the end of
0006 % the function, which have been derived from the literature.
0007 %
0008 % function modelConstraint = physiologicalConstraintsHMDBbased(model,IndividualParameters, ExclList, Type, InputData, Biofluid, setDefault,ExclMet,ExclMetAbbr)
0009 %
0010 % INPUT
0011 % model                     model structure
0012 % IndividualParameters      Structure containing physiological parameters,
0013 %                           as generated in standardPhysiolDefaultParameters
0014 % ExclList                  List of reaction(s) to which no updated bound
0015 %                           should be assigned to
0016 % Type                      Input type (either 'xlsx' (default) --> loads by default
0017 %                           'Parsed_hmdbConc.xlsx' or 'direct'). If
0018 %                           'direct' InputData must be provided
0019 % InputData                 first column corresponds to vmh id's of
0020 %                           metabolites, 2nd to data points (will be set as lb and ub)
0021 % Biofluid                  'all' (default if type is xlsx). For direct:
0022 %                            'bc','u','csf'
0023 % setDefault                If input data does not contain concentration information for a given metabolite
0024 %                           then a default concentration ranges will be used to calculate the constraints (default: 1)
0025 %                           Note that the default metabolite concentration
0026 %                           ranges are specified in IndividualParameters
0027 %                           for the different biofluid compartments.
0028 % ExclMet                   Specify if certain metabolites, and thus their associated reactions, should be
0029 %                           excluded from the constraint application (default: 0)
0030 % ExclMetAbbr               Provide list of metabolites that should be
0031 %                           excluded
0032 %
0033 % OUTPUT
0034 % modelConstraint           model structure with updated constraints
0035 %
0036 % Ines Thiele, 2015-2019
0037 
0038 modelConstraint = model;
0039 
0040 setLB = 0;
0041 
0042 
0043 %% Input physiological data
0044 %standardPhysiolDefaultParameters;
0045 sex = IndividualParameters.sex;
0046 CardiacOutput = IndividualParameters.CardiacOutput;
0047 
0048 % default maximum concentration of a metabolite in blood plasma
0049 MConDefaultBc = IndividualParameters.MConDefaultBc;
0050 
0051 % default maximum concentration of a metabolite in csf
0052 MConDefaultCSF = IndividualParameters.MConDefaultCSF;
0053 
0054 % default maximum concentration of a metabolite in Ur
0055 MConDefaultUrMax = IndividualParameters.MConDefaultUrMax;
0056 MConDefaultUrMin = IndividualParameters.MConDefaultUrMin;
0057 
0058 % creatinine concentration in urine
0059 MConDefaultUrCreatinineMax = IndividualParameters.MConUrCreatinineMax;
0060 MConDefaultUrCreatinineMin = IndividualParameters.MConUrCreatinineMin;
0061 
0062 % CSF Flow rate
0063 CSFFlowRate = IndividualParameters.CSFFlowRate;
0064 
0065 % CSF Blood Flow rate
0066 CSFBloodFlowRate = IndividualParameters.CSFBloodFlowRate;
0067 
0068 % Urine flow rate
0069 UrFlowRate = IndividualParameters.UrFlowRate;
0070 
0071 % Hematocrite
0072 Hematocrit = IndividualParameters.Hematocrit;
0073 
0074 % lower concentration limit for setting a concentration constraint
0075 MinConcConstraint = 5;
0076 MaxConcConstraint = 50;
0077 bloodFlowData = IndividualParameters.bloodFlowData;
0078 bloodFlowPercCol = IndividualParameters.bloodFlowPercCol;
0079 bloodFlowOrganCol = IndividualParameters.bloodFlowOrganCol;
0080 % List of organs
0081 OrganLists;
0082 
0083 %% calculate GFR = Glomerular filtration rate
0084 % the filtration fraction should be 20% of the renal plasma flow
0085 %GlomerularFiltrationRate = IndividualParameters.GlomerularFiltrationRate; % in ml/min
0086 RenalFiltrationFraction  = 0.2; %20%
0087 
0088 % blood flow percentage that Kidney gets
0089 if strcmp(sex,'male')
0090     BK = bloodFlowData{strmatch('Kidney',bloodFlowData(:,1),'exact'),bloodFlowPercCol(1)};
0091 elseif strcmp(sex,'female')
0092     BK = bloodFlowData{strmatch('Kidney',bloodFlowData(:,1),'exact'),bloodFlowPercCol(2)};
0093     % BK = bloodFlowData{strmatch('Kidney',bloodFlowData(:,1),'exact'),4};
0094 end
0095 BK = str2num(BK(2:end-1));
0096 RenalFlowRate=BK*CardiacOutput*(1-Hematocrit); % k_plasma_organ in ml/min
0097 GlomerularFiltrationRate = RenalFlowRate*RenalFiltrationFraction;% in ml/min
0098 %% read data
0099 % read metabolic concentrations from HMDB if no input data are defined
0100 if ~exist('InputData','var') || length(InputData)==0
0101     Type = 'HMDB';
0102     Biofluid = 'all';
0103 end
0104 if ~exist('Type','var')|| strcmp(Type,'')
0105     Type = 'HMDB';
0106 end
0107 if ~exist('ExclList','var')
0108     ExclList = '';
0109 end
0110 % default concentrations will be applied as constraints if not specified
0111 % differently
0112 if ~exist('setDefault','var')
0113     setDefault = 1; % default is true
0114 end
0115 
0116 if ~exist('ExclMet','var')
0117     ExclMet =0; % no further excluded metabolite
0118     ExclMetAbbr = '';
0119 end
0120 if strcmp(Type,'HMDB')
0121     % BLOOD
0122     Biofluid = 'all';
0123     fileName='NormalBloodConcExtractedHMDB.txt';
0124     [Data] =importdata(fileName);
0125     
0126     % find start/header of data - Blood
0127     % find data start
0128     Start= (find(~cellfun(@isempty,strfind(Data.textdata(:,1),'####'))))+1;
0129     % find rxn abbr
0130     VMHIDCol= (find(~cellfun(@isempty,strfind(Data.textdata(Start,:),'VMH'))));
0131     MetConMin = (Data.data(:,1));% min = 1st col, max = 2nd col
0132     MetConMin = cellstr(num2str(MetConMin));
0133     MetConMin = regexprep(MetConMin,' ','');
0134     for j = 1 : size(MetConMin,1)
0135         MetConMin{j,1} = (MetConMin(j,1));
0136     end
0137     
0138     MetConMax = (Data.data(:,2));% min = 1st col, max = 2nd col
0139     MetConMax = cellstr(num2str(MetConMax));
0140     MetConMax = regexprep(MetConMax,' ','');
0141     for j = 1 : size(MetConMax,1)
0142         MetConMax{j,1} = (MetConMax(j,1));
0143     end
0144     metConcDataBc=[Data.textdata(Start+1:end,VMHIDCol) MetConMin MetConMax];
0145     maxConcColBc = 3;
0146     minConcColBc = 2;
0147     VMHIDCol = 1;
0148     
0149     % CSF;
0150     fileName='NormalCSFConcExtractedHMDB.txt';
0151     [Data] =importdata(fileName);
0152     
0153     % find start/header of data - Blood
0154     % find data start
0155     Start= (find(~cellfun(@isempty,strfind(Data.textdata(:,1),'####'))))+1;
0156     % find rxn abbr
0157     VMHIDCol= (find(~cellfun(@isempty,strfind(Data.textdata(Start,:),'VMH'))));
0158     MetConMin = (Data.data(:,1));% min = 1st col, max = 2nd col
0159     MetConMin = cellstr(num2str(MetConMin));
0160     MetConMin = regexprep(MetConMin,' ','');
0161     for j = 1 : size(MetConMin,1)
0162         MetConMin{j,1} = (MetConMin(j,1));
0163     end
0164     
0165     MetConMax = (Data.data(:,2));% min = 1st col, max = 2nd col
0166     MetConMax = cellstr(num2str(MetConMax));
0167     MetConMax = regexprep(MetConMax,' ','');
0168     for j = 1 : size(MetConMax,1)
0169         MetConMax{j,1} = (MetConMax(j,1));
0170     end
0171     metConcDataCSF=[Data.textdata(Start+1:end,VMHIDCol) MetConMin MetConMax];
0172     maxConcColCSF = 3;
0173     minConcColCSF = 2;
0174     VMHIDCol = 1;
0175     
0176     % URINE
0177     fileName='NormalUrineConcExtractedHMDB.txt';
0178     [Data] =importdata(fileName);
0179     
0180     % find start/header of data -
0181     % find data start
0182     Start= (find(~cellfun(@isempty,strfind(Data.textdata(:,1),'####'))))+1;
0183     % find rxn abbr
0184     VMHIDCol= (find(~cellfun(@isempty,strfind(Data.textdata(Start,:),'VMH'))));
0185     MetConMin = (Data.data(:,1));% min = 1st col, max = 2nd col
0186     MetConMin = cellstr(num2str(MetConMin));
0187     MetConMin = regexprep(MetConMin,' ','');
0188     for j = 1 : size(MetConMin,1)
0189         MetConMin{j,1} = (MetConMin(j,1));
0190     end
0191     
0192     MetConMax = (Data.data(:,2));% min = 1st col, max = 2nd col
0193     MetConMax = cellstr(num2str(MetConMax));
0194     MetConMax = regexprep(MetConMax,' ','');
0195     for j = 1 : size(MetConMax,1)
0196         MetConMax{j,1} = (MetConMax(j,1));
0197     end
0198     metConcDataUr=[Data.textdata(Start+1:end,VMHIDCol) MetConMin MetConMax];
0199     maxConcColUr = 3;
0200     minConcColUr = 2;
0201     VMHIDCol = 1;
0202     
0203 elseif strcmp(Type,'direct')%direct data input
0204     VMHIDCol = 1; % first column corresponds to vmh id's, 2nd to data points (min), 3rd to data points (max)
0205     setLB = 1;
0206     if strcmp(Biofluid,'bc')%blood data
0207         minConcColBc = 2;
0208         maxConcColBc = 3;
0209         metConcDataBc = InputData;
0210     elseif strcmp(Biofluid,'csf')%blood data
0211         minConcColCSF = 2;
0212         maxConcColCSF = 3;
0213         metConcDataCSF = InputData;
0214     elseif strcmp(Biofluid,'u')%blood data
0215         minConcColUr = 2;
0216         maxConcColUr = 3;
0217         metConcDataUr = InputData;
0218     end
0219 end
0220 %% compute constraints
0221 % Feher, p. 550 Q8!
0222 % the following assumptions are made
0223 % 1. steady-state
0224 % 2. transport is bulk flow limited, not diffusion limited,  which is at
0225 %    least true for higher blood flow rates
0226 % 3. the metabolites is maximally consumed by tissue (gives an upper bound),
0227 %    for metabolites for which the venal concentration is known the
0228 %    difference between arterial and venal concentration should be rather used.
0229 % Equation:
0230 % Q = (ca-cv)*Qv - Feher, p 550, Ex 8
0231 % vmax_met_organ = conc_met_max * k_blood_organ % no organ scaling
0232 % necessary as those numbers are already adjusted to organs (in part to
0233 % weight but also requirements)
0234 
0235 % cardiac output and organ-specific blood flow rate
0236 for i = 1 : length(OrgansListExt)
0237     BloodFlowOrgan(i,1)=OrgansListExt(i); %percentage of blood flow going to each organ
0238     tmp = strmatch(OrgansListExt(i),bloodFlowData(:,bloodFlowOrganCol),'exact');
0239     if ~isempty(tmp) && tmp>0
0240         if strcmp(IndividualParameters.sex,'male') % use first col
0241             B = bloodFlowData{tmp,bloodFlowPercCol(1)};
0242         elseif strcmp(IndividualParameters.sex,'female') % use 2nd col
0243             B = bloodFlowData{tmp,bloodFlowPercCol(2)};
0244         end
0245         B = str2num(B(2:end-1));
0246         if  ~isempty(B)
0247             BloodFlowRate(i,1)=(B)*CardiacOutput; % k_blood_organ in ml/min
0248             PlasmaFlowRate(i,1)=(B)*CardiacOutput*(1-Hematocrit); % k_plasma_organ in ml/min
0249         else
0250             % if no information is provided for percentage, assume 1%
0251             BloodFlowRate(i,1)=0.01*CardiacOutput; % k_blood_organ in ml/min
0252             PlasmaFlowRate(i,1)=0.01*CardiacOutput*(1-Hematocrit); % k_blood_organ in ml/min
0253         end
0254     elseif strcmp('BBB',OrgansListExt(i))% exception of BBB which gets the flow rate of Scord and Brain
0255         Scord = strmatch('Scord',bloodFlowData(:,bloodFlowOrganCol),'exact');
0256         Brain = strmatch('Brain',bloodFlowData(:,bloodFlowOrganCol),'exact');
0257         
0258         if strcmp(IndividualParameters.sex,'male') % use first col
0259             BScord = bloodFlowData{Scord,bloodFlowPercCol(1)};
0260             BBrain = bloodFlowData{Brain,bloodFlowPercCol(1)};
0261         elseif strcmp(IndividualParameters.sex,'female') % use 2nd col
0262             BScord = bloodFlowData{Scord,bloodFlowPercCol(2)};
0263             BBrain = bloodFlowData{Brain,bloodFlowPercCol(2)};
0264         end
0265         
0266         BScord = str2num(BScord(2:end-1));
0267         BBrain = str2num(BBrain(2:end-1));
0268         B = BBrain + BScord;
0269         
0270         BloodFlowRate(i,1)=(B)*CardiacOutput; % k_blood_organ in ml/min
0271         PlasmaFlowRate(i,1)=(B)*CardiacOutput*(1-Hematocrit); % k_plasma_organ in ml/min
0272     else
0273         % if no information is provided for percentage, assume 1%
0274         BloodFlowRate(i,1)=0.01*CardiacOutput; % k_blood_organ in ml/min
0275         PlasmaFlowRate(i,1)=0.01*CardiacOutput*(1-Hematocrit); % k_blood_organ in ml/min
0276     end
0277 end
0278 
0279 % Organs excluded from secretion into [bc]
0280 ExclOrgan={'sIEC', 'Colon','Spleen','Pancreas','Gall','Brain'};
0281 
0282 % compute maximal possible uptake and secretion rate for each metabolite in
0283 % each organ
0284 if strcmp( Biofluid, 'bc') || strcmp( Biofluid, 'all')
0285     for i = 1 : length(OrgansListExt)
0286         % find uptake and secretion reactions
0287         if ~isempty(strmatch('BBB',OrgansListExt{i})) %BBB for brain constraints
0288             ExR = find(~cellfun(@isempty,strfind(modelConstraint.rxns,'[CSF]upt'))); % uptake from [bc] into [csf] only
0289         else
0290             ExR = strmatch(strcat(OrgansListExt{i},'_EX_'),modelConstraint.rxns);
0291         end
0292         if ~isempty(ExR)
0293             for j = 1 : length(ExR)
0294                 % ensure that the exchange is FROM BLOOD
0295                 if (length(strfind(modelConstraint.rxns{ExR(j)},'[bc]'))>0 || length(strfind(modelConstraint.rxns{ExR(j)},'[CSF]upt'))>0) ...
0296                         && length(strfind(modelConstraint.rxns{ExR(j)},'_o2s(e)'))==0    ...
0297                         && length(strfind(modelConstraint.rxns{ExR(j)},'_h2o(e)'))==0 ...
0298                         && length(strfind(modelConstraint.rxns{ExR(j)},'_H2O[CSF]upt'))==0 %...
0299                     %% allow to exclude more metabolites from being constrained
0300                     % not nice programming but I had problems with other
0301                     % versions
0302                     goOn = 1;
0303                     if ExclMet ==1 %length(strfind(modelConstraint.rxns{ExR(j)},ExclMetAbbr))==0
0304                         for q =1 : length(ExclMetAbbr)
0305                             if length(strfind(modelConstraint.rxns{ExR(j)},ExclMetAbbr{q}))>0 % metabolite is found
0306                                 goOn = 0;
0307                             end
0308                         end
0309                     end
0310                     if goOn ==1
0311                         %       && length(strfind(modelConstraint.rxns{ExR(j)},'_co2(e)'))==0
0312                         %&& length(strfind(modelConstraint.rxns{ExR(j)},'_o2(e)'))==0 ... %no oxygen constraint
0313                         
0314                         %  && length(strfind(modelConstraint.rxns{ExR(j)},'_aicar(e)'))==0    ...
0315                         % && length(strfind(modelConstraint.rxns{ExR(j)},'_CE2705(e)'))==0   % ...
0316                         
0317                         %&& length(strfind(modelConstraint.rxns{ExR(j)},'_h2o2(e)'))==0 % avoids setting uptake for o2s, co2, h2o2
0318                         %&& length(strfind(modelConstraint.rxns{ExR(j)},'_o2(e)'))==0 ... %no oxygen constraint
0319                         
0320                         % all reactions are written such that negative flux means
0321                         % uptake from (bc) and positive flux corresponds to secretion
0322                         % into (bc)
0323                         %
0324                         % get metabolite associated with reaction
0325                         ExM = modelConstraint.mets(find(modelConstraint.S(:,ExR(j))>0));
0326                         MCon = [];
0327                         if ~isempty(ExM)
0328                             % KIDNEY IS TREATED DIFFERENTLY - checked
0329                             % 29.04.2016 - IT
0330                             clear X
0331                             if ~isempty(strmatch(OrgansListExt(i),'Kidney','exact')) && length(strfind(modelConstraint.rxns{ExR(j)},'[bcK]'))==0
0332                                 % get maximal concentration for metabolite
0333                                 X = find(ismember(strcat(metConcDataBc(:,VMHIDCol),'[bc]'),ExM));
0334                                 clear MConMin MConMax
0335                                 if isempty(X) && ~strcmp(Type,'direct') % no concentration range/maximum defined in input data
0336                                     MConMin = 0;
0337                                     MConMax =  MConDefaultBc;
0338                                 elseif ~isempty(X)
0339                                     MConMin1 = metConcDataBc{X,minConcColBc};
0340                                     MConMax1 = metConcDataBc{X,maxConcColBc};
0341                                     if ischar(MConMin1)
0342                                         MConMin = str2num(MConMin1(2:end-1));
0343                                         MConMax = str2num(MConMax1(2:end-1));
0344                                     elseif iscell(MConMin1)
0345                                         MConMin = MConMin1{1};
0346                                         MConMin = str2num(MConMin);
0347                                         MConMax = MConMax1{1};
0348                                         MConMax = str2num(MConMax);
0349                                     else
0350                                         MConMin = MConMin1;
0351                                         MConMax = MConMax1;
0352                                     end
0353                                 else
0354                                     continue;
0355                                 end
0356                                 % MCon is in (umol/L) --> : 1000 to be in mmol/L
0357                                 % PlasmaFlowRate is in (ml/min)
0358                                 % PlasmaFlowRate*60*24/1000 (L/day)
0359                                 % Q = (ca-cv)*Qv; where ca is the aterial
0360                                 % concentration, which is typically not measured, and
0361                                 % cv the venous concentration, reported in HMDB and
0362                                 % measured in general experiments.
0363                                 % in the kidney the flux can go only from
0364                                 % [e]<--[bc] !!!!!!
0365                                 
0366                                 %  UPPER BOUND
0367                                 MSecretRateKidney = 1*(MConMin/1000)*GlomerularFiltrationRate*60*24/1000; % in mmol/day/person
0368                                 R = {'Kidney_EX_na1(e)_[bc]'
0369                                     'Kidney_EX_hco3(e)_[bc]'
0370                                     'Kidney_EX_urea(e)_[bc]'
0371                                     'Kidney_EX_k(e)_[bc]'
0372                                     'Kidney_EX_cl(e)_[bc]'
0373                                     'Kidney_EX_ca2(e)_[bc]'
0374                                     'Kidney_EX_HC02172(e)_[bc]'
0375                                     'Kidney_EX_avite1(e)_[bc]'
0376                                     };
0377                                 R = unique([R;ExclList]);
0378                                 if ~ismember(modelConstraint.rxns(ExR(j)),R)
0379                                     if setLB == 1
0380                                         modelConstraint.ub(ExR(j)) = -MSecretRateKidney; % maximal possible secretion rate
0381                                     else
0382                                         if MConMax>=MaxConcConstraint % set lower constraint if max is higher or equal to MinConcConstraint
0383                                             modelConstraint.ub(ExR(j)) = -MSecretRateKidney; % maximal possible secretion rate
0384                                         else
0385                                             modelConstraint.ub(ExR(j)) = 0; % maximal possible secretion rate
0386                                         end
0387                                     end
0388                                 else
0389                                     modelConstraint.ub(ExR(j)) = 0;
0390                                 end
0391                                 % LOWER BOUND
0392                                 MSecretRateKidney = 1*(MConMax/1000)*GlomerularFiltrationRate*60*24/1000; % in mmol/day/person
0393                                 
0394                                 modelConstraint.lb(ExR(j)) = -MSecretRateKidney; % maximal possible secretion rate
0395                                 
0396                             else
0397                                 %% checked this part of the code - 29.04. IT
0398                                 % get maximal concentration for metabolite
0399                                 X = find(ismember(strcat(metConcDataBc(:,VMHIDCol),'[bc]'),ExM));
0400                                    if isempty(X) %co2 and o2 are now getting in straight from rbc
0401                             X = find(ismember(strcat('RBC_',metConcDataBc(:,VMHIDCol),'[bc]'),ExM));
0402                             end
0403                                 clear MCon
0404                                 if isempty(X) && setDefault == 1 && ~strcmp(Type,'direct')% no concentration range/maximum defined in input data; only if requested
0405                                     MCon =  MConDefaultBc;
0406                                 elseif ~isempty(X)
0407                                     MCon1 = metConcDataBc{X,maxConcColBc};
0408                                     if ischar(MCon1)
0409                                         MCon = str2num(MCon1(2:end-1));
0410                                     elseif iscell(MCon1)
0411                                         MCon = MCon1{1};
0412                                         MCon = str2num(MCon);
0413                                     else
0414                                         MCon = MCon1;
0415                                     end
0416                                 else
0417                                     continue;
0418                                 end
0419                                 R = {
0420                                     % added to the list of  rxns excluded to be
0421                                     % constraint - otw infeasible - Nov 2017 -
0422                                     % IT
0423                                     'BBB_NH4[CSF]upt'
0424                                     'BBB_CHOL[CSF]upt'
0425                                     'BBB_PI[CSF]upt'
0426                                     'BBB_STRDNC[CSF]upt'
0427                                     'BBB_HC00250[CSF]upt'
0428                                     'BBB_PYDXN[CSF]upt'
0429                                     'BBB_5MTHF[CSF]upt'
0430                                     'BBB_SO3[CSF]upt'
0431                                     
0432                                     };
0433                                 
0434                                 if ~ismember(modelConstraint.rxns(ExR(j)),R)
0435                                     % MCon is in (umol/L) --> : 1000 to be in mmol/L
0436                                     % PlasmaFlowRate is in (ml/min)
0437                                     % PlasmaFlowRate*60*24/1000 (L/day)
0438                                     % Q = (ca-cv)*Qv; where ca is the aterial
0439                                     % concentration, which is typically not measured, and
0440                                     % cv the venous concentration, reported in HMDB and
0441                                     % measured in general experiments. ca is assumed to be
0442                                     % 30% higher than cv, allowing the tissue to take up
0443                                     % maximally 30% of the maximally reported cv value
0444                                     if ~isempty(MCon)
0445                                         %  MUptakeRateBc = ((MCon*(100/70)-MCon)/1000)*PlasmaFlowRate(i,1)*60*24/1000; % in mmol/day/person
0446                                         MUptakeRateBc = ((MCon)/1000)*PlasmaFlowRate(i,1)*60*24/1000; % in mmol/day/person
0447                                         if  modelConstraint.lb(ExR(j)) < 0;
0448                                             if MCon>1e-3%abs(MUptakeRateBc)>1e-3; % at least 1 nM
0449                                                 modelConstraint.lb(ExR(j)) = -1*MUptakeRateBc; % maximal possible uptake rate
0450                                             else
0451                                                 MUptakeRateBc = ((1e-3)/1000)*PlasmaFlowRate(i,1)*60*24/1000; % in mmol/day/person
0452                                                 
0453                                                 modelConstraint.lb(ExR(j)) = -1*MUptakeRateBc;
0454                                             end
0455                                         end
0456                                         % I cannot set this constraint as an organ
0457                                         % could secrete a metabolite at a higher
0458                                         % local concentration than in the blood but
0459                                         % this gets then balance through the
0460                                         % constribution (or rather lack of) by other
0461                                         % organs
0462                                         %                                 % allowing the tissue to secrete maximally 30% of the maximally reported cv value
0463                                         %                                 if isempty(strmatch(OrgansListExt(i),ExclOrgan,'exact')) &&  modelConstraint.ub(ExR(j))>0
0464                                         %                                     % MSecretRateBc = -1*((MCon*(100/130)-MCon)/1000)*PlasmaFlowRate(i,1)*60*24/1000; % in mmol/day/person
0465                                         %                                     MSecretRateBc = ((MCon)/1000)*PlasmaFlowRate(i,1)*60*24/1000; % in mmol/day/person
0466                                         %                                     % secretion rate does not apply to organs that are only taking up from [bc]
0467                                         %                                     if abs(MCon)>1e-3; % at least 1 nM
0468                                         %                                         modelConstraint.ub(ExR(j)) = MSecretRateBc; % maximal possible secretion rate
0469                                         %                                     else
0470                                         %                                          MSecretRateBc = ((1e-3)/1000)*PlasmaFlowRate(i,1)*60*24/1000; % in mmol/day/person
0471                                         %
0472                                         %                                         modelConstraint.ub(ExR(j)) = MSecretRateBc; % maximal possible secretion rate
0473                                         %                                     end
0474                                         %                                 end0
0475                                     end
0476                                 end
0477                                 
0478                             end
0479                         else
0480                             modelConstraint.rxns(ExR(j));
0481                         end
0482                     end
0483                 end
0484             end
0485         end
0486     end
0487     
0488 end
0489 %% BBB/Brain
0490 % compute maximal possible uptake and secretion rate for each metabolite in
0491 % each organ
0492 if strcmp( Biofluid, 'csf') || strcmp( Biofluid, 'all')
0493     for i = 1 : length(OrgansListExt)
0494         % find uptake and secretion reactions
0495         ExR = strmatch('BBB_',modelConstraint.rxns);
0496         if ~isempty(ExR)
0497             for j = 1 : length(ExR)
0498                 % ensure that only the export (exp) from csf-> bc receives
0499                 % constraints
0500                 if length(strfind(modelConstraint.rxns{ExR(j)},'[CSF]'))>0 && length(strfind(modelConstraint.rxns{ExR(j)},'exp'))>0 && length(strfind(modelConstraint.rxns{ExR(j)},'_o2(e)'))==0 ... %no oxygen constraint
0501                         && length(strfind(modelConstraint.rxns{ExR(j)},'_o2s(e)'))==0   && length(strfind(modelConstraint.rxns{ExR(j)},'_co2(e)'))==0
0502                     % all reactions are written such that negative flux means
0503                     % uptake from (bc) and positive flux corresponds to secretion
0504                     % into (bc)
0505                     
0506                     % get metabolite associated with reaction
0507                     ExM = modelConstraint.mets(find(modelConstraint.S(:,ExR(j))<0)); % reactions are written as [csf] <=> [bc]
0508                     if ~isempty(ExM)
0509                         % get maximal concentration for metabolite
0510                         X = find(ismember(strcat(metConcDataCSF(:,VMHIDCol),'[csf]'),ExM));
0511                         MConMin = [];
0512                         MConMax = [];
0513                         if isempty(X) && ~strcmp(Type,'direct') % no concentration range/maximum defined in input data
0514                             MConMin = 0;
0515                             MConMax =  MConDefaultCSF;
0516                         elseif ~isempty(X)
0517                             MConMin1 = metConcDataCSF{X,minConcColCSF};
0518                             MConMax1 = metConcDataCSF{X,maxConcColCSF};
0519                             if ischar(MConMin1)
0520                                 MConMin = str2num(MConMin1(2:end-1));
0521                                 MConMax = str2num(MConMax1(2:end-1));
0522                             elseif iscell(MConMin1)
0523                                 MConMin = MConMin1{1};
0524                                 MConMin = str2num(MConMin);
0525                                 MConMax = MConMax1{1};
0526                                 MConMax = str2num(MConMax);
0527                             else
0528                                 MConMin = MConMin1;
0529                                 MConMax = MConMax1;
0530                             end
0531                         else
0532                             continue;
0533                         end
0534                         if ~isempty(MConMin)
0535                             % MCon is in (umol/L) --> : 1000 to be in mmol/L
0536                             % PlasmaFlowRate is in (ml/min)
0537                             % PlasmaFlowRate*60*24/1000 (L/day)
0538                             % Q = (ca-cv)*Qv; where ca is the aterial
0539                             % concentration, which is typically not measured, and
0540                             % cv the venous concentration, reported in HMDB and
0541                             % measured in general experiments.
0542                             % LOWER BOUND
0543                             % flux will be positive as reaction is written as
0544                             % csf --> bc
0545                             MSecretRateCSF = (MConMin/1000)*CSFBloodFlowRate*60*24/1000; % in mmol/day/person
0546                             R = { 'na1[csf]'
0547                                 'cl[csf]'
0548                                 'k[csf]'
0549                                 'h2o[csf]'
0550                                 'sucsal[csf]'
0551                                 'ca2[csf]'
0552                                 'ser_D[csf]'};
0553                             if setLB == 1
0554                                 modelConstraint.lb(ExR(j)) = MSecretRateCSF; % maximal possible secretion rate
0555                             else
0556                                 if MConMin>=MinConcConstraint && MConMax>=MaxConcConstraint && isempty(find(ismember(R,ExM))) &&isempty(find(ismember(ExclList,modelConstraint.rxns(ExR(j))))) % || ismember(MustBeInCSF,ExM) % ismember(MustBeInCSF,ExM)
0557                                     modelConstraint.lb(ExR(j)) = MSecretRateCSF; % maximal possible uptake rate
0558                                 else
0559                                     modelConstraint.lb(ExR(j)) = 0;
0560                                 end
0561                             end
0562                             % UPPER BOUND
0563                             MSecretRateCSF = (MConMax/1000)*CSFFlowRate*60*24/1000; % in mmol/day/person
0564                             modelConstraint.ub(ExR(j)) = MSecretRateCSF; % maximal possible secretion rate
0565                             
0566                         end
0567                     else
0568                         modelConstraint.rxns(ExR(j));
0569                         
0570                     end
0571                 end
0572                 
0573             end
0574         end
0575     end
0576 end
0577 %% Urine excretion
0578 % constraints are set on Exchange reactions for urine
0579 if strcmp( Biofluid, 'u') || strcmp( Biofluid, 'all')
0580     
0581     % convert creatinine from mg/dL into mmol/L
0582     MWCreat = 113.1179;% g/mol
0583     MConDefaultUrCreatinineMax = MConDefaultUrCreatinineMax*10/MWCreat;
0584     MConDefaultUrCreatinineMin = MConDefaultUrCreatinineMin*10/MWCreat;
0585     ExR = strmatch('EX_',modelConstraint.rxns);
0586     if ~isempty(ExR)
0587         for j = 1 : length(ExR)
0588             if length(strfind(modelConstraint.rxns{ExR(j)},'[u]'))>0 && length(strfind(modelConstraint.rxns{ExR(j)},'_o2(e)'))==0 ... %no oxygen constraint
0589                     && length(strfind(modelConstraint.rxns{ExR(j)},'_o2s(e)'))==0   && length(strfind(modelConstraint.rxns{ExR(j)},'_co2(e)'))==0
0590                 
0591                 % all reactions are written such that positive flux corresponds to secretion
0592                 % into urine (u)
0593                 %
0594                 % get metabolite associated with reaction
0595                 ExM = modelConstraint.mets(find(modelConstraint.S(:,ExR(j))<0)); % this is a typical exchange reaction
0596                 MConMin = [];
0597                 MConMax = [];
0598                 if ~isempty(ExM)
0599                     % get maximal concentration for metabolite
0600                     X = find(ismember(strcat(metConcDataUr(:,VMHIDCol),'[u]'),ExM));
0601                     if isempty(X) && ~strcmp(Type,'direct')%&& setDefault == 1 % no concentration range/maximum defined in input data, only if requested
0602                         MConMin =  MConDefaultUrMin;
0603                         MConMax =  MConDefaultUrMax;
0604                     elseif ~isempty(X)
0605                         MConMin1 = metConcDataUr{X,minConcColUr};
0606                         MConMax1 = metConcDataUr{X,maxConcColUr};
0607                         if ischar(MConMin1)
0608                             MConMin = str2num(MConMin1(2:end-1));
0609                             MConMax = str2num(MConMax1(2:end-1));
0610                         elseif iscell(MConMin1)
0611                             MConMin = MConMin1{1};
0612                             MConMin = str2num(MConMin);
0613                             MConMax = MConMax1{1};
0614                             MConMax = str2num(MConMax);
0615                         else
0616                             MConMin = MConMin1;
0617                             MConMax = MConMax1;
0618                         end
0619                     else
0620                         continue;
0621                     end
0622                     % Urine excretion
0623                     % lower bound based on min concentration
0624                     if ~isempty(MConMin) && ~isempty(MConMax)
0625                         MSecrRateUrLB = (MConMin/1000)*MConDefaultUrCreatinineMin*UrFlowRate/1000; % in mmol/day/person
0626                         % upper bound based on max concentration
0627                         MSecrRateUrUB = (MConMax/1000)*MConDefaultUrCreatinineMax*UrFlowRate/1000; % in mmol/day/person
0628                         R = { 'EX_na1[u]'
0629                             'EX_cl[u]'
0630                             'EX_k[u]'
0631                             'EX_ca2[u]'
0632                             % non-unique list!
0633                             %'EX_aldstrn[u]'
0634                             %         'EX_tststerone[u]'
0635                             %         'EX_pydxn[u]'
0636                             %         'EX_3moxtyr[u]'
0637                             %                'EX_cl[u]'
0638                             %               'EX_k[u]'
0639                             %         %         %   'EX_nh4[u]'
0640                             %         'EX_sphgn[u]' % i dont think that this metabolite is routinely secreted
0641                             %         'EX_sphings[u]'
0642                             %         'EX_csn[u]'
0643                             %         'EX_arab_L[u]'
0644                             %         'EX_tststerone[u]'
0645                             %         'EX_tststerone[u]'
0646                             %         'EX_pydxn[u]'
0647                             %         'EX_mma[u]'%Methylamine occurs endogenously from amine catabolism and its tissue levels increase in some pathological conditions, including diabetes.
0648                             %        'EX_tsul[u]'%Thiosulfate occurs naturally in hot springs and geysers, and is produced by certain biochemical processes. In the body, thiosulfate converts small amounts of cyanide ion into harmless products and plays a role in the biosynthesis of cysteine, a sulfur-containing amino acid that locks proteins into their correct three-dimensional shapes. Thiosulfate is not found in large quantities in nature.
0649                             %
0650                             
0651                             % metabolites with lower bound that is non-zero
0652                             % in data but should be not set as lb
0653                             % constraints
0654                             'EX_C05767[u]' %Uroporphyrin I
0655                             'EX_C05770[u]' %Coproporphyrin III
0656                             'EX_C05302[u]'% 2-Methoxyestradiol (2ME2) is a drug that prevents the formation of new blood vessels
0657                             'EX_trypta[u]'%Tryptamine is a monoamine compound that is common precursor molecule to many hormones and neurotransmitters
0658                             'EX_ppbng[u]'% porphobilinogen is produced in excess and excreted in the urine in acute intermittent porphyria and several other porphyrias.
0659                             'EX_13dampp[u]'%  It is a catabolic byproduct of spermidine. "The excretion of these  substances is usually very small compared to the respective amino acids. "http://www.sciencedirect.com/science/article/pii/0009898171904426
0660                             'EX_mhista[u]' %The primary application of urinary N-methylhistamine (NMH) testing is in the diagnosis and monitoring of mast-cell disorders, including mastocytosis, anaphylaxis, and other severe systemic allergic reactions.[1, 2, 3, 4, 5, 6, 7]. The reference range for urinary NMH varies according to subject age, as follows: Age 0-5 years - 120-510 �g/g creatinine; Age 6-16 years - 70-330 �g/g creatinine, Older than16 years - 30-200 �g/g creatinine
0661                             'EX_tym[u]' %Tyramine and its conjugates occur in normal and abnormal urines, although the biological role of tyramine, if any, is obscure. However, it has recently become of interest because severe Parkinsonians excrete raised amounts of tyraminel-R
0662                             'EX_2hyoxplac[u]'%2-Hydroxyphenylacetate
0663                             'EX_pmtcrn[u]'
0664                             'EX_dheas[u]'
0665                             'EX_34dhphe[u]' %L-dopa
0666                             'EX_srtn[u]'
0667                             'EX_gthrd[u]'
0668                             'EX_pcholhep_hs[u]'
0669                             'EX_pcholste_hs[u]'
0670                             'EX_pcholn204_hs[u]'
0671                             'EX_3moxtyr[u]'
0672                             'EX_aldstrn[u]'
0673                             'EX_tststerone[u]'
0674                             'EX_pydxn[u]'
0675                             'EX_sphgn[u]' % i dont think that this metabolite is routinely secreted
0676                             'EX_sphings[u]'
0677                             'EX_csn[u]'
0678                             'EX_arab_L[u]'
0679                             'EX_tststerone[u]'
0680                             'EX_tststerone[u]'
0681                             'EX_pydxn[u]'
0682                             'EX_mma[u]'%Methylamine occurs endogenously from amine catabolism and its tissue levels increase in some pathological conditions, including diabetes.
0683                             'EX_tsul[u]'%Thiosulfate occurs naturally in hot springs and geysers, and is produced by certain biochemical processes. In the body, thiosulfate converts small amounts of cyanide ion into harmless products and plays a role in the biosynthesis of cysteine, a sulfur-containing amino acid that locks proteins into their correct three-dimensional shapes. Thiosulfate is not found in large quantities in nature.
0684                             'EX_5htrp[u]'
0685                             'EX_7dhchsterol'
0686                             'EX_etoh[u]'
0687                             'EX_gsn[u]'
0688                             'EX_5aop[u]'
0689                             'EX_uri[u]';
0690                             'EX_dad_2[u]'
0691                             'EX_ocdca[u]'
0692                             'EX_gua[u]'
0693                             'EX_dcyt[u]'
0694                             'EX_glyleu[u]'
0695                             'EX_acald[u]'
0696                             'EX_HC02191[u]'
0697                             %%
0698                             % 'EX_ethamp[u]'
0699                             };
0700                         R = unique([R;ExclList]);
0701                         MustSecrete = {
0702                             'EX_urea[u]'
0703                             'EX_nh4[u]'
0704                             'EX_etha[u]'
0705                             %   'EX_na1
0706                             'EX_lcts[u]'
0707                             'EX_3hmp[u]'
0708                             'EX_acnam[u]'
0709                             };
0710                         
0711                         if  modelConstraint.ub(ExR(j)) > 0;
0712                             if    setLB == 1
0713                                 if ~ismember(R,modelConstraint.rxns(ExR(j)))
0714                                     modelConstraint.lb(ExR(j)) = MSecrRateUrLB; % maximal possible uptake rate
0715                                     %        modelConstraint.lb(ExR(j)) = 0;
0716                                 else
0717                                     modelConstraint.lb(ExR(j)) = 0;
0718                                 end
0719                             else
0720                                 if MConMax>=MaxConcConstraint  && MConMin>=MinConcConstraint && ~ismember(modelConstraint.rxns(ExR(j)),R)%ismember(modelConstraint.rxns(ExR(j)),MustSecrete)% %
0721                                     modelConstraint.lb(ExR(j)) = MSecrRateUrLB; % maximal possible uptake rate
0722                                 else
0723                                     modelConstraint.lb(ExR(j)) = 0;
0724                                 end
0725                             end
0726                             modelConstraint.ub(ExR(j)) = MSecrRateUrUB; % maximal possible secretion rate
0727                         end
0728                     end
0729                     
0730                 else
0731                     modelConstraint.rxns(ExR(j));
0732                 end
0733             end
0734         end
0735     end
0736 end
0737 
0738 %% woman is not producing milk! - IT 20.12.2016
0739 % hence close all milk producing reactions
0740 tmp = find(~cellfun(@isempty,strfind(modelConstraint.rxns,'(miB)_[mi]')));
0741 modelConstraint.lb(tmp) = 0;
0742 modelConstraint.ub(tmp) = 0;
0743 
0744 if 1
0745     %% set o2[a] and co2[a] constraints
0746     % Put together by Maike
0747     % Composition air in: 78.62%�nitrogen, 21%�oxygen, 0.96%�argon, 0.04%�carbon dioxide, 0.5%�water vapour
0748     % Composition air out: 78.04% nitrogen, 14% - 16% oxygen, 4% - 5.3% carbon dioxide, 1% argon and other gases
0749     % Amount of O2 in:
0750     %   Tidal volume: 500 ml/breath
0751     %   Breathing frequency 12-15x/min
0752     %   Change of O2: 5%
0753     %   Volume of gas: 1mol gas = 22.4 l , 1mmol=22.4ml
0754     %   Volume O2/breath = 5*500 (ml)/100 = 25ml
0755     %   O2 change (mmol) = 25ml/22.4 ml = 1.1mmol
0756     %   Volume 02/day = 1.1mmol*12*60*24 = 19.080mol/day
0757     % Amount of CO2 out:
0758     %     Tidal volume: 500 ml/breath
0759     %     Breathing frequency 12-15x/min
0760     %     Change of CO2: 5.3%
0761     %     Volume of gas: 1mmol=22.4ml
0762     %
0763     %     Volume CO2/breath = 5.3*500 (ml)/100 = 26.5ml
0764     %     CO2 change (mmol) = 26.5ml/22.4 ml = 1.18mmol
0765     %     Volume 02/day = 1.18mmol*12*60*24 = 20.442 mol/day
0766     % Alternative calculation
0767     %     Ratio O2/CO2 = 0.8
0768     %     Tidal volume: 500 ml/breath
0769     %     Breathing frequency 12-15x/min
0770     %     Change of CO2: 4-5.3%
0771     %     Density of CO2 = 1.98g/l
0772     %
0773     %     Volume CO2/breath = 0.8*(0.05*0.5) = 0.02l
0774     %     Volume 02/day = 0.02l*12 = 0.24l
0775     %     Amount CO2/day = 1.98g/l*0.24l*60*24 =  0.475g*60*24= 684.288g/day
0776     %     Volume C02/day = 15.548 mol/day
0777     %
0778     %     Volume 02/day = 0.02l*15
0779     %                    = 0.30l ->19.436mol/day
0780     % Refs: http://biology.stackexchange.com/questions/5642/how-much-gas-is-exchanged-in-one-human-breath
0781     % https://en.wikipedia.org/wiki/Breathing#Breathing_in_gas
0782     % http://cozybeehive.blogspot.lu/2010/03/how-much-co2-do-you-exhale-while.html
0783     % http://www.convertunits.com/from/grams+CO2/to/moles
0784     if 1
0785         modelConstraint = changeRxnBounds(modelConstraint,'EX_o2[a]',-15000,'u');%change to 15k
0786         modelConstraint = changeRxnBounds(modelConstraint,'EX_o2[a]',-25000,'l');
0787         modelConstraint = changeRxnBounds(modelConstraint,'EX_co2[a]',15000*0.8,'l');
0788         modelConstraint = changeRxnBounds(modelConstraint,'EX_co2[a]',25000,'u');
0789     end
0790     % % %% water
0791     % % %breathing out of water
0792     % % % from Ref man
0793     % % % Sweat = 650 ml(water loss)/day - 650g = 650g/day / 18.01528g/mol = 36.0805 mol/day
0794     % % % Insensible(breathing??) 840g = 850/18.01528 = 47.1822 mol/day
0795     % % % Urine = 1400g = 1400/18.01528 = 77.7118 mol/day
0796     % % % Feces = 100g = 100/18.01528 = 5.5508 mol/day
0797     if 1
0798         %  modelConstraint = changeRxnBounds(modelConstraint,'EX_h2o[a]',36080*0.8,'l');%
0799         % modelConstraint = changeRxnBounds(modelConstraint,'EX_h2o[a]',36080*1.2,'u');
0800         
0801         
0802         modelConstraint = changeRxnBounds(modelConstraint,'EX_h2o[a]',47182*0.8,'l');%
0803         modelConstraint = changeRxnBounds(modelConstraint,'EX_h2o[a]',47182*1.2,'u');
0804         
0805         % % % sweating of water
0806         %modelConstraint = changeRxnBounds(modelConstraint,'EX_h2o[sw]',47182*0.8,'l');%
0807         %modelConstraint = changeRxnBounds(modelConstraint,'EX_h2o[sw]',47182*1.2,'u');
0808         
0809         modelConstraint = changeRxnBounds(modelConstraint,'EX_h2o[sw]',36080*0.8,'l');%
0810         modelConstraint = changeRxnBounds(modelConstraint,'EX_h2o[sw]',36080*1.2,'u');
0811         
0812         % % % water in urine
0813         modelConstraint = changeRxnBounds(modelConstraint,'EX_h2o[u]',77711*0.8,'l');%should be much higher
0814         modelConstraint = changeRxnBounds(modelConstraint,'EX_h2o[u]',77711*1.2,'u');
0815         % % % water in feces
0816         modelConstraint = changeRxnBounds(modelConstraint,'Excretion_EX_h2o[fe]',5550*0.8,'l');%
0817         modelConstraint = changeRxnBounds(modelConstraint,'Excretion_EX_h2o[fe]',5550*1.2,'u');
0818         
0819         % limit water secretion into bile duct
0820         modelConstraint = changeRxnBounds(modelConstraint,'Gall_H2Ot[bdG]',1000,'u'); % arbitrary number
0821         modelConstraint = changeRxnBounds(modelConstraint,'Liver_H2Ot[bdL]',1000,'u'); % arbitrary number
0822     end
0823     if 1
0824         % % %% specific reactions
0825         % % Muscle can only take up glc
0826         % set constrain only if the new constrain is tighter than existing one and
0827         % does not get smaller than LB
0828         if modelConstraint.ub(find(ismember(modelConstraint.rxns, 'Muscle_EX_glc_D(e)_[bc]')))>= -0.01*1000 && modelConstraint.lb(find(ismember(modelConstraint.rxns, 'Muscle_EX_glc_D(e)_[bc]')))<= -0.01*1000
0829             modelConstraint = changeRxnBounds(modelConstraint,'Muscle_EX_glc_D(e)_[bc]',-0.01*1000,'u');
0830         elseif modelConstraint.lb(find(ismember(modelConstraint.rxns, 'Muscle_EX_glc_D(e)_[bc]')))> modelConstraint.ub(find(ismember(modelConstraint.rxns, 'Muscle_EX_glc_D(e)_[bc]')))
0831             modelConstraint.ub(find(ismember(modelConstraint.rxns, 'Muscle_EX_glc_D(e)_[bc]')))=0; %reset earlier constraints if lb>ub
0832         end
0833         
0834         % 'Muscle_EX_ala_l(e)_[bc]'    'Muscle_ala_L[e]  <=> ala_L[bc] '    alanine secretion    muscle    12.5 mg alanine/min/person (65 kg)    C3H7NO2    89.09    0.233126398    0.233126398    'Muscle_EX_ala_l(e)_[bc]'    0.187    0.280    postabsorption state    Frayn book
0835         met = 12.5; % mg per min per 65 kg
0836         MW = 89.09; % g�mol?1
0837         met = (met * 60 * 24 *IndividualParameters.bodyWeight/65)/1000; %g per day per person (weight adjusted)
0838         met = met * 1000/ MW ; %mmol per day per person (weight adjusted)
0839         if modelConstraint.lb(find(ismember(modelConstraint.rxns, 'Muscle_EX_ala_L(e)_[bc]')))<met*0.80 && modelConstraint.ub(find(ismember(modelConstraint.rxns, 'Muscle_EX_ala_L(e)_[bc]')))>=met*0.8
0840             modelConstraint = changeRxnBounds(modelConstraint,'Muscle_EX_ala_L(e)_[bc]',met*0.8,'l');% to be in mmol/day/person
0841             modelConstraint = changeRxnBounds(modelConstraint,'Muscle_EX_ala_L(e)_[bc]',met*1.2,'u');
0842         elseif modelConstraint.lb(find(ismember(modelConstraint.rxns, 'Muscle_EX_ala_L(e)_[bc]')))> modelConstraint.ub(find(ismember(modelConstraint.rxns, 'Muscle_EX_ala_L(e)_[bc]')))
0843             modelConstraint.lb(find(ismember(modelConstraint.rxns, 'Muscle_EX_ala_L(e)_[bc]')))=0; %reset earlier constraints if lb>ub
0844         end
0845         
0846         
0847         %     RBC_EX_glc(e)_[bc]'    'RBC_glc_D[e]  <=> glc_D[bc] '    glucose uptake     RBC    25 mg/glc/min/person (65kg)    C6H12O6    180.16    0.230564285    -0.230564285    'RBC_EX_glc(e)_[bc]'    -0.184    -0.277        Frayn book
0848         met = 25; % mg per min per 65 kg
0849         MW = 180.16;% g�mol?1
0850         met = (met * 60 * 24 *IndividualParameters.bodyWeight/65)/1000; %g per day per person (weight adjusted)
0851         met = met * 1000/ MW ; %mmol per day per person (weight adjusted)
0852         % set constraints only if they make the range smaller
0853         if modelConstraint.lb(find(ismember(modelConstraint.rxns, 'RBC_EX_glc_D(e)_[bc]')))<-met*1.2 && modelConstraint.ub(find(ismember(modelConstraint.rxns, 'RBC_EX_glc_D(e)_[bc]')))>=-met*1.2
0854             modelConstraint = changeRxnBounds(modelConstraint,'RBC_EX_glc_D(e)_[bc]',-met*0.8,'u');
0855             modelConstraint = changeRxnBounds(modelConstraint,'RBC_EX_glc_D(e)_[bc]',-met*1.2,'l');
0856         elseif modelConstraint.lb(find(ismember(modelConstraint.rxns, 'RBC_EX_glc_D(e)_[bc]')))> modelConstraint.ub(find(ismember(modelConstraint.rxns, 'RBC_EX_glc_D(e)_[bc]')))
0857             modelConstraint.ub(find(ismember(modelConstraint.rxns, 'RBC_EX_glc_D(e)_[bc]')))=0; %reset earlier constraints if lb>ub
0858         end
0859     end
0860     if 0
0861        % 0.07 � 0.01 mlO2/min/100 g); http://citeseerx.ist.psu.edu/viewdoc/download?doi=10.1.1.558.6328&rep=rep1&type=pdf
0862         % 1.04 kg/l was used for muscle density to convert 1 l to 100 g of skeletal muscle.
0863      %   One mole of Hb carries four moles of O2. Then, 1 mol of gas was converted into 1 l with value of 1 mol
0864      % gas = 22.4 L standard temperature and pressure, dry (STPD) conditions.
0865         met = 0.07; %ml o2/min/100g
0866         met = (met * 1000/22.4) *60 *24; % umol o2/ day/100g;
0867          muscle_weight = (IndividualParameters.OrgansWeights(find(ismember(IndividualParameters.OrgansWeights(:,1),'Muscle')),2));
0868          if ischar(muscle_weight)
0869              muscle_weight =str2num(char(muscle_weight{1})) ;
0870          elseif iscell(muscle_weight)
0871              try
0872                      muscle_weight =str2num((muscle_weight{1})) ;
0873              end
0874              try
0875                  muscle_weight =muscle_weight{1} ;
0876              end
0877          else
0878              muscle_weight =muscle_weight{1} ;
0879          end
0880          met =( met*muscle_weight/100)/1000; % mmol o2/day/person
0881            if modelConstraint.lb(find(ismember(modelConstraint.rxns, 'Muscle_EX_o2(e)_[bc]')))<-met*1.2 && modelConstraint.ub(find(ismember(modelConstraint.rxns, 'RBC_EX_glc_D(e)_[bc]')))>=-met*1.2
0882             modelConstraint = changeRxnBounds(modelConstraint,'Muscle_EX_o2(e)_[bc]',-met*0.8,'u');
0883             modelConstraint = changeRxnBounds(modelConstraint,'Muscle_EX_o2(e)_[bc]',-met*1.2,'l');
0884         elseif modelConstraint.lb(find(ismember(modelConstraint.rxns, 'Muscle_EX_o2(e)_[bc]')))> modelConstraint.ub(find(ismember(modelConstraint.rxns, 'RBC_EX_glc_D(e)_[bc]')))
0885             modelConstraint.ub(find(ismember(modelConstraint.rxns, 'Muscle_EX_o2(e)_[bc]')))=0; %reset earlier constraints if lb>ub
0886            end
0887     end
0888     if 1
0889         if 1
0890             % 'Brain_EX_glc(e)_[csf]'    'Brain_glc_D[e]  <=> glc_D[csf] '    glucose uptake     brain    80 mg glc/min/person    C6H12O6    180.16    0.737805711    -0.737805711    'Brain_EX_glc(e)_[csf]'    -0.590    -0.885    all day    Frayn book
0891             met = 80; % mg per min per 65 kg
0892             MW = 180.16;% g�mol?1
0893             met = (met * 60 * 24 *IndividualParameters.bodyWeight/65)/1000; %g per day per person (weight adjusted)
0894             met = met * 1000/ MW ; %mmol per day per person (weight adjusted)
0895             if modelConstraint.lb(find(ismember(modelConstraint.rxns, 'Brain_EX_glc_D(e)_[csf]')))<-met*1.20 && modelConstraint.ub(find(ismember(modelConstraint.rxns, 'Brain_EX_glc_D(e)_[csf]')))>=-met*1.2
0896                 modelConstraint = changeRxnBounds(modelConstraint,'Brain_EX_glc_D(e)_[csf]',-met*0.8,'u');
0897                 modelConstraint = changeRxnBounds(modelConstraint,'Brain_EX_glc_D(e)_[csf]',-met*1.2,'l');
0898             elseif modelConstraint.lb(find(ismember(modelConstraint.rxns, 'Brain_EX_glc_D(e)_[csf]')))> modelConstraint.ub(find(ismember(modelConstraint.rxns, 'Brain_EX_glc_D(e)_[csf]')))
0899                 modelConstraint.ub(find(ismember(modelConstraint.rxns, 'Brain_EX_glc_D(e)_[csf]')))=0; %reset earlier constraints if lb>ub
0900             end
0901         end
0902         %% addition 21.12.2016
0903         %
0904         if 1
0905             brain_weight = (IndividualParameters.OrgansWeights(find(ismember(IndividualParameters.OrgansWeights(:,1),'Brain')),2));           
0906          if ischar(brain_weight)
0907              brain_weight =str2num(char(brain_weight{1})) ;
0908          elseif iscell(brain_weight)
0909              try
0910              brain_weight =str2num((brain_weight{1})) ;
0911              end
0912              try
0913              brain_weight =brain_weight{1} ;
0914              end
0915          else
0916              brain_weight =brain_weight{1} ;
0917          end
0918             brain_o2 = 156;% umol o2/100g brain/min; REF: http://link.springer.com/chapter/10.1007%2F978-1-59259-108-4_2#page-1
0919             brain_o2 = (brain_o2 * 60 * 24 * brain_weight/100)/1000; %mmol o2/person (brain)/day.
0920             if modelConstraint.lb(find(ismember(modelConstraint.rxns, 'Brain_EX_o2(e)_[csf]')))<-brain_o2*1.2 && modelConstraint.ub(find(ismember(modelConstraint.rxns, 'Brain_EX_o2(e)_[csf]')))>=-brain_o2*1.2
0921                 modelConstraint = changeRxnBounds(modelConstraint,'Brain_EX_o2(e)_[csf]',-brain_o2*1.2,'l');
0922                 modelConstraint = changeRxnBounds(modelConstraint,'Brain_EX_o2(e)_[csf]',-brain_o2*0.7,'u');
0923             elseif modelConstraint.lb(find(ismember(modelConstraint.rxns, 'Brain_EX_o2(e)_[csf]')))> modelConstraint.ub(find(ismember(modelConstraint.rxns, 'Brain_EX_o2(e)_[csf]')))
0924                 modelConstraint.ub(find(ismember(modelConstraint.rxns, 'Brain_EX_o2(e)_[csf]')))=0; %reset earlier constraints if lb>ub
0925             end
0926         end
0927         
0928     end
0929     if 1
0930         
0931         % 'Liver_EX_ala_l(e)_[bc]'    'Liver_ala_L[e]  <=> ala_L[bc] '    alanine uptake    liver    12.5 mg alanine/min/person (65 kg)    C3H7NO2    89.09    0.233126398    -0.233126398    'Liver_EX_ala_l(e)_[bc]'    -0.187    -0.280    postabsorption state    Frayn book
0932         met = 12.5; % mg per min per 65 kg
0933         MW = 89.09; % g�mol?1
0934         met = (met * 60 * 24 *IndividualParameters.bodyWeight/65)/1000; %g per day per person (weight adjusted)
0935         met = met * 1000/ MW ; %mmol per day per person (weight adjusted)
0936         if modelConstraint.lb(find(ismember(modelConstraint.rxns, 'Liver_EX_ala_L(e)_[bc]')))<-met*1.20 && modelConstraint.ub(find(ismember(modelConstraint.rxns, 'Liver_EX_ala_L(e)_[bc]')))>=-met*1.2
0937             modelConstraint = changeRxnBounds(modelConstraint,'Liver_EX_ala_L(e)_[bc]',-met*0.8,'u');
0938             modelConstraint = changeRxnBounds(modelConstraint,'Liver_EX_ala_L(e)_[bc]',-met*1.2,'l');
0939         elseif modelConstraint.lb(find(ismember(modelConstraint.rxns, 'Liver_EX_ala_L(e)_[bc]')))> modelConstraint.ub(find(ismember(modelConstraint.rxns, 'Liver_EX_ala_L(e)_[bc]')))
0940             modelConstraint.ub(find(ismember(modelConstraint.rxns, 'Liver_EX_ala_L(e)_[bc]')))=0; %reset earlier constraints if lb>ub
0941         end
0942         
0943         % 'Liver_EX_glc(e)_[bc]'    'Liver_glc_D[e]  <=> glc_D[bc] '    glucose secretion    Liver    130 mg glc/min/person (65 kg    C6H12O6    180.16    1.198934281    1.198934281    'Liver_EX_glc(e)_[bc]'    0.959    1.439    postabsorption state    Frayn book
0944         met = 130; % mg per min per 65 kg
0945         MW = 180.16;% g�mol?1
0946         met = (met * 60 * 24 *IndividualParameters.bodyWeight/65)/1000; %g per day per person (weight adjusted)
0947         met = met * 1000/ MW ; %mmol per day per person (weight adjusted)
0948         if modelConstraint.lb(find(ismember(modelConstraint.rxns, 'Liver_EX_glc_D(e)_[bc]')))<met*0.80 && modelConstraint.ub(find(ismember(modelConstraint.rxns, 'Liver_EX_glc_D(e)_[bc]')))>=met*0.8
0949             modelConstraint = changeRxnBounds(modelConstraint,'Liver_EX_glc_D(e)_[bc]',met*1.2,'u');
0950             modelConstraint = changeRxnBounds(modelConstraint,'Liver_EX_glc_D(e)_[bc]',met*0.8,'l');
0951         elseif modelConstraint.lb(find(ismember(modelConstraint.rxns, 'Liver_EX_glc_D(e)_[bc]')))> modelConstraint.ub(find(ismember(modelConstraint.rxns, 'Liver_EX_glc_D(e)_[bc]')))
0952             modelConstraint.lb(find(ismember(modelConstraint.rxns, 'Liver_EX_glc_D(e)_[bc]')))=0; %reset earlier constraints if lb>ub
0953         end
0954     end
0955     if 1
0956         
0957         % 'Adipocytes_EX_glyc(e)_[bc]'    'Adipocytes_glyc[e]  <=> glyc[bc] '    glycerol secretion     adipocytes    12 mg glycerol/min/person (65 kg)    C3H8O3    92.09    0.216510604    0.216510604    'Adipocytes_EX_glyc(e)_[bc]'    0.173    0.260    postabsorption state    Frayn book
0958         met = 12; % mg per min per 65 kg
0959         MW =     92.09;% g�mol?1
0960         met = (met * 60 * 24 *IndividualParameters.bodyWeight/65)/1000; %g per day per person (weight adjusted)
0961         met = met * 1000/ MW ; %mmol per day per person (weight adjusted)
0962         if modelConstraint.lb(find(ismember(modelConstraint.rxns, 'Adipocytes_EX_glyc(e)_[bc]')))<met*0.80 && modelConstraint.ub(find(ismember(modelConstraint.rxns, 'Adipocytes_EX_glyc(e)_[bc]')))>=met*0.8
0963             % modelConstraint =     changeRxnBounds(modelConstraint,'Adipocytes_EX_glyc(e)_[bc]',met*0.8,'l');
0964             % the lower bound seems to create troubles so I removed it.
0965             modelConstraint = changeRxnBounds(modelConstraint,'Adipocytes_EX_glyc(e)_[bc]',met*1.2,'u');
0966         elseif modelConstraint.lb(find(ismember(modelConstraint.rxns, 'Adipocytes_EX_glyc(e)_[bc]')))> modelConstraint.ub(find(ismember(modelConstraint.rxns, 'Adipocytes_EX_glyc(e)_[bc]')))
0967             modelConstraint.lb(find(ismember(modelConstraint.rxns, 'Adipocytes_EX_glyc(e)_[bc]')))=0; %reset earlier constraints if lb>ub
0968         end
0969     end
0970     %% addition 19.12.2016
0971     % constrain growth rate of renewing organs
0972     % based on bionumbers
0973     % I am still not convinced that the constraints that I wanted to place
0974     % are correctly capturing the organ weight - I will leave it for the
0975     % moment. - IT 22.12.2-2016
0976     %   modelConstraint = changeRxnBounds(modelConstraint,'sIEC_biomass_reactionIEC01b_trtr',0.25,'l');% every 4 days turn over
0977     %   modelConstraint = changeRxnBounds(modelConstraint,'sIEC_biomass_reactionIEC01b_trtr',0.5,'u');% every 2 days turn over
0978     %   modelConstraint = changeRxnBounds(modelConstraint,'Stomach_biomass_reaction',0.11,'l');% every 9 days turn over
0979     %   modelConstraint = changeRxnBounds(modelConstraint,'Stomach_biomass_reaction',0.5,'u');% every 2 days turn over
0980     %
0981     %   modelConstraint = changeRxnBounds(modelConstraint, 'Colon_biomass_reaction',0.25,'l');% every 4 days turn over
0982     %   modelConstraint = changeRxnBounds(modelConstraint, 'Colon_biomass_reaction',0.33,'u');% every 3 days turn over
0983     %
0984     %   modelConstraint = changeRxnBounds(modelConstraint,  'Skin_biomass_reaction',0.033,'l');% every 30 days turn over
0985     %   modelConstraint = changeRxnBounds(modelConstraint,  'Skin_biomass_reaction',0.1,'u');% every 10 days turn over
0986     
0987     %% brain and liver can do co2 fixation - REF: http://www.jbc.org/content/237/8/2570.full.pdf
0988     
0989     %disallow carbon fixation -- Nov 2017
0990     L = (find(~cellfun(@isempty,strfind(modelConstraint.rxns,'EX_co2(e)_[bc]'))));
0991     %
0992     modelConstraint.lb(find(ismember(modelConstraint.rxns,modelConstraint.rxns(L)))) = 0;
0993     %% Co2 can cross BBB: http://www.sciencedirect.com/science/article/pii/0026286280900205
0994     modelConstraint = changeRxnBounds(modelConstraint,  'Brain_EX_co2(e)_[csf]',-10000,'l'); % arbiratry numbers
0995     modelConstraint = changeRxnBounds(modelConstraint,  'Liver_EX_co2(e)_[bc]',-10000,'l');
0996     % lung is also allowed to take up
0997     modelConstraint = changeRxnBounds(modelConstraint,  'Lung_EX_co2(e)_[bc]',-10000,'l');
0998     modelConstraint = changeRxnBounds(modelConstraint,  'Kidney_EX_co2(e)_[bc]',-10000,'l');
0999     if 1
1000         %% 10.01.17
1001         % brain atp requirement
1002         % apparently the brain consumes about 120g glc per day, corresponding to
1003         % 0.66 mol glc/day/person (MW=180.16)
1004         % 1 mol glc can be converted into 31 mol atp
1005         % hence 20.46 mol ATP could be theoretically produced from 0.66 mol glc
1006         % (if complete ox phos)
1007         % I will set the lower bound on DM_atp to 10 mol/day/person (this is an
1008         % arbitrary number). The GF Harvey under Av EU diet can produce max  12799.7
1009         % mmol ATP/person/day
1010         % ref: https://www.ncbi.nlm.nih.gov/books/NBK22436/, section 30.2
1011         
1012         %  modelConstraint = changeRxnBounds(modelConstraint,'Brain_DM_atp_c_',10000,'l');
1013         modelConstraint = changeRxnBounds(modelConstraint,'Brain_DM_atp_c_',0,'l');
1014     end
1015     
1016     if 1
1017         %% heart energy requirement - minimum
1018         % https://heartmdinstitute.com/heart-health/metabolic-cardiology-basics/
1019         % reports a minimum of 6000g of ATP per day per person, MW_ATP =
1020         % 507.18g/mol
1021         % hence lb = 11830 mmol/day/person
1022         modelConstraint = changeRxnBounds(modelConstraint,'Heart_DM_atp_c_',6000,'l');
1023     end
1024     % Also check this for future efforts: http://hypertextbook.com/facts/2003/IradaMuslumova.shtml
1025     
1026     %% constraint conversion of h2o + co2 to h + hco3
1027     
1028     % R = (find(~cellfun(@isempty,strfind(modelConstraint.rxns, 'RBC_H2CO3D'))));
1029     % modelConstraint.lb(R)=0;
1030     % modelConstraint.ub(R)=150; % no reference for this value except to avoid too high flux through this reaction
1031     
1032     %% o2 uptake lower bound constraints
1033     % each red blood cell contains ~ 270*10^6 haemoglobin, each of which can
1034     % carry up to 4 o2: e.g., https://en.wikipedia.org/wiki/Red_blood_cell
1035     % so one red blood cell carries 4*270*10^6 O2
1036     % The avogadro number is 6.022140857(74)�0^23 mol?1
1037     % The normal range in men is approximately 4.7 to 6.1 million cells/ul (microliter). The normal range in women range from 4.2 to 5.4 million cells/ul, according to NIH (National Institutes of Health) data.
1038     % men: assumed 5.5M/ul and  female: assumed 4.5M/ul
1039     % if strcmp(sex,'male')
1040     %     RBC = 5.5*10^6*
1041 end
```

---

Generated on Thu 14-May-2020 13:05:49 by **m2html** © 2005
