# Supplementary material for: Personalized whole‐body models integrate metabolism, physiology, and the gut microbiome
Source: Mol Syst Biol. 2020 May 28;16(5):e8982. doi: 10.15252/msb.20198982 (PMC7285886; doi:10.15252/msb.20198982)
Supplement: Supplementary file 22 — Dataset EV1 [file MSB-16-e8982-s022.zip › PSCM_toolbox/PSCM_toolbox_doc/src/setConstraints/AAStorage.html]

Description of AAStorage


# AAStorage

## PURPOSE

**This script defines amino acids that can be stored by one or more organs**

## SYNOPSIS

**This is a script file.**

## DESCRIPTION

```
 This script defines amino acids that can be stored by one or more organs
```

## CROSS-REFERENCE INFORMATION

This function calls:


This function is called by:

- setFeedingFastingConstraints This function sets constraints corresponding either to feeding (i.e.,

## SOURCE CODE

```
0001 % This script defines amino acids that can be stored by one or more organs
0002 storageAA = {'sink_his_L(c)';
0003     'sink_ile_L(c)';
0004     'sink_leu_L(c)';
0005     'sink_lys_L(c)';
0006     'sink_met_L(c)';
0007     'sink_phe_L(c)';
0008     'sink_thr_L(c)';
0009     'sink_trp_L(c)';
0010     'sink_val_L(c)';
0011     % non-essential AAs
0012     'sink_ala_L(c)'
0013 'sink_arg_L(c)'
0014 'sink_asn_L(c)'
0015 'sink_asp_L(c)'
0016 'sink_cys_L(c)'
0017 'sink_gln_L(c)'
0018 'sink_glu_L(c)'
0019 'sink_pro_L(c)'
0020 'sink_ser_L(c)'
0021 'sink_tyr_L(c)'
0022 'sink_gly(c)'
0023     }
```

---

Generated on Thu 14-May-2020 13:05:49 by **m2html** © 2005
