# Supplementary material for: Personalized whole‐body models integrate metabolism, physiology, and the gut microbiome
Source: Mol Syst Biol. 2020 May 28;16(5):e8982. doi: 10.15252/msb.20198982 (PMC7285886; doi:10.15252/msb.20198982)
Supplement: Supplementary file 22 — Dataset EV1 [file MSB-16-e8982-s022.zip › PSCM_toolbox/PSCM_toolbox_doc/src/setConstraints/physiologicalConstraintsHMDBbased_old.html]

Description of physiologicalConstraintsHMDBbased\_old


# physiologicalConstraintsHMDBbased\_old

## PURPOSE

**apply constraints to Harvey**

## SYNOPSIS

**function modelConstraint = physiologicalConstraintsHMDBbased(model,IndividualParameters, ExclList, Type, InputData, Biofluid, setDefault)**

## DESCRIPTION

```
 apply constraints to Harvey
 metabolite concentrations have to be given in uM
 organ weights have to be given in g

 INPUT
 model                     model structure
 IndividualParameters      Structure containing physiological parameters,
                           as generated in standardPhysiolDefaultParameters
 Type                      Input type (either 'xlsx' (default) --> loads by default
                           'Parsed_hmdbConc.xlsx' or 'direct'). If
                           'direct' InputData must be provided
 InputData                 first column corresponds to vmh id's of
                           metabolites, 2nd to data points (will be set as lb and ub)
 Biofluid                  'all' (default if type is xlsx). For direct:
                            'bc','u','csf'


 OUTPUT
 modelConstraint           model structure with updated constraints

 Ines Thiele, 2015/2016
```

## CROSS-REFERENCE INFORMATION

This function calls:

- OrganLists This file contains lists of ograns as they are used in the whole-body

This function is called by:

## SOURCE CODE

```
0001 function modelConstraint = physiologicalConstraintsHMDBbased(model,IndividualParameters, ExclList, Type, InputData, Biofluid, setDefault)
0002 % apply constraints to Harvey
0003 % metabolite concentrations have to be given in uM
0004 % organ weights have to be given in g
0005 %
0006 % INPUT
0007 % model                     model structure
0008 % IndividualParameters      Structure containing physiological parameters,
0009 %                           as generated in standardPhysiolDefaultParameters
0010 % Type                      Input type (either 'xlsx' (default) --> loads by default
0011 %                           'Parsed_hmdbConc.xlsx' or 'direct'). If
0012 %                           'direct' InputData must be provided
0013 % InputData                 first column corresponds to vmh id's of
0014 %                           metabolites, 2nd to data points (will be set as lb and ub)
0015 % Biofluid                  'all' (default if type is xlsx). For direct:
0016 %                            'bc','u','csf'
0017 %
0018 %
0019 % OUTPUT
0020 % modelConstraint           model structure with updated constraints
0021 %
0022 % Ines Thiele, 2015/2016
0023 
0024 modelConstraint = model;
0025 
0026 setLB = 0;
0027 
0028 
0029 %% Input physiological data
0030 %standardPhysiolDefaultParameters;
0031 gender = IndividualParameters.gender;
0032 sex = IndividualParameters.sex;
0033 CardiacOutput = IndividualParameters.CardiacOutput;
0034 
0035 % default maximum concentration of a metabolite in blood plasma
0036 MConDefaultBc = IndividualParameters.MConDefaultBc;
0037 
0038 % default maximum concentration of a metabolite in csf
0039 MConDefaultCSF = IndividualParameters.MConDefaultCSF;
0040 
0041 % default maximum concentration of a metabolite in Ur
0042 MConDefaultUrMax = IndividualParameters.MConDefaultUrMax;
0043 MConDefaultUrMin = IndividualParameters.MConDefaultUrMin;
0044 
0045 % creatinine concentration in urine
0046 MConDefaultUrCreatinineMax = IndividualParameters.MConUrCreatinineMax;
0047 MConDefaultUrCreatinineMin = IndividualParameters.MConUrCreatinineMin;
0048 
0049 % CSF Flow rate
0050 CSFFlowRate = IndividualParameters.CSFFlowRate;
0051 
0052 % CSF Blood Flow rate
0053 CSFBloodFlowRate = IndividualParameters.CSFBloodFlowRate;
0054 
0055 % Urine flow rate
0056 UrFlowRate = IndividualParameters.UrFlowRate;
0057 
0058 % Hematocrite
0059 Hematocrit = IndividualParameters.Hematocrit;
0060 
0061 % lower concentration limit for setting a concentration constraint
0062 MinConcConstraint = 5;
0063 MaxConcConstraint = 50;
0064 bloodFlowData = IndividualParameters.bloodFlowData;
0065 bloodFlowPercCol = IndividualParameters.bloodFlowPercCol;
0066 bloodFlowOrganCol = IndividualParameters.bloodFlowOrganCol;
0067 % List of organs
0068 OrganLists;
0069 
0070 %% calculate GFR = Glomerular filtration rate
0071 % the filtration fraction should be 20% of the renal plasma flow
0072 %GlomerularFiltrationRate = IndividualParameters.GlomerularFiltrationRate; % in ml/min
0073 RenalFiltrationFraction  = 0.2; %20%
0074 
0075 % blood flow percentage that Kidney gets
0076 if strcmp(gender,'male')
0077     BK = bloodFlowData{strmatch('Kidney',bloodFlowData(:,1),'exact'),bloodFlowPercCol(1)};
0078 elseif strcmp(gender,'female')
0079     BK = bloodFlowData{strmatch('Kidney',bloodFlowData(:,1),'exact'),bloodFlowPercCol(2)};
0080     % BK = bloodFlowData{strmatch('Kidney',bloodFlowData(:,1),'exact'),4};
0081 end
0082 BK = str2num(BK(2:end-1));
0083 RenalFlowRate=BK*CardiacOutput*(1-Hematocrit); % k_plasma_organ in ml/min
0084 GlomerularFiltrationRate = RenalFlowRate*RenalFiltrationFraction;% in ml/min
0085 %% read data
0086 % read metabolic concentrations from HMDB if no input data are defined
0087 if ~exist('InputData','var')
0088     Type = 'HMDB';
0089     Biofluid = 'all';
0090 end
0091 if ~exist('Type','var')
0092     Type = 'HMDB';
0093 end
0094 if ~exist('ExclList','var')
0095 ExclList = '';
0096 end
0097 % default concentrations will be applied as constraints if not specified
0098 % differently
0099 if ~exist('setDefault','var')
0100     setDefault = 1; % default is true
0101 end
0102 
0103 if strcmp(Type,'HMDB')
0104     % BLOOD
0105     Biofluid = 'all';
0106     fileName='NormalBloodConcExtractedHMDB.txt';
0107     [Data] =importdata(fileName);
0108     
0109     % find start/header of data - Blood
0110     % find data start
0111     Start= (find(~cellfun(@isempty,strfind(Data.textdata(:,1),'####'))))+1;
0112     % find rxn abbr
0113     VMHIDCol= (find(~cellfun(@isempty,strfind(Data.textdata(Start,:),'VMH'))));
0114     MetConMin = (Data.data(:,1));% min = 1st col, max = 2nd col
0115     MetConMin = cellstr(num2str(MetConMin));
0116     MetConMin = regexprep(MetConMin,' ','');
0117     for j = 1 : size(MetConMin,1)
0118         MetConMin{j,1} = (MetConMin(j,1));
0119     end
0120     
0121     MetConMax = (Data.data(:,2));% min = 1st col, max = 2nd col
0122     MetConMax = cellstr(num2str(MetConMax));
0123     MetConMax = regexprep(MetConMax,' ','');
0124     for j = 1 : size(MetConMax,1)
0125         MetConMax{j,1} = (MetConMax(j,1));
0126     end
0127     metConcDataBc=[Data.textdata(Start+1:end,VMHIDCol) MetConMin MetConMax];
0128     maxConcColBc = 3;
0129     minConcColBc = 2;
0130     VMHIDCol = 1;
0131     
0132     % CSF;
0133     fileName='NormalCSFConcExtractedHMDB.txt';
0134     [Data] =importdata(fileName);
0135     
0136     % find start/header of data - Blood
0137     % find data start
0138     Start= (find(~cellfun(@isempty,strfind(Data.textdata(:,1),'####'))))+1;
0139     % find rxn abbr
0140     VMHIDCol= (find(~cellfun(@isempty,strfind(Data.textdata(Start,:),'VMH'))));
0141     MetConMin = (Data.data(:,1));% min = 1st col, max = 2nd col
0142     MetConMin = cellstr(num2str(MetConMin));
0143     MetConMin = regexprep(MetConMin,' ','');
0144     for j = 1 : size(MetConMin,1)
0145         MetConMin{j,1} = (MetConMin(j,1));
0146     end
0147     
0148     MetConMax = (Data.data(:,2));% min = 1st col, max = 2nd col
0149     MetConMax = cellstr(num2str(MetConMax));
0150     MetConMax = regexprep(MetConMax,' ','');
0151     for j = 1 : size(MetConMax,1)
0152         MetConMax{j,1} = (MetConMax(j,1));
0153     end
0154     metConcDataCSF=[Data.textdata(Start+1:end,VMHIDCol) MetConMin MetConMax];
0155     maxConcColCSF = 3;
0156     minConcColCSF = 2;
0157     VMHIDCol = 1;
0158     
0159     % URINE
0160     fileName='NormalUrineConcExtractedHMDB.txt';
0161     [Data] =importdata(fileName);
0162     
0163     % find start/header of data -
0164     % find data start
0165     Start= (find(~cellfun(@isempty,strfind(Data.textdata(:,1),'####'))))+1;
0166     % find rxn abbr
0167     VMHIDCol= (find(~cellfun(@isempty,strfind(Data.textdata(Start,:),'VMH'))));
0168     MetConMin = (Data.data(:,1));% min = 1st col, max = 2nd col
0169     MetConMin = cellstr(num2str(MetConMin));
0170     MetConMin = regexprep(MetConMin,' ','');
0171     for j = 1 : size(MetConMin,1)
0172         MetConMin{j,1} = (MetConMin(j,1));
0173     end
0174     
0175     MetConMax = (Data.data(:,2));% min = 1st col, max = 2nd col
0176     MetConMax = cellstr(num2str(MetConMax));
0177     MetConMax = regexprep(MetConMax,' ','');
0178     for j = 1 : size(MetConMax,1)
0179         MetConMax{j,1} = (MetConMax(j,1));
0180     end
0181     metConcDataUr=[Data.textdata(Start+1:end,VMHIDCol) MetConMin MetConMax];
0182     maxConcColUr = 3;
0183     minConcColUr = 2;
0184     VMHIDCol = 1;
0185     
0186 elseif strcmp(Type,'direct')%direct data input
0187     VMHIDCol = 1; % first column corresponds to vmh id's, 2nd to data points (min), 3rd to data points (max)
0188     setLB = 1;
0189     if strcmp(Biofluid,'bc')%blood data
0190         minConcColBc = 2;
0191         maxConcColBc = 3;
0192         metConcDataBc = InputData;
0193     elseif strcmp(Biofluid,'csf')%blood data
0194         minConcColCSF = 2;
0195         maxConcColCSF = 3;
0196         metConcDataCSF = InputData;
0197     elseif strcmp(Biofluid,'u')%blood data
0198         minConcColUr = 2;
0199         maxConcColUr = 3;
0200         metConcDataUr = InputData;
0201     end
0202 end
0203 %% compute constraints
0204 % Feher, p. 550 Q8!
0205 % the following assumptions are made
0206 % 1. steady-state
0207 % 2. transport is bulk flow limited, not diffusion limited,  which is at
0208 %    least true for higher blood flow rates
0209 % 3. the metabolites is maximally consumed by tissue (gives an upper bound),
0210 %    for metabolites for which the venal concentration is known the
0211 %    difference between arterial and venal concentration should be rather used.
0212 % Equation:
0213 % Q = (ca-cv)*Qv - Feher, p 550, Ex 8
0214 % vmax_met_organ = conc_met_max * k_blood_organ % no organ scaling
0215 % necessary as those numbers are already adjusted to organs (in part to
0216 % weight but also requirements)
0217 
0218 % cardiac output and organ-specific blood flow rate
0219 for i = 1 : length(OrgansListExt)
0220     BloodFlowOrgan(i,1)=OrgansListExt(i); %percentage of blood flow going to each organ
0221     tmp = strmatch(OrgansListExt(i),bloodFlowData(:,bloodFlowOrganCol),'exact');
0222     if ~isempty(tmp) && tmp>0
0223         if strcmp(IndividualParameters.gender,'male') % use first col
0224             B = bloodFlowData{tmp,bloodFlowPercCol(1)};
0225         elseif strcmp(IndividualParameters.gender,'female') % use 2nd col
0226             B = bloodFlowData{tmp,bloodFlowPercCol(2)};
0227         end
0228         B = str2num(B(2:end-1));
0229         if  ~isempty(B)
0230             BloodFlowRate(i,1)=(B)*CardiacOutput; % k_blood_organ in ml/min
0231             PlasmaFlowRate(i,1)=(B)*CardiacOutput*(1-Hematocrit); % k_plasma_organ in ml/min
0232         else
0233             % if no information is provided for percentage, assume 1%
0234             BloodFlowRate(i,1)=0.01*CardiacOutput; % k_blood_organ in ml/min
0235             PlasmaFlowRate(i,1)=0.01*CardiacOutput*(1-Hematocrit); % k_blood_organ in ml/min
0236         end
0237     elseif strcmp('BBB',OrgansListExt(i))% exception of BBB which gets the flow rate of Scord and Brain
0238         Scord = strmatch('Scord',bloodFlowData(:,bloodFlowOrganCol),'exact');
0239         Brain = strmatch('Brain',bloodFlowData(:,bloodFlowOrganCol),'exact');
0240         
0241         if strcmp(IndividualParameters.gender,'male') % use first col
0242             BScord = bloodFlowData{Scord,bloodFlowPercCol(1)};
0243             BBrain = bloodFlowData{Brain,bloodFlowPercCol(1)};
0244         elseif strcmp(IndividualParameters.gender,'female') % use 2nd col
0245             BScord = bloodFlowData{Scord,bloodFlowPercCol(2)};
0246             BBrain = bloodFlowData{Brain,bloodFlowPercCol(2)};
0247         end
0248         
0249         BScord = str2num(BScord(2:end-1));
0250         BBrain = str2num(BBrain(2:end-1));
0251         B = BBrain + BScord;
0252         
0253         BloodFlowRate(i,1)=(B)*CardiacOutput; % k_blood_organ in ml/min
0254         PlasmaFlowRate(i,1)=(B)*CardiacOutput*(1-Hematocrit); % k_plasma_organ in ml/min
0255     else
0256         % if no information is provided for percentage, assume 1%
0257         BloodFlowRate(i,1)=0.01*CardiacOutput; % k_blood_organ in ml/min
0258         PlasmaFlowRate(i,1)=0.01*CardiacOutput*(1-Hematocrit); % k_blood_organ in ml/min
0259     end
0260 end
0261 
0262 % Organs excluded from secretion into [bc]
0263 ExclOrgan={'sIEC', 'Colon','Spleen','Pancreas','Gall','Brain'};
0264 
0265 % compute maximal possible uptake and secretion rate for each metabolite in
0266 % each organ
0267 if strcmp( Biofluid, 'bc') || strcmp( Biofluid, 'all')
0268     for i = 1 : length(OrgansListExt)
0269         % find uptake and secretion reactions
0270         if ~isempty(strmatch('BBB',OrgansListExt{i})) %BBB for brain constraints
0271             ExR = find(~cellfun(@isempty,strfind(modelConstraint.rxns,'[CSF]upt'))); % uptake from [bc] into [csf] only
0272         else
0273             ExR = strmatch(strcat(OrgansListExt{i},'_EX_'),modelConstraint.rxns);
0274         end
0275         if ~isempty(ExR)
0276             for j = 1 : length(ExR)
0277                 % ensure that the exchange is FROM BLOOD
0278                 if (length(strfind(modelConstraint.rxns{ExR(j)},'[bc]'))>0 || length(strfind(modelConstraint.rxns{ExR(j)},'[CSF]upt'))>0) ...
0279                         && length(strfind(modelConstraint.rxns{ExR(j)},'_o2s(e)'))==0    ...
0280                         && length(strfind(modelConstraint.rxns{ExR(j)},'_h2o(e)'))==0 ...
0281                         && length(strfind(modelConstraint.rxns{ExR(j)},'_H2O[CSF]upt'))==0 %...
0282              %       && length(strfind(modelConstraint.rxns{ExR(j)},'_co2(e)'))==0
0283                     %&& length(strfind(modelConstraint.rxns{ExR(j)},'_o2(e)'))==0 ... %no oxygen constraint
0284                     
0285                     %  && length(strfind(modelConstraint.rxns{ExR(j)},'_aicar(e)'))==0    ...
0286                     % && length(strfind(modelConstraint.rxns{ExR(j)},'_CE2705(e)'))==0   % ...
0287                     
0288                     %&& length(strfind(modelConstraint.rxns{ExR(j)},'_h2o2(e)'))==0 % avoids setting uptake for o2s, co2, h2o2
0289                     %&& length(strfind(modelConstraint.rxns{ExR(j)},'_o2(e)'))==0 ... %no oxygen constraint
0290                     
0291                     % all reactions are written such that negative flux means
0292                     % uptake from (bc) and positive flux corresponds to secretion
0293                     % into (bc)
0294                     %
0295                     % get metabolite associated with reaction
0296                     ExM = modelConstraint.mets(find(modelConstraint.S(:,ExR(j))>0));
0297                     MCon = [];
0298                     if ~isempty(ExM)
0299                         % KIDNEY IS TREATED DIFFERENTLY - checked
0300                         % 29.04.2016 - IT
0301                         clear X
0302                         if ~isempty(strmatch(OrgansListExt(i),'Kidney','exact')) && length(strfind(modelConstraint.rxns{ExR(j)},'[bcK]'))==0
0303                             % get maximal concentration for metabolite
0304                             X = find(ismember(strcat(metConcDataBc(:,VMHIDCol),'[bc]'),ExM));
0305                             clear MConMin MConMax
0306                             if isempty(X) && ~strcmp(Type,'direct') % no concentration range/maximum defined in input data
0307                                 MConMin = 0;
0308                                 MConMax =  MConDefaultBc;
0309                             elseif ~isempty(X)
0310                                 MConMin1 = metConcDataBc{X,minConcColBc};
0311                                 MConMax1 = metConcDataBc{X,maxConcColBc};
0312                                 if ischar(MConMin1)
0313                                     MConMin = str2num(MConMin1(2:end-1));
0314                                     MConMax = str2num(MConMax1(2:end-1));
0315                                 elseif iscell(MConMin1)
0316                                     MConMin = MConMin1{1};
0317                                     MConMin = str2num(MConMin);
0318                                     MConMax = MConMax1{1};
0319                                     MConMax = str2num(MConMax);
0320                                 else
0321                                     MConMin = MConMin1;
0322                                     MConMax = MConMax1;
0323                                 end
0324                             else
0325                                 continue;
0326                             end
0327                             % MCon is in (umol/L) --> : 1000 to be in mmol/L
0328                             % PlasmaFlowRate is in (ml/min)
0329                             % PlasmaFlowRate*60*24/1000 (L/day)
0330                             % Q = (ca-cv)*Qv; where ca is the aterial
0331                             % concentration, which is typically not measured, and
0332                             % cv the venous concentration, reported in HMDB and
0333                             % measured in general experiments.
0334                             % in the kidney the flux can go only from
0335                             % [e]<--[bc] !!!!!!
0336                             
0337                             %  UPPER BOUND
0338                             MSecretRateKidney = 1*(MConMin/1000)*GlomerularFiltrationRate*60*24/1000; % in mmol/day/person
0339                             R = {'Kidney_EX_na1(e)_[bc]'
0340                                 'Kidney_EX_hco3(e)_[bc]'
0341                                 'Kidney_EX_urea(e)_[bc]'
0342                                 'Kidney_EX_k(e)_[bc]'
0343                                 'Kidney_EX_cl(e)_[bc]'
0344                                 'Kidney_EX_ca2(e)_[bc]'
0345                                 'Kidney_EX_HC02172(e)_[bc]'
0346                                 'Kidney_EX_avite1(e)_[bc]'
0347                                 };
0348                             R = unique([R;ExclList]);
0349                             if ~ismember(modelConstraint.rxns(ExR(j)),R)
0350                                 if setLB == 1
0351                                     modelConstraint.ub(ExR(j)) = -MSecretRateKidney; % maximal possible secretion rate
0352                                 else
0353                                     if MConMax>=MaxConcConstraint % set lower constraint if max is higher or equal to MinConcConstraint
0354                                         modelConstraint.ub(ExR(j)) = -MSecretRateKidney; % maximal possible secretion rate
0355                                     else
0356                                         modelConstraint.ub(ExR(j)) = 0; % maximal possible secretion rate
0357                                     end
0358                                 end
0359                             else
0360                                 modelConstraint.ub(ExR(j)) = 0;
0361                             end
0362                             % LOWER BOUND
0363                             MSecretRateKidney = 1*(MConMax/1000)*GlomerularFiltrationRate*60*24/1000; % in mmol/day/person
0364                             
0365                             modelConstraint.lb(ExR(j)) = -MSecretRateKidney; % maximal possible secretion rate
0366                             
0367                         else
0368                             %% checked this part of the code - 29.04. IT
0369                             % get maximal concentration for metabolite
0370                             X = find(ismember(strcat(metConcDataBc(:,VMHIDCol),'[bc]'),ExM));
0371                             clear MCon
0372                             if isempty(X) && setDefault == 1 && ~strcmp(Type,'direct')% no concentration range/maximum defined in input data; only if requested
0373                                 MCon =  MConDefaultBc;
0374                             elseif ~isempty(X)
0375                                 MCon1 = metConcDataBc{X,maxConcColBc};
0376                                 if ischar(MCon1)
0377                                     MCon = str2num(MCon1(2:end-1));
0378                                 elseif iscell(MCon1)
0379                                     MCon = MCon1{1};
0380                                     MCon = str2num(MCon);
0381                                 else
0382                                     MCon = MCon1;
0383                                 end
0384                             else
0385                                 continue;
0386                             end
0387                             R = {
0388                                 % added to the list of  rxns excluded to be
0389                                 % constraint - otw infeasible - Nov 2017 -
0390                                 % IT
0391                                 'BBB_NH4[CSF]upt'
0392                                 'BBB_CHOL[CSF]upt'
0393                                 'BBB_PI[CSF]upt'
0394                                 'BBB_STRDNC[CSF]upt'
0395                                 'BBB_HC00250[CSF]upt'
0396                                 'BBB_PYDXN[CSF]upt'
0397                                 'BBB_5MTHF[CSF]upt'
0398                                 'BBB_SO3[CSF]upt'
0399 
0400                                 };
0401                             
0402                             if ~ismember(modelConstraint.rxns(ExR(j)),R)
0403                             % MCon is in (umol/L) --> : 1000 to be in mmol/L
0404                             % PlasmaFlowRate is in (ml/min)
0405                             % PlasmaFlowRate*60*24/1000 (L/day)
0406                             % Q = (ca-cv)*Qv; where ca is the aterial
0407                             % concentration, which is typically not measured, and
0408                             % cv the venous concentration, reported in HMDB and
0409                             % measured in general experiments. ca is assumed to be
0410                             % 30% higher than cv, allowing the tissue to take up
0411                             % maximally 30% of the maximally reported cv value
0412                             if ~isempty(MCon)
0413                                 %  MUptakeRateBc = ((MCon*(100/70)-MCon)/1000)*PlasmaFlowRate(i,1)*60*24/1000; % in mmol/day/person
0414                                 MUptakeRateBc = ((MCon)/1000)*PlasmaFlowRate(i,1)*60*24/1000; % in mmol/day/person
0415                                 if  modelConstraint.lb(ExR(j)) < 0;
0416                                     if MCon>1e-3%abs(MUptakeRateBc)>1e-3; % at least 1 nM
0417                                         modelConstraint.lb(ExR(j)) = -1*MUptakeRateBc; % maximal possible uptake rate
0418                                     else
0419                                         MUptakeRateBc = ((1e-3)/1000)*PlasmaFlowRate(i,1)*60*24/1000; % in mmol/day/person
0420                                         
0421                                         modelConstraint.lb(ExR(j)) = -1*MUptakeRateBc;
0422                                     end
0423                                 end
0424                                 % I cannot set this constraint as an organ
0425                                 % could secrete a metabolite at a higher
0426                                 % local concentration than in the blood but
0427                                 % this gets then balance through the
0428                                 % constribution (or rather lack of) by other
0429                                 % organs
0430                                 %                                 % allowing the tissue to secrete maximally 30% of the maximally reported cv value
0431                                 %                                 if isempty(strmatch(OrgansListExt(i),ExclOrgan,'exact')) &&  modelConstraint.ub(ExR(j))>0
0432                                 %                                     % MSecretRateBc = -1*((MCon*(100/130)-MCon)/1000)*PlasmaFlowRate(i,1)*60*24/1000; % in mmol/day/person
0433                                 %                                     MSecretRateBc = ((MCon)/1000)*PlasmaFlowRate(i,1)*60*24/1000; % in mmol/day/person
0434                                 %                                     % secretion rate does not apply to organs that are only taking up from [bc]
0435                                 %                                     if abs(MCon)>1e-3; % at least 1 nM
0436                                 %                                         modelConstraint.ub(ExR(j)) = MSecretRateBc; % maximal possible secretion rate
0437                                 %                                     else
0438                                 %                                          MSecretRateBc = ((1e-3)/1000)*PlasmaFlowRate(i,1)*60*24/1000; % in mmol/day/person
0439                                 %
0440                                 %                                         modelConstraint.ub(ExR(j)) = MSecretRateBc; % maximal possible secretion rate
0441                                 %                                     end
0442                                 %                                 end0
0443                             end
0444                             end
0445                             
0446                         end
0447                     else
0448                         modelConstraint.rxns(ExR(j));
0449                     end
0450                 end
0451             end
0452         end
0453     end
0454     
0455 end
0456 %% BBB/Brain
0457 % compute maximal possible uptake and secretion rate for each metabolite in
0458 % each organ
0459 if strcmp( Biofluid, 'csf') || strcmp( Biofluid, 'all')
0460     for i = 1 : length(OrgansListExt)
0461         % find uptake and secretion reactions
0462         ExR = strmatch('BBB_',modelConstraint.rxns);
0463         if ~isempty(ExR)
0464             for j = 1 : length(ExR)
0465                 % ensure that only the export (exp) from csf-> bc receives
0466                 % constraints
0467                 if length(strfind(modelConstraint.rxns{ExR(j)},'[CSF]'))>0 && length(strfind(modelConstraint.rxns{ExR(j)},'exp'))>0 && length(strfind(modelConstraint.rxns{ExR(j)},'_o2(e)'))==0 ... %no oxygen constraint
0468                         && length(strfind(modelConstraint.rxns{ExR(j)},'_o2s(e)'))==0   && length(strfind(modelConstraint.rxns{ExR(j)},'_co2(e)'))==0
0469                     % all reactions are written such that negative flux means
0470                     % uptake from (bc) and positive flux corresponds to secretion
0471                     % into (bc)
0472                     
0473                     % get metabolite associated with reaction
0474                     ExM = modelConstraint.mets(find(modelConstraint.S(:,ExR(j))<0)); % reactions are written as [csf] <=> [bc]
0475                     if ~isempty(ExM)
0476                         % get maximal concentration for metabolite
0477                         X = find(ismember(strcat(metConcDataCSF(:,VMHIDCol),'[csf]'),ExM));
0478                         MConMin = [];
0479                         MConMax = [];
0480                         if isempty(X) && ~strcmp(Type,'direct') % no concentration range/maximum defined in input data
0481                             MConMin = 0;
0482                             MConMax =  MConDefaultCSF;
0483                         elseif ~isempty(X)
0484                             MConMin1 = metConcDataCSF{X,minConcColCSF};
0485                             MConMax1 = metConcDataCSF{X,maxConcColCSF};
0486                             if ischar(MConMin1)
0487                                 MConMin = str2num(MConMin1(2:end-1));
0488                                 MConMax = str2num(MConMax1(2:end-1));
0489                             elseif iscell(MConMin1)
0490                                 MConMin = MConMin1{1};
0491                                 MConMin = str2num(MConMin);
0492                                 MConMax = MConMax1{1};
0493                                 MConMax = str2num(MConMax);
0494                             else
0495                                 MConMin = MConMin1;
0496                                 MConMax = MConMax1;
0497                             end
0498                         else
0499                             continue;
0500                         end
0501                         if ~isempty(MConMin)
0502                             % MCon is in (umol/L) --> : 1000 to be in mmol/L
0503                             % PlasmaFlowRate is in (ml/min)
0504                             % PlasmaFlowRate*60*24/1000 (L/day)
0505                             % Q = (ca-cv)*Qv; where ca is the aterial
0506                             % concentration, which is typically not measured, and
0507                             % cv the venous concentration, reported in HMDB and
0508                             % measured in general experiments.
0509                             % LOWER BOUND
0510                             % flux will be positive as reaction is written as
0511                             % csf --> bc
0512                             MSecretRateCSF = (MConMin/1000)*CSFBloodFlowRate*60*24/1000; % in mmol/day/person
0513                             R = { 'na1[csf]'
0514                                 'cl[csf]'
0515                                 'k[csf]'
0516                                 'h2o[csf]'
0517                                 'sucsal[csf]'
0518                                 'ca2[csf]'
0519                                 'ser_D[csf]'};
0520                             if setLB == 1
0521                                 modelConstraint.lb(ExR(j)) = MSecretRateCSF; % maximal possible secretion rate
0522                             else
0523                                 if MConMin>=MinConcConstraint && MConMax>=MaxConcConstraint && isempty(find(ismember(R,ExM))) &&isempty(find(ismember(ExclList,modelConstraint.rxns(ExR(j))))) % || ismember(MustBeInCSF,ExM) % ismember(MustBeInCSF,ExM)
0524                                     modelConstraint.lb(ExR(j)) = MSecretRateCSF; % maximal possible uptake rate
0525                                 else
0526                                     modelConstraint.lb(ExR(j)) = 0;
0527                                 end
0528                             end
0529                             % UPPER BOUND
0530                             MSecretRateCSF = (MConMax/1000)*CSFFlowRate*60*24/1000; % in mmol/day/person
0531                             modelConstraint.ub(ExR(j)) = MSecretRateCSF; % maximal possible secretion rate
0532                             
0533                         end
0534                     else
0535                         modelConstraint.rxns(ExR(j));
0536                         
0537                     end
0538                 end
0539                 
0540             end
0541         end
0542     end
0543 end
0544 %% Urine excretion
0545 % constraints are set on Exchange reactions for urine
0546 if strcmp( Biofluid, 'u') || strcmp( Biofluid, 'all')
0547     
0548     % convert creatinine from mg/dL into mmol/L
0549     MWCreat = 113.1179;% g/mol
0550     MConDefaultUrCreatinineMax = MConDefaultUrCreatinineMax*10/MWCreat;
0551     MConDefaultUrCreatinineMin = MConDefaultUrCreatinineMin*10/MWCreat;
0552     ExR = strmatch('EX_',modelConstraint.rxns);
0553     if ~isempty(ExR)
0554         for j = 1 : length(ExR)
0555             if length(strfind(modelConstraint.rxns{ExR(j)},'[u]'))>0 && length(strfind(modelConstraint.rxns{ExR(j)},'_o2(e)'))==0 ... %no oxygen constraint
0556                     && length(strfind(modelConstraint.rxns{ExR(j)},'_o2s(e)'))==0   && length(strfind(modelConstraint.rxns{ExR(j)},'_co2(e)'))==0
0557                 
0558                 % all reactions are written such that positive flux corresponds to secretion
0559                 % into urine (u)
0560                 %
0561                 % get metabolite associated with reaction
0562                 ExM = modelConstraint.mets(find(modelConstraint.S(:,ExR(j))<0)); % this is a typical exchange reaction
0563                 MConMin = [];
0564                 MConMax = [];
0565                 if ~isempty(ExM)
0566                     % get maximal concentration for metabolite
0567                     X = find(ismember(strcat(metConcDataUr(:,VMHIDCol),'[u]'),ExM));
0568                     if isempty(X) && ~strcmp(Type,'direct')%&& setDefault == 1 % no concentration range/maximum defined in input data, only if requested
0569                         MConMin =  MConDefaultUrMin;
0570                         MConMax =  MConDefaultUrMax;
0571                     elseif ~isempty(X)
0572                         MConMin1 = metConcDataUr{X,minConcColUr};
0573                         MConMax1 = metConcDataUr{X,maxConcColUr};
0574                         if ischar(MConMin1)
0575                             MConMin = str2num(MConMin1(2:end-1));
0576                             MConMax = str2num(MConMax1(2:end-1));
0577                         elseif iscell(MConMin1)
0578                             MConMin = MConMin1{1};
0579                             MConMin = str2num(MConMin);
0580                             MConMax = MConMax1{1};
0581                             MConMax = str2num(MConMax);
0582                         else
0583                             MConMin = MConMin1;
0584                             MConMax = MConMax1;
0585                         end
0586                     else
0587                         continue;
0588                     end
0589                     % Urine excretion
0590                     % lower bound based on min concentration
0591                     if ~isempty(MConMin) && ~isempty(MConMax)
0592                         MSecrRateUrLB = (MConMin/1000)*MConDefaultUrCreatinineMin*UrFlowRate*60*24/1000; % in mmol/day/person
0593                         % upper bound based on max concentration
0594                         MSecrRateUrUB = (MConMax/1000)*MConDefaultUrCreatinineMax*UrFlowRate*60*24/1000; % in mmol/day/person
0595                         R = { 'EX_na1[u]'
0596                             'EX_cl[u]'
0597                             'EX_k[u]'
0598                             'EX_ca2[u]'
0599                             % non-unique list!
0600                             %'EX_aldstrn[u]'
0601                             %         'EX_tststerone[u]'
0602                             %         'EX_pydxn[u]'
0603                             %         'EX_3moxtyr[u]'
0604                             %                'EX_cl[u]'
0605                             %               'EX_k[u]'
0606                             %         %         %   'EX_nh4[u]'
0607                             %         'EX_sphgn[u]' % i dont think that this metabolite is routinely secreted
0608                             %         'EX_sphings[u]'
0609                             %         'EX_csn[u]'
0610                             %         'EX_arab_L[u]'
0611                             %         'EX_tststerone[u]'
0612                             %         'EX_tststerone[u]'
0613                             %         'EX_pydxn[u]'
0614                             %         'EX_mma[u]'%Methylamine occurs endogenously from amine catabolism and its tissue levels increase in some pathological conditions, including diabetes.
0615                             %        'EX_tsul[u]'%Thiosulfate occurs naturally in hot springs and geysers, and is produced by certain biochemical processes. In the body, thiosulfate converts small amounts of cyanide ion into harmless products and plays a role in the biosynthesis of cysteine, a sulfur-containing amino acid that locks proteins into their correct three-dimensional shapes. Thiosulfate is not found in large quantities in nature.
0616                             %
0617                             
0618                             % metabolites with lower bound that is non-zero
0619                             % in data but should be not set as lb
0620                             % constraints
0621                             'EX_C05767[u]' %Uroporphyrin I
0622                             'EX_C05770[u]' %Coproporphyrin III
0623                             'EX_C05302[u]'% 2-Methoxyestradiol (2ME2) is a drug that prevents the formation of new blood vessels
0624                             'EX_trypta[u]'%Tryptamine is a monoamine compound that is common precursor molecule to many hormones and neurotransmitters
0625                             'EX_ppbng[u]'% porphobilinogen is produced in excess and excreted in the urine in acute intermittent porphyria and several other porphyrias.
0626                             'EX_13dampp[u]'%  It is a catabolic byproduct of spermidine. "The excretion of these  substances is usually very small compared to the respective amino acids. "http://www.sciencedirect.com/science/article/pii/0009898171904426
0627                             'EX_mhista[u]' %The primary application of urinary N-methylhistamine (NMH) testing is in the diagnosis and monitoring of mast-cell disorders, including mastocytosis, anaphylaxis, and other severe systemic allergic reactions.[1, 2, 3, 4, 5, 6, 7]. The reference range for urinary NMH varies according to subject age, as follows: Age 0-5 years - 120-510 �g/g creatinine; Age 6-16 years - 70-330 �g/g creatinine, Older than16 years - 30-200 �g/g creatinine
0628                             'EX_tym[u]' %Tyramine and its conjugates occur in normal and abnormal urines, although the biological role of tyramine, if any, is obscure. However, it has recently become of interest because severe Parkinsonians excrete raised amounts of tyraminel-R
0629                             'EX_2hyoxplac[u]'%2-Hydroxyphenylacetate
0630                             'EX_pmtcrn[u]'
0631                             'EX_dheas[u]'
0632                             'EX_34dhphe[u]' %L-dopa
0633                             'EX_srtn[u]'
0634                             'EX_gthrd[u]'
0635                             'EX_pcholhep_hs[u]'
0636                             'EX_pcholste_hs[u]'
0637                             'EX_pcholn204_hs[u]'
0638                             'EX_3moxtyr[u]'
0639                             'EX_aldstrn[u]'
0640                             'EX_tststerone[u]'
0641                             'EX_pydxn[u]'
0642                             'EX_sphgn[u]' % i dont think that this metabolite is routinely secreted
0643                             'EX_sphings[u]'
0644                             'EX_csn[u]'
0645                             'EX_arab_L[u]'
0646                             'EX_tststerone[u]'
0647                             'EX_tststerone[u]'
0648                             'EX_pydxn[u]'
0649                             'EX_mma[u]'%Methylamine occurs endogenously from amine catabolism and its tissue levels increase in some pathological conditions, including diabetes.
0650                             'EX_tsul[u]'%Thiosulfate occurs naturally in hot springs and geysers, and is produced by certain biochemical processes. In the body, thiosulfate converts small amounts of cyanide ion into harmless products and plays a role in the biosynthesis of cysteine, a sulfur-containing amino acid that locks proteins into their correct three-dimensional shapes. Thiosulfate is not found in large quantities in nature.
0651                             'EX_5htrp[u]'
0652                             'EX_7dhchsterol'
0653                             'EX_etoh[u]'
0654                             'EX_gsn[u]'
0655                             'EX_5aop[u]'
0656                             'EX_uri[u]';
0657                             'EX_dad_2[u]'
0658                             'EX_ocdca[u]'
0659                             'EX_gua[u]'
0660                             'EX_dcyt[u]'
0661                             'EX_glyleu[u]'
0662                             'EX_acald[u]'
0663                             'EX_HC02191[u]'
0664                             %%
0665                             % 'EX_ethamp[u]'
0666                             };
0667                         R = unique([R;ExclList]);
0668                         MustSecrete = {
0669                             'EX_urea[u]'
0670                             'EX_nh4[u]'
0671                             'EX_etha[u]'
0672                             %   'EX_na1
0673                             'EX_lcts[u]'
0674                             'EX_3hmp[u]'
0675                             'EX_acnam[u]'
0676                             };
0677                         
0678                         if  modelConstraint.ub(ExR(j)) > 0;
0679                             if    setLB == 1
0680                                 if ~ismember(R,modelConstraint.rxns(ExR(j)))
0681                                     modelConstraint.lb(ExR(j)) = MSecrRateUrLB; % maximal possible uptake rate
0682                                     %        modelConstraint.lb(ExR(j)) = 0;
0683                                 else
0684                                     modelConstraint.lb(ExR(j)) = 0;
0685                                 end
0686                             else
0687                                 if MConMax>=MaxConcConstraint  && MConMin>=MinConcConstraint && ~ismember(modelConstraint.rxns(ExR(j)),R)%ismember(modelConstraint.rxns(ExR(j)),MustSecrete)% %
0688                                     modelConstraint.lb(ExR(j)) = MSecrRateUrLB; % maximal possible uptake rate
0689                                 else
0690                                     modelConstraint.lb(ExR(j)) = 0;
0691                                 end
0692                             end
0693                             modelConstraint.ub(ExR(j)) = MSecrRateUrUB; % maximal possible secretion rate
0694                         end
0695                     end
0696                     
0697                 else
0698                     modelConstraint.rxns(ExR(j));
0699                 end
0700             end
0701         end
0702     end
0703 end
0704 
0705 %% woman is not producing milk! - IT 20.12.2016
0706 % hence close all milk producing reactions
0707 tmp = find(~cellfun(@isempty,strfind(modelConstraint.rxns,'(miB)_[mi]')));
0708 modelConstraint.lb(tmp) = 0;
0709 modelConstraint.ub(tmp) = 0;
0710 
0711 if 1
0712     %% set o2[a] and co2[a] constraints
0713     % Put together by Maike
0714     % Composition air in: 78.62%�nitrogen, 21%�oxygen, 0.96%�argon, 0.04%�carbon dioxide, 0.5%�water vapour
0715     % Composition air out: 78.04% nitrogen, 14% - 16% oxygen, 4% - 5.3% carbon dioxide, 1% argon and other gases
0716     % Amount of O2 in:
0717     %   Tidal volume: 500 ml/breath
0718     %   Breathing frequency 12-15x/min
0719     %   Change of O2: 5%
0720     %   Volume of gas: 1mol gas = 22.4 l , 1mmol=22.4ml
0721     %   Volume O2/breath = 5*500 (ml)/100 = 25ml
0722     %   O2 change (mmol) = 25ml/22.4 ml = 1.1mmol
0723     %   Volume 02/day = 1.1mmol*12*60*24 = 19.080mol/day
0724     % Amount of CO2 out:
0725     %     Tidal volume: 500 ml/breath
0726     %     Breathing frequency 12-15x/min
0727     %     Change of CO2: 5.3%
0728     %     Volume of gas: 1mmol=22.4ml
0729     %
0730     %     Volume CO2/breath = 5.3*500 (ml)/100 = 26.5ml
0731     %     CO2 change (mmol) = 26.5ml/22.4 ml = 1.18mmol
0732     %     Volume 02/day = 1.18mmol*12*60*24 = 20.442 mol/day
0733     % Alternative calculation
0734     %     Ratio O2/CO2 = 0.8
0735     %     Tidal volume: 500 ml/breath
0736     %     Breathing frequency 12-15x/min
0737     %     Change of CO2: 4-5.3%
0738     %     Density of CO2 = 1.98g/l
0739     %
0740     %     Volume CO2/breath = 0.8*(0.05*0.5) = 0.02l
0741     %     Volume 02/day = 0.02l*12 = 0.24l
0742     %     Amount CO2/day = 1.98g/l*0.24l*60*24 =  0.475g*60*24= 684.288g/day
0743     %     Volume C02/day = 15.548 mol/day
0744     %
0745     %     Volume 02/day = 0.02l*15
0746     %                    = 0.30l ->19.436mol/day
0747     % Refs: http://biology.stackexchange.com/questions/5642/how-much-gas-is-exchanged-in-one-human-breath
0748     % https://en.wikipedia.org/wiki/Breathing#Breathing_in_gas
0749     % http://cozybeehive.blogspot.lu/2010/03/how-much-co2-do-you-exhale-while.html
0750     % http://www.convertunits.com/from/grams+CO2/to/moles
0751     if 1
0752         modelConstraint = changeRxnBounds(modelConstraint,'EX_o2[a]',-15000,'u');%change to 15k
0753         modelConstraint = changeRxnBounds(modelConstraint,'EX_o2[a]',-25000,'l');
0754         modelConstraint = changeRxnBounds(modelConstraint,'EX_co2[a]',15000*0.8,'l');
0755         modelConstraint = changeRxnBounds(modelConstraint,'EX_co2[a]',25000,'u');
0756     end
0757     % % %% water
0758     % % %breathing out of water
0759     % % % from Ref man
0760     % % % Sweat = 650 ml(water loss)/day - 650g = 650g/day / 18.01528g/mol = 36.0805 mol/day
0761     % % % Insensible(breathing??) 840g = 850/18.01528 = 47.1822 mol/day
0762     % % % Urine = 1400g = 1400/18.01528 = 77.7118 mol/day
0763     % % % Feces = 100g = 100/18.01528 = 5.5508 mol/day
0764     if 1
0765       %  modelConstraint = changeRxnBounds(modelConstraint,'EX_h2o[a]',36080*0.8,'l');%
0766        % modelConstraint = changeRxnBounds(modelConstraint,'EX_h2o[a]',36080*1.2,'u');
0767         
0768         
0769         modelConstraint = changeRxnBounds(modelConstraint,'EX_h2o[a]',47182*0.8,'l');%
0770         modelConstraint = changeRxnBounds(modelConstraint,'EX_h2o[a]',47182*1.2,'u');
0771         
0772         % % % sweating of water
0773         %modelConstraint = changeRxnBounds(modelConstraint,'EX_h2o[sw]',47182*0.8,'l');%
0774         %modelConstraint = changeRxnBounds(modelConstraint,'EX_h2o[sw]',47182*1.2,'u');
0775         
0776         modelConstraint = changeRxnBounds(modelConstraint,'EX_h2o[sw]',36080*0.8,'l');%
0777         modelConstraint = changeRxnBounds(modelConstraint,'EX_h2o[sw]',36080*1.2,'u');
0778         
0779         % % % water in urine
0780         modelConstraint = changeRxnBounds(modelConstraint,'EX_h2o[u]',77711*0.8,'l');%should be much higher
0781         modelConstraint = changeRxnBounds(modelConstraint,'EX_h2o[u]',77711*1.2,'u');
0782         % % % water in feces
0783         modelConstraint = changeRxnBounds(modelConstraint,'Excretion_EX_h2o[fe]',5550*0.8,'l');%
0784         modelConstraint = changeRxnBounds(modelConstraint,'Excretion_EX_h2o[fe]',5550*1.2,'u');
0785         
0786         % limit water secretion into bile duct
0787         modelConstraint = changeRxnBounds(modelConstraint,'Gall_H2Ot[bdG]',1000,'u'); % arbitrary number
0788         modelConstraint = changeRxnBounds(modelConstraint,'Liver_H2Ot[bdL]',1000,'u'); % arbitrary number
0789     end
0790     if 1
0791         % % %% specific reactions
0792         % % Muscle can only take up glc
0793         % set constrain only if the new constrain is tighter than existing one and
0794         % does not get smaller than LB
0795         if modelConstraint.ub(find(ismember(modelConstraint.rxns, 'Muscle_EX_glc_D(e)_[bc]')))>= -0.01*1000 && modelConstraint.lb(find(ismember(modelConstraint.rxns, 'Muscle_EX_glc_D(e)_[bc]')))<= -0.01*1000
0796             modelConstraint = changeRxnBounds(modelConstraint,'Muscle_EX_glc_D(e)_[bc]',-0.01*1000,'u');
0797         elseif modelConstraint.lb(find(ismember(modelConstraint.rxns, 'Muscle_EX_glc_D(e)_[bc]')))> modelConstraint.ub(find(ismember(modelConstraint.rxns, 'Muscle_EX_glc_D(e)_[bc]')))
0798             modelConstraint.ub(find(ismember(modelConstraint.rxns, 'Muscle_EX_glc_D(e)_[bc]')))=0; %reset earlier constraints if lb>ub
0799         end
0800         
0801         % 'Muscle_EX_ala_l(e)_[bc]'    'Muscle_ala_L[e]  <=> ala_L[bc] '    alanine secretion    muscle    12.5 mg alanine/min/person (65 kg)    C3H7NO2    89.09    0.233126398    0.233126398    'Muscle_EX_ala_l(e)_[bc]'    0.187    0.280    postabsorption state    Frayn book
0802         met = 12.5; % mg per min per 65 kg
0803         MW = 89.09; % g�mol?1
0804         met = (met * 60 * 24 *IndividualParameters.bodyWeight/65)/1000; %g per day per person (weight adjusted)
0805         met = met * 1000/ MW ; %mmol per day per person (weight adjusted)
0806         if modelConstraint.lb(find(ismember(modelConstraint.rxns, 'Muscle_EX_ala_L(e)_[bc]')))<met*0.80 && modelConstraint.ub(find(ismember(modelConstraint.rxns, 'Muscle_EX_ala_L(e)_[bc]')))>=met*0.8
0807             modelConstraint = changeRxnBounds(modelConstraint,'Muscle_EX_ala_L(e)_[bc]',met*0.8,'l');% to be in mmol/day/person
0808             modelConstraint = changeRxnBounds(modelConstraint,'Muscle_EX_ala_L(e)_[bc]',met*1.2,'u');
0809         elseif modelConstraint.lb(find(ismember(modelConstraint.rxns, 'Muscle_EX_ala_L(e)_[bc]')))> modelConstraint.ub(find(ismember(modelConstraint.rxns, 'Muscle_EX_ala_L(e)_[bc]')))
0810             modelConstraint.lb(find(ismember(modelConstraint.rxns, 'Muscle_EX_ala_L(e)_[bc]')))=0; %reset earlier constraints if lb>ub
0811         end
0812         
0813         
0814         %     RBC_EX_glc(e)_[bc]'    'RBC_glc_D[e]  <=> glc_D[bc] '    glucose uptake     RBC    25 mg/glc/min/person (65kg)    C6H12O6    180.16    0.230564285    -0.230564285    'RBC_EX_glc(e)_[bc]'    -0.184    -0.277        Frayn book
0815         met = 25; % mg per min per 65 kg
0816         MW = 180.16;% g�mol?1
0817         met = (met * 60 * 24 *IndividualParameters.bodyWeight/65)/1000; %g per day per person (weight adjusted)
0818         met = met * 1000/ MW ; %mmol per day per person (weight adjusted)
0819         % set constraints only if they make the range smaller
0820         if modelConstraint.lb(find(ismember(modelConstraint.rxns, 'RBC_EX_glc_D(e)_[bc]')))<-met*1.2 && modelConstraint.ub(find(ismember(modelConstraint.rxns, 'RBC_EX_glc_D(e)_[bc]')))>=-met*1.2
0821             modelConstraint = changeRxnBounds(modelConstraint,'RBC_EX_glc_D(e)_[bc]',-met*0.8,'u');
0822             modelConstraint = changeRxnBounds(modelConstraint,'RBC_EX_glc_D(e)_[bc]',-met*1.2,'l');
0823         elseif modelConstraint.lb(find(ismember(modelConstraint.rxns, 'RBC_EX_glc_D(e)_[bc]')))> modelConstraint.ub(find(ismember(modelConstraint.rxns, 'RBC_EX_glc_D(e)_[bc]')))
0824             modelConstraint.ub(find(ismember(modelConstraint.rxns, 'RBC_EX_glc_D(e)_[bc]')))=0; %reset earlier constraints if lb>ub
0825         end
0826     end
0827     
0828     if 1
0829         if 1
0830         % 'Brain_EX_glc(e)_[csf]'    'Brain_glc_D[e]  <=> glc_D[csf] '    glucose uptake     brain    80 mg glc/min/person    C6H12O6    180.16    0.737805711    -0.737805711    'Brain_EX_glc(e)_[csf]'    -0.590    -0.885    all day    Frayn book
0831         met = 80; % mg per min per 65 kg
0832         MW = 180.16;% g�mol?1
0833         met = (met * 60 * 24 *IndividualParameters.bodyWeight/65)/1000; %g per day per person (weight adjusted)
0834         met = met * 1000/ MW ; %mmol per day per person (weight adjusted)
0835         if modelConstraint.lb(find(ismember(modelConstraint.rxns, 'Brain_EX_glc_D(e)_[csf]')))<-met*1.20 && modelConstraint.ub(find(ismember(modelConstraint.rxns, 'Brain_EX_glc_D(e)_[csf]')))>=-met*1.2
0836             modelConstraint = changeRxnBounds(modelConstraint,'Brain_EX_glc_D(e)_[csf]',-met*0.8,'u');
0837             modelConstraint = changeRxnBounds(modelConstraint,'Brain_EX_glc_D(e)_[csf]',-met*1.2,'l');
0838         elseif modelConstraint.lb(find(ismember(modelConstraint.rxns, 'Brain_EX_glc_D(e)_[csf]')))> modelConstraint.ub(find(ismember(modelConstraint.rxns, 'Brain_EX_glc_D(e)_[csf]')))
0839             modelConstraint.ub(find(ismember(modelConstraint.rxns, 'Brain_EX_glc_D(e)_[csf]')))=0; %reset earlier constraints if lb>ub
0840         end
0841         end
0842         %% addition 21.12.2016
0843         %
0844         if 1
0845         brain_weight = cell2mat(IndividualParameters.OrgansWeights(find(ismember(IndividualParameters.OrgansWeights(:,1),'Brain')),2));
0846         brain_o2 = 156;% umol o2/100g brain/min; REF: http://link.springer.com/chapter/10.1007%2F978-1-59259-108-4_2#page-1
0847         brain_o2 = (brain_o2 * 60 * 24 * brain_weight/100)/1000; %mmol o2/person (brain)/day.
0848         if modelConstraint.lb(find(ismember(modelConstraint.rxns, 'Brain_EX_o2(e)_[csf]')))<-brain_o2*1.2 && modelConstraint.ub(find(ismember(modelConstraint.rxns, 'Brain_EX_o2(e)_[csf]')))>=-brain_o2*1.2
0849            modelConstraint = changeRxnBounds(modelConstraint,'Brain_EX_o2(e)_[csf]',-brain_o2*1.2,'l');
0850             modelConstraint = changeRxnBounds(modelConstraint,'Brain_EX_o2(e)_[csf]',-brain_o2*0.7,'u');
0851         elseif modelConstraint.lb(find(ismember(modelConstraint.rxns, 'Brain_EX_o2(e)_[csf]')))> modelConstraint.ub(find(ismember(modelConstraint.rxns, 'Brain_EX_o2(e)_[csf]')))
0852             modelConstraint.ub(find(ismember(modelConstraint.rxns, 'Brain_EX_o2(e)_[csf]')))=0; %reset earlier constraints if lb>ub
0853         end
0854         end
0855         
0856     end
0857     if 1
0858         
0859         % 'Liver_EX_ala_l(e)_[bc]'    'Liver_ala_L[e]  <=> ala_L[bc] '    alanine uptake    liver    12.5 mg alanine/min/person (65 kg)    C3H7NO2    89.09    0.233126398    -0.233126398    'Liver_EX_ala_l(e)_[bc]'    -0.187    -0.280    postabsorption state    Frayn book
0860         met = 12.5; % mg per min per 65 kg
0861         MW = 89.09; % g�mol?1
0862         met = (met * 60 * 24 *IndividualParameters.bodyWeight/65)/1000; %g per day per person (weight adjusted)
0863         met = met * 1000/ MW ; %mmol per day per person (weight adjusted)
0864         if modelConstraint.lb(find(ismember(modelConstraint.rxns, 'Liver_EX_ala_L(e)_[bc]')))<-met*1.20 && modelConstraint.ub(find(ismember(modelConstraint.rxns, 'Liver_EX_ala_L(e)_[bc]')))>=-met*1.2
0865             modelConstraint = changeRxnBounds(modelConstraint,'Liver_EX_ala_L(e)_[bc]',-met*0.8,'u');
0866             modelConstraint = changeRxnBounds(modelConstraint,'Liver_EX_ala_L(e)_[bc]',-met*1.2,'l');
0867         elseif modelConstraint.lb(find(ismember(modelConstraint.rxns, 'Liver_EX_ala_L(e)_[bc]')))> modelConstraint.ub(find(ismember(modelConstraint.rxns, 'Liver_EX_ala_L(e)_[bc]')))
0868             modelConstraint.ub(find(ismember(modelConstraint.rxns, 'Liver_EX_ala_L(e)_[bc]')))=0; %reset earlier constraints if lb>ub
0869         end
0870         
0871         % 'Liver_EX_glc(e)_[bc]'    'Liver_glc_D[e]  <=> glc_D[bc] '    glucose secretion    Liver    130 mg glc/min/person (65 kg    C6H12O6    180.16    1.198934281    1.198934281    'Liver_EX_glc(e)_[bc]'    0.959    1.439    postabsorption state    Frayn book
0872         met = 130; % mg per min per 65 kg
0873         MW = 180.16;% g�mol?1
0874         met = (met * 60 * 24 *IndividualParameters.bodyWeight/65)/1000; %g per day per person (weight adjusted)
0875         met = met * 1000/ MW ; %mmol per day per person (weight adjusted)
0876         if modelConstraint.lb(find(ismember(modelConstraint.rxns, 'Liver_EX_glc_D(e)_[bc]')))<met*0.80 && modelConstraint.ub(find(ismember(modelConstraint.rxns, 'Liver_EX_glc_D(e)_[bc]')))>=met*0.8
0877             modelConstraint = changeRxnBounds(modelConstraint,'Liver_EX_glc_D(e)_[bc]',met*1.2,'u');
0878             modelConstraint = changeRxnBounds(modelConstraint,'Liver_EX_glc_D(e)_[bc]',met*0.8,'l');
0879         elseif modelConstraint.lb(find(ismember(modelConstraint.rxns, 'Liver_EX_glc_D(e)_[bc]')))> modelConstraint.ub(find(ismember(modelConstraint.rxns, 'Liver_EX_glc_D(e)_[bc]')))
0880             modelConstraint.lb(find(ismember(modelConstraint.rxns, 'Liver_EX_glc_D(e)_[bc]')))=0; %reset earlier constraints if lb>ub
0881         end
0882     end
0883     if 1
0884         
0885         % 'Adipocytes_EX_glyc(e)_[bc]'    'Adipocytes_glyc[e]  <=> glyc[bc] '    glycerol secretion     adipocytes    12 mg glycerol/min/person (65 kg)    C3H8O3    92.09    0.216510604    0.216510604    'Adipocytes_EX_glyc(e)_[bc]'    0.173    0.260    postabsorption state    Frayn book
0886         met = 12; % mg per min per 65 kg
0887         MW =     92.09;% g�mol?1
0888         met = (met * 60 * 24 *IndividualParameters.bodyWeight/65)/1000; %g per day per person (weight adjusted)
0889         met = met * 1000/ MW ; %mmol per day per person (weight adjusted)
0890         if modelConstraint.lb(find(ismember(modelConstraint.rxns, 'Adipocytes_EX_glyc(e)_[bc]')))<met*0.80 && modelConstraint.ub(find(ismember(modelConstraint.rxns, 'Adipocytes_EX_glyc(e)_[bc]')))>=met*0.8
0891             % modelConstraint =     changeRxnBounds(modelConstraint,'Adipocytes_EX_glyc(e)_[bc]',met*0.8,'l');
0892             % the lower bound seems to create troubles so I removed it.
0893             modelConstraint = changeRxnBounds(modelConstraint,'Adipocytes_EX_glyc(e)_[bc]',met*1.2,'u');
0894         elseif modelConstraint.lb(find(ismember(modelConstraint.rxns, 'Adipocytes_EX_glyc(e)_[bc]')))> modelConstraint.ub(find(ismember(modelConstraint.rxns, 'Adipocytes_EX_glyc(e)_[bc]')))
0895             modelConstraint.lb(find(ismember(modelConstraint.rxns, 'Adipocytes_EX_glyc(e)_[bc]')))=0; %reset earlier constraints if lb>ub
0896         end
0897     end
0898     %% addition 19.12.2016
0899     % constrain growth rate of renewing organs
0900     % based on bionumbers
0901     % I am still not convinced that the constraints that I wanted to place
0902     % are correctly capturing the organ weight - I will leave it for the
0903     % moment. - IT 22.12.2-2016
0904     %   modelConstraint = changeRxnBounds(modelConstraint,'sIEC_biomass_reactionIEC01b_trtr',0.25,'l');% every 4 days turn over
0905     %   modelConstraint = changeRxnBounds(modelConstraint,'sIEC_biomass_reactionIEC01b_trtr',0.5,'u');% every 2 days turn over
0906     %   modelConstraint = changeRxnBounds(modelConstraint,'Stomach_biomass_reaction',0.11,'l');% every 9 days turn over
0907     %   modelConstraint = changeRxnBounds(modelConstraint,'Stomach_biomass_reaction',0.5,'u');% every 2 days turn over
0908     %
0909     %   modelConstraint = changeRxnBounds(modelConstraint, 'Colon_biomass_reaction',0.25,'l');% every 4 days turn over
0910     %   modelConstraint = changeRxnBounds(modelConstraint, 'Colon_biomass_reaction',0.33,'u');% every 3 days turn over
0911     %
0912     %   modelConstraint = changeRxnBounds(modelConstraint,  'Skin_biomass_reaction',0.033,'l');% every 30 days turn over
0913     %   modelConstraint = changeRxnBounds(modelConstraint,  'Skin_biomass_reaction',0.1,'u');% every 10 days turn over
0914     
0915     %% brain and liver can do co2 fixation - REF: http://www.jbc.org/content/237/8/2570.full.pdf
0916     
0917     %disallow carbon fixation -- Nov 2017
0918      L = (find(~cellfun(@isempty,strfind(modelConstraint.rxns,'EX_co2(e)_[bc]'))));
0919 %
0920    modelConstraint.lb(find(ismember(modelConstraint.rxns,modelConstraint.rxns(L)))) = 0;
0921     %% Co2 can cross BBB: http://www.sciencedirect.com/science/article/pii/0026286280900205
0922     modelConstraint = changeRxnBounds(modelConstraint,  'Brain_EX_co2(e)_[csf]',-10000,'l'); % arbiratry numbers
0923     modelConstraint = changeRxnBounds(modelConstraint,  'Liver_EX_co2(e)_[bc]',-10000,'l');
0924     % lung is also allowed to take up
0925     modelConstraint = changeRxnBounds(modelConstraint,  'Lung_EX_co2(e)_[bc]',-10000,'l');
0926     modelConstraint = changeRxnBounds(modelConstraint,  'Kidney_EX_co2(e)_[bc]',-10000,'l');
0927     if 1
0928         %% 10.01.17
0929         % brain atp requirement
0930         % apparently the brain consumes about 120g glc per day, corresponding to
0931         % 0.66 mol glc/day/person (MW=180.16)
0932         % 1 mol glc can be converted into 31 mol atp
0933         % hence 20.46 mol ATP could be theoretically produced from 0.66 mol glc
0934         % (if complete ox phos)
0935         % I will set the lower bound on DM_atp to 10 mol/day/person (this is an
0936         % arbitrary number). The GF Harvey under Av EU diet can produce max  12799.7
0937         % mmol ATP/person/day
0938         % ref: https://www.ncbi.nlm.nih.gov/books/NBK22436/, section 30.2
0939         
0940       %  modelConstraint = changeRxnBounds(modelConstraint,'Brain_DM_atp_c_',10000,'l');
0941         modelConstraint = changeRxnBounds(modelConstraint,'Brain_DM_atp_c_',0,'l');
0942     end
0943     
0944     if 1
0945     %% heart energy requirement - minimum
0946     % https://heartmdinstitute.com/heart-health/metabolic-cardiology-basics/
0947     % reports a minimum of 6000g of ATP per day per person, MW_ATP =
0948     % 507.18g/mol
0949     % hence lb = 11830 mmol/day/person
0950     modelConstraint = changeRxnBounds(modelConstraint,'Heart_DM_atp_c_',6000,'l');
0951     end
0952     % Also check this for future efforts: http://hypertextbook.com/facts/2003/IradaMuslumova.shtml
0953     
0954     %% constraint conversion of h2o + co2 to h + hco3
0955     
0956     % R = (find(~cellfun(@isempty,strfind(modelConstraint.rxns, 'RBC_H2CO3D'))));
0957     % modelConstraint.lb(R)=0;
0958     % modelConstraint.ub(R)=150; % no reference for this value except to avoid too high flux through this reaction
0959     
0960     %% o2 uptake lower bound constraints
0961     % each red blood cell contains ~ 270*10^6 haemoglobin, each of which can
0962     % carry up to 4 o2: e.g., https://en.wikipedia.org/wiki/Red_blood_cell
0963     % so one red blood cell carries 4*270*10^6 O2
0964     % The avogadro number is 6.022140857(74)�10^23 mol?1
0965     % The normal range in men is approximately 4.7 to 6.1 million cells/ul (microliter). The normal range in women range from 4.2 to 5.4 million cells/ul, according to NIH (National Institutes of Health) data.
0966     % men: assumed 5.5M/ul and  female: assumed 4.5M/ul
0967     % if strcmp(gender,'male')
0968     %     RBC = 5.5*10^6*
0969 end
```

---

Generated on Thu 14-May-2020 13:05:49 by **m2html** © 2005
