# Supplementary material for: Personalized whole‐body models integrate metabolism, physiology, and the gut microbiome
Source: Mol Syst Biol. 2020 May 28;16(5):e8982. doi: 10.15252/msb.20198982 (PMC7285886; doi:10.15252/msb.20198982)
Supplement: Supplementary file 22 — Dataset EV1 [file MSB-16-e8982-s022.zip › PSCM_toolbox/PSCM_toolbox_doc/src/setConstraints/readInDietFromVMH.html]

Description of readInDietFromVMH


# readInDietFromVMH

## PURPOSE

**This function reads in the diet that has been created and downloaded from**

## SYNOPSIS

**function [DietFormulation] = readInDietFromVMH(fileNameDiet)**

## DESCRIPTION

```
 This function reads in the diet that has been created and downloaded from
 the https://www.vmh.life/#nutrition and converts it into the whole-body
 metabolic model consistent format.
 
 [DietFormulation] = readInDietFromVMH(fileNameDiet)
 
 INPUT 
 fileNameDiet      File name

 OUTPUT
 DietFormulation   Diet definition

 Ines Thiele 2016-2019
```

## CROSS-REFERENCE INFORMATION

This function calls:


This function is called by:

## SOURCE CODE

```
0001 function [DietFormulation] = readInDietFromVMH(fileNameDiet)
0002 % This function reads in the diet that has been created and downloaded from
0003 % the https://www.vmh.life/#nutrition and converts it into the whole-body
0004 % metabolic model consistent format.
0005 %
0006 % [DietFormulation] = readInDietFromVMH(fileNameDiet)
0007 %
0008 % INPUT
0009 % fileNameDiet      File name
0010 %
0011 % OUTPUT
0012 % DietFormulation   Diet definition
0013 %
0014 % Ines Thiele 2016-2019
0015 
0016 [Numbers, Strings] = xlsread(fileNameDiet{1});
0017 
0018 ColFlux = 1;% assumes that fluxValues are given in 2nd col
0019 
0020 DietNames = Strings(2:end,6); % assumes that Rxn names are given in 6th column
0021 
0022 DietNames = regexprep(DietNames,'EX_','Diet_EX_');
0023 DietNames = regexprep(DietNames,'\(e\)','\[d\]');
0024 % Diet exchanges for all individuals
0025 Diets = cellstr(num2str((Numbers(:,ColFlux))));
0026 
0027 DietFormulation = [DietNames  Diets];
```

---

Generated on Thu 14-May-2020 13:05:49 by **m2html** © 2005
