# Supplementary material for: Personalized whole‐body models integrate metabolism, physiology, and the gut microbiome
Source: Mol Syst Biol. 2020 May 28;16(5):e8982. doi: 10.15252/msb.20198982 (PMC7285886; doi:10.15252/msb.20198982)
Supplement: Supplementary file 22 — Dataset EV1 [file MSB-16-e8982-s022.zip › PSCM_toolbox/PSCM_toolbox_doc/src/setConstraints/individualizedLabReport.html]

Description of individualizedLabReport


# individualizedLabReport

## PURPOSE

**This function computes personalized physiolgical parameters based on the**

## SYNOPSIS

**function [modelPersonalized,IndividualParametersNew] = individualizedLabReport(model,IndividualParameters, InputData,optionCardiacOutput)**

## DESCRIPTION

```
 This function computes personalized physiolgical parameters based on the
 provided input data.

 function [modelPersonalized,IndividualParametersNew] = individualizedLabReport(model,IndividualParameters, InputData,optionCardiacOutput)

 INPUT
 model                     model structure, whole-body metabolic model
 IndividualParameters      structure with Individual parameters to be
                           personalized or updated based on the input
                           data. This structure, with default parameters, can be obtained using the
                           script standardPhysiolDefaultParameters.m
 InputData                 InputData
 optionCardiacOutput       Different ways of calculatibg the cardiac
                           output based on physiological data have been implemented.
                           - Based on heart rate and stroke volume: CardiacOutput = HeartRate * StrokeVolume. (optionCardiacOutput = 1, default)
                           - Assume that cardiac output = blood volume.(optionCardiacOutput = 2)
                           - Based on the polynomial suggested by Youndg et al [http://www.ams.sunysb.edu/~hahn/psfile/pap_obesity.pdf]: CardiacOutput = 9119-exp(9.164-2.91e-2*Wt+3.91e-4*Wt^2-1.91e-6*Wt^3); Wt = weight in kg (optionCardiacOutput = 0)
                           - Based on Fick's principle, which requires that the oxygen consumption rate is known: VO_2 = (CO* C_a) - (CO *C_v);  where CO = Cardiac Output, Ca = Oxygen concentration of arterial blood and Cv = Oxygen concentration of mixed venous blood. We assume that C_a is 200 ml O2/L and C_v is 150 ml O2/L. (optionCardiacOutput = 3)
                           - Based on Fick's principle, while estimating the VO_2 based on the body surface area (BSA):   We use the Du Bois formula,[4][5]: BSA=0.007184 * Wt^0.425* Ht^0.725; Wt = weight in kg; Ht in cm; (optionCardiacOutput = 4)

 OUTPUT
 modelPersonalized         Updated model structure
 IndividualParametersNew   Updated, personalized individual parameters

 Ines Thiele, 2016-2018
```

## CROSS-REFERENCE INFORMATION

This function calls:

- physiologicalConstraintsHMDBbased This function applies constraints to the whole-body metabolic model

This function is called by:

- perform\_BMR\_newData This script repeats the simulation described in Thiele et al., "Personalized whole-body models integrate metabolism, physiology, and the gut microbiome", Method section 3.9.2 Validation of the parameters in an independent data set.
- perform\_sensi\_BMR\_all This script repeats the simulation described in Thiele et al.,

## SOURCE CODE

```
0001 function [modelPersonalized,IndividualParametersNew] = individualizedLabReport(model,IndividualParameters, InputData,optionCardiacOutput)
0002 % This function computes personalized physiolgical parameters based on the
0003 % provided input data.
0004 %
0005 % function [modelPersonalized,IndividualParametersNew] = individualizedLabReport(model,IndividualParameters, InputData,optionCardiacOutput)
0006 %
0007 % INPUT
0008 % model                     model structure, whole-body metabolic model
0009 % IndividualParameters      structure with Individual parameters to be
0010 %                           personalized or updated based on the input
0011 %                           data. This structure, with default parameters, can be obtained using the
0012 %                           script standardPhysiolDefaultParameters.m
0013 % InputData                 InputData
0014 % optionCardiacOutput       Different ways of calculatibg the cardiac
0015 %                           output based on physiological data have been implemented.
0016 %                           - Based on heart rate and stroke volume: CardiacOutput = HeartRate * StrokeVolume. (optionCardiacOutput = 1, default)
0017 %                           - Assume that cardiac output = blood volume.(optionCardiacOutput = 2)
0018 %                           - Based on the polynomial suggested by Youndg et al [http://www.ams.sunysb.edu/~hahn/psfile/pap_obesity.pdf]: CardiacOutput = 9119-exp(9.164-2.91e-2*Wt+3.91e-4*Wt^2-1.91e-6*Wt^3); Wt = weight in kg (optionCardiacOutput = 0)
0019 %                           - Based on Fick's principle, which requires that the oxygen consumption rate is known: VO_2 = (CO* C_a) - (CO *C_v);  where CO = Cardiac Output, Ca = Oxygen concentration of arterial blood and Cv = Oxygen concentration of mixed venous blood. We assume that C_a is 200 ml O2/L and C_v is 150 ml O2/L. (optionCardiacOutput = 3)
0020 %                           - Based on Fick's principle, while estimating the VO_2 based on the body surface area (BSA):   We use the Du Bois formula,[4][5]: BSA=0.007184 * Wt^0.425* Ht^0.725; Wt = weight in kg; Ht in cm; (optionCardiacOutput = 4)
0021 %
0022 % OUTPUT
0023 % modelPersonalized         Updated model structure
0024 % IndividualParametersNew   Updated, personalized individual parameters
0025 %
0026 % Ines Thiele, 2016-2018
0027 
0028 
0029 if ~exist('optionCardiacOutput','var')
0030     optionCardiacOutput = 1; %CO estimation based on heart rate
0031 end
0032 %% update individual parameters
0033 % ID
0034 ID = ismember(lower(InputData(:,1)),'id');
0035 if ~isempty(find(ID))
0036     ID = InputData(ID,3);
0037     IndividualParameters.ID =ID;
0038 end
0039 
0040 InputDataSex = ismember(lower(InputData(:,1)),'sex');
0041 G = InputData(InputDataSex,3);
0042 if strcmp('male',lower(G))
0043     IndividualParameters.sex = 'male';
0044 else
0045     IndividualParameters.sex = 'female';
0046 end
0047 
0048 % Age
0049 Age = ismember(lower(InputData(:,1)),'age');
0050 updated = 0;
0051 if ~isempty(find(Age))
0052     A = InputData(Age,3);
0053     if ischar(A)
0054         IndividualParameters.age =str2num(char(A{1})) ;
0055     elseif iscell(A)
0056         try
0057             IndividualParameters.age =str2num((A{1})) ;
0058             updated = 1;
0059         end
0060         if updated ==0
0061             try
0062                 IndividualParameters.age =A{1} ;
0063             end
0064         end
0065     else
0066         IndividualParameters.age =A{1} ;
0067     end
0068 end
0069 
0070 % weight
0071 Weight = ismember(lower(InputData(:,1)),'weight');
0072 updated =0;
0073 if ~isempty(find(Weight))
0074     W = InputData(Weight,3);
0075     if ischar(W)
0076         IndividualParameters.bodyWeight =str2num(char(W{1})) ;
0077     elseif iscell(W)
0078         try
0079             IndividualParameters.bodyWeight =str2num((W{1})) ;
0080             updated = 1;
0081         end
0082         if updated ==0
0083             try
0084                 IndividualParameters.bodyWeight =W{1} ;
0085             end
0086         end
0087     else
0088         IndividualParameters.bodyWeight =W{1} ;
0089     end
0090 end
0091 
0092 % Hematocrit given as fraction in IndividualParameters but in percentage in
0093 % InputData
0094 Hematocrit = ismember(lower(InputData(:,1)),'hematocrit');
0095 if ~isempty(find(Hematocrit))
0096     He = InputData(Hematocrit,3);
0097     IndividualParameters.Hematocrit =str2num(char(He{1}))/100 ;
0098 end
0099 
0100 % Creatinine given in mg/dL in InputData and in IndividualParameters
0101 Creatinine = ismember(lower(InputData(:,1)),'creatinine');
0102 if ~isempty(find(Creatinine))
0103     Cr = InputData(Creatinine,3);
0104     IndividualParameters.MConUrCreatinineMin =str2num(char(Cr{1})); % minimum
0105     IndividualParameters.MConUrCreatinineMax =str2num(char(Cr{1})); % maximum
0106 end
0107 
0108 % heart rate given in beats per min in InputData and in IndividualParameters
0109 HeartRate = ismember(lower(InputData(:,1)),'heartrate');
0110 if ~isempty(find(HeartRate))
0111     HR = InputData(HeartRate,3);
0112     if ischar(HR{1})
0113         IndividualParameters.HeartRate =str2num(char(HR{1}));
0114     else
0115         IndividualParameters.HeartRate =HR{1};
0116     end
0117 end
0118 
0119 % VO2 for CO estimation
0120 VO2 = ismember(lower(InputData(:,1)),'vo2');
0121 if ~isempty(find(VO2))
0122     VO2 = InputData(VO2,3);
0123     if ischar(VO2{1})
0124         IndividualParameters.VO2 =str2num(char(VO2{1}));
0125     else
0126         IndividualParameters.VO2 =VO2{1};
0127     end
0128 end
0129 %% estimate blood volume:
0130 %http://www.mc.vanderbilt.edu/documents/vmcpathology/files/TBV%20caclulation.docx.pdf
0131 % Nadler's equation
0132 %For Males = 0.3669 * Ht in M3 + 0.03219 * Wt in kgs + 0.6041 For    Females = 0.3561 * Ht in M3 + 0.03308 x Wt in kgs + 0.1833
0133 %Note: * Ht in M = Height in Meters, which is then cubed * Wt in kgs = Body weight in kilograms
0134 
0135 % read in height given in cm in InputData and in IndividualParameters
0136 Height = ismember(lower(InputData(:,1)),'height');
0137 updated = 0;
0138 if ~isempty(find(Height))
0139     H = InputData(Height,3);
0140     if ischar(H)
0141         IndividualParameters.Height =str2num(char(H{1})) ;
0142     elseif iscell(H)
0143         try
0144             IndividualParameters.Height =str2num((H{1})) ;
0145             updated = 1;
0146         end
0147         if updated ==0
0148             try
0149                 IndividualParameters.Height = H{1} ;
0150             end
0151         end
0152     else
0153         IndividualParameters.Height = H{1} ;
0154     end
0155 end
0156 
0157 % blood volume in ml/min
0158 if strcmp(IndividualParameters.sex, 'male')
0159     IndividualParameters.BloodVolume = (0.3669 * (IndividualParameters.Height/100)^3 + 0.03219 * IndividualParameters.bodyWeight + 0.6041)*1000;
0160 elseif strcmp(IndividualParameters.sex, 'female')
0161     IndividualParameters.BloodVolume = (0.3561 * (IndividualParameters.Height/100)^3 + 0.03308 * IndividualParameters.bodyWeight + 0.1833)*1000;
0162 end
0163 
0164 %% estimate (resting) cardiac output from blood volume in case that no stroke volume is provided
0165 StrokeVolume = ismember(lower(InputData(:,1)),'StrokeVolume');
0166 if optionCardiacOutput ~=-1 % skip adjustment of CO
0167     if ~isempty(find(StrokeVolume))
0168         S = InputData(StrokeVolume,3);
0169         IndividualParameters.StrokeVolume =str2num(char(S{1})) ;
0170         IndividualParameters.CardiacOutput = IndividualParameters.HeartRate * IndividualParameters.StrokeVolume; % in ml/min = beats/min * ml/beat
0171         IndividualParameters.CardiacOutput_Note = 'Calculated from personalized StrokeVolume and heart rate';
0172     elseif optionCardiacOutput == 1
0173         
0174         % actually I think that it makes more sense to keep the cardiac output to
0175         % be calculated based on default strokevolume and heart rate
0176         IndividualParameters.CardiacOutput = IndividualParameters.HeartRate * IndividualParameters.StrokeVolume; % in ml/min = beats/min * ml/beat
0177         IndividualParameters.CardiacOutput_Note = 'Calculated from default StrokeVolume and heart rate';
0178     elseif optionCardiacOutput == 2
0179         IndividualParameters.StrokeVolume ='NaN';
0180         IndividualParameters.CardiacOutput = IndividualParameters.BloodVolume;
0181         IndividualParameters.CardiacOutput_Note = 'Estimated from BloodVolume'; % in ml/min = beats/min * ml/beat
0182     elseif optionCardiacOutput == 0
0183         % With the blood volume estimate the CO gets too low.
0184         % hence I used the equation given here:
0185         % http://www.ams.sunysb.edu/~hahn/psfile/pap_obesity.pdf
0186         % note that the weight here is given in kg rather than g
0187         
0188         Wt = IndividualParameters.bodyWeight;
0189         IndividualParameters.CardiacOutput = 9119-exp(9.164-2.91e-2*Wt+3.91e-4*Wt^2-1.91e-6*Wt^3);
0190         IndividualParameters.CardiacOutput_Note = 'Estimated from CO equation'; % in ml/min = beats/min * ml/beat
0191     elseif optionCardiacOutput == 3
0192         % from wikipedia: https://en.wikipedia.org/wiki/Fick_principle
0193         %     VO_2 = (CO \times\ C_a) - (CO \times\ C_v)
0194         % where CO = Cardiac Output, Ca = Oxygen concentration of arterial blood and Cv = Oxygen concentration of mixed venous blood.
0195         % Note that (Ca ? Cv) is also known as the arteriovenous oxygen difference.
0196         % Cardiac Output = (125 ml O2/minute x 1.9) / (200 ml O2/L - 150 ml O2/L) = 4.75 L/minute
0197         % can be refined to account for haemoglobin content
0198         IndividualParameters.CardiacOutput = ((IndividualParameters.VO2*1000)*60*24/(200 - 150));
0199         IndividualParameters.CardiacOutput_Note = 'Estimated from VO2 ';
0200     elseif optionCardiacOutput == 4 %
0201         %Cardiac Output = (125 ml O2/minute x 1.9) / (200 ml O2/L - 150 ml O2/L) = 4.75 L/minute
0202         % Various calculations have been published to arrive at the BSA without direct measurement. In the following formulae, BSA is in m2, W is mass in kg, and H is height in cm.
0203         % The most widely used is the Du Bois, Du Bois formula,[4][5] which has been shown to be equally as effective in estimating body fat in obese and non-obese patients, something the Body mass index fails to do.[6]
0204         % BSA=0.007184 * W^{0.425}* H^{0.725}}
0205         W = IndividualParameters.bodyWeight;
0206         H = IndividualParameters.Height;
0207         BSA=0.007184 * W^0.425* H^0.725 ;
0208         IndividualParameters.CardiacOutput = ((0.125*1000*BSA)*60*24/(200 - 150));
0209         IndividualParameters.CardiacOutput_Note = 'Estimated from surface area ';
0210     elseif optionCardiacOutput == 5 %
0211         % estimation of vO2max
0212         % file:///Users/ines.thiele/Dropbox/work/Papers/SystemsPhysiology/schneider2013.pdf
0213         % for males: VO2max/kg = -0.42 A + 58, where A is age
0214         % for females: VO2max/kg = -0.35 A + 46, where A is age
0215         % assuming that at low activity the vo2 is 25% of vo2max
0216         % "At low exercise intensities (25% of maximal oxygen uptake (VO2max)), which in an average
0217         % healthy untrained young adult (VO2max per kg body mass = 42 ml kg?1min?1)
0218         % corresponds with level walking at 4?5 km h?1
0219         W = IndividualParameters.bodyWeight;
0220         A = IndividualParameters.age;
0221         if strcmp(IndividualParameters.sex,'male')
0222             VO2max = (-0.42* A + 58)*W; %ml/min
0223         elseif strcmp(IndividualParameters.sex,'female')
0224             VO2max = (-0.35* A + 46)*W;
0225         end
0226         VO2 = 0.07*VO2max;
0227         IndividualParameters.CardiacOutput = ((VO2)*60*24/(200 - 150));
0228     elseif  optionCardiacOutput == 6 %
0229         %estimation of stroke volume based on Frick
0230         % http://circ.ahajournals.org/content/circulationaha/14/2/250.full.pdf
0231         PP = 40; % pulse pressure
0232         DP = 80; % diatstolic blood pressure
0233         IndividualParameters.StrokeVolume = 91.0 + 0.54 * PP - 0.57*DP-0.61 *IndividualParameters.age ;
0234         IndividualParameters.CardiacOutput = IndividualParameters.HeartRate * IndividualParameters.StrokeVolume; % in ml/min = beats/min * ml/beat
0235     elseif  optionCardiacOutput == 7 %
0236         %estimation of stroke volume based on Bridwell
0237         % http://circ.ahajournals.org/content/circulationaha/14/2/250.full.pdf
0238         PP = 40; % pulse pressure
0239         DP = 80; % diatstolic blood pressure
0240         IndividualParameters.StrokeVolume = 66.0 + 0.34 * PP - 0.11*DP-0.36 *IndividualParameters.age ;
0241         IndividualParameters.CardiacOutput = IndividualParameters.HeartRate * IndividualParameters.StrokeVolume; % in ml/min = beats/min * ml/beat
0242         
0243     end
0244 end
0245 %% apply HMDB data based on IndividualParameters
0246 modelPersonalized = model;
0247 %modelPersonalized = physiologicalConstraintsHMDBbased(modelPersonalized,IndividualParameters);
0248 
0249 %% prepare for the urine and blood concentration data
0250 %
0251 if 0
0252     %InputDataMetabolites=[Data.textdata(Start+1:end,VMH) MetConMin MetConMax];
0253     Type = 'direct';
0254     % first blood
0255     % glucose
0256     MW_glc= 180.16;% g�mol?1;
0257     Glc = ismember(InputData(:,1),'Glucose');
0258     if ~isempty(find(Glc))
0259         Glc = InputData(Glc,3);
0260         Glc =str2num(char(Glc{1})) ;
0261         % calculate glucose in mM
0262         GlcMin = Glc * 10 * (1/MW_glc)*1000*0.8; %in mmol/L
0263         GlcMax = Glc * 10 * (1/MW_glc)*1000*1.2; %in mmol/L
0264         GlcMin = cellstr(num2str(GlcMin));
0265         GlcMin = regexprep(GlcMin,' ','');
0266         for j = 1 : size(GlcMin,1)
0267             GlcMin{j,1} = (GlcMin(j,1));
0268         end
0269         GlcMax = cellstr(num2str(GlcMax));
0270         GlcMax = regexprep(GlcMax,' ','');
0271         for j = 1 : size(GlcMax,1)
0272             GlcMax{j,1} = (GlcMax(j,1));
0273         end
0274         InputDataMetabolitesBC(1,:) = ['glc_D'   GlcMin  GlcMax];
0275     end
0276     % Cholesterol
0277     MW_chsterol = 386.65;% g/mol;
0278     Chsterol = ismember(InputData(:,1),'Cholesterol');
0279     if ~isempty(find(Chsterol))
0280         Chsterol = InputData(Chsterol,3);
0281         Chsterol =str2num(char(Chsterol{1})) ;
0282         % calculate Chsterol in mM
0283         ChsterolMin = Chsterol * 10 * (1/MW_chsterol)*1000*0.8; %in mmol/L
0284         ChsterolMax = Chsterol * 10 * (1/MW_chsterol)*1000*1.2; %in mmol/L
0285         ChsterolMin = cellstr(num2str(ChsterolMin));
0286         ChsterolMin = regexprep(ChsterolMin,' ','');
0287         for j = 1 : size(ChsterolMin,1)
0288             ChsterolMin{j,1} = (ChsterolMin(j,1));
0289         end
0290         ChsterolMax = cellstr(num2str(ChsterolMax));
0291         ChsterolMax = regexprep(ChsterolMax,' ','');
0292         for j = 1 : size(ChsterolMax,1)
0293             ChsterolMax{j,1} = (ChsterolMax(j,1));
0294         end
0295         InputDataMetabolitesBC(2,:) = ['chsterol'   ChsterolMin  ChsterolMax];
0296     end
0297     modelPersonalized = physiologicalConstraintsHMDBbased(modelPersonalized,IndividualParameters, Type, InputDataMetabolitesBC, 'bc');
0298     %urea given in mg/dl in input data
0299     % Cholesterol
0300     MW_urea = 60.06;% g/mol;
0301     MW_creatinine = 113.1179;%g/mol
0302     Urea = ismember(InputData(:,1),'Urea');
0303     if ~isempty(find(Urea))
0304         Urea = InputData(Urea,3);
0305         Urea =str2num(char(Urea{1})) ;
0306         % calculate Urea in mM
0307         UreaMin = Urea * 10 * (1/MW_urea)*1000*0.8/(IndividualParameters.MConUrCreatinineMax * 10 * (1/MW_creatinine)); %in umol urea/mmol creatine
0308         UreaMax = Urea * 10 * (1/MW_urea)*1000*1.2/(IndividualParameters.MConUrCreatinineMax * 10 * (1/MW_creatinine)); %in umol urea/mmol creatine
0309         UreaMin = cellstr(num2str(UreaMin));
0310         UreaMin = regexprep(UreaMin,' ','');
0311         for j = 1 : size(UreaMin,1)
0312             UreaMin{j,1} = (UreaMin(j,1));
0313         end
0314         UreaMax = cellstr(num2str(UreaMax));
0315         UreaMax = regexprep(UreaMax,' ','');
0316         for j = 1 : size(UreaMax,1)
0317             UreaMax{j,1} = (UreaMax(j,1));
0318         end
0319         InputDataMetabolitesU(2,:) = ['urea'   UreaMin  UreaMax];
0320     end
0321     modelPersonalized = physiologicalConstraintsHMDBbased(modelPersonalized,IndividualParameters, Type, InputDataMetabolitesU, 'u');
0322 end
0323 IndividualParametersNew =IndividualParameters;
```

---

Generated on Thu 14-May-2020 13:05:49 by **m2html** © 2005
