# Supplementary material for: Personalized whole‐body models integrate metabolism, physiology, and the gut microbiome
Source: Mol Syst Biol. 2020 May 28;16(5):e8982. doi: 10.15252/msb.20198982 (PMC7285886; doi:10.15252/msb.20198982)
Supplement: Supplementary file 22 — Dataset EV1 [file MSB-16-e8982-s022.zip › PSCM_toolbox/PSCM_toolbox_doc/src/setConstraints/standardPhysiolDefaultParameters.html]

Description of standardPhysiolDefaultParameters


# standardPhysiolDefaultParameters

## PURPOSE

**This script creates the IndividualParameters structure which contains**

## SYNOPSIS

**This is a script file.**

## DESCRIPTION

```
 This script creates the IndividualParameters structure which contains
 standard physiological default parameters for the reference man or woman.

 Ines Thiele 2016-2019
```

## CROSS-REFERENCE INFORMATION

This function calls:

- getOrganWeightFraction This script reads in the organ file and assign biomass\_maintenance coefficient according

This function is called by:

- analyzeHMmodel This function performs host-microbiome optimization for a set of defined
- perform\_BMR\_newData This script repeats the simulation described in Thiele et al., "Personalized whole-body models integrate metabolism, physiology, and the gut microbiome", Method section 3.9.2 Validation of the parameters in an independent data set.
- perform\_sensi\_BMR\_all This script repeats the simulation described in Thiele et al.,
- runIEM\_HH This script predicts known biomarker metabolites in

## SOURCE CODE

```
0001 % This script creates the IndividualParameters structure which contains
0002 % standard physiological default parameters for the reference man or woman.
0003 %
0004 % Ines Thiele 2016-2019
0005 
0006 % needs sex to be defined, otherwise this script will error.
0007 
0008 % normal physiological DEFAULT parameters
0009 getOrganWeightFraction;
0010 IndividualParameters.OrgansWeights =  [OrganNames num2cell(OrganWeight) num2cell(OrganWeightFract)];
0011 % these can be personalized if available
0012 IndividualParameters.ID = 'Default';
0013 if strcmp(sex,'male')
0014     IndividualParameters.bodyWeight = BodyWeight/1000; % in kg % as defined in getOrganWeightFraction.m - ref man
0015     IndividualParameters.Height = 170; % in cm
0016     IndividualParameters.sex = 'male'; % alternative female
0017     
0018 elseif strcmp(sex,'female')
0019     IndividualParameters.bodyWeight = BodyWeight/1000; % in kg % as defined in getOrganWeightFraction.m - ref woman
0020     IndividualParameters.Height = 160; % in cm
0021     IndividualParameters.sex = 'female'; % alternative female
0022 end
0023 IndividualParameters.HeartRate = 67; % beats per minute
0024 IndividualParameters.StrokeVolume = 80; %ml/beat
0025 %IndividualParameters.StrokeVolume.unit = 'ml/beat';
0026 IndividualParameters.CardiacOutput = IndividualParameters.HeartRate * IndividualParameters.StrokeVolume; % in ml/min = beats/min * ml/beat
0027 IndividualParameters.Hematocrit = 0.4; % 'packed cell volume; normally men: 46%, women: 41%
0028 % creatinine concentration in urine
0029 IndividualParameters.MConUrCreatinineMax = 1.2; % mg/dL Adult males: 0.5�1.2 mg/dL; Adult females: 0.4 � 1.1 mg/dL; http://emedicine.medscape.com/article/2054342-overview
0030 IndividualParameters.MConUrCreatinineMin = 0.5; % mg/dL
0031 
0032 % default maximum concetration of a metabolite in blood plasma
0033 IndividualParameters.MConDefaultBc = 20; % uM abretary chosen
0034 
0035 % default maximum concetration of a metabolite in csf
0036 IndividualParameters.MConDefaultCSF = 20; % uM abretary chosen
0037 
0038 % default maximum concetration of a metabolite in Ur
0039 IndividualParameters.MConDefaultUrMax = 20; % umol/mmolcreatinine abretary chosen
0040 IndividualParameters.MConDefaultUrMin = 0; % umol/mmolcreatinine
0041 
0042 % CSF Flow rate
0043 IndividualParameters.CSFFlowRate = 0.35;%ml/min based on Sundstrom 2010, Anal Neurol
0044 
0045 % CSF to venous blood flow rate
0046 IndividualParameters.CSFBloodFlowRate = 0.52; % 0.52 ml/min based on Pardridge 2011, Fluids and Barr of CNS
0047 
0048 % Urine flow rate
0049 IndividualParameters.UrFlowRate = 2000; %ml/day,
0050 % https://www.healthline.com/health/urine-24-hour-volume
0051 
0052 % GFR = Glomerular filtration rate
0053 IndividualParameters.GlomerularFiltrationRate = 90;%ml/min, % 90 - 120 ml/min is reported for healthy range: https://www.nlm.nih.gov/medlineplus/ency/article/007305.htm
0054 
0055 % Blood Flow rate per organ
0056 fileNameOrgan = '16_01_26_BloodFlowRatesPercentages.xlsx';
0057 [Numbers, IndividualParameters.bloodFlowData] = xlsread(fileNameOrgan,'BloodFlowPercentage');
0058 % find start of data
0059 for i = 1 : size(IndividualParameters.bloodFlowData,1)
0060     if length(find(ismember(IndividualParameters.bloodFlowData(i,:),'Blood flow percentage')))>0
0061         bloodFlowRow = i+1; % next line is sex
0062         IndividualParameters.bloodFlowRow = bloodFlowRow;
0063         IndividualParameters.bloodFlowPercCol = find(ismember(IndividualParameters.bloodFlowData(i,:),'Blood flow percentage'));
0064         IndividualParameters.bloodFlowOrganCol = find(ismember(IndividualParameters.bloodFlowData(i,:),'Organ'));
0065         break;
0066     end
0067 end
```

---

Generated on Thu 14-May-2020 13:05:49 by **m2html** © 2005
