# Supplementary material for: Personalized whole‐body models integrate metabolism, physiology, and the gut microbiome
Source: Mol Syst Biol. 2020 May 28;16(5):e8982. doi: 10.15252/msb.20198982 (PMC7285886; doi:10.15252/msb.20198982)
Supplement: Supplementary file 23 — Dataset EV2 [file MSB-16-e8982-s023.htm]

Untitled 

## Method Section 3.

#### In this Matlab Live script, we provide the setup and the functions to repeat the simulations and results presented in Thiele et al., "Personalized whole-body models integrate metabolism, physiology, and the gut microbiome".

## 3.0. Prerequisite

- MATLAB, Mathworks, Inc.
- The COBRA Toolbox (0), to be obtained here: https://opencobra.github.io/cobratoolbox/stable/
- The PSCM Toolbox extension for the COBRA Toolbox is required.
- The violin plot functions by Bastian Bechtold, to be obtained here: https://github.com/bastibe/Violinplot-Matlab
- A linear and quadratic programming solver. The code has been tested using Tomlab (TomOpt, Inc.) in conjunction with ILog cplex ('tomlab\_cplex') as well as . IBM ILog cplex (academic version, 'ILOGcomplex').
- Optional: Access to a high-performance computer or computing facility to improve computation time.

Please increase your MATLAB jave heap memory to maximum (see here for details: https://uk.mathworks.com/help/matlab/matlab\_external/java-heap-memory-preferences.html).

Initiate the COBRA toolbox

initCobraToolbox;

Add path to PSCM Toolbox. Requires you to be in the toolbox.

addpath(genpath('.'));

Generate documentation of the PSCM Toolbox using the m2html tool (https://www.artefact.tk/software/matlab/m2html/).

To access the documentation, please open index.html in your preferred web browser.

if 1

currentPath = pwd;

% First, convert Matlab LiveScript into a m file:

matlab.internal.liveeditor.openAndConvert('runPaperSectionSimulationsOct2019b.mlx','runPaperSectionSimulationsOct2019b.m');

% copy the m2html tool from the PSCM Toolbox

copyfile 'addOn/m2html' '../../m2html' 'f'

% delete any pre-existing documentation of the PSCM Toolbox

cd ../;

rmdir PSCM\_toolbox\_doc s

cd ../;

% add path to the m2html tool

addpath(genpath('m2html'));

% generate new documentation in target folder 'PSCM\_toolboxPublication/PSCM\_toolbox\_doc/'

m2html('mfiles','PSCM\_toolboxPublication/PSCM\_toolbox', 'htmldir','PSCM\_toolboxPublication/PSCM\_toolbox\_doc', 'recursive','on', 'global','on','template','brain', 'index','menu');

cd currentPath;

end

Define the LP and QP solver:

if 1

LPSolver = 'tomlab\_cplex';

QPSolver = 'tomlab\_cplex';

else

LPSolver = 'ILOGcomplex';

QPSolver = 'ILOGcomplex';

end

Please note that all simulation results within this lifescript have been generated using Tomlab,

## 1. Load the whole-body metabolic models: Harvetta (female model) and Harvey (male model)

The models have been parameterized using the average European diet, the standard physiological parameters, and metabolite concentrations derived from HMDB (1) for plasma/blood, urine, and CSF. The organ metabolite uptake reactions, the exchange reactions across the blood-brain-barrier, the urine excretion reactions, and the kidney exchange reactions have been set accordingly.

load Harvey\_1\_01c

load Harvetta\_1\_01c

## 3.2. In the next step, we will repeat the parameterization of the provided WBM models.

3.2.1 We will load the standard (default) physiological parameters, which are held on the structure IndividualParameters.

For the male:

gender = 'male';

standardPhysiolDefaultParameters;

Col = 2

IndividualParameters\_male = IndividualParameters

IndividualParameters\_male = struct with fields:

OrgansWeights: {53×3 cell}
ID: 'Default'
bodyWeight: 70
Height: 170
gender: 'male'
HeartRate: 67
StrokeVolume: 80
CardiacOutput: 5360
Hematocrit: 0.4000
MConUrCreatinineMax: 1.2000
MConUrCreatinineMin: 0.5000
MConDefaultBc: 20
MConDefaultCSF: 20
MConDefaultUrMax: 20
MConDefaultUrMin: 0
CSFFlowRate: 0.3500
CSFBloodFlowRate: 0.5200
UrFlowRate: 2000
GlomerularFiltrationRate: 90
bloodFlowData: {32×8 cell}
bloodFlowRow: 2
bloodFlowPercCol: [4 6]
bloodFlowOrganCol: 1

and for the female:

gender = 'female';

standardPhysiolDefaultParameters;

IndividualParameters\_female = IndividualParameters

IndividualParameters\_female = struct with fields:

OrgansWeights: {53×3 cell}
ID: 'Default'
bodyWeight: 58
Height: 160
gender: 'female'
HeartRate: 67
StrokeVolume: 80
CardiacOutput: 5360
Hematocrit: 0.4000
MConUrCreatinineMax: 1.2000
MConUrCreatinineMin: 0.5000
MConDefaultBc: 20
MConDefaultCSF: 20
MConDefaultUrMax: 20
MConDefaultUrMin: 0
CSFFlowRate: 0.3500
CSFBloodFlowRate: 0.5200
UrFlowRate: 2000
GlomerularFiltrationRate: 90
bloodFlowData: {32×8 cell}
bloodFlowRow: 2
bloodFlowPercCol: [4 6]
bloodFlowOrganCol: 1

Note that the percentage of how much blood flow each organ received is defined for the male or female at rest. The values are given in the structure IndividualParameters and defined in the file:

Note that the organ weight percentage is defined in the file:

- Numbers\_organWeightData\_fromxls\_16\_01\_29\_OrganWeigths.txt

and can be accessed via

- getOrganWeightFraction.m

For the reference man (Harvey) and woman (Harvetta), the data have been derived as follows:

Organ weight and fractions (organs in bold are currently considered in the whole-body metabolic model as an organ or cell type):

- Total body weight: male: 70 kg; female: 58 kg.
- Adipocytes: male: 15 kg (21.43%); female: 19 kg (32.76%) (2)
- Adrenal glands: male: 14 g (0.02%); female: 14 g (0.024%) (2, 3)
- Brain: male: 1400 g (2%); female: 1200 g (2.069%) (2, 4)
- Breast: male: 26 g (0.037%); female: 360 g (0.621%) (2, 5)
- Colon: estimated based on weight and length of the small intestine, which has a weight of 600 g for 610 cm: male: 300g (0.429%); female: 290g (0.5%) (2, 6, 7)
- CSF: 100-160 ml CSF, density of CSF: 1.0063 to 1.0075: male: 120 g (0.171%); female: 100 g (0.172%) (2, 8)
- Esophagus: estimated based on weight and length of the small intestine, which has a weight of 600 g for 610 cm: male: 40 g (0.057%); female: 34 g (0.059%) (2, 6, 7)
- Heart: male: 331 g (0.473%); female: 253 g (0.436%) (2, 9)
- Kidney: 129 g (right kindey), 137 g (left kidney): male: 310 g (0.443%); female: 275 g (0.474%) (2, 4)
- Liver: male: 1800 g (2.571%); female: 1400 g (2.414%) (2, 4)
- Lung: bloodless: male: 536 g (0.766%); female: 536 g (0.924%) (2, 4)
- Muscle: 38.4 % of body weight in man, 30.6% in woman: male: 28 kg (40%); female: 17 kg (29.310%) (2, 10)
- Ovary: male: 0 g ; female: 11 g (0.019%) (2, 3)
- Pancreas: male: 100 g (0.143%); female: 85 g (0.147%) (2, 3)
- Placenta: male: 0 g; female: 528 g (0.910%) (11, 12)
- Prostate: male: 16 g (0.023%); female: 0 g (2, 3)
- Parathyroid glands: male: 30 mg each for 4 glands; female: 35 mg each for 4 glands: male: 0.12 g (0.000171%); female 0.14 g (0.000241%) (2, 13)
- Rectum: estimated based on weight and length of the small intestine, which has a weight of 600 g for 610 cm: male: 70 g (0.1 %); female: 70 g (0.121%) (2, 6, 7)
- Retina: male: 0.326 g (0.00047%), female: 0.326 g (0.00056%) (14)
- Spinal cord: male: 30 g (0.043%), female: 28 g (0.0483%) (2, 15)
- sIEC: 610 cm long: male: 640 g (0.914%); female: 600 g (1.0344%) [Ref man, 136PMID: 23492669;9400033] (2)
- Skin: male: 2600 g (3.714%); female: 1790 g (3.086%) (2)
- Spleen: male: 180 g (0.257%); female: 150 g (0.259%) (2, 4)
- Stomach: male: 150 g (0.214%); female: 140 g (0.241%) (2, 16)
- Testis: male: 35 g (0.05 %); female: 0 g (2, 3)
- Thyroid gland: male: 20 g (0.029%); female: 17 g (0.029%) (2, 3)
- Urinary bladder: male: 45 g (0.064%); female: 45 g (0.077%) (2, 17)
- Urine in bladder: 300-400 ml: male: 350 g (0.5 %); female: 350 g (0.603%) (18)
- Uterus: male: 0 g; female: 80 g (0.138%) (2, 3)
- Blood: Blood density = 1.0506 g/ml; blood weight = 1050.0663 g/L; 5 l per person assumed: male: 5500 g (7.857%); female: 4100 g (7.069%) (2, 19, 20)
- White blood cells: WBCs in age over 21 year olds: 4.5 to 11.0 (mean 7.4) (in 10^9/L); WBC make about 1% of blood: male: 55 g (0.079%); female: 41 g (0.071%) (21)
- Lymphocytes: 15-40% of WBCs, assumed 30% of WBC weight: male: 16.5 g (0.024%); female: 12.3 g (0.0212%) (22, 23)
- Bcells: 9% of lymphocytes: male: 1.485 g (0.00212%); female: 1.107 g (0.00191%) (23)
- CD4Tcells: 45-75% of lymphocytes, 4 – 20% of leukocyte, assumed 15% of leukocytes: male: 8.25 g (0.0118%); female: 6.15 g (0.0106%) (24)
- CD8Tcells: 2 – 11% of leukocytes, assumed 8%: male: 4.4 g (0.0063%); female: 3.28 g (0.0057%)
- Nkcells: Human and mouse NK cells constitute approximately 15% of all circulating lymphocytes: male: 2.475 g (0.00353%); female: 1.845 g (0.00318%) (24)
- Monocytes: Monocytes account for 2-8% of peripheral WBCs, assumed 5%: male: 2.75 g (0.00393%); female: 2.05 g (0.00353%) (21)
- Platelets: 150,000 to 400,000/mm3, 1mm3 = 1e-6 l, 10 pg per platelet (wet weight), assumed 400k/mm3 corresponding to 4g/l, hence: 20g/5l: male: 20 g (0.0286%); female: 20 g (0.0345%) (25, 26)
- Red blood cells: The normal range in men is approximately 4.7 to 6.1 million cells/ul. The normal range in women range from 4.2 to 5.4 million cells/ul, according to National Institutes of Health data. 27 pg dry weight of one RBC. Assumed 70% water in RBC, hence 90 pg/RBC or 495g/l hence 2475 g/5l for male (assumed 5.5M) and 2050 g/l for female (assumed 4.5M): male: 2475 g (3.536%); female: 2050 g (3.534%) (27, 28)
- Skeleton: (bone, bone marrow, other tissue: male: 10 kg (14.286%); female: 6800 g (11.724%) (2)
- Tendons etc: male: 850 g (1.214%); female: 700 g (1.207%) (2)
- Cervix: estimated: male: 0 g; female: 50 g (0.086%)
- Gall bladder: male: 10 0.014285714 8 0.013793103 (2)
- Gut: content of the gut: male: 1005 g (1.436%); female: 1005 g (1.733%) (2, 29)
- Salivary glands: male: 85 g (0.121%); female: 70 g (0.120%) (2)
- Ureter: male: 16 g (0.023%); female: 15 g (0.026%) (2)
- Urethra: male: 10 g (0.014%); female: 3 g (0.005%) (2)
- Teeth: male: 46 g (0.065%); female: 41 g (0.071%) (2)
- Nails: male: 3 g (0.004%); female: 3 g (0.005%) (2)
- Nose mucosa: male: 32 g (0.046%); female: 27 g (0.047%) (2)
- Hair: male: 20 g (0.029%); female: 300 g (0.517%) (2)

- Total body weight captured by the whole-body model, when excluding bones, nails, etc: male: 58.6kg (83.8%); female: 49,5 kg (85.4%).

- The blood flow percentage have been retrieved from (30) for: adipocytes, heart, kidney, liver, lung, skin, and from (31) for brain, colon, kidney, muscle, parathyroid glands, pancreas, sIEC, spleen, stomach, rectum, thyroid glands. The remaining organs were assumed to receive 0.01% of the total blood flow, as peripheral organs receive together about 1% of the blood flow (32).

3.2.2. Calculation and application of constraints:

- The conversion of a reconstruction into a model involves the definition of condition-specific constraints. To exploit the unique features of the WBM reconstruction, we constrained it with 14 physiological parameters (Fig. 3A, main text), allowing us to quantitatively integrate metabolomics data as modeling constraints. For instance, the metabolite transport across the capillaries of well-perfused organs is bulk flow limited rather than diffusion limited (20). Using blood flow rates at rest for the difference organs and plasma metabolite concentration, obtained from HMDB (1), an upper bound could be placed on metabolite uptake reactions for each organ. Additionally, 80% of the blood is pushed through the kidney along with all metabolites, irrespective of their nature. Multiplying the glomerular filtration rate (GFR) with the plasma metabolite concentration ranges allowed us to set lower and upper bounds for each metabolite uptake by the kidney. Similarly, the cerebrospinal fluid (CSF) content is unselectively flushed into the bloodstream (20), permitting us to set CSF metabolite concentration, obtained from HMDB (1), as constraints. Urine metabolite concentrations, obtained from HMDB (1), can be applied as constraints, knowing the daily urine excretion. Additionally, we included constraints of organ-specific energy requirements (e.g., brain and heart energy demands) as well as organ- and metabolite-specific uptake rates.
- We then apply these physiological constraints, together with the metabolite ranges obtained for urine, blood, and CSF from HMDB. Note that we calculated the ranges for each metabolite in each biofluid based on the data obtained from HMDB (1) for healthy individuals, combining data points for males and females. We combined reported sex-specific metabolite concentrations as the sex- specific information was not available for all metabolites. We assumed ranges to be two standard deviations from the reported mean if no ranges are provided. The used concentration ranges can be found in these files:
- Blood: NormalBloodConcExtractedHMDB.txt;
- CSF: NormalCSFConcExtractedHMDB.txt;
- Urine: NormalUrineConcExtractedHMDB.txt;
- Alternatively, metabolomic data can be also directly put into the function.
- Note that all metabolite concentrations (must be) are given in uM. Note that if no metabolite concentration is given in the HMDB data, then a default blood concentration of 20 uM was assumed for all biofluids. This default can be changed in the structure IndividualParameters.
- The following assumptions were made:
- 1. The modeled system is in a steady-state
- 2. The metabolite transport from, e.g., the arteria (blood vessel) to the organ/tissue is is bulk flow limited, not diffusion limited, which is at least true at higher blood flow rates
- 3. The metabolites can be maximally consumed by a tissue as given by the upper bound (so one tissue could deplete the entire metabolite concentration). Not considering the potential difference between arterial and venal concentration may result in a too high flux rate for a given metabolite and organ/tissue. However, as the venal concentration for most metabolites is unknown, we proceeded with the looser constraints.
- The upper bound on a reaction flux for a given metabolite and organ/tissue is thus calculated based on:
- Q = (ca-cv)\*Qv (20)
- vmax\_met\_organ = conc\_met\_max \* k\_blood\_organ, where conc\_met\_max is the maximal metabolite concentration in the blood (as given by the ranges obtained from HMDB) and k\_blood\_organ is the blood flow percentage for a given organ.
- Note that no organ-specific scaling is necessary as those numbers are already adjusted to organs (in part to weight but also requirements).
- The blood flow rates and percentages for male and female at rest are defined in this file:
- 16\_01\_26\_BloodFlowRatesPercentages.xlsx along with the corresponding references.

3.2.2.1. Glomerular Filtration Rate

- We calculate the glomerular filtration rate as follows:
- RenalFlowRate=BloodFlowPercentage\_Kidney\*CardiacOutput\*(1-Hematocrit); (in ml/min)
- GlomerularFiltrationRate = RenalFlowRate\*RenalFiltrationFraction; (in ml/min), where the RenalFiltrationFraction = 0.2;

3.2.2.2. Calculation of the blood flow and plasma flow rate for each organ

- BloodFlowRate\_Organ=BloodFlowPercentage\_Organ\*CardiacOutput; (in ml/min)
- PlasmaFlowRate\_Organ=BloodFlowPercentage\_Organ\*CardiacOutput\*(1-Hematocrit); (in ml/min)
- Note than if no information is provided for the organ, 1% was assumed
- BloodFlowRate\_Organ=0.01\*CardiacOutput; % k\_blood\_organ in ml/min
- PlasmaFlowRate\_Organ=0.01\*CardiacOutput\*(1-Hematocrit); % k\_blood\_organ in ml/min
- Flow accross the blood-brain-barrier (exchange from blood compartment to CSF), to supply the spinal cord and the brain
- B = BloodFlowPercentage\_Brain + BloodFlowPercentage\_Scord;
- BloodFlowRate\_BBB=(B)\*CardiacOutput; (in ml/min)
- PlasmaFlowRate\_BBB=(B)\*CardiacOutput\*(1-Hematocrit); (in ml/min)

3.2.2.3. Calculation of the upper bound on each metabolite exchange from the blood compartment ([bc]) to the organ/tissue

- Note that the metabolite concentration is given in umol/L, thus it needs to be multiplied by 1000 to result in mmol/L. The PlasmaFlowRate is in (ml/min), hence
- PlasmaFlowRate = PlasmaFlowRate\*60\*24/1000 to obtain it in L/day.

Kidney:

- The kidney exchange reaction is defined as
- Kidney\_EX\_metabolite(e)\_[bc]: 1 metabolite[e] <--> 1 metabolite[bc]
- Consequently, the upper bound for each kidney reaction is set with
- modelConstraint.ub = -1\*(/1000)\*GlomerularFiltrationRate\*60\*24/1000; (in mmol/day/person)
- and the lower bound:
- modelConstraint.lb = -1\*(MConMax/1000)\*GlomerularFiltrationRate\*60\*24/1000; % in mmol/day/person
- where MConMin and MConMax are the minimum and maximum concentration values, respectively, for a metabolite as given by the ranges obtained from HMDB.
- Note that in the case of the kidney the lower and upper bounds can be set (in contrast to the other organs, as discussed above) as about 80% of the blood is pushed through the kidney independent of the nature of the metabolite.
- Also, note that we only set the upper bound on these reactions, when the MConMax>=MaxConcConstraint, which is currently defined as 50 uM. This softening was introduced during the debugging process and can be changed in the function physiologicalConstraintsHMDBbased.m as needed.

Other organs supplied by the blood compartment:

- The lower bound for each organ exchange reaction is set with
- modelConstraint.lb = -1\*((MConMax)/1000)\*PlasmaFlowRate(i,1)\*60\*24/1000; (in mmol/day/person)
- while the upper bound was unconstrained for all organs that can secrete into the blood compartment or 0 for the remaining organs (i.e., 'sIEC', 'Colon','Spleen','Pancreas','Gall','Brain','Scord').
- Note that if the metabolite concentration was below 1 nM, then the value for MConMax was overwritten to be 1 nM (or 1e-3 uM). This adjustment became necessary during the debugging process.

3.2.2.4. Blood-brain-barrier metabolite exchange reaction constraints

- The constraints for these reactions were based on the blood metabolite concentration and calculated as described in 2.2.3 under "Other organs".

3.2.2.5. CSF concentrations

- No constraints could be put on the metabolite uptake reactions in the brain and the spinal cord as the flux is likely to be diffusion limited, if not active transport system exists, due to the slow flow rate of the CSF (there are about 300 ml of CSF in the brain, which gets completely exchanged about 5 times a day into the blood). However, the exchange/drainage of CSF metabolites into the blood appears to be again independent of the nature of the metabolites and thus bulk flow limitation, rather than diffusion limitation can be assumed. Consequently, we can apply the CSF metabolite concentrations to those CSF excretion reactions, defined as:
- BBB\_METABOLITE(CSF)\_[bc]exp: 1 metabolite[CSF] --> 1 metabolite[bc]
- The constraints were calculated as follows, lower bound:
- modelConstraint.lb = (MConMin/1000)\*CSFBloodFlowRate\*60\*24/1000; (in mmol/day/person)
- and upper bound:
- modelConstraint.ub = (MConMax/1000)\*CSFFlowRate\*60\*24/1000;
- where MConMin and MConMax are the minimum and maximum metabolite concentration in CSF, respectively. If no metabolite concentration value is provided, MConMin was set to 0 mM and MConMax to 20 mM, as defined in the structure IndividualParameters.

3.2.2.6. Urine concentrations

- The urine excretion reactions are defined as
- EX\_metabolite[u]: 1 metabolite[u] -->
- The constraints were calculated as follows, lower bound:
- modelConstraint.lb = (MConMin/1000)\*MConDefaultUrCreatinineMin\*UrFlowRate/1000; (in mmol/day/person)
- and upper bound
- modelConstraint.ub = (MConMax/1000)\*MConDefaultUrCreatinineMax\*UrFlowRate/1000; % in mmol/day/person
- where MConMin and MConMax are the minimum and maximum metabolite concentration in the urine, respectively. If no metabolite concentration value is provided, MConMin was set to 0 mM and MConMax to 20 mM, as defined in the structure IndividualParameters. The MConDefaultUrCreatinineMax is the creatinine concentration in the urine and UrFlowRate is the daily urine flow rate. Both are defined in the structure IndividualParameters (see above).
- Note that the lower bound was only set for those metabolites that had a MConMax of at least 50 mM. This softening of constraints became necessary during the debugging process.

3.2.2.7. Further constraints

- Oxygen uptake bounds (at rest)

- The following assumptions and calculations were done:
- - Composition air in: 78.62% nitrogen, 21% oxygen, 0.96% argon, 0.04% carbon dioxide, 0.5% water vapour
- - Composition air out: 78.04% nitrogen, 14% - 16% oxygen, 4% - 5.3% carbon dioxide, 1% argon and other gases
- - Amount of O2:
- - Tidal volume: 500 ml/breath
- - Breathing frequency 12-15x/min
- - Change of O2: 5%
- - Volume of gas: 1mol gas = 22.4 l , 1mmol=22.4ml
- - Volume O2/breath = 5\*500 (ml)/100 = 25ml
- - O2 change (mmol) = 25ml/22.4 ml = 1.1mmol
- - Volume 02/day = 1.1mmol\*12\*60\*24 = 19080 mmol/day/person
- - We assumed a respiratory quotient of 0.8 to set the CO2 outflux accordingly
- - As the oxygen uptake rate (aka, oxygen consumption at rest) depends on the calculation and differs between individuals, we choose a range to constraint the default Harvey and Harvetta models: (33-35)
- lower bound on O2 uptake = -25000;
- upper bound on O2 uptake = -15000;
- lower bound on CO2 secretion = -15000\*0.8;
- upper bound on CO2 secretion = -25000;
- Water exchange:
- values were taken from (2). For each calculated value, we assumed a 20% error. Consequently, the constraints are:
- Sweat: 650 ml (water loss)/day - 650g = 650g/day / 18.01528g/mol = 36.0805 mol/day/person
- lower bound on EX\_h2o[a] = 36080\*0.8
- upper bound on EX\_h2o[a] = 36080\*1.2
- Insensible (assumed to be breathing): 840g = 850/18.01528 = 47.1822 mol/day/person
- lower bound on EX\_h2o[sw] = 47182\*0.8
- upper bound on EX\_h2o[sw] = 47182\*1.2
- Urine: 1400g = 1400/18.01528 = 77.7118 mol/day/person
- lower bound on EX\_h2o[u] = 77711\*0.8
- upper bound on EX\_h2o[u] = 77711\*1.2
- Feces: 100g = 100/18.01528 = 5.5508 mol/day/person
- lower bound on Excretion\_EX\_h2o[fe = 5550\*0.8
- upper bound on Excretion\_EX\_h2o[fe] = 5550\*1.2
- To limit the water secretion into bile duct, we set an arbitrary value as upper bound on the corresponding reaction
- upper bound on Gall\_H2Ot[bdG] = 1000
- upper bound on Liver\_H2Ot[bdL] = 1000

Specific reactions

- Alanine secretion of the muscle is 12.5 mg alanine/min/person (65 kg) in the postabsorption state (36)

- MW of alanine is MW = 89.09; % g·mol-1
- hence, met = (met \* 60 \* 24 \*IndividualParameters.bodyWeight/65)/1000; %g per day per person (weight adjusted) and met = met \* 1000/ MW ; %mmol per day per person (weight adjusted)
- lower bound on Muscle\_EX\_ala\_L(e)\_[bc] = met\*0.8 (in mmol/day/person)
- upper bound on Muscle\_EX\_ala\_L(e)\_[bc] = met\*1.2

- Glucose uptake of RBCs is 25 mg/glc/min/person (65kg) (36)

- MW of glucose is MW = 180.16; % g·mol-1
- hence, met = (met \* 60 \* 24 \*IndividualParameters.bodyWeight/65)/1000; %g per day per person (weight adjusted) and met = met \* 1000/ MW; %mmol per day per person (weight adjusted)
- lower bound on RBC\_EX\_glc\_D(e)\_[bc] = -met\*0.8
- upper bound on RBC\_EX\_glc\_D(e)\_[bc] = -met\*1.2

- Glucose uptake of the brain is 80 mg glc/min/person (36)

- MW of glucose is MW = 180.16; % g·mol-1
- met = (met \* 60 \* 24 \*IndividualParameters.bodyWeight/65)/1000; %g per day per person (weight adjusted) and met = met \* 1000/ MW; %mmol per day per person (weight adjusted)
- lower bound on Brain\_EX\_glc\_D(e)\_[CSF] = -met\*0.8
- upper bound on Brain\_EX\_glc\_D(e)\_[CSF] = -met\*1.2

- Brain oxygen uptake flux

- brain\_weight is defnined in structure IndividualParameters
- Brain oxygen uptake is 156 umol o2/100g brain/min; (37)
- hence, brain\_o2 = (brain\_o2 \* 60 \* 24 \* brain\_weight/100)/1000; %mmol o2/person (brain)/day.
- lower bound on Brain\_EX\_o2(e)\_[CSF] = -brain\_o2\*1.2
- upper bound on Brain\_EX\_o2(e)\_[CSF] = -brain\_o2\*0.7

- Alanine uptake of the liver is 12.5 mg alanine/min/person (65 kg) in postabsorption state (36)

- MW of alanine is MW = 89.09; % g·mol-1
- hence, met = (met \* 60 \* 24 \*IndividualParameters.bodyWeight/65)/1000; %g per day per person (weight adjusted) and met = met \* 1000/ MW; %mmol per day per person (weight adjusted)
- lower bound on Liver\_EX\_ala\_L(e)\_[bc] = -met\*0.8
- upper bound on Liver\_EX\_ala\_L(e)\_[bc] = -met\*1.2

- Glucose secretion by liver is 130 mg glc/min/person (65 kg) in postabsorption state (36)

- MW of glucose is MW = 180.16; % g·mol-1
- hence, met = (met \* 60 \* 24 \*IndividualParameters.bodyWeight/65)/1000; %g per day per person (weight adjusted) and met = met \* 1000/ MW; %mmol per day per person (weight adjusted)
- lower bound on Liver\_EX\_glc\_D(e)\_[bc] = met\*1.2
- upper bound on Liver\_EX\_glc\_D(e)\_[bc] = met\*0.8

- Glycerol secretion by adipocytes is 12 mg glycerol/min/person (65 kg) in postabsorption state (36)

- MW of glycerol is MW = 92.09; % g·mol-1
- hence, met = (met \* 60 \* 24 \*IndividualParameters.bodyWeight/65)/1000; %g per day per person (weight adjusted) and met = met \* 1000/ MW; %mmol per day per person (weight adjusted)
- lower bound on Adipocytes\_EX\_glyc(e)\_[bc] = met\*0.8
- upper bound on Adipocytes\_EX\_glyc(e)\_[bc] = met\*1.2

- The brain and the liver can do CO2 fixation (38) and Co2 can cross the BBB (39)

- all organs but the liver and the brains are set not to take up CO2:
- lower bound on Organ\_EX\_co2(e)\_[bc] = 0
- lower bound on Brain\_EX\_co2(e)\_[CSF] = -10000 where -10000 is an arbitrary number
- lower bound on Liver\_EX\_co2(e)\_[bc] = -10000 where -10000 is an arbitrary number
- lower bound on Lung\_EX\_co2(e)\_[bc] = -10000 where -10000 is an arbitrary number
- lower bound on Kidney\_EX\_co2(e)\_[bc] = -10000 where -10000 is an arbitrary number (needed for blood circulation)

Brain ATP requirement

- The brain consumes about 120g glucose per day (40), corresponding to 0.66 mol glucose/day/person (MW=180.16).
- 1 mol glucose can be converted into 31 mol atp; hence 20.46 mol ATP could be theoretically produced from 0.66 mol glucose if complete oxidative phosphorylation is assumed.
- However, the reported value also includes the metabolic activity that we capture already in the whole-body metabolic model. Hence, it can be optionally set to:
- lower bound on Brain\_DM\_atp\_c\_ = 5500 (we did not apply this constrain when computing the basal metabolic flux)

Heart energy requirement - minimum based on (41)

- A minimum of 6000g of ATP per day per person is reported. The molecular weight of ATP is 507.18g/mol, hence 11830 mmol ATP/day/person is needed for the pump action of the heart. As the numbers differ between the sources, we enforced a minimum constraint of 6000 mmol ATP/day/person on the Heart\_DM\_atp\_c\_ reaction.
- lower bound on Heart\_DM\_atp\_c\_ = 6000

% apply HMDB metabolomic data based on the female individual parameters

female = physiologicalConstraintsHMDBbased(female,IndividualParameters\_female);

male = physiologicalConstraintsHMDBbased(male,IndividualParameters\_male);

3.2.2.8. Diet constraints

Dietary input defines the possible metabolism of the whole-body model and therefore define the set of attainable metabolic states of the entire body. In total, 1392 dietary components can be defined in the WBM models. However, only limited, quantitative information is currently available for the molecular composition of most food item. For instance, the Österreichische Nährwerttabelle (42) specifies, for more than 16,000 food items, 133 molecular components, 92 of which can be directly mapped onto the whole-body model. Using a newly developed diet designer (43), we defined an average European diet based on an Austrian survey of 1002 people (44) and substituted it with further 23 metabolites, which are known to occur in the diet but for which no quantitative information have been provided in this resource. The VMH lists further diets, which can be used to constrain the WBM models or individual diets can be designed, thereby enabling ultimately assessing the metabolic mechanism of individual dietary regimes on whole-body metabolism or even the prediction of personalized dietary recommendations.

The following diet has been defined. A detailed description can be found in the corresponding mfile and here: http://vmh.life. The diet has been based on food items as suggested by the individual references and a macromolecular composition typical for the corresponding diet.

EUAverageDietNew.m: The "EU diet" was designed based on information from an Austrian Survey. The survey was implemented in the year 2012 and 1002 people from different ages participated being one of the few free available and nutrient-based surveys for European countries (44). The "EU diet" can be used as a control which allows us to examine differences in metabolism due to the variability in the nutritional amounts.

Note that certain additional input constraints were set to enable the growth of gastrointestinal bacteria, as well as to provide the whole-body metabolic model with additional dietary components currently not reported on the used nutritional databases (ONT (42) and USDA (45)). Please read setDietConstraints.m for more details.

EUAverageDietNew

female = setDietConstraints(female, Diet);

male = setDietConstraints(male, Diet);

3.2.2.9. Simulation/paper specific constraints

Please refer to the corresponding functions for details on constraints. These constraints have been introduced during the debugging process to avoid, e.g., cycling between blood and bile of certain R-Group metabolites or to enable the determination of the maximal capacity flux of, e.g., serotonin in the brain.

% set some more constraints

female = setSimulationConstraints(female);

female.lb(strmatch('BBB\_KYNATE[CSF]upt',female.rxns)) = -1000000;

female.lb(strmatch('BBB\_LKYNR[CSF]upt',female.rxns)) = -1000000;

female.lb(strmatch('BBB\_TRP\_L[CSF]upt',female.rxns)) = -1000000;

female.ub(strmatch('Brain\_EX\_glc\_D(',female.rxns)) = -100; % currently -400 rendering many of the models to be infeasible in germfree state

minInf = -1000000;

maxInf = 1000000;

minConstraints = length(intersect(find(female.lb>minInf),find(female.lb)));

maxConstraints =length(intersect(find(female.ub<maxInf),find(female.ub)));

PercentageConstraintRxns\_female = (minConstraints + maxConstraints)\*100/length(female.ub)

PercentageConstraintRxns\_female = 12.5465

% male

% set some more constraints

male = setSimulationConstraints(male);

male.lb(strmatch('BBB\_KYNATE[CSF]upt',male.rxns)) = -1000000; %constrained uptake

male.lb(strmatch('BBB\_LKYNR[CSF]upt',male.rxns)) = -1000000; %constrained uptake

male.lb(strmatch('BBB\_TRP\_L[CSF]upt',male.rxns)) = -1000000; %constrained uptake

male.ub(strmatch('Brain\_EX\_glc\_D(',male.rxns)) = -100; % currently -400 rendering many of the models to be infeasible in germfree state

% count how many reactions have a non-infinity bounds

minInf = -1000000;

maxInf = 1000000;

minConstraints = length(intersect(find(male.lb>minInf),find(male.lb)));

maxConstraints =length(intersect(find(male.ub<maxInf),find(male.ub)));

PercentageConstraintRxns\_male = (minConstraints + maxConstraints)\*100/length(male.ub)

PercentageConstraintRxns\_male = 12.5015

Print the WBM model

With the following code, the WBM models can be printed. Please note that Matlab cannot print the produced table into a spreadsheet as the number of rows exceeds the allowed number of rows. However, the variable 'table' can be copied into a spreadsheet by double-clicking at the variable in the workspace, selecting its content, copying it (ctrl C), and pasting it into a spreadsheet. Please note that the copying may take a while.

% male

% remove coupling constraints from the S matrix and the mets vector

male\_wo\_coupling = male;

male\_wo\_coupling.S(strmatch('slack\_',male\_wo\_coupling.mets),:)=[];

male\_wo\_coupling.mets(strmatch('slack\_',male\_wo\_coupling.mets)) = [];

% print reaction formula into 'rxnFormula'

[rxnFormula]=printRxnFormula(male\_wo\_coupling,'printFlag',false,'directionFlag',true);

% create table

table\_male=[male\_wo\_coupling.rxns,male\_wo\_coupling.rxnNames,rxnFormula,num2cell(male\_wo\_coupling.lb),num2cell(male\_wo\_coupling.ub),male\_wo\_coupling.grRules,male\_wo\_coupling.subSystems];

% convert table into cell format for the Matlab function 'writetable'

cellTable\_male = cell2table(table\_male);

% writing the table produces an error as the number of rows exceeds what is

% permitted by Matlab

if 0

writetable(cellTable\_male,'WBM\_male','FileType','spreadsheet','WriteVariableNames',false);

end

% female

% remove coupling constraints from the S matrix and the mets vector

female\_wo\_coupling = female;

female\_wo\_coupling.S(strmatch('slack\_',female\_wo\_coupling.mets),:)=[];

female\_wo\_coupling.mets(strmatch('slack\_',female\_wo\_coupling.mets)) = [];

% print reaction formula into 'rxnFormula'

[rxnFormula]=printRxnFormula(female\_wo\_coupling,'printFlag',false,'directionFlag',true);

% create table

table\_female=[female\_wo\_coupling.rxns,female\_wo\_coupling.rxnNames,rxnFormula,num2cell(female\_wo\_coupling.lb),num2cell(female\_wo\_coupling.ub),female\_wo\_coupling.grRules,female\_wo\_coupling.subSystems];

% convert table into cell format for the Matlab function 'writetable'

cellTable\_female = cell2table(table\_female);

% writing the table produces an error as the number of rows exceeds what is

% permitted by Matlab

if 0

writetable(cellTable\_female,'WBM\_female','FileType','spreadsheet','WriteVariableNames',false);

end

## 3.3. Compute Basal Metabolic Flux of the whole-body metabolic model

The basal metabolic rate (BMR), or resting energy expenditure, defines the metabolic requirement needed to maintain the basic function of the human body. The BMR varies between individuals and over time. Women have generally a lower BMR than men, due to having more body fat. Generally, the energy use can be split as following: basal metabolism accounts for 60-75%, 15-30% are required for physical activity (depending on the level of activity), 5-10 % for food-induced thermogenesis, and 2-7% others (46).

3.3.1. Whole-body biomass reaction and biomass maintenance

Each organ in the whole-body metabolic model contains a biomass maintenance reaction, representing the energetic requirements to maintain the organ's integral function. As the molecular components, e.g., amino acids and lipids are recycled within the body, the maintenance reaction releases these macromolecular precursors as well. Organ-specific biomass maintenance reactions were taken from published reconstructions for four organs, red blood cell (47), the adipocytes (48), the small intestine (6), and the liver (49), and were formulated based on the biomass reaction present in Recon 3\* for the other organs. Note that organs known to proliferate under healthy conditions contain a biomass reaction in addition, which can be used to represent organ growth if desired.

The whole-body biomass reaction is composed of the biomass maintenance reactions of each organ, scaled by the organ weight percentage as defined in the structure IndividualParameters. As one person has to maintain its body, the lower and upper bound on the whole-body biomass reaction are set to 1, if not noted differently.

% print whole-body biomass reaction

% male

printRxnFormula(male,'Whole\_body\_objective\_rxn');

Whole\_body\_objective\_rxn 21.4286 Adipocytes\_biomass\_maintenance\_dummy\_objective + 0.02 Agland\_biomass\_maintenance\_dummy\_objective + 2 Brain\_biomass\_maintenance\_dummy\_objective + 0.428571 Colon\_biomass\_maintenance\_dummy\_objective + 0.472857 Heart\_biomass\_maintenance\_dummy\_objective + 0.442857 Kidney\_biomass\_maintenance\_dummy\_objective + 2.57143 Liver\_biomass\_maintenance\_dummy\_objective + 0.765714 Lung\_biomass\_maintenance\_dummy\_objective + 40 Muscle\_biomass\_maintenance\_dummy\_objective + 0.142857 Pancreas\_biomass\_maintenance\_dummy\_objective + 0.0228571 Prostate\_biomass\_maintenance\_dummy\_objective + 0.000171429 Pthyroidgland\_biomass\_maintenance\_dummy\_objective + 0.000465714 Retina\_biomass\_maintenance\_dummy\_objective + 0.0428571 Scord\_biomass\_maintenance\_dummy\_objective + 0.914286 sIEC\_biomass\_reactionIEC01b\_dummy\_objective + 3.71429 Skin\_biomass\_maintenance\_dummy\_objective + 0.257143 Spleen\_biomass\_maintenance\_dummy\_objective + 0.214286 Stomach\_biomass\_maintenance\_dummy\_objective + 0.05 Testis\_biomass\_maintenance\_dummy\_objective + 0.0285714 Thyroidgland\_biomass\_maintenance\_dummy\_objective + 0.0642857 Urinarybladder\_biomass\_maintenance\_dummy\_objective + 0.00212143 Bcells\_biomass\_maintenance\_dummy\_objective + 0.0117857 CD4Tcells\_biomass\_maintenance\_dummy\_objective + 0.00353571 Nkcells\_biomass\_maintenance\_dummy\_objective + 0.00392857 Monocyte\_biomass\_maintenance\_dummy\_objective + 0.0285714 Platelet\_biomass\_maintenance\_noTrTr\_dummy\_objective + 3.53571 RBC\_biomass\_maintenance\_noTrTr\_dummy\_objective + 0.0142857 Gall\_biomass\_maintenance\_dummy\_objective ->

% female

printRxnFormula(female,'Whole\_body\_objective\_rxn');

Whole\_body\_objective\_rxn 32.7586 Adipocytes\_biomass\_maintenance\_dummy\_objective + 0.0241379 Agland\_biomass\_maintenance\_dummy\_objective + 2.06897 Brain\_biomass\_maintenance\_dummy\_objective + 0.62069 Breast\_biomass\_maintenance\_dummy\_objective + 0.5 Colon\_biomass\_maintenance\_dummy\_objective + 0.436207 Heart\_biomass\_maintenance\_dummy\_objective + 0.474138 Kidney\_biomass\_maintenance\_dummy\_objective + 2.41379 Liver\_biomass\_maintenance\_dummy\_objective + 0.924138 Lung\_biomass\_maintenance\_dummy\_objective + 29.3103 Muscle\_biomass\_maintenance\_dummy\_objective + 0.0189655 Ovary\_biomass\_maintenance\_dummy\_objective + 0.146552 Pancreas\_biomass\_maintenance\_dummy\_objective + 0.000241379 Pthyroidgland\_biomass\_maintenance\_dummy\_objective + 0.000562069 Retina\_biomass\_maintenance\_dummy\_objective + 0.0482759 Scord\_biomass\_maintenance\_dummy\_objective + 1.03448 sIEC\_biomass\_reactionIEC01b\_dummy\_objective + 3.08621 Skin\_biomass\_maintenance\_dummy\_objective + 0.258621 Spleen\_biomass\_maintenance\_dummy\_objective + 0.241379 Stomach\_biomass\_maintenance\_dummy\_objective + 0.0293103 Thyroidgland\_biomass\_maintenance\_dummy\_objective + 0.0775862 Urinarybladder\_biomass\_maintenance\_dummy\_objective + 0.137931 Uterus\_biomass\_maintenance\_dummy\_objective + 0.00190862 Bcells\_biomass\_maintenance\_dummy\_objective + 0.0106034 CD4Tcells\_biomass\_maintenance\_dummy\_objective + 0.00318103 Nkcells\_biomass\_maintenance\_dummy\_objective + 0.00353448 Monocyte\_biomass\_maintenance\_dummy\_objective + 0.0344828 Platelet\_biomass\_maintenance\_noTrTr\_dummy\_objective + 3.53448 RBC\_biomass\_maintenance\_noTrTr\_dummy\_objective + 0.0862069 Cervix\_biomass\_maintenance\_dummy\_objective + 0.0137931 Gall\_biomass\_maintenance\_dummy\_objective ->

if 0

% compute the minimal and maximal possible flux through the whole-body biomass reaction

% reset the bound on this reaction

male = changeRxnBounds(male,'Whole\_body\_objective\_rxn',0,'l');

male = changeRxnBounds(male,'Whole\_body\_objective\_rxn',100,'u');

female = changeRxnBounds(female,'Whole\_body\_objective\_rxn',0,'l');

female = changeRxnBounds(female,'Whole\_body\_objective\_rxn',100,'u');

% define the objective function

male = changeObjective(male,'Whole\_body\_objective\_rxn');

female = changeObjective(female,'Whole\_body\_objective\_rxn');

% maximization

male.osense = -1;

tic; [solution\_male\_BMF\_Min]=solveCobraLPCPLEX(male,1,0,0,[],0,...

LPSolver); toc;

% minimization

male.osense = 1;

[solution\_female\_BMF\_Min]=solveCobraLPCPLEX(female,1,0,0,[],0,...

LPSolver)

% maximization

male.osense = -1;

[solution\_male\_BMF\_Max]=solveCobraLPCPLEX(male,1,0,0,[],0,...

LPSolver)

% maximization

female.osense = -1;

[solution\_female\_BMF\_Max]=solveCobraLPCPLEX(female,1,0,0,[],0,...

LPSolver)

end

% set lower and upper bound to 1

male = changeRxnBounds(male,'Whole\_body\_objective\_rxn',1,'b');

female = changeRxnBounds(female,'Whole\_body\_objective\_rxn',1,'b');

% clear workspace

clear Numbers i blood\* Body\* IndividualParameters file\* gender Organ\* Object\*

3.3.2. Compute a unique flux distribution through the whole-body metabolic model.

The linear programming problem posed by most metabolic models is underdetermined, meaning that there are more variables (i.e., reactions) then equations (i.e., mass balances). Consequently, alternative optimal solutions exist to this linear programming problem to be solved:

min/max z=c'.v

subject to

S.v = b

v\_lb <= v <= v\_ub

where z is the linear objective function, c a zero vector of length n with at least one non-zero entry at the index of the reaction(s) to be optimized for, and v is the flux vector. S represents the stoichiometric matrix with m rows (number of mass balances) and n number of reactions. v\_lb and v\_ub are the lower and upper bounds set on the model's reactions, respectively.

To obtain a unique flux distribution through the whole-body metabolic model, we minimize the Euclidian distance in addition:

min z = |v|

% set constraints specific for simulating BMR determination

female.lb(strmatch('Brain\_DM\_atp',female.rxns)) = 3500;

female.lb(strmatch('Heart\_DM\_atp',female.rxns)) = 1000;

male.lb(strmatch('Brain\_DM\_atp',male.rxns)) = 3500;

male.lb(strmatch('Heart\_DM\_atp',male.rxns)) = 1000;

male = changeObjective(male,'Whole\_body\_objective\_rxn');

male.osense = -1;

[solution\_male] = computeMin2Norm\_HH(male, QPSolver);

Solution is available, but not proved optimal, due to numeric difficulties, Objective -0
This objective corresponds to a flux with minimum Euclidean norm.
The weighting for minimising the norm was 1.000000e-06.
Check that the objective is the same without minimising the norm.
CPLEX Callable Library Release 12.3.0.0
Multiple threads enabled, disabling callbacks and screen output
Optimal solution found, Objective -0
This objective corresponds to a flux with minimum Euclidean norm.
The weighting for minimising the norm was 1.000000e-06.
Check that the objective is the same without minimising the norm.

female = changeObjective(female,'Whole\_body\_objective\_rxn');

female.osense = -1;

[solution\_female] = computeMin2Norm\_HH(female,QPSolver);

Solution is available, but not proved optimal, due to numeric difficulties, Objective -0
This objective corresponds to a flux with minimum Euclidean norm.
The weighting for minimising the norm was 1.000000e-06.
Check that the objective is the same without minimising the norm.
CPLEX Callable Library Release 12.3.0.0
Multiple threads enabled, disabling callbacks and screen output
Optimal solution found, Objective -0
This objective corresponds to a flux with minimum Euclidean norm.
The weighting for minimising the norm was 1.000000e-06.
Check that the objective is the same without minimising the norm.

3.3.2.1. Basal metabolic flux (BMF)

This flux distribution for each whole-body metabolic model can then be used to determine its overall energy consumption, representing its basal metabolic flux (BMF) in order to approximate the BMR. Note that we assumed that under physiological conditions, the hydrolysis of 1 ATP yields 64 kJ (52).

% Calculate BMF for the male model

S = male.A;

F = max(-S,0);

R = max(S,0);

vf = max(solution\_male.full,0);

vr = max(-solution\_male.full,0);

production=[R, F]\*[vf ;vr];

consumption=[F, R]\*[vf; vr];

% find all reactions in the model that involve atp

atp = (find(~cellfun(@isempty,strfind(male.mets,'\_atp['))));

% sum of atp consumption in the flux distribution

Sum\_atp\_male=sum(consumption(atp,1)); % (in mmol ATP/day/person)

% compute the energy release in kJ

Energy\_kJ\_male = Sum\_atp\_male/1000 \* 64; % (in kJ/day/person)

% compute the energy release in kcal, where 1 kJ = 0.239006 kcal

Energy\_kcal\_male = Energy\_kJ\_male\*0.239006 % (in kcal/day/person)

Energy\_kcal\_male = 1.4558e+03

% Calculate BMF for the female model

S = female.A;

F = max(-S,0);

R = max(S,0);

vf = max(solution\_female.full,0);

vr = max(-solution\_female.full,0);

production=[R F]\*[vf; vr];

consumption=[F R]\*[vf; vr];

% find all reactions in the model that involve atp

atp = (find(~cellfun(@isempty,strfind(female.mets,'\_atp['))));

% sum of atp consumption in the flux distribution

Sum\_atp\_female=sum(consumption(atp,1)); % (in mmol ATP/day/person)

% compute the energy release in kJ

Energy\_kJ\_female = Sum\_atp\_female/1000 \* 64; % (in kJ/day/person)

% compute the energy release in kcal, where 1 kJ = 0.239006 kcal

Energy\_kcal\_female = Energy\_kJ\_female\*0.239006 % (in kcal/day/person)

Energy\_kcal\_female = 1.3449e+03

The computed BMF values for the female and male compare well with those obtained using phenomenological models, such as the Mifflin - St Jeor equations (53):

Male: BMR = 10 \* weight (in kg) + 6.25\*height (in cm) - 5\*age (in years) + 5;

Female: BMR = 10\*weight (in kg) + 6.25\*height (in cm) - 5\*age (in years) - 161

As there is no age reported for the reference man and woman, which the physiological parameters were based on, we compute the BMR at age 20 and at age 40:

% male

BMR\_male\_20 = calculateBMR('male', 70, 170, 20);

BMR\_male\_40 = calculateBMR('male', 70, 170, 40);

{'Based on Harvey' 'Mifflin-St Jeor at age 20' 'Mifflin-St Jeor at age 40';num2str(Energy\_kcal\_male) num2str(BMR\_male\_20(3,:)) num2str(BMR\_male\_40(3,:))}

ans =

'Based on Harvey' 'Mifflin-St Jeor at age 20' 'Mifflin-St Jeor at age 40'
'1455.8238' '1667.5' '1567.5'

% female

BMR\_female\_20 = calculateBMR('female', 58, 160, 20);

BMR\_female\_40 = calculateBMR('female', 58, 160, 40);

{'Based on Harvetta' 'Mifflin-St Jeor at age 20' 'Mifflin-St Jeor at age 40'; num2str(Energy\_kcal\_female) num2str( BMR\_female\_20(3,:)) num2str( BMR\_female\_40(3,:))}

ans =

'Based on Harvetta' 'Mifflin-St Jeor at age 20' 'Mifflin-St Jeor at age 40'
'1344.862' '1319' '1219'

% calculate the atp consumed by organ biomass reactions

% find all biomass reactions

BM = (find(~cellfun(@isempty,strfind(male.rxns,'\_biomass\_'))));

% find all reactions in the model that involve atp

atp = (find(~cellfun(@isempty,strfind(male.mets,'\_atp['))));

BMatpSum = 0;

for i = 1: length(BM)

% grab atp

BMmets = (find(male.S(:,BM(i))));

BMatp(i,1) = intersect(BMmets,atp);

BMatpCoeff(i,1) = male.S(BMatp(i,1),BM(i));

BMatpSum = BMatpSum + BMatpCoeff(i,1)\*solution\_male.full(BM(i));

end

% sum of atp consumption in the flux distribution

Sum\_BM\_atp\_male=abs(BMatpSum); % (in mmol ATP/day/person)

% compute the energy release in kJ

Energy\_BM\_atp\_kJ\_male = Sum\_BM\_atp\_male/1000 \* 64; % (in kJ/day/person)

% compute the energy release in kcal, where 1 kJ = 0.239006 kcal

Energy\_BM\_atp\_kcal\_male = Energy\_BM\_atp\_kJ\_male\*0.239006 % (in kcal/day/person)

Energy\_BM\_atp\_kcal\_male = 26.9637

% female

BM = (find(~cellfun(@isempty,strfind(female.rxns,'\_biomass\_'))));

% find all reactions in the model that involve atp

atp = (find(~cellfun(@isempty,strfind(female.mets,'\_atp['))));

BMatpSum = 0;

for i = 1: length(BM)

% grab atp

BMmets = (find(female.S(:,BM(i))));

BMatp(i,1) = intersect(BMmets,atp);

BMatpCoeff(i,1) = female.S(BMatp(i,1),BM(i));

BMatpSum = BMatpSum + BMatpCoeff(i,1)\*solution\_female.full(BM(i));

end

% sum of atp consumption in the flux distribution

Sum\_BM\_atp\_female=abs(BMatpSum); % (in mmol ATP/day/person)

% compute the energy release in kJ

Energy\_BM\_atp\_kJ\_female = Sum\_BM\_atp\_female/1000 \* 64; % (in kJ/day/person)

% compute the energy release in kcal, where 1 kJ = 0.239006 kcal

Energy\_BM\_atp\_kcal\_female = Energy\_BM\_atp\_kJ\_female\*0.239006 % (in kcal/day/person)

Energy\_BM\_atp\_kcal\_female = 21.5525

% clear workspace

clear F R vf vr S prod\* consu\* ans atp

3.3.2.2. Flux through the Cori and Cahill-cycles

The computed unique flux distribution can be used to verify that both the Cori (glucose-lactate) and the Cahill (glucose-alanine) cycles are active in the whole-body metabolic models (and in this flux distribution).

% female model

% Cori cycle flux

CoriCycleRxns = {

% Liver:

'Liver\_GALU';'Liver\_HMR\_0761';'Liver\_GBA';...

'Liver\_EX\_glc\_D(e)\_[bc]'; 'Liver\_PGMT'; 'Liver\_PGI'; ...

'Liver\_LDH\_L'; 'Liver\_EX\_lac\_L(e)\_[bc]';...

%Muscle:

'Muscle\_EX\_glc\_D(e)\_[bc]'; 'Muscle\_HEX1'; 'Muscle\_PYK'; 'Muscle\_LDH\_L';'Muscle\_EX\_lac\_L(e)\_[bc]' };

for i = 1 : length(CoriCycleRxns)

CoriCycleRxns(i,2) = printRxnFormula(female,female.rxns(strmatch(CoriCycleRxns{i,1},...

female.rxns,'exact')),0);

CoriCycleRxns{i,3} = num2str(solution\_female.full(strmatch(CoriCycleRxns{i,1},...

female.rxns,'exact')));

end

CoriCycleRxns

CoriCycleRxns =

'Liver\_GALU' 'Liver\_h[c] + Liver\_g1p[c] + Liver\_utp[c] <=> Liver\_ppi[c] + Liver\_udpg[c] + slack\_Liver\_GALU + slack\_Liver\_GALU\_R ' '103.2117'
'Liver\_HMR\_0761' 'Liver\_crm\_hs[c] + Liver\_udpg[c] -> Liver\_h[c] + Liver\_udp[c] + Liver\_gluside\_hs[c] + slack\_Liver\_HMR\_0761 ' '82.0247'
'Liver\_GBA' 'Liver\_h2o[c] + Liver\_gluside\_hs[c] -> Liver\_glc\_D[c] + Liver\_crm\_hs[c] + slack\_Liver\_GBA ' '88.0535'
'Liver\_EX\_glc\_D(e)\_[bc]' 'Liver\_glc\_D[e] -> glc\_D[bc] + slack\_Liver\_EX\_glc\_D(e)\_[bc] + slack\_Liver\_EX\_glc\_D(e)\_[bc]\_R ' '967.6931'
'Liver\_PGMT' 'Liver\_g1p[c] <=> Liver\_g6p[c] + slack\_Liver\_PGMT + slack\_Liver\_PGMT\_R ' '40.2787'
'Liver\_PGI' 'Liver\_g6p[c] <=> Liver\_f6p[c] + slack\_Liver\_PGI + slack\_Liver\_PGI\_R ' '-755.2442'
'Liver\_LDH\_L' 'Liver\_nad[c] + Liver\_lac\_L[c] <=> Liver\_h[c] + Liver\_pyr[c] + Liver\_nadh[c] + slack\_Liver\_LDH\_L + slack\_Liver\_LDH\_L\_R ' '855.0597'
'Liver\_EX\_lac\_L(e)\_[bc]' 'Liver\_lac\_L[e] <=> lac\_L[bc] + slack\_Liver\_EX\_lac\_L(e)\_[bc] + slack\_Liver\_EX\_lac\_L(e)\_[bc]\_R ' '-1144.3428'
'Muscle\_EX\_glc\_D(e)\_[bc]' 'Muscle\_glc\_D[e] <=> glc\_D[bc] + slack\_Muscle\_EX\_glc\_D(e)\_[bc] + slack\_Muscle\_EX\_glc\_D(e)\_[bc]\_R ' '-10'
'Muscle\_HEX1' 'Muscle\_atp[c] + Muscle\_glc\_D[c] -> Muscle\_h[c] + Muscle\_adp[c] + Muscle\_g6p[c] + slack\_Muscle\_HEX1 ' '49.7872'
'Muscle\_PYK' 'Muscle\_h[c] + Muscle\_adp[c] + Muscle\_pep[c] -> Muscle\_atp[c] + Muscle\_pyr[c] + slack\_Muscle\_PYK ' '14.0791'
'Muscle\_LDH\_L' 'Muscle\_nad[c] + Muscle\_lac\_L[c] <=> Muscle\_h[c] + Muscle\_pyr[c] + Muscle\_nadh[c] + slack\_Muscle\_LDH\_L + slack\_Muscle\_LDH\_L\_R ' '-321.3929'
'Muscle\_EX\_lac\_L(e)\_[bc]' 'Muscle\_lac\_L[e] <=> lac\_L[bc] + slack\_Muscle\_EX\_lac\_L(e)\_[bc] + slack\_Muscle\_EX\_lac\_L(e)\_[bc]\_R ' '531.6516'

CahillCycleRxns = {

% Liver:

'Liver\_EX\_glc\_D(e)\_[bc]'; 'Liver\_PGMT'; 'Liver\_PGI'; 'Liver\_ALATA\_L';...

'Liver\_EX\_ala\_L(e)\_[bc]';...

'Liver\_GALU';'Liver\_HMR\_0761';'Liver\_GBA';...

% Muscle

'Muscle\_EX\_glc\_D(e)\_[bc]'; 'Muscle\_HEX1'; 'Muscle\_PYK'; 'Muscle\_ALATA\_L'; ...

'Muscle\_EX\_ala\_L(e)\_[bc]';

};

for i = 1 : length(CahillCycleRxns)

CahillCycleRxns(i,2) = printRxnFormula(female,female.rxns(strmatch(CahillCycleRxns{i,1},...

female.rxns,'exact')),0);

CahillCycleRxns{i,3} = num2str(solution\_female.full(strmatch(CahillCycleRxns{i,1},...

female.rxns,'exact')));

end

CahillCycleRxns

CahillCycleRxns =

'Liver\_EX\_glc\_D(e)\_[bc]' 'Liver\_glc\_D[e] -> glc\_D[bc] + slack\_Liver\_EX\_glc\_D(e)\_[bc] + slack\_Liver\_EX\_glc\_D(e)\_[bc]\_R ' '967.6931'
'Liver\_PGMT' 'Liver\_g1p[c] <=> Liver\_g6p[c] + slack\_Liver\_PGMT + slack\_Liver\_PGMT\_R ' '40.2787'
'Liver\_PGI' 'Liver\_g6p[c] <=> Liver\_f6p[c] + slack\_Liver\_PGI + slack\_Liver\_PGI\_R ' '-755.2442'
'Liver\_ALATA\_L' 'Liver\_akg[c] + Liver\_ala\_L[c] <=> Liver\_pyr[c] + Liver\_glu\_L[c] + slack\_Liver\_ALATA\_L + slack\_Liver\_ALATA\_L\_R ' '112.4347'
'Liver\_EX\_ala\_L(e)\_[bc]' 'Liver\_ala\_L[e] <=> ala\_L[bc] + slack\_Liver\_EX\_ala\_L(e)\_[bc] + slack\_Liver\_EX\_ala\_L(e)\_[bc]\_R ' '-144.2275'
'Liver\_GALU' 'Liver\_h[c] + Liver\_g1p[c] + Liver\_utp[c] <=> Liver\_ppi[c] + Liver\_udpg[c] + slack\_Liver\_GALU + slack\_Liver\_GALU\_R ' '103.2117'
'Liver\_HMR\_0761' 'Liver\_crm\_hs[c] + Liver\_udpg[c] -> Liver\_h[c] + Liver\_udp[c] + Liver\_gluside\_hs[c] + slack\_Liver\_HMR\_0761 ' '82.0247'
'Liver\_GBA' 'Liver\_h2o[c] + Liver\_gluside\_hs[c] -> Liver\_glc\_D[c] + Liver\_crm\_hs[c] + slack\_Liver\_GBA ' '88.0535'
'Muscle\_EX\_glc\_D(e)\_[bc]' 'Muscle\_glc\_D[e] <=> glc\_D[bc] + slack\_Muscle\_EX\_glc\_D(e)\_[bc] + slack\_Muscle\_EX\_glc\_D(e)\_[bc]\_R ' '-10'
'Muscle\_HEX1' 'Muscle\_atp[c] + Muscle\_glc\_D[c] -> Muscle\_h[c] + Muscle\_adp[c] + Muscle\_g6p[c] + slack\_Muscle\_HEX1 ' '49.7872'
'Muscle\_PYK' 'Muscle\_h[c] + Muscle\_adp[c] + Muscle\_pep[c] -> Muscle\_atp[c] + Muscle\_pyr[c] + slack\_Muscle\_PYK ' '14.0791'
'Muscle\_ALATA\_L' 'Muscle\_akg[c] + Muscle\_ala\_L[c] <=> Muscle\_pyr[c] + Muscle\_glu\_L[c] + slack\_Muscle\_ALATA\_L + slack\_Muscle\_ALATA\_L\_R ' '-130.3344'
'Muscle\_EX\_ala\_L(e)\_[bc]' 'Muscle\_ala\_L[e] -> ala\_L[bc] + slack\_Muscle\_EX\_ala\_L(e)\_[bc] + slack\_Muscle\_EX\_ala\_L(e)\_[bc]\_R ' '144.2275'

The physiogical constraints applied to the WBM models enforce muscle glucose uptake and muscle alanine secretion, as well as liver glucose secretion and liver alanine uptake. No constraints have been placed to enforce lactate exchange between liver and muscle. Here, we show that the liver glucose secretion constraint is not needed for having active Cori- and Cahill cycles.

if 0

maleMod = male;

Bounds = [male.lb(find(ismember(male.rxns,CoriCycleRxns))) male.ub(find(ismember(male.rxns,CoriCycleRxns)))];

%maleMod = changeRxnBounds(maleMod,'Muscle\_EX\_glc\_D(e)\_[bc]',0,'u'); % uptake enforcement needed for Cori & Cahill cycles

%maleMod = changeRxnBounds(maleMod,'Muscle\_EX\_ala\_L(e)\_[bc]',0,'l'); % secretion enforcement needed for Cahill cycle

maleMod = changeRxnBounds(maleMod,'Liver\_EX\_glc\_D(e)\_[bc]',0,'l'); % secretion enforcement NOT needed for Cori & Cahill cycles

%maleMod = changeRxnBounds(maleMod,'Liver\_EX\_ala\_L(e)\_[bc]',0,'u'); % uptake enforcement needed for Cahill cycle

maleMod = changeObjective(maleMod,'Whole\_body\_objective\_rxn');

maleMod.osense = -1;

[solution\_maleMod] = computeMin2Norm\_HH(maleMod,QPSolver);

for i = 1 : length(CoriCycleRxns)

CoriCycleRxns(i,2) = printRxnFormula(male,male.rxns(strmatch(CoriCycleRxns{i,1},...

male.rxns,'exact')),0);

CoriCycleRxns{i,3} = num2str(solution\_maleMod.full(strmatch(CoriCycleRxns{i,1},...

male.rxns,'exact')));

end

CoriCycleRxns

for i = 1 : length(CahillCycleRxns)

CahillCycleRxns(i,2) = printRxnFormula(male,male.rxns(strmatch(CahillCycleRxns{i,1},...

male.rxns,'exact')),0);

CahillCycleRxns{i,3} = num2str(solution\_maleMod.full(strmatch(CahillCycleRxns{i,1},...

male.rxns,'exact')));

end

CahillCycleRxns

femaleMod = female;

%femaleMod = changeRxnBounds(femaleMod,'Muscle\_EX\_glc\_D(e)\_[bc]',0,'u'); % uptake enforcement needed for Cori & Cahill cycles

%femaleMod = changeRxnBounds(femaleMod,'Muscle\_EX\_ala\_L(e)\_[bc]',0,'l'); % secretion enforcement needed for Cahill cycle

femaleMod = changeRxnBounds(femaleMod,'Liver\_EX\_glc\_D(e)\_[bc]',0,'l'); % secretion enforcement NOT needed for Cori & Cahill cycles

%femaleMod = changeRxnBounds(femaleMod,'Liver\_EX\_ala\_L(e)\_[bc]',0,'u'); % uptake enforcement needed for Cahill cycle

femaleMod = changeObjective(femaleMod,'Whole\_body\_objective\_rxn');

femaleMod.osense = -1;

[solution\_femaleMod] = computeMin2Norm\_HH(femaleMod,QPSolver);

for i = 1 : length(CoriCycleRxns)

CoriCycleRxns(i,2) = printRxnFormula(female,female.rxns(strmatch(CoriCycleRxns{i,1},...

female.rxns,'exact')),0);

CoriCycleRxns{i,3} = num2str(solution\_femaleMod.full(strmatch(CoriCycleRxns{i,1},...

female.rxns,'exact')));

end

CoriCycleRxns

for i = 1 : length(CahillCycleRxns)

CahillCycleRxns(i,2) = printRxnFormula(female,female.rxns(strmatch(CahillCycleRxns{i,1},...

female.rxns,'exact')),0);

CahillCycleRxns{i,3} = num2str(solution\_femaleMod.full(strmatch(CahillCycleRxns{i,1},...

female.rxns,'exact')));

end

CahillCycleRxns

end

3.3.2.3. Brain energy consumption

The human brain consumes the equivalent of 120 g of glucose consumption per day of the brain (40), which corresponds to 20.65 mole ATP/ person/day, when a conversion of 1 mole of glucose is converted into 31 mole ATP is assumed. Using the unique flux distribution computed in 3.2., we can determine the overall brain ATP consumption rate.

% set Brain non-metabolic energy requirement to 0

male.lb(strmatch('Brain\_DM\_atp',male.rxns)) = 0;

male = changeObjective(male,'Whole\_body\_objective\_rxn');

male.osense = -1;

[solution\_male] = computeMin2Norm\_HH(male,QPSolver);

Solution is available, but not proved optimal, due to numeric difficulties, Objective -0
This objective corresponds to a flux with minimum Euclidean norm.
The weighting for minimising the norm was 1.000000e-06.
Check that the objective is the same without minimising the norm.
CPLEX Callable Library Release 12.3.0.0
Multiple threads enabled, disabling callbacks and screen output
Optimal solution found, Objective -0
This objective corresponds to a flux with minimum Euclidean norm.
The weighting for minimising the norm was 1.000000e-06.
Check that the objective is the same without minimising the norm.

% Calculate Brain energy consumption for the male model

S = male.A;

F = max(-S,0);

R = max(S,0);

vf = max(solution\_male.full,0);

vr = max(-solution\_male.full,0);

production=[R,F]\*[vf; vr];

consumption=[F,R]\*[vf; vr];

% find all reactions in the model that involve atp

atp = (find(~cellfun(@isempty,strfind(male.mets,'Brain\_atp['))));

% sum of atp consumption in the flux distribution

Sum\_brain\_atp\_male=sum(consumption(atp,1)) % (in mmol ATP/day/person)

Sum\_brain\_atp\_male = 1.1621e+04

female.lb(strmatch('Brain\_DM\_atp',female.rxns)) = 0;

female = changeObjective(female,'Whole\_body\_objective\_rxn');

female.osense = -1;

[solution\_female] = computeMin2Norm\_HH(female,QPSolver);

Solution is available, but not proved optimal, due to numeric difficulties, Objective -0
This objective corresponds to a flux with minimum Euclidean norm.
The weighting for minimising the norm was 1.000000e-06.
Check that the objective is the same without minimising the norm.
CPLEX Callable Library Release 12.3.0.0
Multiple threads enabled, disabling callbacks and screen output
Optimal solution found, Objective -0
This objective corresponds to a flux with minimum Euclidean norm.
The weighting for minimising the norm was 1.000000e-06.
Check that the objective is the same without minimising the norm.

% Calculate Brain energy consumption for the female model

S = female.A;

F = max(-S,0);

R = max(S,0);

vf = max(solution\_female.full,0);

vr = max(-solution\_female.full,0);

production=[R,F]\*[vf; vr];

consumption=[F,R]\*[vf; vr];

% find all reactions in the model that involve atp

atp = (find(~cellfun(@isempty,strfind(female.mets,'Brain\_atp['))));

% sum of atp consumption in the flux distribution

Sum\_brain\_atp\_female=sum(consumption(atp,1)) % (in mmol ATP/day/person)

Sum\_brain\_atp\_female = 1.0551e+04

female.lb(strmatch('Brain\_DM\_atp',female.rxns)) = 3500;

female.lb(strmatch('Heart\_DM\_atp',female.rxns)) = 1000;

male.lb(strmatch('Brain\_DM\_atp',male.rxns)) = 3500;

male.lb(strmatch('Heart\_DM\_atp',male.rxns)) = 1000;

male = changeObjective(male,'Whole\_body\_objective\_rxn');

male.osense = -1;

[solution\_male] = computeMin2Norm\_HH(male,QPSolver);

Solution is available, but not proved optimal, due to numeric difficulties, Objective -0
This objective corresponds to a flux with minimum Euclidean norm.
The weighting for minimising the norm was 1.000000e-06.
Check that the objective is the same without minimising the norm.
CPLEX Callable Library Release 12.3.0.0
Multiple threads enabled, disabling callbacks and screen output
Optimal solution found, Objective -0
This objective corresponds to a flux with minimum Euclidean norm.
The weighting for minimising the norm was 1.000000e-06.
Check that the objective is the same without minimising the norm.

% Calculate Brain energy consumption for the male model

S = male.A;

F = max(-S,0);

R = max(S,0);

vf = max(solution\_male.full,0);

vr = max(-solution\_male.full,0);

production=[R,F]\*[vf; vr];

consumption=[F,R]\*[vf; vr];

% find all reactions in the model that involve atp

atp = (find(~cellfun(@isempty,strfind(male.mets,'Brain\_atp['))));

% sum of atp consumption in the flux distribution

Sum\_brain\_atp\_male=sum(consumption(atp,1)) % (in mmol ATP/day/person)

Sum\_brain\_atp\_male = 2.0437e+04

female = changeObjective(female,'Whole\_body\_objective\_rxn');

female.osense = -1;

[solution\_female] = computeMin2Norm\_HH(female,QPSolver);

Solution is available, but not proved optimal, due to numeric difficulties, Objective -0
This objective corresponds to a flux with minimum Euclidean norm.
The weighting for minimising the norm was 1.000000e-06.
Check that the objective is the same without minimising the norm.
CPLEX Callable Library Release 12.3.0.0
Multiple threads enabled, disabling callbacks and screen output
Optimal solution found, Objective -0
This objective corresponds to a flux with minimum Euclidean norm.
The weighting for minimising the norm was 1.000000e-06.
Check that the objective is the same without minimising the norm.

% Calculate Brain energy consumption for the female model

S = female.A;

F = max(-S,0);

R = max(S,0);

vf = max(solution\_female.full,0);

vr = max(-solution\_female.full,0);

production=[R,F]\*[vf; vr];

consumption=[F,R]\*[vf; vr];

% find all reactions in the model that involve atp

atp = (find(~cellfun(@isempty,strfind(female.mets,'Brain\_atp['))));

% sum of atp consumption in the flux distribution

Sum\_brain\_atp\_female=sum(consumption(atp,1)) % (in mmol ATP/day/person)

Sum\_brain\_atp\_female = 2.0006e+04

3.4. Compute fat storage and muscle energy expenditure under different dietary constraints

The human brain consumes the equivalent of 120 g of glucose consumption per day of the brain (40), which corresponds to 20.65 mole ATP/ person/day, when a conversion of 1 mole of glucose is converted into 31 mole ATP is assumed. Using the unique flux distribution computed in 3.2., we can determine the overall brain ATP consumption rate.

printRxnFormula(female,'Adipocytes\_DM\_lipid\_storage');

Adipocytes\_DM\_lipid\_storage Adipocytes\_tag\_hs[c] -> slack\_Adipocytes\_DM\_lipid\_storage

Display all reactions that produce Adipocytes\_tag\_hs[c] :

Tag = findRxnsFromMets(female, 'Adipocytes\_tag\_hs[c]');

for i = 1 : length(Tag)

if female.A(strmatch('Adipocytes\_tag\_hs[c]',female.mets,'exact'),strmatch(Tag{i},female.rxns,'exact')) < 0 %not produced

del(i,1) =1;

end

end

Tag(find(del)) = [];

printRxnFormula(female,Tag);

Adipocytes\_DGAT Adipocytes\_Rtotal3coa[c] + Adipocytes\_dag\_hs[c] -> Adipocytes\_coa[c] + Adipocytes\_tag\_hs[c] + slack\_Adipocytes\_DGAT
Adipocytes\_TAGt Adipocytes\_tag\_hs[e] <=> Adipocytes\_tag\_hs[c] + slack\_Adipocytes\_TAGt + slack\_Adipocytes\_TAGt\_R
Adipocytes\_TAG\_HSad 2 Adipocytes\_h2o[c] + Adipocytes\_pmtcoa[c] + 2 Adipocytes\_lnlccoa[c] + Adipocytes\_stcoa[c] + 2 Adipocytes\_odecoa[c] + 2 Adipocytes\_glyc3p[c] -> 2 Adipocytes\_pi[c] + 6 Adipocytes\_coa[c] + 2 Adipocytes\_tag\_hs[c] + slack\_Adipocytes\_TAG\_HSad
Adipocytes\_TAG\_HSad\_NE 2 Adipocytes\_h2o[c] + Adipocytes\_pmtcoa[c] + Adipocytes\_hdcoa[c] + Adipocytes\_tdcoa[c] + Adipocytes\_stcoa[c] + 2 Adipocytes\_odecoa[c] + 2 Adipocytes\_glyc3p[c] -> 2 Adipocytes\_pi[c] + 6 Adipocytes\_coa[c] + 2 Adipocytes\_tag\_hs[c] + slack\_Adipocytes\_TAG\_HSad\_NE
Adipocytes\_TAG\_HSad\_E 2 Adipocytes\_h2o[c] + Adipocytes\_arachdcoa[c] + Adipocytes\_lnlncacoa[c] + Adipocytes\_tmndnccoa[c] + Adipocytes\_lnlccoa[c] + Adipocytes\_c226coa[c] + 2 Adipocytes\_glyc3p[c] -> 2 Adipocytes\_pi[c] + 6 Adipocytes\_coa[c] + 2 Adipocytes\_tag\_hs[c] + slack\_Adipocytes\_TAG\_HSad\_E
Adipocytes\_MEMBRANE\_DEGRx Adipocytes\_lipid\_membrane[c] -> 2e-06 Adipocytes\_pail\_hs[c] + 0.000119 Adipocytes\_pchol\_hs[c] + 5.9e-05 Adipocytes\_pe\_hs[c] + 0.2784 Adipocytes\_chsterol[c] + 9e-06 Adipocytes\_clpn\_hs[c] + 0.000815 Adipocytes\_tag\_hs[c] + 2e-06 Adipocytes\_ps\_hs[c] + 1.2e-05 Adipocytes\_sphmyln\_hs[c]
Adipocytes\_TAG\_dcacoa 0.3 Adipocytes\_h2o[c] + Adipocytes\_dcacoa[c] + 0.3 Adipocytes\_glyc3p[c] -> 0.3 Adipocytes\_pi[c] + Adipocytes\_coa[c] + 0.3 Adipocytes\_tag\_hs[c] + slack\_Adipocytes\_TAG\_dcacoa
Adipocytes\_TAG\_ddcacoa 0.3 Adipocytes\_h2o[c] + Adipocytes\_ddcacoa[c] + 0.3 Adipocytes\_glyc3p[c] -> 0.3 Adipocytes\_pi[c] + Adipocytes\_coa[c] + 0.3 Adipocytes\_tag\_hs[c] + slack\_Adipocytes\_TAG\_ddcacoa
Adipocytes\_TAG\_ttdceacoa 0.3 Adipocytes\_h2o[c] + 0.3 Adipocytes\_glyc3p[c] + Adipocytes\_ttdceacoa[c] -> 0.3 Adipocytes\_pi[c] + Adipocytes\_coa[c] + 0.3 Adipocytes\_tag\_hs[c] + slack\_Adipocytes\_TAG\_ttdceacoa
Adipocytes\_TAG\_ptdcacoa 0.3 Adipocytes\_h2o[c] + 0.3 Adipocytes\_glyc3p[c] + Adipocytes\_ptdcacoa[c] -> 0.3 Adipocytes\_pi[c] + Adipocytes\_coa[c] + 0.3 Adipocytes\_tag\_hs[c] + slack\_Adipocytes\_TAG\_ptdcacoa
Adipocytes\_TAG\_hpdcacoa 0.3 Adipocytes\_h2o[c] + 0.3 Adipocytes\_glyc3p[c] + Adipocytes\_hpdcacoa[c] -> 0.3 Adipocytes\_pi[c] + Adipocytes\_coa[c] + 0.3 Adipocytes\_tag\_hs[c] + slack\_Adipocytes\_TAG\_hpdcacoa
Adipocytes\_TAG\_strdnccoa 0.3 Adipocytes\_h2o[c] + Adipocytes\_strdnccoa[c] + 0.3 Adipocytes\_glyc3p[c] -> 0.3 Adipocytes\_pi[c] + Adipocytes\_coa[c] + 0.3 Adipocytes\_tag\_hs[c] + slack\_Adipocytes\_TAG\_strdnccoa
Adipocytes\_TAG\_CE5151 0.3 Adipocytes\_h2o[c] + 0.3 Adipocytes\_glyc3p[c] + Adipocytes\_CE5151[c] -> 0.3 Adipocytes\_pi[c] + Adipocytes\_coa[c] + 0.3 Adipocytes\_tag\_hs[c] + slack\_Adipocytes\_TAG\_CE5151
Adipocytes\_TAG\_btcoa 0.3 Adipocytes\_h2o[c] + 0.3 Adipocytes\_glyc3p[c] + Adipocytes\_btcoa[c] -> 0.3 Adipocytes\_pi[c] + Adipocytes\_coa[c] + 0.3 Adipocytes\_tag\_hs[c] + slack\_Adipocytes\_TAG\_btcoa
Adipocytes\_TAG\_c226coa 0.3 Adipocytes\_h2o[c] + Adipocytes\_c226coa[c] + 0.3 Adipocytes\_glyc3p[c] -> 0.3 Adipocytes\_pi[c] + Adipocytes\_coa[c] + 0.3 Adipocytes\_tag\_hs[c] + slack\_Adipocytes\_TAG\_c226coa
Adipocytes\_TAG\_clpndcoa 0.3 Adipocytes\_h2o[c] + Adipocytes\_clpndcoa[c] + 0.3 Adipocytes\_glyc3p[c] -> 0.3 Adipocytes\_pi[c] + Adipocytes\_coa[c] + 0.3 Adipocytes\_tag\_hs[c] + slack\_Adipocytes\_TAG\_clpndcoa
Adipocytes\_TAG\_adrncoa 0.3 Adipocytes\_h2o[c] + Adipocytes\_adrncoa[c] + 0.3 Adipocytes\_glyc3p[c] -> 0.3 Adipocytes\_pi[c] + Adipocytes\_coa[c] + 0.3 Adipocytes\_tag\_hs[c] + slack\_Adipocytes\_TAG\_adrncoa
Adipocytes\_TAG\_doco13ecoa 0.3 Adipocytes\_h2o[c] + 0.3 Adipocytes\_glyc3p[c] + Adipocytes\_doco13ecoa[c] -> 0.3 Adipocytes\_pi[c] + Adipocytes\_coa[c] + 0.3 Adipocytes\_tag\_hs[c] + slack\_Adipocytes\_TAG\_doco13ecoa
Adipocytes\_TAG\_docoscoa 0.3 Adipocytes\_h2o[c] + 0.3 Adipocytes\_glyc3p[c] + Adipocytes\_docoscoa[c] -> 0.3 Adipocytes\_pi[c] + Adipocytes\_coa[c] + 0.3 Adipocytes\_tag\_hs[c] + slack\_Adipocytes\_TAG\_docoscoa

Note that the same reactions are present in the male model.

The muscle energy expenditure needed for physical activity is defined as:

printRxnFormula(female,'Muscle\_DM\_atp\_c\_');

Muscle\_DM\_atp\_c\_ Muscle\_h2o[c] + Muscle\_atp[c] -> Muscle\_h[c] + Muscle\_adp[c] + Muscle\_pi[c] + slack\_Muscle\_DM\_atp\_c\_

Note that the current constraints on the whole-body models correspond to 'at rest'. Hence, the maximal possible muscle energy expenditure calculated here represents activity with low metabolic equivalents (METs).

In the following, we calculate the maximal possible fat storage capability and muscle energy expenditure for each sex under different dietary conditions.

o=1;

EUAverageDietNew;

male = setDietConstraints(male,Diet);

female = setDietConstraints(female,Diet);

% set constraints specific for activity-induced activity

female.lb(strmatch('Brain\_DM\_atp\_c\_',female.rxns)) = 3500;

male.lb(strmatch('Brain\_DM\_atp\_c\_',male.rxns)) = 3500;

male.lb(strmatch('Heart\_DM\_atp\_c\_',male.rxns)) = 6000;

female.lb(strmatch('Heart\_DM\_atp\_c\_',female.rxns)) = 6000;

male\_EU = changeObjective(male,'Muscle\_DM\_atp\_c\_');

male\_EU.osense = -1;

tic;[solution,LPProblem]=solveCobraLPCPLEX(male\_EU,1,0,0,[],0,...

LPSolver);toc

CPLEX Callable Library Release 12.3.0.0
Multiple threads enabled, disabling callbacks and screen output
Optimal solution found, Objective 32849.5
Elapsed time is 43.303793 seconds.

Table\_Muscle\_DM\_atp\_c\_(o,1) = solution.full(find(male\_EU.c));

female\_EU = setDietConstraints(female,Diet);

female\_EU = changeObjective(female\_EU,'Muscle\_DM\_atp\_c\_');

female\_EU.osense = -1;

tic;[solution,LPProblem]=solveCobraLPCPLEX(female\_EU,1,0,0,[],0,...

LPSolver);toc

CPLEX Callable Library Release 12.3.0.0
Multiple threads enabled, disabling callbacks and screen output
Optimal solution found, Objective 33711.2
Elapsed time is 46.479591 seconds.

Table\_Muscle\_DM\_atp\_c\_(o,2) = solution.full(find(female\_EU.c));

o=o+1;

%% fat storage

o = 1;

EUAverageDietNew;

male = setDietConstraints(male,Diet);

female = setDietConstraints(female,Diet);

male\_EU = changeObjective(male,'Adipocytes\_DM\_lipid\_storage');

male\_EU.osense = -1;

tic;[solution,LPProblem]=solveCobraLPCPLEX(male\_EU,1,0,0,[],0,...

LPSolver);toc

CPLEX Callable Library Release 12.3.0.0
Multiple threads enabled, disabling callbacks and screen output
No conflict resolution file. Consider to set conflictResolve = 1 next time.
Optimal solution is available, but with infeasibilities after unscaling, Objective 81.7571
Elapsed time is 756.282647 seconds.

Table\_Adipocytes\_DM\_lipid\_storage(o,1) = solution.full(find(male\_EU.c));

female\_EU = setDietConstraints(female,Diet);

female\_EU = changeObjective(female\_EU,'Adipocytes\_DM\_lipid\_storage');

female\_EU.osense = -1;

tic;[solution,LPProblem]=solveCobraLPCPLEX(female\_EU,1,0,0,[],0,...

LPSolver);toc

CPLEX Callable Library Release 12.3.0.0
Multiple threads enabled, disabling callbacks and screen output
Optimal solution found, Objective 77.1451
Elapsed time is 40.075571 seconds.

Table\_Adipocytes\_DM\_lipid\_storage(o,2) = solution.full(find(female\_EU.c));

o=o+1;

% give results: male = first column; female = second column

Table\_Muscle\_DM\_atp\_c\_

Table\_Muscle\_DM\_atp\_c\_ =

1.0e+04 \*
3.2850 3.3711

Table\_Adipocytes\_DM\_lipid\_storage

Table\_Adipocytes\_DM\_lipid\_storage =

81.7571 77.1451

To put the muscle energy expenditure into context, one can convert them into, e.g., number of steps. Note that the model setup only captures physical activities with low metabolic equivalents, e.g., slow walking.

Table\_Muscle\_DM\_atp\_c\_expanded(1,:) = {'Diet' 'Male-muscle ATP' 'Male-Energy (kcal)' 'Female-muscle ATP' 'Female-Energy (kcal)'};

Table\_Muscle\_DM\_atp\_c\_expanded(2,1) = {'Av. European'};

for i = 1 : size(Table\_Muscle\_DM\_atp\_c\_,1)

[Energy\_kJ,Energy\_kcal\_male] = convertATPflux2StepNumer(Table\_Muscle\_DM\_atp\_c\_(i,1), 'male', 70, 170);

[Energy\_kJ,Energy\_kcal\_female] = convertATPflux2StepNumer(Table\_Muscle\_DM\_atp\_c\_(i,2), 'female', 58, 160);

Table\_Muscle\_DM\_atp\_c\_expanded(i+1,2:5) = {num2str(Table\_Muscle\_DM\_atp\_c\_(i,1)) num2str(Energy\_kcal\_male) num2str(Table\_Muscle\_DM\_atp\_c\_(i,2)) num2str(Energy\_kcal\_female)};

end

Table\_Muscle\_DM\_atp\_c\_expanded'

ans =

'Diet' 'Av. European'
'Male-muscle ATP' '32849.5482'
'Male-Energy (kcal)' '502.4793'
'Female-muscle ATP' '33711.2203'
'Female-Energy (kcal)' '515.6598'

%clear female\_\* male\_\*

The following calculation has been done for this:

- The gross energy cost of walking is assumed to be 3 J/kg/m (54).
- In the human muscle, the hydrolysis of 1 mol ATP yields 64KJ based on (52) [Table 1]
- Hence, Energy\_kJ = ATP\_hydrolysis\_flux/1000 \* 64; %in kJ per person per day and with 1 kJ = 0.239006 kcal: Energy\_kcal = Energy\_kJ\*0.239006; %in kcal per person per day
- Consequently, Distance (in meter) = Energy\_kJ\*1000/(3\*weight); % per person per day
- The stride length was calculated using the formula (55)
- Women: 0.413 \* height (in cm)
- Men: 0.415 \* height ( in cm)
- Finally, the StepNumber = Meter\*(Stride/100);

3.5. Compute metabolic organ essentiality

To determine the essentiality of each organ in the whole-body model, we first remove each organ separately from the whole-body biomass reaction and then set the upper and lower bounds of all corresponding organ-specific reactions to zero. An organ is defined non-essential if the maximal possible flux value through the whole-body biomass reactions is equal or greater than 1.

The output of the function organEssentiality is a table, ResultsOrganEss, where the 1st column is the organ name, the 2nd column is the maximal possible flux value for the whole-body biomass reaction, the 3rd column contains its minimal possible flux value, and the 4th column reports the solver status1 = feasible, 5 = feasible with numerical difficulties (rescaling issues), 3 = infeasible.

% require Brain\_dm\_atp\_c\_ to be at least 5000 mmol/day/person

male2 = changeRxnBounds(male,'Brain\_DM\_atp\_c\_',0,'l');

female2 = changeRxnBounds(female,'Brain\_DM\_atp\_c\_',0,'l');

male2 = changeRxnBounds(male2,'Brain\_EX\_o2(e)\_[csf]',-1000,'u');

female2 = changeRxnBounds(female2,'Brain\_EX\_o2(e)\_[csf]',-1000,'u');

if 0

[ResultsOrganEss\_male] = organEssentiality(male2)

[ResultsOrganEss\_female] = organEssentiality(female2)

save ResultsOrganEss\_female\_male ResultsOrganEss\_female ResultsOrganEss\_male

else

load ResultsOrganEss\_female\_male

ResultsOrganEss\_male

ResultsOrganEss\_female

end

ResultsOrganEss\_male =

'Heart' '2.6571' [] '1'
'Muscle' '2.6571' [] '1'
'Lung' [ NaN] [NaN] '3'
'Skin' [ NaN] [NaN] '3'
'Stomach' '2.6571' [] '1'
'sIEC' '4.1807' [] '1'
'Colon' '2.6571' [] '1'
'Urinarybladder' '2.6571' [] '1'
'Retina' '2.6571' [] '1'
'Scord' '2.6571' [] '1'
'Brain' '2.6571' [] '1'
'Adipocytes' [ NaN] [NaN] '3'
'Liver' [ NaN] [NaN] '3'
'Gall' '2.011' [] '1'
'Kidney' [ NaN] [NaN] '3'
'Pancreas' '2.0744' [] '1'
'Spleen' '2.6571' [] '1'
'Agland' [ NaN] [NaN] '3'
'Thyroidgland' '2.6571' [] '1'
'Pthyroidgland' '2.6571' [] '1'
'Ovary' '2.6571' [] '1'
'Uterus' '2.6571' [] '1'
'Breast' '2.6571' [] '1'
'Cervix' '2.6571' [] '1'
'Testis' '2.6571' [] '1'
'Prostate' '2.6571' [] '1'
'Bcells' '2.6571' [] '1'
'CD4Tcells' '2.6571' [] '1'
'Nkcells' '2.6571' [] '1'
'Monocyte' '2.6571' [] '1'
'Platelet' '2.6571' [] '1'
'RBC' [ NaN] [NaN] '3'

ResultsOrganEss\_female =

'Heart' '1.2749' [] '1'
'Muscle' '1.2749' [] '1'
'Lung' [ NaN] [NaN] '3'
'Skin' [ NaN] [NaN] '3'
'Stomach' '1.2749' [] '1'
'sIEC' '4.1821' [] '1'
'Colon' '1.2749' [] '1'
'Urinarybladder' '1.2749' [] '1'
'Retina' '1.2749' [] '1'
'Scord' '1.2749' [] '1'
'Brain' '1.2749' [] '1'
'Adipocytes' [ NaN] [NaN] '3'
'Liver' [ NaN] [NaN] '3'
'Gall' '1.2715' [] '1'
'Kidney' [ NaN] [NaN] '3'
'Pancreas' '1.0065' [] '1'
'Spleen' '1.2749' [] '1'
'Agland' [ NaN] [NaN] '3'
'Thyroidgland' '1.2749' [] '1'
'Pthyroidgland' '1.2749' [] '1'
'Ovary' '1.2749' [] '1'
'Uterus' '1.2749' [] '1'
'Breast' '1.2749' [] '1'
'Cervix' '1.2749' [] '1'
'Testis' '1.2749' [] '1'
'Prostate' '1.2749' [] '1'
'Bcells' '1.2749' [] '1'
'CD4Tcells' '1.2749' [] '1'
'Nkcells' '1.2749' [] '1'
'Monocyte' '1.2749' [] '1'
'Platelet' '1.2749' [] '1'
'RBC' [ NaN] [NaN] '3'

3.6. Obtain statistics on the whole-body metabolic reconstructions

% Basic statistical analysis

[TableHHStats] = getBasicHarveyStats(male,female)

TableHHStats =

'' 'Harvetta' 'Harvey'
'Number of Reactions' '83521' '81094'
'Number of Metabolites' '58851' '56452'
'Number of Genes (transcripts)' '2070' '2070'
'Number of Genes (unique)' '1723' '1723'
'Number of Subsystems' '104' '104'
'Blood compartment metabolites' '978' '979'
'Urine metabolites' '920' '923'
'Portal vein metabolites' '487' '488'
'Bile duct metabolites' '262' '261'
'CSF metabolites' '508' '517'
'Diet metabolites' '1391' '1392'
'Fecal metabolites' '1398' '1400'
'Sweat metabolites' '57' '57'
'Air metabolites' '3' '3'
'Milk metabolites' '84' '0'

[ResultsMaleFemale] = compareMaleFemale(male,female)

ResultsMaleFemale =

MaleOnly: {7879×1 cell}
FemaleOnly: {10306×1 cell}
BothGender: {73215×1 cell}
OrgansNumRxnMale: [38×3 double]
OrgansNumRxnFemale: [40×3 double]
maleOrgans: {38×1 cell}
femaleOrgans: {40×1 cell}
FemaleGallSSEnrich: {36×2 cell}
MaleGallSSEnrich: {35×2 cell}
MaleOnlyBiofluid: {1183×1 cell}
FemaleOnlyBiofluid: {1590×1 cell}

3.7. Generate organ compendium from whole-body metabolic models

At this point, we create the organ compendium from the whole-body metabolic models and perform basic tests on the models. A different organ compendium is generated for each sex.

Therefore, each organ is extracted from the WBM model, while maintaining the biolfuid compartments. For each metabolite in a biofluid compartment, a corresponding exchange reaction is added in the form of EX\_A: 1 A[e] <->. Note that constraints on the biofluid transport reactions in Harvey are not conserved but reset to default bounds (0, 1000, and -1000).

The different organs are stored in the OrganCompendium structure (see Figure above). We then perform basic tests on each organ, such as leak test, metabolic function test, and carbon source ATP yield under aerobic and anaerobic conditions. Please refer to performSanityChecksonRecon.m for more details (or Method Section 1.5). For each organ, a brief report is provided and whether the organ model passed a test or not (see Figure above, Sanity checks, on where to find the report in the OrganCompendium structure).

Recon3\* is also used and the same basic tests are performed. The aim here is to allow comparison of the organ's performance with the orginal input model.

The overview of all carbon source yields, including those for Recon 3\*, are provided in TableCSources.

Using the organ compendium for a given sex, we then generate statistics for each organ as well as comparison between the sexes. The TableProp contains the information and some plots are generated.

clear female2 femaleB\* male2 maleM\* a A ans atp B BM BR\* production R S F consumption solution\* o minInf minConstraints

clear maxInf maxConstraints i del BMmets BMatp BMatp\* Bounds femaleMod LPProblem vf vr Tag

% get organs from the male whole-body metabolic model and performs basic sanity checks. Note that single gene deletion analysis is not performed.

if 0

[OrganCompendium\_male,TableCSources\_male] = getOrgansFromHarvey(male);

save OrganAtlas\_Harvey OrganCompendium\_male TableCSources\_male

% get organs from the female whole-body metabolic model

[OrganCompendium\_female,TableCSources\_female] = getOrgansFromHarvey(female);

save OrganAtlas\_Harvetta OrganCompendium\_female TableCSources\_female

else

load OrganAtlas\_Harvetta.mat;

% OrganCompendium\_female = OrganCompendium;

load OrganAtlas\_Harvey.mat

% OrganCompendium\_male = OrganCompendium;

end

Compute statistics on each organ in the organ compendium

A more comprehensive comparison output is provided in the file 'Results\_StatsOrganComp.mat' that is created at the end of the function getStatsOrganComp.m. TableGRM contains the information displayed in Figure 2. TableProp\_female and TableProp\_male contains the information summarized in Figure 2 and detailed in Table SX.

% if you wish to display also the violin plots from Figure 2 in the paper please set the following option to 1

violinPlots = 0;

[TableProp\_female,TableProp\_male,TableGRM] = getStatsOrganComp(female, male, OrganCompendium\_female, OrganCompendium\_male,violinPlots);

All statistics calculated in the function (getStatsOrganComp) are save in the file: Results\_StatsOrganComp.mat

load Results\_StatsOrganComp;

Calculate the number of metabolites present in only one organ:

sumMetsOrgan = sum(TableMetsNum\_female')';

length(find(sumMetsOrgan==1))

ans = 212

Calculate the number of metabolites present in two organ:

sumMetsOrgan = sum(TableMetsNum\_female')';

length(find(sumMetsOrgan==2))

ans = 166

Calculate the number of genes present in all organs:

sumGenesOrgan = sum(TableGenes\_femaleNum')';

HouseHoldGenes = length(find(sumGenesOrgan==size(TableGenes\_femaleNum,2)))

HouseHoldGenes = 204

Calculate the number of genes present in all organs, excluding the RBC:

sumGenesOrgan = sum(TableGenes\_femaleNum(:,1:size(TableGenes\_femaleNum,2)-1)')';

length(find(sumGenesOrgan==size(TableGenes\_femaleNum,2)-1))-HouseHoldGenes

ans = 171

Create table with all results from the metabolic functions for all organs: TableMetFuncTests (Table SZ).

% Create table with all results from the metabolic functions for all

% organs:

Omale = fieldnames(OrganCompendium\_male);

Ofemale = fieldnames(OrganCompendium\_female);

clear TableMetFuncTests

cnt = 1;

for i = 1 : length(Omale)

if ~strcmp('gender',Omale{i}) %&& ~strcmp('Recon3DHarvey',Omale{i})

if i == 1

TableMetFuncTests{2,cnt} = 'Metabolic function names';

L = length(OrganCompendium\_male.(Omale{i}).Sanity.TestSolutionNameOpenSinks(:,1));

TableMetFuncTests(3:L+2,cnt) =OrganCompendium\_male.(Omale{i}).Sanity.TestSolutionNameOpenSinks(:,1);

end

TableMetFuncTests{1,cnt+1} = 'male WBM model';

TableMetFuncTests{2,cnt+1} = Omale{i};

TableMetFuncTests(3:L+2,cnt+1)= OrganCompendium\_male.(Omale{i}).Sanity.TestSolutionNameOpenSinks(:,2);

cnt = cnt + 1;

end

end

for i = 1 : length(Ofemale)

if ~strcmp('gender',Ofemale{i}) %&& ~strcmp('Recon3DHarvey',Omale{i})

TableMetFuncTests{1,cnt+1} = 'female WBM model';

TableMetFuncTests{2,cnt+1} = Ofemale{i};

TableMetFuncTests(3:L+2,cnt+1)= OrganCompendium\_female.(Ofemale{i}).Sanity.TestSolutionNameOpenSinks(:,2);

cnt = cnt + 1;

end

end

3.8. Predict metabolite biomarkers for 57 inborn-errors of metabolism (IEMs)

The WBM models can be used predict known and novel biomarker metabolites. The script (runIEM\_HH.m) can be used to run the simulations for 49 pre-defined IEMs.

In the script, for each IEM, the reaction(s) are defined that are connected to the defective gene(s) as well as the reported biomarker metabolites in the respective biofluid compartment. First, all organs that contain the reaction(s) are identified. Second, for each reported biomarker metabolite a demand reaction was added to the model in the corresponding biofluid compartment. Third, the maximal possible sum of fluxes through these organ-specific reactions is calculated for the healthy state. For healthy state, the lower and upper bound constrain on the sum of fluxes was set to the calculated maximum and the maximally possible flux through each of the known biomarker metabolite demand reaction was calcuated. For the disease state, the lower and upper bound constrain on the sum of fluxes was set to zero and the maximally possible flux through each of the known biomarker metabolite demand reaction was calcuated. Finally, if the computed flux through a biomarker metabolite demand was smaller in the disease state than in the healthy state, the biomarker was deemed to be decreased (down) in the disease state, if both flux values were identical the biomarker was deemed unchanged/inconclusive, and if the disease flux value was higher than in the healthy state, the biomarker was deemed to be increased. We then compared with the reported changes in biomarker concentrations for the respective IEM.

if 0

% uncomment one of the following three:

gender = 'male'; % for Harvey

gender = 'female'; % for Harvetta

gender = 'Recon3D'; % for Recon3D\*

runIEM\_HH;

end

The simulation results can be found in the Supplemental Table S14 or in the precomputed files.

load Results\_IEM\_Harvetta\_1\_01

load Results\_IEM\_Harvey\_1\_01

load Results\_IEM\_Recon3DStar

3.9. Determine BMF for measured BMR data

3.9.1. Identify parameters that affect prediction accuracy

We performed a sensitivity analysis on parameters that could change the predicted BMR. For benchmarking, we used published data of 13 healthy woman [64] with a normal BMR: mean age 29 (standard deviation, SD, 5), mean height 161 cm (SD 8), mean weight 57.5 kg (SD 6.3), mean BMI 22.1 (SD 2.4), mean fat-free mass 41.3 kg (SD 23.9), and mean measured basal metabolic rate (BMR) 1350.4 kcal (SD 31.1). The values were taken from Table I and II from the original publication [64].

Please note that this is not an exhaustive analysis and other factors can affect the predicted BMR and the agreement with measured BMRs. However, in all cases the predicted BMRs are within the expected range.

Needed personalized physiological data:

- age, weight, height
- measured BMR -for comparison reasons
- measured fat-free body mass (optional)

Considered parameters include:

- adjustment of organ-weights based on measured fat-free body mass
- increasing the amount of atp hydrolysis accounted for in the muscle biomass maintenance reaction

Not considered parameters that may alter the predicted BMR value include

- increasing the amount of atp hydrolysis accounted for in the adipocyte biomass maintenance reaction
- lower bound on brain- and heart atp hydrolysis (DM\_atp) for non metabolic processes
- way to estimate the cardiac output (default uses the polynomial suggested by Young et al [62]: CardiacOutput = 9119-exp(9.164-2.91e-2\*Wt+3.91e-4\*Wt^2-1.91e-6\*Wt^3); Wt = weight in kg (optionCardiacOutput = 0))

The code can be re-run using this comment (replace 0 by 1). Please note that the simulation takes time. The first file contains the results when varying the muscle atp hydrolysis by a factor of 1, 5, and 10 in the muscle biomass maintenance reaction as well as when adjusting for fat-free mass as measured in the individuals.

if 0

perform\_sensi\_BMR\_all;

end

This function saves two files. The first file contains the results when varying the muscle atp hydrolysis by a factor of 1, 5, and 10 in the muscle biomass maintenance reaction BUT the fat-free mass was not adjusted. This means that the organ distribution is as defined by the phenomenoligcal models listed in section 3.2.

The simulation results as well as the measured BMR and the BMR predicted using phenomenological models are stored in BMR\_all.

load('Results\_sensi\_2018\_12\_14\_no\_fatAdj.mat')

% Display input data

InputDataAll

InputDataAll =

'gender' '' 'female' 'female' 'female' 'female' 'female' 'female' 'female' 'female' 'female' 'female' 'female' 'female' 'female' 'female' 'female' 'female' 'female' 'female' 'female' 'female' 'female' 'female'
'age' '' '37' '33' '31' '23' '23' '32' '28' '26' '24' '24' '31' '29' '38' '32' '27' '40' '39' '36' '38' '26' '37' '39'
'Height' '' '161' '158' '163' '165' '163' '158' '163' '160' '156' '180' '151' '150' '170' '161' '160' '168' '161' '164' '162' '160' '169' '165'
'Weight' '' '57.6' '49.5' '55.7' '56.2' '53.2' '49.7' '64.6' '60.6' '59' '71.4' '62.9' '51' '56.4' '74.9' '88.6' '120.1' '83.1' '80.5' '80.3' '79.7' '82.4' '102.1'
'Fat-free' '' '41' '43.6' '42.9' '41.5' '37.6' '35' '40.8' '43.5' '41.9' '50.2' '43.3' '35.3' '40.7' '44.4' '47.5' '60.6' '47.5' '48.8' '45.9' '44.8' '48.4' '54.2'
'BMR' '' '5.37' '6.19' '5.91' '5.85' '5.06' '5.31' '5.54' '5.65' '5.76' '6.4' '5.9' '4.67' '5.86' '6.7' '6.77' '8.2' '6.64' '6.54' '6.42' '6.43' '5.59' '6.73'

Calculate BMI = weight (kg)/height(m)^2:

for i = 3 : 15 % only use the first 15 entries corresponding to normal BMI

BMI(i,1) = str2num(InputDataAll{4,i})/((str2num(InputDataAll{3,i})/100)^2);

BMR\_measured(i,1) = str2num(InputDataAll{6,i})\*0.239006\*1000; % in kcal

end

Plot BMI against measured BMR (in kcal):

figure;

scatter(BMI(3:end),BMR\_measured(3:end),'o');

xlabel('BMI');

ylabel('Measured BMR (in kcal)');

title('BMR of healthy women with normal BMI')

Store the data of the simulation results (without fat-free mass adjustment).

clear BMR\_all Rsqr

BMR\_all(:,1) = BMR\_measured;

BMR\_all(1:15,2:4) = Energy\_kcal\_femaleBMR2(1:15,1:3); % no fat adjustment

The second file contains the results when varying the muscle atp hydrolysis by a factor of 1, 5, and 10 in the muscle biomass maintenance reaction as well as when adjusting for fat-free mass as measured in the individuals.

load('Results\_sensi\_2018\_12\_14\_fatAdj.mat')

BMR\_all(:,5:7) = Energy\_kcal\_femaleBMR2(:,1:3);

Calculate and store the BMR calculated basd on the phenomenological data:

for i = 3 : 15 % size(InputDataAll,2) - only normal BMI's

BMR\_female\_All(:,i) = calculateBMR('female', str2num(InputDataAll{4,i}), str2num(InputDataAll{3,i}), str2num(InputDataAll{2,i}));

end

BMR\_all(:,8:10) = BMR\_female\_All';

% Display all stored data

BMR\_all(3:end,:) % first two rows are empty

ans =

1.0e+03 \*
1.2835 1.1173 1.2613 1.4411 1.1163 1.2559 1.4272 1.3308 1.3188 1.2363
1.4794 1.1005 1.2393 1.4031 1.1126 1.3004 1.5500 1.2665 1.2519 1.1565
1.4125 1.1100 1.2540 1.4308 1.1146 1.2784 1.4791 1.3444 1.3334 1.2597
1.3982 1.1112 1.2555 1.4328 1.1127 1.2605 1.4436 1.3902 1.3789 1.3173
1.2094 1.1031 1.2478 1.7291 1.0992 1.2285 1.3674 1.3579 1.3449 1.2748
1.2691 1.1009 1.2400 1.4043 1.0952 1.2057 1.6751 1.2730 1.2581 1.1635
1.3241 1.1187 1.2630 1.4458 1.1164 1.2373 1.3838 1.4435 1.4287 1.3638
1.3504 1.1139 1.2568 1.4389 1.1144 1.2650 1.4551 1.4090 1.3911 1.3150
1.3767 1.1098 1.2545 1.4357 1.1095 1.2532 1.4320 1.3957 1.3725 1.2840
1.5296 1.1075 1.2593 1.4479 1.1101 1.2934 1.5274 1.5587 1.5615 1.5580

Now, we calculate the linear regression (of the form y = a0+a1\*x) and display the in silico and phenomenologically predicted BMRs (y axis) against the measured BMRs (x axis).

figure1 = figure;

for i = 2 : 10

subplot1 = subplot(3,3,i-1,'Parent',figure1);

% calculate the linear regression of the form y = a0+a1\*x.

[a0(i-1,1),a1(i-1,1),Rsqr(i-1,1), Residuals] = linearRegression(BMR\_all(3:end,1),BMR\_all(3:end,i));

scatter(BMR\_all(3:end,1),BMR\_all(3:end,i));

hold on

plot(BMR\_all(3:end,1),(a0(i-1)+BMR\_all(3:end,1)\*a1(i-1)));

xlim(subplot1,[1000 1700]);

ylim(subplot1,[1050 1700]);

if i==2

% Muscle atp hydrolysis adjustment in muscle biomass maintenance: factor 1

title(strcat('M1: R2= ', num2str(round(Rsqr(1),2))));

elseif i==3

% Muscle atp hydrolysis adjustment in muscle biomass maintenance: factor 5

title(strcat('M5: R2= ', num2str(round(Rsqr(2),2))));

elseif i ==4

% Muscle atp hydrolysis adjustment in muscle biomass maintenance: factor 10

title(strcat('M10: R2= ', num2str(round(Rsqr(3),2))))

elseif i==5

% Muscle atp hydrolysis adjustment in muscle biomass maintenance: factor 1, fat-free mass adjusted

title(strcat('M1, FFA: R2= ', num2str(round(Rsqr(4),2))));

elseif i==6

% Muscle atp hydrolysis adjustment in muscle biomass maintenance: factor 5, fat-free mass adjusted

title(strcat('M5, FFA: R2= ', num2str(round(Rsqr(5),2))));

elseif i ==7

% Muscle atp hydrolysis adjustment in muscle biomass maintenance: factor 10, fat-free mass adjusted

title(strcat('M10, FFA: R2= ', num2str(round(Rsqr(6),2))))

elseif i ==8

% phenomenological model for BMR estimation based on original Harris & Benedict equations.

title(strcat('H & B: R2= ', num2str(round(Rsqr(7),2))))

elseif i ==9

% phenomenological model for BMR estimation based on original Harris & Benedict equations

% revised by Roza and Shizgal.

title(strcat('R & S: R2= ','', num2str(round(Rsqr(8),2))))

elseif i ==10

% phenomenological model for BMR estimation based on original Harris & Benedict equations

% revised by Mifflin and St Jeor.

title(strcat('M & S: R2= ', num2str(round(Rsqr(9),2))))

end

xlabel('measured BMR (kcal)');

ylabel('Predicted BMR (kcal)');

end

The x-axis represents the measured BMR in kcal. The y-axis is the predicted BMR in kcal. M1: muscle atp hydrolysis in the muscle biomass maintenance was multiplied by the factor 1. M5: muscle atp hydrolysis in the muscle biomass maintenance was multiplied by the factor 5. M10: muscle atp hydrolysis in the muscle biomass maintenance was multiplied by the factor 10. FFA: Fat-free mass was adjusted for in each personalized model. H & B: muscle atp hydrolysis in the muscle biomass maintenance was multiplied by the factor 10. R & S: phenomenological model for BMR estimation based on original Harris & Benedict equations revised by Roza and Shizgal. M & S: phenomenological model for BMR estimation based on original Harris & Benedict equations revised by Mifflin and St Jeor.

As you can see, the best R2 was obtained when the muscle atp hydrolysis in the muscle biomass maintenance was multiplied by the factor 5 and the measured fat-free mass was adjusted for.

3.9.2 Validation of the parameters in an independent data set

As the adjustment for fat-free mass was important, we selected an independent data set that is publically available and has the fat-free mass measured in addition to the BMR. The data set [65] has been derived from 28 athletes from the Modern Pentathlon Rio de Janeiro Federation. The data set included one pre-pubertal individual (female) and two individuals of Asian decent (1 female, 1 male), which we excluded from the comparison as the phenomenoligcal models used to derive the physiological constraints for Harvetta and Harvey do not capture these populations well. The considered data contains 9 females and 16 males. The mean age was 14.7 years (SD 2.3) for females and 14.8 years (SD 1.7) for males. The mean height was 159.3 cm (SD 9.9) for females and 169.3 cm (SD 8.5) for males. The mean weight was 50.5 kg (SD 8.9) for females and 57.3 kg (SD 8.6) for males. The fat-free body mass was 39.15 kg (SD 9.6) for females and 48.6 kg (SD 7.8) for males. The mean BMI was consequently 19.7 (SD 1.5) for females and 19.9 (SD 1.5) for males. The measured BMR was 1353.7 kcal (SD 143.3) for females and 1564.2 kcal (SD 208.3) for males.

We scaled the atp hydrolysis coefficients of the muscle biomass maintenance reaction in Harvey and Harvetta by the factor 5 (as determined in 3.9.1) and adjusted the fat-free body mass according to the measurements.

The simulation can be repeated using this script:

% Please set to 1 if you want to repeat the simulation. Note that this may take time.

if 0

perform\_BMR\_newData;

end

We now load the (pre-)computed results:

load Results\_sensi2\_2018\_12\_14;

This time, the females and males are present in the entire data set.

InputDataAll ={ 'gender' '' 'male' 'female' 'female' 'female' 'female' 'female' 'male' 'female' 'male' 'male' 'male' 'female' 'male' 'male' 'male' 'male' 'male' 'female' 'female' 'female' 'male' 'male' 'male' 'female' 'male' 'male' 'male' 'male'

'age' '' '15' '14' '12' '18' '16' '15' '17' '11' '13' '14' '16' '16' '12' '15' '16' '18' '13' '11' '12' '17' '12' '15' '17' '12' '15' '15' '14' '15'

'Height' '' '168' '164' '152' '162' '166' '173' '172' '161' '164' '165' '170' '156' '172' '175' '177' '173' '161' '155' '157' '165' '150' '174' '175' '139' '184' '162' '159.5' '177'

'Weight' '' '60.8' '56.8' '40.2' '53.2' '54.1' '62.9' '64.4' '56.2' '47.9' '52.8' '65.6' '49.9' '58' '69.1' '64.8' '63.5' '48' '50.4' '44.3' '58' '40.1' '61.2' '64.6' '35.4' '65.8' '47.4' '48.2' '59.6'

'Fat-free' '' '51.66' '40.08' '30.08' '43.71' '42.88' '53.44' '58.18' '36.4' '41.4' '47' '55.91' '39.52' '48.23' '59.75' '56' '53.86' '42' '33.75' '37.31' '45.31' '33.62' '53.22' '56.62' '20' '49' '38.56' '39' '50'

'BMI' '' '21.54' '21.12' '17.4' '20.27' '19.63' '21.02' '21.77' '21.68' '17.81' '19.39' '22.7' '20.5' '19.61' '22.56' '20.68' '21.22' '18.52' '20.98' '17.97' '21.3' '17.82' '20.21' '21.09' '18.32' '19.44' '18.06' '18.95' '19.02'

'BMR' '' '1661.16' '1414.97' '1282.42' '1489.45' '1277.24' '1582.33' '1515.13' '1495.3' '1775.51' '1576.21' '1476.97' '1271.78' '1551.96' '2039.63' '1808.9' '1537.86' '1457.89' '1243.59' '1443.9' '1318.07' '1249.74' '1537.93' '1633.33' '1103.2' '1508.41' '1393.85' '1167.48' '1612.75'

'Ethnicity' '' '2' '2' '1' '2' '2' '2' '1' '3' '1' '1' '3' '1' '1' '1' '2' '2' '1' '1' '2' '1' '2' '1' '1' '1' '1' '1' '2' '2'

'Sexual Maturation' '' '2' '2' '2' '3' '2' '2' '2' '2' '2' '2' '2' '2' '2' '2' '3' '3' '2' '1' '2' '2' '2' '2' '2' '2' '3' '2' '2' '3'

};

We will first sort the results. Females will be stored in BMR\_all\_female and males in BMR\_all\_males.

Note that three female models were infeasible, i.e., no solution could be calculated. These females have in common that they have a very low BMI. Consequently, only six females were compared with the measured BMR values.

m=1; f=1;

clear BMR\_all\_male BMR\_all\_female

for i = 3 : size(InputDataAll,2)

if strcmp(InputDataAll{1,i},'male')

if str2num(InputDataAll{8,i})~=3 && str2num(InputDataAll{9,i})~=1 % exclude individuals of Asian decent (3) or pre-puberty(1)

BMR\_all\_male(m,1) = str2num(InputDataAll{7,i});

BMR\_all\_male(m,2) = Energy\_kcal\_maleBMR2(i);

BMR\_all\_male(m,3) = BMR\_male\_20(3,i);

m=m+1;

end

elseif strcmp(InputDataAll{1,i},'female')

if Energy\_kcal\_femaleBMR2(i)>0 && Energy\_kcal\_femaleBMR2(i)<3000 % exclude infeasible models

if str2num(InputDataAll{8,i})~=3 && str2num(InputDataAll{9,i})~=1 % exclude individuals of Asian decent (3) or pre-puberty(1)

BMR\_all\_female(f,1) = str2num(InputDataAll{7,i});

BMR\_all\_female(f,2) = Energy\_kcal\_femaleBMR2(i);

BMR\_all\_female(f,3) = BMR\_female\_20(3,i);

f=f+1;

end

end

end

end

figure1 =figure;

subplot1 = subplot(2,2,1,'Parent',figure1);

[a0,a1,Rsqr\_female(1,1), Residuals] = linearRegression(BMR\_all\_female(1:end,1),BMR\_all\_female(1:end,2));

scatter(BMR\_all\_female(1:end,1),BMR\_all\_female(1:end,2));

hold on

plot(BMR\_all\_female(1:end,1),(a0+BMR\_all\_female(1:end,1)\*a1));

title(strcat('Harvetta: R2= ', num2str(round(Rsqr\_female(1),2))));

xlim(subplot1,[1000 2000]);

ylim(subplot1,[1050 2000]);

xlabel('measured BMR (kcal)');

ylabel('Predicted BMR (kcal)');

subplot1 = subplot(2,2,2,'Parent',figure1);

[a0,a1,Rsqr\_female(1,2)] = linearRegression(BMR\_all\_female(1:end,1),BMR\_all\_female(1:end,3));

scatter(BMR\_all\_female(1:end,1),BMR\_all\_female(1:end,3));

hold on

plot(BMR\_all\_female(1:end,1),(a0+BMR\_all\_female(1:end,1)\*a1));

title(strcat('Female: Mifflin & St Jeor: R2= ', num2str(round(Rsqr\_female(2),2))));

xlim(subplot1,[1000 2000]);

ylim(subplot1,[1050 2000]);

xlabel('measured BMR (kcal)');

ylabel('Predicted BMR (kcal)');

subplot1 = subplot(2,2,3,'Parent',figure1);

[a0,a1,Rsq\_male(1,1)] = linearRegression(BMR\_all\_male(1:end,1),BMR\_all\_male(1:end,2));

scatter(BMR\_all\_male(1:end,1),BMR\_all\_male(1:end,2));

hold on

plot(BMR\_all\_male(1:end,1),(a0+BMR\_all\_male(1:end,1)\*a1));

title(strcat('Harvey: R2= ', num2str(round(Rsq\_male(1),2))));

xlim(subplot1,[1000 2000]);

ylim(subplot1,[1050 2000]);

xlabel('measured BMR (kcal)');

ylabel('Predicted BMR (kcal)');

subplot1 = subplot(2,2,4,'Parent',figure1);

[a0,a1,Rsq\_male(1,2)] = linearRegression(BMR\_all\_male(1:end,1),BMR\_all\_male(1:end,3));

scatter(BMR\_all\_male(1:end,1),BMR\_all\_male(1:end,3));

hold on

plot(BMR\_all\_male(1:end,1),(a0+BMR\_all\_male(1:end,1)\*a1))

title(strcat('Male: Mifflin & St Jeor: R2= ', num2str(round(Rsq\_male(2),2))));

xlim(subplot1,[1000 2000]);

ylim(subplot1,[1050 2000]);

xlabel('measured BMR (kcal)');

ylabel('Predicted BMR (kcal)');

3.10. Host-microbiome modeling

Note that for practical purposes, we only show the procedure for one host-microbiome model.

3.10.1 . Generate personalized host-microbiome model (modelHM)

Note that for practical purposes, we only show the procedure for one host-microbiome model.

The microbiome models were obtained from a previous study (57, 63). Briefly, we obtained from human microbiome project (http://hmpdacc.org/) for 149 gastrointestinal tract samples the identified strain names and their relative abundance as well as associated meta-data (sex, weight, height, and heart rate). These samples were originally obtained from healthy US individuals aged 18-45 . We mapped the strain names manually onto the AGORA microbial genome-scale reconstruction collections. All unmatched strains were ignored.

Using the presence and absence information for each sample, we generated the corresponding microbiota models. Therefore, we used the Cobra Toolbox (0) function: mergeTwoModels. We added one by one the identified microbial model to the microbiota model by joining them on the level of shared exchange metabolites ([e]) and exchange reactions. Note that this compartment will be renamed to [luM] in 3.10.1.3. Once the microbiota model was constructed, we added a community biomass reaction to the model, which is the linear combination of the biomass reactions of all microbes present in the microbiota model. The stoichiometric coefficients for each microbe biomass reaction in this community microbiota biomass reaction corresponds to the relative abundance found for the strain in the given sample. To enforce that flux througn a microbial reaction only occurs when its biomass reaction has a non-zero value, we included so called coupling constraints. Effectively, these coupling constraints enforce the flux through all metabolic and transport reactions to be proportional to an organism’s biomass objective function (58).

3.10.1.1. Load microbiome model

files = {'SRS011239'};

% load microbiome model

S= load(strcat('microbiota\_model\_samp\_',files{1}));

microbiota\_model = S.microbiota\_model;

This is how the microbiota\_model looks like. The first horizontal line corresponds to the exchange metabolites that are shared between the different microbial models. The first seemingly diagonal line corresponds to the microbes. The second diagonal "line" corresponds to the coupling constraints,

figure;

spy(microbiota\_model.A)

Number of rows and number of reactions in this microbiota model:

size(microbiota\_model.A)

ans =

400612 173353

Number of non-zero entries:

nnz(microbiota\_model.A)

ans = 1246713

clear S;

Alternatively the microbiome models can be generated using the Microbiome Toolbox

if 0

% path where to save results

mkdir('microbiomeModels');

resPath=(strcat(currentDir,'/microbiomeModels'));

% path to where the COBRA Toolbox is located

global CBTDIR

toolboxPath=CBTDIR;

% path to and name of the file with dietary information.

dietFilePath=[CBTDIR filesep 'papers' filesep '2018\_microbiomeModelingToolbox' filesep 'resource' filesep 'AverageEuropeanDiet'];

% path to and name of the file with abundance information.

abunFilePath=[CBTDIR filesep 'papers' filesep '2018\_microbiomeModelingToolbox' filesep 'examples' filesep 'normCoverage.csv'];

indInfoFilePath='';

% name of objective function of organisms

objre={'EX\_biomass(e)'};

%the output is vectorized picture, change to '-dpng' for .png

figForm = '-depsc';

% number of cores dedicated for parallelization

numWorkers = 12;

% autofix for names mismatch

autoFix = 1;

% if outputs in open formats should be produced for each section (1=T)

compMod = 0;

% if documentations on patient health status is provided (0 not 1 yes)

patStat = 0;

% to enable also rich diet simulations

rDiet = 0;

% if if to use an external solver and save models with diet

extSolve = 0;

% the type of FVA function to use to solve

fvaType = 1;

% To tourn off the autorun to be able to manually execute each part of the pipeline.

autorun=1;

%END OF REQUIRED INPUT VARIABLES

%

%PIPELINE LAUNCHER

[init, modelPath, toolboxPath, resPath, dietFilePath, abunFilePath, indInfoFilePath, objre, figForm, numWorkers, autoFix, compMod, rDiet, extSolve, fvaType, autorun] = initMgPipe(modPath, toolboxPath, resPath, dietFilePath, abunFilePath, indInfoFilePath, objre, figForm, numWorkers, autoFix, compMod, rDiet, extSolve, fvaType, autorun);

end

3.10.1.2. Load the metadata from the HMP study

This file contains information on sex, heart rate (pulse), weight, height - which we will use for the personalization of the whole-body metabolic models as well as information on blood pressure, dietary habits (meat vs. vegetarian), and race. We did not use these latter features for the personalization of the WBM models.

We decided not to distinguish between vegetarian and meat eaters as the number of vegetarians was very small.

% load in HMP meta-data

load InputDataHMP\_fromxlsx;

% Example on how the input data look like:

InputData(:,1:3)

ans =

'ID' ' ' 'SRS011061'
'Gender' ' ' 'Female'
'Weight' 'kg' [ 84]
'Height' 'cm' [ 170.2000]
'BMI' ' ' [ 29]
'Age' ' ' [ 30]
'Diet' ' ' '01-Meat/fish/poultry at least three days per week'
'HeartRate' ' ' [ 79]

% 1. match filename with ID

ID = strmatch(files{1},InputData(1,:));

% 2. Sex

G = strmatch('Gender',InputData(:,1));

gender = lower(InputData(G,ID));

% get an overview on the input data

a = 1;

b = 1;

for i = 3:size(InputData,2)

if strcmp(lower(InputData(2,i)),'female')

IP\_female(a,:)=[InputData{3,i} InputData{4,i} InputData{6,i} InputData{8,i}];

a = a + 1;

elseif strcmp(lower(InputData(2,i)),'male')

IP\_male(b,:)=[InputData{3,i} InputData{4,i} InputData{6,i} InputData{8,i}];

b = b + 1;

end

end

Tabele\_Input\_Stats(1,:) = {'' 'female' 'male'};

Tabele\_Input\_Stats(2,:) = {'n' num2str(size(IP\_female,1)) num2str(size(IP\_male,1))};

Tabele\_Input\_Stats(3,:) = {'av(weight)' num2str(mean(IP\_female(:,1))) num2str(mean(IP\_male(:,1)))};

Tabele\_Input\_Stats(4,:) = {'std(weight)' num2str(std(IP\_female(:,1))) num2str(std(IP\_male(:,1)))};

Tabele\_Input\_Stats(5,:) = {'av(height)' num2str(mean(IP\_female(:,2))) num2str(mean(IP\_male(:,2)))};

Tabele\_Input\_Stats(6,:) = {'std(height)' num2str(std(IP\_female(:,2))) num2str(std(IP\_male(:,2)))};

Tabele\_Input\_Stats(7,:) = {'av(age)' num2str(mean(IP\_female(:,3))) num2str(mean(IP\_male(:,3)))};

Tabele\_Input\_Stats(8,:) = {'std(age)' num2str(std(IP\_female(:,3))) num2str(std(IP\_male(:,3)))};

Tabele\_Input\_Stats(9,:) = {'av(HR)' num2str(mean(IP\_female(:,4))) num2str(mean(IP\_male(:,4)))};

Tabele\_Input\_Stats(10,:) = {'std(HR)' num2str(std(IP\_female(:,4))) num2str(std(IP\_male(:,4)))};

Tabele\_Input\_Stats

Tabele\_Input\_Stats =

'' 'female' 'male'
'n' '66' '83'
'av(weight)' '65.5242' '80.3458'
'std(weight)' '13.1465' '11.2721'
'av(height)' '163.1091' '178.6313'
'std(height)' '6.6301' '7.0985'
'av(age)' '25.8636' '27.0361'
'std(age)' '4.9237' '5.0035'
'av(HR)' '75.2576' '71.5904'
'std(HR)' '11.129' '11.6358'

3.10.1.3. Combine the whole-body metabolic model with the microbiome model

As a next step, we join the whole-body metabolic model with the microbiome model. The whole-body metabolic model contains a compartment [luLI] representing the large intestinal lumen. It is connected to the lumen of the small intestine ([luSI]), from where dietary components and the output of the bile duct, come to the luLI. Metabolites in the luLI are either taken up by colonocytes or transported to the feces ([fe]), which is then excreted via exchange reactions (Excretion\_metabolite[fe]: 1 metabolite[fe] -->) .

Similarly, the microbiome model contains a luminal compartment ([luM]), into which each microbe can excrete or take up metabolites. Note that each microbe is an independent functional unit within this community model, meaning that the metabolic models have been joined while maintaining their own metabolic networks. The microbial interactions occur through the luminal compartment.

We join the whole-body metabolic model with the microbiome model by connecting both stoichiometric matrices and adding transport reactions between [luM] and [luLI] to the joint model, called modelHM. Any exchange reactions present in the microbiome model are removed as metabolites are provided and eliminated now through these new transport reactions and subsequently, through the reactions present in the whole-body metabolic model (e.g., excretion reactions). To represent the excretion of microbial mass, which is ultimately measured by the metagenomic tools, we add a transport reaction of the community-biomass reaction from [luM] to [luLI], from [luLI] to [fe], and its excretion via an excretion reaction.

Assumptions:

- We assumed that all identified microbes in a metagenomic sample were present in the large intestine, and not in the small intestine.
- We do not account for metabolite release in the luLI due to cell lysis.
- Due to the steady-state assumption, we cannot place concentration constraints on the microbes (abundance of each strain). However, most microbiome studies do not measure or predict the absolute microbial abundance, but instead infer the relative microbial abundance from the fecal matter. The relative abundance in the feces thus corresponds to living or dead cells, which under steady state have to be replenished in the colon through microbial growth. By placing constraints on the microbial community biomass fecal excretion reaction, we mimic precisely that. Hence, we do not need to know the absolute abundance of each strain.
- If not stated differently, we assume that the whole-body metabolic model has fecal excretion once a day (by setting the bounds on the corresponding exchange reaction to 1).
- For consistency with the coupling constraints used in the whole-body metabolic model, we used a coupling constraints of 20000 also for coupling microbial reactions with their respective biomass reaction.

% create HM model

if strcmp(gender,'male')

modelHM = combineHarveyMicrotiota(male,microbiota\_model);

elseif strcmp(gender,'female')

modelHM = combineHarveyMicrotiota(female,microbiota\_model);

end

25% [.......... ]Combining metabolite lists: 70% [............................ ]test
90% [.................................... ]

modelHM.gender = gender;

modelHM.ID = InputData(1,ID);

Let's have look at the models that we just created. Note that all non-zero entry in the stoichiometric matrix are shown as blue dots.

figure;

subplot(1,3,1)

if strcmp(gender,'male')

spy(male.A)

elseif strcmp(gender,'female')

spy(female.A)

end

title('Whole-body model')

subplot(1,3,2)

spy(microbiota\_model.A)

title('Microbiota model')

subplot(1,3,3)

spy(modelHM.A)

title('modelHM')

3.10.1.4. Personalize the host-microbiota model

Note that the personalization described in the following section does not require the presence of the microbiota but can also be done on the vanilla male and female model.

Based on the metadata, we personalize the IndividualParameters.

Assumptions:

- We assume that the heart rate refers to the resting (sitting up) heart rate.

Blood volume estimation

The blood volume (BV) of the personalized model is calculated based on the Nadler's equations (59):

- Male: BV = 0.3669 \* Ht^3 + 0.03219 \* Wt + 0.6041
- Female: BV = 0.3561 \* Ht^3 + 0.03308 \* Wt + 0.1833
- Note: Ht = height in meters, Wt = body weight in kg, BV = blood volume in ml

Cardiac output estimation

We implemented different ways of estimating the cardiac output (CO). The resulting CO is given in ml/min

- Based on heart rate and stroke volume: CardiacOutput = HeartRate \* StrokeVolume. (optionCardiacOutput = 1)
- Assume that cardiac output = blood volume. (optionCardiacOutput = 2)
- Based on the polynomial suggested by Youndg et al [http://www.ams.sunysb.edu/~hahn/psfile/pap\_obesity.pdf]: CardiacOutput = 9119-exp(9.164-2.91e-2\*Wt+3.91e-4\*Wt^2-1.91e-6\*Wt^3); Wt = weight in kg (optionCardiacOutput = 0)
- Based on Fick's principle, which requires that the oxygen consumption rate is known: VO\_2 = (CO\* C\_a) - (CO \*C\_v); where CO = Cardiac Output, Ca = Oxygen concentration of arterial blood and Cv = Oxygen concentration of mixed venous blood. We assume that C\_a is 200 ml O2/L and C\_v is 150 ml O2/L. (optionCardiacOutput = 3)
- Based on Fick's principle, while estimating the VO\_2 based on the body surface area (BSA): We use the Du Bois formula (60, 61): BSA=0.007184 \* Wt^0.425\* Ht^0.725; Wt = weight in kg; Ht in cm; (optionCardiacOutput = 4)

In this study, we applied optionCardiacOutput = 1 using the default stroke volume of SV = 80 ml/beat.

% load standard physiological data, depending on the sex

gender = female;

standardPhysiolDefaultParameters;

% set personalized constraints as given by the input data

Input = [InputData(:,1) InputData(:,2) InputData(:,ID)]

Input =

'ID' ' ' 'SRS011239'
'Gender' ' ' 'Female'
'Weight' 'kg' [ 71.5000]
'Height' 'cm' [ 171.2000]
'BMI' ' ' [ 24]
'Age' ' ' [ 27]
'Diet' ' ' '01-Meat/fish/poultry at least three days per week'
'HeartRate' ' ' [ 77]

[modelHM,IndividualParametersPersonalized] = individualizedLabReport(modelHM,IndividualParameters, Input);

IndividualParametersPersonalized

IndividualParametersPersonalized =

OrgansWeights: {53×3 cell}
ID: {'SRS011239'}
bodyWeight: 71.5000
Height: 171.2000
gender: 'female'
HeartRate: 77
StrokeVolume: 80
CardiacOutput: 6160
Hematocrit: 0.4000
MConUrCreatinineMax: 1.2000
MConUrCreatinineMin: 0.5000
MConDefaultBc: 20
MConDefaultCSF: 20
MConDefaultUrMax: 20
MConDefaultUrMin: 0
CSFFlowRate: 0.3500
CSFBloodFlowRate: 0.5200
UrFlowRate: 2000
GlomerularFiltrationRate: 90
bloodFlowData: {32×8 cell}
bloodFlowRow: 2
bloodFlowPercCol: [4 6]
bloodFlowOrganCol: 1
age: 27
BloodVolume: 4.3354e+03
CardiacOutput\_Note: 'Calculated from default StrokeVolume and heart rate'

modelHM.IndividualParametersPersonalized = IndividualParametersPersonalized;

3.10.1.5. Calculate the organ percentage based on weight and height

We update the stoichiometric coefficients of whole-body biomass reaction based on a person's estimated body composition estimated using published polynomials, as proposed by (62). We use the polynomials to compute thus the organ weight fraction and adjust all other organs, for which no data is available, accordingly, while maintaining the numbers provided for the reference man/woman.

Male:

- Brain: 1.41e-01 + (-5.54e-06 \* Wt) + 9.30e-11 \* Wt^2 + (-6.83e-16 \* Wt^3) + 1.80e-21 \* Wt^4 + 0.0 \* Wt^5;
- Heart: 6.32e-03 + (-1.67e-08 \* Wt) + 0.0 \* Wt^2 + 0.0 \* Wt^3 + 0.0 \* Wt^4 + 0.0 \* Wt^5;
- Kidney: 7.26e-03 + (-6.69e-08 \* Wt) + 3.33e-13 \* Wt^2 + 0.0 \* Wt^3 + 0.0 \* Wt^4 + 0.0 \* Wt^5;
- Liver: 4.25e-02 + (-1.01e-06 \* Wt) + 1.99e-11 \* Wt^2 + (-1.66e-16 \* Wt^3) + 4.83e-22 \* Wt^4 + 0.0 \* Wt^5;
- Lungs: 1.86e-02 + (-4.55e-08 \* Wt) + 0.0 \* Wt^2 + 0.0 \* Wt^3 + 0.0 \* Wt^4 + 0.0 \* Wt^5;
- Spleen: 3.12e-03 + (-5.57e-09 \* Wt) + 0.0 \* Wt^2 + 0.0 \* Wt^3 + 0.0 \* Wt^4 + 0.0 \* Wt^5;
- Agland: 8.04e-04 + (-1.98e-08 \* Wt) + 2.01e-13 \* Wt^2 + (-6.11e-19 \* Wt^3) + 0.0 \* Wt^4 + 0.0 \* Wt^5;
- Pancreas: 1.48e-03 + 0.0 \* Wt + 0.0 \* Wt^2 + 0.0 \* Wt^3 + 0.0 \* Wt^4 + 0.0 \* Wt^5;
- Thymus: 3.70e-03 + (-1.05e-07 \* Wt) + 7.94e-13 \* Wt^2 + 0.0 \* Wt^3 + 0.0 \* Wt^4 + 0.0 \* Wt^5;
- Thyroidgland: 2.42e-04 + 0.0 \* Wt + 0.0 \* Wt^2 + 0.0 \* Wt^3 + 0.0 \* Wt^4 + 0.0 \* Wt^5;
- Adipocytes: 1.61e-01 + (-3.59e-06 \* Wt) + 8.28e-11 \* Wt^2 + (-3.57e-16 \* Wt^3) + 4.73e-22 \* Wt^4 + 0.0 \* Wt^5;
- Muscle: 9.68e-02 + (-3.32e-06 \* Wt) + 1.83e-10 \* Wt^2 + (-1.24e-15 \* Wt^3) + 0.0 \* Wt^4 + 0.0 \* Wt^5;
- Skin: 1.03e-01 + (-2.56e-06 \* Wt) + 3.68e-11 \* Wt^2 + (-2.58e-16 \* Wt^3) + 8.62e-22 \* Wt^4 + (-1.10e-27 \* Wt^5);
- Blood: 8.97e-02 + (-3.50e-07 \* Wt) + 6.54e-13 \* Wt^2 + 0.0 \* Wt^3 + 0.0 \* Wt^4 + 0.0 \* Wt^5;

Female:

- Brain: 1.12e-01 + (-3.33e-06 \* Wt) + 4.30e-11 \* Wt^2 + (-2.45e-16 \* Wt^3) + 5.03e-22 \* Wt^4 + 0.0 \* Wt^5;
- Heart: 5.40e-03 + (-1.07e-08 \* Wt) + 0.0 \* Wt^2 + 0.0 \* Wt^3 + 0.0 \* Wt^4 + 0.0 \* Wt^5;
- Kidney: 7.56e-03 + (-5.58e-08 \* Wt) + 1.54e-13 \* Wt^2 + 0.0 \* Wt^3 + 0.0 \* Wt^4 + 0.0 \* Wt^5;
- Liver: 3.34e-02 + (-1.89e-07 \* Wt) + 5.34e-13 \* Wt^2 + 0.0 \* Wt^3 + 0.0 \* Wt^4 + 0.0 \* Wt^5;
- Lungs: 1.89e-02 + (-5.94e-08 \* Wt) + 0.0 \* Wt^2 + 0.0 \* Wt^3 + 0.0 \* Wt^4 + 0.0 \* Wt^5;
- Spleen: 2.96e-03 + (-7.72e-09 \* Wt) + 0.0 \* Wt^2 + 0.0 \* Wt^3 + 0.0 \* Wt^4 + 0.0 \* Wt^5;
- Agland: 8.04e-04 + (-1.98e-08 \* Wt) + 2.01e-13 \* Wt^2 + (-6.11e-19 \* Wt^3) + 0.0 \* Wt^4 + 0.0 \* Wt^5;
- Pancreas: 1.48e-03 + 0.0 \* Wt + 0.0 \* Wt^2 + 0.0 \* Wt^3 + 0.0 \* Wt^4 + 0.0 \* Wt^5;
- Thymus: 3.70e-03 + (-1.05e-07 \* Wt) + 7.94e-13 \* Wt^2 + 0.0 \* Wt^3 + 0.0 \* Wt^4 + 0.0 \* Wt^5;
- Thyroidgland: 2.42e-04 + 0.0 \* Wt + 0.0 \* Wt^2 + 0.0 \* Wt^3 + 0.0 \* Wt^4 + 0.0 \* Wt^5;
- Adipocytes: 1.84e-01 + (-6.86e-06 \* Wt) + 2.46e-10 \* Wt^2 + (-2.11e-15 \* Wt^3) + 7.58e-21 \* Wt^4 + (-9.94e-27 \* Wt^5);
- Muscle: 3.65e-02 + 7.91e-06 \* Wt + (-5.74e-11 \* Wt^2) + 0.0 \* Wt^3 + 0.0 \* Wt^4 + 0.0 \* Wt^5;
- Skin: 9.81e-02 + (-2.28e-06 \* Wt) + 2.74e-11 \* Wt^2 + (-1.58e-16 \* Wt^3) + 4.30e-22 \* Wt^4 + (-4.43e-28 \* Wt^5);
- Blood: 8.97e-02 + (-3.50e-07 \* Wt) + 6.54e-13 \* Wt^2 + 0.0 \* Wt^3 + 0.0 \* Wt^4 + 0.0 \* Wt^5;
- Breast: 0.01 + 0.0 \* Wt + 0.0 \* Wt^2 + 0.0 \* Wt^3 + 0.0 \* Wt^4 + 0.0 \* Wt^5;

Blood cells

Use the estimated blood weight to determine the fraction of the different blood cells.

- White blood cells (WBCs) make about 1% of blood (21) for numbers: WBCWeight = 0.01\*BloodWeight;
- Lymphocytes represnt about 15-40% of the white blood cells (21) for numbers. We assumed 30% of WBC weight: LympWeight = 0.3\*WBCWeight;
- Bcells represent about 9% of the lymphocytes (23): BcellsWeight = 0.09\*LympWeight;
- CD4Tcells represent 45-75% of the lymphocytes or 4 – 20% of leukocyte (24). We assumed 15% of leukocytes: CD4TcellsWeight = 0.15\*WBCWeight;
- Human and mouse NK cells constitute approximately 15% of all circulating lymphocytes (21) for numbers]: NkcellsWeight = 0.15\*LympWeight;
- Monocytes represent about 2-8% of the peripheral WBCs (24). We assumed 5%: MonocyteWeight = 0.05\*WBCWeight;
- There are about 150,000 to 400,000 platelets/mm3; 1mm3 = 1e-6 l; 10 pg per platelet (wet weight) (25) for weight; (26). We assumed 400k platelets/mm3; corresponding to 4g platelets/l blood; PlateletWeight= 4\*BloodVolume/1000;
- The normal range of red blood cells (RBCs) in men is approximately 4.7 to 6.1 million cells/ul. The normal range in women range from 4.2 to 5.4 million cells/ul, according to National Institutes of Health data. 27 pg dry weight of one RBC (27) for numbers; (28) for dry weight;]. We assumed 70% water in RBCs, hence 90 pg/RBC corresponding to 495g/l: RBCWeigth = 495\*BloodVolume/1000; %495g/l

We took the computed cardiac output to estimate the blood volume for the calculations of the blood cell fractions.

[listOrgan,OrganWeight,OrganWeightFract] = calcOrganFract(modelHM,IndividualParametersPersonalized);

% adjust whole body maintenance reaction based on new organ weight

% fractions

[modelHM] = adjustWholeBodyRxnCoeff(modelHM, listOrgan, OrganWeightFract);

Whole\_body\_objective\_rxn 35.939349 Adipocytes\_biomass\_maintenance\_dummy\_objective + 0.019253 Agland\_biomass\_maintenance\_dummy\_objective + 1.732387 Brain\_biomass\_maintenance\_dummy\_objective + Breast\_biomass\_maintenance\_dummy\_objective + 0.405594 Colon\_biomass\_maintenance\_dummy\_objective + 0.463495 Heart\_biomass\_maintenance\_dummy\_objective + 0.435759 Kidney\_biomass\_maintenance\_dummy\_objective + 2.261644 Liver\_biomass\_maintenance\_dummy\_objective + 0.749650 Lung\_biomass\_maintenance\_dummy\_objective + 30.862185 Muscle\_biomass\_maintenance\_dummy\_objective + 0.015385 Ovary\_biomass\_maintenance\_dummy\_objective + 0.148000 Pancreas\_biomass\_maintenance\_dummy\_objective + 0.000196 Pthyroidgland\_biomass\_maintenance\_dummy\_objective + 0.000456 Retina\_biomass\_maintenance\_dummy\_objective + 0.039161 Scord\_biomass\_maintenance\_dummy\_objective + 0.839161 sIEC\_biomass\_reactionIEC01b\_dummy\_objective + 2.781284 Skin\_biomass\_maintenance\_dummy\_objective + 0.240802 Spleen\_biomass\_maintenance\_dummy\_objective + 0.195804 Stomach\_biomass\_maintenance\_dummy\_objective + 0.024200 Thyroidgland\_biomass\_maintenance\_dummy\_objective + 0.062937 Urinarybladder\_biomass\_maintenance\_dummy\_objective + 0.111888 Uterus\_biomass\_maintenance\_dummy\_objective + 0.001836 Bcells\_biomass\_maintenance\_dummy\_objective + 0.010203 CD4Tcells\_biomass\_maintenance\_dummy\_objective + 0.003061 Nkcells\_biomass\_maintenance\_dummy\_objective + 0.003401 Monocyte\_biomass\_maintenance\_dummy\_objective + 0.025897 Platelet\_biomass\_maintenance\_noTrTr\_dummy\_objective + 3.204751 RBC\_biomass\_maintenance\_noTrTr\_dummy\_objective + 0.069930 Cervix\_biomass\_maintenance\_dummy\_objective + 0.011189 Gall\_biomass\_maintenance\_dummy\_objective ->

Please note that at this stage, we still assume the same blood flow distribution for each organ as in the reference man and woman (at rest).

% apply HMDB metabolomic data based on personalized individual parameters

modelHM = physiologicalConstraintsHMDBbased(modelHM,IndividualParametersPersonalized);

Set simulation specific constraints, store personalized data, and enforce that the whole-body metabolic model excretes feces ones a day.

% set some more constraints

modelHM = setSimulationConstraints(modelHM);

modelHM.status = 'personalized microbiota, personalized Harvey/Harvetta';

modelHM.InputData = [InputData(:,1) InputData(:,2) InputData(:,ID)];

% require Brain\_dm\_atp\_c\_ to be at least 5000 mmol/day/person

modelHM = changeRxnBounds(modelHM,'Brain\_DM\_atp\_c\_',5000,'l');

% set microbial excretion constraint

modelHM.lb(find(ismember(modelHM.rxns,...

'Excretion\_EX\_microbiota\_LI\_biomass[fe]')))=1; %

modelHM.ub(find(ismember(modelHM.rxns,...

'Excretion\_EX\_microbiota\_LI\_biomass[fe]')))=1; %

3.10.2. Compute different metabolic objectives with the personalized host-microbiome model

The following reactions have been used as objective functions. Note that the slack\_ refers to the coupling constraints that we mentioned above.

Rxn = {'Liver\_PCSF';

'Liver\_ALCD2if';

'Liver\_EX\_val\_L(e)\_[bc]'

'Liver\_EX\_ile\_L(e)\_[bc]'

'Colon\_HMR\_0156'

% neurotransmitter

'Brain\_DM\_dopa[c]';

'Brain\_DM\_srtn[c]';

'Brain\_DM\_adrnl[c]'

'Brain\_DM\_4abut[c]'

'Brain\_DM\_hista[c]'

'Brain\_DM\_kynate[c]'

'Brain\_DM\_nrpphr[c]'

'Brain\_DM\_Lkynr[c]'

'Muscle\_DM\_atp\_c\_'

'Adipocytes\_DM\_lipid\_storage'

};

printRxnFormula(modelHM,Rxn);

Liver\_PCSF Liver\_paps[c] + Liver\_pcresol[c] -> Liver\_pap[c] + Liver\_pcs[c] + slack\_Liver\_PCSF
Liver\_ALCD2if Liver\_nad[c] + Liver\_etoh[c] -> Liver\_h[c] + Liver\_nadh[c] + Liver\_acald[c] + slack\_Liver\_ALCD2if
Liver\_EX\_val\_L(e)\_[bc] Liver\_val\_L[e] <=> val\_L[bc] + slack\_Liver\_EX\_val\_L(e)\_[bc] + slack\_Liver\_EX\_val\_L(e)\_[bc]\_R
Liver\_EX\_ile\_L(e)\_[bc] Liver\_ile\_L[e] <=> ile\_L[bc] + slack\_Liver\_EX\_ile\_L(e)\_[bc] + slack\_Liver\_EX\_ile\_L(e)\_[bc]\_R
Colon\_HMR\_0156 Colon\_atp[c] + Colon\_coa[c] + Colon\_but[c] -> Colon\_amp[c] + Colon\_ppi[c] + Colon\_btcoa[c] + slack\_Colon\_HMR\_0156
Brain\_DM\_dopa[c] Brain\_dopa[c] -> slack\_Brain\_DM\_dopa[c]
Brain\_DM\_srtn[c] Brain\_srtn[c] -> slack\_Brain\_DM\_srtn[c]
Brain\_DM\_adrnl[c] Brain\_adrnl[c] -> slack\_Brain\_DM\_adrnl[c]
Brain\_DM\_4abut[c] Brain\_4abut[c] -> slack\_Brain\_DM\_4abut[c]
Brain\_DM\_hista[c] Brain\_hista[c] -> slack\_Brain\_DM\_hista[c]
Brain\_DM\_kynate[c] Brain\_kynate[c] -> slack\_Brain\_DM\_kynate[c]
Brain\_DM\_nrpphr[c] Brain\_nrpphr[c] -> slack\_Brain\_DM\_nrpphr[c]
Brain\_DM\_Lkynr[c] Brain\_Lkynr[c] -> slack\_Brain\_DM\_Lkynr[c]
Muscle\_DM\_atp\_c\_ Muscle\_h2o[c] + Muscle\_atp[c] -> Muscle\_h[c] + Muscle\_adp[c] + Muscle\_pi[c] + slack\_Muscle\_DM\_atp\_c\_
Adipocytes\_DM\_lipid\_storage Adipocytes\_tag\_hs[c] -> slack\_Adipocytes\_DM\_lipid\_storage

% run simulations for host-microbiome model

for k = 1 : length(Rxn)

modelHM = changeObjective(modelHM,Rxn{k});

modelHM.osense = -1;%max

tic;[sol]=solveCobraLPCPLEX(modelHM,0,0,0,[],0,...

LPSolver);toc

RxnF(k,1) = sol.full(find(ismember(modelHM.rxns,Rxn{k})));

% interrupt simulations when infeasible

if isempty(sol.full) || sol.origStat==3

break

end

end

Elapsed time is 71.637102 seconds.

Elapsed time is 53.901493 seconds.

Elapsed time is 93.567342 seconds.

Elapsed time is 71.538267 seconds.

Elapsed time is 80.830747 seconds.

Elapsed time is 91.697452 seconds.

Elapsed time is 62.585703 seconds.

Elapsed time is 47.751517 seconds.

Elapsed time is 56.509764 seconds.

Elapsed time is 112.160231 seconds.

Elapsed time is 46.081721 seconds.

Elapsed time is 61.723962 seconds.

Elapsed time is 68.874753 seconds.

Elapsed time is 213.039127 seconds.

Elapsed time is 88.519199 seconds.

% define germ-free version by disallowing the excretion of microbes

modelHM\_GF = modelHM;

modelHM\_GF.lb(find(ismember(modelHM\_GF.rxns,...

'Excretion\_EX\_microbiota\_LI\_biomass[fe]')))=0; %

modelHM\_GF.ub(find(ismember(modelHM\_GF.rxns,...

'Excretion\_EX\_microbiota\_LI\_biomass[fe]')))=0; %

for k = 1 : length(Rxn)

modelHM\_GF = changeObjective(modelHM\_GF,Rxn{k});

modelHM\_GF.osense = -1;%max

tic;[sol]=solveCobraLPCPLEX(modelHM\_GF,0,0,0,[],0,...

LPSolver);toc

RxnF\_GF(k,1) = sol.full(find(ismember(modelHM\_GF.rxns,Rxn{k})));

if isempty(sol.full) || sol.origStat==3

break

end

end

Elapsed time is 102.952274 seconds.

Elapsed time is 64.591528 seconds.

Elapsed time is 79.306190 seconds.

Elapsed time is 75.066039 seconds.

Elapsed time is 68.112290 seconds.

Elapsed time is 72.214991 seconds.

Elapsed time is 89.616448 seconds.

Elapsed time is 81.033622 seconds.

Elapsed time is 111.334201 seconds.

Elapsed time is 127.510938 seconds.

Elapsed time is 83.858025 seconds.

Elapsed time is 81.980986 seconds.

Elapsed time is 64.552943 seconds.

Elapsed time is 106.577637 seconds.

Elapsed time is 90.310430 seconds.

% print Results

[RxnF RxnF\_GF RxnF./RxnF\_GF]

ans =

1.0e+03 \*
0.0000 0.0000 0.0010
1.8873 0.2822 0.0067
1.2262 0.2473 0.0050
0.1200 0.0722 0.0017
1.8074 0.0320 0.0565
0.1948 0.0440 0.0044
0.0918 0.0058 0.0159
0.1435 0.0440 0.0033
1.2821 0.9917 0.0013
0.1079 0.0610 0.0018

% clear up workspace

clear microbiota\_model G h h2o i ID k m male 2 maxInf minConstraints maxConstraints minInf Numbers pi R production solution sol set suplot1 vf vr y z a a0 a1 A\_\* adp ans atp axes1 b bar1 bar2 BC\_\* BD\_\* cnt D D\_\* f EX EX\_D figure1 M\_\* U\_\* U violinPlots

clear BCM\_MY BM BMatp BMatpSum BMmets BPmets BP\_mets\* Diseases Energy\_BM\* F FE\_\*

3.10.3. Computation of strain-level contributions to amino acids

To determine the microbial contribution to neurotransmitters and their amino acid precursors, the strain-level contributions to GABA, L-glutamate, histamine, L-histidine, L-tryptophan, and L-tyrosine were computed for each microbiome as follows: First, the corresponding internal exchange reaction IDs were retrieved for each strain in the microbiome model with an exchange reaction for the corresponding amino acid. Second, the minimal and maximal fluxes in mmol \* g dry weight-1 \* hr-1 through the retrieved internal exchange reactions were determined using distributed flux balance analysis (Heirendt ref) using the IBM CPLEX solver (IBM, Inc.) through the CPLEX interface for Julia. Finally, the absolute minimal fluxes through the internal exchanges for the six amino acids were retrieved in each microbiome for all strains that could secrete the metabolite in at least one microbiome.

3.10.4. Statistical analysis

For the 11 metabolic functions, the fold changes in maximal flux between microbiome-associated and germfree WBM model were calculated for the 149 individuals. The Spearman correlation between fold changes and species abundances was calculated for each metabolic function using the Microbiome Modeling Toolbox (67) function correlateFluxWithTaxonAbundance.

In the next step, the maximal fluxes in the brain neurotransmitter production between the microbiome-associated and germfree models were compared via paired t-tests, and the corresponding p-values were corrected for multiple testing using Bonferoni correction. Then, for liver sulfotransferase and liver alcohol dehydrogenase, the pattern of association between maximal flux and bacteroidia/clostridia abundance was investigated graphically, indicating non-linear relations. Thus, multivariable fractional polynomials (68) were applied, revealing (invers) hyperbolic functions to improve strongly on the linear fit as revealed by likelihood ratio tests. Finally, the best fitting transformation was extracted and used for graphical presentation. These steps were performed within STATA 14/MP.

3.11. References:

0. L. Heirendt et al., Creation and analysis of biochemical constraint-based models: the COBRA Toolbox v3.0., Nature Protocols (accepted), arXiv preprint, (2018).

1. D. S. Wishart et al., HMDB 3.0--The Human Metabolome Database in 2013. Nucleic Acids Res 41, D801-807 (2013).

2. Book: W. S. Snyder et al., Report on the Task Group on Reference Man. INTERNATIONAL COMMISSION ON RADIOLOGICAL PROTECTION No 23 (1975).

3. A. Sheikhazadi et al., Study of the normal internal organ weights in Tehran's population. Journal of forensic and legal medicine 17, 78-83 (2010).

4. D. K. Molina, V. J. DiMaio, Normal organ weights in men: part II-the brain, lungs, liver, spleen, and kidneys. The American journal of forensic medicine and pathology 33, 368-372 (2012).

5. V. L. Katch et al., Contribution of breast volume and weight to body fat distribution in females. Am J Phys Anthropol 53, 93-100 (1980).

6. S. Sahoo, I. Thiele, Predicting the impact of diet and enzymopathies on human small intestinal epithelial cells. Hum Mol Genet 22, 2705-2722 (2013).

7. M. F. Paine et al., Characterization of interintestinal and intraintestinal variations in human CYP3A-dependent metabolism. J Pharmacol Exp Ther 283, 1552-1562 (1997).

8. E. Levin, S. Muravchick, M. I. Gold, Density of normal human cerebrospinal fluid and tetracaine solutions. Anesthesia and analgesia 60, 814-817 (1981).

9. D. K. Molina, V. J. DiMaio, Normal organ weights in men: part I-the heart. The American journal of forensic medicine and pathology 33, 362-367 (2012).

10. I. Janssen, S. B. Heymsfield, Z. M. Wang, R. Ross, Skeletal muscle mass and distribution in 468 men and women aged 18-88 yr. Journal of applied physiology 89, 81-88 (2000).

11. G. Raghunath, V. S. Vijayalakshmi, A study on the Morphology and the Morphometry of the Human Placenta and its Clinical Relevance in a population in Tamilnadu. Journal of Clinical and Diagnostic Research 5, 282-286 (2011).

12. R. L. Balihallimath, V. S. Shirol, A. M. Gan, N. K. Tyagi, M. R. Bandankar, Placental morphometry determines the birth weight. Journal of clinical and diagnostic research : JCDR 7, 2428-2431 (2013).

13. S. J. Johnson, E. A. Sheffield, A. M. McNicol, Best practice no 183. Examination of parathyroid gland specimens. J Clin Pathol 58, 338-342 (2005).

14. G. T. Feke et al., Blood flow in the normal human retina. Invest Ophthalmol Vis Sci 30, 58-65 (1989).

15. .https://faculty.washington.edu/chudler/facts.html - spinal

16. A. Csendes, A. M. Burgos, Size, volume and weight of the stomach in patients with morbid obesity compared to controls. Obes Surg 15, 1133-1136 (2005).

17. V. Chalana, S. Dudycha, J. T. Yuk, G. McMorrow, Automatic Measurement of Ultrasound-Estimated Bladder Weight (UEBW) from Three-Dimensional Ultrasound. Reviews in urology 7 Suppl 6, S22-28 (2005).

18. .http://www.continence.org.au/pages/bladder-training.html

19. .http://www.hematology.org/Patients/Basics/

20. Book: J. Feher, Quantitative Human Physiology. (Elsevier, USA, ed. 1st, 2012).

21. .http://www.fpnotebook.com/HemeOnc/Lab/WhtBldCl.htm

22. .http://www.fpnotebook.com/hemeonc/lab/lymphcytcnt.htm - fpnContent-panel-id\_1

23. H. Morbach, E. M. Eichhorn, J. G. Liese, H. J. Girschick, Reference values for B cell subpopulations from infancy to adulthood. Clin Exp Immunol 162, 271-279 (2010).

24. Report: S. C. Technologies, "Frequencies of cell types in human peripheral blood."

25. J. Kiem et al., Elemental composition of platelets. Part II. Water content of normal human platelets and measurements of their concentrations of Cu, Fe, K, and Zn by neutron activation analysis. Clin Chem 25, 705-710 (1979).

26. .http://www.fpnotebook.com/HemeOnc/Lab/PltltCnt.htm

27. .http://www.medicinenet.com/polycythemia\_high\_red\_blood\_cell\_count/page2.htm

28. K. G. Phillips, S. L. Jacques, O. J. McCarty, Measurement of single cell refractive index, dry mass, volume, and density using a transillumination microscope. Physical review letters 109, 118105 (2012).

29. .http://www.nih.gov/news-events/news-releases/nih-human-microbiome-project-defines-normal-bacterial-makeup-body

30. .http://www.arn.org/docs/glicksman/eyw\_1301.htm

31. P. S. Price et al., Modeling interindividual variation in physiological factors used in PBPK models of humans. Crit Rev Toxicol 33, 469-503 (2003).

32. Book: F. H. Martini, E. F. Bartholomew, Essentials of Anatomy & Physiology. (Pearson, San Francisco, CA, USA, ed. 7th, 2006), pp. 808.

33. .http://biology.stackexchange.com/questions/5642/how-much-gas-is-exchanged-in-one-human-breath

34. .https://en.wikipedia.org/wiki/Breathing - Breathing\_in\_gas

35. .http://cozybeehive.blogspot.lu/2010/03/how-much-co2-do-you-exhale-while.html

36. K. N. Frayn, Ed., Metabolic Regulation: A Human Perspective, (Wiley-Blackwell, United Kingdom, 2010).

37. A. Schurr, in The Neuronal Environment: Brain Homeostasis in Health and Disease, W. Walz, Ed. (Humana Press, Totowa, NJ, 2002), pp. 25-54.

38. S. Berl, G. Takagaki, D. D. Clarke, H. Waelsch, Carbon dioxide fixation in the brain. J Biol Chem 237, 2570-2573 (1962).

39. M. L. Friis, O. B. Paulson, M. M. Hertz, Carbon dioxide permeability of the blood-brain barrier in man. The effect of acetazolamide. Microvasc Res 20, 71-80 (1980).

40. Book: J. M. Berg, J. L. Tymoczko, L. Stryer, Biochemistry. (W H Freeman, New York, ed. 5th 2002).

41. .https://heartmdinstitute.com/heart-health/metabolic-cardiology-basics/

42. K. Gatternig, B. Maierhofer.http://www.oenwt.at/

43. A. Noronha, Virtual Metabolic Human database: integration of human and gut microbiome metabolism with nutrition and disease (in preparation).

44. Book: I. Elmadfa, Österreichischer Ernährungsbericht 2012. (Vienna, ed. 1., 2012).

45. N. a. l. USDA: Agricultural research service, Nutrient data laboratory. (2012).http://ndb.nal.usda.gov/ndb/foods/show/3490?fg=&man=&lfacet=&count=&max=25&qlookup=&offset=600&sort=fg&format=Full&\_action\_show=Apply+Changes&Qv=1&Q6410=1.0&Q6411=3

46. Book: H. K. Biesalski, P. Grimm, Pocket Atlas of Nutrition. (Thieme, 2005).

47. A. Bordbar, N. Jamshidi, B. O. Palsson, iAB-RBC-283: A proteomically derived knowledge-base of erythrocyte metabolism that can be used to simulate its physiological and patho-physiological states. BMC systems biology 5, 110 (2011).

48. A. Bordbar et al., A multi-tissue type genome-scale metabolic network for analysis of whole-body systems physiology. BMC systems biology 5, 180 (2011).

49. C. Gille et al., HepatoNet1: a comprehensive metabolic reconstruction of the human hepatocyte for the analysis of liver physiology. Molecular systems biology 6, 411 (2010).

50. R. Milo, P. Jorgensen, U. Moran, G. Weber, M. Springer, BioNumbers--the database of key numbers in molecular and cell biology. Nucleic Acids Res 38, D750-753 (2010).

51. T. Skotland et al., Determining the Turnover of Glycosphingolipid Species by Stable-Isotope Tracer Lipidomics. J Mol Biol 428, 4856-4866 (2016).

52. H. Wackerhage et al., Recovery of free ADP, Pi, and free energy of ATP hydrolysis in human skeletal muscle. Journal of applied physiology 85, 2140-2145 (1998).

53. M. D. Mifflin et al., A new predictive equation for resting energy expenditure in healthy individuals. Am J Clin Nutr 51, 241-247 (1990).

54. J. P. DeLany, D. E. Kelley, K. C. Hames, J. M. Jakicic, B. H. Goodpaster, High energy expenditure masks low physical activity in obesity. International journal of obesity 37, 1006-1011 (2013).

55. .http://didyouknowstuff.blogspot.lu/p/how-many-kilometers-in-10000-steps.html

56. T. H. M. P. Consortium, A framework for human microbiome research. Nature 486, 215-221 (2012).

57. S. Magnusdottir et al., Generation of genome-scale metabolic reconstructions for 773 members of the human gut microbiota. Nat Biotechnol 35, 81-89 (2017).

58. A. Heinken, S. Sahoo, R. M. Fleming, I. Thiele, Systems-level characterization of a host-microbe metabolic symbiosis in the mammalian gut. Gut Microbes 4, 28-40 (2013).

59. Report: "http://www.mc.vanderbilt.edu/documents/vmcpathology/files/TBV caclulation.docx.pdf."

60. D. Du Bois, E. F. Du Bois, A formula to estimate the approximate surface area if height and weight be known. 1916. Nutrition 5, 303-311; discussion 312-303 (1989).

61. J. Verbraecken, P. Van de Heyning, W. De Backer, L. Van Gaal, Body surface area in normal-weight, overweight, and obese adults. A comparison study. Metabolism 55, 515-524 (2006).

62. J. F. Young et al., Human organ/tissue growth algorithms that include obese individuals and black/white population organ weight similarities from autopsy data. J Toxicol Environ Health A 72, 527-540 (2009).

63. A. Heinken et al., Personalized modeling of the human gut microbiome reveals distinct bile acid deconjugation and biotransformation potential in healthy and IBD individuals. bioRxiv, doi: https://doi.org/10.1101/229138.

64. A. M. Prentice et al., High levels of energy expenditure in obese women. Brit Med J, 292: 983-287 (1986).

65. L. L. Loureiro et al., Basal Metabolic Rate of Adolescent Modern Pentathlon Athletes: Agreement between Indirect Calorimetry and Predictive Equations and the Correlation with Body Parameters. PLoS One, 10(11):e0142859 (2015).

66. L. Heirendt et al. , DistributedFBA.jl: High-level, high-performance flux balance analysis in Julia., Bioinformatics, 33(9):1421–1423 (2017),

67. F. Baldini et al., The Microbiome Modeling Toolbox: from microbial interactions to personalized microbial communities, Bioinformatics, (2018).

68. P. Royston and W. Sauerbrei, Multivariable Model-building, Biometrical Journal 51(5):874-875.

  
