# Supplementary material for: Personalized whole‐body models integrate metabolism, physiology, and the gut microbiome
Source: Mol Syst Biol. 2020 May 28;16(5):e8982. doi: 10.15252/msb.20198982 (PMC7285886; doi:10.15252/msb.20198982)
Supplement: Supplementary file 22 — Dataset EV1 [file MSB-16-e8982-s022.zip › PSCM_toolbox/ext/m2html/templates/blue/mfile.tpl]

Description of {NAME}


Home >  {PATHDIR} >  {NAME}.m

# {NAME}

## PURPOSE

**{H1LINE}**

## SYNOPSIS

**{SYNOPSIS}  This is a script file.**

## DESCRIPTION

```
{DESCRIPTION}
```

## CROSS-REFERENCE INFORMATION

This function calls:

- {NAME\_CALL} {H1LINE\_CALL}

This function is called by:

- {NAME\_CALLED} {H1LINE\_CALLED}


## SUBFUNCTIONS

- {SUB}


## DOWNLOAD

{NAME}.m


## SOURCE CODE

```
{SOURCECODE}
```


---

Generated on {DATE} by **m2html** © 2005
