# Supplementary material for: Personalized whole‐body models integrate metabolism, physiology, and the gut microbiome
Source: Mol Syst Biol. 2020 May 28;16(5):e8982. doi: 10.15252/msb.20198982 (PMC7285886; doi:10.15252/msb.20198982)
Supplement: Supplementary file 22 — Dataset EV1 [file MSB-16-e8982-s022.zip › PSCM_toolbox/ext/m2html/templates/frame/master.tpl]

Matlab Index


# Matlab Index

## Matlab Directories

- {DIR}


## Search Engine

 


## Dependency Graph

- View the Graph.


---

Generated by **m2html** © 2005
