# Supplementary material for: Personalized whole‐body models integrate metabolism, physiology, and the gut microbiome
Source: Mol Syst Biol. 2020 May 28;16(5):e8982. doi: 10.15252/msb.20198982 (PMC7285886; doi:10.15252/msb.20198982)
Supplement: Supplementary file 22 — Dataset EV1 [file MSB-16-e8982-s022.zip › PSCM_toolbox/ext/m2html/templates/frame/mdir.tpl]

Index for Directory {MDIR}


 Master index 

# Index for {MDIR}

## Matlab files in this directory:

- {NAME}

## Other Matlab-specific files in this directory:

- {OTHERFILE}


## Subsequent directories:

- {SUBDIRECTORY}


## Dependency Graph

- View the Graph.


## TODO List

- View the TODO list.


---

Generated by **m2html** © 2005
