# Supplementary material for: Personalized whole‐body models integrate metabolism, physiology, and the gut microbiome
Source: Mol Syst Biol. 2020 May 28;16(5):e8982. doi: 10.15252/msb.20198982 (PMC7285886; doi:10.15252/msb.20198982)
Supplement: Supplementary file 22 — Dataset EV1 [file MSB-16-e8982-s022.zip › PSCM_toolbox/ext/m2html/doc/m2html/mwizard2.html]

Description of mwizard2


Home > m2html > mwizard2.m

# mwizard2

## PURPOSE

**MWIZARD - M2HTML Graphical User Interface**

## SYNOPSIS

**function mwizard(file)**

## DESCRIPTION

```
MWIZARD - M2HTML Graphical User Interface
  MWIZARD launches a Matlab GUI front-end to edit parameters
  that are then used by M2HTML to generate HTML documentation.
  MWIZARD(FILE) allows to specify a mat-file FILE from which
  default parameters are extracted and can be updated.  

  For more information, please read the M2HTML tutorial and FAQ at:
    <http://www.artefact.tk/software/matlab/m2html/>

  See also M2HTML
```

## CROSS-REFERENCE INFORMATION

This function calls:

- m2html M2HTML - Documentation Generator for Matlab M-files and Toolboxes in HTML

This function is called by:


## SUBFUNCTIONS

- function h = initWindow
- function buildWindow(h)
- function doClose(fig,evd,h)
- function doNewFile(fig,evd,h)
- function doOpenFile(fig,evd,h)
- function status = doSaveFile(fig,evd,h)
- function status = doSaveAsFile(fig,evd,h)
- function doRunFile(fig,evd,h)
- function status = doCheckSave(h)
- function doHelp(fig,evd,h)
- function varargout = initOptions(h)
- function refreshOptions(h)
- function doInitHTMLDir(fig,evd,h)
- function doInitTpl(fig,evd,h)
- function doInitMfiles(fig,evd,h)
- function doInitGraphs(fig,evd,h)
- function doBrowseButton(fig,evd,h)
- function doSelectMfiles(fig,evd,h)
- function doSetRecursive(fig,evd,h)
- function doSetOutputDir(fig,evd,h)
- function doSetIndex(fig,evd,h)
- function doSetExtension(fig,evd,h)
- function doSetTemplate(fig,evd,h)
- function doSetSource(fig,evd,h)
- function doSetHighlight(fig,evd,h)
- function doSetGraph(fig,evd,h)
- function doSetSearch(fig,evd,h)
- function doSetGlobal(fig,evd,h)
- function doSetDownload(fig,evd,h)
- function doSetTodo(fig,evd,h)
- function doSetVerbose(fig,evd,h)
- function doSetSaveAsMat(fig,evd,h)
- function doSetLoadMat(fig,evd,h)
- function doSetTabs(fig,evd,h)
- function doSetNbColumns(fig,evd,h)
- function text2 = shortenText(text, l)

## SOURCE CODE

```
0001 function mwizard(file)
0002 %MWIZARD - M2HTML Graphical User Interface
0003 %  MWIZARD launches a Matlab GUI front-end to edit parameters
0004 %  that are then used by M2HTML to generate HTML documentation.
0005 %  MWIZARD(FILE) allows to specify a mat-file FILE from which
0006 %  default parameters are extracted and can be updated.
0007 %
0008 %  For more information, please read the M2HTML tutorial and FAQ at:
0009 %    <http://www.artefact.tk/software/matlab/m2html/>
0010 %
0011 %  See also M2HTML
0012 
0013 %  Copyright (C) 2004 Guillaume Flandin <Guillaume@artefact.tk>
0014 %  $Revision: 0.5 $Date: 2004/05/24 20:12:17 $
0015 
0016 %  This program is free software; you can redistribute it and/or
0017 %  modify it under the terms of the GNU General Public License
0018 %  as published by the Free Software Foundation; either version 2
0019 %  of the License, or any later version.
0020 %
0021 %  This program is distributed in the hope that it will be useful,
0022 %  but WITHOUT ANY WARRANTY; without even the implied warranty of
0023 %  MERCHANTABILITY or FITNESS FOR A PARTICULAR PURPOSE.  See the
0024 %  GNU General Public License for more details.
0025 %
0026 %  You should have received a copy of the GNU General Public License
0027 %  along with this program; if not, write to the Free Software
0028 %  Foundation Inc, 59 Temple Pl. - Suite 330, Boston, MA 02111-1307, USA.
0029 
0030 %  Suggestions for improvement and fixes are always welcome, although no
0031 %  guarantee is made whether and when they will be implemented.
0032 %  Send requests to Guillaume@artefact.tk
0033 
0034 error(nargchk(0,1,nargin));
0035 
0036 disp('This is a beta version of mwizard.');
0037 disp('Please use the online version m2html instead.');
0038 
0039 h = initWindow;
0040 
0041 initOptions(h);
0042 
0043 buildWindow(h);
0044 
0045 setappdata(h, 'handles', guihandles(h));
0046 % M. Krauski
0047 % setappdata(h, 'pwd',     pwd);
0048 
0049 if nargin == 0
0050     setappdata(h, 'file', '');
0051     setappdata(h, 'needsave', 1);
0052 else
0053     setappdata(h, 'file', file);
0054     setappdata(h, 'needsave', 0);
0055     opt = load(file, 'options');
0056     setappdata(h, 'options', opt.options);
0057     refreshOptions(h);
0058 end
0059 
0060 set(h, 'HandleVisibility', 'callback');
0061 
0062 %===============================================================================
0063 
0064 function h = initWindow
0065 
0066 h = figure('Resize',      'on',...
0067            'MenuBar',     'none',...
0068            'NumberTitle', 'off',...
0069            'Name',        ':: M2HTML Wizard ::',...
0070            'Position',    [200 200 500 650],...
0071            'Tag',         mfilename);
0072            
0073 set(h, 'CloseRequestFcn', {@doClose,h});
0074 
0075 %===============================================================================
0076 
0077 function buildWindow(h)
0078 
0079 wincolor = struct('bg',    [0.9 0.9 0.9], ...
0080                   'fg',    [0.8 0.8 0.8], ...
0081                   'title', [0.8 0.8 0.9]);
0082 
0083 set(h, 'Color', wincolor.bg);
0084               
0085 %-------------------------------------------------------------------------------
0086 %- Menu
0087 %-------------------------------------------------------------------------------
0088 
0089 icons = load(fullfile(fileparts(which(mfilename)),'private', ...
0090             'm2htmltoolbarimages.mat'));
0091 
0092 uipushtool('CData',icons.newIcon,...
0093     'enable','on',...
0094     'Separator','off',...
0095     'ToolTipString','New File',...
0096     'ClickedCallback',{@doNewFile,h},...
0097     'Tag','NewTool');
0098 
0099 uipushtool('CData',icons.openIcon,...
0100     'enable','on',...
0101     'Separator','off',...
0102     'ToolTipString','Open File',...
0103     'ClickedCallback',{@doOpenFile,h},...
0104     'Tag','OpenTool');
0105 
0106 uipushtool('CData',icons.saveIcon,...
0107     'enable','on',...
0108     'Separator','off',...
0109     'ToolTipString','Save File',...
0110     'ClickedCallback',{@doSaveFile,h},...
0111     'Tag','SaveTool');
0112 
0113 uipushtool('CData',icons.saveAsIcon,...
0114     'enable','on',...
0115     'Separator','off',...
0116     'ToolTipString','Save File As',...
0117     'ClickedCallback',{@doSaveAsFile,h},...
0118     'Tag','SaveAsTool');
0119     
0120 uipushtool('CData',icons.wheelIcon,...
0121     'enable','on',...
0122     'Separator','on',...
0123     'ToolTipString','Save and Run M2HTML',...
0124     'ClickedCallback',{@doRunFile,h},...
0125     'Tag','RunTool');
0126 
0127 uipushtool('CData',icons.webIcon,...
0128     'enable','on',...
0129     'Separator','on',...
0130     'ToolTipString','Online Tutorial',...
0131     'ClickedCallback',...
0132         'web(''http://www.artefact.tk/software/matlab/m2html/'')',...
0133     'Tag','WebTool');
0134 
0135 uipushtool('CData',icons.helpIcon,...
0136     'enable','on',...
0137     'Separator','off',...
0138     'ToolTipString','Help',...
0139     'ClickedCallback',{@doHelp,h},...
0140     'Tag','HelpTool');
0141 
0142 %-------------------------------------------------------------------------------
0143 %- Title
0144 %-------------------------------------------------------------------------------
0145 
0146 uicontrol('Style','Frame',...
0147     'Units','Normalized',...
0148     'Position',[0.02,0.92,0.96,0.06],...
0149     'BackgroundColor',wincolor.title);
0150 
0151 uicontrol('Style','Text',...
0152     'Units','Normalized',...
0153     'String','M2HTML Wizard',...
0154     'FontSize',18,...
0155     'HorizontalAlignment','center',...
0156     'Position',[0.03,0.93,0.94,0.038],...
0157     'BackgroundColor',wincolor.title);
0158 
0159 %-------------------------------------------------------------------------------
0160 %- Input
0161 %-------------------------------------------------------------------------------
0162 
0163 uicontrol('Style','Frame',...
0164     'Units','Normalized',...
0165     'Position',[0.02,0.74,0.96,0.16],...
0166     'BackgroundColor',wincolor.fg);
0167     
0168 uicontrol('Style','Frame',...
0169     'Units','Normalized',...
0170     'HorizontalAlignment','center',...
0171     'Position',[0.02,0.87,0.96,0.03],...
0172     'BackgroundColor',wincolor.title);
0173     
0174 uicontrol('Style','Text',...
0175     'Units','Normalized',...
0176     'String','M-Files Input',...
0177     'HorizontalAlignment','left',...
0178     'Position',[0.03,0.875,0.94,0.02],...
0179     'BackgroundColor',wincolor.title);
0180 
0181 uicontrol('Style','Text',...
0182     'Units','Normalized',...
0183     'String','Root directory:',...
0184     'FontAngle','oblique',...
0185     'HorizontalAlignment','left',...
0186     'Position',[0.04,0.825,0.6,0.03],...
0187     'BackgroundColor',wincolor.fg);
0188 
0189 % M. Krauski 7/20/04
0190 % changed and resized control to work with browse push button
0191 
0192 % uicontrol('Style','edit',...
0193 %     'Units','Normalized',...
0194 %     'Position',[0.21,0.83,0.74,0.03],...
0195 %     'String',pwd,...
0196 %     'Enable','inactive',...
0197 %     'HorizontalAlignment','left',...
0198 %     'Callback','uigetfile;',...%uigetdir
0199 %     'BackgroundColor',wincolor.bg,...
0200 %     'Tag','rootdir');
0201 
0202 uicontrol('Style','edit',...
0203     'Units','Normalized',...
0204     'Position',[0.21,0.83,0.59,0.03],...
0205     'String',pwd,... %this will be over written in refreshOptions
0206     'Enable','inactive',...
0207     'HorizontalAlignment','left',...
0208     'BackgroundColor',wincolor.bg,...
0209     'Tag','rootdir');
0210 
0211 % M. Krauski 7/20/04
0212 % added control
0213 uicontrol('Style','pushbutton',...
0214     'Units','Normalized',...
0215     'Position',[0.81,0.83,0.14,0.03],...
0216     'String','Browse...',...
0217     'HorizontalAlignment','left',...
0218     'Callback',{@doBrowseButton,h},...
0219     'BackgroundColor',wincolor.bg,...
0220     'Tag','browsebutton');
0221 
0222 uicontrol('Style','Text',...
0223     'Units','Normalized',...
0224     'String','Relative pathes:',...
0225     'HorizontalAlignment','left',...
0226     'Position',[0.04,0.785,0.6,0.03],...
0227     'BackgroundColor',wincolor.fg);
0228 
0229 % M. Krauski 7/20/04
0230 % changed and resized control to work with select push button
0231 
0232 % uicontrol('Style','edit',...
0233 %     'Units','Normalized',...
0234 %     'Position',[0.21,0.79,0.74,0.03],...
0235 %     'String','',...
0236 %     'HorizontalAlignment','left',...
0237 %     'Callback',{@doSetMfiles,h},...
0238 %     'CreateFcn',{@doInitMfiles,h},...
0239 %     'BackgroundColor',wincolor.bg,...
0240 %     'Tag','mfiles');
0241 
0242 uicontrol('Style','edit',...
0243     'Units','Normalized',...
0244     'Position',[0.21,0.79,0.59,0.03],...
0245     'String','',...
0246     'Enable','inactive',...
0247     'HorizontalAlignment','left',...
0248     'CreateFcn',{@doInitMfiles,h},...
0249     'BackgroundColor',wincolor.bg,...
0250     'Tag','mfiles');
0251 
0252 uicontrol('Style','pushbutton',...
0253     'Units','Normalized',...
0254     'Position',[0.81,0.79,0.14,0.03],...
0255     'String','Select...',...
0256     'HorizontalAlignment','left',...
0257     'Callback',{@doSelectMfiles,h},...
0258     'BackgroundColor',wincolor.bg,...
0259     'Tag','selectbutton');
0260 
0261 uicontrol('Style','CheckBox',...
0262     'Units','Normalized',...
0263     'Position',[0.04,0.749,0.42,0.032],...
0264     'String',' Recursive',...
0265     'HorizontalAlignment','left',...
0266     'Callback',{@doSetRecursive,h},...
0267     'Value',0,...
0268     'BackgroundColor',wincolor.bg,...
0269     'Tag','recursive');
0270 
0271 %-------------------------------------------------------------------------------
0272 %- Output
0273 %-------------------------------------------------------------------------------
0274 
0275 uicontrol('Style','Frame',...
0276     'Units','Normalized',...
0277     'Position',[0.02, 0.56,0.96,0.16],...
0278     'BackgroundColor',wincolor.fg);
0279 
0280 uicontrol('Style','Frame',...
0281     'Units','Normalized',...
0282     'HorizontalAlignment','center',...
0283     'Position',[0.02,0.69,0.96,0.03],...
0284     'BackgroundColor',wincolor.title);
0285     
0286 uicontrol('Style','Text',...
0287     'Units','Normalized',...
0288     'String','HTML Output',...
0289     'HorizontalAlignment','left',...
0290     'Position',[0.03,0.695,0.94,0.02],...
0291     'BackgroundColor',wincolor.title);
0292 
0293 uicontrol('Style','Text',...
0294     'Units','Normalized',...
0295     'String','Output Directory:',...
0296     'HorizontalAlignment','left',...
0297     'Position',[0.04,0.645,0.6,0.03],...
0298     'BackgroundColor',wincolor.fg);
0299 
0300 uicontrol('Style','edit',...
0301     'Units','Normalized',...
0302     'Position',[0.21,0.65,0.74,0.03],...
0303     'String','',...
0304     'HorizontalAlignment','left',...
0305     'Callback',{@doSetOutputDir,h},...
0306     'CreateFcn',{@doInitHTMLDir,h},...
0307     'BackgroundColor',wincolor.bg,...
0308     'Tag','htmldir');
0309 
0310 uicontrol('Style','Text',...
0311     'Units','Normalized',...
0312     'String','HTML Index:',...
0313     'HorizontalAlignment','left',...
0314     'Position',[0.04,0.605,0.6,0.03],...
0315     'BackgroundColor',wincolor.fg);
0316 
0317 uicontrol('Style','edit',...
0318     'Units','Normalized',...
0319     'Position',[0.21,0.61,0.25,0.03],...
0320     'String','index',...
0321     'HorizontalAlignment','left',...
0322     'Callback',{@doSetIndex,h},...
0323     'BackgroundColor',wincolor.bg,...
0324     'Tag','index');
0325 
0326 uicontrol('Style','Text',...
0327     'Units','Normalized',...
0328     'String','Extension:',...
0329     'HorizontalAlignment','left',...
0330     'Position',[0.53,0.605,0.3,0.03],...
0331     'BackgroundColor',wincolor.fg);
0332 
0333 uicontrol('Style','edit',...
0334     'Units','Normalized',...
0335     'Position',[0.70,0.61,0.25,0.03],...
0336     'String','html',...
0337     'HorizontalAlignment','left',...
0338     'Callback',{@doSetExtension,h},...
0339     'BackgroundColor',wincolor.bg,...
0340     'Tag','extension');
0341 
0342 uicontrol('Style','Text',...
0343     'Units','Normalized',...
0344     'String','Template:',...
0345     'HorizontalAlignment','left',...
0346     'Position',[0.04,0.565,0.3,0.03],...
0347     'BackgroundColor',wincolor.fg);
0348 
0349 uicontrol('Style','popupmenu',...
0350     'Units','Normalized',...
0351     'Position',[0.21,0.57,0.25,0.03],...
0352     'String','',...
0353     'HorizontalAlignment','center',...
0354     'Callback',{@doSetTemplate,h},...
0355     'CreateFcn',{@doInitTpl,h},...
0356     'BackgroundColor',wincolor.bg,...
0357     'Tag','template');
0358 
0359 %-------------------------------------------------------------------------------
0360 %- Other options
0361 %-------------------------------------------------------------------------------
0362 
0363 uicontrol('Style','Frame',...
0364     'Units','Normalized',...
0365     'Position',[0.02,0.24,0.96,0.30],...
0366     'BackgroundColor',wincolor.fg);
0367 
0368 uicontrol('Style','Frame',...
0369     'Units','Normalized',...
0370     'HorizontalAlignment','center',...
0371     'Position',[0.02,0.51,0.96,0.03],...
0372     'BackgroundColor',wincolor.title);
0373     
0374 uicontrol('Style','Text',...
0375     'Units','Normalized',...
0376     'String','Other Options',...
0377     'HorizontalAlignment','left',...
0378     'Position',[0.03,0.515,0.94,0.02],...
0379     'BackgroundColor',wincolor.title);
0380 
0381 uicontrol('Style','checkbox',...
0382     'Units','Normalized',...
0383     'Position',[0.04,0.464,0.42,0.032],...
0384     'String',' Include Source Code',...
0385     'HorizontalAlignment','left',...
0386     'Callback',{@doSetSource,h},...
0387     'Value',1,...
0388     'TooltipString','Include Source Code of each M-file',...
0389     'BackgroundColor',wincolor.bg,...
0390     'Tag','source');
0391 
0392 uicontrol('Style','checkbox',...
0393     'Units','Normalized',...
0394     'Position',[0.53,0.464,0.42,0.032],...
0395     'String',' Syntax Highlighting',...
0396     'HorizontalAlignment','left',...
0397     'Callback',{@doSetHighlight,h},...
0398     'Value',1,...
0399     'TooltipString','Source Code Syntax Highlighting',...
0400     'BackgroundColor',wincolor.bg,...
0401     'Tag','highlight');
0402 
0403 uicontrol('Style','checkbox',...
0404     'Units','Normalized',...
0405     'Position',[0.04,0.42,0.42,0.032],...
0406     'String',' Create Dependency Graphs',...
0407     'HorizontalAlignment','left',...
0408     'Callback',{@doSetGraph,h},...
0409     'CreateFcn',{@doInitGraphs,h},...
0410     'Value',0,...
0411     'TooltipString','Compute a Dependency Graph using GraphViz',...
0412     'BackgroundColor',wincolor.bg,...
0413     'Tag','graph');
0414 
0415 uicontrol('Style','checkbox',...
0416     'Units','Normalized',...
0417     'Position',[0.53,0.42,0.42,0.032],...
0418     'String',' PHP Search Engine',...
0419     'HorizontalAlignment','left',...
0420     'Callback',{@doSetSearch,h},...
0421     'Value',0,...
0422     'TooltipString','Create an Index for a PHP Search Engine',...
0423     'BackgroundColor',wincolor.bg,...
0424     'Tag','search');
0425 
0426 uicontrol('Style','checkbox',...
0427     'Units','Normalized',...
0428     'Position',[0.04,0.378,0.42,0.032],...
0429     'String',' Global Hyperlinks',...
0430     'HorizontalAlignment','left',...
0431     'Callback',{@doSetGlobal,1},...
0432     'Value',0,...
0433     'TooltipString','Hypertext links among separate Matlab Directories',...
0434     'BackgroundColor',wincolor.bg,...
0435     'Tag','globalhypertext');
0436 
0437 uicontrol('Style','checkbox',...
0438     'Units','Normalized',...
0439     'Position',[0.53,0.378,0.42,0.032],...
0440     'String',' Downloadable M-files',...
0441     'HorizontalAlignment','left',...
0442     'Callback',{@doSetDownload,h},...
0443     'TooltipString','Add a link to download each M-file separately',...
0444     'Value',0,...
0445     'BackgroundColor',wincolor.bg,...
0446     'Tag','download');
0447 
0448 uicontrol('Style','checkbox',...
0449     'Units','Normalized',...
0450     'Position',[0.04,0.336,0.42,0.032],...
0451     'String',' To Do List',...
0452     'HorizontalAlignment','left',...
0453     'Callback',{@doSetTodo,h},...
0454     'TooltipString',['Create a TODO list in each directory summarizing'...
0455     ' all the ''% TODO %'' lines found in Matlab code'],...
0456     'Value',0,...
0457     'BackgroundColor',wincolor.bg,...
0458     'Tag','todo');
0459 
0460 uicontrol('Style','checkbox',...
0461     'Units','Normalized',...
0462     'Position',[0.53,0.336,0.42,0.032],...
0463     'String',' Verbose Mode',...
0464     'HorizontalAlignment','left',...
0465     'Callback',{@doSetVerbose,h},...
0466     'TooltipString','Verbose mode',...
0467     'Value',1,...
0468     'BackgroundColor',wincolor.bg,...
0469     'Tag','verbose');
0470 
0471 uicontrol('Style','checkbox',...
0472     'Units','Normalized',...
0473     'Position',[0.04,0.294,0.42,0.032],...
0474     'String',' Save M-files Parsing',...
0475     'HorizontalAlignment','left',...
0476     'Callback',{@doSetSaveAsMat,h},...
0477     'TooltipString',['Save current state after M-files parsing in '...
0478     '''m2html.mat'' in the Output directory'],...
0479     'Value',0,...
0480     'BackgroundColor',wincolor.bg,...
0481     'Tag','save');
0482 
0483 uicontrol('Style','Text',...
0484     'Units','Normalized',...
0485     'String','Load File:',...
0486     'HorizontalAlignment','left',...
0487     'Position',[0.53,0.289,0.3,0.03],...
0488     'BackgroundColor',wincolor.fg);
0489 
0490 uicontrol('Style','edit',...
0491     'Units','Normalized',...
0492     'Position',[0.70,0.294,0.25,0.03],...
0493     'String','',...
0494     'HorizontalAlignment','left',...
0495     'Callback',{@doSetLoadMat,h},...
0496     'TooltipString',['Load a previously saved MAT file '...
0497     'to generate HTML files once again'],...
0498     'BackgroundColor',wincolor.bg,...
0499     'Tag','load');
0500 
0501 uicontrol('Style','Text',...
0502     'Units','Normalized',...
0503     'String','Tabs Length:',...
0504     'HorizontalAlignment','left',...
0505     'Position',[0.04,0.247,0.3,0.03],...
0506     'BackgroundColor',wincolor.fg);
0507 
0508 uicontrol('Style','edit',...
0509     'Units','Normalized',...
0510     'Position',[0.21,0.252,0.25,0.03],...
0511     'String','4',...
0512     'HorizontalAlignment','right',...
0513     'Callback',{@doSetTabs,h},...
0514     'TooltipString',['Replace horizontal tabs in source code '...
0515     'by N white space characters'],...
0516     'BackgroundColor',wincolor.bg,...
0517     'Tag','tabs');
0518 
0519 uicontrol('Style','Text',...
0520     'Units','Normalized',...
0521     'String','Nb Columns:',...
0522     'FontAngle','oblique',...
0523     'HorizontalAlignment','left',...
0524     'Position',[0.53,0.247,0.3,0.03],...
0525     'BackgroundColor',wincolor.fg);
0526 
0527 uicontrol('Style','edit',...
0528     'Units','Normalized',...
0529     'Position',[0.70,0.252,0.25,0.03],...
0530     'String','4',...
0531     'HorizontalAlignment','right',...
0532     'Callback',{@doSetNbColumns,h},...
0533     'TooltipString','Number of columns for M-files output - not available',...
0534     'Enable','inactive',...
0535     'BackgroundColor',wincolor.bg,...
0536     'Tag','column');
0537 
0538 
0539 %-------------------------------------------------------------------------------
0540 %- Space available
0541 %-------------------------------------------------------------------------------
0542 
0543 % uicontrol('Style','Frame',...
0544 %     'Units','Normalized',...
0545 %     'Position',[0.02,0.07,0.96,0.14],...
0546 %     'BackgroundColor',wincolor.fg);
0547 
0548 % simulate a frame using an axes
0549 % http://www.mathworks.com/support/solutions/data/1-15P9E.html
0550 axes('Color',wincolor.fg,...
0551     'XTick',[],'YTick',[],...
0552     'Units','Normalized',...
0553     'Box','on',...
0554     'Position',[0.02,0.07,0.9585,0.14]);
0555 
0556 uicontrol('Style','Frame',...
0557     'Units','Normalized',...
0558     'HorizontalAlignment','center',...
0559     'Position',[0.02,0.19,0.96,0.03],...
0560     'BackgroundColor',wincolor.title);
0561 
0562 uicontrol('Style','Text',...
0563     'Units','Normalized',...
0564     'String','M2HTML status',...
0565     'HorizontalAlignment','left',...
0566     'Position',[0.03,0.195,0.94,0.02],...
0567     'BackgroundColor',wincolor.title);
0568 
0569 uicontrol('Style','Text',...
0570     'Units','Normalized',...
0571     'String','Click on the wheel in the toolbar to launch M2HTML...',...
0572     'HorizontalAlignment','left',... % center
0573     'Position',[0.12,0.135,0.76,0.02],...
0574     'Visible','on',...
0575     'BackgroundColor',wincolor.fg,...
0576     'Tag','textmisc');
0577 
0578 axes('XLim',[0 100],...
0579     'YLim',[0 1],...
0580     'Box','on', ...
0581     'Units','Normalized',...
0582     'Position',[0.07,0.09,0.86,0.03],...
0583     'XTickMode','manual',...
0584     'YTickMode','manual',...
0585     'layer','top',...
0586     'XTick',[],...
0587     'YTick',[],...
0588     'XTickLabelMode','manual',...
0589     'XTickLabel',[],...
0590     'YTickLabelMode','manual',...
0591     'Visible','on',...
0592     'YTickLabel',[],...
0593     'Color',wincolor.bg);
0594 
0595 x = 0; % between 0 and 100
0596 xpatch = [0 x x 0];
0597 ypatch = [0 0 1 1];
0598   
0599 p = patch(xpatch,ypatch,'r',...
0600     'EdgeColor','r',...
0601     'Visible','on',...
0602     'EraseMode','none',...
0603     'Tag','waitbarmisc');
0604   
0605 l = line([100 0 0 100 100], [0 0 1 1 0], ...
0606     'EraseMode','none', ...
0607     'Visible','on',...
0608     'Color',get(gca,'XColor'));
0609   
0610 % for i=10:5:100
0611 %     set(p,'Xdata',[0 i i 0]); pause(0.02);
0612 % end
0613 % set(p,'EraseMode','normal');
0614 % set(p,'Xdata',[0 0 0 0]);
0615 % set(p,'EraseMode','none');
0616 
0617 %-------------------------------------------------------------------------------
0618 %- Footnote
0619 %-------------------------------------------------------------------------------
0620 
0621 uicontrol('Style','Frame',...
0622     'Units','Normalized',...
0623     'Position',[0.02,0.02,0.96,0.03],...
0624     'BackgroundColor',[0.8 0.8 0.9]);
0625 
0626 uicontrol('Style','Text',...
0627     'Units','Normalized',...
0628     'String',['M2HTML � 2004 Guillaume Flandin <Guillaume@artefact.tk>'],...
0629     'HorizontalAlignment','right',...
0630     'Position',[0.03,0.025,0.94,0.02],...
0631     'BackgroundColor',[0.8 0.8 0.9]);
0632 
0633 %===============================================================================
0634 
0635 function doClose(fig,evd,h)
0636     status = doCheckSave(h);
0637     if status
0638         delete(h);
0639     end
0640     
0641 function doNewFile(fig,evd,h)
0642     status = doCheckSave(h);
0643     if status
0644         initOptions(h);
0645         setappdata(h, 'needsave', 1);
0646         % refresh options in GUI...
0647         refreshOptions(h);
0648     end
0649 
0650 function doOpenFile(fig,evd,h)
0651     status = doCheckSave(h);
0652     if status
0653         [filename, pathname] = uigetfile('*.mat','Open File');
0654         if ~(isequal(filename,0)|isequal(pathname,0))
0655             opt = load(fullfile(pathname,filename),'options');
0656             setappdata(h,'options',opt.options);
0657             setappdata(h,'file',fullfile(pathname,filename));
0658         end
0659     end
0660     % refresh options in GUI...
0661     refreshOptions(h);
0662 
0663 function status = doSaveFile(fig,evd,h)
0664     file = getappdata(h,'file');
0665     status = 1;
0666     if isempty(file)
0667         status = doSaveAsFile(fig,evd,h);
0668     else
0669         options = getappdata(h,'options');
0670         save(file, 'options');
0671     end
0672     setappdata(h,'needsave',0);
0673 
0674 function status = doSaveAsFile(fig,evd,h)
0675     opt = getappdata(h,'options');
0676     [filename, pathname] = uiputfile(strcat(opt.rootdir,filesep,'matlab.mat'),...
0677         'Save File as');
0678     if ~(isequal(filename,0)|isequal(pathname,0))
0679         setappdata(h,'file',fullfile(pathname,filename));
0680         status = doSaveFile(fig,evd,h);
0681     else
0682         status = 0;
0683     end
0684 
0685 function doRunFile(fig,evd,h)
0686     status = doSaveFile(fig,evd,h);
0687     if status
0688         opt = getappdata(h,'options');
0689         file = getappdata(h,'file');
0690         r = {'off' 'on'};
0691         % opt could be directly given to m2html (no need for file saving)
0692         % just need to convert on/off using opt.param = r{opt.param+1}
0693         m2html('load',file,'recursive',r{opt.recursive+1});
0694         % 'recursive' is specified to force m2html to parse M-files
0695     end
0696     
0697 function status = doCheckSave(h)
0698     file = getappdata(h,'file');
0699     if isempty(file), file = 'Untitled'; end
0700     needsave = getappdata(h,'needsave');
0701     status = 1;
0702     if needsave
0703         button = questdlg(sprintf('Save changes to %s?',file),...
0704             'Mwizard','Yes','No','Cancel','Yes');
0705         if strcmp(button,'Yes')
0706             status = doSaveFile([],[],h);
0707         elseif strcmp(button,'Cancel')
0708             status = 0;
0709         end
0710     end
0711 
0712 function doHelp(fig,evd,h)
0713     helpdlg(sprintf(['M2HTML by Guillaume Flandin\n'...
0714         'Copyright � 2003-2004\nGuillaume@artefact.tk\n'...
0715         '<http://www.artefact.tk/>']),'M2HTML Wizard');
0716 
0717 %===============================================================================
0718 
0719 %-------------------------------------------------------------------------------
0720 %- Default parameters
0721 %-------------------------------------------------------------------------------
0722 
0723 function varargout = initOptions(h)
0724     options = struct('verbose', 1,...
0725         'mFiles', {{''}},...
0726         'htmlDir', 'doc',...
0727         'recursive', 0,...
0728         'source', 1,...
0729         'download',0,...
0730         'syntaxHighlighting', 1,...
0731         'tabs', 4,...
0732         'globalHypertextLinks', 0,...
0733         'graph', 0,...
0734         'todo', 0,...
0735         'load', 0,...
0736         'save', 0,...
0737         'search', 0,...
0738         'helptocxml', 0,...
0739         'indexFile', 'index',...
0740         'extension', '.html',...
0741         'template', 'blue',...
0742         'rootdir', pwd,...
0743         'ignoredDir', {{'.svn' 'cvs'}}, ...
0744         'language','english');
0745     
0746     if nargin == 1,
0747         setappdata(h,'options',options);
0748     else
0749         varargout{1} = options;    
0750     end
0751 
0752 function refreshOptions(h)
0753     opt = getappdata(h,'options');
0754     handles = getappdata(h,'handles');
0755     
0756     doInitTpl(handles.template,    0, h);
0757     doInitMfiles(handles.mfiles,   0, h);
0758     doInitHTMLDir(handles.htmldir, 0, h)
0759     
0760     set(handles.recursive,       'Value',  opt.recursive);
0761     set(handles.graph,           'Value',  opt.graph); %doInitGraphs(handles.graph,0,h);
0762     set(handles.save,            'Value',  opt.save);
0763     set(handles.verbose,         'Value',  opt.verbose);
0764     set(handles.todo,            'Value',  opt.todo);
0765     set(handles.download,        'Value',  opt.download);
0766     set(handles.search,          'Value',  opt.search);
0767     set(handles.highlight,       'Value',  opt.syntaxHighlighting);
0768     set(handles.source,          'Value',  opt.source);
0769     set(handles.globalhypertext, 'Value',  opt.globalHypertextLinks);
0770     
0771     set(handles.index,           'String', opt.indexFile);
0772     set(handles.extension,       'String', opt.extension(2:end)); %remove the '.'
0773     set(handles.tabs,            'String', num2str(opt.tabs));
0774 %     if ~strcmp(opt.rootdir, pwd)
0775 %         warning('[M2HTML] You should ''cd %s'' before...',opt.rootdir);
0776 %     end
0777     set(handles.rootdir,         'String', opt.rootdir); % need to 'cd' if different...
0778     set(handles.column,          'String', num2str(4)); %- not saved... default here
0779     if ischar(opt.load)
0780         set(handles.load,        'String', opt.load);
0781     else
0782         set(handles.load,        'String', '');
0783     end
0784     
0785     set(handles.textmisc,        'String', ...
0786         'Click on the wheel in the toolbar to launch M2HTML...'); %- not saved... default here
0787     set(handles.waitbarmisc,     'EraseMode','normal');
0788     set(handles.waitbarmisc,     'Xdata',[0 0 0 0]);
0789     set(handles.waitbarmisc,     'EraseMode','none');
0790 
0791 
0792 %-------------------------------------------------------------------------------
0793 %- CreateFcn Callbacks
0794 %-------------------------------------------------------------------------------
0795 
0796 function doInitHTMLDir(fig,evd,h)
0797     opt = getappdata(h,'options');
0798     [path,name] = fileparts(opt.htmlDir);
0799     if isempty(path)
0800         opt.htmlDir = fullfile(opt.rootdir, opt.htmlDir);
0801     end
0802     set(fig,'String',opt.htmlDir);
0803     setappdata(h,'options',opt);
0804 
0805 function doInitTpl(fig,evd,h)
0806     %- problems when templates are still in full format
0807     opt = getappdata(h,'options');
0808     d = dir(fullfile(fileparts(which(mfilename)),'templates'));
0809     d = {d([d.isdir]).name};
0810     d = {d{~ismember(d,{'.' '..'})}};
0811     if ~isempty(d)
0812         tpl = sprintf('%s|',d{:});
0813         set(fig,'String',tpl(1:end-1));
0814         i = strmatch(opt.template,d,'exact');
0815         if ~isempty(i)
0816             set(fig,'Value',i(1));
0817         else
0818             %- where is the default template ?
0819             warning('[M2HTML] Default template ''%s'' not found.',opt.template);
0820             set(fig,'Value',1);
0821             opt.template = d{1};
0822             setappdata(h,'options',opt);
0823             warning('[M2HTML] Using template ''%s'' instead.',opt.template);
0824         end
0825     else
0826         error('[M2HTML] No template found.');
0827     end
0828 
0829  function doInitMfiles(fig,evd,h)
0830     opt = getappdata(h,'options');
0831     if ~isempty(opt.mFiles{1})
0832         s = sprintf('''%s'', ',opt.mFiles{:}); s = s(1:end-2);
0833         set(fig,'String',['{' s '}']);
0834         return;
0835     end
0836     d = dir(opt.rootdir); d = {d([d.isdir]).name};
0837     d = {d{~ismember(d,{'.' '..'})}};
0838     if length(d) == 0
0839         warning('[M2HTML] No subsequent directory found. Check your cwd.');
0840         set(fig,'String',''); %- maybe open a uigetdir ?
0841         opt.mFiles = {''};
0842     elseif length(d) == 1
0843         set(fig,'String',d{1});
0844         opt.mFiles = d;
0845     else
0846         s = sprintf('''%s'', ',d{:}); s = s(1:end-2);
0847         set(fig,'String',['{' s '}']);
0848         opt.mFiles = d;
0849     end
0850     setappdata(h,'options',opt);
0851     
0852 function doInitGraphs(fig,evd,h)
0853     opt = getappdata(h,'options');
0854     [s, w] = system('dot -V');
0855     if s
0856         disp('GraphViz not installed: Generation of dependency graphs desactivated.');
0857         disp('See http://www.graphviz.org/ to get ''dot'' tool.');
0858         set(fig,'FontAngle','Oblique','Enable','inactive');
0859         set(fig,'Value',0);
0860         opt.graph = 0;
0861         setappdata(h,'options',opt);
0862     else
0863         set(fig,'Value',opt.graph);
0864     end
0865 
0866 
0867 %===============================================================================
0868 
0869 %-------------------------------------------------------------------------------
0870 %- M-Files Input Callbacks
0871 %-------------------------------------------------------------------------------
0872 
0873 function doBrowseButton(fig,evd,h)
0874     opt = getappdata(h,'options');
0875     handles = getappdata(h,'handles');
0876     d = uigetdir(pwd,'Select the Root directory');
0877     if ~d, return; end;
0878     opt.rootdir = d;
0879     set(handles.rootdir,'String',d);
0880     
0881     % set mfiles
0882     d = dir(opt.rootdir); d = {d([d.isdir]).name};
0883     d = {d{~ismember(d,{'.' '..'})}};
0884     if length(d) == 0
0885         warning('[M2HTML] No subsequent directory found. Check your cwd.');
0886         set(handles.mfiles,'String','');
0887         opt.mFiles = {''};
0888     elseif length(d) == 1
0889         set(handles.mfiles,'String',d{1});
0890         opt.mFiles = d;
0891     else
0892         s = sprintf('''%s'', ',d{:}); s = s(1:end-2);
0893         set(handles.mfiles,'String',['{' s '}']);
0894         opt.mFiles = d;
0895     end
0896     
0897     % set htmldir
0898     [path,name] = fileparts(opt.htmlDir);
0899     opt.htmlDir = fullfile(opt.rootdir, name);
0900     set(handles.htmldir,'String',opt.htmlDir);
0901     
0902     setappdata(h,'options',opt);
0903         
0904 function doSelectMfiles(fig,evd,h)
0905     opt = getappdata(h,'options');
0906     handles = getappdata(h,'handles');
0907     
0908     d = dir(opt.rootdir); d = {d([d.isdir]).name};
0909     d = {d{~ismember(d,{'.' '..'})}};
0910     
0911     [i,v] = listdlg('ListString',d,...
0912         'PromptString','Select folder(s):',...
0913         'Name',':: M2HTML :: M-files',...
0914         'SelectionMode','multiple');
0915     if v == 1
0916         d = {d{i}};
0917         s = sprintf('''%s'', ',d{:}); s = s(1:end-2);
0918         set(handles.mfiles,'String',['{' s '}']);
0919     end
0920     opt.mFiles = d;
0921 
0922     setappdata(h,'options',opt);
0923 
0924 function doSetRecursive(fig,evd,h)
0925     opt = getappdata(h,'options');
0926     opt.recursive = get(fig,'Value');
0927     setappdata(h,'options',opt);
0928 
0929 %-------------------------------------------------------------------------------
0930 %- HTML Output Callbacks
0931 %-------------------------------------------------------------------------------
0932 
0933 function doSetOutputDir(fig,evd,h)
0934     opt = getappdata(h,'options');
0935     opt.htmlDir = get(fig,'String');
0936     setappdata(h,'options',opt);
0937 
0938 function doSetIndex(fig,evd,h)
0939     opt = getappdata(h,'options');
0940     opt.indexFile = get(fig,'String');
0941     setappdata(h,'options',opt);
0942     
0943 function doSetExtension(fig,evd,h)
0944     opt = getappdata(h,'options');
0945     e = get(fig,'String');
0946     if ~isempty(e) & e(1) ~= '.'
0947         e = ['.' e];
0948     end
0949     opt.extension = e;
0950     setappdata(h,'options',opt);
0951     
0952 function doSetTemplate(fig,evd,h)
0953     opt = getappdata(h,'options');
0954     s = get(fig,'String');
0955     v = get(fig,'Value');
0956     opt.template = deblank(s(v,:));
0957     setappdata(h,'options',opt);
0958 
0959 %-------------------------------------------------------------------------------
0960 %- Options Callbacks
0961 %-------------------------------------------------------------------------------
0962 
0963 function doSetSource(fig,evd,h)
0964     opt = getappdata(h,'options');
0965     opt.source = get(fig,'Value');
0966     setappdata(h,'options',opt);
0967 
0968 function doSetHighlight(fig,evd,h)
0969     opt = getappdata(h,'options');
0970     opt.syntaxHighlighting = get(fig,'Value');
0971     setappdata(h,'options',opt);
0972 
0973 function doSetGraph(fig,evd,h)
0974     opt = getappdata(h,'options');
0975     opt.graph = get(fig,'Value');
0976     setappdata(h,'options',opt);
0977 
0978 function doSetSearch(fig,evd,h)
0979     opt = getappdata(h,'options');
0980     opt.search = get(fig,'Value');
0981     setappdata(h,'options',opt);
0982 
0983 function doSetGlobal(fig,evd,h)
0984     opt = getappdata(h,'options');
0985     opt.globalHypertextLinks = get(fig,'Value');
0986     setappdata(h,'options',opt);
0987 
0988 function doSetDownload(fig,evd,h)
0989     opt = getappdata(h,'options');
0990     opt.download = get(fig,'Value');
0991     setappdata(h,'options',opt);
0992 
0993 function doSetTodo(fig,evd,h)
0994     opt = getappdata(h,'options');
0995     opt.todo = get(fig,'Value');
0996     setappdata(h,'options',opt);
0997 
0998 function doSetVerbose(fig,evd,h)
0999     opt = getappdata(h,'options');
1000     opt.verbose = get(fig,'Value');
1001     setappdata(h,'options',opt);
1002 
1003 function doSetSaveAsMat(fig,evd,h)
1004     opt = getappdata(h,'options');
1005     opt.save = get(fig,'Value');
1006     setappdata(h,'options',opt);
1007 
1008 function doSetLoadMat(fig,evd,h)
1009     opt = getappdata(h,'options');
1010     [fname, pname, findex] = uigetfile('m2html.mat',...
1011         'Load a m2html MAT-file');
1012     if findex
1013         opt.load = fullfile(pname,fname);
1014         set(fig,'String',fullfile(pname,fname));
1015     end
1016     setappdata(h,'options',opt);
1017 
1018 function doSetTabs(fig,evd,h)
1019     opt = getappdata(h,'options');
1020     t = str2num(get(fig,'String'));
1021     if t >= 0 & length(t) == 1 
1022         opt.tabs = t; 
1023     else
1024         set(fig,'String',num2str(opt.tabs));
1025     end
1026     setappdata(h,'options',opt);
1027 
1028 function doSetNbColumns(fig,evd,h)
1029     opt = getappdata(h,'options');
1030     disp 'Not available';
1031     setappdata(h,'options',opt);
1032     
1033 %===============================================================================
1034 
1035 function text2 = shortenText(text, l)
1036 
1037     if nargin == 1, l = 64; end
1038     m = length(text);
1039     text2 = text;
1040     if m > l
1041         s = floor((l - 3) / 2);
1042         text2 = [text(1:s) '...' text(end-(l-s-3)+1:end)];
1043     end
```

---

Generated on Tue 29-Oct-2019 21:04:54 by **m2html** © 2005
