# Supplementary material for: Personalized whole‐body models integrate metabolism, physiology, and the gut microbiome
Source: Mol Syst Biol. 2020 May 28;16(5):e8982. doi: 10.15252/msb.20198982 (PMC7285886; doi:10.15252/msb.20198982)
Supplement: Supplementary file 22 — Dataset EV1 [file MSB-16-e8982-s022.zip › PSCM_toolbox/ext/m2html/doc/m2html/index.html]

Index for Directory m2html


|  |  |
| --- | --- |
| Master index | Index for m2html |

# Index for m2html

## Matlab files in this directory:

|  |  |
| --- | --- |
| Contents | M2HTML Toolbox - A Documentation Generator for Matlab in HTML |
| m2html | M2HTML - Documentation Generator for Matlab M-files and Toolboxes in HTML |
| mdot | MDOT - Export a dependency graph into DOT language |
| mwizard | MWIZARD - M2HTML Graphical User Interface |
| mwizard2 | MWIZARD - M2HTML Graphical User Interface |

## Subsequent directories:

- @template
- private
- templates

---

Generated on Tue 29-Oct-2019 21:04:53 by **m2html** © 2005
