# Supplementary material for: Personalized whole‐body models integrate metabolism, physiology, and the gut microbiome
Source: Mol Syst Biol. 2020 May 28;16(5):e8982. doi: 10.15252/msb.20198982 (PMC7285886; doi:10.15252/msb.20198982)
Supplement: Supplementary file 22 — Dataset EV1 [file MSB-16-e8982-s022.zip › PSCM_toolbox/ext/m2html/doc/m2html/mwizard.html]

Description of mwizard


Home > m2html > mwizard.m

# mwizard

## PURPOSE

**MWIZARD - M2HTML Graphical User Interface**

## SYNOPSIS

**function mwizard(file)**

## DESCRIPTION

```
MWIZARD - M2HTML Graphical User Interface
  MWIZARD launches a Matlab GUI front-end to edit parameters
  that are then used by M2HTML to generate HTML documentation.
  MWIZARD(FILE) allows to specify a mat-file FILE from which
  default parameters are extracted and can be updated.  

  For more information, please read the M2HTML tutorial and FAQ at:
    <http://www.artefact.tk/software/matlab/m2html/>

  See also M2HTML
```

## CROSS-REFERENCE INFORMATION

This function calls:

- m2html M2HTML - Documentation Generator for Matlab M-files and Toolboxes in HTML

This function is called by:


## SUBFUNCTIONS

- function h = initWindow
- function buildWindow(h)
- function doClose(fig,evd,h)
- function doNewFile(fig,evd,h)
- function doOpenFile(fig,evd,h)
- function status = doSaveFile(fig,evd,h)
- function status = doSaveAsFile(fig,evd,h)
- function doRunFile(fig,evd,h)
- function status = doCheckSave(h)
- function doHelp(fig,evd,h)
- function varargout = initOptions(h)
- function refreshOptions(h)
- function doInitHTMLDir(fig,evd,h)
- function doInitTpl(fig,evd,h)
- function doInitMfiles(fig,evd,h)
- function doInitGraphs(fig,evd,h)
- function doSetMfiles(fig,evd,h)
- function doSetRecursive(fig,evd,h)
- function doSetOutputDir(fig,evd,h)
- function doSetIndex(fig,evd,h)
- function doSetExtension(fig,evd,h)
- function doSetTemplate(fig,evd,h)
- function doSetSource(fig,evd,h)
- function doSetHighlight(fig,evd,h)
- function doSetGraph(fig,evd,h)
- function doSetSearch(fig,evd,h)
- function doSetGlobal(fig,evd,h)
- function doSetDownload(fig,evd,h)
- function doSetTodo(fig,evd,h)
- function doSetVerbose(fig,evd,h)
- function doSetSaveAsMat(fig,evd,h)
- function doSetLoadMat(fig,evd,h)
- function doSetTabs(fig,evd,h)
- function doSetNbColumns(fig,evd,h)
- function text2 = shortenText(text, l)

## SOURCE CODE

```
0001 function mwizard(file)
0002 %MWIZARD - M2HTML Graphical User Interface
0003 %  MWIZARD launches a Matlab GUI front-end to edit parameters
0004 %  that are then used by M2HTML to generate HTML documentation.
0005 %  MWIZARD(FILE) allows to specify a mat-file FILE from which
0006 %  default parameters are extracted and can be updated.
0007 %
0008 %  For more information, please read the M2HTML tutorial and FAQ at:
0009 %    <http://www.artefact.tk/software/matlab/m2html/>
0010 %
0011 %  See also M2HTML
0012 
0013 %  Copyright (C) 2003-2005 Guillaume Flandin <Guillaume@artefact.tk>
0014 %  $Revision: 0.5 $Date: 2004/05/24 20:12:17 $
0015 
0016 %  This program is free software; you can redistribute it and/or
0017 %  modify it under the terms of the GNU General Public License
0018 %  as published by the Free Software Foundation; either version 2
0019 %  of the License, or any later version.
0020 %
0021 %  This program is distributed in the hope that it will be useful,
0022 %  but WITHOUT ANY WARRANTY; without even the implied warranty of
0023 %  MERCHANTABILITY or FITNESS FOR A PARTICULAR PURPOSE.  See the
0024 %  GNU General Public License for more details.
0025 %
0026 %  You should have received a copy of the GNU General Public License
0027 %  along with this program; if not, write to the Free Software
0028 %  Foundation Inc, 59 Temple Pl. - Suite 330, Boston, MA 02111-1307, USA.
0029 
0030 %  Suggestions for improvement and fixes are always welcome, although no
0031 %  guarantee is made whether and when they will be implemented.
0032 %  Send requests to Guillaume@artefact.tk
0033 
0034 error(nargchk(0,1,nargin));
0035 
0036 disp('This is a beta version of mwizard.');
0037 disp('Please use the online version m2html instead.');
0038 
0039 h = initWindow;
0040 
0041 initOptions(h);
0042 
0043 buildWindow(h);
0044 
0045 setappdata(h, 'handles', guihandles(h));
0046 setappdata(h, 'pwd',     pwd);
0047 
0048 if nargin == 0
0049     setappdata(h, 'file', '');
0050     setappdata(h, 'needsave', 1);
0051 else
0052     setappdata(h, 'file', file);
0053     setappdata(h, 'needsave', 0);
0054     opt = load(file, 'options');
0055     setappdata(h, 'options', opt.options);
0056     refreshOptions(h);
0057 end
0058 
0059 set(h, 'HandleVisibility', 'callback');
0060 
0061 %===============================================================================
0062 
0063 function h = initWindow
0064 
0065 h = figure('Resize',      'on',...
0066            'MenuBar',     'none',...
0067            'NumberTitle', 'off',...
0068            'Name',        ':: M2HTML Wizard ::',...
0069            'Position',    [200 200 500 650],...
0070            'Tag',         mfilename);
0071            
0072 set(h, 'CloseRequestFcn', {@doClose,h});
0073 
0074 %===============================================================================
0075 
0076 function buildWindow(h)
0077 
0078 wincolor = struct('bg',    [0.9 0.9 0.9], ...
0079                   'fg',    [0.8 0.8 0.8], ...
0080                   'title', [0.8 0.8 0.9]);
0081 
0082 set(h, 'Color', wincolor.bg);
0083               
0084 %-------------------------------------------------------------------------------
0085 %- Menu
0086 %-------------------------------------------------------------------------------
0087 
0088 icons = load(fullfile(fileparts(which(mfilename)),'private', ...
0089             'm2htmltoolbarimages.mat'));
0090 
0091 uipushtool('CData',icons.newIcon,...
0092     'enable','on',...
0093     'Separator','off',...
0094     'ToolTipString','New File',...
0095     'ClickedCallback',{@doNewFile,h},...
0096     'Tag','NewTool');
0097 
0098 uipushtool('CData',icons.openIcon,...
0099     'enable','on',...
0100     'Separator','off',...
0101     'ToolTipString','Open File',...
0102     'ClickedCallback',{@doOpenFile,h},...
0103     'Tag','OpenTool');
0104 
0105 uipushtool('CData',icons.saveIcon,...
0106     'enable','on',...
0107     'Separator','off',...
0108     'ToolTipString','Save File',...
0109     'ClickedCallback',{@doSaveFile,h},...
0110     'Tag','SaveTool');
0111 
0112 uipushtool('CData',icons.saveAsIcon,...
0113     'enable','on',...
0114     'Separator','off',...
0115     'ToolTipString','Save File As',...
0116     'ClickedCallback',{@doSaveAsFile,h},...
0117     'Tag','SaveAsTool');
0118     
0119 uipushtool('CData',icons.wheelIcon,...
0120     'enable','on',...
0121     'Separator','on',...
0122     'ToolTipString','Save and Run M2HTML',...
0123     'ClickedCallback',{@doRunFile,h},...
0124     'Tag','RunTool');
0125 
0126 uipushtool('CData',icons.webIcon,...
0127     'enable','on',...
0128     'Separator','on',...
0129     'ToolTipString','Online Tutorial',...
0130     'ClickedCallback',...
0131         'web(''http://www.artefact.tk/software/matlab/m2html/'')',...
0132     'Tag','WebTool');
0133 
0134 uipushtool('CData',icons.helpIcon,...
0135     'enable','on',...
0136     'Separator','off',...
0137     'ToolTipString','Help',...
0138     'ClickedCallback',{@doHelp,h},...
0139     'Tag','HelpTool');
0140 
0141 %-------------------------------------------------------------------------------
0142 %- Title
0143 %-------------------------------------------------------------------------------
0144 
0145 uicontrol('Style','Frame',...
0146     'Units','Normalized',...
0147     'Position',[0.02,0.92,0.96,0.06],...
0148     'BackgroundColor',wincolor.title);
0149 
0150 uicontrol('Style','Text',...
0151     'Units','Normalized',...
0152     'String','M2HTML Wizard',...
0153     'FontSize',18,...
0154     'HorizontalAlignment','center',...
0155     'Position',[0.03,0.93,0.94,0.038],...
0156     'BackgroundColor',wincolor.title);
0157 
0158 %-------------------------------------------------------------------------------
0159 %- Input
0160 %-------------------------------------------------------------------------------
0161 
0162 uicontrol('Style','Frame',...
0163     'Units','Normalized',...
0164     'Position',[0.02,0.74,0.96,0.16],...
0165     'BackgroundColor',wincolor.fg);
0166     
0167 uicontrol('Style','Frame',...
0168     'Units','Normalized',...
0169     'HorizontalAlignment','center',...
0170     'Position',[0.02,0.87,0.96,0.03],...
0171     'BackgroundColor',wincolor.title);
0172     
0173 uicontrol('Style','Text',...
0174     'Units','Normalized',...
0175     'String','M-Files Input',...
0176     'HorizontalAlignment','left',...
0177     'Position',[0.03,0.875,0.94,0.02],...
0178     'BackgroundColor',wincolor.title);
0179 
0180 uicontrol('Style','Text',...
0181     'Units','Normalized',...
0182     'String','Root directory:',...
0183     'FontAngle','oblique',...
0184     'HorizontalAlignment','left',...
0185     'Position',[0.04,0.825,0.6,0.03],...
0186     'BackgroundColor',wincolor.fg);
0187 
0188 uicontrol('Style','edit',...
0189     'Units','Normalized',...
0190     'Position',[0.21,0.83,0.74,0.03],...
0191     'String',pwd,...
0192     'Enable','inactive',...
0193     'HorizontalAlignment','left',...
0194     'Callback','uigetfile;',...%uigetdir
0195     'BackgroundColor',wincolor.bg,...
0196     'Tag','rootdir');
0197 
0198 uicontrol('Style','Text',...
0199     'Units','Normalized',...
0200     'String','Relative pathes:',...
0201     'HorizontalAlignment','left',...
0202     'Position',[0.04,0.785,0.6,0.03],...
0203     'BackgroundColor',wincolor.fg);
0204 
0205 uicontrol('Style','edit',...
0206     'Units','Normalized',...
0207     'Position',[0.21,0.79,0.74,0.03],...
0208     'String','',...
0209     'HorizontalAlignment','left',...
0210     'Callback',{@doSetMfiles,h},...
0211     'CreateFcn',{@doInitMfiles,h},...
0212     'BackgroundColor',wincolor.bg,...
0213     'Tag','mfiles');
0214 
0215 uicontrol('Style','CheckBox',...
0216     'Units','Normalized',...
0217     'Position',[0.04,0.749,0.42,0.032],...
0218     'String',' Recursive',...
0219     'HorizontalAlignment','left',...
0220     'Callback',{@doSetRecursive,h},...
0221     'Value',0,...
0222     'BackgroundColor',wincolor.bg,...
0223     'Tag','recursive');
0224 
0225 %-------------------------------------------------------------------------------
0226 %- Output
0227 %-------------------------------------------------------------------------------
0228 
0229 uicontrol('Style','Frame',...
0230     'Units','Normalized',...
0231     'Position',[0.02, 0.56,0.96,0.16],...
0232     'BackgroundColor',wincolor.fg);
0233 
0234 uicontrol('Style','Frame',...
0235     'Units','Normalized',...
0236     'HorizontalAlignment','center',...
0237     'Position',[0.02,0.69,0.96,0.03],...
0238     'BackgroundColor',wincolor.title);
0239     
0240 uicontrol('Style','Text',...
0241     'Units','Normalized',...
0242     'String','HTML Output',...
0243     'HorizontalAlignment','left',...
0244     'Position',[0.03,0.695,0.94,0.02],...
0245     'BackgroundColor',wincolor.title);
0246 
0247 uicontrol('Style','Text',...
0248     'Units','Normalized',...
0249     'String','Output Directory:',...
0250     'HorizontalAlignment','left',...
0251     'Position',[0.04,0.645,0.6,0.03],...
0252     'BackgroundColor',wincolor.fg);
0253 
0254 uicontrol('Style','edit',...
0255     'Units','Normalized',...
0256     'Position',[0.21,0.65,0.74,0.03],...
0257     'String','',...
0258     'HorizontalAlignment','left',...
0259     'Callback',{@doSetOutputDir,h},...
0260     'CreateFcn',{@doInitHTMLDir,h},...
0261     'BackgroundColor',wincolor.bg,...
0262     'Tag','htmldir');
0263 
0264 uicontrol('Style','Text',...
0265     'Units','Normalized',...
0266     'String','HTML Index:',...
0267     'HorizontalAlignment','left',...
0268     'Position',[0.04,0.605,0.6,0.03],...
0269     'BackgroundColor',wincolor.fg);
0270 
0271 uicontrol('Style','edit',...
0272     'Units','Normalized',...
0273     'Position',[0.21,0.61,0.25,0.03],...
0274     'String','index',...
0275     'HorizontalAlignment','left',...
0276     'Callback',{@doSetIndex,h},...
0277     'BackgroundColor',wincolor.bg,...
0278     'Tag','index');
0279 
0280 uicontrol('Style','Text',...
0281     'Units','Normalized',...
0282     'String','Extension:',...
0283     'HorizontalAlignment','left',...
0284     'Position',[0.53,0.605,0.3,0.03],...
0285     'BackgroundColor',wincolor.fg);
0286 
0287 uicontrol('Style','edit',...
0288     'Units','Normalized',...
0289     'Position',[0.70,0.61,0.25,0.03],...
0290     'String','html',...
0291     'HorizontalAlignment','left',...
0292     'Callback',{@doSetExtension,h},...
0293     'BackgroundColor',wincolor.bg,...
0294     'Tag','extension');
0295 
0296 uicontrol('Style','Text',...
0297     'Units','Normalized',...
0298     'String','Template:',...
0299     'HorizontalAlignment','left',...
0300     'Position',[0.04,0.565,0.3,0.03],...
0301     'BackgroundColor',wincolor.fg);
0302 
0303 uicontrol('Style','popupmenu',...
0304     'Units','Normalized',...
0305     'Position',[0.21,0.57,0.25,0.03],...
0306     'String','',...
0307     'HorizontalAlignment','center',...
0308     'Callback',{@doSetTemplate,h},...
0309     'CreateFcn',{@doInitTpl,h},...
0310     'BackgroundColor',wincolor.bg,...
0311     'Tag','template');
0312 
0313 %-------------------------------------------------------------------------------
0314 %- Other options
0315 %-------------------------------------------------------------------------------
0316 
0317 uicontrol('Style','Frame',...
0318     'Units','Normalized',...
0319     'Position',[0.02,0.24,0.96,0.30],...
0320     'BackgroundColor',wincolor.fg);
0321 
0322 uicontrol('Style','Frame',...
0323     'Units','Normalized',...
0324     'HorizontalAlignment','center',...
0325     'Position',[0.02,0.51,0.96,0.03],...
0326     'BackgroundColor',wincolor.title);
0327     
0328 uicontrol('Style','Text',...
0329     'Units','Normalized',...
0330     'String','Other Options',...
0331     'HorizontalAlignment','left',...
0332     'Position',[0.03,0.515,0.94,0.02],...
0333     'BackgroundColor',wincolor.title);
0334 
0335 uicontrol('Style','checkbox',...
0336     'Units','Normalized',...
0337     'Position',[0.04,0.464,0.42,0.032],...
0338     'String',' Include Source Code',...
0339     'HorizontalAlignment','left',...
0340     'Callback',{@doSetSource,h},...
0341     'Value',1,...
0342     'TooltipString','Include Source Code of each M-file',...
0343     'BackgroundColor',wincolor.bg,...
0344     'Tag','source');
0345 
0346 uicontrol('Style','checkbox',...
0347     'Units','Normalized',...
0348     'Position',[0.53,0.464,0.42,0.032],...
0349     'String',' Syntax Highlighting',...
0350     'HorizontalAlignment','left',...
0351     'Callback',{@doSetHighlight,h},...
0352     'Value',1,...
0353     'TooltipString','Source Code Syntax Highlighting',...
0354     'BackgroundColor',wincolor.bg,...
0355     'Tag','highlight');
0356 
0357 uicontrol('Style','checkbox',...
0358     'Units','Normalized',...
0359     'Position',[0.04,0.42,0.42,0.032],...
0360     'String',' Create Dependency Graphs',...
0361     'HorizontalAlignment','left',...
0362     'Callback',{@doSetGraph,h},...
0363     'CreateFcn',{@doInitGraphs,h},...
0364     'Value',0,...
0365     'TooltipString','Compute a Dependency Graph using GraphViz',...
0366     'BackgroundColor',wincolor.bg,...
0367     'Tag','graph');
0368 
0369 uicontrol('Style','checkbox',...
0370     'Units','Normalized',...
0371     'Position',[0.53,0.42,0.42,0.032],...
0372     'String',' PHP Search Engine',...
0373     'HorizontalAlignment','left',...
0374     'Callback',{@doSetSearch,h},...
0375     'Value',0,...
0376     'TooltipString','Create an Index for a PHP Search Engine',...
0377     'BackgroundColor',wincolor.bg,...
0378     'Tag','search');
0379 
0380 uicontrol('Style','checkbox',...
0381     'Units','Normalized',...
0382     'Position',[0.04,0.378,0.42,0.032],...
0383     'String',' Global Hyperlinks',...
0384     'HorizontalAlignment','left',...
0385     'Callback',{@doSetGlobal,1},...
0386     'Value',0,...
0387     'TooltipString','Hypertext links among separate Matlab Directories',...
0388     'BackgroundColor',wincolor.bg,...
0389     'Tag','globalhypertext');
0390 
0391 uicontrol('Style','checkbox',...
0392     'Units','Normalized',...
0393     'Position',[0.53,0.378,0.42,0.032],...
0394     'String',' Downloadable M-files',...
0395     'HorizontalAlignment','left',...
0396     'Callback',{@doSetDownload,h},...
0397     'TooltipString','Add a link to download each M-file separately',...
0398     'Value',0,...
0399     'BackgroundColor',wincolor.bg,...
0400     'Tag','download');
0401 
0402 uicontrol('Style','checkbox',...
0403     'Units','Normalized',...
0404     'Position',[0.04,0.336,0.42,0.032],...
0405     'String',' To Do List',...
0406     'HorizontalAlignment','left',...
0407     'Callback',{@doSetTodo,h},...
0408     'TooltipString',['Create a TODO list in each directory summarizing'...
0409     ' all the ''% TODO %'' lines found in Matlab code'],...
0410     'Value',0,...
0411     'BackgroundColor',wincolor.bg,...
0412     'Tag','todo');
0413 
0414 uicontrol('Style','checkbox',...
0415     'Units','Normalized',...
0416     'Position',[0.53,0.336,0.42,0.032],...
0417     'String',' Verbose Mode',...
0418     'HorizontalAlignment','left',...
0419     'Callback',{@doSetVerbose,h},...
0420     'TooltipString','Verbose mode',...
0421     'Value',1,...
0422     'BackgroundColor',wincolor.bg,...
0423     'Tag','verbose');
0424 
0425 uicontrol('Style','checkbox',...
0426     'Units','Normalized',...
0427     'Position',[0.04,0.294,0.42,0.032],...
0428     'String',' Save M-files Parsing',...
0429     'HorizontalAlignment','left',...
0430     'Callback',{@doSetSaveAsMat,h},...
0431     'TooltipString',['Save current state after M-files parsing in '...
0432     '''m2html.mat'' in the Output directory'],...
0433     'Value',0,...
0434     'BackgroundColor',wincolor.bg,...
0435     'Tag','save');
0436 
0437 uicontrol('Style','Text',...
0438     'Units','Normalized',...
0439     'String','Load File:',...
0440     'HorizontalAlignment','left',...
0441     'Position',[0.53,0.289,0.3,0.03],...
0442     'BackgroundColor',wincolor.fg);
0443 
0444 uicontrol('Style','edit',...
0445     'Units','Normalized',...
0446     'Position',[0.70,0.294,0.25,0.03],...
0447     'String','',...
0448     'HorizontalAlignment','left',...
0449     'Callback',{@doSetLoadMat,h},...
0450     'TooltipString',['Load a previously saved MAT file '...
0451     'to generate HTML files once again'],...
0452     'BackgroundColor',wincolor.bg,...
0453     'Tag','load');
0454 
0455 uicontrol('Style','Text',...
0456     'Units','Normalized',...
0457     'String','Tabs Length:',...
0458     'HorizontalAlignment','left',...
0459     'Position',[0.04,0.247,0.3,0.03],...
0460     'BackgroundColor',wincolor.fg);
0461 
0462 uicontrol('Style','edit',...
0463     'Units','Normalized',...
0464     'Position',[0.21,0.252,0.25,0.03],...
0465     'String','4',...
0466     'HorizontalAlignment','right',...
0467     'Callback',{@doSetTabs,h},...
0468     'TooltipString',['Replace horizontal tabs in source code '...
0469     'by N white space characters'],...
0470     'BackgroundColor',wincolor.bg,...
0471     'Tag','tabs');
0472 
0473 uicontrol('Style','Text',...
0474     'Units','Normalized',...
0475     'String','Nb Columns:',...
0476     'FontAngle','oblique',...
0477     'HorizontalAlignment','left',...
0478     'Position',[0.53,0.247,0.3,0.03],...
0479     'BackgroundColor',wincolor.fg);
0480 
0481 uicontrol('Style','edit',...
0482     'Units','Normalized',...
0483     'Position',[0.70,0.252,0.25,0.03],...
0484     'String','4',...
0485     'HorizontalAlignment','right',...
0486     'Callback',{@doSetNbColumns,h},...
0487     'TooltipString','Number of columns for M-files output - not available',...
0488     'Enable','inactive',...
0489     'BackgroundColor',wincolor.bg,...
0490     'Tag','column');
0491 
0492 
0493 %-------------------------------------------------------------------------------
0494 %- Space available
0495 %-------------------------------------------------------------------------------
0496 
0497 % uicontrol('Style','Frame',...
0498 %     'Units','Normalized',...
0499 %     'Position',[0.02,0.07,0.96,0.14],...
0500 %     'BackgroundColor',wincolor.fg);
0501 
0502 % simulate a frame using an axes
0503 % http://www.mathworks.com/support/solutions/data/1-15P9E.html
0504 axes('Color',wincolor.fg,...
0505     'XTick',[],'YTick',[],...
0506     'Units','Normalized',...
0507     'Box','on',...
0508     'Position',[0.02,0.07,0.9585,0.14]);
0509 
0510 uicontrol('Style','Frame',...
0511     'Units','Normalized',...
0512     'HorizontalAlignment','center',...
0513     'Position',[0.02,0.19,0.96,0.03],...
0514     'BackgroundColor',wincolor.title);
0515 
0516 uicontrol('Style','Text',...
0517     'Units','Normalized',...
0518     'String','M2HTML status',...
0519     'HorizontalAlignment','left',...
0520     'Position',[0.03,0.195,0.94,0.02],...
0521     'BackgroundColor',wincolor.title);
0522 
0523 uicontrol('Style','Text',...
0524     'Units','Normalized',...
0525     'String','Click on the wheel in the toolbar to launch M2HTML...',...
0526     'HorizontalAlignment','left',... % center
0527     'Position',[0.12,0.135,0.76,0.02],...
0528     'Visible','on',...
0529     'BackgroundColor',wincolor.fg,...
0530     'Tag','textmisc');
0531 
0532 axes('XLim',[0 100],...
0533     'YLim',[0 1],...
0534     'Box','on', ...
0535     'Units','Normalized',...
0536     'Position',[0.07,0.09,0.86,0.03],...
0537     'XTickMode','manual',...
0538     'YTickMode','manual',...
0539     'layer','top',...
0540     'XTick',[],...
0541     'YTick',[],...
0542     'XTickLabelMode','manual',...
0543     'XTickLabel',[],...
0544     'YTickLabelMode','manual',...
0545     'Visible','on',...
0546     'YTickLabel',[],...
0547     'Color',wincolor.bg);
0548 
0549 x = 0; % between 0 and 100
0550 xpatch = [0 x x 0];
0551 ypatch = [0 0 1 1];
0552   
0553 p = patch(xpatch,ypatch,'r',...
0554     'EdgeColor','r',...
0555     'Visible','on',...
0556     'EraseMode','none',...
0557     'Tag','waitbarmisc');
0558   
0559 l = line([100 0 0 100 100], [0 0 1 1 0], ...
0560     'EraseMode','none', ...
0561     'Visible','on',...
0562     'Color',get(gca,'XColor'));
0563   
0564 % for i=10:5:100
0565 %     set(p,'Xdata',[0 i i 0]); pause(0.02);
0566 % end
0567 % set(p,'EraseMode','normal');
0568 % set(p,'Xdata',[0 0 0 0]);
0569 % set(p,'EraseMode','none');
0570 
0571 %-------------------------------------------------------------------------------
0572 %- Footnote
0573 %-------------------------------------------------------------------------------
0574 
0575 uicontrol('Style','Frame',...
0576     'Units','Normalized',...
0577     'Position',[0.02,0.02,0.96,0.03],...
0578     'BackgroundColor',[0.8 0.8 0.9]);
0579 
0580 uicontrol('Style','Text',...
0581     'Units','Normalized',...
0582     'String',['M2HTML � 2003-2005 Guillaume Flandin <Guillaume@artefact.tk>'],...
0583     'HorizontalAlignment','right',...
0584     'Position',[0.03,0.025,0.94,0.02],...
0585     'BackgroundColor',[0.8 0.8 0.9]);
0586 
0587 %===============================================================================
0588 
0589 function doClose(fig,evd,h)
0590     status = doCheckSave(h);
0591     if status
0592         delete(h);
0593     end
0594     
0595 function doNewFile(fig,evd,h)
0596     status = doCheckSave(h);
0597     if status
0598         initOptions(h);
0599         setappdata(h, 'needsave', 1);
0600         % refresh options in GUI...
0601         refreshOptions(h);
0602     end
0603 
0604 function doOpenFile(fig,evd,h)
0605     status = doCheckSave(h);
0606     if status
0607         [filename, pathname] = uigetfile('*.mat','Open File');
0608         if ~(isequal(filename,0)|isequal(pathname,0))
0609             opt = load(fullfile(pathname,filename),'options');
0610             setappdata(h,'options',opt.options);
0611             setappdata(h,'file',fullfile(pathname,filename));
0612         end
0613     end
0614     % refresh options in GUI...
0615     refreshOptions(h);
0616 
0617 function status = doSaveFile(fig,evd,h)
0618     file = getappdata(h,'file');
0619     status = 1;
0620     if isempty(file)
0621         status = doSaveAsFile(fig,evd,h);
0622     else
0623         options = getappdata(h,'options');
0624         save(file, 'options');
0625     end
0626     setappdata(h,'needsave',0);
0627 
0628 function status = doSaveAsFile(fig,evd,h)
0629     [filename, pathname] = uiputfile('matlab.mat', 'Save File as');
0630     if ~(isequal(filename,0)|isequal(pathname,0))
0631         setappdata(h,'file',fullfile(pathname,filename));
0632         status = doSaveFile(fig,evd,h);
0633     else
0634         status = 0;
0635     end
0636 
0637 function doRunFile(fig,evd,h)
0638     status = doSaveFile(fig,evd,h);
0639     if status
0640         opt = getappdata(h,'options');
0641         file = getappdata(h,'file');
0642         r = {'off' 'on'};
0643         % opt could be directly given to m2html (no need for file saving)
0644         % just need to convert on/off using opt.param = r{opt.param+1}
0645         m2html('load',file,'recursive',r{opt.recursive+1});
0646         % 'recursive' is specified to force m2html to parse M-files
0647     end
0648     
0649 function status = doCheckSave(h)
0650     file = getappdata(h,'file');
0651     if isempty(file), file = 'Untitled'; end
0652     needsave = getappdata(h,'needsave');
0653     status = 1;
0654     if needsave
0655         button = questdlg(sprintf('Save changes to %s?',file),...
0656             'Mwizard','Yes','No','Cancel','Yes');
0657         if strcmp(button,'Yes')
0658             status = doSaveFile([],[],h);
0659         elseif strcmp(button,'Cancel')
0660             status = 0;
0661         end
0662     end
0663 
0664 function doHelp(fig,evd,h)
0665     helpdlg(sprintf(['M2HTML by Guillaume Flandin\n'...
0666         'Copyright � 2003-2005\nGuillaume@artefact.tk\n'...
0667         '<http://www.artefact.tk/>']),'M2HTML Wizard');
0668 
0669 %===============================================================================
0670 
0671 %-------------------------------------------------------------------------------
0672 %- Default parameters
0673 %-------------------------------------------------------------------------------
0674 
0675 function varargout = initOptions(h)
0676     options = struct('verbose', 1,...
0677         'mFiles', {{''}},...
0678         'htmlDir', 'doc',...
0679         'recursive', 0,...
0680         'source', 1,...
0681         'download',0,...
0682         'syntaxHighlighting', 1,...
0683         'tabs', 4,...
0684         'globalHypertextLinks', 0,...
0685         'graph', 0,...
0686         'todo', 0,...
0687         'load', 0,...
0688         'save', 0,...
0689         'search', 0,...
0690         'helptocxml', 0,...
0691         'indexFile', 'index',...
0692         'extension', '.html',...
0693         'template', 'blue',...
0694         'rootdir', pwd,...
0695         'ignoredDir', {{'.svn' 'cvs'}}, ...
0696         'language','english');
0697     
0698     if nargin == 1,
0699         setappdata(h,'options',options);
0700     else
0701         varargout{1} = options;    
0702     end
0703 
0704 function refreshOptions(h)
0705     opt = getappdata(h,'options');
0706     handles = getappdata(h,'handles');
0707     
0708     doInitTpl(handles.template,    0, h);
0709     doInitMfiles(handles.mfiles,   0, h);
0710     doInitHTMLDir(handles.htmldir, 0, h)
0711     
0712     set(handles.recursive,       'Value',  opt.recursive);
0713     set(handles.graph,           'Value',  opt.graph); %doInitGraphs(handles.graph,0,h);
0714     set(handles.save,            'Value',  opt.save);
0715     set(handles.verbose,         'Value',  opt.verbose);
0716     set(handles.todo,            'Value',  opt.todo);
0717     set(handles.download,        'Value',  opt.download);
0718     set(handles.search,          'Value',  opt.search);
0719     set(handles.highlight,       'Value',  opt.syntaxHighlighting);
0720     set(handles.source,          'Value',  opt.source);
0721     set(handles.globalhypertext, 'Value',  opt.globalHypertextLinks);
0722     
0723     set(handles.index,           'String', opt.indexFile);
0724     set(handles.extension,       'String', opt.extension(2:end)); %remove the '.'
0725     set(handles.tabs,            'String', num2str(opt.tabs));
0726     if ~strcmp(opt.rootdir, pwd)
0727         warning('[M2HTML] You should ''cd %s'' before...',opt.rootdir);    
0728     end
0729     set(handles.rootdir,         'String', opt.rootdir); % need to 'cd' if different...
0730     set(handles.column,          'String', num2str(4)); %- not saved... default here
0731     if ischar(opt.load)
0732         set(handles.load,        'String', opt.load);
0733     else
0734         set(handles.load,        'String', '');
0735     end
0736     
0737     set(handles.textmisc,        'String', ...
0738         'Click on the wheel in the toolbar to launch M2HTML...'); %- not saved... default here
0739     set(handles.waitbarmisc,     'EraseMode','normal');
0740     set(handles.waitbarmisc,     'Xdata',[0 0 0 0]);
0741     set(handles.waitbarmisc,     'EraseMode','none');
0742 
0743 
0744 %-------------------------------------------------------------------------------
0745 %- CreateFcn Callbacks
0746 %-------------------------------------------------------------------------------
0747 
0748 function doInitHTMLDir(fig,evd,h)
0749     %- problems when htmlDir is still a full path
0750     opt = getappdata(h,'options');
0751     if isempty(strmatch(lower(pwd),lower(opt.htmlDir)))
0752         opt.htmlDir = fullfile(pwd, opt.htmlDir);
0753     end
0754     set(fig,'String',opt.htmlDir);
0755     setappdata(h,'options',opt);
0756 
0757 function doInitTpl(fig,evd,h)
0758     %- problems when templates are still in full format
0759     opt = getappdata(h,'options');
0760     d = dir(fullfile(fileparts(which(mfilename)),'templates'));
0761     d = {d([d.isdir]).name};
0762     d = {d{~ismember(d,{'.' '..'})}};
0763     if ~isempty(d)
0764         tpl = sprintf('%s|',d{:});
0765         set(fig,'String',tpl(1:end-1));
0766         i = strmatch(opt.template,d,'exact');
0767         if ~isempty(i)
0768             set(fig,'Value',i(1));
0769         else
0770             %- where is the default template ?
0771             warning('[M2HTML] Default template ''%s'' not found.',opt.template);
0772             set(fig,'Value',1);
0773             opt.template = d{1};
0774             setappdata(h,'options',opt);
0775             warning('[M2HTML] Using template ''%s'' instead.',opt.template);
0776         end
0777     else
0778         error('[M2HTML] No template found.');
0779     end
0780 
0781  function doInitMfiles(fig,evd,h)
0782     opt = getappdata(h,'options');
0783     if ~isempty(opt.mFiles{1})
0784         s = sprintf('''%s'', ',opt.mFiles{:}); s = s(1:end-2);
0785         set(fig,'String',['{' s '}']);
0786         return;
0787     end
0788     d = dir(pwd); d = {d([d.isdir]).name};
0789     d = {d{~ismember(d,{'.' '..'})}};
0790     if length(d) == 0
0791         warning('[M2HTML] No subsequent directory found. Check your cwd.');
0792         set(fig,'String',''); %- maybe open a uigetdir ?
0793         opt.mFiles = {''};
0794     elseif length(d) == 1
0795         set(fig,'String',d{1});
0796         opt.mFiles = d;
0797     else
0798         s = sprintf('''%s'', ',d{:}); s = s(1:end-2);
0799         set(fig,'String',['{' s '}']);
0800         opt.mFiles = d;
0801     end
0802     setappdata(h,'options',opt);
0803     
0804 function doInitGraphs(fig,evd,h)
0805     opt = getappdata(h,'options');
0806     [s, w] = system('dot -V');
0807     if s
0808         disp('GraphViz not installed: Generation of dependency graphs desactivated.');
0809         disp('See http://www.graphviz.org/ to get ''dot'' tool.');
0810         set(fig,'FontAngle','Oblique','Enable','inactive');
0811         set(fig,'Value',0);
0812         opt.graph = 0;
0813         setappdata(h,'options',opt);
0814     else
0815         set(fig,'Value',opt.graph);
0816     end
0817 
0818 
0819 %===============================================================================
0820 
0821 %-------------------------------------------------------------------------------
0822 %- M-Files Input Callbacks
0823 %-------------------------------------------------------------------------------
0824 
0825 function doSetMfiles(fig,evd,h)
0826     opt = getappdata(h,'options');
0827     l = get(fig,'String');
0828     l = fliplr(deblank(fliplr(l)));
0829     if isempty(l) | l(1) ~= '{'
0830         opt.mFiles = {l};
0831     else
0832         try,
0833             d = eval(l);
0834         catch,
0835             disp('[M2HTML] The list of M-files is corrupted. Please check it.');
0836             return;
0837         end
0838         [i,v] = listdlg('ListString',d,...
0839                         'PromptString','Select folder(s):',...
0840                         'Name',':: M2HTML :: M-files',...
0841                         'SelectionMode','multiple');
0842         if v == 1
0843             d = {d{i}};
0844             s = sprintf('''%s'', ',d{:}); s = s(1:end-2);
0845             set(fig,'String',['{' s '}']);
0846         end
0847         opt.mFiles = d;
0848     end
0849     setappdata(h,'options',opt);
0850 
0851 function doSetRecursive(fig,evd,h)
0852     opt = getappdata(h,'options');
0853     opt.recursive = get(fig,'Value');
0854     setappdata(h,'options',opt);
0855 
0856 %-------------------------------------------------------------------------------
0857 %- HTML Output Callbacks
0858 %-------------------------------------------------------------------------------
0859 
0860 function doSetOutputDir(fig,evd,h)
0861     opt = getappdata(h,'options');
0862     opt.htmlDir = get(fig,'String');
0863     setappdata(h,'options',opt);
0864 
0865 function doSetIndex(fig,evd,h)
0866     opt = getappdata(h,'options');
0867     opt.indexFile = get(fig,'String');
0868     setappdata(h,'options',opt);
0869     
0870 function doSetExtension(fig,evd,h)
0871     opt = getappdata(h,'options');
0872     e = get(fig,'String');
0873     if ~isempty(e) & e(1) ~= '.'
0874         e = ['.' e];
0875     end
0876     opt.extension = e;
0877     setappdata(h,'options',opt);
0878     
0879 function doSetTemplate(fig,evd,h)
0880     opt = getappdata(h,'options');
0881     s = get(fig,'String');
0882     v = get(fig,'Value');
0883     opt.template = deblank(s(v,:));
0884     setappdata(h,'options',opt);
0885 
0886 %-------------------------------------------------------------------------------
0887 %- Options Callbacks
0888 %-------------------------------------------------------------------------------
0889 
0890 function doSetSource(fig,evd,h)
0891     opt = getappdata(h,'options');
0892     opt.source = get(fig,'Value');
0893     setappdata(h,'options',opt);
0894 
0895 function doSetHighlight(fig,evd,h)
0896     opt = getappdata(h,'options');
0897     opt.syntaxHighlighting = get(fig,'Value');
0898     setappdata(h,'options',opt);
0899 
0900 function doSetGraph(fig,evd,h)
0901     opt = getappdata(h,'options');
0902     opt.graph = get(fig,'Value');
0903     setappdata(h,'options',opt);
0904 
0905 function doSetSearch(fig,evd,h)
0906     opt = getappdata(h,'options');
0907     opt.search = get(fig,'Value');
0908     setappdata(h,'options',opt);
0909 
0910 function doSetGlobal(fig,evd,h)
0911     opt = getappdata(h,'options');
0912     opt.globalHypertextLinks = get(fig,'Value');
0913     setappdata(h,'options',opt);
0914 
0915 function doSetDownload(fig,evd,h)
0916     opt = getappdata(h,'options');
0917     opt.download = get(fig,'Value');
0918     setappdata(h,'options',opt);
0919 
0920 function doSetTodo(fig,evd,h)
0921     opt = getappdata(h,'options');
0922     opt.todo = get(fig,'Value');
0923     setappdata(h,'options',opt);
0924 
0925 function doSetVerbose(fig,evd,h)
0926     opt = getappdata(h,'options');
0927     opt.verbose = get(fig,'Value');
0928     setappdata(h,'options',opt);
0929 
0930 function doSetSaveAsMat(fig,evd,h)
0931     opt = getappdata(h,'options');
0932     opt.save = get(fig,'Value');
0933     setappdata(h,'options',opt);
0934 
0935 function doSetLoadMat(fig,evd,h)
0936     opt = getappdata(h,'options');
0937     [fname, pname, findex] = uigetfile('m2html.mat',...
0938         'Load a m2html MAT-file');
0939     if findex
0940         opt.load = fullfile(pname,fname);
0941         set(fig,'String',fullfile(pname,fname));
0942     end
0943     setappdata(h,'options',opt);
0944 
0945 function doSetTabs(fig,evd,h)
0946     opt = getappdata(h,'options');
0947     t = str2num(get(fig,'String'));
0948     if t >= 0 & length(t) == 1 
0949         opt.tabs = t; 
0950     else
0951         set(fig,'String',num2str(opt.tabs));
0952     end
0953     setappdata(h,'options',opt);
0954 
0955 function doSetNbColumns(fig,evd,h)
0956     opt = getappdata(h,'options');
0957     disp 'Not available';
0958     setappdata(h,'options',opt);
0959     
0960 %===============================================================================
0961 
0962 function text2 = shortenText(text, l)
0963 
0964     if nargin == 1, l = 64; end
0965     m = length(text);
0966     text2 = text;
0967     if m > l
0968         s = floor((l - 3) / 2);
0969         text2 = [text(1:s) '...' text(end-(l-s-3)+1:end)];
0970     end
```

---

Generated on Tue 29-Oct-2019 21:04:54 by **m2html** © 2005
