# Supplementary material for: Personalized whole‐body models integrate metabolism, physiology, and the gut microbiome
Source: Mol Syst Biol. 2020 May 28;16(5):e8982. doi: 10.15252/msb.20198982 (PMC7285886; doi:10.15252/msb.20198982)
Supplement: Supplementary file 22 — Dataset EV1 [file MSB-16-e8982-s022.zip › PSCM_toolbox/ext/m2html/doc/m2html/Contents.html]

Description of Contents


Home > m2html > Contents.m

# Contents

## PURPOSE

**M2HTML Toolbox - A Documentation Generator for Matlab in HTML**

## SYNOPSIS

**This is a script file.**

## DESCRIPTION

```
 M2HTML Toolbox - A Documentation Generator for Matlab in HTML
 Version 1.5 01-May-2005
 
 M2HTML main functions.
   m2html   - Documentation System for Matlab M-files in HTML.
   mdot     - Wrapper to GraphViz's <mdot> for dependency graphs.
   mwizard  - Graphical user interface for m2html.
   private  - Internal functions.

 Template toolbox.
   @template  - HTML template class

 Templates files.
   templates/blue   - Default HTML template.
   templates/frame  - Identical to <blue> but using frames.
   templates/brain  - Another frames-enabled template

 Others.
   Changelog, GPL, INSTALL, LICENSE, README, TODO.
```

## CROSS-REFERENCE INFORMATION

This function calls:


This function is called by:


---

Generated on Tue 29-Oct-2019 21:04:54 by **m2html** © 2005
