# Supplementary material for: Personalized whole‐body models integrate metabolism, physiology, and the gut microbiome
Source: Mol Syst Biol. 2020 May 28;16(5):e8982. doi: 10.15252/msb.20198982 (PMC7285886; doi:10.15252/msb.20198982)
Supplement: Supplementary file 22 — Dataset EV1 [file MSB-16-e8982-s022.zip › PSCM_toolbox/ext/m2html/doc/m2html/m2html.html]

Description of m2html


Home > m2html > m2html.m

# m2html

## PURPOSE

**M2HTML - Documentation Generator for Matlab M-files and Toolboxes in HTML**

## SYNOPSIS

**function m2html(varargin)**

## DESCRIPTION

```
M2HTML - Documentation Generator for Matlab M-files and Toolboxes in HTML
  M2HTML by itself generates an HTML documentation of the Matlab M-files found
  in the direct subdirectories of the current directory. HTML files are 
  written in a 'doc' directory (created if necessary). All the others options
  are set to default (in brackets in the following).
  M2HTML('PropertyName1',PropertyValue1,'PropertyName2',PropertyValue2,...)
  sets multiple option values. The list of option names and default values is:
    o mFiles - Cell array of strings or character array containing the
       list of M-files and/or directories of M-files for which an HTML
       documentation will be built (use relative paths without backtracking).
       Launch M2HTML one directory above the directory your wanting to
       generate documentation for  [ <all direct subdirectories> ]
    o htmlDir - Top level directory for generated HTML files [ 'doc' ]
    o recursive - Process subdirectories recursively [ on | {off} ]
    o source - Include Matlab source code in the generated documentation
                               [ {on} | off ]
    o download - Add a link to download each M-file separately [ on | {off} ]
    o syntaxHighlighting - Source Code Syntax Highlighting [ {on} | off ]
    o tabs - Replace '\t' (horizontal tab) in source code by n white space
        characters [ 0 ... {4} ... n ]
    o globalHypertextLinks - Hypertext links among separate Matlab 
        directories [ on | {off} ]
    o todo - Create a TODO list in each directory summarizing all the
        '% TODO %' lines found in Matlab code [ on | {off}]
    o graph - Compute a dependency graph using GraphViz [ on | {off}]
        'dot' required, see <http://www.graphviz.org/>
    o indexFile - Basename of the HTML index file [ 'index' ]
    o extension - Extension of generated HTML files [ '.html' ]
    o template - HTML template name to use [ {'blue'} | 'frame' | ... ]
    o search - Add a PHP search engine [ on | {off}] - beta version!
    o ignoredDir - List of directories to be ignored [ {'.svn' 'cvs'} ]
    o save - Save current state after M-files parsing in 'm2html.mat' 
        in directory htmlDir [ on | {off}]
    o load - Load a previously saved '.mat' M2HTML state to generate HTML 
        files once again with possibly other options [ <none> ]
    o verbose - Verbose mode [ {on} | off ]

  For more information, please read the M2HTML tutorial and FAQ at:
    <http://www.artefact.tk/software/matlab/m2html/>

  Examples:
    >> m2html('mfiles','matlab', 'htmldir','doc');
    >> m2html('mfiles',{'matlab/signal' 'matlab/image'}, 'htmldir','doc');
    >> m2html('mfiles','matlab', 'htmldir','doc', 'recursive','on');
    >> m2html('mfiles','mytoolbox', 'htmldir','doc', 'source','off');
    >> m2html('mfiles','matlab', 'htmldir','doc', 'global','on');
    >> m2html( ... , 'template','frame', 'index','menu');

  See also MWIZARD, MDOT, TEMPLATE.
```

## CROSS-REFERENCE INFORMATION

This function calls:

- mdot MDOT - Export a dependency graph into DOT language

This function is called by:

- mwizard MWIZARD - M2HTML Graphical User Interface
- mwizard2 MWIZARD - M2HTML Graphical User Interface

## SUBFUNCTIONS

- function mfiles = getmfiles(mdirs, mfiles, recursive, ignoredDir)
- function calldot(dotexec, mdotfile, mapfile, pngfile, opt)
- function s = backtomaster(mdir)
- function ldir = splitpath(p)
- function name = extractname(synopsis)
- function f = fullurl(varargin)
- function str = escapeblank(str)
- function str = entity(str)
- function str = horztab(str,n)

## SOURCE CODE

```
0001 function m2html(varargin)
0002 %M2HTML - Documentation Generator for Matlab M-files and Toolboxes in HTML
0003 %  M2HTML by itself generates an HTML documentation of the Matlab M-files found
0004 %  in the direct subdirectories of the current directory. HTML files are
0005 %  written in a 'doc' directory (created if necessary). All the others options
0006 %  are set to default (in brackets in the following).
0007 %  M2HTML('PropertyName1',PropertyValue1,'PropertyName2',PropertyValue2,...)
0008 %  sets multiple option values. The list of option names and default values is:
0009 %    o mFiles - Cell array of strings or character array containing the
0010 %       list of M-files and/or directories of M-files for which an HTML
0011 %       documentation will be built (use relative paths without backtracking).
0012 %       Launch M2HTML one directory above the directory your wanting to
0013 %       generate documentation for  [ <all direct subdirectories> ]
0014 %    o htmlDir - Top level directory for generated HTML files [ 'doc' ]
0015 %    o recursive - Process subdirectories recursively [ on | {off} ]
0016 %    o source - Include Matlab source code in the generated documentation
0017 %                               [ {on} | off ]
0018 %    o download - Add a link to download each M-file separately [ on | {off} ]
0019 %    o syntaxHighlighting - Source Code Syntax Highlighting [ {on} | off ]
0020 %    o tabs - Replace '\t' (horizontal tab) in source code by n white space
0021 %        characters [ 0 ... {4} ... n ]
0022 %    o globalHypertextLinks - Hypertext links among separate Matlab
0023 %        directories [ on | {off} ]
0024 %    o todo - Create a TODO list in each directory summarizing all the
0025 %        '% TODO %' lines found in Matlab code [ on | {off}]
0026 %    o graph - Compute a dependency graph using GraphViz [ on | {off}]
0027 %        'dot' required, see <http://www.graphviz.org/>
0028 %    o indexFile - Basename of the HTML index file [ 'index' ]
0029 %    o extension - Extension of generated HTML files [ '.html' ]
0030 %    o template - HTML template name to use [ {'blue'} | 'frame' | ... ]
0031 %    o search - Add a PHP search engine [ on | {off}] - beta version!
0032 %    o ignoredDir - List of directories to be ignored [ {'.svn' 'cvs'} ]
0033 %    o save - Save current state after M-files parsing in 'm2html.mat'
0034 %        in directory htmlDir [ on | {off}]
0035 %    o load - Load a previously saved '.mat' M2HTML state to generate HTML
0036 %        files once again with possibly other options [ <none> ]
0037 %    o verbose - Verbose mode [ {on} | off ]
0038 %
0039 %  For more information, please read the M2HTML tutorial and FAQ at:
0040 %    <http://www.artefact.tk/software/matlab/m2html/>
0041 %
0042 %  Examples:
0043 %    >> m2html('mfiles','matlab', 'htmldir','doc');
0044 %    >> m2html('mfiles',{'matlab/signal' 'matlab/image'}, 'htmldir','doc');
0045 %    >> m2html('mfiles','matlab', 'htmldir','doc', 'recursive','on');
0046 %    >> m2html('mfiles','mytoolbox', 'htmldir','doc', 'source','off');
0047 %    >> m2html('mfiles','matlab', 'htmldir','doc', 'global','on');
0048 %    >> m2html( ... , 'template','frame', 'index','menu');
0049 %
0050 %  See also MWIZARD, MDOT, TEMPLATE.
0051 
0052 %  Copyright (C) 2005 Guillaume Flandin <Guillaume@artefact.tk>
0053 %  $Revision: 1.5 $Date: 2005/05/01 16:15:30 $
0054 
0055 %  This program is free software; you can redistribute it and/or
0056 %  modify it under the terms of the GNU General Public License
0057 %  as published by the Free Software Foundation; either version 2
0058 %  of the License, or any later version.
0059 %
0060 %  This program is distributed in the hope that it will be useful,
0061 %  but WITHOUT ANY WARRANTY; without even the implied warranty of
0062 %  MERCHANTABILITY or FITNESS FOR A PARTICULAR PURPOSE.  See the
0063 %  GNU General Public License for more details.
0064 %
0065 %  You should have received a copy of the GNU General Public License
0066 %  along with this program; if not, write to the Free Software
0067 %  Foundation Inc, 59 Temple Pl. - Suite 330, Boston, MA 02111-1307, USA.
0068 
0069 %  Suggestions for improvement and fixes are always welcome, although no
0070 %  guarantee is made whether and when they will be implemented.
0071 %  Send requests to <Guillaume@artefact.tk>
0072 
0073 %  For tips on how to write Matlab code, see:
0074 %     * MATLAB Programming Style Guidelines, by R. Johnson:
0075 %       <http://www.datatool.com/prod02.htm>
0076 %     * For tips on creating help for your m-files 'type help.m'.
0077 %     * Matlab documentation on M-file Programming:
0078 %  <http://www.mathworks.com/access/helpdesk/help/techdoc/matlab_prog/ch_funh8.html>
0079 
0080 %  This function uses the Template class so that you can fully customize
0081 %  the output. You can modify .tpl files in templates/blue/ or create new
0082 %  templates in a new directory.
0083 %  See the template class documentation for more details.
0084 %  <http://www.artefact.tk/software/matlab/template/>
0085 
0086 %  Latest information on M2HTML is available on the web through:
0087 %  <http://www.artefact.tk/software/matlab/m2html/>
0088 
0089 %  Other Matlab to HTML converters available on the web:
0090 %  1/ mat2html.pl, J.C. Kantor, in Perl, 1995:
0091 %     <http://fresh.t-systems-sfr.com/unix/src/www/mat2html>
0092 %  2/ htmltools, B. Alsberg, in Matlab, 1997:
0093 %     <http://www.mathworks.com/matlabcentral/fileexchange/loadFile.do?objectId=175>
0094 %  3/ mtree2html2001, H. Pohlheim, in Perl, 1996, 2001:
0095 %     <http://www.pohlheim.com/perl_main.html#matlabdocu>
0096 %  4/ MatlabCodeColorizer, S. Faridani, in C#, 2005:
0097 %     <http://www.pitchup.com/matlabcolorizer/>
0098 %  5/ Highlight, G. Flandin, in Matlab, 2003:
0099 %     <http://www.artefact.tk/software/matlab/highlight/>
0100 %  6/ mdoc, P. Brinkmann, in Matlab, 2003:
0101 %     <http://www.math.uiuc.edu/~brinkman/software/mdoc/>
0102 %  7/ Ocamaweb, Miriad Technologies, in Ocaml, 2002:
0103 %     <http://ocamaweb.sourceforge.net/>
0104 %  8/ Matdoc, M. Kaminsky, in Perl, 2003:
0105 %     <http://www.mathworks.com/matlabcentral/fileexchange/loadFile.do?objectId=3498>
0106 %  9/ Matlab itself, The Mathworks Inc, with HELPWIN, DOC and PUBLISH (R14)
0107 
0108 %-------------------------------------------------------------------------------
0109 %- Set up options and default parameters
0110 %-------------------------------------------------------------------------------
0111 t0 = clock; % for statistics
0112 msgInvalidPair = 'Bad value for argument: ''%s''';
0113 
0114 options = struct('verbose', 1,...
0115                  'mFiles', {{'.'}},...
0116                  'htmlDir', 'doc',...
0117                  'recursive', 0,...
0118                  'source', 1,...
0119                  'download',0,...
0120                  'syntaxHighlighting', 1,...
0121                  'tabs', 4,...
0122                  'globalHypertextLinks', 0,...
0123                  'graph', 0,...
0124                  'todo', 0,...
0125                  'load', 0,...
0126                  'save', 0,...
0127                  'search', 0,...
0128                  'helptocxml', 0,...
0129                  'indexFile', 'index',...
0130                  'extension', '.html',...
0131                  'template', 'blue',...
0132                  'rootdir', pwd,...
0133                  'ignoredDir', {{'.svn' 'cvs'}}, ...
0134                  'language', 'english');
0135 
0136 if nargin == 1 & isstruct(varargin{1})
0137     paramlist = [ fieldnames(varargin{1}) ...
0138                   struct2cell(varargin{1}) ]';
0139     paramlist = { paramlist{:} };
0140 else
0141     if mod(nargin,2)
0142         error('Invalid parameter/value pair arguments.');
0143     end
0144     paramlist = varargin;
0145 end
0146 
0147 optionsnames = lower(fieldnames(options));
0148 for i=1:2:length(paramlist)
0149     pname = paramlist{i};
0150     pvalue = paramlist{i+1};
0151     ind = strmatch(lower(pname),optionsnames);
0152     if isempty(ind)
0153         error(['Invalid parameter: ''' pname '''.']);
0154     elseif length(ind) > 1
0155         error(['Ambiguous parameter: ''' pname '''.']);
0156     end
0157     switch(optionsnames{ind})
0158         case 'verbose'
0159             if strcmpi(pvalue,'on')
0160                 options.verbose = 1;
0161             elseif strcmpi(pvalue,'off')
0162                 options.verbose = 0;
0163             else
0164                 error(sprintf(msgInvalidPair,pname));
0165             end
0166         case 'mfiles'
0167             if iscellstr(pvalue)
0168                 options.mFiles = pvalue;
0169             elseif ischar(pvalue)
0170                 options.mFiles = cellstr(pvalue);
0171             else
0172                 error(sprintf(msgInvalidPair,pname));
0173             end
0174             options.load = 0;
0175         case 'htmldir'
0176             if ischar(pvalue)
0177                 if isempty(pvalue),
0178                     options.htmlDir = '.';
0179                 else
0180                     options.htmlDir = pvalue;
0181                 end
0182             else
0183                 error(sprintf(msgInvalidPair,pname));
0184             end
0185         case 'recursive'
0186             if strcmpi(pvalue,'on')
0187                 options.recursive = 1;
0188             elseif strcmpi(pvalue,'off')
0189                 options.recursive = 0;
0190             else
0191                 error(sprintf(msgInvalidPair,pname));
0192             end
0193             options.load = 0;
0194         case 'source'
0195             if strcmpi(pvalue,'on')
0196                 options.source = 1;
0197             elseif strcmpi(pvalue,'off')
0198                 options.source = 0;
0199             else
0200                 error(sprintf(msgInvalidPair,pname));
0201             end
0202         case 'download'
0203             if strcmpi(pvalue,'on')
0204                 options.download = 1;
0205             elseif strcmpi(pvalue,'off')
0206                 options.download = 0;
0207             else
0208                 error(sprintf(msgInvalidPair,pname));
0209             end
0210         case 'syntaxhighlighting'
0211             if strcmpi(pvalue,'on')
0212                 options.syntaxHighlighting = 1;
0213             elseif strcmpi(pvalue,'off')
0214                 options.syntaxHighlighting = 0;
0215             else
0216                 error(sprintf(msgInvalidPair,pname));
0217             end
0218         case 'tabs'
0219             if pvalue >= 0
0220                 options.tabs = pvalue;
0221             else
0222                 error(sprintf(msgInvalidPair,pname));
0223             end
0224         case 'globalhypertextlinks'
0225             if strcmpi(pvalue,'on')
0226                 options.globalHypertextLinks = 1;
0227             elseif strcmpi(pvalue,'off')
0228                 options.globalHypertextLinks = 0;
0229             else
0230                 error(sprintf(msgInvalidPair,pname));
0231             end
0232             options.load = 0;
0233         case 'graph'
0234             if strcmpi(pvalue,'on')
0235                 options.graph = 1;
0236             elseif strcmpi(pvalue,'off')
0237                 options.graph = 0;
0238             else
0239                 error(sprintf(msgInvalidPair,pname));
0240             end
0241         case 'todo'
0242             if strcmpi(pvalue,'on')
0243                 options.todo = 1;
0244             elseif strcmpi(pvalue,'off')
0245                 options.todo = 0;
0246             else
0247                 error(sprintf(msgInvalidPair,pname));
0248             end
0249         case 'load'
0250             if ischar(pvalue)
0251                 if exist(pvalue) == 7 % directory provided
0252                     pvalue = fullfile(pvalue,'m2html.mat');
0253                 end         
0254                 try
0255                     load(pvalue);
0256                 catch
0257                     error(sprintf('Unable to load %s.', pvalue));
0258                 end
0259                 options.load = 1;
0260                 [dummy options.template] = fileparts(options.template);
0261             else
0262                 error(sprintf(msgInvalidPair,pname));
0263             end
0264         case 'save'
0265             if strcmpi(pvalue,'on')
0266                 options.save = 1;
0267             elseif strcmpi(pvalue,'off')
0268                 options.save = 0;
0269             else
0270                 error(sprintf(msgInvalidPair,pname));
0271             end
0272         case 'search'
0273             if strcmpi(pvalue,'on')
0274                 options.search = 1;
0275             elseif strcmpi(pvalue,'off')
0276                 options.search = 0;
0277             else
0278                 error(sprintf(msgInvalidPair,pname));
0279             end
0280         case 'helptocxml'
0281             if strcmpi(pvalue,'on')
0282                 options.helptocxml = 1;
0283             elseif strcmpi(pvalue,'off')
0284                 options.helptocxml = 0;
0285             else
0286                 error(sprintf(msgInvalidPair,pname));
0287             end
0288         case 'indexfile'
0289             if ischar(pvalue)
0290                 options.indexFile = pvalue;
0291             else
0292                 error(sprintf(msgInvalidPair,pname));
0293             end
0294         case 'extension'
0295             if ischar(pvalue) & pvalue(1) == '.'
0296                 options.extension = pvalue;
0297             else
0298                 error(sprintf(msgInvalidPair,pname));
0299             end
0300         case 'template'
0301             if ischar(pvalue)
0302                 options.template = pvalue;
0303             else
0304                 error(sprintf(msgInvalidPair,pname));
0305             end
0306         case 'ignoreddir'
0307             if iscellstr(pvalue)
0308                 options.ignoredDir = pvalue;
0309             elseif ischar(pvalue)
0310                 options.ignoredDir = cellstr(pvalue);
0311             else
0312                 error(sprintf(msgInvalidPair,pname));
0313             end
0314         case 'language'
0315             if ischar(pvalue)
0316                 options.language = pvalue;
0317             else
0318                 error(sprintf(msgInvalidPair,pname));
0319             end
0320         otherwise
0321             error(['Invalid parameter: ''' pname '''.']);
0322     end
0323 end
0324 
0325 %-------------------------------------------------------------------------------
0326 %- Get template files location
0327 %-------------------------------------------------------------------------------
0328 s = fileparts(which(mfilename));
0329 options.template = fullfile(s,'templates',options.template);
0330 if exist(options.template) ~= 7
0331     error('[Template] Unknown template.');
0332 end
0333 
0334 %-------------------------------------------------------------------------------
0335 %- Get list of M-files
0336 %-------------------------------------------------------------------------------
0337 if ~options.load
0338     if strcmp(options.mFiles,'.')
0339         d = dir(pwd); d = {d([d.isdir]).name};
0340         options.mFiles = {d{~ismember(d,{'.' '..' options.ignoredDir{:}})}};
0341     end
0342     mfiles = getmfiles(options.mFiles,{},options.recursive,options.ignoredDir);
0343     if ~length(mfiles), fprintf('Nothing to be done.\n'); return; end
0344     if options.verbose,
0345         fprintf('Found %d M-files.\n',length(mfiles));
0346     end
0347     mfiles = sort(mfiles); % sort list of M-files in dictionary order
0348 end
0349 
0350 %-------------------------------------------------------------------------------
0351 %- Get list of (unique) directories and (unique) names
0352 %-------------------------------------------------------------------------------
0353 if ~options.load
0354     mdirs = {};
0355     names = {};
0356     for i=1:length(mfiles)
0357         [mdirs{i}, names{i}] = fileparts(mfiles{i});
0358         if isempty(mdirs{i}), mdirs{i} = '.'; end
0359     end
0360 
0361     mdir = unique(mdirs);
0362     if options.verbose,
0363         fprintf('Found %d unique Matlab directories.\n',length(mdir));
0364     end
0365 
0366     name = names;
0367     %name = unique(names); % output is sorted
0368     %if options.verbose,
0369     %    fprintf('Found %d unique Matlab files.\n',length(name));
0370     %end
0371 end
0372 
0373 %-------------------------------------------------------------------------------
0374 %- Create output directory, if necessary
0375 %-------------------------------------------------------------------------------
0376 if isempty(dir(options.htmlDir))                                               
0377     %- Create the top level output directory
0378     if options.verbose                                                         
0379         fprintf('Creating directory %s...\n',options.htmlDir);                 
0380     end                                                                        
0381     if options.htmlDir(end) == filesep,                                        
0382         options.htmlDir(end) = [];                                             
0383     end                                                                        
0384     [pathdir, namedir] = fileparts(options.htmlDir);                           
0385     if isempty(pathdir)                                                        
0386         [status, msg] = mkdir(escapeblank(namedir));                                        
0387     else                                                                       
0388         [status, msg] = mkdir(escapeblank(pathdir), escapeblank(namedir));                               
0389     end                                                                        
0390     if ~status, error(msg); end                                                                
0391 end                                                                            
0392 
0393 %-------------------------------------------------------------------------------
0394 %- Get synopsis, H1 line, script/function, subroutines, cross-references, todo
0395 %-------------------------------------------------------------------------------
0396 if ~options.load
0397     synopsis   = cell(size(mfiles));
0398     h1line     = cell(size(mfiles));
0399     subroutine = cell(size(mfiles));
0400     hrefs      = sparse(length(mfiles), length(mfiles));
0401     todo       = struct('mfile',[], 'line',[], 'comment',{{}});
0402     ismex      = zeros(length(mfiles), length(mexexts));
0403     statlist   = {};
0404     statinfo   = sparse(1,length(mfiles));
0405     kw         = cell(size(mfiles));
0406     freq       = cell(size(mfiles));
0407 
0408     for i=1:length(mfiles)
0409         if options.verbose
0410             fprintf('Processing file %s...',mfiles{i});
0411         end
0412         s = mfileparse(mfiles{i}, mdirs, names, options);
0413         synopsis{i}   = s.synopsis;
0414         h1line{i}     = s.h1line;
0415         subroutine{i} = s.subroutine;
0416         hrefs(i,:)    = s.hrefs;
0417         todo.mfile    = [todo.mfile repmat(i,1,length(s.todo.line))];
0418         todo.line     = [todo.line s.todo.line];
0419         todo.comment  = {todo.comment{:} s.todo.comment{:}};
0420         ismex(i,:)    = s.ismex;
0421         if options.search
0422             if options.verbose, fprintf('search...'); end
0423             [kw{i}, freq{i}] = searchindex(mfiles{i});
0424             statlist = union(statlist, kw{i});
0425         end
0426         if options.verbose, fprintf('\n'); end
0427     end
0428     hrefs = hrefs > 0;
0429     if options.search
0430         if options.verbose
0431             fprintf('Creating the search index...');
0432         end
0433         statinfo = sparse(length(statlist),length(mfiles));
0434         for i=1:length(mfiles)
0435             i1 = find(ismember(statlist, kw{i}));
0436             i2 = repmat(i,1,length(i1));
0437             if ~isempty(i1)
0438                 statinfo(sub2ind(size(statinfo),i1,i2)) = freq{i};
0439             end
0440             if options.verbose, fprintf('.'); end
0441         end
0442         clear kw freq;
0443         if options.verbose, fprintf('\n'); end
0444     end
0445 end
0446 
0447 %-------------------------------------------------------------------------------
0448 %- Save M-filenames and cross-references for further analysis
0449 %-------------------------------------------------------------------------------
0450 matfilesave = 'm2html.mat';
0451 
0452 if options.save
0453     if options.verbose
0454         fprintf('Saving MAT file %s...\n',matfilesave);
0455     end
0456     save(fullfile(options.htmlDir,matfilesave), ...
0457         'mfiles', 'names', 'mdirs', 'name', 'mdir', 'options', ...
0458         'hrefs', 'synopsis', 'h1line', 'subroutine', 'todo', 'ismex', ...
0459         'statlist', 'statinfo');
0460 end
0461 
0462 %-------------------------------------------------------------------------------
0463 %- Setup the output directories
0464 %-------------------------------------------------------------------------------
0465 for i=1:length(mdir)
0466     if exist(fullfile(options.htmlDir,mdir{i})) ~= 7
0467         ldir = splitpath(mdir{i});
0468         for j=1:length(ldir)
0469             if exist(fullfile(options.htmlDir,ldir{1:j})) ~= 7
0470                 %- Create the output directory
0471                 if options.verbose
0472                     fprintf('Creating directory %s...\n',...
0473                             fullfile(options.htmlDir,ldir{1:j}));
0474                 end
0475                 if j == 1
0476                     [status, msg] = mkdir(escapeblank(options.htmlDir), ...
0477                         escapeblank(ldir{1}));
0478                 else
0479                     [status, msg] = mkdir(escapeblank(options.htmlDir), ...
0480                         escapeblank(fullfile(ldir{1:j})));
0481                 end
0482                 error(msg);
0483             end
0484         end
0485     end
0486 end
0487 
0488 %-------------------------------------------------------------------------------
0489 %- Write the master index file
0490 %-------------------------------------------------------------------------------
0491 tpl_master = 'master.tpl';
0492 tpl_master_identifier_nbyline = 4;
0493 php_search = 'search.php';
0494 dotbase = 'graph';
0495 
0496 %- Create the HTML template
0497 tpl = template(options.template,'remove');
0498 tpl = set(tpl,'file','TPL_MASTER',tpl_master);
0499 tpl = set(tpl,'block','TPL_MASTER','rowdir','rowdirs');
0500 tpl = set(tpl,'block','TPL_MASTER','idrow','idrows');
0501 tpl = set(tpl,'block','idrow','idcolumn','idcolumns');
0502 tpl = set(tpl,'block','TPL_MASTER','search','searchs');
0503 tpl = set(tpl,'block','TPL_MASTER','graph','graphs');
0504 
0505 %- Open for writing the HTML master index file
0506 curfile = fullfile(options.htmlDir,[options.indexFile options.extension]);
0507 if options.verbose
0508     fprintf('Creating HTML file %s...\n',curfile);
0509 end
0510 fid = openfile(curfile,'w');
0511 
0512 %- Set some template variables
0513 tpl = set(tpl,'var','DATE',[datestr(now,8) ' ' datestr(now,1) ' ' ...
0514                             datestr(now,13)]);
0515 tpl = set(tpl,'var','MASTERPATH', './');
0516 tpl = set(tpl,'var','DIRS', sprintf('%s ',mdir{:}));
0517 
0518 %- Print list of unique directories
0519 for i=1:length(mdir)
0520     tpl = set(tpl,'var','L_DIR',...
0521               fullurl(mdir{i},[options.indexFile options.extension]));
0522     tpl = set(tpl,'var','DIR',mdir{i});
0523     tpl = parse(tpl,'rowdirs','rowdir',1);
0524 end
0525 
0526 %- Print full list of M-files (sorted by column)
0527 [sortnames, ind] = sort(names);
0528 m_mod = mod(length(sortnames), tpl_master_identifier_nbyline);
0529 ind = [ind zeros(1,tpl_master_identifier_nbyline-m_mod)];
0530 m_floor = floor(length(ind) / tpl_master_identifier_nbyline);
0531 ind = reshape(ind,m_floor,tpl_master_identifier_nbyline)';
0532 
0533 for i=1:prod(size(ind))
0534     if ind(i)
0535         tpl = set(tpl,'var','L_IDNAME',...
0536             fullurl(mdirs{ind(i)},[names{ind(i)} options.extension]));
0537         tpl = set(tpl,'var','T_IDNAME',mdirs{ind(i)});
0538         tpl = set(tpl,'var','IDNAME',names{ind(i)});
0539         tpl = parse(tpl,'idcolumns','idcolumn',1);
0540     else
0541         tpl = set(tpl,'var','L_IDNAME','');
0542         tpl = set(tpl,'var','T_IDNAME','');
0543         tpl = set(tpl,'var','IDNAME','');
0544         tpl = parse(tpl,'idcolumns','idcolumn',1);
0545     end
0546     if mod(i,tpl_master_identifier_nbyline) == 0
0547         tpl = parse(tpl,'idrows','idrow',1);
0548         tpl = set(tpl,'var','idcolumns','');
0549     end
0550 end
0551 
0552 %- Add a search form if necessary
0553 tpl = set(tpl,'var','searchs','');
0554 if options.search
0555     tpl = set(tpl,'var','PHPFILE',php_search);
0556     tpl = parse(tpl,'searchs','search',1);
0557 end
0558 
0559 %- Link to a full dependency graph, if necessary
0560 tpl = set(tpl,'var','graphs','');
0561 if options.graph & options.globalHypertextLinks & length(mdir) > 1
0562     tpl = set(tpl,'var','LGRAPH',[dotbase options.extension]);
0563     tpl = parse(tpl,'graphs','graph',1);
0564 end
0565 
0566 %- Print the template in the HTML file
0567 tpl = parse(tpl,'OUT','TPL_MASTER');
0568 fprintf(fid,'%s',get(tpl,'OUT'));
0569 fclose(fid);
0570 
0571 %-------------------------------------------------------------------------------
0572 %- Copy template files (CSS, images, ...)
0573 %-------------------------------------------------------------------------------
0574 % Get list of files
0575 d = dir(options.template);
0576 d = {d(~[d.isdir]).name};
0577 % Copy files
0578 for i=1:length(d)
0579     [p, n, ext] = fileparts(d{i});
0580     if ~strcmp(ext,'.tpl') ... % do not copy .tpl files
0581        & ~strcmp([n ext],'Thumbs.db') % do not copy this Windows generated file
0582         if isempty(dir(fullfile(options.htmlDir,d{i})))
0583             if options.verbose
0584                 fprintf('Copying template file %s...\n',d{i});
0585             end
0586             %- there is a bug with <copyfile> in Matlab 6.5 :
0587             %   http://www.mathworks.com/support/solutions/data/1-1B5JY.html
0588             %- and <copyfile> does not overwrite files even if newer...
0589             [status, errmsg] = copyfile(fullfile(options.template,d{i}),...
0590                                         options.htmlDir);
0591             %- If you encounter this bug, please uncomment one of the following lines
0592             % eval(['!cp -rf ' fullfile(options.template,d{i}) ' ' options.htmlDir]);
0593             % eval(['!copy ' fullfile(options.template,d{i}) ' ' options.htmlDir]);
0594             % status = 1;
0595             if ~status
0596                 if ~isempty(errmsg)
0597                     error(errmsg)
0598                 else
0599                     warning(sprintf(['<copyfile> failed to do its job...\n' ...
0600                 'This is a known bug in Matlab 6.5 (R13).\n' ...
0601                 'See http://www.mathworks.com/support/solutions/data/1-1B5JY.html']));
0602                 end
0603             end
0604         end
0605     end
0606 end
0607 
0608 %-------------------------------------------------------------------------------
0609 %- Search engine (index file and PHP script)
0610 %-------------------------------------------------------------------------------
0611 tpl_search = 'search.tpl';
0612 idx_search = 'search.idx';
0613 
0614 % TODO % improving the fill in of 'statlist' and 'statinfo'
0615 % TODO % improving the search template file and update the CSS file
0616 
0617 if options.search
0618     %- Write the search index file in output directory
0619     if options.verbose
0620         fprintf('Creating Search Index file %s...\n', idx_search);
0621     end
0622     docinfo = cell(length(mfiles),2);
0623     for i=1:length(mfiles)
0624         docinfo{i,1} = h1line{i};
0625         docinfo{i,2} = fullurl(mdirs{i}, [names{i} options.extension]);
0626     end
0627     doxywrite(fullfile(options.htmlDir,idx_search),statlist,statinfo,docinfo);
0628     
0629     %- Create the PHP template
0630     tpl = template(options.template,'remove');
0631     tpl = set(tpl,'file','TPL_SEARCH',tpl_search);
0632     
0633     %- Open for writing the PHP search script
0634     curfile = fullfile(options.htmlDir, php_search); 
0635     if options.verbose
0636         fprintf('Creating PHP script %s...\n',curfile);
0637     end
0638     fid = openfile(curfile,'w');
0639     
0640     %- Set template fields
0641     tpl = set(tpl,'var','INDEX',[options.indexFile options.extension]);
0642     tpl = set(tpl,'var','MASTERPATH','./');
0643     tpl = set(tpl,'var','DATE',[datestr(now,8) ' ' datestr(now,1) ' ' ...
0644                                 datestr(now,13)]);
0645     tpl = set(tpl,'var','IDXFILE',idx_search);
0646     tpl = set(tpl,'var','PHPFILE',php_search);
0647     
0648     %- Print the template in the HTML file
0649     tpl = parse(tpl,'OUT','TPL_SEARCH');
0650     fprintf(fid,'%s',get(tpl,'OUT'));
0651     fclose(fid);
0652 end
0653 
0654 %-------------------------------------------------------------------------------
0655 %- Create <helptoc.xml> needed to display hierarchical entries in Contents panel
0656 %-------------------------------------------------------------------------------
0657 % See http://www.mathworks.com/access/helpdesk/help/techdoc/matlab_env/guiref16.html
0658 % and http://www.mathworks.com/support/solutions/data/1-18U6Q.html?solution=1-18U6Q
0659 
0660 % TODO % display directories in TOC hierarchically instead of linearly
0661 if options.helptocxml
0662     curfile = fullfile(options.htmlDir, 'helptoc.xml');
0663     if options.verbose
0664         fprintf('Creating XML Table-Of-Content %s...\n',curfile);
0665     end
0666     fid = openfile(curfile,'w');
0667     fprintf(fid,'<?xml version=''1.0'' encoding=''ISO-8859-1'' ?>\n');
0668     fprintf(fid,'<!-- $Date: %s $ -->\n\n', datestr(now,31));
0669     fprintf(fid,'<toc version="1.0">\n\n');
0670     fprintf(fid,['<tocitem target="%s" ',...
0671         'image="$toolbox/matlab/icons/book_mat.gif">%s\n'], ...
0672         [options.indexFile options.extension],'Toolbox');
0673     for i=1:length(mdir)
0674         fprintf(fid,['<tocitem target="%s" ',...
0675             'image="$toolbox/matlab/icons/reficon.gif">%s\n'], ...
0676             fullfile(mdir{i}, ...
0677                 [options.indexFile options.extension]),mdir{i});
0678         if options.graph
0679             fprintf(fid,['\t<tocitem target="%s" ',...
0680             'image="$toolbox/matlab/icons/simulinkicon.gif">%s</tocitem>\n'], ...
0681                 fullfile(mdir{i},...
0682                 [dotbase options.extension]),'Dependency Graph');
0683         end
0684         if options.todo
0685             if ~isempty(intersect(find(strcmp(mdir{i},mdirs)),todo.mfile))
0686                 fprintf(fid,['\t<tocitem target="%s" ',...
0687                 'image="$toolbox/matlab/icons/demoicon.gif">%s</tocitem>\n'], ...
0688                     fullfile(mdir{i},...
0689                     ['todo' options.extension]),'Todo list');
0690             end
0691         end
0692         for j=1:length(mdirs)
0693             if strcmp(mdirs{j},mdir{i})
0694                 curfile = fullfile(mdir{i},...
0695                     [names{j} options.extension]);
0696                 fprintf(fid,'\t<tocitem target="%s">%s</tocitem>\n', ...
0697                     curfile,names{j});
0698             end
0699         end
0700         fprintf(fid,'</tocitem>\n');
0701     end
0702     fprintf(fid,'</tocitem>\n');
0703     fprintf(fid,'\n</toc>\n');
0704     fclose(fid);
0705 end
0706 
0707 %-------------------------------------------------------------------------------
0708 %- Write an index for each output directory
0709 %-------------------------------------------------------------------------------
0710 tpl_mdir = 'mdir.tpl';
0711 tpl_mdir_link = '<a href="%s">%s</a>';
0712 %dotbase defined earlier
0713 
0714 %- Create the HTML template
0715 tpl = template(options.template,'remove');
0716 tpl = set(tpl,'file','TPL_MDIR',tpl_mdir);
0717 tpl = set(tpl,'block','TPL_MDIR','row-m','rows-m');
0718 tpl = set(tpl,'block','row-m','mexfile','mex');
0719 tpl = set(tpl,'block','TPL_MDIR','othermatlab','other');
0720 tpl = set(tpl,'block','othermatlab','row-other','rows-other');
0721 tpl = set(tpl,'block','TPL_MDIR','subfolder','subfold');
0722 tpl = set(tpl,'block','subfolder','subdir','subdirs');
0723 tpl = set(tpl,'block','TPL_MDIR','todolist','todolists');
0724 tpl = set(tpl,'block','TPL_MDIR','graph','graphs');
0725 tpl = set(tpl,'var','DATE',[datestr(now,8) ' ' datestr(now,1) ' ' ...
0726                             datestr(now,13)]);
0727 
0728 for i=1:length(mdir)
0729     %- Open for writing each output directory index file
0730     curfile = fullfile(options.htmlDir,mdir{i},...
0731                        [options.indexFile options.extension]);
0732     if options.verbose
0733         fprintf('Creating HTML file %s...\n',curfile);
0734     end
0735     fid = openfile(curfile,'w');
0736 
0737     %- Set template fields
0738     tpl = set(tpl,'var','INDEX',     [options.indexFile options.extension]);
0739     tpl = set(tpl,'var','MASTERPATH',backtomaster(mdir{i}));
0740     tpl = set(tpl,'var','MDIR',      mdir{i});
0741     
0742     %- Display Matlab m-files, their H1 line and their Mex status
0743     tpl = set(tpl,'var','rows-m','');
0744     for j=1:length(mdirs)
0745         if strcmp(mdirs{j},mdir{i})
0746             tpl = set(tpl,'var','L_NAME', [names{j} options.extension]);
0747             tpl = set(tpl,'var','NAME',   names{j});
0748             tpl = set(tpl,'var','H1LINE', h1line{j});
0749             if any(ismex(j,:))
0750                 tpl = parse(tpl,'mex','mexfile');
0751             else
0752                 tpl = set(tpl,'var','mex','');
0753             end
0754             tpl = parse(tpl,'rows-m','row-m',1);
0755         end
0756     end
0757     
0758     %- Display other Matlab-specific files (.mat,.mdl,.p)
0759     tpl = set(tpl,'var','other','');
0760     tpl = set(tpl,'var','rows-other','');
0761     w = what(mdir{i}); w = w(1);
0762     w = {w.mat{:} w.mdl{:} w.p{:}};
0763     for j=1:length(w)
0764         tpl = set(tpl,'var','OTHERFILE',w{j});
0765         tpl = parse(tpl,'rows-other','row-other',1);
0766     end
0767     if ~isempty(w)
0768         tpl = parse(tpl,'other','othermatlab');
0769     end
0770     
0771     %- Display subsequent directories and classes
0772     tpl = set(tpl,'var','subdirs','');
0773     tpl = set(tpl,'var','subfold','');
0774     d = dir(mdir{i});
0775     d = {d([d.isdir]).name};
0776     d = {d{~ismember(d,{'.' '..' options.ignoredDir{:}})}};
0777     for j=1:length(d)
0778         if ismember(fullfile(mdir{i},d{j}),mdir)
0779             tpl = set(tpl,'var','SUBDIRECTORY',...
0780                 sprintf(tpl_mdir_link,...
0781                 fullurl(d{j},[options.indexFile options.extension]),d{j}));
0782         else
0783             tpl = set(tpl,'var','SUBDIRECTORY',d{j});
0784         end
0785         tpl = parse(tpl,'subdirs','subdir',1);
0786     end
0787     if ~isempty(d)
0788         tpl = parse(tpl,'subfold','subfolder');
0789     end
0790     
0791     %- Link to the TODO list if necessary
0792     tpl = set(tpl,'var','todolists','');
0793     if options.todo
0794         if ~isempty(intersect(find(strcmp(mdir{i},mdirs)),todo.mfile))
0795             tpl = set(tpl,'var','LTODOLIST',['todo' options.extension]);
0796             tpl = parse(tpl,'todolists','todolist',1);
0797         end
0798     end
0799     
0800     %- Link to the dependency graph if necessary
0801     tpl = set(tpl,'var','graphs','');
0802     if options.graph
0803         tpl = set(tpl,'var','LGRAPH',[dotbase options.extension]);
0804         tpl = parse(tpl,'graphs','graph',1);
0805     end
0806     
0807     %- Print the template in the HTML file
0808     tpl = parse(tpl,'OUT','TPL_MDIR');
0809     fprintf(fid,'%s',get(tpl,'OUT'));
0810     fclose(fid);
0811 end
0812 
0813 %-------------------------------------------------------------------------------
0814 %- Write a TODO list file for each output directory, if necessary
0815 %-------------------------------------------------------------------------------
0816 tpl_todo = 'todo.tpl';
0817 
0818 if options.todo
0819     %- Create the HTML template
0820     tpl = template(options.template,'remove');
0821     tpl = set(tpl,'file','TPL_TODO',tpl_todo);
0822     tpl = set(tpl,'block','TPL_TODO','filelist','filelists');
0823     tpl = set(tpl,'block','filelist','row','rows');
0824     tpl = set(tpl,'var','DATE',[datestr(now,8) ' ' datestr(now,1) ' ' ...
0825                                 datestr(now,13)]);
0826 
0827     for i=1:length(mdir)
0828         mfilestodo = intersect(find(strcmp(mdir{i},mdirs)),todo.mfile);
0829         if ~isempty(mfilestodo)
0830             %- Open for writing each TODO list file
0831             curfile = fullfile(options.htmlDir,mdir{i},...
0832                                ['todo' options.extension]);
0833             if options.verbose
0834                 fprintf('Creating HTML file %s...\n',curfile);
0835             end
0836             fid = openfile(curfile,'w');
0837             
0838             %- Set template fields
0839             tpl = set(tpl,'var','INDEX',[options.indexFile options.extension]);
0840             tpl = set(tpl,'var','MASTERPATH', backtomaster(mdir{i}));
0841             tpl = set(tpl,'var','MDIR',       mdir{i});
0842             tpl = set(tpl,'var','filelists',  '');
0843     
0844             for k=1:length(mfilestodo)
0845                 tpl = set(tpl,'var','MFILE',names{mfilestodo(k)});
0846                 tpl = set(tpl,'var','rows','');
0847                 nbtodo = find(todo.mfile == mfilestodo(k));
0848                 for l=1:length(nbtodo)
0849                     tpl = set(tpl,'var','L_NBLINE',...
0850                         [names{mfilestodo(k)} ...
0851                             options.extension ...
0852                             '#l' num2str(todo.line(nbtodo(l)))]);
0853                     tpl = set(tpl,'var','NBLINE',num2str(todo.line(nbtodo(l))));
0854                     tpl = set(tpl,'var','COMMENT',todo.comment{nbtodo(l)});
0855                     tpl = parse(tpl,'rows','row',1);
0856                 end
0857                 tpl = parse(tpl,'filelists','filelist',1);
0858             end
0859     
0860             %- Print the template in the HTML file
0861             tpl = parse(tpl,'OUT','TPL_TODO');
0862             fprintf(fid,'%s',get(tpl,'OUT'));
0863             fclose(fid);
0864         end
0865     end
0866 end
0867 
0868 %-------------------------------------------------------------------------------
0869 %- Create dependency graphs using GraphViz, if requested
0870 %-------------------------------------------------------------------------------
0871 tpl_graph = 'graph.tpl';
0872 % You may have to modify the following line with Matlab7 (R14) to specify
0873 % the full path to where GraphViz is installed
0874 dot_exec  = 'dot';
0875 %dotbase defined earlier
0876 
0877 if options.graph
0878     %- Create the HTML template
0879     tpl = template(options.template,'remove');
0880     tpl = set(tpl,'file','TPL_GRAPH',tpl_graph);
0881     tpl = set(tpl,'var','DATE',[datestr(now,8) ' ' datestr(now,1) ' ' ...
0882                                 datestr(now,13)]);
0883     
0884     %- Create a full dependency graph for all directories if possible
0885     if options.globalHypertextLinks & length(mdir) > 1
0886         mdotfile = fullfile(options.htmlDir,[dotbase '.dot']);
0887         if options.verbose
0888             fprintf('Creating full dependency graph %s...',mdotfile);
0889         end
0890         mdot({hrefs, names, options, mdirs}, mdotfile); %mfiles
0891         calldot(dot_exec, mdotfile, ...
0892                 fullfile(options.htmlDir,[dotbase '.map']), ...
0893                 fullfile(options.htmlDir,[dotbase '.png']));
0894         if options.verbose, fprintf('\n'); end
0895         fid = openfile(fullfile(options.htmlDir, [dotbase options.extension]),'w');
0896         tpl = set(tpl,'var','INDEX',[options.indexFile options.extension]);
0897         tpl = set(tpl,'var','MASTERPATH', './');
0898         tpl = set(tpl,'var','MDIR',       'the whole toolbox');
0899         tpl = set(tpl,'var','GRAPH_IMG',  [dotbase '.png']);
0900         try % if <dot> failed...
0901             fmap = openfile(fullfile(options.htmlDir,[dotbase '.map']),'r');
0902             tpl = set(tpl,'var','GRAPH_MAP',  fscanf(fmap,'%c'));
0903             fclose(fmap);
0904         end
0905         tpl = parse(tpl,'OUT','TPL_GRAPH');
0906         fprintf(fid,'%s', get(tpl,'OUT'));
0907         fclose(fid);
0908     end
0909     
0910     %- Create a dependency graph for each output directory
0911     for i=1:length(mdir)
0912         mdotfile = fullfile(options.htmlDir,mdir{i},[dotbase '.dot']);
0913         if options.verbose
0914             fprintf('Creating dependency graph %s...',mdotfile);
0915         end
0916         ind = find(strcmp(mdirs,mdir{i}));
0917         href1 = zeros(length(ind),length(hrefs));
0918         for j=1:length(hrefs), href1(:,j) = hrefs(ind,j); end
0919         href2 = zeros(length(ind));
0920         for j=1:length(ind), href2(j,:) = href1(j,ind); end
0921         mdot({href2, {names{ind}}, options}, mdotfile); %{mfiles{ind}}
0922         calldot(dot_exec, mdotfile, ...
0923                 fullfile(options.htmlDir,mdir{i},[dotbase '.map']), ...
0924                 fullfile(options.htmlDir,mdir{i},[dotbase '.png']));
0925         if options.verbose, fprintf('\n'); end
0926         fid = openfile(fullfile(options.htmlDir,mdir{i},...
0927             [dotbase options.extension]),'w');
0928         tpl = set(tpl,'var','INDEX',[options.indexFile options.extension]);
0929         tpl = set(tpl,'var','MASTERPATH', backtomaster(mdir{i}));
0930         tpl = set(tpl,'var','MDIR',       mdir{i});
0931         tpl = set(tpl,'var','GRAPH_IMG',  [dotbase '.png']);
0932         try % if <dot> failed, no '.map' file has been created
0933             fmap = openfile(fullfile(options.htmlDir,mdir{i},[dotbase '.map']),'r');
0934             tpl = set(tpl,'var','GRAPH_MAP',  fscanf(fmap,'%c'));
0935             fclose(fmap);
0936         end
0937         tpl = parse(tpl,'OUT','TPL_GRAPH');
0938         fprintf(fid,'%s', get(tpl,'OUT'));
0939         fclose(fid);
0940     end
0941 end
0942 
0943 %-------------------------------------------------------------------------------
0944 %- Write an HTML file for each M-file
0945 %-------------------------------------------------------------------------------
0946 %- List of Matlab keywords (output from iskeyword)
0947 matlabKeywords = {'break', 'case', 'catch', 'continue', 'elseif', 'else', ...
0948                   'end', 'for', 'function', 'global', 'if', 'otherwise', ...
0949                   'persistent', 'return', 'switch', 'try', 'while'};
0950                   %'keyboard', 'pause', 'eps', 'NaN', 'Inf'
0951 
0952 tpl_mfile = 'mfile.tpl';
0953 
0954 tpl_mfile_code     = '<a href="%s" class="code" title="%s">%s</a>';
0955 tpl_mfile_keyword  = '<span class="keyword">%s</span>';
0956 tpl_mfile_comment  = '<span class="comment">%s</span>';
0957 tpl_mfile_string   = '<span class="string">%s</span>';
0958 tpl_mfile_aname    = '<a name="%s" href="#_subfunctions" class="code">%s</a>';
0959 tpl_mfile_line     = '%04d %s\n';
0960 
0961 %- Delimiters used in strtok: some of them may be useless (% " .), removed '.'
0962 strtok_delim = sprintf(' \t\n\r(){}[]<>+-*~!|\\@&/,:;="''%%');
0963 
0964 %- Create the HTML template
0965 tpl = template(options.template,'remove');
0966 tpl = set(tpl,'file','TPL_MFILE',tpl_mfile);
0967 tpl = set(tpl,'block','TPL_MFILE','pathline','pl');
0968 tpl = set(tpl,'block','TPL_MFILE','mexfile','mex');
0969 tpl = set(tpl,'block','TPL_MFILE','script','scriptfile');
0970 tpl = set(tpl,'block','TPL_MFILE','crossrefcall','crossrefcalls');
0971 tpl = set(tpl,'block','TPL_MFILE','crossrefcalled','crossrefcalleds');
0972 tpl = set(tpl,'block','TPL_MFILE','subfunction','subf');
0973 tpl = set(tpl,'block','subfunction','onesubfunction','onesubf');
0974 tpl = set(tpl,'block','TPL_MFILE','source','thesource');
0975 tpl = set(tpl,'block','TPL_MFILE','download','downloads');
0976 tpl = set(tpl,'var','DATE',[datestr(now,8) ' ' datestr(now,1) ' ' ...
0977                             datestr(now,13)]);
0978 
0979 nblinetot = 0;
0980 for i=1:length(mdir)
0981     for j=1:length(mdirs)
0982         if strcmp(mdirs{j},mdir{i})
0983         
0984             curfile = fullfile(options.htmlDir,mdir{i},...
0985                                [names{j} options.extension]);
0986                                
0987             %- Copy M-file for download, if necessary
0988             if options.download
0989                 if options.verbose
0990                     fprintf('Copying M-file %s.m to %s...\n',names{j},...
0991                         fullfile(options.htmlDir,mdir{i}));
0992                 end
0993                 [status, errmsg] = copyfile(mfiles{j},...
0994                     fullfile(options.htmlDir,mdir{i}));
0995                 error(errmsg);
0996             end
0997             
0998             %- Open for writing the HTML file
0999             if options.verbose
1000                 fprintf('Creating HTML file %s...\n',curfile);
1001             end
1002             fid = openfile(curfile,'w');
1003             if strcmp(names{j},options.indexFile)
1004                 fprintf(['Warning: HTML index file %s will be ' ...
1005                         'overwritten by Matlab function %s.\n'], ...
1006                         [options.indexFile options.extension], mfiles{j});
1007             end
1008             
1009             %- Open for reading the M-file
1010             fid2 = openfile(mfiles{j},'r');
1011             
1012             %- Set some template fields
1013             tpl = set(tpl,'var','INDEX', [options.indexFile options.extension]);
1014             tpl = set(tpl,'var','MASTERPATH',       backtomaster(mdir{i}));
1015             tpl = set(tpl,'var','MDIR',             mdirs{j});
1016             tpl = set(tpl,'var','NAME',             names{j});
1017             tpl = set(tpl,'var','H1LINE',           entity(h1line{j}));
1018             tpl = set(tpl,'var','scriptfile',       '');
1019             if isempty(synopsis{j})
1020                 tpl = set(tpl,'var','SYNOPSIS',get(tpl,'var','script'));
1021             else
1022                 tpl = set(tpl,'var','SYNOPSIS', synopsis{j});
1023             end
1024             s = splitpath(mdir{i});
1025             tpl = set(tpl,'var','pl','');
1026             for k=1:length(s)
1027                 c = cell(1,k); for l=1:k, c{l} = filesep; end
1028                 cpath = {s{1:k};c{:}}; cpath = [cpath{:}];
1029                 if ~isempty(cpath), cpath = cpath(1:end-1); end
1030                 if ismember(cpath,mdir)
1031                     tpl = set(tpl,'var','LPATHDIR',[repmat('../',...
1032                         1,length(s)-k) options.indexFile options.extension]);
1033                 else
1034                     tpl = set(tpl,'var','LPATHDIR','#');
1035                 end
1036                 tpl = set(tpl,'var','PATHDIR',s{k});
1037                 tpl = parse(tpl,'pl','pathline',1);
1038             end
1039             
1040             %- Handle mex files
1041             tpl = set(tpl,'var','mex', '');
1042             samename = dir(fullfile(mdir{i},[names{j}    '.*']));
1043             samename = {samename.name};
1044             tpl = set(tpl,'var','MEXTYPE', 'mex');
1045             for k=1:length(samename)
1046                 [dummy, dummy, ext] = fileparts(samename{k});
1047                 switch ext
1048                     case '.c'
1049                         tpl = set(tpl,'var','MEXTYPE', 'c');
1050                     case {'.cpp' '.c++' '.cxx' '.C'}
1051                         tpl = set(tpl,'var','MEXTYPE', 'c++');
1052                     case {'.for' '.f' '.FOR' '.F'}
1053                         tpl = set(tpl,'var','MEXTYPE', 'fortran');
1054                     otherwise
1055                         %- Unknown mex file source
1056                 end
1057             end
1058             [exts, platform] = mexexts;
1059             mexplatforms = sprintf('%s, ',platform{find(ismex(j,:))});
1060             if ~isempty(mexplatforms)
1061                 tpl = set(tpl,'var','PLATFORMS', mexplatforms(1:end-2));
1062                 tpl = parse(tpl,'mex','mexfile');
1063             end
1064             
1065             %- Set description template field
1066             descr = '';
1067             flagsynopcont = 0;
1068             flag_seealso  = 0;
1069             while 1
1070                 tline = fgets(fid2);
1071                 if ~ischar(tline), break, end
1072                 tline = entity(fliplr(deblank(fliplr(tline))));
1073                 %- Synopsis line
1074                 if ~isempty(strmatch('function',tline))
1075                     if ~isempty(strmatch('...',fliplr(deblank(tline))))
1076                         flagsynopcont = 1;
1077                     end
1078                 %- H1 line and description
1079                 elseif ~isempty(strmatch('%',tline))
1080                     %- Hypertext links on the "See also" line
1081                     ind = findstr(lower(tline),'see also');
1082                     if ~isempty(ind) | flag_seealso
1083                         %- "See also" only in files in the same directory
1084                         indsamedir = find(strcmp(mdirs{j},mdirs));
1085                         hrefnames = {names{indsamedir}};
1086                         r = deblank(tline);
1087                         flag_seealso = 1; %(r(end) == ',');
1088                         tline = '';
1089                         while 1
1090                             [t,r,q] = strtok(r,sprintf(' \t\n\r.,;%%'));
1091                             tline = [tline q];
1092                             if isempty(t), break, end;
1093                             ii = strcmpi(hrefnames,t);
1094                             if any(ii)
1095                                 jj = find(ii);
1096                                 tline = [tline sprintf(tpl_mfile_code,...
1097                                     [hrefnames{jj(1)} options.extension],...
1098                                     synopsis{indsamedir(jj(1))},t)];
1099                             else
1100                                 tline = [tline t];
1101                             end
1102                         end
1103                         tline = sprintf('%s\n',tline);
1104                     end
1105                     descr = [descr tline(2:end)];
1106                 elseif isempty(tline)
1107                     if ~isempty(descr), break, end;
1108                 else
1109                     if flagsynopcont
1110                         if isempty(strmatch('...',fliplr(deblank(tline))))
1111                             flagsynopcont = 0;
1112                         end
1113                     else
1114                         break;
1115                     end
1116                 end
1117             end
1118             tpl = set(tpl,'var','DESCRIPTION',...
1119                 horztab(descr,options.tabs));
1120             
1121             %- Set cross-references template fields:
1122             %  Function called
1123             ind = find(hrefs(j,:) == 1);
1124             tpl = set(tpl,'var','crossrefcalls','');
1125             for k=1:length(ind)
1126                 if strcmp(mdirs{j},mdirs{ind(k)})
1127                     tpl = set(tpl,'var','L_NAME_CALL', ...
1128                         [names{ind(k)} options.extension]);
1129                 else
1130                     tpl = set(tpl,'var','L_NAME_CALL', ...
1131                               fullurl(backtomaster(mdirs{j}), ...
1132                                          mdirs{ind(k)}, ...
1133                                        [names{ind(k)} options.extension]));
1134                 end
1135                 tpl = set(tpl,'var','SYNOP_CALL',   synopsis{ind(k)});
1136                 tpl = set(tpl,'var','NAME_CALL',   names{ind(k)});
1137                 tpl = set(tpl,'var','H1LINE_CALL', h1line{ind(k)});
1138                 tpl = parse(tpl,'crossrefcalls','crossrefcall',1);
1139             end
1140             %  Callers
1141             ind = find(hrefs(:,j) == 1);
1142             tpl = set(tpl,'var','crossrefcalleds','');
1143             for k=1:length(ind)
1144                 if strcmp(mdirs{j},mdirs{ind(k)})
1145                     tpl = set(tpl,'var','L_NAME_CALLED', ...
1146                         [names{ind(k)} options.extension]);
1147                 else
1148                     tpl = set(tpl,'var','L_NAME_CALLED', ...
1149                         fullurl(backtomaster(mdirs{j}),...
1150                             mdirs{ind(k)}, ...
1151                             [names{ind(k)} options.extension]));
1152                 end
1153                 tpl = set(tpl,'var','SYNOP_CALLED',   synopsis{ind(k)});
1154                 tpl = set(tpl,'var','NAME_CALLED',   names{ind(k)});
1155                 tpl = set(tpl,'var','H1LINE_CALLED', h1line{ind(k)});
1156                 tpl = parse(tpl,'crossrefcalleds','crossrefcalled',1);
1157             end
1158             
1159             %- Set subfunction template field
1160             tpl = set(tpl,'var',{'subf' 'onesubf'},{'' ''});
1161             if ~isempty(subroutine{j}) & options.source
1162                 for k=1:length(subroutine{j})
1163                     tpl = set(tpl, 'var', 'L_SUB', ['#_sub' num2str(k)]);
1164                     tpl = set(tpl, 'var', 'SUB',   subroutine{j}{k});
1165                     tpl = parse(tpl, 'onesubf', 'onesubfunction',1);
1166                 end
1167                 tpl = parse(tpl,'subf','subfunction');
1168             end
1169             subname = extractname(subroutine{j});
1170             
1171             %- Link to M-file (for download)
1172             tpl = set(tpl,'var','downloads','');
1173             if options.download
1174                 tpl = parse(tpl,'downloads','download',1);
1175             end
1176             
1177             %- Display source code with cross-references
1178             if options.source & ~strcmpi(names{j},'contents')
1179                 fseek(fid2,0,-1);
1180                 it = 1;
1181                 matlabsource = '';
1182                 nbsubroutine = 1;
1183                 %- Get href function names of this file
1184                 indhrefnames = find(hrefs(j,:) == 1);
1185                 hrefnames = {names{indhrefnames}};
1186                 %- Loop over lines
1187                 while 1
1188                     tline = fgetl(fid2);
1189                     if ~ischar(tline), break, end
1190                     myline = '';
1191                     splitc = splitcode(entity(tline));
1192                     for k=1:length(splitc)
1193                         if isempty(splitc{k})
1194                         elseif ~isempty(strmatch('function', ...
1195                                         fliplr(deblank(fliplr(splitc{k})))))
1196                             if isspace(splitc{k}(1))
1197                                 nbs = find(diff(isspace(splitc{k}))==-1);
1198                                 myline = [myline splitc{k}(1:nbs(1))];
1199                                 splitc{k} = splitc{k}(nbs(1)+1:end);
1200                             end
1201                             %- Subfunctions definition
1202                             myline = [myline ...
1203                                 sprintf(tpl_mfile_aname,...
1204                                     ['_sub' num2str(nbsubroutine-1)],splitc{k})];
1205                             nbsubroutine = nbsubroutine + 1;
1206                         elseif splitc{k}(1) == ''''
1207                             myline = [myline ...
1208                                 sprintf(tpl_mfile_string,splitc{k})];
1209                         elseif splitc{k}(1) == '%'
1210                             myline = [myline ...
1211                                 sprintf(tpl_mfile_comment,deblank(splitc{k}))];
1212                         elseif ~isempty(strmatch('...',splitc{k}))
1213                             myline = [myline sprintf(tpl_mfile_keyword,'...')];
1214                             if ~isempty(splitc{k}(4:end))
1215                                 myline = [myline ...
1216                                     sprintf(tpl_mfile_comment,splitc{k}(4:end))];
1217                             end
1218                         else
1219                             %- Look for keywords
1220                             r = splitc{k};
1221                             while 1
1222                                 [t,r,q] = strtok(r,strtok_delim);
1223                                 myline = [myline q];
1224                                 if isempty(t), break, end;
1225                                 %- Highlight Matlab keywords &
1226                                 %  cross-references on known functions
1227                                 if options.syntaxHighlighting & ...
1228                                         any(strcmp(matlabKeywords,t))
1229                                     if strcmp('end',t)
1230                                         rr = fliplr(deblank(fliplr(r)));
1231                                         icomma = strmatch(',',rr);
1232                                         isemicolon = strmatch(';',rr);
1233                                         if ~(isempty(rr) | ~isempty([icomma isemicolon]))
1234                                             myline = [myline t];
1235                                         else
1236                                             myline = [myline sprintf(tpl_mfile_keyword,t)];
1237                                         end
1238                                     else
1239                                         myline = [myline sprintf(tpl_mfile_keyword,t)];
1240                                     end
1241                                 elseif any(strcmp(hrefnames,t))
1242                                     indt = indhrefnames(logical(strcmp(hrefnames,t)));
1243                                     flink = [t options.extension];
1244                                     ii = ismember({mdirs{indt}},mdirs{j});
1245                                     if ~any(ii)
1246                                         % take the first one...
1247                                         flink = fullurl(backtomaster(mdirs{j}),...
1248                                                           mdirs{indt(1)}, flink);
1249                                     else
1250                                         indt = indt(logical(ii));
1251                                     end
1252                                     myline = [myline sprintf(tpl_mfile_code,...
1253                                               flink, synopsis{indt(1)}, t)];
1254                                 elseif any(strcmp(subname,t))
1255                                     ii = find(strcmp(subname,t));
1256                                     myline = [myline sprintf(tpl_mfile_code,...
1257                                         ['#_sub' num2str(ii)],...
1258                                         ['sub' subroutine{j}{ii}],t)];
1259                                 else
1260                                     myline = [myline t];
1261                                 end
1262                             end
1263                         end
1264                     end
1265                     matlabsource = [matlabsource sprintf(tpl_mfile_line,it,myline)];
1266                     it = it + 1;
1267                 end
1268                 nblinetot = nblinetot + it - 1;
1269                 tpl = set(tpl,'var','SOURCECODE',...
1270                           horztab(matlabsource,options.tabs));
1271                 tpl = parse(tpl,'thesource','source');
1272             else
1273                 tpl = set(tpl,'var','thesource','');
1274             end
1275             tpl = parse(tpl,'OUT','TPL_MFILE');
1276             fprintf(fid,'%s',get(tpl,'OUT'));
1277             fclose(fid2);
1278             fclose(fid);
1279         end
1280     end
1281 end
1282 
1283 %-------------------------------------------------------------------------------
1284 %- Display Statistics
1285 %-------------------------------------------------------------------------------
1286 if options.verbose
1287     prnbline = '';
1288     if options.source
1289         prnbline = sprintf('(%d lines) ', nblinetot);
1290     end
1291     fprintf('Stats: %d M-files %sin %d directories documented in %d s.\n', ...
1292             length(mfiles), prnbline, length(mdir), round(etime(clock,t0)));
1293 end
1294 
1295 %===============================================================================
1296 function mfiles = getmfiles(mdirs, mfiles, recursive, ignoredDir)
1297     %- Extract M-files from a list of directories and/or M-files
1298 
1299     if nargin < 4, ignoredDir = {}; end
1300     for i=1:length(mdirs)
1301         currentdir = fullfile(pwd, mdirs{i});
1302         if exist(currentdir) == 2 % M-file
1303             mfiles{end+1} = mdirs{i};
1304         elseif exist(currentdir) == 7 % Directory
1305             d = dir(fullfile(currentdir, '*.m'));
1306             d = {d(~[d.isdir]).name};
1307             for j=1:length(d)
1308                 %- don't take care of files containing ','
1309                 %  probably a sccs file...
1310                 if isempty(findstr(',',d{j}))
1311                     mfiles{end+1} = fullfile(mdirs{i}, d{j});
1312                 end
1313             end
1314             if recursive
1315                 d = dir(currentdir);
1316                 d = {d([d.isdir]).name};
1317                 d = {d{~ismember(d,{'.' '..' ignoredDir{:}})}};
1318                 for j=1:length(d)
1319                     mfiles = getmfiles(cellstr(fullfile(mdirs{i},d{j})), ...
1320                                        mfiles, recursive, ignoredDir);
1321                 end
1322             end
1323         else
1324             fprintf('Warning: Unprocessed file %s.\n',mdirs{i});
1325             if ~isempty(strmatch('/',mdirs{i})) | findstr(':',mdirs{i})
1326                 fprintf('         Use relative paths in ''mfiles'' option\n');
1327             end 
1328         end
1329     end
1330 
1331 %===============================================================================
1332 function calldot(dotexec, mdotfile, mapfile, pngfile, opt)
1333     %- Draw a dependency graph in a PNG image using <dot> from GraphViz
1334 
1335     if nargin == 4, opt = ''; end
1336     try
1337         %- See <http://www.graphviz.org/>
1338         %  <dot> must be in your system path, see M2HTML FAQ:
1339         %  <http://www.artefact.tk/software/matlab/m2html/faq.php>
1340 
1341         eval(['!"' dotexec '" ' opt ' -Tcmap -Tpng "' mdotfile ...
1342               '" -o "' mapfile ... 
1343               '" -o "' pngfile '"']);
1344         % use '!' rather than 'system' for backward compability with Matlab 5.3
1345     catch % use of '!' prevents errors to be catched...
1346         fprintf('<dot> failed.');
1347     end
1348     
1349 %===============================================================================
1350 function s = backtomaster(mdir)
1351     %- Provide filesystem path to go back to the root folder
1352 
1353     ldir = splitpath(mdir);
1354     s = repmat('../',1,length(ldir));
1355     
1356 %===============================================================================
1357 function ldir = splitpath(p)
1358     %- Split a filesystem path into parts using filesep as separator
1359 
1360     ldir = {};
1361     p = deblank(p);
1362     while 1
1363         [t,p] = strtok(p,filesep);
1364         if isempty(t), break; end
1365         if ~strcmp(t,'.')
1366             ldir{end+1} = t;
1367         end
1368     end
1369     if isempty(ldir)
1370         ldir{1} = '.'; % should be removed
1371     end
1372 
1373 %===============================================================================
1374 function name = extractname(synopsis)
1375     %- Extract function name in a synopsis
1376 
1377     if ischar(synopsis), synopsis = {synopsis}; end
1378     name = cell(size(synopsis));
1379     for i=1:length(synopsis)
1380         ind = findstr(synopsis{i},'=');
1381         if isempty(ind)
1382             ind = findstr(synopsis{i},'function');
1383             s = synopsis{i}(ind(1)+8:end);
1384         else
1385             s = synopsis{i}(ind(1)+1:end);
1386         end
1387         name{i} = strtok(s,[9:13 32 40]); % white space characters and '('
1388     end
1389     if length(name) == 1, name = name{1}; end
1390 
1391 %===============================================================================
1392 function f = fullurl(varargin)
1393     %- Build full url from parts (using '/' and not filesep)
1394     
1395     f = strrep(fullfile(varargin{:}),'\','/');
1396 
1397 %===============================================================================
1398 function str = escapeblank(str)
1399     %- Escape white spaces using '\'
1400     
1401     str = deblank(fliplr(deblank(fliplr(str))));
1402     str = strrep(str,' ','\ ');
1403 
1404 %===============================================================================
1405 function str = entity(str)
1406     %- Escape HTML special characters
1407     %- See http://www.w3.org/TR/html4/charset.html#h-5.3.2
1408     
1409     str = strrep(str,'&','&amp;');
1410     str = strrep(str,'<','&lt;');
1411     str = strrep(str,'>','&gt;');
1412     str = strrep(str,'"','&quot;');
1413     
1414 %===============================================================================
1415 function str = horztab(str,n)
1416     %- For browsers, the horizontal tab character is the smallest non-zero
1417     %- number of spaces necessary to line characters up along tab stops that are
1418     %- every 8 characters: behaviour obtained when n = 0.
1419     
1420     if n > 0
1421         str = strrep(str,sprintf('\t'),blanks(n));
1422     end
```

---

Generated on Tue 29-Oct-2019 21:04:54 by **m2html** © 2005
