# Supplementary material for: Personalized whole‐body models integrate metabolism, physiology, and the gut microbiome
Source: Mol Syst Biol. 2020 May 28;16(5):e8982. doi: 10.15252/msb.20198982 (PMC7285886; doi:10.15252/msb.20198982)
Supplement: Supplementary file 22 — Dataset EV1 [file MSB-16-e8982-s022.zip › PSCM_toolbox/ext/m2html/doc/m2html/mdot.html]

Description of mdot


Home > m2html > mdot.m

# mdot

## PURPOSE

**MDOT - Export a dependency graph into DOT language**

## SYNOPSIS

**function mdot(mmat, dotfile,f)**

## DESCRIPTION

```
MDOT - Export a dependency graph into DOT language
  MDOT(MMAT, DOTFILE) loads a .mat file generated by M2HTML using option
  ('save','on') and writes an ascii file using the DOT language that can
  be drawn using <dot> or <neato> .
  MDOT(MMAT, DOTFILE,F) builds the graph containing M-file F and its
  neighbors only.
  See the following page for more details:
  <http://www.graphviz.org/>

  Example:
    mdot('m2html.mat','m2html.dot');
    !dot -Tps m2html.dot -o m2html.ps
    !neato -Tps m2html.dot -o m2html.ps

  See also M2HTML
```

## CROSS-REFERENCE INFORMATION

This function calls:


This function is called by:

- m2html M2HTML - Documentation Generator for Matlab M-files and Toolboxes in HTML

## SUBFUNCTIONS

- function f = fullurl(varargin)

## SOURCE CODE

```
0001 function mdot(mmat, dotfile,f)
0002 %MDOT - Export a dependency graph into DOT language
0003 %  MDOT(MMAT, DOTFILE) loads a .mat file generated by M2HTML using option
0004 %  ('save','on') and writes an ascii file using the DOT language that can
0005 %  be drawn using <dot> or <neato> .
0006 %  MDOT(MMAT, DOTFILE,F) builds the graph containing M-file F and its
0007 %  neighbors only.
0008 %  See the following page for more details:
0009 %  <http://www.graphviz.org/>
0010 %
0011 %  Example:
0012 %    mdot('m2html.mat','m2html.dot');
0013 %    !dot -Tps m2html.dot -o m2html.ps
0014 %    !neato -Tps m2html.dot -o m2html.ps
0015 %
0016 %  See also M2HTML
0017 
0018 %  Copyright (C) 2004 Guillaume Flandin <Guillaume@artefact.tk>
0019 %  $Revision: 1.1 $Date: 2004/05/05 17:14:09 $
0020 
0021 error(nargchk(2,3,nargin));
0022 
0023 if ischar(mmat)
0024     load(mmat);
0025 elseif iscell(mmat)
0026     hrefs  = mmat{1};
0027     names  = mmat{2};
0028     options = mmat{3};
0029     if nargin == 3, mfiles = mmat{4}; end
0030     mdirs = cell(size(names));
0031     [mdirs{:}] = deal('');
0032     if nargin == 2 & length(mmat) > 3, 
0033         mdirs = mmat{4};
0034     end;
0035 else
0036     error('[mdot] Invalid argument: mmat.');
0037 end
0038 
0039 fid = fopen(dotfile,'wt');
0040 if fid == -1, error(sprintf('[mdot] Cannot open %s.',dotfile)); end
0041 
0042 fprintf(fid,'/* Created by mdot for Matlab */\n');
0043 fprintf(fid,'digraph m2html {\n');
0044 
0045 % if 'names' contains '.' then they should be surrounded by '"'
0046 
0047 if nargin == 2
0048     for i=1:size(hrefs,1)
0049         n = find(hrefs(i,:) == 1);
0050         m{i} = n;
0051         for j=1:length(n)
0052             fprintf(fid,['  ' names{i} ' -> ' names{n(j)} ';\n']);
0053         end
0054     end
0055     %m = unique([m{:}]);
0056     fprintf(fid,'\n');
0057     for i=1:size(hrefs,1)
0058         fprintf(fid,['  ' names{i} ' [URL="' ...
0059             fullurl(mdirs{i},[names{i} options.extension]) '"];\n']);
0060     end
0061 else
0062     i = find(strcmp(f,mfiles));
0063     if length(i) ~= 1
0064         error(sprintf('[mdot] Cannot find %s.',f));
0065     end
0066     n = find(hrefs(i,:) == 1);
0067     for j=1:length(n)
0068         fprintf(fid,['  ' names{i} ' -> ' names{n(j)} ';\n']);
0069     end
0070     m = find(hrefs(:,i) == 1);
0071     for j=1:length(m)
0072         if n(j) ~= i
0073             fprintf(fid,['  ' names{m(j)} ' -> ' names{i} ';\n']);
0074         end
0075     end
0076     n = unique([n(:)' m(:)']);
0077     fprintf(fid,'\n');
0078     for i=1:length(n)
0079         fprintf(fid,['  ' names{n(i)} ' [URL="' fullurl(mdirs{i}, ...
0080             [names{n(i)} options.extension]) '"];\n']);
0081     end
0082 end
0083 
0084 fprintf(fid,'}');
0085 
0086 fid = fclose(fid);
0087 if fid == -1, error(sprintf('[mdot] Cannot close %s.',dotfile)); end
0088 
0089 %===========================================================================
0090 function f = fullurl(varargin)
0091     %- Build full url from parts (using '/' and not filesep)
0092     
0093     f = strrep(fullfile(varargin{:}),'\','/');
```

---

Generated on Tue 29-Oct-2019 21:04:54 by **m2html** © 2005
