# Supplementary material for: Personalized whole‐body models integrate metabolism, physiology, and the gut microbiome
Source: Mol Syst Biol. 2020 May 28;16(5):e8982. doi: 10.15252/msb.20198982 (PMC7285886; doi:10.15252/msb.20198982)
Supplement: Supplementary file 22 — Dataset EV1 [file MSB-16-e8982-s022.zip › PSCM_toolbox/PSCM_toolbox_doc/src/scripts/BMRcalculations/perform_BMR_newData.html]

Description of perform\_BMR\_newData


# perform\_BMR\_newData

## PURPOSE

**This script repeats the simulation described in Thiele et al., "Personalized whole-body models integrate metabolism, physiology, and the gut microbiome", Method section 3.9.2 Validation of the parameters in an independent data set.**

## SYNOPSIS

**This is a script file.**

## DESCRIPTION

```
 This script repeats the simulation described in Thiele et al., "Personalized whole-body models integrate metabolism, physiology, and the gut microbiome", Method section 3.9.2 Validation of the parameters in an independent data set.
 The input data come from  L. L. Loureiro et al., Basal Metabolic Rate of Adolescent Modern Pentathlon Athletes: Agreement between Indirect Calorimetry and Predictive Equations and the Correlation with Body Parameters. PLoS One, 10(11):e0142859 (2015).
```

## CROSS-REFERENCE INFORMATION

This function calls:

- adjustWholeBodyRxnCoeff [model] = adjustWholeBodyRxnCoeff(model, listOrgan, listCoeff)
- calcOrganFract This function extrapolates the organ weight fractions based on polynomials given in
- calculateBMR This function calculates the basal metabolic rate using the
- setSimulationConstraints This function sets the remaining simulation constraints that I identified during the
- optimizeWBModel Solves flux balance analysis problems, and variants thereof
- EUAverageDietNew Average European Diet defintion. For details, please see https://www.vmh.life/#nutrition
- individualizedLabReport This function computes personalized physiolgical parameters based on the
- physiologicalConstraintsHMDBbased This function applies constraints to the whole-body metabolic model
- setDietConstraints This function sets diet constraints onto the bounds of the diet uptale
- standardPhysiolDefaultParameters This script creates the IndividualParameters structure which contains

This function is called by:

## SOURCE CODE

```
0001 % This script repeats the simulation described in Thiele et al., "Personalized whole-body models integrate metabolism, physiology, and the gut microbiome", Method section 3.9.2 Validation of the parameters in an independent data set.
0002 % The input data come from  L. L. Loureiro et al., Basal Metabolic Rate of Adolescent Modern Pentathlon Athletes: Agreement between Indirect Calorimetry and Predictive Equations and the Correlation with Body Parameters. PLoS One, 10(11):e0142859 (2015).
0003 
0004 global useSolveCobraLPCPLEX
0005 
0006 clear Energy_kcal_femaleBMR2 %avoid adding another column to this variable
0007 
0008 sex = female.sex;
0009 standardPhysiolDefaultParameters;
0010 IndividualParameters_female = IndividualParameters;
0011 
0012 female = physiologicalConstraintsHMDBbased(female,IndividualParameters_female);
0013 EUAverageDietNew;
0014 female = setDietConstraints(female, Diet);
0015 
0016 female = setSimulationConstraints(female);
0017 
0018 minInf = -1000000;
0019 maxInf = 1000000;
0020 minConstraints = length(intersect(find(female.lb>minInf),find(female.lb)));
0021 maxConstraints =length(intersect(find(female.ub<maxInf),find(female.ub)));
0022 
0023 female = changeObjective(female,'Whole_body_objective_rxn');
0024 female.osenseStr = 'max';
0025 
0026 % adjust Muscle and fat atp in biomass - compute and compare BMF's with
0027 % BMRs
0028 
0029 sensi_analysis = [1; 5; 10;50;100 ];
0030 
0031 cnt = 1;
0032 for y =2 %1 : length(sensi_analysis)
0033     femaleBMR = female;
0034     clear female
0035     
0036     if useSolveCobraLPCPLEX
0037         femaleBMR.S = femaleBMR.A;
0038     end
0039     % femaleBMR
0040     BM = (find(~cellfun(@isempty,strfind(femaleBMR.rxns,'Muscle_biomass_'))));
0041     
0042     % find all reactions in the model that involve atp
0043     atp = (find(~cellfun(@isempty,strfind(femaleBMR.mets,'_atp[c'))));
0044     adp = (find(~cellfun(@isempty,strfind(femaleBMR.mets,'_adp[c'))));
0045     pi = (find(~cellfun(@isempty,strfind(femaleBMR.mets,'_pi[c'))));
0046     h = (find(~cellfun(@isempty,strfind(femaleBMR.mets,'_h[c'))));
0047     h2o = (find(~cellfun(@isempty,strfind(femaleBMR.mets,'_h2o[c'))));
0048     BMatpSum = 0;
0049     for i = 1: length(BM)
0050         % grab atp
0051         BMmets = (find(femaleBMR.S(:,BM(i))));
0052         BMatp = intersect(BMmets,atp);
0053         femaleBMR.S(BMatp,BM(i))= femaleBMR.S(BMatp,BM(i)) *sensi_analysis(y);
0054         BMatp = intersect(BMmets,adp);
0055         femaleBMR.S(BMatp,BM(i))= femaleBMR.S(BMatp,BM(i)) *sensi_analysis(y);
0056         BMatp = intersect(BMmets,pi);
0057         femaleBMR.S(BMatp,BM(i))= femaleBMR.S(BMatp,BM(i)) *sensi_analysis(y);
0058         BMatp = intersect(BMmets,h);
0059         femaleBMR.S(BMatp,BM(i))= femaleBMR.S(BMatp,BM(i)) *sensi_analysis(y);
0060         BMatp= intersect(BMmets,h2o);
0061         femaleBMR.S(BMatp,BM(i))= femaleBMR.S(BMatp,BM(i)) *sensi_analysis(y);
0062     end
0063     
0064     Energy_kJ_femaleBMR2(1,cnt) = sensi_analysis(y);
0065     for z =1%1: length(sensi_analysis)
0066         femaleBMR2 = femaleBMR;
0067         BM = (find(~cellfun(@isempty,strfind(femaleBMR2.rxns,'Adipocytes_biomass_'))));
0068         for i = 1: length(BM)
0069             % grab atp
0070             BMmets = (find(femaleBMR2.S(:,BM(i))));
0071             BMatp = intersect(BMmets,atp);
0072             femaleBMR2.S(BMatp,BM(i))= femaleBMR2.S(BMatp,BM(i)) *sensi_analysis(z);
0073             BMatp = intersect(BMmets,adp);
0074             femaleBMR2.S(BMatp,BM(i))= femaleBMR2.S(BMatp,BM(i)) *sensi_analysis(z);
0075             BMatp = intersect(BMmets,pi);
0076             femaleBMR2.S(BMatp,BM(i))= femaleBMR2.S(BMatp,BM(i))*sensi_analysis(z);
0077             BMatp = intersect(BMmets,h);
0078             femaleBMR2.S(BMatp,BM(i))= femaleBMR2.S(BMatp,BM(i)) *sensi_analysis(z);
0079             BMatp= intersect(BMmets,h2o);
0080             femaleBMR2.S(BMatp,BM(i))= femaleBMR2.S(BMatp,BM(i)) *sensi_analysis(z);
0081         end
0082         
0083         Energy_kJ_femaleBMR2(2,cnt) = sensi_analysis(z);
0084         
0085         if useSolveCobraLPCPLEX
0086             femaleBMR2.A = femaleBMR2.S;
0087         end
0088         InputDataAll =  {
0089             'sex' ''    'male'        'female'        'female'        'female'        'female'        'female'        'male'        'female'        'male'        'male'        'male'        'female'        'male'        'male'        'male'        'male'        'male'        'female'        'female'        'female'        'male'        'male'        'male'        'female'        'male'        'male'        'male'        'male'
0090             'age' '' num2str(15) num2str(14) num2str(12) num2str(18) num2str(16) num2str(15) num2str(17) num2str(11) num2str(13) num2str(14) num2str(16) num2str(16) num2str(12) num2str(15) num2str(16) num2str(18) num2str(13) num2str(11) num2str(12) num2str(17) num2str(12) num2str(15) num2str(17) num2str(12) num2str(15) num2str(15) num2str(14) num2str(15)
0091             'Height' '' num2str(168) num2str(164) num2str(152) num2str(162) num2str(166) num2str(173) num2str(172) num2str(161) num2str(164) num2str(165) num2str(170) num2str(156) num2str(172) num2str(175) num2str(177) num2str(173) num2str(161) num2str(155) num2str(157) num2str(165) num2str(150) num2str(174) num2str(175) num2str(139) num2str(184) num2str(162) num2str(159.5) num2str(177)
0092             'Weight' '' num2str(60.8) num2str(56.8) num2str(40.2) num2str(53.2) num2str(54.1) num2str(62.9) num2str(64.4) num2str(56.2) num2str(47.9) num2str(52.8) num2str(65.6) num2str(49.9) num2str(58) num2str(69.1) num2str(64.8) num2str(63.5) num2str(48) num2str(50.4) num2str(44.3) num2str(58) num2str(40.1) num2str(61.2) num2str(64.6) num2str(35.4) num2str(65.8) num2str(47.4) num2str(48.2) num2str(59.6)
0093             'Fat-free' '' num2str(51.66) num2str(40.08) num2str(30.08) num2str(43.71) num2str(42.88) num2str(53.44) num2str(58.18) num2str(36.4) num2str(41.4) num2str(47) num2str(55.91) num2str(39.52) num2str(48.23) num2str(59.75) num2str(56) num2str(53.86) num2str(42) num2str(33.75) num2str(37.31) num2str(45.31) num2str(33.62) num2str(53.22) num2str(56.62) num2str(20) num2str(49) num2str(38.56) num2str(39) num2str(50)
0094             'BMI' '' num2str(21.54) num2str(21.12) num2str(17.4) num2str(20.27) num2str(19.63) num2str(21.02) num2str(21.77) num2str(21.68) num2str(17.81) num2str(19.39) num2str(22.7) num2str(20.5) num2str(19.61) num2str(22.56) num2str(20.68) num2str(21.22) num2str(18.52) num2str(20.98) num2str(17.97) num2str(21.3) num2str(17.82) num2str(20.21) num2str(21.09) num2str(18.32) num2str(19.44) num2str(18.06) num2str(18.95) num2str(19.02)
0095             'BMR' '' num2str(1661.16) num2str(1414.97) num2str(1282.42) num2str(1489.45) num2str(1277.24) num2str(1582.33) num2str(1515.13) num2str(1495.3) num2str(1775.51) num2str(1576.21) num2str(1476.97) num2str(1271.78) num2str(1551.96) num2str(2039.63) num2str(1808.9) num2str(1537.86) num2str(1457.89) num2str(1243.59) num2str(1443.9) num2str(1318.07) num2str(1249.74) num2str(1537.93) num2str(1633.33) num2str(1103.2) num2str(1508.41) num2str(1393.85) num2str(1167.48) num2str(1612.75)
0096             };
0097         
0098         femaleBMR2O=femaleBMR2;
0099         for i = 3 :  size(InputDataAll,2) %
0100             InputData =  [InputDataAll(:,1), InputDataAll(:,2), InputDataAll(:,i)];
0101             femaleBMR2 = femaleBMR2O;
0102             
0103             sex =  InputDataAll(1,i);
0104             femaleBMR2.sex = sex;
0105             if strcmp(sex,'female')
0106                 optionCardiacOutput = 0; %0 4 2
0107                 [femaleBMR2,IndividualParametersPersonalized] = individualizedLabReport(femaleBMR2,IndividualParameters_female, InputData,optionCardiacOutput);
0108                 % 3. set personalized constraints
0109                 femaleBMR2.IndividualParametersPersonalized=IndividualParametersPersonalized;
0110                 % 3. set personalized constraints
0111                 
0112                 % calculate new organ weight fraction for a given personalized weight
0113                 [listOrgan,OrganWeight,OrganWeightFract,IndividualParametersPersonalized] = calcOrganFract(femaleBMR2,IndividualParametersPersonalized);
0114                 
0115                 % I repeat this step, as I calculate in some options the CO based on
0116                 % bloodVol which is only calculated in calcOrganFract
0117                 [femaleBMR2,IndividualParametersPersonalized] = individualizedLabReport(femaleBMR2,IndividualParametersPersonalized, InputData,optionCardiacOutput);
0118                 femaleBMR2.IndividualParametersPersonalized=IndividualParametersPersonalized;
0119                 
0120                 if 1
0121                     % adjust whole body maintenance reaction based on new organ weight
0122                     % fractions
0123                     % adjust adipocyte and muscle weight to measured one
0124                     Fat_fraction = 1 - str2num(InputData{5,3})/str2num(InputData{4,3});
0125                     FatDiff = Fat_fraction-OrganWeightFract(11)
0126                     OrganWeightFract(11) = OrganWeightFract(11)+FatDiff;
0127                     OrganWeightFract(12) = OrganWeightFract(12)-FatDiff;
0128                     
0129                     Fat_weight =  (str2num(InputData{4,3})-str2num(InputData{5,3}))*1000;
0130                     Fat_weightOri=str2num(IndividualParametersPersonalized.OrgansWeights{11,2});
0131                     Muscle_weightOri=str2num(IndividualParametersPersonalized.OrgansWeights{12,2});
0132                     % substract higher fat_weight from Muscle_weight
0133                     Muscle_weight = Muscle_weightOri-(Fat_weight - Fat_weightOri);
0134                     
0135                     IndividualParametersPersonalized.OrgansWeights{11,2} = num2str(Fat_weight);
0136                     IndividualParametersPersonalized.OrgansWeights{11,3} = num2str(OrganWeightFract(11)*100);
0137                     IndividualParametersPersonalized.OrgansWeights{12,2} = num2str(Muscle_weight);
0138                     IndividualParametersPersonalized.OrgansWeights{12,3} = num2str(OrganWeightFract(12)*100);
0139                 end
0140                 [femaleBMR2] = adjustWholeBodyRxnCoeff(femaleBMR2, listOrgan, OrganWeightFract);
0141                 femaleBMR2.listOrgan = listOrgan;
0142                 femaleBMR2.OrganWeightFract = OrganWeightFract;
0143                 femaleBMR2 = setDietConstraints(femaleBMR2);
0144                 % set some more constraints
0145                 %femaleBMR2 = setSimulationConstraints(femaleBMR2);
0146                 femaleBMR2 = physiologicalConstraintsHMDBbased(femaleBMR2,IndividualParametersPersonalized);
0147                 
0148                 %femaleBMR2.ub(strmatch('Brain_EX_glc_D(',femaleBMR2.rxns)) = -100; % currently -400 rendering many of the models to be infeasible in germfree state
0149                 
0150                 CO(i,cnt) = IndividualParametersPersonalized.CardiacOutput;
0151                 BM = (find(~cellfun(@isempty,strfind(femaleBMR2.rxns,'Heart_DM_atp'))));
0152                 femaleBMR2.lb(BM) = 1000;
0153                 
0154                 if useSolveCobraLPCPLEX
0155                     femaleBMR2 = changeObjective(femaleBMR2,'Whole_body_objective_rxn');
0156                     femaleBMR2.osense = -1;
0157                     [solution] = computeMin2Norm_HH(femaleBMR2,'tomlab_cplex');
0158                 else
0159                     femaleBMR2.c(:)=0;
0160                     param.minNorm = 1e-6;
0161                     femaleBMR2.osenseStr='min';
0162                     solution = optimizeWBModel(femaleBMR2,param);
0163                 end
0164                 if solution.stat == 1 || solution.stat == 3
0165                     F = max(-femaleBMR2.S,0);
0166                     R = max(femaleBMR2.S,0);
0167                     vf = max(solution.v,0);
0168                     vr = max(-solution.v,0);
0169                     production=[R F]*[vf; vr];
0170                     consumption=[F R]*[vf; vr];
0171                     % find all reactions in the model that involve atp
0172                     atp = (find(~cellfun(@isempty,strfind(femaleBMR2.mets,'_atp['))));
0173                     % sum of atp consumption in the flux distribution
0174                     Sum_atp_femaleBMR2=sum(consumption(atp,1)); % (in mmol ATP/day/person)
0175                     % compute the energy release in kJ
0176                     Energy_kJ_femaleBMR2(i,cnt) = Sum_atp_femaleBMR2/1000 * 64; % (in kJ/day/person)
0177                 else
0178                     Energy_kJ_femaleBMR2(i,cnt) = NaN;
0179                 end
0180                 
0181                 % compute the energy release in kcal, where 1 kJ = 0.239006 kcal
0182                 Energy_kcal_femaleBMR2(i,cnt) =  Energy_kJ_femaleBMR2(i,cnt) *0.239006; % (in kcal/day/person)
0183                 Energy_kcal_femaleBMR2_origStatText{i,cnt} = solution.origStatText;
0184             end
0185         end
0186         cnt = cnt +1;
0187         %save Results_sensi2_2018_12_14
0188         %save([resultsPath 'Results_BMR_newData'])
0189     end
0190 end
0191 for i = 3 : size(InputDataAll,2)
0192     BMR_female_20(:,i) = calculateBMR('female', str2num(InputDataAll{4,i}), str2num(InputDataAll{3,i}), str2num(InputDataAll{2,i}));
0193 end
0194 
0195 clearvars -except computeResults useSolveCobraLPCPLEX InputDataAll Energy_kcal_femaleBMR2 BMR_female_20 male Energy_kcal_femaleBMR2_origStatText
0196 
0197 %%
0198 % test multiple BMR's
0199 if 1
0200     
0201     sex = male.sex;
0202     standardPhysiolDefaultParameters;
0203     IndividualParameters_male = IndividualParameters;
0204     
0205     male = physiologicalConstraintsHMDBbased(male,IndividualParameters_male);
0206     EUAverageDietNew;
0207     male = setDietConstraints(male, Diet);
0208     
0209     male = setSimulationConstraints(male);
0210     
0211     minInf = -1000000;
0212     maxInf = 1000000;
0213     minConstraints = length(intersect(find(male.lb>minInf),find(male.lb)));
0214     maxConstraints =length(intersect(find(male.ub<maxInf),find(male.ub)));
0215     
0216     male = changeObjective(male,'Whole_body_objective_rxn');
0217     male.osense = -1;
0218     
0219     % adjust Muscle and fat atp in biomass - compute and compare BMF's with
0220     % BMRs
0221     
0222     sensi_analysis = [1; 5; 10;50;100 ];
0223     
0224     cnt = 1;
0225     for y =2 %1 : length(sensi_analysis)
0226         maleBMR = male;
0227         
0228         if useSolveCobraLPCPLEX
0229             maleBMR.S = maleBMR.A;
0230         end 
0231         
0232         % maleBMR
0233         BM = (find(~cellfun(@isempty,strfind(maleBMR.rxns,'Muscle_biomass_'))));
0234         
0235         % find all reactions in the model that involve atp
0236         atp = (find(~cellfun(@isempty,strfind(maleBMR.mets,'_atp[c'))));
0237         adp = (find(~cellfun(@isempty,strfind(maleBMR.mets,'_adp[c'))));
0238         pi = (find(~cellfun(@isempty,strfind(maleBMR.mets,'_pi[c'))));
0239         h = (find(~cellfun(@isempty,strfind(maleBMR.mets,'_h[c'))));
0240         h2o = (find(~cellfun(@isempty,strfind(maleBMR.mets,'_h2o[c'))));
0241         BMatpSum = 0;
0242         for i = 1: length(BM)
0243             % grab atp
0244             BMmets = (find(maleBMR.S(:,BM(i))));
0245             BMatp = intersect(BMmets,atp);
0246             maleBMR.S(BMatp,BM(i))= maleBMR.S(BMatp,BM(i)) *sensi_analysis(y);
0247             BMatp = intersect(BMmets,adp);
0248             maleBMR.S(BMatp,BM(i))= maleBMR.S(BMatp,BM(i)) *sensi_analysis(y);
0249             BMatp = intersect(BMmets,pi);
0250             maleBMR.S(BMatp,BM(i))= maleBMR.S(BMatp,BM(i)) *sensi_analysis(y);
0251             BMatp = intersect(BMmets,h);
0252             maleBMR.S(BMatp,BM(i))= maleBMR.S(BMatp,BM(i)) *sensi_analysis(y);
0253             BMatp= intersect(BMmets,h2o);
0254             maleBMR.S(BMatp,BM(i))= maleBMR.S(BMatp,BM(i)) *sensi_analysis(y);
0255         end
0256         
0257         Energy_kJ_maleBMR2(1,cnt) = sensi_analysis(y);
0258         for z =1%1: length(sensi_analysis)
0259             maleBMR2 = maleBMR;
0260             BM = (find(~cellfun(@isempty,strfind(maleBMR2.rxns,'Adipocytes_biomass_'))));
0261             for i = 1: length(BM)
0262                 % grab atp
0263                 BMmets = (find(maleBMR2.S(:,BM(i))));
0264                 BMatp = intersect(BMmets,atp);
0265                 maleBMR2.S(BMatp,BM(i))= maleBMR2.S(BMatp,BM(i)) *sensi_analysis(z);
0266                 BMatp = intersect(BMmets,adp);
0267                 maleBMR2.S(BMatp,BM(i))= maleBMR2.S(BMatp,BM(i)) *sensi_analysis(z);
0268                 BMatp = intersect(BMmets,pi);
0269                 maleBMR2.S(BMatp,BM(i))= maleBMR2.S(BMatp,BM(i))*sensi_analysis(z);
0270                 BMatp = intersect(BMmets,h);
0271                 maleBMR2.S(BMatp,BM(i))= maleBMR2.S(BMatp,BM(i)) *sensi_analysis(z);
0272                 BMatp= intersect(BMmets,h2o);
0273                 maleBMR2.S(BMatp,BM(i))= maleBMR2.S(BMatp,BM(i)) *sensi_analysis(z);
0274             end
0275             
0276             Energy_kJ_maleBMR2(2,cnt) = sensi_analysis(z);
0277             
0278             if useSolveCobraLPCPLEX
0279                 maleBMR2.A = maleBMR2.S;
0280             end
0281             
0282             InputDataAll =  {
0283                 'sex' ''    'male'        'female'        'female'        'female'        'female'        'female'        'male'        'female'        'male'        'male'        'male'        'female'        'male'        'male'        'male'        'male'        'male'        'female'        'female'        'female'        'male'        'male'        'male'        'female'        'male'        'male'        'male'        'male'
0284                 'age' '' num2str(15) num2str(14) num2str(12) num2str(18) num2str(16) num2str(15) num2str(17) num2str(11) num2str(13) num2str(14) num2str(16) num2str(16) num2str(12) num2str(15) num2str(16) num2str(18) num2str(13) num2str(11) num2str(12) num2str(17) num2str(12) num2str(15) num2str(17) num2str(12) num2str(15) num2str(15) num2str(14) num2str(15)
0285                 'Height' '' num2str(168) num2str(164) num2str(152) num2str(162) num2str(166) num2str(173) num2str(172) num2str(161) num2str(164) num2str(165) num2str(170) num2str(156) num2str(172) num2str(175) num2str(177) num2str(173) num2str(161) num2str(155) num2str(157) num2str(165) num2str(150) num2str(174) num2str(175) num2str(139) num2str(184) num2str(162) num2str(159.5) num2str(177)
0286                 'Weight' '' num2str(60.8) num2str(56.8) num2str(40.2) num2str(53.2) num2str(54.1) num2str(62.9) num2str(64.4) num2str(56.2) num2str(47.9) num2str(52.8) num2str(65.6) num2str(49.9) num2str(58) num2str(69.1) num2str(64.8) num2str(63.5) num2str(48) num2str(50.4) num2str(44.3) num2str(58) num2str(40.1) num2str(61.2) num2str(64.6) num2str(35.4) num2str(65.8) num2str(47.4) num2str(48.2) num2str(59.6)
0287                 'Fat-free' '' num2str(51.66) num2str(40.08) num2str(30.08) num2str(43.71) num2str(42.88) num2str(53.44) num2str(58.18) num2str(36.4) num2str(41.4) num2str(47) num2str(55.91) num2str(39.52) num2str(48.23) num2str(59.75) num2str(56) num2str(53.86) num2str(42) num2str(33.75) num2str(37.31) num2str(45.31) num2str(33.62) num2str(53.22) num2str(56.62) num2str(20) num2str(49) num2str(38.56) num2str(39) num2str(50)
0288                 'BMI' '' num2str(21.54) num2str(21.12) num2str(17.4) num2str(20.27) num2str(19.63) num2str(21.02) num2str(21.77) num2str(21.68) num2str(17.81) num2str(19.39) num2str(22.7) num2str(20.5) num2str(19.61) num2str(22.56) num2str(20.68) num2str(21.22) num2str(18.52) num2str(20.98) num2str(17.97) num2str(21.3) num2str(17.82) num2str(20.21) num2str(21.09) num2str(18.32) num2str(19.44) num2str(18.06) num2str(18.95) num2str(19.02)
0289                 'BMR' '' num2str(1661.16) num2str(1414.97) num2str(1282.42) num2str(1489.45) num2str(1277.24) num2str(1582.33) num2str(1515.13) num2str(1495.3) num2str(1775.51) num2str(1576.21) num2str(1476.97) num2str(1271.78) num2str(1551.96) num2str(2039.63) num2str(1808.9) num2str(1537.86) num2str(1457.89) num2str(1243.59) num2str(1443.9) num2str(1318.07) num2str(1249.74) num2str(1537.93) num2str(1633.33) num2str(1103.2) num2str(1508.41) num2str(1393.85) num2str(1167.48) num2str(1612.75)
0290                 };
0291             
0292             maleBMR2O=maleBMR2;
0293             for i = 3 :  size(InputDataAll,2) %
0294                 InputData =  [InputDataAll(:,1), InputDataAll(:,2), InputDataAll(:,i)];
0295                 maleBMR2 = maleBMR2O;
0296                 
0297                 sex =  InputDataAll(1,i);
0298                 maleBMR2.sex = sex;
0299                 if strcmp(sex,'male')
0300                     optionCardiacOutput = 0; %0 4 2
0301                     [maleBMR2,IndividualParametersPersonalized] = individualizedLabReport(maleBMR2,IndividualParameters_male, InputData,optionCardiacOutput);
0302                     % 3. set personalized constraints
0303                     maleBMR2.IndividualParametersPersonalized=IndividualParametersPersonalized;
0304                     % 3. set personalized constraints
0305                     
0306                     % calculate new organ weight fraction for a given personalized weight
0307                     [listOrgan,OrganWeight,OrganWeightFract,IndividualParametersPersonalized] = calcOrganFract(maleBMR2,IndividualParametersPersonalized);
0308                     
0309                     % I repeat this step, as I calculate in some options the CO based on
0310                     % bloodVol which is only calculated in calcOrganFract
0311                     [maleBMR2,IndividualParametersPersonalized] = individualizedLabReport(maleBMR2,IndividualParametersPersonalized, InputData,optionCardiacOutput);
0312                     maleBMR2.IndividualParametersPersonalized=IndividualParametersPersonalized;
0313                     
0314                     if 1
0315                         % adjust whole body maintenance reaction based on new organ weight
0316                         % fractions
0317                         % adjust adipocyte and muscle weight to measured one
0318                         Fat_fraction = 1 - str2num(InputData{5,3})/str2num(InputData{4,3});
0319                         FatDiff = Fat_fraction-OrganWeightFract(11);
0320                         OrganWeightFract(11) = OrganWeightFract(11)+FatDiff;
0321                         OrganWeightFract(12) = OrganWeightFract(12)-FatDiff;
0322                         
0323                         Fat_weight =  (str2num(InputData{4,3})-str2num(InputData{5,3}))*1000;
0324                         Fat_weightOri=str2num(IndividualParametersPersonalized.OrgansWeights{11,2});
0325                         Muscle_weightOri=str2num(IndividualParametersPersonalized.OrgansWeights{12,2});
0326                         % substract higher fat_weight from Muscle_weight
0327                         Muscle_weight = Muscle_weightOri-(Fat_weight - Fat_weightOri);
0328                         
0329                         IndividualParametersPersonalized.OrgansWeights{11,2} = num2str(Fat_weight);
0330                         IndividualParametersPersonalized.OrgansWeights{11,3} = num2str(OrganWeightFract(11)*100);
0331                         IndividualParametersPersonalized.OrgansWeights{12,2} = num2str(Muscle_weight);
0332                         IndividualParametersPersonalized.OrgansWeights{12,3} = num2str(OrganWeightFract(12)*100);
0333                     end
0334                     [maleBMR2] = adjustWholeBodyRxnCoeff(maleBMR2, listOrgan, OrganWeightFract);
0335                     maleBMR2.listOrgan = listOrgan;
0336                     maleBMR2.OrganWeightFract = OrganWeightFract;
0337                     maleBMR2 = setDietConstraints(maleBMR2);
0338                     % set some more constraints
0339                     %maleBMR2 = setSimulationConstraints(maleBMR2);
0340                     maleBMR2 = physiologicalConstraintsHMDBbased(maleBMR2,IndividualParametersPersonalized);
0341                     
0342                     %maleBMR2.ub(strmatch('Brain_EX_glc_D(',maleBMR2.rxns)) = -100; % currently -400 rendering many of the models to be infeasible in germfree state
0343                     
0344                     CO(i,cnt) = IndividualParametersPersonalized.CardiacOutput;
0345                     BM = (find(~cellfun(@isempty,strfind(maleBMR2.rxns,'Heart_DM_atp'))));
0346                     maleBMR2.lb(BM) = 6000;
0347                     
0348                     if useSolveCobraLPCPLEX
0349                         maleBMR2 = changeObjective(maleBMR2,'Whole_body_objective_rxn');
0350                         maleBMR2.osenseStr = 'max';
0351                         maleBMR2.osense = -1;
0352                         [solution] = computeMin2Norm_HH(maleBMR2);
0353                     else
0354                         maleBMR2.c(:)=0;
0355                         param.minNorm=1e-6;
0356                         maleBMR2.osenseStr = 'min';
0357                         solution = optimizeWBModel(maleBMR2,param);
0358                     end
0359                     if solution.stat == 1 || solution.stat == 3
0360                         F = max(-maleBMR2.S,0);
0361                         R = max(maleBMR2.S,0);
0362                         vf = max(solution.v,0);
0363                         vr = max(-solution.v,0);
0364                         production=[R F]*[vf; vr];
0365                         consumption=[F R]*[vf; vr];
0366                         % find all reactions in the model that involve atp
0367                         atp = (find(~cellfun(@isempty,strfind(maleBMR2.mets,'_atp['))));
0368                         % sum of atp consumption in the flux distribution
0369                         Sum_atp_maleBMR2=sum(consumption(atp,1)); % (in mmol ATP/day/person)
0370                         % compute the energy release in kJ
0371                         Energy_kJ_maleBMR2(i,cnt) = Sum_atp_maleBMR2/1000 * 64; % (in kJ/day/person)
0372                         % compute the energy release in kcal, where 1 kJ = 0.239006 kcal
0373                         Energy_kcal_maleBMR2(i,cnt) =  Energy_kJ_maleBMR2(i,cnt) *0.239006; % (in kcal/day/person)
0374                     else
0375                         Energy_kJ_maleBMR2(i,cnt) = NaN;
0376                     end
0377                     Energy_kcal_maleBMR2_origStatText{i,cnt} = solution.origStatText;
0378                 end
0379             end
0380             cnt = cnt +1;
0381             %save Results_sensi2_2018_12_14
0382             %save([resultsPath 'Results_BMR_newData'])
0383         end
0384     end
0385 end
0386 for i = 3 : size(InputDataAll,2)
0387     BMR_male_20(:,i) = calculateBMR('male', str2num(InputDataAll{4,i}), str2num(InputDataAll{3,i}), str2num(InputDataAll{2,i}));
0388 end
0389 clearvars -except computeResults useSolveCobraLPCPLEX InputDataAll male BMR_male_20 Energy_kcal_maleBMR2 Energy_kcal_maleBMR2_origStatText female BMR_female_20 Energy_kcal_femaleBMR2 Energy_kcal_femaleBMR2_origStatText
```

---

Generated on Thu 14-May-2020 13:05:49 by **m2html** © 2005
