# Supplementary material for: Personalized whole‐body models integrate metabolism, physiology, and the gut microbiome
Source: Mol Syst Biol. 2020 May 28;16(5):e8982. doi: 10.15252/msb.20198982 (PMC7285886; doi:10.15252/msb.20198982)
Supplement: Supplementary file 22 — Dataset EV1 [file MSB-16-e8982-s022.zip › PSCM_toolbox/PSCM_toolbox_doc/src/scripts/BMRcalculations/perform_sensi_BMR_all.html]

Description of perform\_sensi\_BMR\_all


# perform\_sensi\_BMR\_all

## PURPOSE

**This script repeats the simulation described in Thiele et al.,**

## SYNOPSIS

**This is a script file.**

## DESCRIPTION

```
 This script repeats the simulation described in Thiele et al.,
 "Personalized whole-body models integrate metabolism, physiology, and the gut microbiome", Method section 3.9.1. Identify  parameters that affect prediction accuracy.
 The input data come from A. M. Prentice et al., High levels of energy expenditure in obese women. Brit Med J, 292: 983-287 (1986).
```

## CROSS-REFERENCE INFORMATION

This function calls:

- adjustWholeBodyRxnCoeff [model] = adjustWholeBodyRxnCoeff(model, listOrgan, listCoeff)
- calcOrganFract This function extrapolates the organ weight fractions based on polynomials given in
- calculateBMR This function calculates the basal metabolic rate using the
- setSimulationConstraints This function sets the remaining simulation constraints that I identified during the
- loadPSCMfile Loads a mat file into the workspace, given a nickname or a full filename
- optimizeWBModel Solves flux balance analysis problems, and variants thereof
- EUAverageDietNew Average European Diet defintion. For details, please see https://www.vmh.life/#nutrition
- individualizedLabReport This function computes personalized physiolgical parameters based on the
- physiologicalConstraintsHMDBbased This function applies constraints to the whole-body metabolic model
- setDietConstraints This function sets diet constraints onto the bounds of the diet uptale
- standardPhysiolDefaultParameters This script creates the IndividualParameters structure which contains

This function is called by:

## SOURCE CODE

```
0001 % This script repeats the simulation described in Thiele et al.,
0002 % "Personalized whole-body models integrate metabolism, physiology, and the gut microbiome", Method section 3.9.1. Identify  parameters that affect prediction accuracy.
0003 % The input data come from A. M. Prentice et al., High levels of energy expenditure in obese women. Brit Med J, 292: 983-287 (1986).
0004 
0005 if ~exist('fatFreeAdj','var')
0006     fatFreeAdj = 0; % adjust for fat free mass measured
0007 end
0008 
0009 %%%%%%%%%%%%%%%%%%%%%%%%%%%%%%%%%%%%
0010 if ~exist('useSolveCobraLPCPLEX','var')
0011     global useSolveCobraLPCPLEX
0012 end
0013 
0014 sex = female.sex;
0015 standardPhysiolDefaultParameters;
0016 IndividualParameters_female = IndividualParameters;
0017 
0018 if ~exist('female','var')
0019     % only female data were available
0020     female = loadPSCMfile('Harvetta');
0021 end
0022 
0023 female = physiologicalConstraintsHMDBbased(female,IndividualParameters_female);
0024 EUAverageDietNew;
0025 female = setDietConstraints(female, Diet);
0026 
0027 female = setSimulationConstraints(female);
0028 
0029 % female.lb(strmatch('BBB_KYNATE[CSF]upt',female.rxns)) = -1000000;
0030 % female.lb(strmatch('BBB_LKYNR[CSF]upt',female.rxns)) = -1000000;
0031 % female.lb(strmatch('BBB_TRP_L[CSF]upt',female.rxns)) = -1000000;
0032 % female.ub(strmatch('Brain_EX_glc_D(',female.rxns)) = -100; % currently -400 rendering many of the models to be infeasible in germfree state
0033 
0034 
0035 minInf = -1000000;
0036 maxInf = 1000000;
0037 minConstraints = length(intersect(find(female.lb>minInf),find(female.lb)));
0038 maxConstraints =length(intersect(find(female.ub<maxInf),find(female.ub)));
0039 
0040 if 0
0041     female = changeObjective(female,'Whole_body_objective_rxn');
0042     female.osenseStr = 'max';
0043 else
0044     female.c(:) = 0;
0045 end
0046 
0047 Sum_atp_femaleBMR2 = [];
0048 
0049 % adjust Muscle and fat atp in biomass - compute and compare BMF's with
0050 % BMRs
0051 
0052 sensi_analysis = [1; 5; 10; ];
0053 
0054 cnt = 1;
0055 for y = 1 : length(sensi_analysis)
0056     femaleBMR = female;
0057     
0058     if useSolveCobraLPCPLEX
0059         femaleBMR.S = femaleBMR.A;
0060     end
0061     
0062     % femaleBMR
0063     BM = (find(~cellfun(@isempty,strfind(femaleBMR.rxns,'Muscle_biomass_'))));
0064     
0065     % find all reactions in the model that involve atp
0066     atp = (find(~cellfun(@isempty,strfind(femaleBMR.mets,'_atp[c'))));
0067     adp = (find(~cellfun(@isempty,strfind(femaleBMR.mets,'_adp[c'))));
0068     pi = (find(~cellfun(@isempty,strfind(femaleBMR.mets,'_pi[c'))));
0069     h = (find(~cellfun(@isempty,strfind(femaleBMR.mets,'_h[c'))));
0070     h2o = (find(~cellfun(@isempty,strfind(femaleBMR.mets,'_h2o[c'))));
0071     BMatpSum = 0;
0072     for i = 1: length(BM)
0073         % grab atp
0074         BMmets = (find(femaleBMR.S(:,BM(i))));
0075         BMatp = intersect(BMmets,atp);
0076         femaleBMR.S(BMatp,BM(i))= femaleBMR.S(BMatp,BM(i)) *sensi_analysis(y);
0077         BMatp = intersect(BMmets,adp);
0078         femaleBMR.S(BMatp,BM(i))= femaleBMR.S(BMatp,BM(i)) *sensi_analysis(y);
0079         BMatp = intersect(BMmets,pi);
0080         femaleBMR.S(BMatp,BM(i))= femaleBMR.S(BMatp,BM(i)) *sensi_analysis(y);
0081         BMatp = intersect(BMmets,h);
0082         femaleBMR.S(BMatp,BM(i))= femaleBMR.S(BMatp,BM(i)) *sensi_analysis(y);
0083         BMatp= intersect(BMmets,h2o);
0084         femaleBMR.S(BMatp,BM(i))= femaleBMR.S(BMatp,BM(i)) *sensi_analysis(y);
0085     end
0086     
0087     Energy_kJ_femaleBMR2(1,cnt) = sensi_analysis(y);
0088     for z = 1%: length(sensi_analysis)
0089         femaleBMR2 = femaleBMR;
0090         BM = (find(~cellfun(@isempty,strfind(femaleBMR2.rxns,'Adipocytes_biomass_'))));
0091         for i = 1: length(BM)
0092             % grab atp
0093             BMmets = (find(femaleBMR2.S(:,BM(i))));
0094             BMatp = intersect(BMmets,atp);
0095             femaleBMR2.S(BMatp,BM(i))= femaleBMR2.S(BMatp,BM(i)) *sensi_analysis(z);
0096             BMatp = intersect(BMmets,adp);
0097             femaleBMR2.S(BMatp,BM(i))= femaleBMR2.S(BMatp,BM(i)) *sensi_analysis(z);
0098             BMatp = intersect(BMmets,pi);
0099             femaleBMR2.S(BMatp,BM(i))= femaleBMR2.S(BMatp,BM(i))*sensi_analysis(z);
0100             BMatp = intersect(BMmets,h);
0101             femaleBMR2.S(BMatp,BM(i))= femaleBMR2.S(BMatp,BM(i)) *sensi_analysis(z);
0102             BMatp= intersect(BMmets,h2o);
0103             femaleBMR2.S(BMatp,BM(i))= femaleBMR2.S(BMatp,BM(i)) *sensi_analysis(z);
0104         end
0105         
0106         Energy_kJ_femaleBMR2(2,cnt) = sensi_analysis(z);
0107         
0108         if useSolveCobraLPCPLEX
0109             femaleBMR2.A = femaleBMR2.S;
0110         end
0111         
0112         InputDataAll =  {
0113             'sex', '','female', 'female', 'female', 'female', 'female', 'female', 'female', 'female', 'female', 'female', 'female', 'female', 'female', 'female', 'female', 'female', 'female', 'female', 'female', 'female', 'female', 'female';
0114             'age' , '', num2str(37),  num2str(33), num2str(31), num2str(23), num2str(23), num2str(32), num2str(28), num2str(26), num2str(24), num2str(24), num2str(31), num2str(29), num2str(38), num2str(32), num2str(27), num2str(40), num2str(39), num2str(36), num2str(38), num2str(26), num2str(37), num2str(39);
0115             'Height' , '', num2str(161), num2str(158), num2str(163), num2str(165), num2str(163), num2str(158), num2str(163), num2str(160), num2str(156), num2str(180), num2str(151), num2str(150), num2str(170), num2str(161), num2str(160), num2str(168), num2str(161), num2str(164), num2str(162), num2str(160), num2str(169), num2str(165);
0116             'Weight', '', num2str(57.6), num2str(49.5), num2str(55.7), num2str(56.2), num2str(53.2), num2str(49.7), num2str(64.6), num2str(60.6), num2str(59), num2str(71.4), num2str(62.9), num2str(51), num2str(56.4), num2str(74.9), num2str(88.6), num2str(120.1), num2str(83.1), num2str(80.5), num2str(80.3), num2str(79.7), num2str(82.4), num2str(102.1);
0117             'Fat-free','',  num2str(41.0),  num2str(43.6), num2str(42.9), num2str(41.5), num2str(37.6), num2str(35), num2str(40.8), num2str(43.5), num2str(41.9), num2str(50.2), num2str(43.3), num2str(35.3), num2str(40.7), num2str(44.4), num2str(47.5), num2str(60.6), num2str(47.5), num2str(48.8), num2str(45.9), num2str(44.8), num2str(48.4), num2str(54.2);
0118             'BMR','', num2str(5.37), num2str(6.19), num2str(5.91), num2str(5.85), num2str(5.06), num2str(5.31), num2str(5.54), num2str(5.65), num2str(5.76), num2str(6.4), num2str(5.9), num2str(4.67), num2str(5.86), num2str(6.7), num2str(6.77), num2str(8.2), num2str(6.64), num2str(6.54), num2str(6.42), num2str(6.43), num2str(5.59), num2str(6.73);
0119             };
0120         femaleBMR2O=femaleBMR2;
0121         for i = 3 :  15% size(InputDataAll,2) % only normal BMI's
0122             InputData =  [InputDataAll(:,1), InputDataAll(:,2), InputDataAll(:,i)];
0123             femaleBMR2 = femaleBMR2O;
0124             
0125             sex =  InputDataAll(1,i);
0126             femaleBMR2.sex = sex;
0127             
0128             optionCardiacOutput = 0; %0 4 2
0129             [femaleBMR2,IndividualParametersPersonalized] = individualizedLabReport(femaleBMR2,IndividualParameters_female, InputData,optionCardiacOutput);
0130             % 3. set personalized constraints
0131             femaleBMR2.IndividualParametersPersonalized=IndividualParametersPersonalized;
0132             % 3. set personalized constraints
0133             
0134             % calculate new organ weight fraction for a given personalized weight
0135             [listOrgan,OrganWeight,OrganWeightFract,IndividualParametersPersonalized] = calcOrganFract(femaleBMR2,IndividualParametersPersonalized);
0136             
0137             % I repeat this step, as I calculate in some options the CO based on
0138             % bloodVol which is only calculated in calcOrganFract
0139             [femaleBMR2,IndividualParametersPersonalized] = individualizedLabReport(femaleBMR2,IndividualParametersPersonalized, InputData,optionCardiacOutput);
0140             femaleBMR2.IndividualParametersPersonalized=IndividualParametersPersonalized;
0141             
0142             if fatFreeAdj ==1
0143                 % adjust whole body maintenance reaction based on new organ weight
0144                 % fractions
0145                 % adjust adipocyte and muscle weight to measured one
0146                 Fat_fraction = 1 - str2num(InputData{5,3})/str2num(InputData{4,3});
0147                 FatDiff = Fat_fraction-OrganWeightFract(11)
0148                 OrganWeightFract(11) = OrganWeightFract(11)+FatDiff;
0149                 OrganWeightFract(12) = OrganWeightFract(12)-FatDiff;
0150                 
0151                 Fat_weight =  (str2num(InputData{4,3})-str2num(InputData{5,3}))*1000;
0152                 Fat_weightOri=str2num(IndividualParametersPersonalized.OrgansWeights{11,2});
0153                 Muscle_weightOri=str2num(IndividualParametersPersonalized.OrgansWeights{12,2});
0154                 % substract higher fat_weight from Muscle_weight
0155                 Muscle_weight = Muscle_weightOri-(Fat_weight - Fat_weightOri);
0156                 
0157                 IndividualParametersPersonalized.OrgansWeights{11,2} = num2str(Fat_weight);
0158                 IndividualParametersPersonalized.OrgansWeights{11,3} = num2str(OrganWeightFract(11)*100);
0159                 IndividualParametersPersonalized.OrgansWeights{12,2} = num2str(Muscle_weight);
0160                 IndividualParametersPersonalized.OrgansWeights{12,3} = num2str(OrganWeightFract(12)*100);
0161             end
0162             %adjusts whole body reaction coefficients and prints out 'Whole_body_objective_rxn'
0163             [femaleBMR2] = adjustWholeBodyRxnCoeff(femaleBMR2, listOrgan, OrganWeightFract);
0164             femaleBMR2.listOrgan = listOrgan;
0165             femaleBMR2.OrganWeightFract = OrganWeightFract;
0166             femaleBMR2 = setDietConstraints(femaleBMR2);
0167             % set some more constraints
0168             %femaleBMR2 = setSimulationConstraints(femaleBMR2);
0169             femaleBMR2 = physiologicalConstraintsHMDBbased(femaleBMR2,IndividualParametersPersonalized);
0170             
0171             %femaleBMR2.ub(strmatch('Brain_EX_glc_D(',femaleBMR2.rxns)) = -100; % currently -400 rendering many of the models to be infeasible in germfree state
0172             
0173             CO(i,cnt) = IndividualParametersPersonalized.CardiacOutput;
0174             BM = (find(~cellfun(@isempty,strfind(femaleBMR2.rxns,'Heart_DM_atp'))));
0175             femaleBMR2.lb(BM) = 1000; % as BMR is determined at absolute rest
0176 
0177             if useSolveCobraLPCPLEX
0178                 femaleBMR2 = changeObjective(femaleBMR2,'Whole_body_objective_rxn');
0179                 femaleBMR2.osense = -1;
0180                 [solution] = computeMin2Norm_HH(femaleBMR2);
0181             else
0182                 param.minNorm = 1e-6;
0183                 solution = optimizeWBModel(femaleBMR2, param);
0184             end
0185             %stat(i,cnt) = solution.stat;
0186             %origStat(i,cnt) = solution.origStat;
0187             
0188             if solution.stat==1 || solution.stat==3
0189                 if useSolveCobraLPCPLEX
0190                     F = max(-femaleBMR2.A,0);
0191                     R = max(femaleBMR2.A,0);
0192                 else
0193                     F = max(-femaleBMR2.S,0);
0194                     R = max(femaleBMR2.S,0);
0195                 end
0196                 vf = max(solution.v,0);
0197                 vr = max(-solution.v,0);
0198                 production=[R F]*[vf; vr];
0199                 consumption=[F R]*[vf; vr];
0200                 % find all reactions in the model that involve atp
0201                 atp = (find(~cellfun(@isempty,strfind(femaleBMR2.mets,'_atp['))));
0202                 % sum of atp consumption in the flux distribution
0203                 Sum_atp_femaleBMR2=sum(consumption(atp,1)); % (in mmol ATP/day/person)
0204                 % compute the energy release in kJ
0205                 Energy_kJ_femaleBMR2(i,cnt) = Sum_atp_femaleBMR2/1000 * 64; % (in kJ/day/person)
0206            else
0207                % NaN if no solution reported
0208                Energy_kJ_femaleBMR2(i,cnt) = NaN;
0209            end
0210             
0211             % compute the energy release in kcal, where 1 kJ = 0.239006 kcal
0212             Energy_kcal_femaleBMR2(i,cnt) =  Energy_kJ_femaleBMR2(i,cnt) *0.239006; % (in kcal/day/person)
0213             Energy_kcal_femaleBMR2_origStatText{i,cnt}  = solution.origStatText;
0214         end
0215         cnt = cnt +1;
0216         if 0
0217             if fatFreeAdj ==1
0218                 %save Results_sensi_2018_12_14_fatAdj
0219                 save([resultsPath 'Results_sensi_fatAdj'])
0220                 
0221             else
0222                 %save Results_sensi_2018_12_14_no_fatAdj
0223                 save([resultsPath 'Results_sensi_no_fatAdj'])
0224             end
0225         end
0226     end
0227 end
0228 for i = 3 : 15 % size(InputDataAll,2) - only normal BMI's
0229     BMR_female_All(:,i) = calculateBMR('female', str2num(InputDataAll{4,i}), str2num(InputDataAll{3,i}), str2num(InputDataAll{2,i}));
0230 end
0231 % if fatFreeAdj ==1
0232 %     save Results_sensi_2018_12_14_fatAdj
0233 % else
0234 %     save Results_sensi_2018_12_14_no_fatAdj
0235 % end
0236 
0237 if ~exist('resultsPath','var')
0238     global resultsPath
0239     resultsPath = which('MethodSection3.mlx');
0240     resultsPath = strrep(resultsPath,'MethodSection3.mlx',['Results' filesep]);
0241 end
0242 
0243 if fatFreeAdj ==1
0244     %save Results_sensi_2018_12_14_fatAdj
0245     save([resultsPath 'Results_sensi_fatAdj'],'BMR_female_All','InputDataAll','Energy_kcal_femaleBMR2','Sum_atp_femaleBMR2','Energy_kcal_femaleBMR2_origStatText')
0246 else
0247     %save Results_sensi_2018_12_14_no_fatAdj
0248     save([resultsPath 'Results_sensi_no_fatAdj'],'BMR_female_All','InputDataAll','Energy_kcal_femaleBMR2','Sum_atp_femaleBMR2','Energy_kcal_femaleBMR2_origStatText')
0249 end
```

---

Generated on Thu 14-May-2020 13:05:49 by **m2html** © 2005
