# Supplementary material for: Personalized whole‐body models integrate metabolism, physiology, and the gut microbiome
Source: Mol Syst Biol. 2020 May 28;16(5):e8982. doi: 10.15252/msb.20198982 (PMC7285886; doi:10.15252/msb.20198982)
Supplement: Supplementary file 22 — Dataset EV1 [file MSB-16-e8982-s022.zip › PSCM_toolbox/PSCM_toolbox_doc/src/setConstraints/organWeight/getOrganWeightFraction.html]

Description of getOrganWeightFraction


# getOrganWeightFraction

## PURPOSE

**This script reads in the organ file and assign biomass\_maintenance coefficient according**

## SYNOPSIS

**This is a script file.**

## DESCRIPTION

```
 This script reads in the organ file and assign biomass_maintenance coefficient according
 to relative fraction of organs.

 Ines Thiele - 2015/2016
```

## CROSS-REFERENCE INFORMATION

This function calls:


This function is called by:

- calcOrganFract This function extrapolates the organ weight fractions based on polynomials given in
- organEssentiality This function computes the organ essentiality in a whole-body model by
- standardPhysiolDefaultParameters This script creates the IndividualParameters structure which contains

## SOURCE CODE

```
0001 % This script reads in the organ file and assign biomass_maintenance coefficient according
0002 % to relative fraction of organs.
0003 %
0004 % Ines Thiele - 2015/2016
0005 
0006 % fileNameOrgan = '16_01_29_OrganWeights.xlsx';
0007 % [Numbers, organWeightData] = xlsread(fileNameOrgan,'OrganWeights');
0008 load Numbers_organWeightData_fromxls_16_01_29_OrganWeigths;
0009 
0010 % find start of data
0011 for i = 1 : size(organWeightData,1)
0012     Cols = find(ismember(organWeightData(i,:),'% of body weight'));
0013     if length(Cols)>0
0014         if strcmp(sex,'male')
0015             if  exist('weigth', 'var') && weigth == 100 % 100 kg male
0016                 Col = Cols(3)-2;
0017             elseif  exist('weigth', 'var') && weigth ==125
0018                 Col = Cols(4)-2;
0019             else
0020                 Col = Cols(1)-2
0021             end
0022         elseif strcmp(sex,'female')
0023             Col = Cols(2)-2;
0024         end
0025     end
0026     if length(find(ismember(organWeightData(i,1),'Body weight')))>0
0027         organRow = i+1;
0028         break;
0029     end
0030 end
0031 
0032 OrganWeightFract = Numbers(2:end,Col)/100; % assumes that the relative organ weights are given in %
0033 OrganWeight = Numbers(2:end,Col-1); % assumes that the organ weights appear in the col before the percentage
0034 OrganNames = organWeightData(organRow:end,1);
0035 BodyWeight = Numbers(1,Col-1); %total body weight is in first row of numbers
0036 ObjectiveComponents = strcat(OrganNames,'_biomass_maintenance');
0037 %make exception for non-translating organs
0038 ObjectiveComponents(strmatch('Platelet',OrganNames)) ={'Platelet_biomass_maintenance_noTrTr'};
0039 ObjectiveComponents(strmatch('RBC',OrganNames)) ={'RBC_biomass_maintenance_noTrTr'};
0040 ObjectiveComponents(strmatch('sIEC',OrganNames)) ={'sIEC_biomass_reactionIEC01b'};
0041 
0042 %clear Col* Numbers ans i organ*
```

---

Generated on Thu 14-May-2020 13:05:49 by **m2html** © 2005
