# Supplementary material for: Personalized whole‐body models integrate metabolism, physiology, and the gut microbiome
Source: Mol Syst Biol. 2020 May 28;16(5):e8982. doi: 10.15252/msb.20198982 (PMC7285886; doi:10.15252/msb.20198982)
Supplement: Supplementary file 22 — Dataset EV1 [file MSB-16-e8982-s022.zip › PSCM_toolbox/PSCM_toolbox_doc/src/setConstraints/diets/Mediterranian.html]

Description of Mediterranian


# Mediterranian

## PURPOSE

**Mediterranian diet defintion. For details, please see https://www.vmh.life/#nutrition**

## SYNOPSIS

**This is a script file.**

## DESCRIPTION

```
 Mediterranian diet defintion. For details, please see https://www.vmh.life/#nutrition
 Ines Thiele 2016-2019
```

## CROSS-REFERENCE INFORMATION

This function calls:


This function is called by:

## SOURCE CODE

```
0001 % Mediterranian diet defintion. For details, please see https://www.vmh.life/#nutrition
0002 % Ines Thiele 2016-2019
0003 
0004 Diet = {%'Reaction'    'Flux Value'
0005 'EX_etoh(e)'    '355.9923939'
0006 'EX_h2o(e)'    '220593.0188'
0007 'EX_caro(e)'    '0.005979072'
0008 'EX_retinol(e)'    '0.558558584'
0009 'EX_thm(e)'    '8.328478195'
0010 'EX_adpcbl(e)'    '1.22881E-05'
0011 'EX_ribflv(e)'    '0.006111107'
0012 'EX_nac(e)'    '0.445244325'
0013 'EX_ncam(e)'    '0.211750949'
0014 'EX_pnto_R(e)'    '0.085186526'
0015 'EX_pydam(e)'    '0.004669003'
0016 'EX_pydxn(e)'    '0.004669643'
0017 'EX_pydx(e)'    '0.004725954'
0018 'EX_btn(e)'    '0.00036173'
0019 'EX_10fthf(e)'    '0.000264396'
0020 'EX_5mthf(e)'    '0.000271879'
0021 'EX_thf(e)'    '0.000281098'
0022 'EX_ascb_L(e)'    '0.782052146'
0023 'EX_vitd3(e)'    '3.18352E-05'
0024 'EX_avite1(e)'    '0.054573176'
0025 'EX_phyQ(e)'    '0.000301578'
0026 'EX_ca2(e)'    '44.23174809'
0027 'EX_cl(e)'    '543.2352692'
0028 'EX_k(e)'    '101.3033815'
0029 'EX_mg2(e)'    '25.58362477'
0030 'EX_na1(e)'    '150.9506232'
0031 'EX_pi(e)'    '23.46662249'
0032 'EX_cu2(e)'    '0.04957039'
0033 'EX_fe2(e)'    '0.200734175'
0034 'EX_fe3(e)'    '0.200734175'
0035 'EX_mn2(e)'    '0.136335394'
0036 'EX_zn2(e)'    '0.261394922'
0037 'EX_mnl(e)'    '0.779483982'
0038 'EX_xylt(e)'    '0'
0039 'EX_lcts(e)'    '35.94427638'
0040 'EX_malt(e)'    '4.206879124'
0041 'EX_sucr(e)'    '110.5182203'
0042 'EX_fru(e)'    '125.4517893'
0043 'EX_gal(e)'    '10.8426091'
0044 'EX_cellul(e)'    '0.14529578'
0045 'EX_ala_L(e)'    '61.65072082'
0046 'EX_arg_L(e)'    '42.32821506'
0047 'EX_asp_L(e)'    '80.87258611'
0048 'EX_cys_L(e)'    '13.29163028'
0049 'EX_glu_L(e)'    '168.7925032'
0050 'EX_gly(e)'    '67.07363861'
0051 'EX_urate(e)'    '5.780252608'
0052 'EX_his_L(e)'    '18.55149638'
0053 'EX_ile_L(e)'    '45.97634115'
0054 'EX_leu_L(e)'    '73.5657289'
0055 'EX_lys_L(e)'    '49.72502556'
0056 'EX_met_L(e)'    '15.16359686'
0057 'EX_phe_L(e)'    '34.89104305'
0058 'EX_pro_L(e)'    '78.89334277'
0059 'EX_ser_L(e)'    '59.78051737'
0060 'EX_thr_L(e)'    '39.41362937'
0061 'EX_trp_L(e)'    '6.728111908'
0062 'EX_tyr_L(e)'    '23.68527804'
0063 'EX_val_L(e)'    '54.75068355'
0064 'EX_dca(e)'    '3.170503694'
0065 'EX_ddca(e)'    '3.503490546'
0066 'EX_ttdca(e)'    '12.65654482'
0067 'EX_ttdcea(e)'    '1.377519391'
0068 'EX_ptdca(e)'    '1.183522405'
0069 'EX_hdca(e)'    '56.41911375'
0070 'EX_hpdca(e)'    '0.886830484'
0071 'EX_ocdca(e)'    '13.64408774'
0072 'EX_ocdcea(e)'    '128.1607541'
0073 'EX_lnlc(e)'    '73.59427421'
0074 'EX_lnlnca(e)'    '7.87609621'
0075 'EX_strdnc(e)'    '0.614547697'
0076 'EX_arach(e)'    '1.876943078'
0077 'EX_CE2510(e)'    '5.347026525'
0078 'EX_CE4843(e)'    '0.130800704'
0079 'EX_arachd(e)'    '0.268570142'
0080 'EX_docosac(e)'    '2.557574808'
0081 'EX_doco13ac(e)'    '4.673543672'
0082 'EX_adrn(e)'    '0.025338442'
0083 'EX_clpnd(e)'    '0.108954216'
0084 'EX_crvnc(e)'    '3.860995608'
0085 'EX_but(e)'    '8.165474895'
0086 'EX_octa(e)'    '1.800374991'
0087 'EX_chsterol(e)'    '0.687954461'
0088 'EX_sbt_D(e)'    '8.441207695'
0089 'EX_glc_D(e)'    '96.02127935'
0090 'EX_hdcea(e)'    '5.649717068'
0091 'EX_lgnc(e)'    '0.909993994'
0092 'EX_fol(e)'    '0.000283028'
0093 'EX_strch1(e)'    '41.02641436'
0094 'EX_i(e)'    '0.003914204'
0095 'EX_starch1200(e)'    '0.379838969'
0096 
0097 
0098     };
0099 Diet = regexprep(Diet,'EX_','Diet_EX_');
0100 Diet = regexprep(Diet,'\(e\)','\[d\]');
```

---

Generated on Thu 14-May-2020 13:05:49 by **m2html** © 2005
