# Supplementary material for: Personalized whole‐body models integrate metabolism, physiology, and the gut microbiome
Source: Mol Syst Biol. 2020 May 28;16(5):e8982. doi: 10.15252/msb.20198982 (PMC7285886; doi:10.15252/msb.20198982)
Supplement: Supplementary file 22 — Dataset EV1 [file MSB-16-e8982-s022.zip › PSCM_toolbox/PSCM_toolbox_doc/src/setConstraints/diets/DACH.html]

Description of DACH


# DACH

## PURPOSE

**DACH diet definition. For details, please refer to https://www.vmh.life/#diet/DACH**

## SYNOPSIS

**This is a script file.**

## DESCRIPTION

```
 DACH diet definition. For details, please refer to https://www.vmh.life/#diet/DACH
 Ines Thiele 2016 - 2019
```

## CROSS-REFERENCE INFORMATION

This function calls:


This function is called by:

## SOURCE CODE

```
0001 % DACH diet definition. For details, please refer to https://www.vmh.life/#diet/DACH
0002 % Ines Thiele 2016 - 2019
0003 
0004 Diet = {%'Reaction'    'Flux Value'
0005 'EX_etoh(e)'    '0'
0006 'EX_h2o(e)'    '197316.5002'
0007 'EX_caro(e)'    '0.024810355'
0008 'EX_retinol(e)'    '0.907657699'
0009 'EX_thm(e)'    '5.351329881'
0010 'EX_adpcbl(e)'    '8.0591E-06'
0011 'EX_ribflv(e)'    '0.004490335'
0012 'EX_nac(e)'    '0.351408459'
0013 'EX_ncam(e)'    '0.167881819'
0014 'EX_pnto_R(e)'    '0.027631778'
0015 'EX_pydam(e)'    '0.004688704'
0016 'EX_pydxn(e)'    '0.004689347'
0017 'EX_pydx(e)'    '0.004745895'
0018 'EX_btn(e)'    '0.000235509'
0019 'EX_10fthf(e)'    '0.000222995'
0020 'EX_5mthf(e)'    '0.000229306'
0021 'EX_thf(e)'    '0.000237081'
0022 'EX_ascb_L(e)'    '1.086421473'
0023 'EX_vitd3(e)'    '8.04913E-05'
0024 'EX_avite1(e)'    '0.054677656'
0025 'EX_phyQ(e)'    '0.000938105'
0026 'EX_ca2(e)'    '41.54149409'
0027 'EX_cl(e)'    '293.8058838'
0028 'EX_k(e)'    '108.4415435'
0029 'EX_mg2(e)'    '28.8006583'
0030 'EX_na1(e)'    '190.5912994'
0031 'EX_pi(e)'    '21.44837481'
0032 'EX_cu2(e)'    '0.036304409'
0033 'EX_fe2(e)'    '0.328964097'
0034 'EX_fe3(e)'    '0.328964097'
0035 'EX_mn2(e)'    '0.143234074'
0036 'EX_zn2(e)'    '0.219027225'
0037 'EX_mnl(e)'    '0.702633448'
0038 'EX_xylt(e)'    '0.026290571'
0039 'EX_lcts(e)'    '10.15610735'
0040 'EX_malt(e)'    '0.584288767'
0041 'EX_sucr(e)'    '137.424718'
0042 'EX_fru(e)'    '99.23349721'
0043 'EX_gal(e)'    '0.034969712'
0044 'EX_cellul(e)'    '0.105778496'
0045 'EX_ala_L(e)'    '57.90823542'
0046 'EX_arg_L(e)'    '29.80738992'
0047 'EX_asp_L(e)'    '68.76755843'
0048 'EX_cys_L(e)'    '9.567656172'
0049 'EX_glu_L(e)'    '145.9493585'
0050 'EX_gly(e)'    '57.38690709'
0051 'EX_urate(e)'    '4.474443267'
0052 'EX_his_L(e)'    '18.57282994'
0053 'EX_ile_L(e)'    '39.35347926'
0054 'EX_leu_L(e)'    '64.6490243'
0055 'EX_lys_L(e)'    '47.49194099'
0056 'EX_met_L(e)'    '15.84980494'
0057 'EX_phe_L(e)'    '26.9572266'
0058 'EX_pro_L(e)'    '69.75223768'
0059 'EX_ser_L(e)'    '49.13314544'
0060 'EX_thr_L(e)'    '36.45793457'
0061 'EX_trp_L(e)'    '5.551077928'
0062 'EX_tyr_L(e)'    '19.13195374'
0063 'EX_val_L(e)'    '50.52741743'
0064 'EX_dca(e)'    '3.816493019'
0065 'EX_ddca(e)'    '3.95514922'
0066 'EX_ttdca(e)'    '18.44275454'
0067 'EX_ttdcea(e)'    '2.058158281'
0068 'EX_ptdca(e)'    '1.662457703'
0069 'EX_hdca(e)'    '45.44310245'
0070 'EX_hpdca(e)'    '1.031016984'
0071 'EX_ocdca(e)'    '13.11006165'
0072 'EX_ocdcea(e)'    '58.34962377'
0073 'EX_lnlc(e)'    '52.96354283'
0074 'EX_lnlnca(e)'    '4.217406209'
0075 'EX_strdnc(e)'    '5.429079562'
0076 'EX_arach(e)'    '0.486642217'
0077 'EX_CE2510(e)'    '10.28572573'
0078 'EX_CE4843(e)'    '0.287163156'
0079 'EX_arachd(e)'    '0.261320396'
0080 'EX_docosac(e)'    '0.362216838'
0081 'EX_doco13ac(e)'    '14.0043376'
0082 'EX_adrn(e)'    '0.065155993'
0083 'EX_clpnd(e)'    '0.452509012'
0084 'EX_crvnc(e)'    '2.740012147'
0085 'EX_but(e)'    '10.49517091'
0086 'EX_octa(e)'    '2.131232826'
0087 'EX_chsterol(e)'    '0.51337955'
0088 'EX_sbt_D(e)'    '0.33429982'
0089 'EX_glc_D(e)'    '90.01092942'
0090 'EX_hdcea(e)'    '9.200462667'
0091 'EX_lgnc(e)'    '0.085956269'
0092 'EX_fol(e)'    '0.000238709'
0093 'EX_strch1(e)'    '37.50276357'
0094 'EX_i(e)'    '0.004352408'
0095 'EX_starch1200(e)'    '0.3472156'
0096 
0097     };
0098 Diet = regexprep(Diet,'EX_','Diet_EX_');
0099 Diet = regexprep(Diet,'\(e\)','\[d\]');
```

---

Generated on Thu 14-May-2020 13:05:49 by **m2html** © 2005
