# Supplementary material for: Personalized whole‐body models integrate metabolism, physiology, and the gut microbiome
Source: Mol Syst Biol. 2020 May 28;16(5):e8982. doi: 10.15252/msb.20198982 (PMC7285886; doi:10.15252/msb.20198982)
Supplement: Supplementary file 22 — Dataset EV1 [file MSB-16-e8982-s022.zip › PSCM_toolbox/PSCM_toolbox_doc/src/setConstraints/diets/VegetarianDiet.html]

Description of VegetarianDiet


# VegetarianDiet

## PURPOSE

**Vegetarian diet defintion. For details, please see https://www.vmh.life/#nutrition**

## SYNOPSIS

**This is a script file.**

## DESCRIPTION

```
 Vegetarian diet defintion. For details, please see https://www.vmh.life/#nutrition
 Ines Thiele 2016-2019
```

## CROSS-REFERENCE INFORMATION

This function calls:


This function is called by:

- analyzeHMmodel This function performs host-microbiome optimization for a set of defined
- setDietConstraints This function sets diet constraints onto the bounds of the diet uptale

## SOURCE CODE

```
0001 % Vegetarian diet defintion. For details, please see https://www.vmh.life/#nutrition
0002 % Ines Thiele 2016-2019
0003 
0004 Diet = {%'Reaction'    'Flux Value'
0005 'EX_etoh(e)'    '26.80796381'
0006 'EX_h2o(e)'    '188463.6264'
0007 'EX_caro(e)'    '0.007916217'
0008 'EX_retinol(e)'    '0.209459469'
0009 'EX_thm(e)'    '2.958305603'
0010 'EX_adpcbl(e)'    '9.31E-07'
0011 'EX_ribflv(e)'    '0.001873187'
0012 'EX_nac(e)'    '0.20869932'
0013 'EX_ncam(e)'    '0.097461936'
0014 'EX_pnto_R(e)'    '0.018214978'
0015 'EX_pydam(e)'    '0.003309673'
0016 'EX_pydxn(e)'    '0.003310127'
0017 'EX_pydx(e)'    '0.003350044'
0018 'EX_btn(e)'    '0.000202793'
0019 'EX_10fthf(e)'    '0.000184725'
0020 'EX_5mthf(e)'    '0.000189953'
0021 'EX_thf(e)'    '0.000196394'
0022 'EX_ascb_L(e)'    '0.791103279'
0023 'EX_vitd3(e)'    '7.04559E-06'
0024 'EX_avite1(e)'    '0.041269441'
0025 'EX_phyQ(e)'    '0.000410099'
0026 'EX_ca2(e)'    '18.48183542'
0027 'EX_cl(e)'    '203.5486419'
0028 'EX_k(e)'    '75.81327065'
0029 'EX_mg2(e)'    '15.67537544'
0030 'EX_na1(e)'    '106.469968'
0031 'EX_pi(e)'    '10.71283079'
0032 'EX_cu2(e)'    '0.0373745'
0033 'EX_fe2(e)'    '0.108559405'
0034 'EX_fe3(e)'    '0.108559405'
0035 'EX_mn2(e)'    '0.125687035'
0036 'EX_zn2(e)'    '0.116396452'
0037 'EX_mnl(e)'    '0.474277578'
0038 'EX_xylt(e)'    '0.075388213'
0039 'EX_lcts(e)'    '8.533946447'
0040 'EX_malt(e)'    '0.262929945'
0041 'EX_sucr(e)'    '87.59949342'
0042 'EX_fru(e)'    '204.9397772'
0043 'EX_gal(e)'    '0.979151946'
0044 'EX_cellul(e)'    '0.079528166'
0045 'EX_ala_L(e)'    '25.69337503'
0046 'EX_arg_L(e)'    '17.86199217'
0047 'EX_asp_L(e)'    '35.14974484'
0048 'EX_cys_L(e)'    '6.746097251'
0049 'EX_glu_L(e)'    '83.42890462'
0050 'EX_gly(e)'    '30.26206062'
0051 'EX_urate(e)'    '2.124379054'
0052 'EX_his_L(e)'    '7.81807307'
0053 'EX_ile_L(e)'    '18.99565383'
0054 'EX_leu_L(e)'    '31.40671587'
0055 'EX_lys_L(e)'    '17.25395817'
0056 'EX_met_L(e)'    '6.125172825'
0057 'EX_phe_L(e)'    '15.54751494'
0058 'EX_pro_L(e)'    '36.97121962'
0059 'EX_ser_L(e)'    '25.34169865'
0060 'EX_thr_L(e)'    '16.51476001'
0061 'EX_trp_L(e)'    '3.023108803'
0062 'EX_tyr_L(e)'    '10.1519964'
0063 'EX_val_L(e)'    '25.01799032'
0064 'EX_dca(e)'    '1.806294294'
0065 'EX_ddca(e)'    '2.105641569'
0066 'EX_ttdca(e)'    '5.801977455'
0067 'EX_ttdcea(e)'    '0.715429664'
0068 'EX_ptdca(e)'    '0.621609216'
0069 'EX_hdca(e)'    '39.53952002'
0070 'EX_hpdca(e)'    '0.466147348'
0071 'EX_ocdca(e)'    '12.48152798'
0072 'EX_ocdcea(e)'    '82.41643199'
0073 'EX_lnlc(e)'    '88.19503467'
0074 'EX_lnlnca(e)'    '7.659818969'
0075 'EX_strdnc(e)'    '0'
0076 'EX_arach(e)'    '1.11155695'
0077 'EX_CE2510(e)'    '0.596998578'
0078 'EX_CE4843(e)'    '0.014504504'
0079 'EX_arachd(e)'    '0.001779483'
0080 'EX_docosac(e)'    '0.304806941'
0081 'EX_doco13ac(e)'    '0.124955675'
0082 'EX_adrn(e)'    '0'
0083 'EX_clpnd(e)'    '0'
0084 'EX_crvnc(e)'    '0.077867279'
0085 'EX_but(e)'    '4.691654841'
0086 'EX_octa(e)'    '1.040826516'
0087 'EX_chsterol(e)'    '0.104124235'
0088 'EX_sbt_D(e)'    '18.1276685'
0089 'EX_glc_D(e)'    '102.1380649'
0090 'EX_hdcea(e)'    '4.088531071'
0091 'EX_lgnc(e)'    '0.090254082'
0092 'EX_fol(e)'    '0.000197743'
0093 'EX_strch1(e)'    '41.33622192'
0094 'EX_i(e)'    '0.001071751'
0095 'EX_starch1200(e)'    '0.382707292'
0096 
0097     };
0098 Diet = regexprep(Diet,'EX_','Diet_EX_');
0099 Diet = regexprep(Diet,'\(e\)','\[d\]');
```

---

Generated on Thu 14-May-2020 13:05:49 by **m2html** © 2005
