# Supplementary material for: Personalized whole‐body models integrate metabolism, physiology, and the gut microbiome
Source: Mol Syst Biol. 2020 May 28;16(5):e8982. doi: 10.15252/msb.20198982 (PMC7285886; doi:10.15252/msb.20198982)
Supplement: Supplementary file 22 — Dataset EV1 [file MSB-16-e8982-s022.zip › PSCM_toolbox/PSCM_toolbox_doc/src/setConstraints/diets/UnhealthyDiet.html]

Description of UnhealthyDiet


# UnhealthyDiet

## PURPOSE

**Unhealthy diet defintion. For details, please see https://www.vmh.life/#nutrition**

## SYNOPSIS

**This is a script file.**

## DESCRIPTION

```
 Unhealthy diet defintion. For details, please see https://www.vmh.life/#nutrition
 Ines Thiele 2016-2019
```

## CROSS-REFERENCE INFORMATION

This function calls:


This function is called by:

- analyzeHMmodel This function performs host-microbiome optimization for a set of defined
- setDietConstraints This function sets diet constraints onto the bounds of the diet uptale

## SOURCE CODE

```
0001 % Unhealthy diet defintion. For details, please see https://www.vmh.life/#nutrition
0002 % Ines Thiele 2016-2019
0003 
0004 Diet = {%'Reaction'    'Flux Value'
0005 'EX_etoh(e)'    '294.3449306'
0006 'EX_h2o(e)'    '152721.301'
0007 'EX_caro(e)'    '0.002894541'
0008 'EX_retinol(e)'    '4.887387608'
0009 'EX_thm(e)'    '6.206788953'
0010 'EX_adpcbl(e)'    '8.24775E-06'
0011 'EX_ribflv(e)'    '0.006315696'
0012 'EX_nac(e)'    '0.455874825'
0013 'EX_ncam(e)'    '0.243513592'
0014 'EX_pnto_R(e)'    '0.029331843'
0015 'EX_pydam(e)'    '0.003973578'
0016 'EX_pydxn(e)'    '0.003974123'
0017 'EX_pydx(e)'    '0.004022046'
0018 'EX_btn(e)'    '0.0002698'
0019 'EX_10fthf(e)'    '0.000127851'
0020 'EX_5mthf(e)'    '0.00013147'
0021 'EX_thf(e)'    '0.000135927'
0022 'EX_ascb_L(e)'    '0.745870456'
0023 'EX_vitd3(e)'    '1.7055E-05'
0024 'EX_avite1(e)'    '0.053170828'
0025 'EX_phyQ(e)'    '0.000276422'
0026 'EX_ca2(e)'    '53.0445631'
0027 'EX_cl(e)'    '617.9611316'
0028 'EX_k(e)'    '89.05371845'
0029 'EX_mg2(e)'    '17.17514915'
0030 'EX_na1(e)'    '309.750825'
0031 'EX_pi(e)'    '28.47781761'
0032 'EX_cu2(e)'    '0.03298398'
0033 'EX_fe2(e)'    '0.170006267'
0034 'EX_fe3(e)'    '0.170006267'
0035 'EX_mn2(e)'    '0.056026748'
0036 'EX_zn2(e)'    '0.335041297'
0037 'EX_mnl(e)'    '0.133489377'
0038 'EX_sbt_D(e)'    '1.561163693'
0039 'EX_xylt(e)'    '0'
0040 'EX_lcts(e)'    '43.07096333'
0041 'EX_malt(e)'    '15.74658228'
0042 'EX_sucr(e)'    '223.6277613'
0043 'EX_fru(e)'    '92.43134974'
0044 'EX_gal(e)'    '0.330269505'
0045 'EX_glc_D(e)'    '119.070988'
0046 'EX_cellul(e)'    '0.033451281'
0047 'EX_ala_L(e)'    '86.3692852'
0048 'EX_arg_L(e)'    '46.64146627'
0049 'EX_asp_L(e)'    '101.0677416'
0050 'EX_cys_L(e)'    '16.97235515'
0051 'EX_glu_L(e)'    '220.123986'
0052 'EX_gly(e)'    '84.45734854'
0053 'EX_urate(e)'    '3.845195684'
0054 'EX_his_L(e)'    '30.96118968'
0055 'EX_ile_L(e)'    '61.08776279'
0056 'EX_leu_L(e)'    '101.9602982'
0057 'EX_lys_L(e)'    '79.12945029'
0058 'EX_met_L(e)'    '26.31822121'
0059 'EX_phe_L(e)'    '44.76969122'
0060 'EX_pro_L(e)'    '106.9964084'
0061 'EX_ser_L(e)'    '77.42338661'
0062 'EX_thr_L(e)'    '56.78264293'
0063 'EX_trp_L(e)'    '9.950912032'
0064 'EX_tyr_L(e)'    '35.59289911'
0065 'EX_val_L(e)'    '80.96521188'
0066 'EX_dca(e)'    '16.85243847'
0067 'EX_ddca(e)'    '20.46177358'
0068 'EX_ttdca(e)'    '57.29967937'
0069 'EX_ttdcea(e)'    '7.459891873'
0070 'EX_ptdca(e)'    '5.58183534'
0071 'EX_hdca(e)'    '183.1090366'
0072 'EX_hpdca(e)'    '4.168890083'
0073 'EX_ocdca(e)'    '71.37693218'
0074 'EX_ocdcea(e)'    '219.9278353'
0075 'EX_lnlc(e)'    '82.60523373'
0076 'EX_lnlnca(e)'    '7.908537796'
0077 'EX_strdnc(e)'    '0.085691732'
0078 'EX_arach(e)'    '2.056875507'
0079 'EX_CE2510(e)'    '3.910866521'
0080 'EX_CE4843(e)'    '0.308903651'
0081 'EX_arachd(e)'    '1.033968686'
0082 'EX_docosac(e)'    '0.876841563'
0083 'EX_doco13ac(e)'    '2.215486624'
0084 'EX_adrn(e)'    '0'
0085 'EX_clpnd(e)'    '0.74689784'
0086 'EX_crvnc(e)'    '1.41810057'
0087 'EX_but(e)'    '44.40320699'
0088 'EX_octa(e)'    '10.2894622'
0089 'EX_chsterol(e)'    '2.763523413'
0090 'EX_hdcea(e)'    '21.78982424'
0091 'EX_lgnc(e)'    '0.354020142'
0092 'EX_fol(e)'    '0.000136861'
0093 'EX_strch1(e)'    '40.11699515'
0094 'EX_i(e)'    '0.002644572'
0095 'EX_starch1200(e)'    '0.371419202'
0096 
0097     };
0098 Diet = regexprep(Diet,'EX_','Diet_EX_');
0099 Diet = regexprep(Diet,'\(e\)','\[d\]');
```

---

Generated on Thu 14-May-2020 13:05:49 by **m2html** © 2005
