# Supplementary material for: Personalized whole‐body models integrate metabolism, physiology, and the gut microbiome
Source: Mol Syst Biol. 2020 May 28;16(5):e8982. doi: 10.15252/msb.20198982 (PMC7285886; doi:10.15252/msb.20198982)
Supplement: Supplementary file 22 — Dataset EV1 [file MSB-16-e8982-s022.zip › PSCM_toolbox/PSCM_toolbox_doc/src/setConstraints/diets/HighFatLowCarbDiet.html]

Description of HighFatLowCarbDiet


# HighFatLowCarbDiet

## PURPOSE

**High Fat, Low Carb Diet defintion. For details, please see https://www.vmh.life/#nutrition**

## SYNOPSIS

**This is a script file.**

## DESCRIPTION

```
 High Fat, Low Carb Diet defintion. For details, please see https://www.vmh.life/#nutrition
 Ines Thiele 2016-2019
```

## CROSS-REFERENCE INFORMATION

This function calls:


This function is called by:

## SOURCE CODE

```
0001 % High Fat, Low Carb Diet defintion. For details, please see https://www.vmh.life/#nutrition
0002 % Ines Thiele 2016-2019
0003 
0004 Diet = {%'Reaction'    'Flux Value'
0005 'EX_etoh(e)'    '0'
0006 'EX_h2o(e)'    '158601.9201'
0007 'EX_caro(e)'    '0.001620496'
0008 'EX_retinol(e)'    '3.106982122'
0009 'EX_thm(e)'    '1.808900241'
0010 'EX_adpcbl(e)'    '9.06569E-06'
0011 'EX_ribflv(e)'    '0.004623185'
0012 'EX_nac(e)'    '0.318014111'
0013 'EX_ncam(e)'    '0.181535907'
0014 'EX_pnto_R(e)'    '0.022453684'
0015 'EX_pydam(e)'    '0.003309673'
0016 'EX_pydxn(e)'    '0.003310127'
0017 'EX_pydx(e)'    '0.003350044'
0018 'EX_btn(e)'    '0.000173488'
0019 'EX_10fthf(e)'    '0.000111466'
0020 'EX_5mthf(e)'    '0.00011462'
0021 'EX_thf(e)'    '0.000118507'
0022 'EX_ascb_L(e)'    '0.281984191'
0023 'EX_vitd3(e)'    '6.63222E-05'
0024 'EX_avite1(e)'    '0.05574567'
0025 'EX_phyQ(e)'    '0.000219439'
0026 'EX_ca2(e)'    '40.26049204'
0027 'EX_cl(e)'    '734.5753533'
0028 'EX_k(e)'    '56.59299765'
0029 'EX_mg2(e)'    '15.62929438'
0030 'EX_na1(e)'    '361.0149323'
0031 'EX_pi(e)'    '18.95283671'
0032 'EX_cu2(e)'    '0.019875366'
0033 'EX_fe2(e)'    '0.220189811'
0034 'EX_fe3(e)'    '0.220189811'
0035 'EX_mn2(e)'    '0.018129513'
0036 'EX_zn2(e)'    '0.226215968'
0037 'EX_mnl(e)'    '0.149309608'
0038 'EX_sbt_D(e)'    '0'
0039 'EX_xylt(e)'    '0.118307571'
0040 'EX_lcts(e)'    '0.063103187'
0041 'EX_malt(e)'    '0'
0042 'EX_sucr(e)'    '3.38887485'
0043 'EX_fru(e)'    '9.193259838'
0044 'EX_gal(e)'    '0'
0045 'EX_glc_D(e)'    '34.76738758'
0046 'EX_cellul(e)'    '0.030160202'
0047 'EX_ala_L(e)'    '84.47041974'
0048 'EX_arg_L(e)'    '44.96997584'
0049 'EX_asp_L(e)'    '99.83890345'
0050 'EX_cys_L(e)'    '12.42945174'
0051 'EX_glu_L(e)'    '171.793024'
0052 'EX_gly(e)'    '79.21419113'
0053 'EX_urate(e)'    '5.034849144'
0054 'EX_his_L(e)'    '25.7788683'
0055 'EX_ile_L(e)'    '56.42392598'
0056 'EX_leu_L(e)'    '92.15729773'
0057 'EX_lys_L(e)'    '79.59591156'
0058 'EX_met_L(e)'    '26.03656694'
0059 'EX_phe_L(e)'    '37.49127515'
0060 'EX_pro_L(e)'    '80.77894216'
0061 'EX_ser_L(e)'    '69.57987527'
0062 'EX_thr_L(e)'    '52.2778024'
0063 'EX_trp_L(e)'    '8.196441967'
0064 'EX_tyr_L(e)'    '28.11740259'
0065 'EX_val_L(e)'    '72.01490785'
0066 'EX_dca(e)'    '10.33699703'
0067 'EX_ddca(e)'    '10.69611228'
0068 'EX_ttdca(e)'    '34.08237048'
0069 'EX_ttdcea(e)'    '4.15261612'
0070 'EX_ptdca(e)'    '3.382498666'
0071 'EX_hdca(e)'    '131.5679787'
0072 'EX_hpdca(e)'    '2.080924813'
0073 'EX_ocdca(e)'    '38.17326956'
0074 'EX_ocdcea(e)'    '251.1268651'
0075 'EX_lnlc(e)'    '64.55826437'
0076 'EX_lnlnca(e)'    '5.190653795'
0077 'EX_strdnc(e)'    '2.657896094'
0078 'EX_arach(e)'    '1.399192675'
0079 'EX_CE2510(e)'    '6.299445634'
0080 'EX_CE4843(e)'    '0.296789464'
0081 'EX_arachd(e)'    '0.854810981'
0082 'EX_docosac(e)'    '0.497621148'
0083 'EX_doco13ac(e)'    '6.872206605'
0084 'EX_adrn(e)'    '0.036197774'
0085 'EX_clpnd(e)'    '0.422827335'
0086 'EX_crvnc(e)'    '1.687979399'
0087 'EX_but(e)'    '26.80912819'
0088 'EX_octa(e)'    '5.751395741'
0089 'EX_chsterol(e)'    '2.277749975'
0090 'EX_hdcea(e)'    '17.34283661'
0091 'EX_lgnc(e)'    '0.205669414'
0092 'EX_fol(e)'    '0.000119321'
0093 'EX_strch1(e)'    '0.068690396'
0094 'EX_i(e)'    '0.007685781'
0095 'EX_starch1200(e)'    '0.000635963'
0096 
0097     };
0098 
0099 Diet = regexprep(Diet,'EX_','Diet_EX_');
0100 Diet = regexprep(Diet,'\(e\)','\[d\]');
```

---

Generated on Thu 14-May-2020 13:05:49 by **m2html** © 2005
