# Supplementary material for: Personalized whole‐body models integrate metabolism, physiology, and the gut microbiome
Source: Mol Syst Biol. 2020 May 28;16(5):e8982. doi: 10.15252/msb.20198982 (PMC7285886; doi:10.15252/msb.20198982)
Supplement: Supplementary file 22 — Dataset EV1 [file MSB-16-e8982-s022.zip › PSCM_toolbox/PSCM_toolbox_doc/src/setConstraints/diets/EUAverageDietNew.html]

Description of EUAverageDietNew


# EUAverageDietNew

## PURPOSE

**Average European Diet defintion. For details, please see https://www.vmh.life/#nutrition**

## SYNOPSIS

**This is a script file.**

## DESCRIPTION

```
 Average European Diet defintion. For details, please see https://www.vmh.life/#nutrition
 Ines Thiele 2016-2019
```

## CROSS-REFERENCE INFORMATION

This function calls:


This function is called by:

- analyzeHMmodel This function performs host-microbiome optimization for a set of defined
- perform\_BMR\_newData This script repeats the simulation described in Thiele et al., "Personalized whole-body models integrate metabolism, physiology, and the gut microbiome", Method section 3.9.2 Validation of the parameters in an independent data set.
- perform\_sensi\_BMR\_all This script repeats the simulation described in Thiele et al.,
- runIEM\_HH This script predicts known biomarker metabolites in
- setDietConstraints This function sets diet constraints onto the bounds of the diet uptale

## SOURCE CODE

```
0001 % Average European Diet defintion. For details, please see https://www.vmh.life/#nutrition
0002 % Ines Thiele 2016-2019
0003 
0004 Diet = {%'Exchange reactions'    'Total in mmol/(human*day)'
0005 %'reaction'    'fluxValue'
0006 'EX_10fthf(e)'    '0.000125752'
0007 'EX_5mthf(e)'    '0.000129311'
0008 'EX_adpcbl(e)'    '3.61E-06'
0009 'EX_ala_L(e)'    '37.20856362'
0010 'EX_arach(e)'    '1.033055397'
0011 'EX_arachd(e)'    '0.536810751'
0012 'EX_arg_L(e)'    '20.3518771'
0013 'EX_ascb_L(e)'    '0.583441166'
0014 'EX_asp_L(e)'    '47.08674913'
0015 'EX_avite1(e)'    '0.03685808'
0016 'EX_btn(e)'    '0.000178666'
0017 'EX_but(e)'    '26.33425644'
0018 'EX_ca2(e)'    '18.5490793'
0019 'EX_caro(e)'    '0.003585581'
0020 'EX_CE2510(e)'    '1.086665034'
0021 'EX_CE4843(e)'    '0.114865263'
0022 'EX_cellul(e)'    '0.052346876'
0023 'EX_chsterol(e)'    '1.23218851'
0024 'EX_cl(e)'    '187.7787493'
0025 'EX_clpnd(e)'    '0.048376886'
0026 'EX_crvnc(e)'    '0.16630008'
0027 'EX_cu2(e)'    '0.035674944'
0028 'EX_cys_L(e)'    '7.511831638'
0029 'EX_dca(e)'    '10.74842619'
0030 'EX_ddca(e)'    '13.45904717'
0031 'EX_doco13ac(e)'    '0.560730443'
0032 'EX_docosac(e)'    '0.454096231'
0033 'EX_etoh(e)'    '234.4340155'
0034 'EX_fe2(e)'    '0.109248814'
0035 'EX_fe3(e)'    '0.109248814'
0036 'EX_fol(e)'    '0.000134614'
0037 'EX_fru(e)'    '106.5058652'
0038 'EX_gal(e)'    '0.029974039'
0039 'EX_glc_D(e)'    '135.5803501'
0040 'EX_glu_L(e)'    '103.1222005'
0041 'EX_gly(e)'    '36.76468629'
0042     
0043 'EX_h2o(e)'    '165892.3425'
0044 'EX_hdca(e)'    '92.78506719'
0045 'EX_hdcea(e)'    '7.120473022'
0046 'EX_his_L(e)'    '14.02407663'
0047 'EX_hpdca(e)'    '1.779524923'
0048 'EX_i(e)'    '0.00201238'
0049 'EX_ile_L(e)'    '28.92167513'
0050 'EX_k(e)'    '83.43380658'
0051 'EX_lcts(e)'    '4.126714705'
0052 'EX_leu_L(e)'    '46.88780991'
0053 'EX_lgnc(e)'    '0.222615856'
0054 'EX_lnlc(e)'    '54.98546187'
0055 'EX_lnlnca(e)'    '4.163336898'
0056 'EX_lys_L(e)'    '33.31154825'
0057 'EX_malt(e)'    '8.413758248'
0058 'EX_met_L(e)'    '10.72740469'
0059 'EX_mg2(e)'    '14.52993211'
0060 'EX_mn2(e)'    '0.074702331'
0061 'EX_mnl(e)'    '0.170169038'
0062 'EX_na1(e)'    '172.4462717'
0063 'EX_nac(e)'    '0.308616192'
0064 'EX_ncam(e)'    '0.1455276'
0065 'EX_ocdca(e)'    '35.85086639'
0066 'EX_ocdcea(e)'    '101.5927326'
0067 'EX_octa(e)'    '6.845572908'
0068 'EX_phe_L(e)'    '20.71498664'
0069 'EX_phyQ(e)'    '0.000136877'
0070 'EX_pi(e)'    '14.22660928'
0071 'EX_pnto_R(e)'    '0.025569705'
0072 'EX_pro_L(e)'    '53.81527918'
0073 'EX_ptdca(e)'    '2.89407663'
0074 'EX_pydam(e)'    '0.00317177'
0075 'EX_pydx(e)'    '0.003210459'
0076 'EX_pydxn(e)'    '0.003172205'
0077 'EX_retinol(e)'    '3.002252387'
0078 'EX_ribflv(e)'    '0.003693234'
0079 'EX_sbt_D(e)'    '0.032935943'
0080 'EX_ser_L(e)'    '37.37922556'
0081 'EX_strch1(e)'    '33.03699973'
0082 'EX_strdnc(e)'    '0'
0083 'EX_sucr(e)'    '202.3830217'
0084 'EX_thf(e)'    '0.000133696'
0085 'EX_thm(e)'    '5.407858013'
0086 'EX_thr_L(e)'    '25.88105024'
0087 'EX_trp_L(e)'    '4.554824772'
0088 'EX_ttdca(e)'    '30.21138004'
0089 'EX_ttdcea(e)'    '3.783984795'
0090 'EX_tyr_L(e)'    '15.64685397'
0091 'EX_urate(e)'    '2.919392803'
0092 'EX_val_L(e)'    '37.78437731'
0093 'EX_vitd3(e)'    '8.76E-06'
0094 'EX_xylt(e)'    '0.141969085'
0095 'EX_zn2(e)'    '0.150963597'
0096 'EX_adrn(e)'    '0'
0097 
0098 % add bile acids
0099 'EX_adrn(e)'    '0'
0100 
0101     };
0102 Diet = regexprep(Diet,'EX_','Diet_EX_');
0103 Diet = regexprep(Diet,'\(e\)','\[d\]');
```

---

Generated on Thu 14-May-2020 13:05:49 by **m2html** © 2005
