# Supplementary material for: Personalized whole‐body models integrate metabolism, physiology, and the gut microbiome
Source: Mol Syst Biol. 2020 May 28;16(5):e8982. doi: 10.15252/msb.20198982 (PMC7285886; doi:10.15252/msb.20198982)
Supplement: Supplementary file 22 — Dataset EV1 [file MSB-16-e8982-s022.zip › PSCM_toolbox/PSCM_toolbox_doc/src/setConstraints/diets/VeganDiet.html]

Description of VeganDiet


# VeganDiet

## PURPOSE

**Vegan diet defintion. For details, please see https://www.vmh.life/#nutrition**

## SYNOPSIS

**This is a script file.**

## DESCRIPTION

```
 Vegan diet defintion. For details, please see https://www.vmh.life/#nutrition
 Ines Thiele 2016-2019
```

## CROSS-REFERENCE INFORMATION

This function calls:


This function is called by:

## SOURCE CODE

```
0001 % Vegan diet defintion. For details, please see https://www.vmh.life/#nutrition
0002 % Ines Thiele 2016-2019
0003 
0004 Diet = {%'Reaction'    'Flux Value'
0005 'EX_etoh(e)'    '0'
0006 'EX_h2o(e)'    '204712.0555'
0007 'EX_caro(e)'    '0.015022186'
0008 'EX_retinol(e)'    '1.256756813'
0009 'EX_thm(e)'    '10.36349097'
0010 'EX_adpcbl(e)'    '4.81E-07'
0011 'EX_ribflv(e)'    '0.005314006'
0012 'EX_nac(e)'    '0.264656863'
0013 'EX_ncam(e)'    '0.117298235'
0014 'EX_pnto_R(e)'    '0.035032329'
0015 'EX_pydam(e)'    '0.005851029'
0016 'EX_pydxn(e)'    '0.005851832'
0017 'EX_pydx(e)'    '0.005922399'
0018 'EX_btn(e)'    '0.000477944'
0019 'EX_10fthf(e)'    '0.000278028'
0020 'EX_5mthf(e)'    '0.000285897'
0021 'EX_thf(e)'    '0.00029559'
0022 'EX_ascb_L(e)'    '1.599252382'
0023 'EX_vitd3(e)'    '2.20987E-06'
0024 'EX_avite1(e)'    '0.051694183'
0025 'EX_phyQ(e)'    '0.000843219'
0026 'EX_ca2(e)'    '21.05120016'
0027 'EX_cl(e)'    '219.1563478'
0028 'EX_k(e)'    '107.0774177'
0029 'EX_mg2(e)'    '26.06418432'
0030 'EX_na1(e)'    '113.966781'
0031 'EX_pi(e)'    '11.35468794'
0032 'EX_cu2(e)'    '0.043669153'
0033 'EX_fe2(e)'    '0.162727191'
0034 'EX_fe3(e)'    '0.162727191'
0035 'EX_mn2(e)'    '0.208871648'
0036 'EX_zn2(e)'    '0.116396452'
0037 'EX_mnl(e)'    '5.357580043'
0038 'EX_xylt(e)'    '0.254361277'
0039 'EX_lcts(e)'    '0.102542679'
0040 'EX_malt(e)'    '0.555074329'
0041 'EX_sucr(e)'    '216.9464193'
0042 'EX_fru(e)'    '143.7152488'
0043 'EX_gal(e)'    '0.013321795'
0044 'EX_cellul(e)'    '0.132648766'
0045 'EX_ala_L(e)'    '29.5717855'
0046 'EX_arg_L(e)'    '23.8655114'
0047 'EX_asp_L(e)'    '46.52393321'
0048 'EX_cys_L(e)'    '7.484016765'
0049 'EX_glu_L(e)'    '85.21447592'
0050 'EX_gly(e)'    '36.45129791'
0051 'EX_urate(e)'    '2.792482079'
0052 'EX_his_L(e)'    '9.224122263'
0053 'EX_ile_L(e)'    '19.6492187'
0054 'EX_leu_L(e)'    '32.41164905'
0055 'EX_lys_L(e)'    '19.66510525'
0056 'EX_met_L(e)'    '4.98705527'
0057 'EX_phe_L(e)'    '17.16451025'
0058 'EX_pro_L(e)'    '33.5136215'
0059 'EX_ser_L(e)'    '30.30946042'
0060 'EX_thr_L(e)'    '17.59749898'
0061 'EX_trp_L(e)'    '3.103167484'
0062 'EX_tyr_L(e)'    '10.36660715'
0063 'EX_val_L(e)'    '26.41150425'
0064 'EX_dca(e)'    '2.280377936'
0065 'EX_ddca(e)'    '15.14335973'
0066 'EX_ttdca(e)'    '5.913649099'
0067 'EX_ttdcea(e)'    '0.029687535'
0068 'EX_ptdca(e)'    '0.034549956'
0069 'EX_hdca(e)'    '32.86754437'
0070 'EX_hpdca(e)'    '0.13373901'
0071 'EX_ocdca(e)'    '7.757541998'
0072 'EX_ocdcea(e)'    '123.5422809'
0073 'EX_lnlc(e)'    '69.80093939'
0074 'EX_lnlnca(e)'    '6.235993796'
0075 'EX_strdnc(e)'    '0'
0076 'EX_arach(e)'    '1.253488913'
0077 'EX_CE2510(e)'    '1.137714026'
0078 'EX_CE4843(e)'    '0.003642387'
0079 'EX_arachd(e)'    '0.006261144'
0080 'EX_docosac(e)'    '1.260882702'
0081 'EX_doco13ac(e)'    '0.261835166'
0082 'EX_adrn(e)'    '0'
0083 'EX_clpnd(e)'    '0'
0084 'EX_crvnc(e)'    '0'
0085 'EX_but(e)'    '0.177388022'
0086 'EX_octa(e)'    '3.674770519'
0087 'EX_chsterol(e)'    '0.003672539'
0088 'EX_sbt_D(e)'    '3.933100513'
0089 'EX_glc_D(e)'    '112.0613036'
0090 'EX_hdcea(e)'    '2.074287205'
0091 'EX_lgnc(e)'    '0.441749939'
0092 'EX_fol(e)'    '0.00029762'
0093 'EX_strch1(e)'    '30.86817888'
0094 'EX_i(e)'    '0.001422803'
0095 'EX_starch1200(e)'    '0.285789958'
0096 
0097     };
0098 Diet = regexprep(Diet,'EX_','Diet_EX_');
0099 Diet = regexprep(Diet,'\(e\)','\[d\]');
```

---

Generated on Thu 14-May-2020 13:05:49 by **m2html** © 2005
