# Supplementary material for: Personalized whole‐body models integrate metabolism, physiology, and the gut microbiome
Source: Mol Syst Biol. 2020 May 28;16(5):e8982. doi: 10.15252/msb.20198982 (PMC7285886; doi:10.15252/msb.20198982)
Supplement: Supplementary file 22 — Dataset EV1 [file MSB-16-e8982-s022.zip › PSCM_toolbox/PSCM_toolbox_doc/src/setConstraints/diets/HighFiberDiet.html]

Description of HighFiberDiet


# HighFiberDiet

## PURPOSE

**High fiber diet defintion. For details, please see https://www.vmh.life/#nutrition**

## SYNOPSIS

**This is a script file.**

## DESCRIPTION

```
 High fiber diet defintion. For details, please see https://www.vmh.life/#nutrition
 Ines Thiele 2016-2019
```

## CROSS-REFERENCE INFORMATION

This function calls:


This function is called by:

- analyzeHMmodel This function performs host-microbiome optimization for a set of defined
- setDietConstraints This function sets diet constraints onto the bounds of the diet uptale

## SOURCE CODE

```
0001 % High fiber diet defintion. For details, please see https://www.vmh.life/#nutrition
0002 % Ines Thiele 2016-2019
0003 
0004 Diet = {%'Reaction'    'Flux Value'
0005 'EX_etoh(e)'    '22.79219595'
0006 'EX_h2o(e)'    '181671.3368'
0007 'EX_caro(e)'    '0.013131607'
0008 'EX_retinol(e)'    '1.099662212'
0009 'EX_thm(e)'    '7.235600966'
0010 'EX_adpcbl(e)'    '2.56714E-06'
0011 'EX_ribflv(e)'    '0.006894923'
0012 'EX_nac(e)'    '0.479928584'
0013 'EX_ncam(e)'    '0.216377372'
0014 'EX_pnto_R(e)'    '0.054851141'
0015 'EX_pydam(e)'    '0.00499406'
0016 'EX_pydxn(e)'    '0.004994745'
0017 'EX_pydx(e)'    '0.005054977'
0018 'EX_btn(e)'    '0.000479218'
0019 'EX_10fthf(e)'    '0.000254697'
0020 'EX_5mthf(e)'    '0.000261905'
0021 'EX_thf(e)'    '0.000270786'
0022 'EX_ascb_L(e)'    '1.513851945'
0023 'EX_vitd3(e)'    '1.53391E-05'
0024 'EX_avite1(e)'    '0.068538616'
0025 'EX_phyQ(e)'    '0.000936308'
0026 'EX_ca2(e)'    '33.02011078'
0027 'EX_cl(e)'    '231.4450117'
0028 'EX_k(e)'    '116.4477739'
0029 'EX_mg2(e)'    '31.15531784'
0030 'EX_na1(e)'    '147.9118802'
0031 'EX_pi(e)'    '22.33606621'
0032 'EX_cu2(e)'    '0.04732792'
0033 'EX_fe2(e)'    '0.424456084'
0034 'EX_fe3(e)'    '0.424456084'
0035 'EX_mn2(e)'    '0.174378247'
0036 'EX_zn2(e)'    '0.244799633'
0037 'EX_mnl(e)'    '2.691195893'
0038 'EX_xylt(e)'    '0.276050998'
0039 'EX_lcts(e)'    '26.57468014'
0040 'EX_malt(e)'    '0.803397055'
0041 'EX_sucr(e)'    '137.1179664'
0042 'EX_fru(e)'    '157.3272926'
0043 'EX_gal(e)'    '1.010236134'
0044 'EX_cellul(e)'    '0.237688253'
0045 'EX_ala_L(e)'    '59.06129761'
0046 'EX_arg_L(e)'    '40.67170675'
0047 'EX_asp_L(e)'    '80.1605212'
0048 'EX_cys_L(e)'    '13.82679835'
0049 'EX_glu_L(e)'    '130.8154595'
0050 'EX_gly(e)'    '60.78249181'
0051 'EX_urate(e)'    '6.319184488'
0052 'EX_his_L(e)'    '17.21966348'
0053 'EX_ile_L(e)'    '41.93225125'
0054 'EX_leu_L(e)'    '63.31791856'
0055 'EX_lys_L(e)'    '46.34676332'
0056 'EX_met_L(e)'    '12.66995194'
0057 'EX_phe_L(e)'    '29.69793406'
0058 'EX_pro_L(e)'    '50.90466905'
0059 'EX_ser_L(e)'    '52.11494434'
0060 'EX_thr_L(e)'    '38.25894566'
0061 'EX_trp_L(e)'    '6.192991854'
0062 'EX_tyr_L(e)'    '21.2683752'
0063 'EX_val_L(e)'    '50.97864807'
0064 'EX_dca(e)'    '0.043443556'
0065 'EX_ddca(e)'    '0.101851489'
0066 'EX_ttdca(e)'    '0.787507202'
0067 'EX_ttdcea(e)'    '0.031285071'
0068 'EX_ptdca(e)'    '0.072103355'
0069 'EX_hdca(e)'    '34.44714331'
0070 'EX_hpdca(e)'    '0.202603374'
0071 'EX_ocdca(e)'    '9.424142226'
0072 'EX_ocdcea(e)'    '130.2124615'
0073 'EX_lnlc(e)'    '66.47282487'
0074 'EX_lnlnca(e)'    '22.76317966'
0075 'EX_strdnc(e)'    '0'
0076 'EX_arach(e)'    '0.667592228'
0077 'EX_CE2510(e)'    '1.006715204'
0078 'EX_CE4843(e)'    '0.038033671'
0079 'EX_arachd(e)'    '0.35958741'
0080 'EX_docosac(e)'    '0.394315728'
0081 'EX_doco13ac(e)'    '0.049887472'
0082 'EX_adrn(e)'    '0'
0083 'EX_clpnd(e)'    '0.050167498'
0084 'EX_crvnc(e)'    '0.384328462'
0085 'EX_but(e)'    '0.07233298'
0086 'EX_octa(e)'    '0.015502414'
0087 'EX_chsterol(e)'    '1.053035858'
0088 'EX_sbt_D(e)'    '6.5699521'
0089 'EX_glc_D(e)'    '120.210995'
0090 'EX_hdcea(e)'    '3.616984668'
0091 'EX_lgnc(e)'    '0.166975493'
0092 'EX_fol(e)'    '0.000272645'
0093 'EX_strch1(e)'    '45.11396195'
0094 'EX_i(e)'    '0.003660628'
0095 'EX_starch1200(e)'    '0.417683121'
0096 
0097 
0098 };
0099 
0100 Diet = regexprep(Diet,'EX_','Diet_EX_');
0101 Diet = regexprep(Diet,'\(e\)','\[d\]');
```

---

Generated on Thu 14-May-2020 13:05:49 by **m2html** © 2005
