# Supplementary material for: Personalized whole‐body models integrate metabolism, physiology, and the gut microbiome
Source: Mol Syst Biol. 2020 May 28;16(5):e8982. doi: 10.15252/msb.20198982 (PMC7285886; doi:10.15252/msb.20198982)
Supplement: Supplementary file 22 — Dataset EV1 [file MSB-16-e8982-s022.zip › PSCM_toolbox/PSCM_toolbox_doc/src/setConstraints/diets/HighProteinDiet.html]

Description of HighProteinDiet


# HighProteinDiet

## PURPOSE

**High protein diet defintion. For details, please see https://www.vmh.life/#nutrition**

## SYNOPSIS

**This is a script file.**

## DESCRIPTION

```
 High protein diet defintion. For details, please see https://www.vmh.life/#nutrition
 Ines Thiele 2016-2019
```

## CROSS-REFERENCE INFORMATION

This function calls:


This function is called by:

- analyzeHMmodel This function performs host-microbiome optimization for a set of defined
- setDietConstraints This function sets diet constraints onto the bounds of the diet uptale

## SOURCE CODE

```
0001 % High protein diet defintion. For details, please see https://www.vmh.life/#nutrition
0002 % Ines Thiele 2016-2019
0003 
0004 % HighProteinDiet
0005 Diet = {%'Reaction'    'Flux Value'
0006 'EX_etoh(e)'    '0'
0007 'EX_h2o(e)'    '179031.7997'
0008 'EX_caro(e)'    '0.009518087'
0009 'EX_retinol(e)'    '2.077139733'
0010 'EX_thm(e)'    '6.764533194'
0011 'EX_adpcbl(e)'    '3.55474E-06'
0012 'EX_ribflv(e)'    '0.005274151'
0013 'EX_nac(e)'    '0.506422935'
0014 'EX_ncam(e)'    '0.24511851'
0015 'EX_pnto_R(e)'    '0.041172724'
0016 'EX_pydam(e)'    '0.005407769'
0017 'EX_pydxn(e)'    '0.005408511'
0018 'EX_pydx(e)'    '0.005473732'
0019 'EX_btn(e)'    '0.000493685'
0020 'EX_10fthf(e)'    '0.000251926'
0021 'EX_5mthf(e)'    '0.000259056'
0022 'EX_thf(e)'    '0.00026784'
0023 'EX_ascb_L(e)'    '1.122397585'
0024 'EX_vitd3(e)'    '1.46112E-05'
0025 'EX_avite1(e)'    '0.039586159'
0026 'EX_phyQ(e)'    '0.000512863'
0027 'EX_ca2(e)'    '25.41943211'
0028 'EX_cl(e)'    '482.1654021'
0029 'EX_k(e)'    '98.62768969'
0030 'EX_mg2(e)'    '25.57663032'
0031 'EX_na1(e)'    '249.2580474'
0032 'EX_pi(e)'    '18.0555599'
0033 'EX_cu2(e)'    '0.038601958'
0034 'EX_fe2(e)'    '0.329062584'
0035 'EX_fe3(e)'    '0.329062584'
0036 'EX_mn2(e)'    '0.108959101'
0037 'EX_zn2(e)'    '0.16862955'
0038 'EX_mnl(e)'    '0.158092526'
0039 'EX_xylt(e)'    '0.078871714'
0040 'EX_lcts(e)'    '17.11513264'
0041 'EX_malt(e)'    '8.355329371'
0042 'EX_sucr(e)'    '82.69146778'
0043 'EX_fru(e)'    '65.80686505'
0044 'EX_gal(e)'    '0.291858329'
0045 'EX_cellul(e)'    '0.09665326'
0046 'EX_ala_L(e)'    '83.24782363'
0047 'EX_arg_L(e)'    '42.99858626'
0048 'EX_asp_L(e)'    '96.14821791'
0049 'EX_cys_L(e)'    '17.48486689'
0050 'EX_glu_L(e)'    '162.2197448'
0051 'EX_gly(e)'    '75.17877458'
0052 'EX_urate(e)'    '4.551862676'
0053 'EX_his_L(e)'    '25.53968751'
0054 'EX_ile_L(e)'    '54.39774527'
0055 'EX_leu_L(e)'    '81.99799654'
0056 'EX_lys_L(e)'    '62.62953011'
0057 'EX_met_L(e)'    '23.6294101'
0058 'EX_phe_L(e)'    '38.256368'
0059 'EX_pro_L(e)'    '63.86778482'
0060 'EX_ser_L(e)'    '69.71237747'
0061 'EX_thr_L(e)'    '48.65567432'
0062 'EX_trp_L(e)'    '8.220067847'
0063 'EX_tyr_L(e)'    '27.95329174'
0064 'EX_val_L(e)'    '68.63750712'
0065 'EX_dca(e)'    '1.155224876'
0066 'EX_ddca(e)'    '1.20606212'
0067 'EX_ttdca(e)'    '4.25546373'
0068 'EX_ttdcea(e)'    '0.48811367'
0069 'EX_ptdca(e)'    '0.364452321'
0070 'EX_hdca(e)'    '37.66248486'
0071 'EX_hpdca(e)'    '0.4103841'
0072 'EX_ocdca(e)'    '12.33521584'
0073 'EX_ocdcea(e)'    '99.50130288'
0074 'EX_lnlc(e)'    '29.27309327'
0075 'EX_lnlnca(e)'    '1.658125518'
0076 'EX_strdnc(e)'    '0.081334525'
0077 'EX_arach(e)'    '0.672744344'
0078 'EX_CE2510(e)'    '0.809271919'
0079 'EX_CE4843(e)'    '0.061725444'
0080 'EX_arachd(e)'    '0.589371411'
0081 'EX_docosac(e)'    '0.187248436'
0082 'EX_doco13ac(e)'    '0.276691797'
0083 'EX_adrn(e)'    '0.026545034'
0084 'EX_clpnd(e)'    '0.279517639'
0085 'EX_crvnc(e)'    '3.231492093'
0086 'EX_but(e)'    '2.932126406'
0087 'EX_octa(e)'    '0.642267822'
0088 'EX_chsterol(e)'    '1.595356049'
0089 'EX_sbt_D(e)'    '2.059594295'
0090 'EX_glc_D(e)'    '107.3525763'
0091 'EX_hdcea(e)'    '3.967674861'
0092 'EX_lgnc(e)'    '0.068098582'
0093 'EX_fol(e)'    '0.000269679'
0094 'EX_strch1(e)'    '27.08644094'
0095 'EX_i(e)'    '0.003562207'
0096 'EX_starch1200(e)'    '0.250777115'
0097 
0098     };
0099 Diet = regexprep(Diet,'EX_','Diet_EX_');
0100 Diet = regexprep(Diet,'\(e\)','\[d\]');
```

---

Generated on Thu 14-May-2020 13:05:49 by **m2html** © 2005
