# Supplementary material for: Personalized whole‐body models integrate metabolism, physiology, and the gut microbiome
Source: Mol Syst Biol. 2020 May 28;16(5):e8982. doi: 10.15252/msb.20198982 (PMC7285886; doi:10.15252/msb.20198982)
Supplement: Supplementary file 22 — Dataset EV1 [file MSB-16-e8982-s022.zip › PSCM_toolbox/ext/m2html/doc/m2html/@template/index.html]

Index for Directory m2html/@template


|  |  |
| --- | --- |
| Master index | Index for m2html/@template |

# Index for m2html/@template

## Matlab files in this directory:

|  |  |
| --- | --- |
| char | TEMPLATE Convert a template object in a one line description string |
| display | TEMPLATE Display a template object in Matlab window |
| get | TEMPLATE/GET Access data stored in a Template object |
| parse | TEMPLATE/PARSE Fill in replacement fields with the class properties |
| set | TEMPLATE/SET Edit data stored in a Template object |
| template | TEMPLATE HTML Template Toolbox Constructor |

## Subsequent directories:

- private

---

Generated on Tue 29-Oct-2019 21:04:53 by **m2html** © 2005
