# Supplementary material for: Personalized whole‐body models integrate metabolism, physiology, and the gut microbiome
Source: Mol Syst Biol. 2020 May 28;16(5):e8982. doi: 10.15252/msb.20198982 (PMC7285886; doi:10.15252/msb.20198982)
Supplement: Supplementary file 22 — Dataset EV1 [file MSB-16-e8982-s022.zip › PSCM_toolbox/ext/m2html/doc/m2html/@template/display.html]

Description of display


Home > m2html > @template > display.m

# display

## PURPOSE

**TEMPLATE Display a template object in Matlab window**

## SYNOPSIS

**function display(tpl)**

## DESCRIPTION

```
TEMPLATE Display a template object in Matlab window
  DISPLAY(TPL) displays informations about the content of template 
  object TPL:
     Template Object: root '.', 2 files, 9 keys, comment unknowns.
  root element of template files, number of template files, number of 
  keywords defined and the way of handling unknowns tags.
```

## CROSS-REFERENCE INFORMATION

This function calls:

- char TEMPLATE Convert a template object in a one line description string

This function is called by:


## SOURCE CODE

```
0001 function display(tpl)
0002 %TEMPLATE Display a template object in Matlab window
0003 %  DISPLAY(TPL) displays informations about the content of template
0004 %  object TPL:
0005 %     Template Object: root '.', 2 files, 9 keys, comment unknowns.
0006 %  root element of template files, number of template files, number of
0007 %  keywords defined and the way of handling unknowns tags.
0008 
0009 %  Copyright (C) 2003 Guillaume Flandin <Guillaume@artefact.tk>
0010 %  $Revision: 1.0 $Date: 2003/05/05 22:19:51 $
0011 
0012 disp(' ');
0013 disp([inputname(1),' = ']);
0014 disp(' ');
0015 for i=1:prod(size(tpl))
0016     disp([blanks(length(inputname(1))+3) char(tpl(i))]);
0017 end
0018 disp(' ');
```

---

Generated on Tue 29-Oct-2019 21:04:54 by **m2html** © 2005
