# Supplementary material for: Personalized whole‐body models integrate metabolism, physiology, and the gut microbiome
Source: Mol Syst Biol. 2020 May 28;16(5):e8982. doi: 10.15252/msb.20198982 (PMC7285886; doi:10.15252/msb.20198982)
Supplement: Supplementary file 22 — Dataset EV1 [file MSB-16-e8982-s022.zip › PSCM_toolbox/ext/m2html/doc/m2html/@template/char.html]

Description of char


Home > m2html > @template > char.m

# char

## PURPOSE

**TEMPLATE Convert a template object in a one line description string**

## SYNOPSIS

**function s = char(tpl)**

## DESCRIPTION

```
TEMPLATE Convert a template object in a one line description string
  S = CHAR(TPL) is a class convertor from Template to a string, used
  in online display.
  
  See also DISPLAY
```

## CROSS-REFERENCE INFORMATION

This function calls:


This function is called by:

- display TEMPLATE Display a template object in Matlab window
- get TEMPLATE/GET Access data stored in a Template object

## SOURCE CODE

```
0001 function s = char(tpl)
0002 %TEMPLATE Convert a template object in a one line description string
0003 %  S = CHAR(TPL) is a class convertor from Template to a string, used
0004 %  in online display.
0005 %
0006 %  See also DISPLAY
0007 
0008 %  Copyright (C) 2003 Guillaume Flandin <Guillaume@artefact.tk>
0009 %  $Revision: 1.0 $Date: 2003/05/05 22:19:51 $
0010 
0011 s = ['Template Object: root ''',...
0012         tpl.root,''', ',...
0013         num2str(length(tpl.file)), ' files, ',...
0014         num2str(length(tpl.varkeys)), ' keys, ',...
0015         tpl.unknowns, ' unknowns.'];
```

---

Generated on Tue 29-Oct-2019 21:04:54 by **m2html** © 2005
