# Supplementary material for: Personalized whole‐body models integrate metabolism, physiology, and the gut microbiome
Source: Mol Syst Biol. 2020 May 28;16(5):e8982. doi: 10.15252/msb.20198982 (PMC7285886; doi:10.15252/msb.20198982)
Supplement: Supplementary file 22 — Dataset EV1 [file MSB-16-e8982-s022.zip › PSCM_toolbox/ext/m2html/doc/m2html/@template/template.html]

Description of template


Home > m2html > @template > template.m

# template

## PURPOSE

**TEMPLATE HTML Template Toolbox Constructor**

## SYNOPSIS

**function tpl = template(root,unknowns)**

## DESCRIPTION

```
TEMPLATE HTML Template Toolbox Constructor
  TPL = TEMPLATE returns a template object using default values for the
  root path of the template files ('.') and for the way of handling unknown
  replacement fields (default is 'remove').
  TPL = TEMPLATE(ROOT) allows to specify the root path of the template files
  that will then be provided relative to this path.
  TPL = TEMPLATE(ROOT,UNKNOWNS) also allows to specify the strategy to apply
  to unkown fields. UNKNOWNS may be:
    * 'keep' to do nothing
    * 'remove' to remove all undefined fields
    * 'comment' to replace undefined fields by a warning HTML comment.

  The template class allows you to keep your HTML code in some external 
  files which are completely free of Matlab code, but contain replacement 
  fields. The class provides you with functions which can fill in the 
  replacement fields with arbitrary strings. These strings can become very 
  large, e.g. entire tables.
  See the PHPLib: <http://www.sanisoft.com/phplib/manual/template.php>
  See also GET, SET, PARSE
```

## CROSS-REFERENCE INFORMATION

This function calls:

- set TEMPLATE/SET Edit data stored in a Template object
- template TEMPLATE HTML Template Toolbox Constructor

This function is called by:

- template TEMPLATE HTML Template Toolbox Constructor

## SOURCE CODE

```
0001 function tpl = template(root,unknowns)
0002 %TEMPLATE HTML Template Toolbox Constructor
0003 %  TPL = TEMPLATE returns a template object using default values for the
0004 %  root path of the template files ('.') and for the way of handling unknown
0005 %  replacement fields (default is 'remove').
0006 %  TPL = TEMPLATE(ROOT) allows to specify the root path of the template files
0007 %  that will then be provided relative to this path.
0008 %  TPL = TEMPLATE(ROOT,UNKNOWNS) also allows to specify the strategy to apply
0009 %  to unkown fields. UNKNOWNS may be:
0010 %    * 'keep' to do nothing
0011 %    * 'remove' to remove all undefined fields
0012 %    * 'comment' to replace undefined fields by a warning HTML comment.
0013 %
0014 %  The template class allows you to keep your HTML code in some external
0015 %  files which are completely free of Matlab code, but contain replacement
0016 %  fields. The class provides you with functions which can fill in the
0017 %  replacement fields with arbitrary strings. These strings can become very
0018 %  large, e.g. entire tables.
0019 %  See the PHPLib: <http://www.sanisoft.com/phplib/manual/template.php>
0020 %  See also GET, SET, PARSE
0021 
0022 %  Copyright (C) 2003 Guillaume Flandin <Guillaume@artefact.tk>
0023 %  $Revision: 1.0 $Date: 2003/05/05 22:19:51 $
0024 
0025 error(nargchk(0,2,nargin));
0026 
0027 switch nargin
0028     case 0
0029         tpl = struct('root','.',...
0030                      'file',{{}},...
0031                      'handles',{{}},...
0032                      'varkeys',{{}},...
0033                      'varvals',{{}},...
0034                      'unknowns','remove');
0035         tpl = class(tpl,'template');
0036     case 1
0037         if isa(root,'template')
0038             tpl = root;
0039         else
0040             tpl = template;
0041             tpl = set(tpl,'root',root);
0042         end
0043     case 2
0044         tpl = template;
0045         tpl = set(tpl,'root',root);
0046         tpl = set(tpl,'unknowns',unknowns);
0047 end
```

---

Generated on Tue 29-Oct-2019 21:04:54 by **m2html** © 2005
