# Supplementary material for: Personalized whole‐body models integrate metabolism, physiology, and the gut microbiome
Source: Mol Syst Biol. 2020 May 28;16(5):e8982. doi: 10.15252/msb.20198982 (PMC7285886; doi:10.15252/msb.20198982)
Supplement: Supplementary file 22 — Dataset EV1 [file MSB-16-e8982-s022.zip › PSCM_toolbox/ext/m2html/doc/m2html/@template/parse.html]

Description of parse


Home > m2html > @template > parse.m

# parse

## PURPOSE

**TEMPLATE/PARSE Fill in replacement fields with the class properties**

## SYNOPSIS

**function [tpl, str] = parse(tpl,target,handle,append)**

## DESCRIPTION

```
TEMPLATE/PARSE Fill in replacement fields with the class properties
  [TPL, STR] = PARSE(TPL,TARGET,HANDLE) fills in the replacement field
  HANDLE using previously defined variables of template TPL and store
  it in field TARGET. HANDLE can also be a cell array of field names.
  Output is also provided in output STR (content of TARGET).
  [TPL, STR] = PARSE(TPL,TARGET,HANDLE,APPEND) allows to specify if
  TARGET field is reseted before being filled or if new content is
  appended to the previous one.
```

## CROSS-REFERENCE INFORMATION

This function calls:

- get TEMPLATE/GET Access data stored in a Template object
- set TEMPLATE/SET Edit data stored in a Template object

This function is called by:


## SOURCE CODE

```
0001 function [tpl, str] = parse(tpl,target,handle,append)
0002 %TEMPLATE/PARSE Fill in replacement fields with the class properties
0003 %  [TPL, STR] = PARSE(TPL,TARGET,HANDLE) fills in the replacement field
0004 %  HANDLE using previously defined variables of template TPL and store
0005 %  it in field TARGET. HANDLE can also be a cell array of field names.
0006 %  Output is also provided in output STR (content of TARGET).
0007 %  [TPL, STR] = PARSE(TPL,TARGET,HANDLE,APPEND) allows to specify if
0008 %  TARGET field is reseted before being filled or if new content is
0009 %  appended to the previous one.
0010 
0011 %  Copyright (C) 2003 Guillaume Flandin <Guillaume@artefact.tk>
0012 %  $Revision: 1.0 $Date: 2003/05/05 22:19:51 $
0013 
0014 error(nargchk(3,4,nargin));
0015 if nargin == 3
0016     append = 0;
0017 end
0018 
0019 if iscellstr(handle)
0020     for i=1:length(handle)
0021         [tpl, str] = subst(tpl,handle{i});
0022         tpl = set(tpl,'var',target,str);
0023     end
0024 elseif ischar(handle)
0025     [tpl, str] = subst(tpl,handle);
0026     if append
0027         tpl = set(tpl,'var',target,[get(tpl,'var',target) str]);
0028     else
0029         tpl = set(tpl,'var',target,str);
0030     end
0031 else
0032     error('[Template] Badly formed handle.');
0033 end
```

---

Generated on Tue 29-Oct-2019 21:04:54 by **m2html** © 2005
