# Supplementary material for: Personalized whole‐body models integrate metabolism, physiology, and the gut microbiome
Source: Mol Syst Biol. 2020 May 28;16(5):e8982. doi: 10.15252/msb.20198982 (PMC7285886; doi:10.15252/msb.20198982)
Supplement: Supplementary file 22 — Dataset EV1 [file MSB-16-e8982-s022.zip › PSCM_toolbox/ext/m2html/doc/m2html/@template/set.html]

Description of set


Home > m2html > @template > set.m

# set

## PURPOSE

**TEMPLATE/SET Edit data stored in a Template object**

## SYNOPSIS

**function tpl = set(tpl,action,varargin)**

## DESCRIPTION

```
TEMPLATE/SET Edit data stored in a Template object
  TPL = SET(TPL,ACTION,VARARGIN)
     ACTION 'root'
     ACTION 'unknowns'
     ACTION 'file'
     ACTION 'block'
     ACTION 'var'
```

## CROSS-REFERENCE INFORMATION

This function calls:

- get TEMPLATE/GET Access data stored in a Template object
- set TEMPLATE/SET Edit data stored in a Template object

This function is called by:

- parse TEMPLATE/PARSE Fill in replacement fields with the class properties
- set TEMPLATE/SET Edit data stored in a Template object
- template TEMPLATE HTML Template Toolbox Constructor

## SOURCE CODE

```
0001 function tpl = set(tpl,action,varargin)
0002 %TEMPLATE/SET Edit data stored in a Template object
0003 %  TPL = SET(TPL,ACTION,VARARGIN)
0004 %     ACTION 'root'
0005 %     ACTION 'unknowns'
0006 %     ACTION 'file'
0007 %     ACTION 'block'
0008 %     ACTION 'var'
0009 
0010 %  Copyright (C) 2003 Guillaume Flandin <Guillaume@artefact.tk>
0011 %  $Revision: 1.0 $Date: 2003/05/05 22:19:51 $
0012 
0013 error(nargchk(3,5,nargin));
0014 
0015 switch lower(action)
0016     case 'root'
0017         error(nargchk(3,3,nargin));
0018         if exist(varargin{1},'dir')
0019             tpl.root = varargin{1};
0020         else
0021             error('[Template] No such directory.');
0022         end
0023     case 'unknowns'
0024         error(nargchk(3,3,nargin));
0025         if ismember(varargin{1},{'remove' 'comment' 'keep'})
0026             tpl.unknowns = varargin{1};
0027         else
0028             error('[Template] Unknowns: ''remove'', ''comment'' or ''keep''.');
0029         end
0030     case 'file'
0031         error(nargchk(4,4,nargin));
0032         if iscellstr(varargin{1})
0033             for i=1:length(varargin{1})
0034                 ind = find(ismember(tpl.handles,varargin{1}{i}));
0035                 if isempty(ind)
0036                     tpl.handles{end+1} = varargin{1}{i};
0037                     if strcmp(varargin{2}{i}(1),filesep) %- absolute path (Unix)
0038                         tpl.file{end+1} = varargin{2}{i};
0039                     else %- relative path
0040                         tpl.file{end+1} = fullfile(tpl.root,varargin{2}{i});
0041                     end
0042                 else
0043                     if strcmp(varargin{2}{i}(1),filesep) %- absolute path (Unix)
0044                         tpl.file{ind} = varargin{2}{i};
0045                     else %- relative path
0046                         tpl.file{ind} = fullfile(tpl.root,varargin{2}{i});
0047                     end
0048                 end
0049             end
0050         elseif ischar(varargin{1})
0051             tpl = set(tpl,'file',cellstr(varargin{1}),cellstr(varargin{2}));
0052         else
0053             error('[Template] Badly formed handles.');
0054         end
0055     case 'block'
0056         error(nargchk(4,5,nargin));
0057         tpl = loadtpl(tpl,varargin{1});
0058         if nargin == 4
0059             name = varargin{2};
0060         else
0061             name = varargin{3};
0062         end
0063         str = get(tpl,'var',varargin{1});
0064         blk = '';
0065         %- look like this (keep the same number (1) of spaces between characters!)
0066         %  <!-- BEGIN ??? -->
0067         %  <!-- END ??? -->
0068         
0069         %%%%%%%%%%%%%%%%%%%%%%%%% WIH REGEXP %%%%%%%%%%%%%%%%%%%%%%%%
0070         % reg = ['<!--\s+BEGIN ' varargin{2} '\s+-->(.*)\n\s*<!--\s+END ' varargin{2} '\s+-->'];
0071         % [b, e] = regexp(str,reg,'once');
0072         % if ~isempty(b), blk = str(b:e); end %- should also remove BEGIN and END comments
0073         % str = regexprep(str,reg,['{' name '}']);
0074         %%%%%%%%%%%%%%%%%%%%%%%%%%%%%%%%%%%%%%%%%%%%%%%%%%%%%%%%%%%%%
0075         
0076         %%%%%%%%%%%%%%%%%%%%%% WIHTOUT REGEXP %%%%%%%%%%%%%%%%%%%%%%%
0077         indbegin = findstr(str,['<!-- BEGIN ' varargin{2} ' -->']);
0078         indend   = findstr(str,['<!-- END ' varargin{2} ' -->']);
0079         if ~isempty(indbegin) & ~isempty(indend)
0080            blk = str(indbegin+length(['<!-- BEGIN ' varargin{2} ' -->'])+1:indend-1);
0081            str = [str(1:indbegin-1) '{' name '}' str(indend+length(['<!-- END ' varargin{2} ' -->'])+1:end)];
0082         end
0083         %%%%%%%%%%%%%%%%%%%%%%%%%%%%%%%%%%%%%%%%%%%%%%%%%%%%%%%%%%%%%
0084         
0085         tpl = set(tpl,'var',varargin{2},blk);
0086         tpl = set(tpl,'var',varargin{1},str);
0087     case 'var'
0088         error(nargchk(3,4,nargin));
0089         if iscellstr(varargin{1})
0090             for i=1:length(varargin{1})
0091                 ind = find(ismember(tpl.varkeys,varargin{1}{i}));
0092                 if isempty(ind)
0093                     tpl.varkeys{end+1} = varargin{1}{i};
0094                     if nargin == 4
0095                         tpl.varvals{end+1} = varargin{2}{i};
0096                     else
0097                         tpl.varvals{end+1} = '';
0098                     end
0099                 else
0100                     tpl.varvals{ind} = varargin{2}{i};
0101                 end
0102             end
0103         elseif ischar(varargin{1})
0104             tpl = set(tpl,'var',cellstr(varargin{1}),cellstr(varargin{2}));
0105         else
0106             error('[Template] Badly formed variable names.');
0107         end
0108     otherwise
0109         error('[Template] Unknown action to perform.');
0110 end
```

---

Generated on Tue 29-Oct-2019 21:04:54 by **m2html** © 2005
