# Supplementary material for: Personalized whole‐body models integrate metabolism, physiology, and the gut microbiome
Source: Mol Syst Biol. 2020 May 28;16(5):e8982. doi: 10.15252/msb.20198982 (PMC7285886; doi:10.15252/msb.20198982)
Supplement: Supplementary file 22 — Dataset EV1 [file MSB-16-e8982-s022.zip › PSCM_toolbox/ext/m2html/doc/m2html/@template/get.html]

Description of get


Home > m2html > @template > get.m

# get

## PURPOSE

**TEMPLATE/GET Access data stored in a Template object**

## SYNOPSIS

**function varargout = get(tpl,action,varargin)**

## DESCRIPTION

```
TEMPLATE/GET Access data stored in a Template object
  TPL = GET(TPL,ACTION,VARARGIN)
     ACTION 'var'
     ACTION 'undefined'
```

## CROSS-REFERENCE INFORMATION

This function calls:

- char TEMPLATE Convert a template object in a one line description string
- get TEMPLATE/GET Access data stored in a Template object

This function is called by:

- get TEMPLATE/GET Access data stored in a Template object
- parse TEMPLATE/PARSE Fill in replacement fields with the class properties
- set TEMPLATE/SET Edit data stored in a Template object

## SOURCE CODE

```
0001 function varargout = get(tpl,action,varargin)
0002 %TEMPLATE/GET Access data stored in a Template object
0003 %  TPL = GET(TPL,ACTION,VARARGIN)
0004 %     ACTION 'var'
0005 %     ACTION 'undefined'
0006 
0007 %  Copyright (C) 2003 Guillaume Flandin <Guillaume@artefact.tk>
0008 %  $Revision: 1.0 $Date: 2003/05/05 22:19:51 $
0009 
0010 error(nargchk(2,3,nargin));
0011 
0012 switch lower(action)
0013     case 'var'
0014         error(nargchk(2,3,nargin));
0015         if nargin == 2
0016             varargout{1} = tpl.varvals;
0017         elseif iscellstr(varargin{1})
0018             varargout{1} = {};
0019             for i=1:length(varargin{1})
0020                 key = find(ismember(tpl.varkeys,varargin{1}{i}));
0021                 if isempty(key)
0022                     %error('[Template] No such variable name.');
0023                     varargout{1}{end+1} = '';
0024                 else
0025                     varargout{1}{end+1} = tpl.varvals{key};
0026                 end
0027             end
0028         elseif ischar(varargin{1})
0029             varargout{1} = char(get(tpl,'var',cellstr(varargin{1})));
0030         else
0031             varargout{1} = '';
0032         end
0033     case 'undefined'
0034         error(nargchk(3,3,nargin));
0035         tpl = loadtpl(tpl,varargin{1});
0036         str = get(tpl,'var',varargin{1});
0037         varargout{1} = {};
0038         
0039         %%%%%%%%%%%%%%%%%%%%%%%% WIH REGEXP ONLY %%%%%%%%%%%%%%%%%%%%
0040         % [b, e] = regexp(str,'{[^ \t\r\n}]+}');
0041         % for i=1:length(b)
0042         %     if ~any(ismember(tpl.varkeys,str(b(i)+1:e(i)-1)))
0043         %         varargout{1}{end+1} = str(b(i)+1:e(i)-1);
0044         %    end
0045         % end
0046         %%%%%%%%%%%%%%%%%%%%%%%%%%%%%%%%%%%%%%%%%%%%%%%%%%%%%%%%%%%%%
0047 
0048     otherwise
0049         varargout{1} = finish(get(tpl,'var',action),tpl.unknowns);
0050 end
```

---

Generated on Tue 29-Oct-2019 21:04:54 by **m2html** © 2005
