# Supplementary material for: Personalized whole‐body models integrate metabolism, physiology, and the gut microbiome
Source: Mol Syst Biol. 2020 May 28;16(5):e8982. doi: 10.15252/msb.20198982 (PMC7285886; doi:10.15252/msb.20198982)
Supplement: Supplementary file 22 — Dataset EV1 [file MSB-16-e8982-s022.zip › PSCM_toolbox/ext/m2html/doc/m2html/private/searchindex.html]

Description of searchindex


Home > m2html > private > searchindex.m

# searchindex

## PURPOSE

**SEARCHINDEX Compute keywords statistics of an M-file**

## SYNOPSIS

**function [s, freq] = searchindex(mfile, szmin)**

## DESCRIPTION

```
SEARCHINDEX Compute keywords statistics of an M-file
  S = SEARCHINDEX(MFILE) returns a cell array of char S containing
  all the keywords (variables, function names, words in comments or
  char arrays) found in M-file MFILE, of more than 2 characters.
  S = SEARCHINDEX(MFILE, SZMIN) allows to specify the minimum size
  SZMIN of the keywords.
  [S, FREQ] = SEARCHINDEX(...) also returns the occurency frequence
  of each keyword in the M-file.

  See also M2HTML
```

## CROSS-REFERENCE INFORMATION

This function calls:

- openfile OPENFILE Open a file in read/write mode, catching errors
- strtok Modified version of STRTOK to also return the quotient

This function is called by:


## SOURCE CODE

```
0001 function [s, freq] = searchindex(mfile, szmin)
0002 %SEARCHINDEX Compute keywords statistics of an M-file
0003 %  S = SEARCHINDEX(MFILE) returns a cell array of char S containing
0004 %  all the keywords (variables, function names, words in comments or
0005 %  char arrays) found in M-file MFILE, of more than 2 characters.
0006 %  S = SEARCHINDEX(MFILE, SZMIN) allows to specify the minimum size
0007 %  SZMIN of the keywords.
0008 %  [S, FREQ] = SEARCHINDEX(...) also returns the occurency frequence
0009 %  of each keyword in the M-file.
0010 %
0011 %  See also M2HTML
0012 
0013 %  Copyright (C) 2003 Guillaume Flandin <Guillaume@artefact.tk>
0014 %  $Revision: 1.0 $Date: 2003/04/10 18:32:48 $
0015 
0016 error(nargchk(1,2,nargin));
0017 if nargin == 1, szmin = 2; end
0018 
0019 %- Delimiters used in strtok
0020 strtok_delim = sprintf(' \t\n\r(){}[]<>+-*^$~#!|\\@&/.,:;="''%%');
0021 
0022 %- Open for reading the M-file
0023 fid = openfile(mfile,'r');
0024 
0025 %- Initialize keywords list
0026 s = {};
0027 
0028 %- Loop over lines
0029 while 1
0030     tline = fgetl(fid);
0031     if ~ischar(tline), break, end
0032     
0033     %- Extract keywords in each line
0034     while 1
0035         [w, tline] = strtok(tline,strtok_delim);
0036         if isempty(w), break, end;
0037         %- Check the length of the keyword
0038         if length(w) > szmin
0039             s{end+1} = w;
0040         end
0041     end
0042 end
0043 
0044 %- Close the M-file
0045 fclose(fid);
0046 
0047 %- Remove repeted keywords
0048 [s, i, j] = unique(s);
0049 
0050 %- Compute occurency frenquency if required
0051 if nargout == 2,
0052     if ~isempty(s)
0053         freq = histc(j,1:length(i));
0054     else
0055         freq = [];
0056     end
0057 end
```

---

Generated on Tue 29-Oct-2019 21:04:54 by **m2html** © 2005
