# Supplementary material for: Personalized whole‐body models integrate metabolism, physiology, and the gut microbiome
Source: Mol Syst Biol. 2020 May 28;16(5):e8982. doi: 10.15252/msb.20198982 (PMC7285886; doi:10.15252/msb.20198982)
Supplement: Supplementary file 22 — Dataset EV1 [file MSB-16-e8982-s022.zip › PSCM_toolbox/ext/m2html/doc/m2html/private/index.html]

Index for Directory m2html/private


|  |  |
| --- | --- |
| Master index | Index for m2html/private |

# Index for m2html/private

## Matlab files in this directory:

|  |  |
| --- | --- |
| doxyread | DOXYREAD Read a 'search.idx' file generated by DOXYGEN |
| doxysearch | DOXYSEARCH Search a query in a 'search.idx' file |
| doxywrite | DOXYWRITE Write a 'search.idx' file compatible with DOXYGEN |
| mexexts | MEXEXTS List of Mex files extensions |
| mfileparse | MFILEPARSE Parsing of an M-file to obtain synopsis, help and references |
| openfile | OPENFILE Open a file in read/write mode, catching errors |
| searchindex | SEARCHINDEX Compute keywords statistics of an M-file |
| splitcode | SPLITCODE Split a line of Matlab code in string, comment and other |
| strtok | Modified version of STRTOK to also return the quotient |

## Other Matlab-specific files in this directory:

- m2htmltoolbarimages.mat

---

Generated on Tue 29-Oct-2019 21:04:53 by **m2html** © 2005
