# Supplementary material for: Personalized whole‐body models integrate metabolism, physiology, and the gut microbiome
Source: Mol Syst Biol. 2020 May 28;16(5):e8982. doi: 10.15252/msb.20198982 (PMC7285886; doi:10.15252/msb.20198982)
Supplement: Supplementary file 22 — Dataset EV1 [file MSB-16-e8982-s022.zip › PSCM_toolbox/ext/m2html/doc/m2html/private/openfile.html]

Description of openfile


Home > m2html > private > openfile.m

# openfile

## PURPOSE

**OPENFILE Open a file in read/write mode, catching errors**

## SYNOPSIS

**function fid = openfile(filename,permission)**

## DESCRIPTION

```
OPENFILE Open a file in read/write mode, catching errors
  FID = OPENFILE(FILENAME,PERMISSION) opens file FILENAME
  in PERMISSION mode ('r' or 'w') and return a file identifier FID.
  File is opened in text mode: no effect on Unix but useful on Windows.
```

## CROSS-REFERENCE INFORMATION

This function calls:


This function is called by:

- mfileparse MFILEPARSE Parsing of an M-file to obtain synopsis, help and references
- searchindex SEARCHINDEX Compute keywords statistics of an M-file

## SOURCE CODE

```
0001 function fid = openfile(filename,permission)
0002 %OPENFILE Open a file in read/write mode, catching errors
0003 %  FID = OPENFILE(FILENAME,PERMISSION) opens file FILENAME
0004 %  in PERMISSION mode ('r' or 'w') and return a file identifier FID.
0005 %  File is opened in text mode: no effect on Unix but useful on Windows.
0006 
0007 %  Copyright (C) 2004 Guillaume Flandin <Guillaume@artefact.tk>
0008 %  $Revision: 1.1 $Date: 2004/05/05 17:14:09 $
0009 
0010 [fid, errmsg] = fopen(filename,[permission 't']);
0011 if ~isempty(errmsg)
0012     switch permission
0013         case 'r'
0014             error(sprintf('Cannot open %s in read mode.',filename));
0015         case 'w'
0016             error(sprintf('Cannot open %s in write mode.',filename));
0017         otherwise
0018             error(errmsg);
0019     end
0020 end
```

---

Generated on Tue 29-Oct-2019 21:04:54 by **m2html** © 2005
