# Supplementary material for: Personalized whole‐body models integrate metabolism, physiology, and the gut microbiome
Source: Mol Syst Biol. 2020 May 28;16(5):e8982. doi: 10.15252/msb.20198982 (PMC7285886; doi:10.15252/msb.20198982)
Supplement: Supplementary file 22 — Dataset EV1 [file MSB-16-e8982-s022.zip › PSCM_toolbox/ext/m2html/doc/m2html/private/mfileparse.html]

Description of mfileparse


Home > m2html > private > mfileparse.m

# mfileparse

## PURPOSE

**MFILEPARSE Parsing of an M-file to obtain synopsis, help and references**

## SYNOPSIS

**function s = mfileparse(mfile, mdirs, names, options)**

## DESCRIPTION

```
MFILEPARSE Parsing of an M-file to obtain synopsis, help and references
  S = MFILEPARSE(MFILE, MDIRS, NAMES, OPTIONS) parses the M-file MFILE looking
  for synopsis (function), H1 line, subroutines and todo tags (if any).
  It also fills in a boolean array indicating whether MFILE calls M-files 
  defined by MDIRS (M-files directories) AND NAMES (M-file names).
  The input OPTIONS comes from M2HTML: fields used are 'verbose', 'global'
  and 'todo'.
  Output S is a structure whose fields are:
     o synopsis: char array (empty if MFILE is a script)
     o h1line: short one-line description into the first help line
     o subroutine: cell array of char containing subroutines synopsis
     o hrefs: boolean array with hrefs(i) = 1 if MFILE calls mdirs{i}/names{i}
     o todo: structure containing information about potential todo tags

  See also M2HTML
```

## CROSS-REFERENCE INFORMATION

This function calls:

- mexexts MEXEXTS List of Mex files extensions
- openfile OPENFILE Open a file in read/write mode, catching errors
- splitcode SPLITCODE Split a line of Matlab code in string, comment and other
- strtok Modified version of STRTOK to also return the quotient

This function is called by:


## SOURCE CODE

```
0001 function s = mfileparse(mfile, mdirs, names, options)
0002 %MFILEPARSE Parsing of an M-file to obtain synopsis, help and references
0003 %  S = MFILEPARSE(MFILE, MDIRS, NAMES, OPTIONS) parses the M-file MFILE looking
0004 %  for synopsis (function), H1 line, subroutines and todo tags (if any).
0005 %  It also fills in a boolean array indicating whether MFILE calls M-files
0006 %  defined by MDIRS (M-files directories) AND NAMES (M-file names).
0007 %  The input OPTIONS comes from M2HTML: fields used are 'verbose', 'global'
0008 %  and 'todo'.
0009 %  Output S is a structure whose fields are:
0010 %     o synopsis: char array (empty if MFILE is a script)
0011 %     o h1line: short one-line description into the first help line
0012 %     o subroutine: cell array of char containing subroutines synopsis
0013 %     o hrefs: boolean array with hrefs(i) = 1 if MFILE calls mdirs{i}/names{i}
0014 %     o todo: structure containing information about potential todo tags
0015 %
0016 %  See also M2HTML
0017 
0018 %  Copyright (C) 2003 Guillaume Flandin <Guillaume@artefact.tk>
0019 %  $Revision: 1.0 $Date: 2003/29/04 17:33:43 $
0020 
0021 error(nargchk(3,4,nargin));
0022 if nargin == 3,
0023     options = struct('verbose',1, 'globalHypertextLinks',0, 'todo',0);
0024 end
0025 
0026 %- Delimiters used in strtok: some of them may be useless (% " .), removed '.'
0027 strtok_delim = sprintf(' \t\n\r(){}[]<>+-*~!|\\@&/,:;="''%%');
0028 
0029 %- Open for reading the M-file
0030 fid = openfile(mfile,'r');
0031 it = 0; % line number
0032 
0033 %- Initialize Output
0034 s = struct('synopsis',   '', ...
0035            'h1line',     '', ...
0036            'subroutine', {{}}, ...
0037            'hrefs',      sparse(1,length(names)), ...
0038            'todo',       struct('line',[],'comment',{{}}), ...
0039            'ismex',      zeros(size(mexexts)));
0040 
0041 %- Initialize flag for synopsis cont ('...')
0042 flagsynopcont = 0;
0043 %- Look for synopsis and H1 line
0044 %  Help is the first set of contiguous comment lines in an m-file
0045 %  The H1 line is a short one-line description into the first help line
0046 while 1
0047     tline = fgetl(fid);
0048     if ~ischar(tline), break, end
0049     it = it + 1;
0050     tline = deblank(fliplr(deblank(fliplr(tline))));
0051     %- Synopsis line
0052     if ~isempty(strmatch('function',tline))
0053         s.synopsis = tline;
0054         if ~isempty(strmatch('...',fliplr(tline)))
0055             flagsynopcont = 1;
0056             s.synopsis = deblank(s.synopsis(1:end-3));
0057         end
0058     %- H1 Line
0059     elseif ~isempty(strmatch('%',tline))
0060         % allow for the help lines to be before the synopsis
0061         if isempty(s.h1line)
0062             s.h1line = fliplr(deblank(tline(end:-1:2)));
0063         end
0064         if ~isempty(s.synopsis), break, end
0065     %- Go through empty lines
0066     elseif isempty(tline)
0067         
0068     %- Code found. Stop.
0069     else
0070         if flagsynopcont
0071             if isempty(strmatch('...',fliplr(tline)))
0072                 s.synopsis = [s.synopsis tline];
0073                 flagsynopcont = 0;
0074             else
0075                 s.synopsis = [s.synopsis deblank(tline(1:end-3))];
0076             end
0077         else
0078             break;
0079         end
0080     end
0081 end
0082 
0083 %- Global Hypertext Links option
0084 %  If false, hypertext links are done only among functions in the same
0085 %  directory.
0086 if options.globalHypertextLinks
0087     hrefnames = names;
0088 else
0089     indhref = find(strcmp(fileparts(mfile),mdirs));
0090     hrefnames = {names{indhref}};
0091 end
0092 
0093 %- Compute cross-references and extract subroutines
0094 %  hrefs(i) is 1 if mfile calls mfiles{i} and 0 otherwise
0095 while ischar(tline)
0096     % Remove blanks at both ends
0097     tline = deblank(fliplr(deblank(fliplr(tline))));
0098     
0099     % Split code into meaningful chunks
0100     splitc = splitcode(tline);
0101     for j=1:length(splitc)
0102         if isempty(splitc{j}) | ...
0103             splitc{j}(1) == '''' | ...
0104             ~isempty(strmatch('...',splitc{j}))
0105             % Forget about empty lines, char strings or conts
0106         elseif splitc{j}(1) == '%'
0107             % Cross-references are not taken into account in comments
0108             % Just look for potential TODO or FIXME line
0109             if options.todo
0110                 if ~isempty(strmatch('% TODO',splitc{j})) | ...
0111                    ~isempty(strmatch('% FIXME',splitc{j}))
0112                     s.todo.line   = [s.todo.line it];
0113                     s.todo.comment{end+1} = splitc{j}(9:end);
0114                 end
0115             end
0116         else
0117             % detect if this line is a declaration of a subroutine
0118             if ~isempty(strmatch('function',splitc{j}))
0119                 s.subroutine{end+1} = splitc{j};
0120             else
0121                 % get list of variables and functions
0122                 symbol = {};
0123                 while 1
0124                     [t,splitc{j}] = strtok(splitc{j},strtok_delim);
0125                     if isempty(t), break, end;
0126                     symbol{end+1} = t;
0127                 end
0128                 if options.globalHypertextLinks
0129                     s.hrefs = s.hrefs + ismember(hrefnames,symbol);
0130                 else
0131                     if ~isempty(indhref)
0132                         s.hrefs(indhref) = s.hrefs(1,indhref) + ...
0133                                            ismember(hrefnames,symbol);
0134                     end
0135                 end
0136             end
0137         end
0138     end
0139     tline = fgetl(fid);
0140     it = it + 1;
0141 end    
0142 
0143 fclose(fid);
0144 
0145 %- Look for Mex files
0146 [pathstr,name] = fileparts(mfile);
0147 samename = dir(fullfile(pathstr,[name    '.*']));
0148 samename = {samename.name};
0149 ext = {};
0150 for i=1:length(samename)
0151     [dummy, dummy, ext{i}] = fileparts(samename{i});
0152 end
0153 s.ismex = ismember(mexexts,ext);
```

---

Generated on Tue 29-Oct-2019 21:04:54 by **m2html** © 2005
