# Supplementary material for: Personalized whole‐body models integrate metabolism, physiology, and the gut microbiome
Source: Mol Syst Biol. 2020 May 28;16(5):e8982. doi: 10.15252/msb.20198982 (PMC7285886; doi:10.15252/msb.20198982)
Supplement: Supplementary file 22 — Dataset EV1 [file MSB-16-e8982-s022.zip › PSCM_toolbox/ext/m2html/doc/m2html/private/splitcode.html]

Description of splitcode


Home > m2html > private > splitcode.m

# splitcode

## PURPOSE

**SPLITCODE Split a line of Matlab code in string, comment and other**

## SYNOPSIS

**function splitc = splitcode(code)**

## DESCRIPTION

```
SPLITCODE Split a line of Matlab code in string, comment and other
  SPLITC = SPLITCODE(CODE) splits line of Matlab code CODE into a cell
  array SPLITC where each element is either a character array ('...'),
  a comment (%...), a continuation (...) or something else.
  Note that CODE = [SPLITC{:}]

  See also M2HTML, HIGHLIGHT
```

## CROSS-REFERENCE INFORMATION

This function calls:


This function is called by:

- mfileparse MFILEPARSE Parsing of an M-file to obtain synopsis, help and references

## SOURCE CODE

```
0001 function splitc = splitcode(code)
0002 %SPLITCODE Split a line of Matlab code in string, comment and other
0003 %  SPLITC = SPLITCODE(CODE) splits line of Matlab code CODE into a cell
0004 %  array SPLITC where each element is either a character array ('...'),
0005 %  a comment (%...), a continuation (...) or something else.
0006 %  Note that CODE = [SPLITC{:}]
0007 %
0008 %  See also M2HTML, HIGHLIGHT
0009 
0010 %  Copyright (C) 2003 Guillaume Flandin <Guillaume@artefact.tk>
0011 %  $Revision: 1.0 $Date: 2003/29/04 17:33:43 $
0012 
0013 %- Label quotes in {'transpose', 'beginstring', 'midstring', 'endstring'}
0014 iquote = findstr(code,'''');
0015 quotetransp = [double('_''.)}]') ...
0016                double('A'):double('Z') ...
0017                double('0'):double('9') ...
0018                double('a'):double('z')];
0019 flagstring = 0;
0020 flagdoublequote = 0;
0021 jquote = [];
0022 for i=1:length(iquote)
0023     if ~flagstring
0024         if iquote(i) > 1 & any(quotetransp == double(code(iquote(i)-1)))
0025             % => 'transpose';
0026         else
0027             % => 'beginstring';
0028             jquote(size(jquote,1)+1,:) = [iquote(i) length(code)];
0029             flagstring = 1;
0030         end
0031     else % if flagstring
0032         if flagdoublequote | ...
0033            (iquote(i) < length(code) & strcmp(code(iquote(i)+1),''''))
0034             % => 'midstring';
0035             flagdoublequote = ~flagdoublequote;
0036         else
0037             % => 'endstring';
0038             jquote(size(jquote,1),2) = iquote(i);
0039             flagstring = 0;
0040         end
0041     end
0042 end
0043 
0044 %- Find if a portion of code is a comment
0045 ipercent = findstr(code,'%');
0046 jpercent = [];
0047 for i=1:length(ipercent)
0048     if isempty(jquote) | ...
0049        ~any((ipercent(i) > jquote(:,1)) & (ipercent(i) < jquote(:,2)))
0050         jpercent = [ipercent(i) length(code)];
0051         break;
0052     end
0053 end
0054 
0055 %- Find continuation punctuation '...'
0056 icont = findstr(code,'...');
0057 for i=1:length(icont)
0058     if (isempty(jquote) | ...
0059         ~any((icont(i) > jquote(:,1)) & (icont(i) < jquote(:,2)))) & ...
0060         (isempty(jpercent) | ...
0061         icont(i) < jpercent(1))
0062         jpercent = [icont(i) length(code)];
0063         break;
0064     end
0065 end
0066 
0067 %- Remove strings inside comments
0068 if ~isempty(jpercent) & ~isempty(jquote)
0069     jquote(find(jquote(:,1) > jpercent(1)),:) = [];
0070 end
0071 
0072 %- Split code in a cell array of strings
0073 icode = [jquote ; jpercent];
0074 splitc = {};
0075 if isempty(icode)
0076     splitc{1} = code;
0077 elseif icode(1,1) > 1
0078     splitc{1} = code(1:icode(1,1)-1);
0079 end
0080 for i=1:size(icode,1)
0081     splitc{end+1} = code(icode(i,1):icode(i,2));
0082     if i < size(icode,1) & icode(i+1,1) > icode(i,2) + 1
0083         splitc{end+1} = code((icode(i,2)+1):(icode(i+1,1)-1));
0084     elseif i == size(icode,1) & icode(i,2) < length(code)
0085         splitc{end+1} = code(icode(i,2)+1:end);
0086     end
0087 end
```

---

Generated on Tue 29-Oct-2019 21:04:54 by **m2html** © 2005
