# Supplementary material for: Personalized whole‐body models integrate metabolism, physiology, and the gut microbiome
Source: Mol Syst Biol. 2020 May 28;16(5):e8982. doi: 10.15252/msb.20198982 (PMC7285886; doi:10.15252/msb.20198982)
Supplement: Supplementary file 22 — Dataset EV1 [file MSB-16-e8982-s022.zip › PSCM_toolbox/ext/m2html/doc/m2html/private/doxywrite.html]

Description of doxywrite


Home > m2html > private > doxywrite.m

# doxywrite

## PURPOSE

**DOXYWRITE Write a 'search.idx' file compatible with DOXYGEN**

## SYNOPSIS

**function doxywrite(filename, kw, statinfo, docinfo)**

## DESCRIPTION

```
DOXYWRITE Write a 'search.idx' file compatible with DOXYGEN
  DOXYWRITE(FILENAME, KW, STATINFO, DOCINFO) writes file FILENAME
  (Doxygen search.idx. format) using the cell array KW containing the
  word list, the sparse matrix (nbword x nbfile) with non-null values
  in (i,j) indicating the frequency of occurence of word i in file j
  and the cell array (nbfile x 2) containing the list of urls and names
  of each file.

  See also DOXYREAD
```

## CROSS-REFERENCE INFORMATION

This function calls:


This function is called by:


## SUBFUNCTIONS

- function writeString(fid, s)
- function writeInt(fid, i)

## SOURCE CODE

```
0001 function doxywrite(filename, kw, statinfo, docinfo)
0002 %DOXYWRITE Write a 'search.idx' file compatible with DOXYGEN
0003 %  DOXYWRITE(FILENAME, KW, STATINFO, DOCINFO) writes file FILENAME
0004 %  (Doxygen search.idx. format) using the cell array KW containing the
0005 %  word list, the sparse matrix (nbword x nbfile) with non-null values
0006 %  in (i,j) indicating the frequency of occurence of word i in file j
0007 %  and the cell array (nbfile x 2) containing the list of urls and names
0008 %  of each file.
0009 %
0010 %  See also DOXYREAD
0011 
0012 %  Copyright (C) 2003 Guillaume Flandin <Guillaume@artefact.tk>
0013 %  $Revision: 1.0 $Date: 2003/23/10 15:52:56 $
0014 
0015 %  This program is free software; you can redistribute it and/or
0016 %  modify it under the terms of the GNU General Public License
0017 %  as published by the Free Software Foundation; either version 2
0018 %  of the License, or any later version.
0019 %
0020 %  This program is distributed in the hope that it will be useful,
0021 %  but WITHOUT ANY WARRANTY; without even the implied warranty of
0022 %  MERCHANTABILITY or FITNESS FOR A PARTICULAR PURPOSE.  See the
0023 %  GNU General Public License for more details.
0024 %
0025 %  You should have received a copy of the GNU General Public License
0026 %  along with this program; if not, write to the Free Software
0027 %  Foundation Inc, 59 Temple Pl. - Suite 330, Boston, MA 02111-1307, USA.
0028 
0029 %  Suggestions for improvement and fixes are always welcome, although no
0030 %  guarantee is made whether and when they will be implemented.
0031 %  Send requests to <Guillaume@artefact.tk>
0032 
0033 %  See <http://www.doxygen.org/> for more details.
0034 
0035 error(nargchk(4,4,nargin));
0036 
0037 %- Open the search index file
0038 [fid, errmsg] = fopen(filename,'w','ieee-be');
0039 if fid == -1, error(errmsg); end
0040 
0041 %- Write 4 byte header (DOXS)
0042 fwrite(fid,'DOXS','uchar');
0043 pos = ftell(fid);
0044 
0045 %- Write 256 * 256 header
0046 idx = zeros(256);
0047 writeInt(fid, idx);
0048 
0049 %- Write word lists
0050 i = 1;
0051 idx2 = zeros(1,length(kw));
0052 while 1
0053     s = kw{i}(1:2);
0054     idx(double(s(2)+1), double(s(1)+1)) = ftell(fid);
0055     while i <= length(kw) & strmatch(s, kw{i})
0056         writeString(fid,kw{i});
0057         idx2(i) = ftell(fid);
0058         writeInt(fid,0);
0059         i = i + 1;
0060     end
0061     fwrite(fid, 0, 'int8');
0062     if i > length(kw), break; end
0063 end
0064 
0065 %- Write extra padding bytes
0066 pad = mod(4 - mod(ftell(fid),4), 4);
0067 for i=1:pad, fwrite(fid,0,'int8'); end
0068 pos2 = ftell(fid);
0069 
0070 %- Write 256*256 header again
0071   fseek(fid, pos, 'bof');
0072   writeInt(fid, idx);
0073 
0074 % Write word statistics
0075 fseek(fid,pos2,'bof');
0076 idx3 = zeros(1,length(kw));
0077 for i=1:length(kw)
0078     idx3(i) = ftell(fid);
0079     [ia, ib, v] = find(statinfo(i,:));
0080     counter = length(ia); % counter
0081     writeInt(fid,counter);
0082     for j=1:counter
0083         writeInt(fid,ib(j)); % index
0084         writeInt(fid,v(j));  % freq
0085     end
0086 end
0087 pos3 = ftell(fid);
0088 
0089 %- Set correct handles to keywords
0090   for i=1:length(kw)
0091       fseek(fid,idx2(i),'bof');
0092     writeInt(fid,idx3(i));
0093   end
0094 
0095 % Write urls
0096 fseek(fid,pos3,'bof');
0097 idx4 = zeros(1,length(docinfo));
0098 for i=1:length(docinfo)
0099     idx4(i) = ftell(fid);
0100     writeString(fid, docinfo{i,1}); % name
0101     writeString(fid, docinfo{i,2}); % url
0102 end
0103 
0104 %- Set corrext handles to word statistics
0105 fseek(fid,pos2,'bof');
0106 for i=1:length(kw)
0107     [ia, ib, v] = find(statinfo(i,:));
0108     counter = length(ia);
0109     fseek(fid,4,'cof'); % counter
0110     for m=1:counter
0111         writeInt(fid,idx4(ib(m)));% index
0112         fseek(fid,4,'cof'); % freq
0113     end
0114 end
0115 
0116 %- Close the search index file
0117 fclose(fid);
0118 
0119 %===========================================================================
0120 function writeString(fid, s)
0121 
0122     fwrite(fid,s,'uchar');
0123     fwrite(fid,0,'int8');
0124 
0125 %===========================================================================
0126 function writeInt(fid, i)
0127     
0128     fwrite(fid,i,'uint32');
```

---

Generated on Tue 29-Oct-2019 21:04:54 by **m2html** © 2005
