# Supplementary material for: Personalized whole‐body models integrate metabolism, physiology, and the gut microbiome
Source: Mol Syst Biol. 2020 May 28;16(5):e8982. doi: 10.15252/msb.20198982 (PMC7285886; doi:10.15252/msb.20198982)
Supplement: Supplementary file 22 — Dataset EV1 [file MSB-16-e8982-s022.zip › PSCM_toolbox/ext/m2html/doc/m2html/private/doxyread.html]

Description of doxyread


Home > m2html > private > doxyread.m

# doxyread

## PURPOSE

**DOXYREAD Read a 'search.idx' file generated by DOXYGEN**

## SYNOPSIS

**function [statlist, docinfo] = doxyread(filename)**

## DESCRIPTION

```
DOXYREAD Read a 'search.idx' file generated by DOXYGEN
  STATLIST = DOXYREAD(FILENAME) reads FILENAME (Doxygen search.idx
  format) and returns the list of keywords STATLIST as a cell array.
  [STATLIST, DOCINFO] = DOXYREAD(FILENAME) also returns a cell array
  containing details for each keyword (frequency in each file where it
  appears and the URL).

  See also DOXYSEARCH, DOXYWRITE
```

## CROSS-REFERENCE INFORMATION

This function calls:


This function is called by:


## SUBFUNCTIONS

- function s = readString(fid)
- function i = readInt(fid)

## SOURCE CODE

```
0001 function [statlist, docinfo] = doxyread(filename)
0002 %DOXYREAD Read a 'search.idx' file generated by DOXYGEN
0003 %  STATLIST = DOXYREAD(FILENAME) reads FILENAME (Doxygen search.idx
0004 %  format) and returns the list of keywords STATLIST as a cell array.
0005 %  [STATLIST, DOCINFO] = DOXYREAD(FILENAME) also returns a cell array
0006 %  containing details for each keyword (frequency in each file where it
0007 %  appears and the URL).
0008 %
0009 %  See also DOXYSEARCH, DOXYWRITE
0010 
0011 %  Copyright (C) 2003 Guillaume Flandin <Guillaume@artefact.tk>
0012 %  $Revision: 1.0 $Date: 2003/05/10 17:41:21 $
0013 
0014 %  This program is free software; you can redistribute it and/or
0015 %  modify it under the terms of the GNU General Public License
0016 %  as published by the Free Software Foundation; either version 2
0017 %  of the License, or any later version.
0018 %
0019 %  This program is distributed in the hope that it will be useful,
0020 %  but WITHOUT ANY WARRANTY; without even the implied warranty of
0021 %  MERCHANTABILITY or FITNESS FOR A PARTICULAR PURPOSE.  See the
0022 %  GNU General Public License for more details.
0023 %
0024 %  You should have received a copy of the GNU General Public License
0025 %  along with this program; if not, write to the Free Software
0026 %  Foundation Inc, 59 Temple Pl. - Suite 330, Boston, MA 02111-1307, USA.
0027 
0028 %  Suggestions for improvement and fixes are always welcome, although no
0029 %  guarantee is made whether and when they will be implemented.
0030 %  Send requests to <Guillaume@artefact.tk>
0031 
0032 %  See <http://www.doxygen.org/> for more details.
0033 
0034 error(nargchk(0,1,nargin));
0035 if nargin == 0,
0036     filename = 'search.idx';
0037 end
0038 
0039 %- Open the search index file
0040 [fid, errmsg] = fopen(filename,'r','ieee-be');
0041 if fid == -1, error(errmsg); end
0042 
0043 %- 4 byte header (DOXS)
0044 header = char(fread(fid,4,'uchar'))';
0045 
0046 %- 256*256*4 byte index
0047 idx = fread(fid,256*256,'uint32');
0048 idx = reshape(idx,256,256);
0049 
0050 %- Extract list of words
0051 i = find(idx);
0052 statlist = cell(0,2);    
0053 for j=1:length(i) 
0054     fseek(fid, idx(i(j)), 'bof');    
0055     statw    = readString(fid);
0056     while ~isempty(statw)
0057         statidx  = readInt(fid);
0058         statlist{end+1,1} = statw; % word
0059         statlist{end,2}   = statidx; % index
0060         statw   = readString(fid);
0061     end
0062 end
0063     
0064 %- Extract occurence frequency of each word and docs info (name and url)
0065 docinfo = cell(size(statlist,1),1);
0066 for k=1:size(statlist,1)
0067     fseek(fid, statlist{k,2}, 'bof');
0068     numdoc = readInt(fid);
0069     docinfo{k} = cell(numdoc,4);
0070     for m=1:numdoc
0071         docinfo{k}{m,1} = readInt(fid); % idx
0072         docinfo{k}{m,2} = readInt(fid); % freq
0073     end
0074     for m=1:numdoc
0075         fseek(fid, docinfo{k}{m,1}, 'bof');
0076         docinfo{k}{m,3} = readString(fid); % name
0077         docinfo{k}{m,4} = readString(fid); % url
0078     end
0079     docinfo{k} = reshape({docinfo{k}{:,2:4}},numdoc,[]);
0080 end
0081 
0082 %- Close the search index file
0083 fclose(fid);
0084 
0085 %- Remove indexes
0086 statlist = {statlist{:,1}}';
0087 
0088 %===========================================================================
0089 function s = readString(fid)
0090 
0091     s = '';
0092     while 1
0093         w = fread(fid,1,'uchar');
0094         if w == 0, break; end
0095         s(end+1) = char(w);
0096     end
0097 
0098 %===========================================================================
0099 function i = readInt(fid)
0100 
0101     i = fread(fid,1,'uint32');
```

---

Generated on Tue 29-Oct-2019 21:04:54 by **m2html** © 2005
