# Supplementary material for: Personalized whole‐body models integrate metabolism, physiology, and the gut microbiome
Source: Mol Syst Biol. 2020 May 28;16(5):e8982. doi: 10.15252/msb.20198982 (PMC7285886; doi:10.15252/msb.20198982)
Supplement: Supplementary file 22 — Dataset EV1 [file MSB-16-e8982-s022.zip › PSCM_toolbox/ext/m2html/doc/m2html/private/doxysearch.html]

Description of doxysearch


Home > m2html > private > doxysearch.m

# doxysearch

## PURPOSE

**DOXYSEARCH Search a query in a 'search.idx' file**

## SYNOPSIS

**function result = doxysearch(query,filename)**

## DESCRIPTION

```
DOXYSEARCH Search a query in a 'search.idx' file
  RESULT = DOXYSEARCH(QUERY,FILENAME) looks for request QUERY
  in FILENAME (Doxygen search.idx format) and returns a list of
  files responding to the request in RESULT.

  See also DOXYREAD, DOXYWRITE
```

## CROSS-REFERENCE INFORMATION

This function calls:

- strtok Modified version of STRTOK to also return the quotient

This function is called by:


## SUBFUNCTIONS

- function res = searchAgain(fid, word,res)
- function docs = combineResults(result)
- function filtdocs = filterResults(docs,requiredWords,forbiddenWords)
- function docs = normalizeResults(docs);
- function result = sortResults(docs);
- function i = computeIndex(word)
- function s = readString(fid)
- function i = readInt(fid)

## SOURCE CODE

```
0001 function result = doxysearch(query,filename)
0002 %DOXYSEARCH Search a query in a 'search.idx' file
0003 %  RESULT = DOXYSEARCH(QUERY,FILENAME) looks for request QUERY
0004 %  in FILENAME (Doxygen search.idx format) and returns a list of
0005 %  files responding to the request in RESULT.
0006 %
0007 %  See also DOXYREAD, DOXYWRITE
0008 
0009 %  Copyright (C) 2004 Guillaume Flandin <Guillaume@artefact.tk>
0010 %  $Revision: 1.1 $Date: 2004/05/05 14:33:55 $
0011 
0012 %  This program is free software; you can redistribute it and/or
0013 %  modify it under the terms of the GNU General Public License
0014 %  as published by the Free Software Foundation; either version 2
0015 %  of the License, or any later version.
0016 %
0017 %  This program is distributed in the hope that it will be useful,
0018 %  but WITHOUT ANY WARRANTY; without even the implied warranty of
0019 %  MERCHANTABILITY or FITNESS FOR A PARTICULAR PURPOSE.  See the
0020 %  GNU General Public License for more details.
0021 %
0022 %  You should have received a copy of the GNU General Public License
0023 %  along with this program; if not, write to the Free Software
0024 %  Foundation Inc, 59 Temple Pl. - Suite 330, Boston, MA 02111-1307, USA.
0025 
0026 %  Suggestions for improvement and fixes are always welcome, although no
0027 %  guarantee is made whether and when they will be implemented.
0028 %  Send requests to <Guillaume@artefact.tk>
0029 
0030 %  See <http://www.doxygen.org/> for more details.
0031 
0032 error(nargchk(1,2,nargin));
0033 if nargin == 1,
0034     filename = 'search.idx';
0035 end
0036 
0037 %- Open the search index file
0038 [fid, errmsg] = fopen(filename,'r','ieee-be');
0039 if fid == -1, error(errmsg); end
0040 
0041 %- 4 byte header (DOXS)
0042 header = char(fread(fid,4,'uchar'))';
0043 if ~all(header == 'DOXS')
0044     error('[doxysearch] Header of index file is invalid!');
0045 end
0046 
0047 %- many thanks to <doxyread.m> and <doxysearch.php>
0048 r = query;
0049 requiredWords  = {};
0050 forbiddenWords = {};
0051 foundWords     = {};
0052 res            = {};
0053 while 1
0054     % extract each word of the query
0055     [t,r] = strtok(r);
0056     if isempty(t), break, end;
0057     if t(1) == '+'
0058         t = t(2:end); requiredWords{end+1} = t;
0059     elseif t(1) == '-'
0060         t = t(2:end); forbiddenWords{end+1} = t;
0061     end
0062     if ~ismember(t,foundWords)
0063         foundWords{end+1} = t;
0064         res = searchAgain(fid,t,res);
0065     end
0066 end
0067 
0068 %- Filter and sort results
0069 docs = combineResults(res);
0070 filtdocs = filterResults(docs,requiredWords,forbiddenWords);
0071 filtdocs = normalizeResults(filtdocs);
0072 res = sortResults(filtdocs);
0073 
0074 %-
0075 if nargout
0076     result = res;
0077 else
0078     for i=1:size(res,1)
0079         fprintf('   %d. %s - %s\n      ',i,res{i,1},res{i,2});
0080         for j=1:size(res{i,4},1)
0081             fprintf('%s ',res{i,4}{j,1});
0082         end
0083         fprintf('\n');
0084     end
0085 end
0086 
0087 %- Close the search index file
0088 fclose(fid);
0089 
0090 %===========================================================================
0091 function res = searchAgain(fid, word,res)
0092 
0093     i = computeIndex(word);
0094     if i > 0
0095         
0096         fseek(fid,i*4+4,'bof'); % 4 bytes per entry, skip header
0097         start = size(res,1);
0098         idx = readInt(fid);
0099         
0100         if idx > 0
0101             
0102             fseek(fid,idx,'bof');
0103             statw = readString(fid);
0104             while ~isempty(statw)
0105                 statidx  = readInt(fid);
0106                 if length(statw) >= length(word) & ...
0107                     strcmp(statw(1:length(word)),word)
0108                     res{end+1,1} = statw;   % word
0109                     res{end,2}   = word;    % match
0110                     res{end,3}   = statidx; % index
0111                     res{end,4}   = (length(statw) == length(word)); % full
0112                     res{end,5}   = {};      % doc
0113                 end
0114                 statw = readString(fid);
0115             end
0116         
0117             totalfreq = 0;
0118             for j=start+1:size(res,1)
0119                 fseek(fid,res{j,3},'bof');
0120                 numdoc = readInt(fid);
0121                 docinfo = {};
0122                 for m=1:numdoc
0123                     docinfo{m,1} = readInt(fid); % idx
0124                     docinfo{m,2} = readInt(fid); % freq
0125                     docinfo{m,3} = 0;            % rank
0126                     totalfreq = totalfreq + docinfo{m,2};
0127                     if res{j,2}, 
0128                         totalfreq = totalfreq + docinfo{m,2};
0129                     end;
0130                 end
0131                 for m=1:numdoc
0132                     fseek(fid, docinfo{m,1}, 'bof');
0133                     docinfo{m,4} = readString(fid); % name
0134                     docinfo{m,5} = readString(fid); % url
0135                 end
0136                 res{j,5} = docinfo;
0137             end
0138         
0139             for j=start+1:size(res,1)
0140                 for m=1:size(res{j,5},1)
0141                     res{j,5}{m,3} = res{j,5}{m,2} / totalfreq;
0142                 end
0143             end
0144             
0145         end % if idx > 0
0146         
0147     end % if i > 0
0148 
0149 %===========================================================================
0150 function docs = combineResults(result)
0151 
0152     docs = {};
0153     for i=1:size(result,1)
0154         for j=1:size(result{i,5},1)
0155             key = result{i,5}{j,5};
0156             rank = result{i,5}{j,3};
0157             if ~isempty(docs) & ismember(key,{docs{:,1}})
0158                 l = find(ismember({docs{:,1}},key));
0159                 docs{l,3} = docs{l,3} + rank;
0160                 docs{l,3} = 2 * docs{l,3};
0161             else
0162                 l = size(docs,1)+1;
0163                 docs{l,1} = key; % key
0164                 docs{l,2} = result{i,5}{j,4}; % name
0165                 docs{l,3} = rank; % rank
0166                 docs{l,4} = {}; %words
0167             end
0168             n = size(docs{l,4},1);
0169             docs{l,4}{n+1,1} = result{i,1}; % word
0170             docs{l,4}{n+1,2} = result{i,2}; % match
0171             docs{l,4}{n+1,3} = result{i,5}{j,2}; % freq
0172         end
0173     end
0174 
0175 %===========================================================================
0176 function filtdocs = filterResults(docs,requiredWords,forbiddenWords)
0177 
0178     filtdocs = {};
0179     for i=1:size(docs,1)
0180         words = docs{i,4};
0181         c = 1;
0182         j = size(words,1);
0183         % check required
0184         if ~isempty(requiredWords)
0185             found = 0;
0186             for k=1:j
0187                 if ismember(words{k,1},requiredWords)
0188                     found = 1; 
0189                     break;  
0190                 end
0191             end
0192             if ~found, c = 0; end
0193         end
0194         % check forbidden
0195         if ~isempty(forbiddenWords)
0196             for k=1:j
0197                 if ismember(words{k,1},forbiddenWords)
0198                     c = 0;
0199                     break;
0200                 end
0201             end
0202         end
0203         % keep it or not
0204         if c, 
0205             l = size(filtdocs,1)+1;
0206             filtdocs{l,1} = docs{i,1};
0207             filtdocs{l,2} = docs{i,2};
0208             filtdocs{l,3} = docs{i,3};
0209             filtdocs{l,4} = docs{i,4};
0210         end;
0211     end
0212 
0213 %===========================================================================
0214 function docs = normalizeResults(docs);
0215 
0216     m = max([docs{:,3}]);
0217     for i=1:size(docs,1)
0218         docs{i,3} = 100 * docs{i,3} / m;
0219     end
0220 
0221 %===========================================================================
0222 function result = sortResults(docs);
0223 
0224     [y, ind] = sort([docs{:,3}]);
0225     result = {};
0226     ind = fliplr(ind);
0227     for i=1:size(docs,1)
0228         result{i,1} = docs{ind(i),1};
0229         result{i,2} = docs{ind(i),2};
0230         result{i,3} = docs{ind(i),3};
0231         result{i,4} = docs{ind(i),4};
0232     end
0233 
0234 %===========================================================================
0235 function i = computeIndex(word)
0236 
0237     if length(word) < 2,
0238        i = -1;
0239     else
0240         i = double(word(1)) * 256 + double(word(2));
0241     end
0242     
0243 %===========================================================================
0244 function s = readString(fid)
0245 
0246     s = '';
0247     while 1
0248         w = fread(fid,1,'uchar');
0249         if w == 0, break; end
0250         s(end+1) = char(w);
0251     end
0252 
0253 %===========================================================================
0254 function i = readInt(fid)
0255 
0256     i = fread(fid,1,'uint32');
```

---

Generated on Tue 29-Oct-2019 21:04:54 by **m2html** © 2005
