# Supplementary material for: Personalized whole‐body models integrate metabolism, physiology, and the gut microbiome
Source: Mol Syst Biol. 2020 May 28;16(5):e8982. doi: 10.15252/msb.20198982 (PMC7285886; doi:10.15252/msb.20198982)
Supplement: Supplementary file 22 — Dataset EV1 [file MSB-16-e8982-s022.zip › PSCM_toolbox/ext/m2html/doc/m2html/private/strtok.html]

Description of strtok


Home > m2html > private > strtok.m

# strtok

## PURPOSE

**Modified version of STRTOK to also return the quotient**

## SYNOPSIS

**function [token, remainder, quotient] = strtok(string, delimiters)**

## DESCRIPTION

```
Modified version of STRTOK to also return the quotient
  string = [quotient token remainder]
STRTOK Find token in string.
   STRTOK(S) returns the first token in the string S delimited
   by "white space".   Any leading white space characters are ignored.

   STRTOK(S,D) returns the first token delimited by one of the 
   characters in D.  Any leading delimiter characters are ignored.

   [T,R] = STRTOK(...) also returns the remainder of the original
   string.
   If the token is not found in S then R is an empty string and T
   is same as S. 

   Copyright 1984-2002 The MathWorks, Inc. 
   $Revision: 5.14 $  $Date: 2002/04/09 00:33:38 $
```

## CROSS-REFERENCE INFORMATION

This function calls:


This function is called by:

- doxysearch DOXYSEARCH Search a query in a 'search.idx' file
- mfileparse MFILEPARSE Parsing of an M-file to obtain synopsis, help and references
- searchindex SEARCHINDEX Compute keywords statistics of an M-file

## SOURCE CODE

```
0001 function [token, remainder, quotient] = strtok(string, delimiters)
0002 %Modified version of STRTOK to also return the quotient
0003 %  string = [quotient token remainder]
0004 %STRTOK Find token in string.
0005 %   STRTOK(S) returns the first token in the string S delimited
0006 %   by "white space".   Any leading white space characters are ignored.
0007 %
0008 %   STRTOK(S,D) returns the first token delimited by one of the
0009 %   characters in D.  Any leading delimiter characters are ignored.
0010 %
0011 %   [T,R] = STRTOK(...) also returns the remainder of the original
0012 %   string.
0013 %   If the token is not found in S then R is an empty string and T
0014 %   is same as S.
0015 %
0016 %   Copyright 1984-2002 The MathWorks, Inc.
0017 %   $Revision: 5.14 $  $Date: 2002/04/09 00:33:38 $
0018 
0019 token = []; remainder = []; quotient = string;
0020 
0021 len = length(string);
0022 if len == 0
0023     return
0024 end
0025 
0026 if (nargin == 1)
0027     delimiters = [9:13 32]; % White space characters
0028 end
0029 
0030 i = 1;
0031 while (any(string(i) == delimiters))
0032     i = i + 1;
0033     if (i > len), return, end
0034 end
0035 start = i;
0036 while (~any(string(i) == delimiters))
0037     i = i + 1;
0038     if (i > len), break, end
0039 end
0040 sfinish = i - 1;
0041 
0042 token = string(start:sfinish);
0043 
0044 if (nargout >= 2)
0045     remainder = string(sfinish + 1:length(string));
0046 end
0047 
0048 if (nargout == 3 & start > 1)
0049     quotient = string(1:start-1);
0050 else
0051     quotient = [];
0052 end
```

---

Generated on Tue 29-Oct-2019 21:04:54 by **m2html** © 2005
