# Supplementary material for: Personalized whole‐body models integrate metabolism, physiology, and the gut microbiome
Source: Mol Syst Biol. 2020 May 28;16(5):e8982. doi: 10.15252/msb.20198982 (PMC7285886; doi:10.15252/msb.20198982)
Supplement: Supplementary file 22 — Dataset EV1 [file MSB-16-e8982-s022.zip › PSCM_toolbox/ext/m2html/doc/m2html/private/mexexts.html]

Description of mexexts


Home > m2html > private > mexexts.m

# mexexts

## PURPOSE

**MEXEXTS List of Mex files extensions**

## SYNOPSIS

**function [ext, platform] = mexexts**

## DESCRIPTION

```
MEXEXTS List of Mex files extensions
  MEXEXTS returns a cell array containing the Mex files platform
  dependent extensions and another cell array containing the full names
  of the corresponding platforms.

  See also MEX, MEXEXT
```

## CROSS-REFERENCE INFORMATION

This function calls:


This function is called by:

- mfileparse MFILEPARSE Parsing of an M-file to obtain synopsis, help and references

## SOURCE CODE

```
0001 function [ext, platform] = mexexts
0002 %MEXEXTS List of Mex files extensions
0003 %  MEXEXTS returns a cell array containing the Mex files platform
0004 %  dependent extensions and another cell array containing the full names
0005 %  of the corresponding platforms.
0006 %
0007 %  See also MEX, MEXEXT
0008 
0009 %  Copyright (C) 2003 Guillaume Flandin <Guillaume@artefact.tk>
0010 %  $Revision: 1.0 $Date: 2003/29/04 17:33:43 $
0011 
0012 ext = {'.mexsol' '.mexhpux' '.mexhp7' '.mexglx' '.mexa64' '.mexi64' '.mexmac' '.dll' '.mexw32' '.mexw64'};
0013 
0014 platform = {'Sun Solaris' 'HP-UX' 'HP-UX' 'Linux PC' 'Linux AMD Opteron' 'Linux Intel Itanium2' 'MacIntosh' 'Windows' 'Windows 32' 'Windows 64'};
```

---

Generated on Tue 29-Oct-2019 21:04:54 by **m2html** © 2005
