# Supplementary material for: Personalized whole‐body models integrate metabolism, physiology, and the gut microbiome
Source: Mol Syst Biol. 2020 May 28;16(5):e8982. doi: 10.15252/msb.20198982 (PMC7285886; doi:10.15252/msb.20198982)
Supplement: Supplementary file 22 — Dataset EV1 [file MSB-16-e8982-s022.zip › PSCM_toolbox/ext/m2html/doc/m2html/@template/private/index.html]

Index for Directory m2html/@template/private


|  |  |
| --- | --- |
| Master index | Index for m2html/@template/private |

# Index for m2html/@template/private

## Matlab files in this directory:

|  |  |
| --- | --- |
| finish | TEMPLATE/FINISH Apply given strategy to unknown fields in a string |
| loadtpl | TEMPLATE/LOADTPL Read a template from file |
| subst | TEMPLATE/SUBST Substitute a replacement field by its value |

---

Generated on Tue 29-Oct-2019 21:04:53 by **m2html** © 2005
