# Supplementary material for: Personalized whole‐body models integrate metabolism, physiology, and the gut microbiome
Source: Mol Syst Biol. 2020 May 28;16(5):e8982. doi: 10.15252/msb.20198982 (PMC7285886; doi:10.15252/msb.20198982)
Supplement: Supplementary file 22 — Dataset EV1 [file MSB-16-e8982-s022.zip › PSCM_toolbox/ext/m2html/doc/m2html/@template/private/finish.html]

Description of finish


Home > m2html > @template > private > finish.m

# finish

## PURPOSE

**TEMPLATE/FINISH Apply given strategy to unknown fields in a string**

## SYNOPSIS

**function str = finish(str,unknowns)**

## DESCRIPTION

```
TEMPLATE/FINISH Apply given strategy to unknown fields in a string
  STR = FINISH(STR,UNKNOWNS) applies on string STR the strategy defined
  in UNKNOWNS to unknowns fields '{UNKNOWNS_FIELDS}'.
  UNKNOWNS may be:
    * 'keep' to do nothing
    * 'remove' to remove all undefined fields
    * 'comment' to replace undefined fields by a warning HTML comment.
  This function uses Matlab REGEXPREP function coming with R13. If you
  hold an older version, please comment lines 38 and 42: then you can 
  only apply the 'keep' strategy.
```

## CROSS-REFERENCE INFORMATION

This function calls:


This function is called by:


## SOURCE CODE

```
0001 function str = finish(str,unknowns)
0002 %TEMPLATE/FINISH Apply given strategy to unknown fields in a string
0003 %  STR = FINISH(STR,UNKNOWNS) applies on string STR the strategy defined
0004 %  in UNKNOWNS to unknowns fields '{UNKNOWNS_FIELDS}'.
0005 %  UNKNOWNS may be:
0006 %    * 'keep' to do nothing
0007 %    * 'remove' to remove all undefined fields
0008 %    * 'comment' to replace undefined fields by a warning HTML comment.
0009 %  This function uses Matlab REGEXPREP function coming with R13. If you
0010 %  hold an older version, please comment lines 38 and 42: then you can
0011 %  only apply the 'keep' strategy.
0012 
0013 %  Copyright (C) 2003 Guillaume Flandin <Guillaume@artefact.tk>
0014 %  $Revision: 1.0 $Date: 2003/05/05 22:19:51 $
0015 
0016 error(nargchk(2,2,nargin));
0017 
0018 switch lower(unknowns)
0019     case 'keep'
0020         %- do nothing
0021     case 'remove'
0022         %%%%%%%%%%%%%%%%%%%%%%%% WIH REGEXP ONLY %%%%%%%%%%%%%%%%%%%%
0023         % str = regexprep(str,'{[^ \t\r\n}]+}','');
0024         %%%%%%%%%%%%%%%%%%%%%%%%%%%%%%%%%%%%%%%%%%%%%%%%%%%%%%%%%%%%%
0025     case 'comment'
0026         %%%%%%%%%%%%%%%%%%%%%%%% WIH REGEXP ONLY %%%%%%%%%%%%%%%%%%%%
0027         % str = regexprep(str,'{[^ \t\r\n}]+}','<!-- Template variable undefined -->');
0028         %%%%%%%%%%%%%%%%%%%%%%%%%%%%%%%%%%%%%%%%%%%%%%%%%%%%%%%%%%%%%
0029     otherwise
0030         error('[Template] Unknown action.');
0031 end
```

---

Generated on Tue 29-Oct-2019 21:04:54 by **m2html** © 2005
