# Supplementary material for: Personalized whole‐body models integrate metabolism, physiology, and the gut microbiome
Source: Mol Syst Biol. 2020 May 28;16(5):e8982. doi: 10.15252/msb.20198982 (PMC7285886; doi:10.15252/msb.20198982)
Supplement: Supplementary file 22 — Dataset EV1 [file MSB-16-e8982-s022.zip › PSCM_toolbox/ext/m2html/doc/m2html/@template/private/loadtpl.html]

Description of loadtpl


Home > m2html > @template > private > loadtpl.m

# loadtpl

## PURPOSE

**TEMPLATE/LOADTPL Read a template from file**

## SYNOPSIS

**function tpl = loadtpl(tpl,handle)**

## DESCRIPTION

```
TEMPLATE/LOADTPL Read a template from file
  TPL = LOADTPL(TPL,HANDLE) read the template file associated with the
  handle HANDLE in the template TPL and store it in the variable HANDLE.
```

## CROSS-REFERENCE INFORMATION

This function calls:


This function is called by:

- subst TEMPLATE/SUBST Substitute a replacement field by its value

## SOURCE CODE

```
0001 function tpl = loadtpl(tpl,handle)
0002 %TEMPLATE/LOADTPL Read a template from file
0003 %  TPL = LOADTPL(TPL,HANDLE) read the template file associated with the
0004 %  handle HANDLE in the template TPL and store it in the variable HANDLE.
0005 
0006 %  Copyright (C) 2003 Guillaume Flandin <Guillaume@artefact.tk>
0007 %  $Revision: 1.0 $Date: 2003/05/05 22:19:51 $
0008 
0009 if ~isempty(get(tpl,'var',handle))
0010     return;
0011 else
0012     ind = find(ismember(tpl.handles,handle));
0013     if isempty(ind)
0014         error('[Template] No such template handle.');
0015     else
0016         filename = tpl.file{ind};
0017         [fid, errmsg] = fopen(filename,'rt');
0018         if ~isempty(errmsg)
0019             error(sprintf('Cannot open template file %s.',filename));
0020         end
0021         tpl = set(tpl,'var',handle,fscanf(fid,'%c'));
0022         fclose(fid);
0023     end
0024 end
```

---

Generated on Tue 29-Oct-2019 21:04:54 by **m2html** © 2005
