# Supplementary material for: Personalized whole‐body models integrate metabolism, physiology, and the gut microbiome
Source: Mol Syst Biol. 2020 May 28;16(5):e8982. doi: 10.15252/msb.20198982 (PMC7285886; doi:10.15252/msb.20198982)
Supplement: Supplementary file 22 — Dataset EV1 [file MSB-16-e8982-s022.zip › PSCM_toolbox/ext/m2html/doc/m2html/@template/private/subst.html]

Description of subst


Home > m2html > @template > private > subst.m

# subst

## PURPOSE

**TEMPLATE/SUBST Substitute a replacement field by its value**

## SYNOPSIS

**function [tpl, str] = subst(tpl,handle)**

## DESCRIPTION

```
TEMPLATE/SUBST Substitute a replacement field by its value
  STR = SUBST(TPL,HANDLE) substitute all the known fields of variable HANDLE
  in the template TPL.
```

## CROSS-REFERENCE INFORMATION

This function calls:

- loadtpl TEMPLATE/LOADTPL Read a template from file

This function is called by:


## SOURCE CODE

```
0001 function [tpl, str] = subst(tpl,handle)
0002 %TEMPLATE/SUBST Substitute a replacement field by its value
0003 %  STR = SUBST(TPL,HANDLE) substitute all the known fields of variable HANDLE
0004 %  in the template TPL.
0005 
0006 %  Copyright (C) 2003 Guillaume Flandin <Guillaume@artefact.tk>
0007 %  $Revision: 1.0 $Date: 2003/05/05 22:19:51 $
0008 
0009 tpl = loadtpl(tpl,handle);
0010 
0011 str = get(tpl,'var',handle);
0012 for i=1:length(tpl.varkeys)
0013     str = strrep(str, strcat('{',tpl.varkeys{i},'}'), tpl.varvals{i});
0014 end
```

---

Generated on Tue 29-Oct-2019 21:04:54 by **m2html** © 2005
